# A Topological Map of the Compartmentalized *Arabidopsis thaliana* Leaf Metabolome

**DOI:** 10.1371/journal.pone.0017806

**Published:** 2011-03-15

**Authors:** Stephan Krueger, Patrick Giavalisco, Leonard Krall, Marie-Caroline Steinhauser, Dirk Büssis, Bjoern Usadel, Ulf-Ingo Flügge, Alisdair R. Fernie, Lothar Willmitzer, Dirk Steinhauser

**Affiliations:** 1 Botanical Institute, University of Cologne, Cologne, Germany; 2 Max Planck Institute of Molecular Plant Physiology, Potsdam-Golm, Germany; 3 GABI Managing Office, c/o Max Planck Institute of Molecular Plant Physiology, Potsdam-Golm, Germany; Dana-Farber Cancer Institute, United States of America

## Abstract

**Background:**

The extensive subcellular compartmentalization of metabolites and metabolism in eukaryotic cells is widely acknowledged and represents a key factor of metabolic activity and functionality. In striking contrast, the knowledge of actual compartmental distribution of metabolites from experimental studies is surprisingly low. However, a precise knowledge of, possibly all, metabolites and their subcellular distributions remains a key prerequisite for the understanding of any cellular function.

**Methodology/Principal Findings:**

Here we describe results for the subcellular distribution of 1,117 polar and 2,804 lipophilic mass spectrometric features associated to known and unknown compounds from leaves of the model plant *Arabidopsis thaliana*. Using an optimized non-aqueous fractionation protocol in conjunction with GC/MS- and LC/MS-based metabolite profiling, 81.5% of the metabolic data could be associated to one of three subcellular compartments: the cytosol (including the mitochondria), vacuole, or plastids. Statistical analysis using a marker-‘free’ approach revealed that 18.5% of these metabolites show intermediate distributions, which can either be explained by transport processes or by additional subcellular compartments.

**Conclusion/Significance:**

Next to a functional and conceptual workflow for the efficient, highly resolved metabolite analysis of the fractionated *Arabidopsis thaliana* leaf metabolome, a detailed survey of the subcellular distribution of several compounds, in the graphical format of a topological map, is provided. This complex data set therefore does not only contain a rich repository of metabolic information, but due to thorough validation and testing by statistical methods, represents an initial step in the analysis of metabolite dynamics and fluxes within and between subcellular compartments.

## Introduction

The partitioning of cellular functions and metabolism into subcellular compartments is a fundamental feature of all eukaryotic cells. Subcellular compartments are usually delineated by a lipid bilayer to maintain compartment integrity and specific microenvironments. Though physically and biochemically distinct, these compartments and their metabolic contents are interlinked by inter-compartmentally transported metabolites [1], [2], [3]. This translocation, as well as the turnover of metabolites, can be exceptionally fast [2], [4], making the reliable determination of metabolites in subcellular compartments challenging. Consequently, the development of methods and strategies to determine the metabolic composition of these compartments is required to gain a comprehensive understanding of the cellular biochemistry.

While subcellular distributions have been determined for a limited number of metabolites using genetically encoded metabolic sensors [5], [6] or direct mass imaging methods on surface tissues [7], the number of approaches devoted towards deciphering subcellular distributions of multiple metabolites is rather limited. The main challenge using destructive approaches is that in order to prevent the leakage of metabolites out of organelles the analysis needs to be performed under anhydrous conditions thus rendering subcellular metabolite analyses strikingly different from e.g. organelle oriented proteomic studies [8].

Non-aqueous fractionation (NAF) is a powerful technique to separate subcellular compartments, and their molecular compositions, under conditions where biological activities are completely arrested due to rapid freezing and dehydration of the sample material [9], [10], [11]. Cellular constituents in proximity to each other aggregate to small particles during lyophilization of the ground sample material. These particles, mainly fragments of cellular compartments, are then separated by their composition-dependent density using equilibrium centrifugation in a non-aqueous gradient. Using compartment-specific marker abundances throughout collected gradient fractions, compartment enrichment and compartmental separation can be assessed. As well, subcellular metabolite distributions can be calculated, usually by applying a two- or three-compartmental calculation strategy [10], [11].

While NAF was first applied to study animal nuclei [9] and mitochondrial high-energy phosphates in mammalian cells [12], [13], [14], later on it has been used mostly in plant sciences in order to determine the partitioning of photosynthetic assimilates in leaves [10], [11], [15], [16], [17], storage organs [18], [19], [20], rose petals [21] or for analysis of specific pathways [22].

In the past decade, technological breakthroughs in mass spectrometry (MS) and nuclear magnetic resonance spectrometry (NMR) [23] have paved the way for comprehensive analyses of an organism's metabolic composition [24], [25]. Even though NMR provides advantages for quantitative and structural metabolomics [26], LC/MS- and GC/MS-based metabolite profiling have become the methods of choice for a general overview of cellular metabolism due to their high throughput, compound coverage, and sensitivity [27]. Despite the increasing use of MS-based metabolite profiling, it has only been combined with NAF in a limited number of studies, basically to unravel the subcellular location of primary metabolites in soybean leaves and potato tubers by means of targeted analyses [20], [28], [29].

Here, we describe the subcellular distribution of a broad range of polar and lipophilic compounds in leaves of the model plant *Arabidopsis thaliana* obtained using three orthogonal MS-based analytical approaches, namely GC-TOF/MS for primary and LC-FT/MS analyses for lipids and semipolar, secondary metabolites. The provided data, which can be regarded as a resource documenting a metabolomic survey of a compartmentally separated leaf, clearly distinguishes the cytosol, the plastids, and the vacuole from one another. Using statistical approaches we were able to demonstrate the robustness of our analyses, assign chemical compounds to the resolved compartments, and to validate our results using structurally annotated (known) metabolites. We further demonstrate that the localizations of several known metabolites and structurally undetermined compounds (unknowns) are difficult to unambiguously explain on the basis of three compartments due to either unresolved compartments, or the interconnections of subcellular metabolic networks.

## Results and Discussion

### Non-aqueous fractionation of *Arabidopsis* leaves allows clear separation of three subcellular compartments

**Figure 1:**
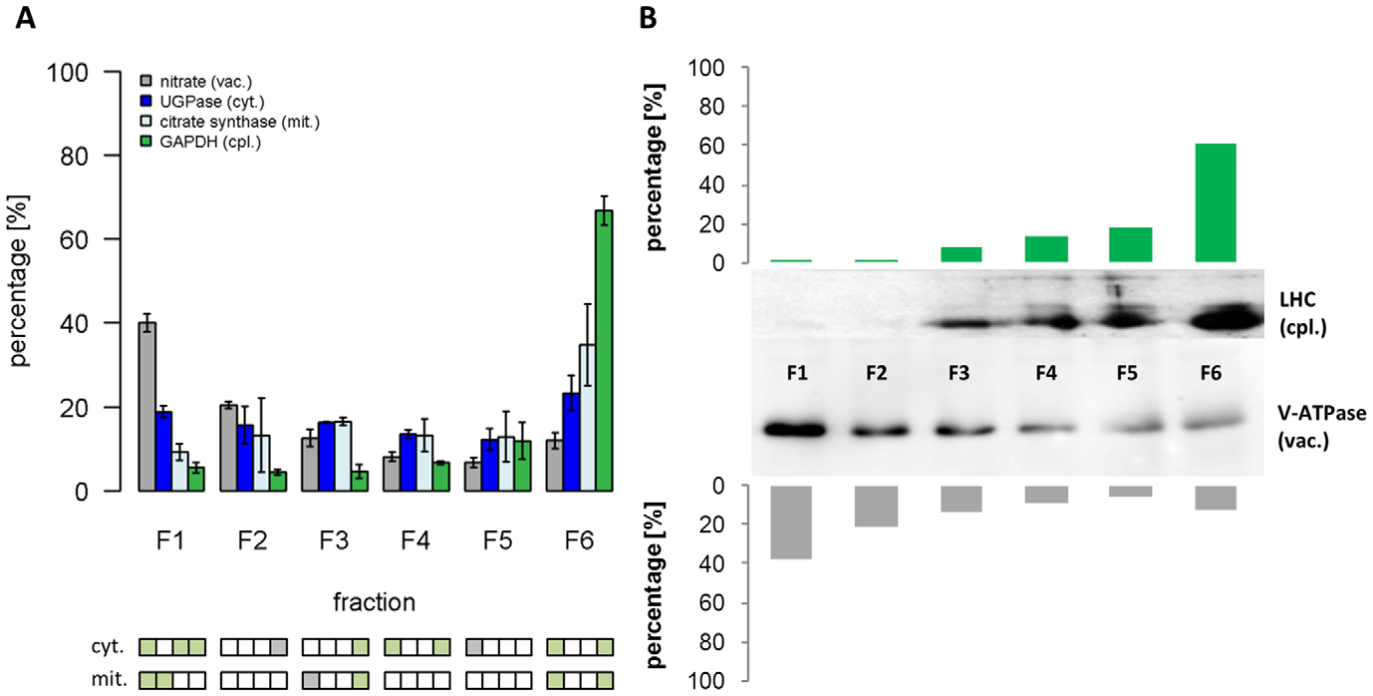
Distribution of compartment-specific markers in non-aqueous gradients from *Arabidopsis thaliana* leaves. (**A**) The distribution of vacuolar (nitrate), cytosolic (UGPase), mitochondrial (citrate synthase), and plastidic (GAPDH) markers are shown as the average of three independent gradients. The mean values and standard deviations of marker enzyme activities (UGPase, citrate synthase, GAPDH) or relative concentrations (nitrate) in each fraction are depicted as percentage from total (scaled data). Significant differences (*P*_BH_<0.05, Benjamini-Hochberg corrected) using *t*-test within each fraction compared to the cytosolic (cyt.) or mitochondrial (mit.) marker are shown as green colored boxes below the graph. Grey boxes illustrate uncorrected significant (*P*<0.05) differences. (**B**) Western blots detecting LHC (plastidic) and vacuolar H^+^-ATPase (vacuolar) membrane proteins in each fraction are shown for one representative gradient to confirm the distribution of the plastidic and vacuolar compartment throughout the gradients. The pixel intensities quantified using ImageJ (http://rsb.info.nih.gov/ij) are drawn as bar diagrams.

In order to analye the subcellular compartmentalization of the plant metabolome, NAF was performed on three independent replicates of pooled *Arabidopsis* leaves from soil grown plants harvested three hours after the onset of light using an optimized NAF protocol [22] (Text S1). NAF separates fragments of subcellular compartments and organelles in a continuous density gradient. Due to the variable composition-dependent density of the fragments, their segregation reflects continuous compartmental distributions throughout the gradient [11]. To unambiguously assign a specific compartment to these distributions, abundances of compartment-specific markers within the six collected gradient fractions were determined. These marker distributions, which must be sufficiently distinct from each other, were then used to evaluate the compartmental enrichment and separation of distinct organelles or subcellular spaces (Figure 1). Nitrate, as vacuolar marker [22], showed a clear enrichment in the densest fraction F1 with 40.1±2.1% (as mean ± SD) which is in agreement with the vacuolar H^+^-ATPase abundance (Figure 1). The cytosolic marker UGPase [30] was relatively equally distributed across the gradients with abundances ranging from 12.3±2.6% to 23.3±4.2%, showing slight increases (F1: 18.9±1.5%; F6: 23.3±4.2%) in the most distant fractions (Figure 1). Contrarily to nitrate, the plastidic marker NADP-GAPDH [11] was clearly enriched in the lightest fraction F6, with 66.8±3.4%, which is in agreement with the abundance of the light harvesting complex (LHC) (Figure 1). Citrate synthase, used as a mitochondrial marker [31] was detected throughout the gradients and revealed a similar distribution as observed for the cytosol, but with decreased abundance in fraction F1 (9.3±2%) and an enrichment in fraction F6 (34.8±9.7%; Figure 1).

Despite the clear intermediate distribution between the cytosolic and plastidic compartment, the mitochondrial marker revealed a relatively large standard deviation and was not enriched in any fraction as compared to the other markers (Figure 1). Therefore, and in agreement with previous reports [e.g. 10], [20], [22], the mitochondrial compartment was not, even though a clear trend could be observed, considered to be unambiguously delineated from the cytosolic compartment. However, with the broad separation of the other markers we were clearly able to obtain an excellent separation of the vacuolar, the cytosolic, and the plastidic compartments by non-aqueous fractionation of *Arabidopsis* leaf material.

### Non-aqueous fractionation produces consistent fraction separation

A total of 18 fractions, resulting from three independent gradients comprised of six fractions each, were subjected to the three MS platforms for polar and lipophilic metabolite analysis (Data S1, S2, S3). In the following, the MS data refers to the analytical approach applied rather than the exact chemical properties of the detected metabolites.

Using GC/MS, 203 analytes, comprising 88 unique metabolites, were consistently identified with 93 (45.8%) and 110 (54.2%) analytes of known and unknown chemical structure (Data S4). High-resolution LC/MS analyses of lipophilic and secondary metabolites resulted in the consistent monitoring of 2,804 and 910 mass spectrometric features (afterwards analytes) comprising 726 and 461 non-redundant peaks (T/S clusters, Text S1). Database searches revealed that 88 (3.1%) and 31 (3.4%) analytes of lipophilic and secondary metabolite profiling represented known metabolites and further 362 (12.9%) and 224 (24.6%) produced database hits with single or multiple potential chemical structures (Data S4).

**Figure 2:**
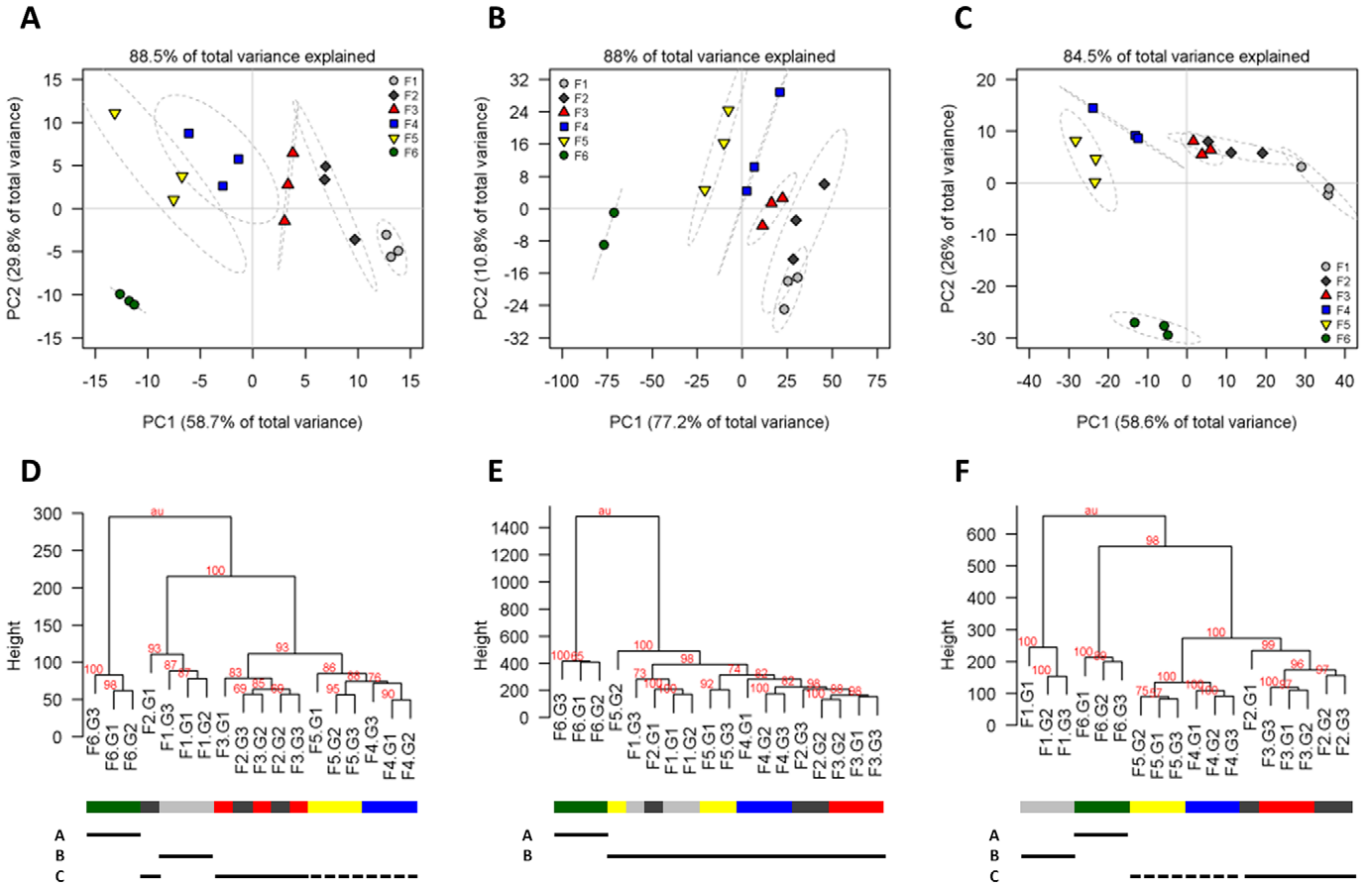
Principal component (PCA; A–C) and hierarchical cluster (HCA; D–F) analyses of metabolite data. Both PCA and HCA plots demonstrate a good separation of the six fractions from each other independent of the three major compound classes, (**A**, **D**) primary, (**B**, **E**) lipophilic and (**C**, **F**) secondary metabolites. PCA and HCA were performed on scaled data. For PCA, data were additionally log_2_-transformed; HCA analyses are based on Euclidean distances among samples. Identical gradient fractions are encoded with the same color and shape as depicted in the graph legend. The 95% confidence ellipse is drawn as a grey dotted line on the basis of the mean, standard deviation, and correlation of the three independent gradient replicates per fraction. To aid interpretation of HCA graphs (D–F) same fractions are identically color-coded (see PCA legend) at the bottom sidebar. The unbiased cluster *P*-values, calculated using multiscale bootstrap resampling, are depicted as red-colored numbers at each node. The fraction groups explaining the highest variance of data and revealing a good cohesion within and separation among assigned fractions are depicted at the bottom of the HCA plots. Fraction groups, evaluated using resampling and gap statistic, were assembled by sequential merging of neighboring sample clusters with fractions assigned using membership majority voting (Figure S1). All graphs clearly support that the plastidic enriched, lowest density fraction F6 (group A) and to a lesser extent the vacuolar enriched, most dense fraction F1 are separated from the intermediately-dense fractions F2 to F5, even though for primary and secondary metabolite data two less-well separated fraction groups (F2–F3 and F4–F5) can be assumed (solid and dotted line; Figure S1).

In order to test whether the individual metabolome data are consistent among the independent gradients and discriminative with respect to fraction separation, principal component (PCA) and hierarchical cluster (HCA) analyses were performed on the scaled and, for the PCA, additionally log_2_-transformed metabolite data (Figure 2). Non-parametric ANOVA using the Mantel test [32] supported separation of the six fraction groups despite low matrix correlations of r = 0.43, r = 0.28, and r = 0.45 (*P*<0.001) for primary, lipophilic, and secondary metabolite data, respectively. Sequential expanding of the fraction grouping (Figure S1) showed significant (*P*<0.001, r = 0.97) differences between the F6 and the other fractions for lipophilic metabolites (Figure 2E). Primary (r = 0.84) and secondary (r = 0.89) metabolite data statistically (*P*<0.001) support the distance of the plastidic (F6) and vacuolar (F1) enriched fractions from the remaining ones, even though relatively high matrix correlations (r = 0.63 and r = 0.66, *P*<0.001) are observed if the two further clusters, comprising the intermediate-dense fractions F2-F3 and F4-F5, are not merged (Figure S1). Mantel tests between sample distance matrices, to determine the overall similarity in terms of similar fraction separation underlying the different metabolome data, showed a very high correlation of r = 0.92 (*P*<0.001) between primary and secondary metabolite data. However, both primary and secondary metabolite data revealed significant but lower correlations with r = 0.82 (*P*<0.001) and r = 0.63 (*P*<0.005), respectively, when compared to the lipophilic metabolite data.

In essence, the three metabolite data sets showed consistency among the data derived from the independent gradients and supported, visually (Figure 2) and statistically (see above; Figure S1), the separation of compartmental-enriched fractions. The plastidic (F6) and to a lesser extent the vacuolar enriched fraction (F1) are distinct from the majority of the intermediate-dense fractions (F2–5). Gap statistics suggested overall less well-separated clusters (Figure S1D–F), likely because there is a continuous distribution of compartments and their metabolite content throughout the gradients (Figure 1).

### Non-aqueous fractionation results in robust compartmental fractionation

**Figure 3:**
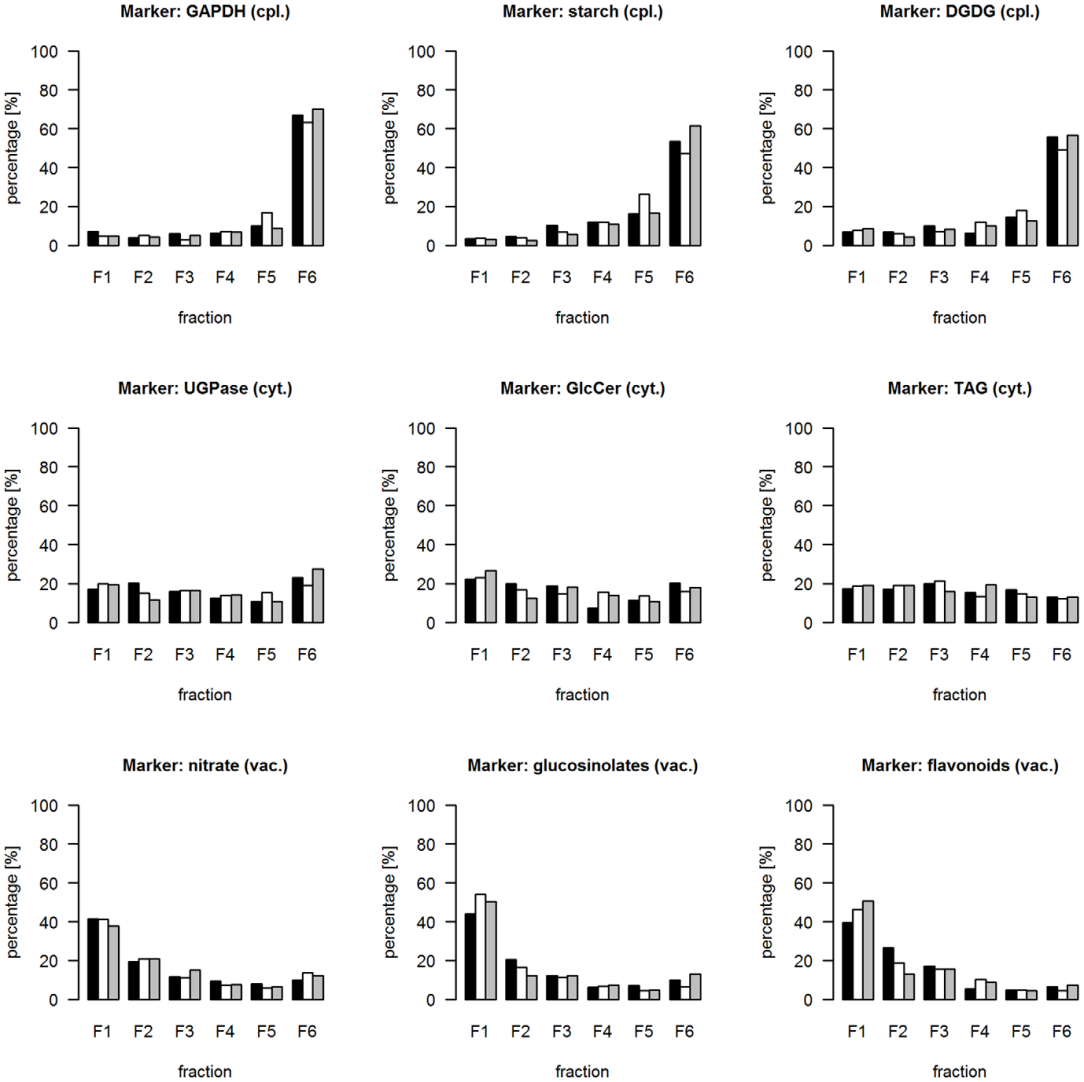
Side-by-side bar plots of marker distribution representing the same subcellular compartment throughout the three independent gradients. For all graphs scaled data were used. Marker names are provided as graph headers including the compartmental representation, i.e. cpl. - plastidic, vac. - vacuolar, and cyt. – cytosolic compartment. The bars are colored as follows: gradient 1 - black, gradient 2 – white, and gradient 3 – grey. For DGDG (cpl.), GlcCer (cyt.), TAG (cyt.), glucosinolates (vac.), and flavonoids (vac.) the abundance per fraction is based on the robust average of multiple analytes representing the individual compound class (Data S4).

As NAF in combination with the MS-based analysis is a complex procedure, various error sources can affect the compartmental separation and downstream estimation of subcellular distributions. To evaluate the consistency and robustness of NAF-derived data further markers were measured or selected from our MS data. Starch was used as additional plastidic marker, as it is synthesized and stored as semi-crystalline granules in plastids during the day [33]. Digalactosyldiacylglycerol (DGDG), a group of galactolipids with high abundances in both envelope and thylakoid membranes [34] were further utilized as plastidic markers. Many classes of secondary metabolites like glucosinolates and flavonoids are reported to be commonly stored in the vacuole (or vacuolar inclusions) of several different plant species [e.g. 35], [36], [37], [38], [39], [40], [41], [42] and thus, represent ideal vacuolar markers. Therefore, based on a targeted analysis we selected a number of glucosinolates and flavonoids/sinapate esters (afterwards for simplicity called flavonoids) (cf. Data S4) reported to be found in *Arabidopsis* (KNApSAcK database and references therein [43]; [44]). As additional markers for the cytosol, triacylglycerides [45] and glyceroceramids, a class of lipids within the sphingolipid group localized to the plasma membrane and to a lesser extent to the tonoplast [46], were used. The results for all nine marker distributions are shown in Figure 3 and demonstrate a high reproducibility of the marker distribution between the three gradients. Likewise, the between-gradient variation of markers designating the same compartment are relatively small with coefficients of variation of 29±8.5%, 19.1±5.8%, and 21.8±7.7% for the plastidic, cytosolic, and vacuolar compartment, respectively (all as mean ± SD; Figure S2A).

### Use of multiple markers results in robust compartmental designation and assignment

As described above, in addition to the three markers used to assign subcellular compartments we took advantage of the fact that within our metabolite data several compounds could be assigned to a specific compartment in an unambiguous way. The availability of these additional markers allowed us to rigorously test the reproducibility of the fractionation procedure and to assess the magnitude of cohesion within and separation between the three delineated compartments.

**Figure 4:**
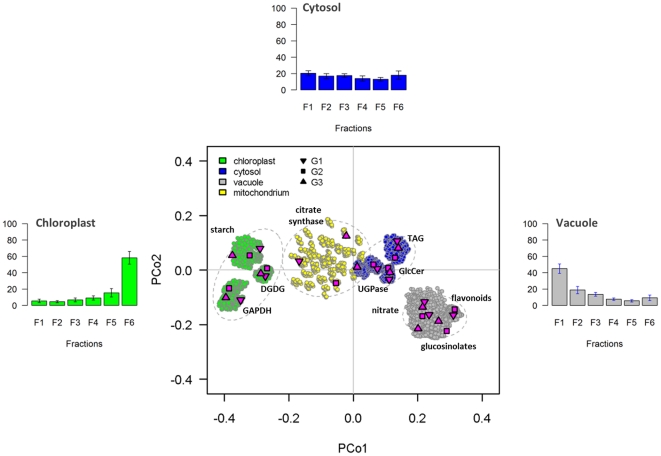
Consistency and robustness of compartmental separation within and between gradients. Manhattan distances among markers for each gradient were converted into a principal coordinates (PCo) space using classical multidimensional scaling (CMD) for the three independent and 729 non-redundant combinations of randomly assembled gradients. Shapes colored in magenta show the data points for the three independent gradients (G1, G2, and G3 as depicted in the figure) of each selected marker. Data points from simulated gradients are depicted as circles with coloration according to the individual compartments: green – chloroplasts, blue – cytosol, and grey – vacuole. For each compartment the 95% confidence ellipse is drawn as a dashed grey line on the basis of the mean, standard deviation, and correlation of the 729 non-redundant gradient combinations. The mitochondrial compartment (yellow circles) shows an overlapping distribution with the cytosol where the majority of data are in between the plastidic and cytosolic compartments. The principal coordinates 1 and 2 explain together in average about 96.7% of the total variance of the underlying distance matrices. For each of the three resolved compartments the distribution of average abundances including their standard deviations throughout the three independent gradients is depicted as bar plots.

Classical multidimensional scaling (CMD; Figure 4) and HCA on normalized Manhattan distances (Figure S2A) among markers and gradients clearly demonstrate the separation of the three considered compartments (Figures 4 and S2). The individual clusters reveal high silhouette information with 0.71±0.05, 0.60±0.06, and 0.61±0.05 for the plastidic, cytosolic, and vacuolar compartment, respectively, with a cluster-solution average of 0.64±0.07 (all as mean ± SD). Thus, a high cohesion within and separation among the clusters is observed, which is supported by gap statistic (Figure S2B) and non-parametric ANOVA using Mantel test (*P*<0.001, r = 0.77). The spread of compartmental clusters, estimated as the clusterwise average of their normalized Manhattan distances, within- and between-gradients is very similar (data not shown), possessing low between-gradient cluster spreads of 0.12±0.05, 0.11±0.03, and 0.11±0.03 (all as mean ± SD) for the plastidic, cytosolic and vacuolar compartment, respectively. Interestingly, the plastidic compartment revealed the largest cluster diameter based on the maximum clusterwise normalized Manhattan distance (0.16±0.02; 0.24) compared to the cytosolic (0.13±0.03; 0.17) and vacuolar (0.14±0.01; 0.19) compartment (within-gradient diameter as mean ± SD followed by between-gradient diameter).

For further robustness evaluation, fractions were systematically assembled into all possible non-redundant artificial combinations (simulated gradients) and the computed normalized Manhattan distances subjected to CMD analyses. Markers representing the same compartment (Figure 4) are in close proximity to each other and do not reveal large variations within their distribution in principal coordinates space. With respect to compartmental clusters, the 95% confidence ellipses and gap statistic (data not shown) clearly support their separation as neither they, nor any data point overlap with any other cluster (Figure 4).

As initially observed, the mitochondrial marker citrate synthase is clearly distributed between the plastid and cytosol with overlap towards the cytosol (Figure 4). When including the mitochondrial compartment the silhouette information of the cluster solution drops (0.5±0.2) with clusterwise values of 0.55±0.10, −0.01±0.10, 0.50±0.14, and 0.61±0.05 for the plastidic, mitochondrial, cytosolic, and vacuolar compartment, respectively (all as mean ± SD). The mitochondrial compartment itself revealed a large between-gradient cluster spread of 0.20±0.003 and diameter of 0.20 compared to the other compartments (see above; Figure 4). In addition, a fourth cluster (mitochondrial) is not fully supported by gap statistic (Figure S2B).

As we lack further unambiguous markers to clearly distinguish the mitochondrial compartment, we therefore prefer to not consider the contribution of this compartment from our NAF gradients. Interestingly, the problem of unequivocally separating mitochondria was already described in previous NAF studies [10], [20], [22]. Reasons for this might be the small size and dispersed localization of mitochondria in the plant cell, as they exist as a population of physically discrete organelles [47]. They are also highly motile within the cell, associating with specific compartments, as seen under stress conditions [48], through association with the actin cytoskeleton [49]. In consequence, the cytosolic compartment must be considered in a broader sense as it represents metabolites with clear cytosolic and/or possible mitochondrial localization.

### Different computational strategies to estimate subcellular distributions result in similar downstream findings and demonstrate statistical robustness

As the compartment-specific markers support an enrichment and robust separation of the three compartments, they enabled a marker-based determination of subcellular metabolite distributions for these compartments (Data S4).

Two different computational algorithms were used, namely the non-negative least square (NNLS) algorithm by Lawson and Hanson [50] and the best fit algorithm (BFA) by Riens [11]. Essentially, both approaches solve a system of linear equations defined by the subcellular compartments (designated by marker abundances throughout the gradient) to find the relative assignment of a metabolite to the compartments by minimizing the discrepancy between the measured and computationally estimated (fitted) fraction abundances of this metabolite. NNLS is based on an active-set approach and seeks linear least square solutions that are also non-negative by minimizing the Euclidean distance [50], which corresponds to the square root of the residual sum of squares (Eq. 1). BFA is based on a heuristic approach and tests all possible subcellular distributions using 1% intervals, i.e. (1^st^) vacuole 100%, cytosol 0%, plastid 0%, (2^nd^) vacuole 99%, cytosol 1%, plastid 0% and so forth, by minimizing the *Q*-value (Eq. 2), the Euclidean distance divided by the number of fractions – 1 [11]. Whereas BFA solutions add up to 100% across the considered compartments, the NNLS solutions, as no other constraints other than non-negative values are given, can sum to above or below 100% (Data S4).

**Figure 5:**
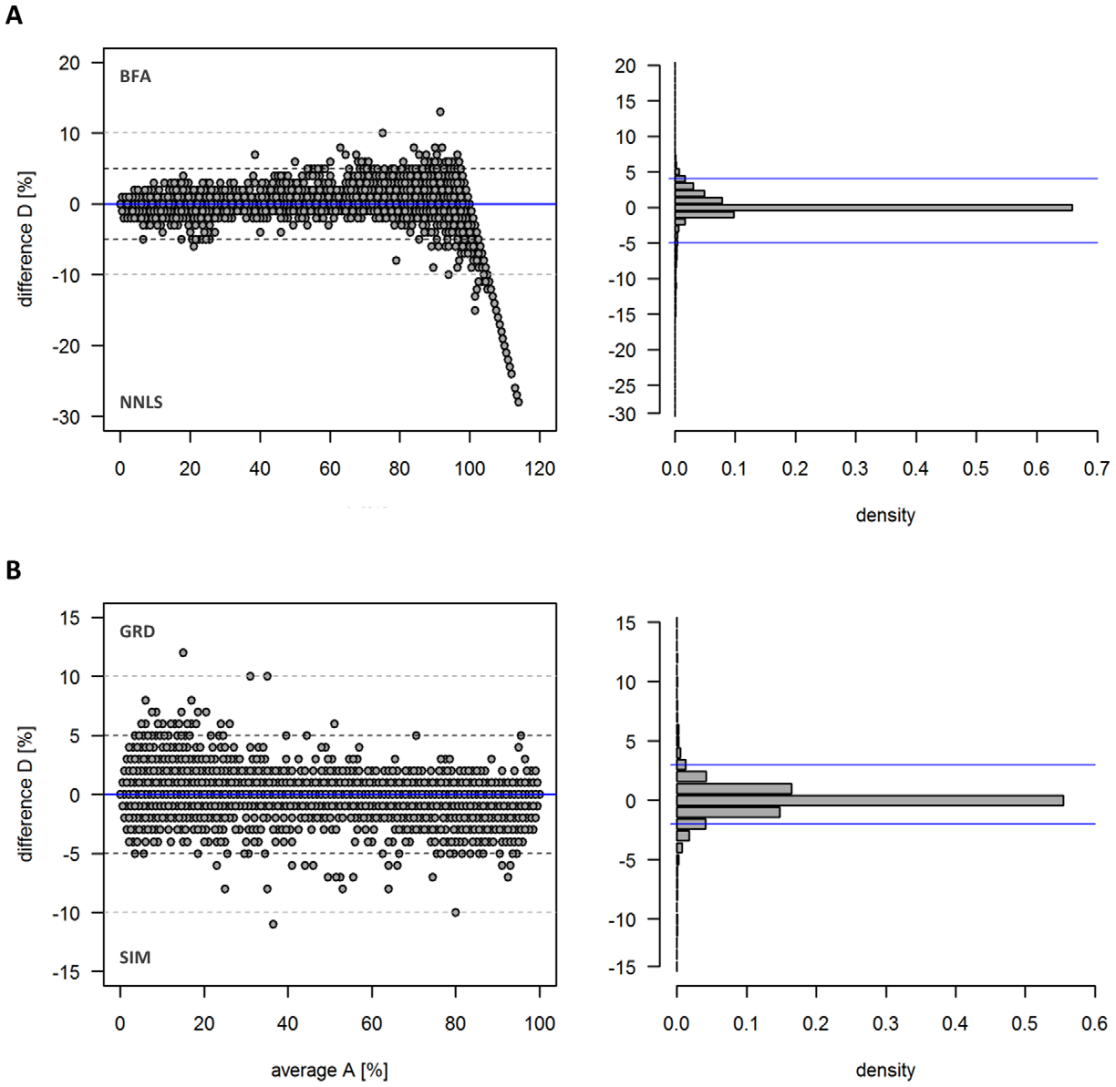
Diagnostic plots showing the differences in estimated subcellular distributions using (A) BFA and NNLS algorithm on the three independent gradients, and (B) using BFA algorithm on the three independent and 729 simulated gradients. The difference versus average plot (left) shows the differences (D) in dependence of the averages (A) of estimated subcellular distributions in a compartment (C, estimated as percentage) between two computational strategies (S): D_i_ = C_i [S1]_ – C_i [S2]_, and A_i_ = 0.5 x (C_i [S1]_ + C_i [S2]_). The corresponding computational strategies depicted in the plots are for (**A**) S1 = BFA and S2 = NNLS solutions, and for (**B**) S1 = 3 (GRD) and S2 = 729 (SIM) gradients. The histogram plot (right) shows the distribution of the M values in 1% intervals with blue solid lines indicating the 2.5% and 97.5% quantiles (95% range of observed differences). For all comparisons the average of estimated subcellular distributions (based on 3 or 729 gradients) are used.

Application of both BFA and NNLS revealed that the average of the estimated subcellular distributions from the three independent gradients are very similar, characterized by a mean difference of about −0.1±2.3% (as mean ± SD) where 95% of the BFA-to-NNLS differences lay in a range of −5% to 4% with tailing at about ≥100% due to the abovementioned algorithms differences (Figure 5A). Comparison of the BFA solutions derived from the independent and simulated gradient data revealed overall small differences of the averaged subcellular distributions with a mean difference of about 0±1.2% (as mean ± SD) where 95% of the differences lay in a range of −3% to 2% (Figure 5B). A similar behavior was observed when using the NNLS solutions (cf. Data S4).

As both BFA and NNLS resulted in similar estimates of subcellular distribution, and computation on simulated gradients revealed the overall statistical robustness, we used the BFA solutions estimated on the three independent gradient data for all further analyses.

### A three-compartmental distribution strategy sufficiently explains the majority of observed analyte distributions

**Figure 6:**
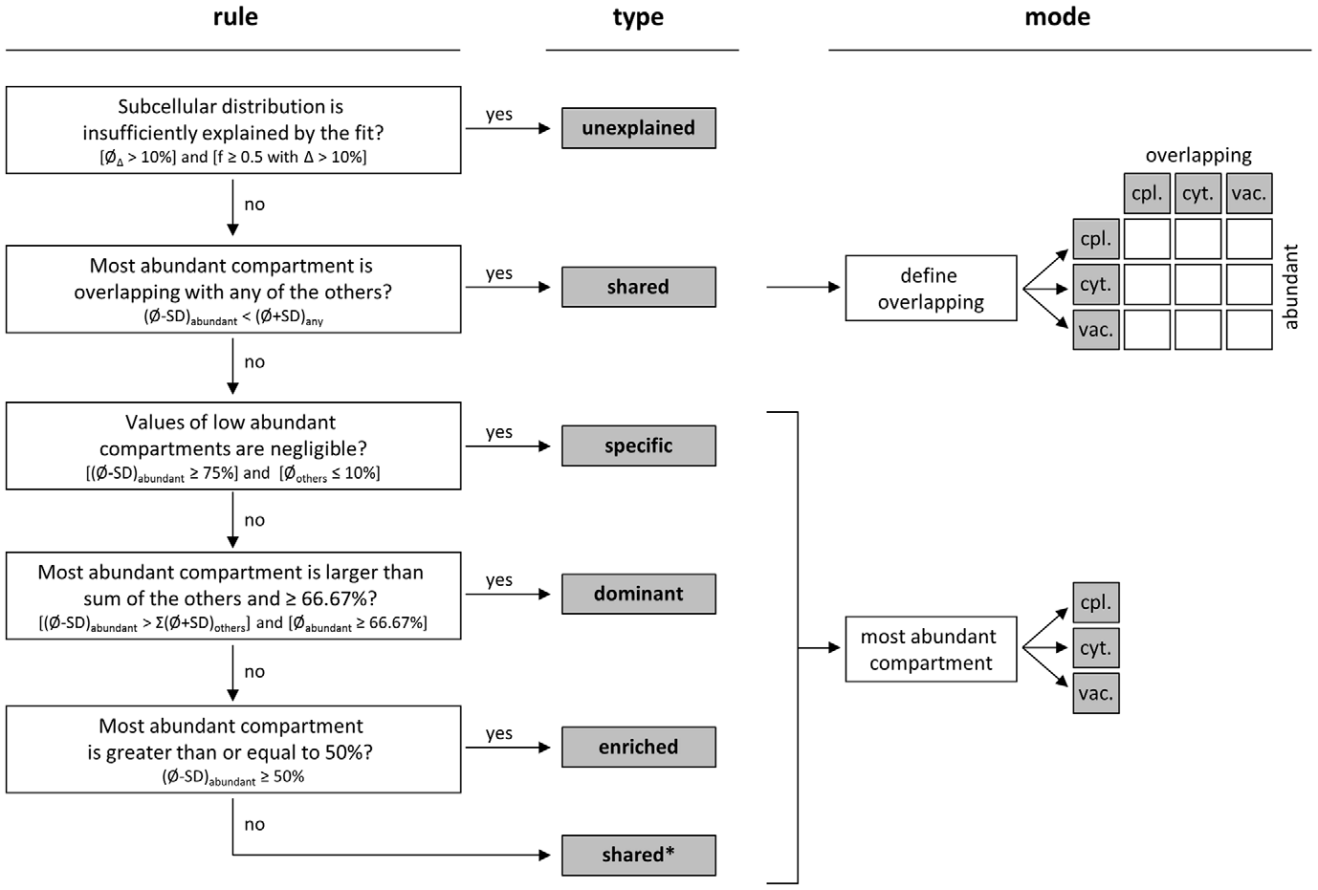
Manually constructed classification tree for the partitioning of analytes into compartments and intermediate units based on their estimated subcellular distribution as well as compartmental abundance and variability. The classification tree was used to assign an analyte into a group defining its subcellular distribution type (type) and an associated mode (mode) which represents one of the resolved compartments or the overlap between them. Assignments are based on the mean and standard deviations of the subcellular distribution, estimated using the best fit algorithm (BFA), for each analyte based on the three independent gradient data. Analytes with insufficiently explained subcellular distributions according to the selected compartment-specific markers are accounted as unexplained and are not further considered in this tree. Analytes revealing sufficiently explained fits are accounted as ‘shared’ if the minimum of the percentage value (min  =  mean - SD) in the most abundant compartment is overlapping with the maximum percentage value (max  =  mean + SD) of any other considered compartment. The corresponding mode is defined by the overlapping compartments regarding the most abundant compartment. Analytes are considered ‘specific’ with the mode according to the estimated most abundant compartment, if the minimum of the most abundant compartment is ≥75% and the values of all other (low abundant) compartments are negligible, i.e. ≤10%. If the minimum in the most abundant compartment is larger than the sum of the maxima among the other compartment and ≥66.67% (2/3 compartments), analytes are accounted as ‘dominantly’ distributed in the respective compartment. Analytes are accounted as ‘enriched’ in a compartment, if the value of the most abundant fraction is ≥50% than the sum of the other compartment. If none of this decisions result in an assignment the analyte is considered as being shared but with enrichment in a particular compartment (‘shared*’).

As criteria for a best fit, the mathematically related measures Euclidean distance, residual sum of squares, or the *Q*-value are usually considered (see above and Eq. 1–3). A tight fit is reflected in small values of the outputs from these equations, however ‘small’ is difficult to define. Therefore we used the normalized Manhattan distance (Eq. 3), the sum of absolute differences between the measured and fitted data, as it ranges from 0 to 100% (or 0.0 to 1.0 if expressed relative) on scaled NAF data. Thus, it describes the total percentage discrepancy (TPD) between the model and the measurements. Subcellular distributions were considered as insufficiently explained (‘unexplained’, cf. Figure 6) if both the average TPD exceeded 10% and the TPD from individual gradients exceeded 10% in ≥50% of the cases. The 10% cutoff was chosen because the difference of individual markers to their respective compartment-specific average was 6.9±1.8% (as mean ± SD) with a maximum of 10.3%.

Using BFA the subcellular distributions of 3,198 (81.5%) out of all 3,922 analytes are considered as sufficiently explained by the averaged compartment-specific markers using a three-compartmental calculation strategy, considering the vacuolar, cytosolic, and plastidic compartments (Table 1; Data S4 and S5). Consequently, the subcellular distributions of 724 (18.5%) analytes are insufficiently explained. Classification based on the compartment-specificity of the three nearest neighboring markers using k-nearest neighbor algorithm (kNN, with k = 3) facilitated the assignment of 487 (67.3%), 174 (24.0%), and 63 (8.7%) of these insufficiently explained analytes into the cytosolic, plastidic, and vacuolar compartments, respectively (Table 1). Interestingly, the mitochondrial marker citrate synthase was considered as insufficiently explained but, as mentioned before, it showed a more cytosol-like distribution and therefore was assigned to the cytosol (Data S4). Similarly, sucrose, a metabolite which is synthesized in the cytosol and transported into sink organs via the phloem, was assigned to the cytosol (Data S4). Earlier conducted NAF studies [10], [11] have already indicated that the observed sucrose distribution could not clearly be ascribed to the cytosolic, plastidic, or vacuolar compartment, most likely due to the greatly higher amounts present in sieve tubes [11].

**Table 1:** Overview of compartmental assignment results.

Compound (cpd.) class	unexplained			specific			dominant			enriched			shared/shared*				total
	*(cpl)*	*(cyt)*	*(vac)*	cpl	cyt	vac	cpl	cyt	vac	cpl	cyt	vac	cpl <> cyt	cyt <> vac	cpl <> vac	other	
**primary cpd.**	8	24	9	7	12	9	11	28	15	4	10	6	15	24	1	20	203
	41 (20.2%)			28 (13.8%)			54 (26.6%)			20 (9.9%)			60 (29.6%)				
**lipophilic cpd.**	146	342	24	326	288	0	284	756	3	49	125	0	270	102	1	88	2804
	512 (18.3%)			614 (21.9%)			1043 (37.2%)			174 (6.2%)			461 (16.4%)				
**secondary cpd.**	20	119	30	10	161	158	39	44	76	14	14	21	48	55	22	79	910
	169 (18.6%)			329 (36.2%)			159 (17.5%)			49 (5.4%)			204 (22.4%)				
**others (+)**	0	2	0	1	0	0	0	0	0	0	0	0	1	0	0	1	5
	2 (40%)			1 (20%)			0 (0%)			0 (0%)			2 (40%)				
**total**	174	487	63	344	461	167	334	828	94	67	149	27	334	181	24	188	3922
	724 (18.5%)			972 (24.8%)			1256 (32%)			243 (6.2%)			727 (18.5%)				

Classification of analytes into classes is based on the BFA-estimated subcellular distributions (Data S4) derived from three independent gradients using a classification tree (Figure 6). Venn diagrams are depicted in Figure S3. Analytes with insufficiently explained (unexplained) distributions using the selected compartment-specific markers are classified by the kNN algorithm using the three nearest neighbor (k = 3) compartment-specific markers.

Therefore, analytes revealing insufficiently explained subcellular distributions may indicate the presence and influence of unconsidered compartments, as the compartment-specific markers used do not encompasses their distribution and thus are inadequate to precisely explain the observed distributions.

### Guilt by association – a three-compartmental distribution strategy facilitates compartmental classification of metabolites

Several metabolites are known to localize to more than one specific compartment; similarly, in our analysis we have observed compounds present in more than one compartment. Explanations for these observations can be the occurrence of biochemical pathways in multiple compartments as well as transport of compounds between compartments. In order to account for these situations, analytes were classified into specific compartments or intermediate assignments based on their compartmental abundance using a classification tree (Figure 6). Five classes were considered:

- ‘specific’,
- ‘dominant’ where, for both, the analyte pool sizes are located dominantly but to different degrees within a designated compartment,
- ‘enriched’ where the pool size in the most abundant compartment is roughly higher than the sum of the others,
- ‘shared’ between compartments,
- shared with enrichment in a specific compartment (‘shared*’).

**Figure 7:**
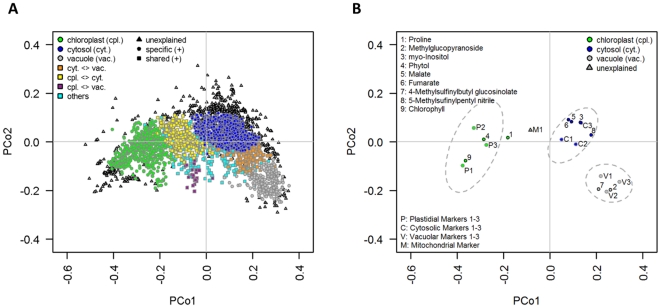
A topological map of the compartmentalized *Arabidopsis thaliana* leaf metabolome for (A) all and (B) selected analytes. The classification for the partitioning of analytes into compartments and intermediate units is based on the best fit - estimated subcellular distribution as well as compartmental abundance and variability for each analyte (for details see Figure 6). The topological map (cf. Data S6 for single analytes) of the classification results for (**A**) all and (**B**) selected metabolites is visualized in principal coordinates (PCo) space on the basis of averaged Manhattan distances among analytes for the three independent gradients. To aid interpretation, analytes of the classes ‘specific’ and ‘dominant’ were both assigned into the respective compartment and color-coded accordingly: green – chloroplast, blue – cytosol, and grey – vacuole. Analytes assigned as being shared between two compartments are color coded as depicted in the figure. With exception of analytes with insufficiently explained (unexplained) subcellular distributions, all other analytes not belonging to one of the above-mentioned classes are defined as ‘others’.

These classification results (Table 1; Figure S3) have been visualized as a topological map of the compartmentalized metabolome (Figure 7; for single analytes see Data S6). In detail, 82 GC/MS analytes (40.4%) were classified as specific or dominant and 47 (23.2%) as shared between compartments. Of the specific or dominant class, 48.8% of analytes were assigned to the cytosol, 22% localized to the plastids, and 29.3% were localized to the vacuole. Of the 488 (53.6%) specific and dominantly assigned analytes derived from secondary metabolism, 48% were assigned to the vacuole, 42% to the cytosol, and only 10% were localized to the plastid. 1,657 (59.1%) out of all lipophilic analytes displayed specific and dominant subcellular distributions. Of them, the majority, 63%, were assigned to the cytosol and 36.8% to the plastids. Lipophilic compounds showing specific or dominant pool sizes in the vacuole are negligible (0.2%).

Overall the compartmental assignment varies regarding the major compound classes (Table 1; Table S1, S2, S3). Many lipophilic metabolites can be found localized to plastids and the cytosol, as both encompass large internal membrane systems. As well, plastids are the site of plant fatty acids synthesis [34], [51]. In contrast, many secondary metabolites are dominant or even specific for the vacuole and cytosol, reflecting their synthesis and storage location as has been supported by protein localization studies [e.g. 36], [41], [52]. Analytes of primary metabolism revealed the largest diversity regarding compartmental class assignments as these compounds are crucial constituents for many pathways. They are localized to each compartment, with 29.6% analytes revealing shared pool sizes.

### Literature confirmation of selected metabolites demonstrates robustness, relevance, and facilitates hypothesis deduction

As described above we followed a comprehensive approach to assign as many analytes into specific compartments as possible. To the best of our knowledge, this study, with respect to its comprehensiveness, is the first of its kind. Therefore we decided to validate the data by linking it to prior knowledge.

In our study, most of the amino acids were highly abundant in the cytosol and chloroplasts (Table S1), which is in agreement with results obtained for leaves of other plant species [11], [53]. Proline, which is synthesized in chloroplasts and the cytosol of mesophyll cells [54], is dominantly plastidic localized (67±6%; Table S1). This localization fits its function as ROS scavenger and singlet oxygen quencher during photosynthesis [55]. Methylglucopyranoside (MeG), a secondary metabolite synthesized by direct transfer of methanol onto glucose in the cytosol of *Geum montanum*, is rapidly transported into the vacuole where it accumulates to more than 95% [56]. The high vacuolar abundance of MeG (95±9%; Table S1) indicates that MeG metabolism in *Arabidopsis* might be similar, at least in terms of storage. Recently, it was shown that myo-inositol accumulates in the cytosol and not in the vacuole of *Mesembryanthemum crystallinum* [57] supporting its cytosolic localization (100±1%) in *Arabidopsis* (Table S1). Phytol is released during chlorophyll degradation by a chloroplast-located pheophytinase [58]. The free phytol residue is redirected into chloroplast lipid metabolism [59] which would support an abundant plastidic pool (90±10%) as shown (Table S1).

Malate and fumarate were localized mainly in the cytosol and did not accumulate in the vacuole (Table S1) which is in contrast to reports indicating a large vacuolar pool [10]. However, in C3 plants malate accumulates during the day with a maximum at the end of the light period, only being transported into the vacuole after reaching a threshold concentration [60]. A further observation concerns the predominant aliphatic glucosinolate in Arabidopsis, 4-methylsulfinylbutyl glucosinolate (glucoraphanin), which revealed a dominant pool size within the vacuole (88±19%; Table S3). Glucoraphanin can be hydrolyzed by myrosinase into 5-methylsufinylpentylnitrile, which was dominantly localized in the cytosol (80±18%). In *Arabidopsis* myrosinase is localized in the vacuole of idioblastic cells of the phloem parenchyma [61], whereas glucosinolates are commonly reported to be stored in the vacuole, indicating that substrate and enzyme are likely not co-localized in the same cell [36], [61], [62]. The detection of glucoraphanin in the vacuole and the degradation product 5-methylsufinylpentylnitrile in the cytosol (Figure 7B) therefore provides evidence for the transport and catabolism of glucosinolates under physiological conditions that does not involve tissue disruption by herbivore attacks. Even though little is known about glucosinolate catabolism in plants, their concentrations can significantly vary in leaves during diurnal cycle [63], [64] or specific glucosinolates can be degraded during developmental processes [65].

Based on the within-compartment distance of the three markers, the plastidic compartment seemed to be resolved to a higher resolution than the others. Whereas starch is stored within the plastidial stroma [33], the galactolipids, MGDG and DGDG (Table S2), are found in both envelope and thylakoid membranes [34]. Surprisingly, NADP-GAPDH, an enzyme found within the stroma, is clearly deviant from both (Figure 4), and also showed a very similar distribution and close proximity to chlorophyll (Figure 7B; Data S6) and the light harvesting complex (Figure 1B). Studies in spinach [66] and *Synechocystis* [67] have provided circumstantial evidences that the Calvin cycle multienzyme complex seems to be bound to thylakoid membranes and thus may indicate a partial separation of the thylakoid and the stroma of plastids under our NAF conditions.

### Recurring distribution patterns throughout the gradients suggests the existence and contribution of previously unconsidered compartments

As described above, the vast majority (81.5%) of analytes could be assigned to one of the five classes. In contrast, the subcellular distributions for another 724 analytes (18.5%) could not be sufficiently estimated as the compartment-specific markers did not encompass their distribution (Table 1; Data S4). These include aspartate, asparagine, glutamate, glutamine, serine as well as the mitochondrial marker citrate synthase amongst others. Considering that the distribution for these metabolites resembles, to some extent, the situation for the mitochondrial compartment, which also could not be unambiguously delineated, therefore we speculated that unresolved or unconsidered compartments may contribute to the recurring and unexplained distribution patterns. To test this hypothesis on the 724 analytes we tried to identify analyte groups characterized by similar, yet distant and reproducible distributions using a marker-‘free’-based classification by k-medoids clustering, allowing only the cytosolic compartment to be partitioned into different clusters without being assigned to another compartment.

**Figure 8:**
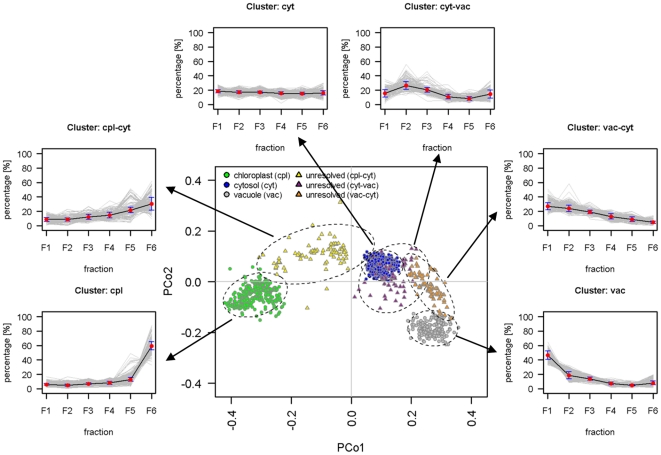
Scatter and distribution plots of analytes with compartment-specific and unresolved subcellular distributions. Analytes with compartment-specific distributions were identified using a classification tree based assignment (Figure 6). Analytes with insufficiently explained subcellular distribution were grouped according to k-medoids clustering (Figure S4A). For visualization, Manhattan distances among analytes for each of the three independent gradients were averaged and then converted into a principal coordinates (PCo) space. Analytes were color-coded according to their cluster membership. For each identified cluster the distribution of members throughout the gradients (grey lines) and the robust average distribution including standard deviations (black lines) are depicted as line plots. Despite the overlap of the intermediate clusters with the resolved compartments, recurring and stable distribution patterns have been observed.

This resulted in the identification of seven clusters of which two are represented by the cytosolic compartment (Figure S4A; Data S4). Out of the 724 analytes with insufficiently explained distributions 339 (46.8%) were assigned into one of the two cytosolic, 125 (17.3%) into the plastidic, and 26 (3.6%) into the vacuolar cluster. Further 234 (32.3%) analytes were assigned into three novel clusters (Figure 8). Mantel tests performed as non-parametric ANOVA revealed significant (*P*<0.001) and intermediate matrix correlations of r = 0.51±0.02 (as mean ± SD). The same approach, but restricted to the clusters reflecting the three considered compartments, resulted in a higher average matrix correlation of r = 0.82±0.01. Using gap static both biologically-driven cluster solutions are supported, however it indicated that the seven considered clusters are less well-separated (Figure S4C). Despite a certain degree of cluster overlap, line plots displayed robust intermediate cluster distributions in between the delineated subcellular compartments (Figure 8).

Specifically, the largest intermediate cluster ‘cpl-cyt’ (n = 94) showed similarity to the robust consensus distribution of the plastidic compartment, displaying increased abundance in fraction F5 and a decreased abundance in fraction F6 (Figure 8). Amongst others aspartate, glutamate, asparagine, and the mitochondrial marker citrate synthase are assigned into this cluster. Interestingly, it was shown that the glutamine synthase GLN2 is targeted between both chloroplasts and mitochondria and facilitates ammonium recovery by transferring ammonium to glutamate during photorespiration [68]. Aspartate aminotransferase activity in mitochondria indicates that aspartate, as its substrate, is also present [69]. Together with the mitochondrial marker citrate synthase this intermediate cluster may represent metabolites captured in transport between the plastids and mitochondria but as well as the cytosol, as serine, involved in photorespiration, is assigned into one of the cytosolic clusters (Data S4).

The cluster ‘vac-cyt’ comprises 80 mainly unknown analytes (Data S4) and has similarity to the robust consensus distribution of the vacuolar compartment. The abundances in the densest fractions F1 and F2 are similar, whereas for the vacuolar compartment the abundance in fraction F1 is about 2-fold higher compared to F2 (Figure 8).

The smallest cluster ‘cyt-vac’ comprises 60 members of which 59 are unknown secondary metabolites (Data S4). It strongly overlaps with the cytosolic and the ‘vac-cyt’ clusters, and shows the highest abundances in the fractions F2 and to a lesser extent F3 (Figure 8). Interestingly, most of these analytes are relatively large (average m/z 640) and have a relatively late retention time (53 with RTs greater than 14 min), indicating that these compounds could be very hydrophobic. At this point it might appear speculative to hypothesize about the provenience of these compounds since many reasonable explanations seem possible, still it is tempting to propose that this unusual cluster with specific distribution (as for the cluster ‘vac-cyt’) could be a derivative of the highly heterogeneous vacuole [70]. Another likely explanation could be that we are capturing some vesicles channeled between compartments [71], [72] or that we simply see an unconsidered compartment like the endoplasmatic reticulum (ER). The later would be supported by the structurally annotated metabolite 4-hydroxybenzoate, a precursor for the synthesis of the antimicrobial metabolite shikonin [73], [74] as well as an intermediate in ubiquinone biosynthesis. In both cases the biosynthetic reactions involving 4-hydroxybenzoate are localized in the ER and Golgi apparatus [75], [76] or in small vesicle derived from the ER [73], [77], [78]. A targeted proteomic or immunological approach towards the enzymes involved in these reactions might strengthen or dismiss this hypothesis.

Nevertheless, despite the identification of robust recurrent distribution patterns (Figure 8), the observed distributions are generally not distinctive enough when compared to the defined subcellular compartments. However, when this approach was applied on all analytes (Figures S4B), the intermediate cluster ‘cpl-cyt’ was supported (cf. Figures S4D), demonstrating that the observed intermediate distributions can be robustly identified. Even though a further subcellular compartment cannot be unambiguously delineated, the subcellular distributions of analytes with sufficiently explained distributions assigned into this cluster might be partially overestimated as this cluster comprises the mitochondrial marker and therefore metabolites shared between the mitochondria and plastids/cytosol (see above; Data S4).

### Concluding remarks

By using an untargeted metabolic approach in combination with the development of an advanced method for critical analysis of NAF-derived metabolic data, we have gathered a comprehensive description of a compartmentalized (with regard to the cytosol (including the mitochondria), chloroplast, and vacuole) metabolome of an eukaryotic organism. The resultant comprehensive metabolic map of *Arabidopsis* leaves provides a resource that can serve as a basis to identify constraints and key processes as targets for biotechnology or for systems-biology driven research.

A precise understanding of how metabolites are synthesized, stored, and transported is critical for a better understanding of subcellular biochemical networks which will be important in biotechnological applications, as well as providing a basis to refine metabolic models by considering the subcellular localization of dominant pool sizes. This fact is of particular importance for plant energy metabolism which is closely linked with the plant plastid, mitochondria, and cytosol. In frame with this it will be of interest to sufficiently delineate not only the mitochondria from the cytosol but also to uncover novel subcellular distributions. While marker-‘free’ reconstructions showed the contribution of unconsidered compartments in our data, an unambiguous designation and biological description for these compartments could not be achieved as they are mainly comprised of structurally unknown analytes. Currently, this represents one of the main limitations in NAF studies, as even the subcellular localization of structurally identified (known) metabolites are often not described in literature and even then their localization might still be variable. Therefore it is clear that a comprehensive framework of markers needs to be established to align and assemble metabolites based on the measurement of known, unambiguously localizable molecules. For this purpose it will be necessary to include, along with the metabolic data, more protein analyses. These could be either provided using more antibody-based assays or by performing proteomic measurements on the gradient fractions. Nevertheless, having developed the presented metabolomics resource we have also laid the groundwork needed in order to perform and analyze more complex experiments, such as a time course or changing environmental conditions.

With the biological validation of the dataset, and the promise in the future to be able to name some of the unknowns, this topographical map can aid in the discovery of novel transporters, biosynthesis enzymes, and generate hypotheses for undiscovered pathways. As NAF and the whole metabolomics platform are applicable to any eukaryotic organism, the provided optimized protocol (Text S1) for *Arabidopsis* and statistical workflow should be adaptable to many other organisms.

## Materials and Methods

### Plant growth

All wild-type *Arabidopsis thaliana* Col-0 plants were grown on soil for two weeks under short day conditions (8 h light) before being transferred for three weeks to long day conditions (16 h light) with 140 µmol m^−2^ s^−1^ photon flux density and a temperature of 21°C at 50% relative humidity. A total of 4–8 g pooled plant leaf material from individual plants was harvested at the beginning of the light period (about 3 h after light switched on), snap-frozen in liquid nitrogen, and stored at −80°C until use.

### Non-aqueous fractionation

For determination of subcellular metabolite levels, cellular compartments were separated using density gradient centrifugation under non-aqueous conditions according to the methods for leaf material [11] with optimized conditions [22] (Text S1). Frozen *Arabidopsis* leaf material was homogenized using a ball mill, pre-cooled in liquid N_2_ to avoid thawing, instead of using a mortar as mortar-ground material was insufficiently filtered through a 20 µm nylon net (used instead of quartz wool (data not shown)). The gradient volume, composed of the non-polar solvents tetrachlorethylene/heptane, was increased from 12 to 28 mL using a much smaller linear density ρ from 1.43 g cm^−3^ to 1.62 g cm^−3^. Most of the sample material was focused in the middle fractions with exception of the plastidic compartment enriched within the top fractions (data not shown). By testing several centrifugation velocities and durations, equilibrium distribution was already achieved at 5,000 g and 50 min instead of 25,000 g and 180 min [cf. 11], shortening the exposure time of sample material to the non-aqueous solvents.

### SDS–PAGE and Western blotting

SDS–PAGE and Western blotting were conducted as described [79]. Western blots were blocked with skimmed milk and probed with polyclonal primary antibody against the light harvesting complex (LHC) from *Pisum sativum* or the subunit E of the vacuolar type H^+^-ATPase (V-ATPase; Abcam plc, Cambridge, UK). Anti-rabbit horse radish peroxidase-conjugated secondary antibodies were used to detect primary antibodies. All blots were developed using ECL Western blotting kit (GE Healthcare, Munich, Germany).

### Enzyme and metabolite assays

Enzyme assay extracts were prepared according to Geigenberger and Stitt [80]. NADP-dependent glyceraldehyde-3-phosphate dehydrogenase (GAPDH, EC 1.2.1.12) was measured as described by Stitt et al. [81]. Uridine diphosphate (UDP)-glucose-pyrophosphorylase (UGPase, EC 2.7.7.9) was assayed according to Zrenner et al. [82]. Citrate synthase (EC 2.3.3.1) activity was determined as described [83]. Chlorophyll was extracted twice with 80% (v/v) and once with 50% (v/v) hot ethanol (30 min, 95°C) and determined as outlined by Arnon [84]. Starch was measured from the remaining pellet of ethanolic extracts according to Hendriks et al. [85]. Nitrate was analyzed by enzymatic reaction as described [86].

### Metabolite profiling

For GC-TOF/MS analyses, dried fraction aliquots were extracted with cold 10∶3∶1 (v/v/v) methanol:chloroform:water (MCW) solution and two extract aliquots (100 µL, 150 µL) were derivatized and analyzed as described [87] with m/z acquisition of 85–750. The established GC/MS protocol allows quantification of sugars, sugar alcohols, organic and amino acids, ascorbate and some lipophilic compounds [87], [88], [89].

For LC/MS analyses lipophilic and secondary metabolites were extracted from dried fraction aliquots with cold 2.5∶1∶1 (v/v/v) MCW solution under shaking and sonication. After phase separation, aliquots of the upper, aqueous phase and lower, organic phase were dried and resuspended in ddH_2_0 (secondary metabolites) or 50∶20∶25 (v/v/v) isopropanol/hexane/water (lipids). Extraction and derivatization of individual soluble thiols (cystein, γ-glutamylcysteine, glutathione) were performed as described [90]. UPLC separation of soluble thiols, secondary, and lipophilic metabolites were performed on a Waters Acquity UPLC system (Waters, Mildford, MA, USA) equipped with a BEH C_18_ (thiols), a HSS T3 C_18_ (secondary metabolites), or a BEH C_8_ (lipids) reversed phase column (Waters) coupled to a Fourier Transform Ion Cyclotron Resonance Mass Spectrometer (thiols) or an Exactive Orbitrap (secondary and lipophilic metabolites) (both Thermo Fisher Scientific, Bremen, Germany). Mass spectra were recorded in full scan, positive ion mode with m/z acquisition of 100–1500 and 200–600 using 25,000 and 50,000 ppm resolution for soluble thiols and secondary or lipophilic metabolites, respectively (Text S1).

### MS data analyses

GC/MS data were processed and aligned as described [87] using a curated library of authentic standards and unknown *Arabidopsis* compounds comprising 1,032 unique spectral entries (Krall et al., in prep.). The aligned data with 413 found library entries, were evaluated and curated (Text S1). The filtered raw GC/MS data comprises 40 samples and 203 curated analytes with 1 (0.01%) missing value. All GC/MS data were expressed relative to U-^13^C-sorbitol and extract replicates averaged after TIC normalization (Data S1).

High-resolution MS data were aligned or peaks extracted using GeneData (v5.3.7, Basel, Swizerland) and Xcalibur (v2.06, Thermo). Aligned FT-MS data, comprising 16,262 and 53,785 time-m/z features (afterwards analytes) of lipophilic and secondary metabolites, were filtered for consistently found analytes (Text S1). These resultant peak lists were then searched against KEGG [91] and KNApSAcK [43] for secondary metabolites using an in-house developed database search tool (GoBioSpace, Hummel et al., unpublished) while the lipid data was searched against an in-house compiled lipid database (Giavalisco et al., submitted). These filtered and uncurated data were derived from 20 samples comprising 2,804 and 910 analytes with 1,125 (2%) and 457 (2.5%) missing values for lipophilic and secondary metabolites, respectively (Data S2–S3). These analytes were annotated onto three levels: unknown, if no database hit could be assigned; match if an unverified database hit was assigned; and known for orthogonally validated database hits. The validation of known metabolites does not include the use of authentic reference standards, but instead relies on previously described compounds for Arabidopsis, the use of validated fragmentation patterns, and mass shifts of ^13^C, ^15^N, and ^34^S isotope labeled *Arabidopsis thaliana* samples (Giavalisco et al., submitted). In order to estimate the number of potential non-redundant analytes within the FT-MS data, a correlative approach similar as described [92] was conducted by defining time/similarity (T/S) clusters (Text S1).

The individual MS data were assembled into a joint data set including metabolites measured by targeted MS approaches (thiols) and metabolic assays (chlorophyll, starch) (Data S4).

### Statistical analyses and visualization

All statistical analyses were performed if not otherwise stated according to Sokal and Rohlf [32] using R 2.9.1.

Metabolite data were normalized to adjust for sample amount variations using the total ion count within and among gradients (Text S1). Analyte abundances were expressed as percentage from total (scaled data). Missing values were imputed by principal component analyses (PCA) [93]. Outliers, extreme deviations from the respective fraction means, were detected by a boxplot approach and replaced with the corresponding fraction mean to promote extraction of biological relevant and robust information (Text S1). The processed, i.e. normalized, imputed, and outlier-removed data are provided as supplemental data (Data S1, S2, S3).

Robust consensus distributions throughout gradients were computed using Tukey's biweight. The *t*-test was performed two-sided with equal or unequal variance determined using *F*-test. *P*-values were adjusted by Benjamini-Hochberg correction (*P*_BH_) [94] to control the false discovery rate. Mantel tests were performed as Pearson's matrix correlations (r) between distance matrices or as non-parametric ANOVA. HCA using average linkage clustering were performed on Euclidean (Eq. 1) or Manhattan distances. *P*-values for cluster nodes were computed with R's pvclust [95]. Classical multidimensional scaling (CMD; [96]) on normalized Manhattan distances (Eq. 3) among analytes was used to reflect distances as points in principal coordinates space. This approach was used to visualize and assess the proximity of a metabolite (or compartment) to the delineated compartments. Gap statistic was performed to estimate the number of clusters [97].

To estimate the robustness of downstream results, fractions were randomly assembled into a total of 729 (726 random +3 original combination) non-redundant artificial gradients and analyses were repeated.

### Compartmental distribution and assignment

Subcellular metabolite distributions were computed using the BestFit command line tool (available upon request) by a three-compartmental distribution strategy utilizing the best fit (BFA) [11] and non-negative least square (NNLS) [50] algorithm. The abundances of all markers delineating the same compartment were averaged for each gradient separately prior to computation.

Analytes were assigned onto the three resolved subcellular compartments using a k-nearest neighbor (kNN) approach [98] with k = 3 nearest neighbors (estimated using cross-evaluation) on normalized Manhattan distances (Data S4). Refined compartmental assignments (Data S4) were performed using a classification tree based on observed subcellular distribution (Figure 6) and marker-‘free’ by means of robust k-medoids clustering (PAM, partitioning around medoids). The number of clusters (k) was determined by allowing only the cytosolic compartment (represented by three compartment-specific markers) to be partitioned into different clusters without being assigned onto another compartment. The validity of identified cluster numbers was evaluated using gap statistics. Non-parametric ANOVA by means of Mantel test was performed on 5 randomly selected cluster members for each cluster and repeated 999 times.

### Equations

(Eq. 1) Euclidean distance *d_E_*

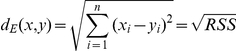



RSS  =  residual sum of squares

(Eq. 2) *Q*-value
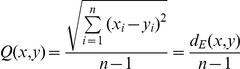



(Eq. 3) Normalized Manhattan distance *d_m_* (on scaled data)
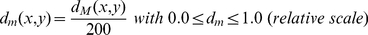
(3a)

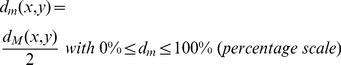
(3b)


with 
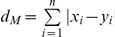
 (Manhattan distance)

## Supporting Information

Figure S1 Box plots illustrating (A–C) the silhouette information and matrix correlation of assembled fraction group solutions as well as (D–F) the gap statistics to estimate the number of sample clusters on the basis of (A, D) primary, (B, E) lipophilic and (C, F) secondary metabolite data.
(DOC)

Figure S2 (A) Heatmap and cluster distribution of selected markers representing the three resolved subcellular compartments and (B) gap curves to estimate the number of marker clusters.
(DOC)

Figure S3 Venn diagrams of compartmental assignments of analytes separated according to the major compound classes (A) primary, (B) lipophilic, and (C) secondary metabolite data.
(DOC)

Figure S4 Graphical visualization of (A, B) classification results and (C, D) gap curves based on k-medoids clustering regarding (A, C) analytes with insufficiently explained (unexplained) subcellular distributions and (B, D) all analytes.
(DOC)

Table S1 Subcellular metabolite distribution and assignment results for selected major compound classes of primary metabolic compounds.
(DOC)

Table S2 Subcellular metabolite distribution and assignment results for selected major compound classes of lipophilic metabolic compounds.
(DOC)

Table S3 Subcellular metabolite distribution and assignment results for selected major compound classes of secondary metabolic compounds.
(DOC)

Text S1 Supplemental extended methods and detailed supplemental data description. Further details of non-aqueous fractionation, mass-spectrometry based metabolome analyses as well as data and statistical analyses are provided.
(PDF)

Data S1 Raw and processed GC-TOF/MS data of primary metabolites.
(XLS)

Data S2 Raw and processed UPLC-FT/MS data of lipophilic metabolites.
(XLS)

Data S3 Raw and processed UPLC-FT/MS data of secondary metabolites.
(XLS)

Data S4 Fused metabolome data set covering analyte annotations as well as results of estimated subcellular distributions and compartmental assignments.
(XLS)

Data S5 Distribution of measured and fitted fraction abundances of analytes across the gradient based on three independent gradient data.
(PDF)

Data S6 Scatter plots of analytes and compartment-specific markers in the principal coordinates space for visual assessment of subcellular location.
(PDF)
