# Supplementary material for: A Topological Map of the Compartmentalized Arabidopsis thaliana Leaf Metabolome
Source: PLoS One. 2011 Mar 15;6(3):e17806. doi: 10.1371/journal.pone.0017806 (PMC3058050; doi:10.1371/journal.pone.0017806)
Supplement: Figure S1 — Box plots illustrating (A–C) the silhouette information and matrix correlation of assembled fraction group solutions as well as (D–F) the gap statistics to estimate the number of sample clusters on the basis of (A, D) primary, (B, E) lipophilic and (C, F) secondary metabolite data. (DOC) [file pone.0017806.s001.doc]

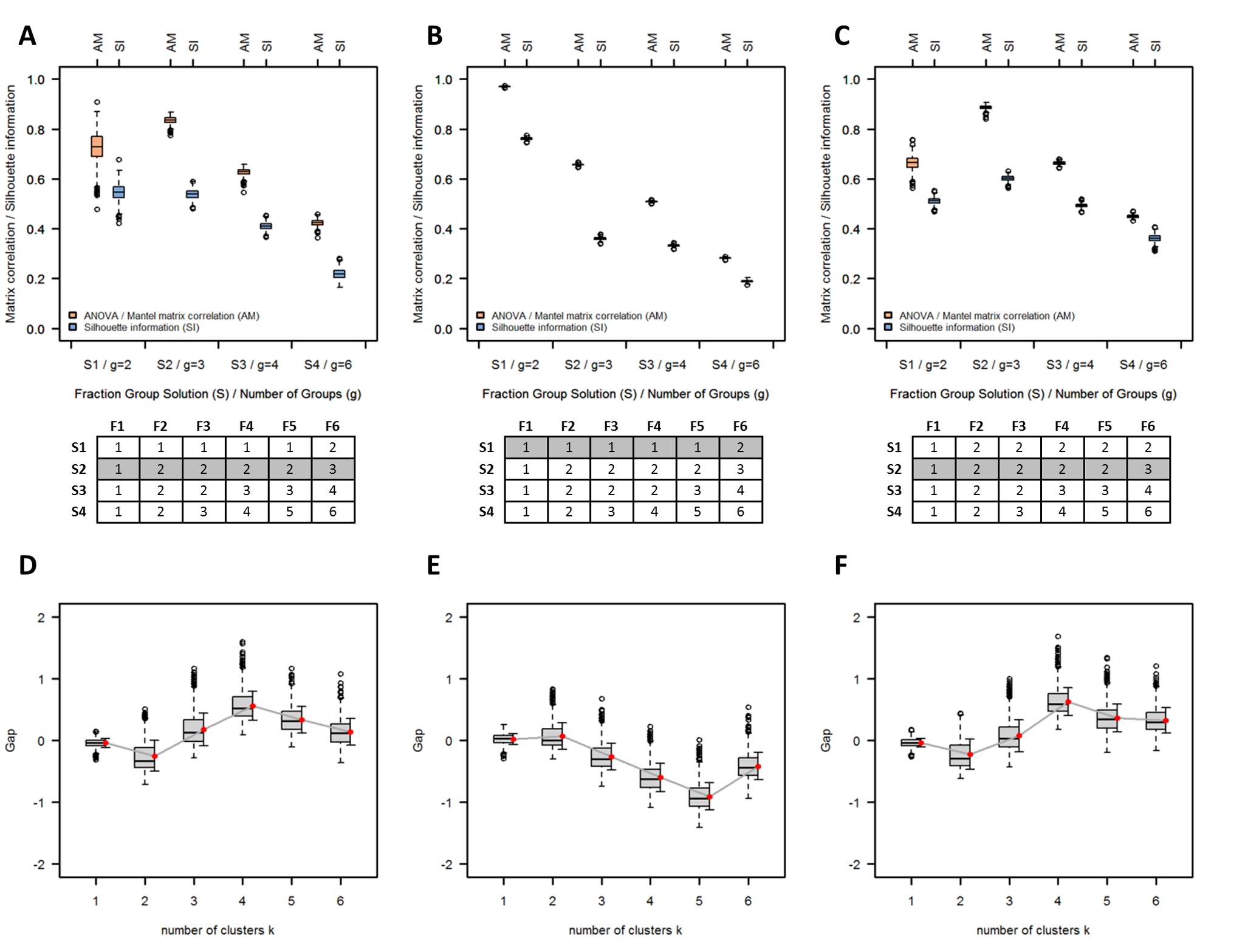


**Figure S1.** **Box plots illustrating (A-C) the silhouette information and matrix correlation of assembled fraction group solutions as well as (D-F) the gap statistics to estimate the number of sample clusters on the basis of (A, D) primary, (B, E) lipophilic and (C, F) secondary metabolite data. (A-C)** Fraction group solutions (S), assembled by sequential merging of nearest neighboring sample clusters with fractions assigned using membership majority voting, are shown below the graphs A-C with the considered best solution depicted in grey boxes. The robustness was assessed 999 times by random selection with resampling of 75% of metabolites; the resulting Euclidean distance matrices among samples and cluster solutions were compared to the expected solutions S1 - S4 by Rand index (RI), a measure of the similarity between two cluster solutions. The solution S2 (3 groups: F6, F1, and F2-F5) for primary and secondary metabolite data and S1 (2 groups: F6 and F1-F5) for lipophilic metabolite data are considered best as they explain the largest fraction of variance (≥ 70%; estimated by non-parametric ANOVA using Mantel test), and reveal the highest silhouette information, a measure of cohesion within and separation among groups, with complete agreement (RI_median_ = 1) of the expected and observed clustering for secondary and lipophilic data (data not shown), while primary metabolite data gave a RI_median_ of 0.85. (**D-E**) Box plots showing the goodness of clustering measure in dependence of the number of clusters (k) using 999 bootstrap samples. The mean values (red dots) and standard deviations are shown. The grey-colored solid line connects the mean values and depicts the gap curve. A gap maximum is reached at k = 4, 2, and 4 clusters for primary, lipophilic and secondary metabolite data, respectively. Since the first gap maximum is observable at k = 1, this suggests that the estimated clusters are less well-separated, likely as the result of the continuous distribution of compartments and their metabolite content throughout the gradient fractions (estimated using compartment-specific markers; Figure 1).
