# Supplementary material for: A Topological Map of the Compartmentalized Arabidopsis thaliana Leaf Metabolome
Source: PLoS One. 2011 Mar 15;6(3):e17806. doi: 10.1371/journal.pone.0017806 (PMC3058050; doi:10.1371/journal.pone.0017806)
Supplement: Figure S2 — (A) Heatmap and cluster distribution of selected markers representing the three resolved subcellular compartments and (B) gap curves to estimate the number of marker clusters. (DOC) [file pone.0017806.s002.doc]

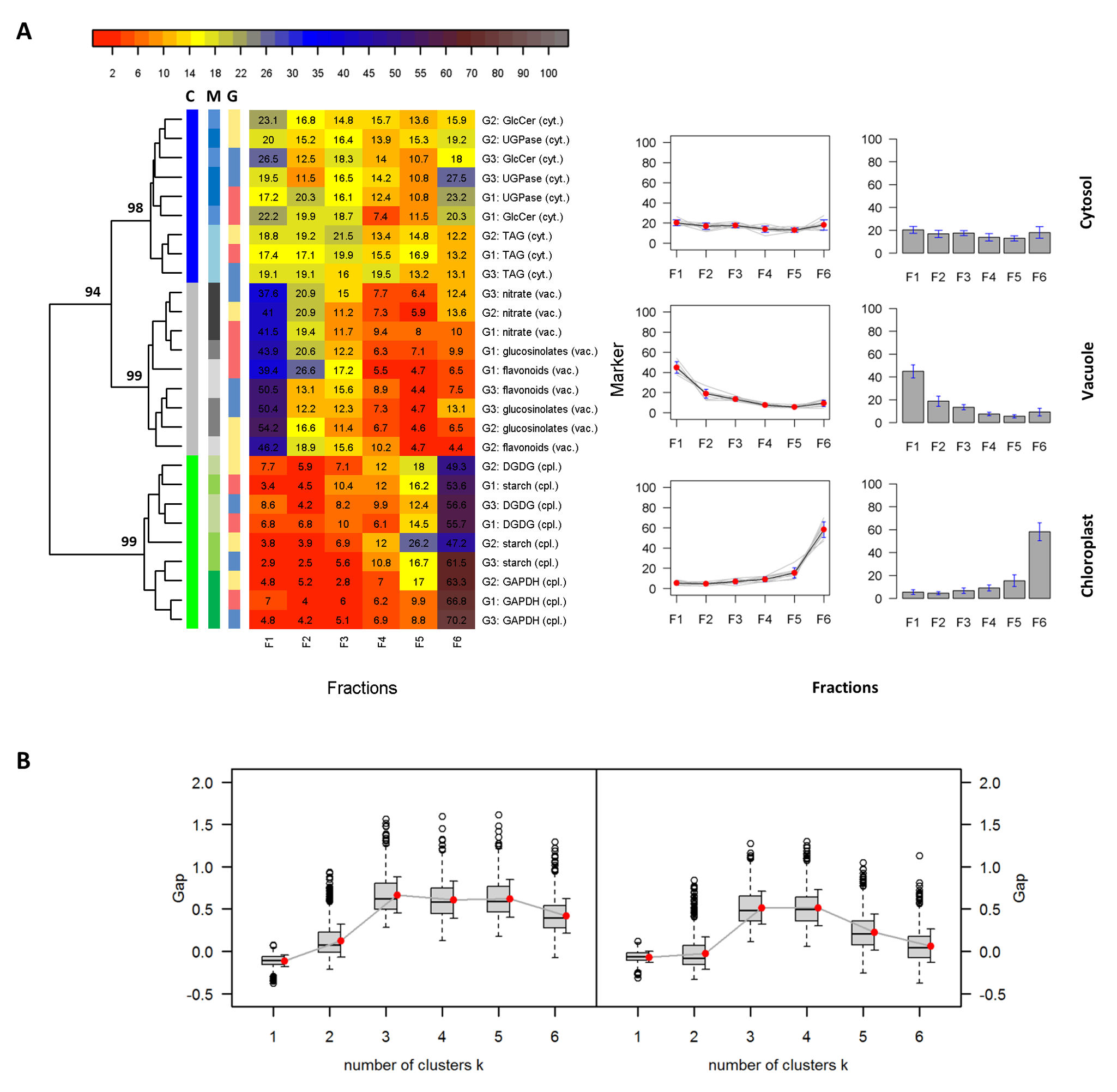


**Figure S2. (A) Heatmap and cluster distribution of selected markers representing the three resolved subcellular compartments and (B) gap curves to estimate the number of marker clusters.** **(A)** Heatmap values are depicted as percentage from total (scaled data) in each cell (Data S4) and color coded according to the top color bar. Cluster trees are drawn on the basis of Manhattan distances hierarchically clustered using average linkage clustering. The approximately unbiased *P*-value, calculated using multiscale bootstrap resampling, of the marker clusters is depicted. The left side bars reflect, using diverse color codes, the ordering of compartments (C), markers (M), and gradients (G). Whereas the compartments are clearly and consistently separated, markers, and especially gradients are essentially randomly ordered within the compartmental clusters. Line and bar plots of the average distributions including standard deviations for each compartmental cluster across the averaged gradient fractions are given on the left side. **(B)** Box plots showing the goodness of clustering measure in dependence of the number of clusters (k) using 999 bootstrap samples for all estimated marker abundances and three independent gradients. Marker abundances were clustered as described above and gap statistics estimated for **(left)** three (cytosol, vacuole, plastids) or **(right)** four compartments (including mitochondria). The mean values (red dots), including standard deviations, are connected by grey-colored solid lines (gap curve). Gap statistics estimated for three compartments (B, plot to the left) in dependence of the number of clusters supported three well-separated uniform clusters as a maximum is just reached at k = 3. Similarly, when including the mitochondrial marker citrate synthase (B, plot to the right), the optimal number of clusters estimated is k=3, even though a first maximum is already observed at k = 1, suggesting less well-separated clusters due to the distribution of the mitochondrial compartment which is in-between the plastids and the cytosol with an overlap with the cytosolic compartment (Figure 4).
