# Supplementary material for: A Topological Map of the Compartmentalized Arabidopsis thaliana Leaf Metabolome
Source: PLoS One. 2011 Mar 15;6(3):e17806. doi: 10.1371/journal.pone.0017806 (PMC3058050; doi:10.1371/journal.pone.0017806)
Supplement: Figure S3 — Venn diagrams of compartmental assignments of analytes separated according to the major compound classes (A) primary, (B) lipophilic, and (C) secondary metabolite data. (DOC) [file pone.0017806.s003.doc]

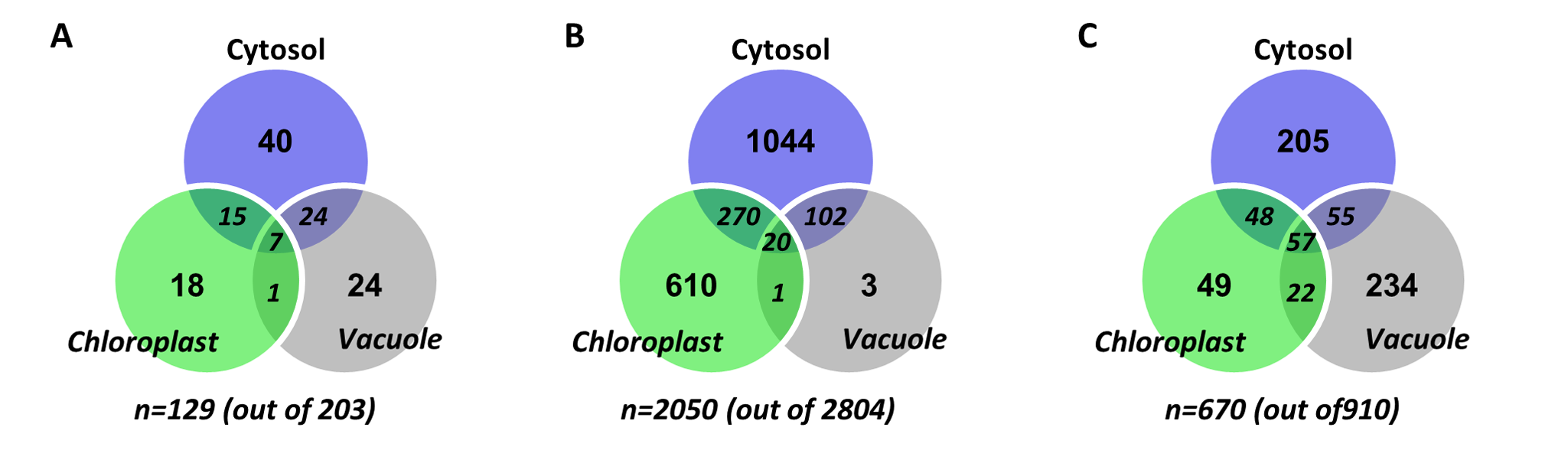


**Figure S3. Venn diagrams of compartmental assignments of analytes separated according to the major compound classes (A) primary, (B) lipophilic, and (C) secondary metabolite data.** Classification of analytes into specific (Venn) or shared between compartments (Venn overlap) is based on the best fit - estimated subcellular distributions (Data S4) derived from three independent gradients using a classification tree based assignment (Figure 6). To aid interpretation the classes ‘specific’ and ‘dominant’ were both assigned into the respective compartment (Venn value); the classes ‘enriched’, ‘shared*’, and ‘unexplained’ were ignored (see Table 1 for details). Overall, each major compound class shows variations in the portion of compartment-specific and -dominant assigned analytes. Whereas primary metabolites are mainly located in the cytosol with approximately equal fractions in the plastidic and vacuolar compartments, lipophilic compounds are primarily assigned in the cytosol and plastid. In contrast, secondary metabolic compounds are located mainly in the cytosol and vacuole and to a lesser extent in the plastids.
