# Supplementary material for: A Topological Map of the Compartmentalized Arabidopsis thaliana Leaf Metabolome
Source: PLoS One. 2011 Mar 15;6(3):e17806. doi: 10.1371/journal.pone.0017806 (PMC3058050; doi:10.1371/journal.pone.0017806)
Supplement: Figure S4 — Graphical visualization of (A, B) classification results and (C, D) gap curves based on k-medoids clustering regarding (A, C) analytes with insufficiently explained (unexplained) subcellular distributions and (B, D) all analytes. (DOC) [file pone.0017806.s004.doc]

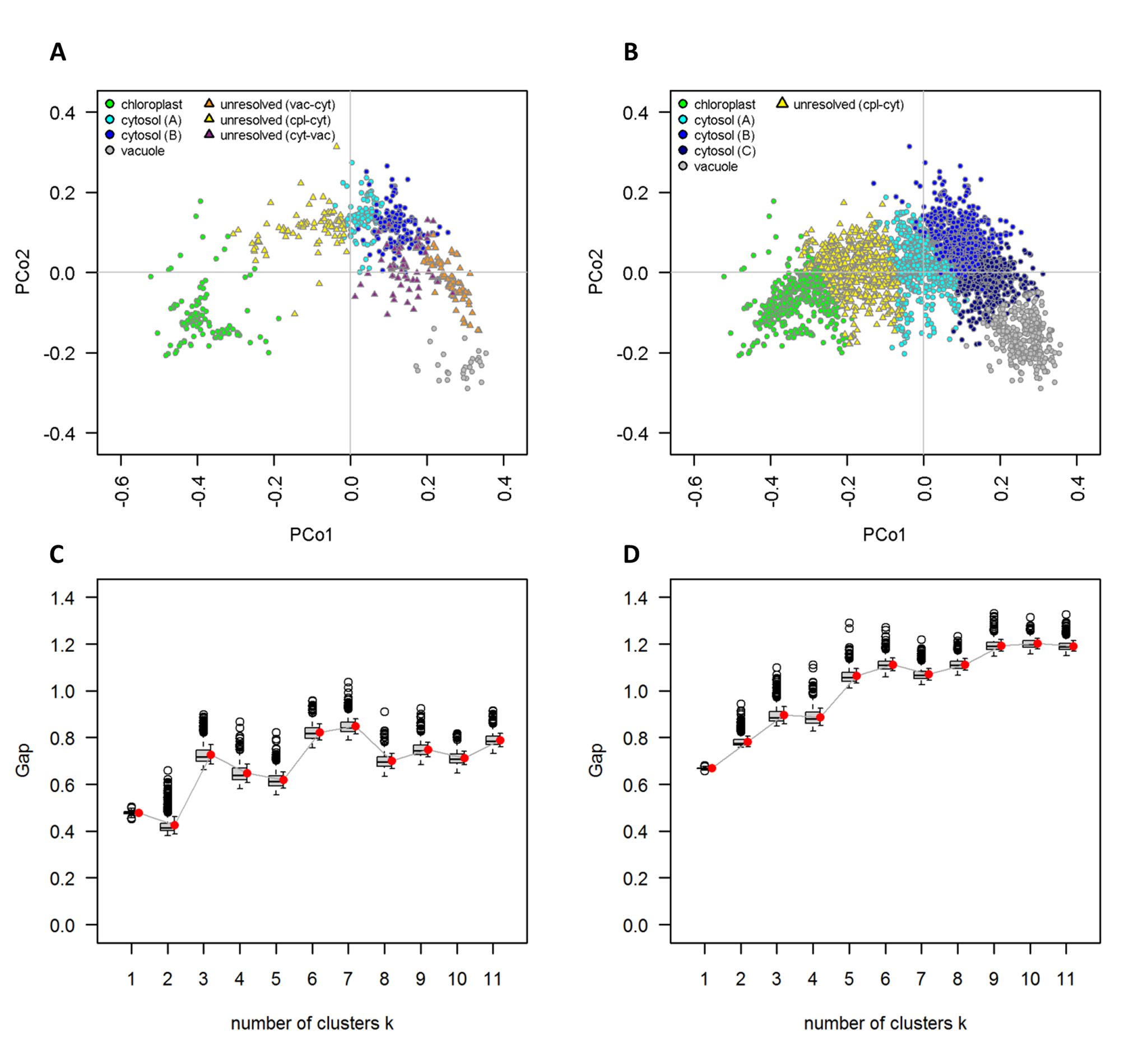


**Figure S4. Graphical visualization of (A, B) classification results and (C, D) gap curves based on k-medoids clustering regarding (A, C) analytes with insufficiently explained (unexplained) subcellular distributions and (B, D) all analytes.** **(A, B)** A topological map of the classification results visualized in principal coordinates (PCo) space on the basis of averaged Manhattan distances among analytes for the three independent gradients. Analytes assigned into classes are based on the membership of compartmental markers (Figures 1 and 4) and are color-coded according to the assignment results (Data S4). **(C, D)** Box plots showing the goodness of clustering measure in dependence of the number of clusters (k) using 999 bootstrap samples. Analytes were clustered as described above and gap statistics estimated for **(C)** analytes with unexplained distributions and **(D)** all analytes. The mean values (red dots), including standard deviations, are connected by grey-colored solid lines (gap curve). The number of clusters, initially determined by allowing only the cytosolic compartment (represented by three compartment-specific markers) to be portioned into different clusters without being assigned onto another compartment, is supported by the gap statistic showing maxima at k = 7 (6) and 6 for when considering only analytes with unexplained distribution and when considering all analytes. However, it should be noted that maxima are already observed at k=1 and k=3 when considering only analytes with unexplained distribution or k = 3 when considering all analytes. This illustrates that subclusters can be identified within the three relatively well-separated clusters, namely plastids, cytosol, and vacuole.
