# Supplementary material for: A Topological Map of the Compartmentalized Arabidopsis thaliana Leaf Metabolome
Source: PLoS One. 2011 Mar 15;6(3):e17806. doi: 10.1371/journal.pone.0017806 (PMC3058050; doi:10.1371/journal.pone.0017806)
Supplement: Table S1 — Subcellular metabolite distribution and assignment results for selected major compound classes of primary metabolic compounds. (DOC) [file pone.0017806.s005.doc]

**Table S1. Subcellular metabolite distribution and assignment results for selected major compound classes of primary metabolic compounds.**

| **Analyte Name** | | **Subcellular distribution**  **[%]** | | | **Classification tree based**  **Assignment** | | | **k-Medoids**  **Cluster** | |
| --- | --- | --- | --- | --- | --- | --- | --- | --- | --- |
|  |  | **chloroplast** | **cytosol** | **vacuole** | **explained** | **type** | **mode** | **unexplained analytes (k=7)** | **all analytes (k=6)** |
| **Amino acids and conjugates** | | | | | | | | | |
|  | Glutamine (P1.1) | 89±10 | 11±10 | 0±0 | no |  |  | cpl | cpl |
|  | Aspartate | 74±21 | 26±21 | 0±0 | no |  |  | cpl-cyt | cpl-cyt |
|  | Glutamate | 58±25 | 42±25 | 0±0 | no |  |  | cpl-cyt | cpl-cyt |
|  | Asparagine | 80±9 | 20±9 | 0±0 | no |  |  | cpl-cyt | cpl-cyt |
|  | Serine | 0±0 | 98±3 | 2±3 | no |  |  | cyt (B) | cyt (B) |
|  | Pyrrole-2-carboxylate | 0±0 | 81±18 | 19±18 | no |  |  | vac-cyt | cyt (C) |
|  | Arginine | 97±3 | 3±3 | 0±0 |  | specific | cpl |  | cpl |
|  | Proline | 67±6 | 26±12 | 7±6 |  | dominant | cpl |  | cpl-cyt |
|  | Tryptophan | 81±11 | 18±14 | 2±3 |  | dominant | cpl |  | cpl-cyt |
|  | Threonine | 3±5 | 97±5 | 0±0 |  | specific | cyt |  | cyt (B) |
|  | Alanine | 7±12 | 85±23 | 8±11 |  | dominant | cyt |  | cyt (B) |
|  | Glycine (P1.1) | 9±1 | 81±17 | 11±17 |  | dominant | cyt |  | cyt (A) |
|  | b-Alanine | 18±3 | 71±3 | 11±1 |  | dominant | cyt |  | cyt (A) |
|  | 5-Oxoproline | 16±7 | 84±7 | 0±0 |  | dominant | cyt |  | cyt (B) |
|  | Nicotinate | 0±1 | 63±5 | 37±4 |  | enriched | cyt |  | cyt (C) |
|  | Homoserine | 41±2 | 54±3 | 5±4 |  | enriched | cyt |  | cyt (A) |
|  | trans-4-Hydroxyproline | 35±7 | 63±8 | 2±4 |  | enriched | cyt |  | cyt (A) |
|  | 2-Aminobutyrate | 23±7 | 57±13 | 20±8 |  | shared* | cyt |  | cyt (A) |
|  | Valine | 18±14 | 67±27 | 15±13 |  | shared* | cyt |  | cyt (A) |
|  | Leucine | 22±14 | 67±19 | 11±6 |  | shared* | cyt |  | cyt (A) |
|  | Phenylalanine | 31±10 | 60±18 | 9±9 |  | shared* | cyt |  | cyt (A) |
|  | 4-Aminobutanoate | 9±5 | 48±11 | 43±6 |  | shared | cyt<>vac |  | cyt (C) |
|  | Isoleucine | 28±8 | 49±14 | 23±6 |  | shared | cpl<>cyt |  | cyt (A) |
|  | Lysine | 54±6 | 45±9 | 2±3 |  | shared | cpl<>cyt |  | cpl-cyt |
|  | Tyrosine | 39±11 | 44±16 | 17±6 |  | shared | cpl<>cyt |  | cyt (A) |
| **Carbohydrates and conjugates** | | | | | | | | | |
|  | Dehydroascorbate (P1.1) | 9±16 | 91±16 | 0±0 | no |  |  | cyt (B) | cyt (B) |
|  | Sucrose | 0±1 | 88±19 | 11±20 | no |  |  | cyt (B) | cyt (B) |
|  | Fructose (|Psicose) | 4±7 | 86±9 | 10±5 |  | specific | cyt |  | cyt (B) |
|  | Fructose|Sorbose | 0±0 | 92±9 | 8±9 |  | specific | cyt |  | cyt (B) |
|  | Glucose (P1.1) | 6±10 | 79±21 | 15±13 |  | dominant | cyt |  | cyt (B) |
|  | Gentiobiose | 0±0 | 82±11 | 18±11 |  | dominant | cyt |  | cyt (C) |
|  | Galactinol | 16±2 | 82±4 | 3±3 |  | dominant | cyt |  | cyt (A) |
|  | Raffinose (|1-Kestose|Inulotriose)) | 0±0 | 77±10 | 23±10 |  | dominant | cyt |  | cyt (C) |
|  | Xylose | 8±3 | 13±6 | 79±7 |  | dominant | vac |  | vac |
|  | Rhamnose | 1±1 | 15±7 | 84±7 |  | dominant | vac |  | vac |
|  | Melibiose | 19±6 | 11±10 | 69±5 |  | dominant | vac |  | cyt (C) |
|  | Trehalose | 23±2 | 64±10 | 12±8 |  | enriched | cyt |  | cyt (A) |
|  | Erythritol|Threitol | 17±3 | 20±4 | 63±7 |  | enriched | vac |  | cyt (C) |
|  | Galactono-1,4-lactone | 55±8 | 27±19 | 18±13 |  | shared* | cpl |  | cpl-cyt |
|  | Arabinose|Lyxose|Xylose | 5±5 | 34±13 | 61±12 |  | shared* | vac |  | cyt (C) |
|  | Ribose (|Ribulose)) | 3±4 | 43±7 | 54±7 |  | shared | cyt<>vac |  | cyt (C) |
|  | Fucose|Epifucose | 5±5 | 43±11 | 51±6 |  | shared | cyt<>vac |  | cyt (C) |
|  | Psicose | 6±6 | 50±28 | 44±32 |  | shared | cyt<>vac |  | cyt (C) |
|  | N-Acetylgalactosamine | 1±2 | 54±8 | 45±8 |  | shared | cyt<>vac |  | cyt (C) |
|  | N-Acetylmannosamine | 9±7 | 33±34 | 58±29 |  | shared | cyt<>vac |  | cyt (C) |
|  | Lactose (|Lactulose) | 8±4 | 23±25 | 69±22 |  | shared | cyt<>vac |  | cyt (C) |
|  | Cellobiose | 0±0 | 56±11 | 44±11 |  | shared | cyt<>vac |  | cyt (C) |
|  | Maltose (P1.2) | 0±0 | 44±17 | 56±17 |  | shared | cyt<>vac |  | cyt (C) |
|  | 2-Deoxygalactose | 49±7 | 16±27 | 36±26 |  | shared | all three |  | cyt (A) |
| **Organic acids** | | | | | | | | | |
|  | 4-Hydroxybenzoate | 5±8 | 70±20 | 25±26 | no |  |  | cyt-vac | cyt (C) |
|  | Fumarate (|Maleate) | 5±8 | 95±8 | 0±0 |  | specific | cyt |  | cyt (B) |
|  | Malate | 2±4 | 98±4 | 0±0 |  | specific | cyt |  | cyt (B) |
|  | Tetracosanoate | 5±9 | 90±14 | 5±5 |  | specific | cyt |  | cyt (B) |
|  | Octacosanoate | 7±8 | 85±7 | 8±11 |  | specific | cyt |  | cyt (B) |
|  | Lactate | 6±10 | 86±21 | 8±11 |  | dominant | cyt |  | cyt (B) |
|  | Benzoate | 15±4 | 67±11 | 18±12 |  | dominant | cyt |  | cyt (A) |
|  | Sinapate (P1.1) | 0±0 | 86±13 | 14±13 |  | dominant | cyt |  | cyt (C) |
|  | Docosanoate | 14±18 | 81±21 | 5±4 |  | dominant | cyt |  | cyt (B) |
|  | Hexacosanoate | 6±7 | 75±16 | 19±9 |  | dominant | cyt |  | cyt (C) |
|  | 2-Hydroxybutyrate | 21±11 | 68±18 | 11±9 |  | enriched | cyt |  | cyt (A) |
|  | Threonate | 64±8 | 34±10 | 2±3 |  | enriched | cpl |  | cpl-cyt |
|  | Ascorbate | 36±4 | 6±11 | 58±7 |  | enriched | vac |  | cyt (A) |
|  | 2-Oxobutanoate | 37±2 | 46±9 | 17±8 |  | shared | cpl<>cyt |  | cyt (A) |
|  | Glycerate | 27±10 | 53±19 | 20±11 |  | shared | cpl<>cyt |  | cyt (A) |
|  | Pyruvate | 19±7 | 47±12 | 34±5 |  | shared | cyt<>vac |  | cyt (A) |
| **Alcohols** | | | | | | | | | |
|  | Phytol (P1.1) | 90±6 | 9±8 | 1±2 |  | specific | cpl |  | cpl |
|  | Ethanolamine | 9±11 | 90±12 | 1±1 |  | specific | cyt |  | cyt (B) |
|  | myo-Inositol | 0±0 | 100±1 | 0±1 |  | specific | cyt |  | cyt (B) |
|  | Glycerol | 27±11 | 73±11 | 0±0 |  | dominant | cyt |  | cyt (A) |

The subcellular distributions were calculated using BFA on data from the three independent gradients and are given as mean ± SD (see Data S4 for complete list). The results of classification tree based assignments (Figure 6) are provided as type and mode with cpl = plastid, cyt = cytosol, and vac = vacuole or its overlap designated by the characters ‘<>’. Analytes with insufficiently explained subcellular distributions were clustered using k-medoids clustering with k = 7 clusters labeled according the compartment or compartmental subcluster based on the marker assignment. Clusters without a marker are named according to their intermediate averaged fraction abundances between markers, e.g. cpl – cyt, a virtual subcellular unit with fraction abundance between the cytosol and the plastids. Also, the result of k-medoids clustering of all analytes (with k=6) is provided. The subcellular distributions of analytes assigned into the cluster cpl-cyt might be partially overestimated as this cluster also encompasses the mitochondrial marker, potentially indicating metabolites shared between the mitochondria and plastids / cytosol.
