# Supplementary material for: A Topological Map of the Compartmentalized Arabidopsis thaliana Leaf Metabolome
Source: PLoS One. 2011 Mar 15;6(3):e17806. doi: 10.1371/journal.pone.0017806 (PMC3058050; doi:10.1371/journal.pone.0017806)
Supplement: Table S2 — Subcellular metabolite distribution and assignment results for selected major compound classes of lipophilic metabolic compounds. (DOC) [file pone.0017806.s006.doc]

**Table S2. Subcellular metabolite distribution and assignment results for selected major compound classes of lipophilic metabolic compounds.**

| **Analyte Name** | | **Subcellular distribution**  **[%]** | | | **Classification tree based**  **Assignment** | | | **k-Medoids**  **Cluster** | |
| --- | --- | --- | --- | --- | --- | --- | --- | --- | --- |
|  |  | **chloroplast** | **cytosol** | **vacuole** | **explained** | **type** | **mode** | **unexplained analytes (k=7)** | **all analytes (k=6)** |
| **Monogalactosyldiacylglycerol (MGDGs)** | | | | | | | | | |
|  | MGDG [OPDA/dnOPDA] (Peak: L117) | 100±0 | 0±0 | 0±0 | no |  |  | cpl | cpl |
|  | MGDG [34:5] (Peak: L1274) | 100±0 | 0±0 | 0±0 | no |  |  | cpl | cpl |
|  | MGDG [36:6] (Peak: L1402) | 100±0 | 0±0 | 0±0 | no |  |  | cpl | cpl |
|  | MGDG [18C ketol/16:3] (Peak: L72) | 100±0 | 0±0 | 0±0 |  | specific | cpl |  | cpl |
|  | MGDG [OPDA/dnOPDA] (Peak: L222) | 100±0 | 0±0 | 0±0 |  | specific | cpl |  | cpl |
|  | MGDG [OPDA/16:3] (Peak: L555) | 99±1 | 0±0 | 1±1 |  | specific | cpl |  | cpl |
|  | MGDG [18:3/dnOPDA] (Peak: L679) | 100±0 | 0±0 | 0±0 |  | specific | cpl |  | cpl |
|  | MGDG [OPDA/18:2] (Peak: L762) | 100±0 | 0±0 | 0±0 |  | specific | cpl |  | cpl |
|  | MGDG [OPDA/18:3] (Peak: L773) | 100±0 | 0±0 | 0±0 |  | specific | cpl |  | cpl |
|  | MGDG [36:5] (Peak: L1558) | 100±0 | 0±0 | 0±0 |  | specific | cpl |  | cpl |
|  | MGDG [36:4] (Peak: L1741) | 99±2 | 0±0 | 1±2 |  | specific | cpl |  | cpl |
|  | MGDG [34:2] (Peak: L1896) | 98±3 | 0±0 | 2±3 |  | specific | cpl |  | cpl |
|  | MGDG [34:1] (Peak: L2065) | 100±0 | 0±0 | 0±0 |  | specific | cpl |  | cpl |
|  | MGDG [34:6] (Peak: L1088) | 58±20 | 42±20 | 0±0 |  | shared | cpl<>cyt |  | cpl-cyt |
| **Digalactosyldiacylglycerol (DGDGs)** | | | | | | | | | |
|  | DGDG [OPDA/18:2] (Peak: L583) | 96±4 | 4±4 | 0±0 |  | specific | cpl |  | cpl |
|  | DGDG [34:6] (Peak: L905) | 96±4 | 4±4 | 0±0 |  | specific | cpl |  | cpl |
|  | DGDG [36:6] (Peak: L1233) | 100±1 | 0±0 | 0±1 |  | specific | cpl |  | cpl |
|  | DGDG [36:5] (Peak: L1293) | 92±7 | 8±7 | 0±0 |  | specific | cpl |  | cpl |
|  | DGDG [36:4] (Peak: L1533) | 91±6 | 7±6 | 2±3 |  | specific | cpl |  | cpl |
|  | DGDG [34:3] (Peak: L1593) | 93±7 | 3±6 | 4±1 |  | specific | cpl |  | cpl |
|  | DGDG [34:3] (Peak: L1594) | 93±7 | 3±6 | 4±1 |  | specific | cpl |  | cpl |
|  | DGDG [36:3] (Peak: L1812) | 98±2 | 0±0 | 2±2 |  | specific | cpl |  | cpl |
|  | DGDG [34:2] (Peak: L1658) | 80±7 | 20±7 | 0±0 |  | dominant | cpl |  | cpl-cyt |
|  | DGDG [32:0] (Peak: L1854) | 78±2 | 21±1 | 1±1 |  | dominant | cpl |  | cpl-cyt |
|  | DGDG [34:1] (Peak: L1880) | 72±7 | 26±8 | 2±1 |  | dominant | cpl |  | cpl-cyt |
|  | DGDG [34:3] (Peak: L1447) | 64±12 | 36±12 | 0±0 |  | enriched | cpl |  | cpl-cyt |
|  | DGDG [36:6] (Peak: L1164) | 52±16 | 48±16 | 0±0 |  | shared | cpl<>cyt |  | cpl-cyt |
| **Phospholipids** | | | | | | | | | |
|  | PC [34:1] (Peak: L1932) | 0±0 | 59±14 | 41±14 | no |  |  | vac-cyt | cyt (C) |
|  | PC [34:1] (Peak: L1951) | 0±0 | 69±13 | 31±13 |  | dominant | cyt |  | cyt (C) |
|  | PC [34:2] (Peak: L1690) | 0±0 | 69±13 | 31±13 |  | dominant | cyt |  | cyt (C) |
|  | PC [34:3] (Peak: L1486) | 2±3 | 83±7 | 15±8 |  | dominant | cyt |  | cyt (C) |
|  | PC [36:2] (Peak: L1962) | 3±5 | 73±15 | 24±15 |  | dominant | cyt |  | cyt (C) |
|  | PC [36:4] (Peak: L1541) | 0±0 | 75±21 | 25±21 |  | dominant | cyt |  | cyt (C) |
|  | PC [36:5] (Peak: L1321) | 10±18 | 78±16 | 11±9 |  | dominant | cyt |  | cyt (A) |
|  | PC [36:5] (Peak: L1308) | 0±0 | 80±11 | 20±11 |  | dominant | cyt |  | cyt (C) |
|  | PC [36:6] (Peak: L1192) | 15±16 | 79±12 | 6±7 |  | dominant | cyt |  | cyt (A) |
|  | PC [36:3] (Peak: L1750) | 0±0 | 57±23 | 43±23 |  | shared | cyt<>vac |  | cyt (C) |
|  | PE [34:2] (Peak: L1786) | 0±0 | 13±18 | 87±18 | no |  |  | vac-cyt | vac |
|  | PE [34:3] (Peak: L1572) | 0±0 | 31±27 | 69±27 | no |  |  | vac-cyt | vac |
|  | PE [36:4] (Peak: L1591) | 8±12 | 51±13 | 41±19 |  | shared | cyt<>vac |  | cyt (C) |
|  | PE [36:5] (Peak: L1377) | 0±0 | 36±19 | 64±19 |  | shared | cyt<>vac |  | vac |
|  | PG [34:1] (Peak: L1616) | 51±14 | 49±14 | 0±0 | no |  |  | cpl-cyt | cpl-cyt |
|  | PG [34:3] (Peak: L1237) | 85±12 | 15±13 | 1±1 |  | dominant | cpl |  | cpl-cyt |
|  | PG [34:2] (Peak: L1401) | 63±9 | 31±19 | 6±10 |  | enriched | cpl |  | cpl-cyt |
| **Sphingolipids** | | | | | | | | | |
|  | Cer [t18:1/h24:0] (Peak: L2439) | 0±0 | 87±2 | 13±2 |  | dominant | cyt |  | cyt (C) |
|  | Cer [t18:1/c22:0] (Peak: L2133) | 0±0 | 72±31 | 28±31 |  | shared | cyt<>vac |  | cyt (C) |
|  | GlcCer [t18:1/h24:0] (Peak: L2419) | 0±0 | 97±6 | 3±6 |  | specific | cyt |  | cyt (B) |
|  | GlcCer [t18:1/h24:1] (Peak: L2156) | 3±4 | 90±12 | 6±8 |  | specific | cyt |  | cyt (B) |
|  | GlcCer [d18:1/h16:0] (Peak: L1483) | 0±0 | 67±6 | 33±6 |  | dominant | cyt |  | cyt (C) |
|  | GlcCer [d18:1/h24:1] (Peak: L2397) | 14±4 | 70±15 | 16±11 |  | dominant | cyt |  | cyt (A) |
|  | GlcCer [t18:1/h24:0] (Peak: L2433) | 1±1 | 85±6 | 14±5 |  | dominant | cyt |  | cyt (C) |
|  | GlcCer [t18:1/h26:1] (Peak: L2420) | 7±5 | 81±9 | 12±4 |  | dominant | cyt |  | cyt (A) |
|  | GlcCer [t18:1/h26:0] (Peak: L2509) | 8±3 | 65±8 | 27±6 |  | enriched | cyt |  | cyt (C) |
|  | GlcCer [t18:1/h16:0] (Peak: L1265) | 4±3 | 63±12 | 33±9 |  | enriched | cyt |  | cyt (C) |
| **Triacylglycerides** | | | | | | | | | |
|  | TG [51:3] (Peak: L2600) | 0±0 | 100±0 | 0±0 | no |  |  | cyt (B) | cyt (B) |
|  | TG [52:1] (Peak: L2690) | 0±0 | 100±0 | 0±0 | no |  |  | cyt (B) | cyt (B) |
|  | TG [54:2] (Peak: L2691) | 0±0 | 100±0 | 0±0 | no |  |  | cyt (B) | cyt (B) |
|  | TG [51:3] (Peak: L2600) | 0±0 | 100±0 | 0±0 | no |  |  | cyt (B) | cyt (B) |
|  | TG [52:4] (Peak: L2565) | 0±0 | 99±2 | 1±2 |  | specific | cyt |  | cyt (B) |
|  | TG [48:2] (Peak: L2566) | 0±0 | 100±0 | 0±0 |  | specific | cyt |  | cyt (B) |
|  | TG [54:5] (Peak: L2568) | 0±0 | 100±0 | 0±0 |  | specific | cyt |  | cyt (B) |
|  | TG [50:3] (Peak: L2569) | 3±3 | 97±3 | 0±0 |  | specific | cyt |  | cyt (B) |
|  | TG [49:2] (Peak: L2598) | 0±0 | 100±0 | 0±0 |  | specific | cyt |  | cyt (B) |
|  | TG [48:1] (Peak: L2604) | 0±0 | 100±0 | 0±0 |  | specific | cyt |  | cyt (B) |
|  | TG [52:3] (Peak: L2617) | 0±0 | 100±0 | 0±0 |  | specific | cyt |  | cyt (B) |
|  | TG [50:1] (Peak: L2606) | 0±0 | 95±9 | 5±9 |  | specific | cyt |  | cyt (B) |
|  | TG [54:4] (Peak: L2610) | 0±0 | 100±0 | 0±0 |  | specific | cyt |  | cyt (B) |
|  | TG [49:1] (Peak: L2622) | 0±0 | 100±0 | 0±0 |  | specific | cyt |  | cyt (B) |
|  | TG [51:2] (Peak: L2624) | 0±0 | 99±1 | 1±1 |  | specific | cyt |  | cyt (B) |
|  | TG [48:0] (Peak: L2643) | 0±0 | 100±1 | 0±1 |  | specific | cyt |  | cyt (B) |
|  | TG [51:1] (Peak: L2683) | 0±0 | 100±0 | 0±0 |  | specific | cyt |  | cyt (B) |
|  | TG [49:0] (Peak: L2681) | 0±0 | 100±0 | 0±0 |  | specific | cyt |  | cyt (B) |
|  | TG [53:2] (Peak: L2684) | 0±0 | 100±0 | 0±0 |  | specific | cyt |  | cyt (B) |
|  | TG [50:0] (Peak: L2687) | 0±0 | 97±2 | 3±2 |  | specific | cyt |  | cyt (B) |
|  | TG [51:0] (Peak: L2707) | 0±0 | 98±1 | 2±1 |  | specific | cyt |  | cyt (B) |
|  | TG [53:1] (Peak: L2708) | 0±0 | 100±0 | 0±0 |  | specific | cyt |  | cyt (B) |
|  | TG [54:1] (Peak: L2731) | 0±0 | 99±1 | 1±1 |  | specific | cyt |  | cyt (B) |
|  | TG [52:0] (Peak: L2730) | 0±0 | 98±2 | 2±2 |  | specific | cyt |  | cyt (B) |
|  | TG [54:0] (Peak: L2738) | 0±0 | 100±0 | 0±0 |  | specific | cyt |  | cyt (B) |
|  | TG [56:1] (Peak: L2740) | 0±0 | 100±0 | 0±0 |  | specific | cyt |  | cyt (B) |
|  | TG [57:1] (Peak: L2762) | 0±0 | 100±1 | 0±1 |  | specific | cyt |  | cyt (B) |

The subcellular distributions were calculated using BFA on data from the three independent gradients and are given as mean ± SD (see Data S4 for complete list). The results of classification tree based assignments (Figure 6) are provided as type and mode with cpl = plastid, cyt = cytosol, and vac = vacuole or its overlap designated by the characters ‘<>’. Analytes with insufficiently explained subcellular distributions were clustered using k-medoids clustering with k = 7 clusters labeled according the compartment or compartmental subcluster based on the marker assignment. Clusters without a marker are named according to their intermediate averaged fraction abundances between markers, e.g. cpl – cyt, a virtual subcellular unit with fraction abundance between the cytosol and the plastids. Also, the result of k-medoids clustering of all analytes (with k=6) is provided. The subcellular distributions of analytes assigned into the cluster cpl-cyt might be partially overestimated as this cluster also encompasses the mitochondrial marker, potentially indicating metabolites shared between the mitochondria and plastids / cytosol.
