# Supplementary material for: A Topological Map of the Compartmentalized Arabidopsis thaliana Leaf Metabolome
Source: PLoS One. 2011 Mar 15;6(3):e17806. doi: 10.1371/journal.pone.0017806 (PMC3058050; doi:10.1371/journal.pone.0017806)
Supplement: Table S3 — Subcellular metabolite distribution and assignment results for selected major compound classes of secondary metabolic compounds. (DOC) [file pone.0017806.s007.doc]

**Table S3. Subcellular metabolite distribution and assignment results for selected major compound classes of secondary metabolic compounds.**

| **Analyte Name** | | **Subcellular distribution**  **[%]** | | | **Classification tree based**  **Assignment** | | | **k-Medoids**  **Cluster** | |
| --- | --- | --- | --- | --- | --- | --- | --- | --- | --- |
|  |  | **chloroplast** | **cytosol** | **vacuole** | **explained** | **type** | **mode** | **unexplained analytes (k=7)** | **all analytes (k=6)** |
| **Glucosinolates** | | | | | | | | | |
|  | 5-Methylsulfinylpentyl glucosinolate (Peak: S16) | 0±0 | 0±0 | 100±0 | no |  |  | vac | vac |
|  | 7-(Methylsulfinyl)heptyl glucosinolate (Peak: S33) | 0±0 | 0±0 | 100±0 | no |  |  | vac | vac |
|  | 3-Indolylmethyl glucosinolate (Peak: S44) | 2±3 | 2±3 | 96±7 |  | specific | vac |  | vac |
|  | 3-Indolylmethyldesulfo glucosinolate (Peak: S45; fragment of S44) | 1±1 | 2±3 | 98±4 |  | specific | vac |  | vac |
|  | 3-Indolylmethyl-thiohydroximate (Peak: S42; fragment of S44) | 3±4 | 1±1 | 96±6 |  | specific | vac |  | vac |
|  | 8-Methylsulfinyloctyl glucosinolate (M+H) (Peak: S60) | 0±1 | 0±0 | 100±1 |  | specific | vac |  | vac |
|  | 8-Methylsulfinyloctyl glucosinolate (M+NH4) (Peak: S62) | 0±0 | 0±0 | 100±0 |  | specific | vac |  | vac |
|  | 4-Benzoyloxybutyl glucosinolate (Peak: S56) | 1±2 | 0±0 | 99±2 |  | specific | vac |  | vac |
|  | 1-Methoxy-3-indolylmethylglucosinolate (Peak: S123) | 2±2 | 5±8 | 94±9 |  | specific | vac |  | vac |
|  | 5-Methylsufinylpentyl nitrile (Peak: S21) | 0±0 | 80±18 | 20±18 |  | dominant | cyt |  | cyt (C) |
|  | 4-Methylsulfinylbutyl glucosinolate (Peak: S11) | 1±1 | 11±19 | 88±19 |  | dominant | vac |  | vac |
| **Flavonoids** | | | | | | | | | |
|  | Kaempferol 3-O-[2''-O-(rhamnosyl) glucoside] 7-O-rhamnoside (Peak: S146) | 0±0 | 4±4 | 96±4 |  | specific | vac |  | vac |
|  | Kaempferol 3-O-[2''-O-(rhamnosyl) glucoside] 7-O-rhamnoside (Peak: S135; fragment of S146) | 0±0 | 0±0 | 100±0 |  | specific | vac |  | vac |
|  | Kaempferol 3-O-[2''-O-(rhamnosyl) glucoside] 7-O-rhamnoside (Peak: S140; fragment of S146) | 0±0 | 2±4 | 98±4 |  | specific | vac |  | vac |
|  | Kaempferol 3-glucoside-7-rhamnoside (Peak: S186) | 0±0 | 4±5 | 96±5 |  | specific | vac |  | vac |
|  | Kaempferol 3-glucoside-7-rhamnoside (Peak: S179; fragment of S186) | 0±0 | 4±5 | 96±5 |  | specific | vac |  | vac |
|  | Kaempferol 3-glucoside-7-rhamnoside (Peak: S181; fragment of S186) | 0±0 | 8±7 | 92±7 |  | specific | vac |  | vac |
|  | Kaempferol 3,7-di-rhamnoside (Peak: S253) | 0±0 | 0±0 | 100±0 |  | specific | vac |  | vac |
|  | Quercetin 3-O-[2''-O-rhamnosyl)glucoside] 7-O-rhamnoside (Peak: S129) | 0±0 | 28±11 | 72±11 |  | dominant | vac |  | vac |
|  | Kaempferol 3,7-di-rhamnoside (Peak: S220) | 0±0 | 12±6 | 88±6 |  | dominant | vac |  | vac |
|  | Kaempferol 3,7-di-rhamnoside (Peak: S224; fragment of S220) | 0±0 | 19±10 | 81±10 |  | dominant | vac |  | vac |
|  | Kaempferol 3,7-di-rhamnoside (Peak: S230; fragment of S220) | 0±0 | 26±10 | 74±10 |  | dominant | vac |  | vac |
|  | Quercetin 3-O-glucoside-7-O-rhamnoside (Peak: S159) | 0±0 | 34±7 | 66±7 |  | enriched | vac |  | vac |
| **Sinapyl-conjugates (+)** | | | | | | | | | |
|  | Dimer-2-O-Sinapoylmalate (Peak: S79; dimer of S70) | 0±0 | 0±0 | 100±0 |  | specific | vac |  | vac |
|  | Dimer-2-O-Sinapoylmalate (Peak: S108; dimer of S115) | 0±0 | 0±0 | 100±0 |  | specific | vac |  | vac |
|  | Sinapoyl-(S)-malate (Peak: S101; proton adduct of S70) | 0±0 | 10±6 | 90±6 |  | specific | vac |  | vac |
|  | Sinapoyl-(S)-malate (Peak: S115) | 0±0 | 3±6 | 97±6 |  | specific | vac |  | vac |
|  | Sinapoyl-(S)-malate (Peak: S70) | 0±0 | 11±3 | 89±3 |  | dominant | vac |  | vac |
|  | 1-O-Sinapoyl-beta-D-glucose (Peak: S131) | 0±0 | 12±10 | 88±10 |  | dominant | vac |  | vac |

The subcellular distributions were calculated using BFA on data from the three independent gradients and are given as mean ± SD (see Data S4 for complete list). The results of classification tree based assignments (Figure 6) are provided as type and mode with cpl = plastid, cyt = cytosol, and vac = vacuole or its overlap designated by the characters ‘<>’. Analytes with insufficiently explained subcellular distributions were clustered using k-medoids clustering with k = 7 clusters labeled according the compartment or compartmental subcluster based on the marker assignment. Clusters without a marker are named according to their intermediate averaged fraction abundances between markers, e.g. cpl – cyt, a virtual subcellular unit with fraction abundance between the cytosol and the plastids. Also, the result of k-medoids clustering of all analytes (with k=6) is provided. The subcellular distributions of analytes assigned into the cluster cpl-cyt might be partially overestimated as this cluster also encompasses the mitochondrial marker, potentially indicating metabolites shared between the mitochondria and plastids / cytosol.
