# Supplementary material for: A Topological Map of the Compartmentalized Arabidopsis thaliana Leaf Metabolome
Source: PLoS One. 2011 Mar 15;6(3):e17806. doi: 10.1371/journal.pone.0017806 (PMC3058050; doi:10.1371/journal.pone.0017806)
Supplement: Text S1 — Supplemental extended methods and detailed supplemental data description. Further details of non-aqueous fractionation, mass-spectrometry based metabolome analyses as well as data and statistical analyses are provided. (PDF) [file pone.0017806.s008.pdf]

A topological map of the compartmentalized *Arabidopsis thaliana* leaf metabolome

- Supporting Information -

Stephan Krueger[1,*], Patrick Giavalisco[2,*], Leonard Krall[2], Marie-Caroline Steinhauser[2], Dirk Büssis[3], Bjoern Usadel[2], Ulf-Ingo Flügge[1], Alisdair R. Fernie[2], Lothar Willmitzer[2], and Dirk Steinhauser[2,*,†]

[1]Botanical Institute, University of Cologne, Zuelpicherstrasse 47b, 50674 Cologne, Germany

[2]Max Planck Institute of Molecular Plant Physiology, Am Mühlenberg 1, 14476 Potsdam-Golm, Germany

[3]GABI Managing Office, c/o Max Planck Institute of Molecular Plant Physiology, Am Mühlenberg 1, 14476 Potsdam-Golm, Germany

* These authors contributed equally to this work.

† Corresponding author.

Corresponding author:

Dirk Steinhauser

Max Planck Institute of Molecular Plant Physiology,

Am Mühlenberg 1, 14476 Potsdam-Golm, Germany

Email: steinhauser@mpimp-golm.mpg.de

Tel: (49)331-5678218

Fax: (49)331-5678236

Supporting Materials and Methods

Materials

Organic and inorganic chemicals were purchased, if not otherwise stated, in analytical grade from Sigma-Aldrich (Taufkirchen, Germany) and Merck (Darmstadt, Germany), respectively. Enzymes and $NADP^+$ used for metabolite or enzyme assays were purchased from Roche (Roche, Mannheim, Germany).

Non-aqueous fractionation

4 g of frozen leaf material was homogenized using a ball mill and then freeze-dried in a pre-cooled lyophilizer. The dried powder was resuspended in 20 mL 66:34 (v/v) tetrachlorethylene (TCE) / heptane (C_7H_{16}/C_2CL_4, density ρ = 1.3 g cm^{-3}) and ultrasonicated on ice for 120 s with 6 x 10 cycle, 65% power (Sonoplus HD 200, MS 73/D, Bandolin, Berlin, Germany). The suspension was poured through a nylon sieve (20 µm pore size) and diluted threefold with heptane. After centrifugation (4°C; 10 min; 3,200 g) the pellet was resuspended in 5 mL 66:34 (v/v) TCE/heptane and 50 µL aliquots (F0 = total extract, non-fractionated sample material) were collected and dried in a speed vac at room temperature (RT).

Separation of cellular compartments was achieved with a linear density gradient (30 mL, ρ = 1.43 – 1.62 g cm^{-3}) after 50 min centrifugation at 5,000 g and 4°C. Six fractions (F6 (4.5 mL empty loading volume including the first fraction that contained material) – F1, each 5 mL) were taken from the top (F6, lowest density) of the gradient, centrifuged (4°C; 10 min; 3,200 g) after addition of 15 mL heptane, and the pellet was then resuspended in 10 mL heptane. For each fraction, ten 1 mL aliquots were centrifuged (4°C; 10 min; 14,000 g) and the remaining pellets dried in a speed vac at RT. The dried aliquots were stored in a silica-gel containing desiccator at RT in the dark.

For all further analysis, dried aliquots were extracted in the respective buffer or solvent by strong vortexing or shaking in a pre-cooled Retsch mill (1 min, f = 25 Hz; Retsch MM200, Retsch, Haan, Germany). A total of three gradients from independent biological plant material were made.

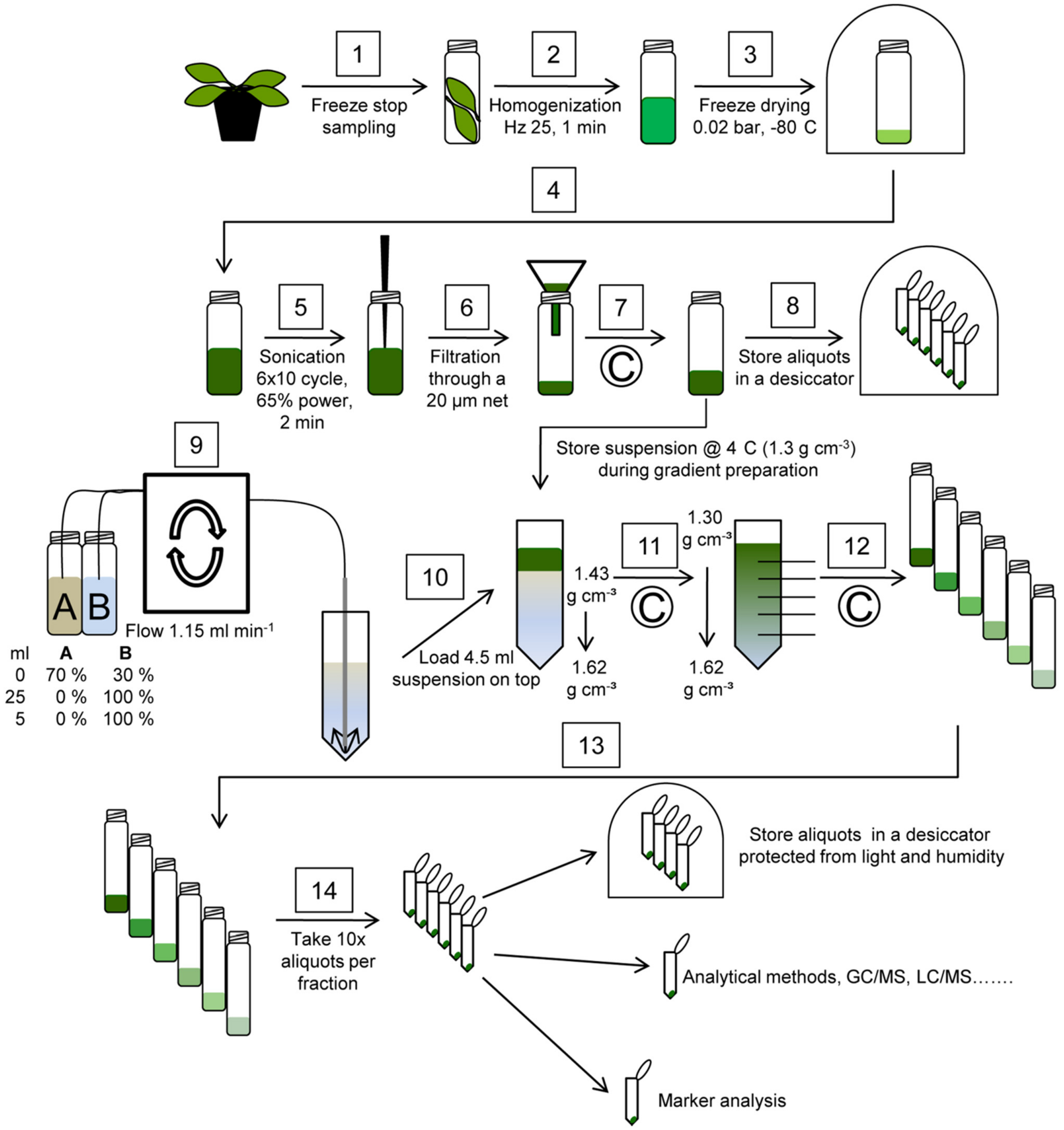

Workflow:

C_2Cl_4 and C_7H_{16} is dried and stored over molecule sieve beads (4 Å) for two days before gradient preparation.

1| Sample 4-8 gram of leaf material into aluminum foil, pre-cooled in liquid nitrogen.

2| For homogenization, place approximately 2 g of leaf material into the 25 mL grinding jar made of hardened steel (pre-cooled in liquid nitrogen). Add the pre-cooled steel ball and homogenize in ball mill for 1.5 min at 25 Hz. Frozen homogenate is placed into pre-cooled 50 mL tubes (3 gram per tube) and can be stored at -80°C up to 3 months. At this point the exact mass of the sample has to be determined.

3| Freeze dry the homogenate, open the 50 mL Falcon tubes and cover them with Kimwipes tissue, fixed with an elastic band. Place the prepared tubes into the lyophilizer at 0.02 bar and -80°C for up to 5 days. Dried material can be stored in a desiccator protected from light and humidity for up to 6 months.

4| Resuspend the dry leaf powder in 20 ml C_2Cl_4/C_7H_{16} mixture 66:34 (v/v); density = 1.3 g cm^{-3}.

5| Ultrasonicate the suspension for a total of 120 s, with 6 x 10 cycle, 65% power. To avoid over heating during ultrasonifcation keep the sample at 4°C (ice water).

6| Pour the suspension through nylon sieve (20 µm pore size) and wash the net with 3 x 10 volumes of heptane. Collect the flow through in a 50 ml Falcon tube.

7| Centrifuge 10 min at 3,200 g and 4°C, discard supernatant and resuspend pellet in 5 ml C_2Cl_4/C_7H_{16} mixture 66:34 (v/v); density ρ = 1.3 g cm^{-3}.

8| Transfer 10 x 50 µl from the suspension into 2 ml Eppendorf tubes and dry in a vacuum concentrator without heating. Store the aliquots, indicated as total extracts (F0) in a dessicator.

9| Fill tube A with C_2Cl_4/C_7H_{16} 66:34 (v/v) and tube B with C_2Cl_4. Fix needle slightly over the bottom of the 38 mL polyallomer centrifugation tube and program the peristaltic gradient pump using following settings:

Flow rate: 1.15 mL min^{-1}

mL	Solvent A C_2Cl_4/C_7H_{16} 66:34 (v/v)	Solvent B C_2Cl_4	Density ρ [g cm^{-3}]
0	70%	30%	1.43
25	0%	100%	1.62
5	0%	100%	1.63

10| Take out the needle from the gradient and load 4.5 mL suspension carefully on top using a Pasteur pipette.

11| Centrifuge for 50 min at 5,000 g and 4°C (use swing out rotor).

12| Take at least 6 gradient fractions and transfer into new 50 mL tubes. Add 3 volumes of heptane and centrifuge at 3,200 g for 10 min.

13| Resuspend the pellet in 10 mL of heptan.

14| Transfer 10 x 1 mL from the fractionated suspension into 2 mL Eppendorf tubes, centrifuge (4°C; 10 min; 14,000 g) and dry pellet in a vacuum concentrator without heating.

Metabolite profiling: Sample preparation

GC/MS samples: For GC/MS based metabolite profiling, dried fraction aliquots were extracted with 600 µL of a cold 10:3:1 (v/v/v) methanol:chloroform:water (MCW) solution supplemented with 0.1 µg mL^{-1} of U-^{13}C-sorbitol as internal standard. The mixtures were shaken for 1 min in a pre-cooled Retsch mill and further extracted for 20 min by shaking at 4°C. Extracts were centrifuged (4°C; 2 min; 10,000 g) and two aliquots (100 µL, 150 µL)

were concentrated to dryness. Extract derivatization was performed according to Krall et al. [1].

LC/MS samples: For the extraction of secondary and lipophilic metabolites, dried fraction aliquots were resuspended and homogenized in 1 mL pre-cooled 2.5:1:1 (v/v/v) MCW solution by vortexing. The samples were incubated for 10 min at 4°C on an orbital shaker, followed by ultrasonication in a bath-type sonicator for 10 min at RT. Finally, the unsoluble plant material was pelleted by centrifugation (5 min; 14,000 g) and the supernatant transferred to a fresh 2 mL tube. To separate the organic from the aqueous phase, 300 µL ddH_20 and 300 µL chloroform were added to the supernatant, vortexed, and centrifuged (2 min; 14,000 g). Subsequently, 1 mL of the upper, aqueous phase and 350 µL of the lower, organic phase were collected for secondary metabolite and lipid analyses, respectively. All samples were concentrated to complete dryness in a speed vac at RT. The dried samples were either resuspended in 100 µL ddH_20 (secondary metabolites) or in 100 µL 50:20:25 (v/v/v) isopropanol/hexane/water solution (lipophilic metabolites) or stored at -80°C until use.

Extraction and derivatization of individual soluble thiols (cystein, γ-glutamylcysteine, glutathione) were performed as described [2].

Metabolite profiling: Chromatography and mass spectrometry

GC/MS: Derivatized samples for GC/MS based metabolomics were analyzed in splitless mode on an Agilent 6890 gas chromatograph (Agilent, Santa Clara, CA, USA) coupled to a Leco Pegasus II TOF mass spectrometer (Leco, St. Joseph, MI, USA) with data acquisition in the mass range m/z of 85-750 as described [1].

LC/MS: UPLC separation of secondary metabolites, lipids, and soluble thiols were performed on a Waters Acquity UPLC system (Waters, Mildford, MA, USA) operated with a 400 µL min^{-1} flow rate of the mobile phase.

LC/MS – secondary metabolites: For secondary metabolites, 2 µL of sample were injected onto a HSS T3 C_{18} reversed phase column (100 x 2.1 mm I.D, 1.8 µm particle size, Waters) at 40°C. The mobile phases consisted of 0.1% (v/v) formic acid in water (solvent A) and 0.1% (v/v) formic acid in acetonitrile (solvent B). The sample was separated using the following gradient profile: After an 1 min isocratic run at 99% A, a 12 min linear gradient was applied to 65% A. This was immediately followed by a 1.5 min gradient to 30% A, and a 1 min gradient to 1% A. Finally, a 1.5 min isocratic period at 1% A was conducted. The column was then re-equilibrated for 2.5 min with 99% A before the next sample was injected.

LC/MS – lipophilic metabolites: For lipophilic compounds, 2 µL of sample were injected onto a BEH C_8 reversed phase column (100 x 2.1 mm I.D., 1.7 µm particle size, Waters) at 60°C. The mobile phases consisted of water with 1% (v/v) 1M NH_4Acetate, 0.1% (v/v) acetic acid (solvent A) and 7:3 (v/v) acetonitrile/isopropanol solution containing 1% (v/v) 1M NH_4Acetate, 0.1% (v/v) acetic acid (solvent B). The gradient profile was as follows: After 1 min of isocratic run at 45% A, a linear 3 min linear gradient was applied to 25% A. This was immediately followed by an 8 min gradient to 11% A, and a 3 min gradient to 0% A. The flow was held for 2 minutes at 0% A to clean the column before stepping back to 45% A within 10 seconds. The column was then re-equilibrated for 4 min with 45% A before the next sample was injected.

LC/MS – soluble thiols: For soluble, derivatized thiols, 2 µL of sample were injected onto a BEH C_{18} reversed phase column (100 x 2.1 mm I.D., 1.8 µm particle size, Waters) at 40°C. The mobile phases consisted of 0.1% (v/v) formic acid in water (solvent A) and 0.1% (v/v) formic acid in acetonitrile (solvent B). The gradient was: 0 - 1 min isocratic 95% A, 1 - 6.5 min linear gradient from 95% to 65% A, 6.5 - 10 min linear gradient from 65% to 20% A, 10 - 10.5 min linear gradient 20% to 1% A, 10.5 - 12 min isocratic 1% A, 12 - 12.5 min linear gradient from 1% to 95% A, 12.5 - 15 min isocratic 95% A.

LC/MS – mass spectrometry: For mass spectrometry based analyses of lipophilic and secondary metabolites, the UPLC was connected to an Exactive Orbitrap (Thermo Fisher Scientific, Bremen, Germany) via a heated electro spray source (Thermo). Positive ion mode mass spectra were recorded using full scan mode in the mass range m/z of 100-1500 from 0 - 19 min of the UPLC gradient. The resolution was set to 25,000 and the maximum loading time for the ICR cell was set to 100 milliseconds. The sheath gas was set to 60 L h^{-1}, while the auxiliary gas was set to 35 L h^{-1}. The transfer capillary temperature was held at 150°C while the heater temperature was set to 300°C. The spray voltage was fixed at 3 kV, while the capillary and the skimmer voltage were set to 25 V and 15 V, respectively.

A Fourier Transform Ion Cyclotron Resonance Mass Spectrometer (LTQ FT-ICR-MS, Thermo) was coupled to the UPLC for analyses of derivatized soluble thiols. Full scan mass spectra in positive ionization mode were recorded in the mass range of m/z 200-600 with a resolution of 50,000 from 0 - 13 min of the UPLC gradient. The sheath gas was set to a value of 50 L h^{-1}. The ion source was set to a voltage of 3.5 kV, while the voltage of the transfer capillary was set to 25 V and a temperature of 200°C. The maximal scan time for the ICR cell was set to 250 ms.

MS data analyses: Data pre-processing

GC/MS data: All GC/MS chromatograms were processed using Leco ChromaTOF software (version 3.25) according to Krall et al. [1]. Compound identification and mass spectral alignments were performed with the msMatch algorithm (Krall et al., in prep.) using a reference library of authentic standards (annotated reference metabolites) and manually curated *Arabidopsis* compounds of unknown chemical structure (unknowns), both measured on the employed GC/MS system (Krall et al., in prep.). The msMatch alignments of the two processing methods were averaged prior further data analyses.

LC/MS data: For high-resolution mass spectrometry, peak picking and spectral alignments were performed using the RefinerMS software (GeneData Version 5.3.7, Basel, Swizerland) or by a targeted manual approach (soluble thiols). Molecular masses, retention times and associated peak intensities were extracted from the raw data using the Expressionist Software (GeneData). Mass and retention time lists were used for various database searches, employing the in-house developed database GoBioSpace (Hummel et al., unpublished), with implemented algorithms for formula parsing and isotopic correct mass calculation. The database search criteria were set to 2 ppm and only chemical formulas containing the elements C, H, N, O, P, or S were allowed for the final results. Secondary metabolites were searched against the KEGG [3] and the KNApSAcK [4] databases, while lipophilic metabolites were searched against an in-house compiled lipid database (Giavalisco et al., submitted). All other spectral manipulations and peak extractions were performed using Xcalibur (Version 2.06, Thermo Fisher Scientific).

MS data analyses: Data post-processing

GC/MS data: The aligned and averaged GC/MS data (18 fractions and 2 total extracts in 2 different extract volumes), were evaluated and filtered to reduce missing or ambiguous spectral assignments. Analytes were considered valid if they pass the following criteria: (I) non-missing values in ≥ 75% (30 out of 40) samples, (II) a stringent spectral matching of ≤ 0.2 to the library entry, (III) ≥ 3-fold increase compared to the averaged blank samples, and (IV) an average peak height of ≥ 5,000 arbitrary units. Furthermore, the remaining data were manually curated to address deconvolution errors, reduce redundant assignments of similar library entries, and to replace missing values (n = 23). The filtered GC/MS data (afterwards raw data) comprises 40 samples and 203 curated analytes with 1 (0.01%) missing value (Data S1). All GC/MS data were expressed relative to U-^{13}C-sorbitol. The two extract volume replicates per fraction were averaged after normalization

(see below; Data S1). The automatically generated compound annotation (see above) for known and unknown metabolites was verified using manual curation by visual evaluation of peak shape and spectral similarity of the observed mass spectra compared to library entries.

LC/MS data: The aligned LC/MS data (18 fractions and 2 total extracts) were filtered to reduce missing values. Analytes were considered valid if they pass the following criteria: (I) average peak height within one fraction group (samples of the 3 NAF gradients belonging to the same fraction) of ≥ 10,000 arbitrary units, (II) ≥ 2 non-missing values per fraction group, and (III) ≥ 16 out of the 18 fractions with non-missing values. The filtered data (afterwards raw data) consists of 20 samples and comprises 2804 and 910 analytes with 1125 (2%) and 457 (2.5%) missing values for lipophilic and secondary metabolites, respectively (Data S2-3). These analytes were annotated onto three levels: unknown, if no database hit could be assigned; match if an unverified database hit was assigned; and known for orthogonally validated database hits. The validation of known metabolites does not include the use of authentic reference standards, but instead relies on previously described compounds for Arabidopsis, the use of validated fragmentation patterns, and mass shifts of ^{13}C, ^{15}N, and ^{34}S isotope labeled *Arabidopsis thaliana* samples (Giavalisco et al., submitted) (Data S4).

Time/similariy (T/S) clusters: To reduce the complexity of aligned LC/MS data towards potential redundant analytes, i.e. peaks revealing mass shifts as the result of isotope incorporation, due to adducts or yet unknown reasons, clustering was performed on time groups. In detail, analytes were grouped according to their retention time into time groups with 1 digit (0.1 min) resolution. The similarities among time group members were computed on $\log_2$-transformed raw data using the parametric Pearson's product moment and non-parametric Spearman's rank-order correlation [5]. Both similarity matrices were averaged ($s_{\varnothing}$) and converted into distance range using the equation $(1 - s_{\varnothing}) / 2$. The

distance matrices were then clustered using average linkage clustering [6] and the resulting trees cut at 0.067 heights ($s_Ø$ = 0.866; Data S2-3). Thus, the defined time/similariy (T/S) clusters reflect about 75% of the observed variance among analytes within a cluster.

To this end, a total of 726 non-redundant time/similarity (T/S) clusters were identified for lipophilic metabolites of which 75 (10.3%) contain at least one known analyte and 196 (27%) at least one matched analyte. For the secondary metabolites, 461 clusters were identified with 17 (3.7%) containing a known analyte and 138 (29.9%) at least one matched analyte (Data S2-4). Thus, the degree of redundancy within high resolution MS data ranges in between 74.1% for the lipophilic data to 49.3% for the secondary metabolite data.

Other data: For some analyses individual metabolite data were assembled into a joint data sets complemented with metabolites measured by targeted approaches (thiols) or metabolic assays (chlorophyll, starch) (Data S4).

Statistical analyses

Scaling: Metabolite data were normalized to adjust for variations in sample amounts by assuming equality and stability of the total ion count (TIC) among the three independent NAF gradients. In detail, the TIC was calculated over the entire fractions (F1-6) of an individual gradient (TIC_g) using 90% non-extreme values by choosing the 5% and 95% quantile cutoffs determined on log_2-scale basis. The three gradient TICs were averaged ($TIC_Ø$) and the peak height data multiplied by the ratio of $TIC_Ø$ / TIC_g. For further downstream analyses data were either scaled, so that the sum of each single analyte across the six fractions of each individual gradient equals 100% (scaled data) or were log_2-transformed.

Missing values: Missing values were imputed by principal component analyses (PCA) on log_2-transformed data using Bayesian model [7] and the resulting complete data were back-transformed using the antilog.

Outliers: Outliers within the data were detected by a global boxplot approach using R. In detail, for each fraction group and analyte the observed peak heights were divided by the fraction group mean. Extreme deviations were detected using boxplot statistic by considering values exceeding the upper or lower whisker as outliers. Fraction groups containing outliers were re-evaluated by (I) identifying the most extreme value and (II) replacing it by the fraction group mean, calculated using all three values including the extreme one.

The normalized, imputed and outlier-removed data are provided as supplemental data (Data S1-3).

Consensus: The processed data were used to compute the robust cluster consensus distributions of members within T/S clusters or selected marker from LC/MS based metabolomics. For this, the cluster consensus was built on the basis of the robust cluster mean computed using Tukey’s biweight across all members.

PCA / HCA: Principal component analyses (PCA) were performed on scaled and log_2-transformed metabolite data with R’s pcaMethods [7]. Euclidean distances were calculated between the samples or Manhattan distances between the variables on scaled metabolite data. HCA using average linkage clustering were performed on Euclidean or Manhattan distances, where Manhattan distances were normalized to lay within the range of 0 to 1 on relative scale (division by 200) or 0 to 100% on a percentage scale (division by 2). *P*-values for cluster nodes were computed with the pvclust package using multiscale bootstrap resampling with 999 replicates [8].

Mantel test / ANOVA: Mantel tests were performed as Pearson's matrix correlation with 999 bootstrap samples either between distance matrices or as non-parametric ANOVA [5].

CMD: Normalized Manhattan distance matrices were converted into coordinates matrices using classical multidimensional scaling (CMD; [9]), and the first two principal coordinates, which reflected > 90% of the variance (data not shown), were visualized. Confidence ellipses were drawn for a 95% region using the correlation, mean and standard deviation of the data points.

Cluster spread: The between-gradient variation (cluster spread) of a measured analyte was estimated by computing the normalized Manhattan distances among all gradients. Similarly, the compartmental spread for a single compartment was estimated using all markers delineating the same compartment within and between gradients. The cluster spread of an analyte was expressed as percentage to the maximum (after outlier-removal by boxplot statistics) of compartmental spreads for all three resolved subcellular compartments (normalized cluster spread).

Robustness: To determine the robustness of the gradient data and the conducted downstream analyses, fractions from the three independent gradients were randomly selected without repetition to assembled artificial gradients, i.e. fraction 1 of gradient 1, then fraction 2 of gradient 3 and so forth. In total 729 (726 + 3 original) non-redundant random combinations were generated and the statistical analyses were repeated for each of those combination.

Compartmental distribution and assignment

BestFit – subcellular distribution: Subcellular metabolite distributions with 1% resolution were computed using a C-language implementation and extension towards n-compartments (with n ≤ 5) of a 3-compartmental distribution algorithm (best fit algorithm,

BFA) according to Riens et al. [10] called BestFit (available upon request). Non-negative least square (NNLS) [11] fit was additionally employed and the Fortran 77 routine complied into the BestFit command line tool.

Compartmental assignment: Analytes were assigned onto the three resolved subcellular compartments using a k-nearest neighbor (k-NN) approach [12] with k = 3 nearest neighbors (estimated using cross-evaluation) on normalized Manhattan distances employing R's knnflex package. For a more flexible assignment a classification tree was manually constructed and analytes assigned accordingly (Figure 6). K-medoids clustering, a more robust version of k-means clustering was employed to partitioning the analytes into 7 (for analytes with insufficiently explained subcellular distributions) or 6 (for all analytes) clusters (Data S4). The number of clusters (k) was determined by allowing only the cytosolic compartment (represented by three compartment-specific markers) to be partitioned into different clusters without being assigned onto another compartment.

Supporting Data

Data S1. Raw and processed GC-TOF/MS data of primary metabolites.

Both MS Excel sheets contain the measurements for 203 measured and validated analytes. The sheet ‘raw’ contains the raw data matrix of 40 measured fraction aliquots including the data for the internal control, U-^{13}C-sorbitol. The sheet ‘proc’ contains the processed, i.e. normalized, averaged, imputed and outlier-handled data matrix, from 18 averaged fraction samples. The fractions and corresponding independent gradients are given as table headers. Each individual sample is labeled by the fraction number followed by the gradient number (e.g. F1.G1: fraction 1 of gradient 1) and also contains the aliquot amount used (e.g. F1.G1-100: 100 µL of fraction 1 from gradient 1). Total extract samples (non-fractionated material) are labeled as F0; fractions were collected from top (F6; chloroplast enriched fraction; lowest density) to the bottom (F1; vacuolar enriched fraction; highest density). Analyte identifiers (‘AnalyteID’) are shown as concatenation of name and retention index (adjusted retention time; in seconds) and, if applicable, the source organism (Exp - found experimentally in multiple organisms; Ath - *A. thaliana*). Multiple metabolite assignments of analytes are separated by vertical bars between compound names. Additionally, a weblink (‘Link’; if available) to online compound databases, the quantification mass (‘Quant.m/z’), and the computed matching factor (‘MF.Spectrum’) is depicted for each analyte. Matching factors can range from a perfect (0.0) to the most dissimilar (1.0) spectral match.

cf. *Krueger_NAF_Supplemental-Data-S1* (attached supporting MS Excel file)

Data S2. Raw and processed UPLC-FT/MS data of lipophilic metabolites.

Both MS Excel sheets contain the measurements for 2804 time-m/z pairs (analytes) and 18 fraction samples (F1-F6) including two total extract samples, i.e. non-fractionated material (F0; only raw data). The fractions and corresponding independent gradients are given as table headers. Each individual sample is labeled by the fraction number followed by the gradient number (e.g. F1.G1: fraction 1 of gradient 1). Analyte identifiers ('AnalyteID') are given by the letter L followed by a unique number including the concatenation of the mass (m/z) and retention time, both parameters as high-resolution values are depicted in the peak information section. T/S cluster assignments to reduce data complexity are provided in the raw data sheet, containing the median intensity across all samples (median intensity), the number of valid measurements (count data) and the median correlation among cluster members. The corresponding T/S cluster identifiers are given as the concatenation of the letter L followed by the time window (resolution 0.1 min) and a unique sub-cluster number.

cf. *Krueger_NAF_Supplemental-Data-S2* (attached supporting MS Excel file)

Data S3. Raw and processed UPLC-FT/MS data of secondary metabolites.

Both MS Excel sheets contain the measurements for 910 time-m/z pairs (analytes) and 18 fraction samples (F1-F6) including two total extract samples, i.e. non-fractionated material (F0; only raw data). The fractions and corresponding independent gradients are given as table headers. Each individual sample is labeled by the fraction number followed by the gradient number (e.g. F1.G1: fraction 1 of gradient 1). Analyte identifiers ('AnalyteID') are given by the letter S followed by a unique number including the concatenation of the mass (m/z) and retention time, both parameters as high-resolution values are depicted in the peak information section. T/S cluster assignments to reduce data complexity are provided in the raw data sheet, containing the median intensity across all samples (median intensity), the number of valid measurements (count data) and the median correlation among cluster members. The corresponding T/S cluster identifiers are given as the concatenation of the letter S followed by the time window (resolution 0.1 min) and a unique sub-cluster number.

cf. *Krueger_NAF_Supplemental-Data-S3* (attached supporting MS Excel file)

Data S4. Fused metabolome data set covering analyte annotations as well as results of estimated subcellular distributions and compartmental assignments.

The Excel sheet 'data' comprises the processed, i.e. normalized, averaged, imputed and outlier-handled, and scaled data matrix for all analytes measured using MS based approaches or specific assays including the selected compartment-specific markers. The fractions and corresponding independent gradients are given as table headers. Each individual sample is labeled by the fraction number followed by the gradient number (e.g. F1.G1: fraction 1 of gradient 1).

The Excel sheet 'comparison' contains the results of the bestfit (BFA) and non-negative least square (NNLS) approach to estimate the subcellular distributions on the basis of the three independent and the 729 randomly assembled gradient data. The estimated subcellular distributions for each computational approach are given as mean including the standard deviation. Error estimates (Q-values, residual sum of squares) as well as further quality estimates for the fit, e.g. the normalized Manhattan distance, accounted as total percentage discrepancy (TPD) between the measured and fitted gradient distributions used to define insufficiently explained distributions, are provided as mean values.

The sheet 'summary' comprises annotation information and results of estimated subcellular distributions and compartmental classification on the basis of the BFA algorithm using the three independent gradients. The peak information section, such as m/z, retention (time or index) and time-similarity (T/S) cluster assignments, are provided according to the specific metabolome platform targeted towards the individual major compounds classes, i.e. primary, lipophilic, secondary metabolites or specific assays used for thiols and others (citrate synthase, chlorophyll). The annotation section contains annotation information and further comments for each analyte, including the metabolite name, chemical class and superclass assignments, chemical superclass predictions and

experimental confirmations by stable isotope labeling (column ‘comments’, Krall et al., in prep.) as well as a broad assignment into marker, known, matched, or unknown analytes (‘type’ column). The comment column can contain further results regarding manual curation. Individual analytes used to estimate the robust gradient distribution for defining robust compartment specific marker are tagged in the marker column. Moreover, the classification of analytes into resolved compartments or unresolved subcellular units are provided with respect to individual classification strategies, i.e. classification tree, k-medoids clustering, and k-nearest neighbor. The estimated subcellular distributions for a three-compartmental fit are provided as described above and include the frequency of unexplained estimates in dependency of the TPD (normalized Manhattan distance computed as the total percentage discrepancy between the measured and fitted gradient data) cutoff chosen.

cf. *Krueger_NAF_Supplemental-Data-S4* (attached supporting MS Excel file)

Data S5. Distribution of measured and fitted fraction abundances of analytes across the gradient based on three independent gradient data.

Each bar plot shows the abundances, based on processed and scaled data, of an analyte in each fraction as average including standard deviation across the gradient. The white bars represent the measured abundances, whereas the grey colored bars depicting the fitted data. The analyte name is shown as plot title. The average total percentage discrepancy (TPD) between the measured and fitted data, estimated using normalized Manhattan distance, is provided on the top left side in each graph followed by the number of insufficiently explained fits (out of 3) and the number of significantly different fractions (uncorrected $P < 0.05$ as * and $P < 0.01$ as ** using t-test), all separated using a vertical bar character. In this study, subcellular distributions were considered as insufficiently explained if both, the average TPD across the three gradients exceeded 10% and the TPD from individual gradients exceeded 10% in ≥ 50% of the cases, i.e. 2 out of 3 independent gradients.

cf. *Krueger_NAF_Supplemental-Data-S5* (attached supporting PDF file)

Data S6. Scatter plots of analytes and compartment-specific markers in the principal coordinates space for visual assessment of subcellular location.

Manhattan distances among markers and analytes for each gradient were converted into a principal coordinates (PCo) space using classical multidimensional scaling (CMD) for the three independent and 726 (+3 original) non-redundant combinations of randomly assembled gradients. Shapes colored in cyan (markers) or magenta (analyte under investigation) show the data points for the three independent gradients G1 – triangle down, G2 - square, and G3 – triangle up. Data points from simulated gradients are depicted as circles with coloration according to the individual compartment, namely plastid – green, cytosol – blue, and vacuole – grey, or analyte under investigation - yellow. To aid interpretation only the most extreme 20 data points (determined using HCA based on Euclidean distances) are depicted for each individual marker and analyte. For each compartment or analyte under investigation the 95% confidence ellipse is drawn as solid grey line. The name of each analyte depicted is given as plot header. For further metabolite details see Data S1 to Data S4. The principal coordinates 1 and 2 explain together in average about 96.7% of the total variance of the underlying distance matrices.

cf. *Krueger_NAF_Supplemental-Data-S6* (attached supporting PDF file)

Supporting References

1. Krall L, Huege J, Catchpole G, Steinhauser D, Willmitzer L (2009) Assessment of sampling strategies for gas chromatography-mass spectrometry (GC-MS) based metabolomics of cyanobacteria. J Chromatogr B Analyt Technol Biomed Life Sci 877: 2952-2960.

2. Hell R, Bergmann L (1990) γ-Glutamylcysteine synthetase in higher plants: catalytic properties and subcellular localization. Planta 180: 603–612.

3. Kanehisa M, Goto S (2000) KEGG: kyoto encyclopedia of genes and genomes. Nucleic Acids Res 28: 27-30.

4. Shinbo Y, Nakamura Y, Altaf-Ul-Amin U, Asahi H, Kurokawa K, et al. (2006) KNApSAcK: A comprehensive species-metabolite relationship database. In: Saito K, Dixon RA, Willmitzer L, editors. Plant Metabolomics (Biotechnology in Agriculture and Forestry) Heidelberg. pp. 165–184.

5. Sokal RR, Rohlf FJ (1995) Biometry: The principles and practice of statistics in biological research. New York: W.H. Freeman and Company. 337 p.

6. Theodoridis S, Koutroumbas K (2009) Pattern Recognition: Academic Press. 984 p.

7. Stacklies W, Redestig H, Scholz M, Walther D, Selbig J (2007) pcaMethods--a bioconductor package providing PCA methods for incomplete data. Bioinformatics 23: 1164-1167.

8. Suzuki R, Shimodaira H (2006) Pvclust: an R package for assessing the uncertainty in hierarchical clustering. Bioinformatics 22: 1540-1542.

9. Cox TF, Cox MAA (1994) Multidimensional Scaling. Monographs on Statistics and Applied Probability. Boca Raton: Chapman and Hall/CRC.

10. Riens B, Lohaus G, Heineke D, Heldt HW (1991) Amino Acid and Sucrose Content Determined in the Cytosolic, Chloroplastic, and Vacuolar Compartments and in the Phloem Sap of Spinach Leaves. Plant Physiol 97: 227-233.

11. Lawson CL, Hanson RJ (1995) Solving Least Squares Problems. Classics in Applied Mathematics. Philadelphia: SIAM.

12. Ripley BD (1996) Pattern Recognition and Neural Networks. Cambridge: Cambridge University Press.
