# Supplementary material for: A Topological Map of the Compartmentalized Arabidopsis thaliana Leaf Metabolome
Source: PLoS One. 2011 Mar 15;6(3):e17806. doi: 10.1371/journal.pone.0017806 (PMC3058050; doi:10.1371/journal.pone.0017806)

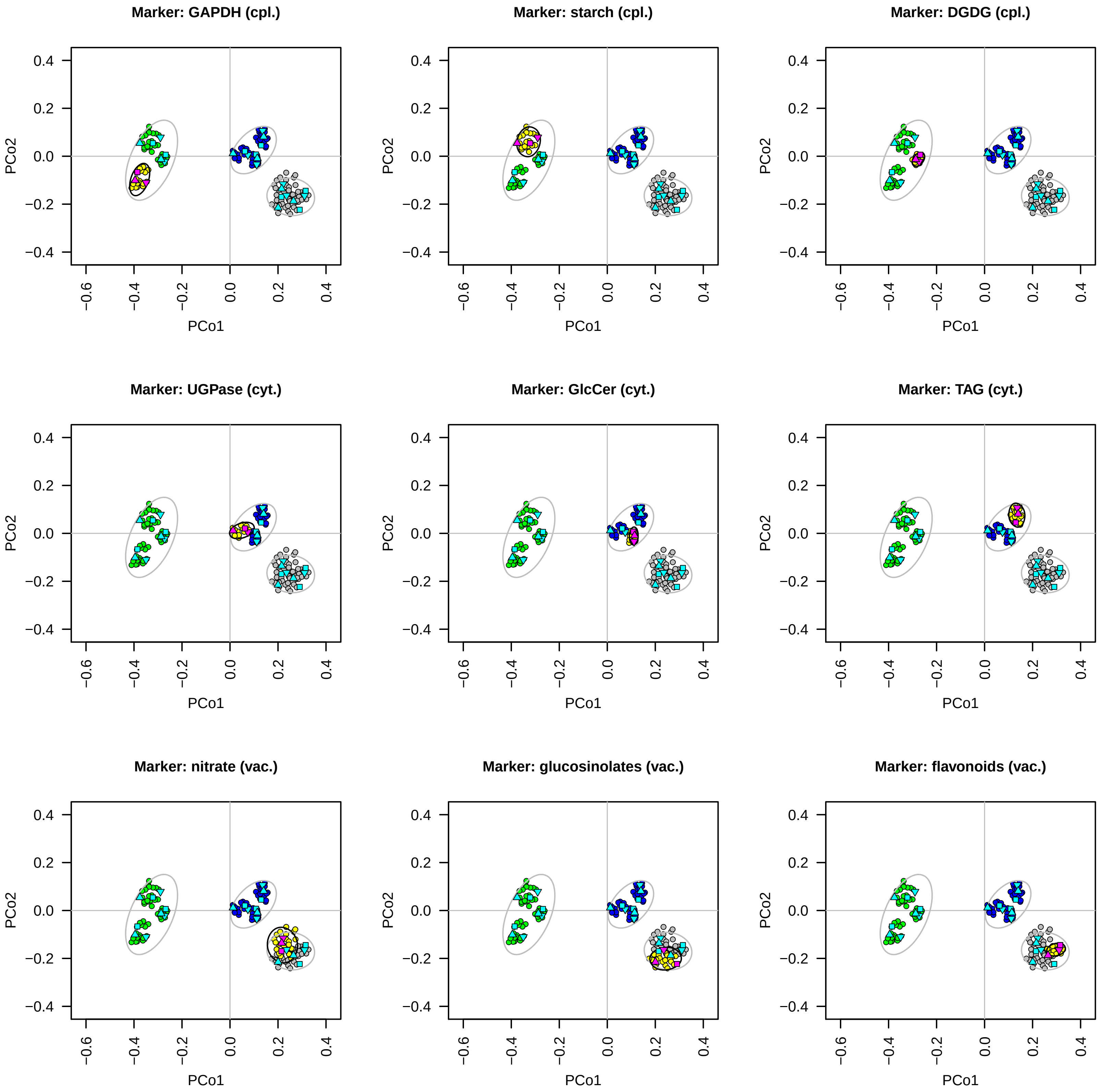
Marker: GAPDH (cpl.)
Marker: starch (cpl.)
Marker: DGDG (cpl.)
Marker: UGPase (cyt.)
Marker: GlcCer (cyt.)
Marker: TAG (cyt.)
Marker: nitrate (vac.)
Marker: glucosinolates (vac.)
Marker: flavonoids (vac.)
PCo1
PCo2
0.4
0.2
0.0
−0.2
−0.4
−0.6

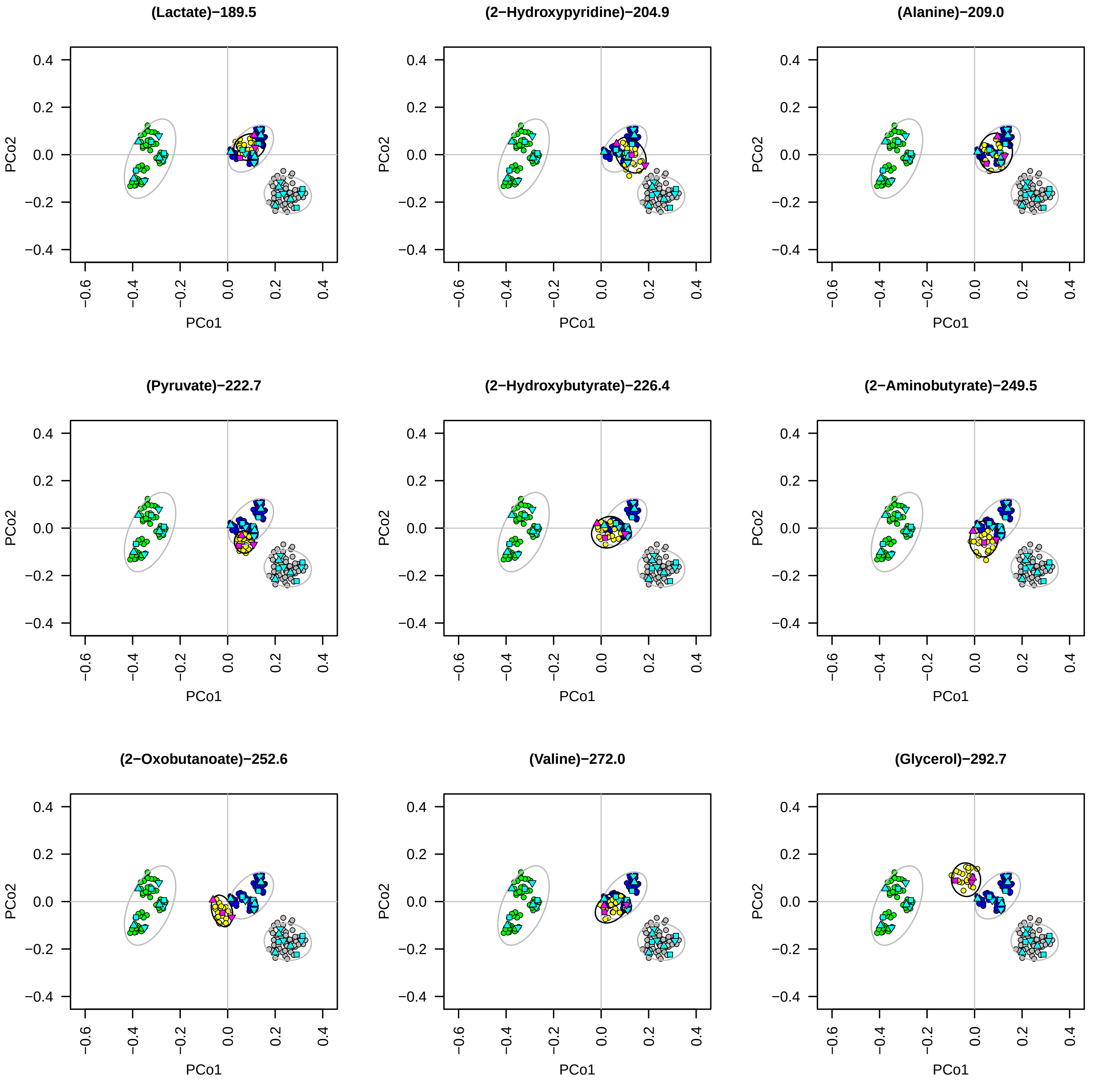
(Lactate)−189.5
(2−Hydroxypyridine)−204.9
(Alanine)−209.0
(Pyruvate)−222.7
(2−Hydroxybutyrate)−226.4
(2−Aminobutyrate)−249.5
(2−Oxobutanoate)−252.6
(Valine)−272.0
(Glycerol)−292.7
PCo1
PCo2
0.4
0.2
0.0
−0.2
−0.4
−0.6

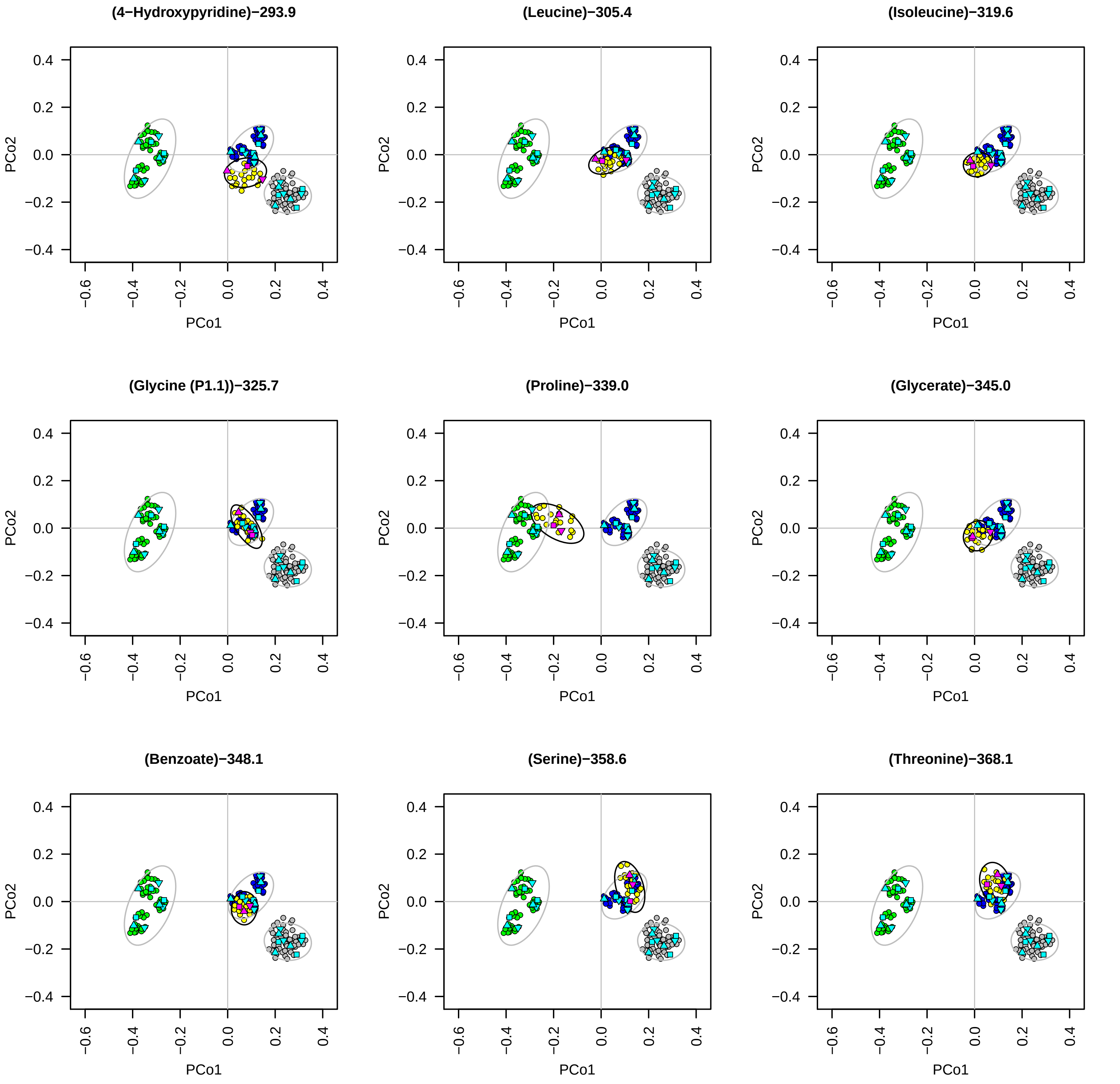
(4−Hydroxypyridine)−293.9
(Leucine)−305.4
(Isoleucine)−319.6
(Glycine (P1.1))−325.7
(Proline)−339.0
(Glycerate)−345.0
(Benzoate)−348.1
(Serine)−358.6
(Threonine)−368.1
PCo1
PCo2
0.4
0.2
0.0
−0.2
−0.4
−0.6

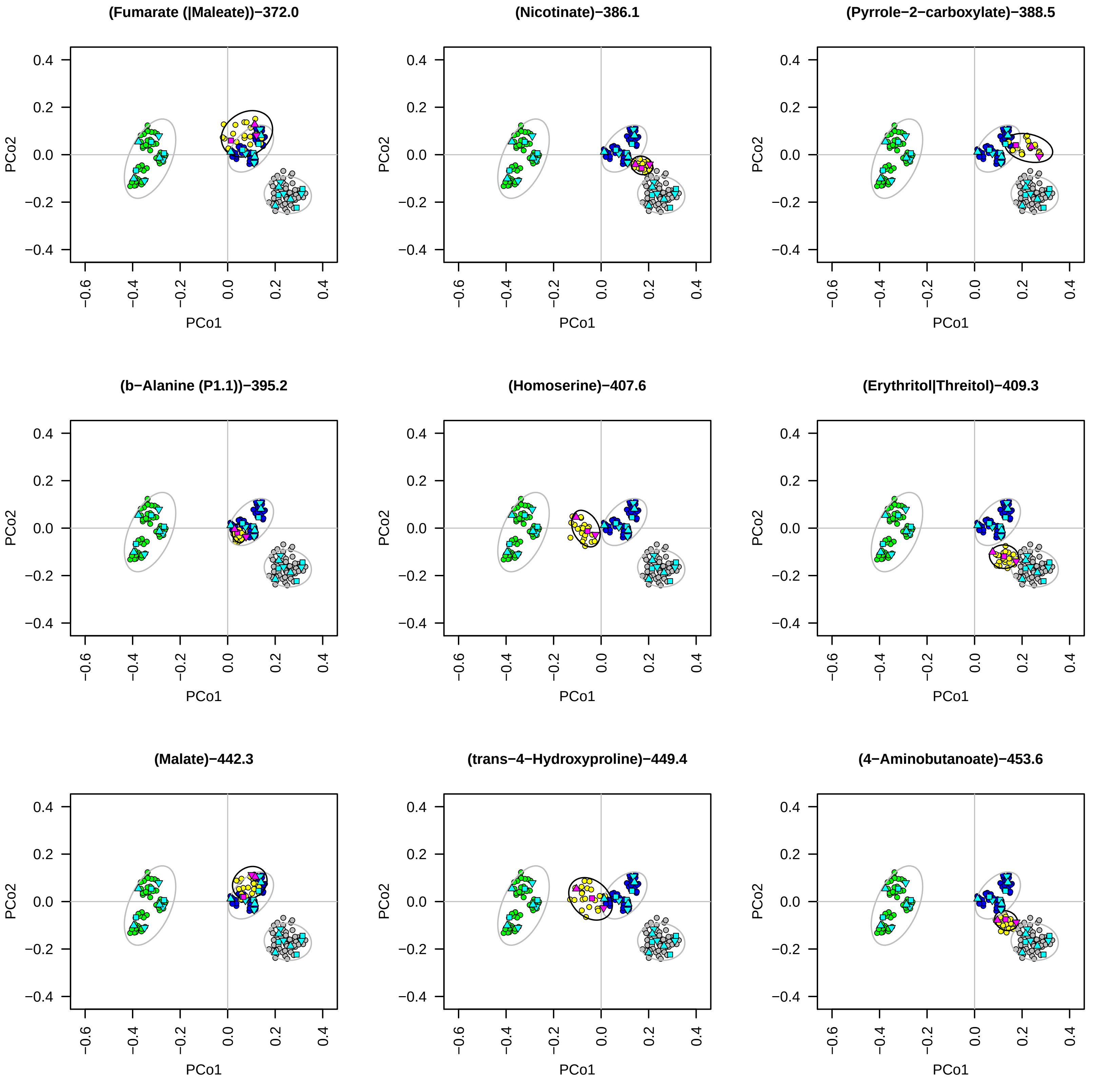
(Fumarate (|Maleate))−372.0
(Nicotinate)−386.1
(Pyrrole−2−carboxylate)−388.5
(b−Alanine (P1.1))−395.2
(Homoserine)−407.6
(Erythritol|Threitol)−409.3
(Malate)−442.3
(trans−4−Hydroxyproline)−449.4
(4−Aminobutanoate)−453.6
PCo1
PCo2

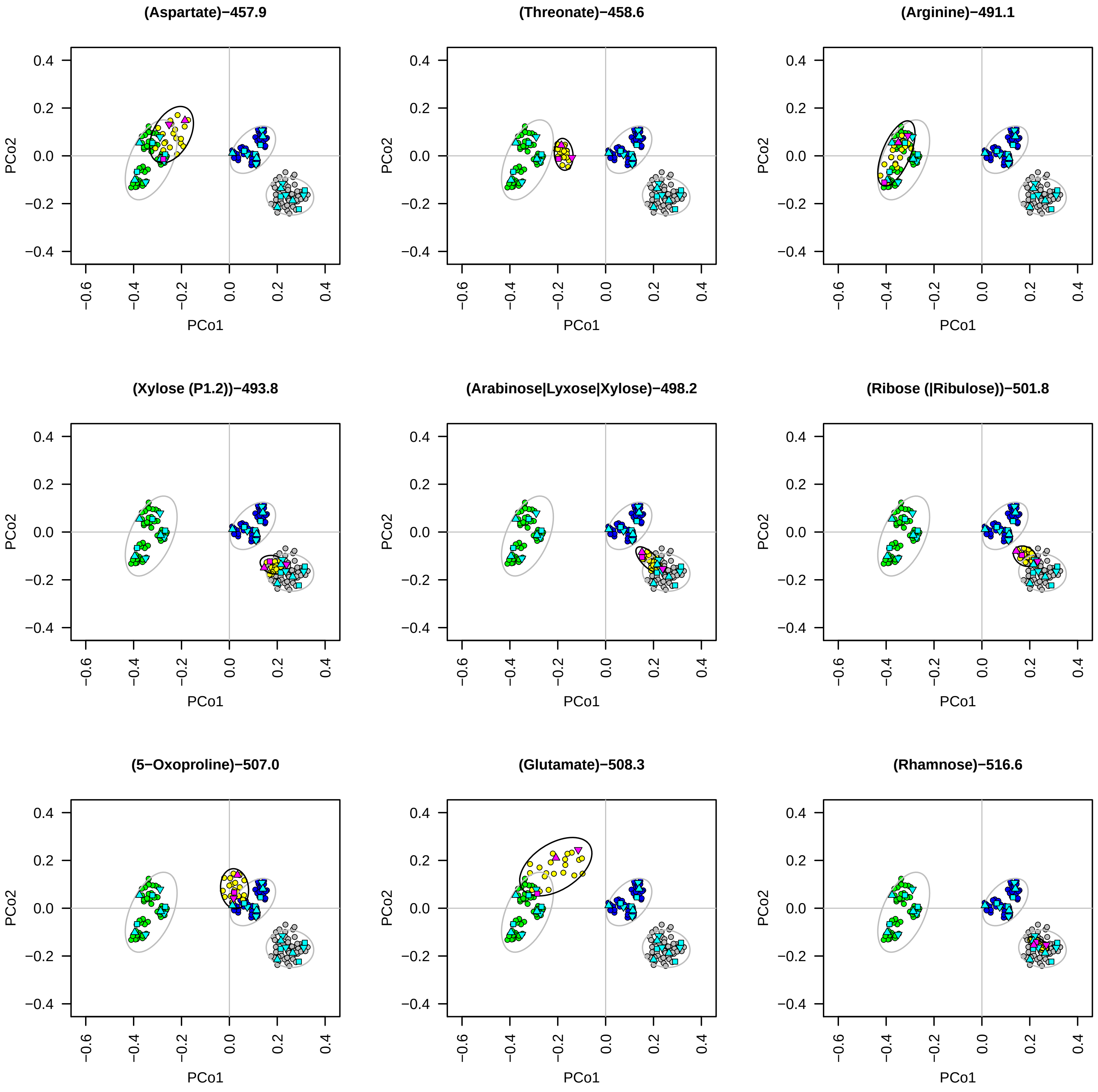
(Aspartate)−457.9
(Threonate)−458.6
(Arginine)−491.1
(Xylose (P1.2))−493.8
(Arabinose|Lyxose|Xylose)−498.2
(Ribose (|Ribulose))−501.8
(5−Oxoproline)−507.0
(Glutamate)−508.3
(Rhamnose)−516.6
PCo1
PCo2
0.4
0.2
0.0
−0.2
−0.4
−0.6

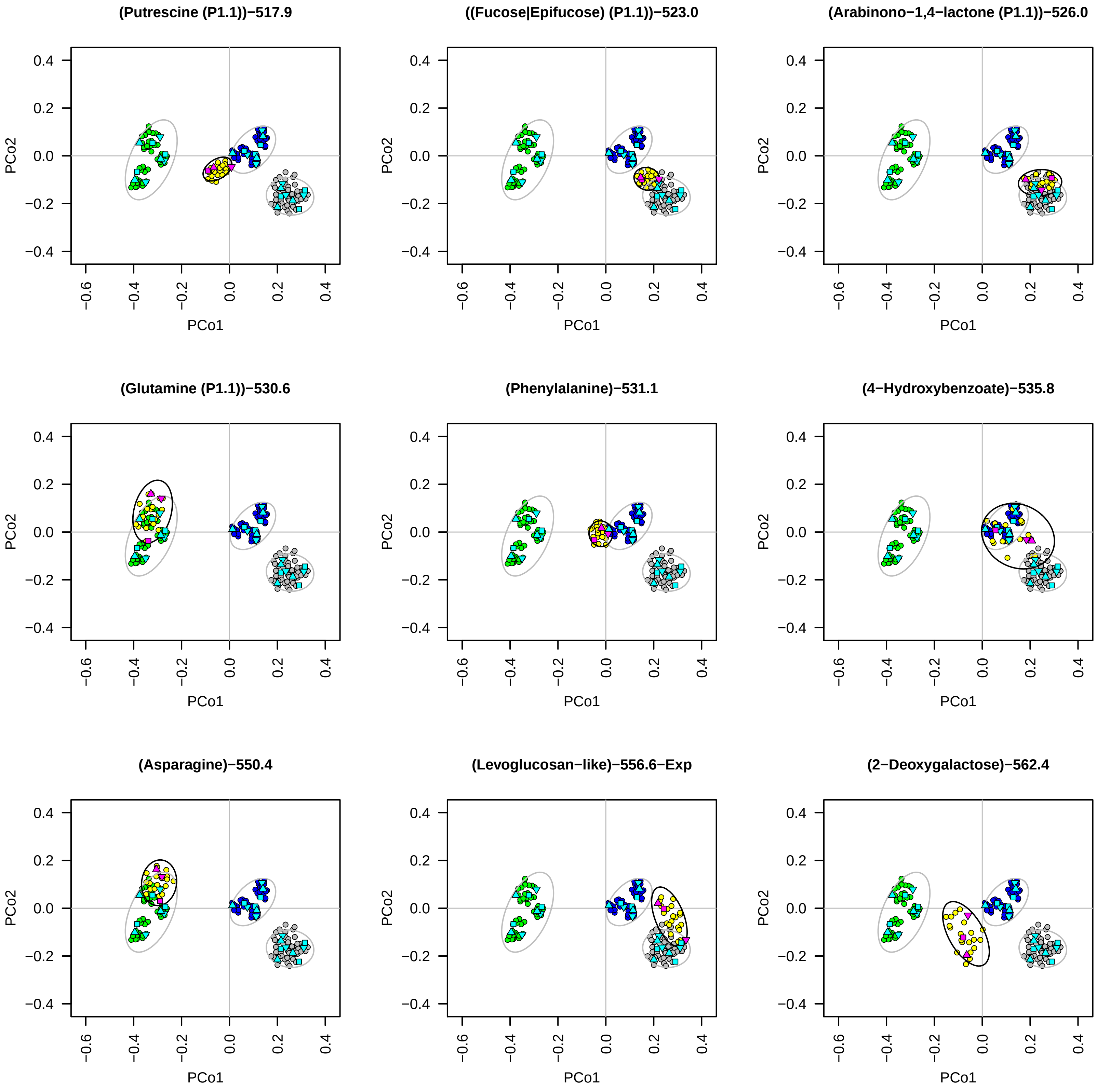

(Putrescine (P1.1))−517.9
((Fucose|Epifucose) (P1.1))−523.0
(Arabinono−1,4−lactone (P1.1))−526.0
(Glutamine (P1.1))−530.6
(Phenylalanine)−531.1
(4−Hydroxybenzoate)−535.8
(Asparagine)−550.4
(Levoglucosan−like)−556.6−Exp
(2−Deoxygalactose)−562.4
PCo1
PCo2
−0.6
−0.4
−0.2
0.0
0.2
0.4

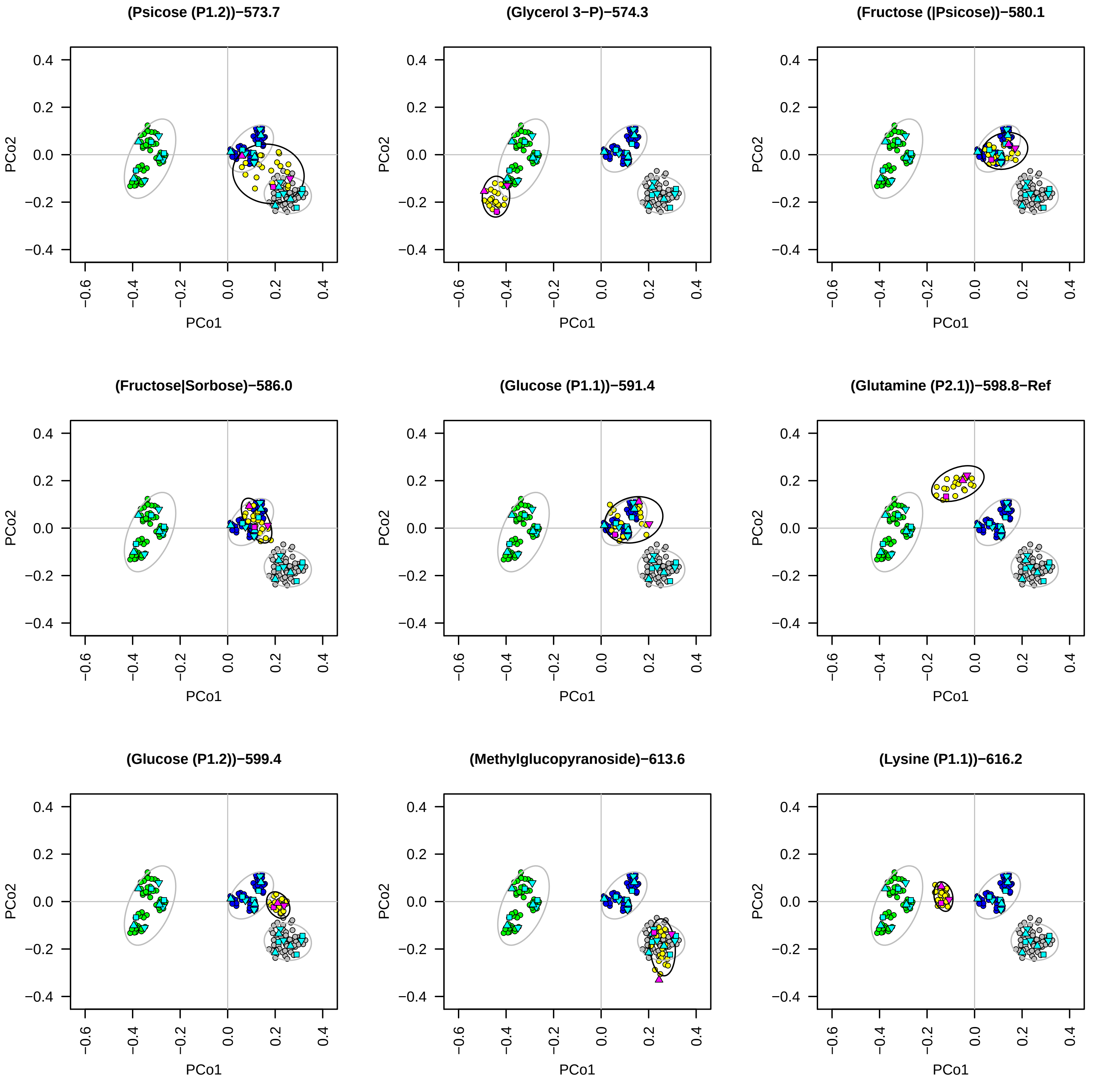
(Psicose (P1.2))−573.7
(Glycerol 3−P)−574.3
(Fructose (|Psicose))−580.1
(Fructose|Sorbose)−586.0
(Glucose (P1.1))−591.4
(Glutamine (P2.1))−598.8−Ref
(Glucose (P1.2))−599.4
(Methylglucopyranoside)−613.6
(Lysine (P1.1))−616.2
PCo1
PCo2
−0.6
−0.4
−0.2
0.0
0.2
0.4

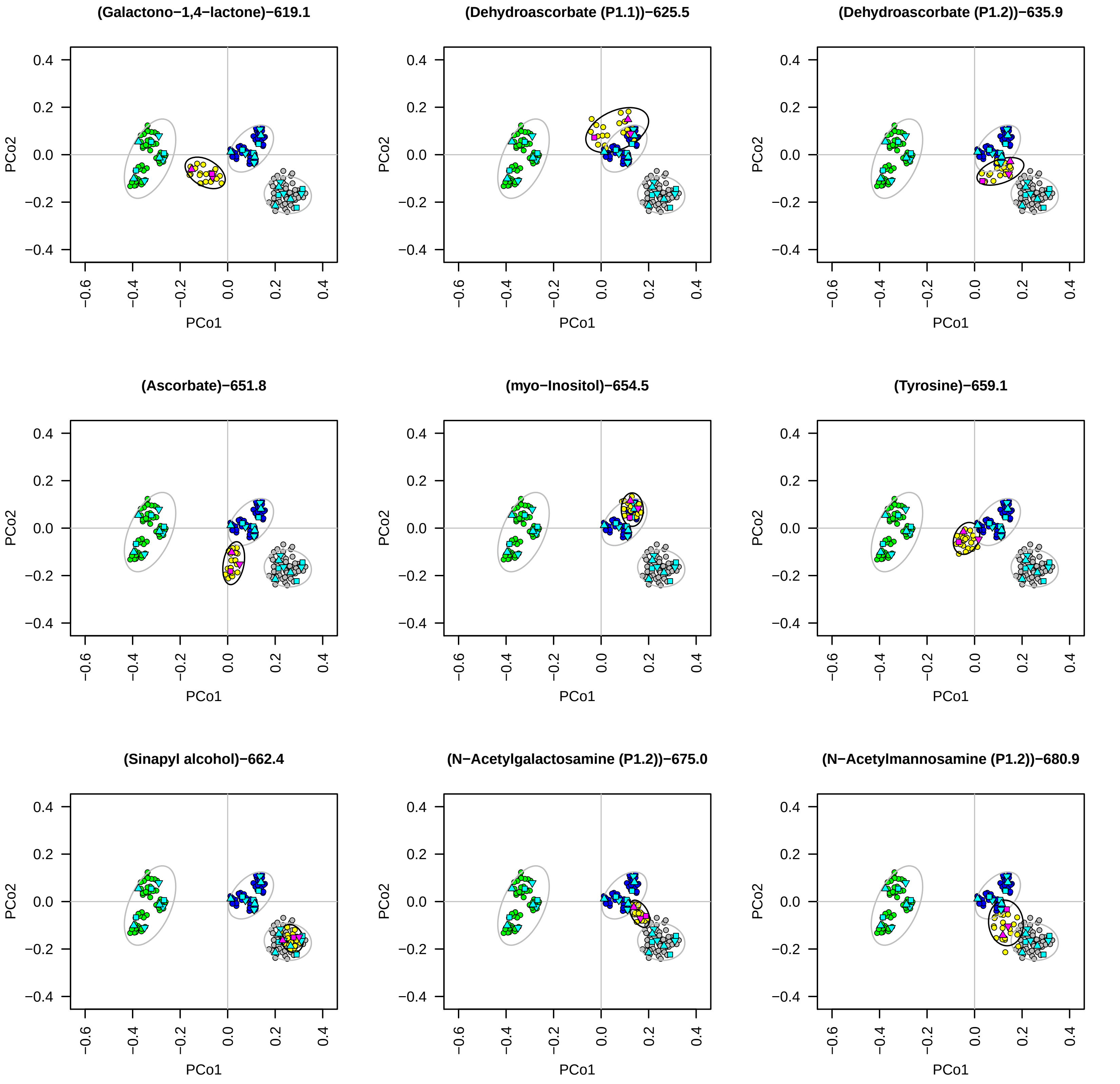
(Galactono−1,4−lactone)−619.1
(Dehydroascorbate (P1.1))−625.5
(Dehydroascorbate (P1.2))−635.9
(Ascorbate)−651.8
(myo−Inositol)−654.5
(Tyrosine)−659.1
(Sinapyl alcohol)−662.4
(N−Acetylgalactosamine (P1.2))−675.0
(N−Acetylmannosamine (P1.2))−680.9
PCo1
PCo2
−0.6
−0.4
−0.2
0.0
0.2
0.4

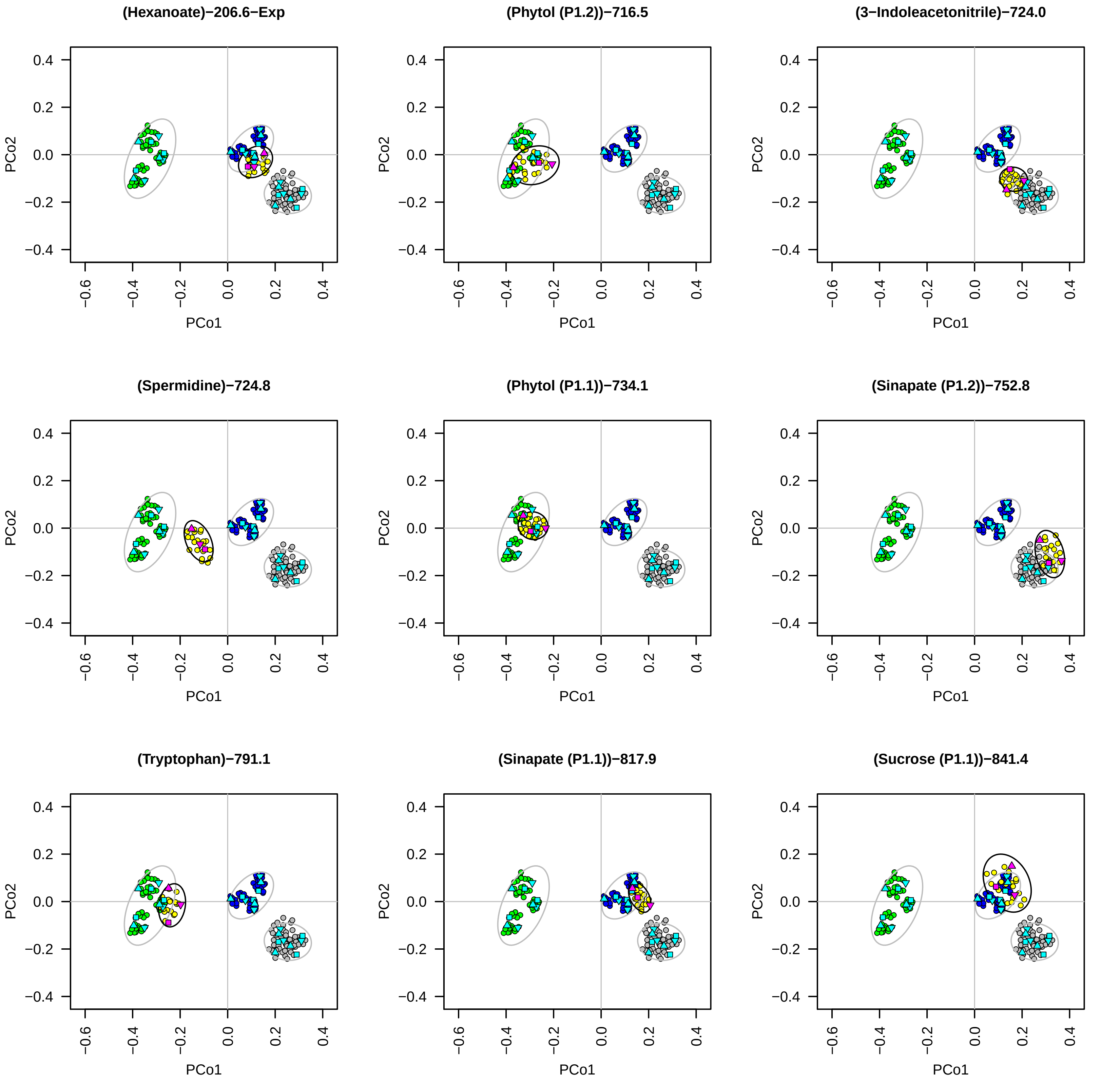
(Hexanoate)−206.6−Exp
(Phytol (P1.2))−716.5
(3−Indoleacetonitrile)−724.0
(Spermidine)−724.8
(Phytol (P1.1))−734.1
(Sinapate (P1.2))−752.8
(Tryptophan)−791.1
(Sinapate (P1.1))−817.9
(Sucrose (P1.1))−841.4
PCo1
PCo2
0.4
0.2
0.0
−0.2
−0.4
−0.6

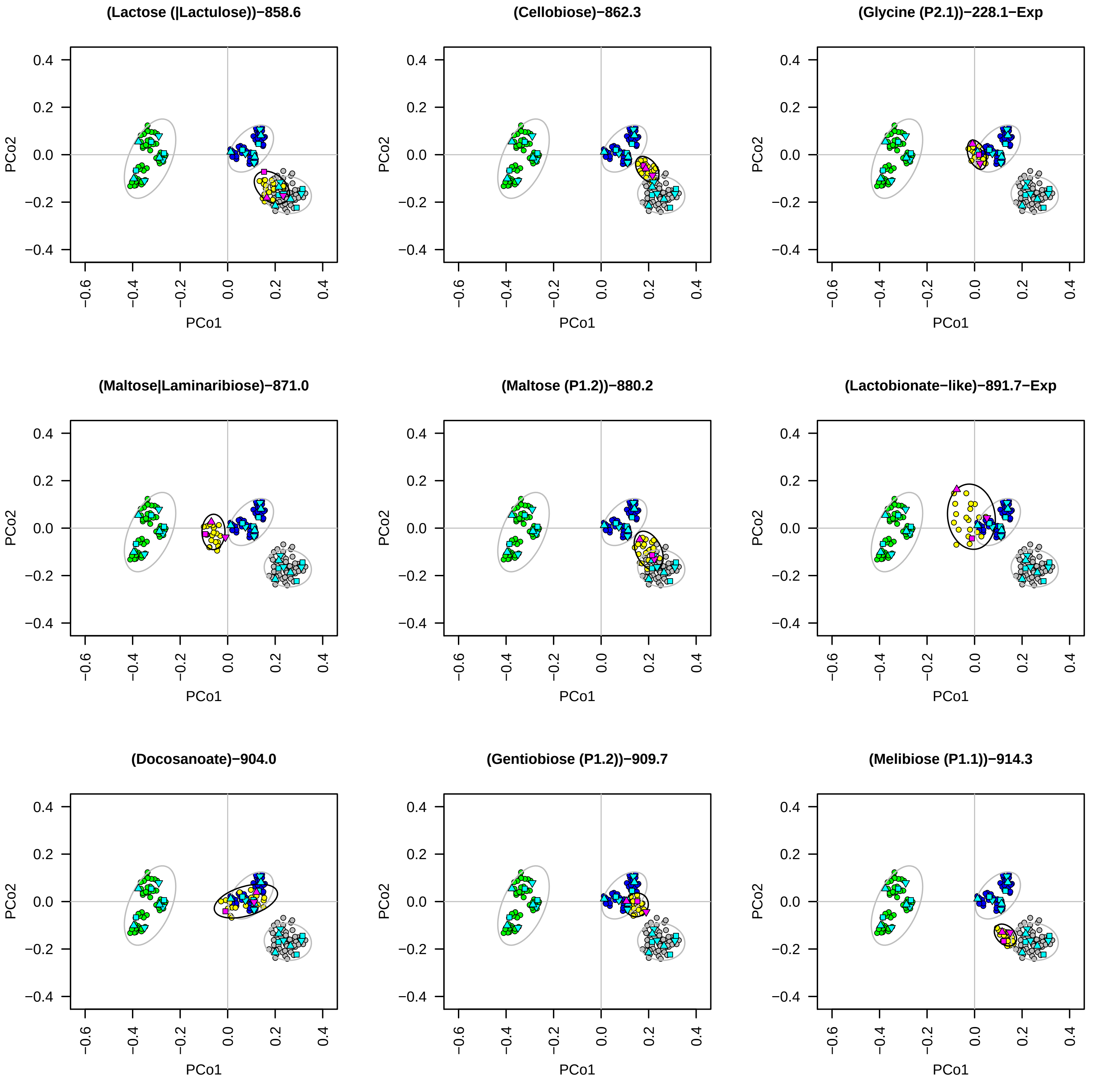
(Lactose (|Lactulose))−858.6
(Cellobiose)−862.3
(Glycine (P2.1))−228.1−Exp
(Maltose|Laminaribiose)−871.0
(Maltose (P1.2))−880.2
(Lactobionate−like)−891.7−Exp
(Docosanoate)−904.0
(Gentiobiose (P1.2))−909.7
(Melibiose (P1.1))−914.3
PCo1
PCo2
0.4
0.2
0.0
−0.2
−0.4
−0.6

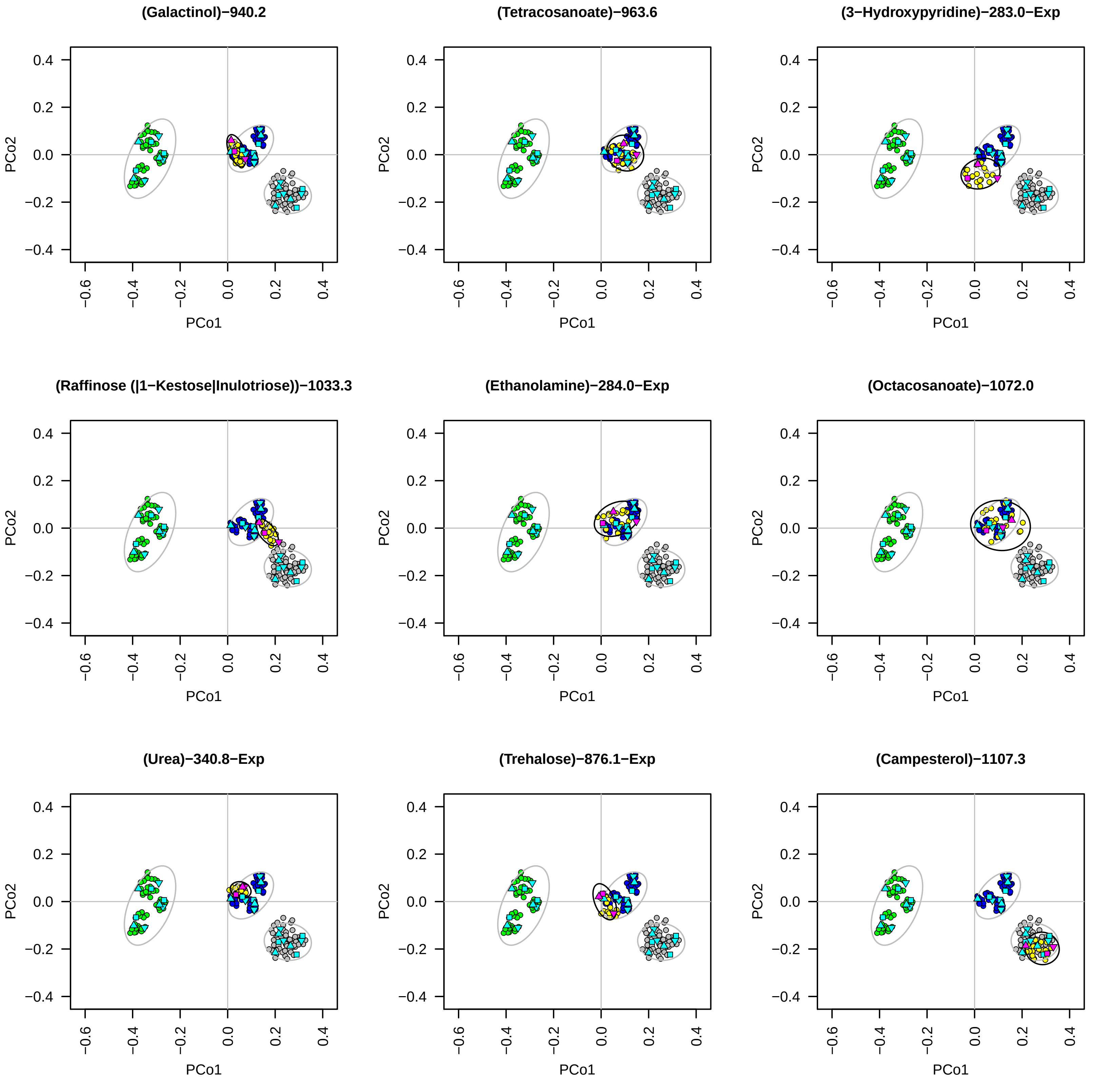
(Galactinol)−940.2
(Tetracosanoate)−963.6
(3−Hydroxypyridine)−283.0−Exp
(Raffinose (|1−Kestose|Inulotriose))−1033.3
(Ethanolamine)−284.0−Exp
(Octacosanoate)−1072.0
(Urea)−340.8−Exp
(Trehalose)−876.1−Exp
(Campesterol)−1107.3
PCo1
PCo2
0.4
0.2
0.0
−0.2
−0.4
−0.6

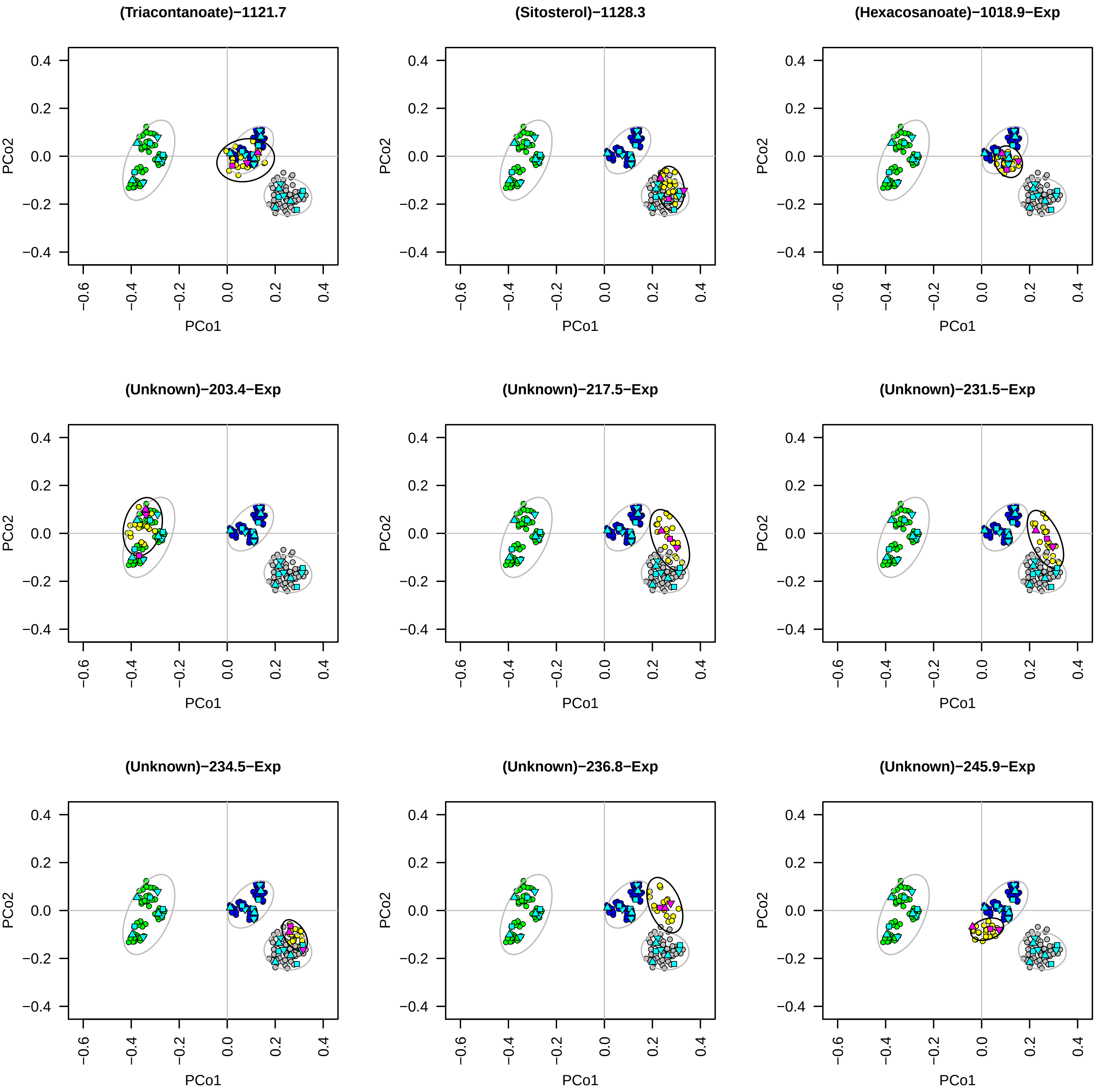
(Triacontanoate)−1121.7
(Sitosterol)−1128.3
(Hexacosanoate)−1018.9−Exp
(Unknown)−203.4−Exp
(Unknown)−217.5−Exp
(Unknown)−231.5−Exp
(Unknown)−234.5−Exp
(Unknown)−236.8−Exp
(Unknown)−245.9−Exp
PCo1
PCo2
0.4
0.2
0.0
−0.2
−0.4
−0.6

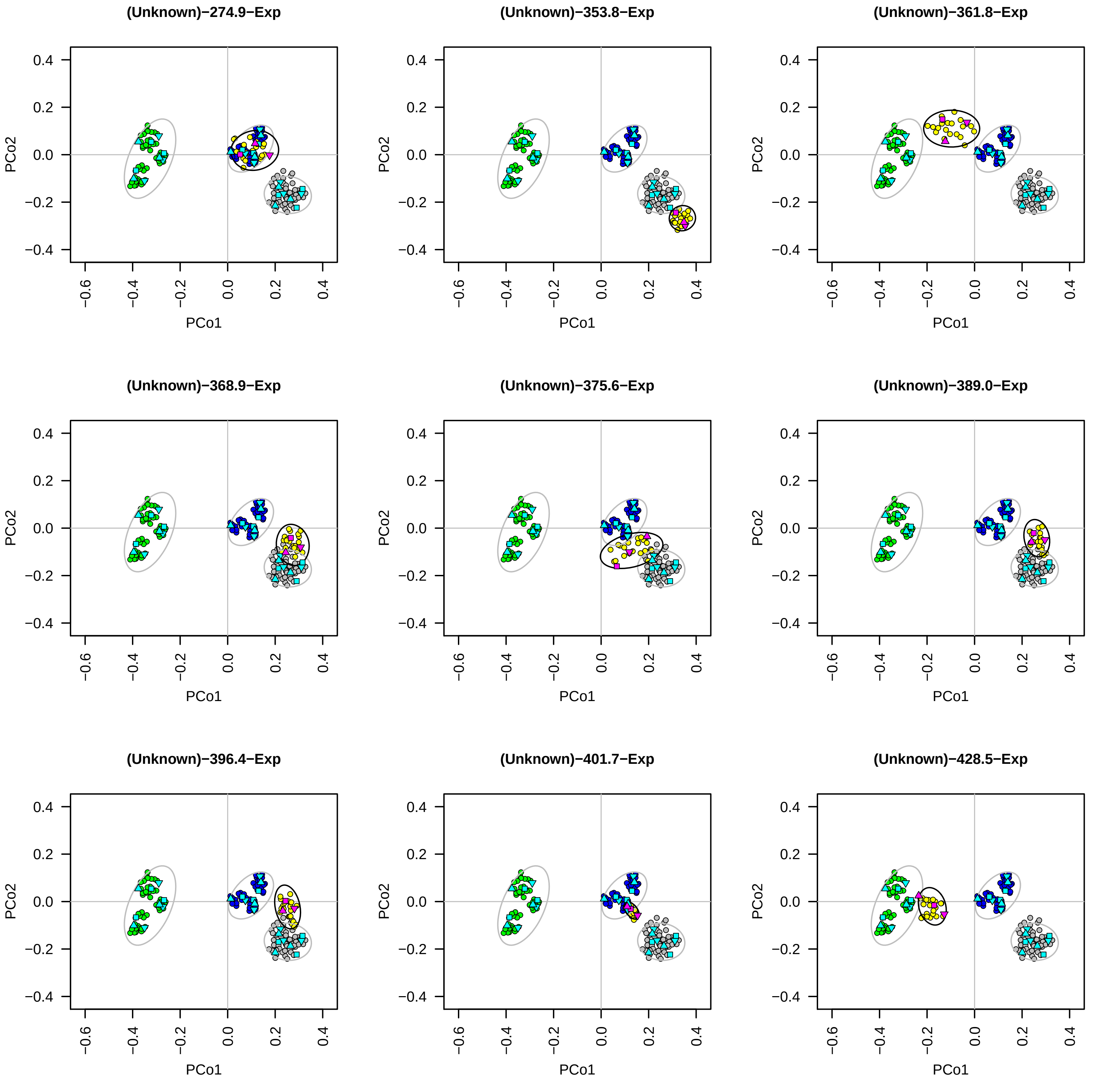
(Unknown)−274.9−Exp
(Unknown)−353.8−Exp
(Unknown)−361.8−Exp
(Unknown)−368.9−Exp
(Unknown)−375.6−Exp
(Unknown)−389.0−Exp
(Unknown)−396.4−Exp
(Unknown)−401.7−Exp
(Unknown)−428.5−Exp
PCo1
PCo2
−0.6
−0.4
−0.2
0.0
0.2
0.4

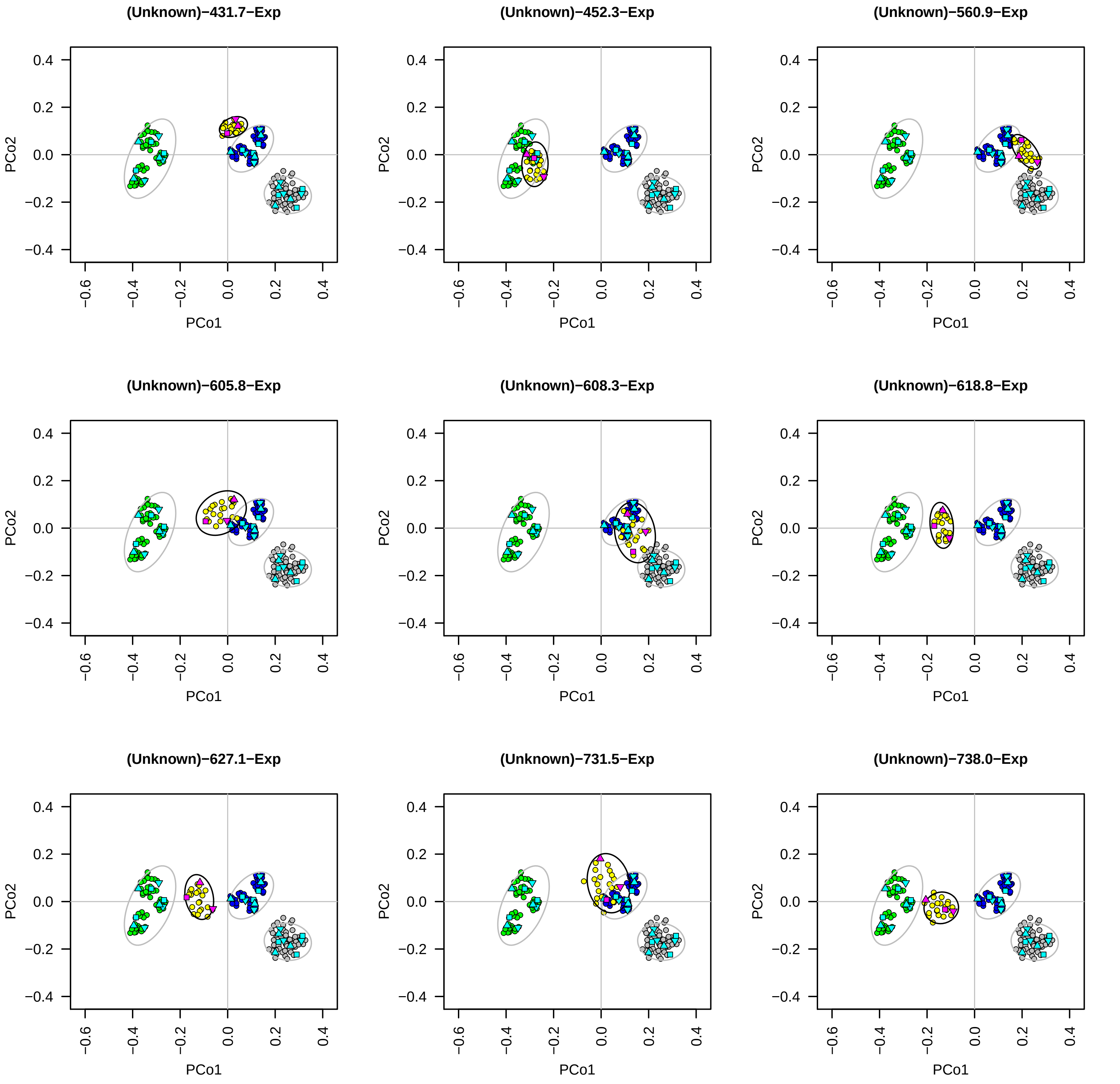

(Unknown)−431.7−Exp
(Unknown)−452.3−Exp
(Unknown)−560.9−Exp
(Unknown)−605.8−Exp
(Unknown)−608.3−Exp
(Unknown)−618.8−Exp
(Unknown)−627.1−Exp
(Unknown)−731.5−Exp
(Unknown)−738.0−Exp
PCo1
PCo2

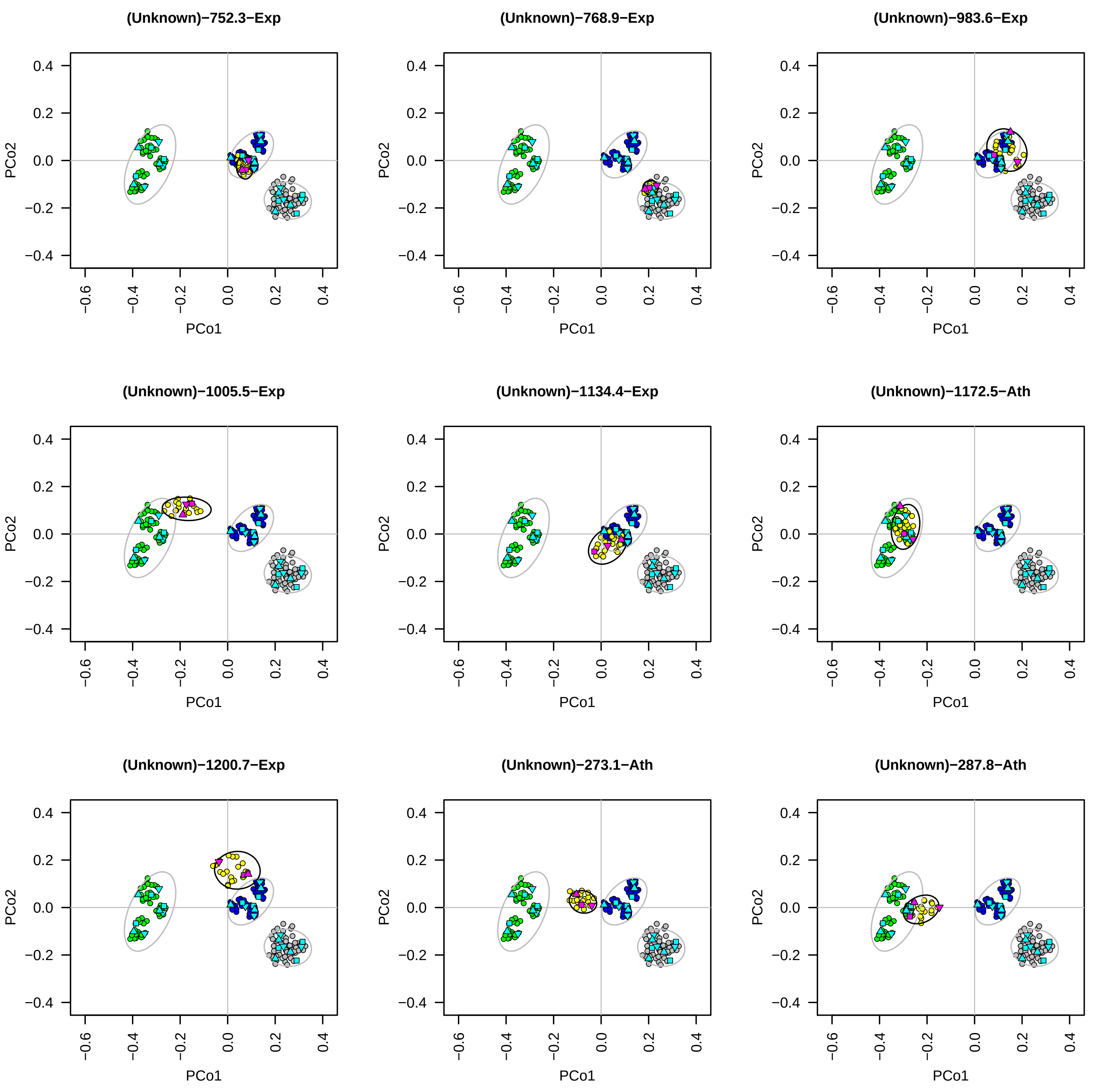
(Unknown)−752.3−Exp
(Unknown)−768.9−Exp
(Unknown)−983.6−Exp
(Unknown)−1005.5−Exp
(Unknown)−1134.4−Exp
(Unknown)−1172.5−Ath
(Unknown)−1200.7−Exp
(Unknown)−273.1−Ath
(Unknown)−287.8−Ath
PCo1
PCo2
−0.6
−0.4
−0.2
0.0
0.2
0.4

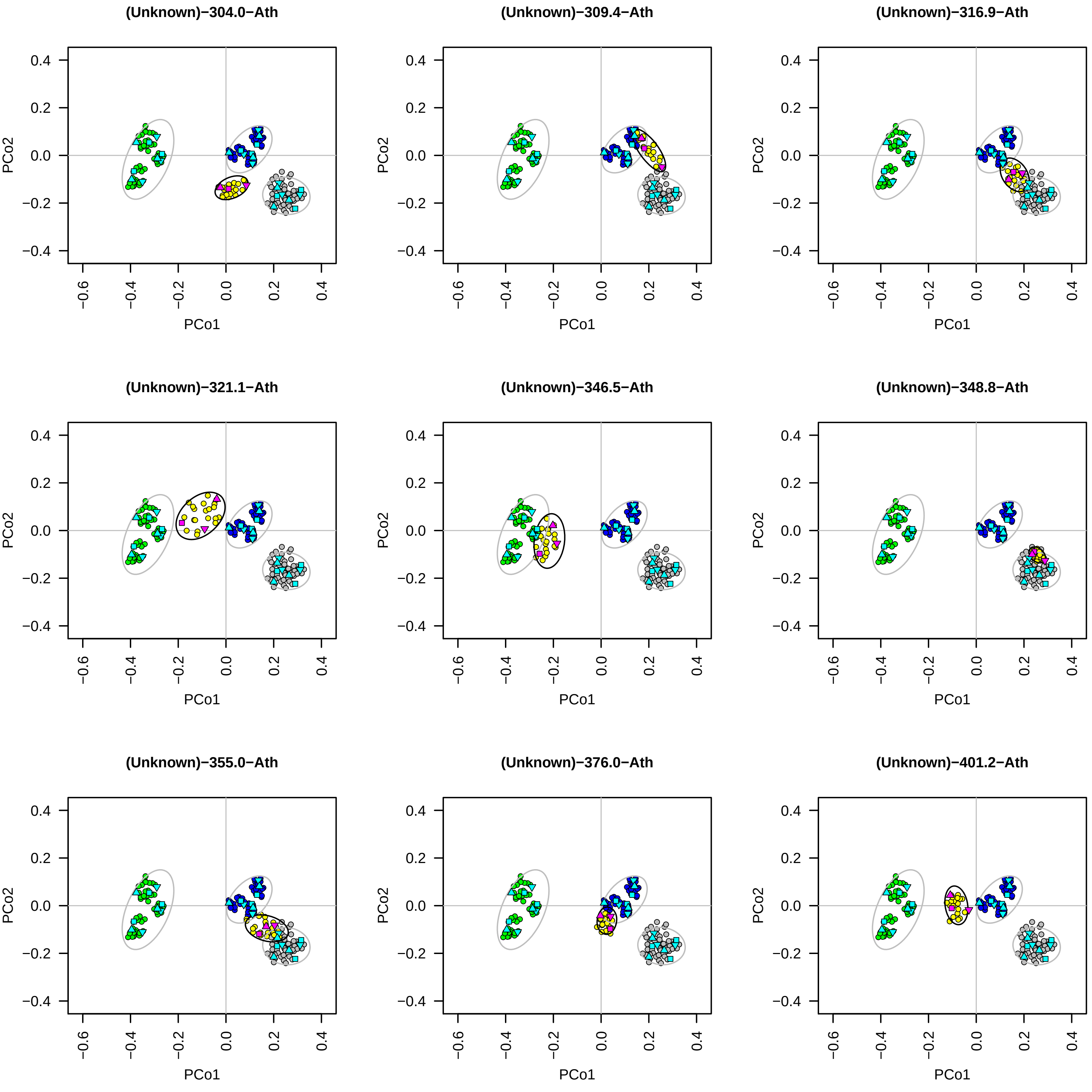
(Unknown)−304.0−Ath
(Unknown)−309.4−Ath
(Unknown)−316.9−Ath
(Unknown)−321.1−Ath
(Unknown)−346.5−Ath
(Unknown)−348.8−Ath
(Unknown)−355.0−Ath
(Unknown)−376.0−Ath
(Unknown)−401.2−Ath
PCo1
PCo2
0.4
0.2
0.0
−0.2
−0.4
−0.6

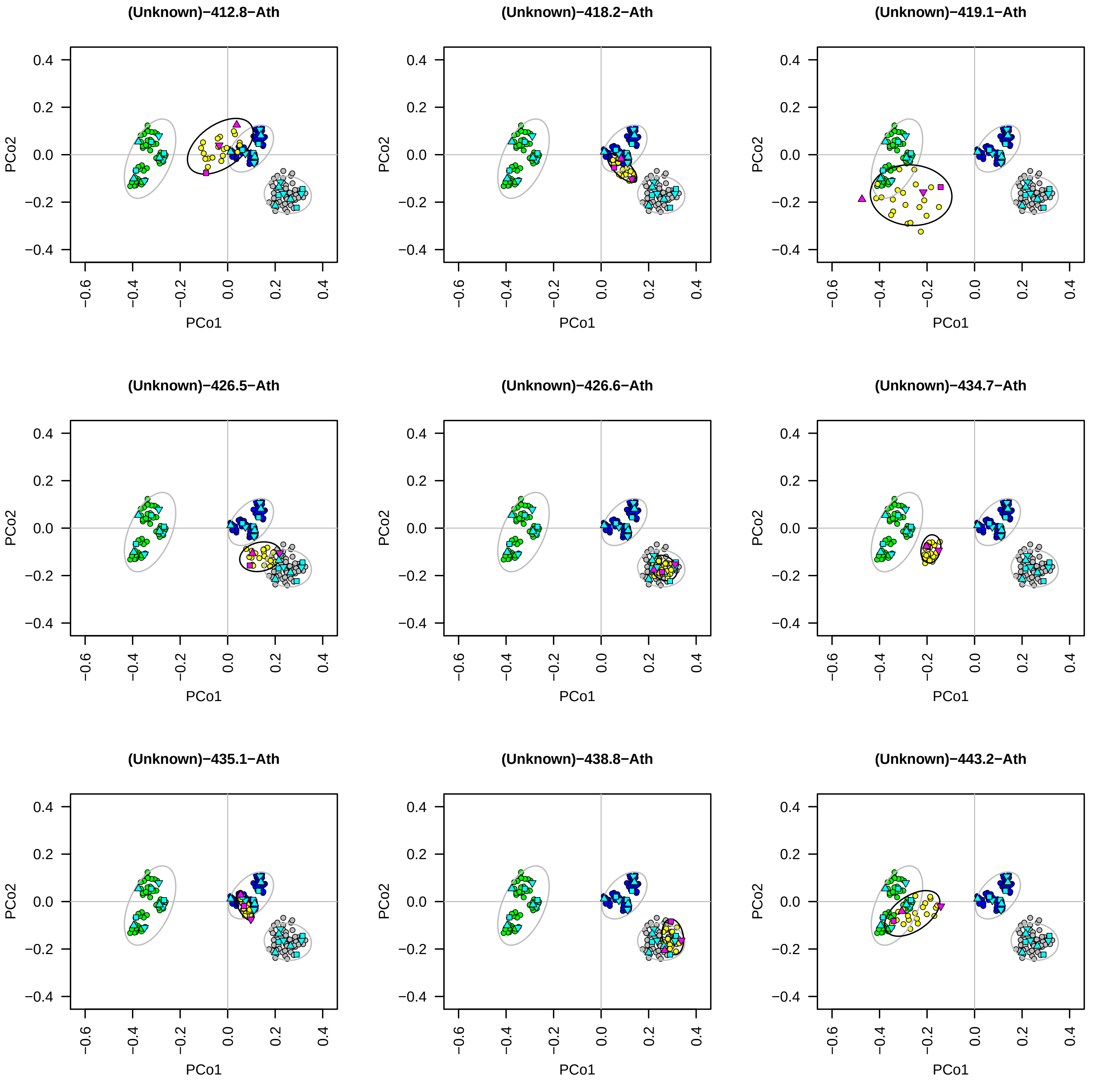
(Unknown)−412.8−Ath
(Unknown)−418.2−Ath
(Unknown)−419.1−Ath
(Unknown)−426.5−Ath
(Unknown)−426.6−Ath
(Unknown)−434.7−Ath
(Unknown)−435.1−Ath
(Unknown)−438.8−Ath
(Unknown)−443.2−Ath
PCo1
PCo2
0.4
0.2
0.0
−0.2
−0.4
−0.6

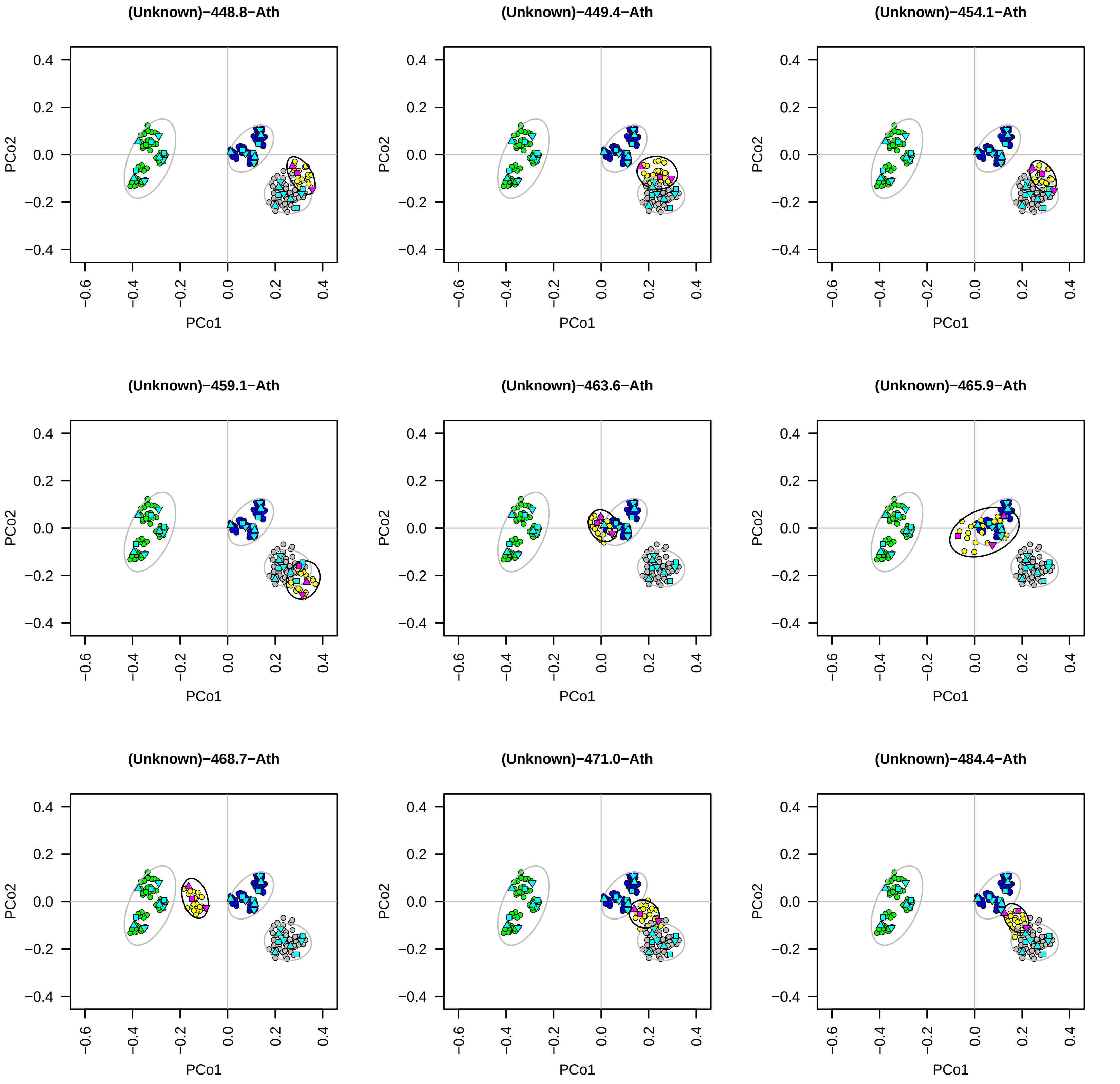
(Unknown)−448.8−Ath
(Unknown)−449.4−Ath
(Unknown)−454.1−Ath
(Unknown)−459.1−Ath
(Unknown)−463.6−Ath
(Unknown)−465.9−Ath
(Unknown)−468.7−Ath
(Unknown)−471.0−Ath
(Unknown)−484.4−Ath
PCo1
PCo2
0.4
0.2
0.0
−0.2
−0.4
−0.6

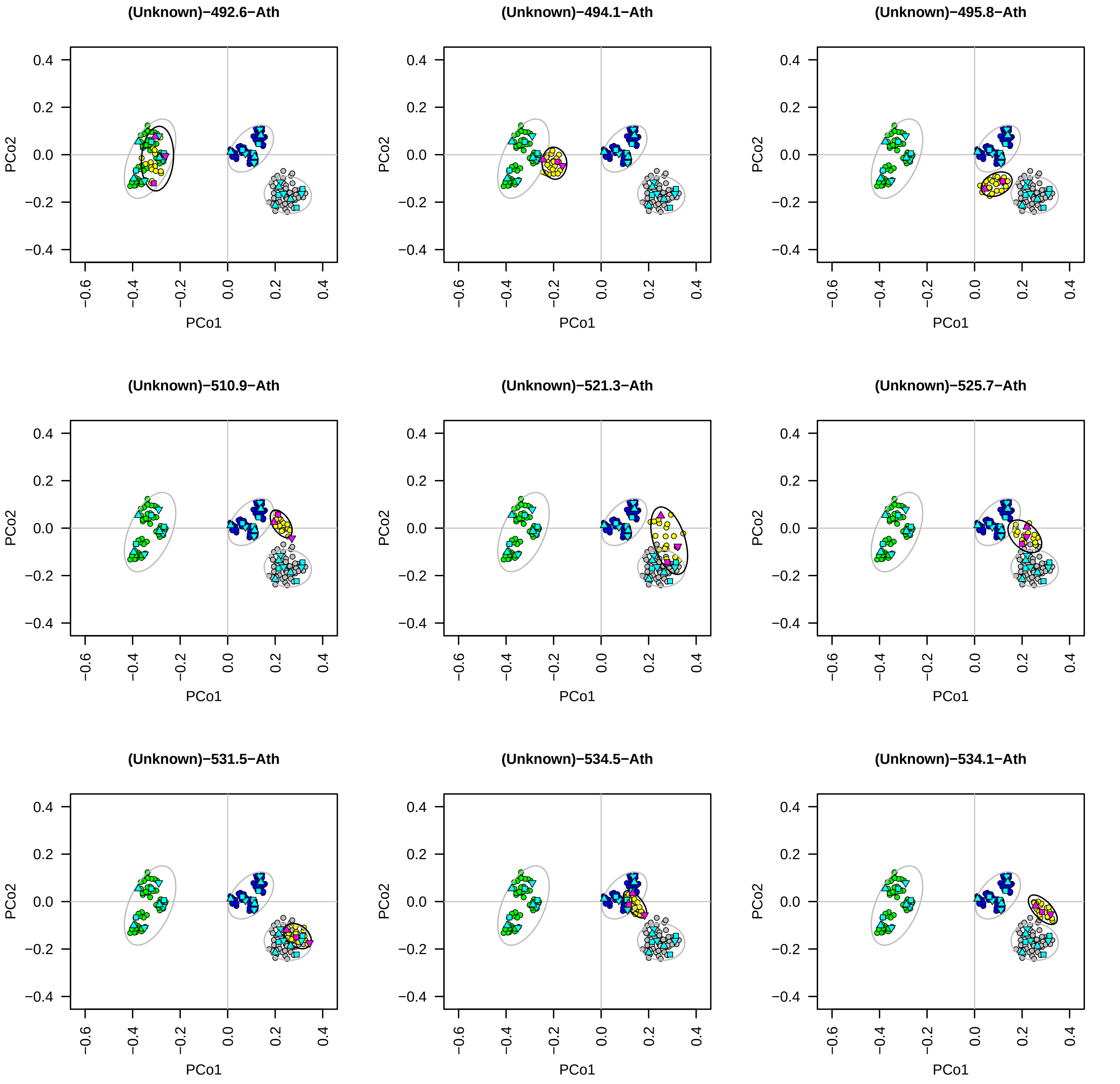
(Unknown)−492.6−Ath
(Unknown)−494.1−Ath
(Unknown)−495.8−Ath
(Unknown)−510.9−Ath
(Unknown)−521.3−Ath
(Unknown)−525.7−Ath
(Unknown)−531.5−Ath
(Unknown)−534.5−Ath
(Unknown)−534.1−Ath
PCo1
PCo2
0.4
0.2
0.0
−0.2
−0.4
−0.6

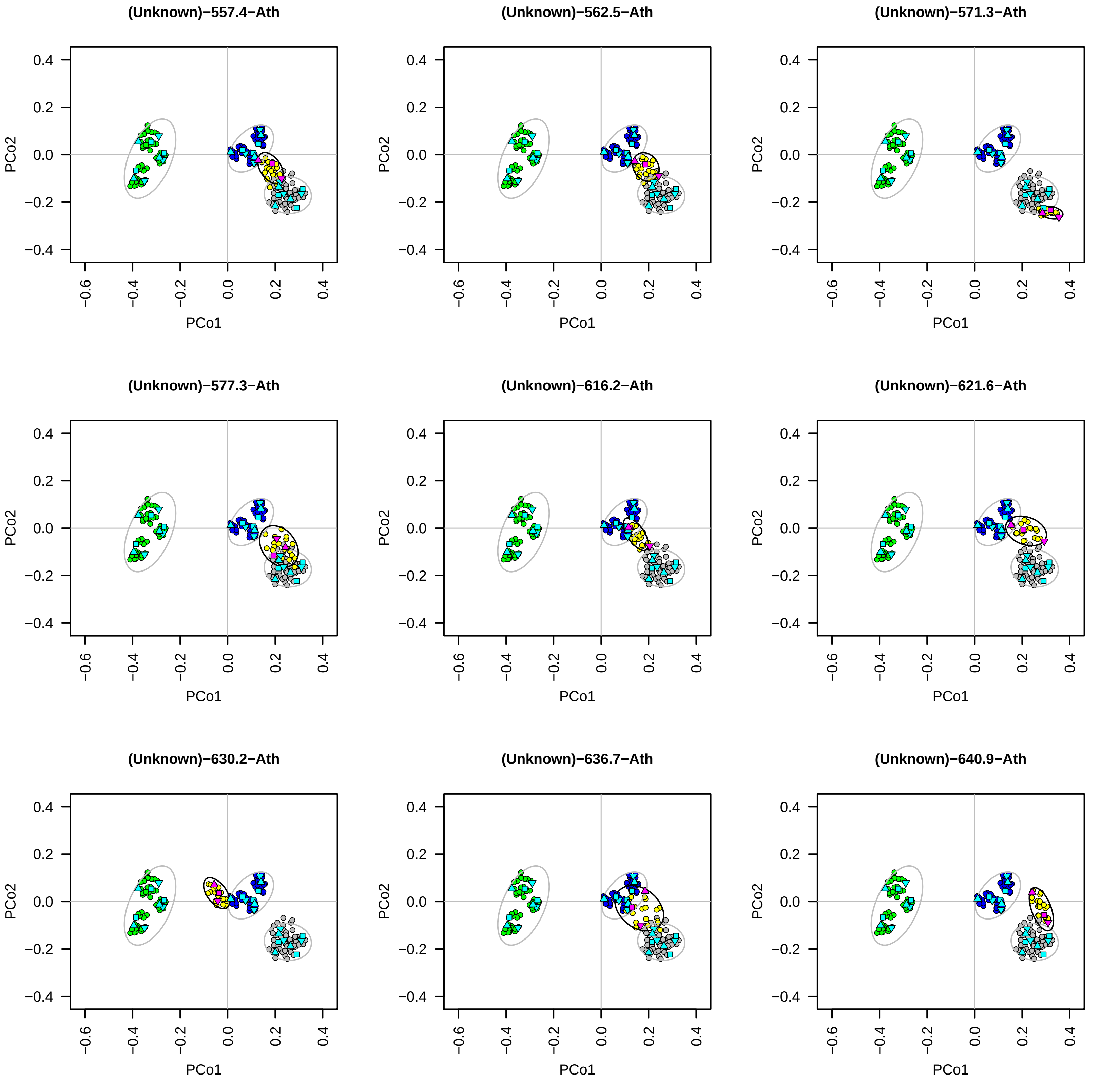
(Unknown)−557.4−Ath
(Unknown)−562.5−Ath
(Unknown)−571.3−Ath
(Unknown)−577.3−Ath
(Unknown)−616.2−Ath
(Unknown)−621.6−Ath
(Unknown)−630.2−Ath
(Unknown)−636.7−Ath
(Unknown)−640.9−Ath
PCo1
PCo2
0.4
0.2
0.0
−0.2
−0.4
−0.6

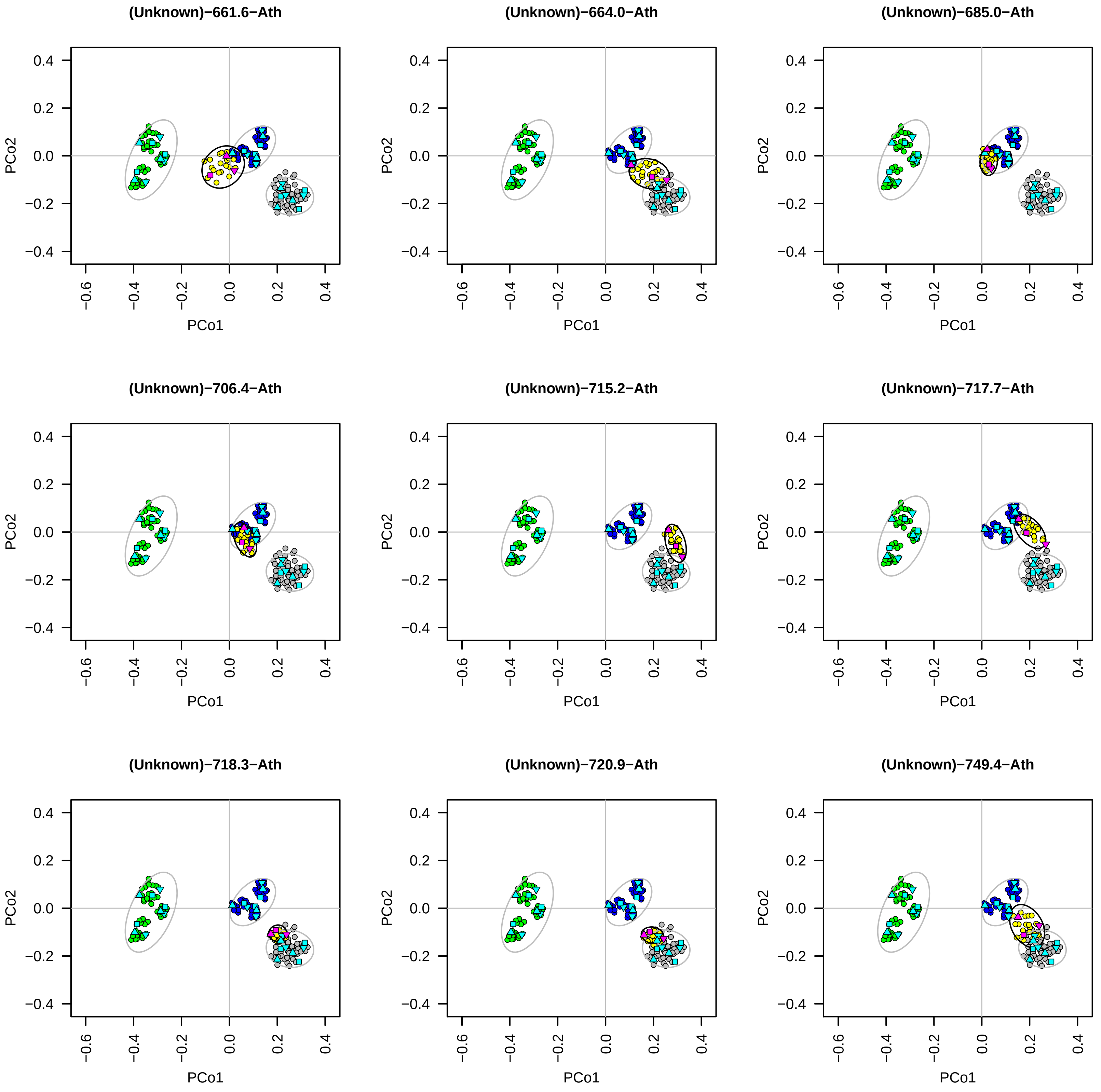
(Unknown)−661.6−Ath
(Unknown)−664.0−Ath
(Unknown)−685.0−Ath
(Unknown)−706.4−Ath
(Unknown)−715.2−Ath
(Unknown)−717.7−Ath
(Unknown)−718.3−Ath
(Unknown)−720.9−Ath
(Unknown)−749.4−Ath
PCo1
PCo2
0.4
0.2
0.0
−0.2
−0.4
−0.6

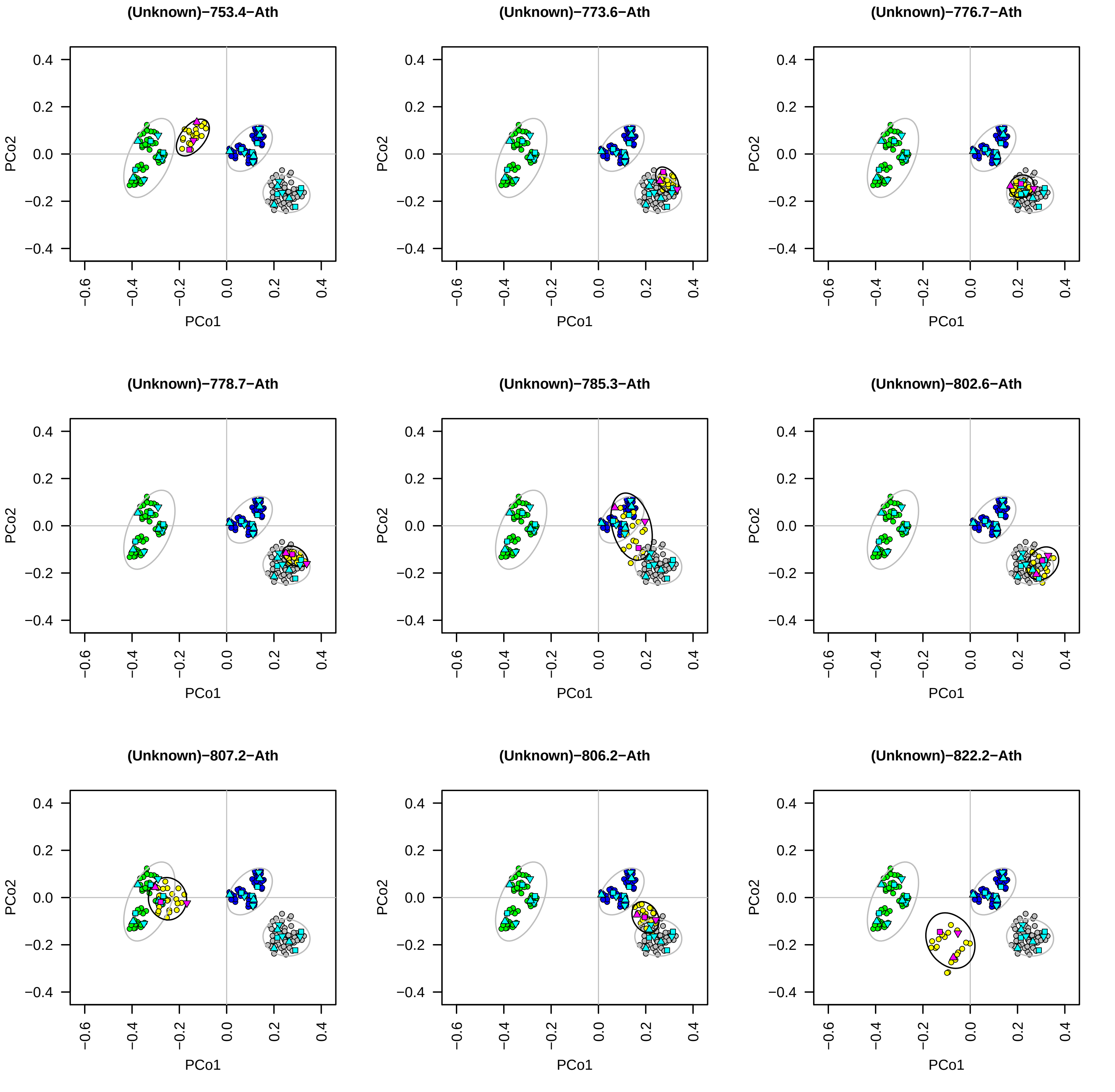

(Unknown)−753.4−Ath
(Unknown)−773.6−Ath
(Unknown)−776.7−Ath
(Unknown)−778.7−Ath
(Unknown)−785.3−Ath
(Unknown)−802.6−Ath
(Unknown)−807.2−Ath
(Unknown)−806.2−Ath
(Unknown)−822.2−Ath
PCo1
PCo2
0.4
0.2
0.0
−0.2
−0.4
−0.6

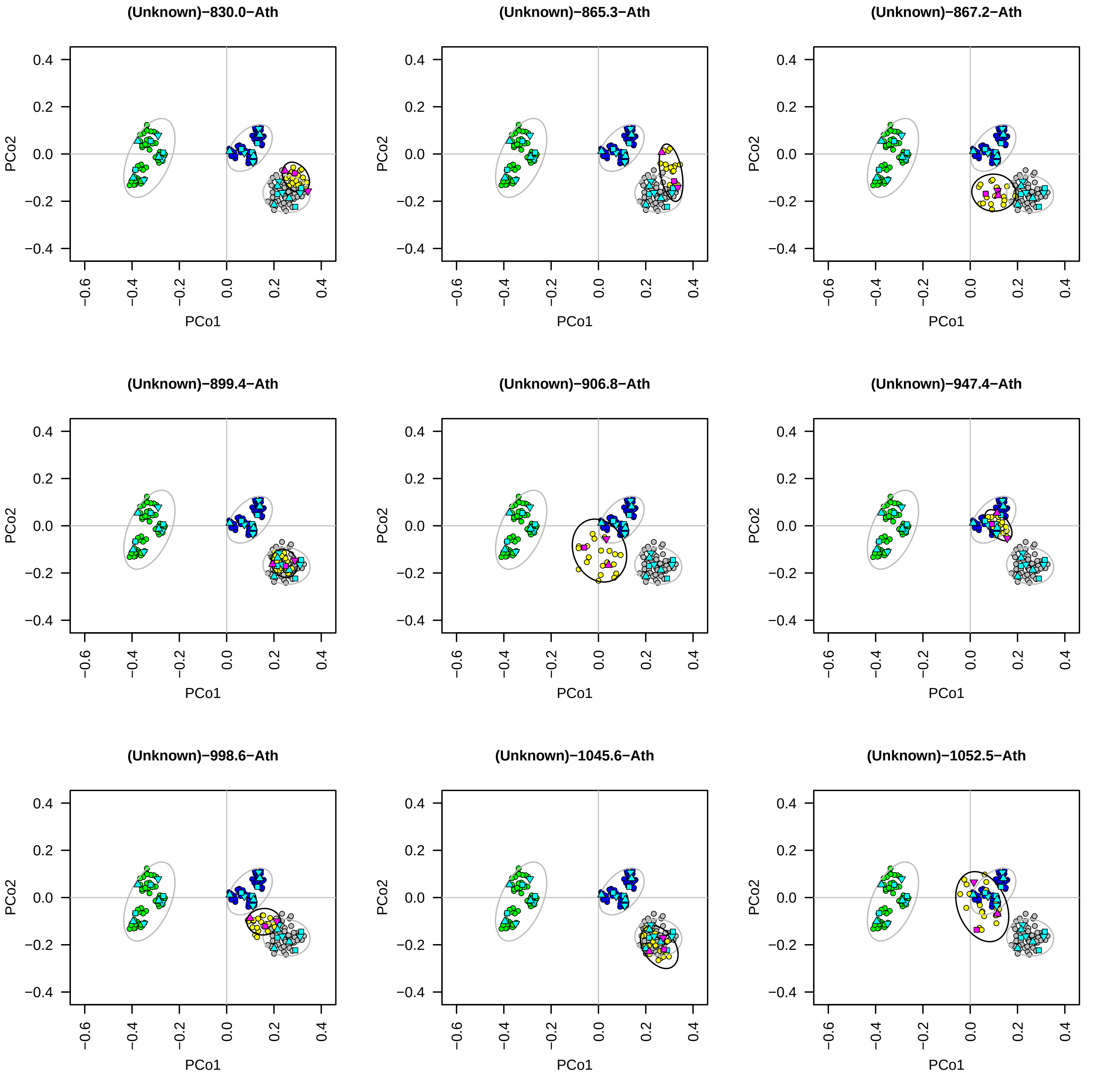
(Unknown)−830.0−Ath
(Unknown)−865.3−Ath
(Unknown)−867.2−Ath
(Unknown)−899.4−Ath
(Unknown)−906.8−Ath
(Unknown)−947.4−Ath
(Unknown)−998.6−Ath
(Unknown)−1045.6−Ath
(Unknown)−1052.5−Ath
PCo1
PCo2
−0.6
−0.4
−0.2
0.0
0.2
0.4

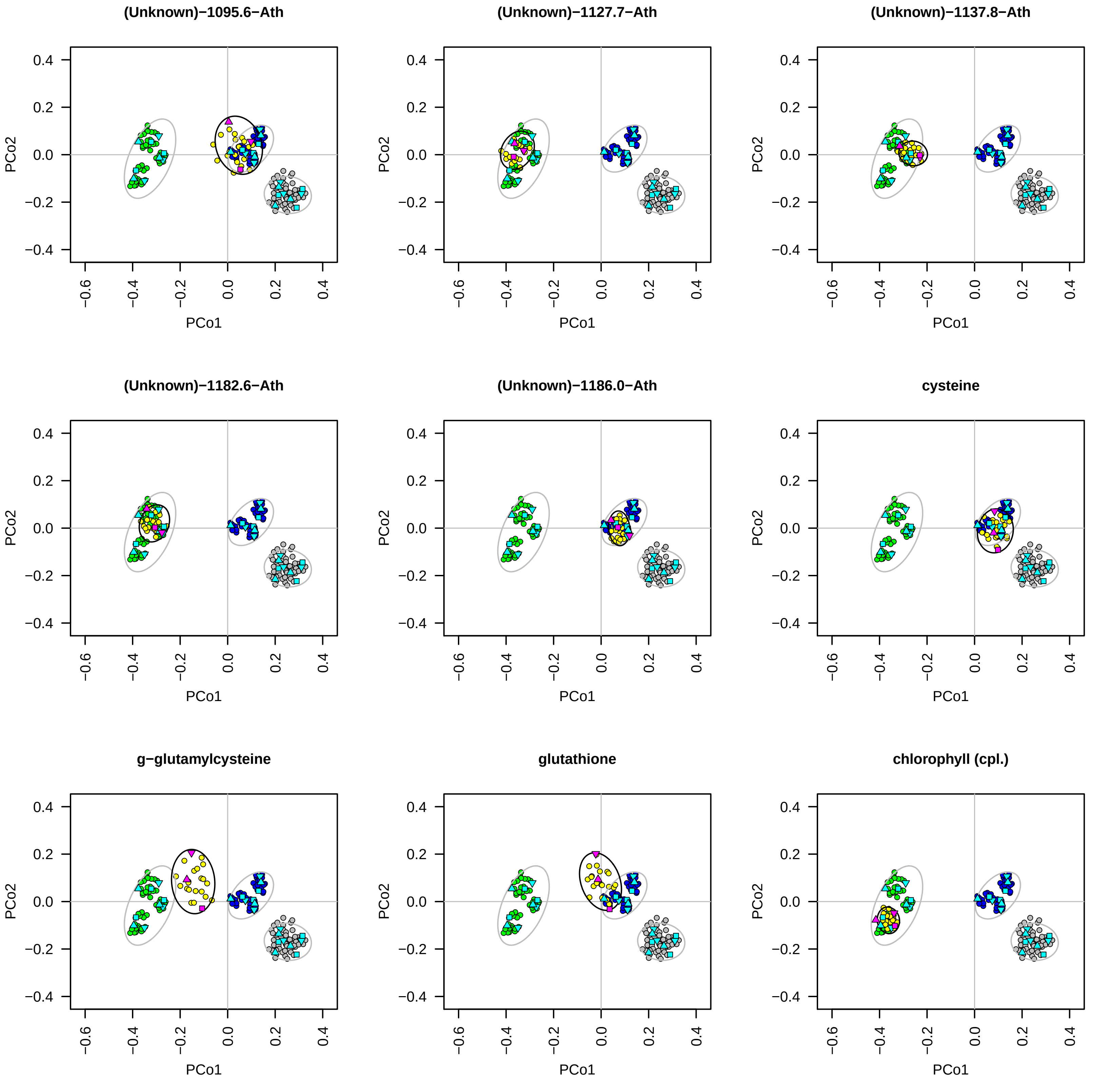

(Unknown)−1095.6−Ath
(Unknown)−1127.7−Ath
(Unknown)−1137.8−Ath
(Unknown)−1182.6−Ath
(Unknown)−1186.0−Ath
cysteine
g−glutamylcysteine
glutathione
chlorophyll (cpl.)
PCo1
PCo2

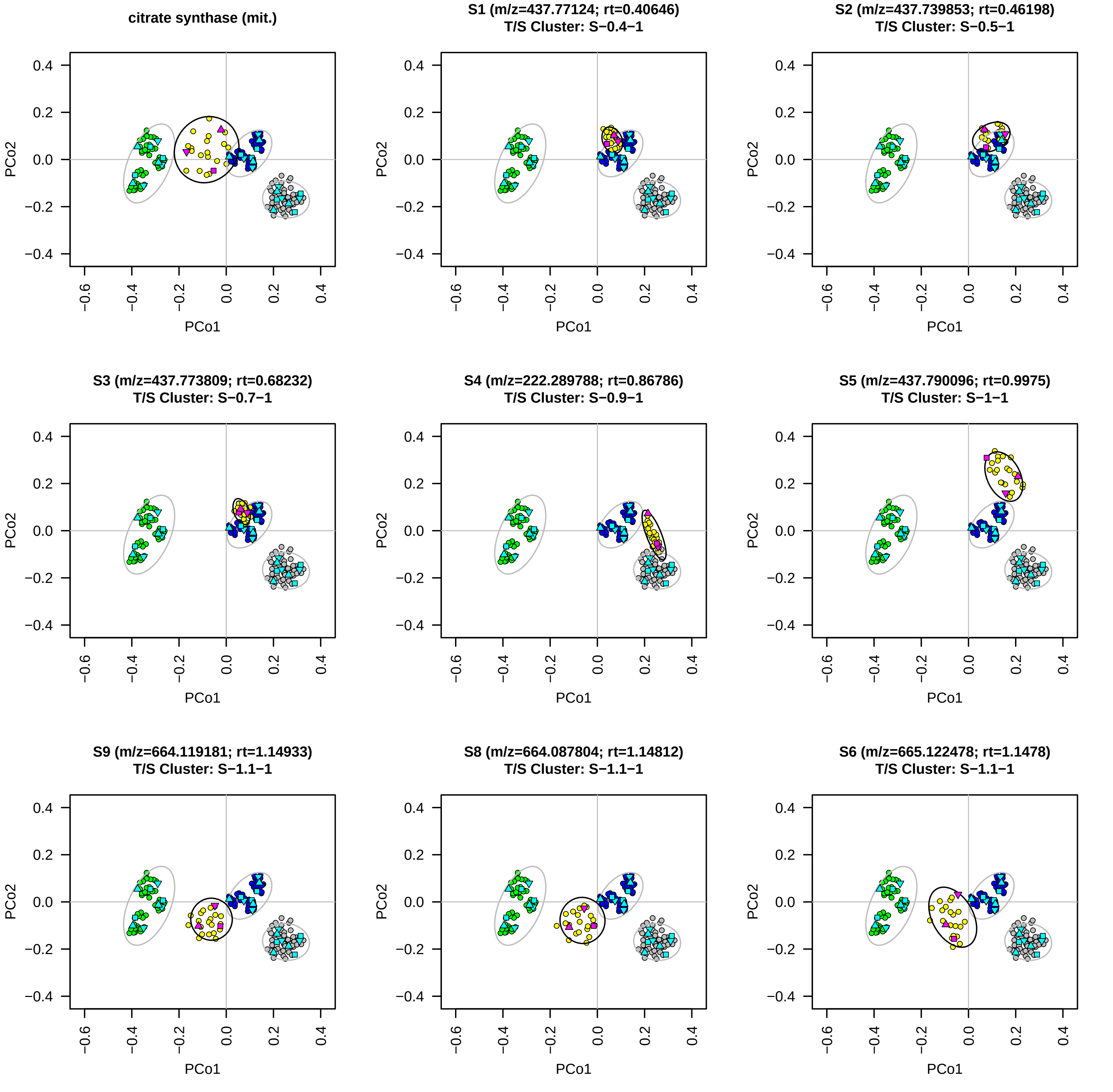
citrate synthase (mit.)
S1 (m/z=437.77124; rt=0.40646)
T/S Cluster: S−0.4−1
S2 (m/z=437.739853; rt=0.46198)
T/S Cluster: S−0.5−1
S3 (m/z=437.773809; rt=0.68232)
T/S Cluster: S−0.7−1
S4 (m/z=222.289788; rt=0.86786)
T/S Cluster: S−0.9−1
S5 (m/z=437.790096; rt=0.9975)
T/S Cluster: S−1−1
S9 (m/z=664.119181; rt=1.14933)
T/S Cluster: S−1.1−1
S8 (m/z=664.087804; rt=1.14812)
T/S Cluster: S−1.1−1
S6 (m/z=665.122478; rt=1.1478)
T/S Cluster: S−1.1−1
PCo1
PCo2
0.4
0.2
0.0
−0.2
−0.4
−0.6

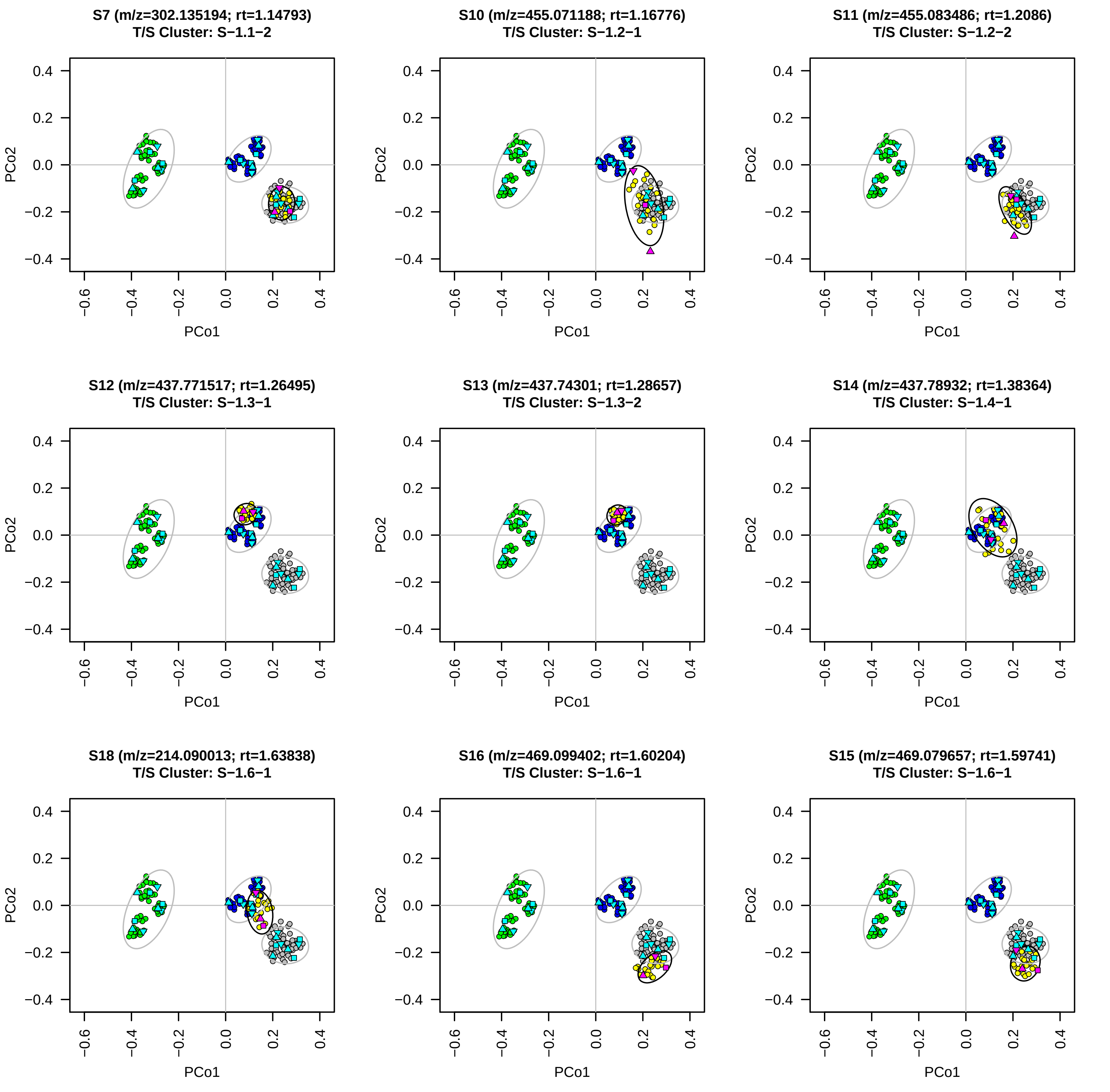
S7 (m/z=302.135194; rt=1.14793)
T/S Cluster: S−1.1−2
S10 (m/z=455.071188; rt=1.16776)
T/S Cluster: S−1.2−1
S11 (m/z=455.083486; rt=1.2086)
T/S Cluster: S−1.2−2
S12 (m/z=437.771517; rt=1.26495)
T/S Cluster: S−1.3−1
S13 (m/z=437.74301; rt=1.28657)
T/S Cluster: S−1.3−2
S14 (m/z=437.78932; rt=1.38364)
T/S Cluster: S−1.4−1
S18 (m/z=214.090013; rt=1.63838)
T/S Cluster: S−1.6−1
S16 (m/z=469.099402; rt=1.60204)
T/S Cluster: S−1.6−1
S15 (m/z=469.079657; rt=1.59741)
T/S Cluster: S−1.6−1
PCo1
PCo2

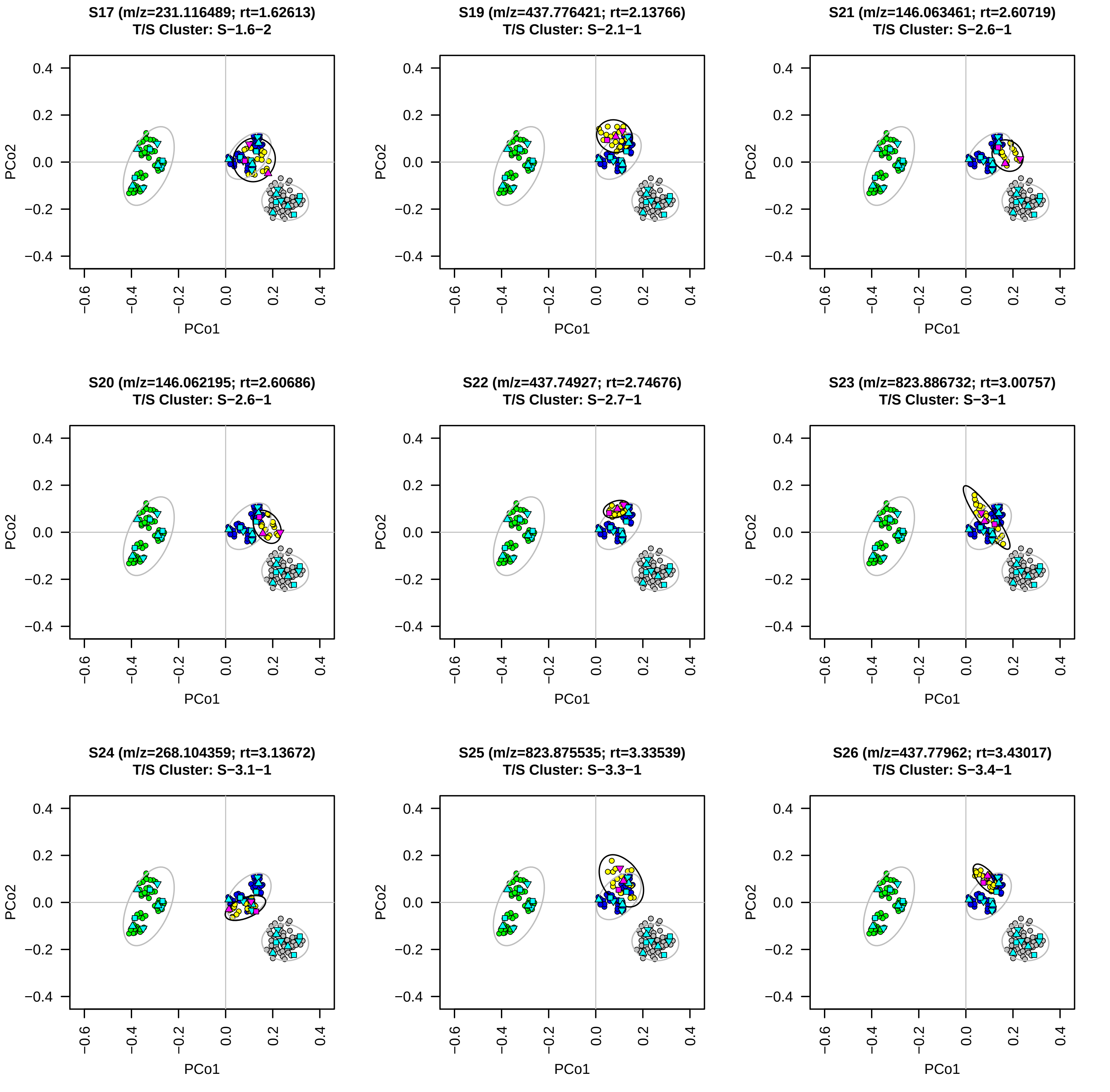

S17 (m/z=231.116489; rt=1.62613)
T/S Cluster: S−1.6−2
S19 (m/z=437.776421; rt=2.13766)
T/S Cluster: S−2.1−1
S21 (m/z=146.063461; rt=2.60719)
T/S Cluster: S−2.6−1
S20 (m/z=146.062195; rt=2.60686)
T/S Cluster: S−2.6−1
S22 (m/z=437.74927; rt=2.74676)
T/S Cluster: S−2.7−1
S23 (m/z=823.886732; rt=3.00757)
T/S Cluster: S−3−1
S24 (m/z=268.104359; rt=3.13672)
T/S Cluster: S−3.1−1
S25 (m/z=823.875535; rt=3.33539)
T/S Cluster: S−3.3−1
S26 (m/z=437.77962; rt=3.43017)
T/S Cluster: S−3.4−1
PCo1
PCo2
0.4
0.2
0.0
−0.2
−0.4
−0.6

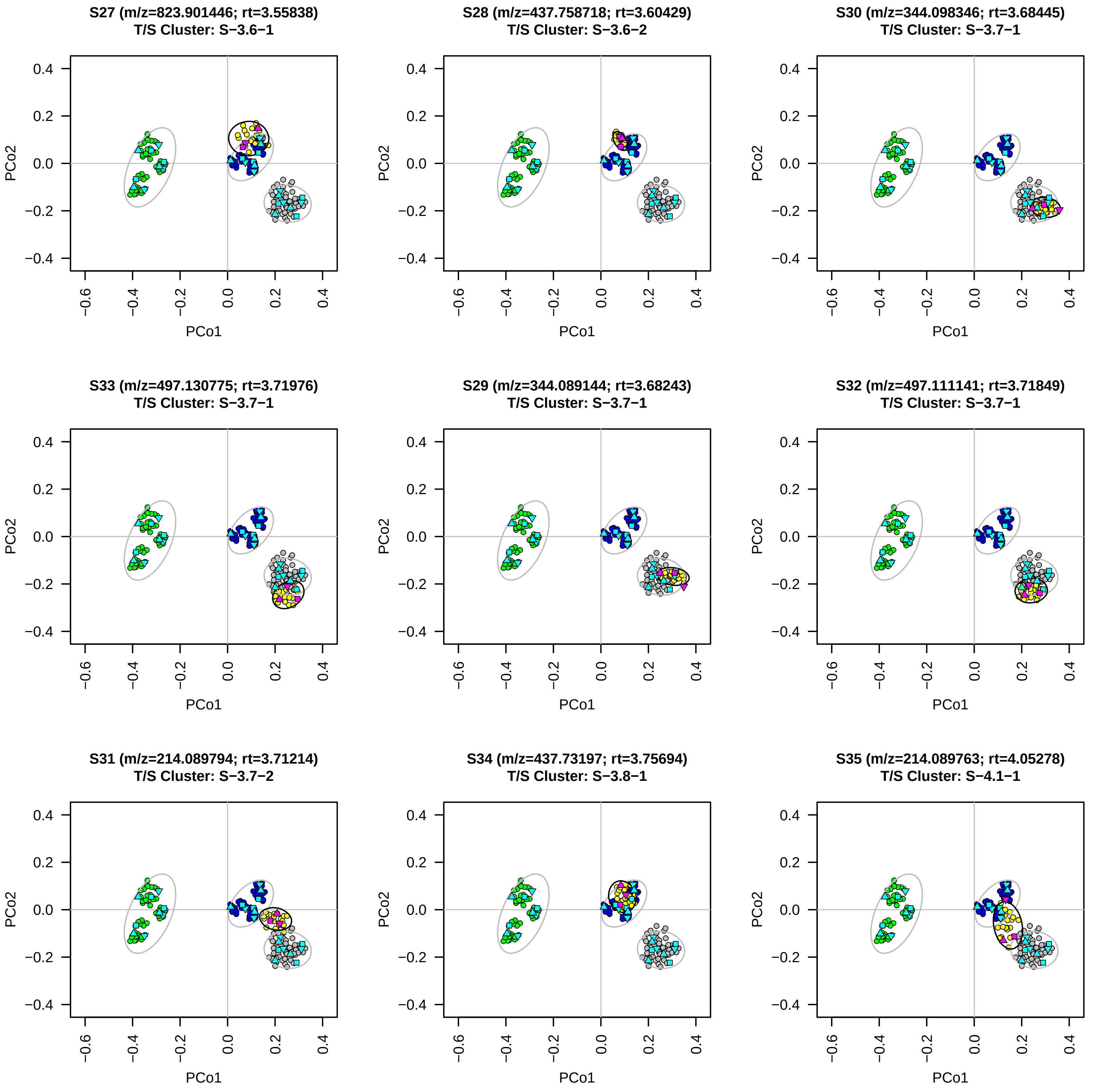
S27 (m/z=823.901446; rt=3.55838)
T/S Cluster: S−3.6−1
S28 (m/z=437.758718; rt=3.60429)
T/S Cluster: S−3.6−2
S30 (m/z=344.098346; rt=3.68445)
T/S Cluster: S−3.7−1
S33 (m/z=497.130775; rt=3.71976)
T/S Cluster: S−3.7−1
S29 (m/z=344.089144; rt=3.68243)
T/S Cluster: S−3.7−1
S32 (m/z=497.111141; rt=3.71849)
T/S Cluster: S−3.7−1
S31 (m/z=214.089794; rt=3.71214)
T/S Cluster: S−3.7−2
S34 (m/z=437.73197; rt=3.75694)
T/S Cluster: S−3.8−1
S35 (m/z=214.089763; rt=4.05278)
T/S Cluster: S−4.1−1
PCo1
PCo2
−0.6
−0.4
−0.2
0.0
0.2
0.4

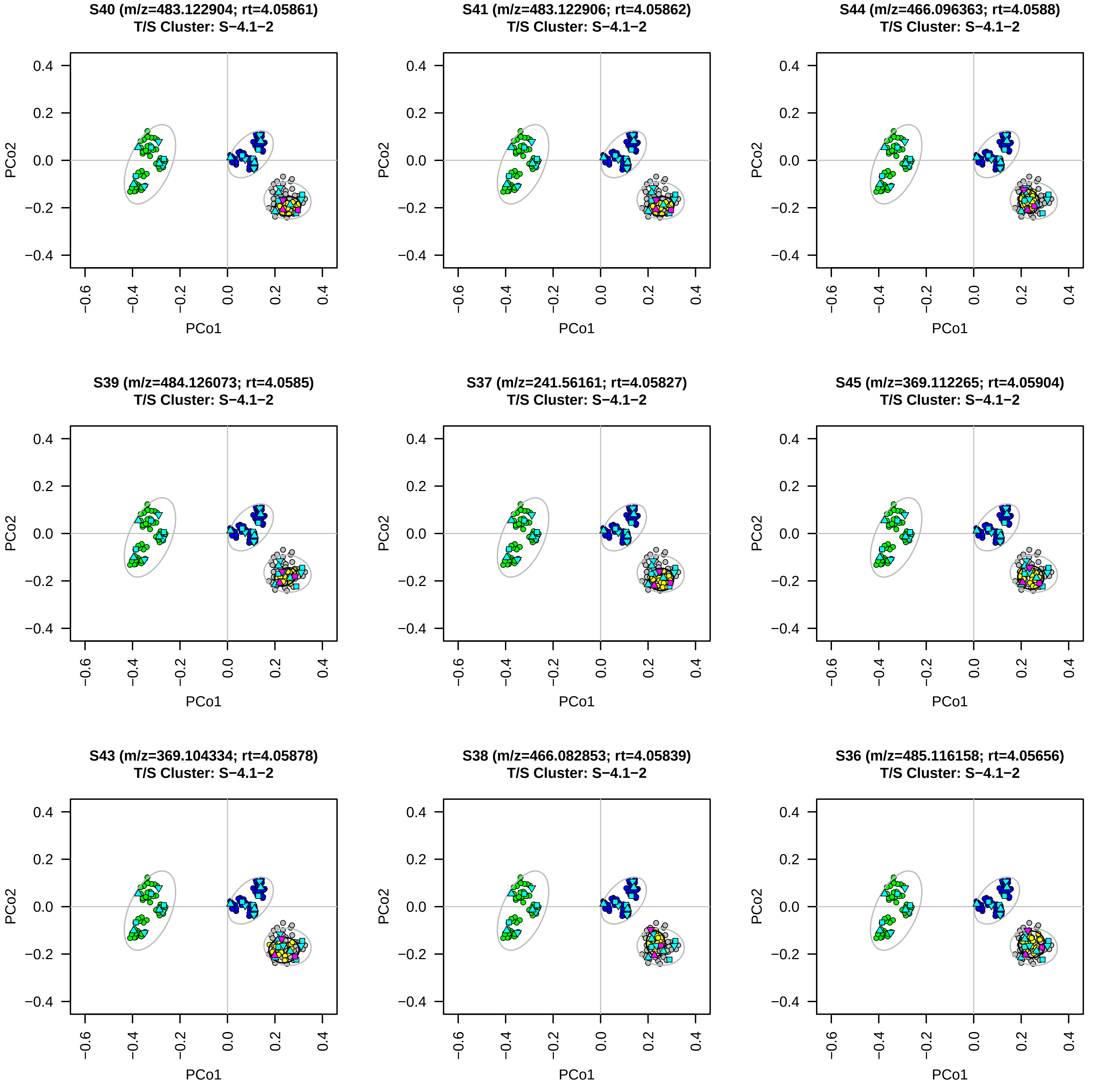
S40 (m/z=483.122904; rt=4.05861)
T/S Cluster: S−4.1−2
S41 (m/z=483.122906; rt=4.05862)
T/S Cluster: S−4.1−2
S44 (m/z=466.096363; rt=4.0588)
T/S Cluster: S−4.1−2
S39 (m/z=484.126073; rt=4.0585)
T/S Cluster: S−4.1−2
S37 (m/z=241.56161; rt=4.05827)
T/S Cluster: S−4.1−2
S45 (m/z=369.112265; rt=4.05904)
T/S Cluster: S−4.1−2
S43 (m/z=369.104334; rt=4.05878)
T/S Cluster: S−4.1−2
S38 (m/z=466.082853; rt=4.05839)
T/S Cluster: S−4.1−2
S36 (m/z=485.116158; rt=4.05656)
T/S Cluster: S−4.1−2
PCo1
PCo2
−0.6
−0.4
−0.2
0.0
0.2
0.4

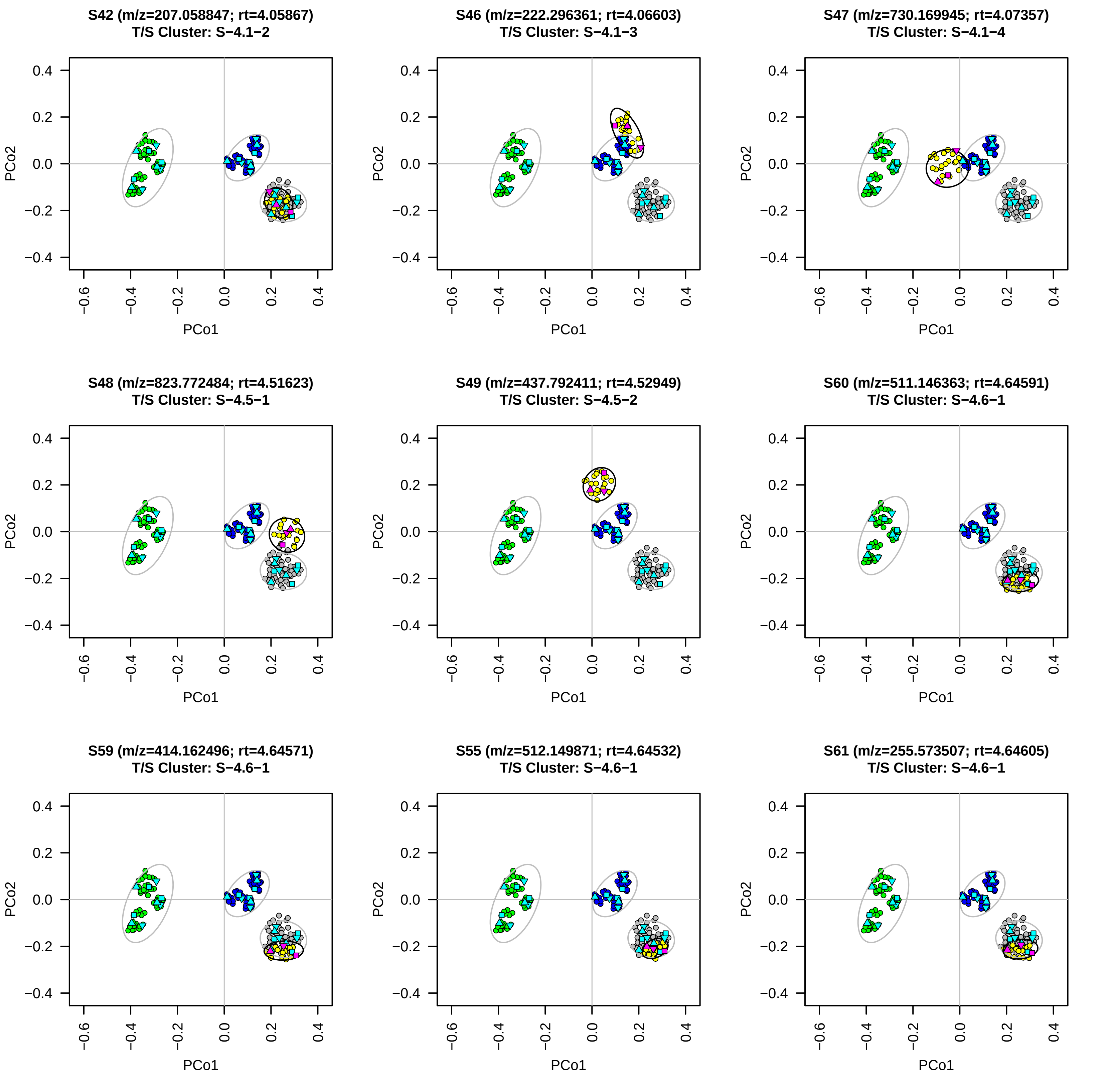
S42 (m/z=207.058847; rt=4.05867)
T/S Cluster: S−4.1−2
S46 (m/z=222.296361; rt=4.06603)
T/S Cluster: S−4.1−3
S47 (m/z=730.169945; rt=4.07357)
T/S Cluster: S−4.1−4
S48 (m/z=823.772484; rt=4.51623)
T/S Cluster: S−4.5−1
S49 (m/z=437.792411; rt=4.52949)
T/S Cluster: S−4.5−2
S60 (m/z=511.146363; rt=4.64591)
T/S Cluster: S−4.6−1
S59 (m/z=414.162496; rt=4.64571)
T/S Cluster: S−4.6−1
S55 (m/z=512.149871; rt=4.64532)
T/S Cluster: S−4.6−1
S61 (m/z=255.573507; rt=4.64605)
T/S Cluster: S−4.6−1
PCo1
PCo2
−0.6
−0.4
−0.2
0.0
0.2
0.4

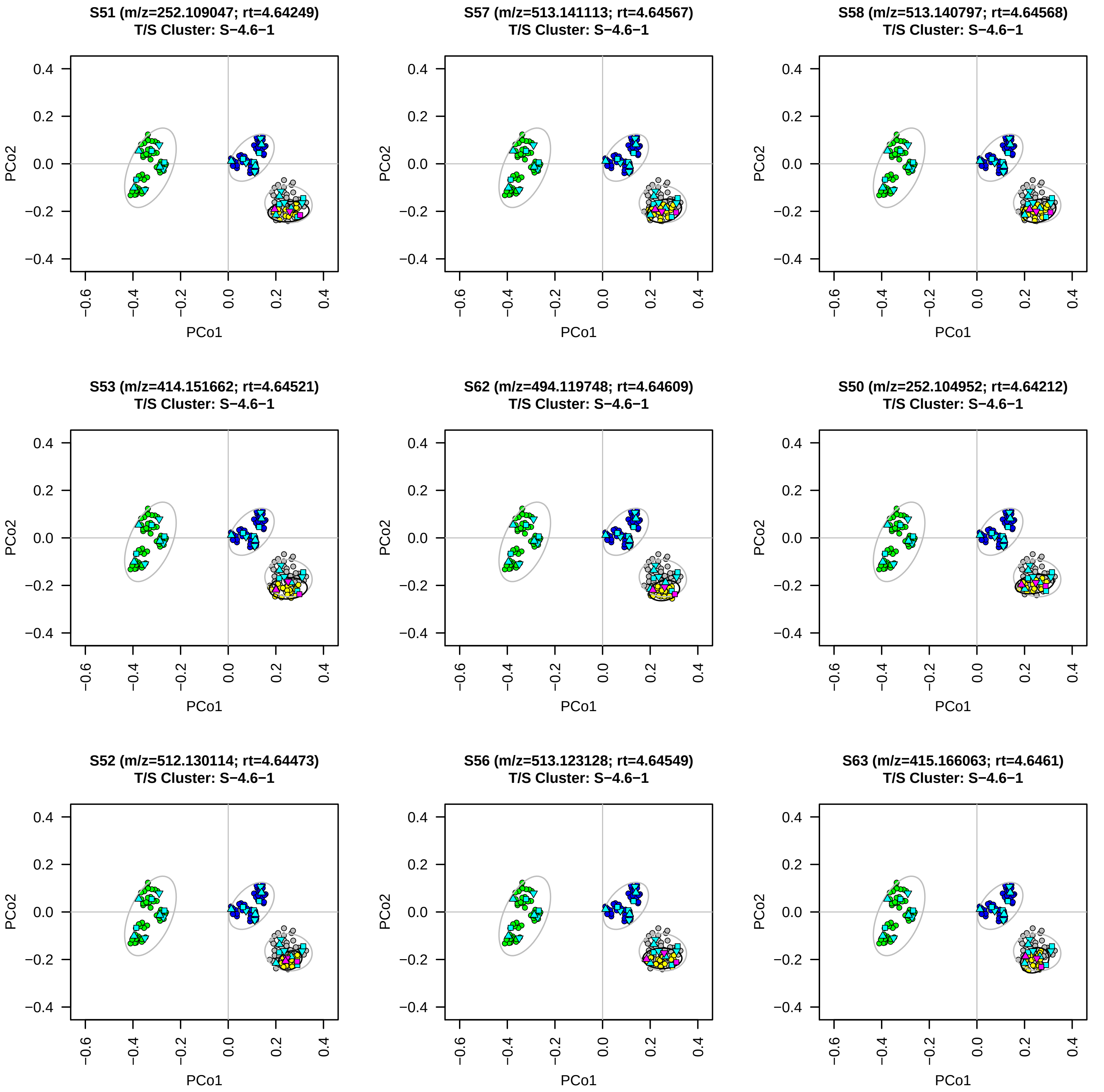
S51 (m/z=252.109047; rt=4.64249)
T/S Cluster: S−4.6−1
S57 (m/z=513.141113; rt=4.64567)
T/S Cluster: S−4.6−1
S58 (m/z=513.140797; rt=4.64568)
T/S Cluster: S−4.6−1
S53 (m/z=414.151662; rt=4.64521)
T/S Cluster: S−4.6−1
S62 (m/z=494.119748; rt=4.64609)
T/S Cluster: S−4.6−1
S50 (m/z=252.104952; rt=4.64212)
T/S Cluster: S−4.6−1
S52 (m/z=512.130114; rt=4.64473)
T/S Cluster: S−4.6−1
S56 (m/z=513.123128; rt=4.64549)
T/S Cluster: S−4.6−1
S63 (m/z=415.166063; rt=4.6461)
T/S Cluster: S−4.6−1
PCo1
PCo2

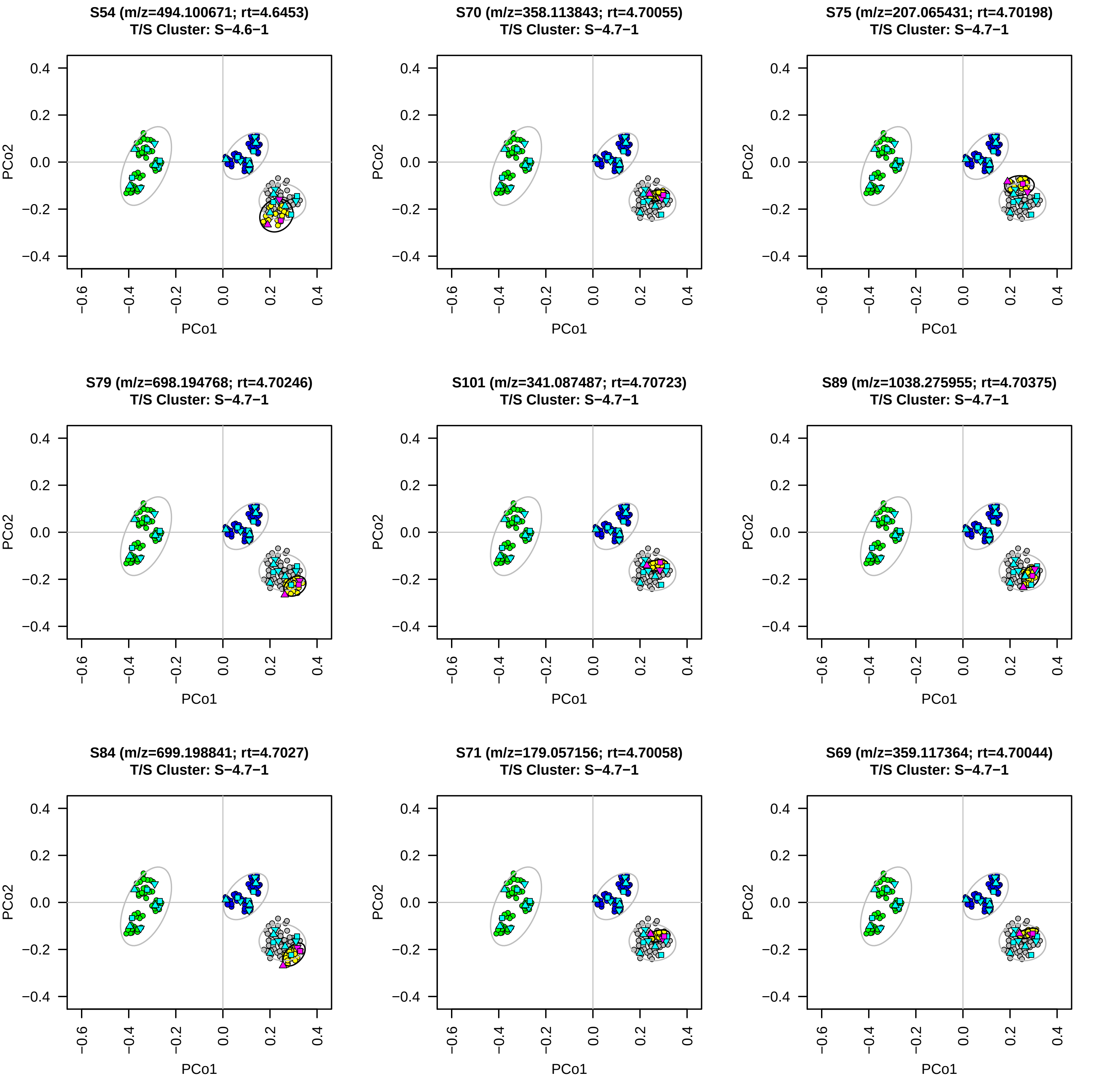
S54 (m/z=494.100671; rt=4.6453)
T/S Cluster: S−4.6−1
S70 (m/z=358.113843; rt=4.70055)
T/S Cluster: S−4.7−1
S75 (m/z=207.065431; rt=4.70198)
T/S Cluster: S−4.7−1
S79 (m/z=698.194768; rt=4.70246)
T/S Cluster: S−4.7−1
S101 (m/z=341.087487; rt=4.70723)
T/S Cluster: S−4.7−1
S89 (m/z=1038.275955; rt=4.70375)
T/S Cluster: S−4.7−1
S84 (m/z=699.198841; rt=4.7027)
T/S Cluster: S−4.7−1
S71 (m/z=179.057156; rt=4.70058)
T/S Cluster: S−4.7−1
S69 (m/z=359.117364; rt=4.70044)
T/S Cluster: S−4.7−1
PCo1
PCo2
0.4
0.2
0.0
−0.2
−0.4
−0.6

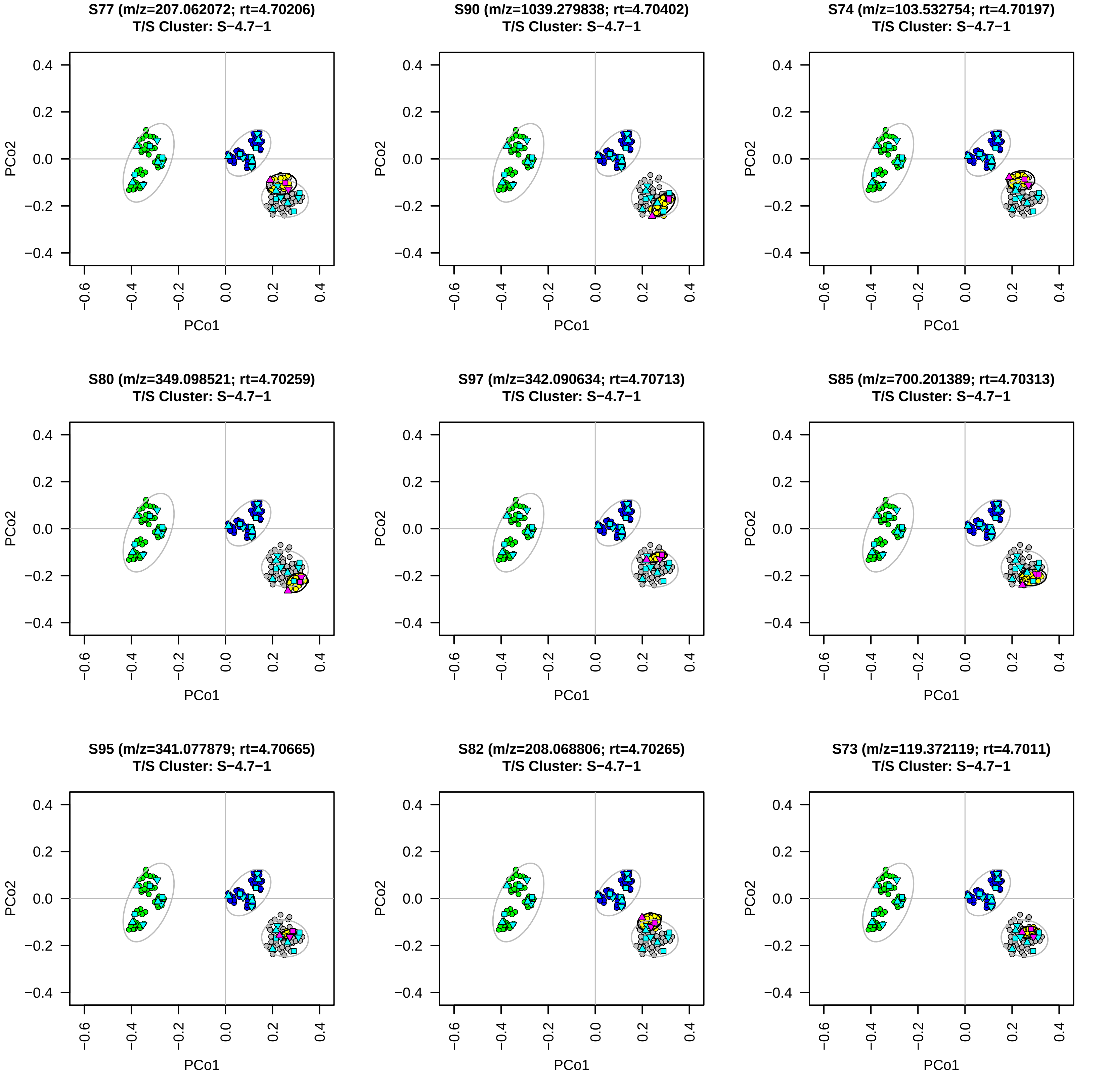
S77 (m/z=207.062072; rt=4.70206)
T/S Cluster: S−4.7−1
S90 (m/z=1039.279838; rt=4.70402)
T/S Cluster: S−4.7−1
S74 (m/z=103.532754; rt=4.70197)
T/S Cluster: S−4.7−1
S80 (m/z=349.098521; rt=4.70259)
T/S Cluster: S−4.7−1
S97 (m/z=342.090634; rt=4.70713)
T/S Cluster: S−4.7−1
S85 (m/z=700.201389; rt=4.70313)
T/S Cluster: S−4.7−1
S95 (m/z=341.077879; rt=4.70665)
T/S Cluster: S−4.7−1
S82 (m/z=208.068806; rt=4.70265)
T/S Cluster: S−4.7−1
S73 (m/z=119.372119; rt=4.7011)
T/S Cluster: S−4.7−1
PCo1
PCo2
−0.6
−0.4
−0.2
0.0
0.2
0.4

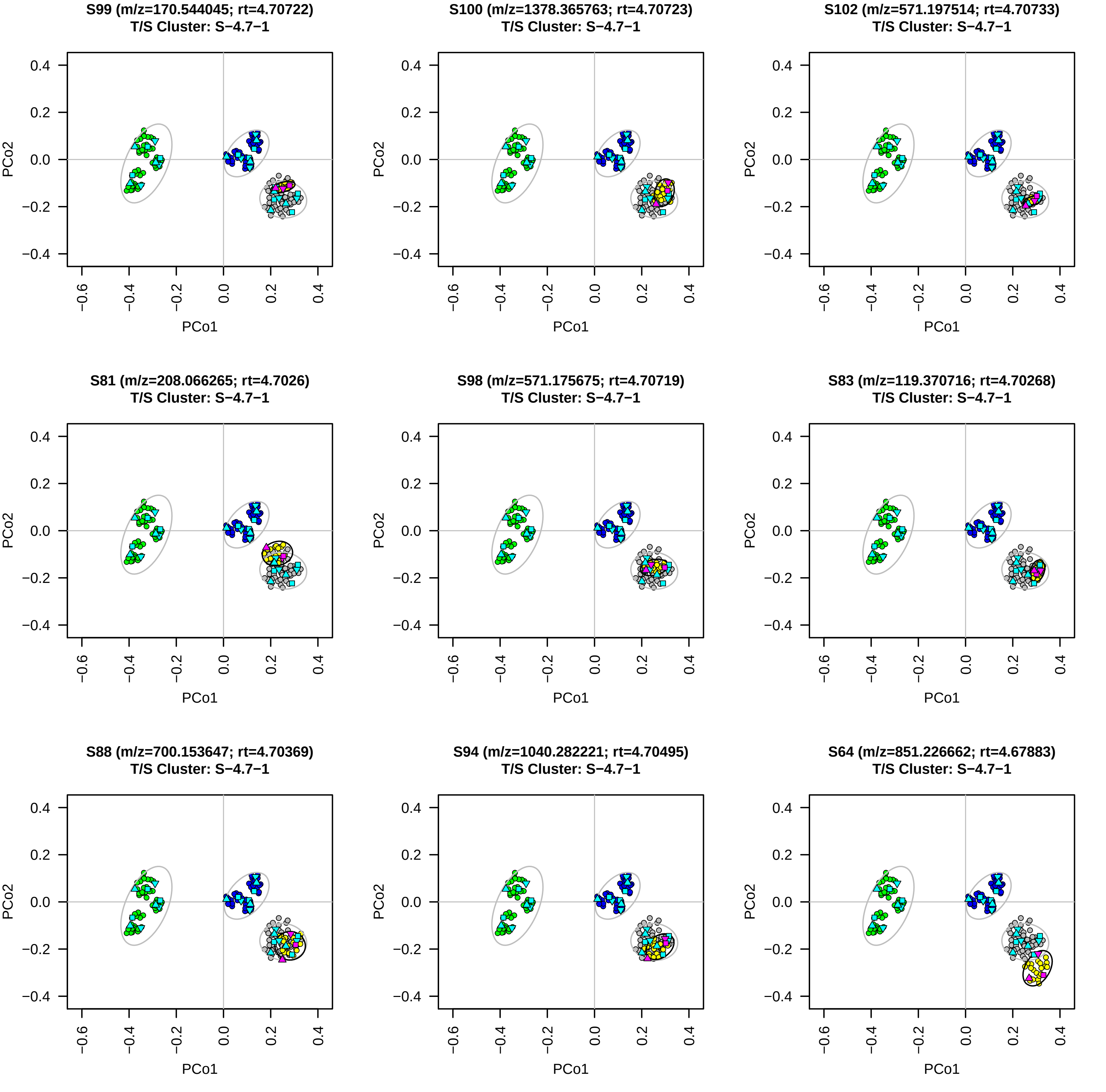

S99 (m/z=170.544045; rt=4.70722)
T/S Cluster: S−4.7−1
S100 (m/z=1378.365763; rt=4.70723)
T/S Cluster: S−4.7−1
S102 (m/z=571.197514; rt=4.70733)
T/S Cluster: S−4.7−1
S81 (m/z=208.066265; rt=4.7026)
T/S Cluster: S−4.7−1
S98 (m/z=571.175675; rt=4.70719)
T/S Cluster: S−4.7−1
S83 (m/z=119.370716; rt=4.70268)
T/S Cluster: S−4.7−1
S88 (m/z=700.153647; rt=4.70369)
T/S Cluster: S−4.7−1
S94 (m/z=1040.282221; rt=4.70495)
T/S Cluster: S−4.7−1
S64 (m/z=851.226662; rt=4.67883)
T/S Cluster: S−4.7−1
PCo1
PCo2
−0.6 −0.4 −0.2 0.0 0.2 0.4
−0.4 −0.2 0.0 0.2 0.4

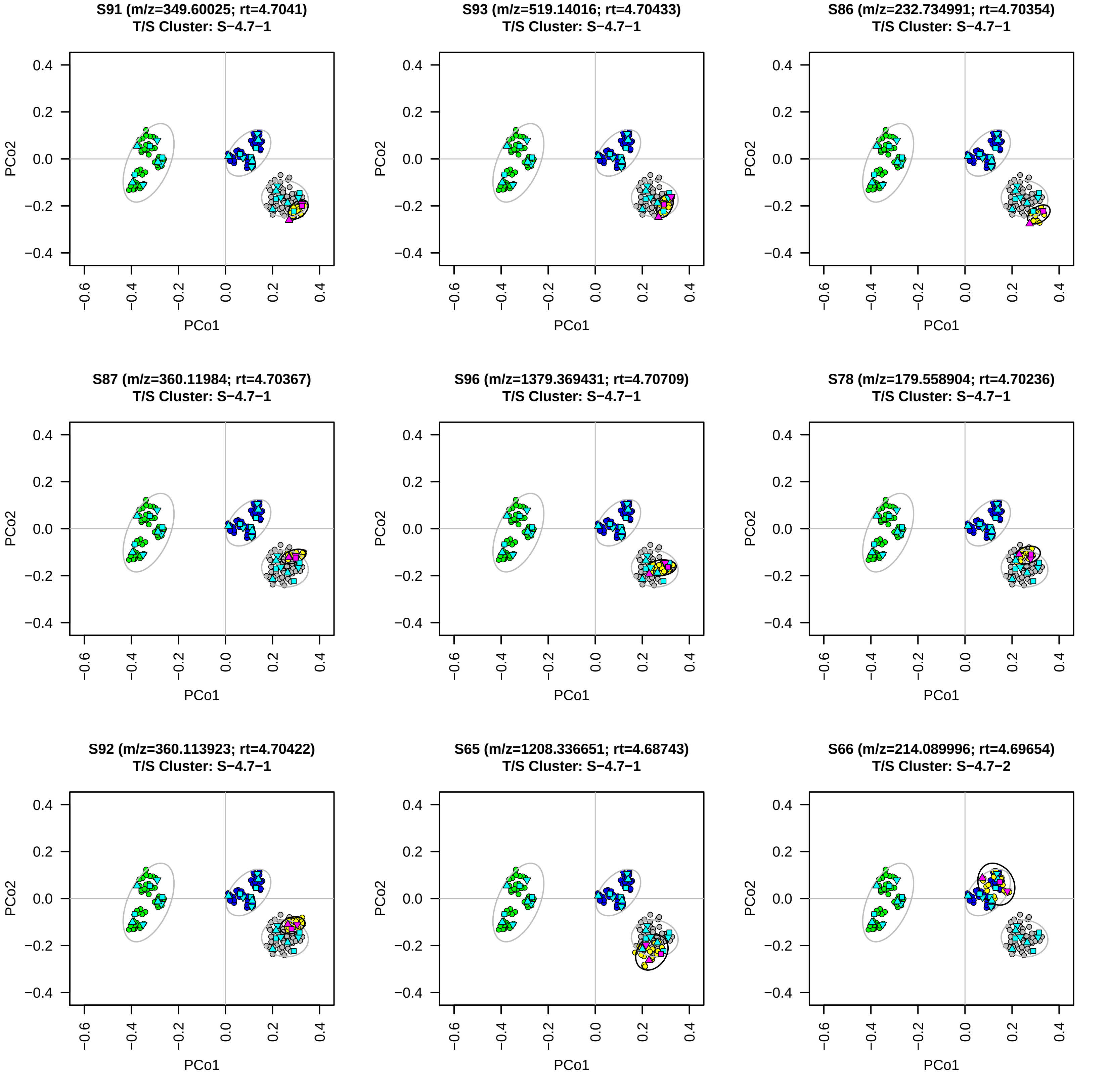
S91 (m/z=349.60025; rt=4.7041)
T/S Cluster: S−4.7−1
S93 (m/z=519.14016; rt=4.70433)
T/S Cluster: S−4.7−1
S86 (m/z=232.734991; rt=4.70354)
T/S Cluster: S−4.7−1
S87 (m/z=360.11984; rt=4.70367)
T/S Cluster: S−4.7−1
S96 (m/z=1379.369431; rt=4.70709)
T/S Cluster: S−4.7−1
S78 (m/z=179.558904; rt=4.70236)
T/S Cluster: S−4.7−1
S92 (m/z=360.113923; rt=4.70422)
T/S Cluster: S−4.7−1
S65 (m/z=1208.336651; rt=4.68743)
T/S Cluster: S−4.7−1
S66 (m/z=214.089996; rt=4.69654)
T/S Cluster: S−4.7−2
PCo1
PCo2
0.4
0.2
0.0
−0.2
−0.4
−0.6

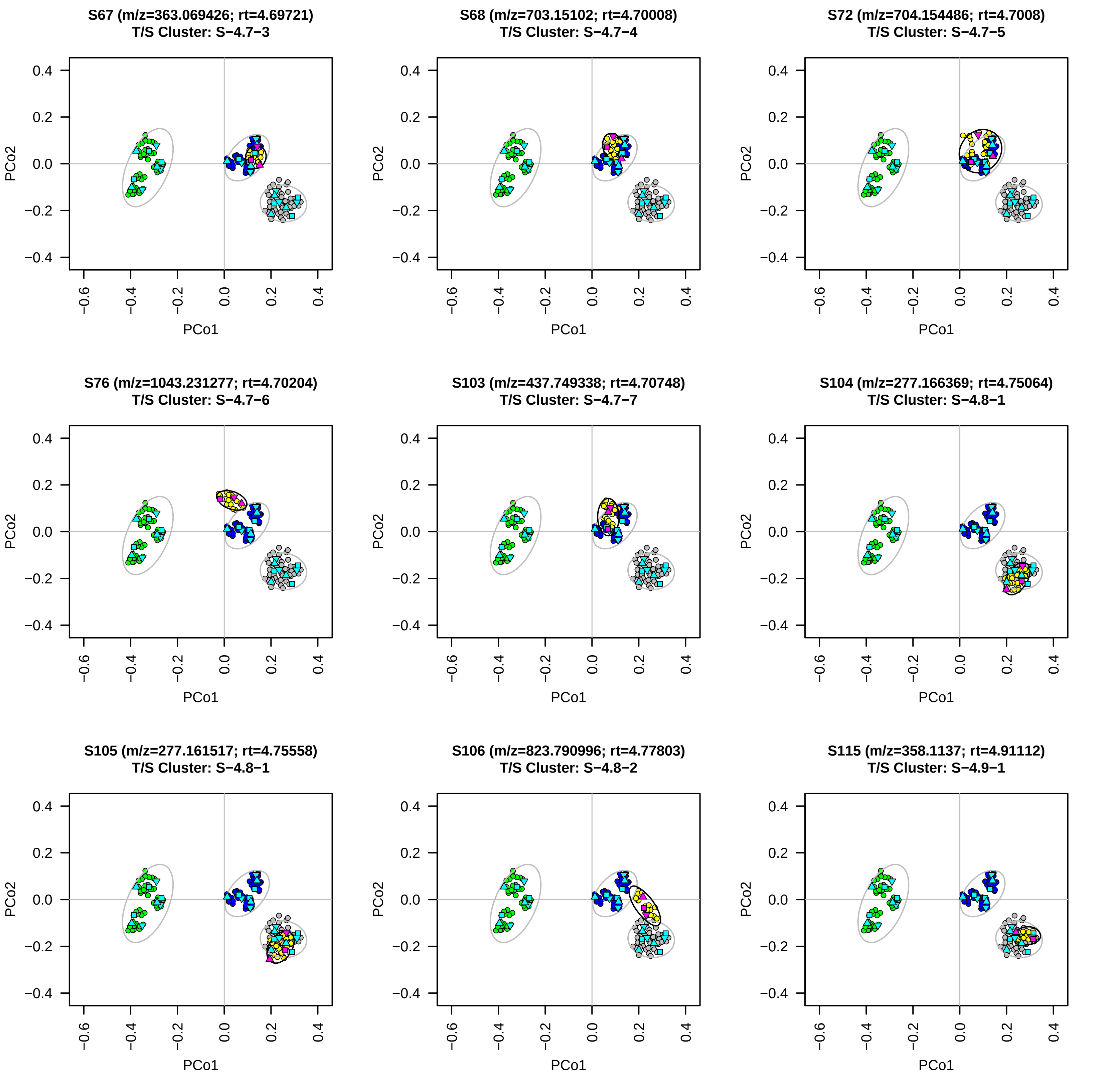
S67 (m/z=363.069426; rt=4.69721)
T/S Cluster: S−4.7−3
S68 (m/z=703.15102; rt=4.70008)
T/S Cluster: S−4.7−4
S72 (m/z=704.154486; rt=4.7008)
T/S Cluster: S−4.7−5
S76 (m/z=1043.231277; rt=4.70204)
T/S Cluster: S−4.7−6
S103 (m/z=437.749338; rt=4.70748)
T/S Cluster: S−4.7−7
S104 (m/z=277.166369; rt=4.75064)
T/S Cluster: S−4.8−1
S105 (m/z=277.161517; rt=4.75558)
T/S Cluster: S−4.8−1
S106 (m/z=823.790996; rt=4.77803)
T/S Cluster: S−4.8−2
S115 (m/z=358.1137; rt=4.91112)
T/S Cluster: S−4.9−1
PCo1
PCo2
−0.6
−0.4
−0.2
0.0
0.2
0.4

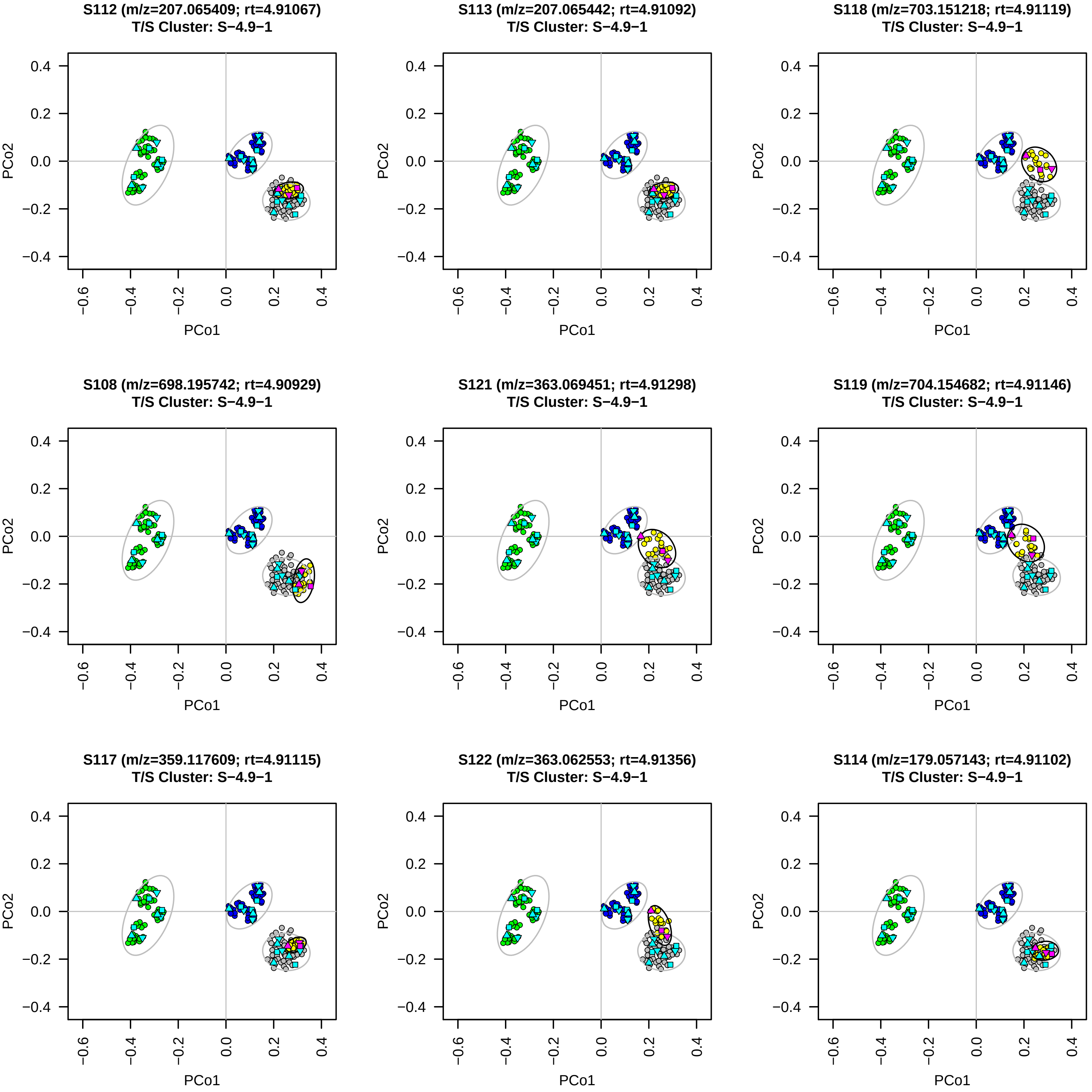
S112 (m/z=207.065409; rt=4.91067)
T/S Cluster: S−4.9−1
S113 (m/z=207.065442; rt=4.91092)
T/S Cluster: S−4.9−1
S118 (m/z=703.151218; rt=4.91119)
T/S Cluster: S−4.9−1
S108 (m/z=698.195742; rt=4.90929)
T/S Cluster: S−4.9−1
S121 (m/z=363.069451; rt=4.91298)
T/S Cluster: S−4.9−1
S119 (m/z=704.154682; rt=4.91146)
T/S Cluster: S−4.9−1
S117 (m/z=359.117609; rt=4.91115)
T/S Cluster: S−4.9−1
S122 (m/z=363.062553; rt=4.91356)
T/S Cluster: S−4.9−1
S114 (m/z=179.057143; rt=4.91102)
T/S Cluster: S−4.9−1
PCo1
PCo2
−0.6
−0.4
−0.2
0.0
0.2
0.4

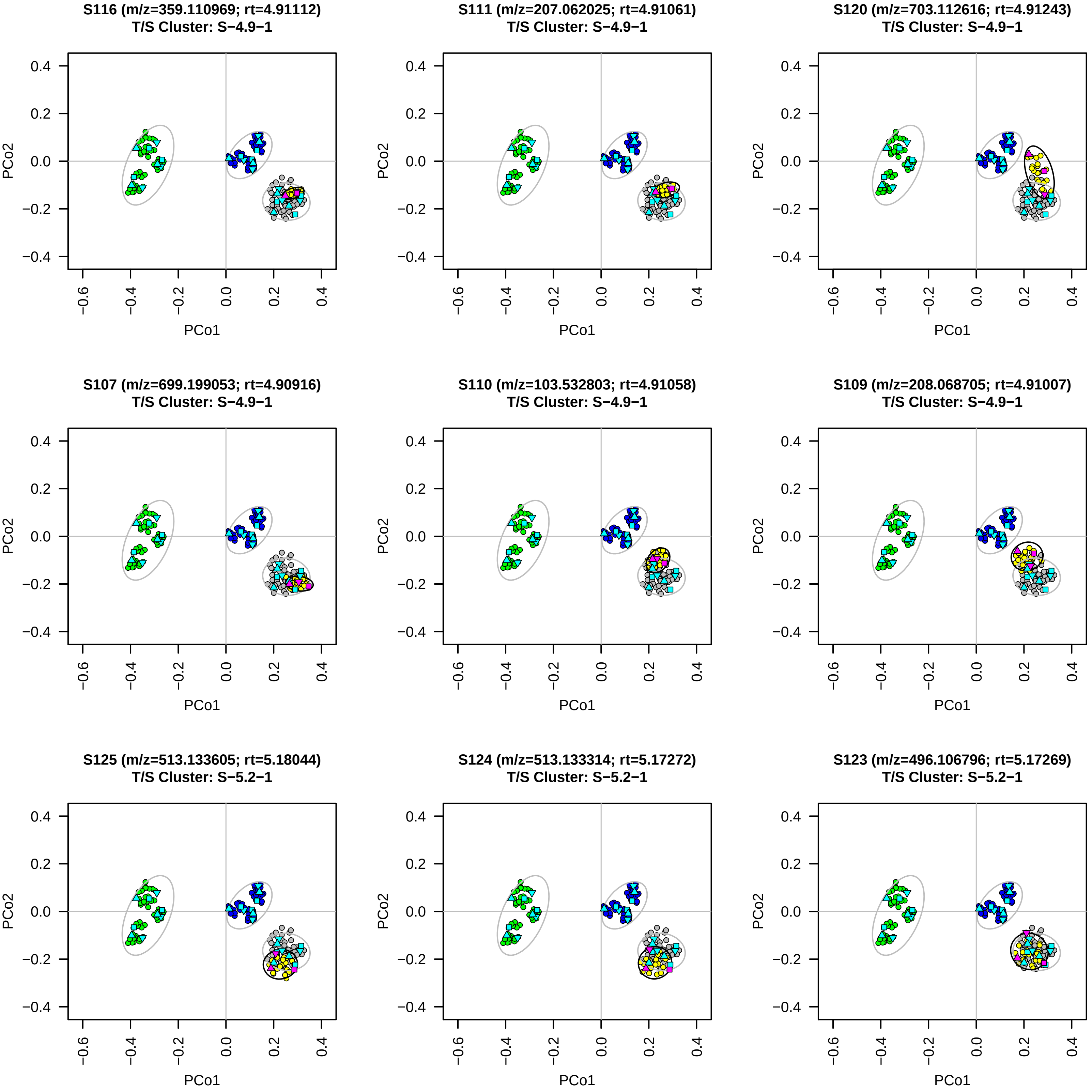
S116 (m/z=359.110969; rt=4.91112)
T/S Cluster: S−4.9−1
S111 (m/z=207.062025; rt=4.91061)
T/S Cluster: S−4.9−1
S120 (m/z=703.112616; rt=4.91243)
T/S Cluster: S−4.9−1
S107 (m/z=699.199053; rt=4.90916)
T/S Cluster: S−4.9−1
S110 (m/z=103.532803; rt=4.91058)
T/S Cluster: S−4.9−1
S109 (m/z=208.068705; rt=4.91007)
T/S Cluster: S−4.9−1
S125 (m/z=513.133605; rt=5.18044)
T/S Cluster: S−5.2−1
S124 (m/z=513.133314; rt=5.17272)
T/S Cluster: S−5.2−1
S123 (m/z=496.106796; rt=5.17269)
T/S Cluster: S−5.2−1
PCo1
PCo2
0.4
0.2
0.0
−0.2
−0.4
−0.6

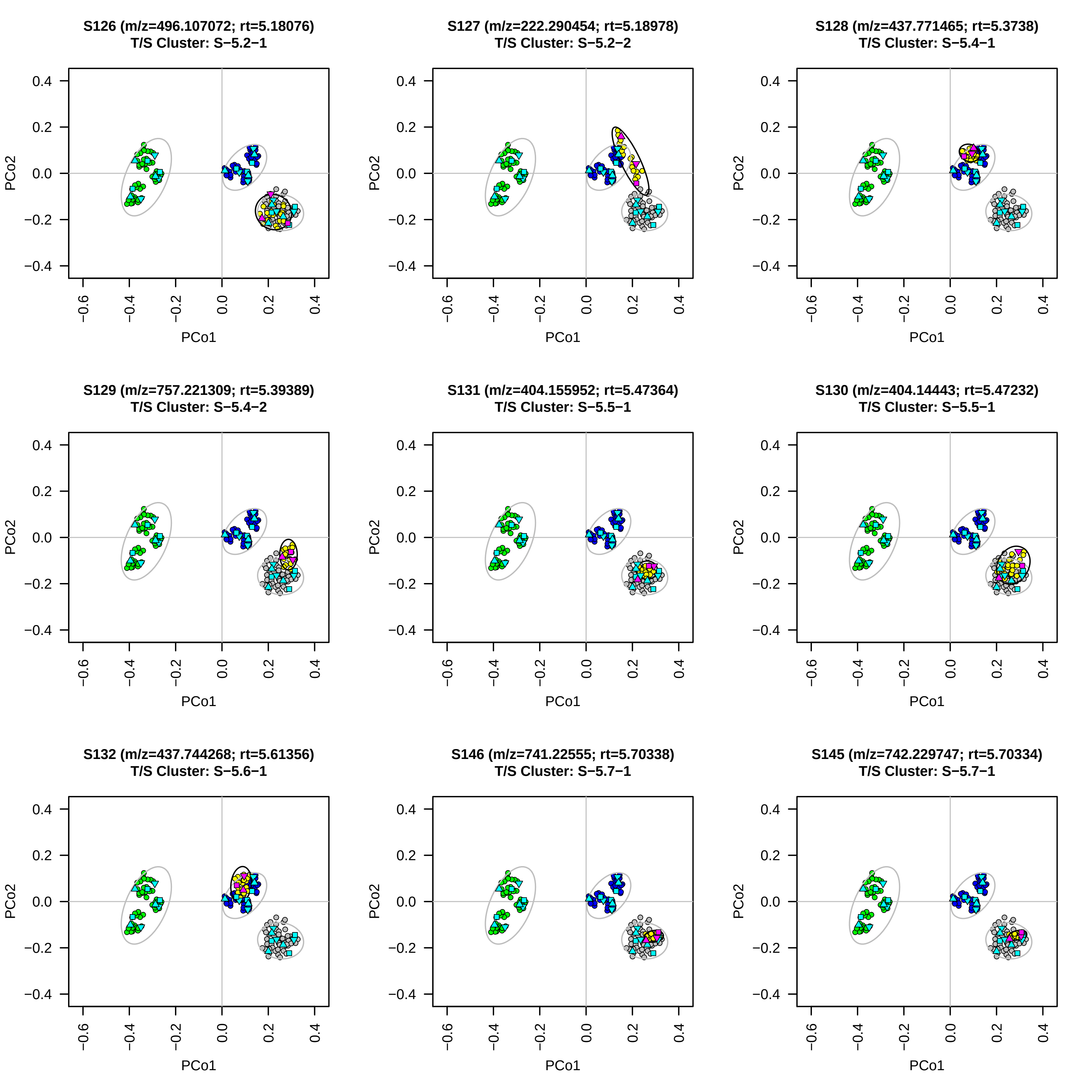
S126 (m/z=496.107072; rt=5.18076)
T/S Cluster: S−5.2−1
S127 (m/z=222.290454; rt=5.18978)
T/S Cluster: S−5.2−2
S128 (m/z=437.771465; rt=5.3738)
T/S Cluster: S−5.4−1
S129 (m/z=757.221309; rt=5.39389)
T/S Cluster: S−5.4−2
S131 (m/z=404.155952; rt=5.47364)
T/S Cluster: S−5.5−1
S130 (m/z=404.14443; rt=5.47232)
T/S Cluster: S−5.5−1
S132 (m/z=437.744268; rt=5.61356)
T/S Cluster: S−5.6−1
S146 (m/z=741.22555; rt=5.70338)
T/S Cluster: S−5.7−1
S145 (m/z=742.229747; rt=5.70334)
T/S Cluster: S−5.7−1
PCo1
PCo2
−0.6
−0.4
−0.2
0.0
0.2
0.4

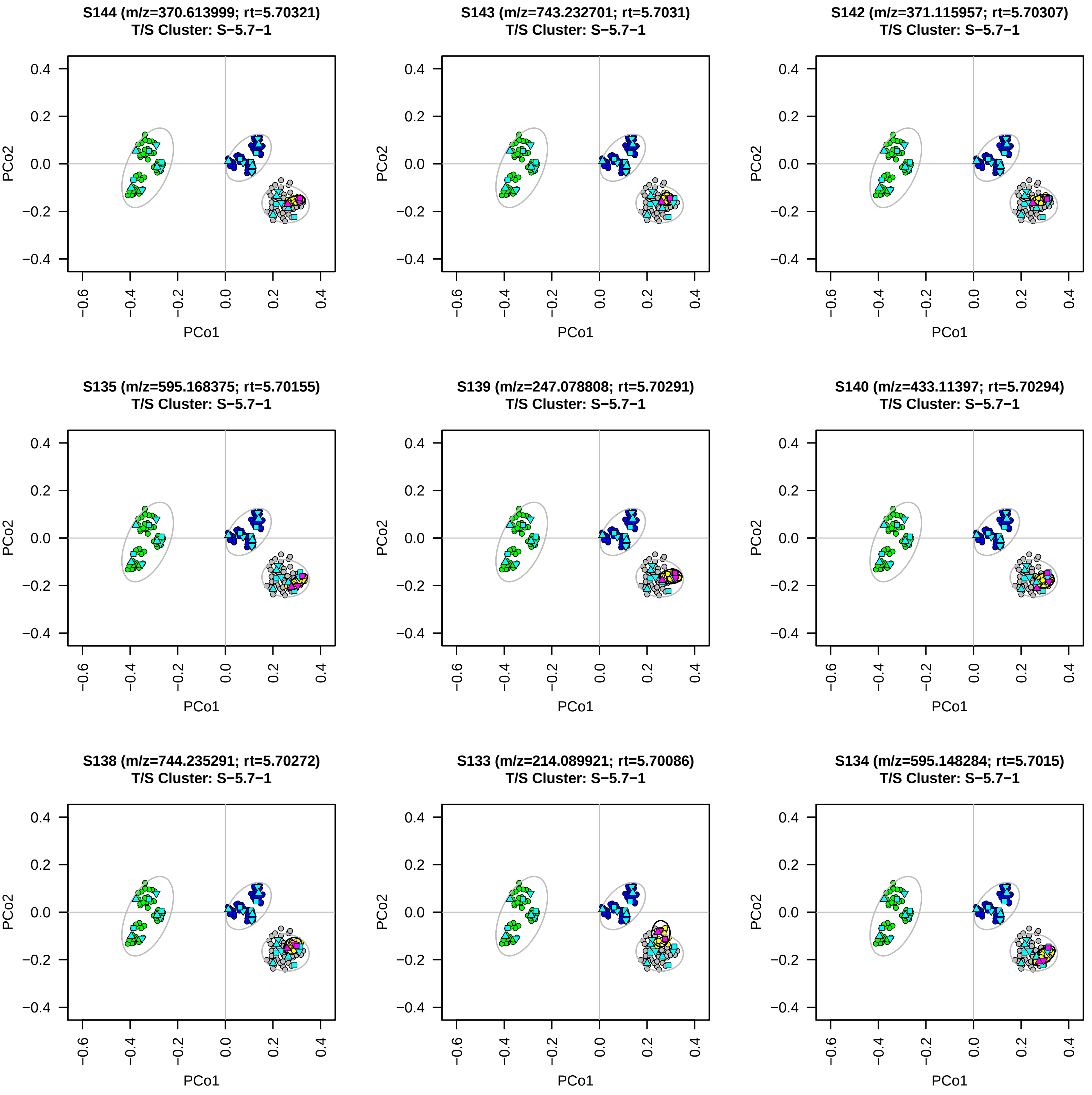
S144 (m/z=370.613999; rt=5.70321)
T/S Cluster: S−5.7−1
S143 (m/z=743.232701; rt=5.7031)
T/S Cluster: S−5.7−1
S142 (m/z=371.115957; rt=5.70307)
T/S Cluster: S−5.7−1
S135 (m/z=595.168375; rt=5.70155)
T/S Cluster: S−5.7−1
S139 (m/z=247.078808; rt=5.70291)
T/S Cluster: S−5.7−1
S140 (m/z=433.11397; rt=5.70294)
T/S Cluster: S−5.7−1
S138 (m/z=744.235291; rt=5.70272)
T/S Cluster: S−5.7−1
S133 (m/z=214.089921; rt=5.70086)
T/S Cluster: S−5.7−1
S134 (m/z=595.148284; rt=5.7015)
T/S Cluster: S−5.7−1
PCo1
PCo2
0.4
0.2
0.0
−0.2
−0.4
−0.6

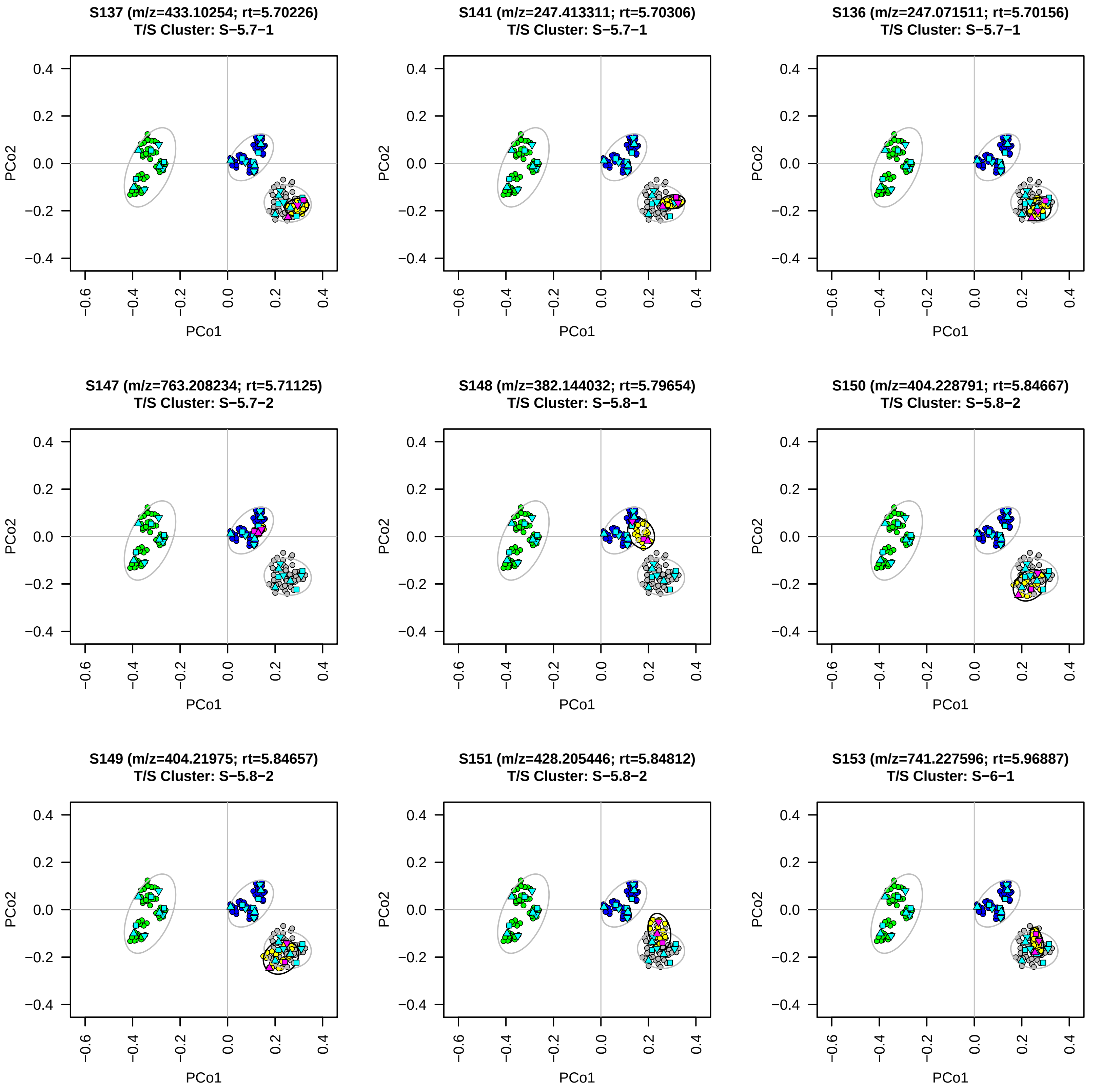
S137 (m/z=433.10254; rt=5.70226)
T/S Cluster: S−5.7−1
S141 (m/z=247.413311; rt=5.70306)
T/S Cluster: S−5.7−1
S136 (m/z=247.071511; rt=5.70156)
T/S Cluster: S−5.7−1
S147 (m/z=763.208234; rt=5.71125)
T/S Cluster: S−5.7−2
S148 (m/z=382.144032; rt=5.79654)
T/S Cluster: S−5.8−1
S150 (m/z=404.228791; rt=5.84667)
T/S Cluster: S−5.8−2
S149 (m/z=404.21975; rt=5.84657)
T/S Cluster: S−5.8−2
S151 (m/z=428.205446; rt=5.84812)
T/S Cluster: S−5.8−2
S153 (m/z=741.227596; rt=5.96887)
T/S Cluster: S−6−1
PCo1
PCo2
0.4
0.2
0.0
−0.2
−0.4
−0.6

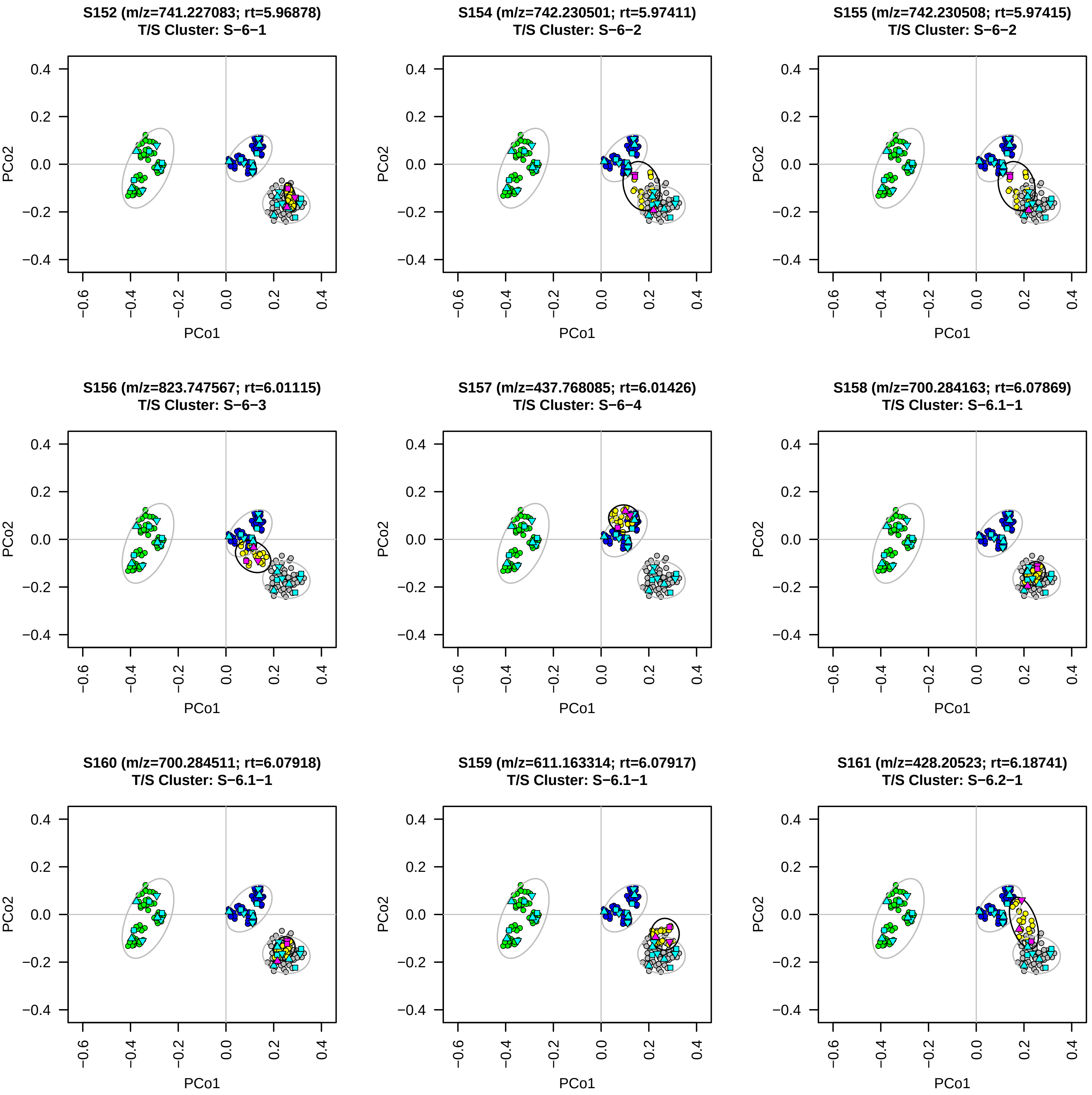
S152 (m/z=741.227083; rt=5.96878)
T/S Cluster: S−6−1
S154 (m/z=742.230501; rt=5.97411)
T/S Cluster: S−6−2
S155 (m/z=742.230508; rt=5.97415)
T/S Cluster: S−6−2
S156 (m/z=823.747567; rt=6.01115)
T/S Cluster: S−6−3
S157 (m/z=437.768085; rt=6.01426)
T/S Cluster: S−6−4
S158 (m/z=700.284163; rt=6.07869)
T/S Cluster: S−6.1−1
S160 (m/z=700.284511; rt=6.07918)
T/S Cluster: S−6.1−1
S159 (m/z=611.163314; rt=6.07917)
T/S Cluster: S−6.1−1
S161 (m/z=428.20523; rt=6.18741)
T/S Cluster: S−6.2−1
PCo1
PCo2
−0.6
−0.4
−0.2
0.0
0.2
0.4

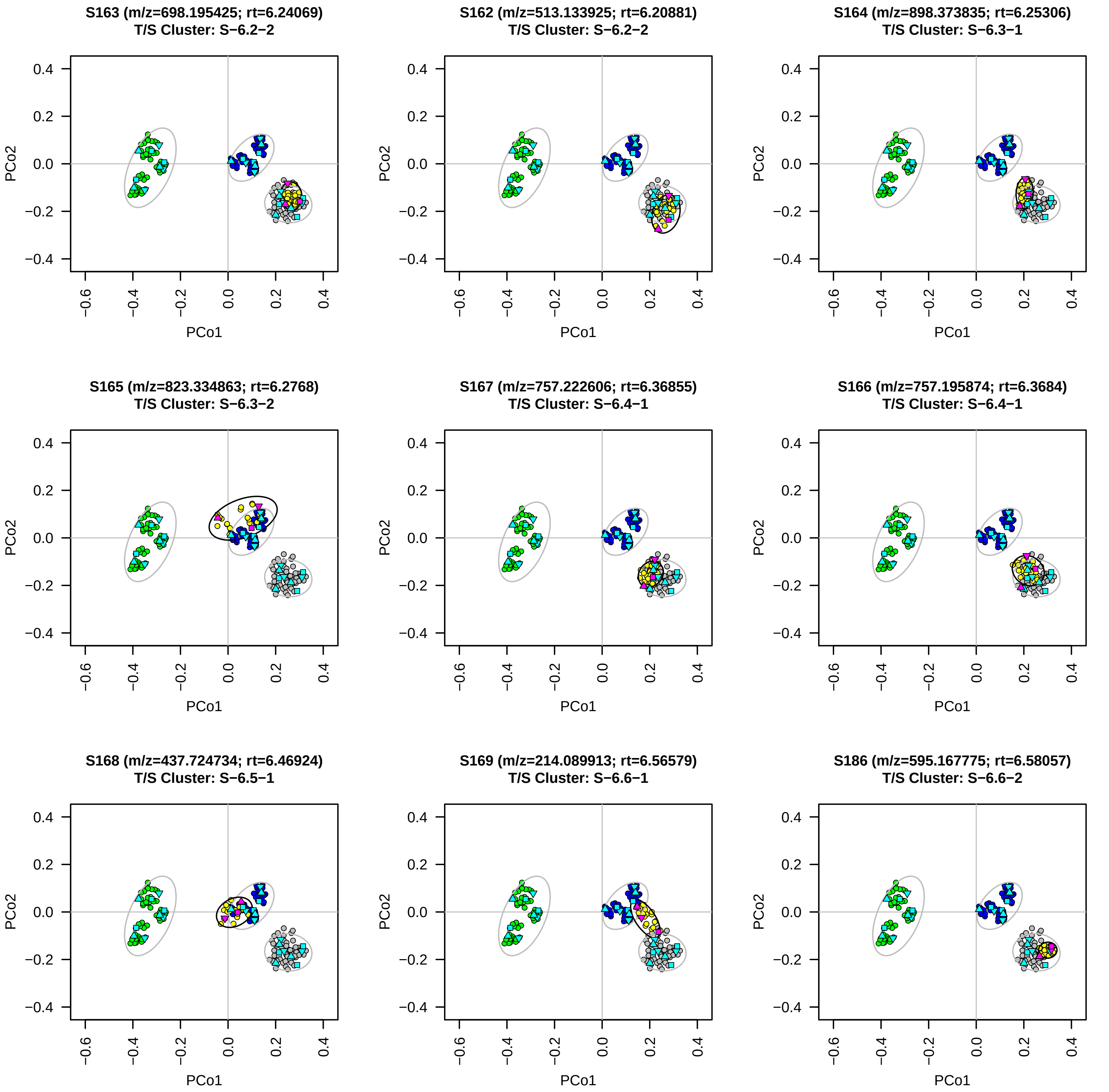
S163 (m/z=698.195425; rt=6.24069)
T/S Cluster: S−6.2−2
S162 (m/z=513.133925; rt=6.20881)
T/S Cluster: S−6.2−2
S164 (m/z=898.373835; rt=6.25306)
T/S Cluster: S−6.3−1
S165 (m/z=823.334863; rt=6.2768)
T/S Cluster: S−6.3−2
S167 (m/z=757.222606; rt=6.36855)
T/S Cluster: S−6.4−1
S166 (m/z=757.195874; rt=6.3684)
T/S Cluster: S−6.4−1
S168 (m/z=437.724734; rt=6.46924)
T/S Cluster: S−6.5−1
S169 (m/z=214.089913; rt=6.56579)
T/S Cluster: S−6.6−1
S186 (m/z=595.167775; rt=6.58057)
T/S Cluster: S−6.6−2
PCo1
PCo2
−0.6
−0.4
−0.2
0.0
0.2
0.4

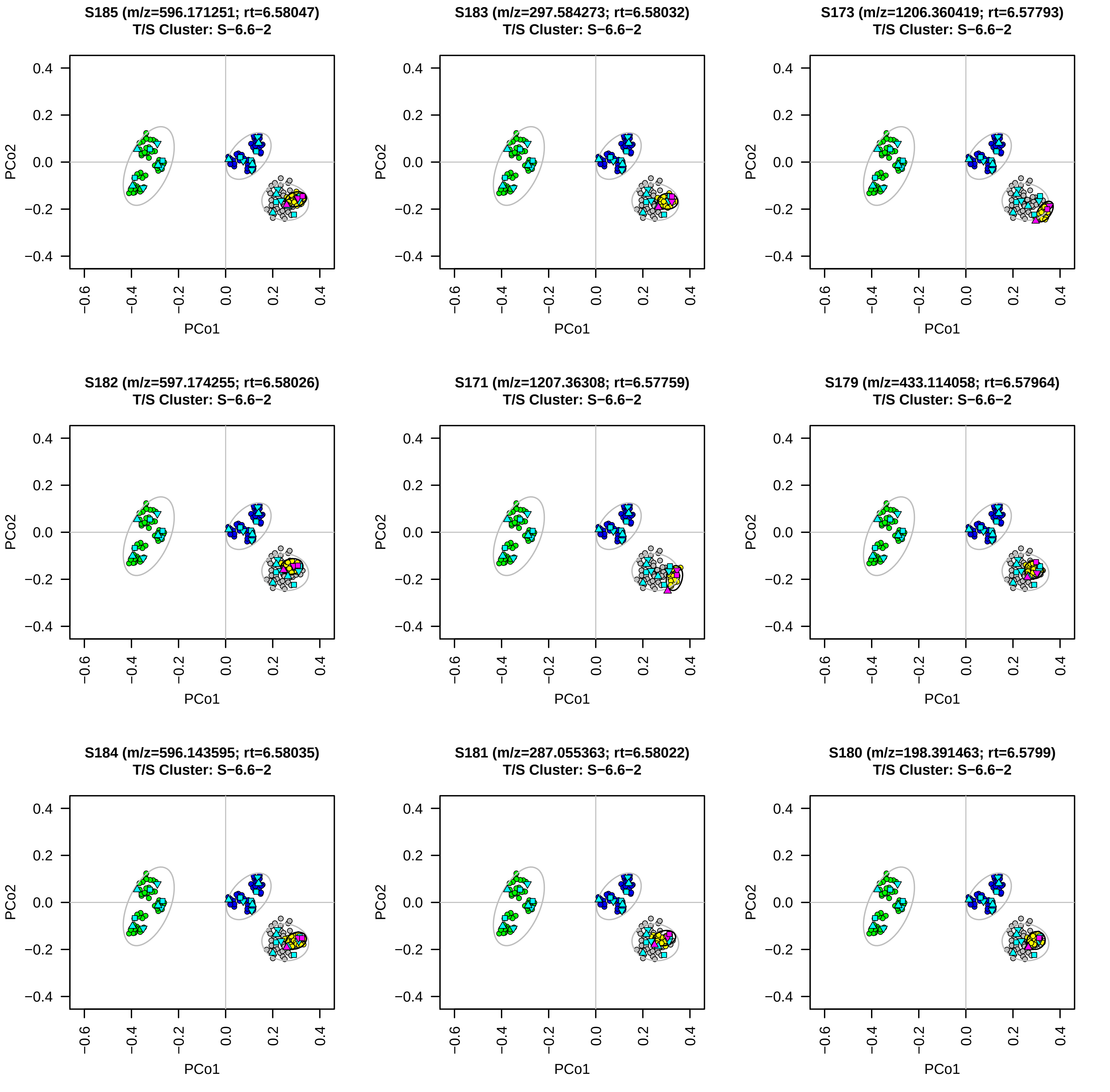

S185 (m/z=596.171251; rt=6.58047)
T/S Cluster: S−6.6−2
S183 (m/z=297.584273; rt=6.58032)
T/S Cluster: S−6.6−2
S173 (m/z=1206.360419; rt=6.57793)
T/S Cluster: S−6.6−2
S182 (m/z=597.174255; rt=6.58026)
T/S Cluster: S−6.6−2
S171 (m/z=1207.36308; rt=6.57759)
T/S Cluster: S−6.6−2
S179 (m/z=433.114058; rt=6.57964)
T/S Cluster: S−6.6−2
S184 (m/z=596.143595; rt=6.58035)
T/S Cluster: S−6.6−2
S181 (m/z=287.055363; rt=6.58022)
T/S Cluster: S−6.6−2
S180 (m/z=198.391463; rt=6.5799)
T/S Cluster: S−6.6−2
PCo1
PCo2
0.4
0.2
0.0
−0.2
−0.4
−0.6

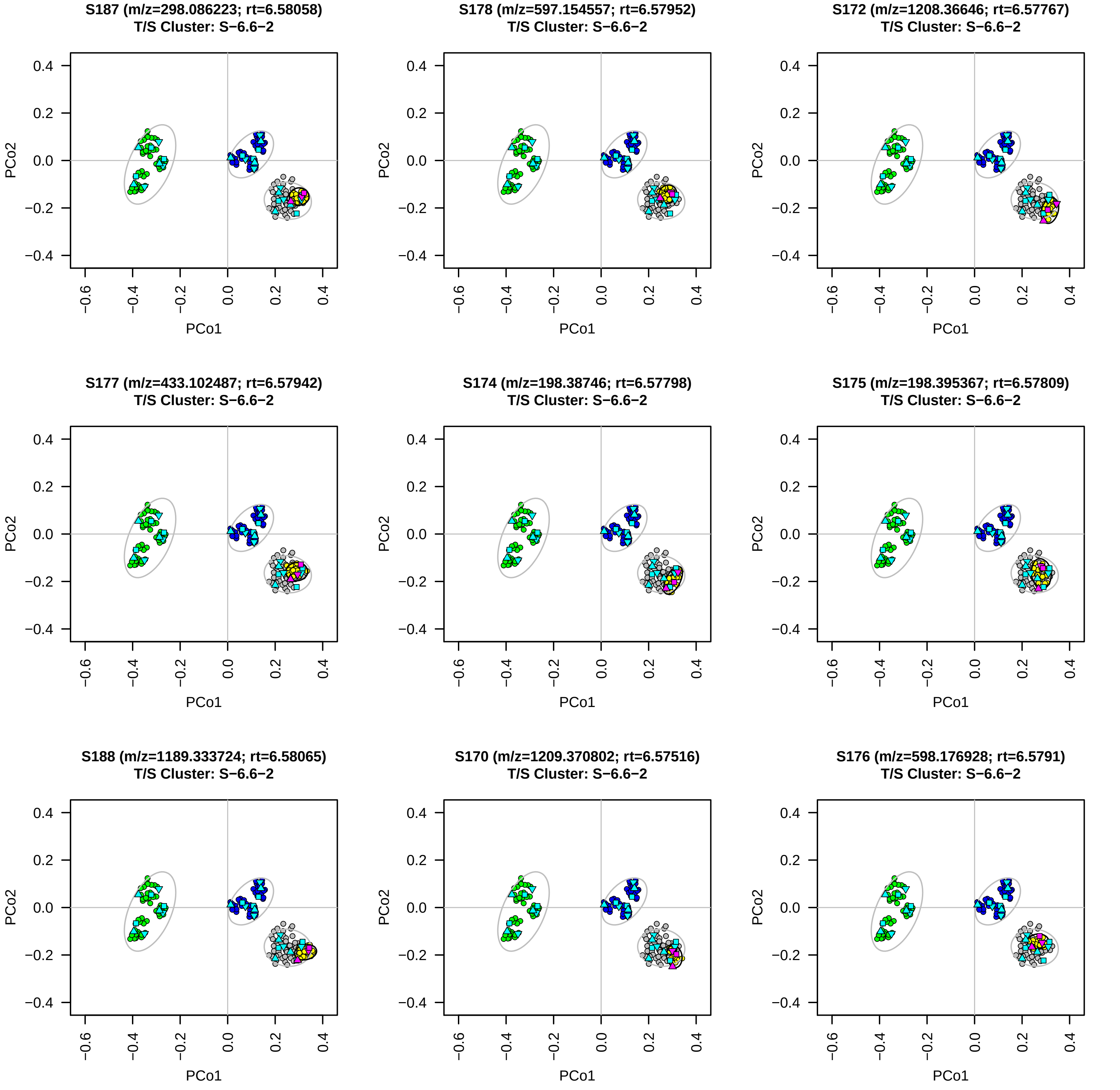
S187 (m/z=298.086223; rt=6.58058)
T/S Cluster: S−6.6−2
S178 (m/z=597.154557; rt=6.57952)
T/S Cluster: S−6.6−2
S172 (m/z=1208.36646; rt=6.57767)
T/S Cluster: S−6.6−2
S177 (m/z=433.102487; rt=6.57942)
T/S Cluster: S−6.6−2
S174 (m/z=198.38746; rt=6.57798)
T/S Cluster: S−6.6−2
S175 (m/z=198.395367; rt=6.57809)
T/S Cluster: S−6.6−2
S188 (m/z=1189.333724; rt=6.58065)
T/S Cluster: S−6.6−2
S170 (m/z=1209.370802; rt=6.57516)
T/S Cluster: S−6.6−2
S176 (m/z=598.176928; rt=6.5791)
T/S Cluster: S−6.6−2
PCo1
PCo2
0.4
0.2
0.0
−0.2
−0.4
−0.6

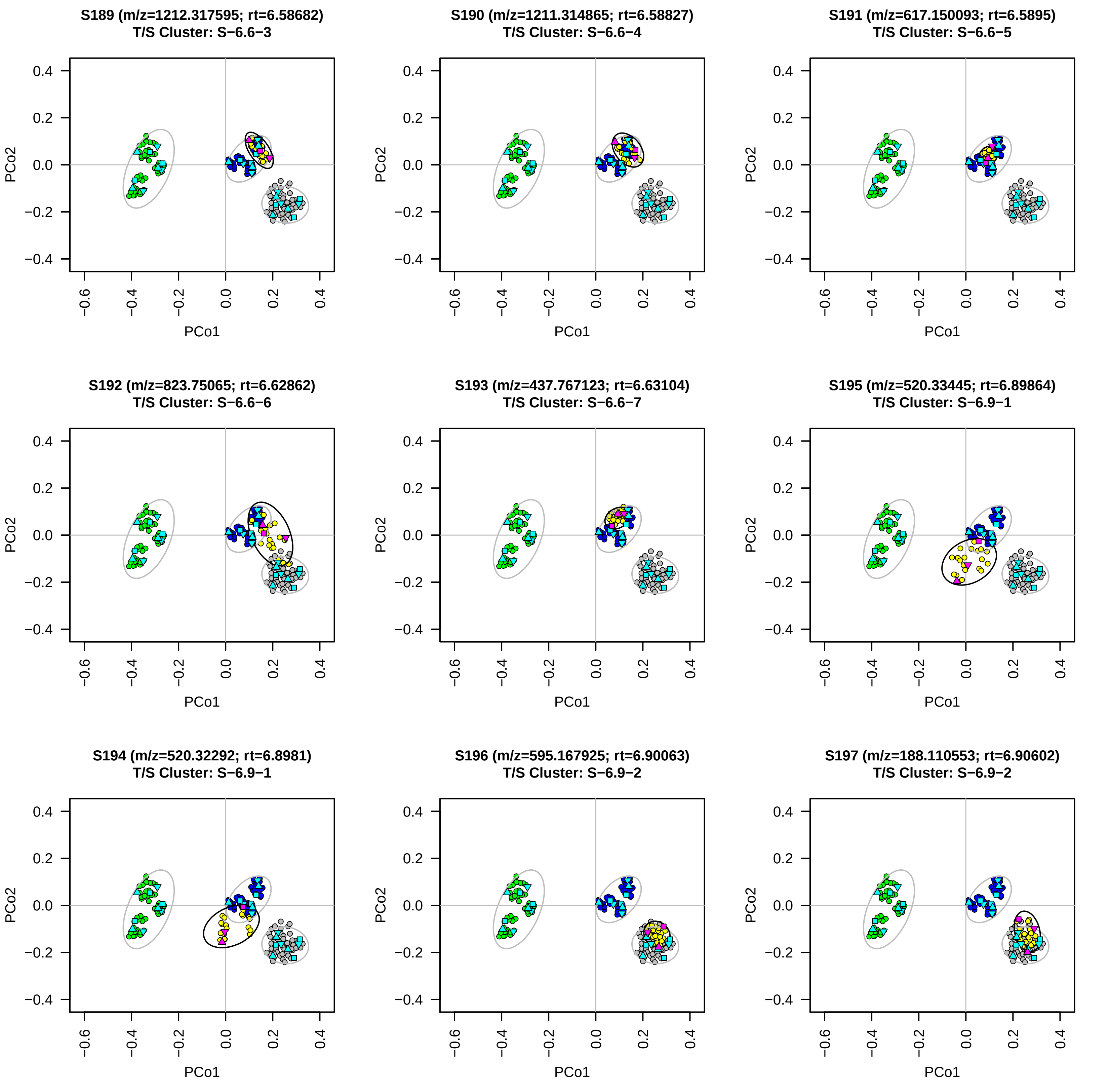
S189 (m/z=1212.317595; rt=6.58682)
T/S Cluster: S−6.6−3
S190 (m/z=1211.314865; rt=6.58827)
T/S Cluster: S−6.6−4
S191 (m/z=617.150093; rt=6.5895)
T/S Cluster: S−6.6−5
S192 (m/z=823.75065; rt=6.62862)
T/S Cluster: S−6.6−6
S193 (m/z=437.767123; rt=6.63104)
T/S Cluster: S−6.6−7
S195 (m/z=520.33445; rt=6.89864)
T/S Cluster: S−6.9−1
S194 (m/z=520.32292; rt=6.8981)
T/S Cluster: S−6.9−1
S196 (m/z=595.167925; rt=6.90063)
T/S Cluster: S−6.9−2
S197 (m/z=188.110553; rt=6.90602)
T/S Cluster: S−6.9−2
PCo1
PCo2
0.4
0.2
0.0
−0.2
−0.4
−0.6

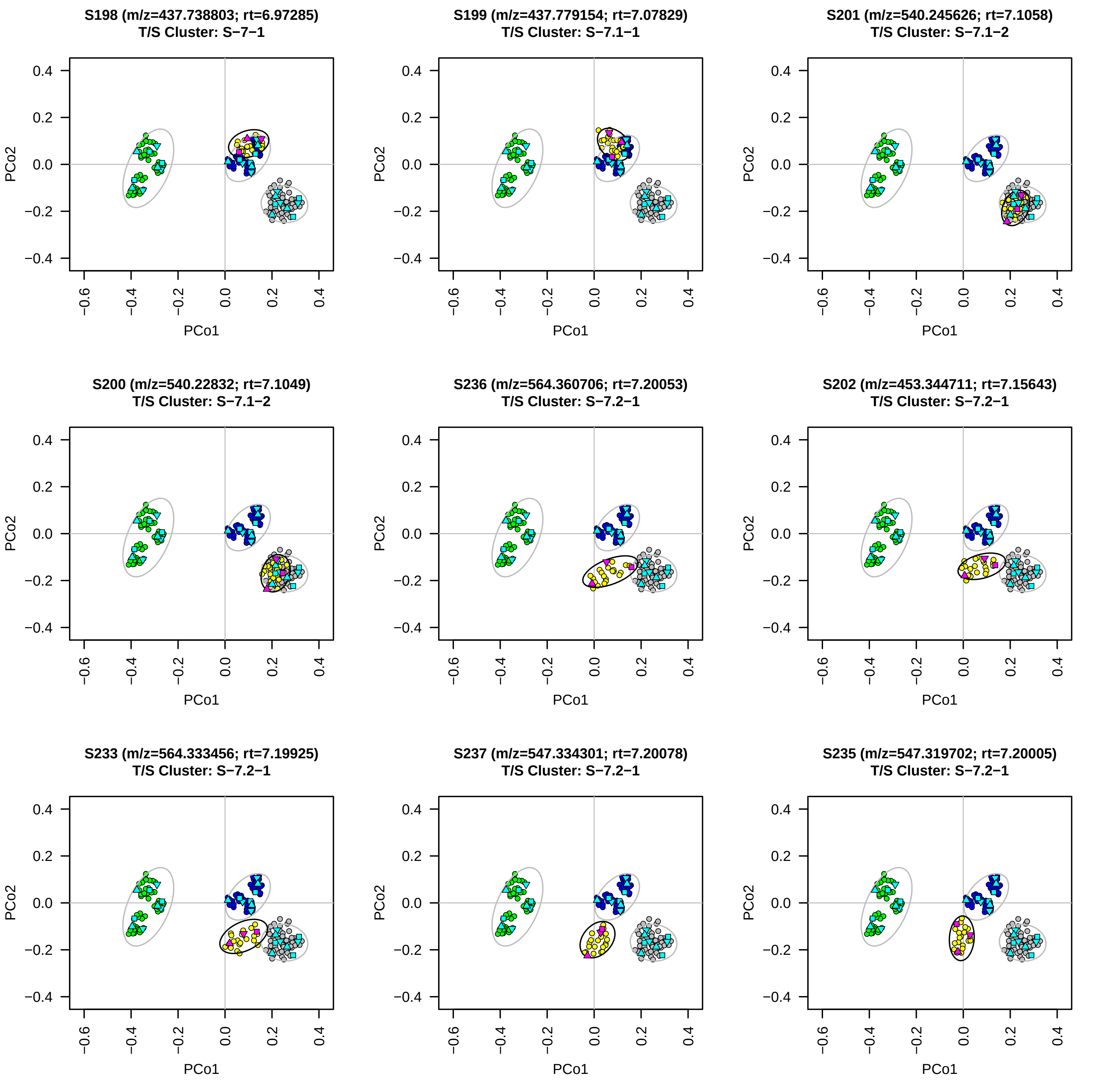
S198 (m/z=437.738803; rt=6.97285)
T/S Cluster: S−7−1
S199 (m/z=437.779154; rt=7.07829)
T/S Cluster: S−7.1−1
S201 (m/z=540.245626; rt=7.1058)
T/S Cluster: S−7.1−2
S200 (m/z=540.22832; rt=7.1049)
T/S Cluster: S−7.1−2
S236 (m/z=564.360706; rt=7.20053)
T/S Cluster: S−7.2−1
S202 (m/z=453.344711; rt=7.15643)
T/S Cluster: S−7.2−1
S233 (m/z=564.333456; rt=7.19925)
T/S Cluster: S−7.2−1
S237 (m/z=547.334301; rt=7.20078)
T/S Cluster: S−7.2−1
S235 (m/z=547.319702; rt=7.20005)
T/S Cluster: S−7.2−1
PCo1
PCo2
−0.6
−0.4
−0.2
0.0
0.2
0.4

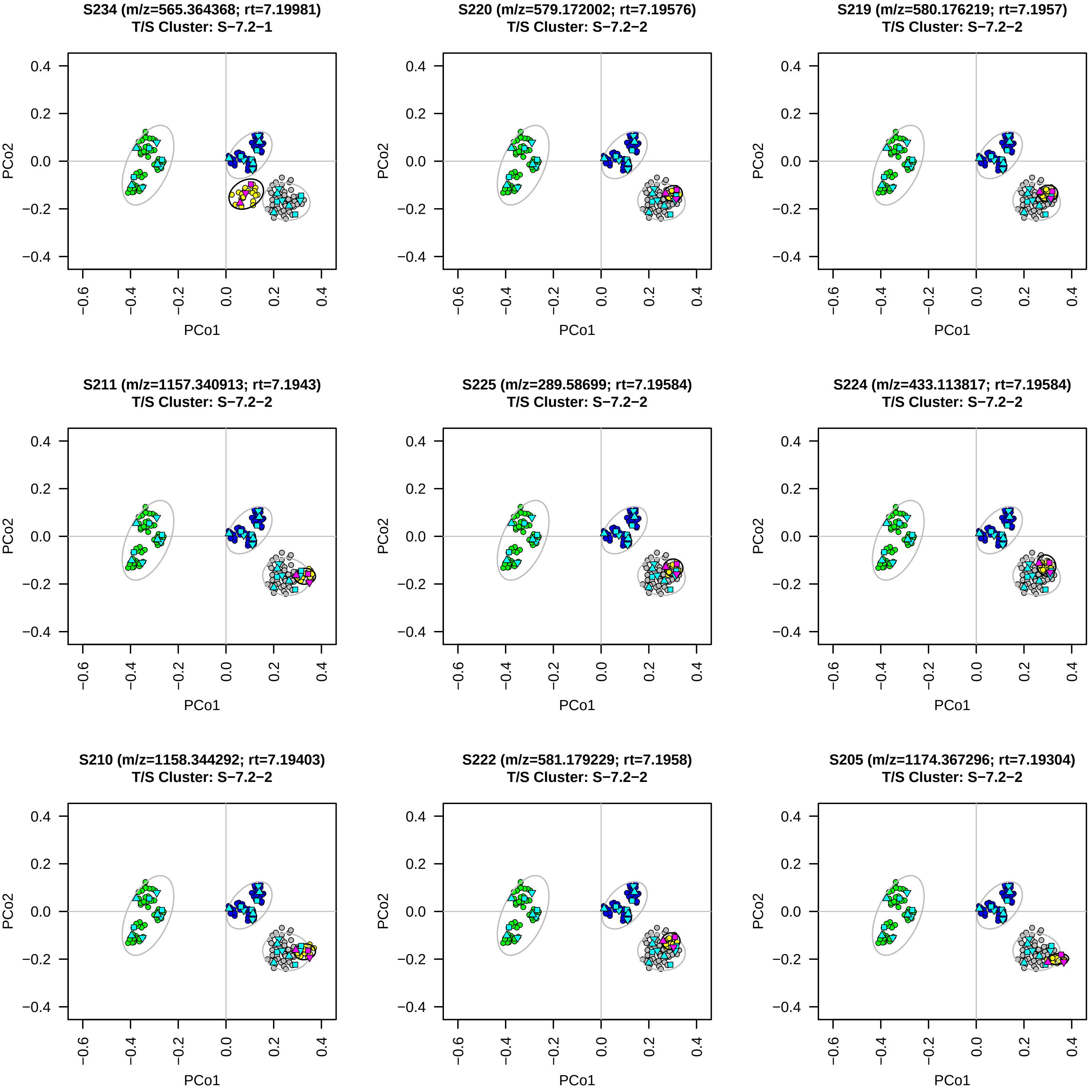
S234 (m/z=565.364368; rt=7.19981)
T/S Cluster: S−7.2−1
S220 (m/z=579.172002; rt=7.19576)
T/S Cluster: S−7.2−2
S219 (m/z=580.176219; rt=7.1957)
T/S Cluster: S−7.2−2
S211 (m/z=1157.340913; rt=7.1943)
T/S Cluster: S−7.2−2
S225 (m/z=289.58699; rt=7.19584)
T/S Cluster: S−7.2−2
S224 (m/z=433.113817; rt=7.19584)
T/S Cluster: S−7.2−2
S210 (m/z=1158.344292; rt=7.19403)
T/S Cluster: S−7.2−2
S222 (m/z=581.179229; rt=7.1958)
T/S Cluster: S−7.2−2
S205 (m/z=1174.367296; rt=7.19304)
T/S Cluster: S−7.2−2
PCo1
PCo2

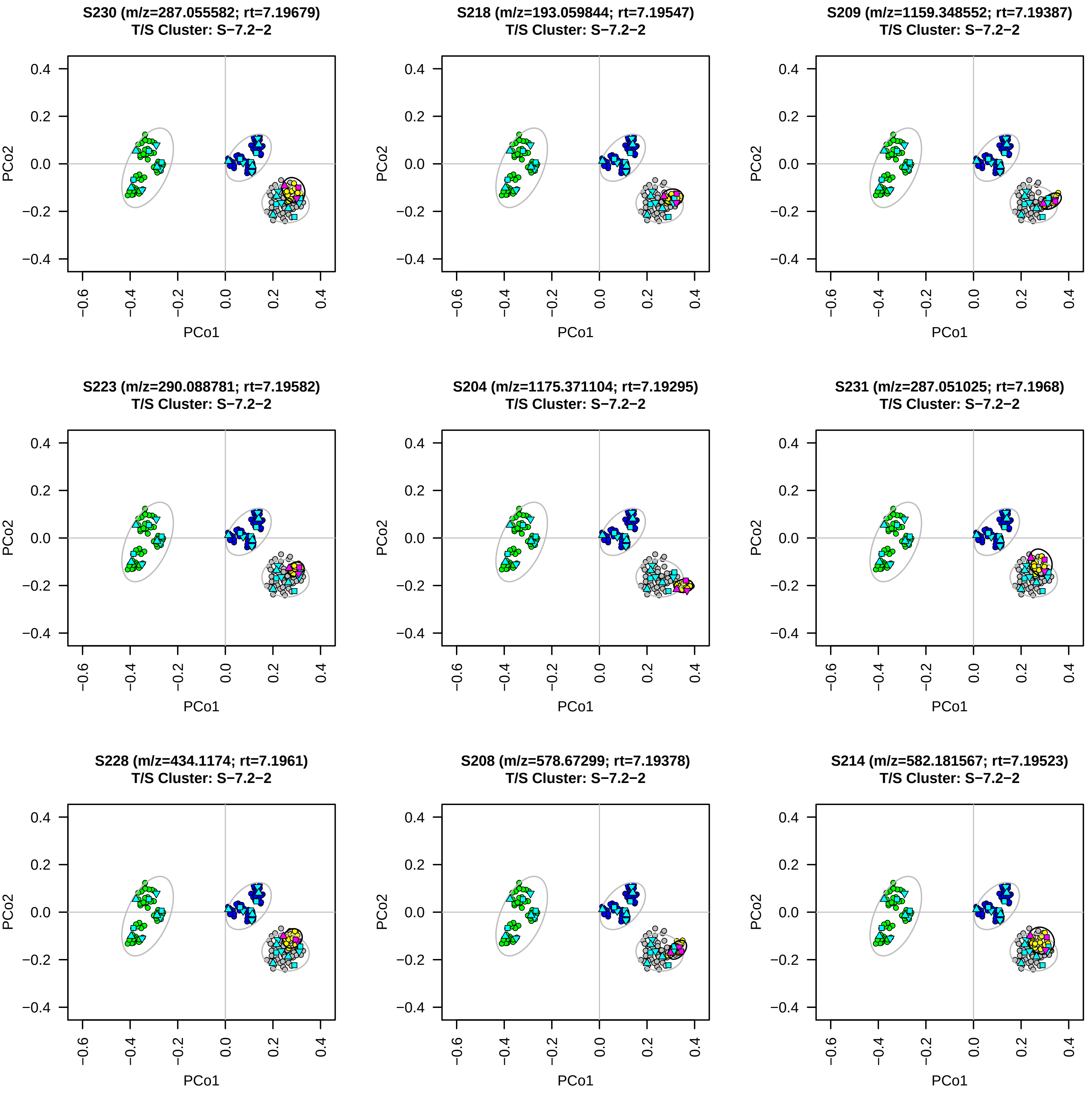
S230 (m/z=287.055582; rt=7.19679)
T/S Cluster: S−7.2−2
S218 (m/z=193.059844; rt=7.19547)
T/S Cluster: S−7.2−2
S209 (m/z=1159.348552; rt=7.19387)
T/S Cluster: S−7.2−2
S223 (m/z=290.088781; rt=7.19582)
T/S Cluster: S−7.2−2
S204 (m/z=1175.371104; rt=7.19295)
T/S Cluster: S−7.2−2
S231 (m/z=287.051025; rt=7.1968)
T/S Cluster: S−7.2−2
S228 (m/z=434.1174; rt=7.1961)
T/S Cluster: S−7.2−2
S208 (m/z=578.67299; rt=7.19378)
T/S Cluster: S−7.2−2
S214 (m/z=582.181567; rt=7.19523)
T/S Cluster: S−7.2−2
PCo1
PCo2
−0.6
−0.4
−0.2
0.0
0.2
0.4

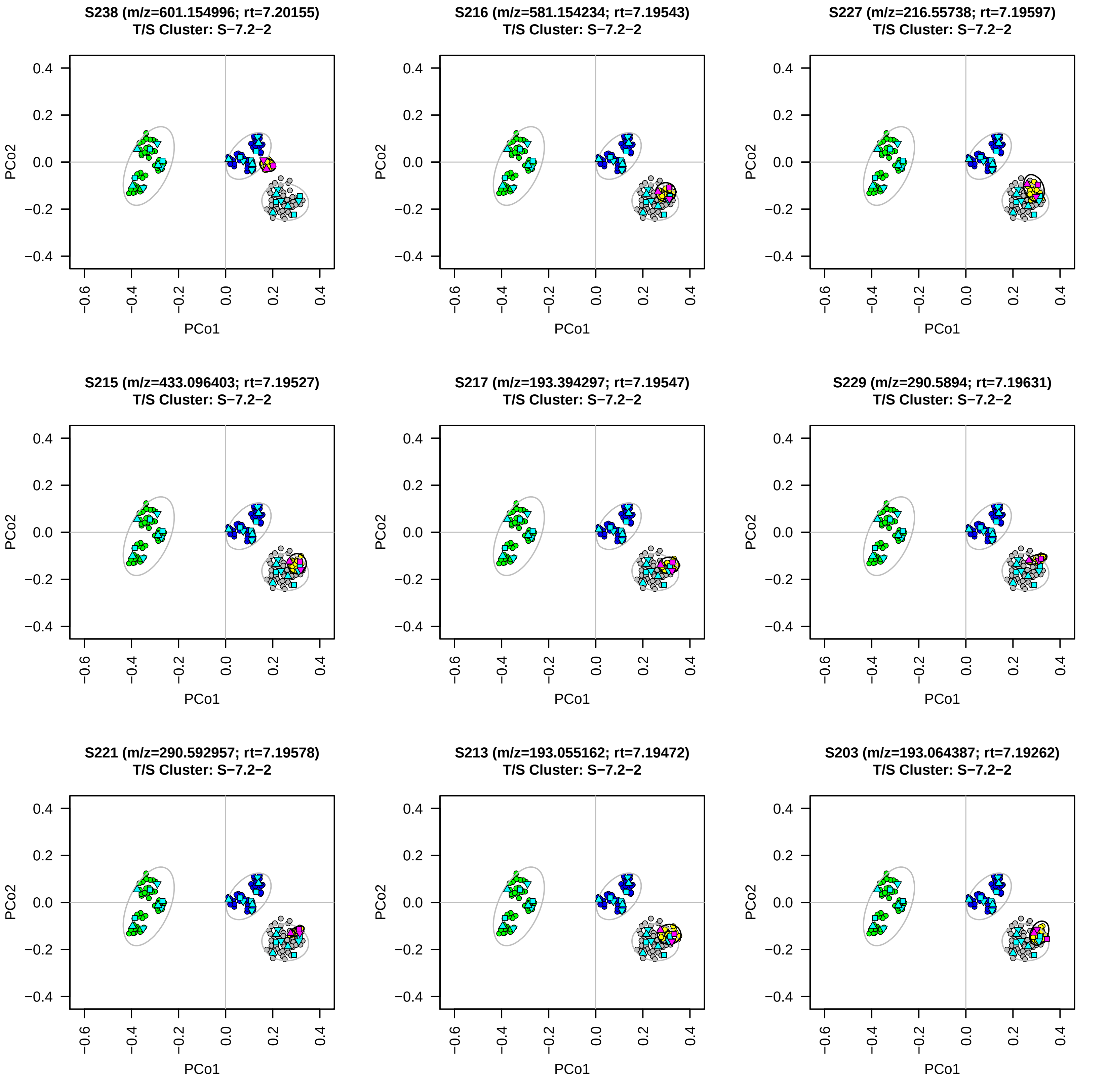
S238 (m/z=601.154996; rt=7.20155)
T/S Cluster: S−7.2−2
S216 (m/z=581.154234; rt=7.19543)
T/S Cluster: S−7.2−2
S227 (m/z=216.55738; rt=7.19597)
T/S Cluster: S−7.2−2
S215 (m/z=433.096403; rt=7.19527)
T/S Cluster: S−7.2−2
S217 (m/z=193.394297; rt=7.19547)
T/S Cluster: S−7.2−2
S229 (m/z=290.5894; rt=7.19631)
T/S Cluster: S−7.2−2
S221 (m/z=290.592957; rt=7.19578)
T/S Cluster: S−7.2−2
S213 (m/z=193.055162; rt=7.19472)
T/S Cluster: S−7.2−2
S203 (m/z=193.064387; rt=7.19262)
T/S Cluster: S−7.2−2
PCo1
PCo2
0.4
0.2
0.0
−0.2
−0.4
−0.6

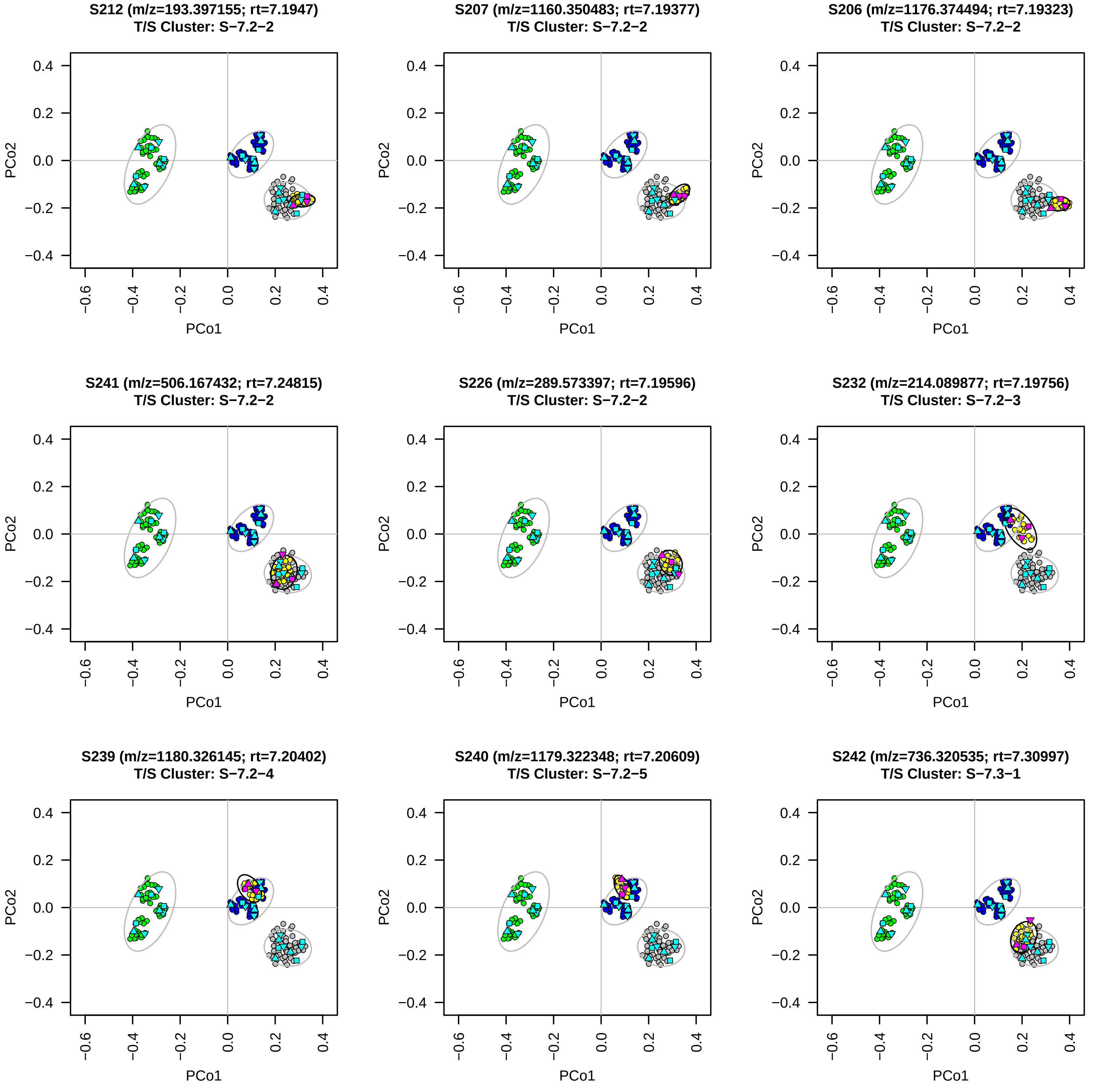
S212 (m/z=193.397155; rt=7.1947)
T/S Cluster: S−7.2−2
S207 (m/z=1160.350483; rt=7.19377)
T/S Cluster: S−7.2−2
S206 (m/z=1176.374494; rt=7.19323)
T/S Cluster: S−7.2−2
S241 (m/z=506.167432; rt=7.24815)
T/S Cluster: S−7.2−2
S226 (m/z=289.573397; rt=7.19596)
T/S Cluster: S−7.2−2
S232 (m/z=214.089877; rt=7.19756)
T/S Cluster: S−7.2−3
S239 (m/z=1180.326145; rt=7.20402)
T/S Cluster: S−7.2−4
S240 (m/z=1179.322348; rt=7.20609)
T/S Cluster: S−7.2−5
S242 (m/z=736.320535; rt=7.30997)
T/S Cluster: S−7.3−1
PCo1
PCo2
0.4
0.2
0.0
−0.2
−0.4
−0.6

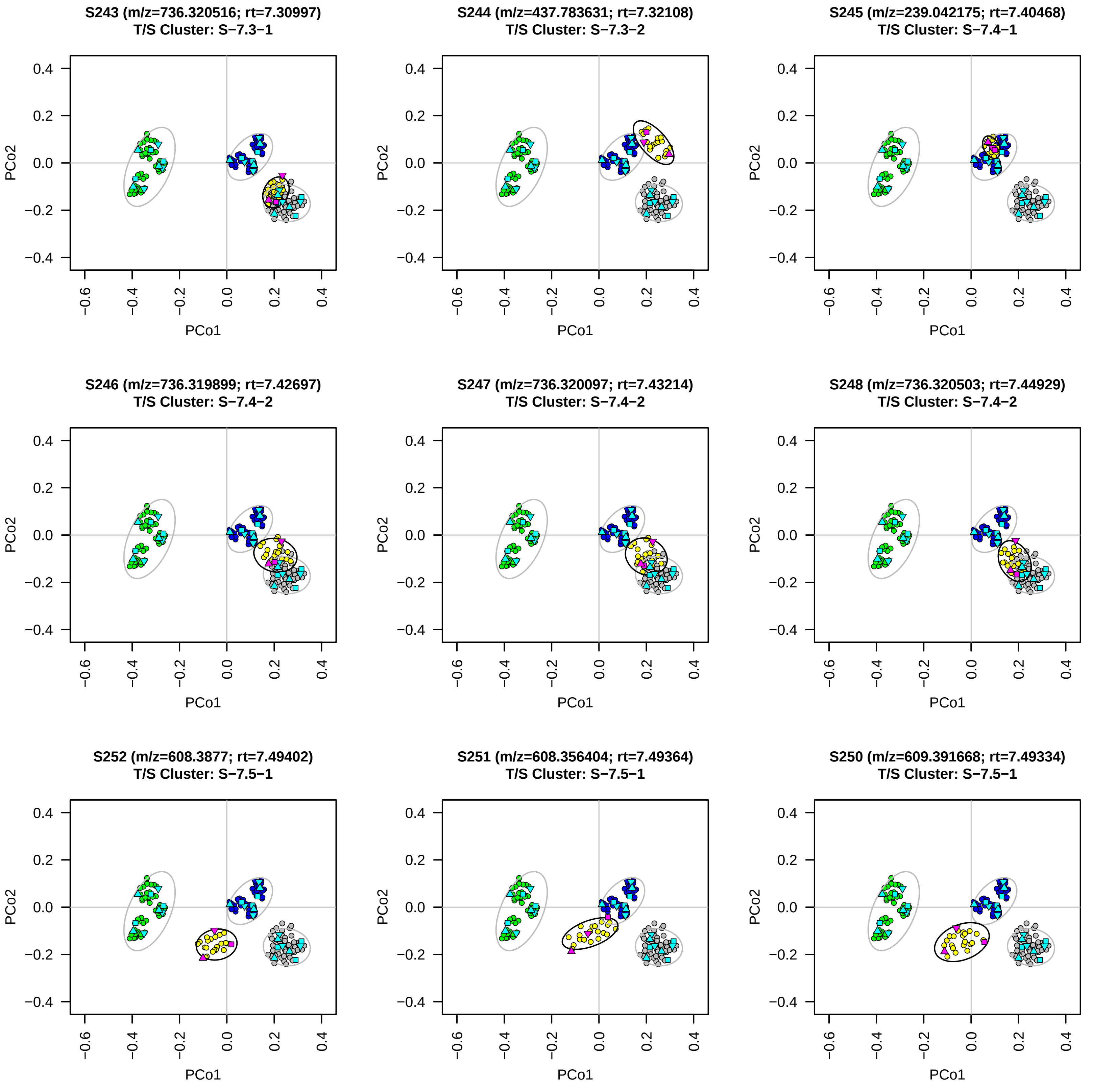
S243 (m/z=736.320516; rt=7.30997)
T/S Cluster: S−7.3−1
S244 (m/z=437.783631; rt=7.32108)
T/S Cluster: S−7.3−2
S245 (m/z=239.042175; rt=7.40468)
T/S Cluster: S−7.4−1
S246 (m/z=736.319899; rt=7.42697)
T/S Cluster: S−7.4−2
S247 (m/z=736.320097; rt=7.43214)
T/S Cluster: S−7.4−2
S248 (m/z=736.320503; rt=7.44929)
T/S Cluster: S−7.4−2
S252 (m/z=608.3877; rt=7.49402)
T/S Cluster: S−7.5−1
S251 (m/z=608.356404; rt=7.49364)
T/S Cluster: S−7.5−1
S250 (m/z=609.391668; rt=7.49334)
T/S Cluster: S−7.5−1
PCo1
PCo2

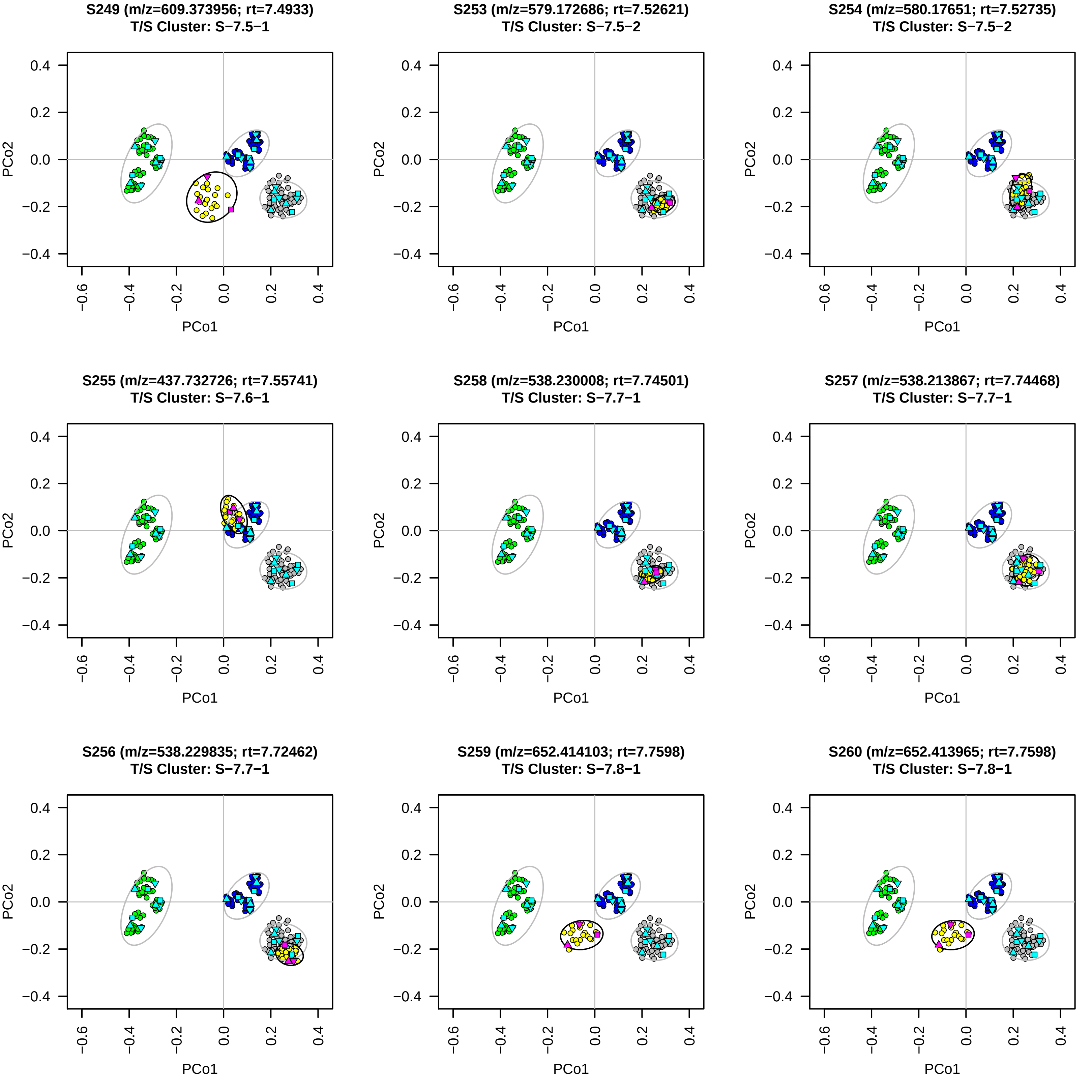
S249 (m/z=609.373956; rt=7.4933)
T/S Cluster: S−7.5−1
S253 (m/z=579.172686; rt=7.52621)
T/S Cluster: S−7.5−2
S254 (m/z=580.17651; rt=7.52735)
T/S Cluster: S−7.5−2
S255 (m/z=437.732726; rt=7.55741)
T/S Cluster: S−7.6−1
S258 (m/z=538.230008; rt=7.74501)
T/S Cluster: S−7.7−1
S257 (m/z=538.213867; rt=7.74468)
T/S Cluster: S−7.7−1
S256 (m/z=538.229835; rt=7.72462)
T/S Cluster: S−7.7−1
S259 (m/z=652.414103; rt=7.7598)
T/S Cluster: S−7.8−1
S260 (m/z=652.413965; rt=7.7598)
T/S Cluster: S−7.8−1
PCo1
PCo2
0.4
0.2
0.0
−0.2
−0.4
−0.6

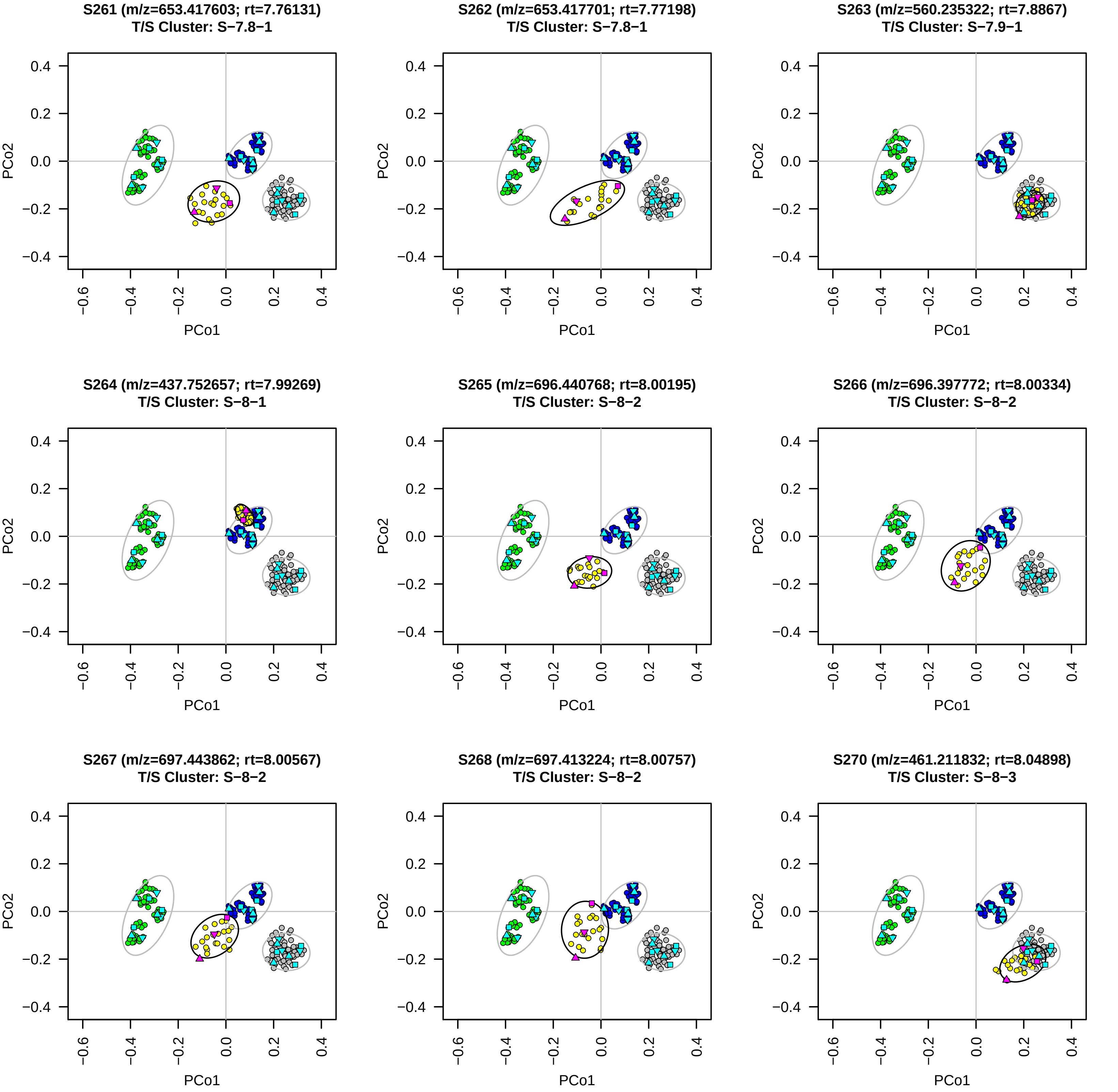

S261 (m/z=653.417603; rt=7.76131)
T/S Cluster: S−7.8−1
S262 (m/z=653.417701; rt=7.77198)
T/S Cluster: S−7.8−1
S263 (m/z=560.235322; rt=7.8867)
T/S Cluster: S−7.9−1
S264 (m/z=437.752657; rt=7.99269)
T/S Cluster: S−8−1
S265 (m/z=696.440768; rt=8.00195)
T/S Cluster: S−8−2
S266 (m/z=696.397772; rt=8.00334)
T/S Cluster: S−8−2
S267 (m/z=697.443862; rt=8.00567)
T/S Cluster: S−8−2
S268 (m/z=697.413224; rt=8.00757)
T/S Cluster: S−8−2
S270 (m/z=461.211832; rt=8.04898)
T/S Cluster: S−8−3
PCo1
PCo2
0.4
0.2
0.0
−0.2
−0.4
−0.6

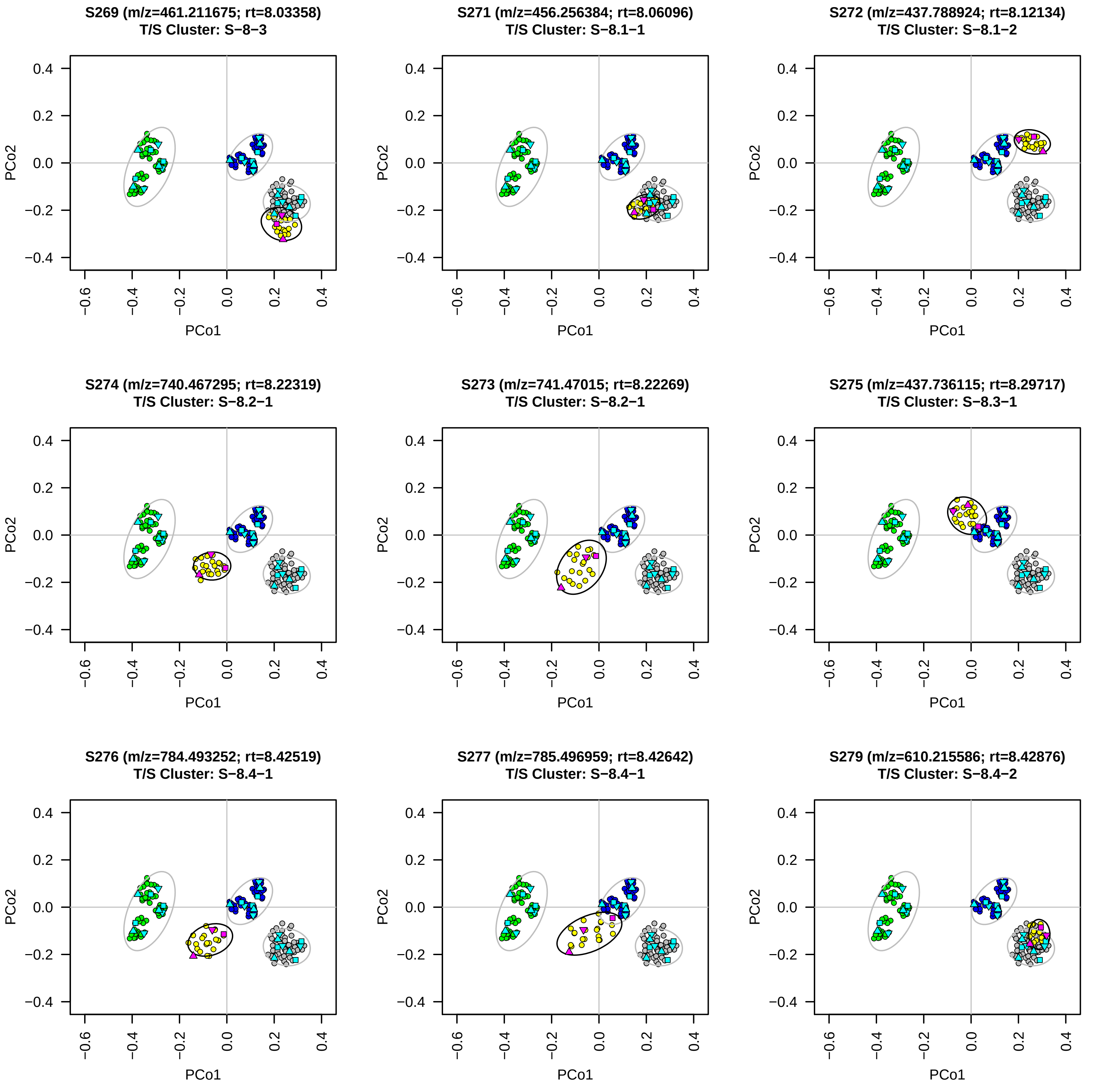

S269 (m/z=461.211675; rt=8.03358)
T/S Cluster: S−8−3
S271 (m/z=456.256384; rt=8.06096)
T/S Cluster: S−8.1−1
S272 (m/z=437.788924; rt=8.12134)
T/S Cluster: S−8.1−2
S274 (m/z=740.467295; rt=8.22319)
T/S Cluster: S−8.2−1
S273 (m/z=741.47015; rt=8.22269)
T/S Cluster: S−8.2−1
S275 (m/z=437.736115; rt=8.29717)
T/S Cluster: S−8.3−1
S276 (m/z=784.493252; rt=8.42519)
T/S Cluster: S−8.4−1
S277 (m/z=785.496959; rt=8.42642)
T/S Cluster: S−8.4−1
S279 (m/z=610.215586; rt=8.42876)
T/S Cluster: S−8.4−2
PCo1
PCo2

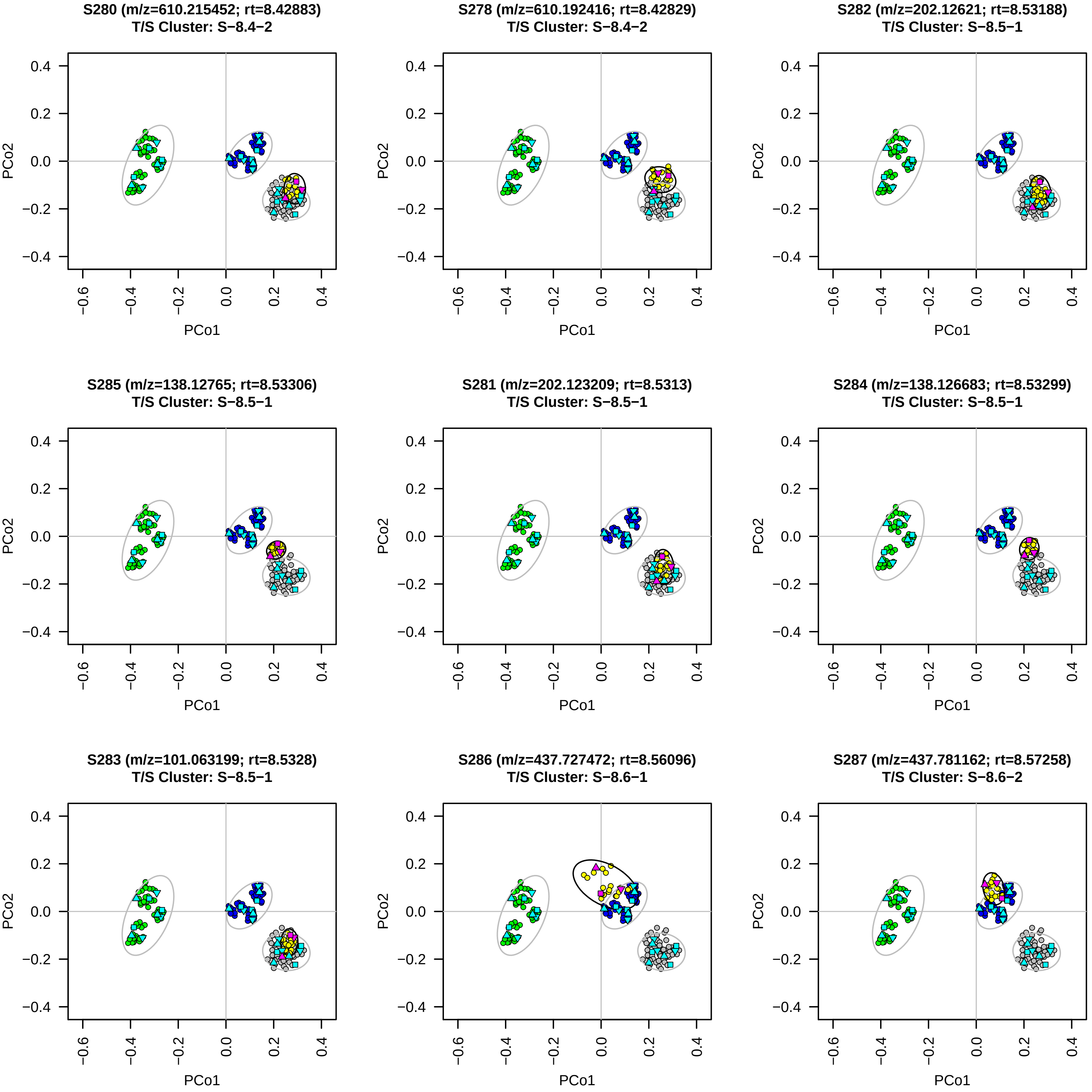
S280 (m/z=610.215452; rt=8.42883)
T/S Cluster: S−8.4−2
S278 (m/z=610.192416; rt=8.42829)
T/S Cluster: S−8.4−2
S282 (m/z=202.12621; rt=8.53188)
T/S Cluster: S−8.5−1
S285 (m/z=138.12765; rt=8.53306)
T/S Cluster: S−8.5−1
S281 (m/z=202.123209; rt=8.5313)
T/S Cluster: S−8.5−1
S284 (m/z=138.126683; rt=8.53299)
T/S Cluster: S−8.5−1
S283 (m/z=101.063199; rt=8.5328)
T/S Cluster: S−8.5−1
S286 (m/z=437.727472; rt=8.56096)
T/S Cluster: S−8.6−1
S287 (m/z=437.781162; rt=8.57258)
T/S Cluster: S−8.6−2
PCo1
PCo2
0.4
0.2
0.0
−0.2
−0.4
−0.6

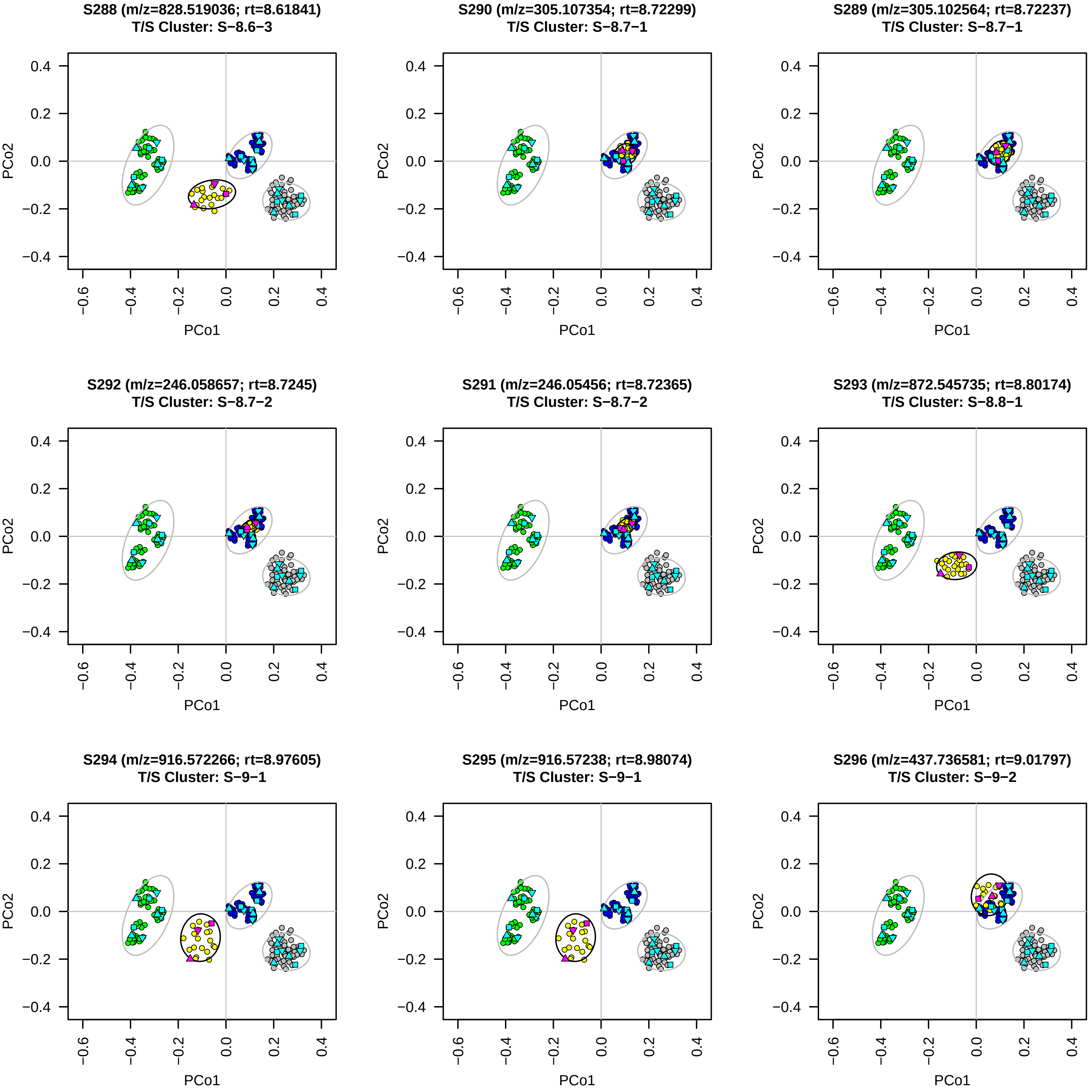
S288 (m/z=828.519036; rt=8.61841)
T/S Cluster: S−8.6−3
S290 (m/z=305.107354; rt=8.72299)
T/S Cluster: S−8.7−1
S289 (m/z=305.102564; rt=8.72237)
T/S Cluster: S−8.7−1
S292 (m/z=246.058657; rt=8.7245)
T/S Cluster: S−8.7−2
S291 (m/z=246.05456; rt=8.72365)
T/S Cluster: S−8.7−2
S293 (m/z=872.545735; rt=8.80174)
T/S Cluster: S−8.8−1
S294 (m/z=916.572266; rt=8.97605)
T/S Cluster: S−9−1
S295 (m/z=916.57238; rt=8.98074)
T/S Cluster: S−9−1
S296 (m/z=437.736581; rt=9.01797)
T/S Cluster: S−9−2
PCo1
PCo2
−0.6
−0.4
−0.2
0.0
0.2
0.4

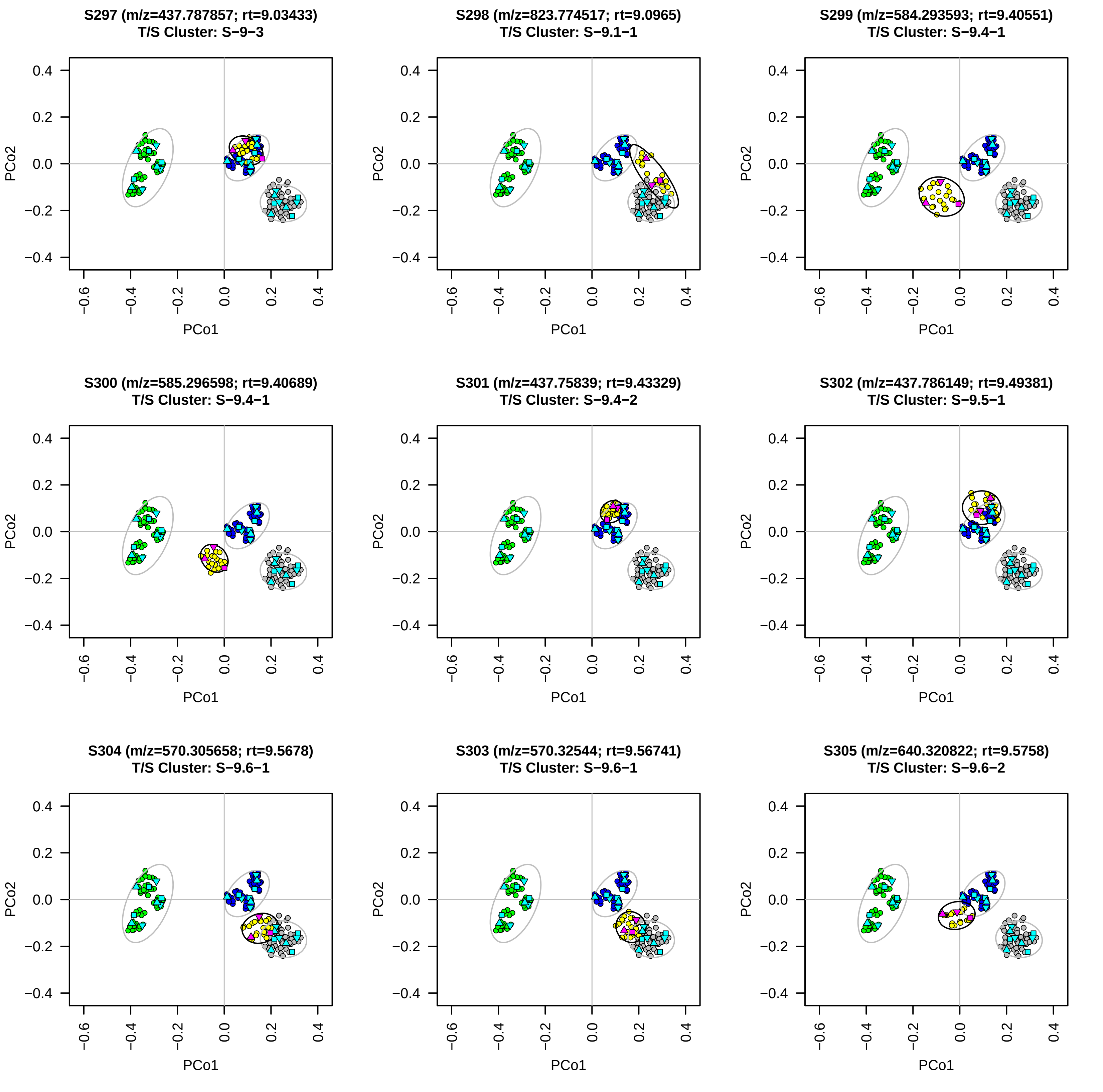
S297 (m/z=437.787857; rt=9.03433)
T/S Cluster: S−9−3
S298 (m/z=823.774517; rt=9.0965)
T/S Cluster: S−9.1−1
S299 (m/z=584.293593; rt=9.40551)
T/S Cluster: S−9.4−1
S300 (m/z=585.296598; rt=9.40689)
T/S Cluster: S−9.4−1
S301 (m/z=437.75839; rt=9.43329)
T/S Cluster: S−9.4−2
S302 (m/z=437.786149; rt=9.49381)
T/S Cluster: S−9.5−1
S304 (m/z=570.305658; rt=9.5678)
T/S Cluster: S−9.6−1
S303 (m/z=570.32544; rt=9.56741)
T/S Cluster: S−9.6−1
S305 (m/z=640.320822; rt=9.5758)
T/S Cluster: S−9.6−2
PCo1
PCo2
−0.6
−0.4
−0.2
0.0
0.2
0.4

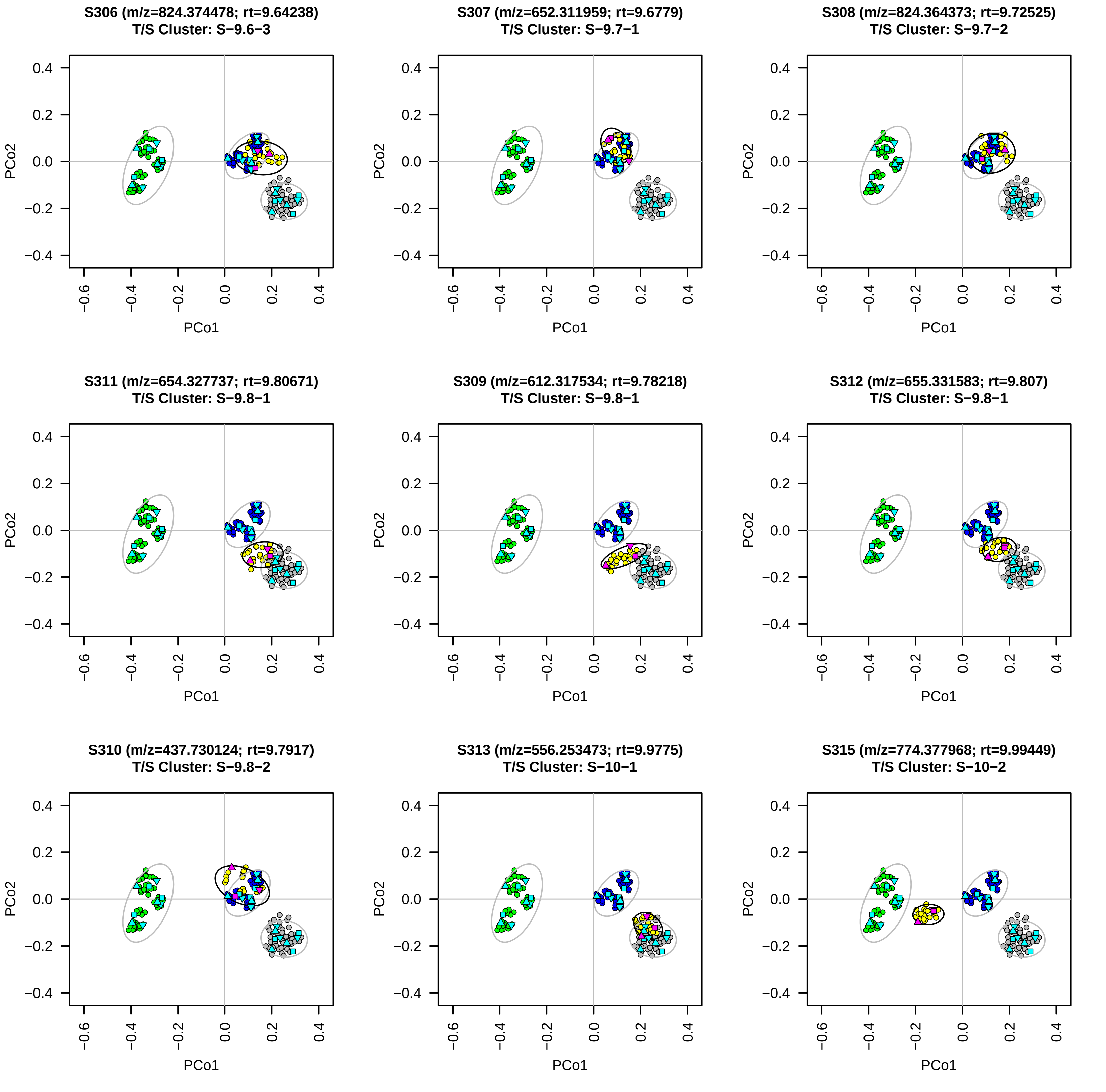
S306 (m/z=824.374478; rt=9.64238)
T/S Cluster: S−9.6−3
S307 (m/z=652.311959; rt=9.6779)
T/S Cluster: S−9.7−1
S308 (m/z=824.364373; rt=9.72525)
T/S Cluster: S−9.7−2
S311 (m/z=654.327737; rt=9.80671)
T/S Cluster: S−9.8−1
S309 (m/z=612.317534; rt=9.78218)
T/S Cluster: S−9.8−1
S312 (m/z=655.331583; rt=9.807)
T/S Cluster: S−9.8−1
S310 (m/z=437.730124; rt=9.7917)
T/S Cluster: S−9.8−2
S313 (m/z=556.253473; rt=9.9775)
T/S Cluster: S−10−1
S315 (m/z=774.377968; rt=9.99449)
T/S Cluster: S−10−2
PCo1
PCo2

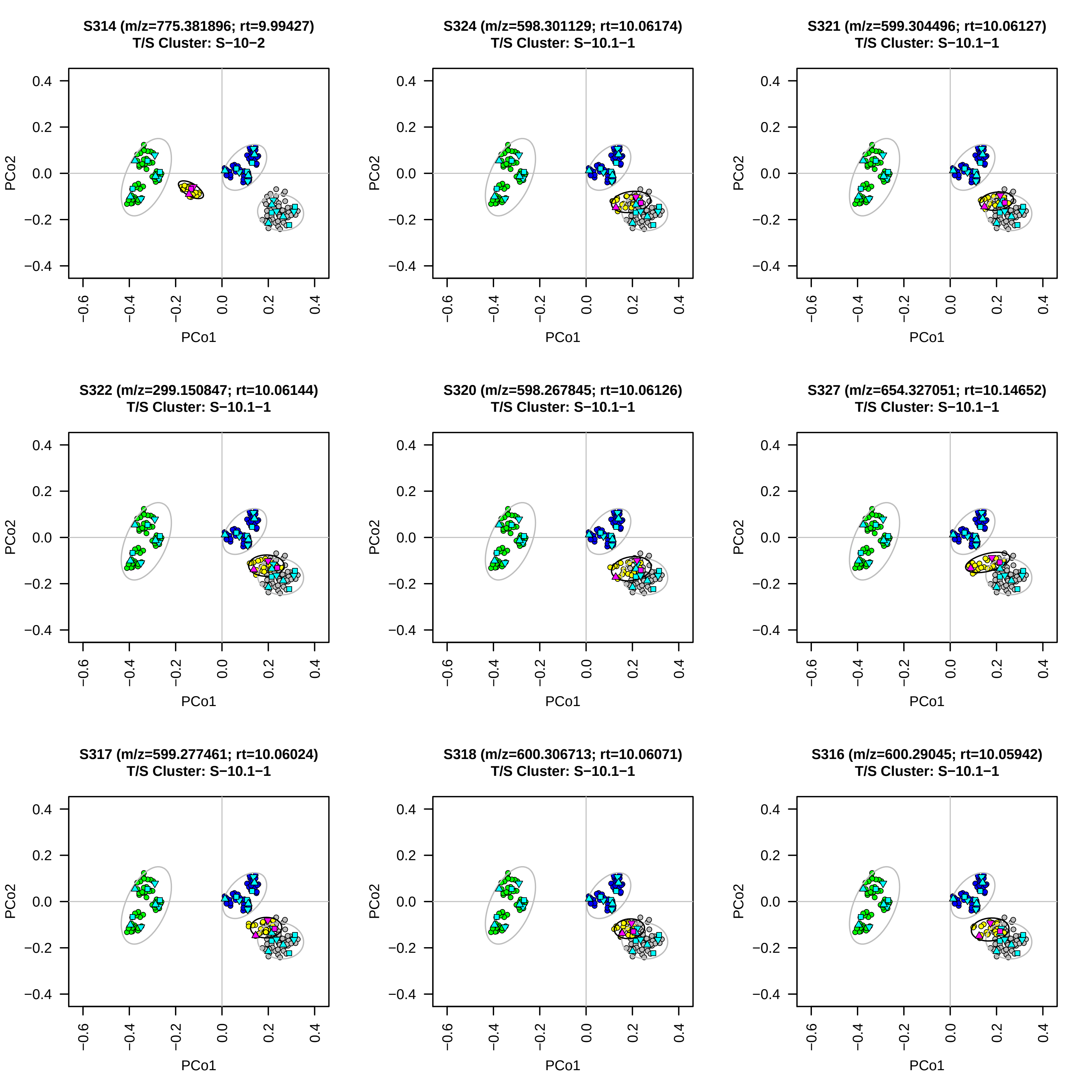
S314 (m/z=775.381896; rt=9.99427)
T/S Cluster: S−10−2
S324 (m/z=598.301129; rt=10.06174)
T/S Cluster: S−10.1−1
S321 (m/z=599.304496; rt=10.06127)
T/S Cluster: S−10.1−1
S322 (m/z=299.150847; rt=10.06144)
T/S Cluster: S−10.1−1
S320 (m/z=598.267845; rt=10.06126)
T/S Cluster: S−10.1−1
S327 (m/z=654.327051; rt=10.14652)
T/S Cluster: S−10.1−1
S317 (m/z=599.277461; rt=10.06024)
T/S Cluster: S−10.1−1
S318 (m/z=600.306713; rt=10.06071)
T/S Cluster: S−10.1−1
S316 (m/z=600.29045; rt=10.05942)
T/S Cluster: S−10.1−1
PCo1
PCo2

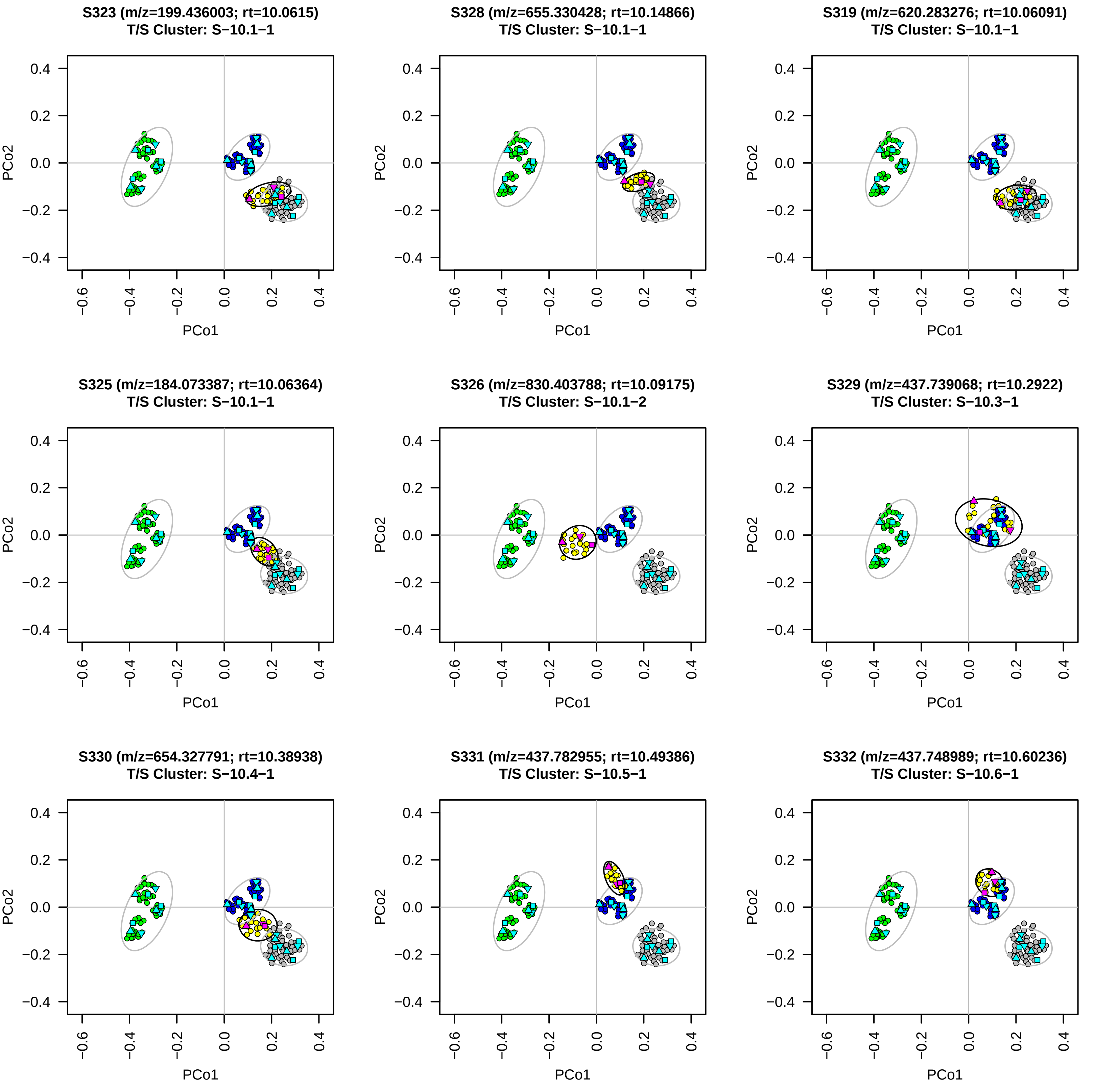
S323 (m/z=199.436003; rt=10.0615)
T/S Cluster: S−10.1−1
S328 (m/z=655.330428; rt=10.14866)
T/S Cluster: S−10.1−1
S319 (m/z=620.283276; rt=10.06091)
T/S Cluster: S−10.1−1
S325 (m/z=184.073387; rt=10.06364)
T/S Cluster: S−10.1−1
S326 (m/z=830.403788; rt=10.09175)
T/S Cluster: S−10.1−2
S329 (m/z=437.739068; rt=10.2922)
T/S Cluster: S−10.3−1
S330 (m/z=654.327791; rt=10.38938)
T/S Cluster: S−10.4−1
S331 (m/z=437.782955; rt=10.49386)
T/S Cluster: S−10.5−1
S332 (m/z=437.748989; rt=10.60236)
T/S Cluster: S−10.6−1
PCo1
PCo2
−0.6
−0.4
−0.2
0.0
0.2
0.4

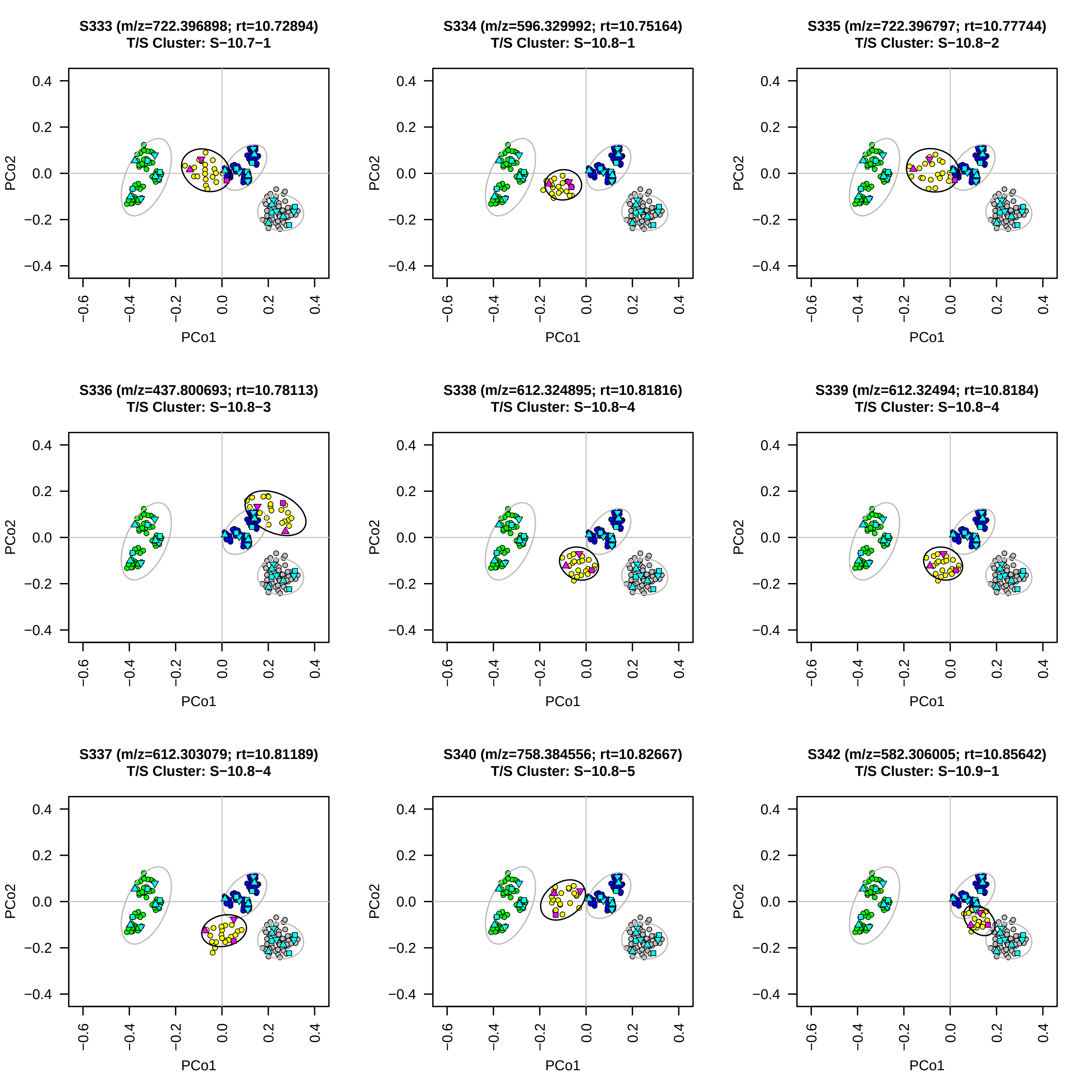
S333 (m/z=722.396898; rt=10.72894)
T/S Cluster: S−10.7−1
S334 (m/z=596.329992; rt=10.75164)
T/S Cluster: S−10.8−1
S335 (m/z=722.396797; rt=10.77744)
T/S Cluster: S−10.8−2
S336 (m/z=437.800693; rt=10.78113)
T/S Cluster: S−10.8−3
S338 (m/z=612.324895; rt=10.81816)
T/S Cluster: S−10.8−4
S339 (m/z=612.32494; rt=10.8184)
T/S Cluster: S−10.8−4
S337 (m/z=612.303079; rt=10.81189)
T/S Cluster: S−10.8−4
S340 (m/z=758.384556; rt=10.82667)
T/S Cluster: S−10.8−5
S342 (m/z=582.306005; rt=10.85642)
T/S Cluster: S−10.9−1
PCo1
PCo2
0.4
0.2
0.0
−0.2
−0.4
−0.6

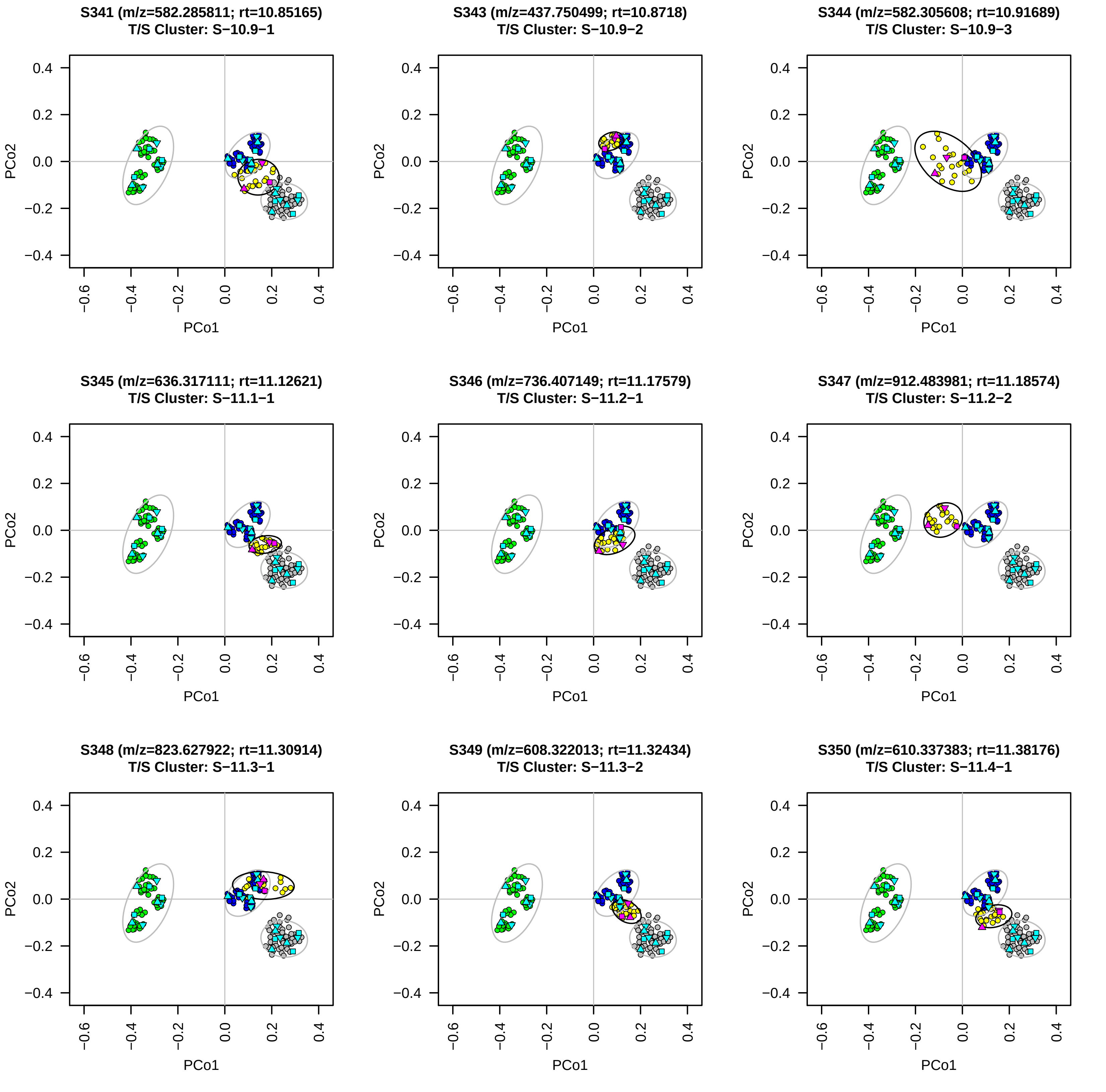
S341 (m/z=582.285811; rt=10.85165)
T/S Cluster: S−10.9−1
S343 (m/z=437.750499; rt=10.8718)
T/S Cluster: S−10.9−2
S344 (m/z=582.305608; rt=10.91689)
T/S Cluster: S−10.9−3
S345 (m/z=636.317111; rt=11.12621)
T/S Cluster: S−11.1−1
S346 (m/z=736.407149; rt=11.17579)
T/S Cluster: S−11.2−1
S347 (m/z=912.483981; rt=11.18574)
T/S Cluster: S−11.2−2
S348 (m/z=823.627922; rt=11.30914)
T/S Cluster: S−11.3−1
S349 (m/z=608.322013; rt=11.32434)
T/S Cluster: S−11.3−2
S350 (m/z=610.337383; rt=11.38176)
T/S Cluster: S−11.4−1
PCo1
PCo2
0.4
0.2
0.0
−0.2
−0.4
−0.6

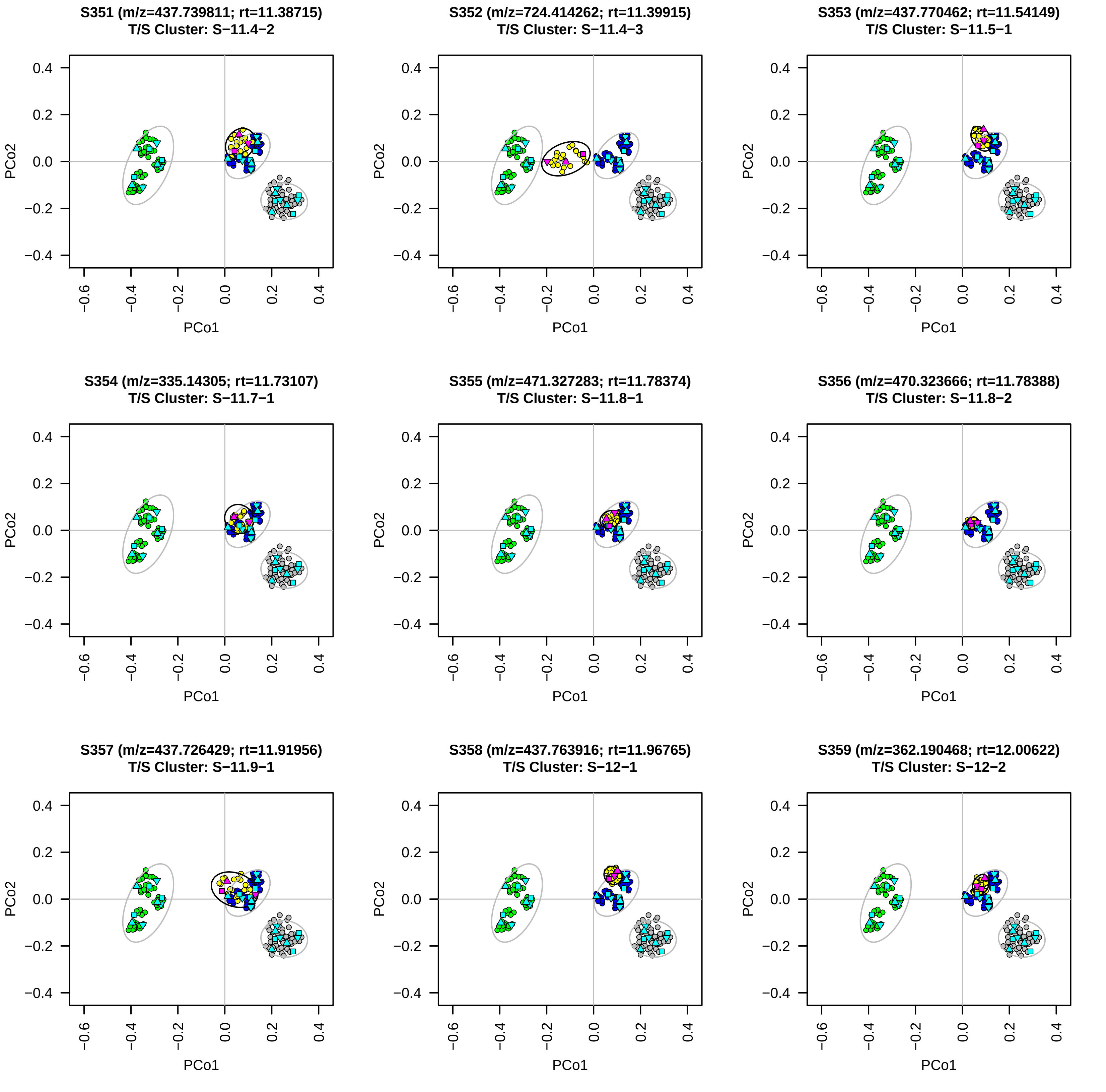
S351 (m/z=437.739811; rt=11.38715)
T/S Cluster: S−11.4−2
S352 (m/z=724.414262; rt=11.39915)
T/S Cluster: S−11.4−3
S353 (m/z=437.770462; rt=11.54149)
T/S Cluster: S−11.5−1
S354 (m/z=335.14305; rt=11.73107)
T/S Cluster: S−11.7−1
S355 (m/z=471.327283; rt=11.78374)
T/S Cluster: S−11.8−1
S356 (m/z=470.323666; rt=11.78388)
T/S Cluster: S−11.8−2
S357 (m/z=437.726429; rt=11.91956)
T/S Cluster: S−11.9−1
S358 (m/z=437.763916; rt=11.96765)
T/S Cluster: S−12−1
S359 (m/z=362.190468; rt=12.00622)
T/S Cluster: S−12−2
PCo1
PCo2

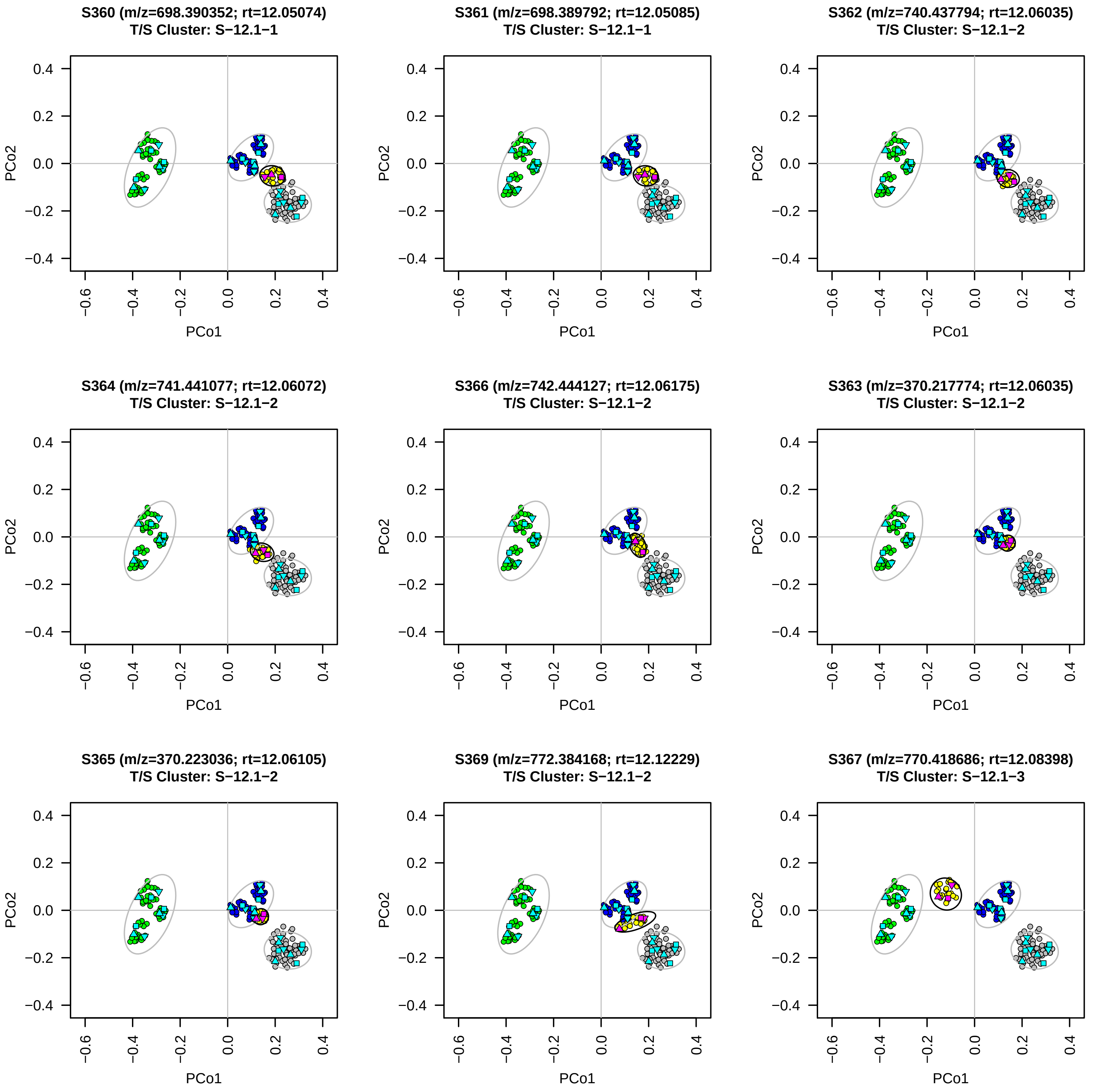
S360 (m/z=698.390352; rt=12.05074)
T/S Cluster: S−12.1−1
S361 (m/z=698.389792; rt=12.05085)
T/S Cluster: S−12.1−1
S362 (m/z=740.437794; rt=12.06035)
T/S Cluster: S−12.1−2
S364 (m/z=741.441077; rt=12.06072)
T/S Cluster: S−12.1−2
S366 (m/z=742.444127; rt=12.06175)
T/S Cluster: S−12.1−2
S363 (m/z=370.217774; rt=12.06035)
T/S Cluster: S−12.1−2
S365 (m/z=370.223036; rt=12.06105)
T/S Cluster: S−12.1−2
S369 (m/z=772.384168; rt=12.12229)
T/S Cluster: S−12.1−2
S367 (m/z=770.418686; rt=12.08398)
T/S Cluster: S−12.1−3
PCo1
PCo2
−0.6
−0.4
−0.2
0.0
0.2
0.4

S368 (m/z=708.418704; rt=12.08746)
T/S Cluster: S−12.1−4

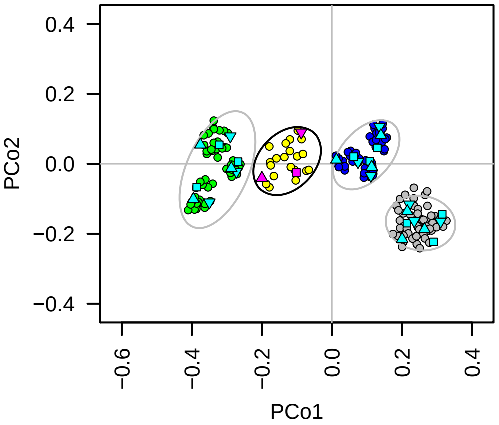

S370 (m/z=722.414594; rt=12.12819)
T/S Cluster: S−12.1−5

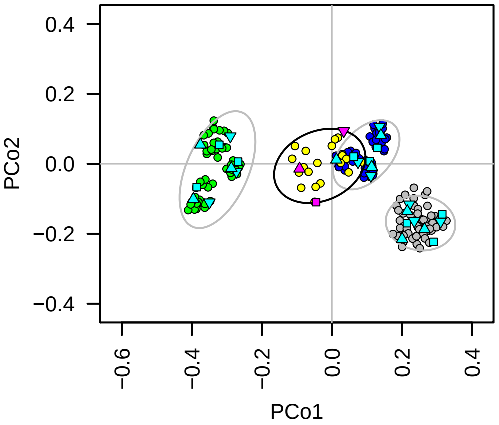

S371 (m/z=752.443916; rt=12.15204)
T/S Cluster: S−12.2−1

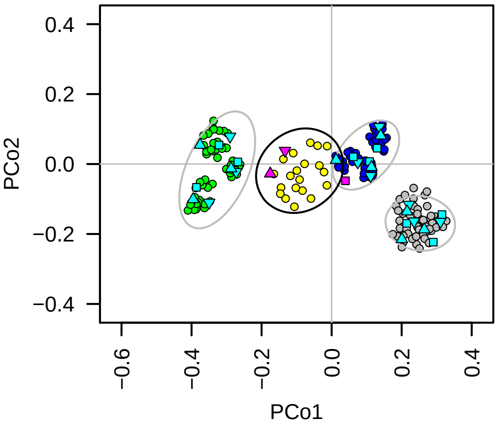

S372 (m/z=722.398853; rt=12.16149)
T/S Cluster: S−12.2−2

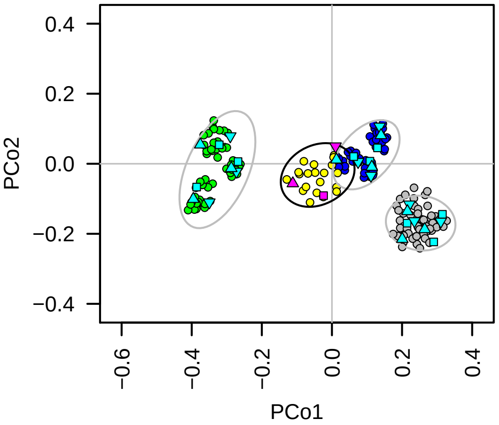

S373 (m/z=249.106007; rt=12.16161)
T/S Cluster: S−12.2−3

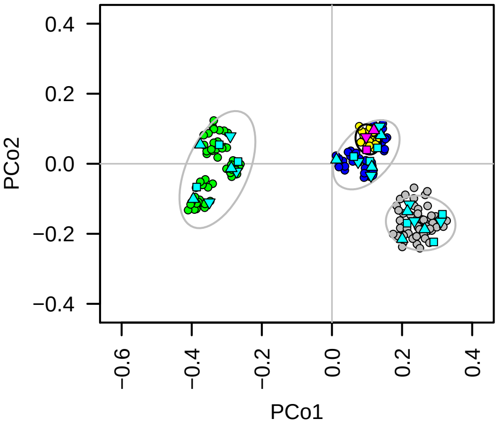

S374 (m/z=249.103192; rt=12.16165)
T/S Cluster: S−12.2−4

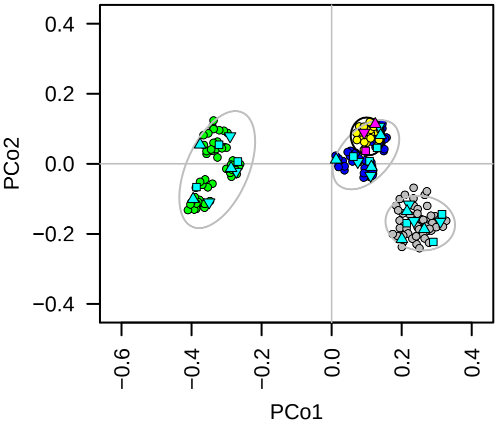

S375 (m/z=710.426472; rt=12.17141)
T/S Cluster: S−12.2−5

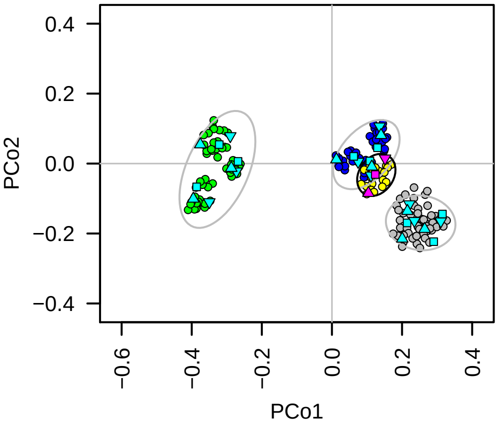

S376 (m/z=898.511282; rt=12.18795)
T/S Cluster: S−12.2−6

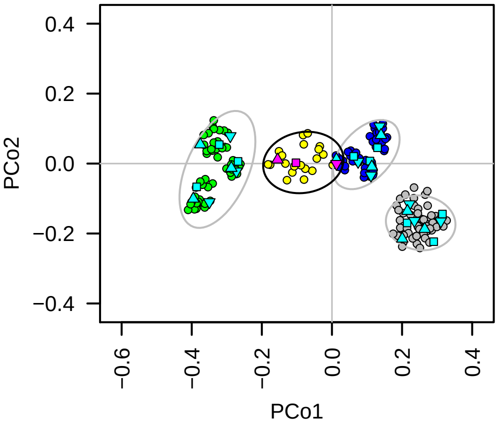

S377 (m/z=722.430685; rt=12.19378)
T/S Cluster: S−12.2−7

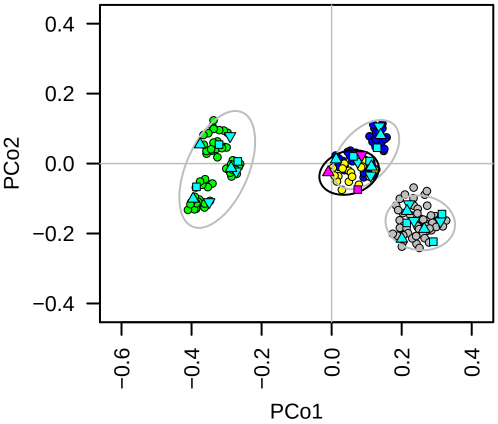

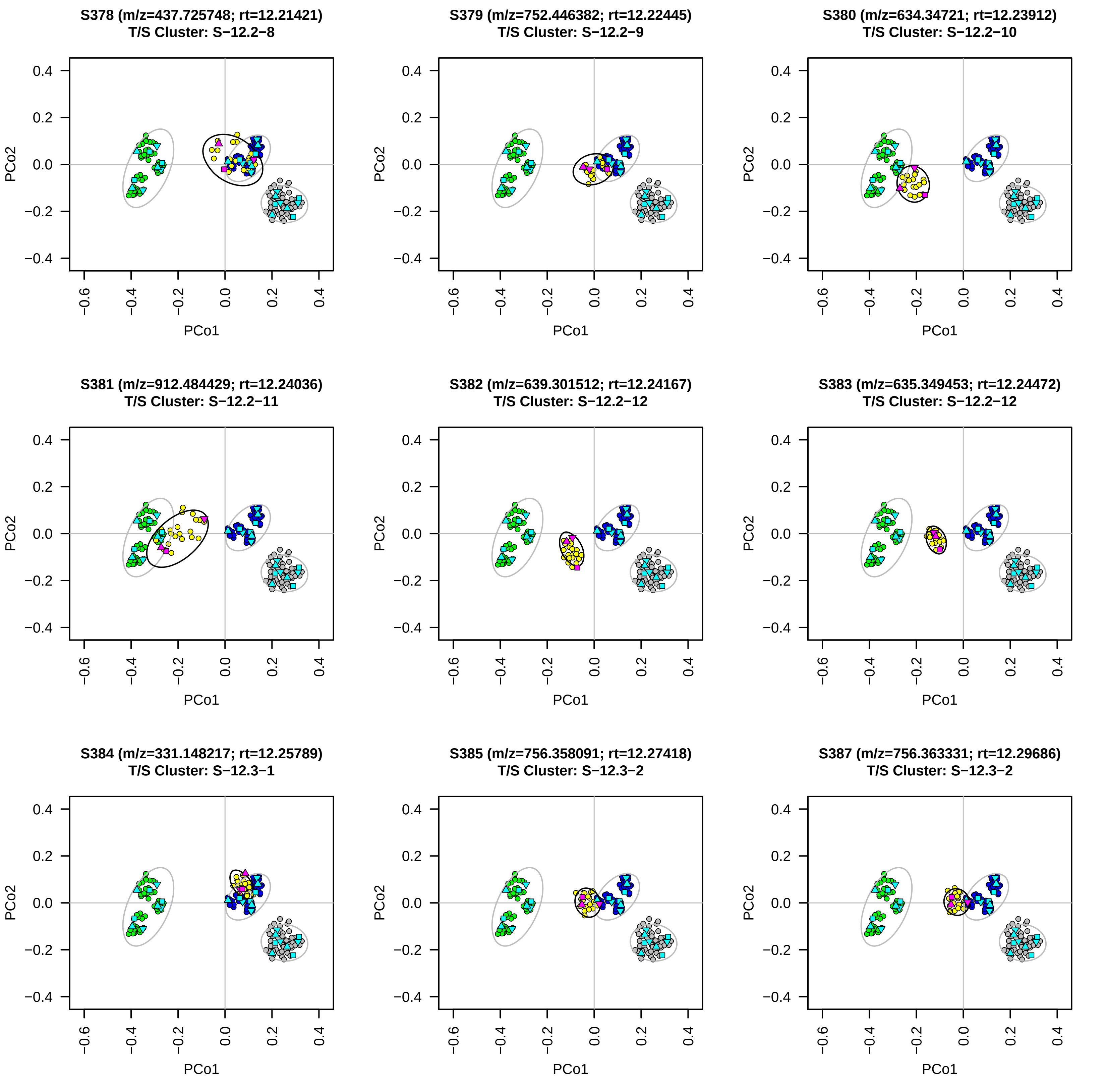
S378 (m/z=437.725748; rt=12.21421)
T/S Cluster: S−12.2−8
S379 (m/z=752.446382; rt=12.22445)
T/S Cluster: S−12.2−9
S380 (m/z=634.34721; rt=12.23912)
T/S Cluster: S−12.2−10
S381 (m/z=912.484429; rt=12.24036)
T/S Cluster: S−12.2−11
S382 (m/z=639.301512; rt=12.24167)
T/S Cluster: S−12.2−12
S383 (m/z=635.349453; rt=12.24472)
T/S Cluster: S−12.2−12
S384 (m/z=331.148217; rt=12.25789)
T/S Cluster: S−12.3−1
S385 (m/z=756.358091; rt=12.27418)
T/S Cluster: S−12.3−2
S387 (m/z=756.363331; rt=12.29686)
T/S Cluster: S−12.3−2
PCo1
PCo2
−0.6
−0.4
−0.2
0.0
0.2
0.4

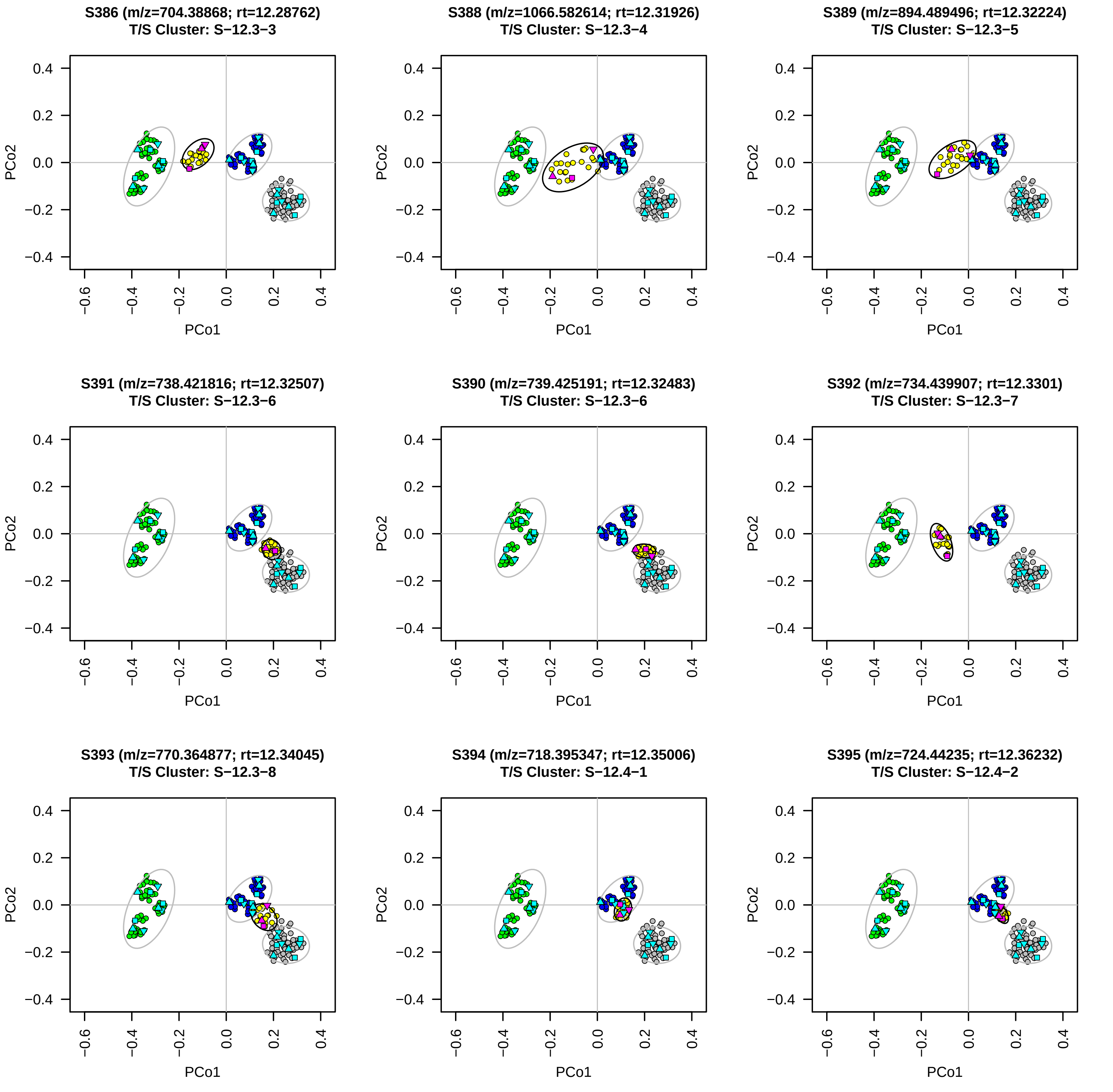
S386 (m/z=704.38868; rt=12.28762)
T/S Cluster: S−12.3−3
S388 (m/z=1066.582614; rt=12.31926)
T/S Cluster: S−12.3−4
S389 (m/z=894.489496; rt=12.32224)
T/S Cluster: S−12.3−5
S391 (m/z=738.421816; rt=12.32507)
T/S Cluster: S−12.3−6
S390 (m/z=739.425191; rt=12.32483)
T/S Cluster: S−12.3−6
S392 (m/z=734.439907; rt=12.3301)
T/S Cluster: S−12.3−7
S393 (m/z=770.364877; rt=12.34045)
T/S Cluster: S−12.3−8
S394 (m/z=718.395347; rt=12.35006)
T/S Cluster: S−12.4−1
S395 (m/z=724.44235; rt=12.36232)
T/S Cluster: S−12.4−2
PCo1
PCo2

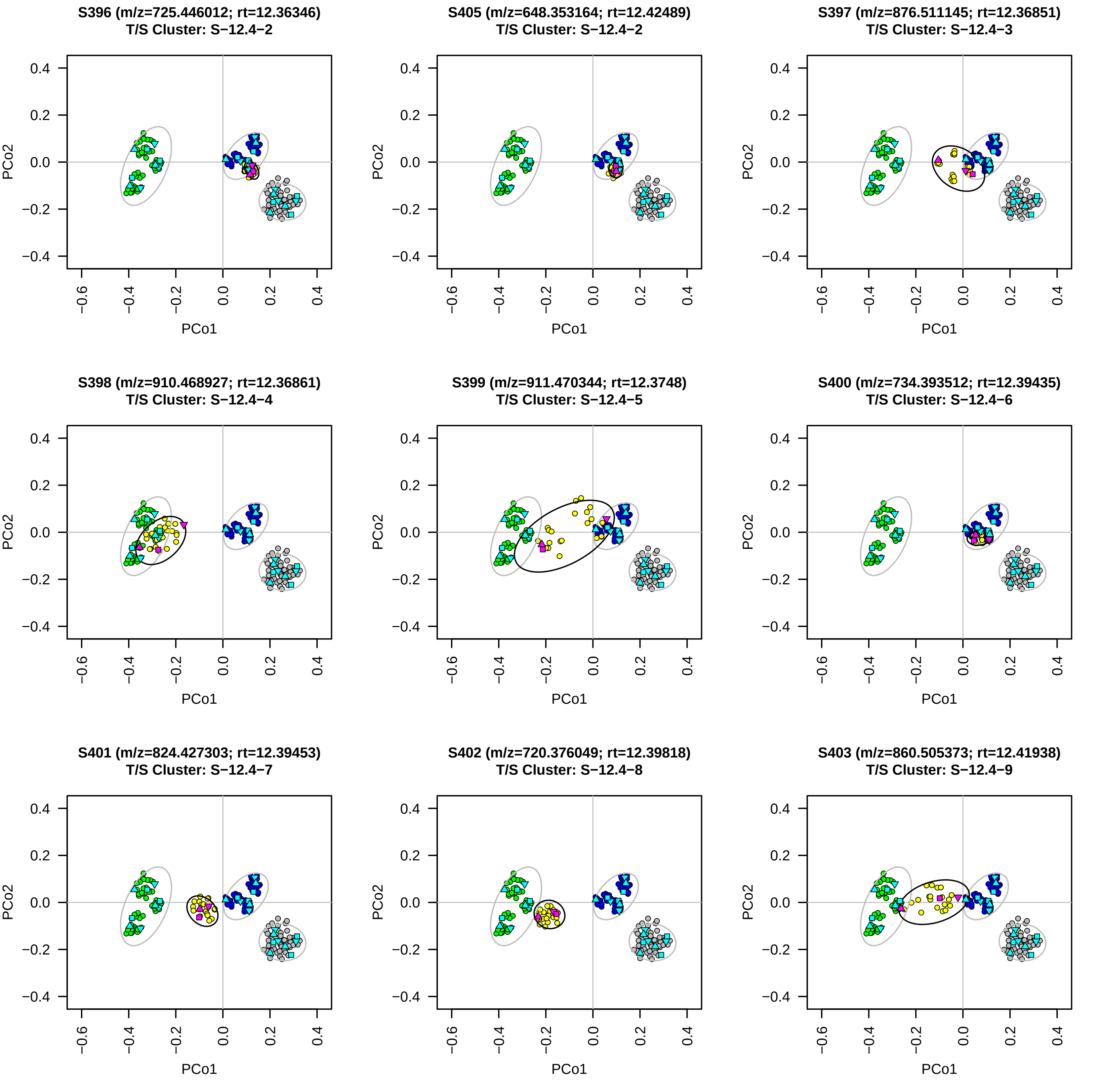
S396 (m/z=725.446012; rt=12.36346)
T/S Cluster: S−12.4−2
S405 (m/z=648.353164; rt=12.42489)
T/S Cluster: S−12.4−2
S397 (m/z=876.511145; rt=12.36851)
T/S Cluster: S−12.4−3
S398 (m/z=910.468927; rt=12.36861)
T/S Cluster: S−12.4−4
S399 (m/z=911.470344; rt=12.3748)
T/S Cluster: S−12.4−5
S400 (m/z=734.393512; rt=12.39435)
T/S Cluster: S−12.4−6
S401 (m/z=824.427303; rt=12.39453)
T/S Cluster: S−12.4−7
S402 (m/z=720.376049; rt=12.39818)
T/S Cluster: S−12.4−8
S403 (m/z=860.505373; rt=12.41938)
T/S Cluster: S−12.4−9
PCo2
PCo1
0.4
0.2
0.0
−0.2
−0.4
−0.6
−0.4
−0.2
0.0
0.2
0.4

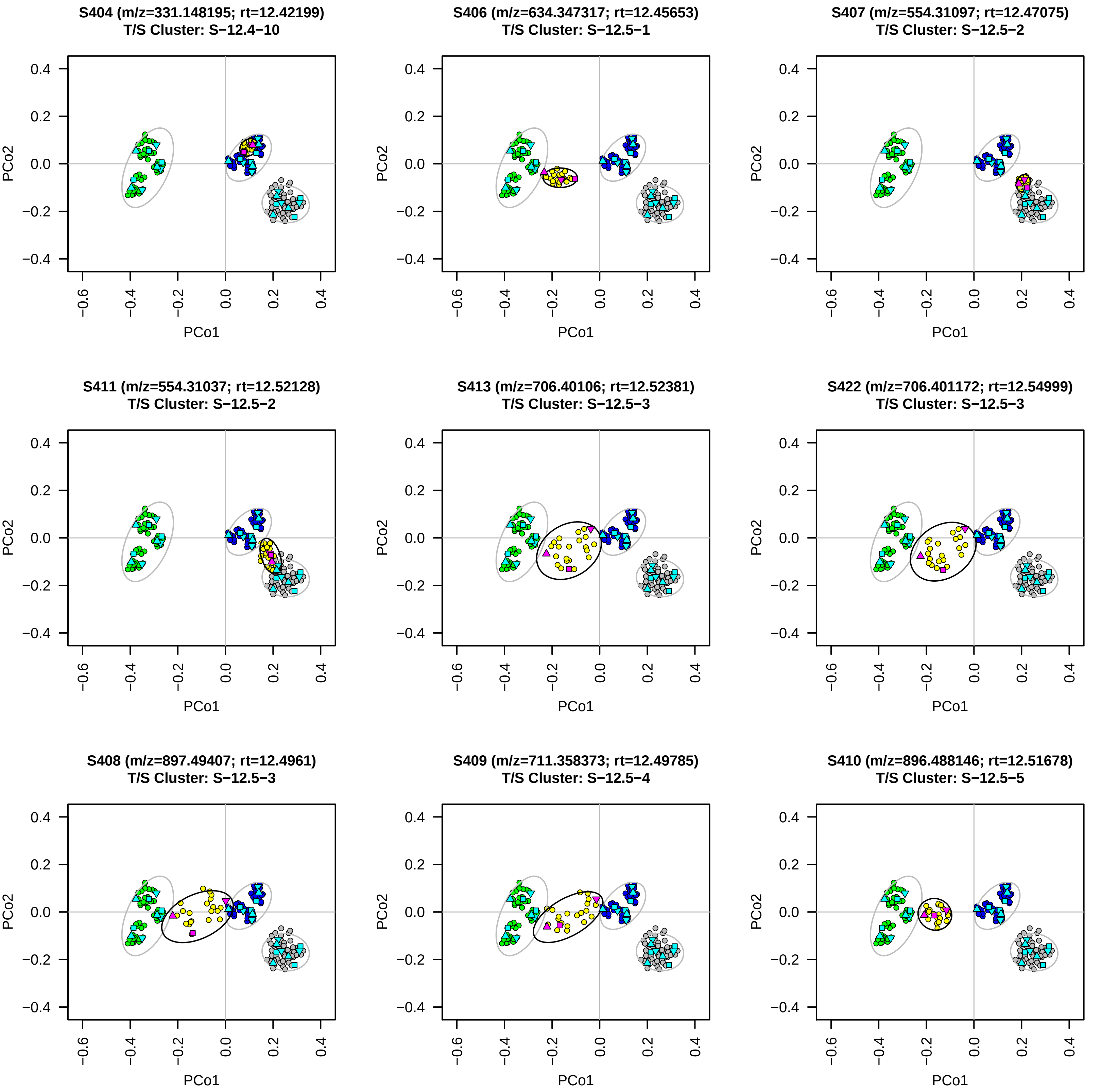
S404 (m/z=331.148195; rt=12.42199)
T/S Cluster: S−12.4−10
S406 (m/z=634.347317; rt=12.45653)
T/S Cluster: S−12.5−1
S407 (m/z=554.31097; rt=12.47075)
T/S Cluster: S−12.5−2
S411 (m/z=554.31037; rt=12.52128)
T/S Cluster: S−12.5−2
S413 (m/z=706.40106; rt=12.52381)
T/S Cluster: S−12.5−3
S422 (m/z=706.401172; rt=12.54999)
T/S Cluster: S−12.5−3
S408 (m/z=897.49407; rt=12.4961)
T/S Cluster: S−12.5−3
S409 (m/z=711.358373; rt=12.49785)
T/S Cluster: S−12.5−4
S410 (m/z=896.488146; rt=12.51678)
T/S Cluster: S−12.5−5
PCo1
PCo2
−0.6
−0.4
−0.2
0.0
0.2
0.4

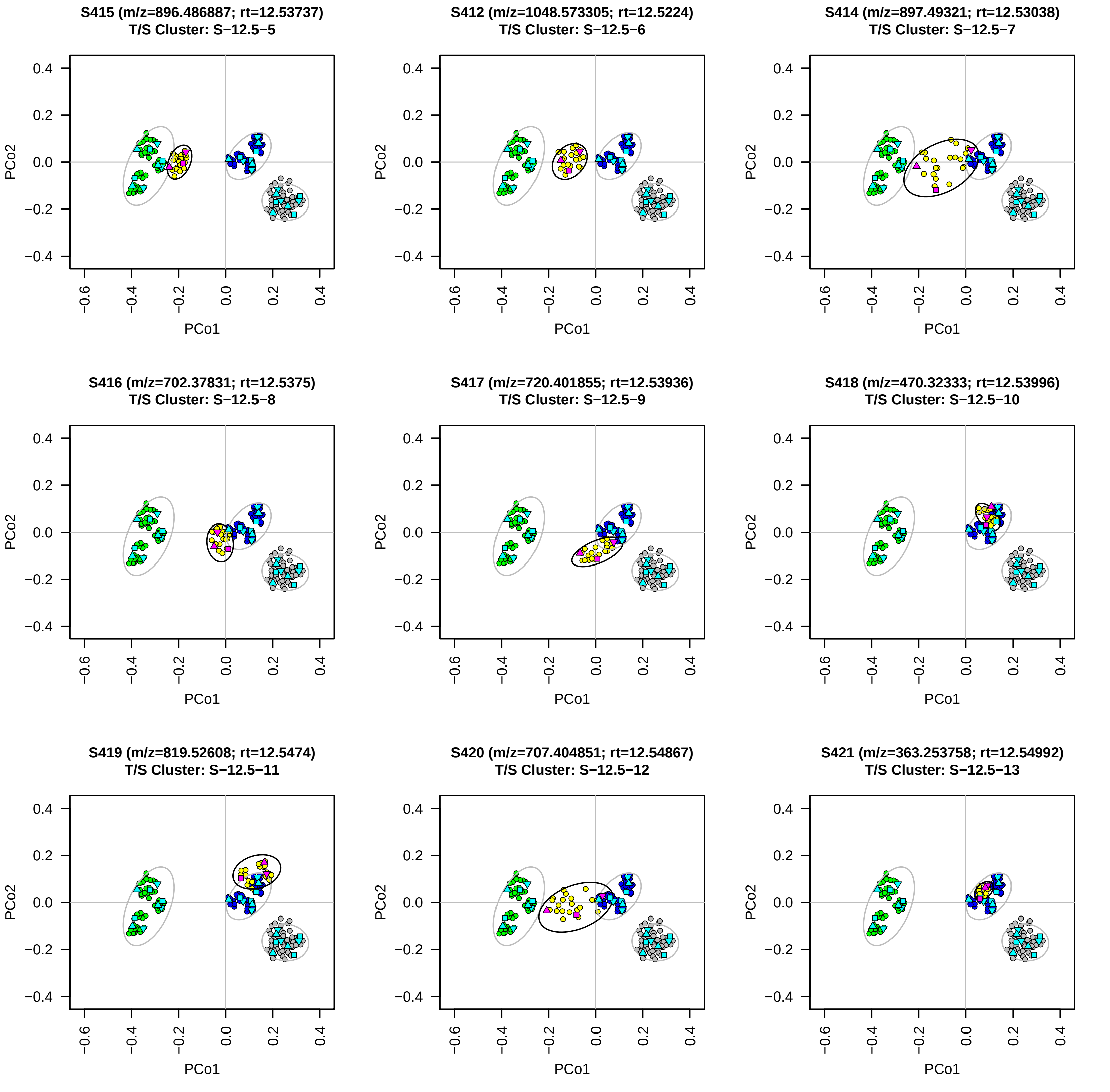
S415 (m/z=896.486887; rt=12.53737)
T/S Cluster: S−12.5−5
S412 (m/z=1048.573305; rt=12.5224)
T/S Cluster: S−12.5−6
S414 (m/z=897.49321; rt=12.53038)
T/S Cluster: S−12.5−7
S416 (m/z=702.37831; rt=12.5375)
T/S Cluster: S−12.5−8
S417 (m/z=720.401855; rt=12.53936)
T/S Cluster: S−12.5−9
S418 (m/z=470.32333; rt=12.53996)
T/S Cluster: S−12.5−10
S419 (m/z=819.52608; rt=12.5474)
T/S Cluster: S−12.5−11
S420 (m/z=707.404851; rt=12.54867)
T/S Cluster: S−12.5−12
S421 (m/z=363.253758; rt=12.54992)
T/S Cluster: S−12.5−13
PCo1
PCo2
0.4
0.2
0.0
−0.2
−0.4
−0.6

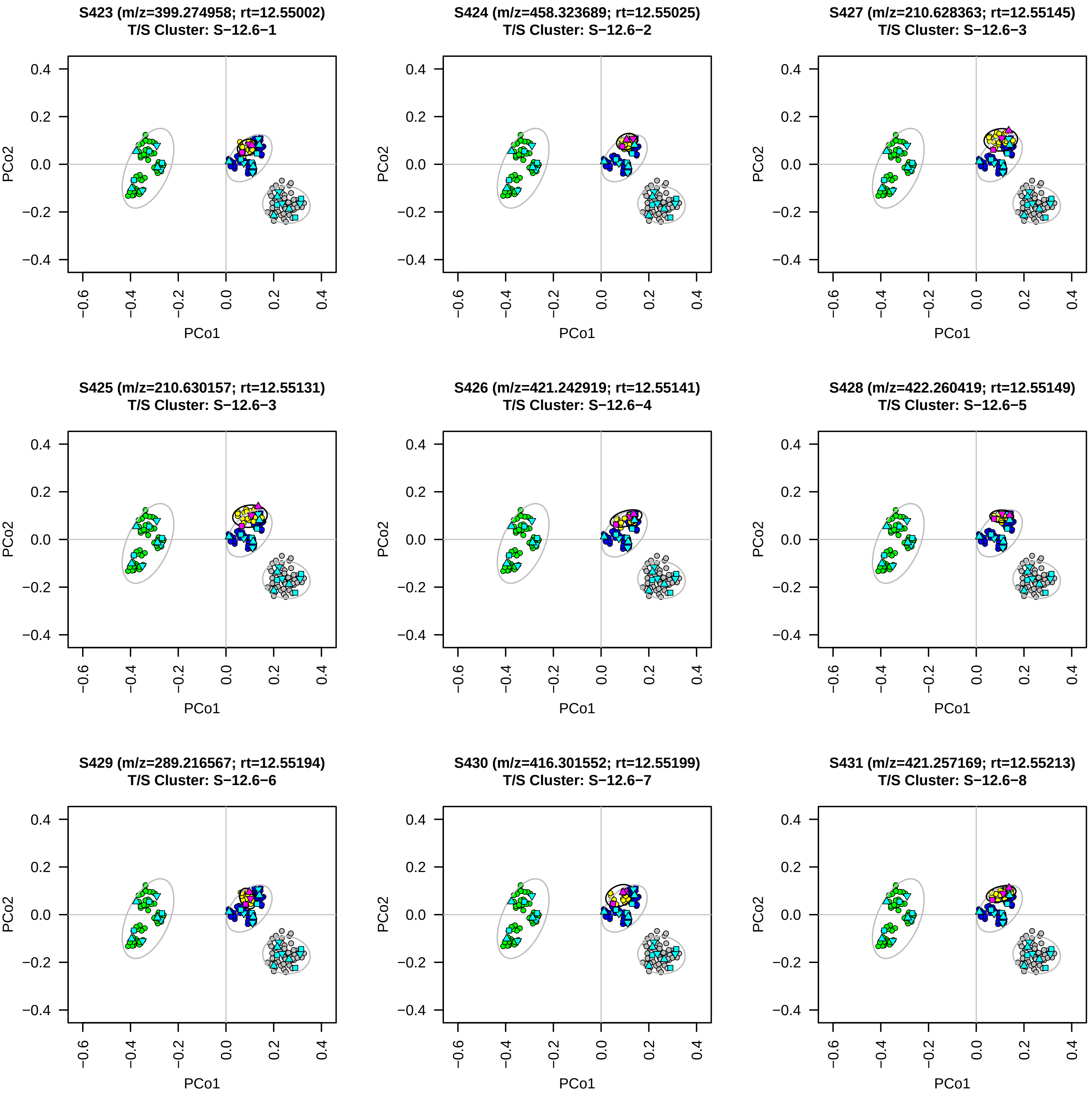
S423 (m/z=399.274958; rt=12.55002)
T/S Cluster: S−12.6−1
S424 (m/z=458.323689; rt=12.55025)
T/S Cluster: S−12.6−2
S427 (m/z=210.628363; rt=12.55145)
T/S Cluster: S−12.6−3
S425 (m/z=210.630157; rt=12.55131)
T/S Cluster: S−12.6−3
S426 (m/z=421.242919; rt=12.55141)
T/S Cluster: S−12.6−4
S428 (m/z=422.260419; rt=12.55149)
T/S Cluster: S−12.6−5
S429 (m/z=289.216567; rt=12.55194)
T/S Cluster: S−12.6−6
S430 (m/z=416.301552; rt=12.55199)
T/S Cluster: S−12.6−7
S431 (m/z=421.257169; rt=12.55213)
T/S Cluster: S−12.6−8
PCo1
PCo2
−0.6
−0.4
−0.2
0.0
0.2
0.4

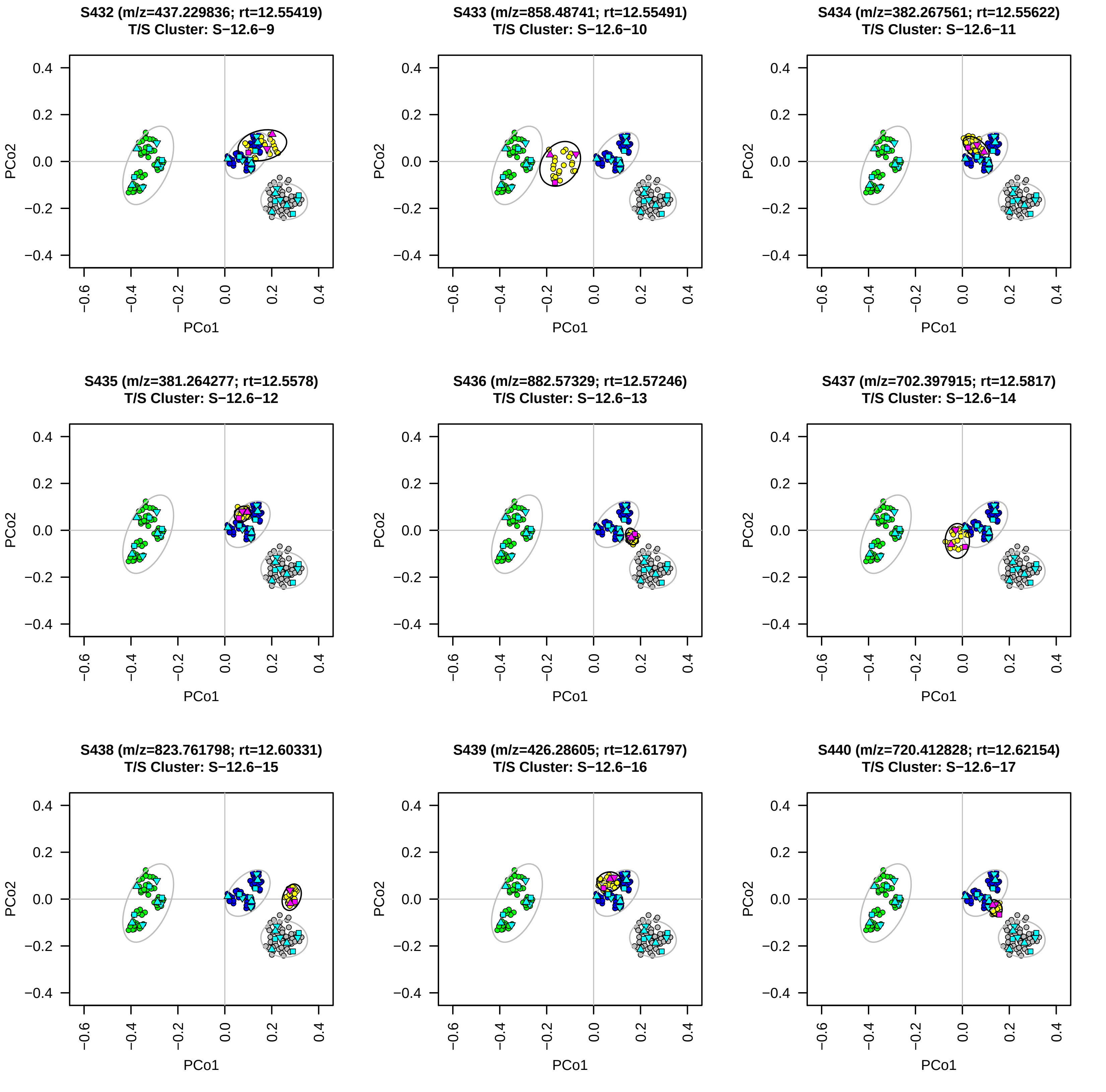
S432 (m/z=437.229836; rt=12.55419)
T/S Cluster: S−12.6−9
S433 (m/z=858.48741; rt=12.55491)
T/S Cluster: S−12.6−10
S434 (m/z=382.267561; rt=12.55622)
T/S Cluster: S−12.6−11
S435 (m/z=381.264277; rt=12.5578)
T/S Cluster: S−12.6−12
S436 (m/z=882.57329; rt=12.57246)
T/S Cluster: S−12.6−13
S437 (m/z=702.397915; rt=12.5817)
T/S Cluster: S−12.6−14
S438 (m/z=823.761798; rt=12.60331)
T/S Cluster: S−12.6−15
S439 (m/z=426.28605; rt=12.61797)
T/S Cluster: S−12.6−16
S440 (m/z=720.412828; rt=12.62154)
T/S Cluster: S−12.6−17
PCo1
PCo2

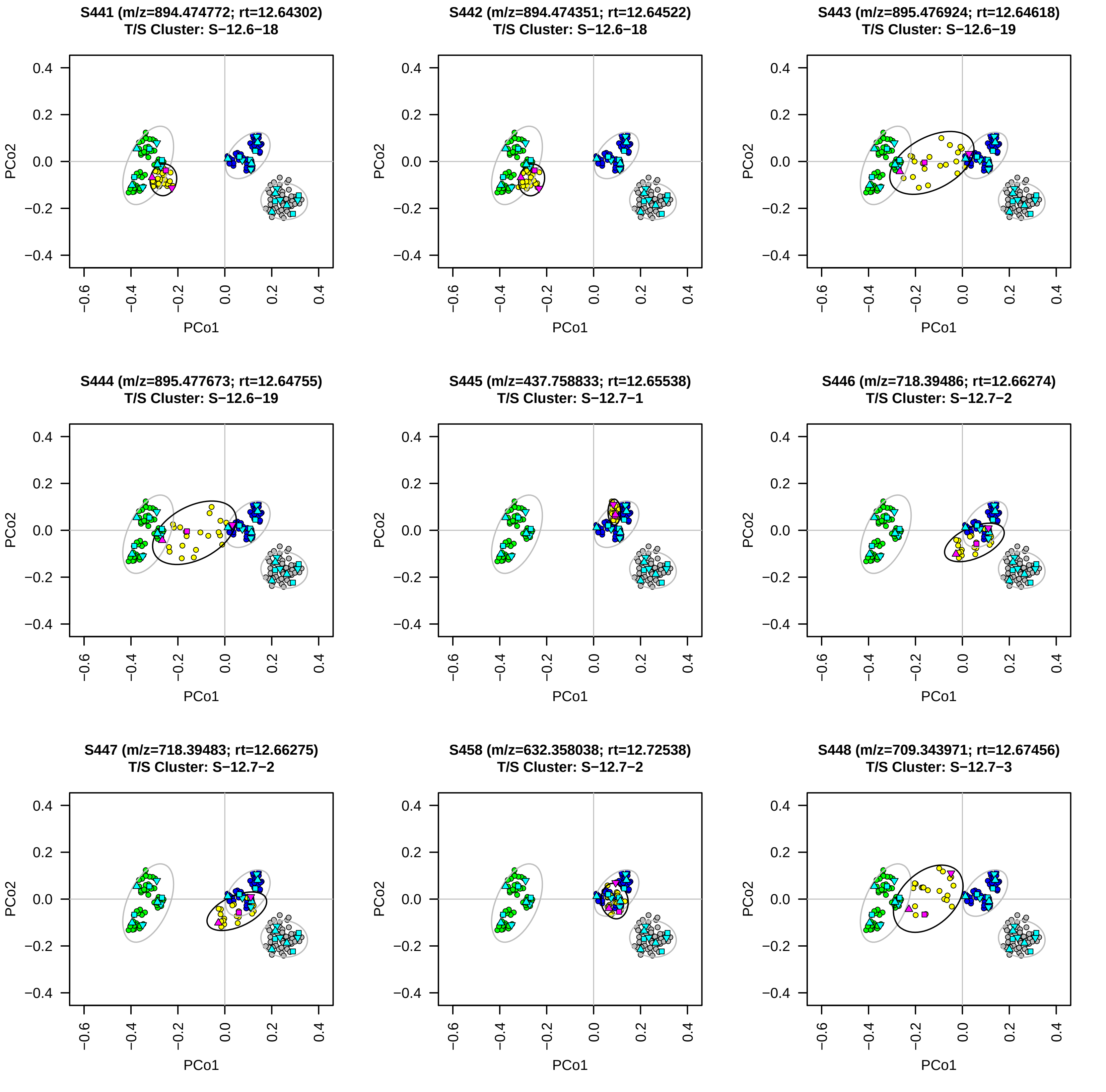
S441 (m/z=894.474772; rt=12.64302)
T/S Cluster: S−12.6−18
S442 (m/z=894.474351; rt=12.64522)
T/S Cluster: S−12.6−18
S443 (m/z=895.476924; rt=12.64618)
T/S Cluster: S−12.6−19
S444 (m/z=895.477673; rt=12.64755)
T/S Cluster: S−12.6−19
S445 (m/z=437.758833; rt=12.65538)
T/S Cluster: S−12.7−1
S446 (m/z=718.39486; rt=12.66274)
T/S Cluster: S−12.7−2
S447 (m/z=718.39483; rt=12.66275)
T/S Cluster: S−12.7−2
S458 (m/z=632.358038; rt=12.72538)
T/S Cluster: S−12.7−2
S448 (m/z=709.343971; rt=12.67456)
T/S Cluster: S−12.7−3
PCo1
PCo2
0.4
0.2
0.0
−0.2
−0.4
−0.6

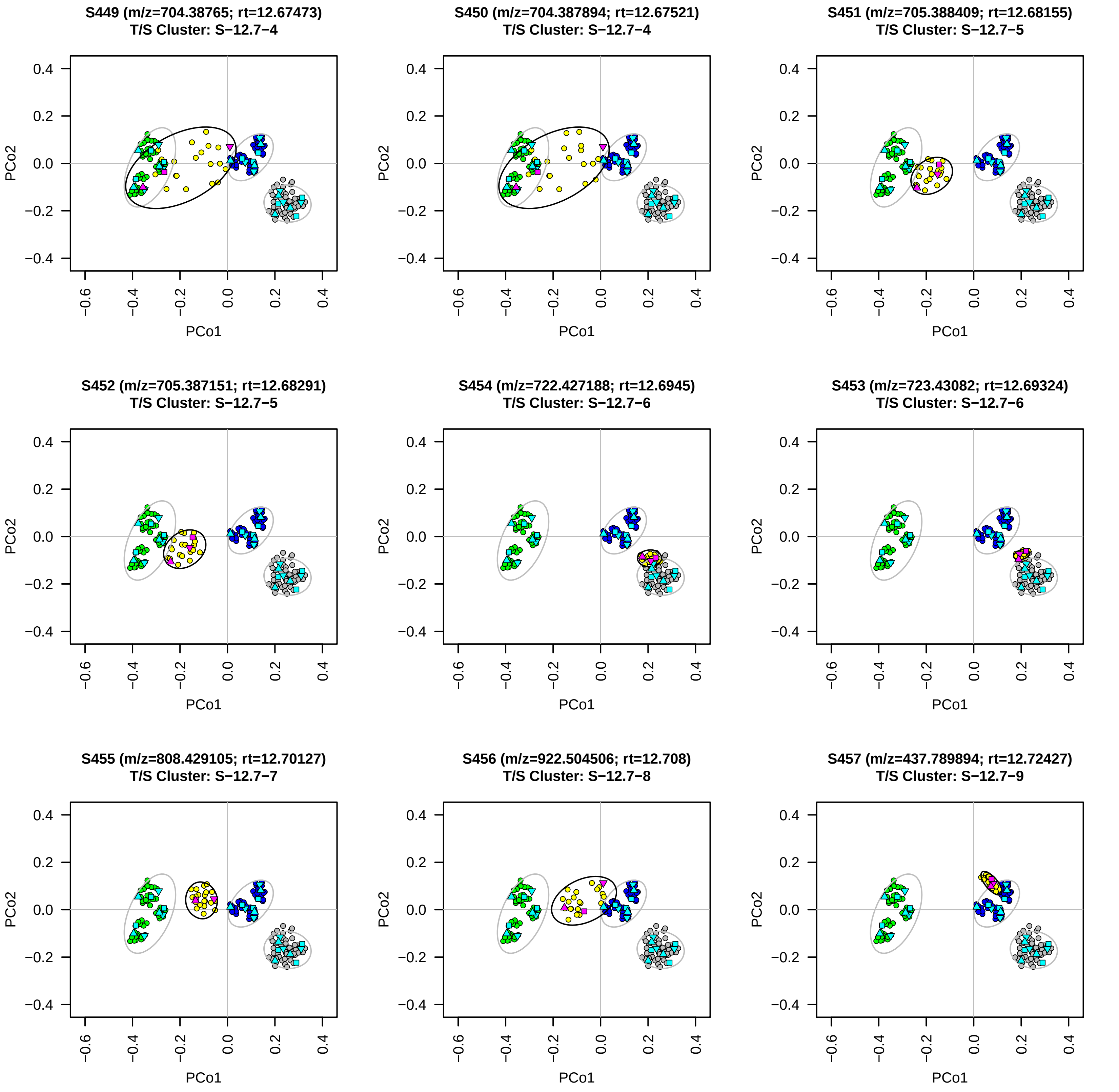
S449 (m/z=704.38765; rt=12.67473)
T/S Cluster: S−12.7−4
S450 (m/z=704.387894; rt=12.67521)
T/S Cluster: S−12.7−4
S451 (m/z=705.388409; rt=12.68155)
T/S Cluster: S−12.7−5
S452 (m/z=705.387151; rt=12.68291)
T/S Cluster: S−12.7−5
S454 (m/z=722.427188; rt=12.6945)
T/S Cluster: S−12.7−6
S453 (m/z=723.43082; rt=12.69324)
T/S Cluster: S−12.7−6
S455 (m/z=808.429105; rt=12.70127)
T/S Cluster: S−12.7−7
S456 (m/z=922.504506; rt=12.708)
T/S Cluster: S−12.7−8
S457 (m/z=437.789894; rt=12.72427)
T/S Cluster: S−12.7−9
PCo1
PCo2
0.4
0.2
0.0
−0.2
−0.4
−0.6

S459 (m/z=374.219087; rt=12.82547)
T/S Cluster: S−12.8−1

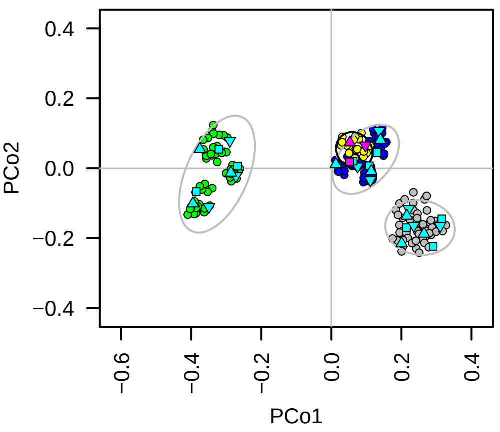

S460 (m/z=366.275916; rt=12.83015)
T/S Cluster: S−12.8−2

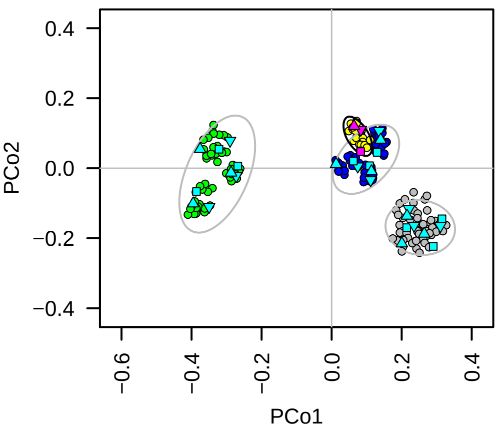

S462 (m/z=366.275717; rt=12.86085)
T/S Cluster: S−12.9−1

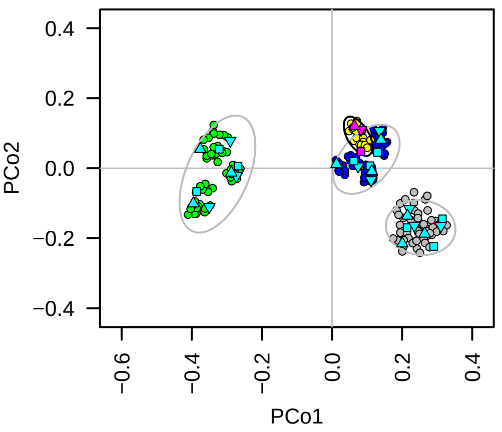

S461 (m/z=366.267965; rt=12.86016)
T/S Cluster: S−12.9−1

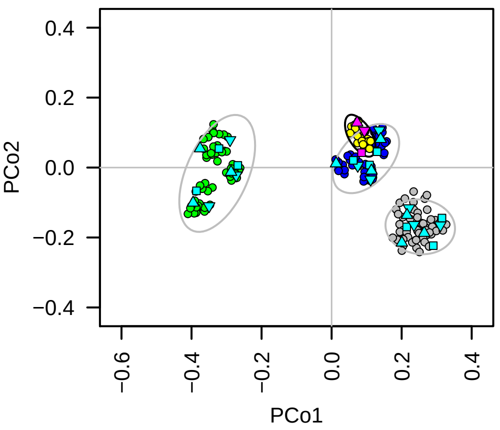

S464 (m/z=263.121651; rt=12.8697)
T/S Cluster: S−12.9−2

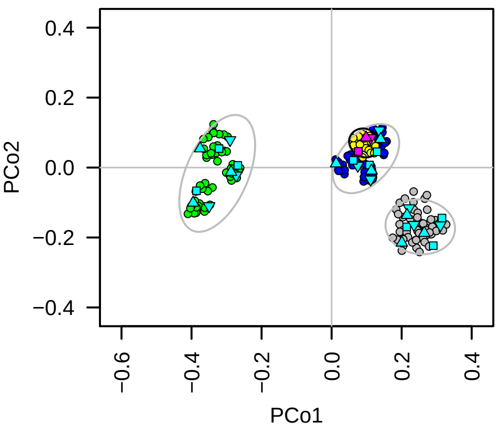

S463 (m/z=263.11771; rt=12.86912)
T/S Cluster: S−12.9−2

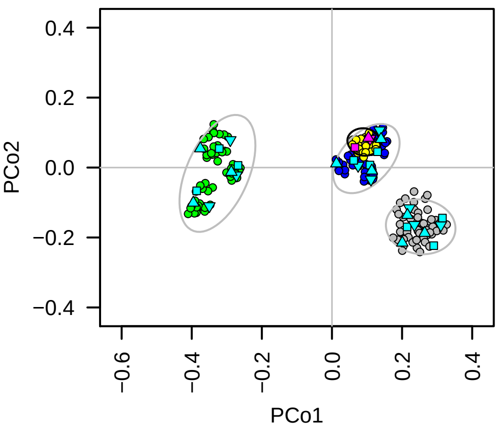

S465 (m/z=687.424499; rt=12.90915)
T/S Cluster: S−12.9−3

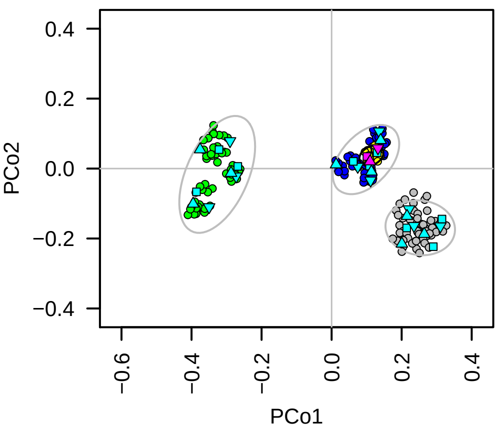

S466 (m/z=666.496271; rt=12.90916)
T/S Cluster: S−12.9−4

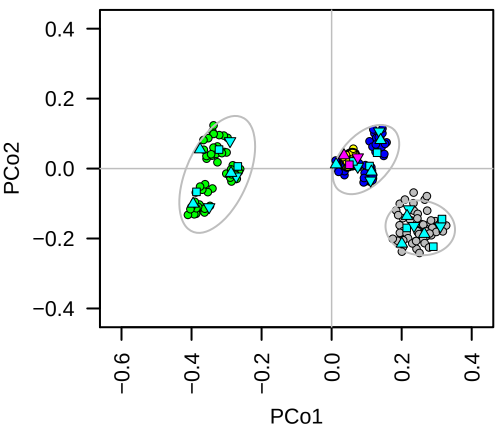

S467 (m/z=108.413383; rt=12.9093)
T/S Cluster: S−12.9−5

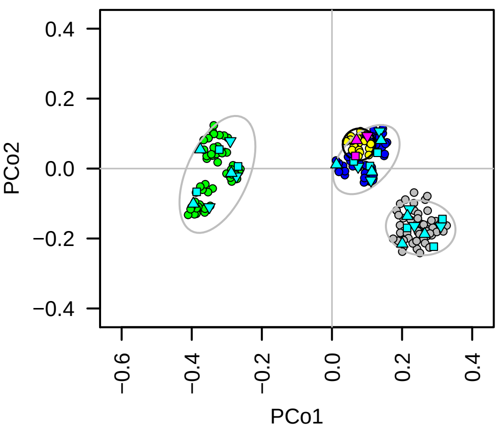

S468 (m/z=667.499311; rt=12.90962)
T/S Cluster: S−12.9−6

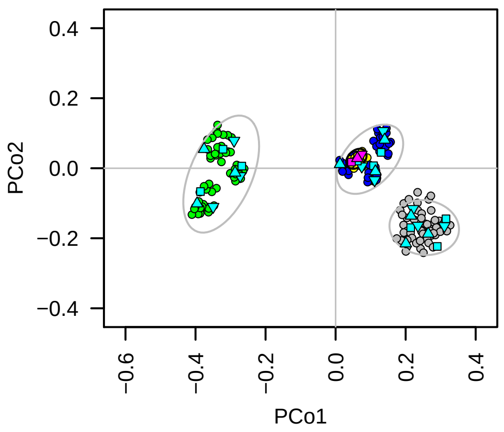

S469 (m/z=326.232949; rt=12.90972)
T/S Cluster: S−12.9−7

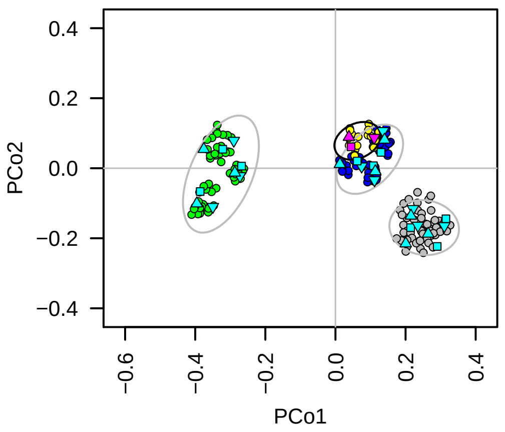

S470 (m/z=325.237934; rt=12.90975)
T/S Cluster: S−12.9−8

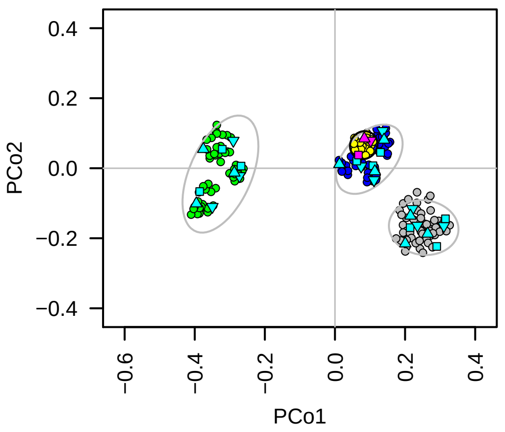

S471 (m/z=162.619305; rt=12.90997)
T/S Cluster: S−12.9−8

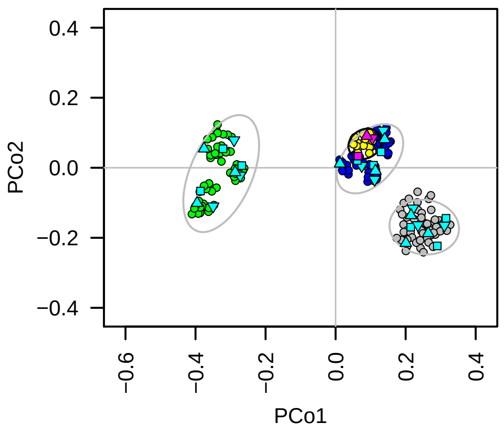

S472 (m/z=326.241208; rt=12.91008)
T/S Cluster: S−12.9−9

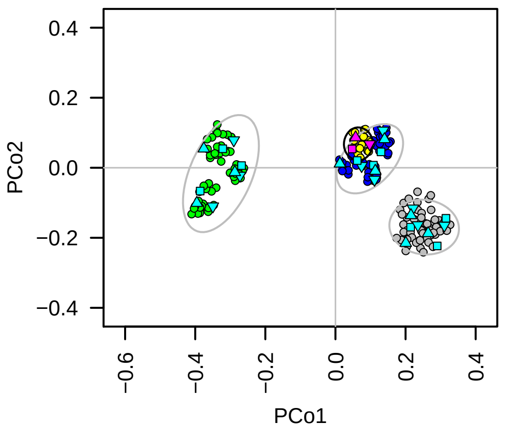

S473 (m/z=671.451641; rt=12.9109)
T/S Cluster: S−12.9−10

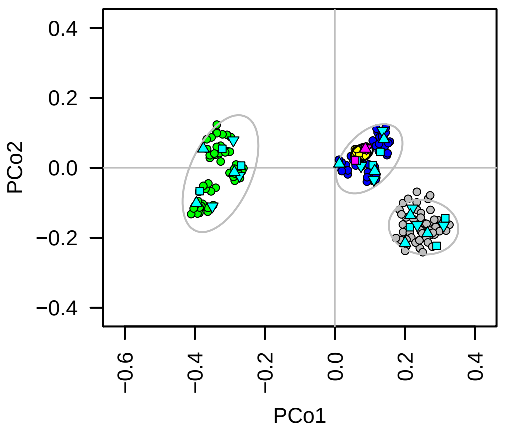

S474 (m/z=672.45503; rt=12.91091)
T/S Cluster: S−12.9−10

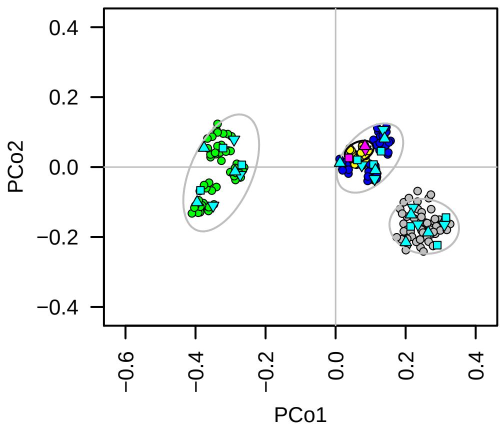

S476 (m/z=335.726842; rt=12.91097)
T/S Cluster: S−12.9−10

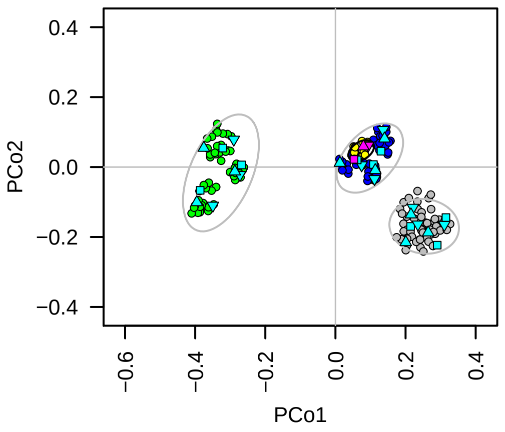

S478 (m/z=672.414907; rt=12.91116)
T/S Cluster: S−12.9−10

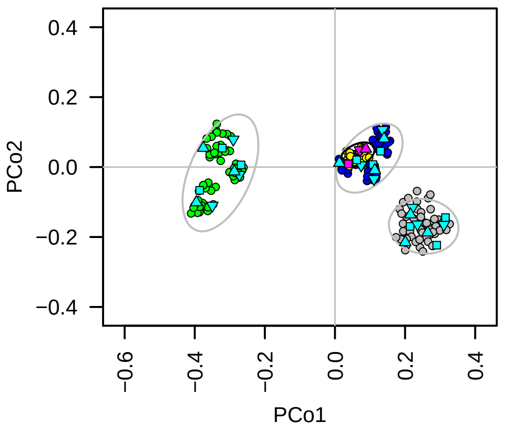

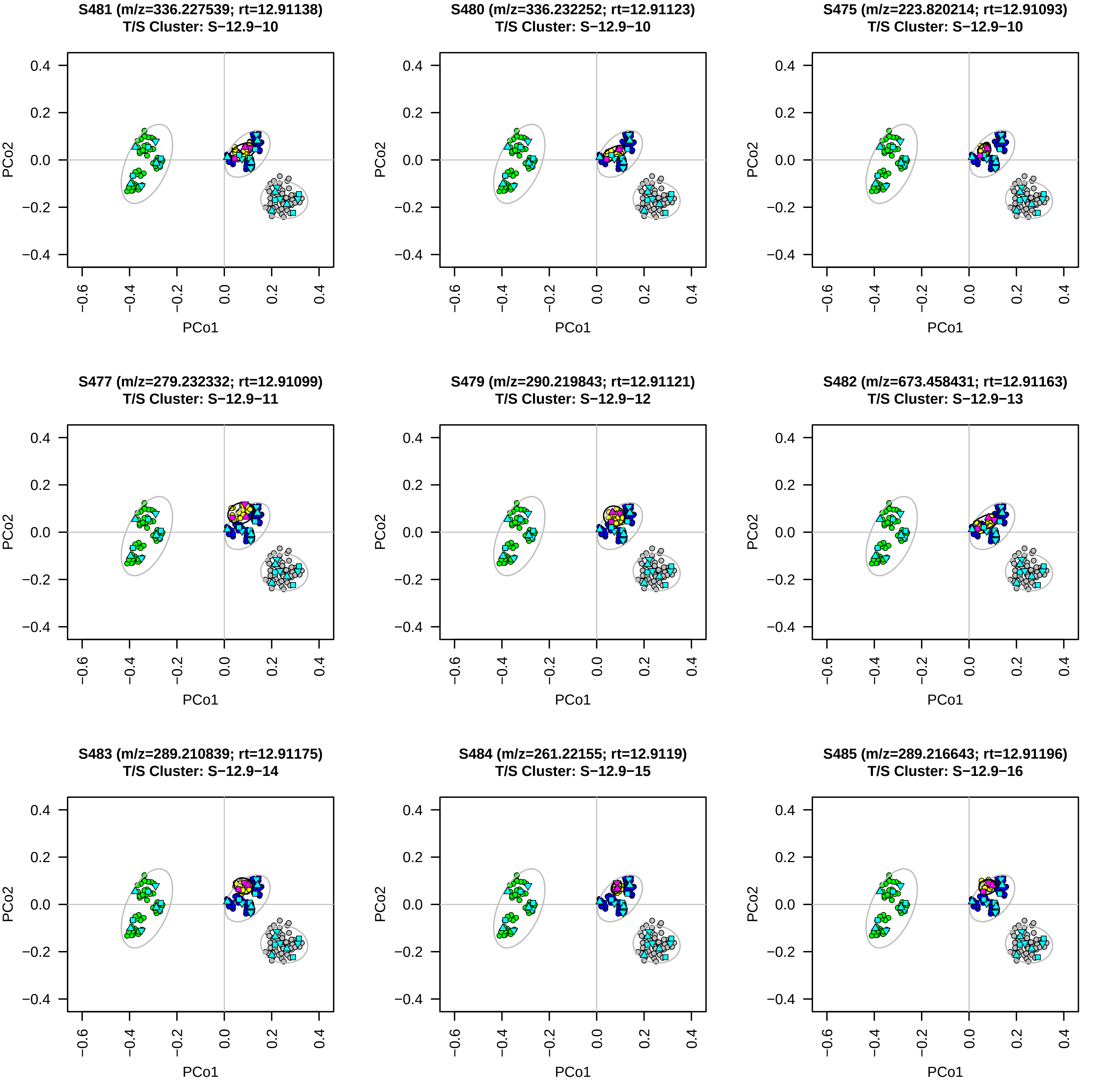

S481 (m/z=336.227539; rt=12.91138)
T/S Cluster: S−12.9−10
S480 (m/z=336.232252; rt=12.91123)
T/S Cluster: S−12.9−10
S475 (m/z=223.820214; rt=12.91093)
T/S Cluster: S−12.9−10
S477 (m/z=279.232332; rt=12.91099)
T/S Cluster: S−12.9−11
S479 (m/z=290.219843; rt=12.91121)
T/S Cluster: S−12.9−12
S482 (m/z=673.458431; rt=12.91163)
T/S Cluster: S−12.9−13
S483 (m/z=289.210839; rt=12.91175)
T/S Cluster: S−12.9−14
S484 (m/z=261.22155; rt=12.9119)
T/S Cluster: S−12.9−15
S485 (m/z=289.216643; rt=12.91196)
T/S Cluster: S−12.9−16
PCo1
PCo2
−0.6
−0.4
−0.2
0.0
0.2
0.4

S486 (m/z=363.19398; rt=12.91223)
T/S Cluster: S−12.9−17

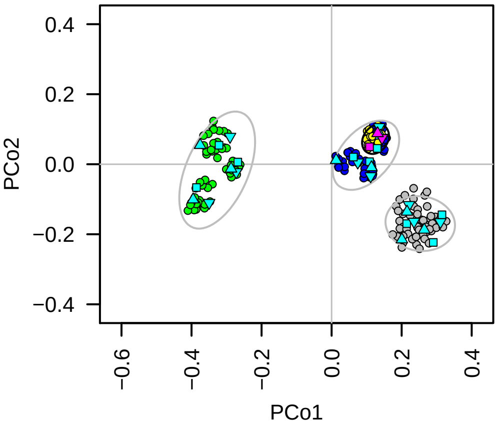

S487 (m/z=342.264794; rt=12.91227)
T/S Cluster: S−12.9−18

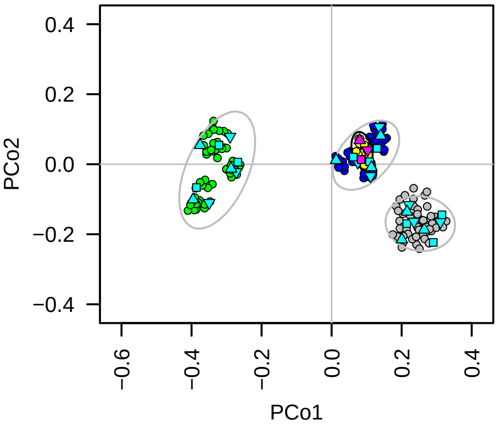

S490 (m/z=308.230632; rt=12.91259)
T/S Cluster: S−12.9−19

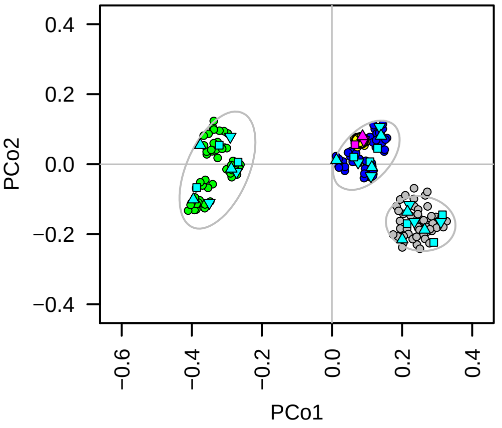

S488 (m/z=308.223684; rt=12.91229)
T/S Cluster: S−12.9−19

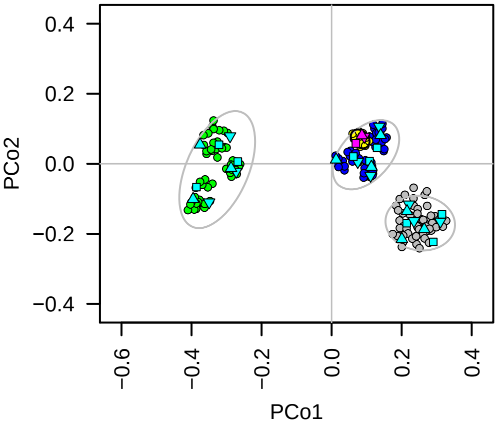

S498 (m/z=384.286632; rt=12.91298)
T/S Cluster: S−12.9−20

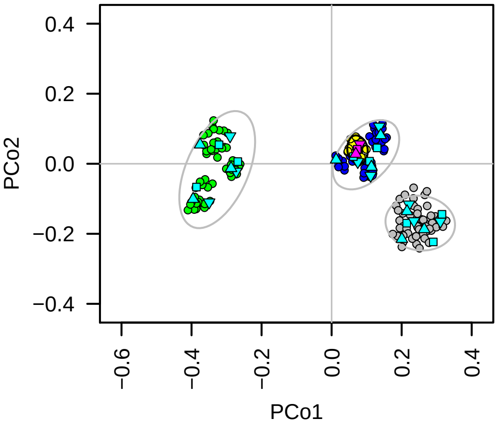

S489 (m/z=384.278791; rt=12.91247)
T/S Cluster: S−12.9−20

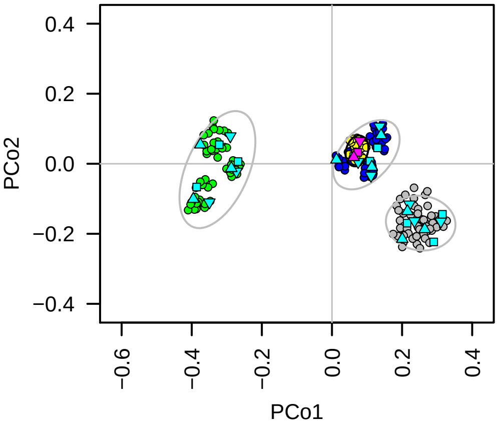

S495 (m/z=307.2272; rt=12.91274)
T/S Cluster: S−12.9−21

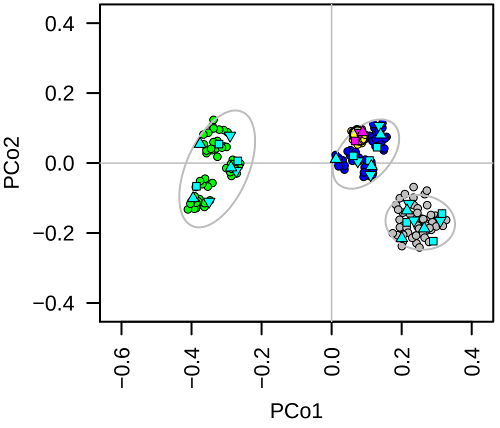

S491 (m/z=307.21851; rt=12.91265)
T/S Cluster: S−12.9−21

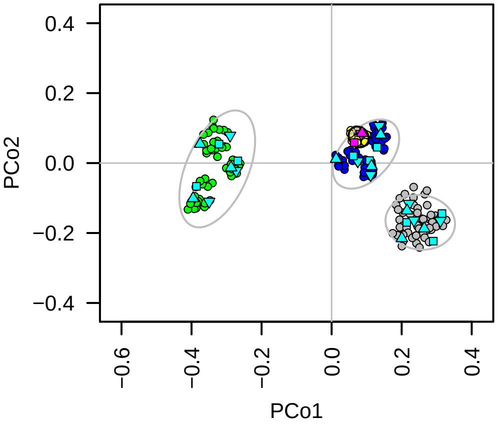

S492 (m/z=153.613887; rt=12.91265)
T/S Cluster: S−12.9−22

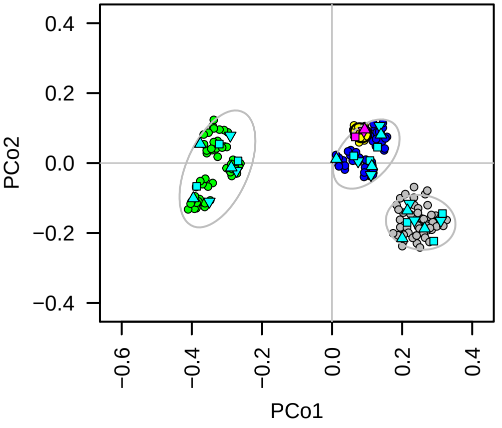

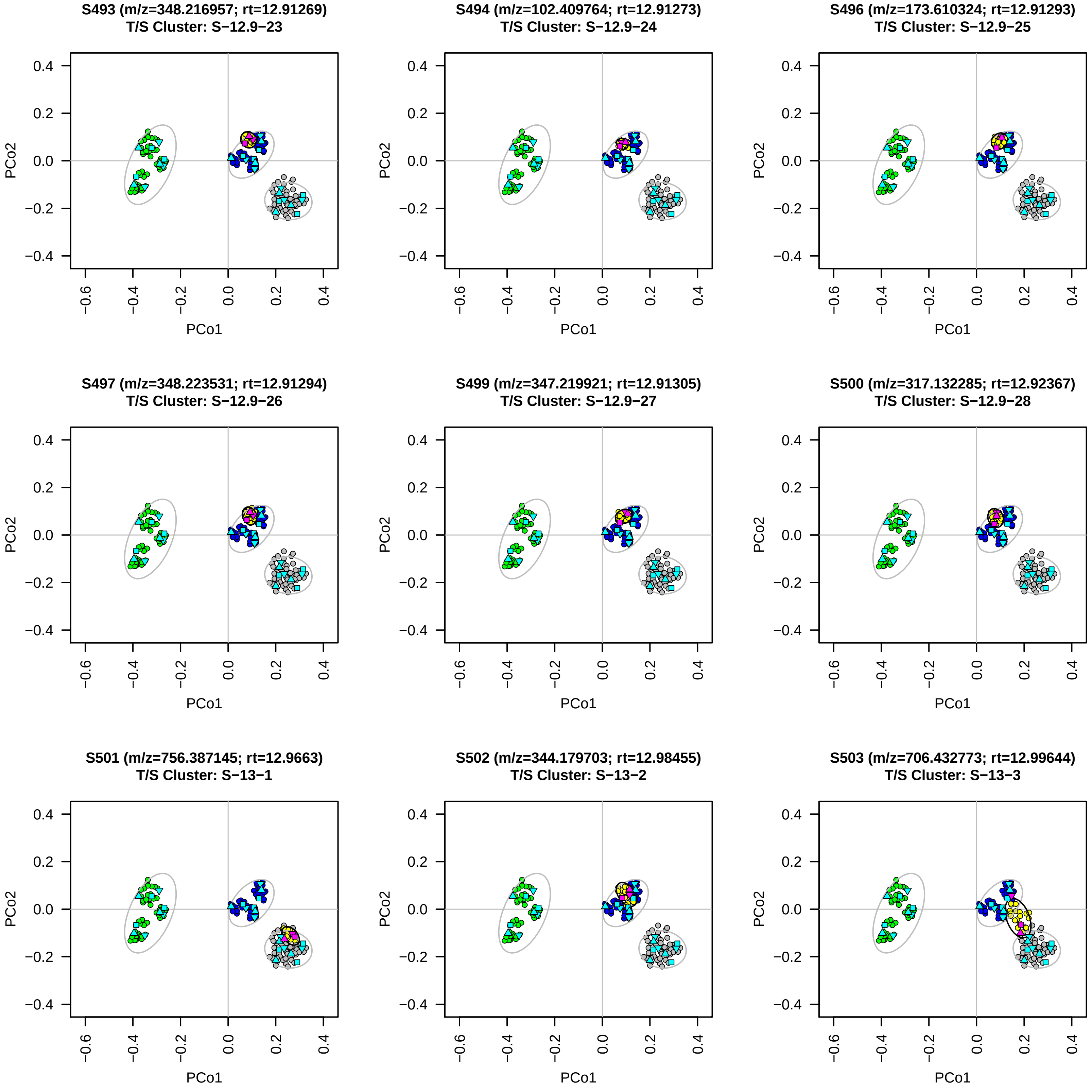
S493 (m/z=348.216957; rt=12.91269)
T/S Cluster: S−12.9−23
S494 (m/z=102.409764; rt=12.91273)
T/S Cluster: S−12.9−24
S496 (m/z=173.610324; rt=12.91293)
T/S Cluster: S−12.9−25
S497 (m/z=348.223531; rt=12.91294)
T/S Cluster: S−12.9−26
S499 (m/z=347.219921; rt=12.91305)
T/S Cluster: S−12.9−27
S500 (m/z=317.132285; rt=12.92367)
T/S Cluster: S−12.9−28
S501 (m/z=756.387145; rt=12.9663)
T/S Cluster: S−13−1
S502 (m/z=344.179703; rt=12.98455)
T/S Cluster: S−13−2
S503 (m/z=706.432773; rt=12.99644)
T/S Cluster: S−13−3
PCo1
PCo2
−0.6
−0.4
−0.2
0.0
0.2
0.4

S504 (m/z=706.43283; rt=12.99644)
T/S Cluster: S−13−3

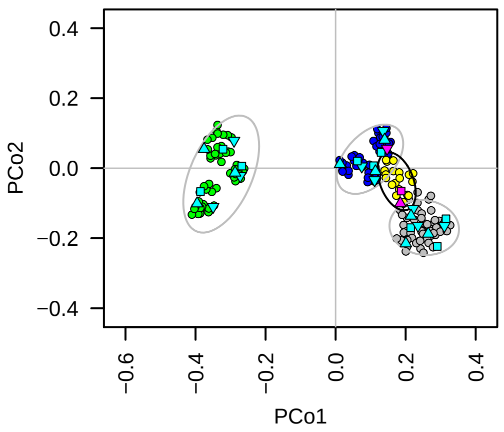

S505 (m/z=334.275069; rt=12.999)
T/S Cluster: S−13−4

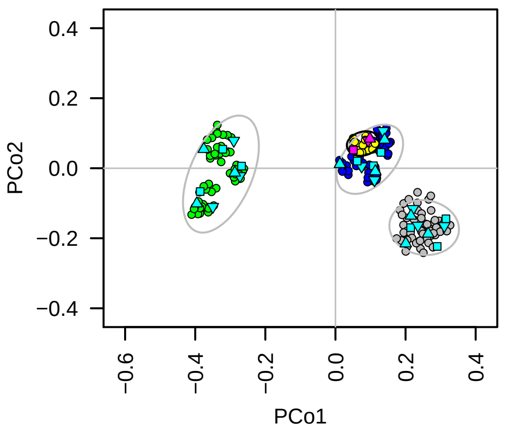

S506 (m/z=880.493309; rt=13.01226)
T/S Cluster: S−13−5

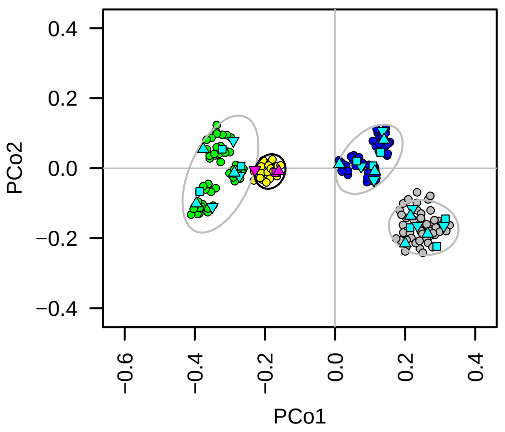

S507 (m/z=880.526406; rt=13.01741)
T/S Cluster: S−13−5

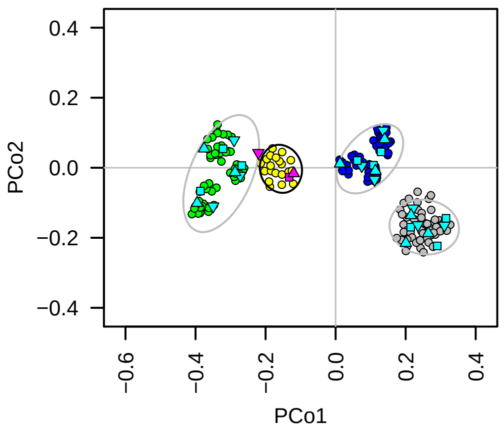

S508 (m/z=366.300967; rt=13.02353)
T/S Cluster: S−13−6

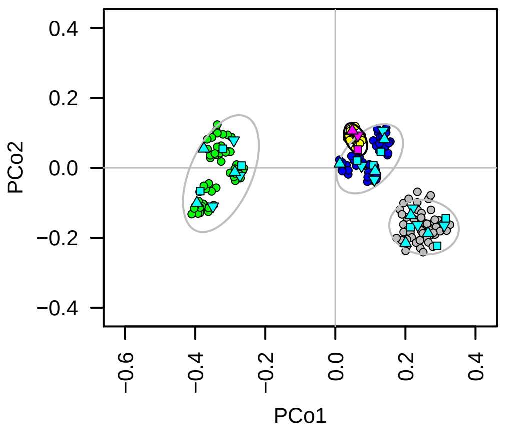

S509 (m/z=307.227147; rt=13.05273)
T/S Cluster: S−13.1−1

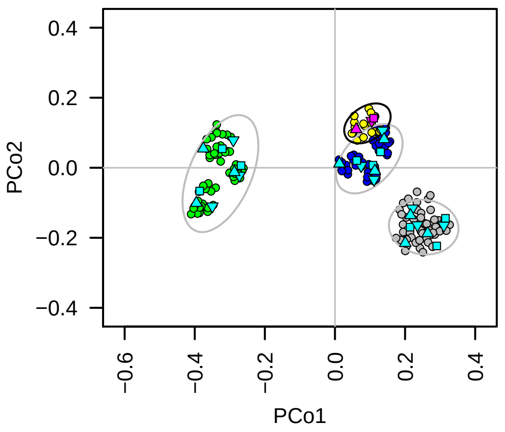

S510 (m/z=325.237852; rt=13.05783)
T/S Cluster: S−13.1−2

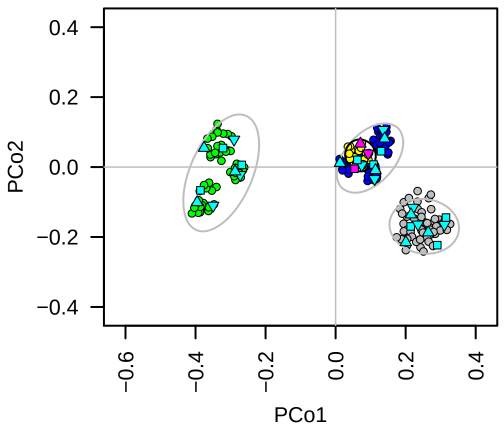

S511 (m/z=736.405106; rt=13.07698)
T/S Cluster: S−13.1−3

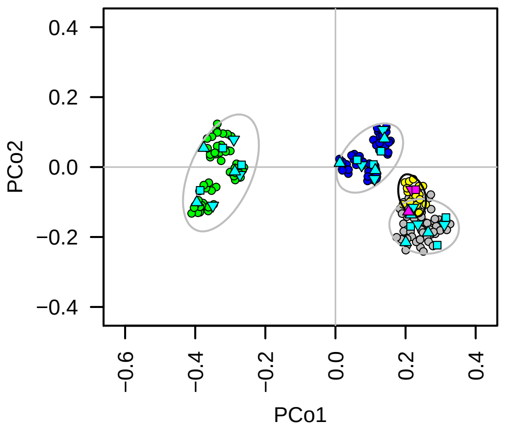

S512 (m/z=424.198663; rt=13.10018)
T/S Cluster: S−13.1−4

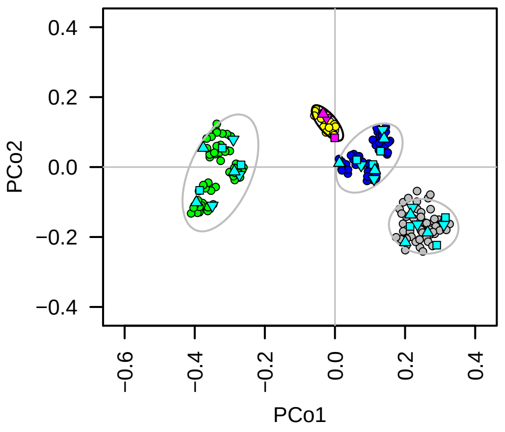

S513 (m/z=400.234576; rt=13.11391)
T/S Cluster: S−13.1−5

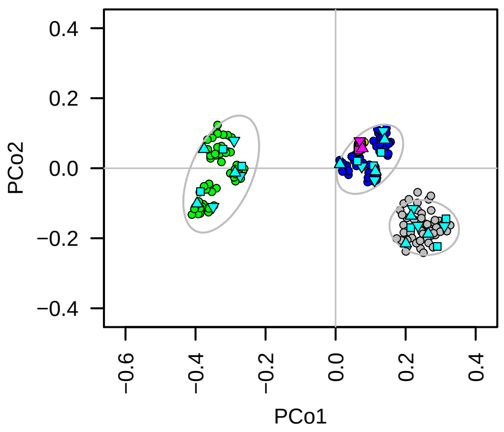

S514 (m/z=421.25703; rt=13.13309)
T/S Cluster: S−13.1−6

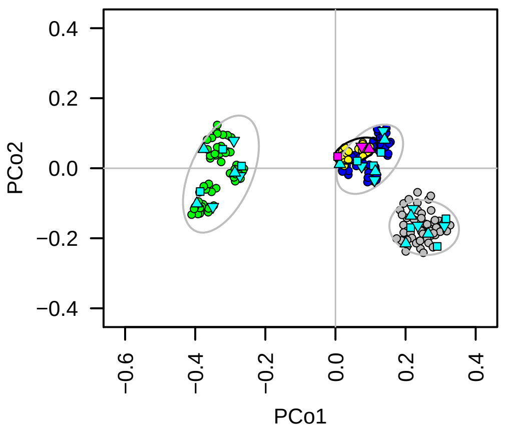

S515 (m/z=734.494082; rt=13.1805)
T/S Cluster: S−13.2−1

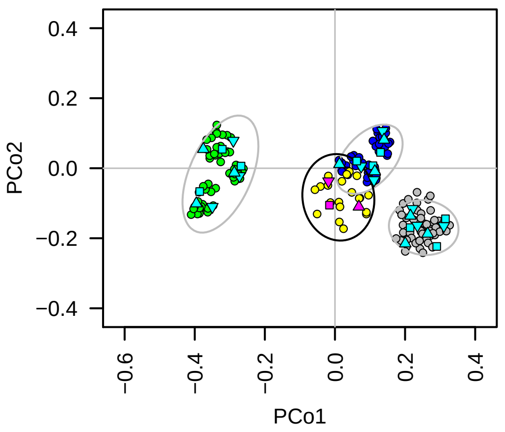

S516 (m/z=437.744462; rt=13.20008)
T/S Cluster: S−13.2−2

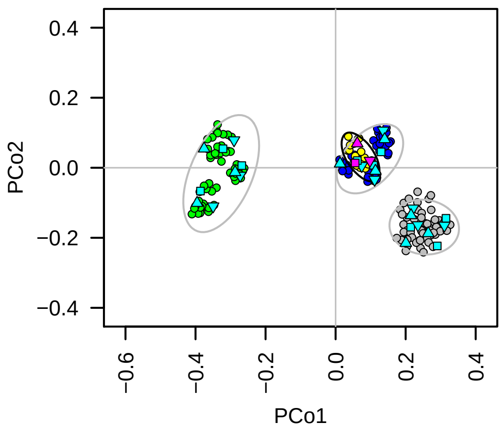

S517 (m/z=437.802958; rt=13.20877)
T/S Cluster: S−13.2−3

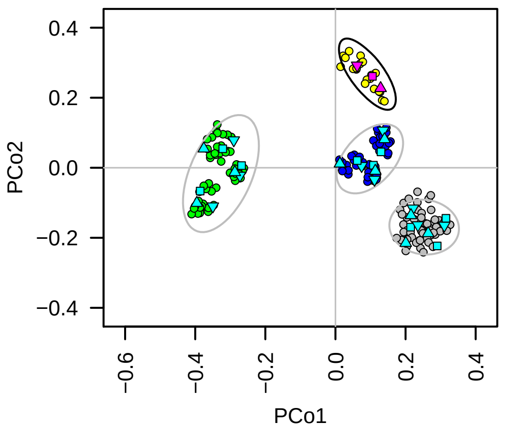

S520 (m/z=299.050765; rt=13.23219)
T/S Cluster: S−13.2−4

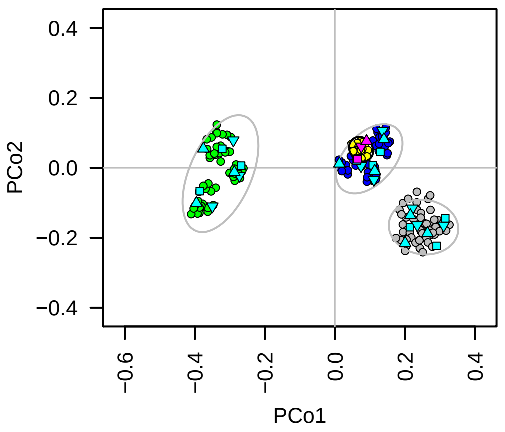

S518 (m/z=299.045914; rt=13.23136)
T/S Cluster: S−13.2−4

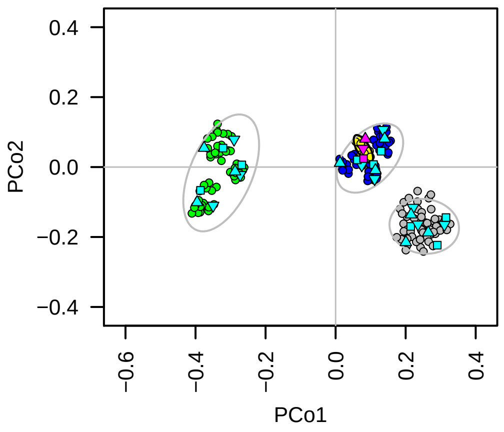

S519 (m/z=300.042948; rt=13.23151)
T/S Cluster: S−13.2−5

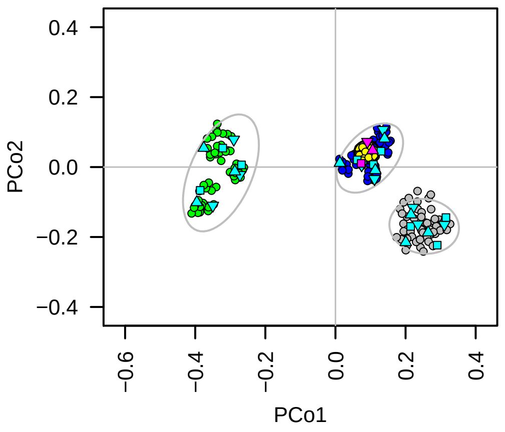

S523 (m/z=298.047281; rt=13.23269)
T/S Cluster: S−13.2−6

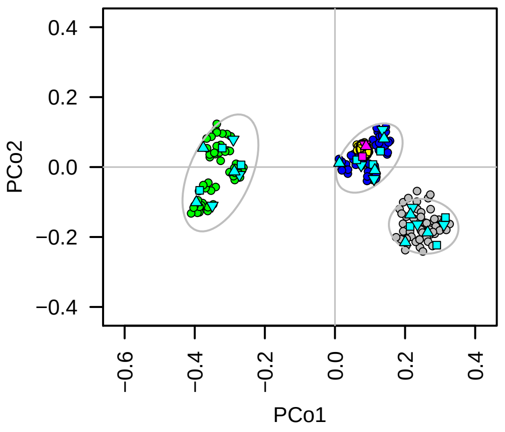

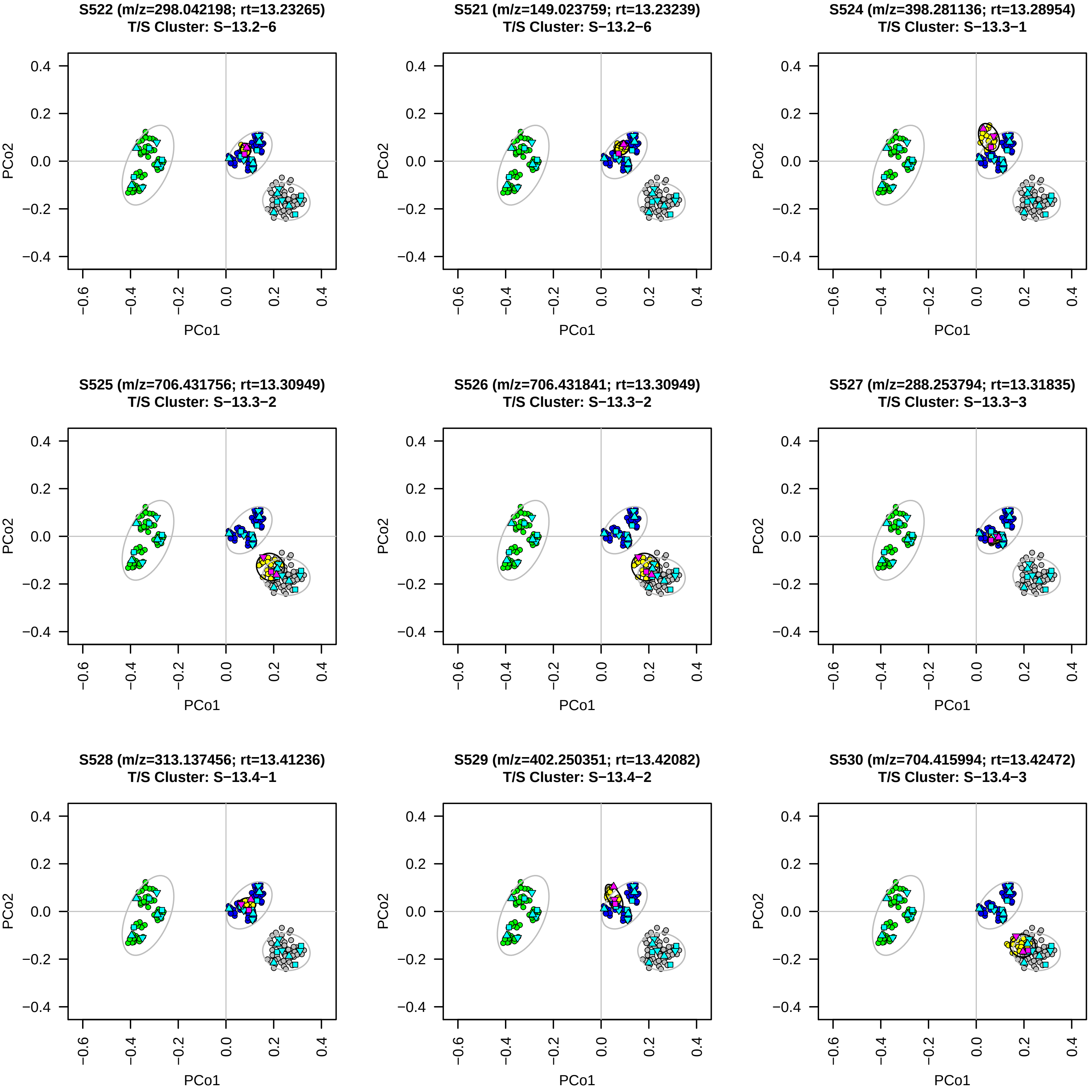
S522 (m/z=298.042198; rt=13.23265)
T/S Cluster: S−13.2−6
S521 (m/z=149.023759; rt=13.23239)
T/S Cluster: S−13.2−6
S524 (m/z=398.281136; rt=13.28954)
T/S Cluster: S−13.3−1
S525 (m/z=706.431756; rt=13.30949)
T/S Cluster: S−13.3−2
S526 (m/z=706.431841; rt=13.30949)
T/S Cluster: S−13.3−2
S527 (m/z=288.253794; rt=13.31835)
T/S Cluster: S−13.3−3
S528 (m/z=313.137456; rt=13.41236)
T/S Cluster: S−13.4−1
S529 (m/z=402.250351; rt=13.42082)
T/S Cluster: S−13.4−2
S530 (m/z=704.415994; rt=13.42472)
T/S Cluster: S−13.4−3
PCo1
PCo2
0.4
0.2
0.0
−0.2
−0.4
−0.6

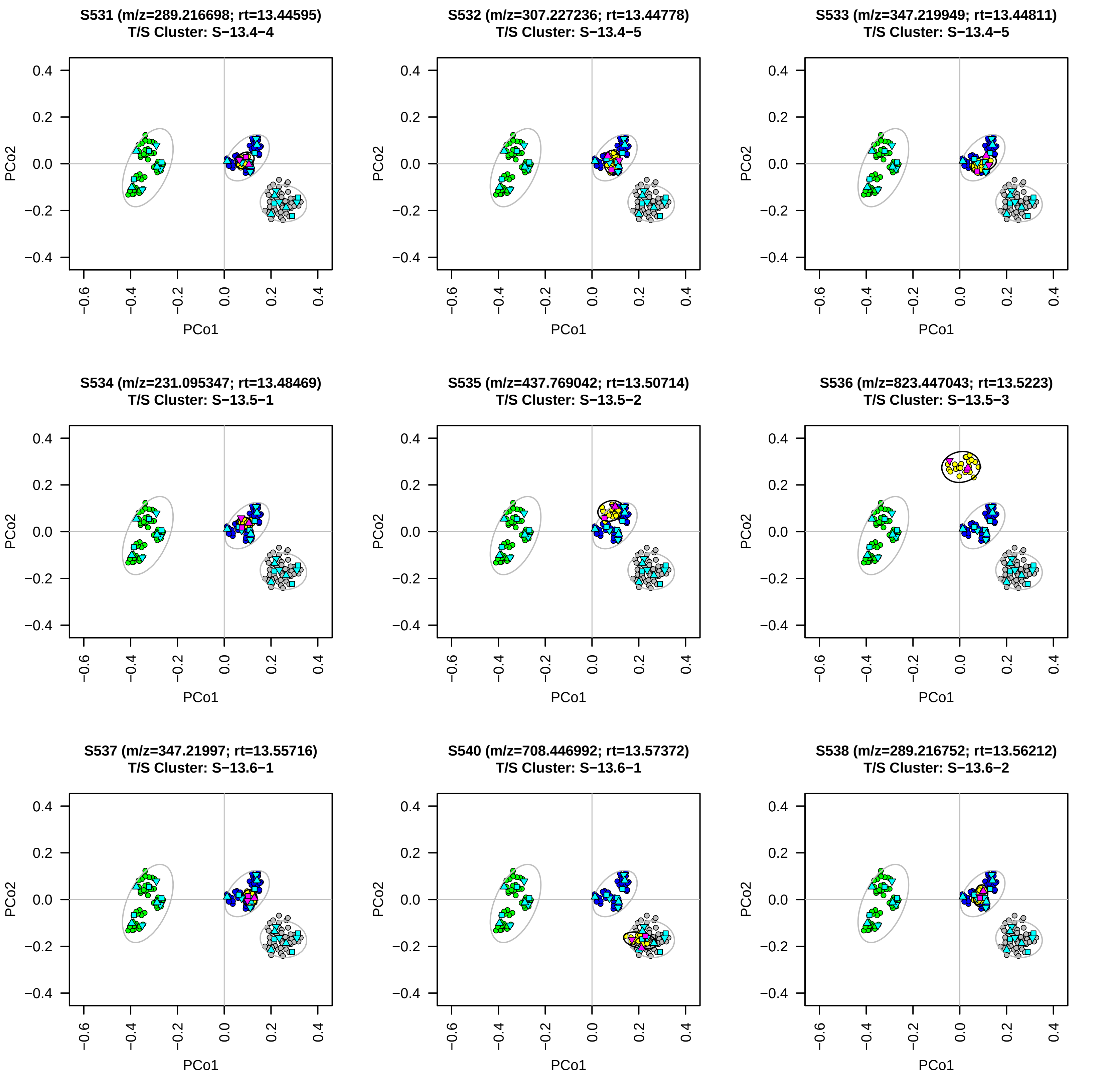
S531 (m/z=289.216698; rt=13.44595)
T/S Cluster: S−13.4−4
S532 (m/z=307.227236; rt=13.44778)
T/S Cluster: S−13.4−5
S533 (m/z=347.219949; rt=13.44811)
T/S Cluster: S−13.4−5
S534 (m/z=231.095347; rt=13.48469)
T/S Cluster: S−13.5−1
S535 (m/z=437.769042; rt=13.50714)
T/S Cluster: S−13.5−2
S536 (m/z=823.447043; rt=13.5223)
T/S Cluster: S−13.5−3
S537 (m/z=347.21997; rt=13.55716)
T/S Cluster: S−13.6−1
S540 (m/z=708.446992; rt=13.57372)
T/S Cluster: S−13.6−1
S538 (m/z=289.216752; rt=13.56212)
T/S Cluster: S−13.6−2
PCo1
PCo2
−0.6
−0.4
−0.2
0.0
0.2
0.4

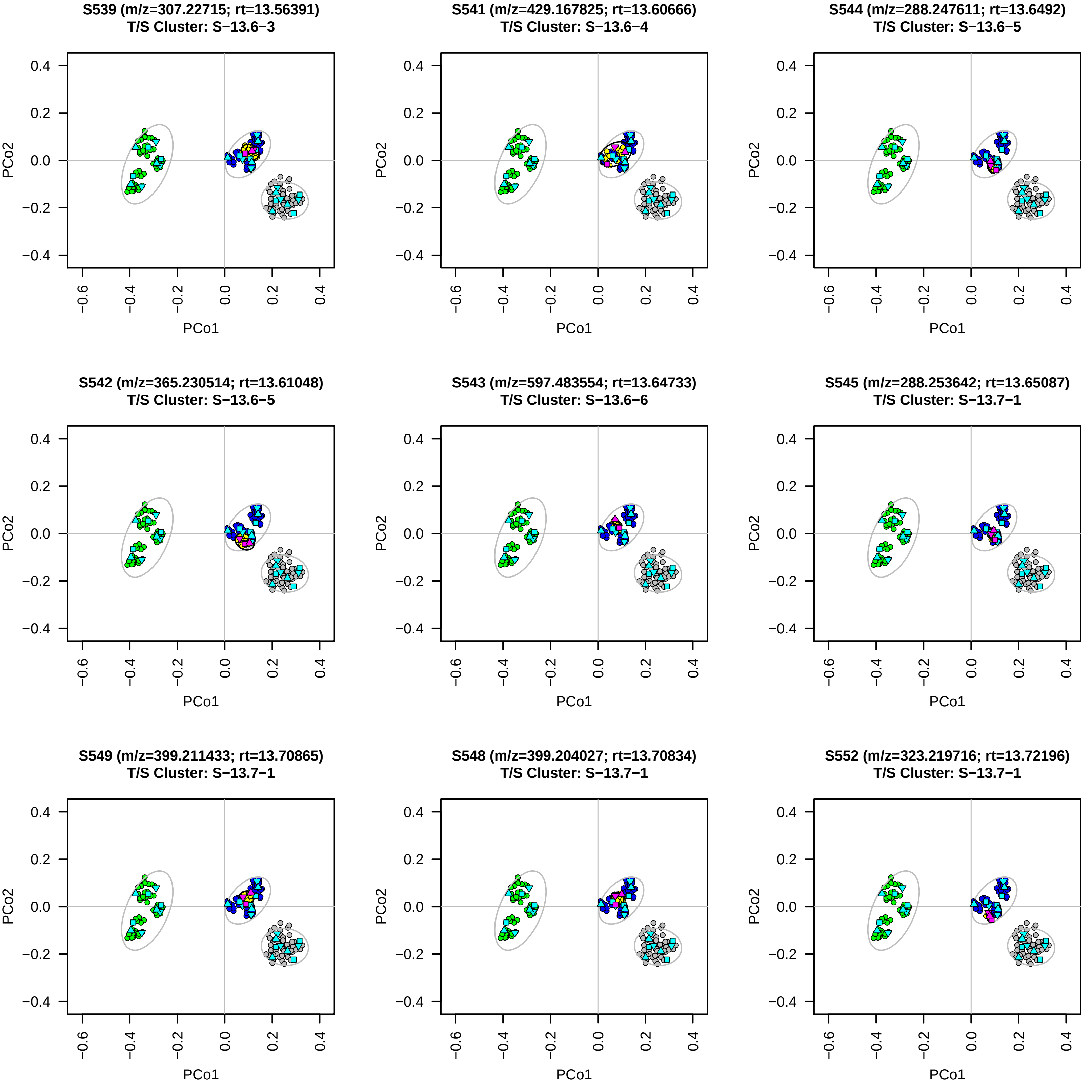
S539 (m/z=307.22715; rt=13.56391)
T/S Cluster: S−13.6−3
S541 (m/z=429.167825; rt=13.60666)
T/S Cluster: S−13.6−4
S544 (m/z=288.247611; rt=13.6492)
T/S Cluster: S−13.6−5
S542 (m/z=365.230514; rt=13.61048)
T/S Cluster: S−13.6−5
S543 (m/z=597.483554; rt=13.64733)
T/S Cluster: S−13.6−6
S545 (m/z=288.253642; rt=13.65087)
T/S Cluster: S−13.7−1
S549 (m/z=399.211433; rt=13.70865)
T/S Cluster: S−13.7−1
S548 (m/z=399.204027; rt=13.70834)
T/S Cluster: S−13.7−1
S552 (m/z=323.219716; rt=13.72196)
T/S Cluster: S−13.7−1
PCo1
PCo2
−0.6
−0.4
−0.2
0.0
0.2
0.4

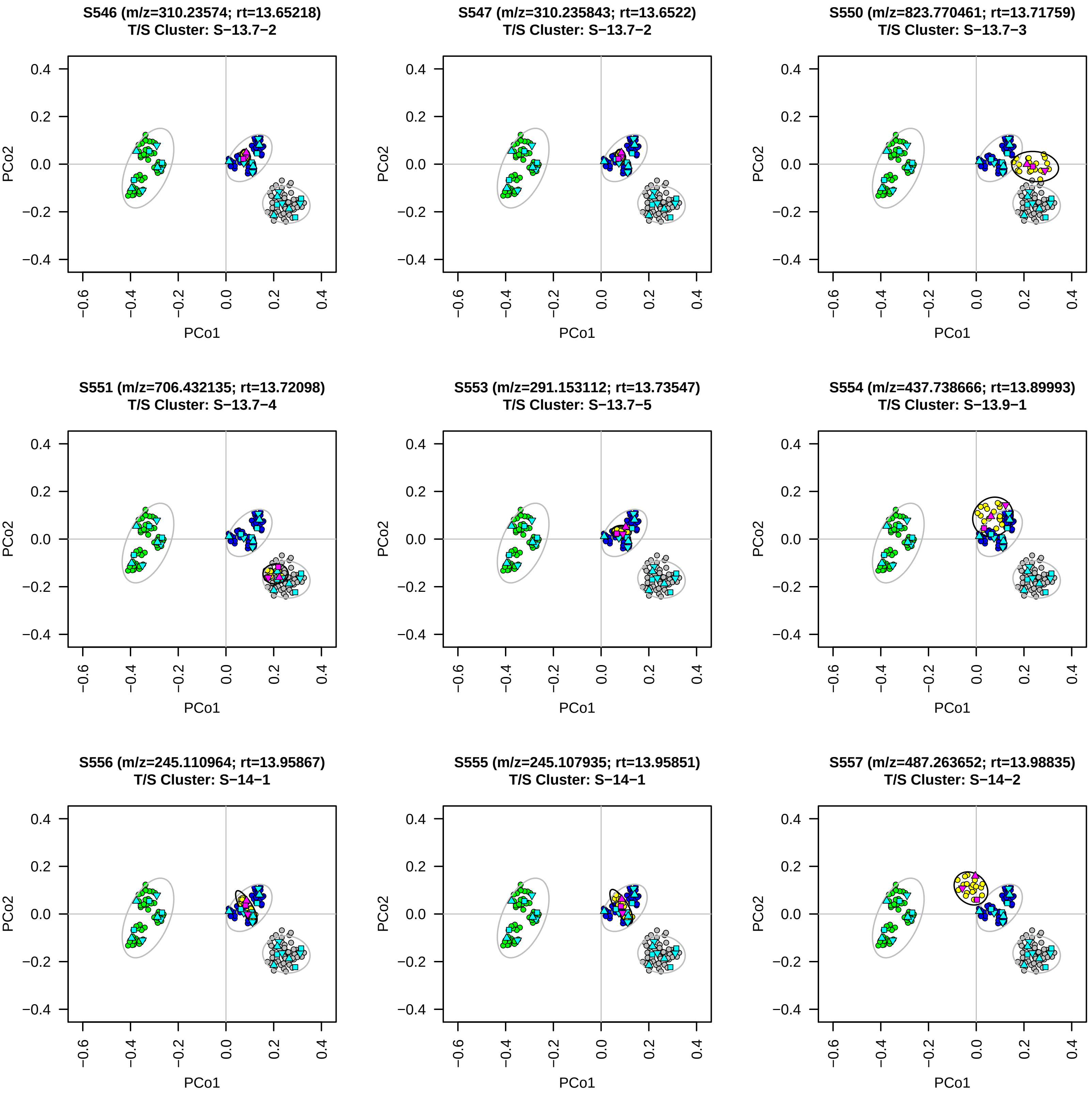
S546 (m/z=310.23574; rt=13.65218)
T/S Cluster: S−13.7−2
S547 (m/z=310.235843; rt=13.6522)
T/S Cluster: S−13.7−2
S550 (m/z=823.770461; rt=13.71759)
T/S Cluster: S−13.7−3
S551 (m/z=706.432135; rt=13.72098)
T/S Cluster: S−13.7−4
S553 (m/z=291.153112; rt=13.73547)
T/S Cluster: S−13.7−5
S554 (m/z=437.738666; rt=13.89993)
T/S Cluster: S−13.9−1
S556 (m/z=245.110964; rt=13.95867)
T/S Cluster: S−14−1
S555 (m/z=245.107935; rt=13.95851)
T/S Cluster: S−14−1
S557 (m/z=487.263652; rt=13.98835)
T/S Cluster: S−14−2
PCo1
PCo2
0.4
0.2
0.0
−0.2
−0.4
−0.6

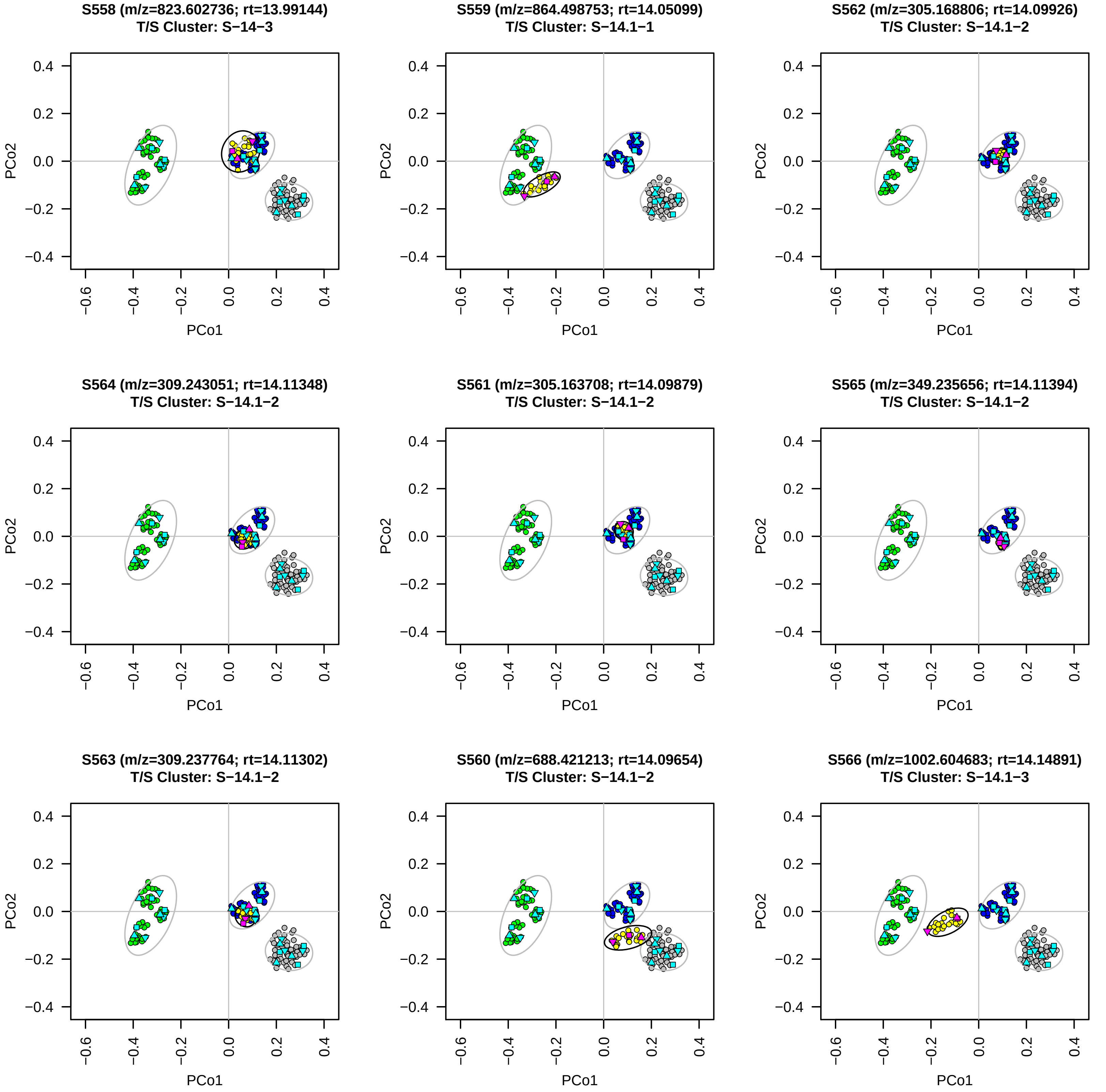
S558 (m/z=823.602736; rt=13.99144)
T/S Cluster: S−14−3
S559 (m/z=864.498753; rt=14.05099)
T/S Cluster: S−14.1−1
S562 (m/z=305.168806; rt=14.09926)
T/S Cluster: S−14.1−2
S564 (m/z=309.243051; rt=14.11348)
T/S Cluster: S−14.1−2
S561 (m/z=305.163708; rt=14.09879)
T/S Cluster: S−14.1−2
S565 (m/z=349.235656; rt=14.11394)
T/S Cluster: S−14.1−2
S563 (m/z=309.237764; rt=14.11302)
T/S Cluster: S−14.1−2
S560 (m/z=688.421213; rt=14.09654)
T/S Cluster: S−14.1−2
S566 (m/z=1002.604683; rt=14.14891)
T/S Cluster: S−14.1−3
PCo1
PCo2
−0.6
−0.4
−0.2
0.0
0.2
0.4

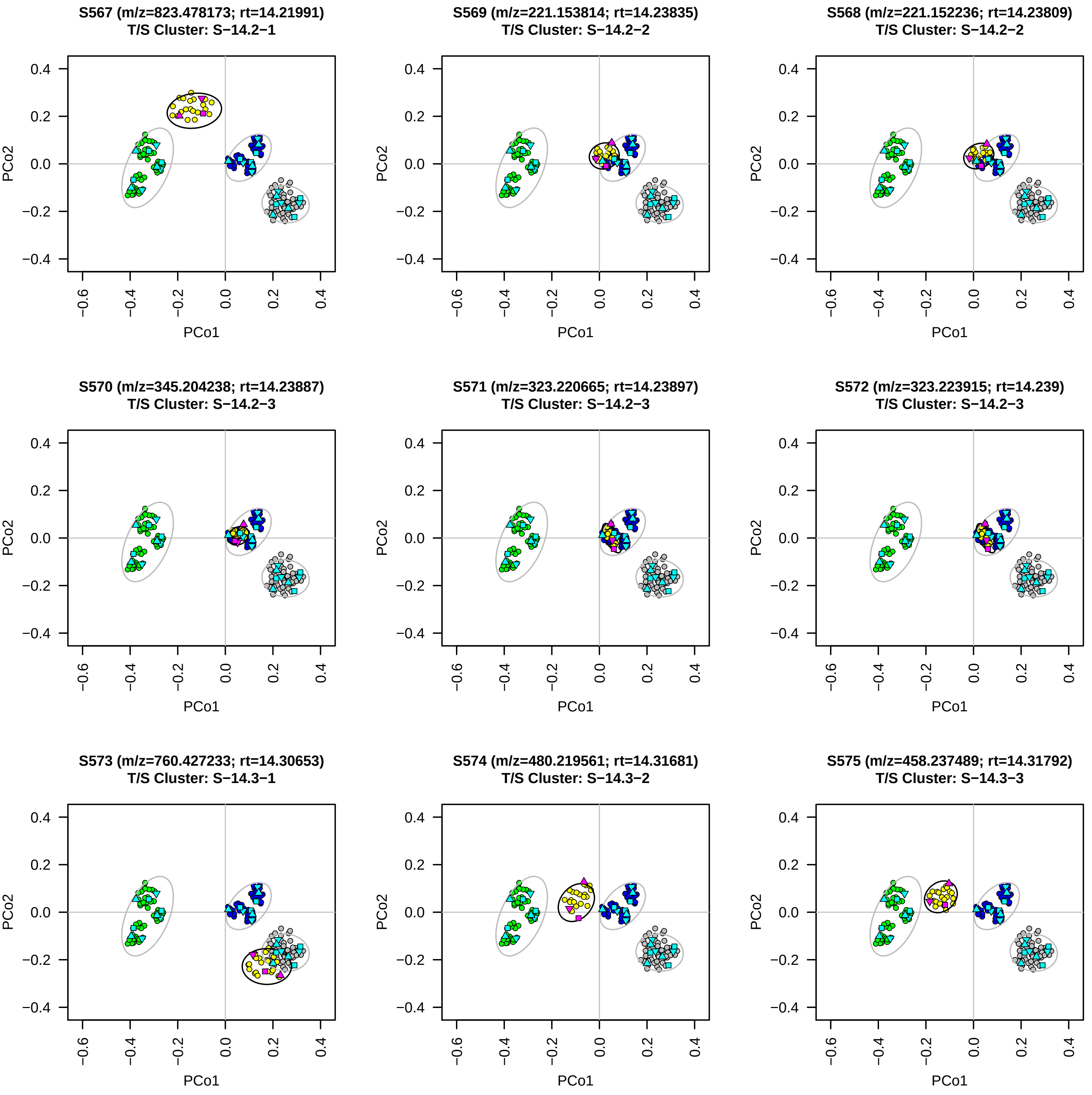
S567 (m/z=823.478173; rt=14.21991)
T/S Cluster: S−14.2−1
S569 (m/z=221.153814; rt=14.23835)
T/S Cluster: S−14.2−2
S568 (m/z=221.152236; rt=14.23809)
T/S Cluster: S−14.2−2
S570 (m/z=345.204238; rt=14.23887)
T/S Cluster: S−14.2−3
S571 (m/z=323.220665; rt=14.23897)
T/S Cluster: S−14.2−3
S572 (m/z=323.223915; rt=14.239)
T/S Cluster: S−14.2−3
S573 (m/z=760.427233; rt=14.30653)
T/S Cluster: S−14.3−1
S574 (m/z=480.219561; rt=14.31681)
T/S Cluster: S−14.3−2
S575 (m/z=458.237489; rt=14.31792)
T/S Cluster: S−14.3−3
PCo1
PCo2
−0.6
−0.4
−0.2
0.0
0.2
0.4

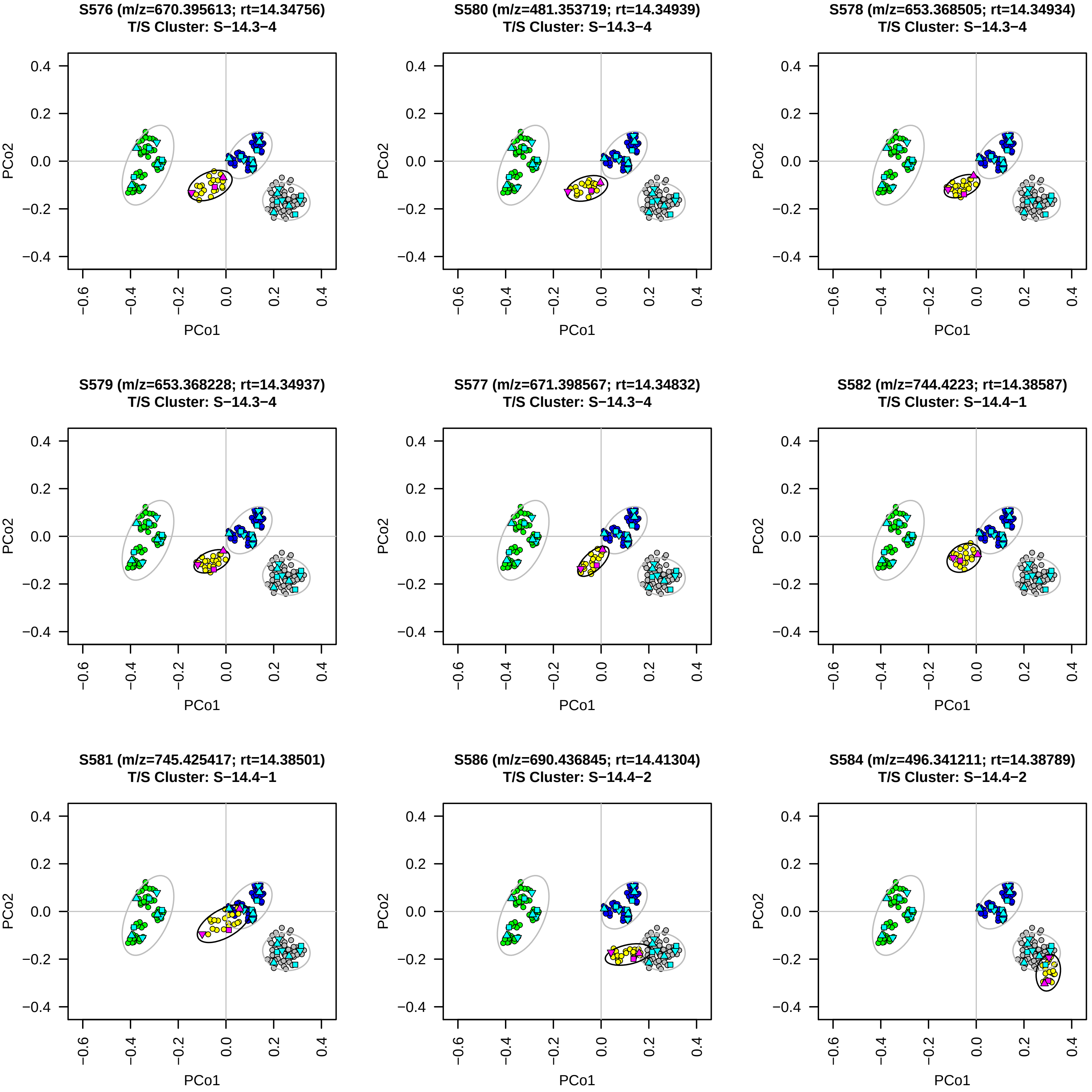
S576 (m/z=670.395613; rt=14.34756)
T/S Cluster: S−14.3−4
S580 (m/z=481.353719; rt=14.34939)
T/S Cluster: S−14.3−4
S578 (m/z=653.368505; rt=14.34934)
T/S Cluster: S−14.3−4
S579 (m/z=653.368228; rt=14.34937)
T/S Cluster: S−14.3−4
S577 (m/z=671.398567; rt=14.34832)
T/S Cluster: S−14.3−4
S582 (m/z=744.4223; rt=14.38587)
T/S Cluster: S−14.4−1
S581 (m/z=745.425417; rt=14.38501)
T/S Cluster: S−14.4−1
S586 (m/z=690.436845; rt=14.41304)
T/S Cluster: S−14.4−2
S584 (m/z=496.341211; rt=14.38789)
T/S Cluster: S−14.4−2
PCo1
PCo2
−0.6
−0.4
−0.2
0.0
0.2
0.4

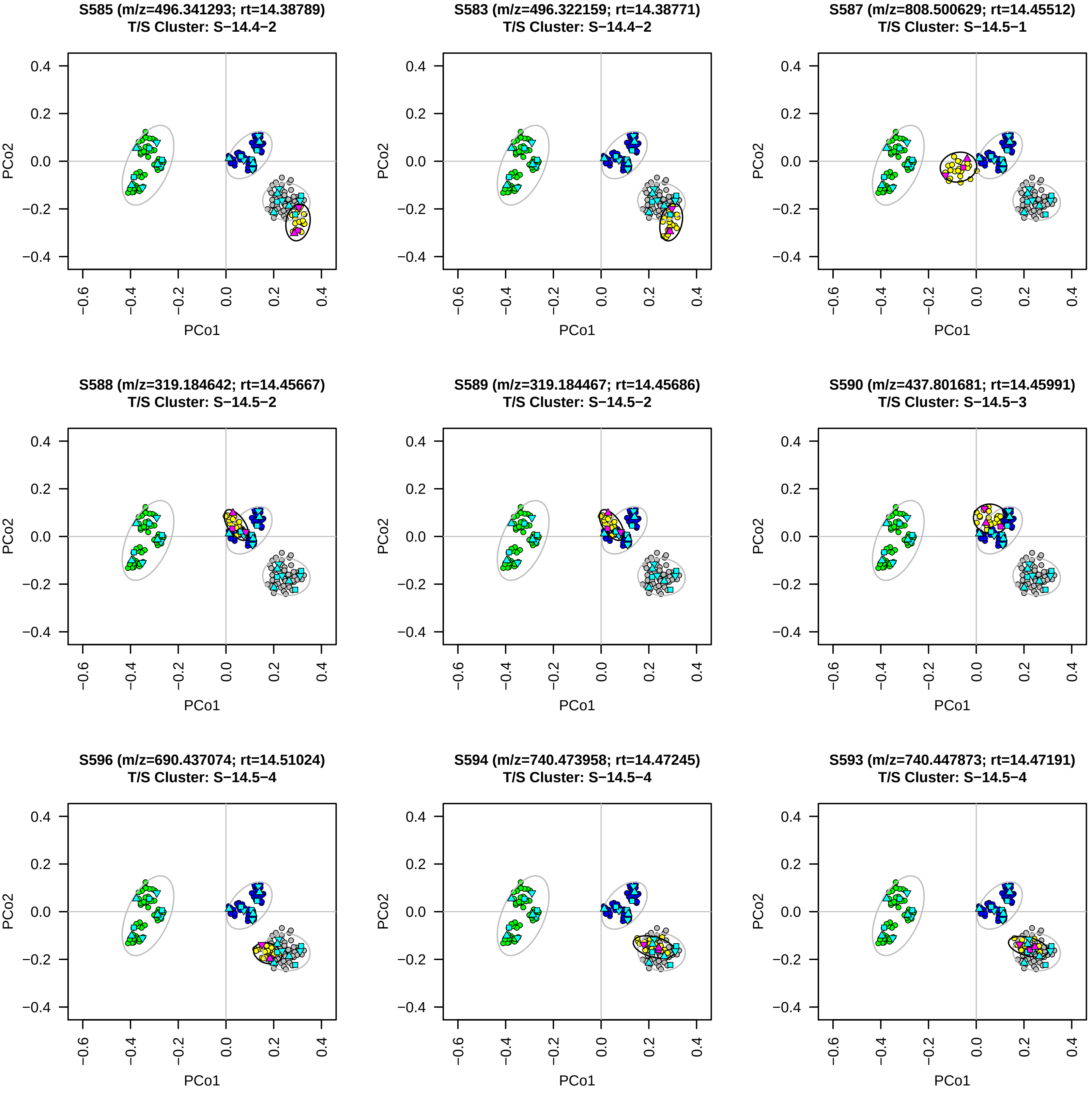
S585 (m/z=496.341293; rt=14.38789)
T/S Cluster: S−14.4−2
S583 (m/z=496.322159; rt=14.38771)
T/S Cluster: S−14.4−2
S587 (m/z=808.500629; rt=14.45512)
T/S Cluster: S−14.5−1
S588 (m/z=319.184642; rt=14.45667)
T/S Cluster: S−14.5−2
S589 (m/z=319.184467; rt=14.45686)
T/S Cluster: S−14.5−2
S590 (m/z=437.801681; rt=14.45991)
T/S Cluster: S−14.5−3
S596 (m/z=690.437074; rt=14.51024)
T/S Cluster: S−14.5−4
S594 (m/z=740.473958; rt=14.47245)
T/S Cluster: S−14.5−4
S593 (m/z=740.447873; rt=14.47191)
T/S Cluster: S−14.5−4
PCo1
PCo2
−0.6
−0.4
−0.2
0.0
0.2
0.4

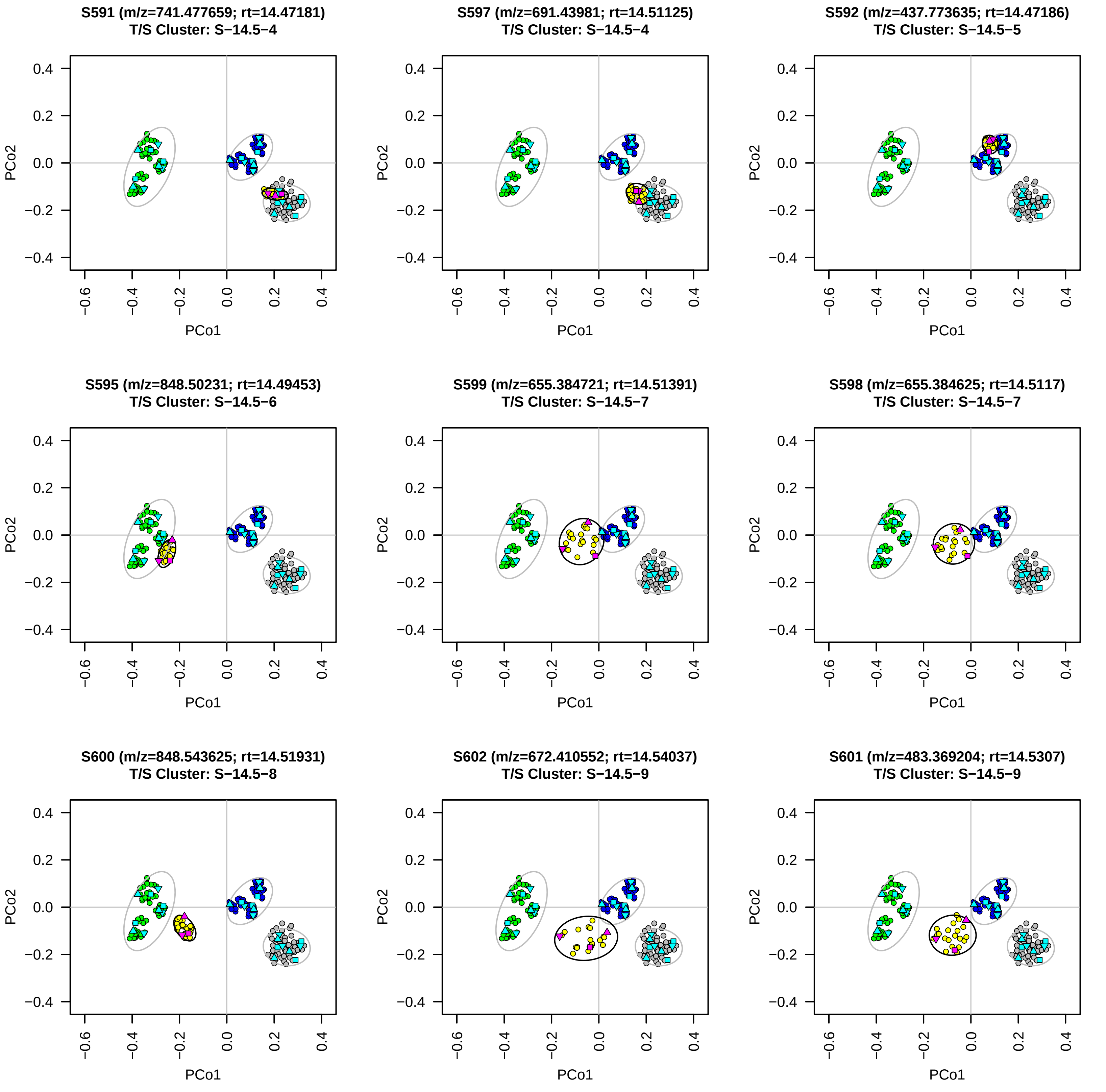
S591 (m/z=741.477659; rt=14.47181)
T/S Cluster: S−14.5−4
S597 (m/z=691.43981; rt=14.51125)
T/S Cluster: S−14.5−4
S592 (m/z=437.773635; rt=14.47186)
T/S Cluster: S−14.5−5
S595 (m/z=848.50231; rt=14.49453)
T/S Cluster: S−14.5−6
S599 (m/z=655.384721; rt=14.51391)
T/S Cluster: S−14.5−7
S598 (m/z=655.384625; rt=14.5117)
T/S Cluster: S−14.5−7
S600 (m/z=848.543625; rt=14.51931)
T/S Cluster: S−14.5−8
S602 (m/z=672.410552; rt=14.54037)
T/S Cluster: S−14.5−9
S601 (m/z=483.369204; rt=14.5307)
T/S Cluster: S−14.5−9
PCo1
PCo2
0.4
0.2
0.0
−0.2
−0.4
−0.6

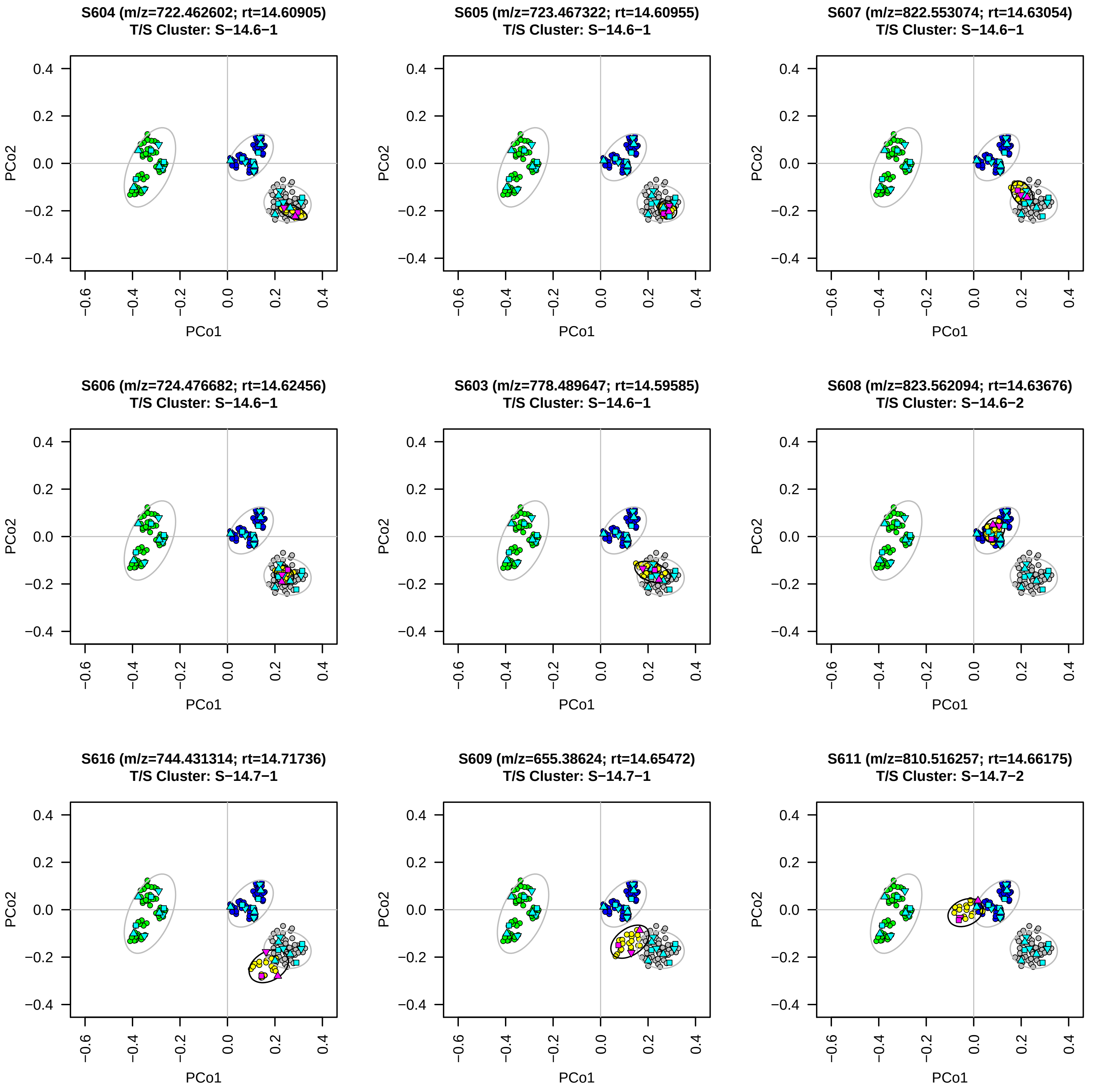

S604 (m/z=722.462602; rt=14.60905)
T/S Cluster: S−14.6−1
S605 (m/z=723.467322; rt=14.60955)
T/S Cluster: S−14.6−1
S607 (m/z=822.553074; rt=14.63054)
T/S Cluster: S−14.6−1
S606 (m/z=724.476682; rt=14.62456)
T/S Cluster: S−14.6−1
S603 (m/z=778.489647; rt=14.59585)
T/S Cluster: S−14.6−1
S608 (m/z=823.562094; rt=14.63676)
T/S Cluster: S−14.6−2
S616 (m/z=744.431314; rt=14.71736)
T/S Cluster: S−14.7−1
S609 (m/z=655.38624; rt=14.65472)
T/S Cluster: S−14.7−1
S611 (m/z=810.516257; rt=14.66175)
T/S Cluster: S−14.7−2
PCo1
PCo2

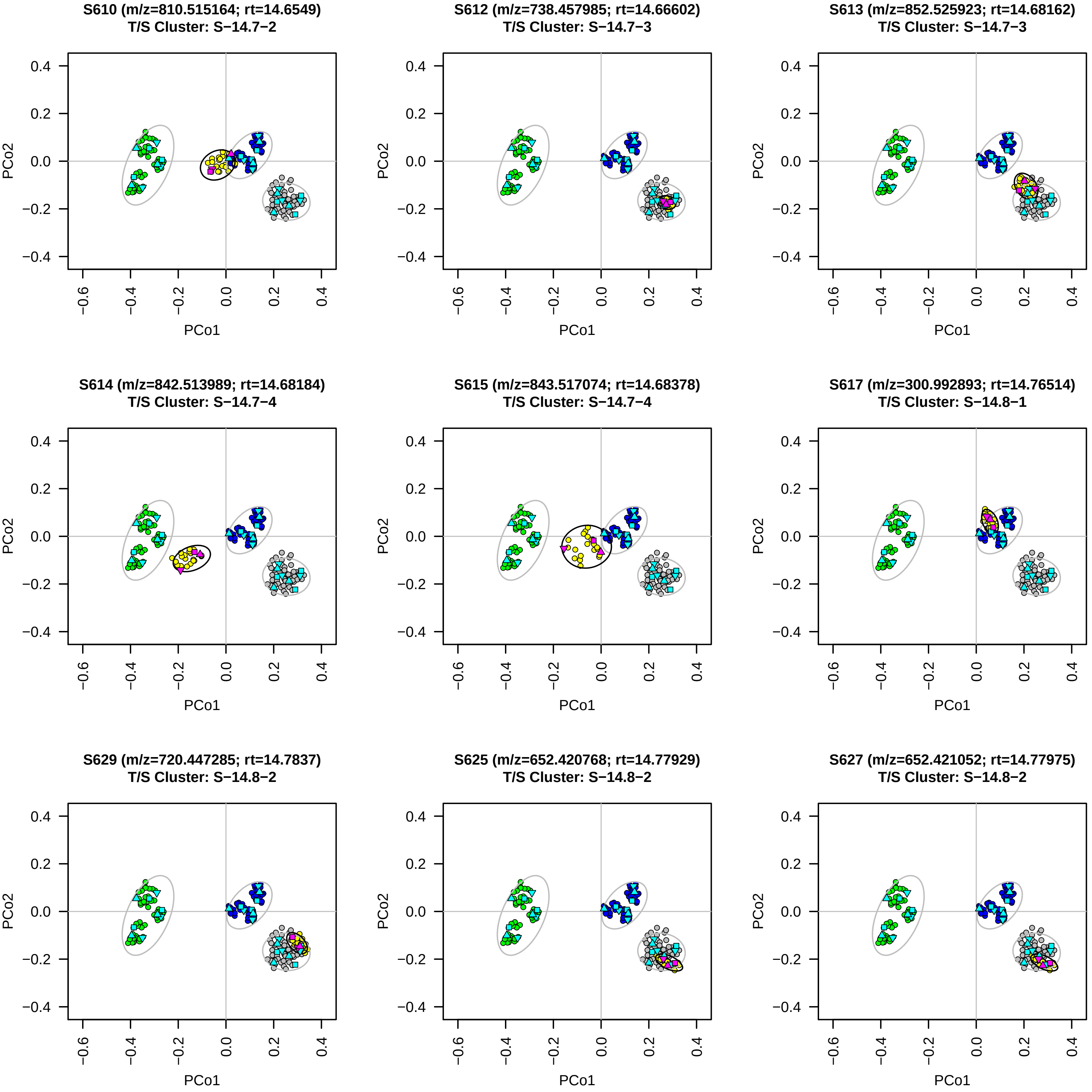
S610 (m/z=810.515164; rt=14.6549)
T/S Cluster: S−14.7−2
S612 (m/z=738.457985; rt=14.66602)
T/S Cluster: S−14.7−3
S613 (m/z=852.525923; rt=14.68162)
T/S Cluster: S−14.7−3
S614 (m/z=842.513989; rt=14.68184)
T/S Cluster: S−14.7−4
S615 (m/z=843.517074; rt=14.68378)
T/S Cluster: S−14.7−4
S617 (m/z=300.992893; rt=14.76514)
T/S Cluster: S−14.8−1
S629 (m/z=720.447285; rt=14.7837)
T/S Cluster: S−14.8−2
S625 (m/z=652.420768; rt=14.77929)
T/S Cluster: S−14.8−2
S627 (m/z=652.421052; rt=14.77975)
T/S Cluster: S−14.8−2
PCo1
PCo2
0.4
0.2
0.0
−0.2
−0.4
−0.6

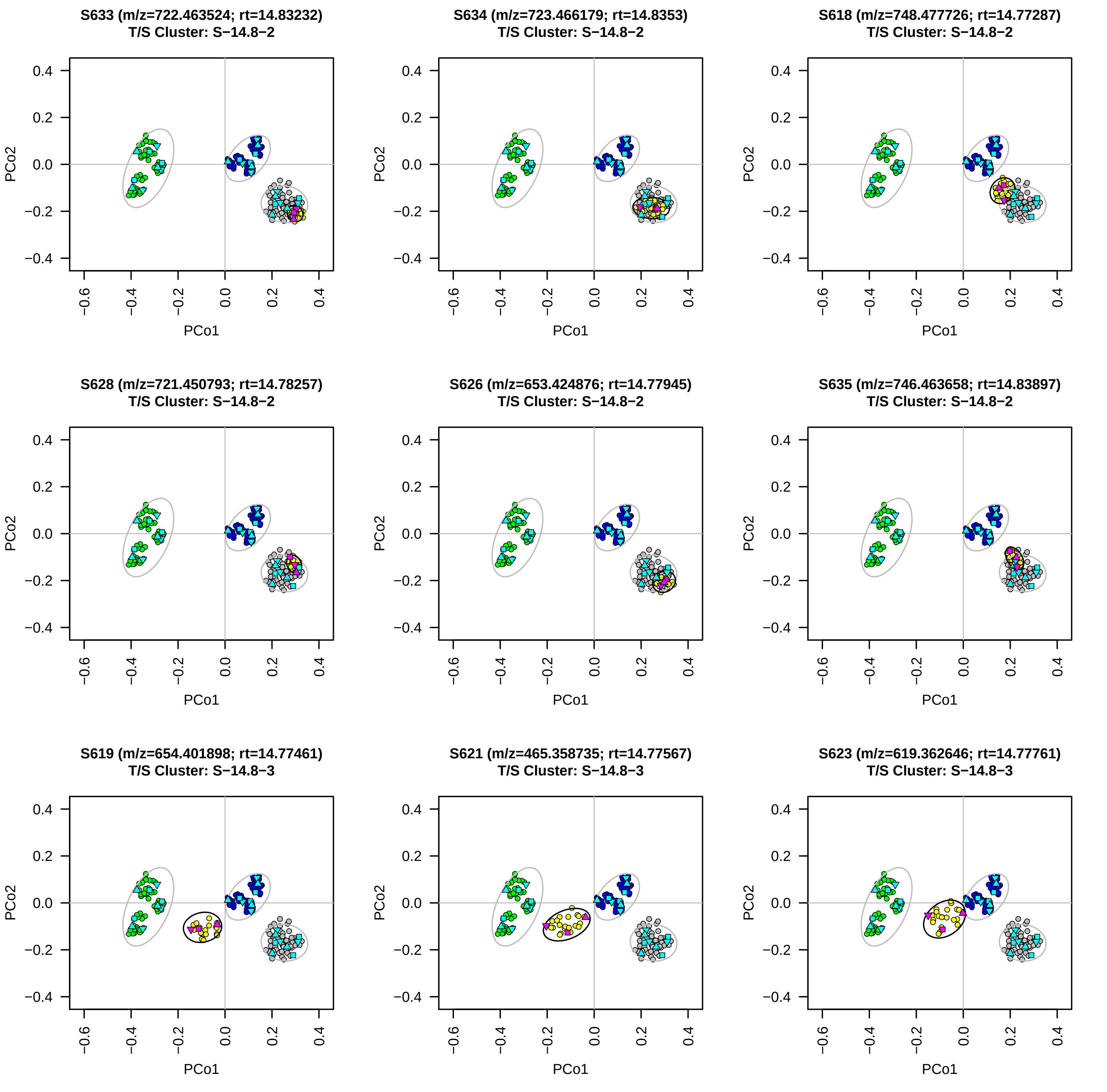
S633 (m/z=722.463524; rt=14.83232)
T/S Cluster: S−14.8−2
S634 (m/z=723.466179; rt=14.8353)
T/S Cluster: S−14.8−2
S618 (m/z=748.477726; rt=14.77287)
T/S Cluster: S−14.8−2
S628 (m/z=721.450793; rt=14.78257)
T/S Cluster: S−14.8−2
S626 (m/z=653.424876; rt=14.77945)
T/S Cluster: S−14.8−2
S635 (m/z=746.463658; rt=14.83897)
T/S Cluster: S−14.8−2
S619 (m/z=654.401898; rt=14.77461)
T/S Cluster: S−14.8−3
S621 (m/z=465.358735; rt=14.77567)
T/S Cluster: S−14.8−3
S623 (m/z=619.362646; rt=14.77761)
T/S Cluster: S−14.8−3
PCo1
PCo2
0.4
0.2
0.0
−0.2
−0.4
−0.6

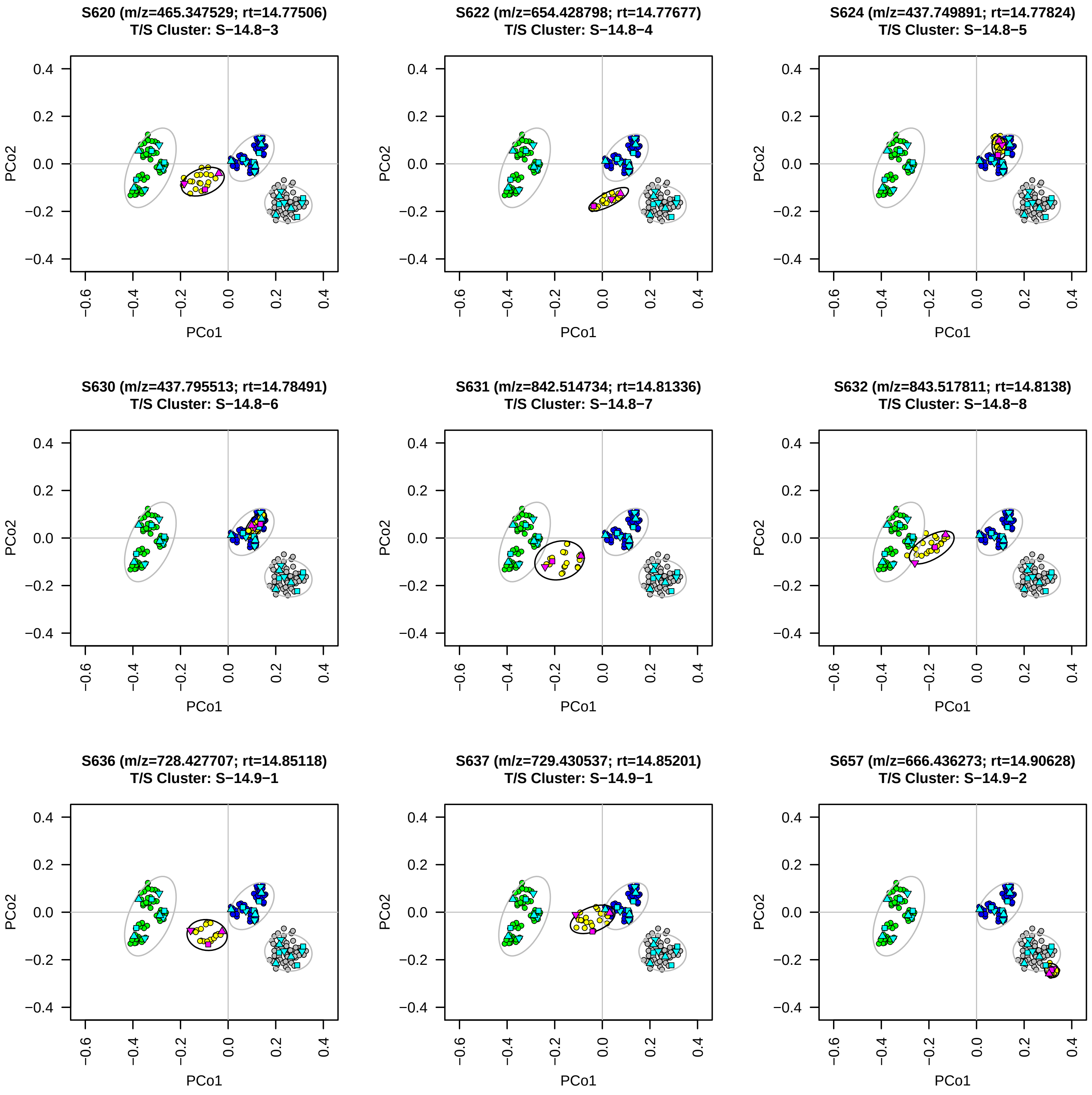
S620 (m/z=465.347529; rt=14.77506)
T/S Cluster: S−14.8−3
S622 (m/z=654.428798; rt=14.77677)
T/S Cluster: S−14.8−4
S624 (m/z=437.749891; rt=14.77824)
T/S Cluster: S−14.8−5
S630 (m/z=437.795513; rt=14.78491)
T/S Cluster: S−14.8−6
S631 (m/z=842.514734; rt=14.81336)
T/S Cluster: S−14.8−7
S632 (m/z=843.517811; rt=14.8138)
T/S Cluster: S−14.8−8
S636 (m/z=728.427707; rt=14.85118)
T/S Cluster: S−14.9−1
S637 (m/z=729.430537; rt=14.85201)
T/S Cluster: S−14.9−1
S657 (m/z=666.436273; rt=14.90628)
T/S Cluster: S−14.9−2
PCo1
PCo2
0.4
0.2
0.0
−0.2
−0.4
−0.6

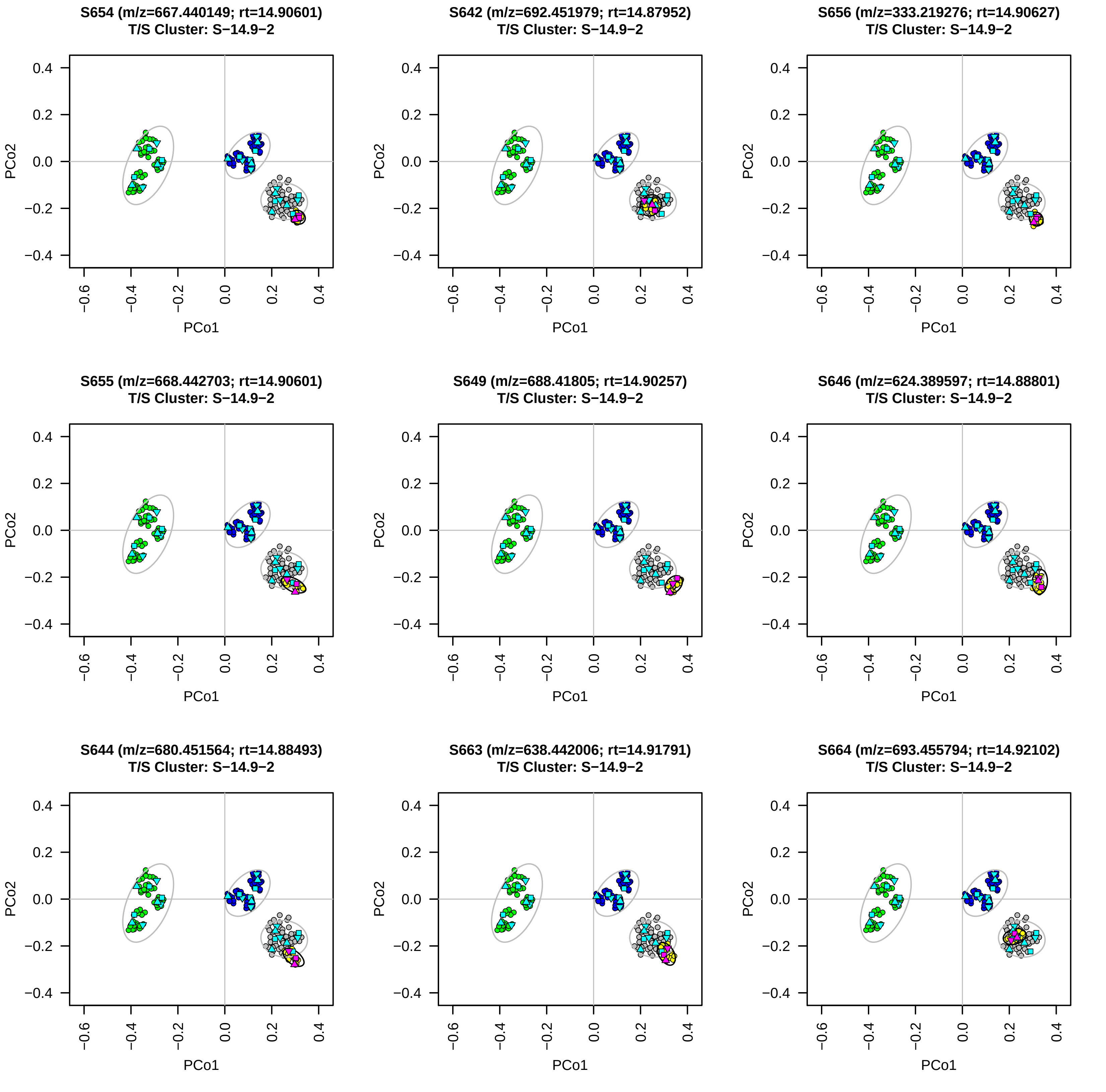
S654 (m/z=667.440149; rt=14.90601)
T/S Cluster: S−14.9−2
S642 (m/z=692.451979; rt=14.87952)
T/S Cluster: S−14.9−2
S656 (m/z=333.219276; rt=14.90627)
T/S Cluster: S−14.9−2
S655 (m/z=668.442703; rt=14.90601)
T/S Cluster: S−14.9−2
S649 (m/z=688.41805; rt=14.90257)
T/S Cluster: S−14.9−2
S646 (m/z=624.389597; rt=14.88801)
T/S Cluster: S−14.9−2
S644 (m/z=680.451564; rt=14.88493)
T/S Cluster: S−14.9−2
S663 (m/z=638.442006; rt=14.91791)
T/S Cluster: S−14.9−2
S664 (m/z=693.455794; rt=14.92102)
T/S Cluster: S−14.9−2
PCo1
PCo2
−0.6
−0.4
−0.2
0.0
0.2
0.4

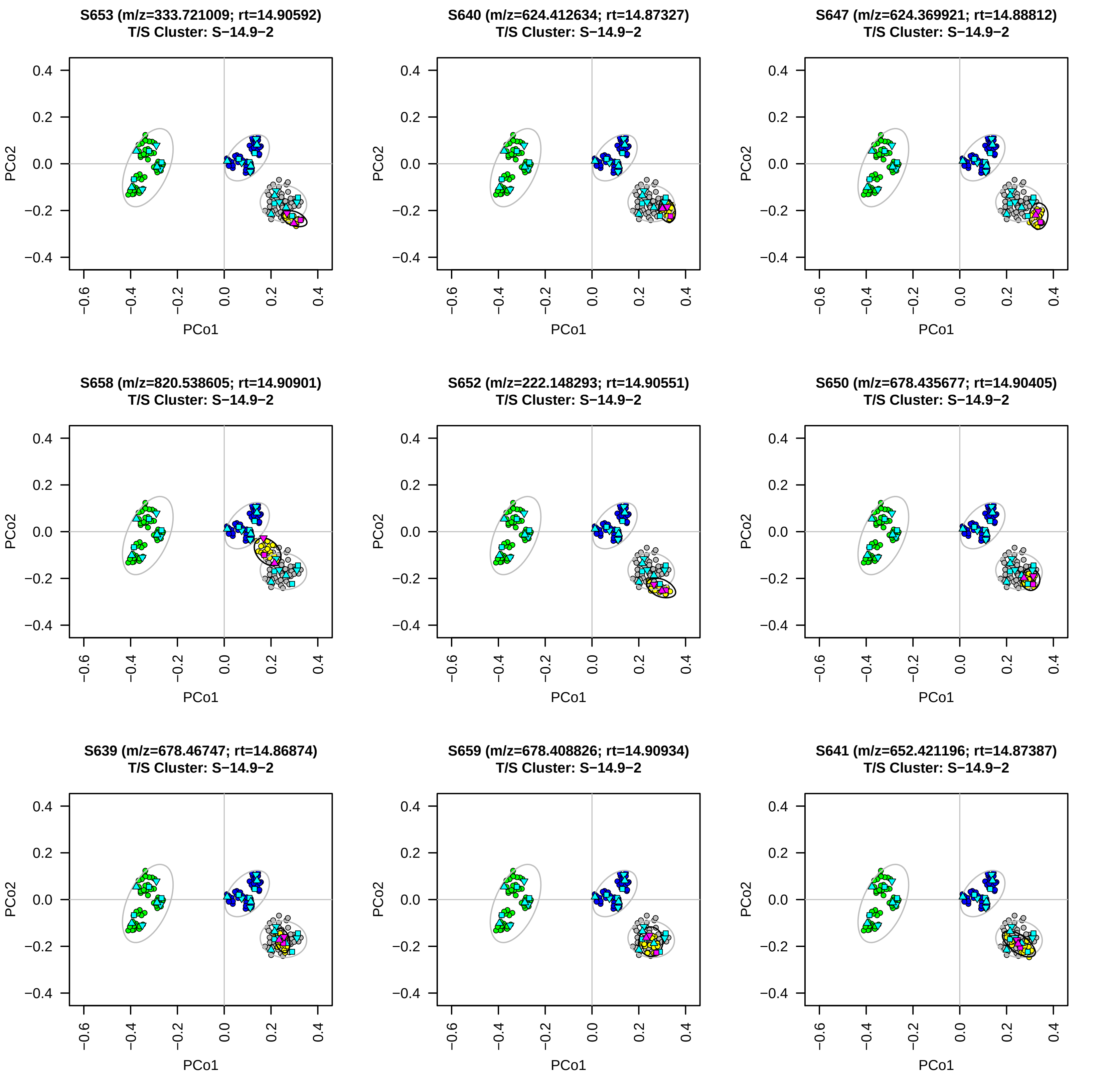
S653 (m/z=333.721009; rt=14.90592)
T/S Cluster: S−14.9−2
S640 (m/z=624.412634; rt=14.87327)
T/S Cluster: S−14.9−2
S647 (m/z=624.369921; rt=14.88812)
T/S Cluster: S−14.9−2
S658 (m/z=820.538605; rt=14.90901)
T/S Cluster: S−14.9−2
S652 (m/z=222.148293; rt=14.90551)
T/S Cluster: S−14.9−2
S650 (m/z=678.435677; rt=14.90405)
T/S Cluster: S−14.9−2
S639 (m/z=678.46747; rt=14.86874)
T/S Cluster: S−14.9−2
S659 (m/z=678.408826; rt=14.90934)
T/S Cluster: S−14.9−2
S641 (m/z=652.421196; rt=14.87387)
T/S Cluster: S−14.9−2
PCo1
PCo2
0.4
0.2
0.0
−0.2
−0.4
−0.6

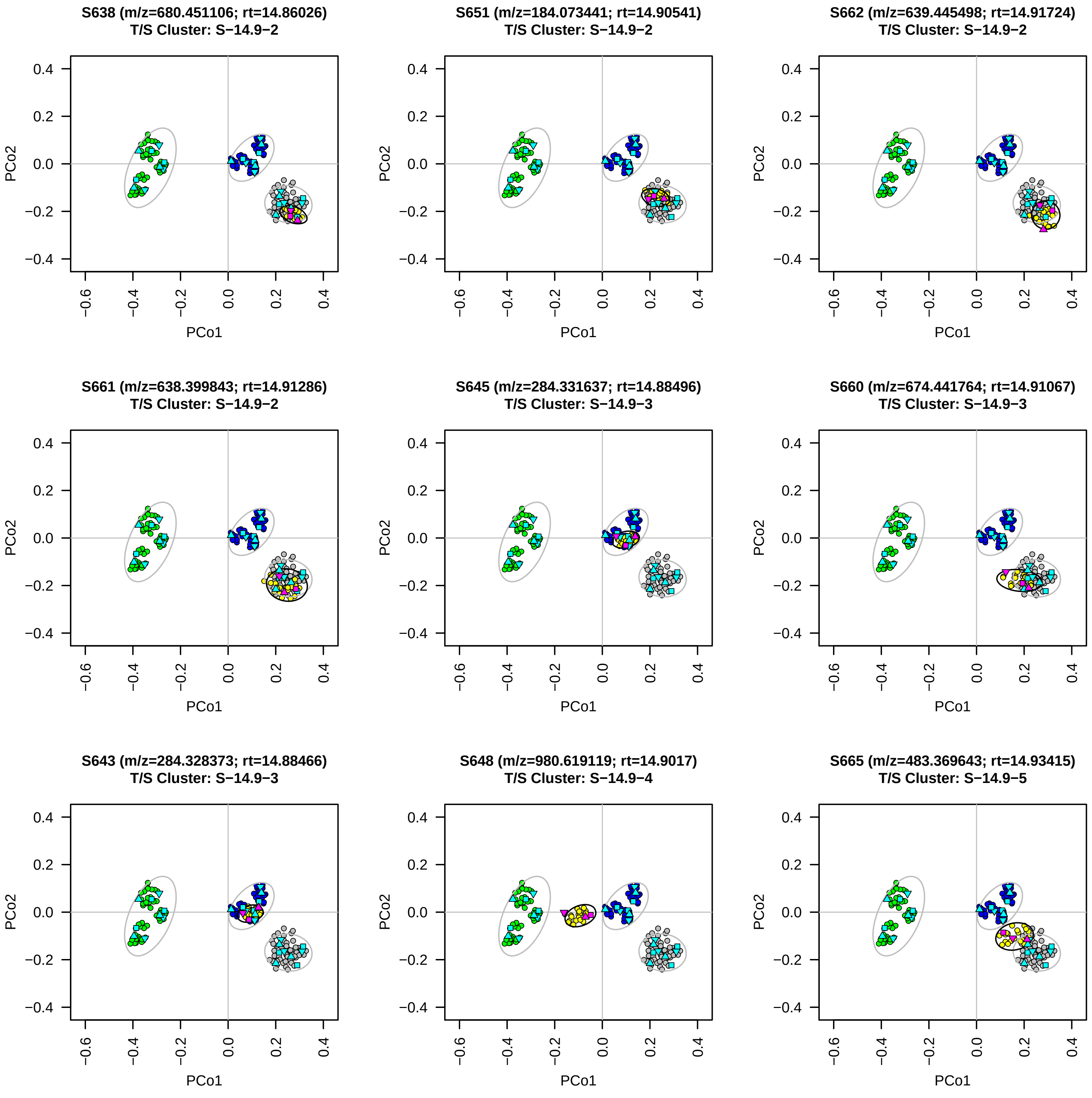
S638 (m/z=680.451106; rt=14.86026)
T/S Cluster: S−14.9−2
S651 (m/z=184.073441; rt=14.90541)
T/S Cluster: S−14.9−2
S662 (m/z=639.445498; rt=14.91724)
T/S Cluster: S−14.9−2
S661 (m/z=638.399843; rt=14.91286)
T/S Cluster: S−14.9−2
S645 (m/z=284.331637; rt=14.88496)
T/S Cluster: S−14.9−3
S660 (m/z=674.441764; rt=14.91067)
T/S Cluster: S−14.9−3
S643 (m/z=284.328373; rt=14.88466)
T/S Cluster: S−14.9−3
S648 (m/z=980.619119; rt=14.9017)
T/S Cluster: S−14.9−4
S665 (m/z=483.369643; rt=14.93415)
T/S Cluster: S−14.9−5
PCo1
PCo2
−0.6 −0.4 −0.2 0.0 0.2 0.4
−0.4 −0.2 0.0 0.2 0.4

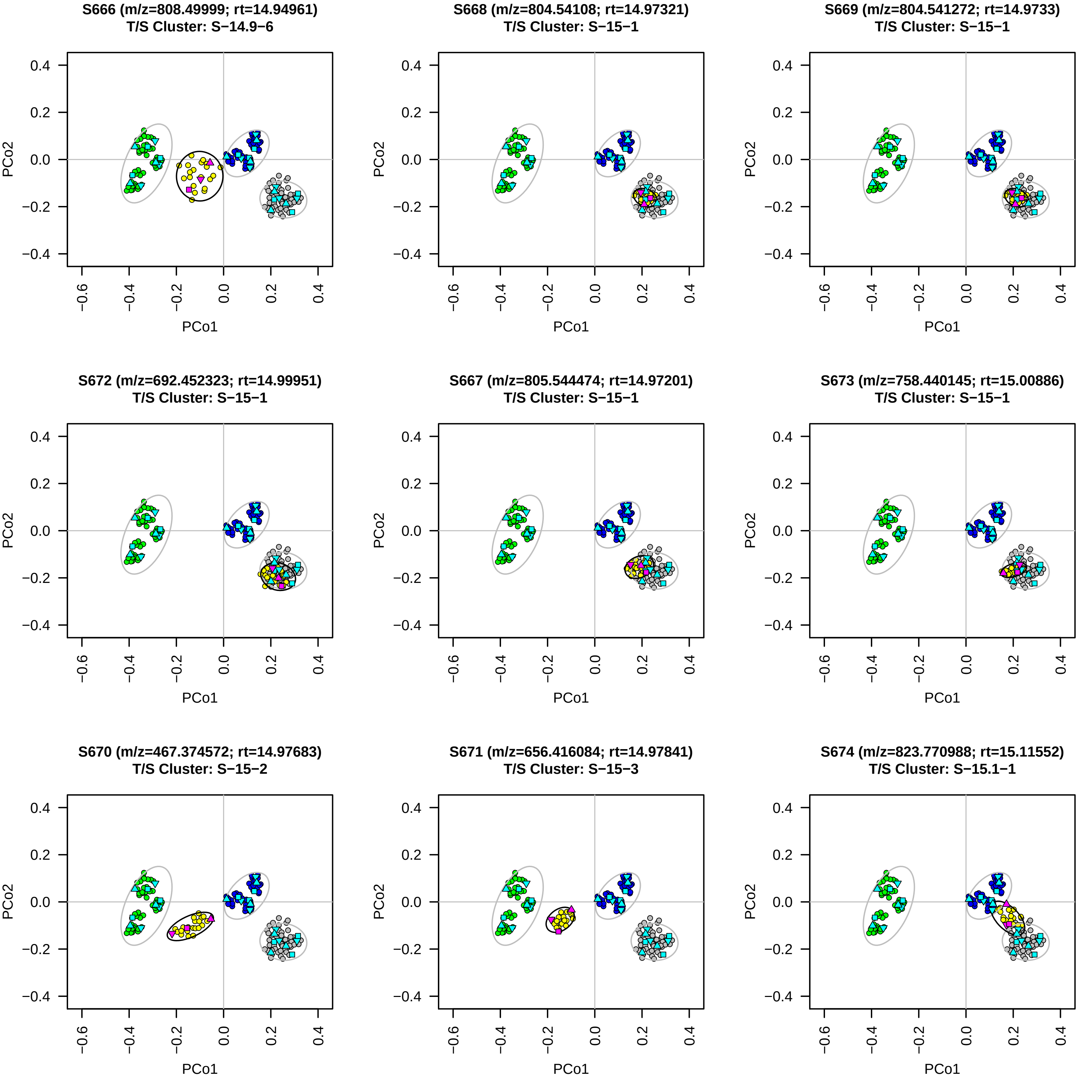
S666 (m/z=808.49999; rt=14.94961)
T/S Cluster: S−14.9−6
S668 (m/z=804.54108; rt=14.97321)
T/S Cluster: S−15−1
S669 (m/z=804.541272; rt=14.9733)
T/S Cluster: S−15−1
S672 (m/z=692.452323; rt=14.99951)
T/S Cluster: S−15−1
S667 (m/z=805.544474; rt=14.97201)
T/S Cluster: S−15−1
S673 (m/z=758.440145; rt=15.00886)
T/S Cluster: S−15−1
S670 (m/z=467.374572; rt=14.97683)
T/S Cluster: S−15−2
S671 (m/z=656.416084; rt=14.97841)
T/S Cluster: S−15−3
S674 (m/z=823.770988; rt=15.11552)
T/S Cluster: S−15.1−1
PCo1
PCo2
−0.6
−0.4
−0.2
0.0
0.2
0.4

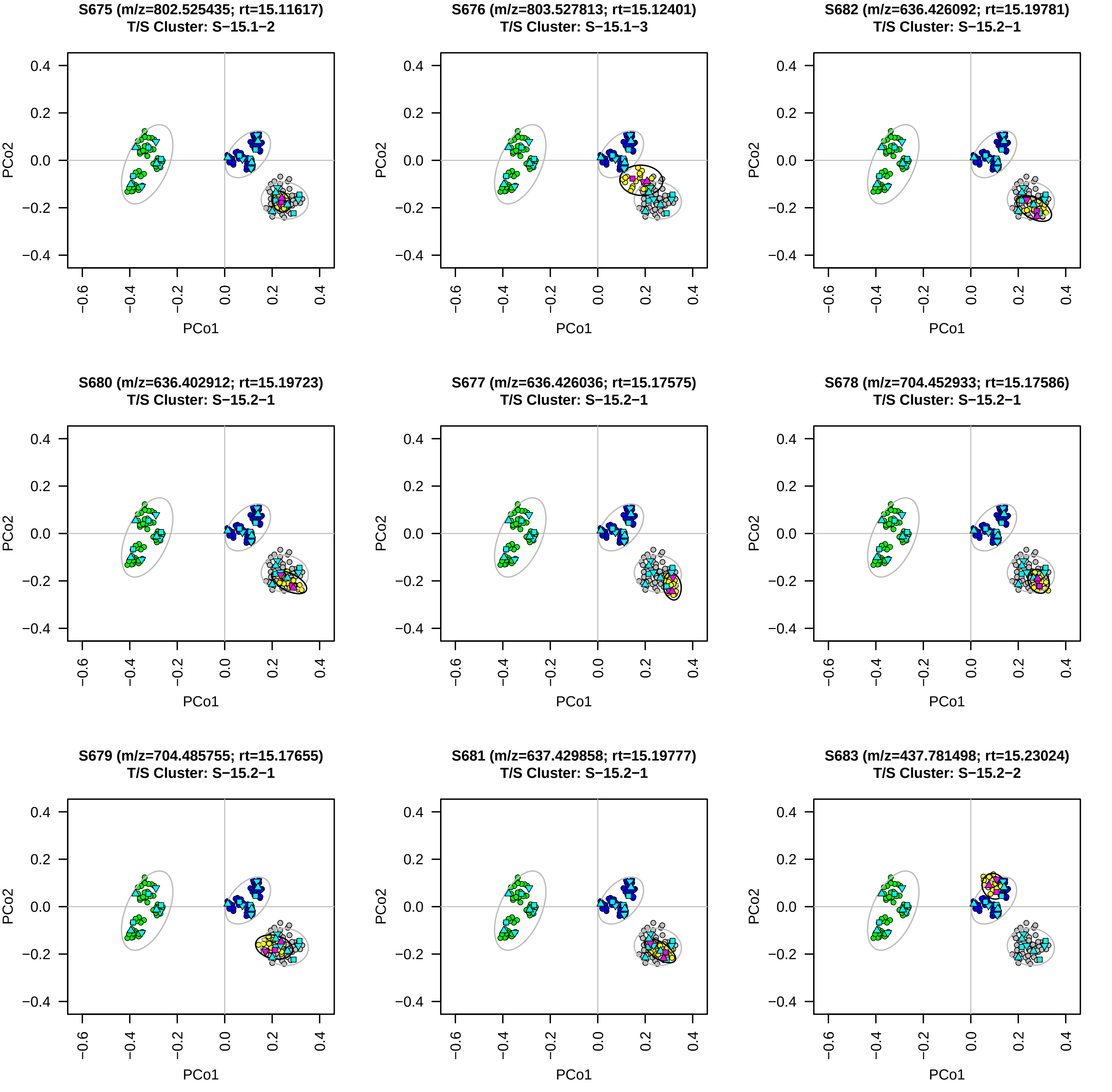
S675 (m/z=802.525435; rt=15.11617)
T/S Cluster: S−15.1−2
S676 (m/z=803.527813; rt=15.12401)
T/S Cluster: S−15.1−3
S682 (m/z=636.426092; rt=15.19781)
T/S Cluster: S−15.2−1
S680 (m/z=636.402912; rt=15.19723)
T/S Cluster: S−15.2−1
S677 (m/z=636.426036; rt=15.17575)
T/S Cluster: S−15.2−1
S678 (m/z=704.452933; rt=15.17586)
T/S Cluster: S−15.2−1
S679 (m/z=704.485755; rt=15.17655)
T/S Cluster: S−15.2−1
S681 (m/z=637.429858; rt=15.19777)
T/S Cluster: S−15.2−1
S683 (m/z=437.781498; rt=15.23024)
T/S Cluster: S−15.2−2
PCo1
PCo2
−0.6
−0.4
−0.2
0.0
0.2
0.4

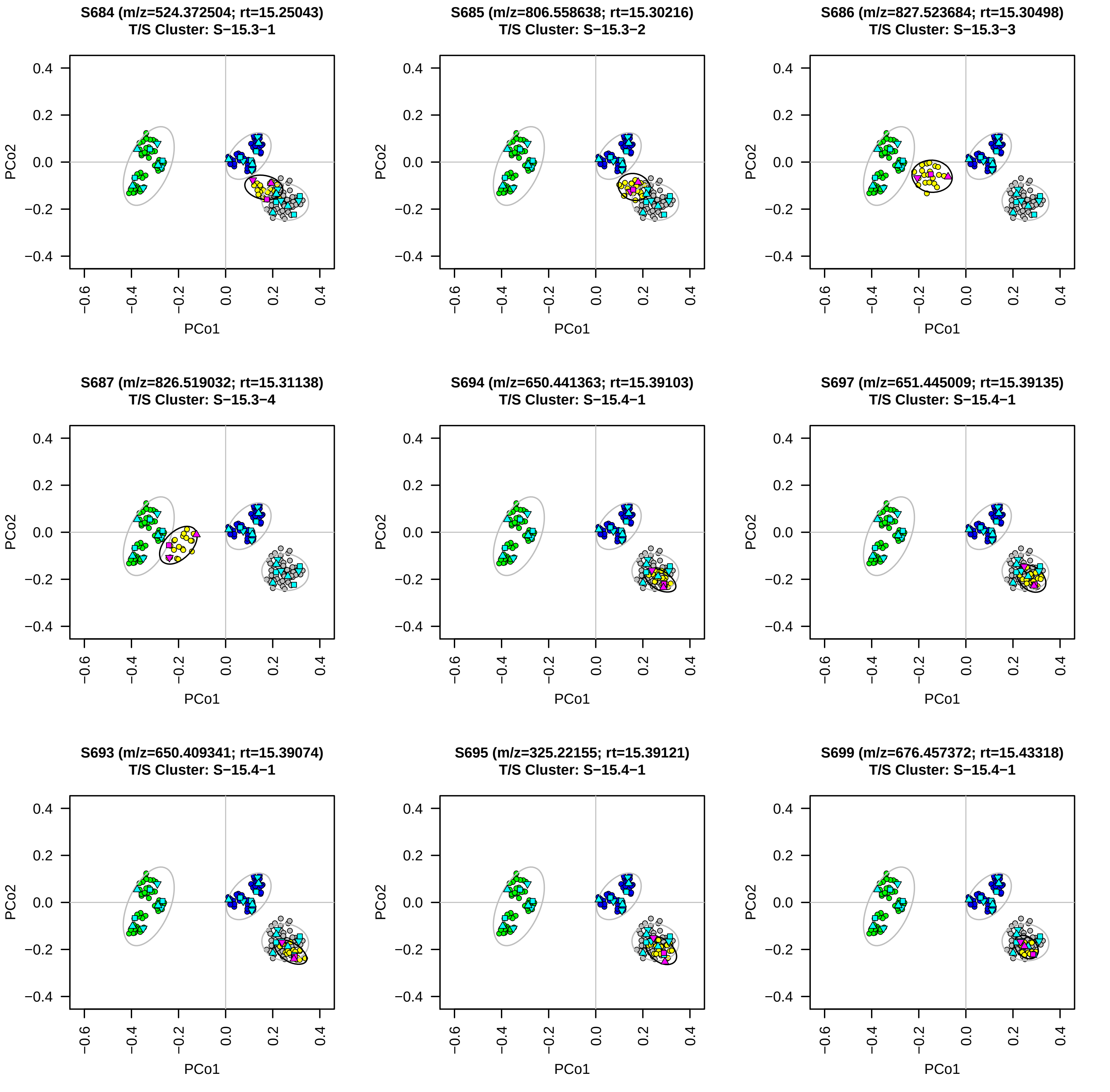
S684 (m/z=524.372504; rt=15.25043)
T/S Cluster: S−15.3−1
S685 (m/z=806.558638; rt=15.30216)
T/S Cluster: S−15.3−2
S686 (m/z=827.523684; rt=15.30498)
T/S Cluster: S−15.3−3
S687 (m/z=826.519032; rt=15.31138)
T/S Cluster: S−15.3−4
S694 (m/z=650.441363; rt=15.39103)
T/S Cluster: S−15.4−1
S697 (m/z=651.445009; rt=15.39135)
T/S Cluster: S−15.4−1
S693 (m/z=650.409341; rt=15.39074)
T/S Cluster: S−15.4−1
S695 (m/z=325.22155; rt=15.39121)
T/S Cluster: S−15.4−1
S699 (m/z=676.457372; rt=15.43318)
T/S Cluster: S−15.4−1
PCo1
PCo2

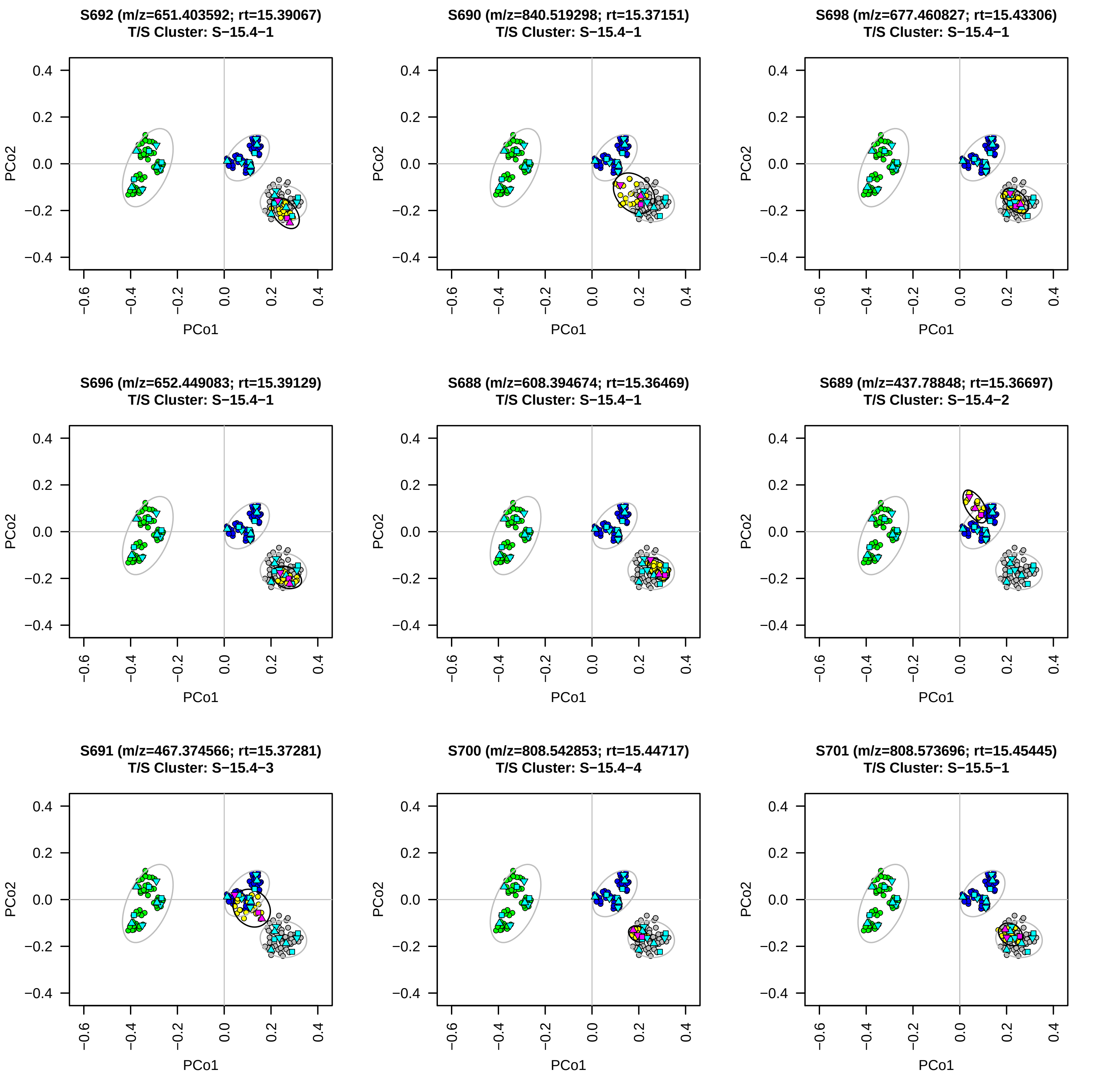
S692 (m/z=651.403592; rt=15.39067)
T/S Cluster: S−15.4−1
PCo2
PCo1
S690 (m/z=840.519298; rt=15.37151)
T/S Cluster: S−15.4−1
PCo2
PCo1
S698 (m/z=677.460827; rt=15.43306)
T/S Cluster: S−15.4−1
PCo2
PCo1
S696 (m/z=652.449083; rt=15.39129)
T/S Cluster: S−15.4−1
PCo2
PCo1
S688 (m/z=608.394674; rt=15.36469)
T/S Cluster: S−15.4−1
PCo2
PCo1
S689 (m/z=437.78848; rt=15.36697)
T/S Cluster: S−15.4−2
PCo2
PCo1
S691 (m/z=467.374566; rt=15.37281)
T/S Cluster: S−15.4−3
PCo2
PCo1
S700 (m/z=808.542853; rt=15.44717)
T/S Cluster: S−15.4−4
PCo2
PCo1
S701 (m/z=808.573696; rt=15.45445)
T/S Cluster: S−15.5−1
PCo2
PCo1

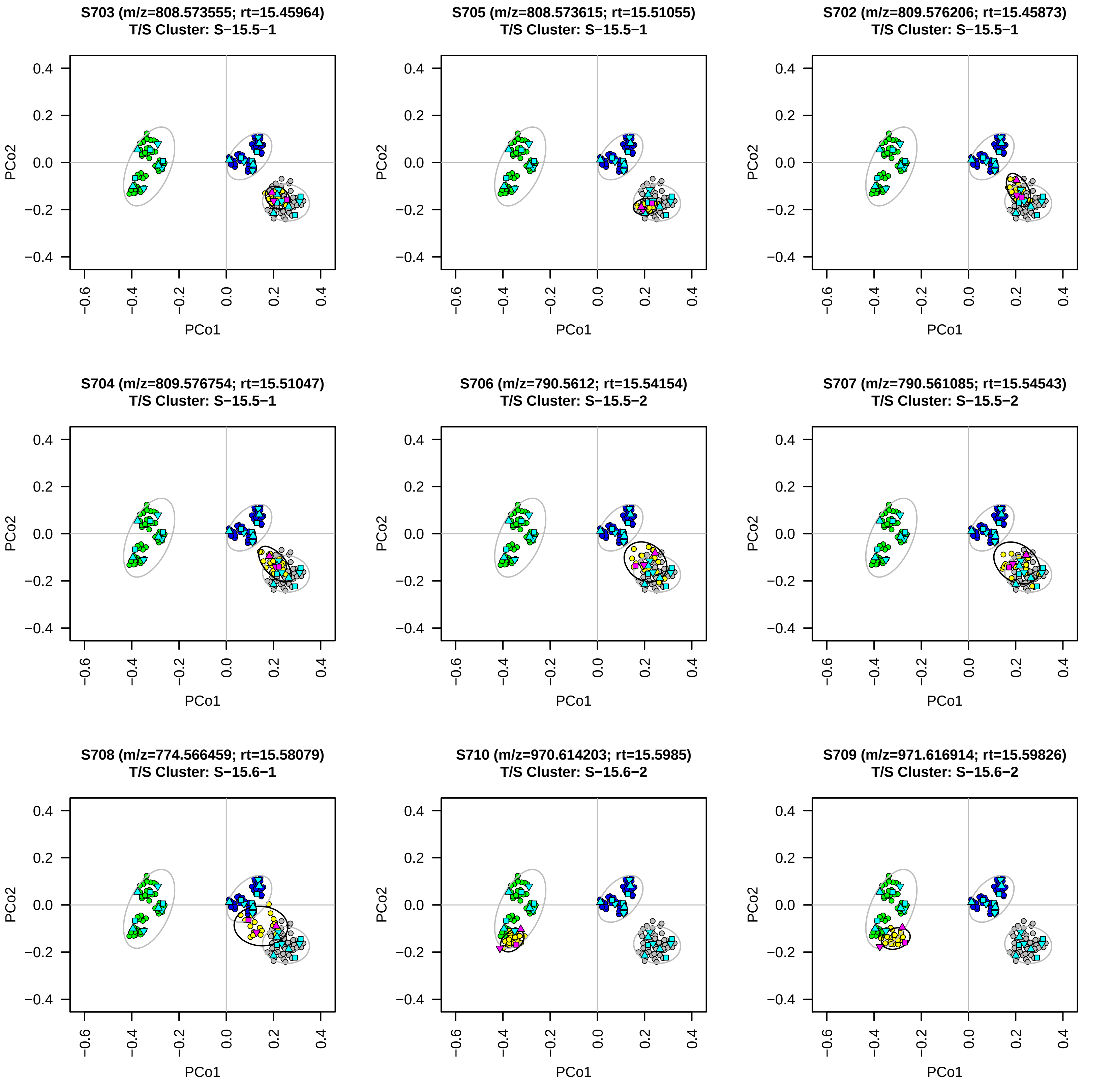
S703 (m/z=808.573555; rt=15.45964)
T/S Cluster: S−15.5−1
S705 (m/z=808.573615; rt=15.51055)
T/S Cluster: S−15.5−1
S702 (m/z=809.576206; rt=15.45873)
T/S Cluster: S−15.5−1
S704 (m/z=809.576754; rt=15.51047)
T/S Cluster: S−15.5−1
S706 (m/z=790.5612; rt=15.54154)
T/S Cluster: S−15.5−2
S707 (m/z=790.561085; rt=15.54543)
T/S Cluster: S−15.5−2
S708 (m/z=774.566459; rt=15.58079)
T/S Cluster: S−15.6−1
S710 (m/z=970.614203; rt=15.5985)
T/S Cluster: S−15.6−2
S709 (m/z=971.616914; rt=15.59826)
T/S Cluster: S−15.6−2
PCo1
PCo2
0.4
0.2
0.0
−0.2
−0.4
−0.6

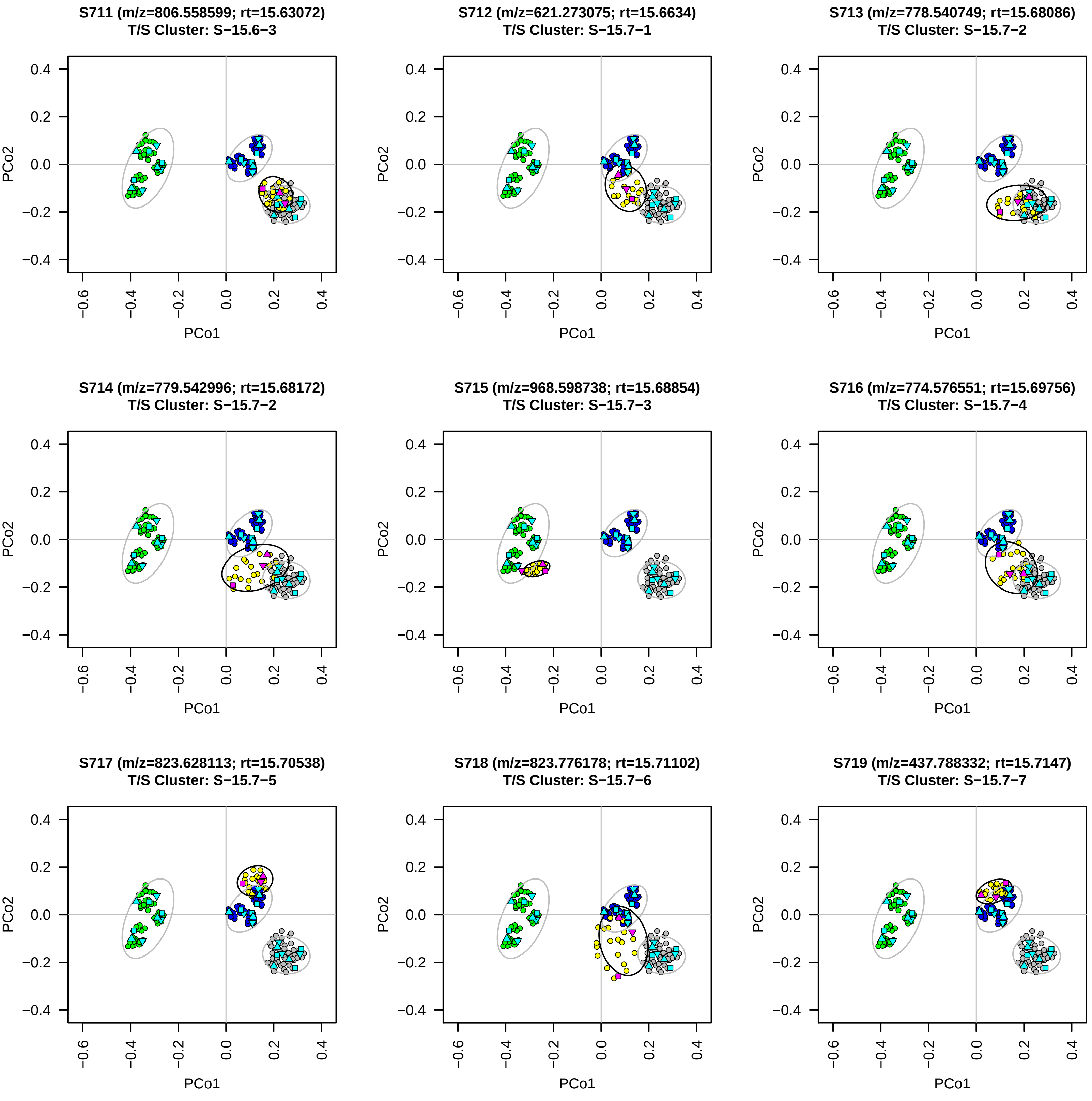
S711 (m/z=806.558599; rt=15.63072)
T/S Cluster: S−15.6−3
S712 (m/z=621.273075; rt=15.6634)
T/S Cluster: S−15.7−1
S713 (m/z=778.540749; rt=15.68086)
T/S Cluster: S−15.7−2
S714 (m/z=779.542996; rt=15.68172)
T/S Cluster: S−15.7−2
S715 (m/z=968.598738; rt=15.68854)
T/S Cluster: S−15.7−3
S716 (m/z=774.576551; rt=15.69756)
T/S Cluster: S−15.7−4
S717 (m/z=823.628113; rt=15.70538)
T/S Cluster: S−15.7−5
S718 (m/z=823.776178; rt=15.71102)
T/S Cluster: S−15.7−6
S719 (m/z=437.788332; rt=15.7147)
T/S Cluster: S−15.7−7
PCo1
PCo2
0.4
0.2
0.0
−0.2
−0.4
−0.6

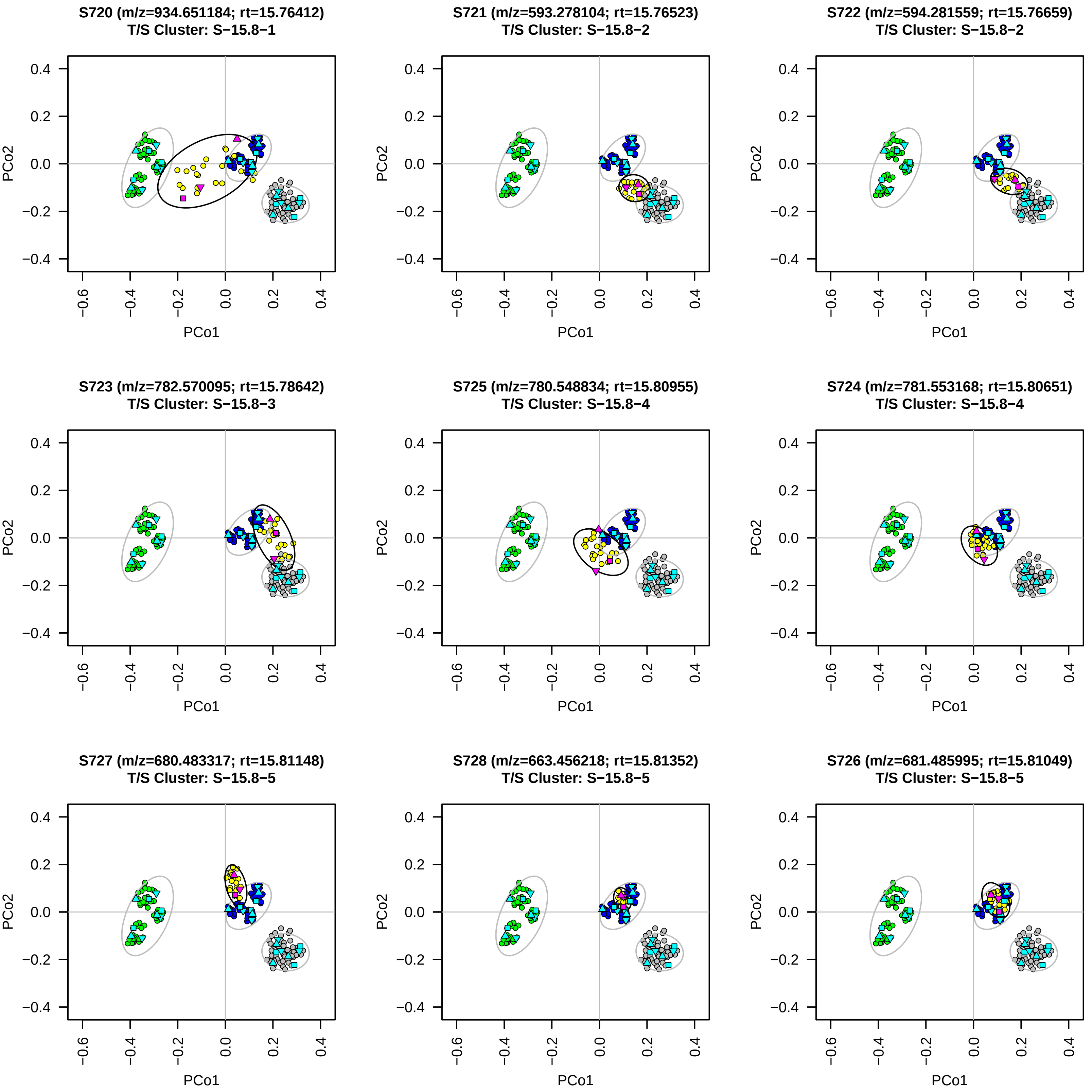
S720 (m/z=934.651184; rt=15.76412)
T/S Cluster: S−15.8−1
S721 (m/z=593.278104; rt=15.76523)
T/S Cluster: S−15.8−2
S722 (m/z=594.281559; rt=15.76659)
T/S Cluster: S−15.8−2
S723 (m/z=782.570095; rt=15.78642)
T/S Cluster: S−15.8−3
S725 (m/z=780.548834; rt=15.80955)
T/S Cluster: S−15.8−4
S724 (m/z=781.553168; rt=15.80651)
T/S Cluster: S−15.8−4
S727 (m/z=680.483317; rt=15.81148)
T/S Cluster: S−15.8−5
S728 (m/z=663.456218; rt=15.81352)
T/S Cluster: S−15.8−5
S726 (m/z=681.485995; rt=15.81049)
T/S Cluster: S−15.8−5
PCo1
PCo2
−0.6
−0.4
−0.2
0.0
0.2
0.4

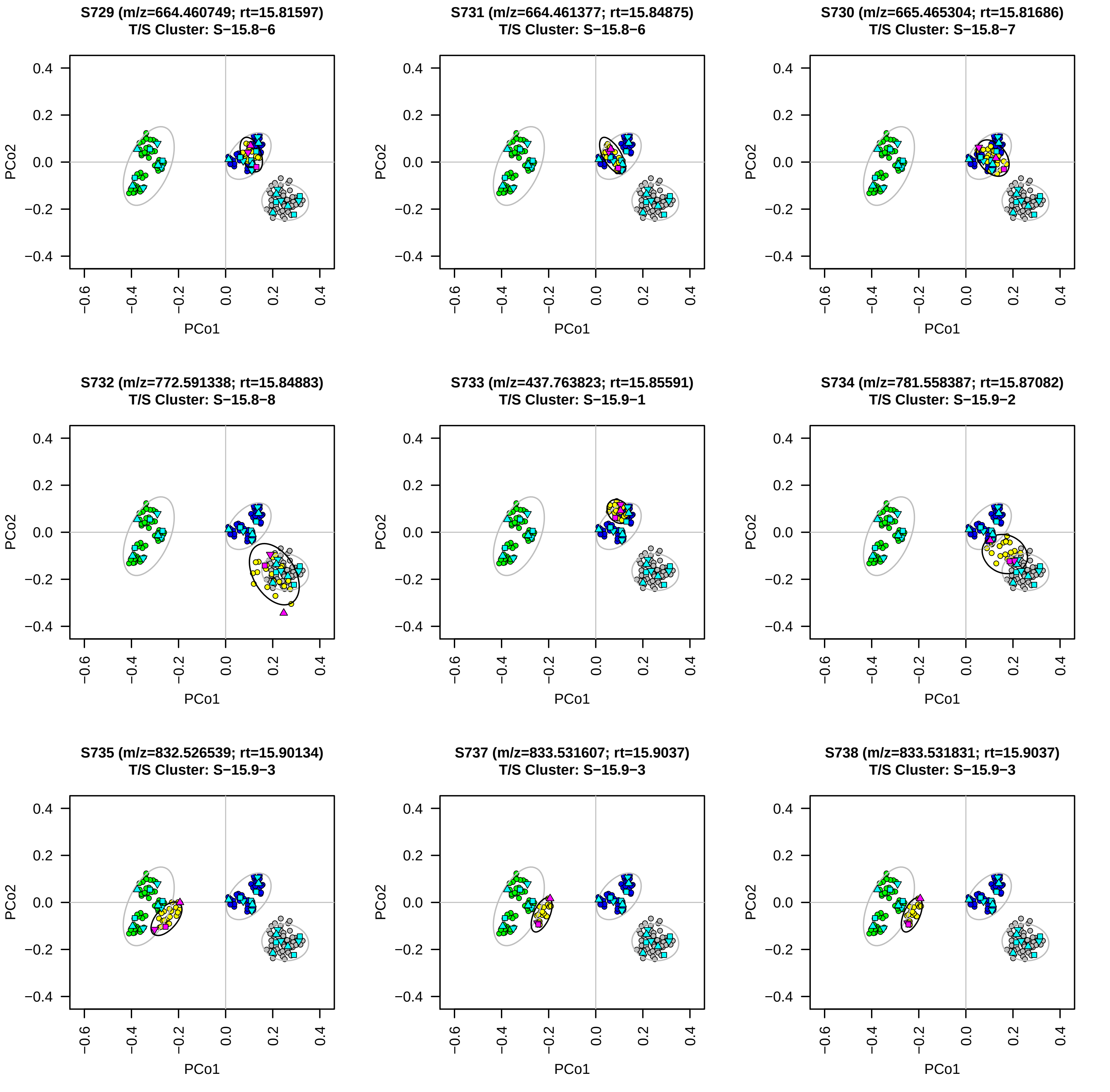
S729 (m/z=664.460749; rt=15.81597)
T/S Cluster: S−15.8−6
S731 (m/z=664.461377; rt=15.84875)
T/S Cluster: S−15.8−6
S730 (m/z=665.465304; rt=15.81686)
T/S Cluster: S−15.8−7
S732 (m/z=772.591338; rt=15.84883)
T/S Cluster: S−15.8−8
S733 (m/z=437.763823; rt=15.85591)
T/S Cluster: S−15.9−1
S734 (m/z=781.558387; rt=15.87082)
T/S Cluster: S−15.9−2
S735 (m/z=832.526539; rt=15.90134)
T/S Cluster: S−15.9−3
S737 (m/z=833.531607; rt=15.9037)
T/S Cluster: S−15.9−3
S738 (m/z=833.531831; rt=15.9037)
T/S Cluster: S−15.9−3
PCo1
PCo2
−0.6
−0.4
−0.2
0.0
0.2
0.4

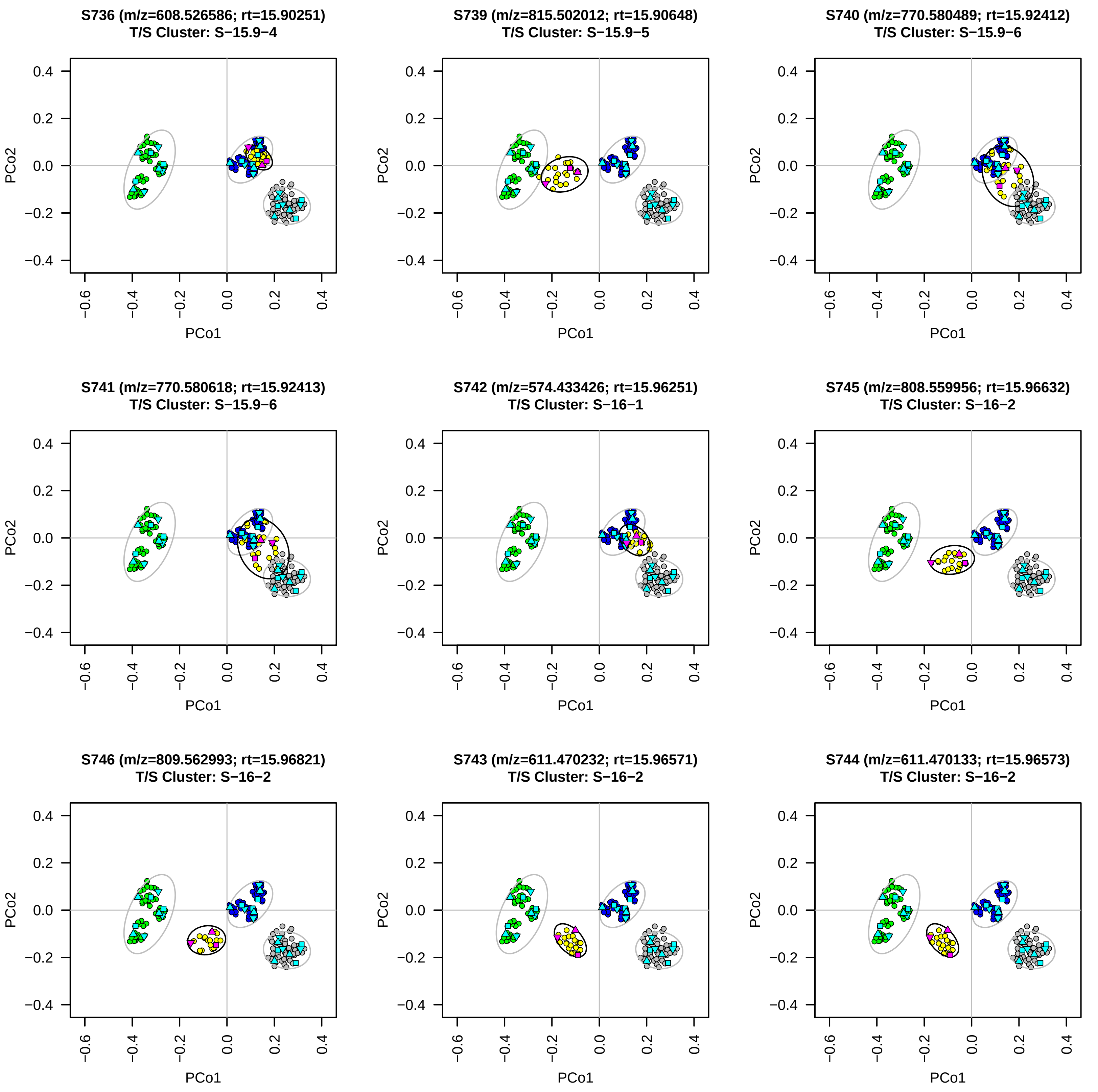
S736 (m/z=608.526586; rt=15.90251)
T/S Cluster: S−15.9−4
S739 (m/z=815.502012; rt=15.90648)
T/S Cluster: S−15.9−5
S740 (m/z=770.580489; rt=15.92412)
T/S Cluster: S−15.9−6
S741 (m/z=770.580618; rt=15.92413)
T/S Cluster: S−15.9−6
S742 (m/z=574.433426; rt=15.96251)
T/S Cluster: S−16−1
S745 (m/z=808.559956; rt=15.96632)
T/S Cluster: S−16−2
S746 (m/z=809.562993; rt=15.96821)
T/S Cluster: S−16−2
S743 (m/z=611.470232; rt=15.96571)
T/S Cluster: S−16−2
S744 (m/z=611.470133; rt=15.96573)
T/S Cluster: S−16−2
PCo1
PCo2

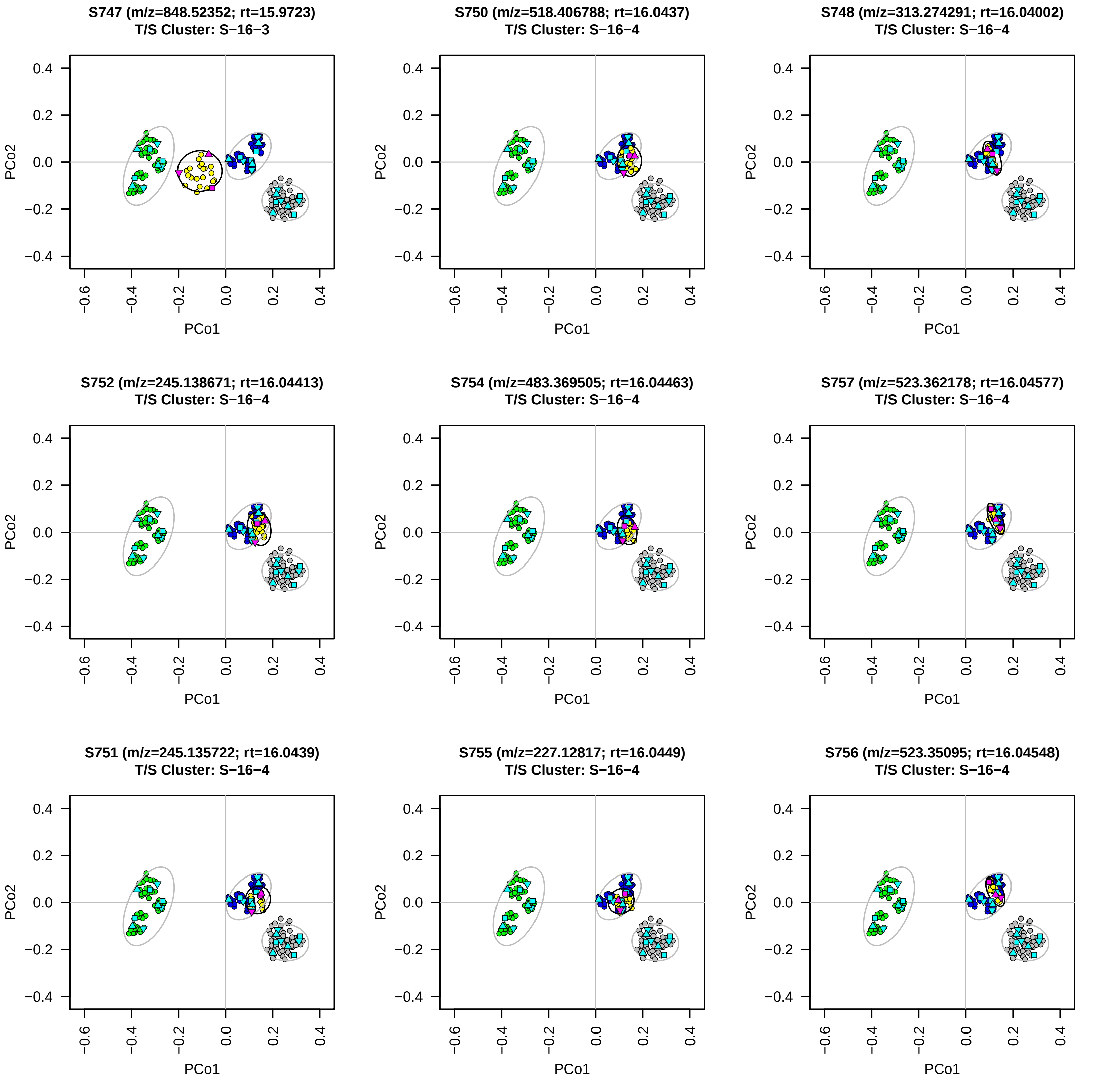
S747 (m/z=848.52352; rt=15.9723)
T/S Cluster: S−16−3
S750 (m/z=518.406788; rt=16.0437)
T/S Cluster: S−16−4
S748 (m/z=313.274291; rt=16.04002)
T/S Cluster: S−16−4
S752 (m/z=245.138671; rt=16.04413)
T/S Cluster: S−16−4
S754 (m/z=483.369505; rt=16.04463)
T/S Cluster: S−16−4
S757 (m/z=523.362178; rt=16.04577)
T/S Cluster: S−16−4
S751 (m/z=245.135722; rt=16.0439)
T/S Cluster: S−16−4
S755 (m/z=227.12817; rt=16.0449)
T/S Cluster: S−16−4
S756 (m/z=523.35095; rt=16.04548)
T/S Cluster: S−16−4
PCo1
PCo2
0.4
0.2
0.0
−0.2
−0.4
−0.6

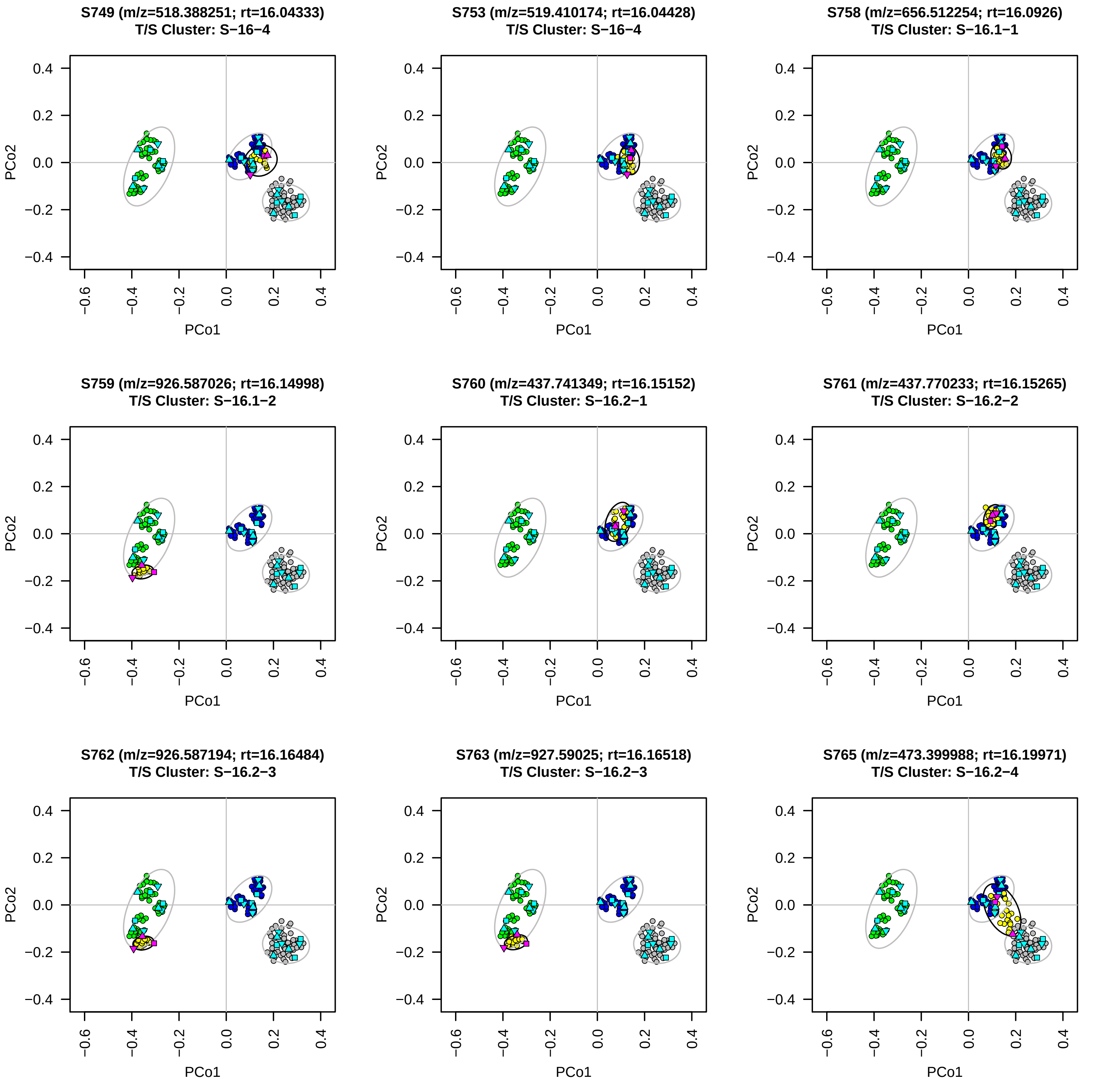
S749 (m/z=518.388251; rt=16.04333)
T/S Cluster: S−16−4
S753 (m/z=519.410174; rt=16.04428)
T/S Cluster: S−16−4
S758 (m/z=656.512254; rt=16.0926)
T/S Cluster: S−16.1−1
S759 (m/z=926.587026; rt=16.14998)
T/S Cluster: S−16.1−2
S760 (m/z=437.741349; rt=16.15152)
T/S Cluster: S−16.2−1
S761 (m/z=437.770233; rt=16.15265)
T/S Cluster: S−16.2−2
S762 (m/z=926.587194; rt=16.16484)
T/S Cluster: S−16.2−3
S763 (m/z=927.59025; rt=16.16518)
T/S Cluster: S−16.2−3
S765 (m/z=473.399988; rt=16.19971)
T/S Cluster: S−16.2−4
PCo1
PCo2
0.4
0.2
0.0
−0.2
−0.4
−0.6

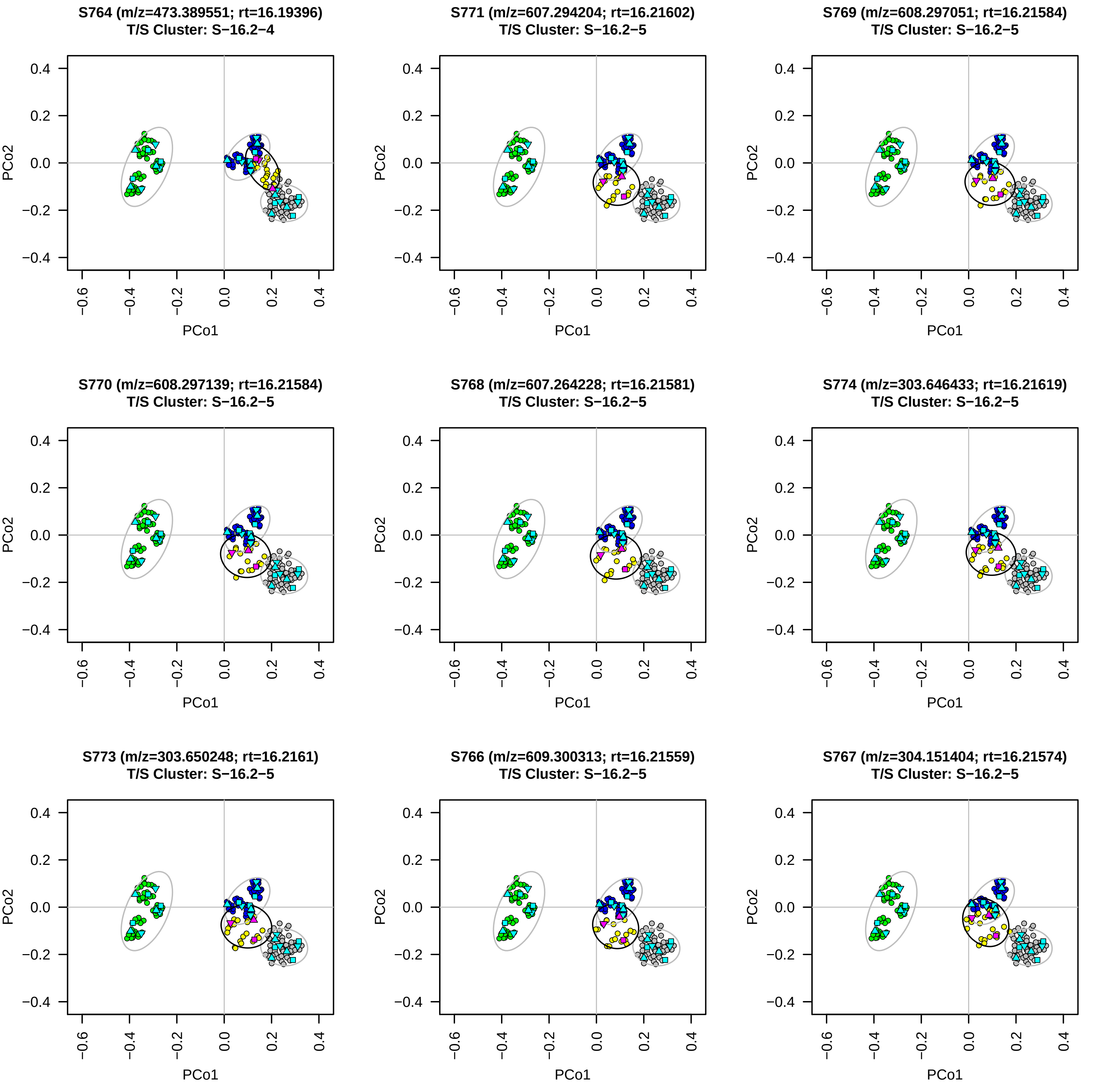
S764 (m/z=473.389551; rt=16.19396)
T/S Cluster: S−16.2−4
S771 (m/z=607.294204; rt=16.21602)
T/S Cluster: S−16.2−5
S769 (m/z=608.297051; rt=16.21584)
T/S Cluster: S−16.2−5
S770 (m/z=608.297139; rt=16.21584)
T/S Cluster: S−16.2−5
S768 (m/z=607.264228; rt=16.21581)
T/S Cluster: S−16.2−5
S774 (m/z=303.646433; rt=16.21619)
T/S Cluster: S−16.2−5
S773 (m/z=303.650248; rt=16.2161)
T/S Cluster: S−16.2−5
S766 (m/z=609.300313; rt=16.21559)
T/S Cluster: S−16.2−5
S767 (m/z=304.151404; rt=16.21574)
T/S Cluster: S−16.2−5
PCo1
PCo2
0.4
0.2
0.0
−0.2
−0.4
−0.6

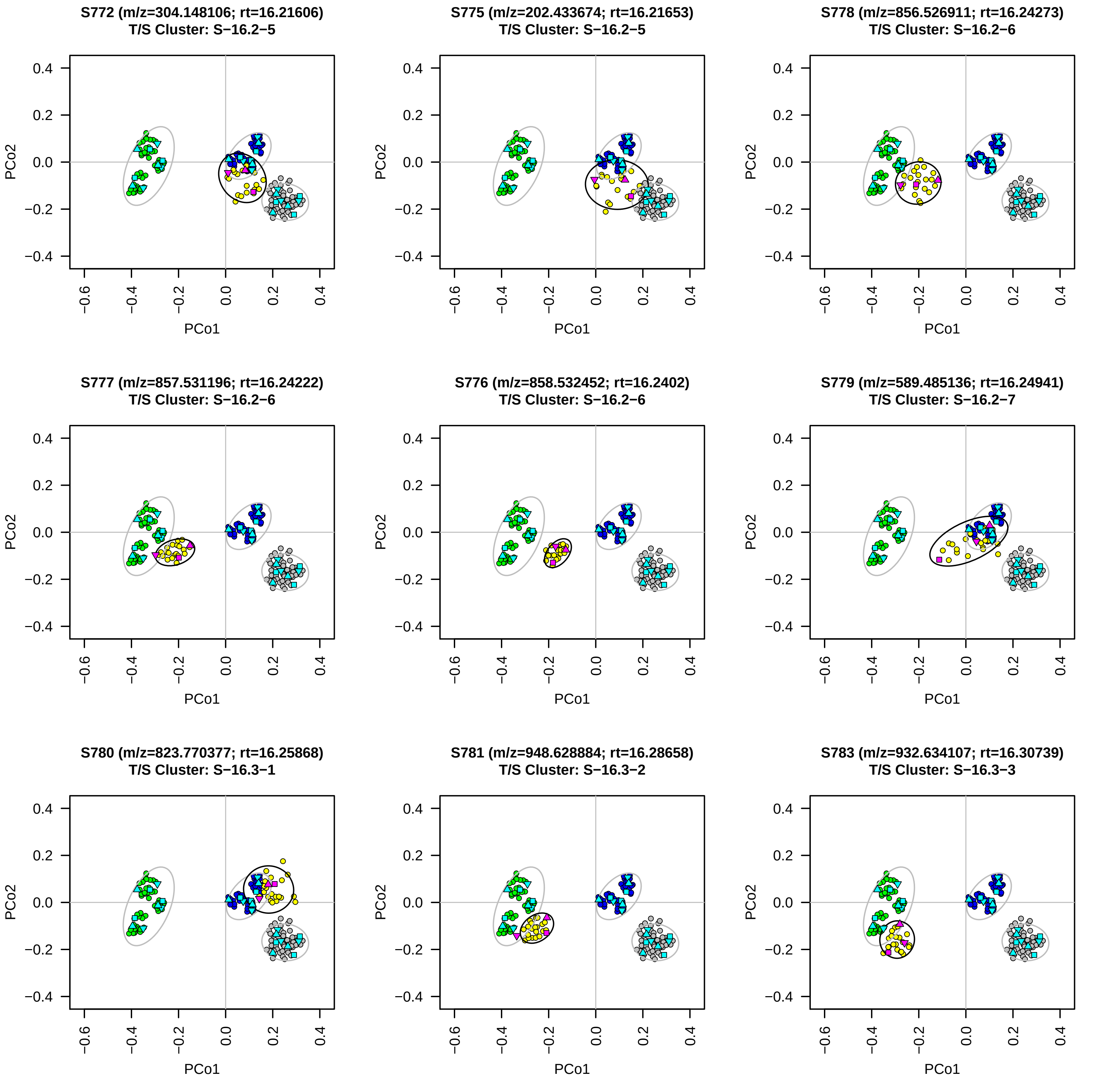
S772 (m/z=304.148106; rt=16.21606)
T/S Cluster: S−16.2−5
S775 (m/z=202.433674; rt=16.21653)
T/S Cluster: S−16.2−5
S778 (m/z=856.526911; rt=16.24273)
T/S Cluster: S−16.2−6
S777 (m/z=857.531196; rt=16.24222)
T/S Cluster: S−16.2−6
S776 (m/z=858.532452; rt=16.2402)
T/S Cluster: S−16.2−6
S779 (m/z=589.485136; rt=16.24941)
T/S Cluster: S−16.2−7
S780 (m/z=823.770377; rt=16.25868)
T/S Cluster: S−16.3−1
S781 (m/z=948.628884; rt=16.28658)
T/S Cluster: S−16.3−2
S783 (m/z=932.634107; rt=16.30739)
T/S Cluster: S−16.3−3
PCo1
PCo2
0.4
0.2
0.0
−0.2
−0.4
−0.6

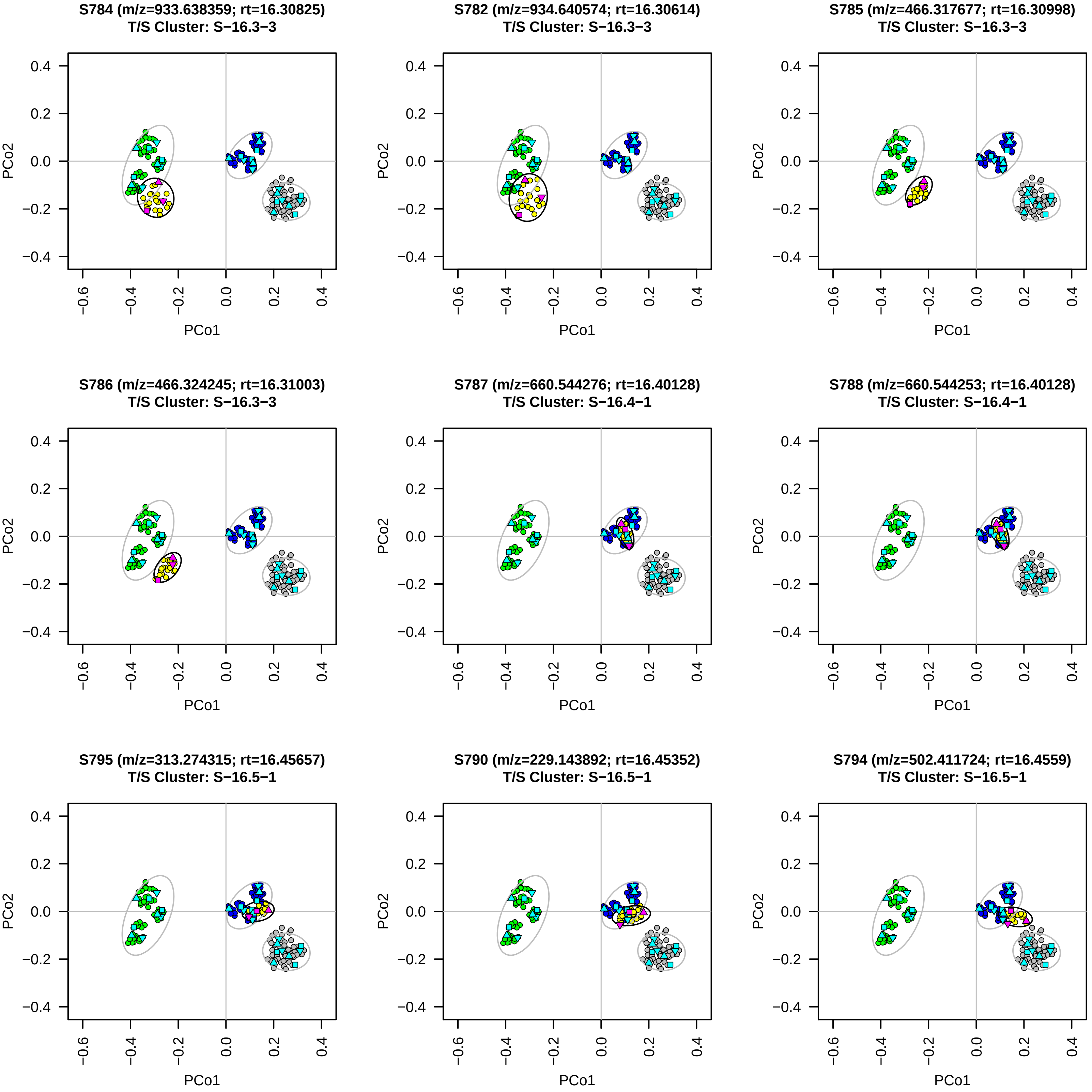
S784 (m/z=933.638359; rt=16.30825)
T/S Cluster: S−16.3−3
S782 (m/z=934.640574; rt=16.30614)
T/S Cluster: S−16.3−3
S785 (m/z=466.317677; rt=16.30998)
T/S Cluster: S−16.3−3
S786 (m/z=466.324245; rt=16.31003)
T/S Cluster: S−16.3−3
S787 (m/z=660.544276; rt=16.40128)
T/S Cluster: S−16.4−1
S788 (m/z=660.544253; rt=16.40128)
T/S Cluster: S−16.4−1
S795 (m/z=313.274315; rt=16.45657)
T/S Cluster: S−16.5−1
S790 (m/z=229.143892; rt=16.45352)
T/S Cluster: S−16.5−1
S794 (m/z=502.411724; rt=16.4559)
T/S Cluster: S−16.5−1
PCo1
PCo2
0.4
0.2
0.0
−0.2
−0.4
−0.6

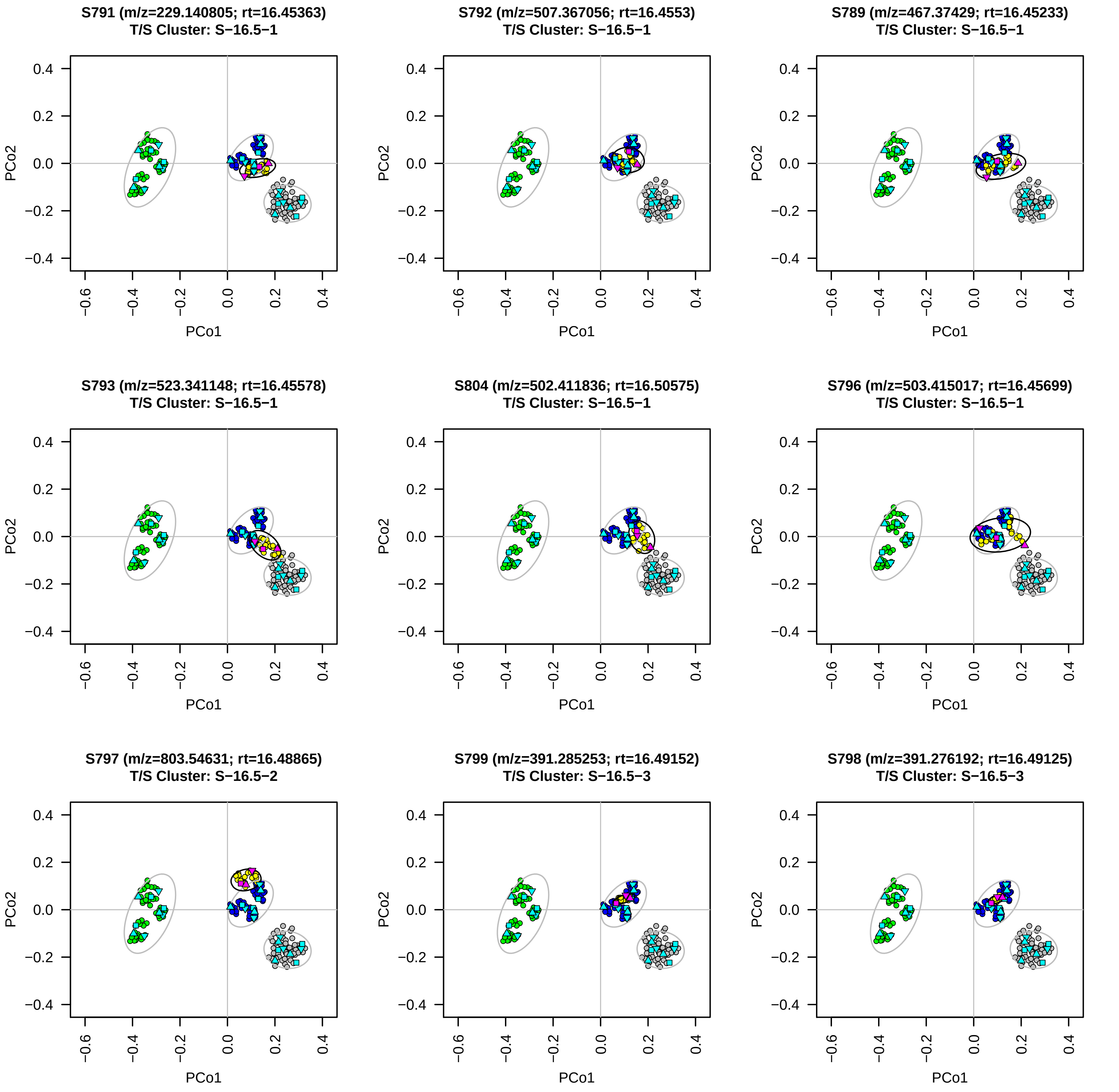
S791 (m/z=229.140805; rt=16.45363)
T/S Cluster: S−16.5−1
S792 (m/z=507.367056; rt=16.4553)
T/S Cluster: S−16.5−1
S789 (m/z=467.37429; rt=16.45233)
T/S Cluster: S−16.5−1
S793 (m/z=523.341148; rt=16.45578)
T/S Cluster: S−16.5−1
S804 (m/z=502.411836; rt=16.50575)
T/S Cluster: S−16.5−1
S796 (m/z=503.415017; rt=16.45699)
T/S Cluster: S−16.5−1
S797 (m/z=803.54631; rt=16.48865)
T/S Cluster: S−16.5−2
S799 (m/z=391.285253; rt=16.49152)
T/S Cluster: S−16.5−3
S798 (m/z=391.276192; rt=16.49125)
T/S Cluster: S−16.5−3
PCo1
PCo2
−0.6
−0.4
−0.2
0.0
0.2
0.4

S800 (m/z=413.267181; rt=16.49196)
T/S Cluster: S−16.5−4

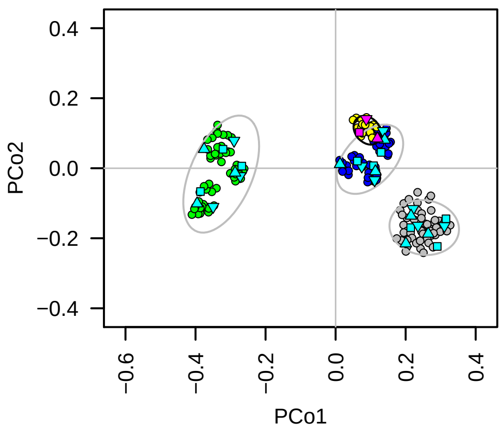

S801 (m/z=392.288689; rt=16.49223)
T/S Cluster: S−16.5−5

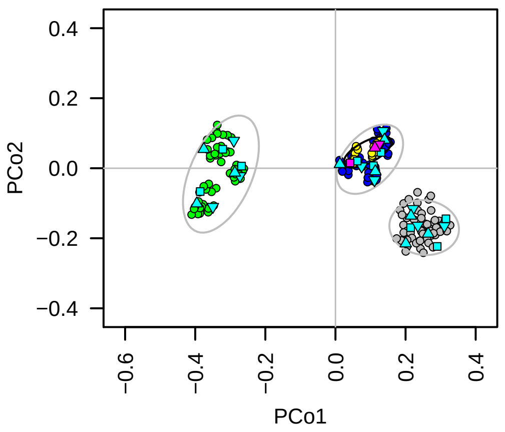

S802 (m/z=429.241105; rt=16.49238)
T/S Cluster: S−16.5−6

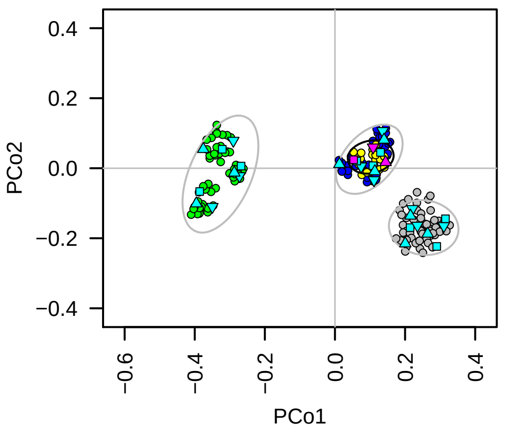

S803 (m/z=190.050041; rt=16.49253)
T/S Cluster: S−16.5−7

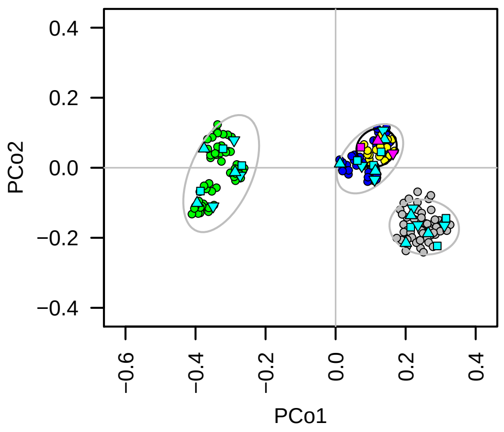

S805 (m/z=313.274143; rt=16.51683)
T/S Cluster: S−16.5−8

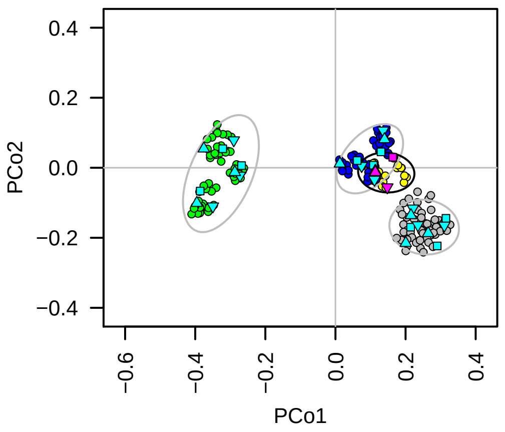

S806 (m/z=437.776845; rt=16.52788)
T/S Cluster: S−16.5−9

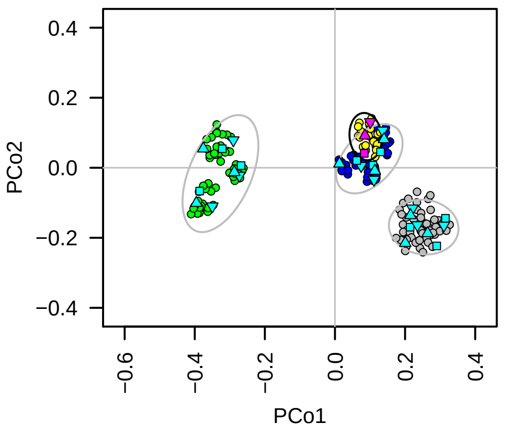

S837 (m/z=764.533664; rt=16.57449)
T/S Cluster: S−16.6−1

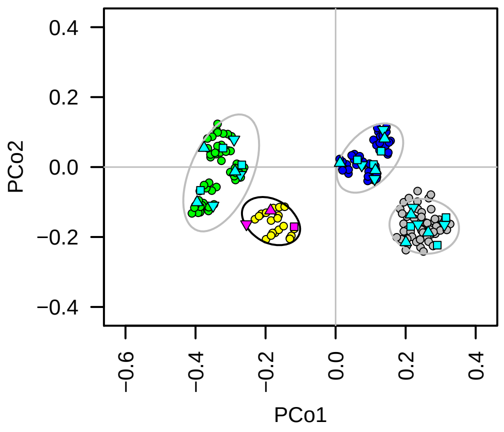

S831 (m/z=765.537908; rt=16.57431)
T/S Cluster: S−16.6−1

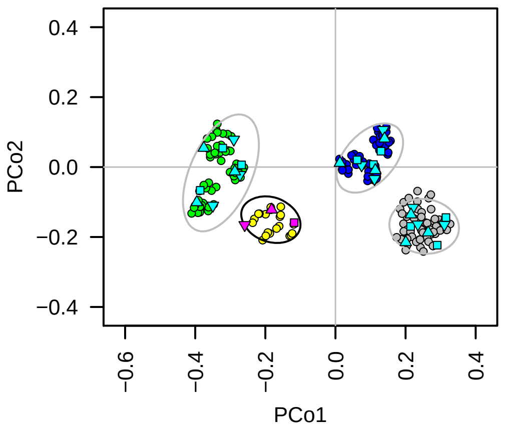

S832 (m/z=765.538344; rt=16.57432)
T/S Cluster: S−16.6−1

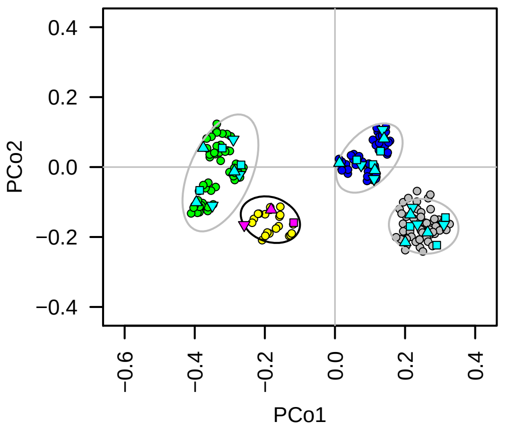

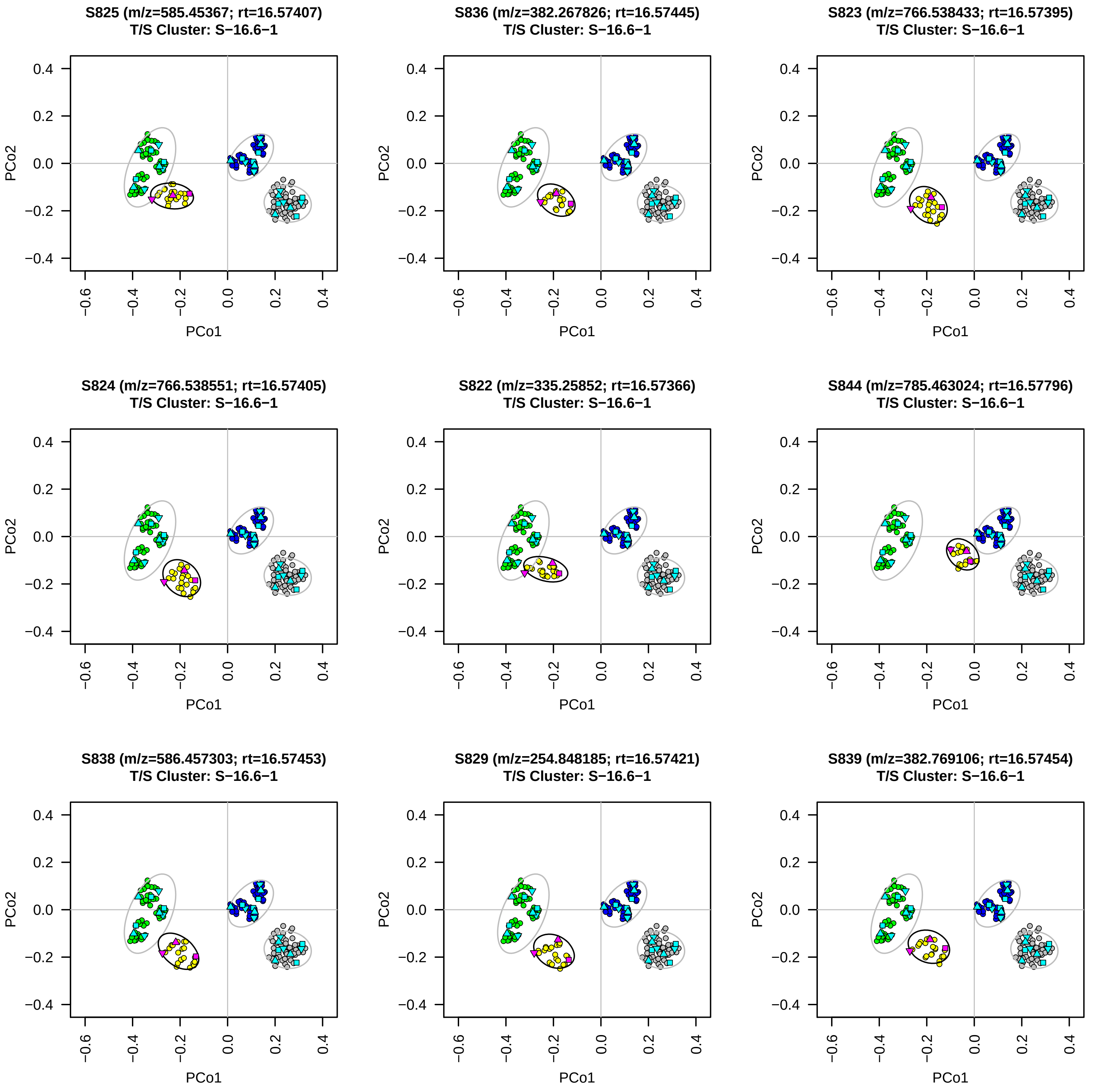
S825 (m/z=585.45367; rt=16.57407)
T/S Cluster: S−16.6−1
S836 (m/z=382.267826; rt=16.57445)
T/S Cluster: S−16.6−1
S823 (m/z=766.538433; rt=16.57395)
T/S Cluster: S−16.6−1
S824 (m/z=766.538551; rt=16.57405)
T/S Cluster: S−16.6−1
S822 (m/z=335.25852; rt=16.57366)
T/S Cluster: S−16.6−1
S844 (m/z=785.463024; rt=16.57796)
T/S Cluster: S−16.6−1
S838 (m/z=586.457303; rt=16.57453)
T/S Cluster: S−16.6−1
S829 (m/z=254.848185; rt=16.57421)
T/S Cluster: S−16.6−1
S839 (m/z=382.769106; rt=16.57454)
T/S Cluster: S−16.6−1
PCo1
PCo2
−0.6
−0.4
−0.2
0.0
0.2
0.4

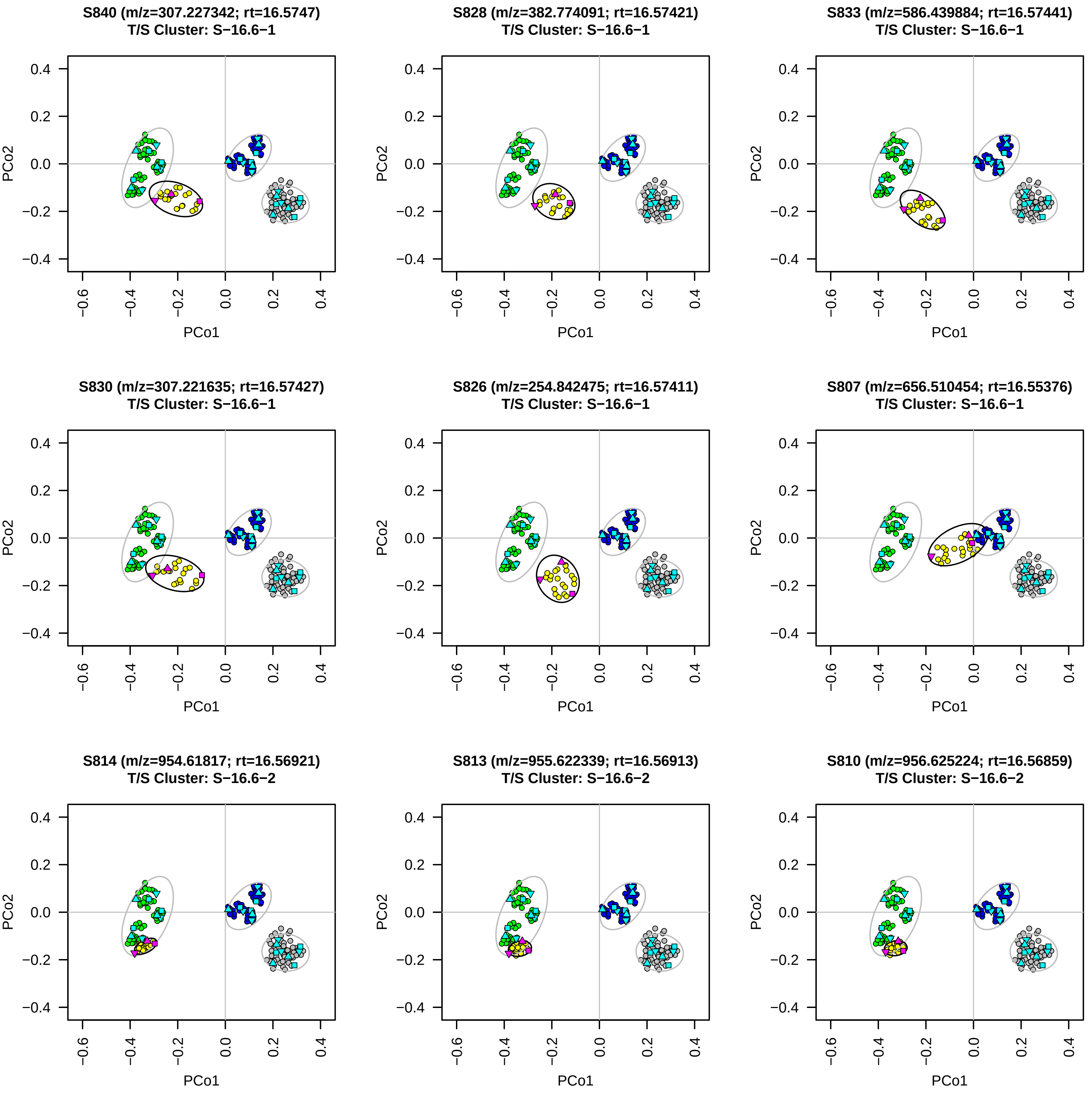
S840 (m/z=307.227342; rt=16.5747)
T/S Cluster: S−16.6−1
S828 (m/z=382.774091; rt=16.57421)
T/S Cluster: S−16.6−1
S833 (m/z=586.439884; rt=16.57441)
T/S Cluster: S−16.6−1
S830 (m/z=307.221635; rt=16.57427)
T/S Cluster: S−16.6−1
S826 (m/z=254.842475; rt=16.57411)
T/S Cluster: S−16.6−1
S807 (m/z=656.510454; rt=16.55376)
T/S Cluster: S−16.6−1
S814 (m/z=954.61817; rt=16.56921)
T/S Cluster: S−16.6−2
S813 (m/z=955.622339; rt=16.56913)
T/S Cluster: S−16.6−2
S810 (m/z=956.625224; rt=16.56859)
T/S Cluster: S−16.6−2
PCo1
PCo2
−0.6
−0.4
−0.2
0.0
0.2
0.4

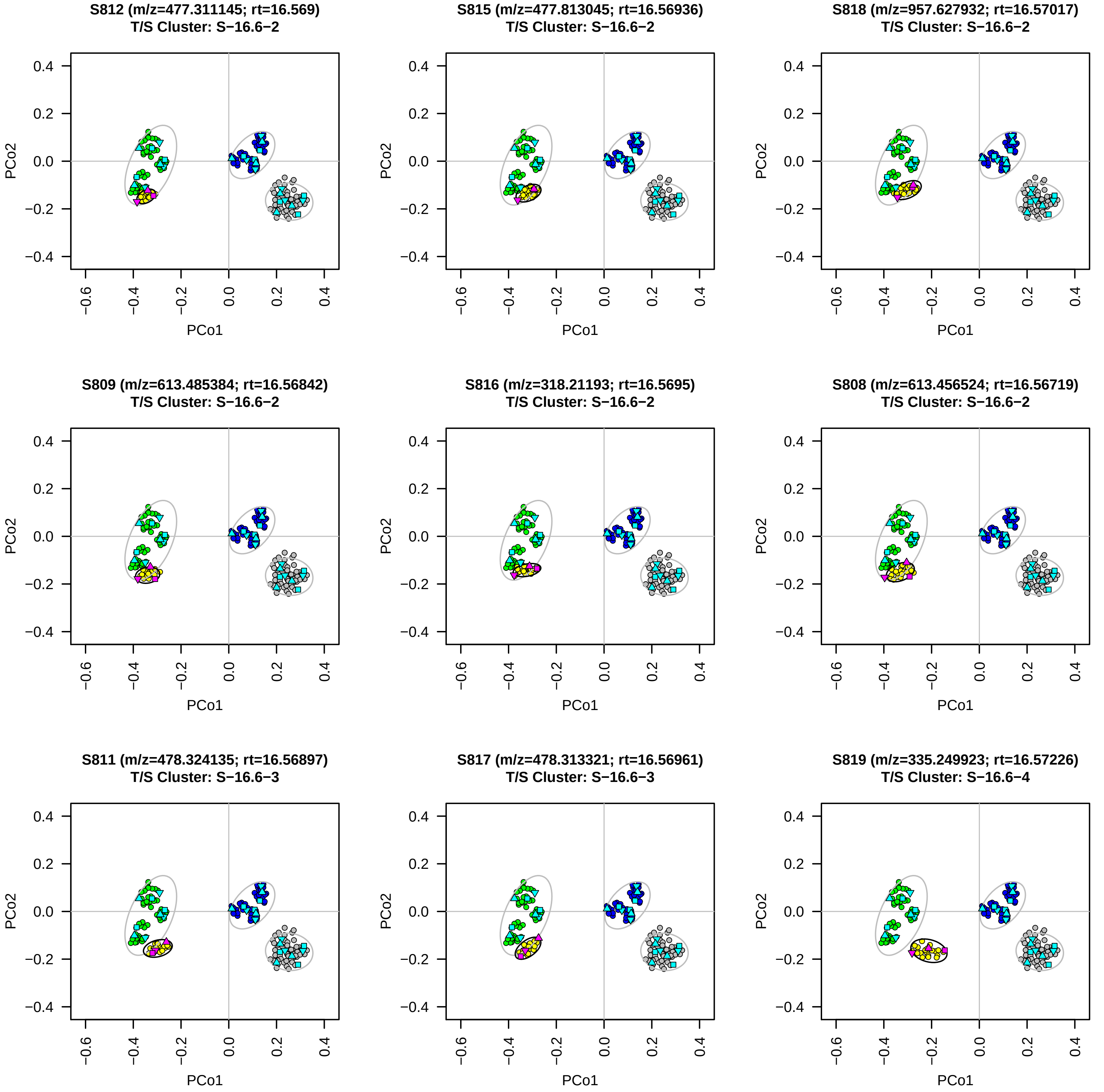
S812 (m/z=477.311145; rt=16.569)
T/S Cluster: S−16.6−2
S815 (m/z=477.813045; rt=16.56936)
T/S Cluster: S−16.6−2
S818 (m/z=957.627932; rt=16.57017)
T/S Cluster: S−16.6−2
S809 (m/z=613.485384; rt=16.56842)
T/S Cluster: S−16.6−2
S816 (m/z=318.21193; rt=16.5695)
T/S Cluster: S−16.6−2
S808 (m/z=613.456524; rt=16.56719)
T/S Cluster: S−16.6−2
S811 (m/z=478.324135; rt=16.56897)
T/S Cluster: S−16.6−3
S817 (m/z=478.313321; rt=16.56961)
T/S Cluster: S−16.6−3
S819 (m/z=335.249923; rt=16.57226)
T/S Cluster: S−16.6−4
PCo1
PCo2
0.4
0.2
0.0
−0.2
−0.4
−0.6

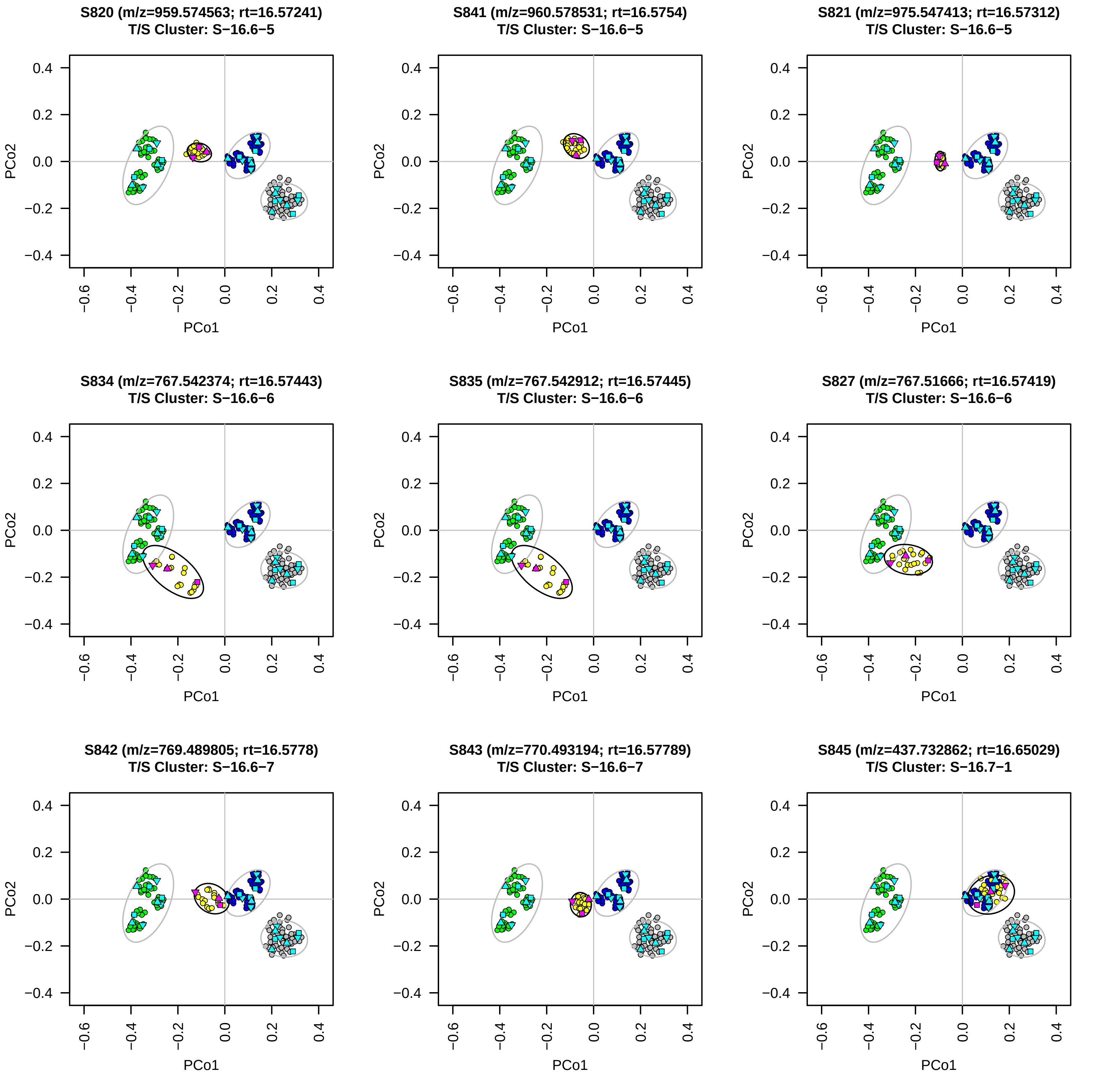
S820 (m/z=959.574563; rt=16.57241)
T/S Cluster: S−16.6−5
S841 (m/z=960.578531; rt=16.5754)
T/S Cluster: S−16.6−5
S821 (m/z=975.547413; rt=16.57312)
T/S Cluster: S−16.6−5
S834 (m/z=767.542374; rt=16.57443)
T/S Cluster: S−16.6−6
S835 (m/z=767.542912; rt=16.57445)
T/S Cluster: S−16.6−6
S827 (m/z=767.51666; rt=16.57419)
T/S Cluster: S−16.6−6
S842 (m/z=769.489805; rt=16.5778)
T/S Cluster: S−16.6−7
S843 (m/z=770.493194; rt=16.57789)
T/S Cluster: S−16.6−7
S845 (m/z=437.732862; rt=16.65029)
T/S Cluster: S−16.7−1
PCo1
PCo2
0.4
0.2
0.0
−0.2
−0.4
−0.6

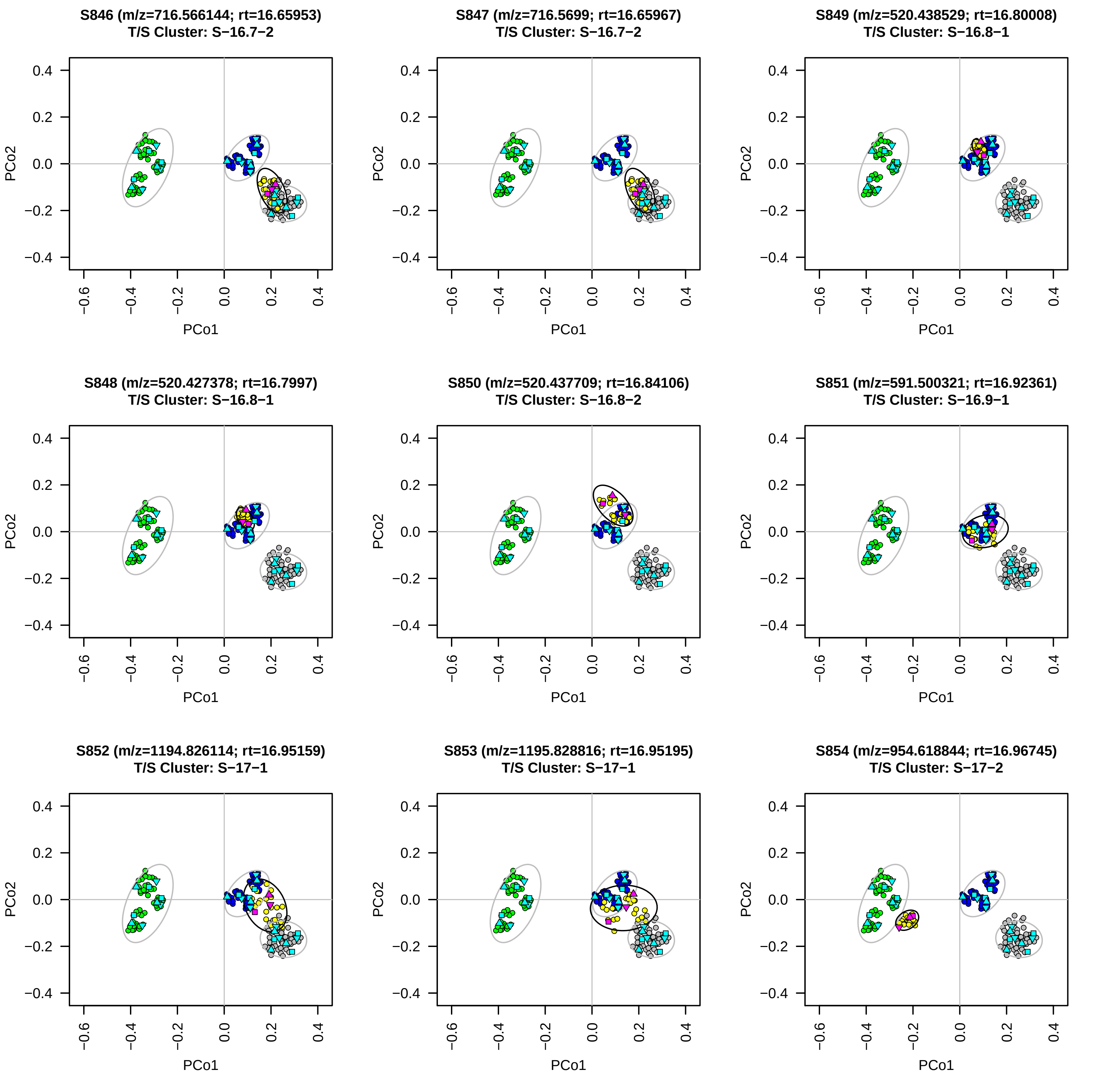
S846 (m/z=716.566144; rt=16.65953)
T/S Cluster: S−16.7−2
S847 (m/z=716.5699; rt=16.65967)
T/S Cluster: S−16.7−2
S849 (m/z=520.438529; rt=16.80008)
T/S Cluster: S−16.8−1
S848 (m/z=520.427378; rt=16.7997)
T/S Cluster: S−16.8−1
S850 (m/z=520.437709; rt=16.84106)
T/S Cluster: S−16.8−2
S851 (m/z=591.500321; rt=16.92361)
T/S Cluster: S−16.9−1
S852 (m/z=1194.826114; rt=16.95159)
T/S Cluster: S−17−1
S853 (m/z=1195.828816; rt=16.95195)
T/S Cluster: S−17−1
S854 (m/z=954.618844; rt=16.96745)
T/S Cluster: S−17−2
PCo1
PCo2
0.4
0.2
0.0
−0.2
−0.4
−0.6

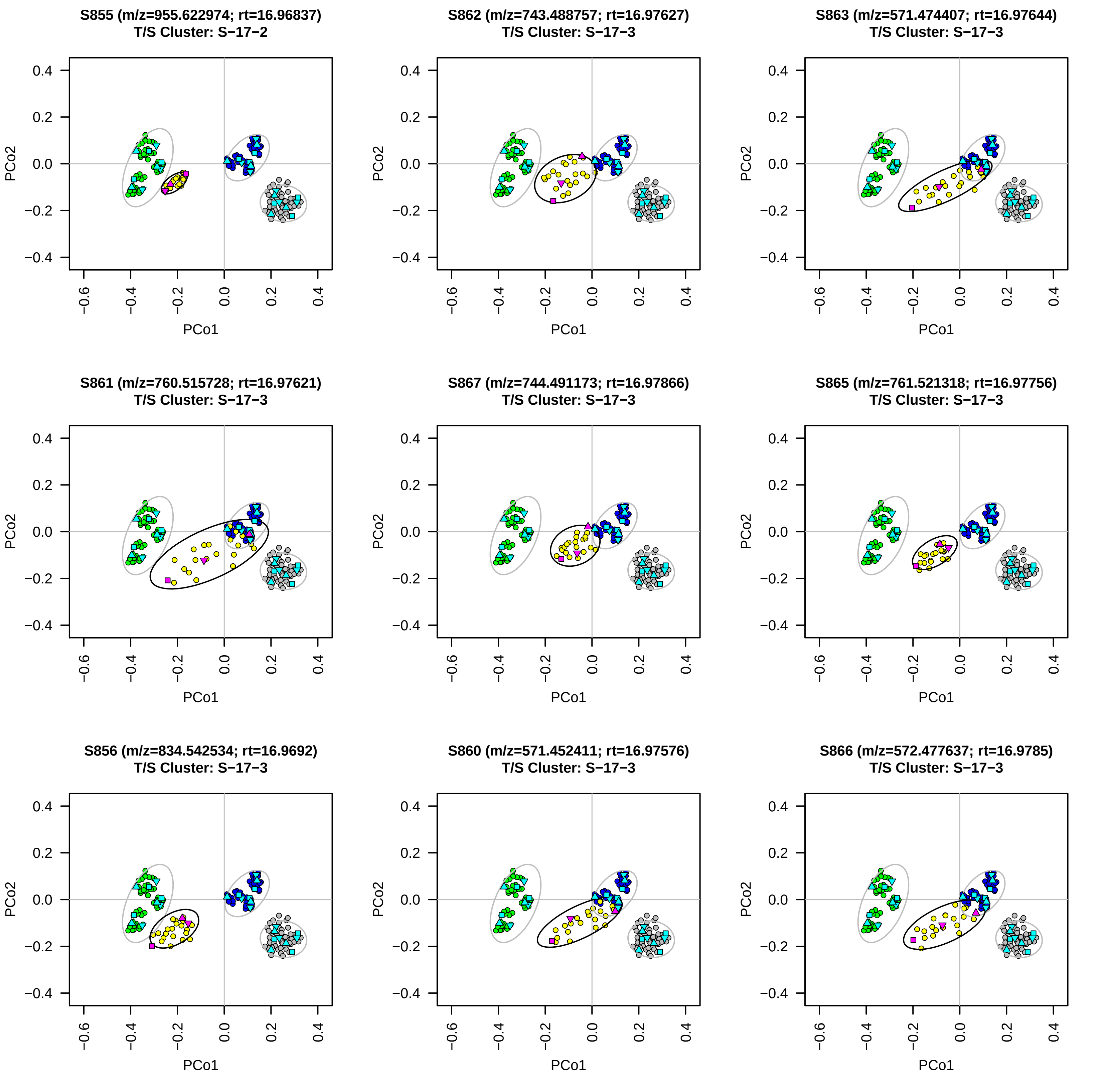
S855 (m/z=955.622974; rt=16.96837)
T/S Cluster: S−17−2
S862 (m/z=743.488757; rt=16.97627)
T/S Cluster: S−17−3
S863 (m/z=571.474407; rt=16.97644)
T/S Cluster: S−17−3
S861 (m/z=760.515728; rt=16.97621)
T/S Cluster: S−17−3
S867 (m/z=744.491173; rt=16.97866)
T/S Cluster: S−17−3
S865 (m/z=761.521318; rt=16.97756)
T/S Cluster: S−17−3
S856 (m/z=834.542534; rt=16.9692)
T/S Cluster: S−17−3
S860 (m/z=571.452411; rt=16.97576)
T/S Cluster: S−17−3
S866 (m/z=572.477637; rt=16.9785)
T/S Cluster: S−17−3
PCo1
PCo2

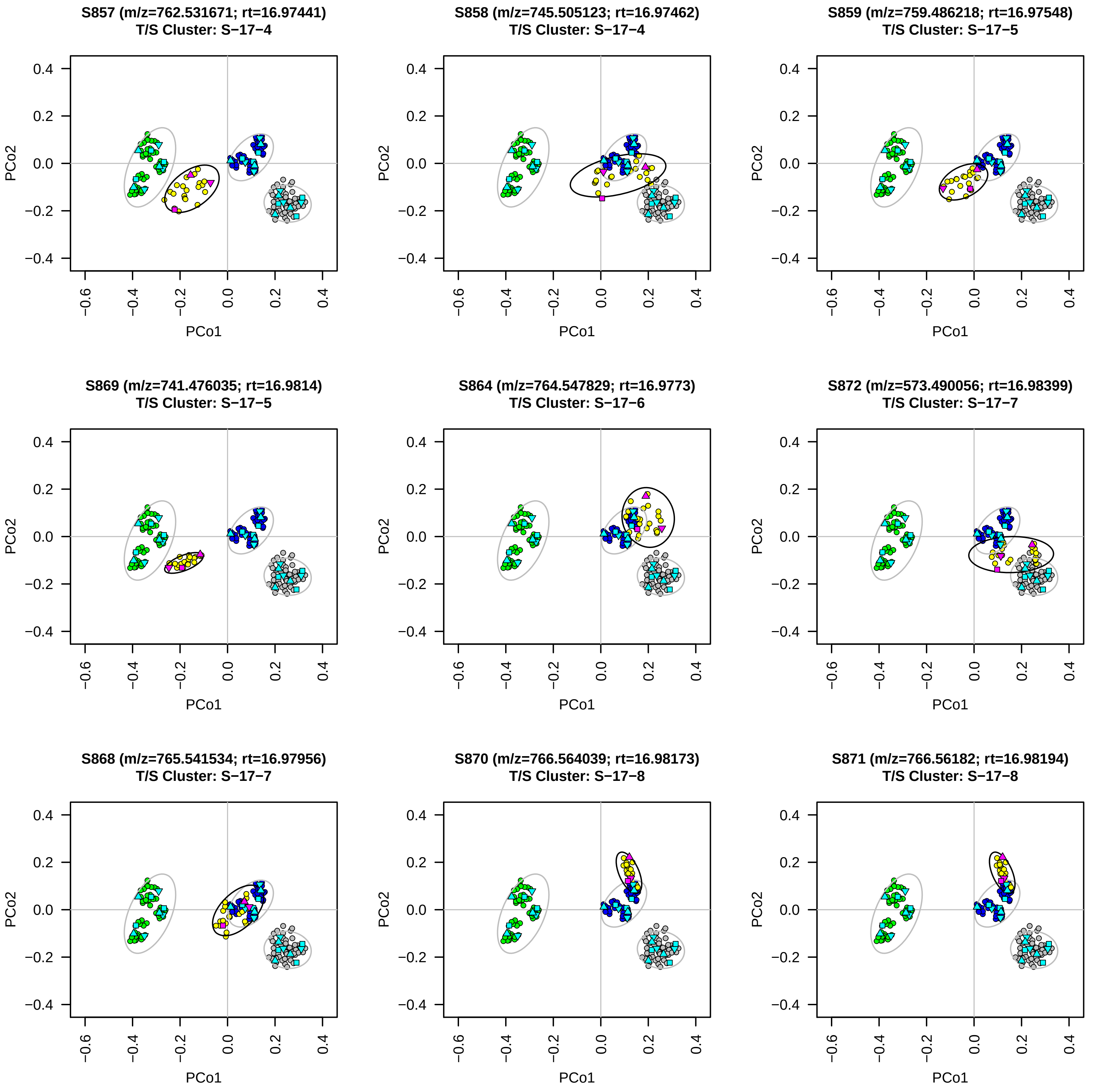
S857 (m/z=762.531671; rt=16.97441)
T/S Cluster: S−17−4
S858 (m/z=745.505123; rt=16.97462)
T/S Cluster: S−17−4
S859 (m/z=759.486218; rt=16.97548)
T/S Cluster: S−17−5
S869 (m/z=741.476035; rt=16.9814)
T/S Cluster: S−17−5
S864 (m/z=764.547829; rt=16.9773)
T/S Cluster: S−17−6
S872 (m/z=573.490056; rt=16.98399)
T/S Cluster: S−17−7
S868 (m/z=765.541534; rt=16.97956)
T/S Cluster: S−17−7
S870 (m/z=766.564039; rt=16.98173)
T/S Cluster: S−17−8
S871 (m/z=766.56182; rt=16.98194)
T/S Cluster: S−17−8
PCo1
PCo2
0.4
0.2
0.0
−0.2
−0.4
−0.6

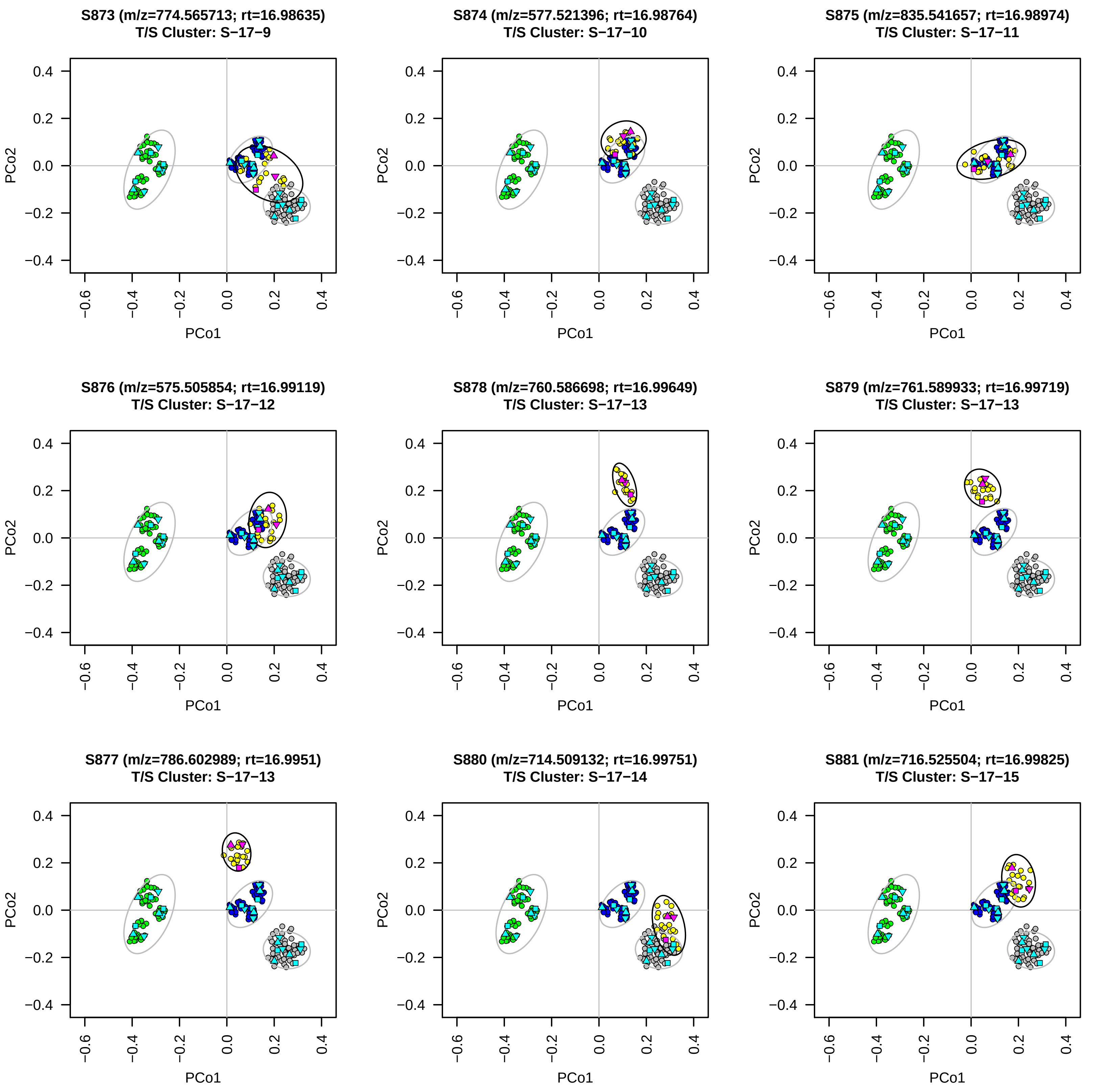
S873 (m/z=774.565713; rt=16.98635)
T/S Cluster: S−17−9
S874 (m/z=577.521396; rt=16.98764)
T/S Cluster: S−17−10
S875 (m/z=835.541657; rt=16.98974)
T/S Cluster: S−17−11
S876 (m/z=575.505854; rt=16.99119)
T/S Cluster: S−17−12
S878 (m/z=760.586698; rt=16.99649)
T/S Cluster: S−17−13
S879 (m/z=761.589933; rt=16.99719)
T/S Cluster: S−17−13
S877 (m/z=786.602989; rt=16.9951)
T/S Cluster: S−17−13
S880 (m/z=714.509132; rt=16.99751)
T/S Cluster: S−17−14
S881 (m/z=716.525504; rt=16.99825)
T/S Cluster: S−17−15
PCo1
PCo2
−0.6
−0.4
−0.2
0.0
0.2
0.4

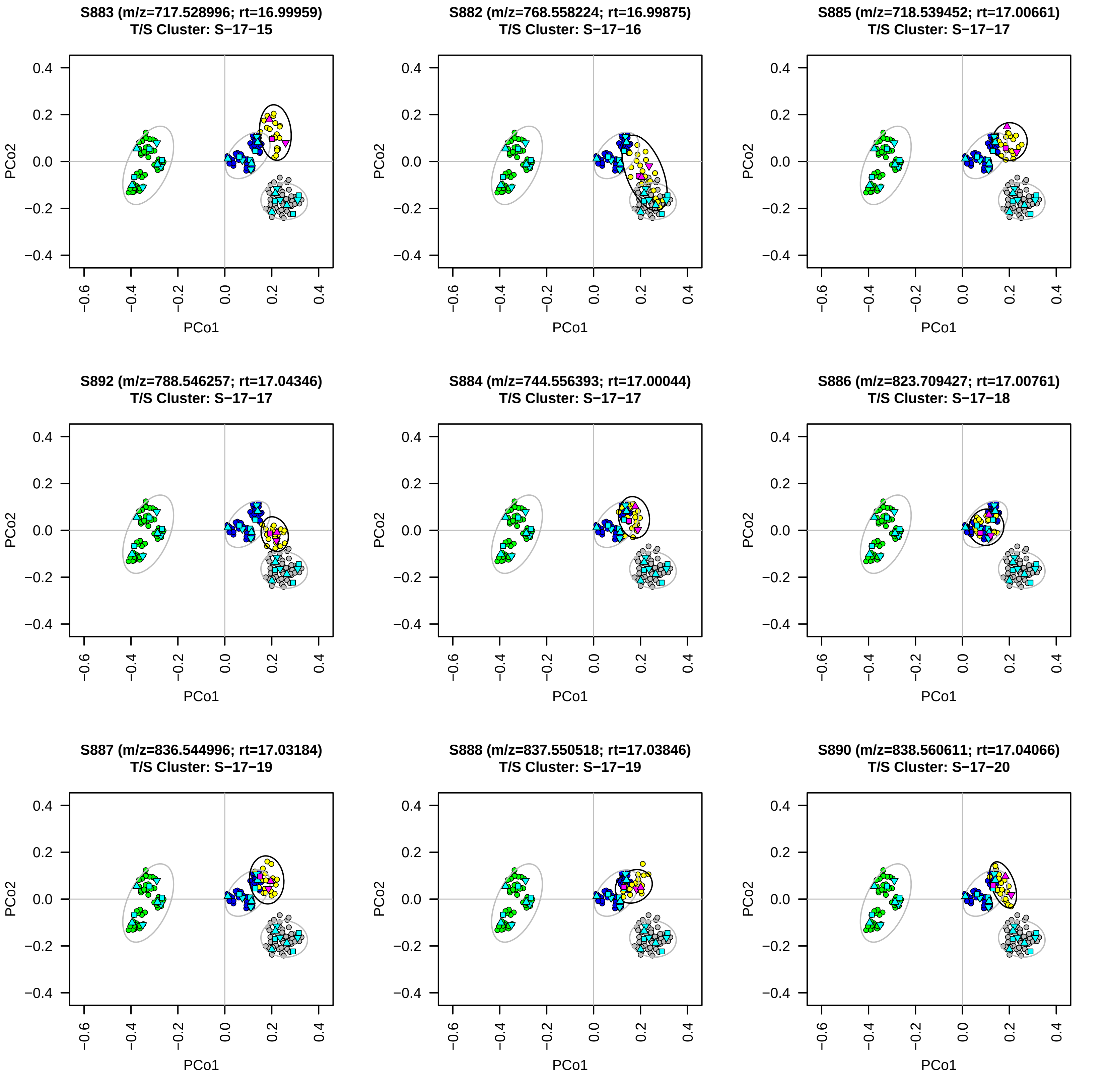

S883 (m/z=717.528996; rt=16.99959)
T/S Cluster: S−17−15
S882 (m/z=768.558224; rt=16.99875)
T/S Cluster: S−17−16
S885 (m/z=718.539452; rt=17.00661)
T/S Cluster: S−17−17
S892 (m/z=788.546257; rt=17.04346)
T/S Cluster: S−17−17
S884 (m/z=744.556393; rt=17.00044)
T/S Cluster: S−17−17
S886 (m/z=823.709427; rt=17.00761)
T/S Cluster: S−17−18
S887 (m/z=836.544996; rt=17.03184)
T/S Cluster: S−17−19
S888 (m/z=837.550518; rt=17.03846)
T/S Cluster: S−17−19
S890 (m/z=838.560611; rt=17.04066)
T/S Cluster: S−17−20
PCo1
PCo2
0.4
0.2
0.0
−0.2
−0.4
−0.6

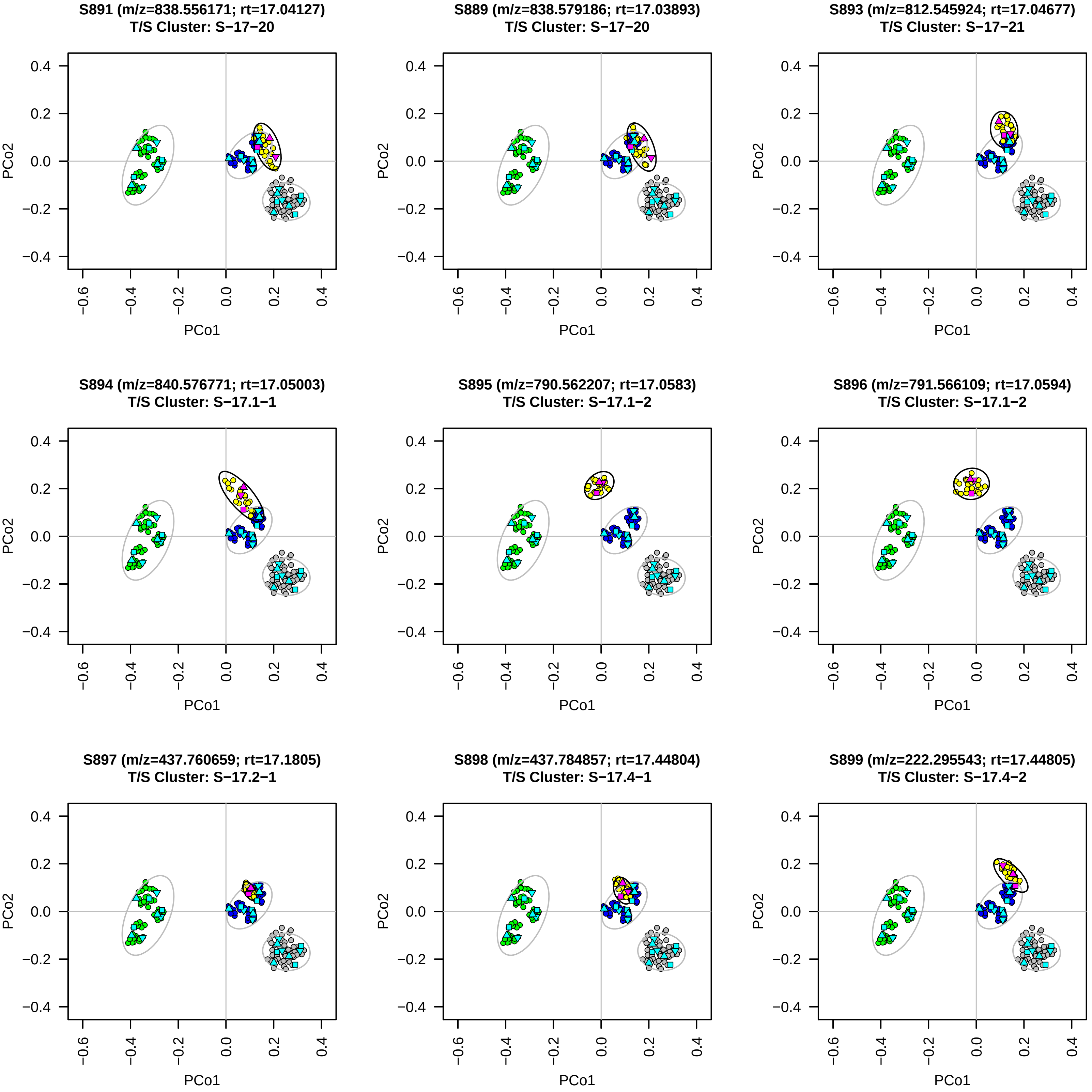
S891 (m/z=838.556171; rt=17.04127)
T/S Cluster: S−17−20
S889 (m/z=838.579186; rt=17.03893)
T/S Cluster: S−17−20
S893 (m/z=812.545924; rt=17.04677)
T/S Cluster: S−17−21
S894 (m/z=840.576771; rt=17.05003)
T/S Cluster: S−17.1−1
S895 (m/z=790.562207; rt=17.0583)
T/S Cluster: S−17.1−2
S896 (m/z=791.566109; rt=17.0594)
T/S Cluster: S−17.1−2
S897 (m/z=437.760659; rt=17.1805)
T/S Cluster: S−17.2−1
S898 (m/z=437.784857; rt=17.44804)
T/S Cluster: S−17.4−1
S899 (m/z=222.295543; rt=17.44805)
T/S Cluster: S−17.4−2
PCo1
PCo2
0.4
0.2
0.0
−0.2
−0.4
−0.6

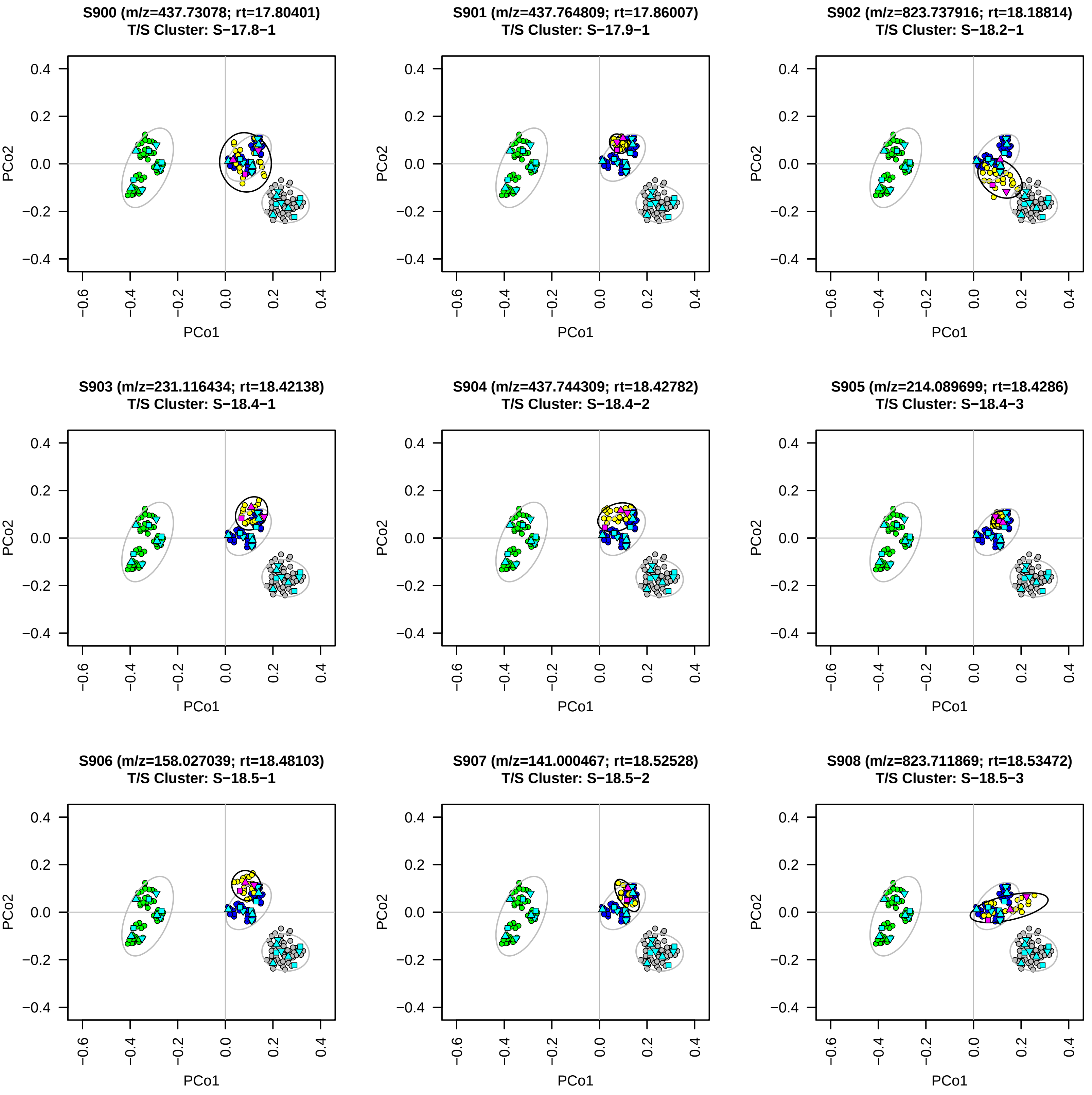
S900 (m/z=437.73078; rt=17.80401)
T/S Cluster: S−17.8−1
S901 (m/z=437.764809; rt=17.86007)
T/S Cluster: S−17.9−1
S902 (m/z=823.737916; rt=18.18814)
T/S Cluster: S−18.2−1
S903 (m/z=231.116434; rt=18.42138)
T/S Cluster: S−18.4−1
S904 (m/z=437.744309; rt=18.42782)
T/S Cluster: S−18.4−2
S905 (m/z=214.089699; rt=18.4286)
T/S Cluster: S−18.4−3
S906 (m/z=158.027039; rt=18.48103)
T/S Cluster: S−18.5−1
S907 (m/z=141.000467; rt=18.52528)
T/S Cluster: S−18.5−2
S908 (m/z=823.711869; rt=18.53472)
T/S Cluster: S−18.5−3
PCo1
PCo2
−0.6
−0.4
−0.2
0.0
0.2
0.4

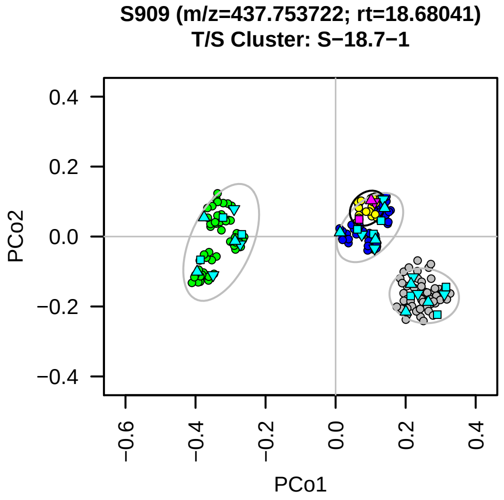
S909 (m/z=437.753722; rt=18.68041)
T/S Cluster: S−18.7−1
PCo2
PCo1
0.4
0.2
0.0
−0.2
−0.4
−0.6
−0.4
−0.2
0.0
0.2
0.4

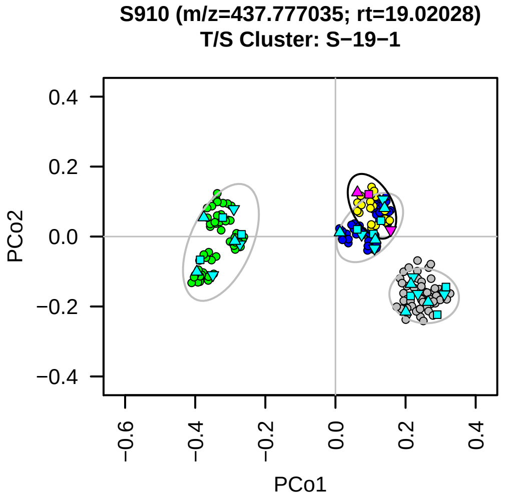
S910 (m/z=437.777035; rt=19.02028)
T/S Cluster: S−19−1
PCo2
PCo1
0.4
0.2
0.0
−0.2
−0.4
−0.6
−0.4
−0.2
0.0
0.2
0.4

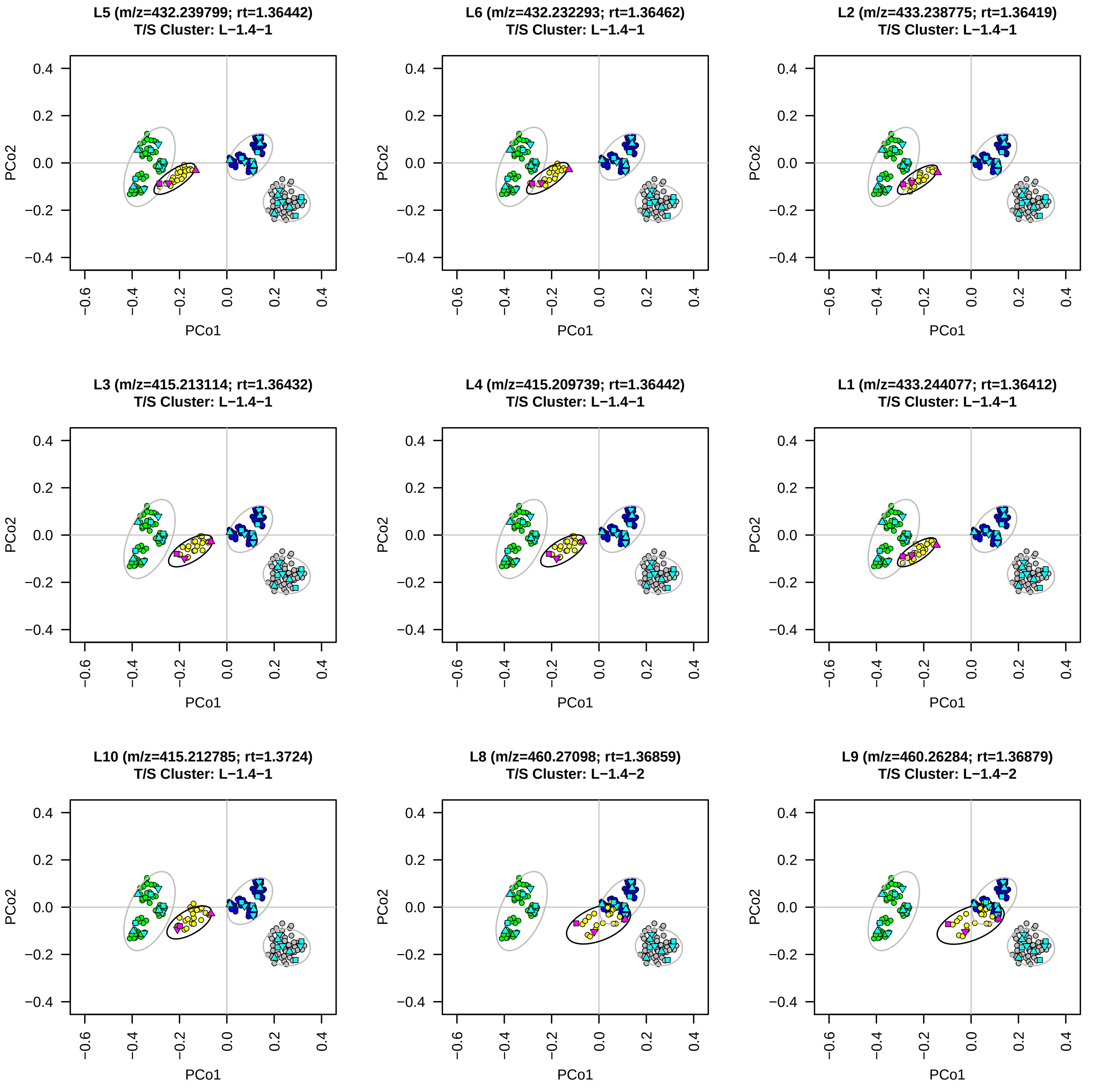
L5 (m/z=432.239799; rt=1.36442)
T/S Cluster: L−1.4−1
L6 (m/z=432.232293; rt=1.36462)
T/S Cluster: L−1.4−1
L2 (m/z=433.238775; rt=1.36419)
T/S Cluster: L−1.4−1
L3 (m/z=415.213114; rt=1.36432)
T/S Cluster: L−1.4−1
L4 (m/z=415.209739; rt=1.36442)
T/S Cluster: L−1.4−1
L1 (m/z=433.244077; rt=1.36412)
T/S Cluster: L−1.4−1
L10 (m/z=415.212785; rt=1.3724)
T/S Cluster: L−1.4−1
L8 (m/z=460.27098; rt=1.36859)
T/S Cluster: L−1.4−2
L9 (m/z=460.26284; rt=1.36879)
T/S Cluster: L−1.4−2
PCo1
PCo2

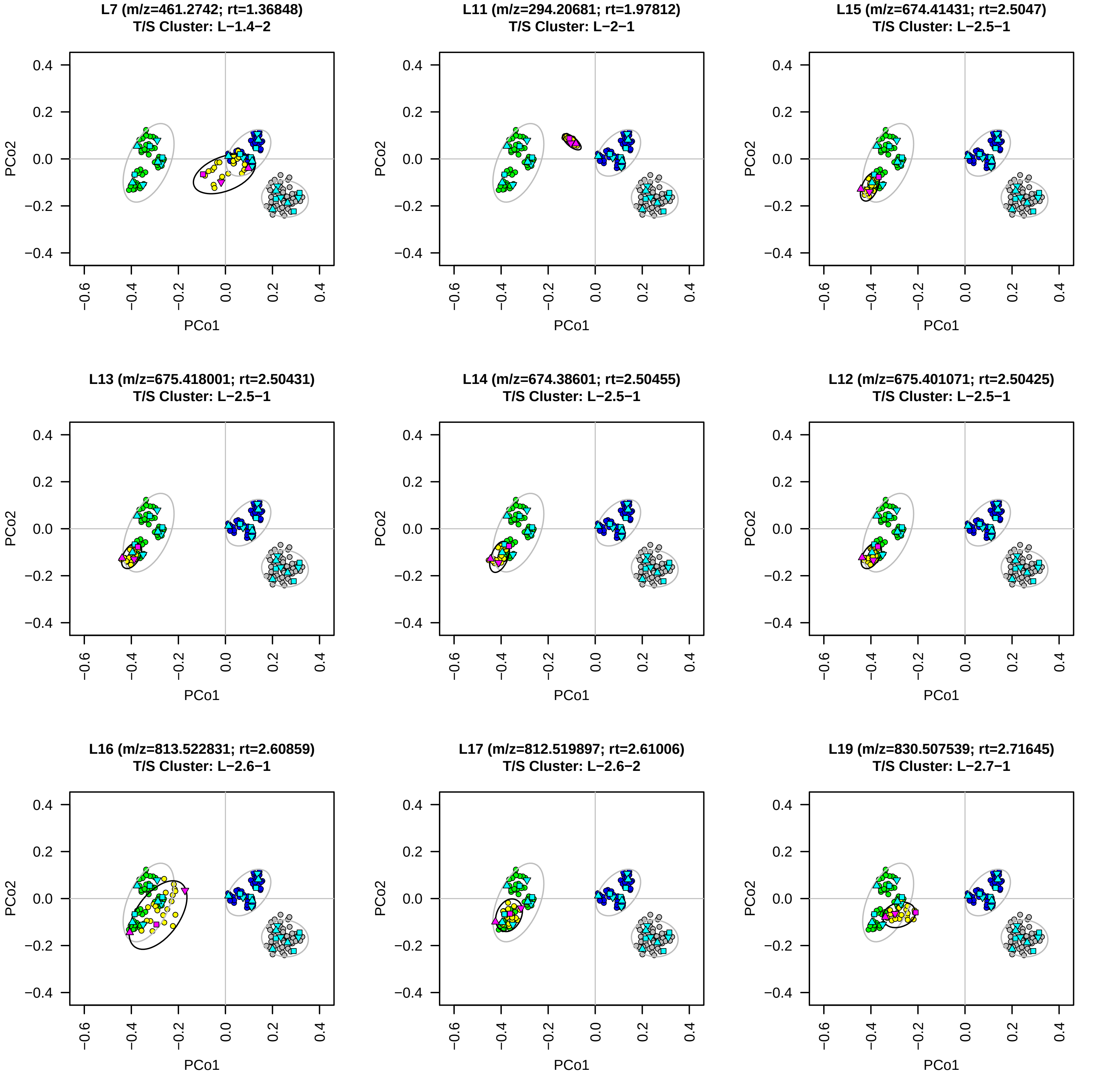
L7 (m/z=461.2742; rt=1.36848)
T/S Cluster: L−1.4−2
L11 (m/z=294.20681; rt=1.97812)
T/S Cluster: L−2−1
L15 (m/z=674.41431; rt=2.5047)
T/S Cluster: L−2.5−1
L13 (m/z=675.418001; rt=2.50431)
T/S Cluster: L−2.5−1
L14 (m/z=674.38601; rt=2.50455)
T/S Cluster: L−2.5−1
L12 (m/z=675.401071; rt=2.50425)
T/S Cluster: L−2.5−1
L16 (m/z=813.522831; rt=2.60859)
T/S Cluster: L−2.6−1
L17 (m/z=812.519897; rt=2.61006)
T/S Cluster: L−2.6−2
L19 (m/z=830.507539; rt=2.71645)
T/S Cluster: L−2.7−1
PCo1
PCo2
−0.6
−0.4
−0.2
0.0
0.2
0.4

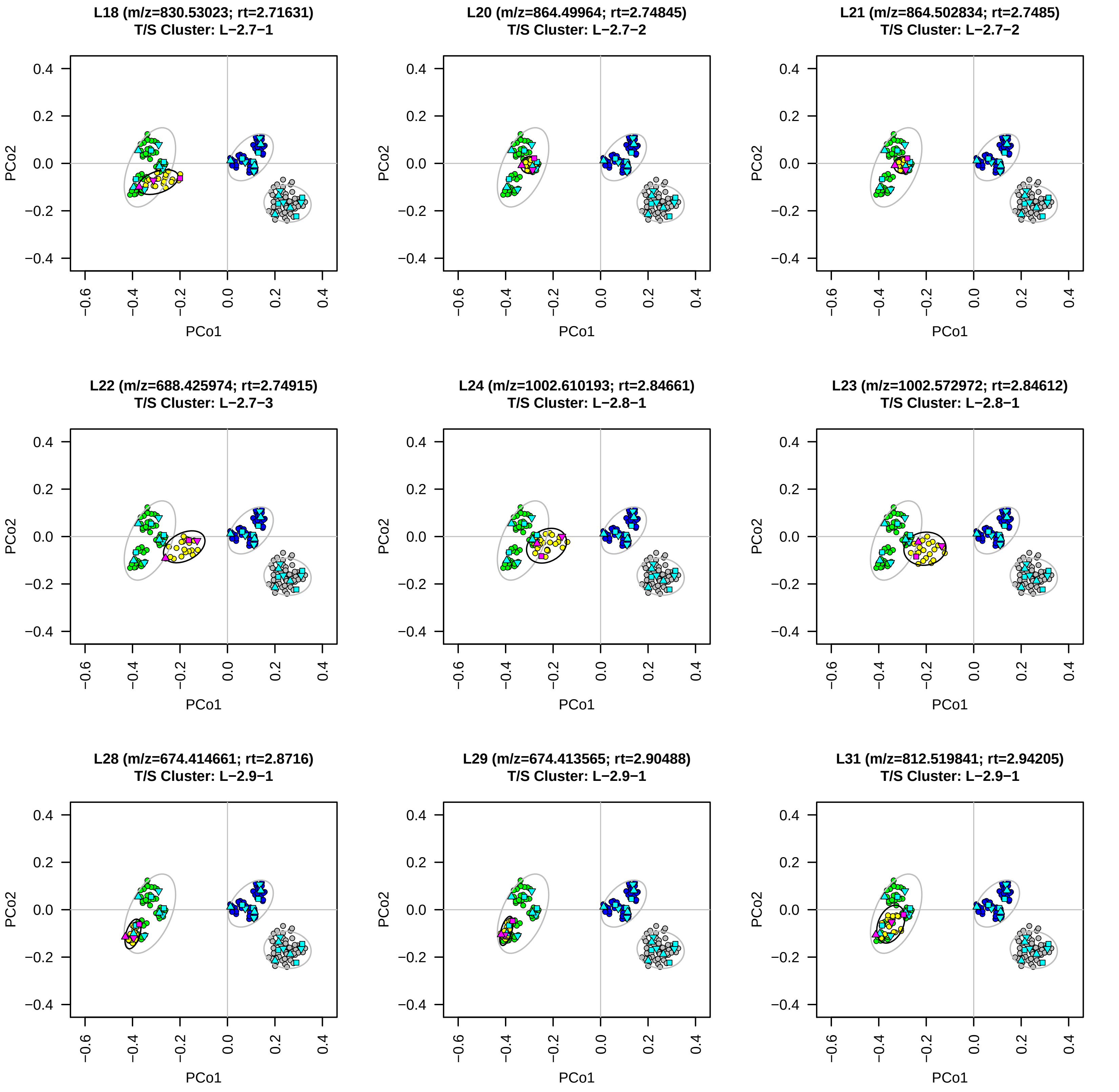
L18 (m/z=830.53023; rt=2.71631)
T/S Cluster: L−2.7−1
L20 (m/z=864.49964; rt=2.74845)
T/S Cluster: L−2.7−2
L21 (m/z=864.502834; rt=2.7485)
T/S Cluster: L−2.7−2
L22 (m/z=688.425974; rt=2.74915)
T/S Cluster: L−2.7−3
L24 (m/z=1002.610193; rt=2.84661)
T/S Cluster: L−2.8−1
L23 (m/z=1002.572972; rt=2.84612)
T/S Cluster: L−2.8−1
L28 (m/z=674.414661; rt=2.8716)
T/S Cluster: L−2.9−1
L29 (m/z=674.413565; rt=2.90488)
T/S Cluster: L−2.9−1
L31 (m/z=812.519841; rt=2.94205)
T/S Cluster: L−2.9−1
PCo1
PCo2
−0.6
−0.4
−0.2
0.0
0.2
0.4

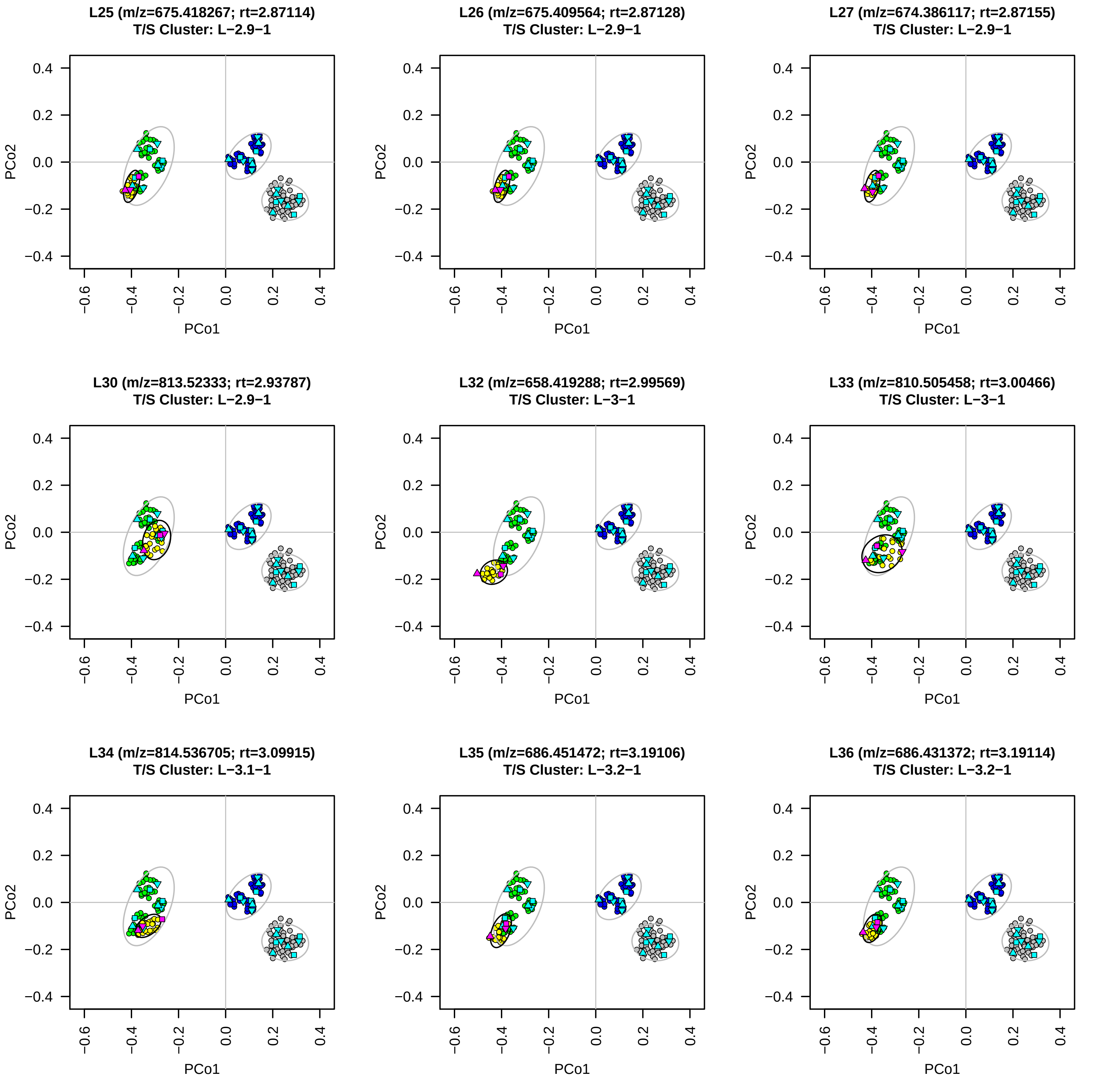
L25 (m/z=675.418267; rt=2.87114)
T/S Cluster: L−2.9−1
L26 (m/z=675.409564; rt=2.87128)
T/S Cluster: L−2.9−1
L27 (m/z=674.386117; rt=2.87155)
T/S Cluster: L−2.9−1
L30 (m/z=813.52333; rt=2.93787)
T/S Cluster: L−2.9−1
L32 (m/z=658.419288; rt=2.99569)
T/S Cluster: L−3−1
L33 (m/z=810.505458; rt=3.00466)
T/S Cluster: L−3−1
L34 (m/z=814.536705; rt=3.09915)
T/S Cluster: L−3.1−1
L35 (m/z=686.451472; rt=3.19106)
T/S Cluster: L−3.2−1
L36 (m/z=686.431372; rt=3.19114)
T/S Cluster: L−3.2−1
PCo1
PCo2
0.4
0.2
0.0
−0.2
−0.4
−0.6

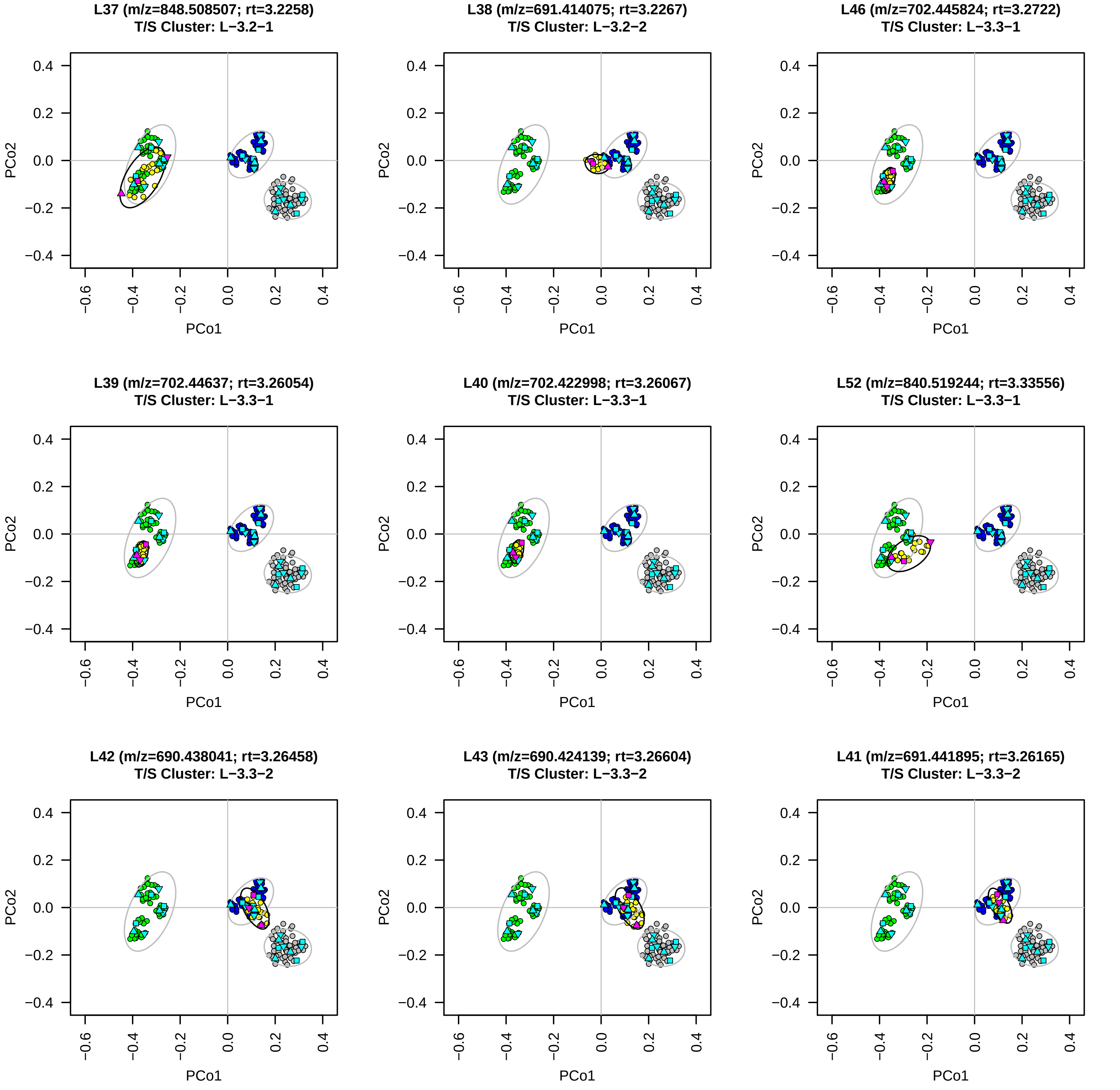

L37 (m/z=848.508507; rt=3.2258)
T/S Cluster: L−3.2−1
L38 (m/z=691.414075; rt=3.2267)
T/S Cluster: L−3.2−2
L46 (m/z=702.445824; rt=3.2722)
T/S Cluster: L−3.3−1
L39 (m/z=702.44637; rt=3.26054)
T/S Cluster: L−3.3−1
L40 (m/z=702.422998; rt=3.26067)
T/S Cluster: L−3.3−1
L52 (m/z=840.519244; rt=3.33556)
T/S Cluster: L−3.3−1
L42 (m/z=690.438041; rt=3.26458)
T/S Cluster: L−3.3−2
L43 (m/z=690.424139; rt=3.26604)
T/S Cluster: L−3.3−2
L41 (m/z=691.441895; rt=3.26165)
T/S Cluster: L−3.3−2
PCo1
PCo2
−0.6
−0.4
−0.2
0.0
0.2
0.4

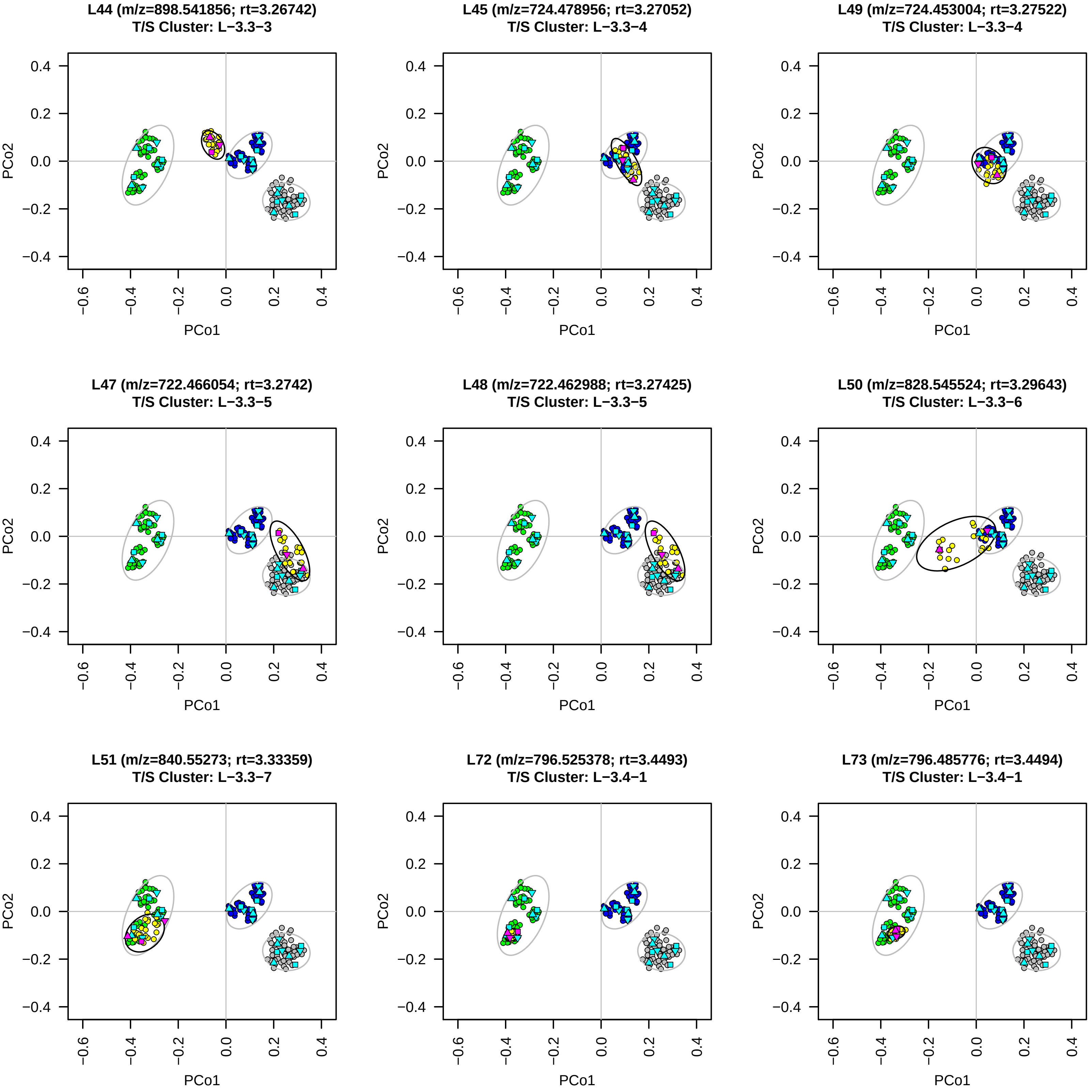

L44 (m/z=898.541856; rt=3.26742)
T/S Cluster: L−3.3−3
L45 (m/z=724.478956; rt=3.27052)
T/S Cluster: L−3.3−4
L49 (m/z=724.453004; rt=3.27522)
T/S Cluster: L−3.3−4
L47 (m/z=722.466054; rt=3.2742)
T/S Cluster: L−3.3−5
L48 (m/z=722.462988; rt=3.27425)
T/S Cluster: L−3.3−5
L50 (m/z=828.545524; rt=3.29643)
T/S Cluster: L−3.3−6
L51 (m/z=840.55273; rt=3.33359)
T/S Cluster: L−3.3−7
L72 (m/z=796.525378; rt=3.4493)
T/S Cluster: L−3.4−1
L73 (m/z=796.485776; rt=3.4494)
T/S Cluster: L−3.4−1
PCo2
0.4
0.2
0.0
−0.2
−0.4
PCo1
−0.6
−0.4
−0.2
0.0
0.2
0.4

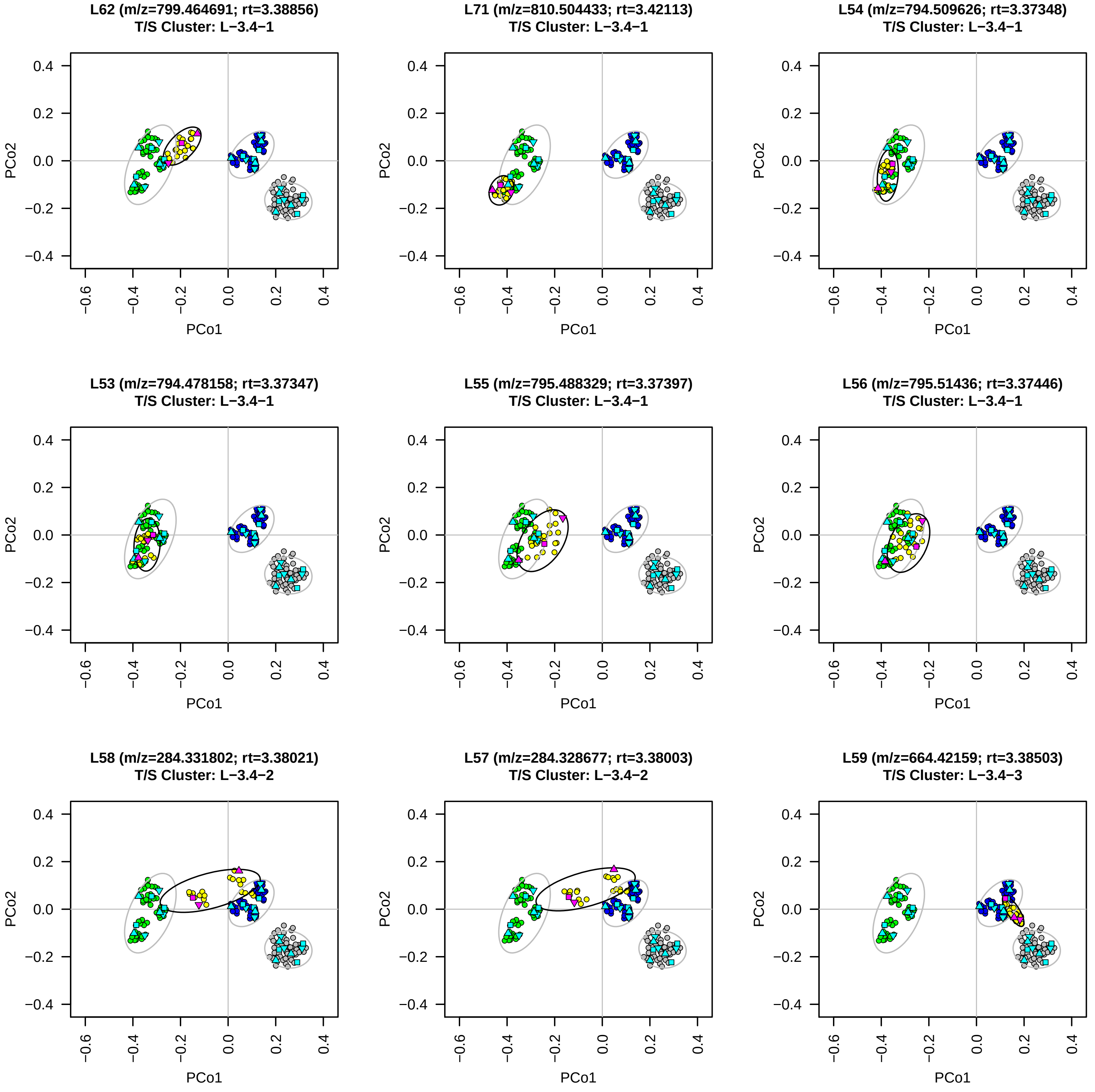
L62 (m/z=799.464691; rt=3.38856)
T/S Cluster: L−3.4−1
L71 (m/z=810.504433; rt=3.42113)
T/S Cluster: L−3.4−1
L54 (m/z=794.509626; rt=3.37348)
T/S Cluster: L−3.4−1
L53 (m/z=794.478158; rt=3.37347)
T/S Cluster: L−3.4−1
L55 (m/z=795.488329; rt=3.37397)
T/S Cluster: L−3.4−1
L56 (m/z=795.51436; rt=3.37446)
T/S Cluster: L−3.4−1
L58 (m/z=284.331802; rt=3.38021)
T/S Cluster: L−3.4−2
L57 (m/z=284.328677; rt=3.38003)
T/S Cluster: L−3.4−2
L59 (m/z=664.42159; rt=3.38503)
T/S Cluster: L−3.4−3
PCo1
PCo2
−0.6
−0.4
−0.2
0.0
0.2
0.4

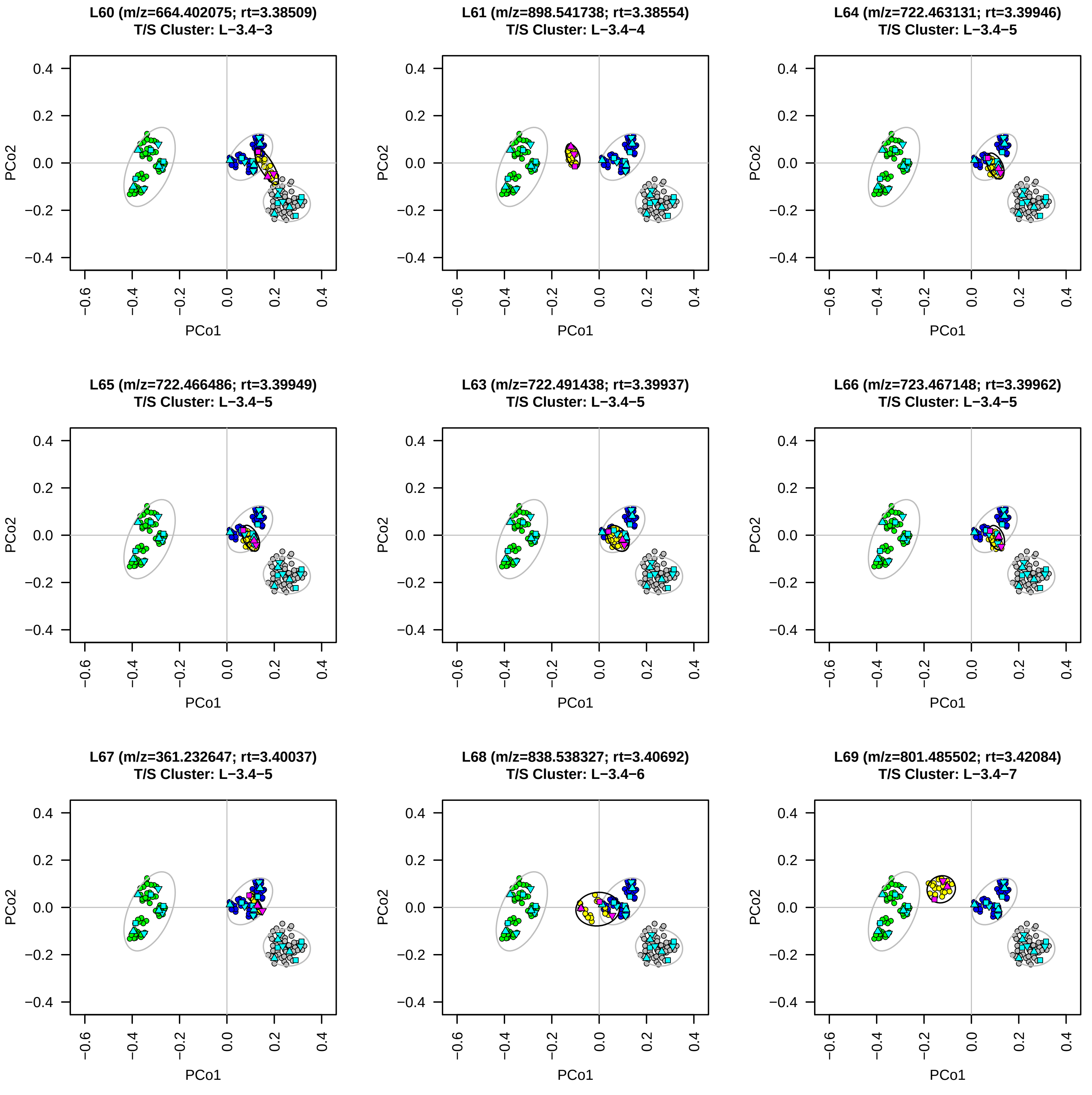

L60 (m/z=664.402075; rt=3.38509)
T/S Cluster: L−3.4−3
L61 (m/z=898.541738; rt=3.38554)
T/S Cluster: L−3.4−4
L64 (m/z=722.463131; rt=3.39946)
T/S Cluster: L−3.4−5
L65 (m/z=722.466486; rt=3.39949)
T/S Cluster: L−3.4−5
L63 (m/z=722.491438; rt=3.39937)
T/S Cluster: L−3.4−5
L66 (m/z=723.467148; rt=3.39962)
T/S Cluster: L−3.4−5
L67 (m/z=361.232647; rt=3.40037)
T/S Cluster: L−3.4−5
L68 (m/z=838.538327; rt=3.40692)
T/S Cluster: L−3.4−6
L69 (m/z=801.485502; rt=3.42084)
T/S Cluster: L−3.4−7
PCo1
PCo2

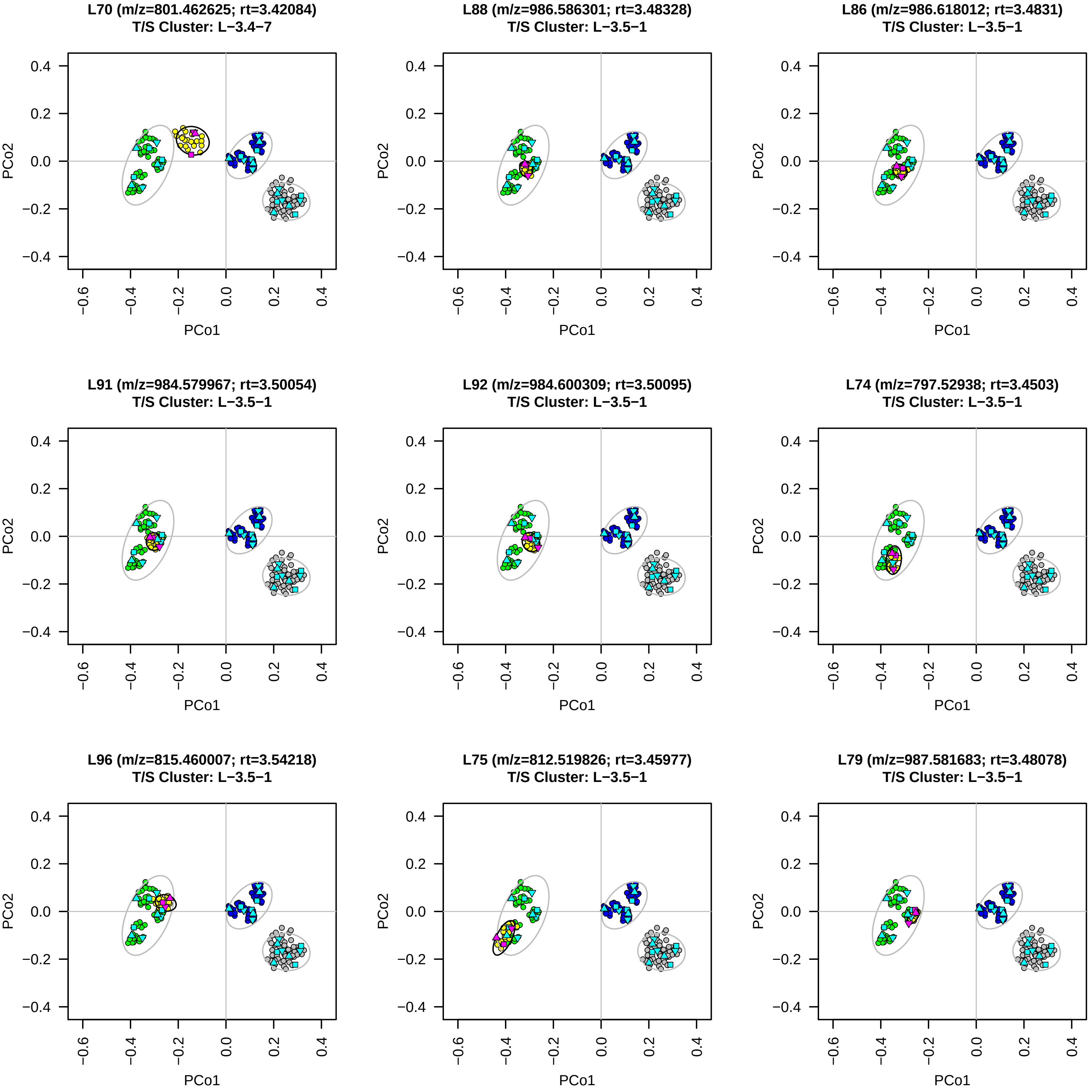
L70 (m/z=801.462625; rt=3.42084)
T/S Cluster: L−3.4−7
L88 (m/z=986.586301; rt=3.48328)
T/S Cluster: L−3.5−1
L86 (m/z=986.618012; rt=3.4831)
T/S Cluster: L−3.5−1
L91 (m/z=984.579967; rt=3.50054)
T/S Cluster: L−3.5−1
L92 (m/z=984.600309; rt=3.50095)
T/S Cluster: L−3.5−1
L74 (m/z=797.52938; rt=3.4503)
T/S Cluster: L−3.5−1
L96 (m/z=815.460007; rt=3.54218)
T/S Cluster: L−3.5−1
L75 (m/z=812.519826; rt=3.45977)
T/S Cluster: L−3.5−1
L79 (m/z=987.581683; rt=3.48078)
T/S Cluster: L−3.5−1
PCo1
PCo2

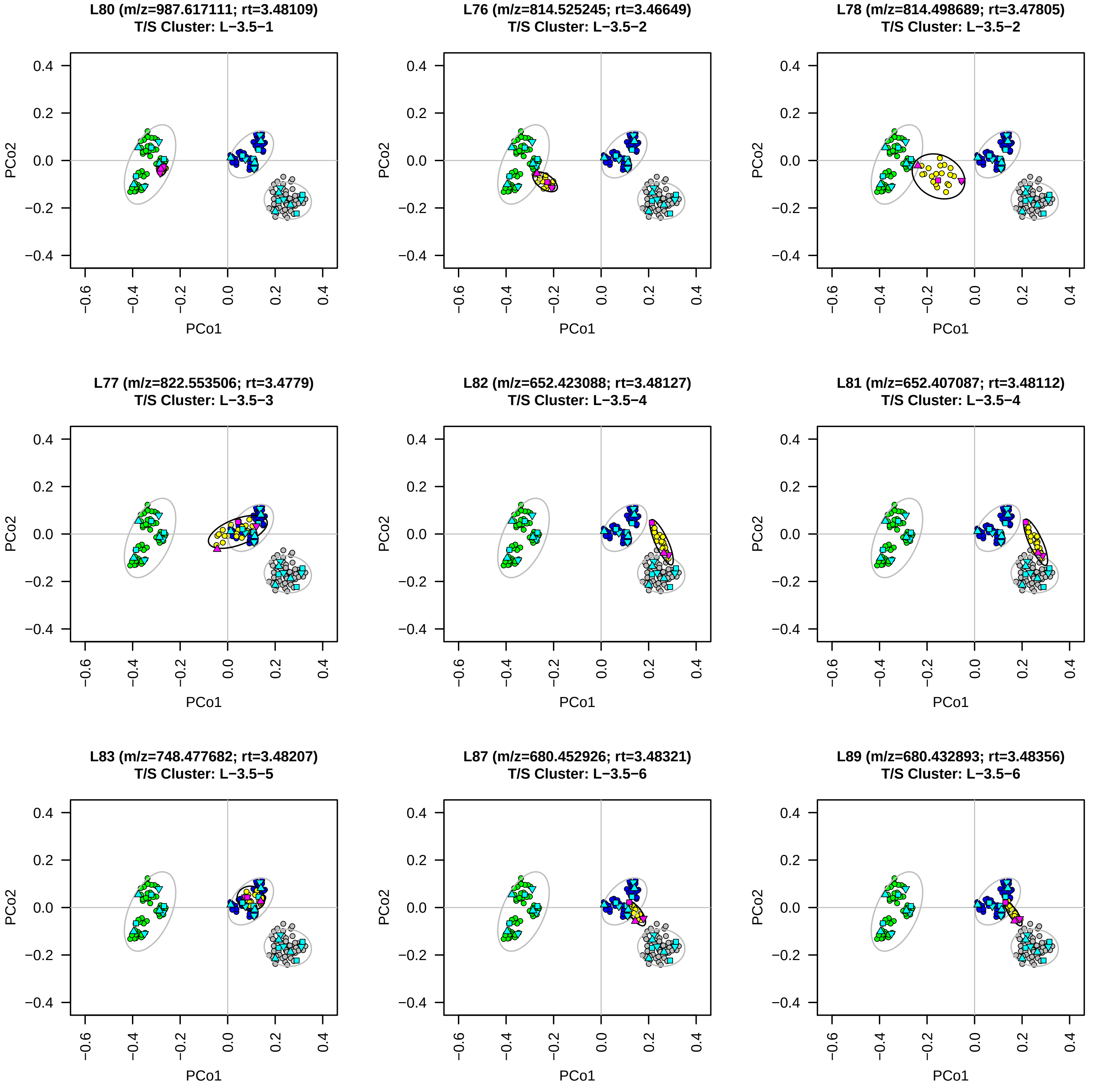
L80 (m/z=987.617111; rt=3.48109)
T/S Cluster: L−3.5−1
L76 (m/z=814.525245; rt=3.46649)
T/S Cluster: L−3.5−2
L78 (m/z=814.498689; rt=3.47805)
T/S Cluster: L−3.5−2
L77 (m/z=822.553506; rt=3.4779)
T/S Cluster: L−3.5−3
L82 (m/z=652.423088; rt=3.48127)
T/S Cluster: L−3.5−4
L81 (m/z=652.407087; rt=3.48112)
T/S Cluster: L−3.5−4
L83 (m/z=748.477682; rt=3.48207)
T/S Cluster: L−3.5−5
L87 (m/z=680.452926; rt=3.48321)
T/S Cluster: L−3.5−6
L89 (m/z=680.432893; rt=3.48356)
T/S Cluster: L−3.5−6
PCo1
PCo2
0.4
0.2
0.0
−0.2
−0.4
−0.6

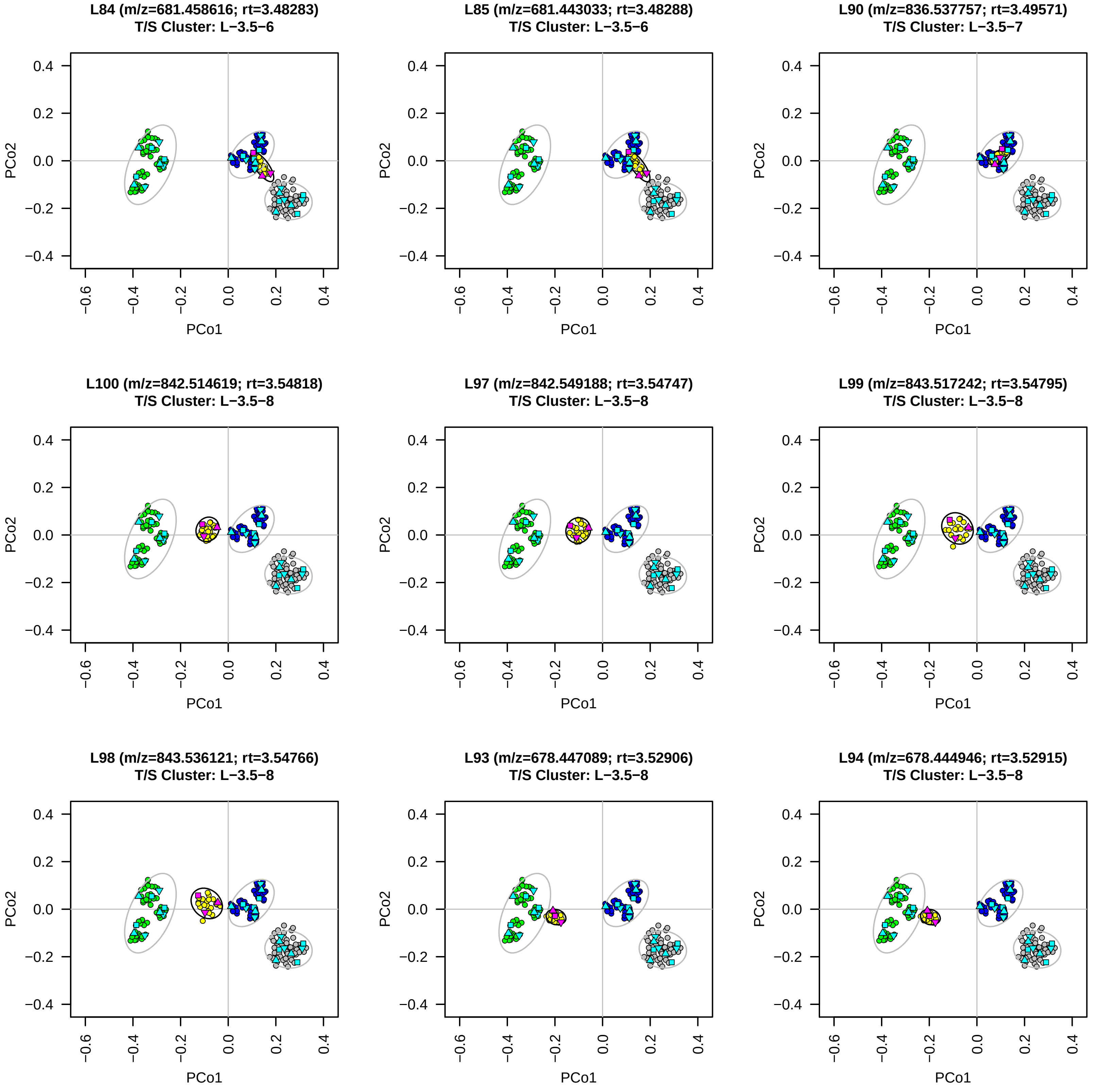
L84 (m/z=681.458616; rt=3.48283)
T/S Cluster: L−3.5−6
L85 (m/z=681.443033; rt=3.48288)
T/S Cluster: L−3.5−6
L90 (m/z=836.537757; rt=3.49571)
T/S Cluster: L−3.5−7
L100 (m/z=842.514619; rt=3.54818)
T/S Cluster: L−3.5−8
L97 (m/z=842.549188; rt=3.54747)
T/S Cluster: L−3.5−8
L99 (m/z=843.517242; rt=3.54795)
T/S Cluster: L−3.5−8
L98 (m/z=843.536121; rt=3.54766)
T/S Cluster: L−3.5−8
L93 (m/z=678.447089; rt=3.52906)
T/S Cluster: L−3.5−8
L94 (m/z=678.444946; rt=3.52915)
T/S Cluster: L−3.5−8
PCo1
PCo2
−0.6
−0.4
−0.2
0.0
0.2
0.4

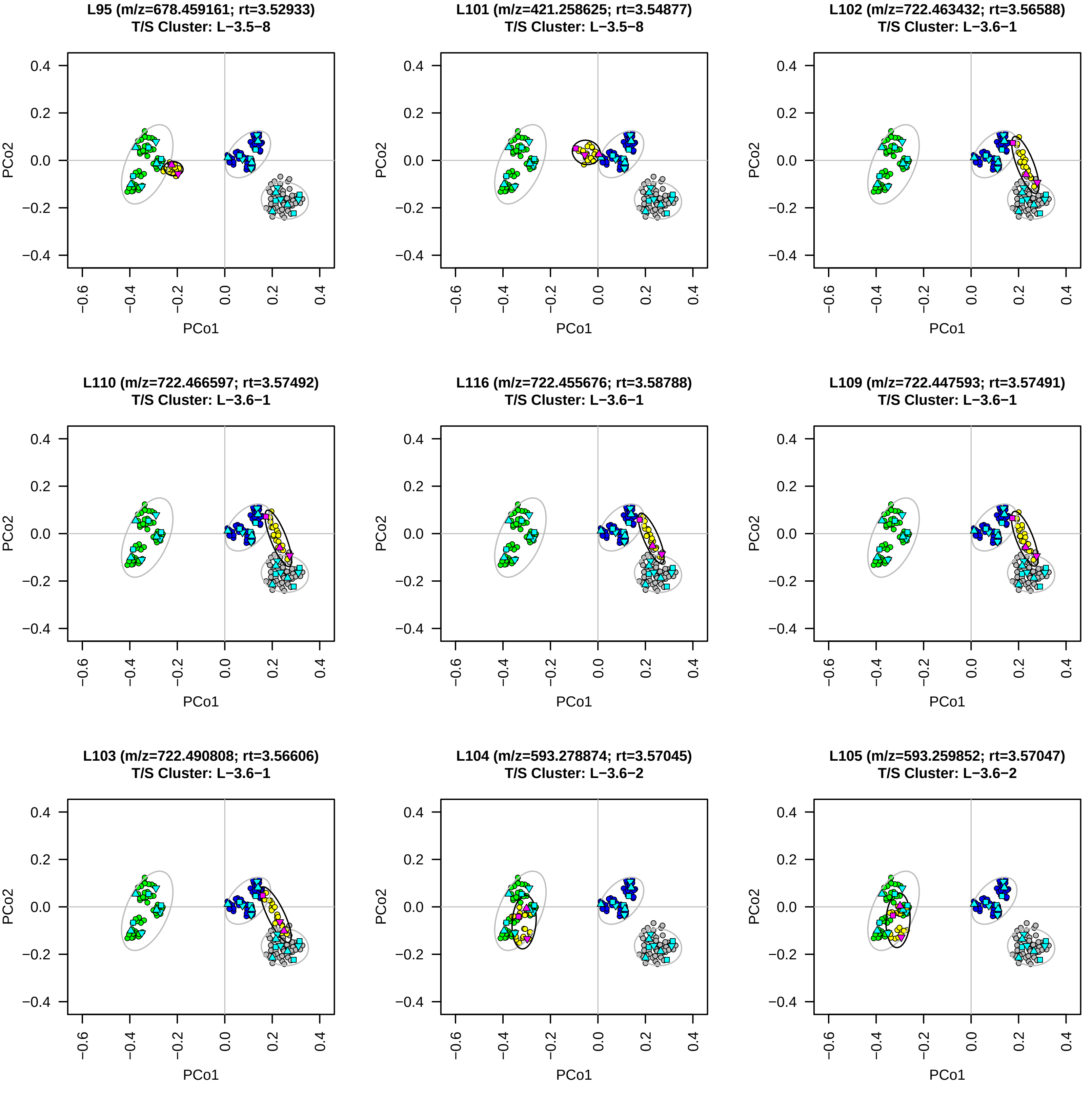
L95 (m/z=678.459161; rt=3.52933)
T/S Cluster: L−3.5−8
L101 (m/z=421.258625; rt=3.54877)
T/S Cluster: L−3.5−8
L102 (m/z=722.463432; rt=3.56588)
T/S Cluster: L−3.6−1
L110 (m/z=722.466597; rt=3.57492)
T/S Cluster: L−3.6−1
L116 (m/z=722.455676; rt=3.58788)
T/S Cluster: L−3.6−1
L109 (m/z=722.447593; rt=3.57491)
T/S Cluster: L−3.6−1
L103 (m/z=722.490808; rt=3.56606)
T/S Cluster: L−3.6−1
L104 (m/z=593.278874; rt=3.57045)
T/S Cluster: L−3.6−2
L105 (m/z=593.259852; rt=3.57047)
T/S Cluster: L−3.6−2
PCo1
PCo2
−0.6
−0.4
−0.2
0.0
0.2
0.4

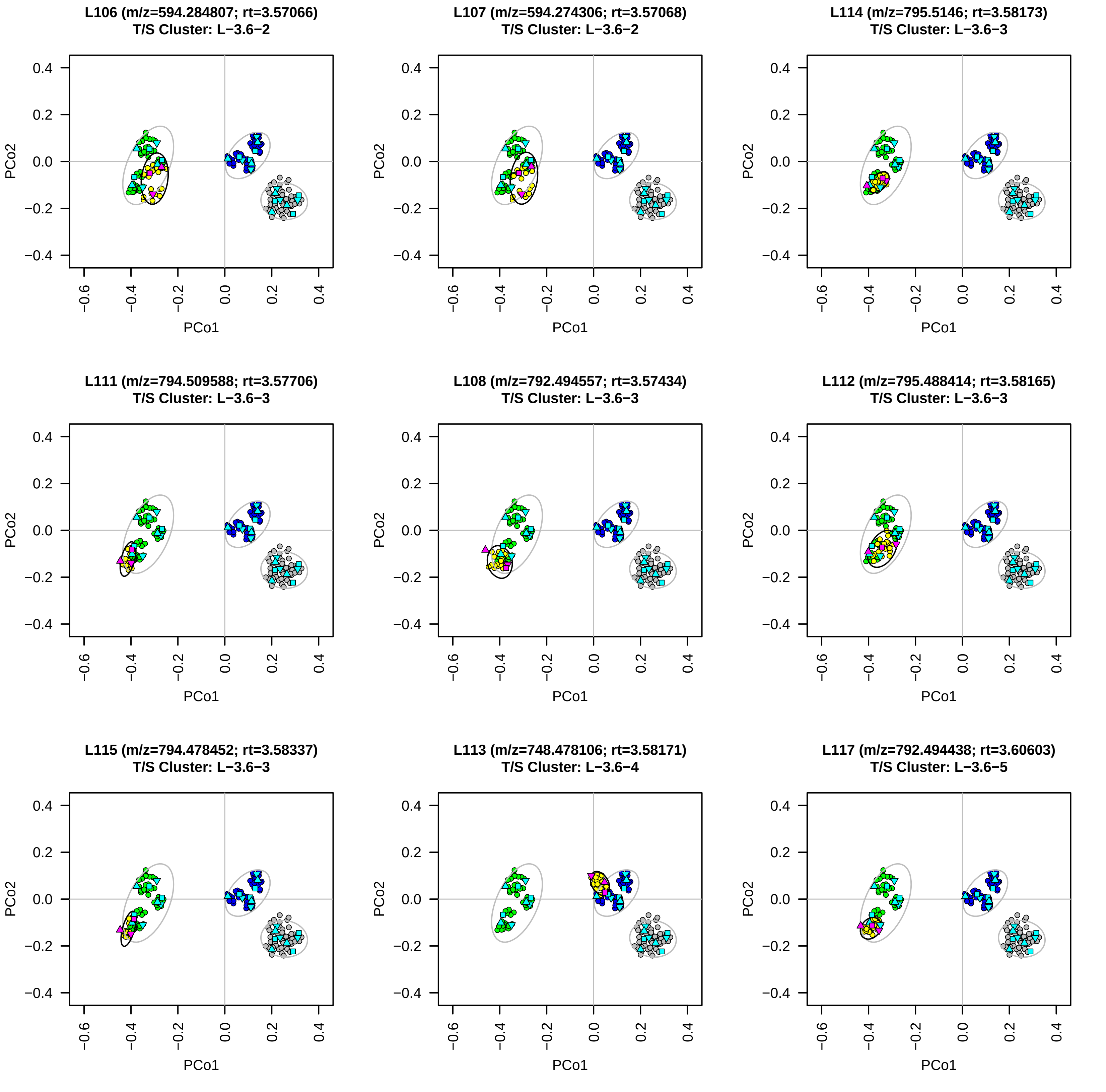
L106 (m/z=594.284807; rt=3.57066)
T/S Cluster: L−3.6−2
L107 (m/z=594.274306; rt=3.57068)
T/S Cluster: L−3.6−2
L114 (m/z=795.5146; rt=3.58173)
T/S Cluster: L−3.6−3
L111 (m/z=794.509588; rt=3.57706)
T/S Cluster: L−3.6−3
L108 (m/z=792.494557; rt=3.57434)
T/S Cluster: L−3.6−3
L112 (m/z=795.488414; rt=3.58165)
T/S Cluster: L−3.6−3
L115 (m/z=794.478452; rt=3.58337)
T/S Cluster: L−3.6−3
L113 (m/z=748.478106; rt=3.58171)
T/S Cluster: L−3.6−4
L117 (m/z=792.494438; rt=3.60603)
T/S Cluster: L−3.6−5
PCo1
PCo2
0.4
0.2
0.0
−0.2
−0.4
−0.6
−0.4
−0.2
0.0
0.2
0.4

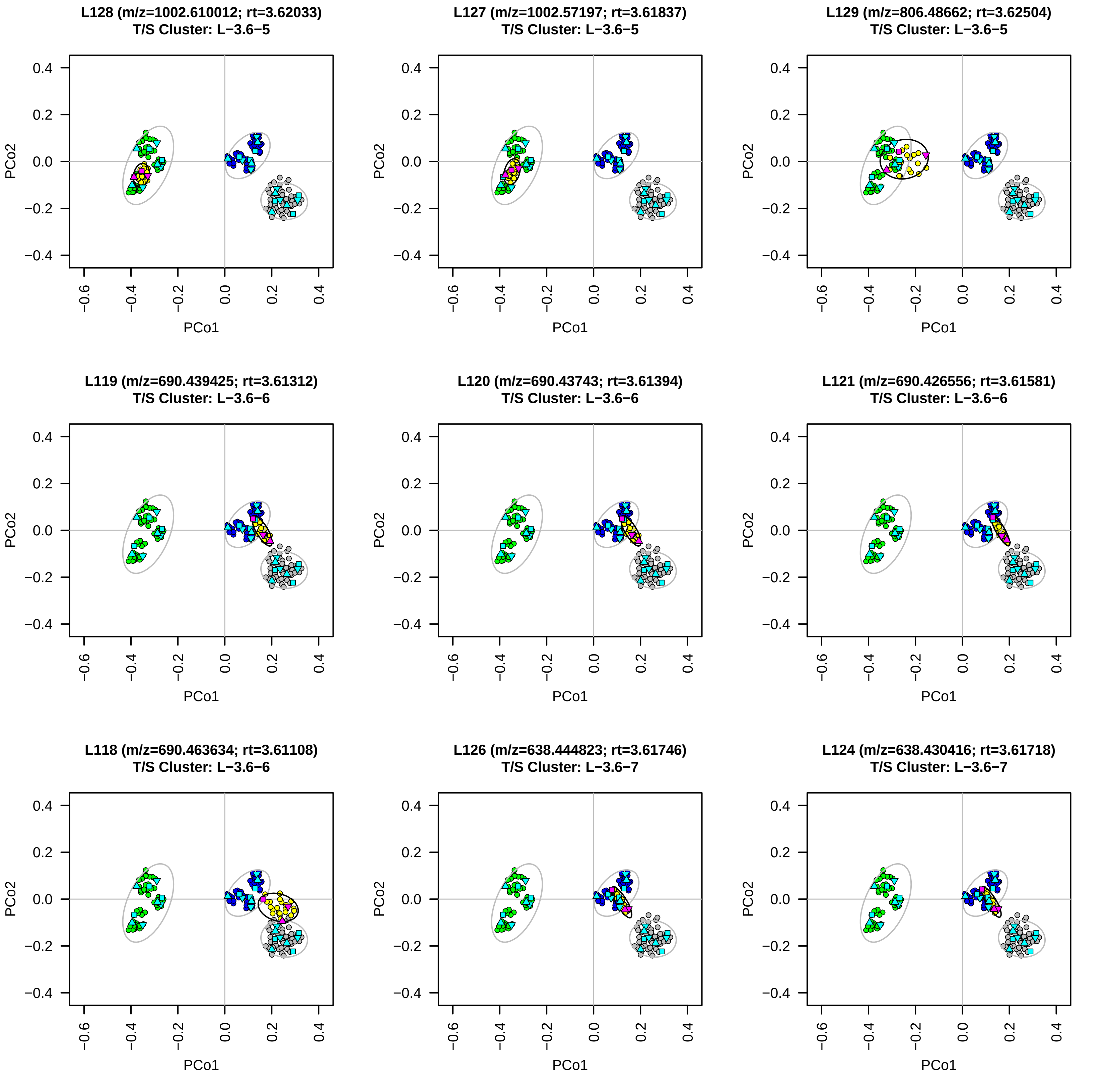
L128 (m/z=1002.610012; rt=3.62033)
T/S Cluster: L−3.6−5
L127 (m/z=1002.57197; rt=3.61837)
T/S Cluster: L−3.6−5
L129 (m/z=806.48662; rt=3.62504)
T/S Cluster: L−3.6−5
L119 (m/z=690.439425; rt=3.61312)
T/S Cluster: L−3.6−6
L120 (m/z=690.43743; rt=3.61394)
T/S Cluster: L−3.6−6
L121 (m/z=690.426556; rt=3.61581)
T/S Cluster: L−3.6−6
L118 (m/z=690.463634; rt=3.61108)
T/S Cluster: L−3.6−6
L126 (m/z=638.444823; rt=3.61746)
T/S Cluster: L−3.6−7
L124 (m/z=638.430416; rt=3.61718)
T/S Cluster: L−3.6−7
PCo1
PCo2
0.4
0.2
0.0
−0.2
−0.4
−0.6

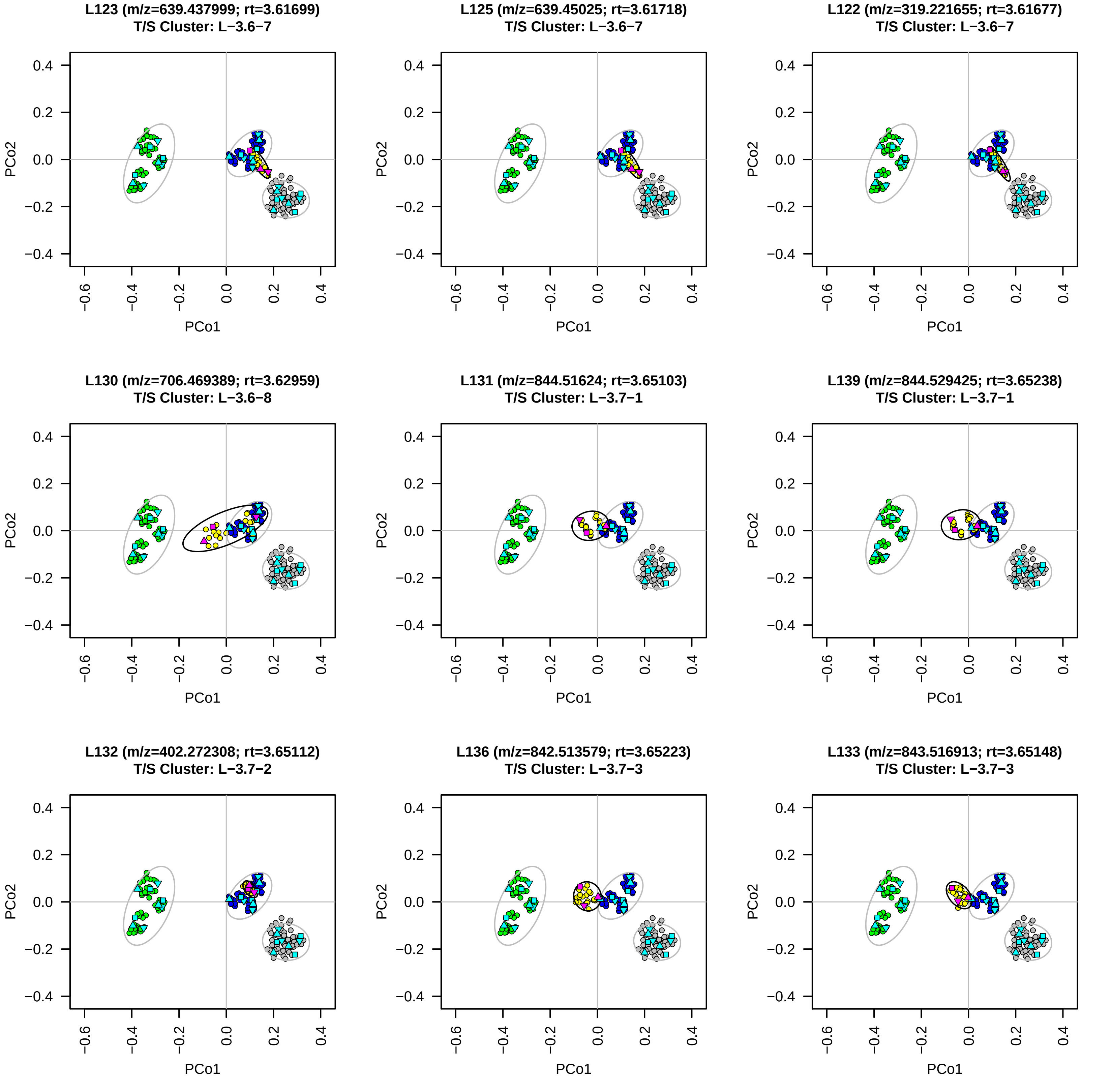
L123 (m/z=639.437999; rt=3.61699)
T/S Cluster: L−3.6−7
L125 (m/z=639.45025; rt=3.61718)
T/S Cluster: L−3.6−7
L122 (m/z=319.221655; rt=3.61677)
T/S Cluster: L−3.6−7
L130 (m/z=706.469389; rt=3.62959)
T/S Cluster: L−3.6−8
L131 (m/z=844.51624; rt=3.65103)
T/S Cluster: L−3.7−1
L139 (m/z=844.529425; rt=3.65238)
T/S Cluster: L−3.7−1
L132 (m/z=402.272308; rt=3.65112)
T/S Cluster: L−3.7−2
L136 (m/z=842.513579; rt=3.65223)
T/S Cluster: L−3.7−3
L133 (m/z=843.516913; rt=3.65148)
T/S Cluster: L−3.7−3
PCo1
PCo2
0.4
0.2
0.0
−0.2
−0.4
−0.6

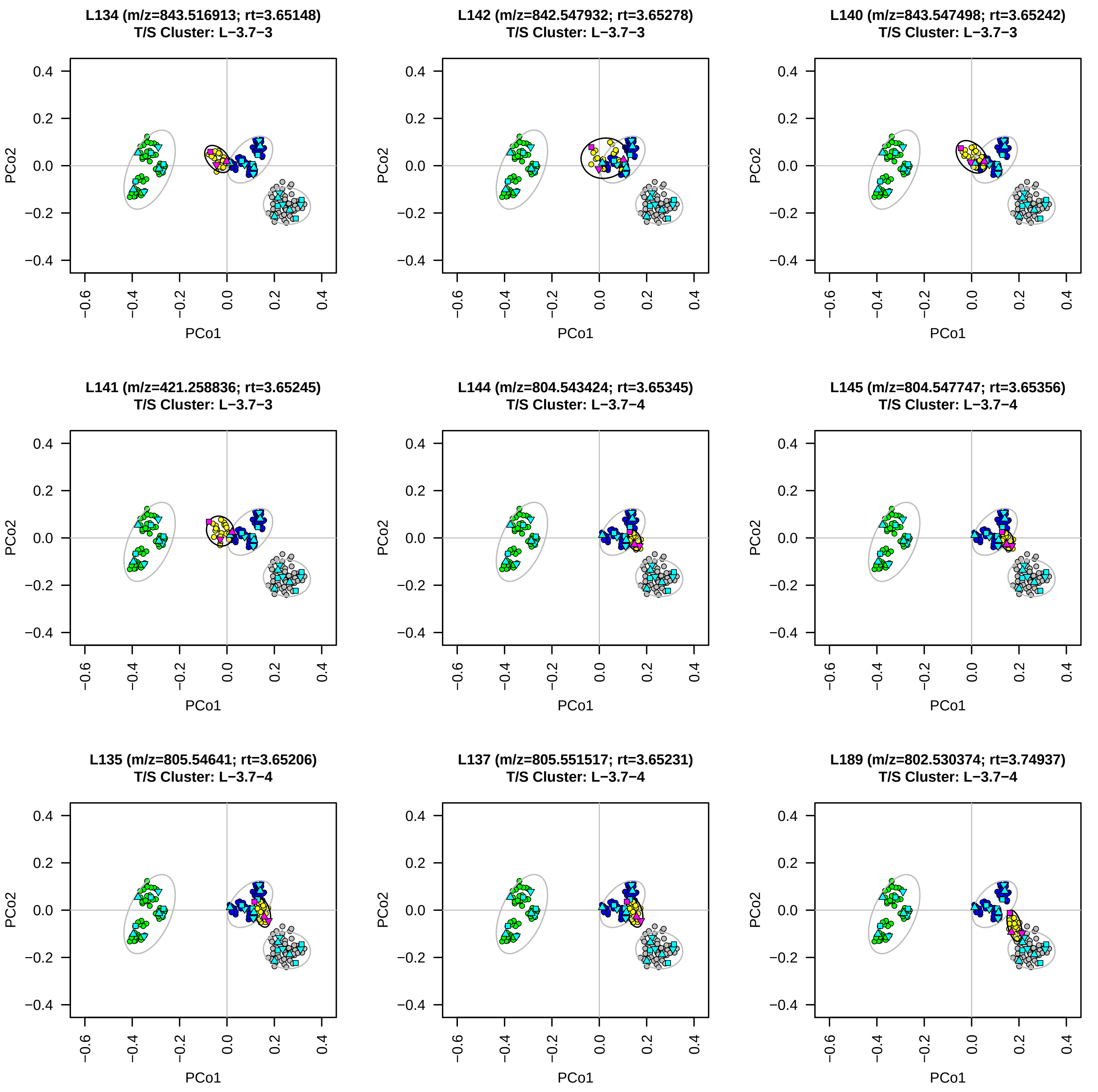
L134 (m/z=843.516913; rt=3.65148)
T/S Cluster: L−3.7−3
L142 (m/z=842.547932; rt=3.65278)
T/S Cluster: L−3.7−3
L140 (m/z=843.547498; rt=3.65242)
T/S Cluster: L−3.7−3
L141 (m/z=421.258836; rt=3.65245)
T/S Cluster: L−3.7−3
L144 (m/z=804.543424; rt=3.65345)
T/S Cluster: L−3.7−4
L145 (m/z=804.547747; rt=3.65356)
T/S Cluster: L−3.7−4
L135 (m/z=805.54641; rt=3.65206)
T/S Cluster: L−3.7−4
L137 (m/z=805.551517; rt=3.65231)
T/S Cluster: L−3.7−4
L189 (m/z=802.530374; rt=3.74937)
T/S Cluster: L−3.7−4
PCo1
PCo2

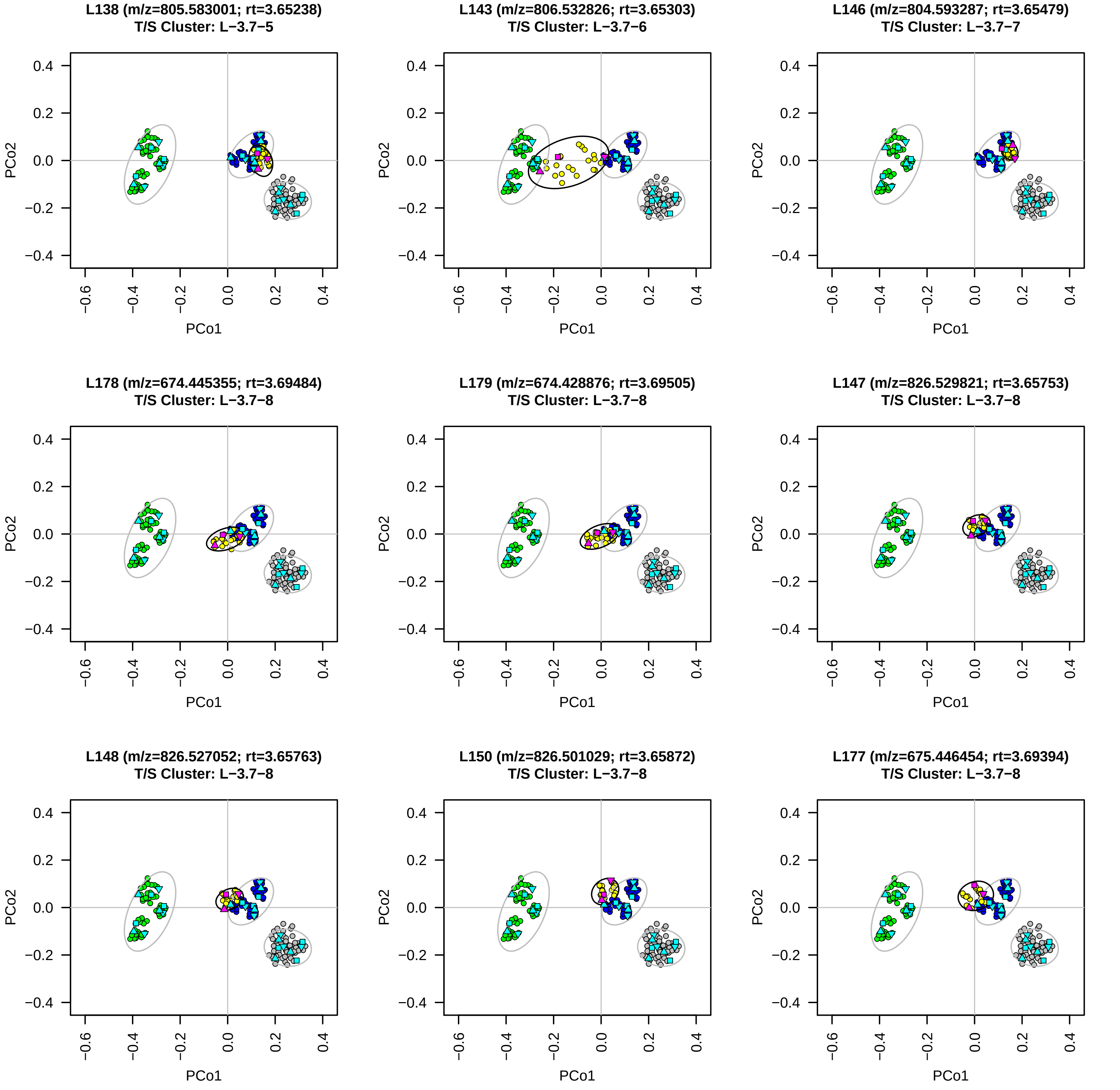
L138 (m/z=805.583001; rt=3.65238)
T/S Cluster: L−3.7−5
L143 (m/z=806.532826; rt=3.65303)
T/S Cluster: L−3.7−6
L146 (m/z=804.593287; rt=3.65479)
T/S Cluster: L−3.7−7
L178 (m/z=674.445355; rt=3.69484)
T/S Cluster: L−3.7−8
L179 (m/z=674.428876; rt=3.69505)
T/S Cluster: L−3.7−8
L147 (m/z=826.529821; rt=3.65753)
T/S Cluster: L−3.7−8
L148 (m/z=826.527052; rt=3.65763)
T/S Cluster: L−3.7−8
L150 (m/z=826.501029; rt=3.65872)
T/S Cluster: L−3.7−8
L177 (m/z=675.446454; rt=3.69394)
T/S Cluster: L−3.7−8
PCo1
PCo2
0.4
0.2
0.0
−0.2
−0.4
−0.6

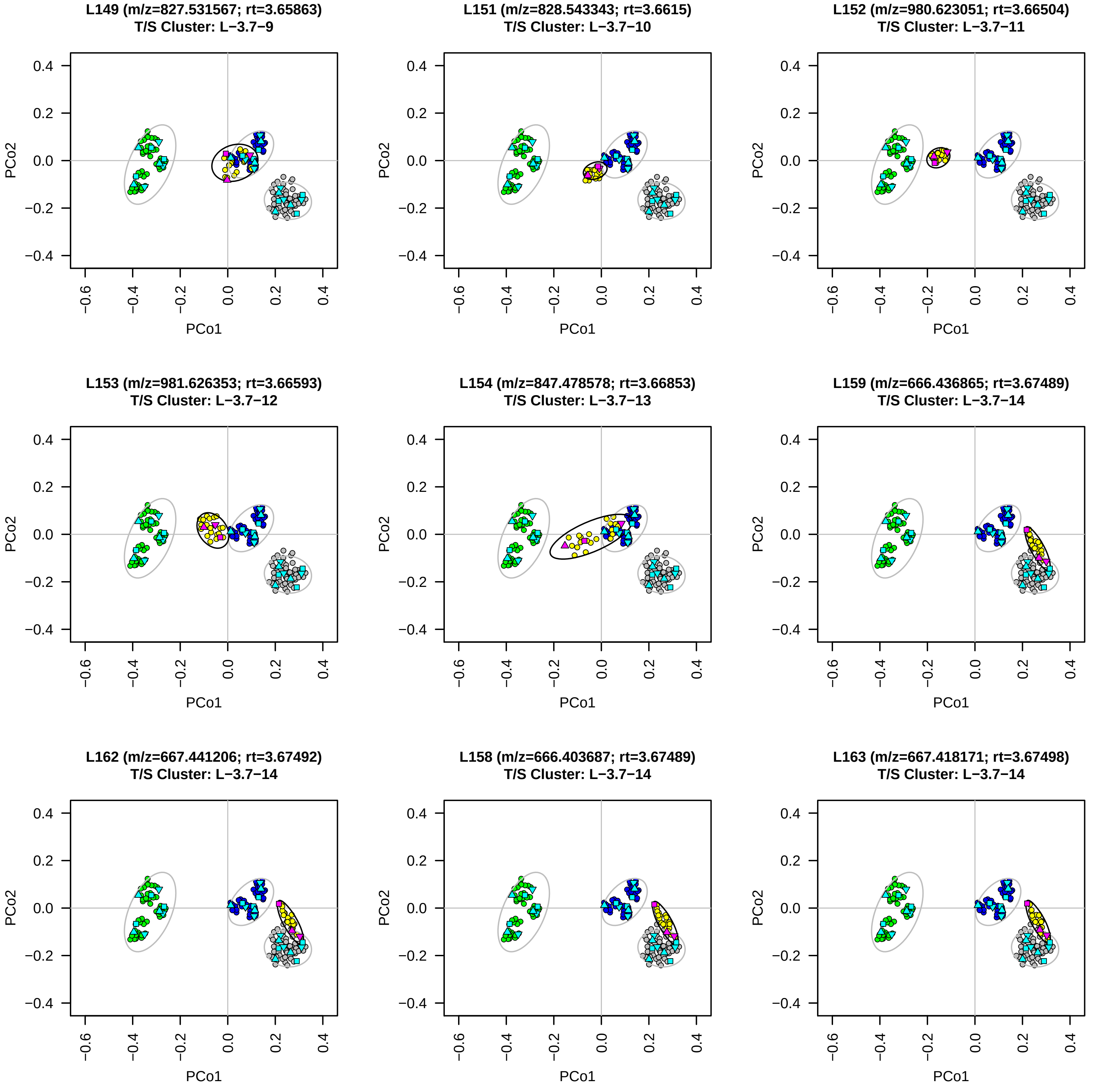
L149 (m/z=827.531567; rt=3.65863)
T/S Cluster: L−3.7−9
L151 (m/z=828.543343; rt=3.6615)
T/S Cluster: L−3.7−10
L152 (m/z=980.623051; rt=3.66504)
T/S Cluster: L−3.7−11
L153 (m/z=981.626353; rt=3.66593)
T/S Cluster: L−3.7−12
L154 (m/z=847.478578; rt=3.66853)
T/S Cluster: L−3.7−13
L159 (m/z=666.436865; rt=3.67489)
T/S Cluster: L−3.7−14
L162 (m/z=667.441206; rt=3.67492)
T/S Cluster: L−3.7−14
L158 (m/z=666.403687; rt=3.67489)
T/S Cluster: L−3.7−14
L163 (m/z=667.418171; rt=3.67498)
T/S Cluster: L−3.7−14
PCo2
0.4
0.2
0.0
−0.2
−0.4
PCo1
−0.6
−0.4
−0.2
0.0
0.2
0.4

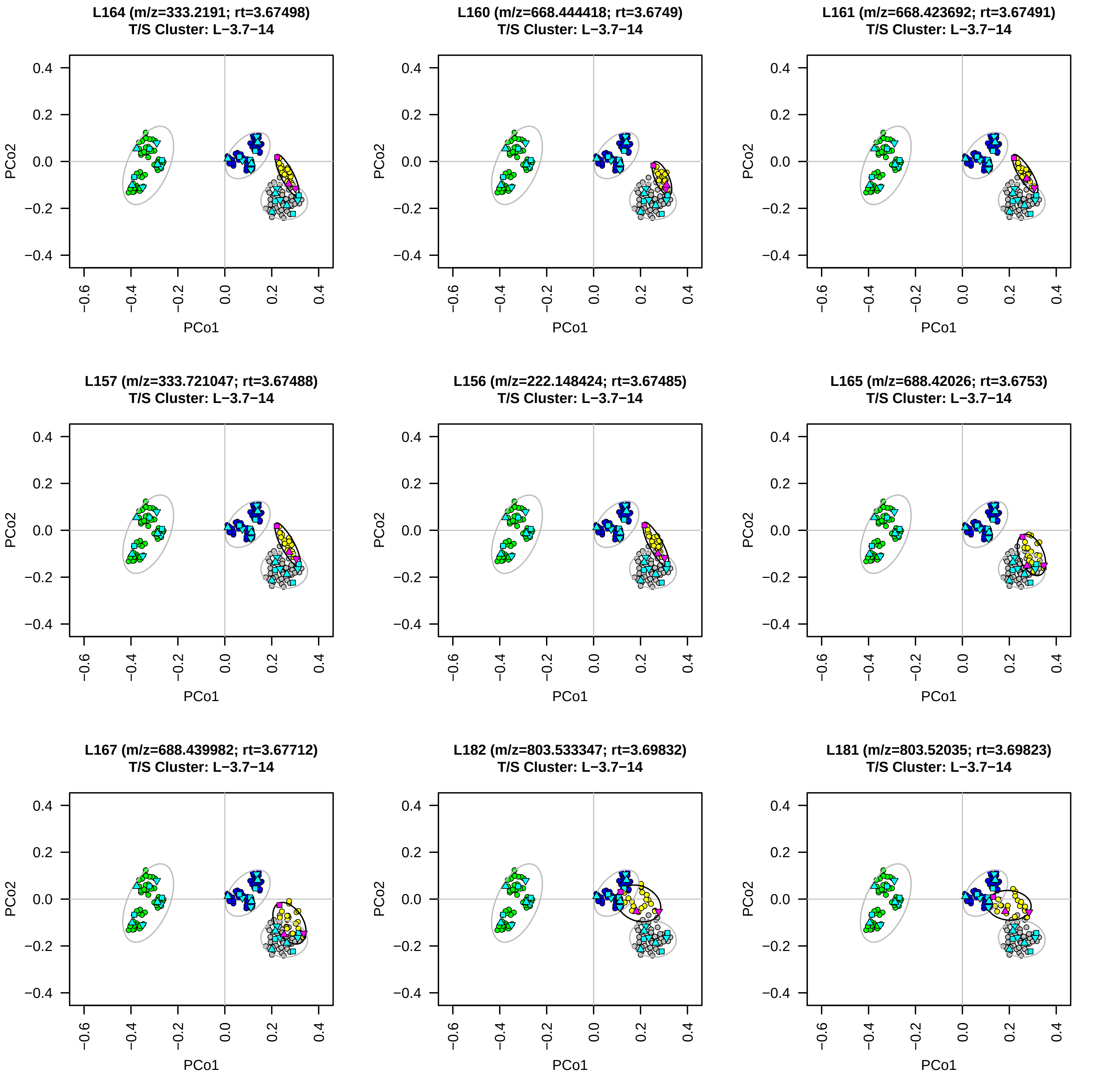

L164 (m/z=333.2191; rt=3.67498)
T/S Cluster: L−3.7−14
PCo2
PCo1
L160 (m/z=668.444418; rt=3.6749)
T/S Cluster: L−3.7−14
PCo2
PCo1
L161 (m/z=668.423692; rt=3.67491)
T/S Cluster: L−3.7−14
PCo2
PCo1
L157 (m/z=333.721047; rt=3.67488)
T/S Cluster: L−3.7−14
PCo2
PCo1
L156 (m/z=222.148424; rt=3.67485)
T/S Cluster: L−3.7−14
PCo2
PCo1
L165 (m/z=688.42026; rt=3.6753)
T/S Cluster: L−3.7−14
PCo2
PCo1
L167 (m/z=688.439982; rt=3.67712)
T/S Cluster: L−3.7−14
PCo2
PCo1
L182 (m/z=803.533347; rt=3.69832)
T/S Cluster: L−3.7−14
PCo2
PCo1
L181 (m/z=803.52035; rt=3.69823)
T/S Cluster: L−3.7−14
PCo2
PCo1

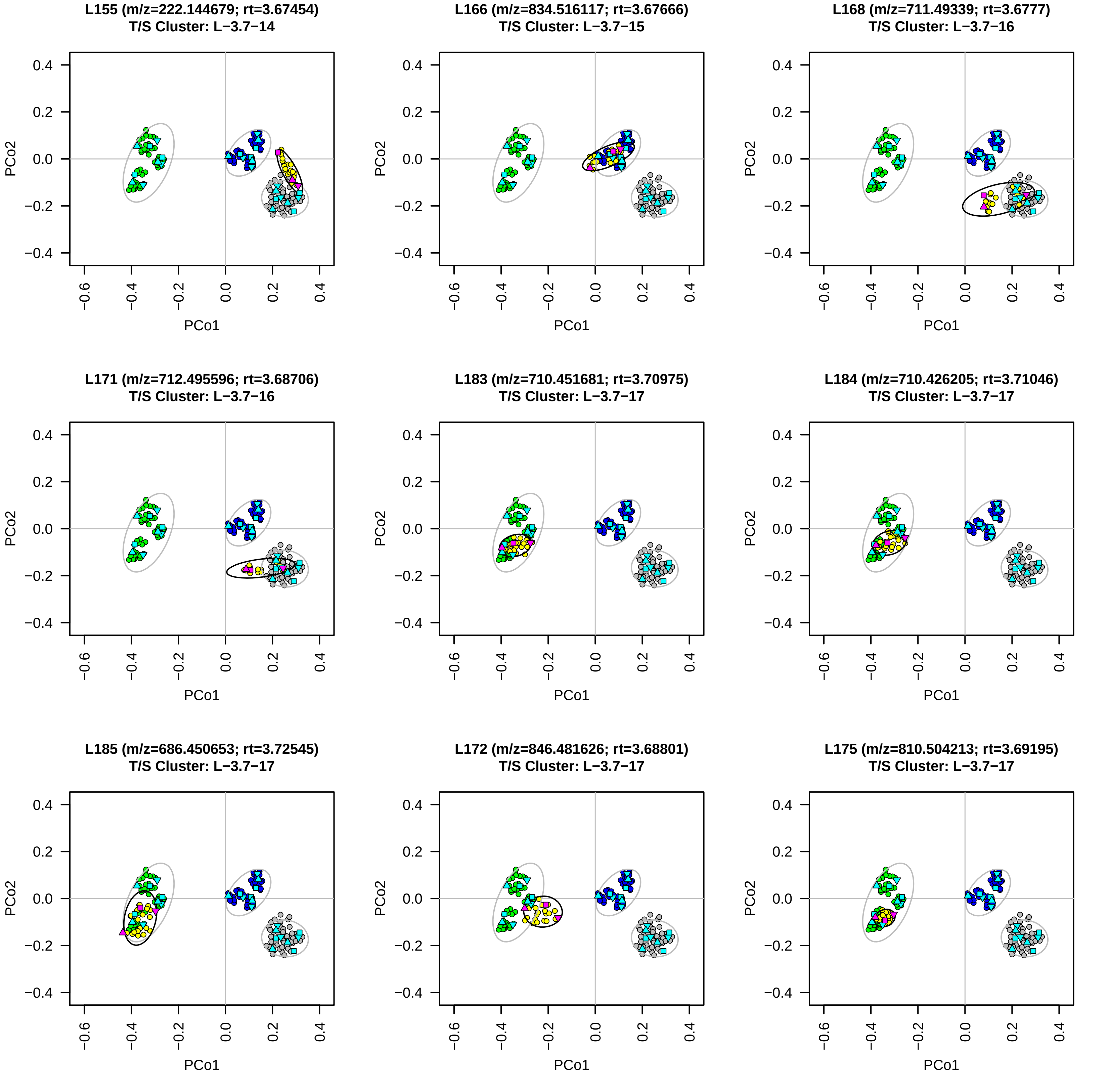
L155 (m/z=222.144679; rt=3.67454)
T/S Cluster: L−3.7−14
L166 (m/z=834.516117; rt=3.67666)
T/S Cluster: L−3.7−15
L168 (m/z=711.49339; rt=3.6777)
T/S Cluster: L−3.7−16
L171 (m/z=712.495596; rt=3.68706)
T/S Cluster: L−3.7−16
L183 (m/z=710.451681; rt=3.70975)
T/S Cluster: L−3.7−17
L184 (m/z=710.426205; rt=3.71046)
T/S Cluster: L−3.7−17
L185 (m/z=686.450653; rt=3.72545)
T/S Cluster: L−3.7−17
L172 (m/z=846.481626; rt=3.68801)
T/S Cluster: L−3.7−17
L175 (m/z=810.504213; rt=3.69195)
T/S Cluster: L−3.7−17
PCo1
PCo2
−0.6
−0.4
−0.2
0.0
0.2
0.4

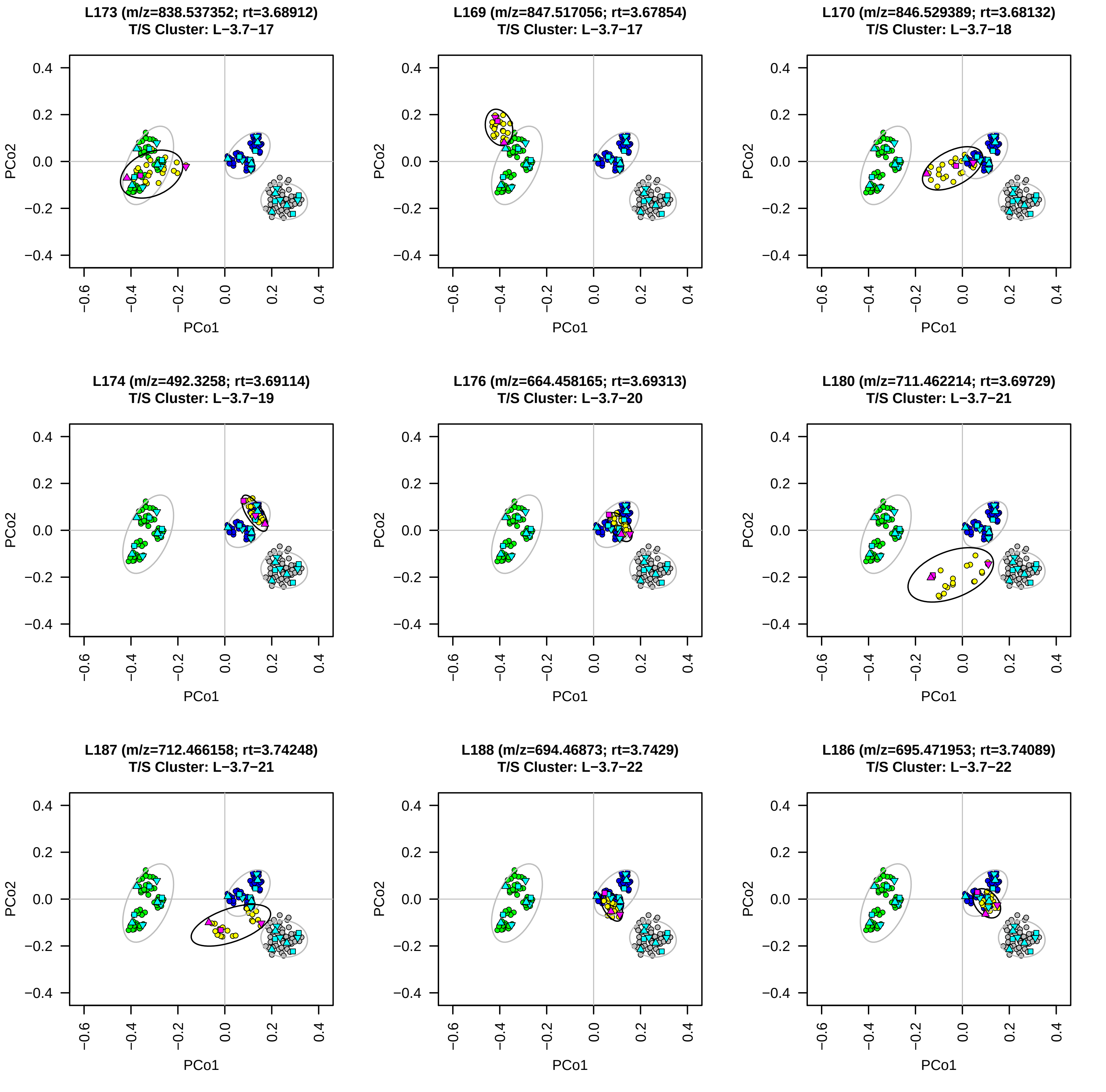
L173 (m/z=838.537352; rt=3.68912)
T/S Cluster: L−3.7−17
L169 (m/z=847.517056; rt=3.67854)
T/S Cluster: L−3.7−17
L170 (m/z=846.529389; rt=3.68132)
T/S Cluster: L−3.7−18
L174 (m/z=492.3258; rt=3.69114)
T/S Cluster: L−3.7−19
L176 (m/z=664.458165; rt=3.69313)
T/S Cluster: L−3.7−20
L180 (m/z=711.462214; rt=3.69729)
T/S Cluster: L−3.7−21
L187 (m/z=712.466158; rt=3.74248)
T/S Cluster: L−3.7−21
L188 (m/z=694.46873; rt=3.7429)
T/S Cluster: L−3.7−22
L186 (m/z=695.471953; rt=3.74089)
T/S Cluster: L−3.7−22
PCo1
PCo2
0.4
0.2
0.0
−0.2
−0.4
−0.6

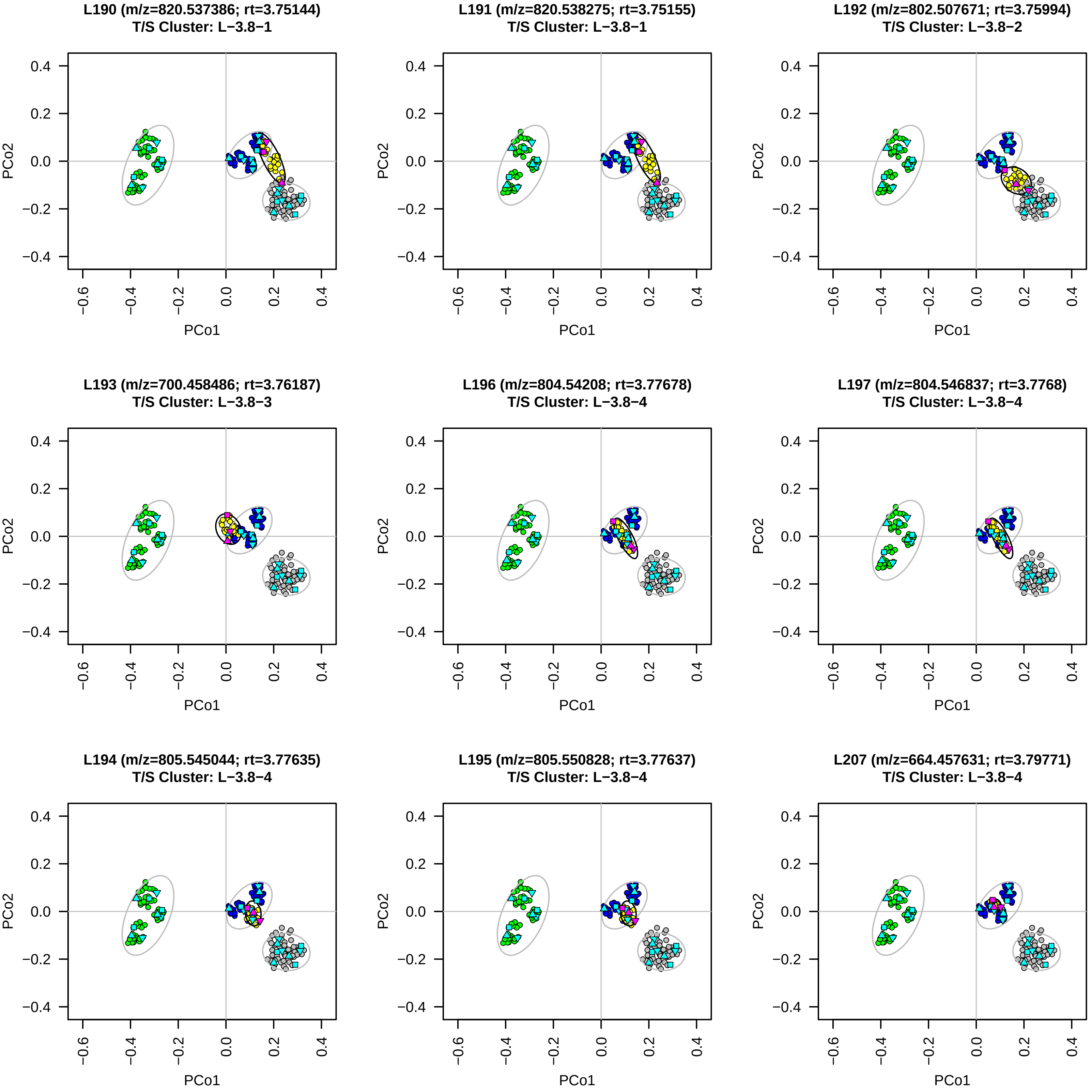
L190 (m/z=820.537386; rt=3.75144)
T/S Cluster: L−3.8−1
L191 (m/z=820.538275; rt=3.75155)
T/S Cluster: L−3.8−1
L192 (m/z=802.507671; rt=3.75994)
T/S Cluster: L−3.8−2
L193 (m/z=700.458486; rt=3.76187)
T/S Cluster: L−3.8−3
L196 (m/z=804.54208; rt=3.77678)
T/S Cluster: L−3.8−4
L197 (m/z=804.546837; rt=3.7768)
T/S Cluster: L−3.8−4
L194 (m/z=805.545044; rt=3.77635)
T/S Cluster: L−3.8−4
L195 (m/z=805.550828; rt=3.77637)
T/S Cluster: L−3.8−4
L207 (m/z=664.457631; rt=3.79771)
T/S Cluster: L−3.8−4
PCo1
PCo2
0.4
0.2
0.0
−0.2
−0.4
−0.6
−0.4
−0.2
0.0
0.2
0.4

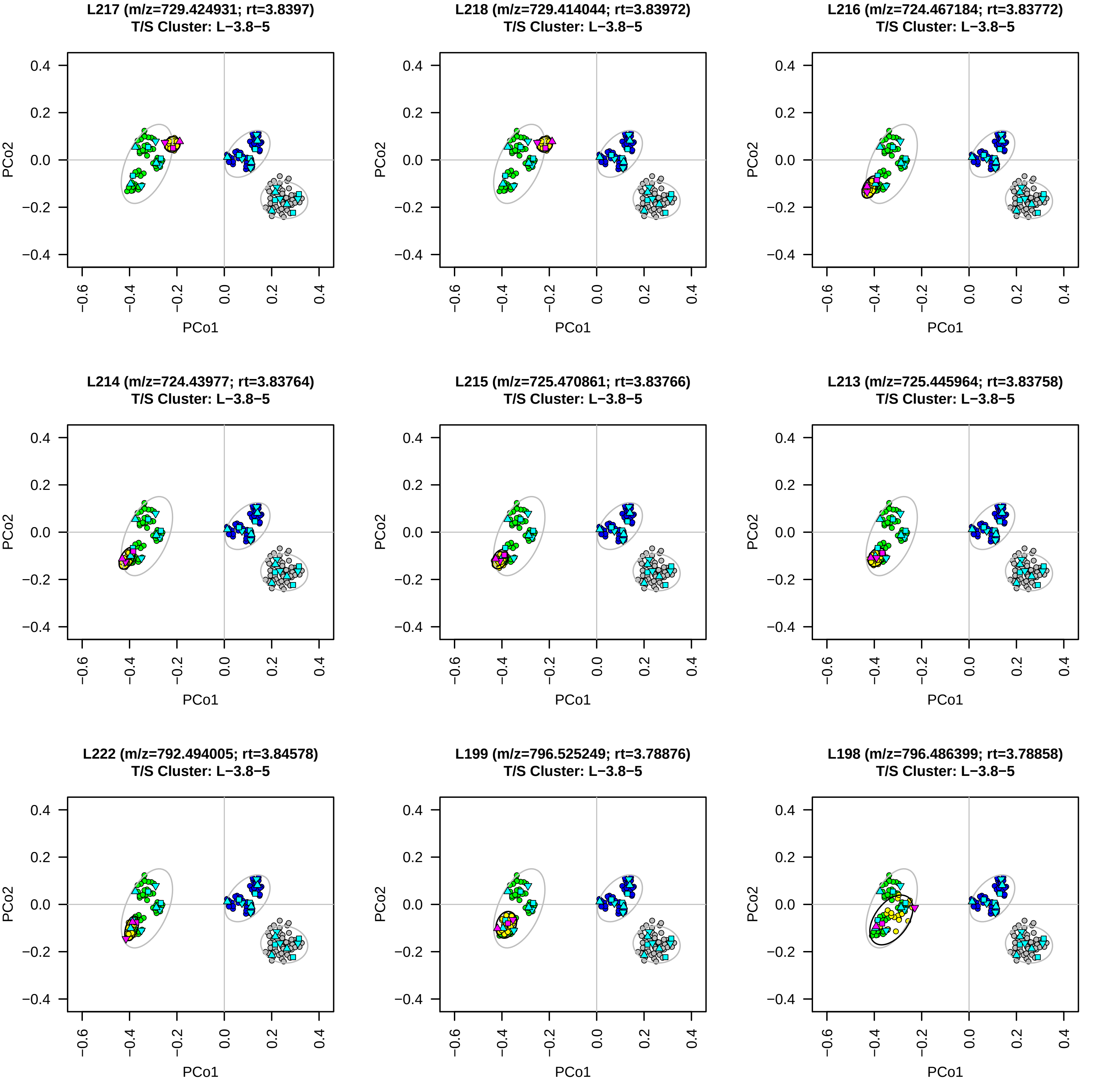
L217 (m/z=729.424931; rt=3.8397)
T/S Cluster: L−3.8−5
L218 (m/z=729.414044; rt=3.83972)
T/S Cluster: L−3.8−5
L216 (m/z=724.467184; rt=3.83772)
T/S Cluster: L−3.8−5
L214 (m/z=724.43977; rt=3.83764)
T/S Cluster: L−3.8−5
L215 (m/z=725.470861; rt=3.83766)
T/S Cluster: L−3.8−5
L213 (m/z=725.445964; rt=3.83758)
T/S Cluster: L−3.8−5
L222 (m/z=792.494005; rt=3.84578)
T/S Cluster: L−3.8−5
L199 (m/z=796.525249; rt=3.78876)
T/S Cluster: L−3.8−5
L198 (m/z=796.486399; rt=3.78858)
T/S Cluster: L−3.8−5
PCo1
PCo2

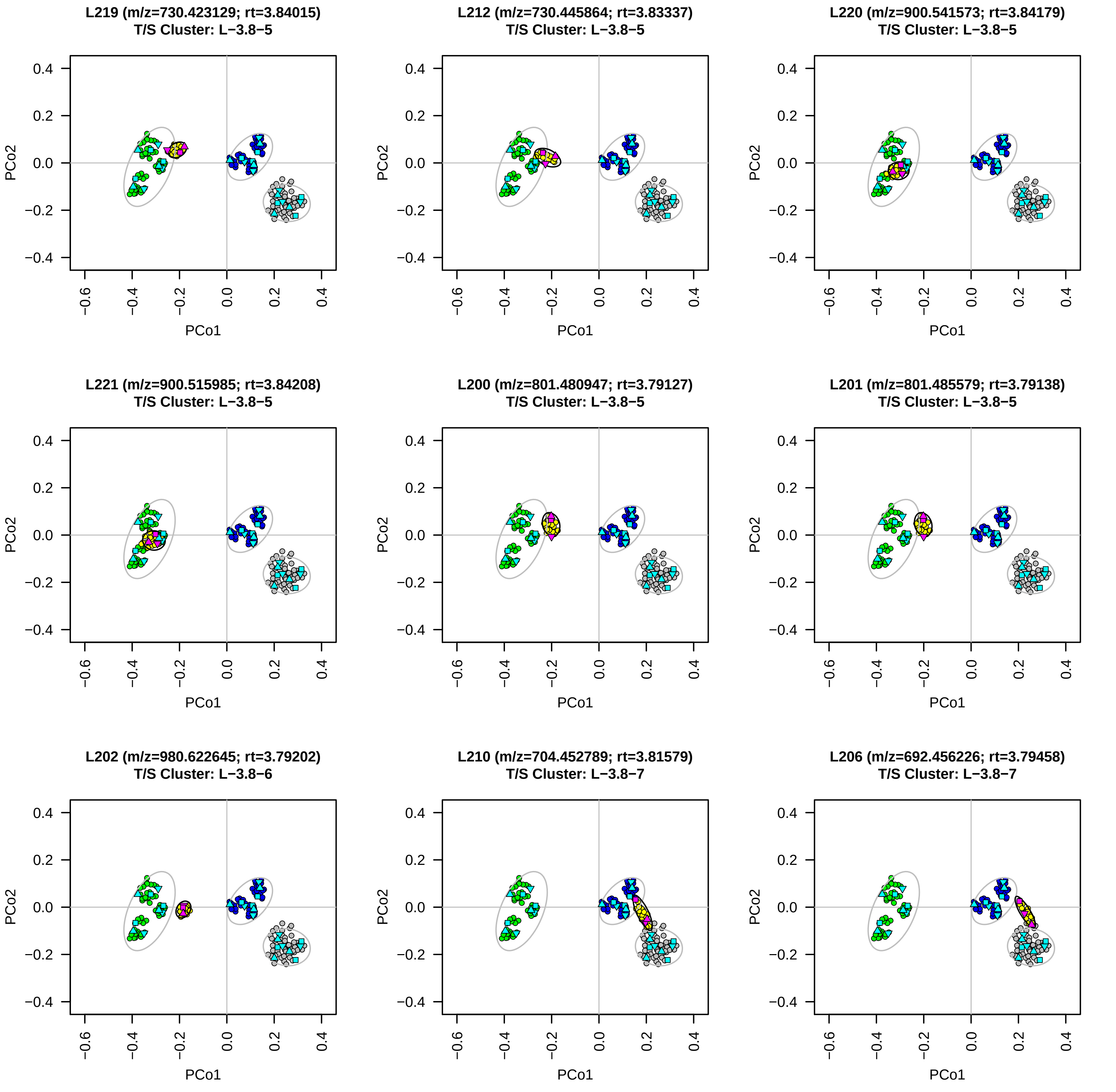
L219 (m/z=730.423129; rt=3.84015)
T/S Cluster: L−3.8−5
L212 (m/z=730.445864; rt=3.83337)
T/S Cluster: L−3.8−5
L220 (m/z=900.541573; rt=3.84179)
T/S Cluster: L−3.8−5
L221 (m/z=900.515985; rt=3.84208)
T/S Cluster: L−3.8−5
L200 (m/z=801.480947; rt=3.79127)
T/S Cluster: L−3.8−5
L201 (m/z=801.485579; rt=3.79138)
T/S Cluster: L−3.8−5
L202 (m/z=980.622645; rt=3.79202)
T/S Cluster: L−3.8−6
L210 (m/z=704.452789; rt=3.81579)
T/S Cluster: L−3.8−7
L206 (m/z=692.456226; rt=3.79458)
T/S Cluster: L−3.8−7
PCo1
PCo2
0.4
0.2
0.0
−0.2
−0.4
−0.6

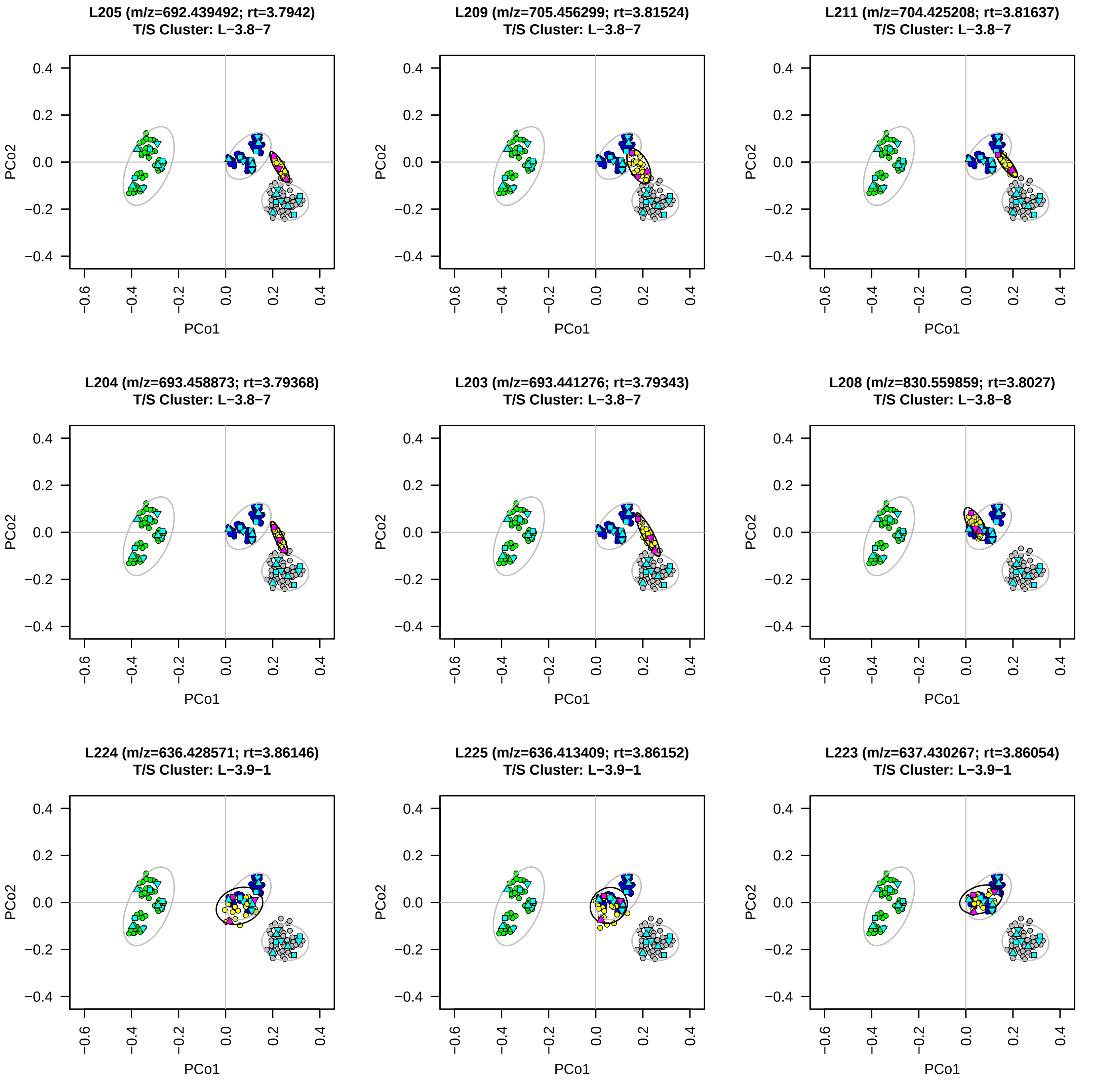
L205 (m/z=692.439492; rt=3.7942)
T/S Cluster: L−3.8−7
L209 (m/z=705.456299; rt=3.81524)
T/S Cluster: L−3.8−7
L211 (m/z=704.425208; rt=3.81637)
T/S Cluster: L−3.8−7
L204 (m/z=693.458873; rt=3.79368)
T/S Cluster: L−3.8−7
L203 (m/z=693.441276; rt=3.79343)
T/S Cluster: L−3.8−7
L208 (m/z=830.559859; rt=3.8027)
T/S Cluster: L−3.8−8
L224 (m/z=636.428571; rt=3.86146)
T/S Cluster: L−3.9−1
L225 (m/z=636.413409; rt=3.86152)
T/S Cluster: L−3.9−1
L223 (m/z=637.430267; rt=3.86054)
T/S Cluster: L−3.9−1
PCo1
PCo2
−0.6
−0.4
−0.2
0.0
0.2
0.4

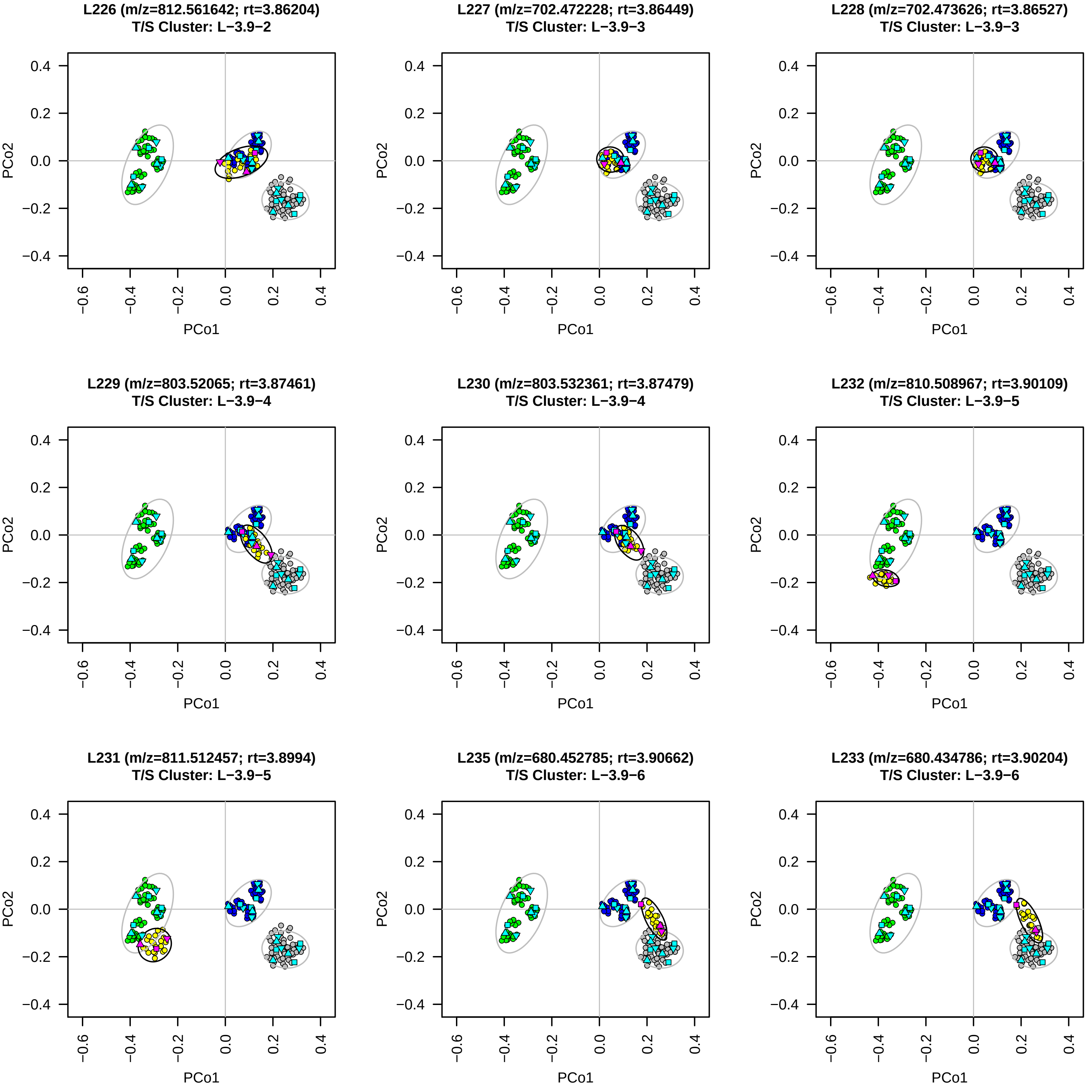

L226 (m/z=812.561642; rt=3.86204)
T/S Cluster: L−3.9−2
L227 (m/z=702.472228; rt=3.86449)
T/S Cluster: L−3.9−3
L228 (m/z=702.473626; rt=3.86527)
T/S Cluster: L−3.9−3
L229 (m/z=803.52065; rt=3.87461)
T/S Cluster: L−3.9−4
L230 (m/z=803.532361; rt=3.87479)
T/S Cluster: L−3.9−4
L232 (m/z=810.508967; rt=3.90109)
T/S Cluster: L−3.9−5
L231 (m/z=811.512457; rt=3.8994)
T/S Cluster: L−3.9−5
L235 (m/z=680.452785; rt=3.90662)
T/S Cluster: L−3.9−6
L233 (m/z=680.434786; rt=3.90204)
T/S Cluster: L−3.9−6
PCo1
PCo2

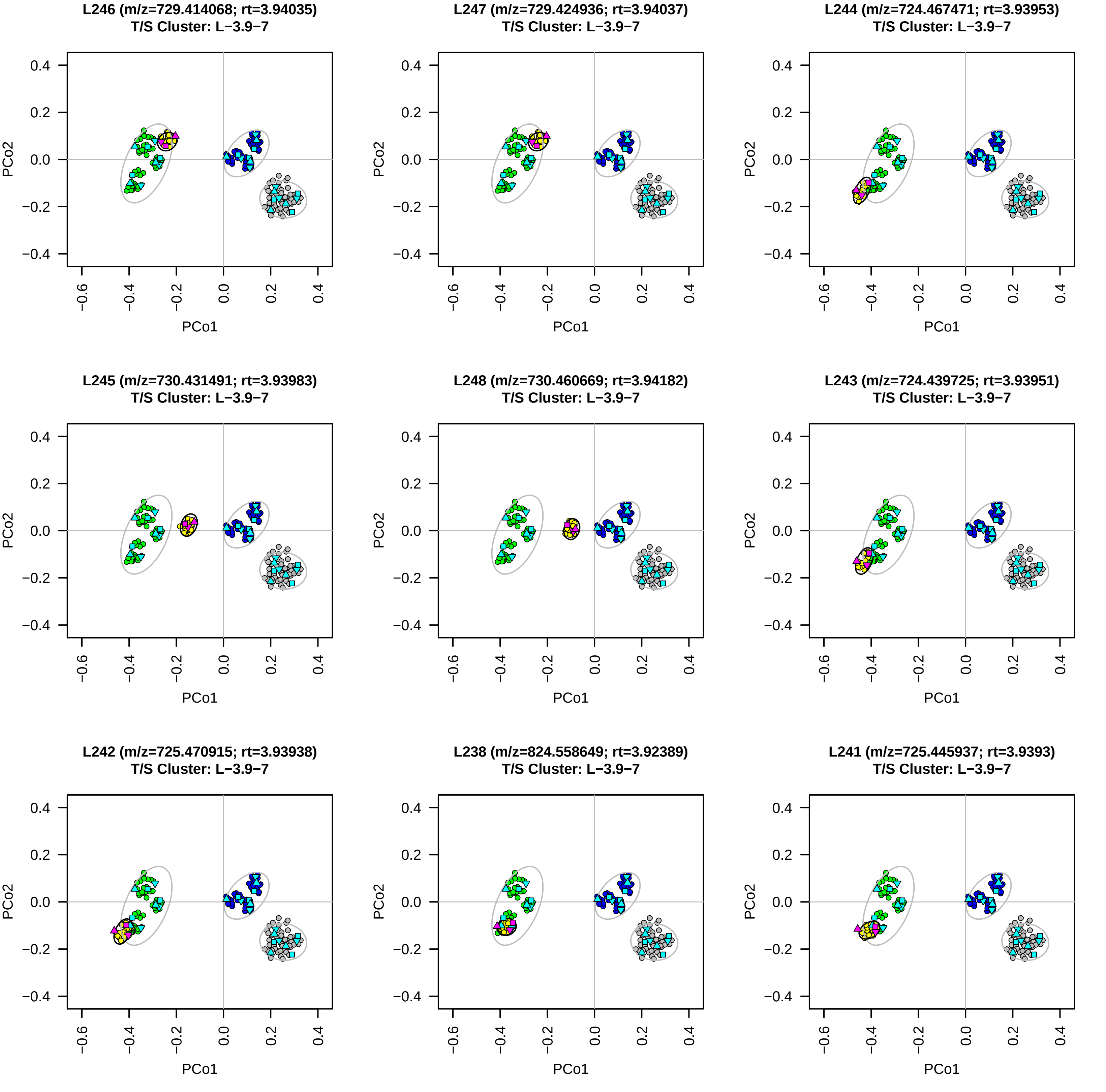
L246 (m/z=729.414068; rt=3.94035)
T/S Cluster: L−3.9−7
L247 (m/z=729.424936; rt=3.94037)
T/S Cluster: L−3.9−7
L244 (m/z=724.467471; rt=3.93953)
T/S Cluster: L−3.9−7
L245 (m/z=730.431491; rt=3.93983)
T/S Cluster: L−3.9−7
L248 (m/z=730.460669; rt=3.94182)
T/S Cluster: L−3.9−7
L243 (m/z=724.439725; rt=3.93951)
T/S Cluster: L−3.9−7
L242 (m/z=725.470915; rt=3.93938)
T/S Cluster: L−3.9−7
L238 (m/z=824.558649; rt=3.92389)
T/S Cluster: L−3.9−7
L241 (m/z=725.445937; rt=3.9393)
T/S Cluster: L−3.9−7
PCo1
PCo2

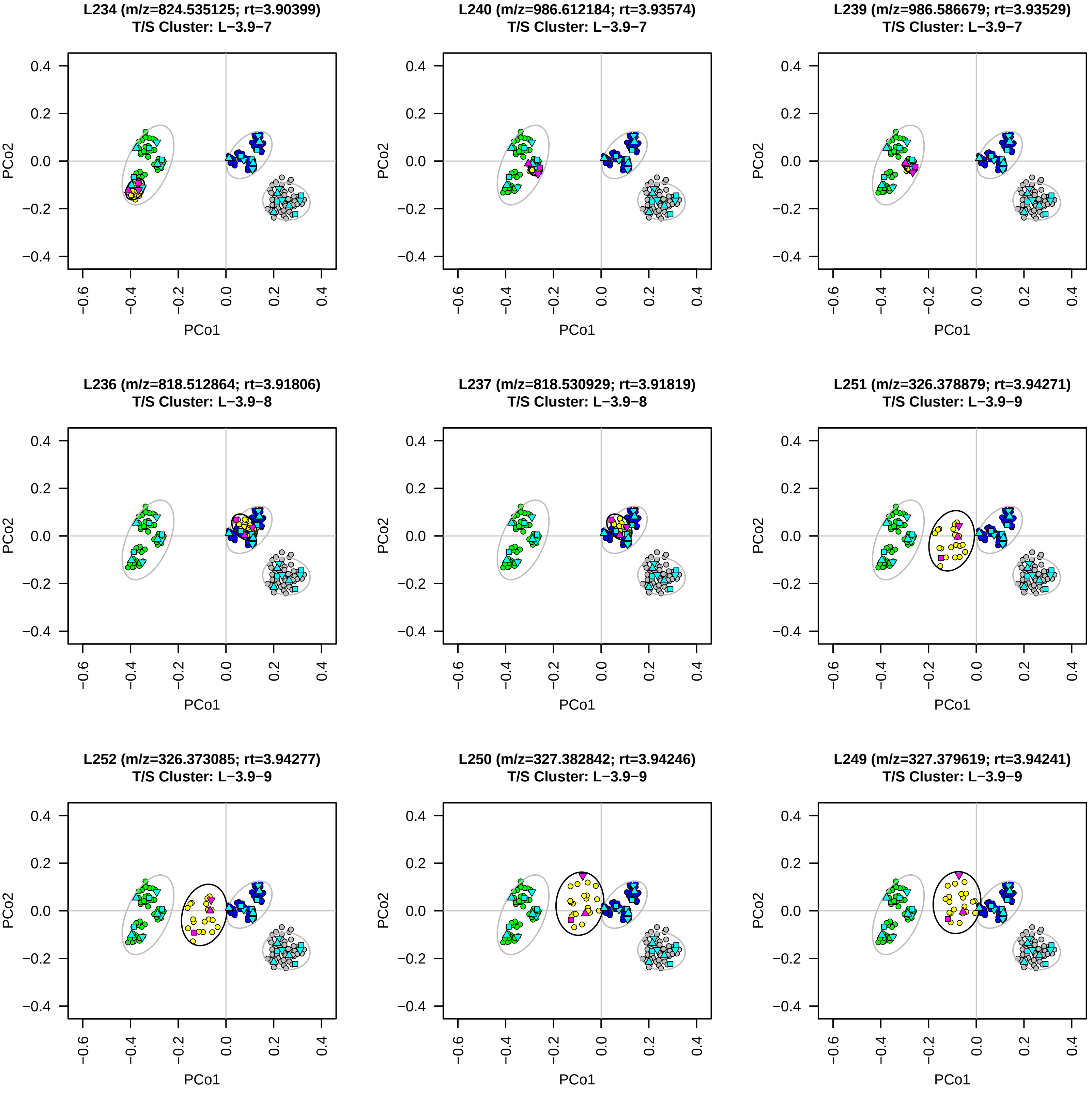
L234 (m/z=824.535125; rt=3.90399)
T/S Cluster: L−3.9−7
L240 (m/z=986.612184; rt=3.93574)
T/S Cluster: L−3.9−7
L239 (m/z=986.586679; rt=3.93529)
T/S Cluster: L−3.9−7
L236 (m/z=818.512864; rt=3.91806)
T/S Cluster: L−3.9−8
L237 (m/z=818.530929; rt=3.91819)
T/S Cluster: L−3.9−8
L251 (m/z=326.378879; rt=3.94271)
T/S Cluster: L−3.9−9
L252 (m/z=326.373085; rt=3.94277)
T/S Cluster: L−3.9−9
L250 (m/z=327.382842; rt=3.94246)
T/S Cluster: L−3.9−9
L249 (m/z=327.379619; rt=3.94241)
T/S Cluster: L−3.9−9
PCo1
PCo2
0.4
0.2
0.0
−0.2
−0.4
−0.6

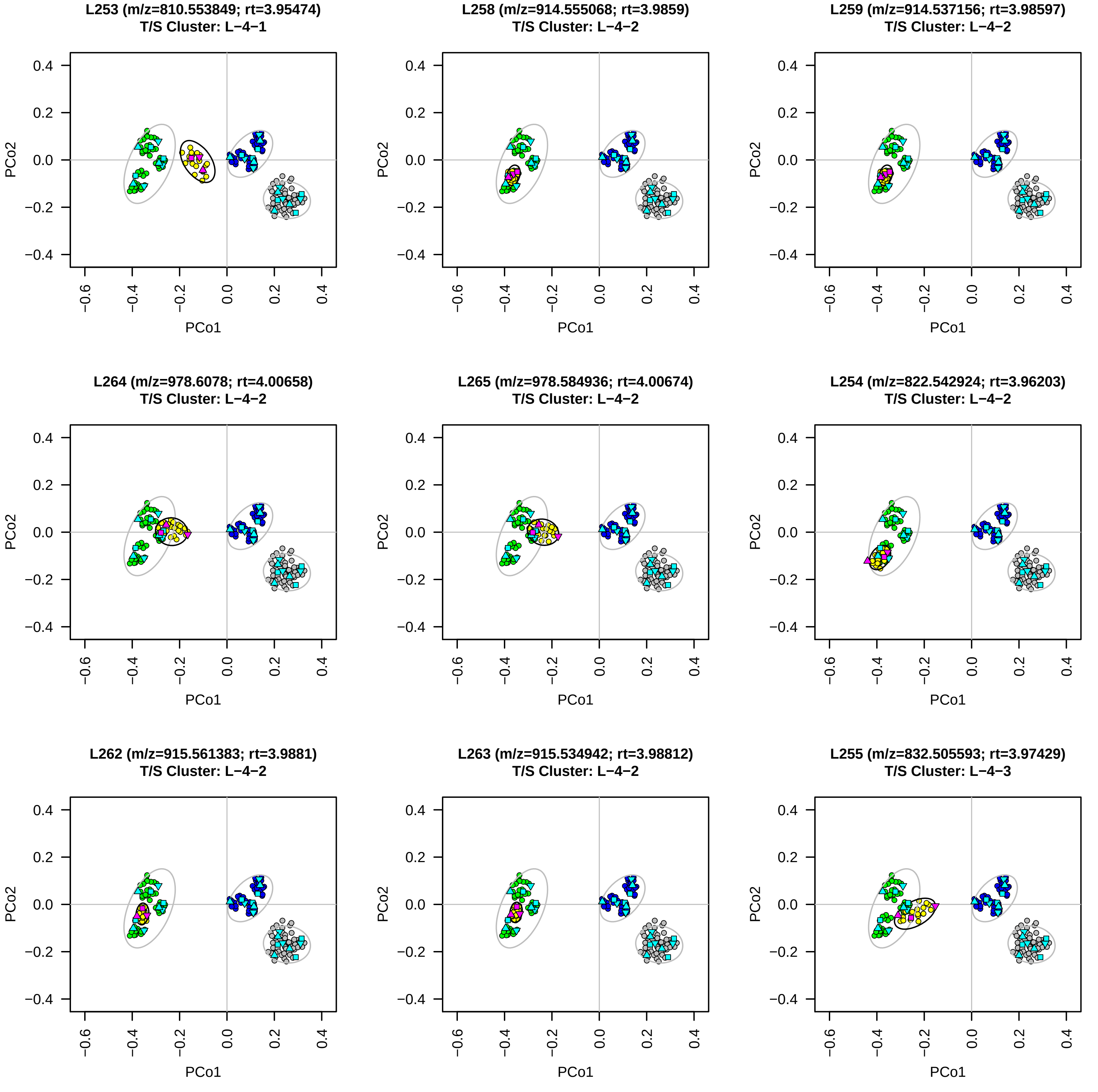
L253 (m/z=810.553849; rt=3.95474)
T/S Cluster: L−4−1
L258 (m/z=914.555068; rt=3.9859)
T/S Cluster: L−4−2
L259 (m/z=914.537156; rt=3.98597)
T/S Cluster: L−4−2
L264 (m/z=978.6078; rt=4.00658)
T/S Cluster: L−4−2
L265 (m/z=978.584936; rt=4.00674)
T/S Cluster: L−4−2
L254 (m/z=822.542924; rt=3.96203)
T/S Cluster: L−4−2
L262 (m/z=915.561383; rt=3.9881)
T/S Cluster: L−4−2
L263 (m/z=915.534942; rt=3.98812)
T/S Cluster: L−4−2
L255 (m/z=832.505593; rt=3.97429)
T/S Cluster: L−4−3
PCo1
PCo2
0.4
0.2
0.0
−0.2
−0.4
−0.6

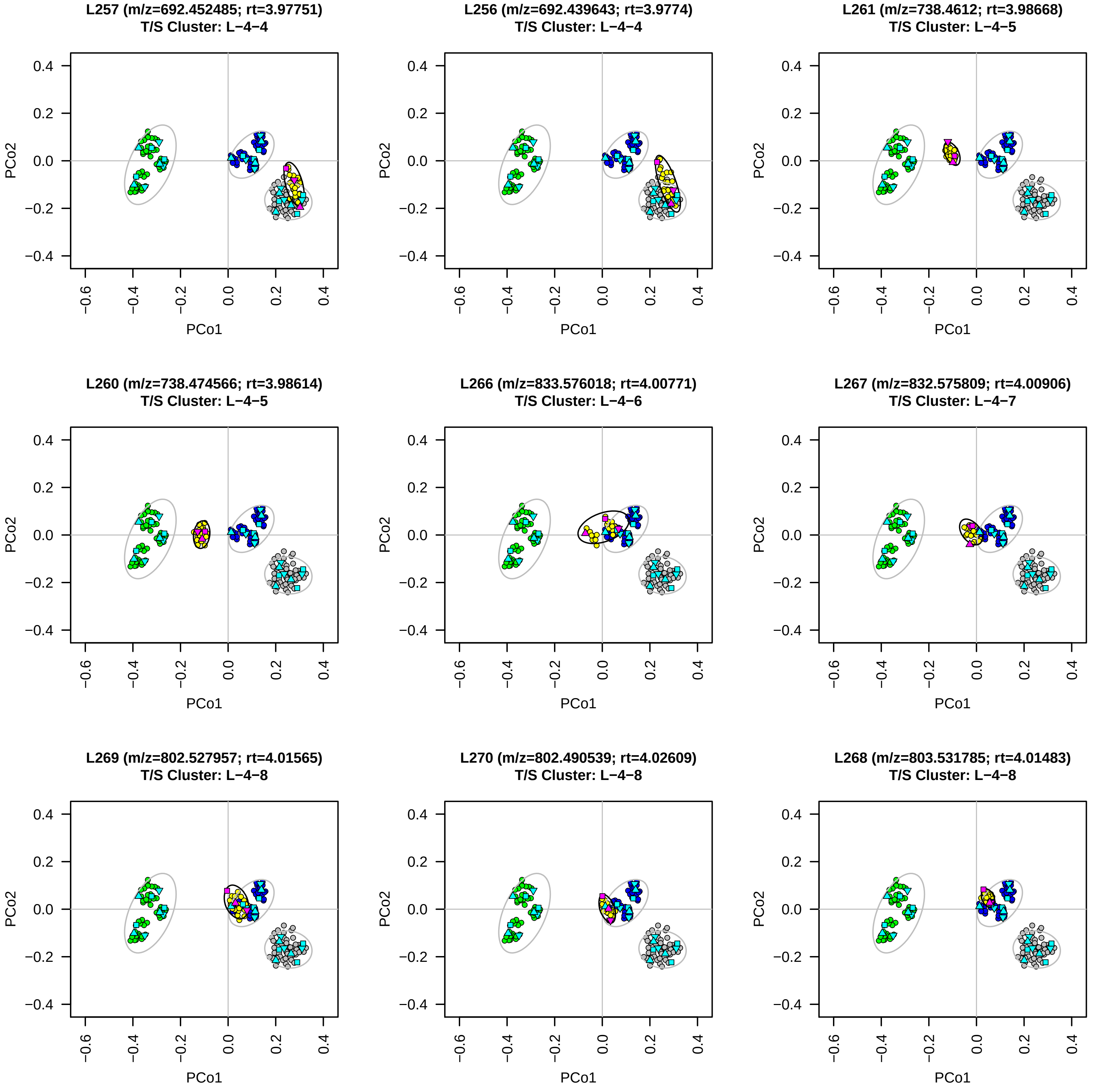

L257 (m/z=692.452485; rt=3.97751)
T/S Cluster: L−4−4
L256 (m/z=692.439643; rt=3.9774)
T/S Cluster: L−4−4
L261 (m/z=738.4612; rt=3.98668)
T/S Cluster: L−4−5
L260 (m/z=738.474566; rt=3.98614)
T/S Cluster: L−4−5
L266 (m/z=833.576018; rt=4.00771)
T/S Cluster: L−4−6
L267 (m/z=832.575809; rt=4.00906)
T/S Cluster: L−4−7
L269 (m/z=802.527957; rt=4.01565)
T/S Cluster: L−4−8
L270 (m/z=802.490539; rt=4.02609)
T/S Cluster: L−4−8
L268 (m/z=803.531785; rt=4.01483)
T/S Cluster: L−4−8
PCo1
PCo2

L271 (m/z=804.553755; rt=4.03316)
T/S Cluster: L−4−9

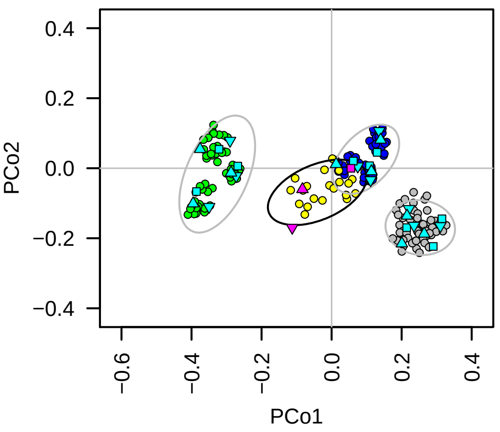

L272 (m/z=826.524257; rt=4.05438)
T/S Cluster: L−4.1−1

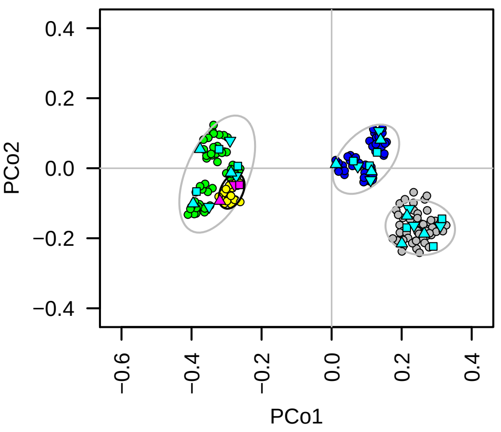

L274 (m/z=826.510338; rt=4.05705)
T/S Cluster: L−4.1−1

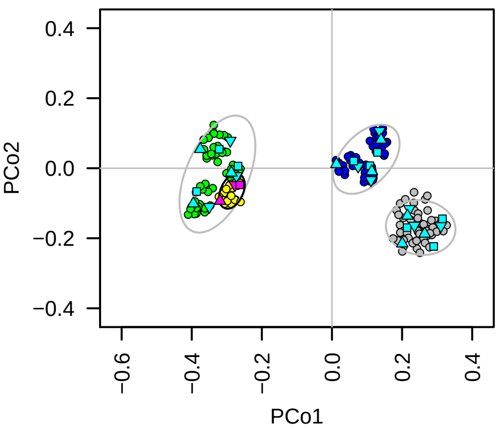

L276 (m/z=827.528072; rt=4.06331)
T/S Cluster: L−4.1−1

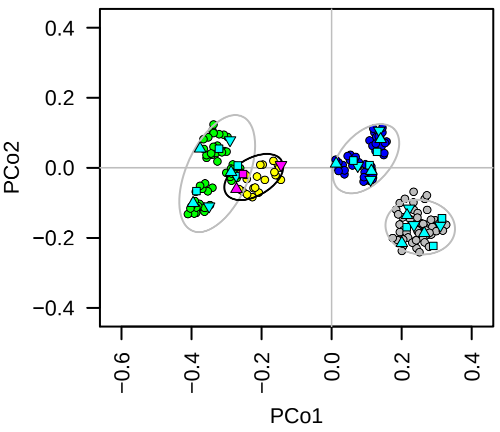

L275 (m/z=796.525848; rt=4.06058)
T/S Cluster: L−4.1−1

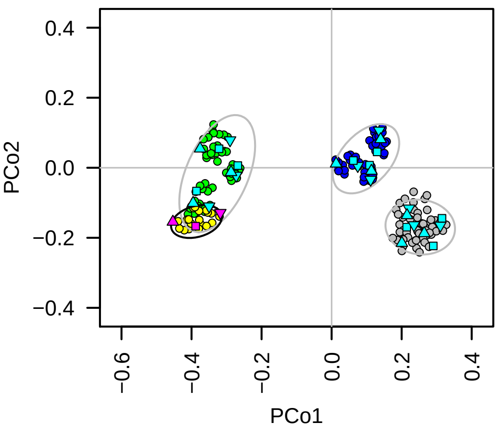

L273 (m/z=801.480595; rt=4.05539)
T/S Cluster: L−4.1−1

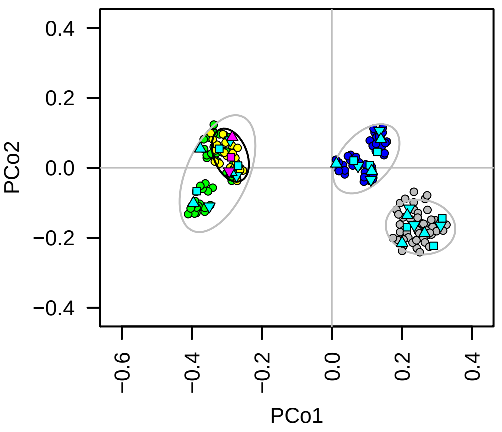

L281 (m/z=650.443074; rt=4.09566)
T/S Cluster: L−4.1−2

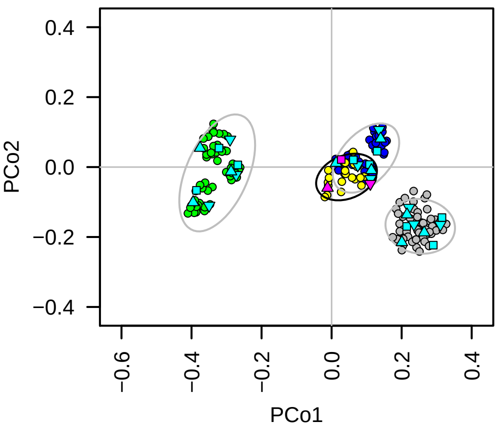

L280 (m/z=650.422665; rt=4.09564)
T/S Cluster: L−4.1−2

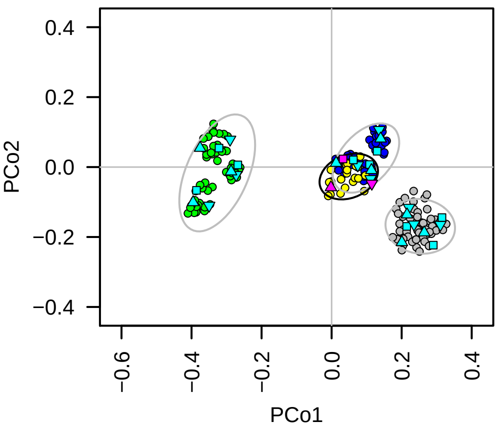

L285 (m/z=651.447207; rt=4.09576)
T/S Cluster: L−4.1−2

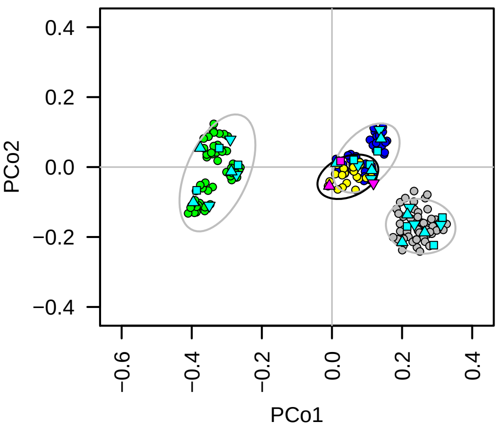

L284 (m/z=651.42864; rt=4.09574)
T/S Cluster: L−4.1−2

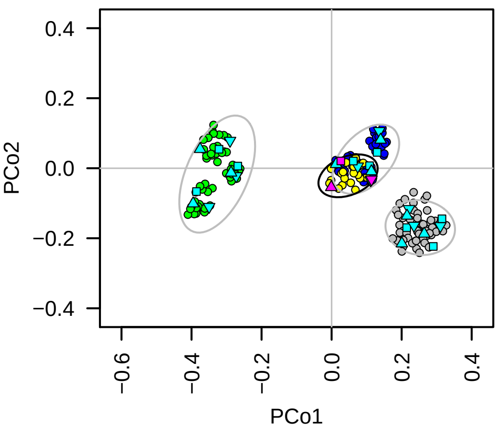

L282 (m/z=325.219619; rt=4.09568)
T/S Cluster: L−4.1−2

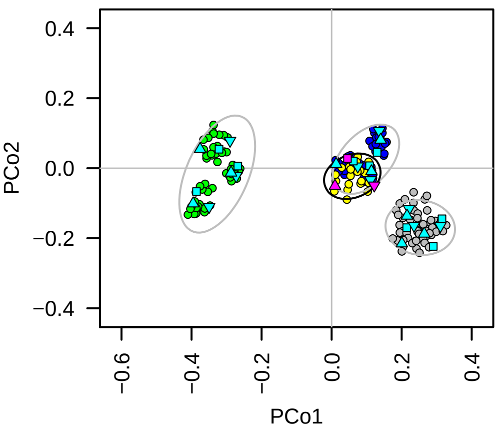

L283 (m/z=325.223766; rt=4.09569)
T/S Cluster: L−4.1−2

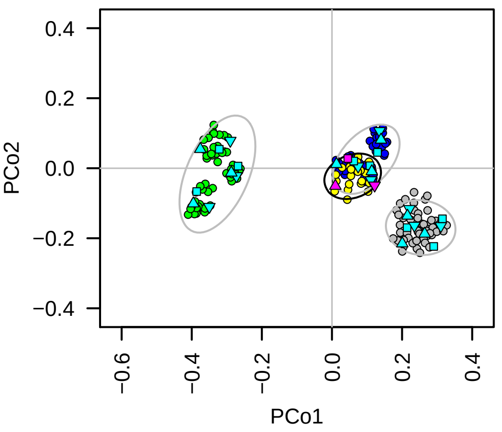

L277 (m/z=662.44363; rt=4.07434)
T/S Cluster: L−4.1−2

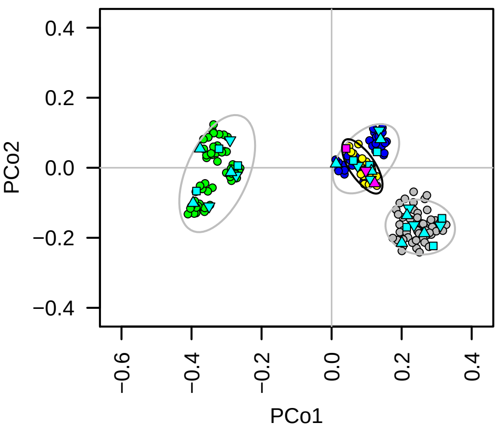

L278 (m/z=662.427147; rt=4.07558)
T/S Cluster: L−4.1−2

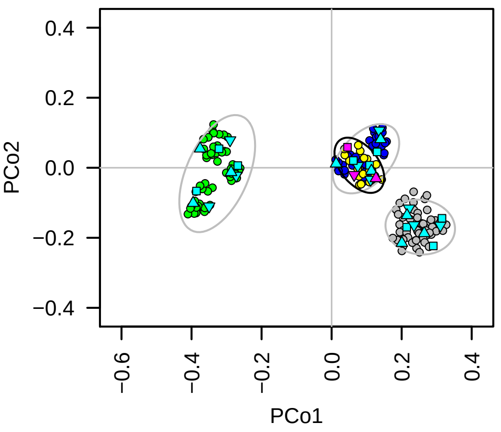

L286 (m/z=652.448069; rt=4.0958)
T/S Cluster: L−4.1−2

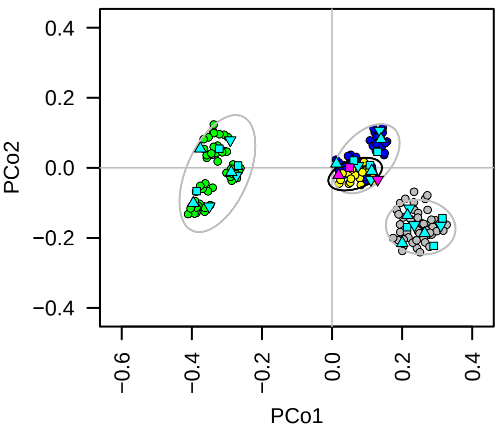

L287 (m/z=325.723394; rt=4.09595)
T/S Cluster: L−4.1−2

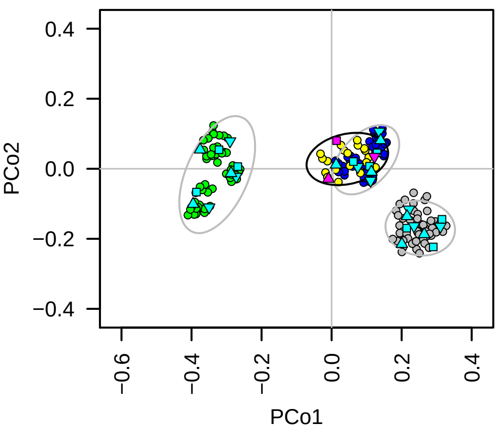

L288 (m/z=216.816679; rt=4.09616)
T/S Cluster: L−4.1−2

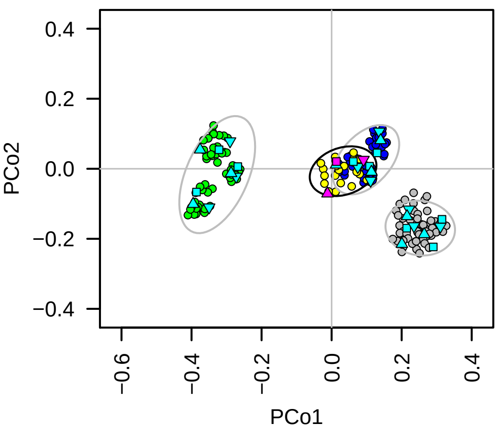

L289 (m/z=216.814993; rt=4.09623)
T/S Cluster: L−4.1−2

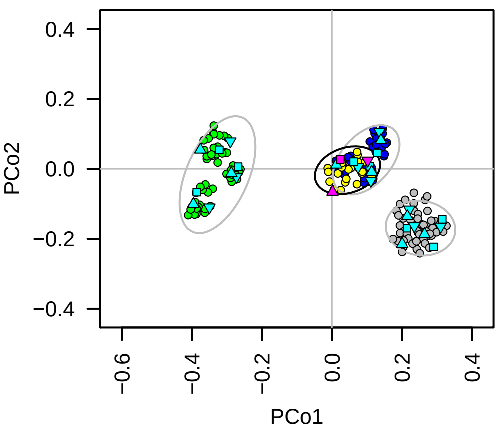

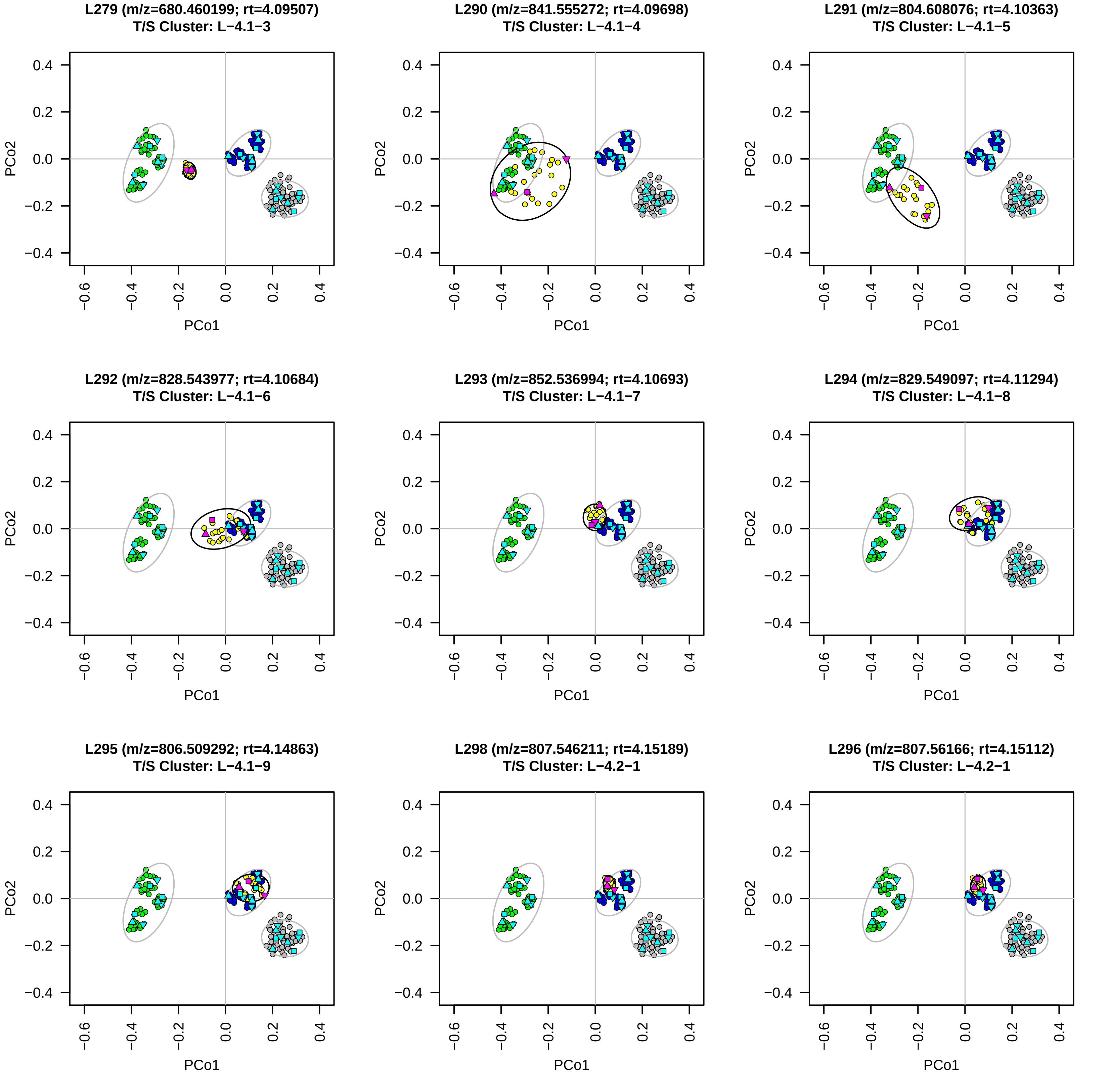
L279 (m/z=680.460199; rt=4.09507)
T/S Cluster: L−4.1−3
L290 (m/z=841.555272; rt=4.09698)
T/S Cluster: L−4.1−4
L291 (m/z=804.608076; rt=4.10363)
T/S Cluster: L−4.1−5
L292 (m/z=828.543977; rt=4.10684)
T/S Cluster: L−4.1−6
L293 (m/z=852.536994; rt=4.10693)
T/S Cluster: L−4.1−7
L294 (m/z=829.549097; rt=4.11294)
T/S Cluster: L−4.1−8
L295 (m/z=806.509292; rt=4.14863)
T/S Cluster: L−4.1−9
L298 (m/z=807.546211; rt=4.15189)
T/S Cluster: L−4.2−1
L296 (m/z=807.56166; rt=4.15112)
T/S Cluster: L−4.2−1
PCo1
PCo2

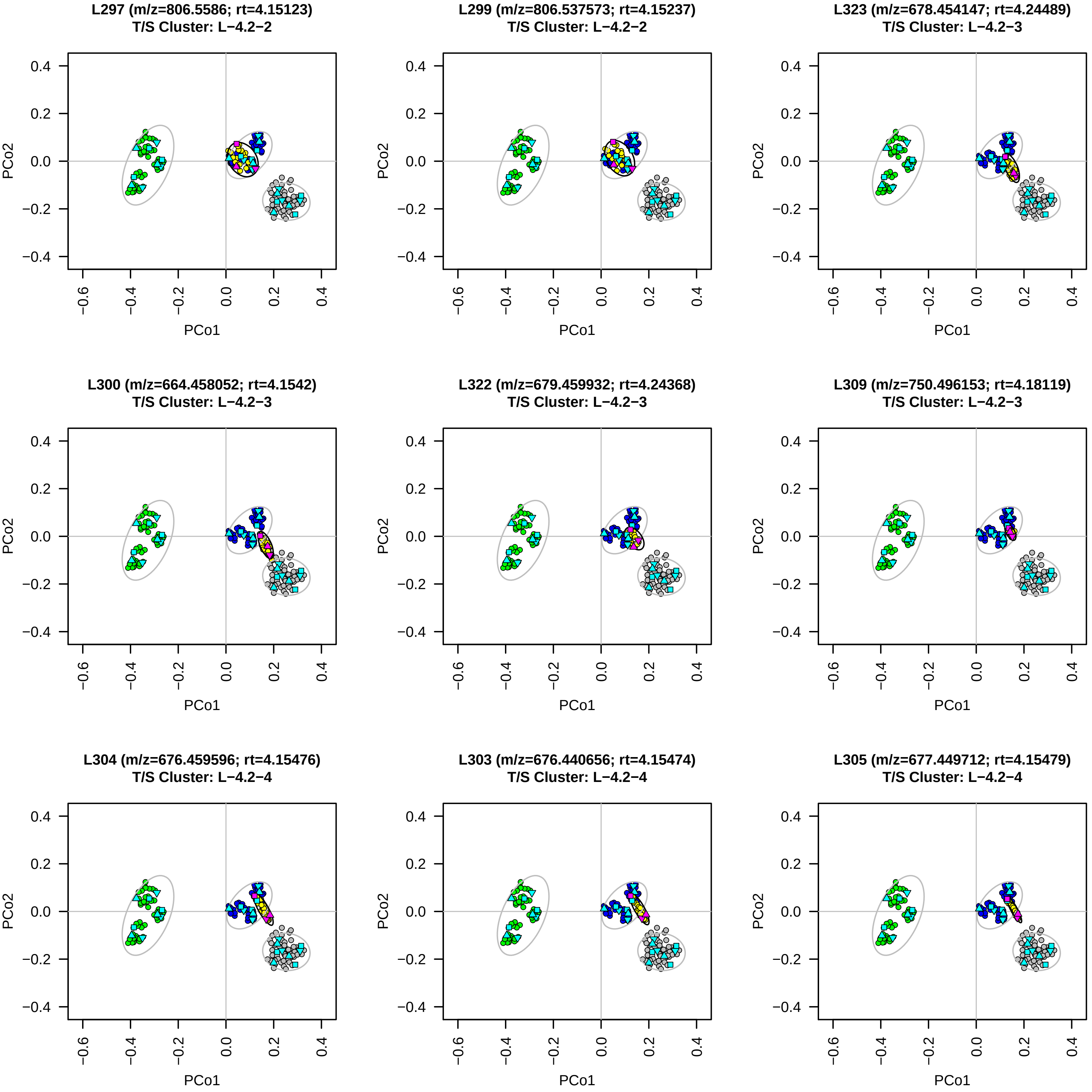

L297 (m/z=806.5586; rt=4.15123)
T/S Cluster: L−4.2−2
L299 (m/z=806.537573; rt=4.15237)
T/S Cluster: L−4.2−2
L323 (m/z=678.454147; rt=4.24489)
T/S Cluster: L−4.2−3
L300 (m/z=664.458052; rt=4.1542)
T/S Cluster: L−4.2−3
L322 (m/z=679.459932; rt=4.24368)
T/S Cluster: L−4.2−3
L309 (m/z=750.496153; rt=4.18119)
T/S Cluster: L−4.2−3
L304 (m/z=676.459596; rt=4.15476)
T/S Cluster: L−4.2−4
L303 (m/z=676.440656; rt=4.15474)
T/S Cluster: L−4.2−4
L305 (m/z=677.449712; rt=4.15479)
T/S Cluster: L−4.2−4
PCo1
PCo2

L306 (m/z=677.465102; rt=4.15481)
T/S Cluster: L−4.2−4

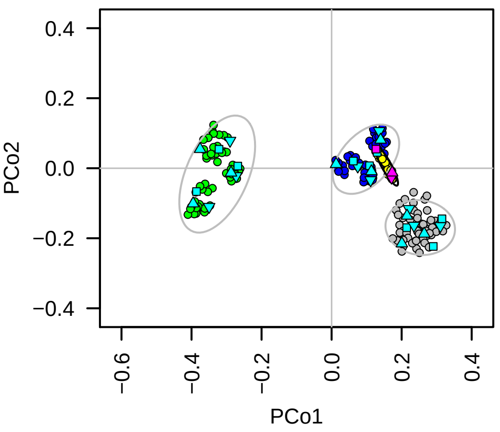

L315 (m/z=692.453165; rt=4.21553)
T/S Cluster: L−4.2−4

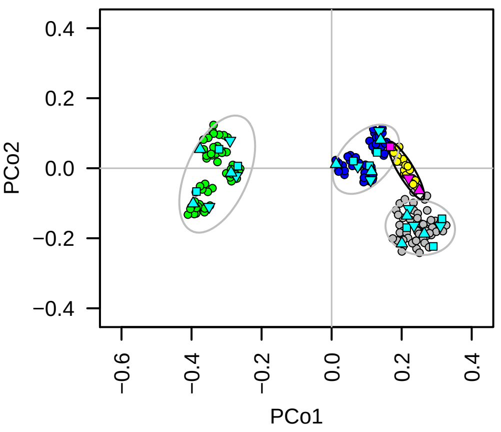

L314 (m/z=692.440741; rt=4.21528)
T/S Cluster: L−4.2−4

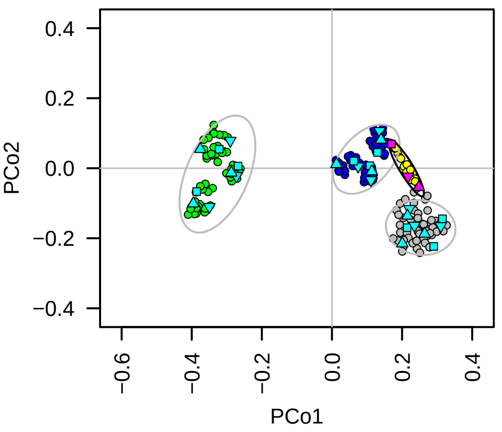

L301 (m/z=338.229676; rt=4.15457)
T/S Cluster: L−4.2−4

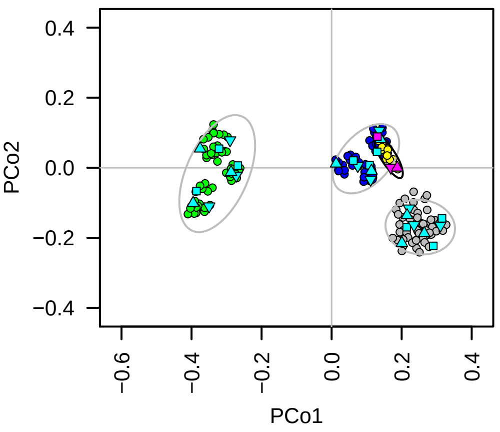

L307 (m/z=867.539361; rt=4.15645)
T/S Cluster: L−4.2−5

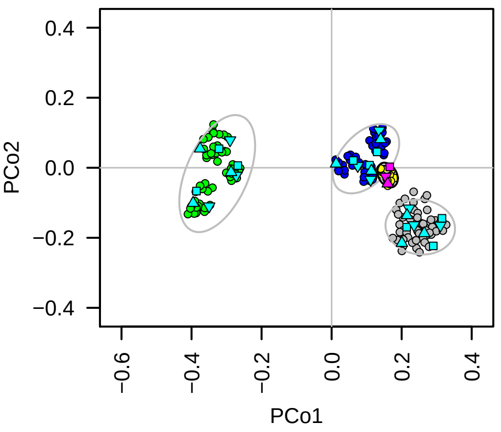

L302 (m/z=868.542251; rt=4.15472)
T/S Cluster: L−4.2−5

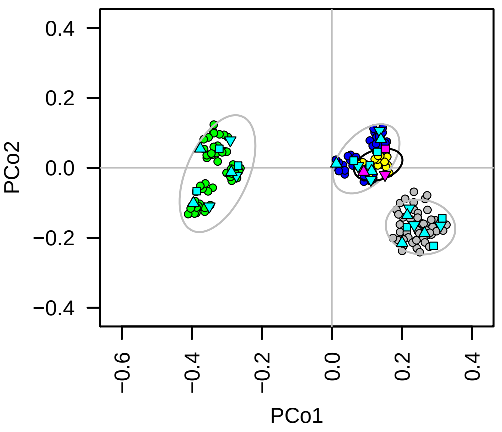

L308 (m/z=830.557181; rt=4.17212)
T/S Cluster: L−4.2−6

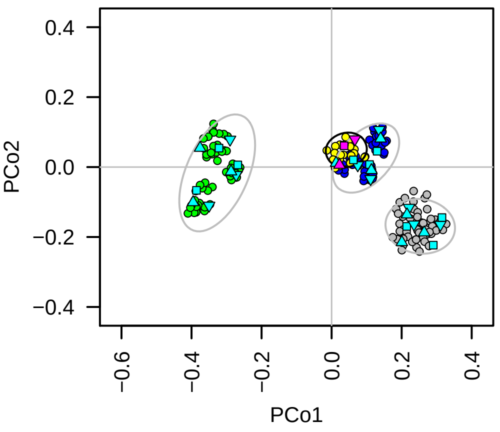

L311 (m/z=810.535032; rt=4.18448)
T/S Cluster: L−4.2−7

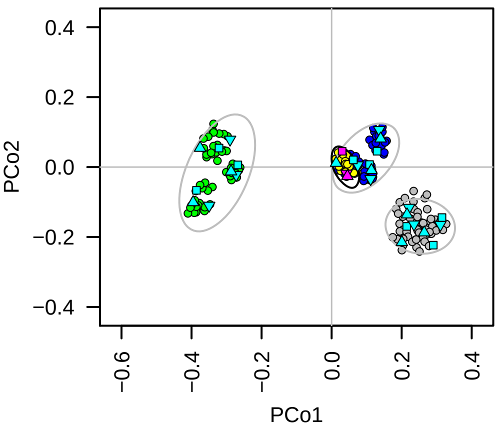

L310 (m/z=810.554037; rt=4.18369)
T/S Cluster: L−4.2−7

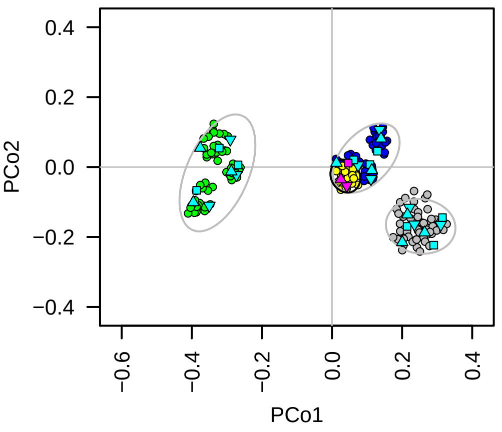

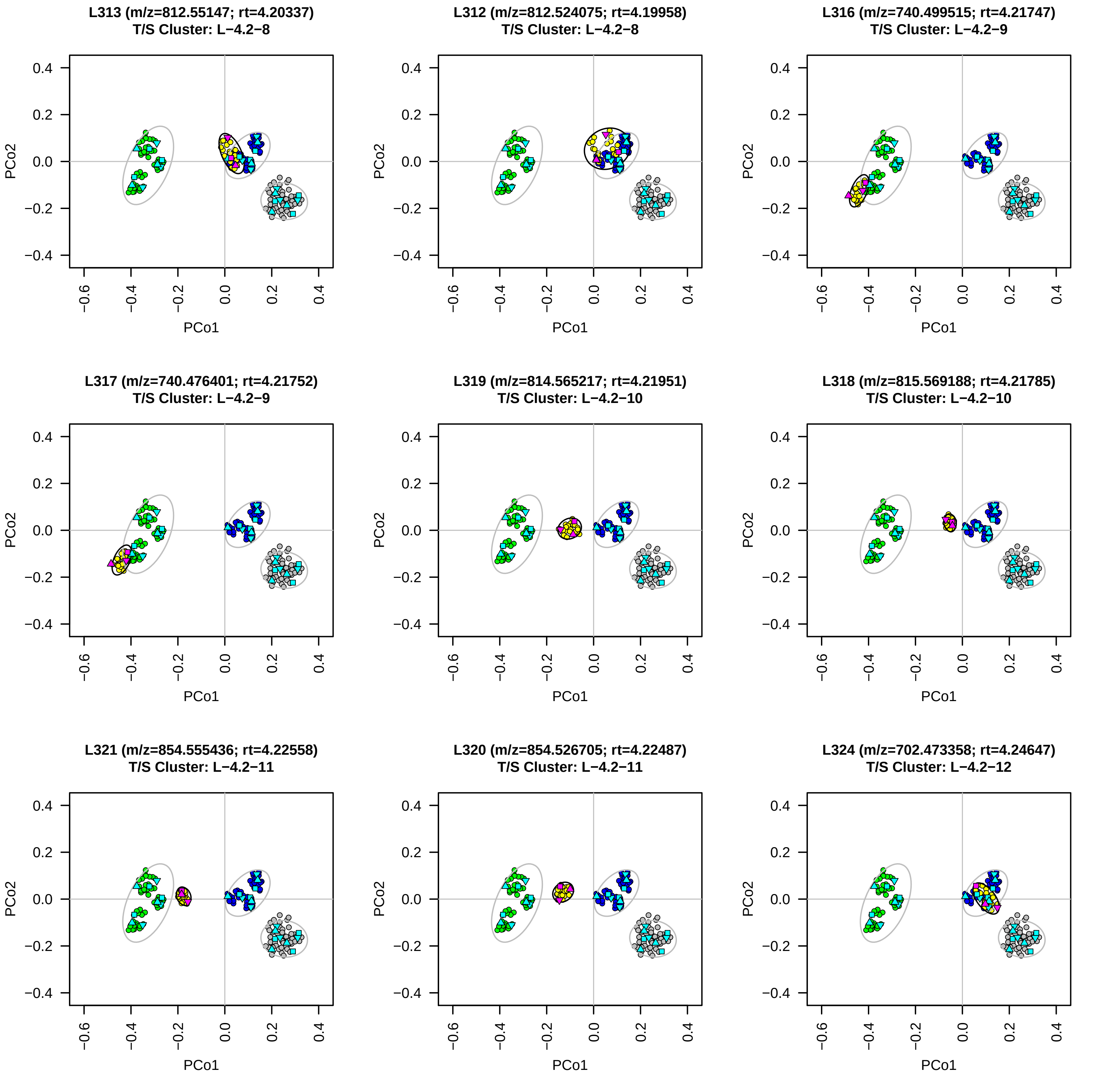
L313 (m/z=812.55147; rt=4.20337)
T/S Cluster: L−4.2−8
L312 (m/z=812.524075; rt=4.19958)
T/S Cluster: L−4.2−8
L316 (m/z=740.499515; rt=4.21747)
T/S Cluster: L−4.2−9
L317 (m/z=740.476401; rt=4.21752)
T/S Cluster: L−4.2−9
L319 (m/z=814.565217; rt=4.21951)
T/S Cluster: L−4.2−10
L318 (m/z=815.569188; rt=4.21785)
T/S Cluster: L−4.2−10
L321 (m/z=854.555436; rt=4.22558)
T/S Cluster: L−4.2−11
L320 (m/z=854.526705; rt=4.22487)
T/S Cluster: L−4.2−11
L324 (m/z=702.473358; rt=4.24647)
T/S Cluster: L−4.2−12
PCo1
PCo2
−0.6
−0.4
−0.2
0.0
0.2
0.4

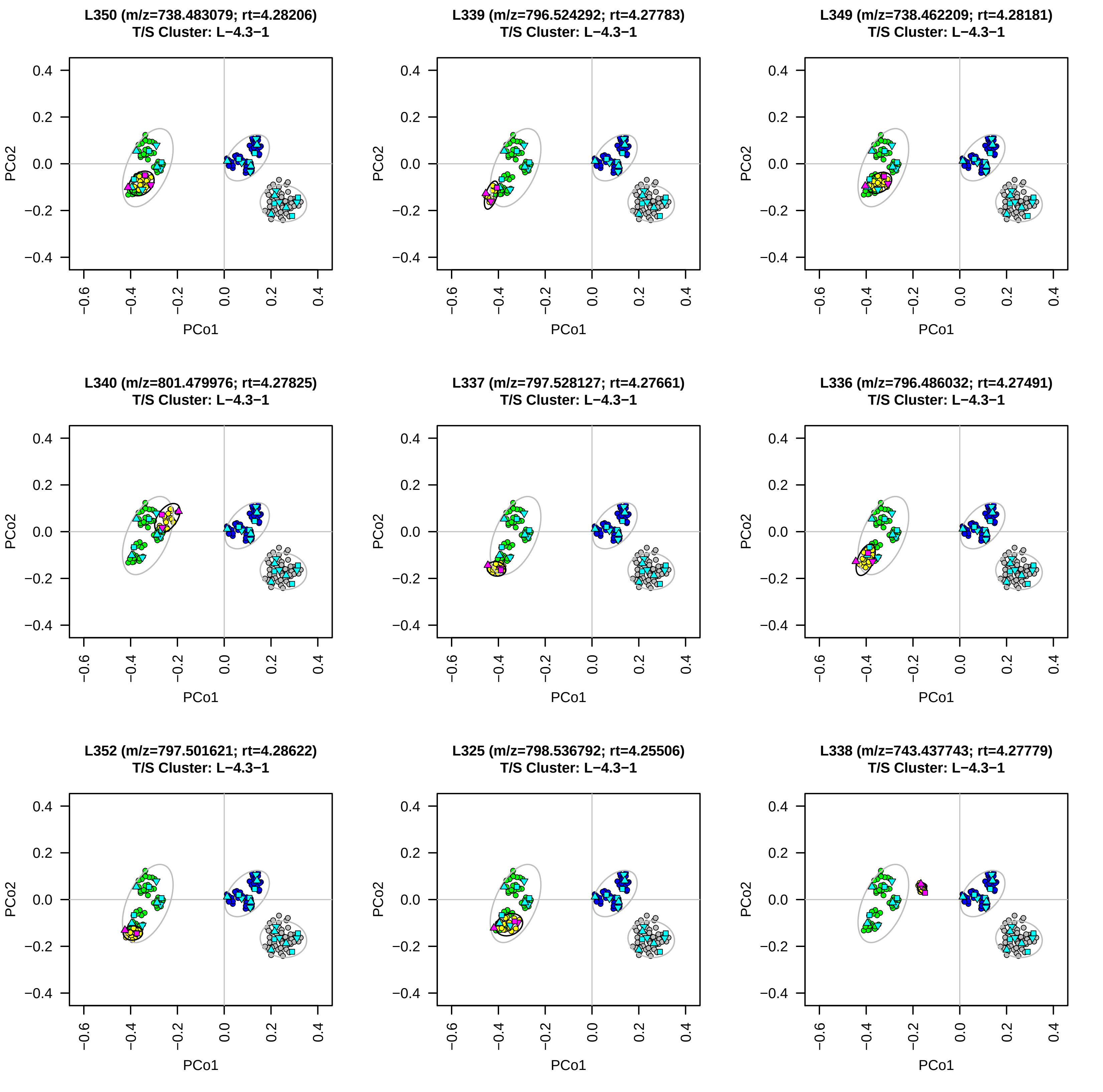
L350 (m/z=738.483079; rt=4.28206)
T/S Cluster: L−4.3−1
L339 (m/z=796.524292; rt=4.27783)
T/S Cluster: L−4.3−1
L349 (m/z=738.462209; rt=4.28181)
T/S Cluster: L−4.3−1
L340 (m/z=801.479976; rt=4.27825)
T/S Cluster: L−4.3−1
L337 (m/z=797.528127; rt=4.27661)
T/S Cluster: L−4.3−1
L336 (m/z=796.486032; rt=4.27491)
T/S Cluster: L−4.3−1
L352 (m/z=797.501621; rt=4.28622)
T/S Cluster: L−4.3−1
L325 (m/z=798.536792; rt=4.25506)
T/S Cluster: L−4.3−1
L338 (m/z=743.437743; rt=4.27779)
T/S Cluster: L−4.3−1
PCo1
PCo2

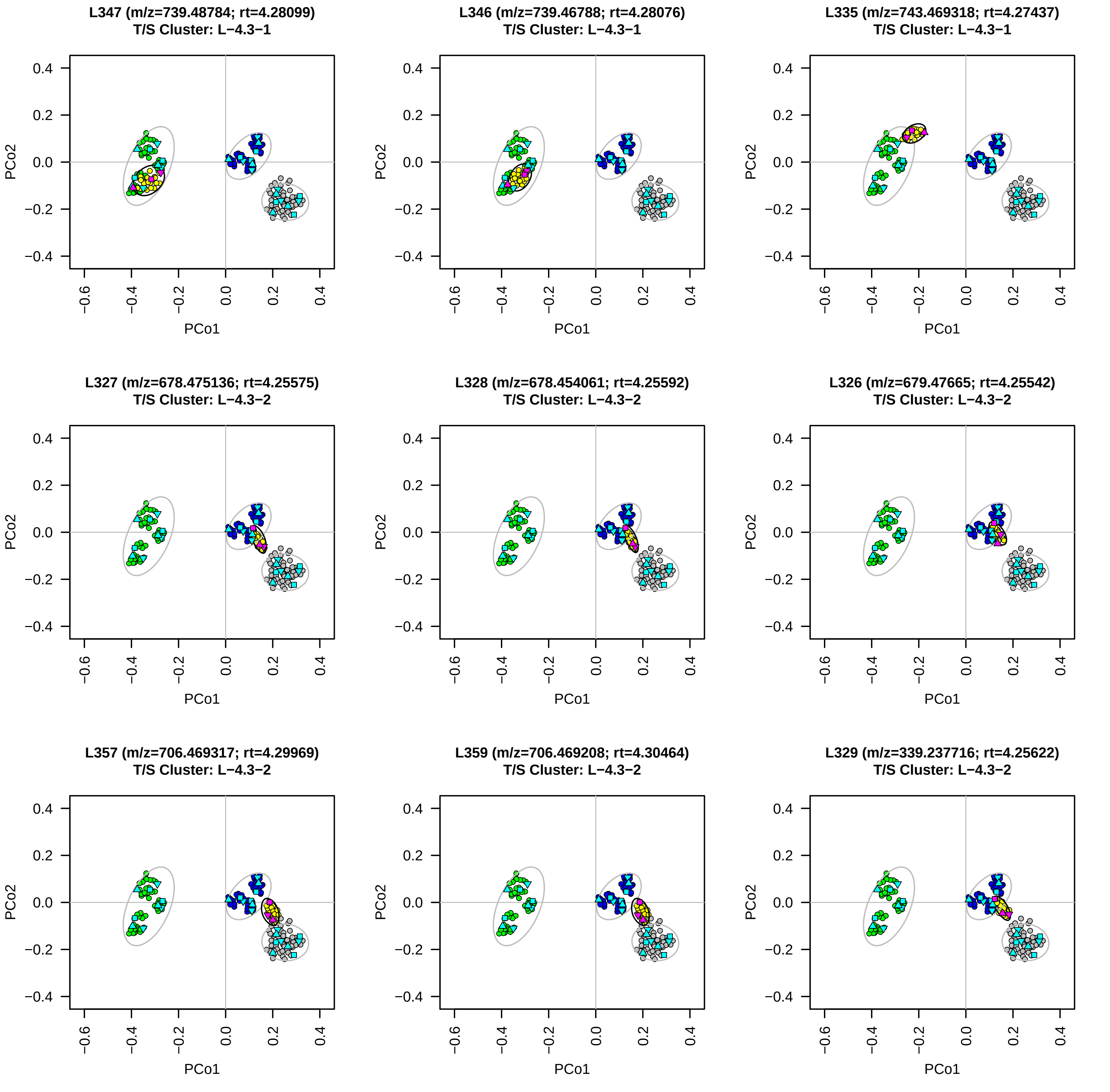
L347 (m/z=739.48784; rt=4.28099)
T/S Cluster: L−4.3−1
L346 (m/z=739.46788; rt=4.28076)
T/S Cluster: L−4.3−1
L335 (m/z=743.469318; rt=4.27437)
T/S Cluster: L−4.3−1
L327 (m/z=678.475136; rt=4.25575)
T/S Cluster: L−4.3−2
L328 (m/z=678.454061; rt=4.25592)
T/S Cluster: L−4.3−2
L326 (m/z=679.47665; rt=4.25542)
T/S Cluster: L−4.3−2
L357 (m/z=706.469317; rt=4.29969)
T/S Cluster: L−4.3−2
L359 (m/z=706.469208; rt=4.30464)
T/S Cluster: L−4.3−2
L329 (m/z=339.237716; rt=4.25622)
T/S Cluster: L−4.3−2
PCo1
PCo2

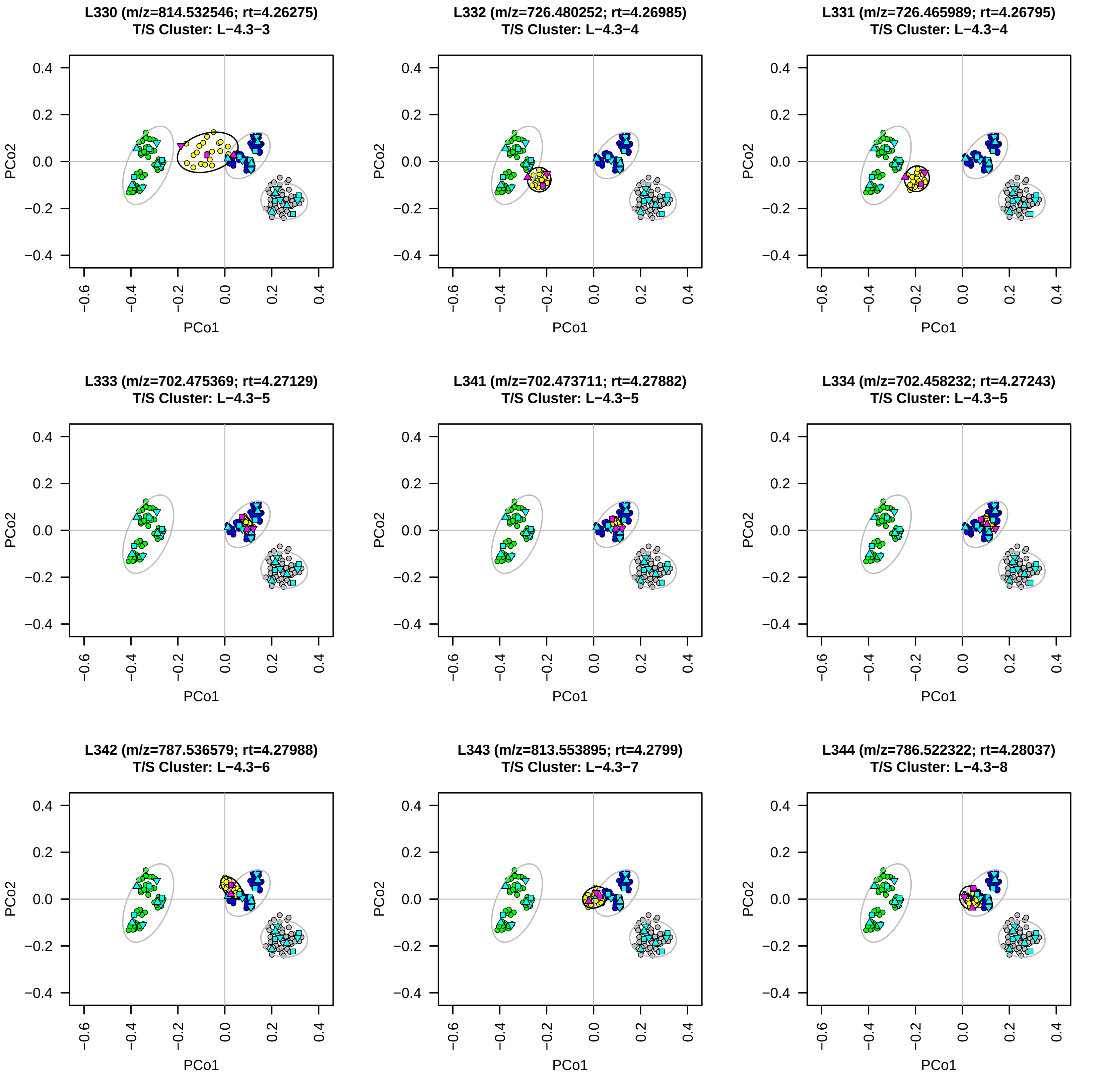
L330 (m/z=814.532546; rt=4.26275)
T/S Cluster: L−4.3−3
L332 (m/z=726.480252; rt=4.26985)
T/S Cluster: L−4.3−4
L331 (m/z=726.465989; rt=4.26795)
T/S Cluster: L−4.3−4
L333 (m/z=702.475369; rt=4.27129)
T/S Cluster: L−4.3−5
L341 (m/z=702.473711; rt=4.27882)
T/S Cluster: L−4.3−5
L334 (m/z=702.458232; rt=4.27243)
T/S Cluster: L−4.3−5
L342 (m/z=787.536579; rt=4.27988)
T/S Cluster: L−4.3−6
L343 (m/z=813.553895; rt=4.2799)
T/S Cluster: L−4.3−7
L344 (m/z=786.522322; rt=4.28037)
T/S Cluster: L−4.3−8
PCo1
PCo2
−0.6
−0.4
−0.2
0.0
0.2
0.4

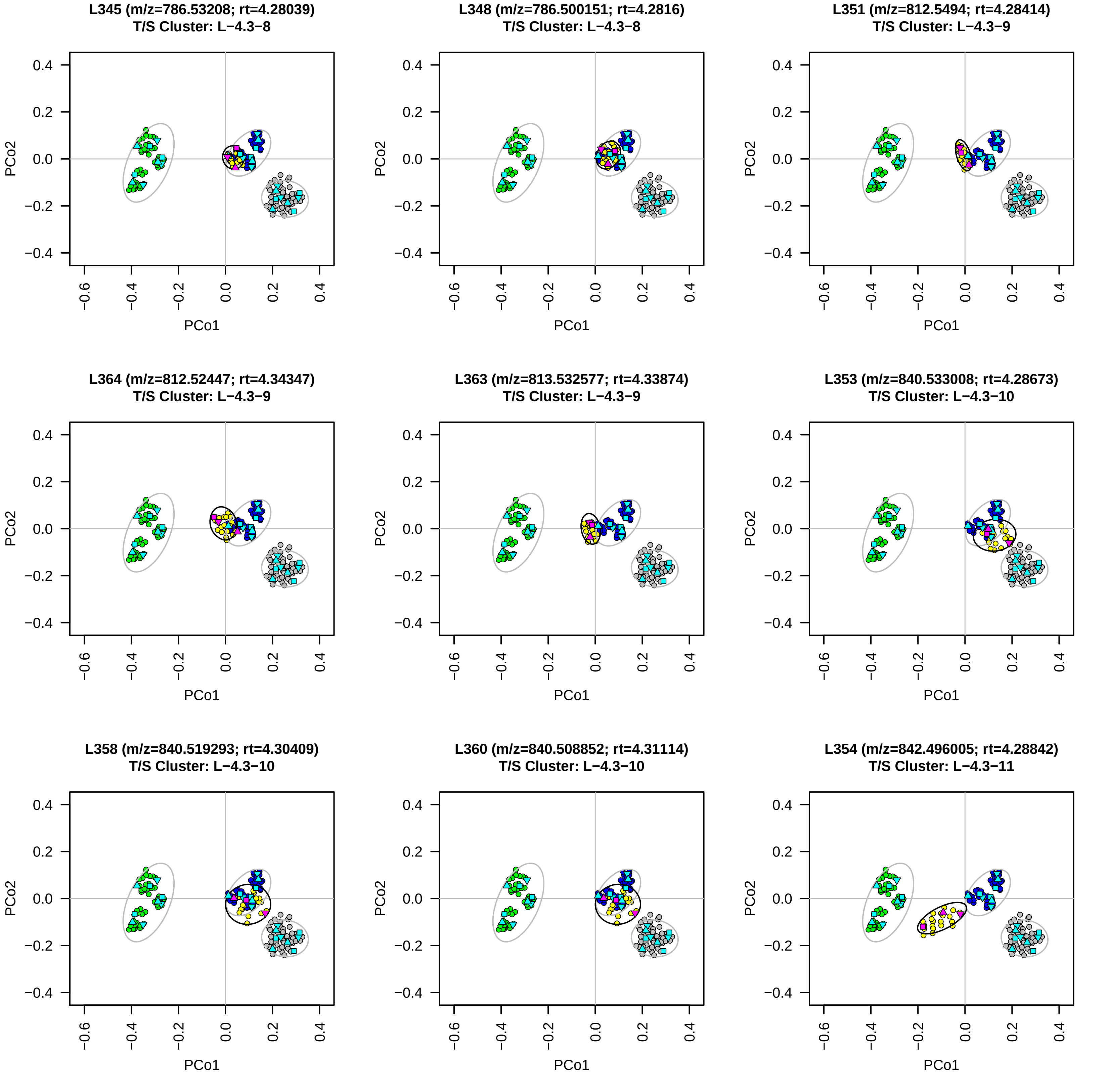

L345 (m/z=786.53208; rt=4.28039)
T/S Cluster: L−4.3−8
L348 (m/z=786.500151; rt=4.2816)
T/S Cluster: L−4.3−8
L351 (m/z=812.5494; rt=4.28414)
T/S Cluster: L−4.3−9
L364 (m/z=812.52447; rt=4.34347)
T/S Cluster: L−4.3−9
L363 (m/z=813.532577; rt=4.33874)
T/S Cluster: L−4.3−9
L353 (m/z=840.533008; rt=4.28673)
T/S Cluster: L−4.3−10
L358 (m/z=840.519293; rt=4.30409)
T/S Cluster: L−4.3−10
L360 (m/z=840.508852; rt=4.31114)
T/S Cluster: L−4.3−10
L354 (m/z=842.496005; rt=4.28842)
T/S Cluster: L−4.3−11
PCo1
PCo2

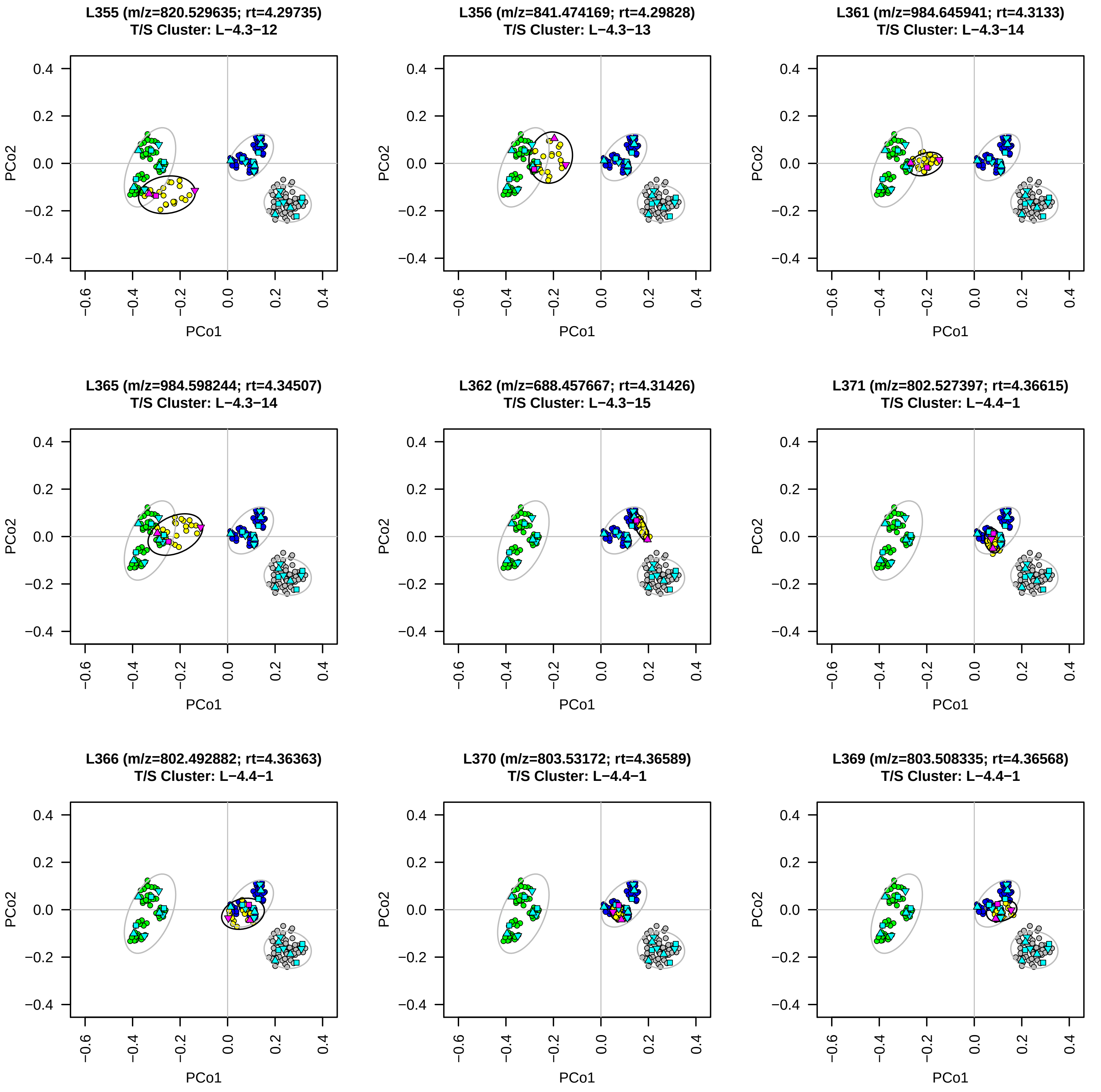
L355 (m/z=820.529635; rt=4.29735)
T/S Cluster: L−4.3−12
L356 (m/z=841.474169; rt=4.29828)
T/S Cluster: L−4.3−13
L361 (m/z=984.645941; rt=4.3133)
T/S Cluster: L−4.3−14
L365 (m/z=984.598244; rt=4.34507)
T/S Cluster: L−4.3−14
L362 (m/z=688.457667; rt=4.31426)
T/S Cluster: L−4.3−15
L371 (m/z=802.527397; rt=4.36615)
T/S Cluster: L−4.4−1
L366 (m/z=802.492882; rt=4.36363)
T/S Cluster: L−4.4−1
L370 (m/z=803.53172; rt=4.36589)
T/S Cluster: L−4.4−1
L369 (m/z=803.508335; rt=4.36568)
T/S Cluster: L−4.4−1
PCo1
PCo2
−0.6
−0.4
−0.2
0.0
0.2
0.4

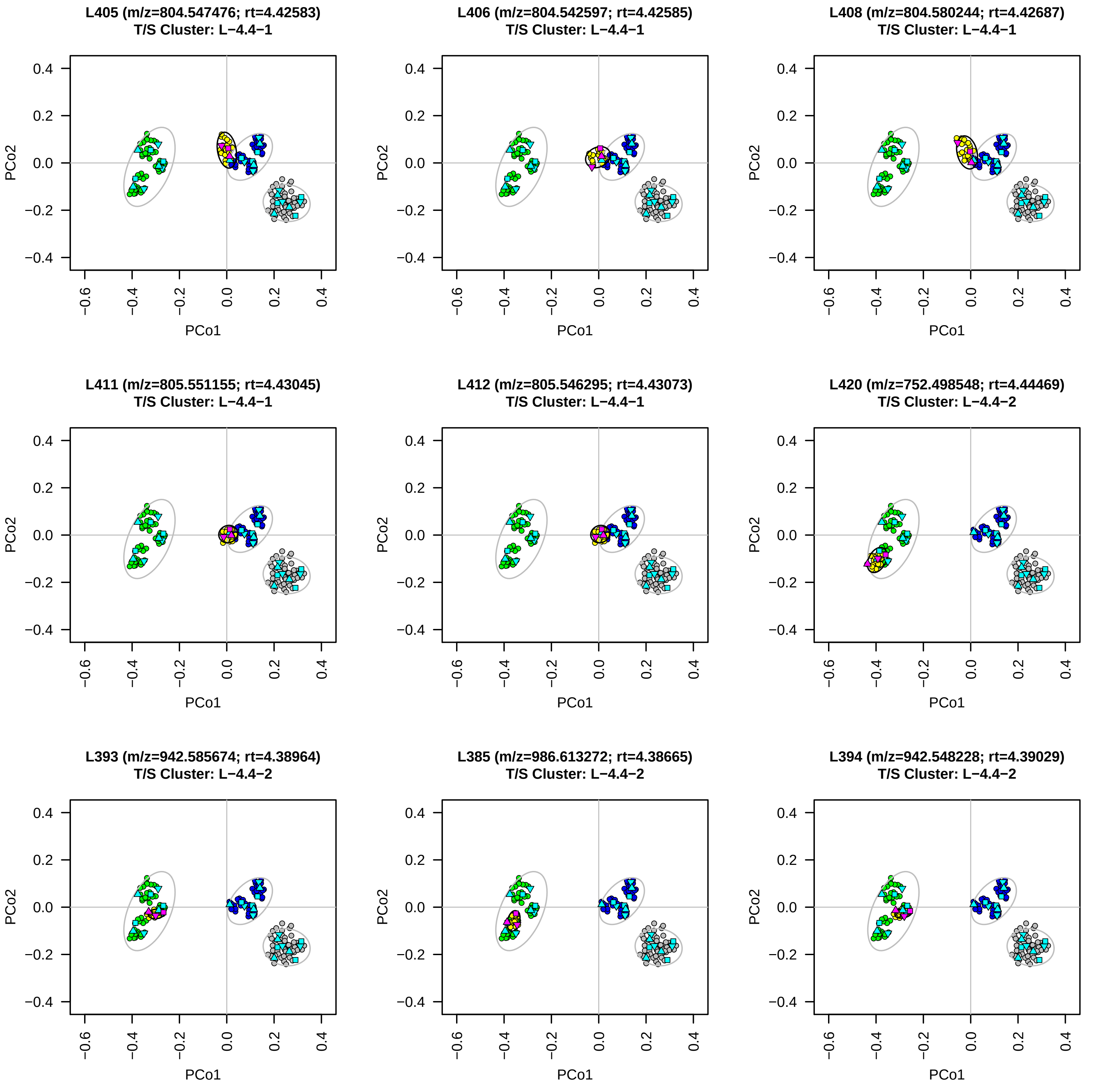
L405 (m/z=804.547476; rt=4.42583)
T/S Cluster: L−4.4−1
L406 (m/z=804.542597; rt=4.42585)
T/S Cluster: L−4.4−1
L408 (m/z=804.580244; rt=4.42687)
T/S Cluster: L−4.4−1
L411 (m/z=805.551155; rt=4.43045)
T/S Cluster: L−4.4−1
L412 (m/z=805.546295; rt=4.43073)
T/S Cluster: L−4.4−1
L420 (m/z=752.498548; rt=4.44469)
T/S Cluster: L−4.4−2
L393 (m/z=942.585674; rt=4.38964)
T/S Cluster: L−4.4−2
L385 (m/z=986.613272; rt=4.38665)
T/S Cluster: L−4.4−2
L394 (m/z=942.548228; rt=4.39029)
T/S Cluster: L−4.4−2
PCo1
PCo2
0.4
0.2
0.0
−0.2
−0.4
−0.6

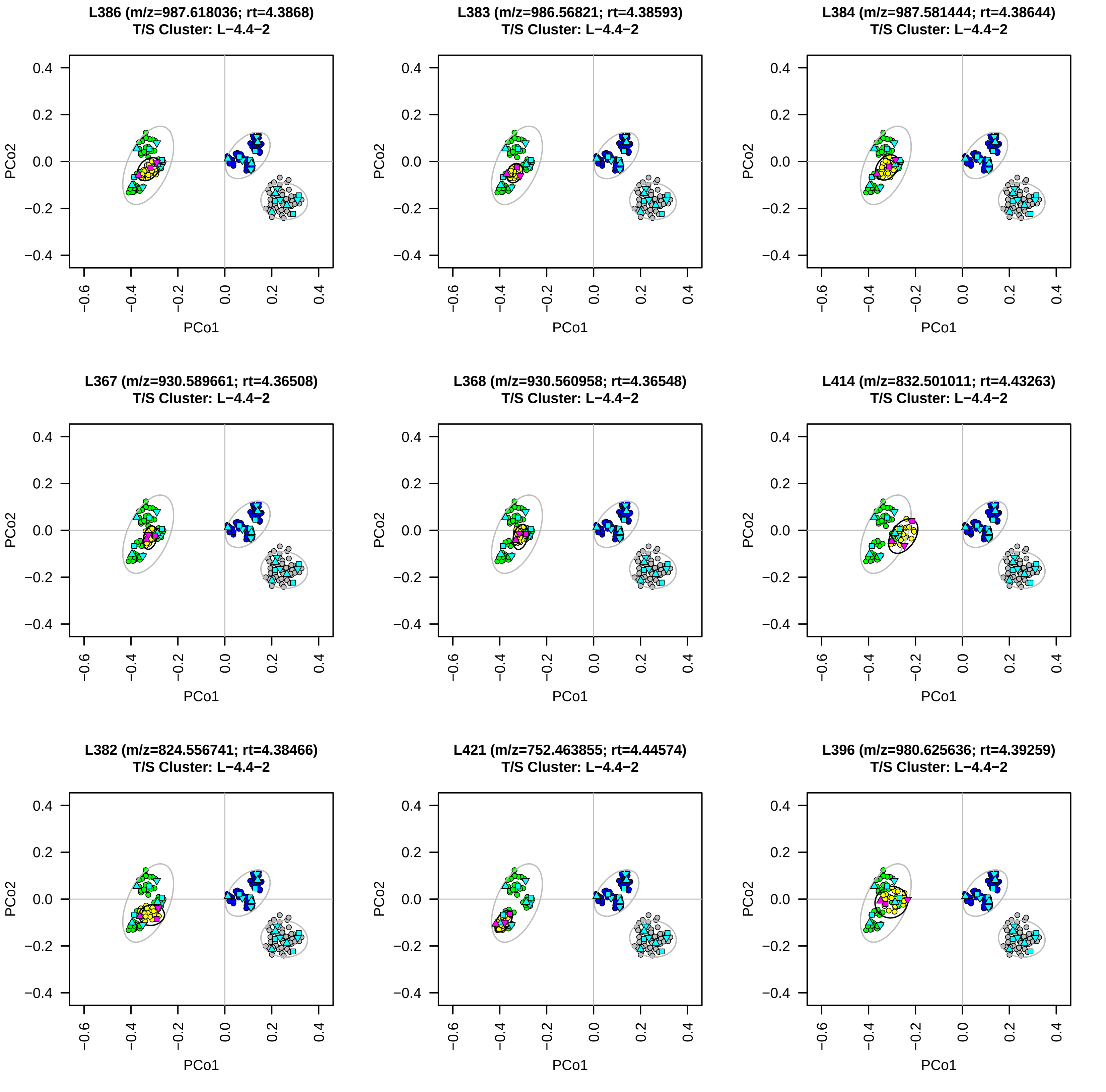
L386 (m/z=987.618036; rt=4.3868)
T/S Cluster: L−4.4−2
L383 (m/z=986.56821; rt=4.38593)
T/S Cluster: L−4.4−2
L384 (m/z=987.581444; rt=4.38644)
T/S Cluster: L−4.4−2
L367 (m/z=930.589661; rt=4.36508)
T/S Cluster: L−4.4−2
L368 (m/z=930.560958; rt=4.36548)
T/S Cluster: L−4.4−2
L414 (m/z=832.501011; rt=4.43263)
T/S Cluster: L−4.4−2
L382 (m/z=824.556741; rt=4.38466)
T/S Cluster: L−4.4−2
L421 (m/z=752.463855; rt=4.44574)
T/S Cluster: L−4.4−2
L396 (m/z=980.625636; rt=4.39259)
T/S Cluster: L−4.4−2
PCo1
PCo2
−0.6
−0.4
−0.2
0.0
0.2
0.4

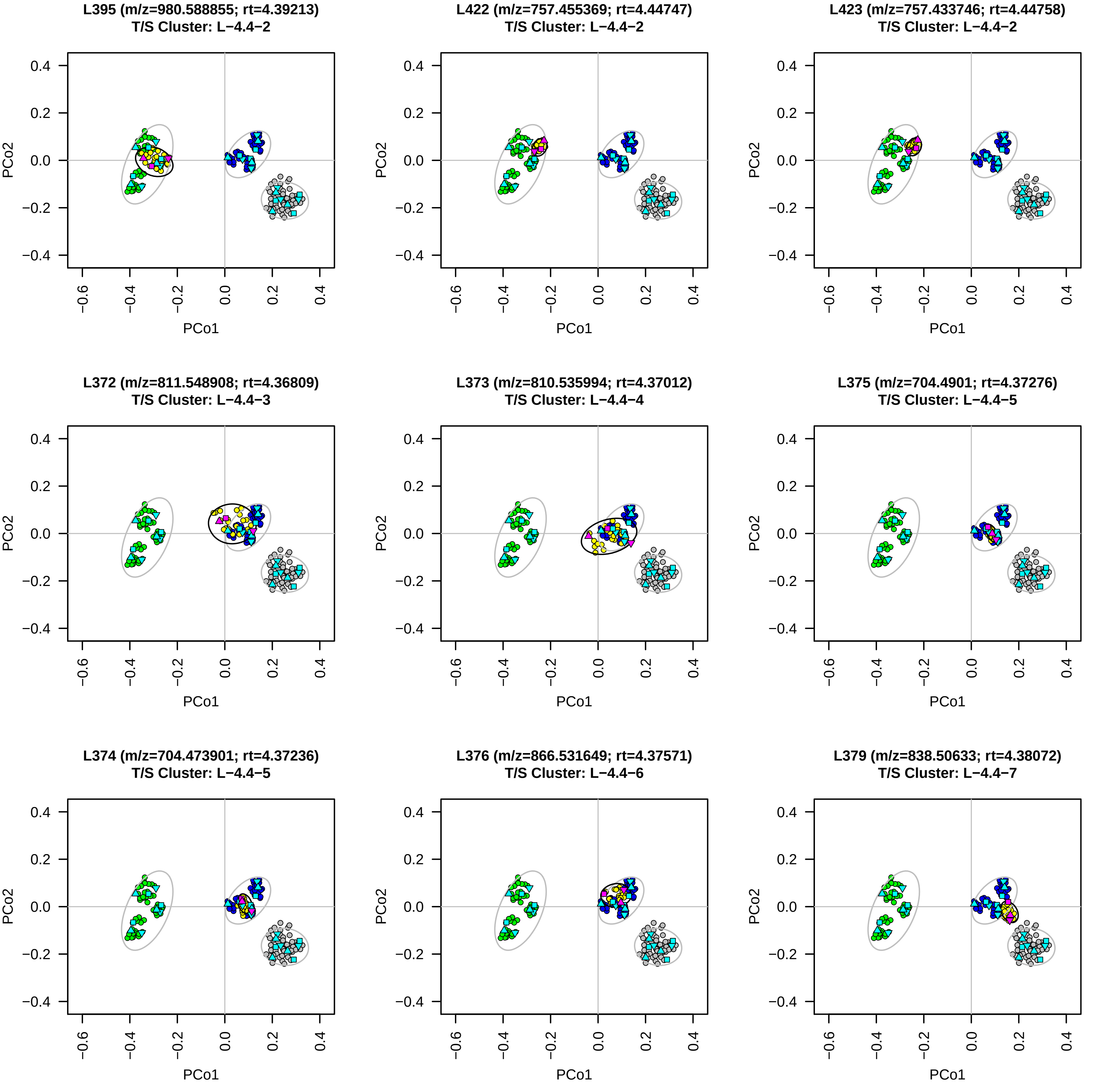
L395 (m/z=980.588855; rt=4.39213)
T/S Cluster: L−4.4−2
L422 (m/z=757.455369; rt=4.44747)
T/S Cluster: L−4.4−2
L423 (m/z=757.433746; rt=4.44758)
T/S Cluster: L−4.4−2
L372 (m/z=811.548908; rt=4.36809)
T/S Cluster: L−4.4−3
L373 (m/z=810.535994; rt=4.37012)
T/S Cluster: L−4.4−4
L375 (m/z=704.4901; rt=4.37276)
T/S Cluster: L−4.4−5
L374 (m/z=704.473901; rt=4.37236)
T/S Cluster: L−4.4−5
L376 (m/z=866.531649; rt=4.37571)
T/S Cluster: L−4.4−6
L379 (m/z=838.50633; rt=4.38072)
T/S Cluster: L−4.4−7
PCo1
PCo2
−0.6
−0.4
−0.2
0.0
0.2
0.4

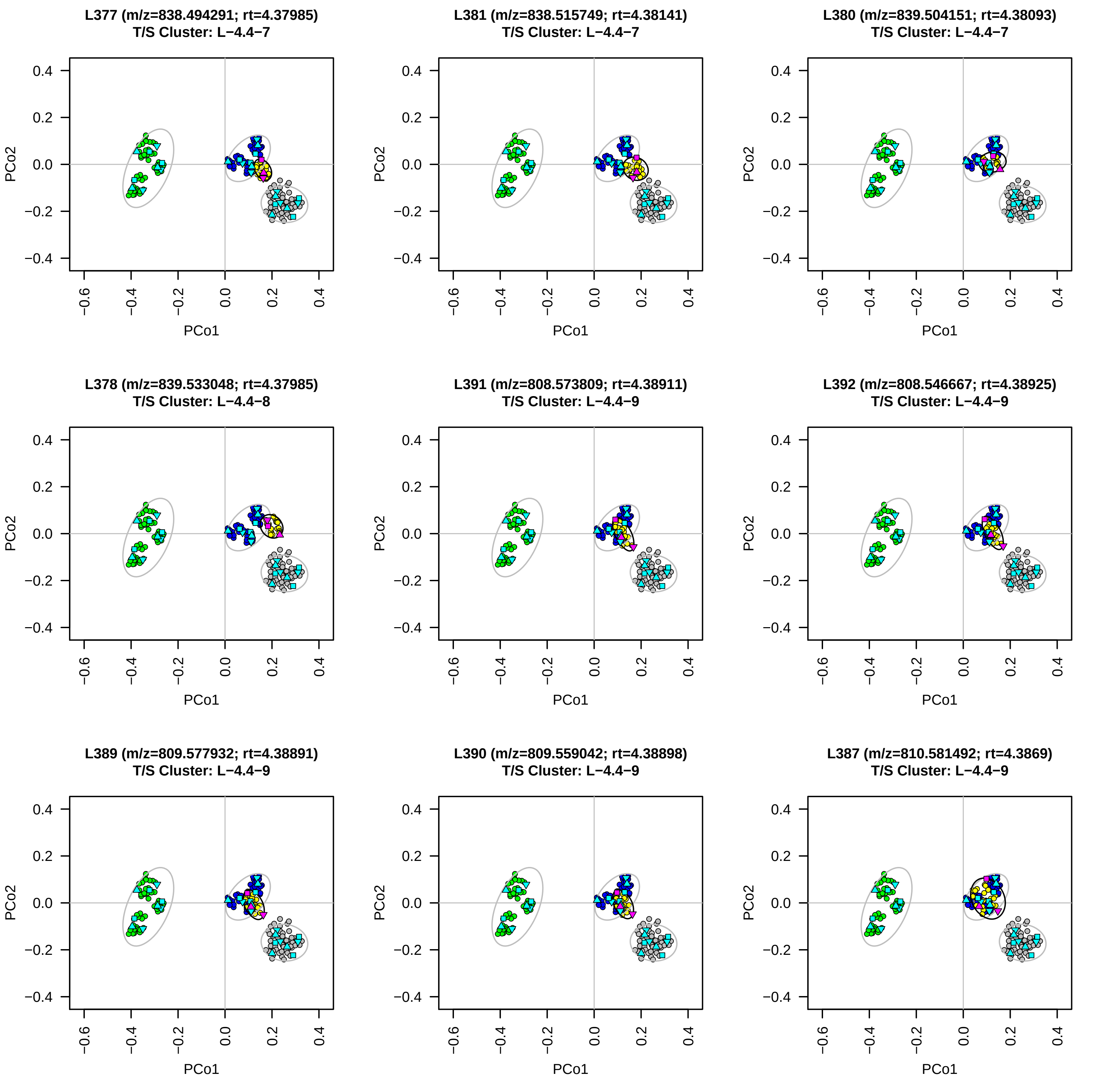
L377 (m/z=838.494291; rt=4.37985)
T/S Cluster: L−4.4−7
L381 (m/z=838.515749; rt=4.38141)
T/S Cluster: L−4.4−7
L380 (m/z=839.504151; rt=4.38093)
T/S Cluster: L−4.4−7
L378 (m/z=839.533048; rt=4.37985)
T/S Cluster: L−4.4−8
L391 (m/z=808.573809; rt=4.38911)
T/S Cluster: L−4.4−9
L392 (m/z=808.546667; rt=4.38925)
T/S Cluster: L−4.4−9
L389 (m/z=809.577932; rt=4.38891)
T/S Cluster: L−4.4−9
L390 (m/z=809.559042; rt=4.38898)
T/S Cluster: L−4.4−9
L387 (m/z=810.581492; rt=4.3869)
T/S Cluster: L−4.4−9
PCo1
PCo2
0.4
0.2
0.0
−0.2
−0.4
−0.6

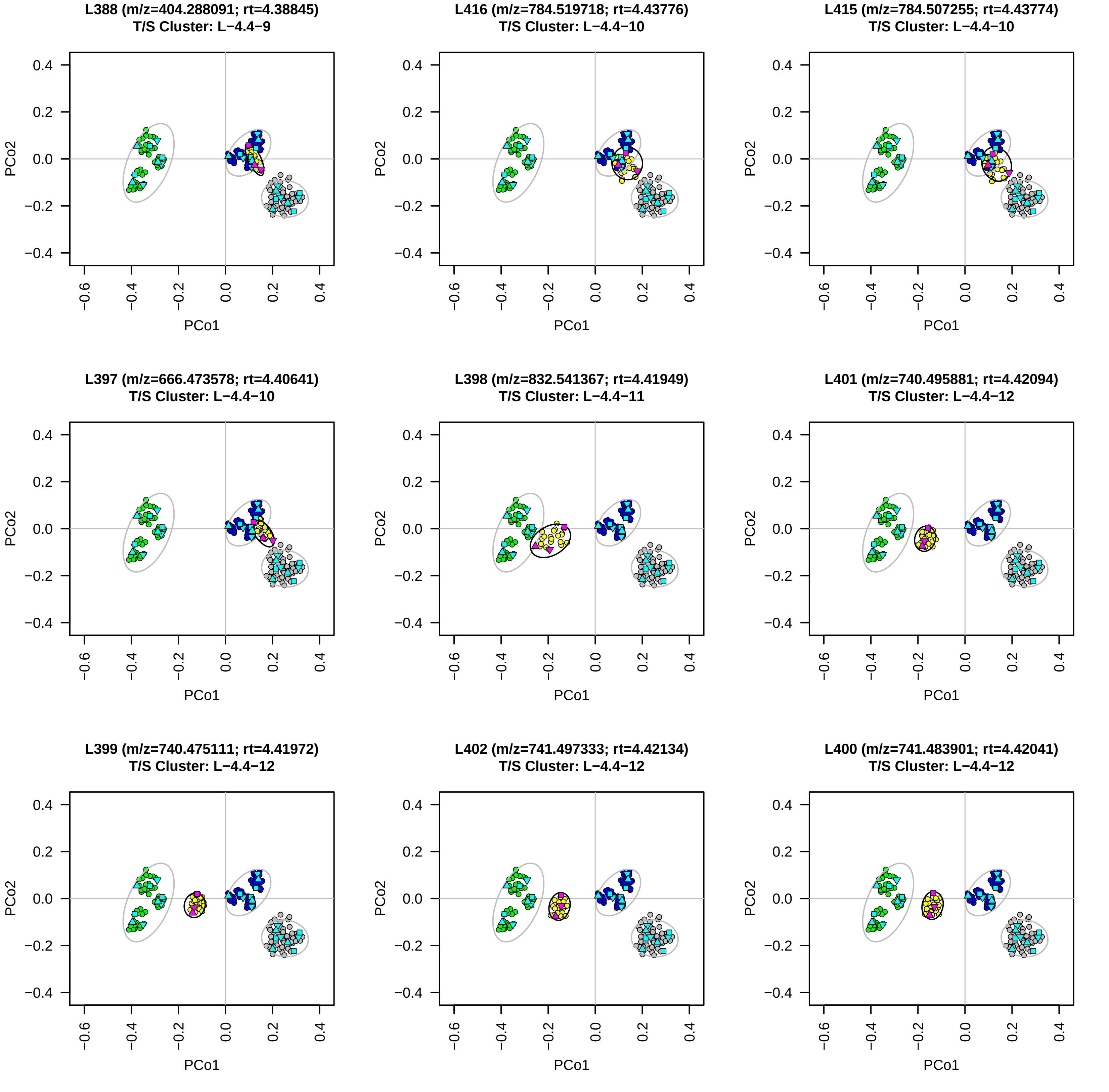
L388 (m/z=404.288091; rt=4.38845)
T/S Cluster: L−4.4−9
L416 (m/z=784.519718; rt=4.43776)
T/S Cluster: L−4.4−10
L415 (m/z=784.507255; rt=4.43774)
T/S Cluster: L−4.4−10
L397 (m/z=666.473578; rt=4.40641)
T/S Cluster: L−4.4−10
L398 (m/z=832.541367; rt=4.41949)
T/S Cluster: L−4.4−11
L401 (m/z=740.495881; rt=4.42094)
T/S Cluster: L−4.4−12
L399 (m/z=740.475111; rt=4.41972)
T/S Cluster: L−4.4−12
L402 (m/z=741.497333; rt=4.42134)
T/S Cluster: L−4.4−12
L400 (m/z=741.483901; rt=4.42041)
T/S Cluster: L−4.4−12
PCo1
PCo2
0.4
0.2
0.0
−0.2
−0.4
−0.6

L403 (m/z=982.602248; rt=4.42349)
T/S Cluster: L−4.4−13

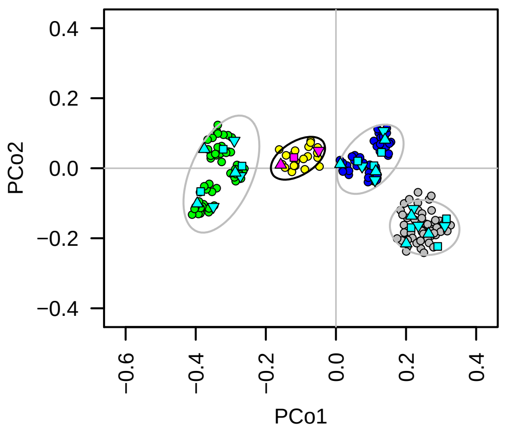

L404 (m/z=982.634197; rt=4.42422)
T/S Cluster: L−4.4−14

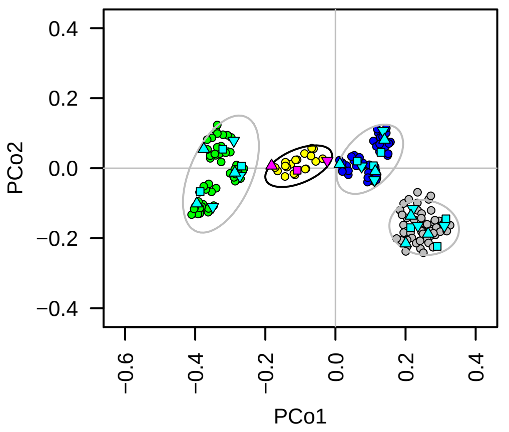

L407 (m/z=789.551703; rt=4.42614)
T/S Cluster: L−4.4−15

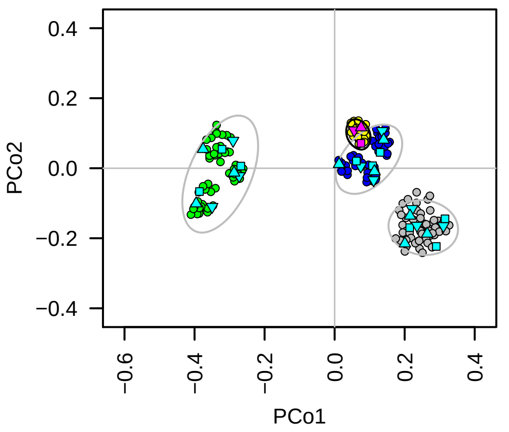

L409 (m/z=788.547415; rt=4.43023)
T/S Cluster: L−4.4−16

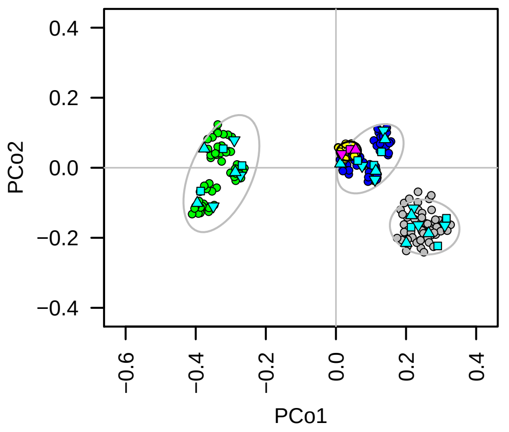

L410 (m/z=788.547461; rt=4.43023)
T/S Cluster: L−4.4−16

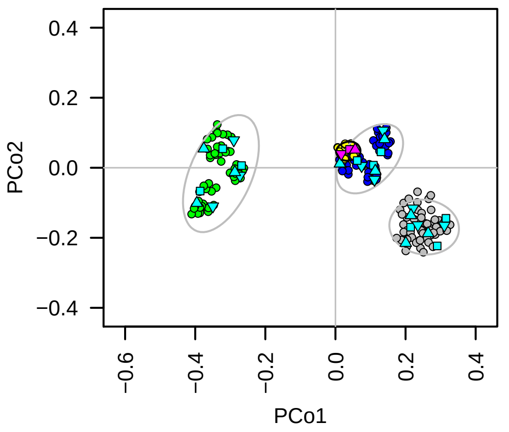

L413 (m/z=805.582905; rt=4.43096)
T/S Cluster: L−4.4−17

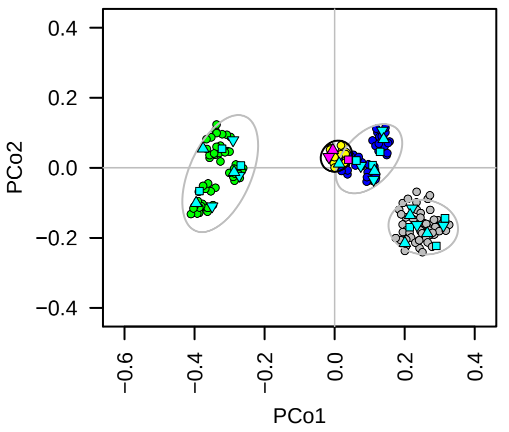

L418 (m/z=694.4695; rt=4.44016)
T/S Cluster: L−4.4−18

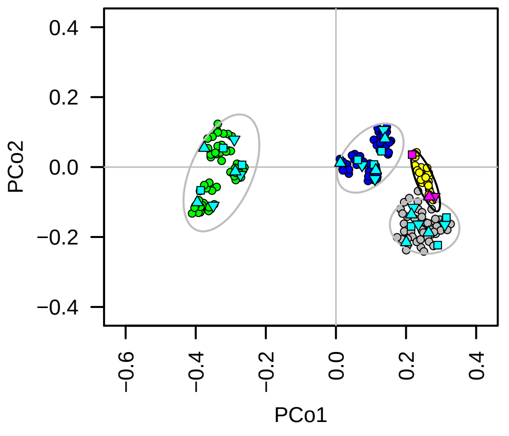

L419 (m/z=694.446053; rt=4.44017)
T/S Cluster: L−4.4−18

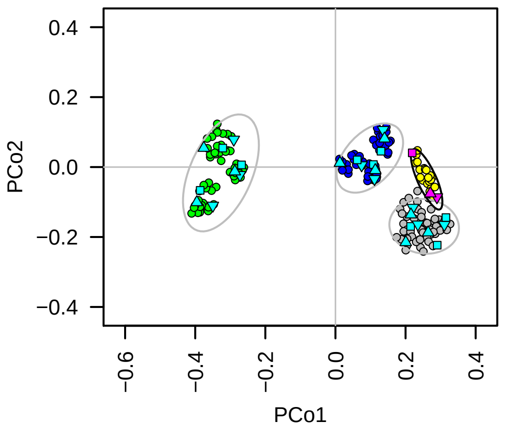

L417 (m/z=695.472326; rt=4.4398)
T/S Cluster: L−4.4−18

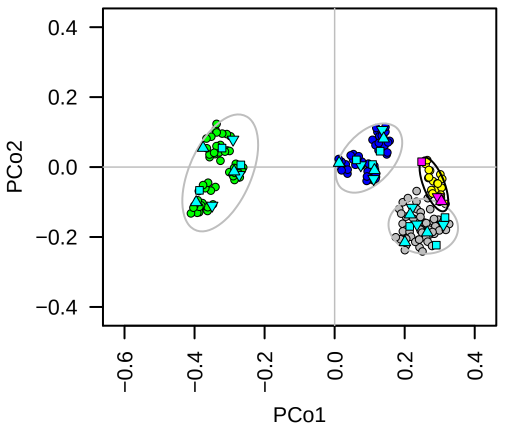

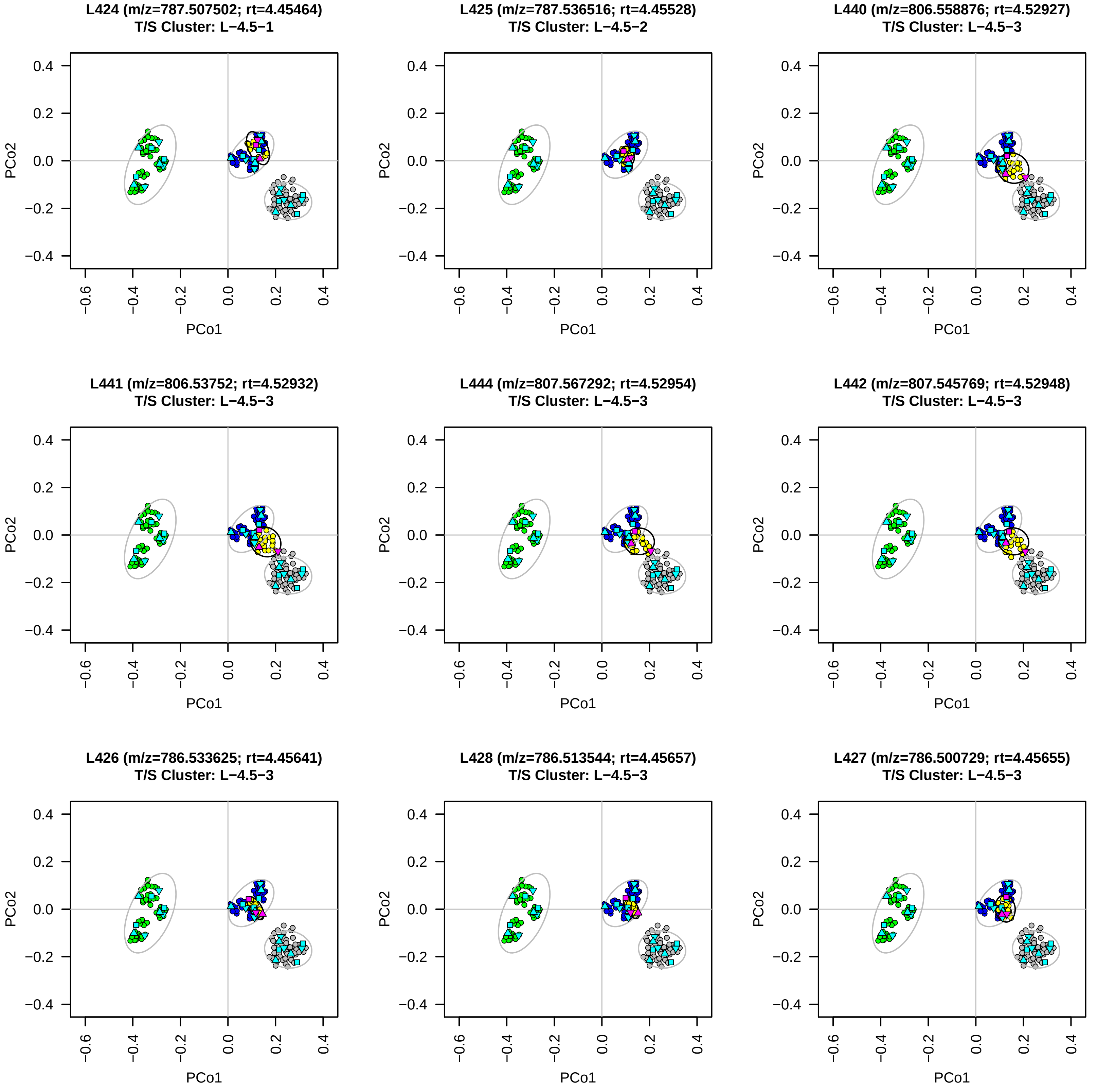
L424 (m/z=787.507502; rt=4.45464)
T/S Cluster: L−4.5−1
L425 (m/z=787.536516; rt=4.45528)
T/S Cluster: L−4.5−2
L440 (m/z=806.558876; rt=4.52927)
T/S Cluster: L−4.5−3
L441 (m/z=806.53752; rt=4.52932)
T/S Cluster: L−4.5−3
L444 (m/z=807.567292; rt=4.52954)
T/S Cluster: L−4.5−3
L442 (m/z=807.545769; rt=4.52948)
T/S Cluster: L−4.5−3
L426 (m/z=786.533625; rt=4.45641)
T/S Cluster: L−4.5−3
L428 (m/z=786.513544; rt=4.45657)
T/S Cluster: L−4.5−3
L427 (m/z=786.500729; rt=4.45655)
T/S Cluster: L−4.5−3
PCo1
PCo2
0.4
0.2
0.0
−0.2
−0.4
−0.6

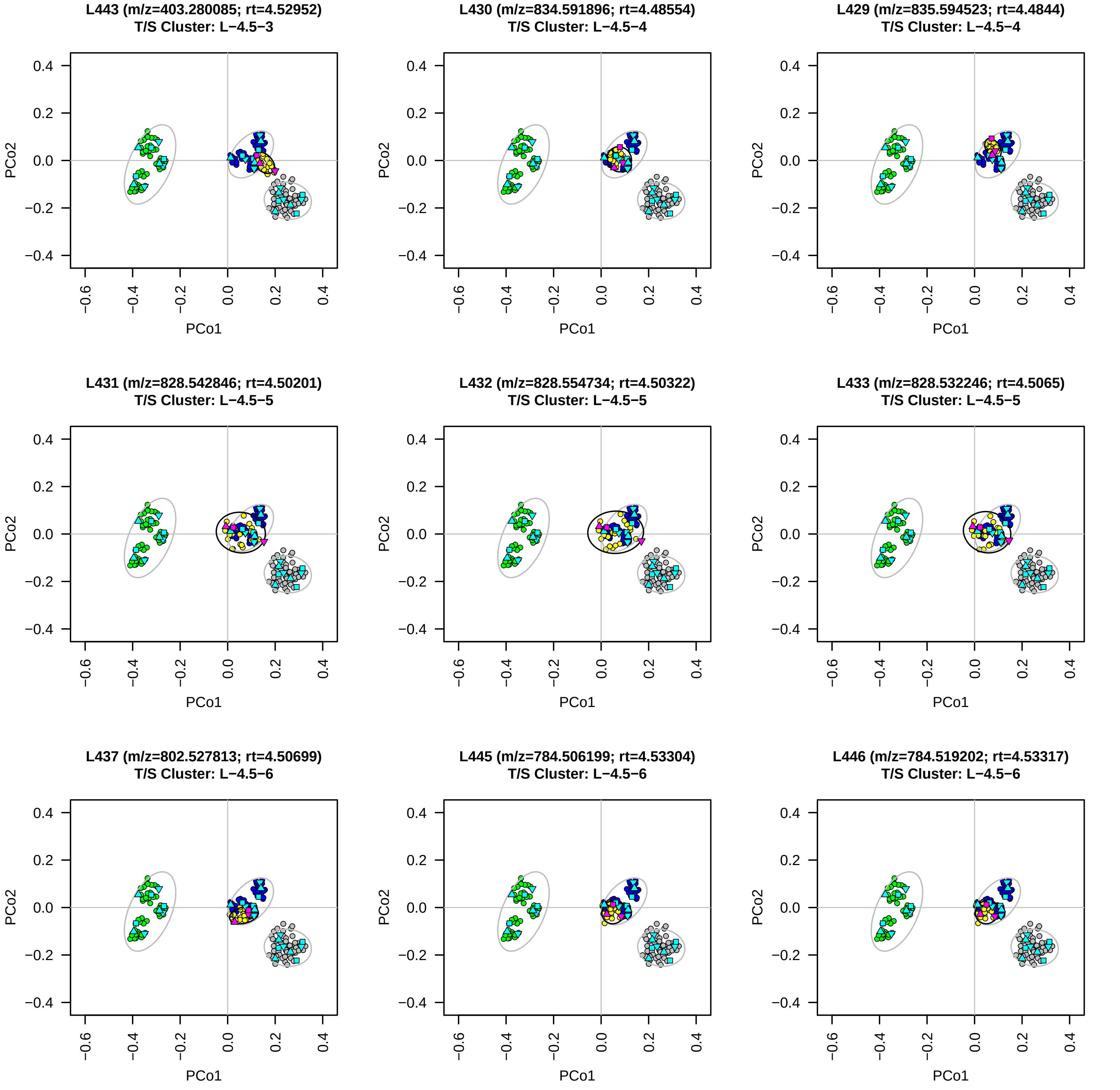
L443 (m/z=403.280085; rt=4.52952)
T/S Cluster: L−4.5−3
L430 (m/z=834.591896; rt=4.48554)
T/S Cluster: L−4.5−4
L429 (m/z=835.594523; rt=4.4844)
T/S Cluster: L−4.5−4
L431 (m/z=828.542846; rt=4.50201)
T/S Cluster: L−4.5−5
L432 (m/z=828.554734; rt=4.50322)
T/S Cluster: L−4.5−5
L433 (m/z=828.532246; rt=4.5065)
T/S Cluster: L−4.5−5
L437 (m/z=802.527813; rt=4.50699)
T/S Cluster: L−4.5−6
L445 (m/z=784.506199; rt=4.53304)
T/S Cluster: L−4.5−6
L446 (m/z=784.519202; rt=4.53317)
T/S Cluster: L−4.5−6
PCo1
PCo2
0.4
0.2
0.0
−0.2
−0.4
−0.6

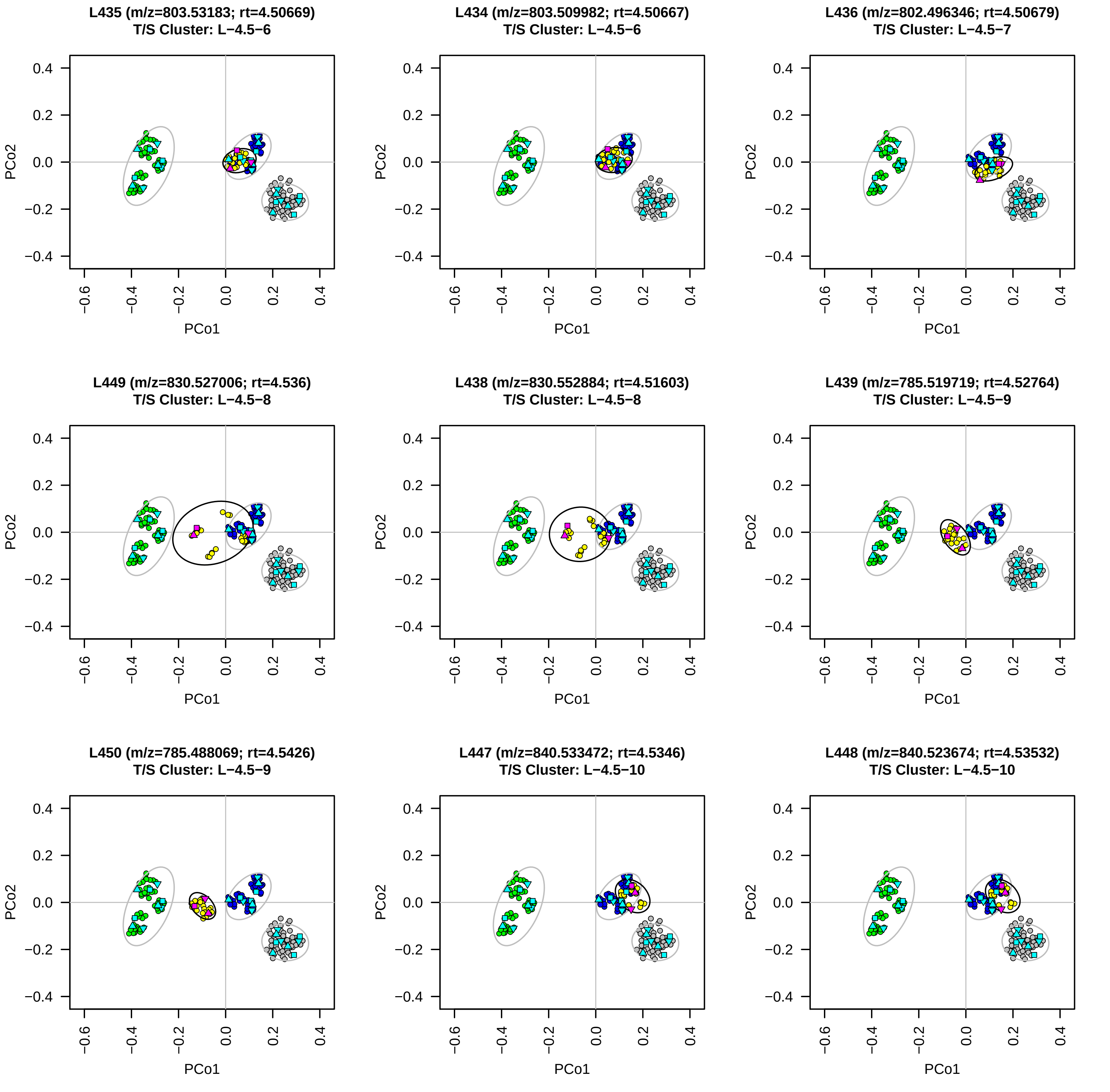
L435 (m/z=803.53183; rt=4.50669)
T/S Cluster: L−4.5−6
L434 (m/z=803.509982; rt=4.50667)
T/S Cluster: L−4.5−6
L436 (m/z=802.496346; rt=4.50679)
T/S Cluster: L−4.5−7
L449 (m/z=830.527006; rt=4.536)
T/S Cluster: L−4.5−8
L438 (m/z=830.552884; rt=4.51603)
T/S Cluster: L−4.5−8
L439 (m/z=785.519719; rt=4.52764)
T/S Cluster: L−4.5−9
L450 (m/z=785.488069; rt=4.5426)
T/S Cluster: L−4.5−9
L447 (m/z=840.533472; rt=4.5346)
T/S Cluster: L−4.5−10
L448 (m/z=840.523674; rt=4.53532)
T/S Cluster: L−4.5−10
PCo1
PCo2
0.4
0.2
0.0
−0.2
−0.4
−0.6

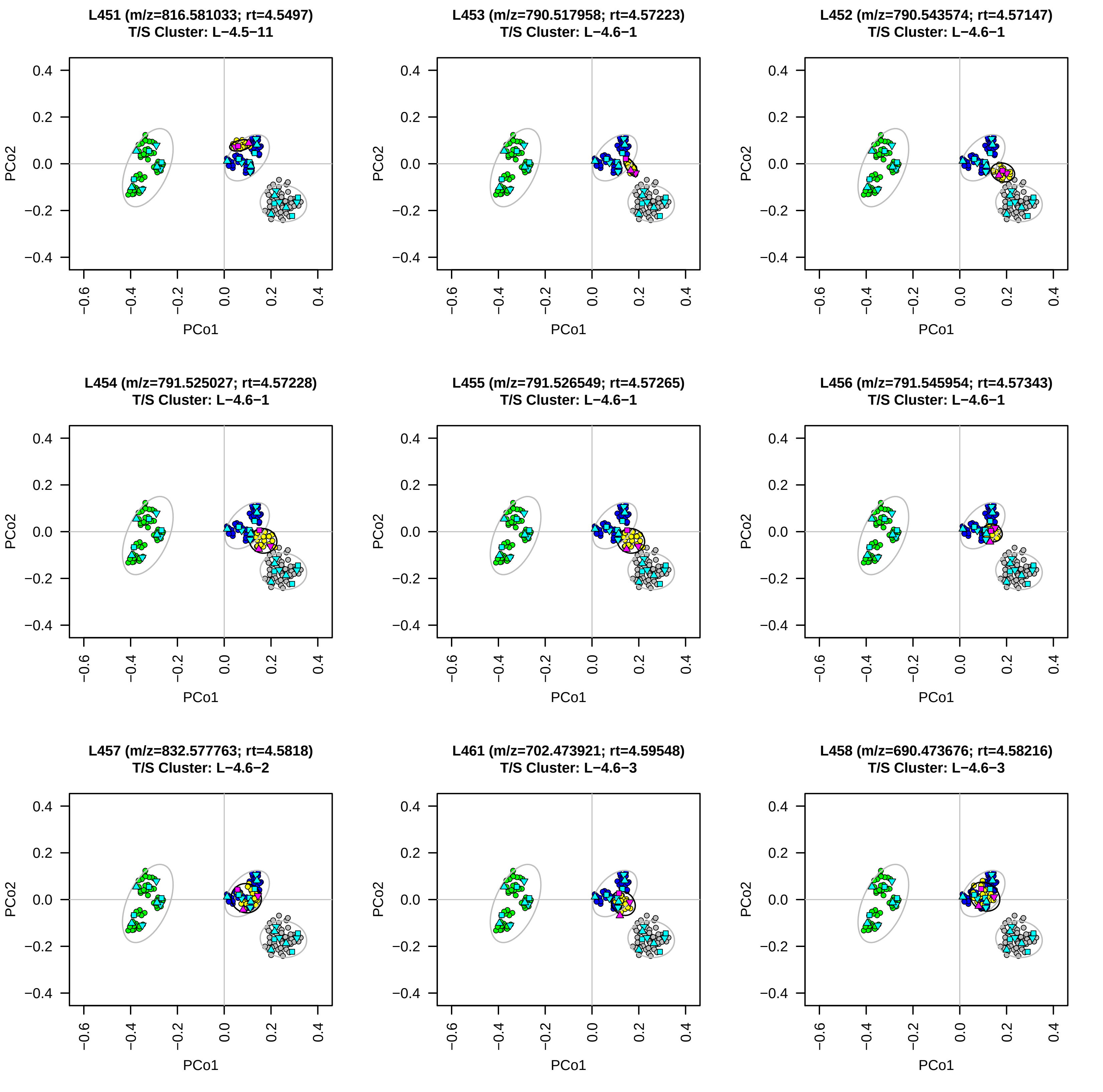
L451 (m/z=816.581033; rt=4.5497)
T/S Cluster: L−4.5−11
L453 (m/z=790.517958; rt=4.57223)
T/S Cluster: L−4.6−1
L452 (m/z=790.543574; rt=4.57147)
T/S Cluster: L−4.6−1
L454 (m/z=791.525027; rt=4.57228)
T/S Cluster: L−4.6−1
L455 (m/z=791.526549; rt=4.57265)
T/S Cluster: L−4.6−1
L456 (m/z=791.545954; rt=4.57343)
T/S Cluster: L−4.6−1
L457 (m/z=832.577763; rt=4.5818)
T/S Cluster: L−4.6−2
L461 (m/z=702.473921; rt=4.59548)
T/S Cluster: L−4.6−3
L458 (m/z=690.473676; rt=4.58216)
T/S Cluster: L−4.6−3
PCo1
PCo2

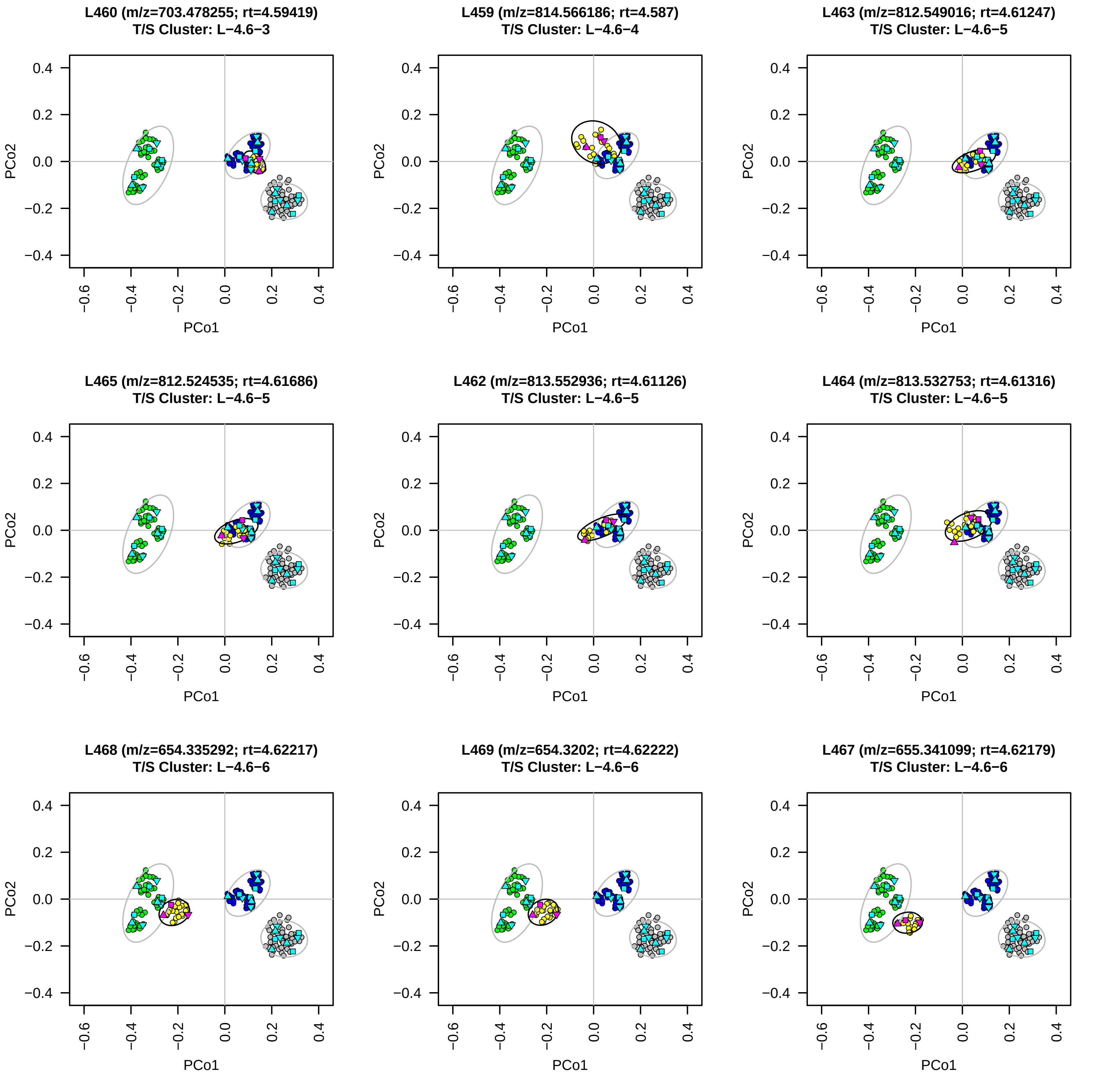
L460 (m/z=703.478255; rt=4.59419)
T/S Cluster: L−4.6−3
L459 (m/z=814.566186; rt=4.587)
T/S Cluster: L−4.6−4
L463 (m/z=812.549016; rt=4.61247)
T/S Cluster: L−4.6−5
L465 (m/z=812.524535; rt=4.61686)
T/S Cluster: L−4.6−5
L462 (m/z=813.552936; rt=4.61126)
T/S Cluster: L−4.6−5
L464 (m/z=813.532753; rt=4.61316)
T/S Cluster: L−4.6−5
L468 (m/z=654.335292; rt=4.62217)
T/S Cluster: L−4.6−6
L469 (m/z=654.3202; rt=4.62222)
T/S Cluster: L−4.6−6
L467 (m/z=655.341099; rt=4.62179)
T/S Cluster: L−4.6−6
PCo1
PCo2
0.4
0.2
0.0
−0.2
−0.4
−0.6

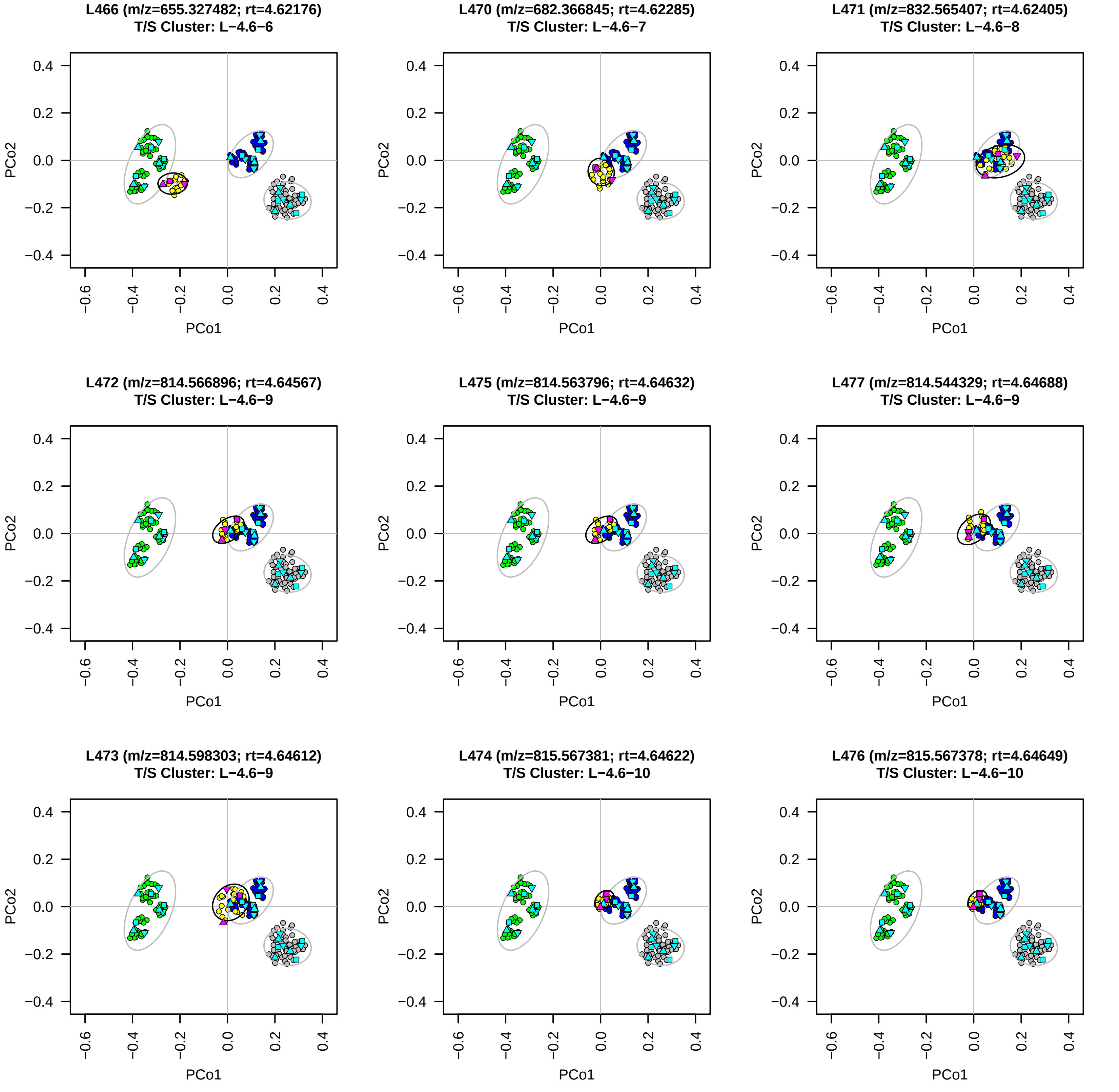
L466 (m/z=655.327482; rt=4.62176)
T/S Cluster: L−4.6−6
L470 (m/z=682.366845; rt=4.62285)
T/S Cluster: L−4.6−7
L471 (m/z=832.565407; rt=4.62405)
T/S Cluster: L−4.6−8
L472 (m/z=814.566896; rt=4.64567)
T/S Cluster: L−4.6−9
L475 (m/z=814.563796; rt=4.64632)
T/S Cluster: L−4.6−9
L477 (m/z=814.544329; rt=4.64688)
T/S Cluster: L−4.6−9
L473 (m/z=814.598303; rt=4.64612)
T/S Cluster: L−4.6−9
L474 (m/z=815.567381; rt=4.64622)
T/S Cluster: L−4.6−10
L476 (m/z=815.567378; rt=4.64649)
T/S Cluster: L−4.6−10
PCo1
PCo2

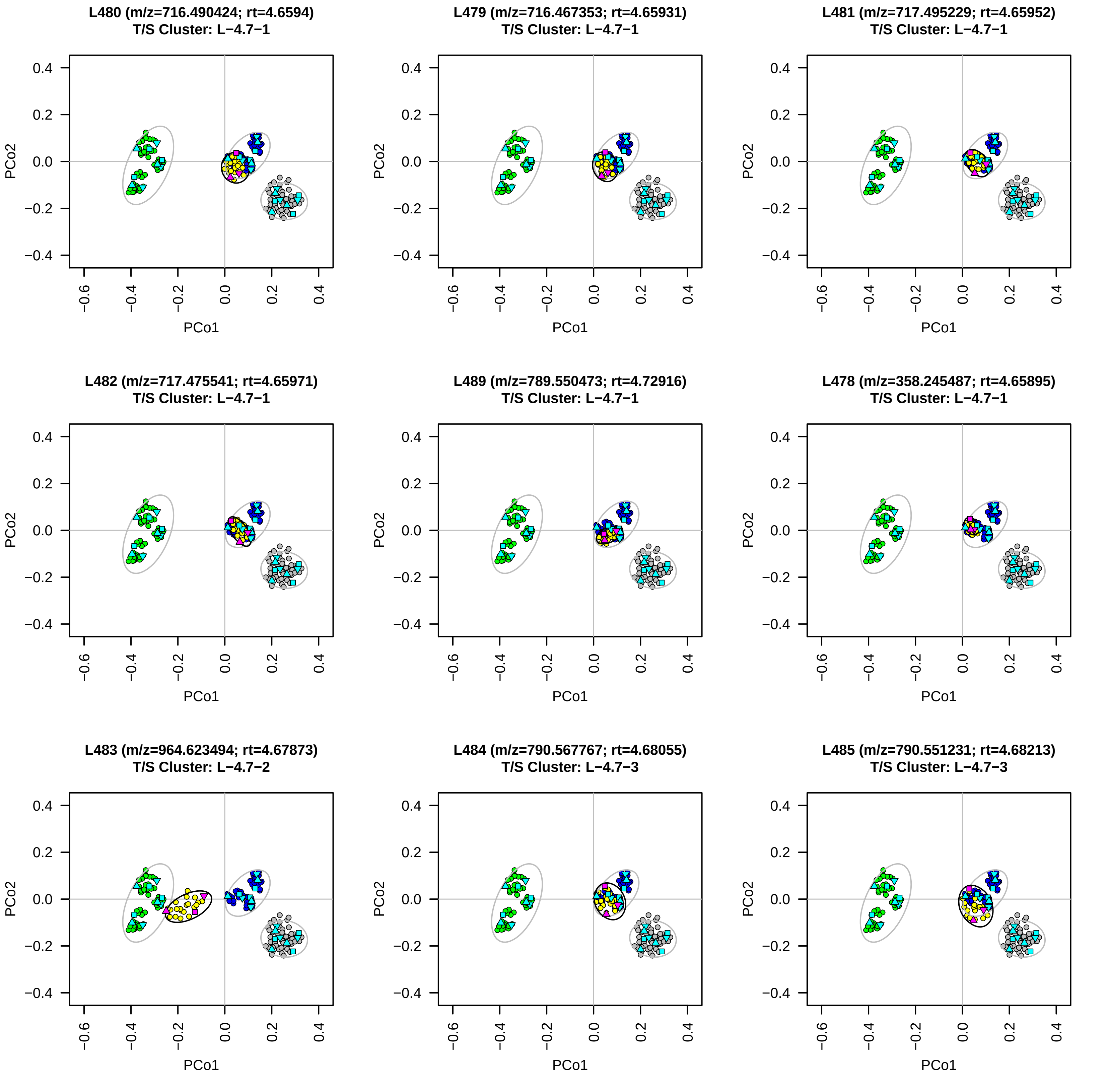
L480 (m/z=716.490424; rt=4.6594)
T/S Cluster: L−4.7−1
L479 (m/z=716.467353; rt=4.65931)
T/S Cluster: L−4.7−1
L481 (m/z=717.495229; rt=4.65952)
T/S Cluster: L−4.7−1
L482 (m/z=717.475541; rt=4.65971)
T/S Cluster: L−4.7−1
L489 (m/z=789.550473; rt=4.72916)
T/S Cluster: L−4.7−1
L478 (m/z=358.245487; rt=4.65895)
T/S Cluster: L−4.7−1
L483 (m/z=964.623494; rt=4.67873)
T/S Cluster: L−4.7−2
L484 (m/z=790.567767; rt=4.68055)
T/S Cluster: L−4.7−3
L485 (m/z=790.551231; rt=4.68213)
T/S Cluster: L−4.7−3
PCo1
PCo2
0.4
0.2
0.0
−0.2
−0.4
−0.6

L487 (m/z=810.528834; rt=4.69109)
T/S Cluster: L−4.7−4

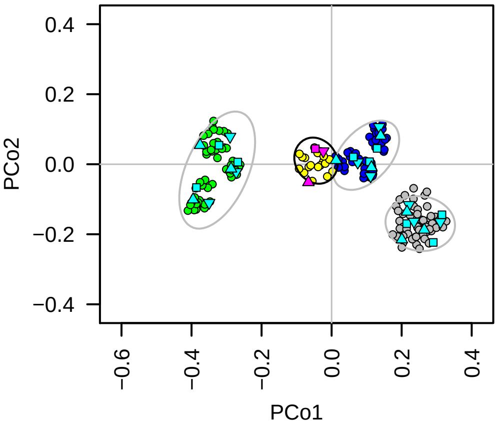

L486 (m/z=810.554675; rt=4.69039)
T/S Cluster: L−4.7−4

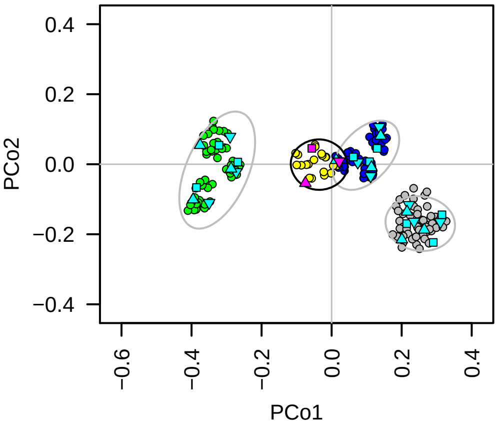

L488 (m/z=820.515641; rt=4.70551)
T/S Cluster: L−4.7−5

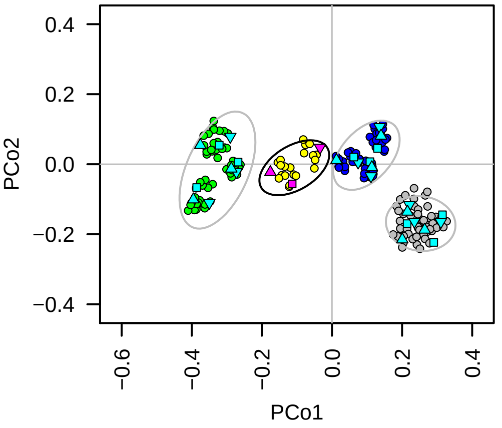

L490 (m/z=788.591712; rt=4.72925)
T/S Cluster: L−4.7−6

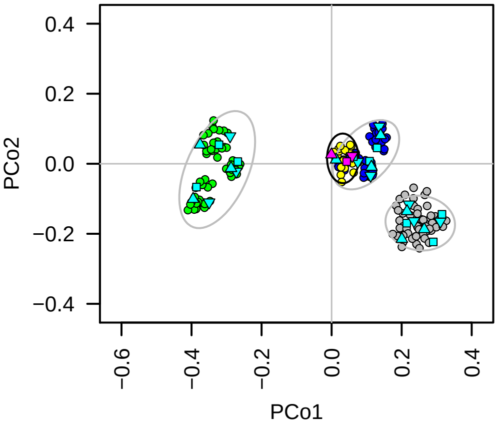

L491 (m/z=788.547762; rt=4.73069)
T/S Cluster: L−4.7−7

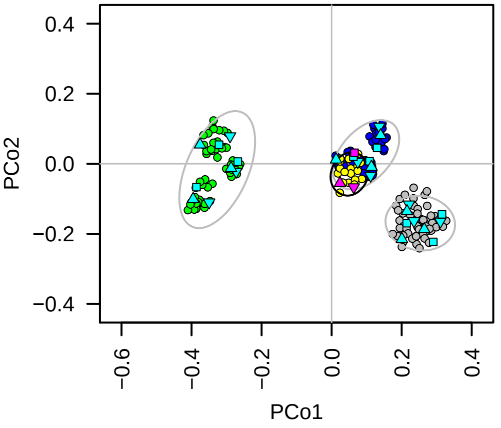

L493 (m/z=892.57477; rt=4.74184)
T/S Cluster: L−4.7−8

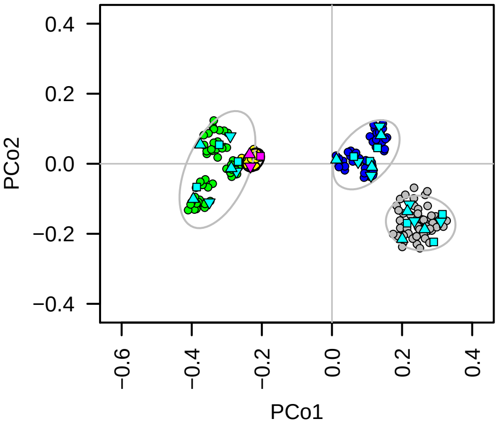

L492 (m/z=892.549887; rt=4.74176)
T/S Cluster: L−4.7−8

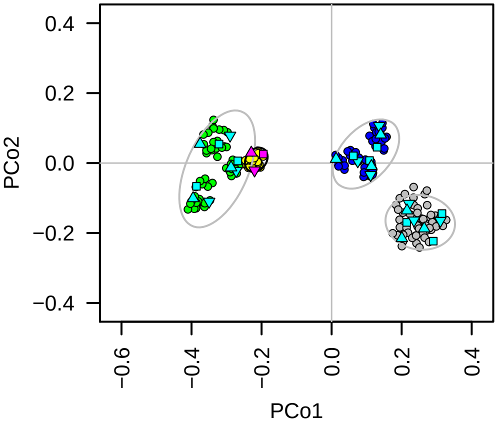

L494 (m/z=774.530527; rt=4.74892)
T/S Cluster: L−4.7−9

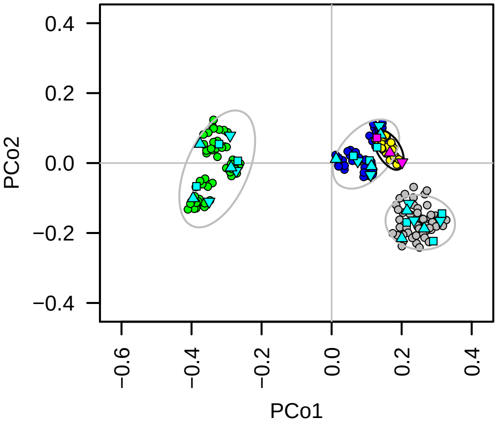

L495 (m/z=774.531637; rt=4.74897)
T/S Cluster: L−4.7−9

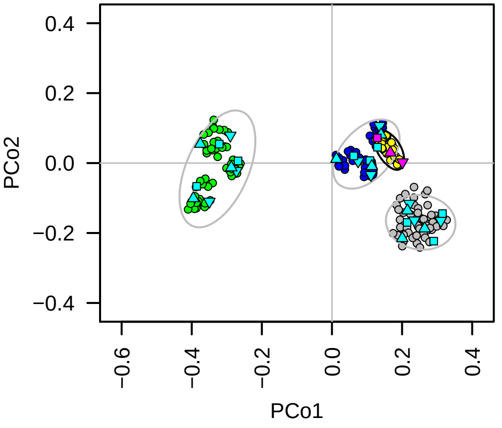

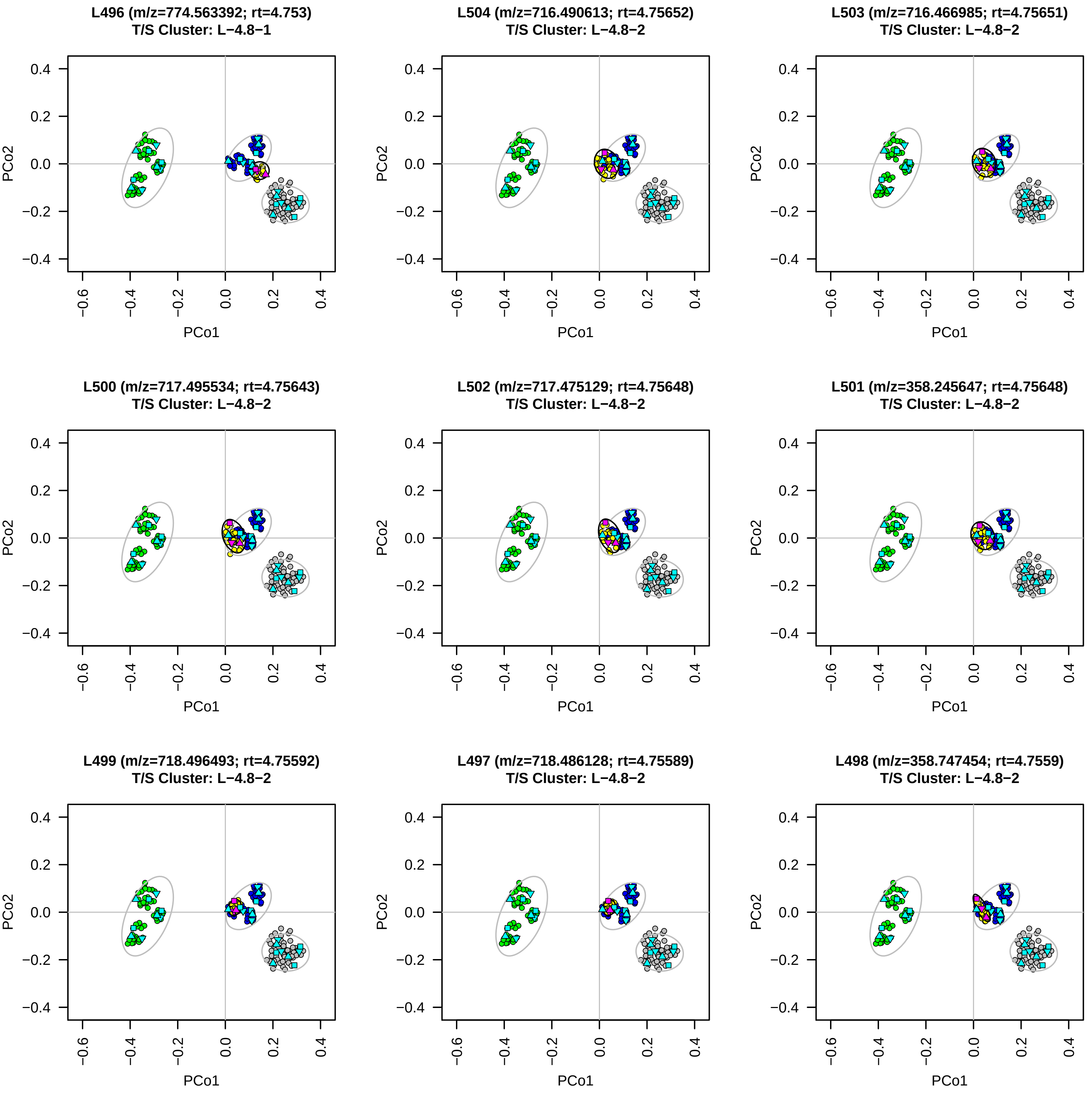
L496 (m/z=774.563392; rt=4.753)
T/S Cluster: L−4.8−1
L504 (m/z=716.490613; rt=4.75652)
T/S Cluster: L−4.8−2
L503 (m/z=716.466985; rt=4.75651)
T/S Cluster: L−4.8−2
L500 (m/z=717.495534; rt=4.75643)
T/S Cluster: L−4.8−2
L502 (m/z=717.475129; rt=4.75648)
T/S Cluster: L−4.8−2
L501 (m/z=358.245647; rt=4.75648)
T/S Cluster: L−4.8−2
L499 (m/z=718.496493; rt=4.75592)
T/S Cluster: L−4.8−2
L497 (m/z=718.486128; rt=4.75589)
T/S Cluster: L−4.8−2
L498 (m/z=358.747454; rt=4.7559)
T/S Cluster: L−4.8−2
PCo1
PCo2

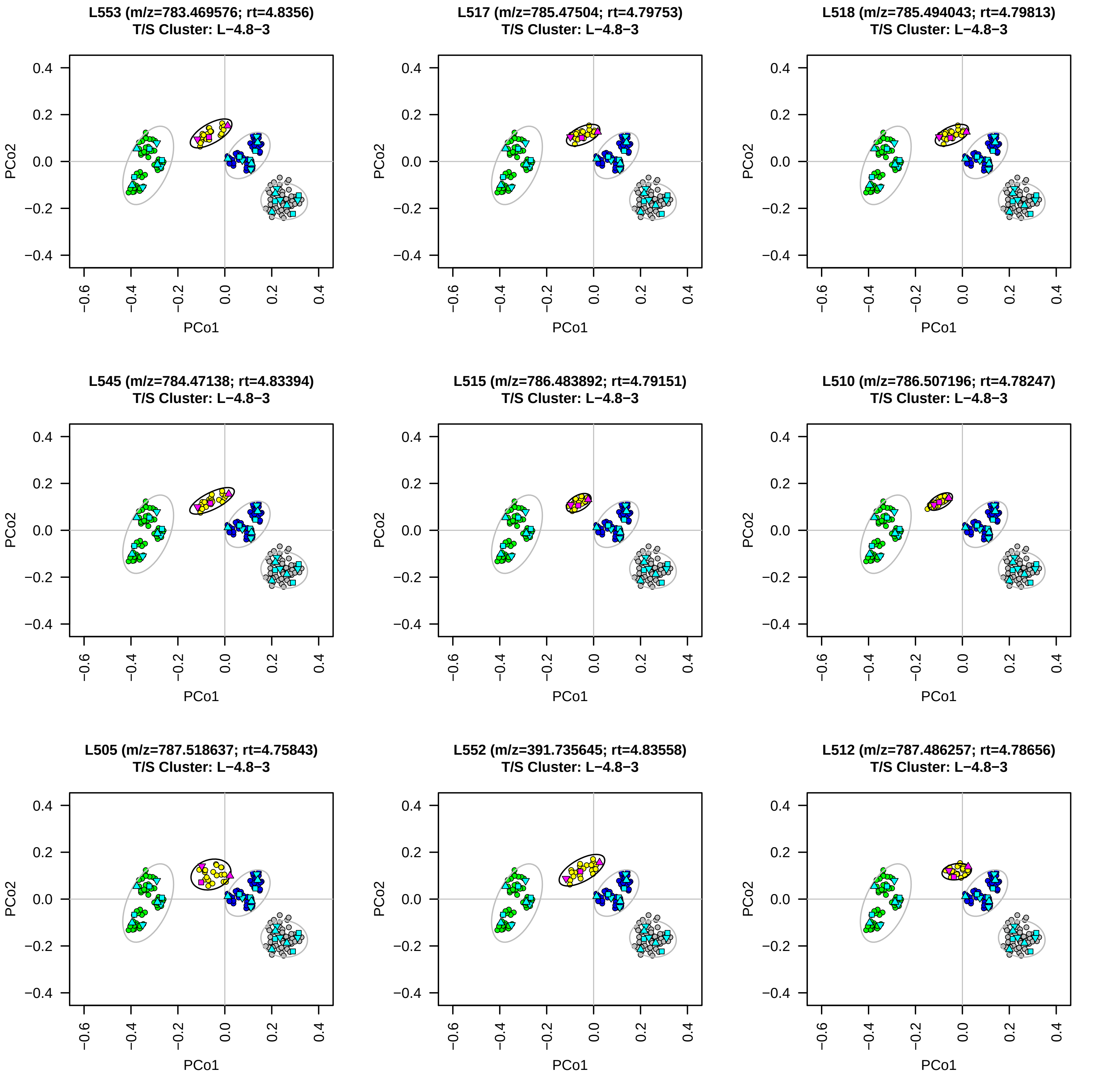
L553 (m/z=783.469576; rt=4.8356)
T/S Cluster: L−4.8−3
L517 (m/z=785.47504; rt=4.79753)
T/S Cluster: L−4.8−3
L518 (m/z=785.494043; rt=4.79813)
T/S Cluster: L−4.8−3
L545 (m/z=784.47138; rt=4.83394)
T/S Cluster: L−4.8−3
L515 (m/z=786.483892; rt=4.79151)
T/S Cluster: L−4.8−3
L510 (m/z=786.507196; rt=4.78247)
T/S Cluster: L−4.8−3
L505 (m/z=787.518637; rt=4.75843)
T/S Cluster: L−4.8−3
L552 (m/z=391.735645; rt=4.83558)
T/S Cluster: L−4.8−3
L512 (m/z=787.486257; rt=4.78656)
T/S Cluster: L−4.8−3
PCo1
PCo2
−0.6
−0.4
−0.2
0.0
0.2
0.4

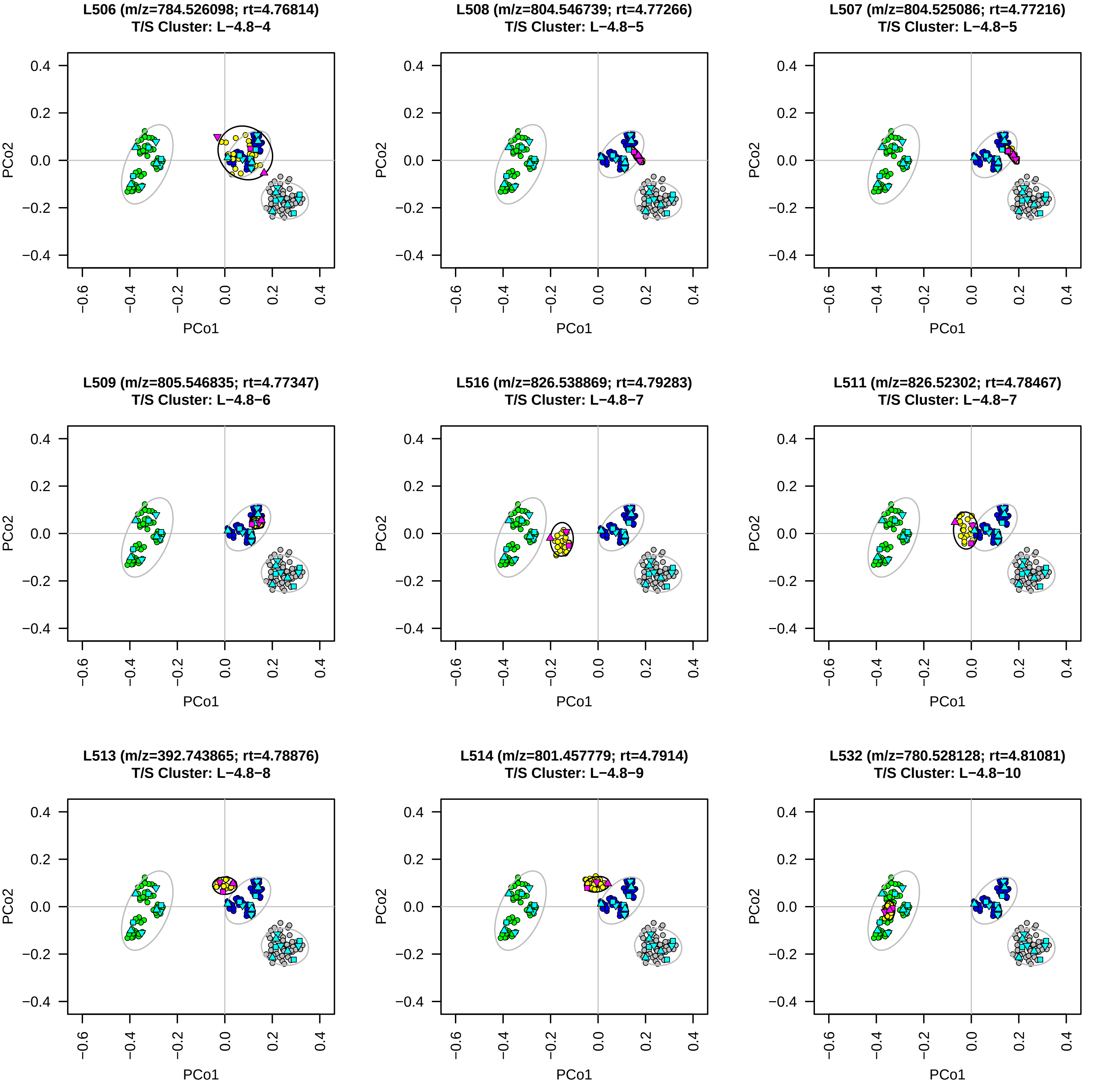
L506 (m/z=784.526098; rt=4.76814)
T/S Cluster: L−4.8−4
L508 (m/z=804.546739; rt=4.77266)
T/S Cluster: L−4.8−5
L507 (m/z=804.525086; rt=4.77216)
T/S Cluster: L−4.8−5
L509 (m/z=805.546835; rt=4.77347)
T/S Cluster: L−4.8−6
L516 (m/z=826.538869; rt=4.79283)
T/S Cluster: L−4.8−7
L511 (m/z=826.52302; rt=4.78467)
T/S Cluster: L−4.8−7
L513 (m/z=392.743865; rt=4.78876)
T/S Cluster: L−4.8−8
L514 (m/z=801.457779; rt=4.7914)
T/S Cluster: L−4.8−9
L532 (m/z=780.528128; rt=4.81081)
T/S Cluster: L−4.8−10
PCo1
PCo2
0.4
0.2
0.0
−0.2
−0.4
−0.6

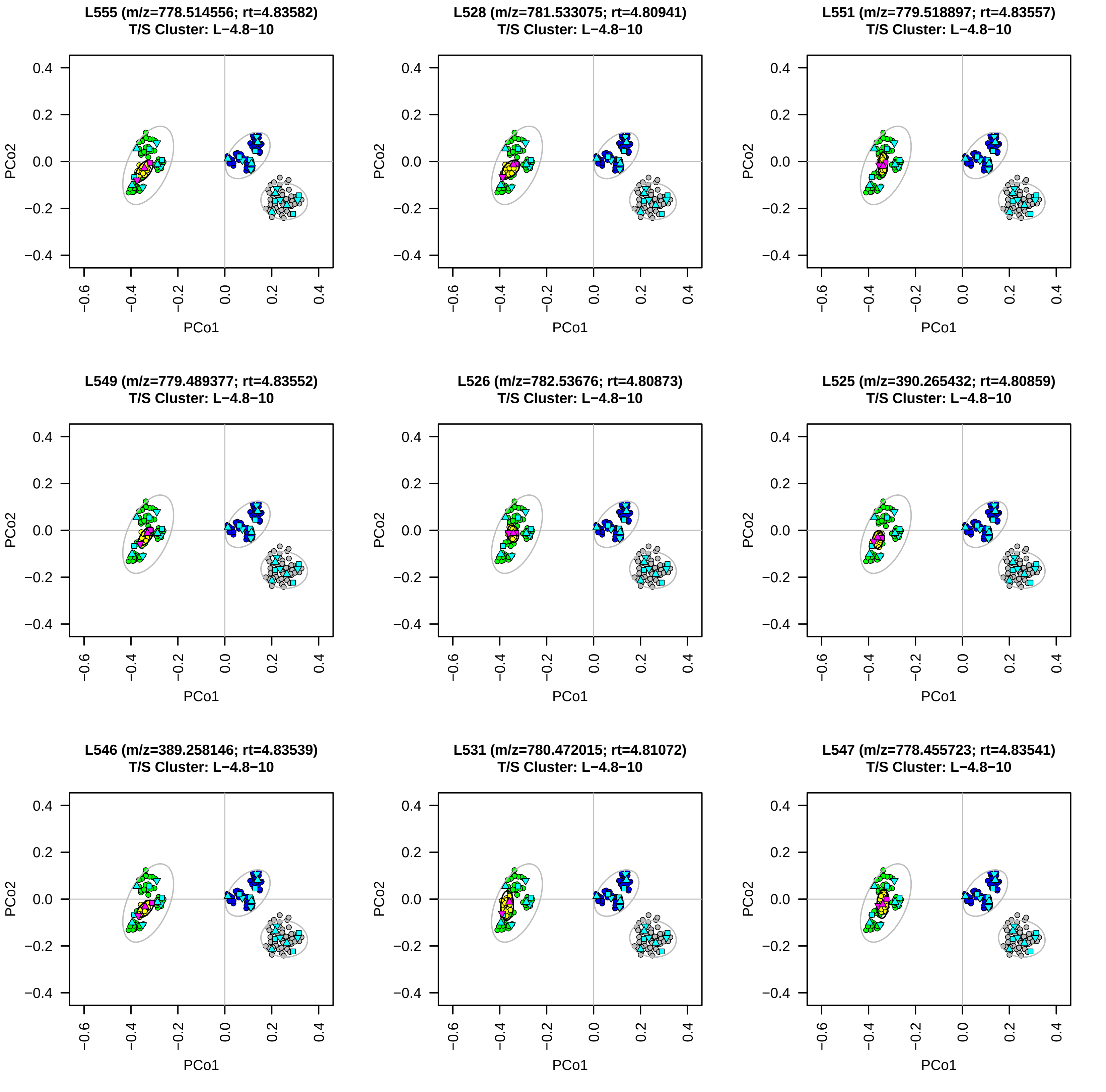
L555 (m/z=778.514556; rt=4.83582)
T/S Cluster: L−4.8−10
L528 (m/z=781.533075; rt=4.80941)
T/S Cluster: L−4.8−10
L551 (m/z=779.518897; rt=4.83557)
T/S Cluster: L−4.8−10
L549 (m/z=779.489377; rt=4.83552)
T/S Cluster: L−4.8−10
L526 (m/z=782.53676; rt=4.80873)
T/S Cluster: L−4.8−10
L525 (m/z=390.265432; rt=4.80859)
T/S Cluster: L−4.8−10
L546 (m/z=389.258146; rt=4.83539)
T/S Cluster: L−4.8−10
L531 (m/z=780.472015; rt=4.81072)
T/S Cluster: L−4.8−10
L547 (m/z=778.455723; rt=4.83541)
T/S Cluster: L−4.8−10
PCo1
PCo2
−0.6
−0.4
−0.2
0.0
0.2
0.4

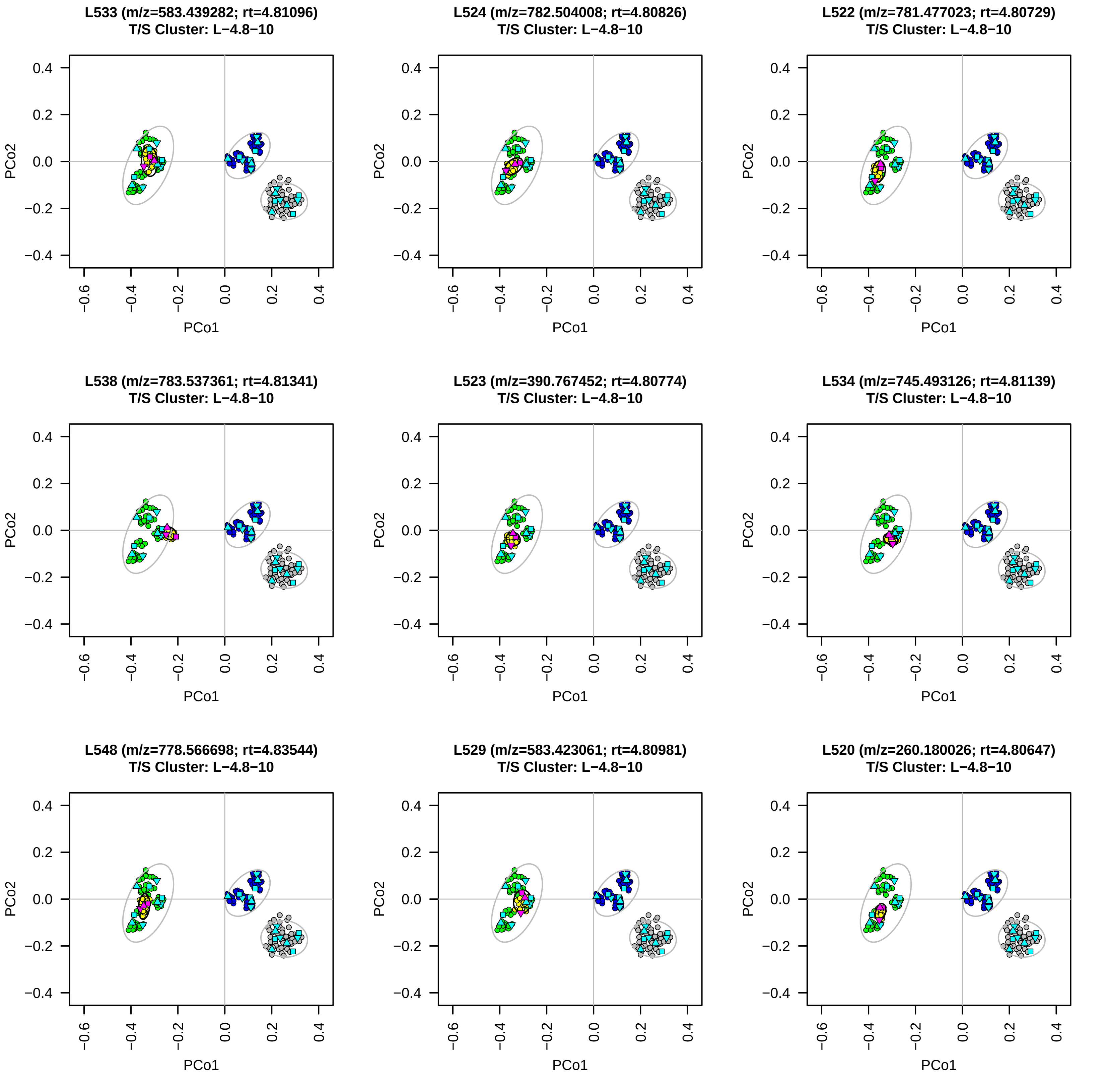
L533 (m/z=583.439282; rt=4.81096)
T/S Cluster: L−4.8−10
L524 (m/z=782.504008; rt=4.80826)
T/S Cluster: L−4.8−10
L522 (m/z=781.477023; rt=4.80729)
T/S Cluster: L−4.8−10
L538 (m/z=783.537361; rt=4.81341)
T/S Cluster: L−4.8−10
L523 (m/z=390.767452; rt=4.80774)
T/S Cluster: L−4.8−10
L534 (m/z=745.493126; rt=4.81139)
T/S Cluster: L−4.8−10
L548 (m/z=778.566698; rt=4.83544)
T/S Cluster: L−4.8−10
L529 (m/z=583.423061; rt=4.80981)
T/S Cluster: L−4.8−10
L520 (m/z=260.180026; rt=4.80647)
T/S Cluster: L−4.8−10
PCo1
PCo2
−0.6
−0.4
−0.2
0.0
0.2
0.4

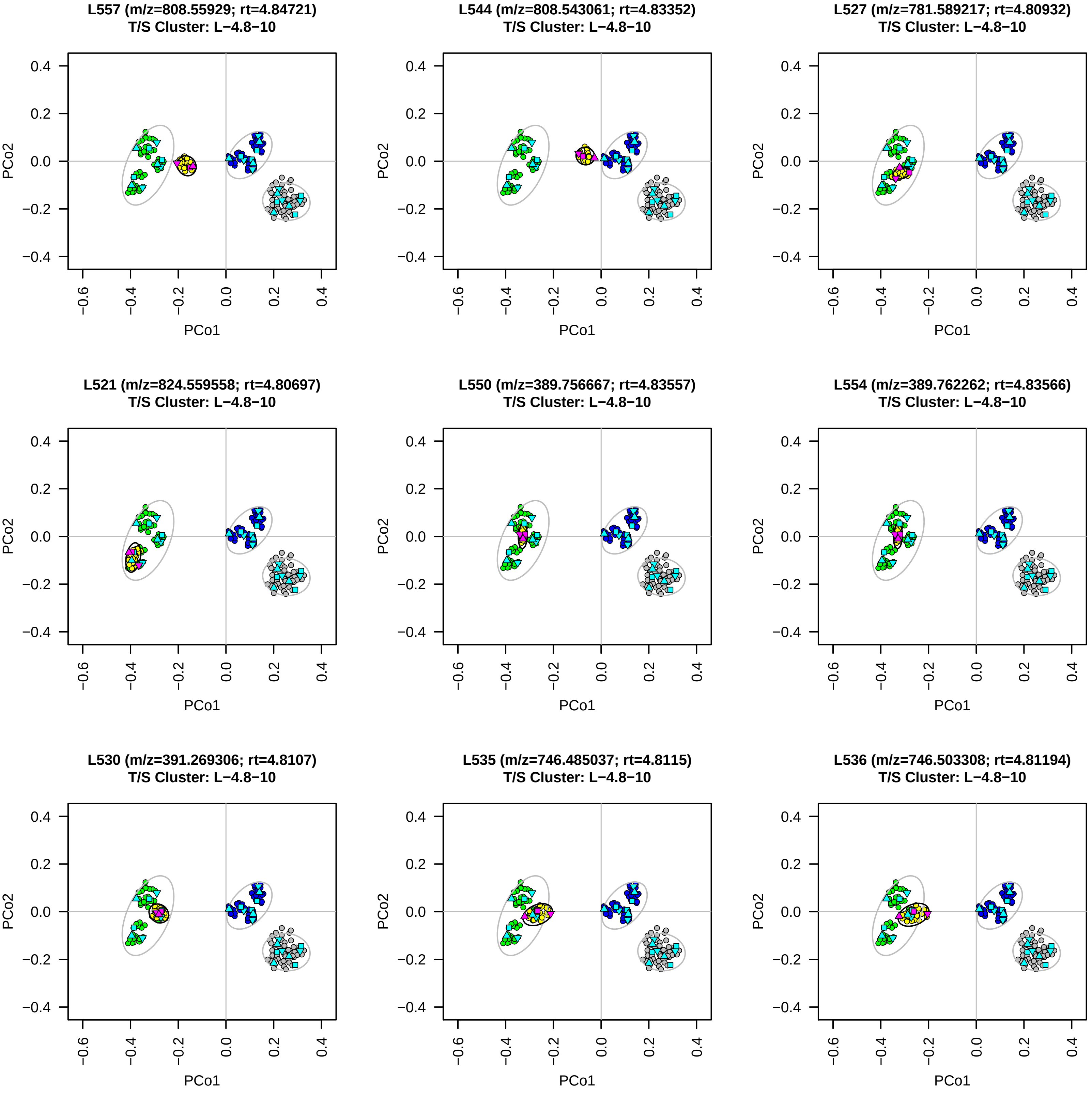
L557 (m/z=808.55929; rt=4.84721)
T/S Cluster: L−4.8−10
L544 (m/z=808.543061; rt=4.83352)
T/S Cluster: L−4.8−10
L527 (m/z=781.589217; rt=4.80932)
T/S Cluster: L−4.8−10
L521 (m/z=824.559558; rt=4.80697)
T/S Cluster: L−4.8−10
L550 (m/z=389.756667; rt=4.83557)
T/S Cluster: L−4.8−10
L554 (m/z=389.762262; rt=4.83566)
T/S Cluster: L−4.8−10
L530 (m/z=391.269306; rt=4.8107)
T/S Cluster: L−4.8−10
L535 (m/z=746.485037; rt=4.8115)
T/S Cluster: L−4.8−10
L536 (m/z=746.503308; rt=4.81194)
T/S Cluster: L−4.8−10
PCo1
PCo2
0.4
0.2
0.0
−0.2
−0.4
−0.6

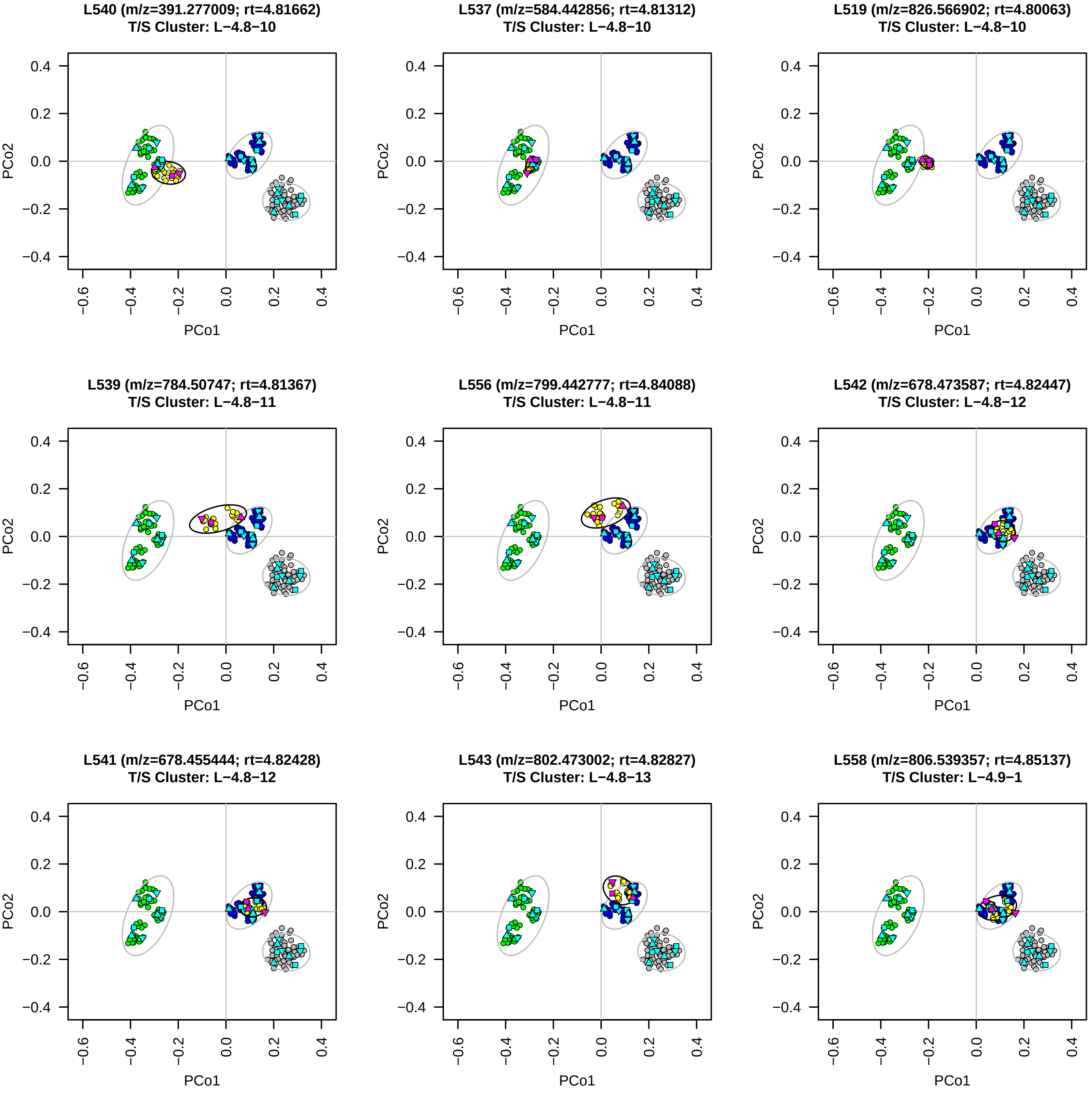
L540 (m/z=391.277009; rt=4.81662)
T/S Cluster: L−4.8−10
L537 (m/z=584.442856; rt=4.81312)
T/S Cluster: L−4.8−10
L519 (m/z=826.566902; rt=4.80063)
T/S Cluster: L−4.8−10
L539 (m/z=784.50747; rt=4.81367)
T/S Cluster: L−4.8−11
L556 (m/z=799.442777; rt=4.84088)
T/S Cluster: L−4.8−11
L542 (m/z=678.473587; rt=4.82447)
T/S Cluster: L−4.8−12
L541 (m/z=678.455444; rt=4.82428)
T/S Cluster: L−4.8−12
L543 (m/z=802.473002; rt=4.82827)
T/S Cluster: L−4.8−13
L558 (m/z=806.539357; rt=4.85137)
T/S Cluster: L−4.9−1
PCo1
PCo2
−0.6
−0.4
−0.2
0.0
0.2
0.4

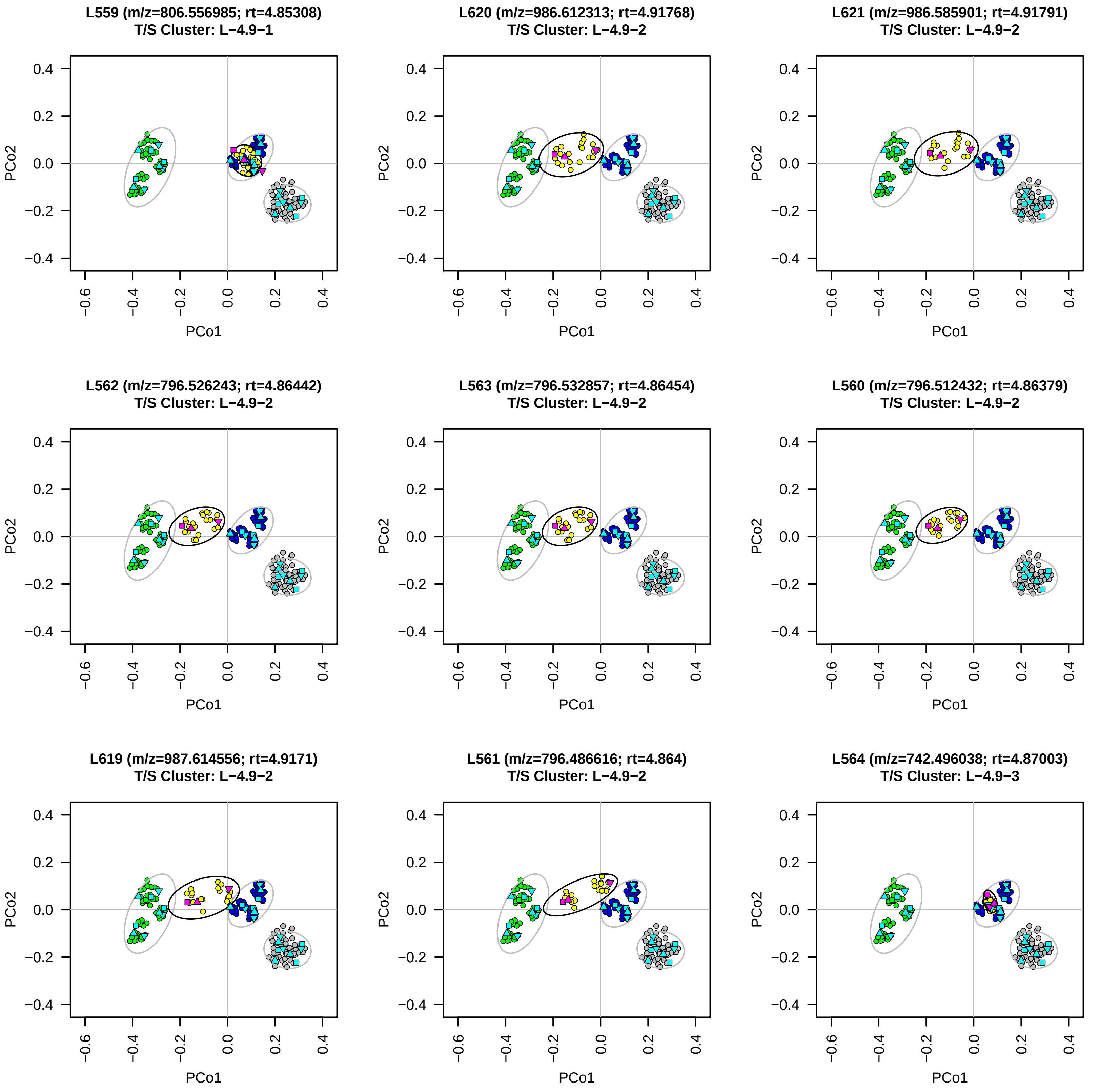
L559 (m/z=806.556985; rt=4.85308)
T/S Cluster: L−4.9−1
L620 (m/z=986.612313; rt=4.91768)
T/S Cluster: L−4.9−2
L621 (m/z=986.585901; rt=4.91791)
T/S Cluster: L−4.9−2
L562 (m/z=796.526243; rt=4.86442)
T/S Cluster: L−4.9−2
L563 (m/z=796.532857; rt=4.86454)
T/S Cluster: L−4.9−2
L560 (m/z=796.512432; rt=4.86379)
T/S Cluster: L−4.9−2
L619 (m/z=987.614556; rt=4.9171)
T/S Cluster: L−4.9−2
L561 (m/z=796.486616; rt=4.864)
T/S Cluster: L−4.9−2
L564 (m/z=742.496038; rt=4.87003)
T/S Cluster: L−4.9−3
PCo1
PCo2
−0.6
−0.4
−0.2
0.0
0.2
0.4

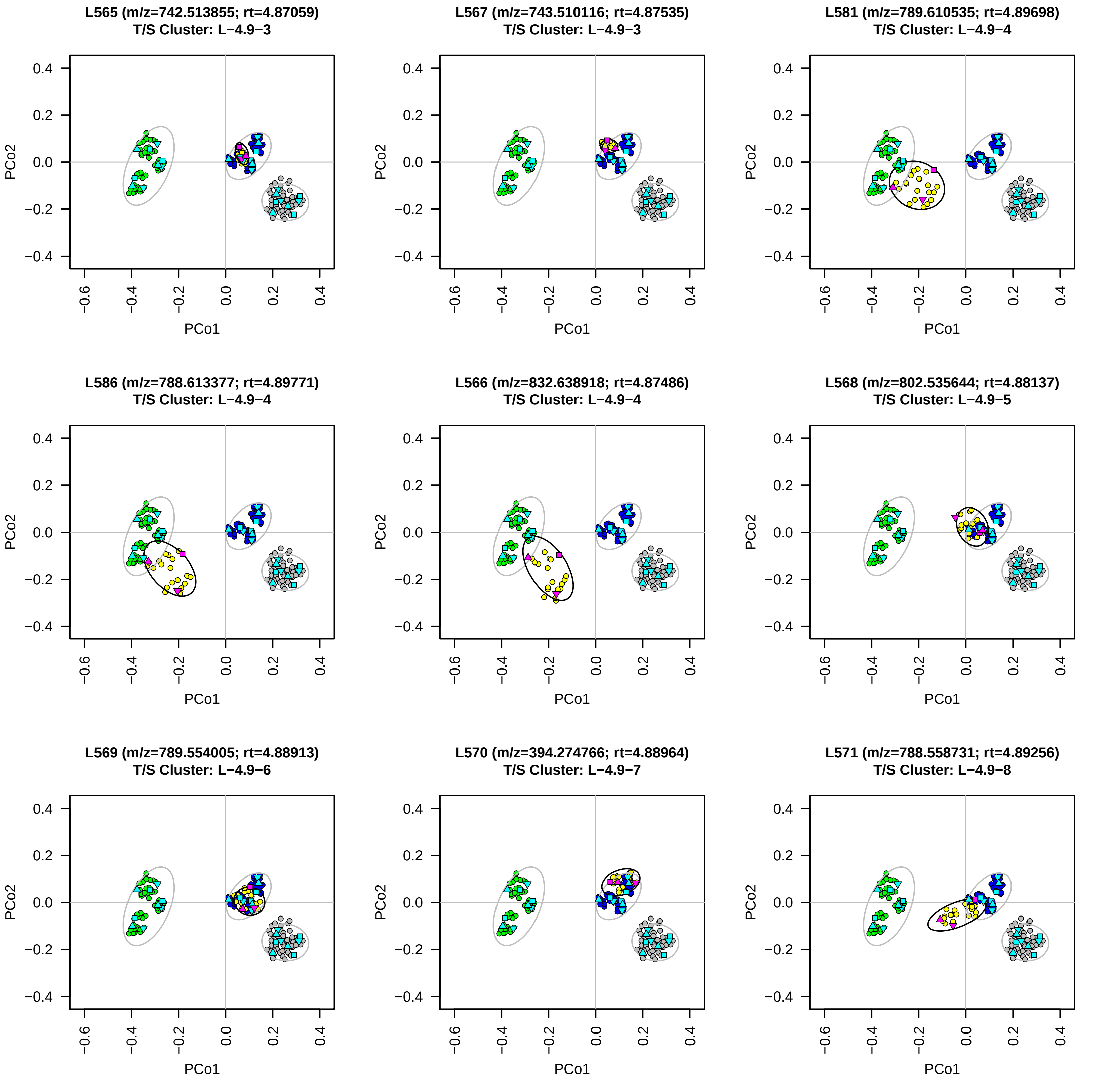
L565 (m/z=742.513855; rt=4.87059)
T/S Cluster: L−4.9−3
L567 (m/z=743.510116; rt=4.87535)
T/S Cluster: L−4.9−3
L581 (m/z=789.610535; rt=4.89698)
T/S Cluster: L−4.9−4
L586 (m/z=788.613377; rt=4.89771)
T/S Cluster: L−4.9−4
L566 (m/z=832.638918; rt=4.87486)
T/S Cluster: L−4.9−4
L568 (m/z=802.535644; rt=4.88137)
T/S Cluster: L−4.9−5
L569 (m/z=789.554005; rt=4.88913)
T/S Cluster: L−4.9−6
L570 (m/z=394.274766; rt=4.88964)
T/S Cluster: L−4.9−7
L571 (m/z=788.558731; rt=4.89256)
T/S Cluster: L−4.9−8
PCo1
PCo2
−0.6
−0.4
−0.2
0.0
0.2
0.4

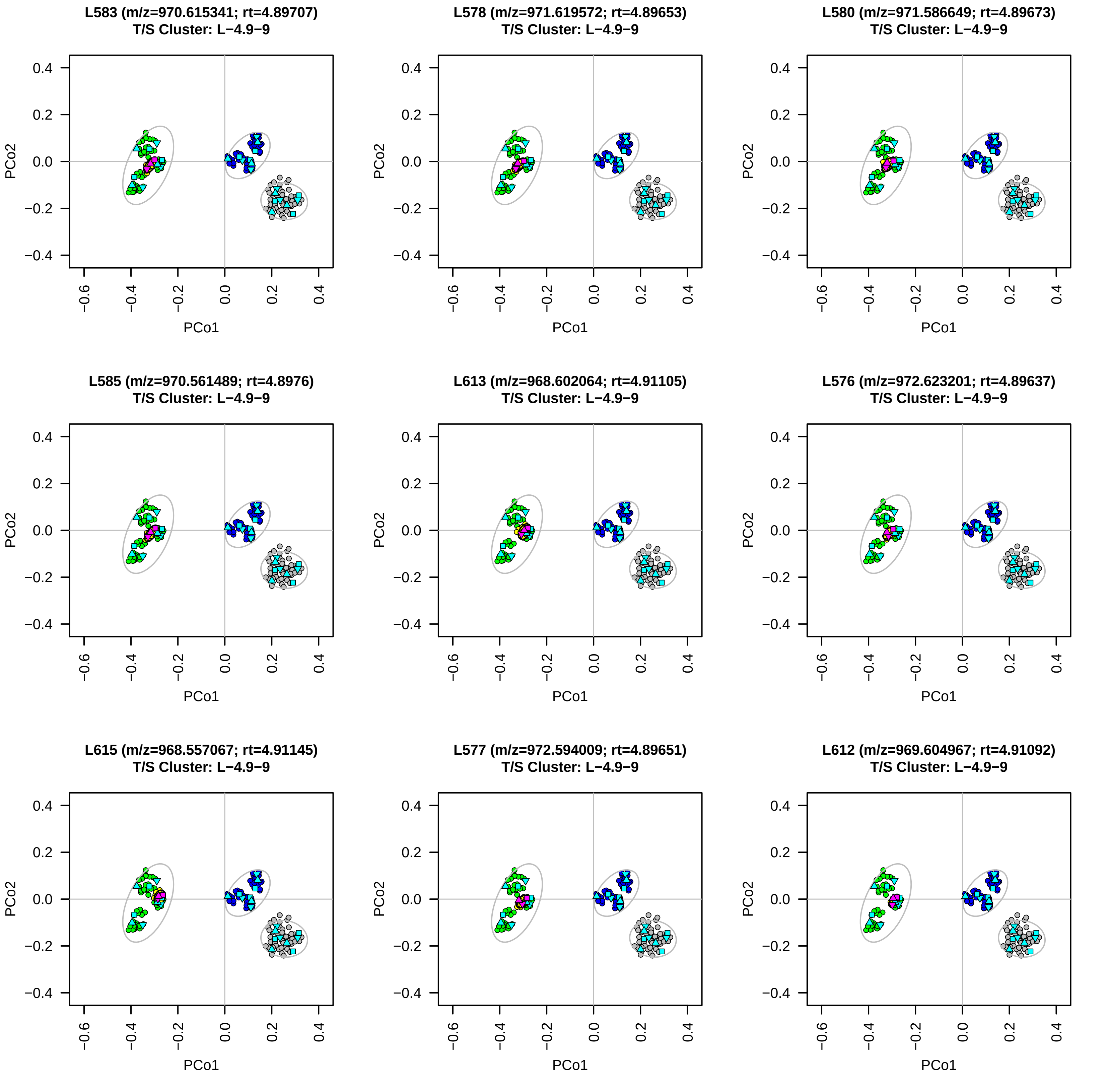
L583 (m/z=970.615341; rt=4.89707)
T/S Cluster: L−4.9−9
L578 (m/z=971.619572; rt=4.89653)
T/S Cluster: L−4.9−9
L580 (m/z=971.586649; rt=4.89673)
T/S Cluster: L−4.9−9
L585 (m/z=970.561489; rt=4.8976)
T/S Cluster: L−4.9−9
L613 (m/z=968.602064; rt=4.91105)
T/S Cluster: L−4.9−9
L576 (m/z=972.623201; rt=4.89637)
T/S Cluster: L−4.9−9
L615 (m/z=968.557067; rt=4.91145)
T/S Cluster: L−4.9−9
L577 (m/z=972.594009; rt=4.89651)
T/S Cluster: L−4.9−9
L612 (m/z=969.604967; rt=4.91092)
T/S Cluster: L−4.9−9
PCo1
PCo2
0.4
0.2
0.0
−0.2
−0.4
−0.6

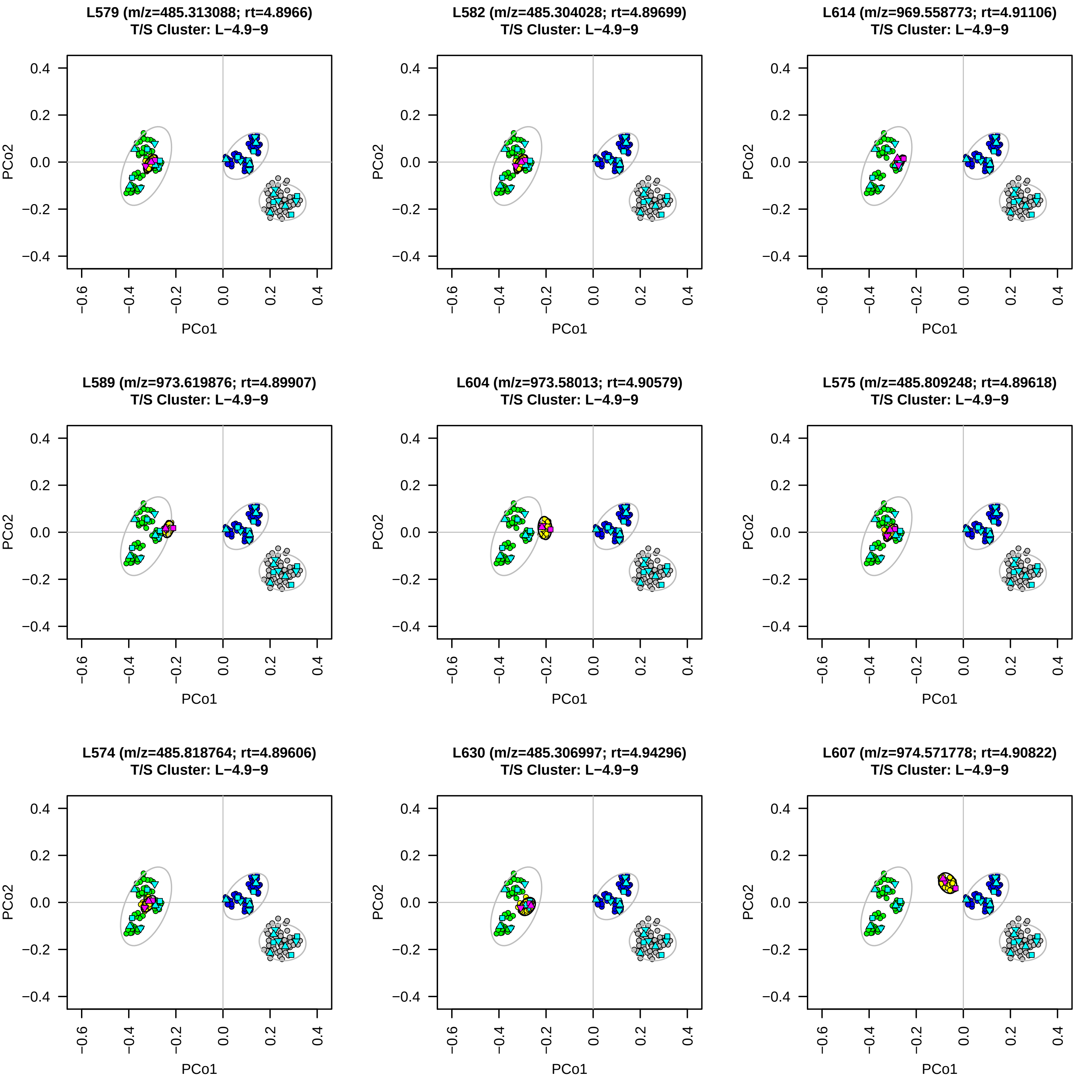
L579 (m/z=485.313088; rt=4.8966)
T/S Cluster: L−4.9−9
L582 (m/z=485.304028; rt=4.89699)
T/S Cluster: L−4.9−9
L614 (m/z=969.558773; rt=4.91106)
T/S Cluster: L−4.9−9
L589 (m/z=973.619876; rt=4.89907)
T/S Cluster: L−4.9−9
L604 (m/z=973.58013; rt=4.90579)
T/S Cluster: L−4.9−9
L575 (m/z=485.809248; rt=4.89618)
T/S Cluster: L−4.9−9
L574 (m/z=485.818764; rt=4.89606)
T/S Cluster: L−4.9−9
L630 (m/z=485.306997; rt=4.94296)
T/S Cluster: L−4.9−9
L607 (m/z=974.571778; rt=4.90822)
T/S Cluster: L−4.9−9
PCo1
PCo2

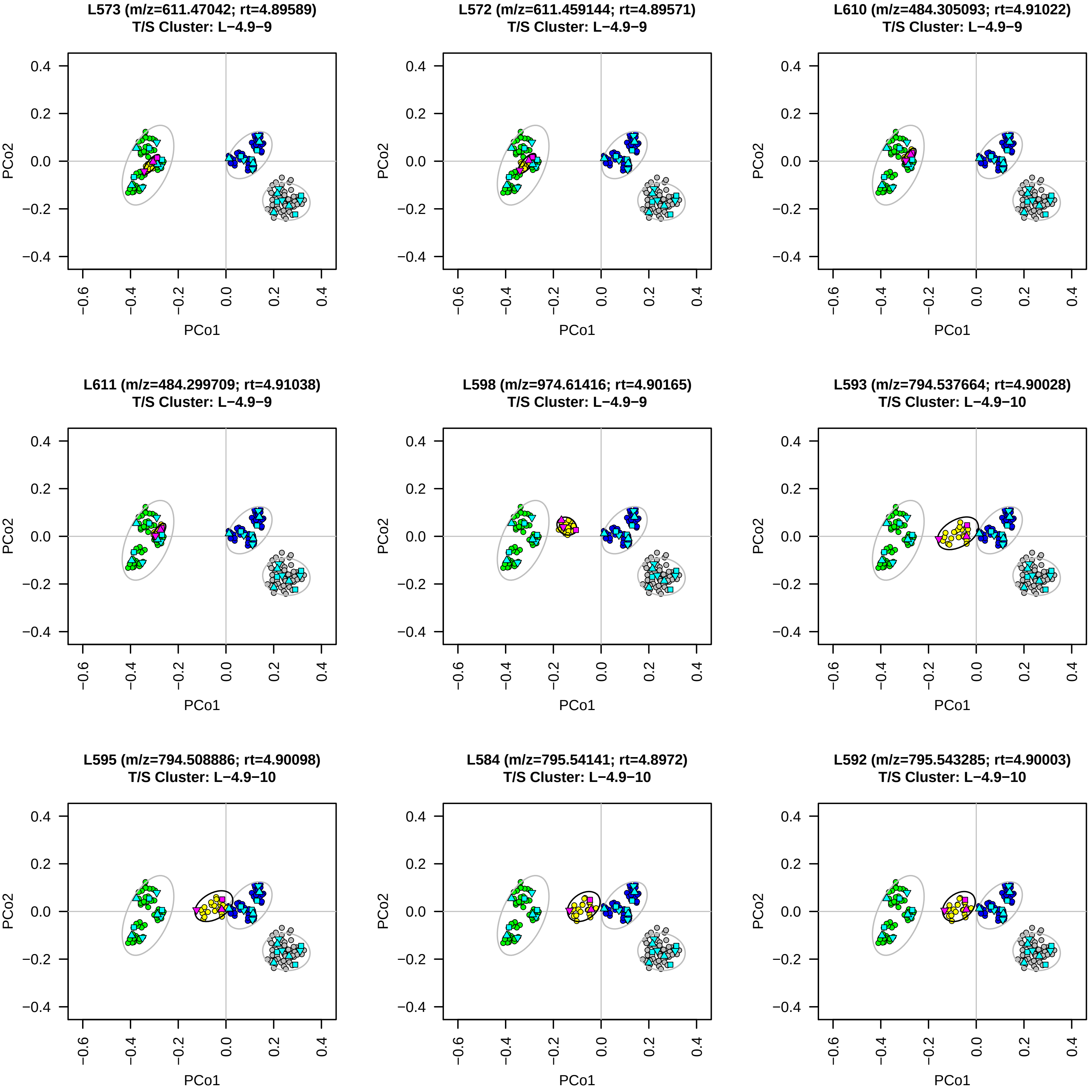
L573 (m/z=611.47042; rt=4.89589)
T/S Cluster: L−4.9−9
L572 (m/z=611.459144; rt=4.89571)
T/S Cluster: L−4.9−9
L610 (m/z=484.305093; rt=4.91022)
T/S Cluster: L−4.9−9
L611 (m/z=484.299709; rt=4.91038)
T/S Cluster: L−4.9−9
L598 (m/z=974.61416; rt=4.90165)
T/S Cluster: L−4.9−9
L593 (m/z=794.537664; rt=4.90028)
T/S Cluster: L−4.9−10
L595 (m/z=794.508886; rt=4.90098)
T/S Cluster: L−4.9−10
L584 (m/z=795.54141; rt=4.8972)
T/S Cluster: L−4.9−10
L592 (m/z=795.543285; rt=4.90003)
T/S Cluster: L−4.9−10
PCo1
PCo2
−0.6
−0.4
−0.2
0.0
0.2
0.4

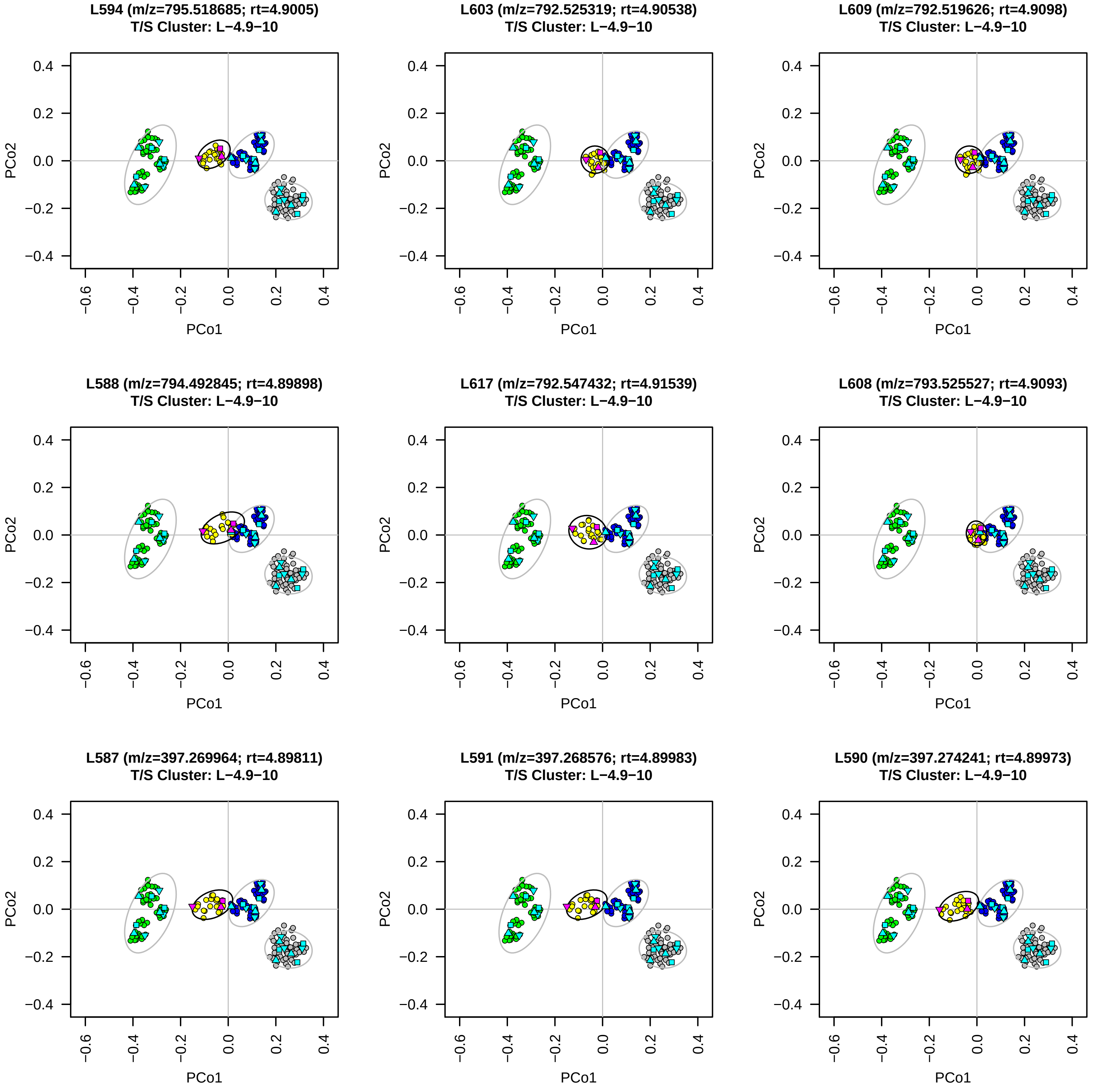
L594 (m/z=795.518685; rt=4.9005)
T/S Cluster: L−4.9−10
L603 (m/z=792.525319; rt=4.90538)
T/S Cluster: L−4.9−10
L609 (m/z=792.519626; rt=4.9098)
T/S Cluster: L−4.9−10
L588 (m/z=794.492845; rt=4.89898)
T/S Cluster: L−4.9−10
L617 (m/z=792.547432; rt=4.91539)
T/S Cluster: L−4.9−10
L608 (m/z=793.525527; rt=4.9093)
T/S Cluster: L−4.9−10
L587 (m/z=397.269964; rt=4.89811)
T/S Cluster: L−4.9−10
L591 (m/z=397.268576; rt=4.89983)
T/S Cluster: L−4.9−10
L590 (m/z=397.274241; rt=4.89973)
T/S Cluster: L−4.9−10
PCo1
PCo2
0.4
0.2
0.0
−0.2
−0.4
−0.6

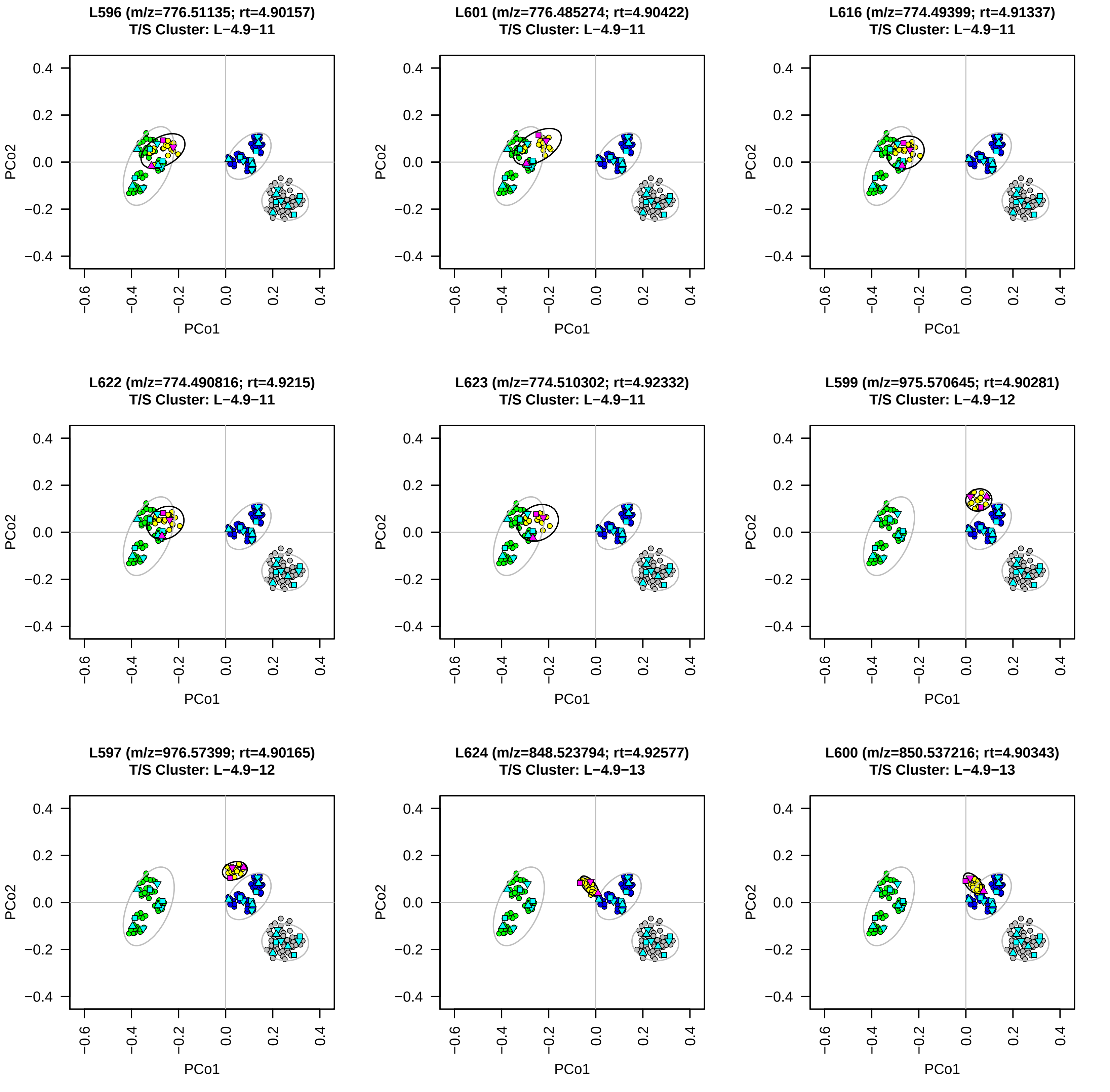
L596 (m/z=776.51135; rt=4.90157)
T/S Cluster: L−4.9−11
L601 (m/z=776.485274; rt=4.90422)
T/S Cluster: L−4.9−11
L616 (m/z=774.49399; rt=4.91337)
T/S Cluster: L−4.9−11
L622 (m/z=774.490816; rt=4.9215)
T/S Cluster: L−4.9−11
L623 (m/z=774.510302; rt=4.92332)
T/S Cluster: L−4.9−11
L599 (m/z=975.570645; rt=4.90281)
T/S Cluster: L−4.9−12
L597 (m/z=976.57399; rt=4.90165)
T/S Cluster: L−4.9−12
L624 (m/z=848.523794; rt=4.92577)
T/S Cluster: L−4.9−13
L600 (m/z=850.537216; rt=4.90343)
T/S Cluster: L−4.9−13
PCo1
PCo2
0.4
0.2
0.0
−0.2
−0.4
−0.6

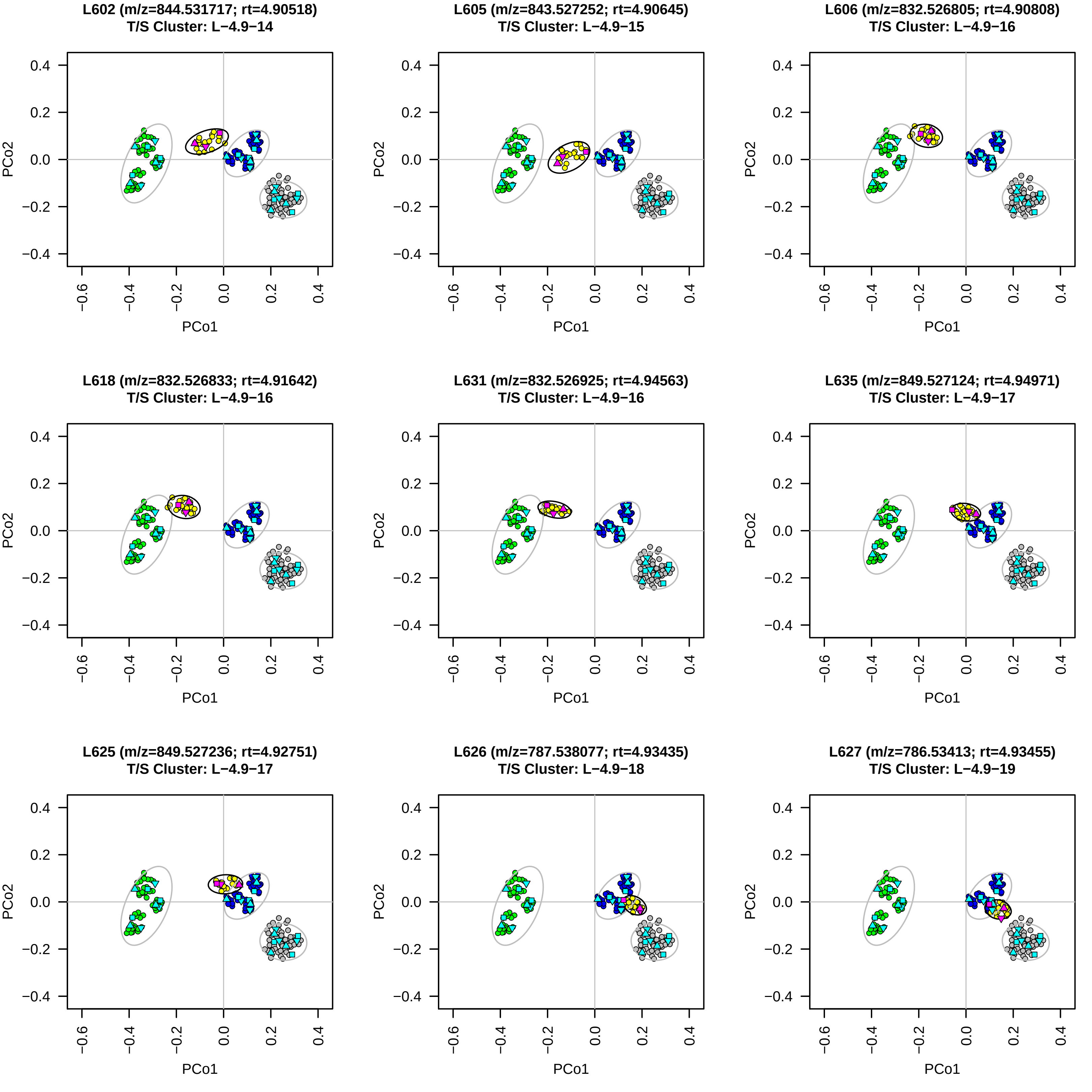
L602 (m/z=844.531717; rt=4.90518)
T/S Cluster: L−4.9−14
L605 (m/z=843.527252; rt=4.90645)
T/S Cluster: L−4.9−15
L606 (m/z=832.526805; rt=4.90808)
T/S Cluster: L−4.9−16
L618 (m/z=832.526833; rt=4.91642)
T/S Cluster: L−4.9−16
L631 (m/z=832.526925; rt=4.94563)
T/S Cluster: L−4.9−16
L635 (m/z=849.527124; rt=4.94971)
T/S Cluster: L−4.9−17
L625 (m/z=849.527236; rt=4.92751)
T/S Cluster: L−4.9−17
L626 (m/z=787.538077; rt=4.93435)
T/S Cluster: L−4.9−18
L627 (m/z=786.53413; rt=4.93455)
T/S Cluster: L−4.9−19
PCo1
PCo2
0.4
0.2
0.0
−0.2
−0.4
−0.6

L628 (m/z=789.555027; rt=4.94026)
T/S Cluster: L−4.9−20

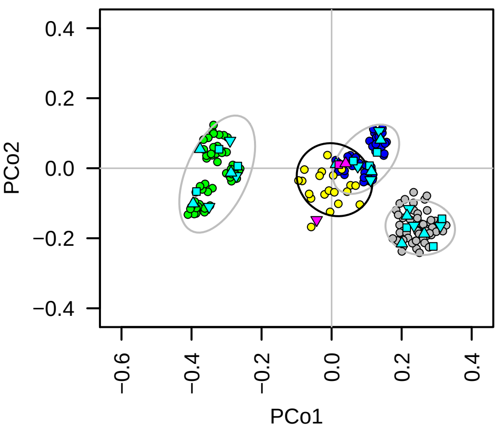

L629 (m/z=787.496937; rt=4.94149)
T/S Cluster: L−4.9−21

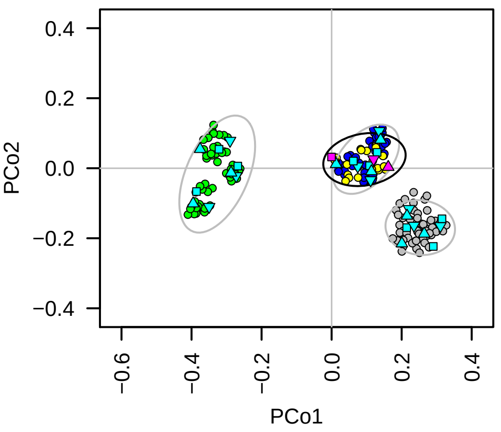

L632 (m/z=810.555224; rt=4.94575)
T/S Cluster: L−4.9−22

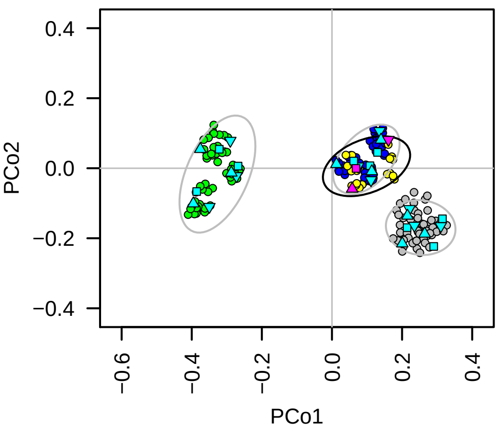

L633 (m/z=810.530376; rt=4.94704)
T/S Cluster: L−4.9−22

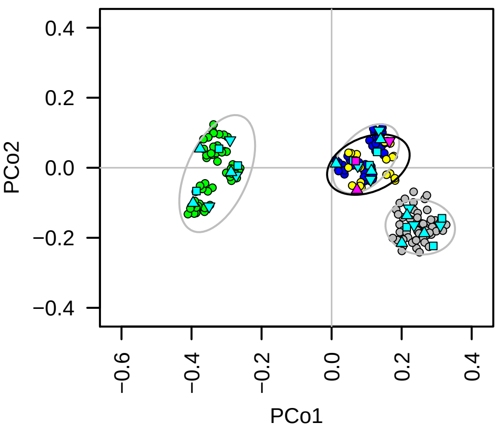

L634 (m/z=786.486972; rt=4.94739)
T/S Cluster: L−4.9−23

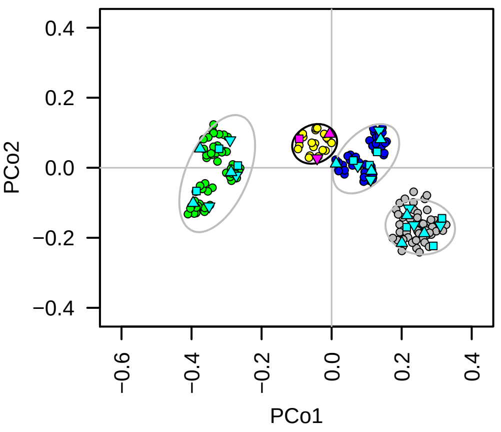

L648 (m/z=780.529036; rt=4.9531)
T/S Cluster: L−5−1

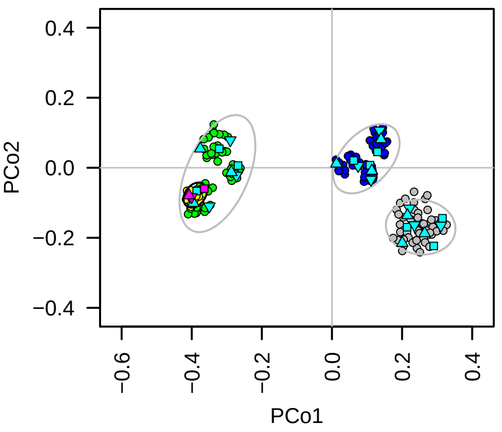

L649 (m/z=780.528818; rt=4.9531)
T/S Cluster: L−5−1

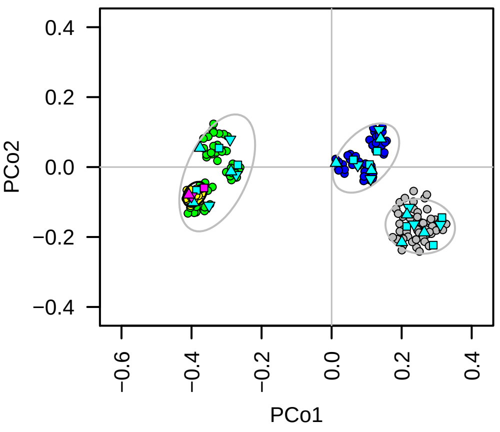

L644 (m/z=781.532906; rt=4.95285)
T/S Cluster: L−5−1

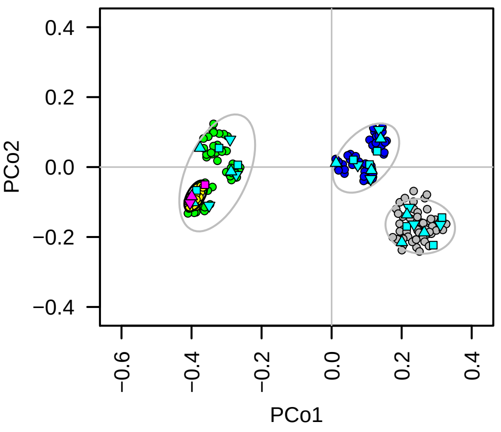

L645 (m/z=781.532657; rt=4.95286)
T/S Cluster: L−5−1

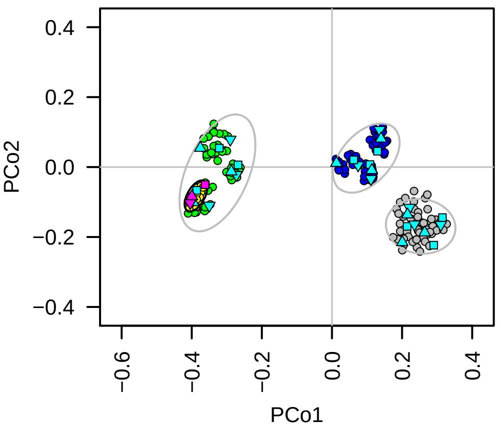

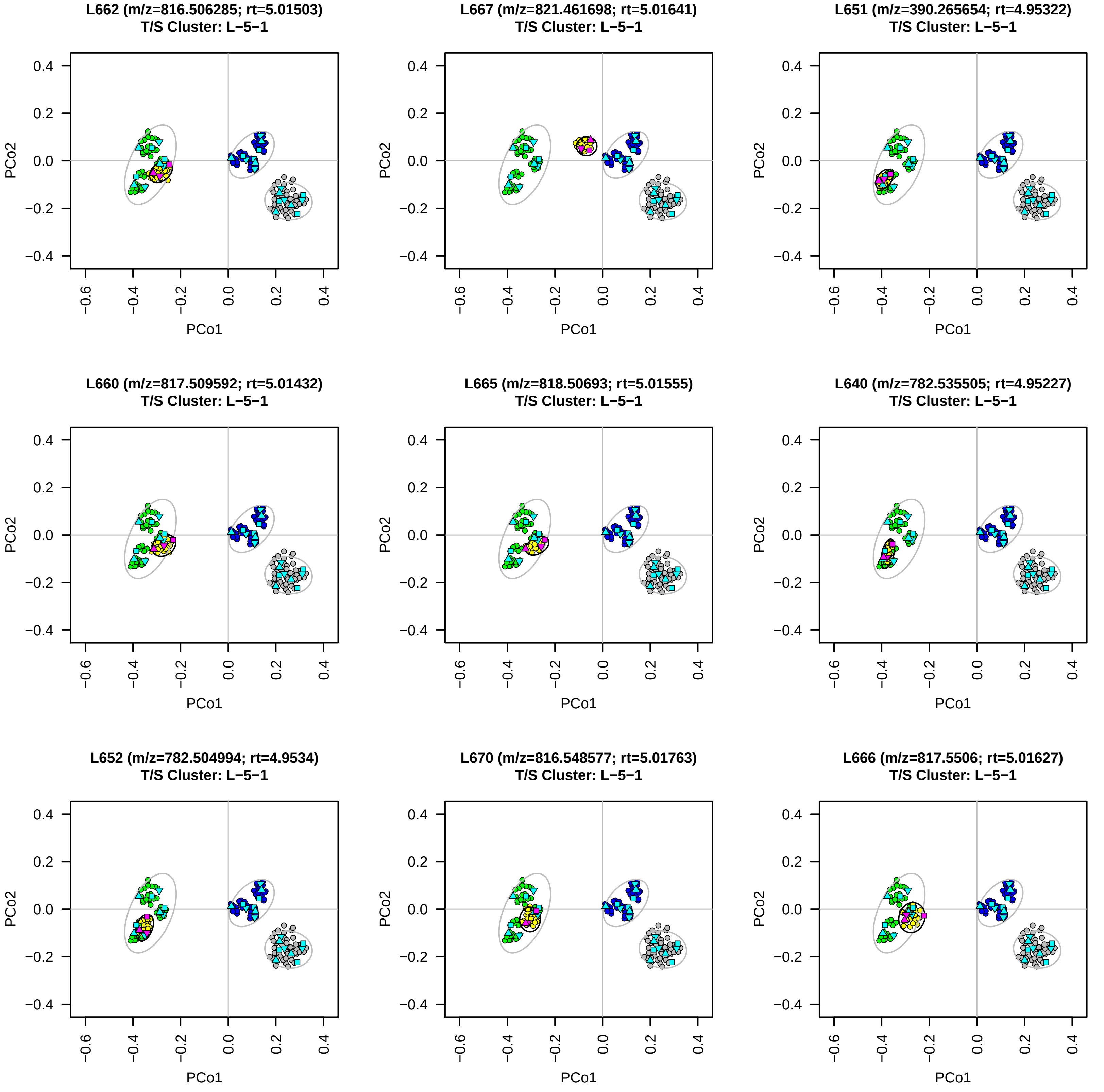

L662 (m/z=816.506285; rt=5.01503)
T/S Cluster: L−5−1
L667 (m/z=821.461698; rt=5.01641)
T/S Cluster: L−5−1
L651 (m/z=390.265654; rt=4.95322)
T/S Cluster: L−5−1
L660 (m/z=817.509592; rt=5.01432)
T/S Cluster: L−5−1
L665 (m/z=818.50693; rt=5.01555)
T/S Cluster: L−5−1
L640 (m/z=782.535505; rt=4.95227)
T/S Cluster: L−5−1
L652 (m/z=782.504994; rt=4.9534)
T/S Cluster: L−5−1
L670 (m/z=816.548577; rt=5.01763)
T/S Cluster: L−5−1
L666 (m/z=817.5506; rt=5.01627)
T/S Cluster: L−5−1
PCo1
PCo2
−0.6
−0.4
−0.2
0.0
0.2
0.4

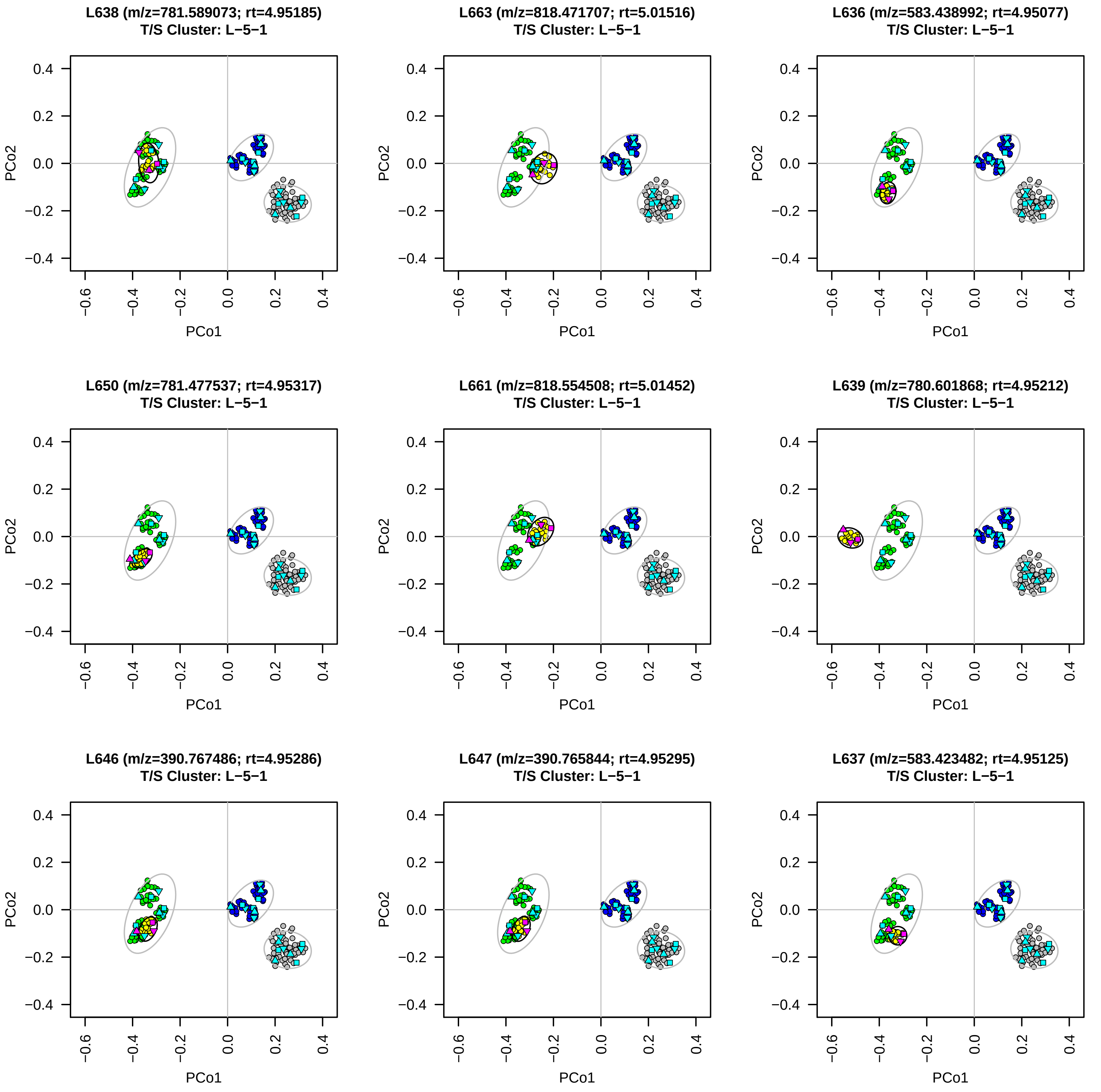

L638 (m/z=781.589073; rt=4.95185)
T/S Cluster: L−5−1
L663 (m/z=818.471707; rt=5.01516)
T/S Cluster: L−5−1
L636 (m/z=583.438992; rt=4.95077)
T/S Cluster: L−5−1
L650 (m/z=781.477537; rt=4.95317)
T/S Cluster: L−5−1
L661 (m/z=818.554508; rt=5.01452)
T/S Cluster: L−5−1
L639 (m/z=780.601868; rt=4.95212)
T/S Cluster: L−5−1
L646 (m/z=390.767486; rt=4.95286)
T/S Cluster: L−5−1
L647 (m/z=390.765844; rt=4.95295)
T/S Cluster: L−5−1
L637 (m/z=583.423482; rt=4.95125)
T/S Cluster: L−5−1
PCo1
PCo2

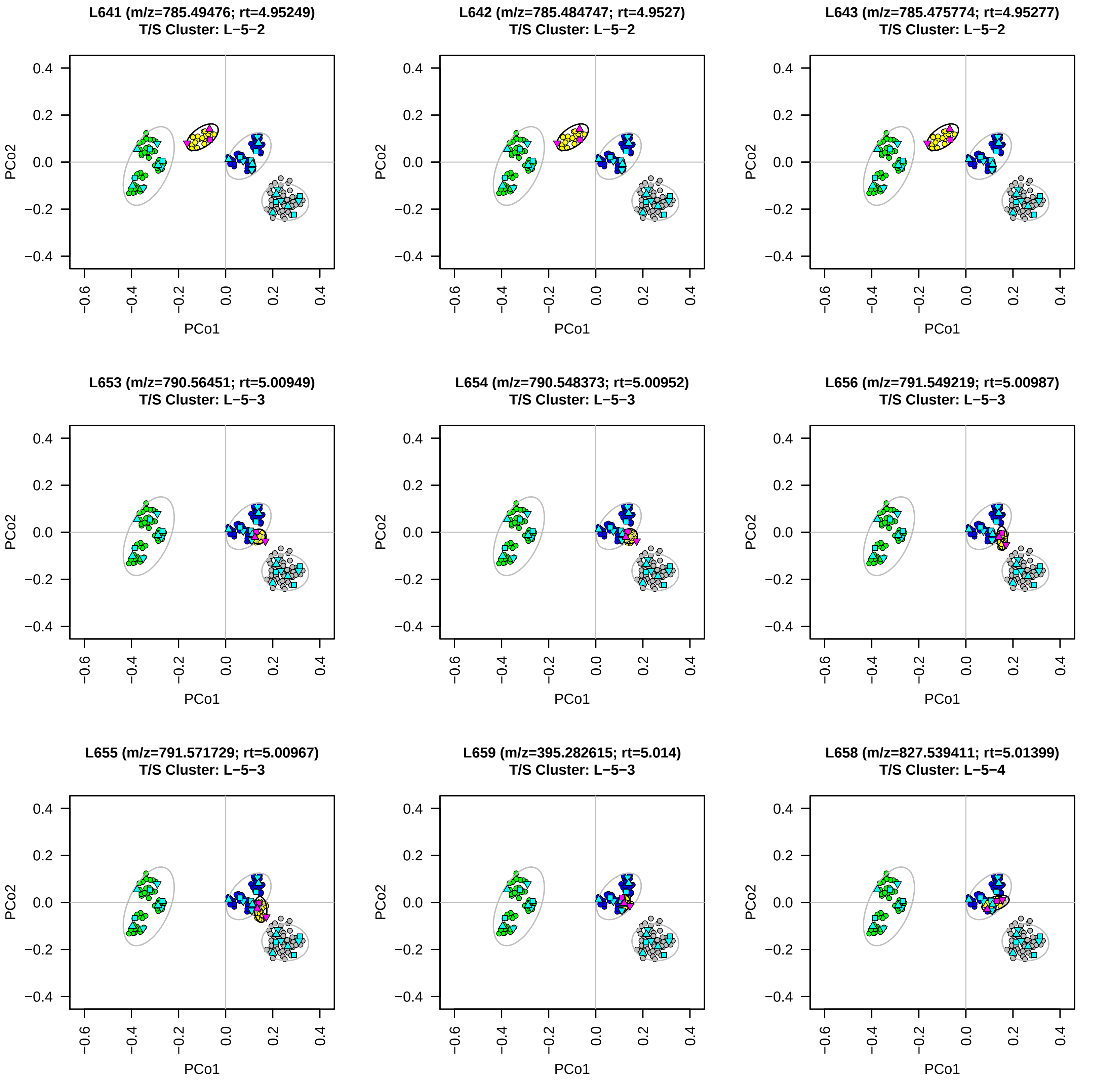
L641 (m/z=785.49476; rt=4.95249)
T/S Cluster: L−5−2
L642 (m/z=785.484747; rt=4.9527)
T/S Cluster: L−5−2
L643 (m/z=785.475774; rt=4.95277)
T/S Cluster: L−5−2
L653 (m/z=790.56451; rt=5.00949)
T/S Cluster: L−5−3
L654 (m/z=790.548373; rt=5.00952)
T/S Cluster: L−5−3
L656 (m/z=791.549219; rt=5.00987)
T/S Cluster: L−5−3
L655 (m/z=791.571729; rt=5.00967)
T/S Cluster: L−5−3
L659 (m/z=395.282615; rt=5.014)
T/S Cluster: L−5−3
L658 (m/z=827.539411; rt=5.01399)
T/S Cluster: L−5−4
PCo1
PCo2

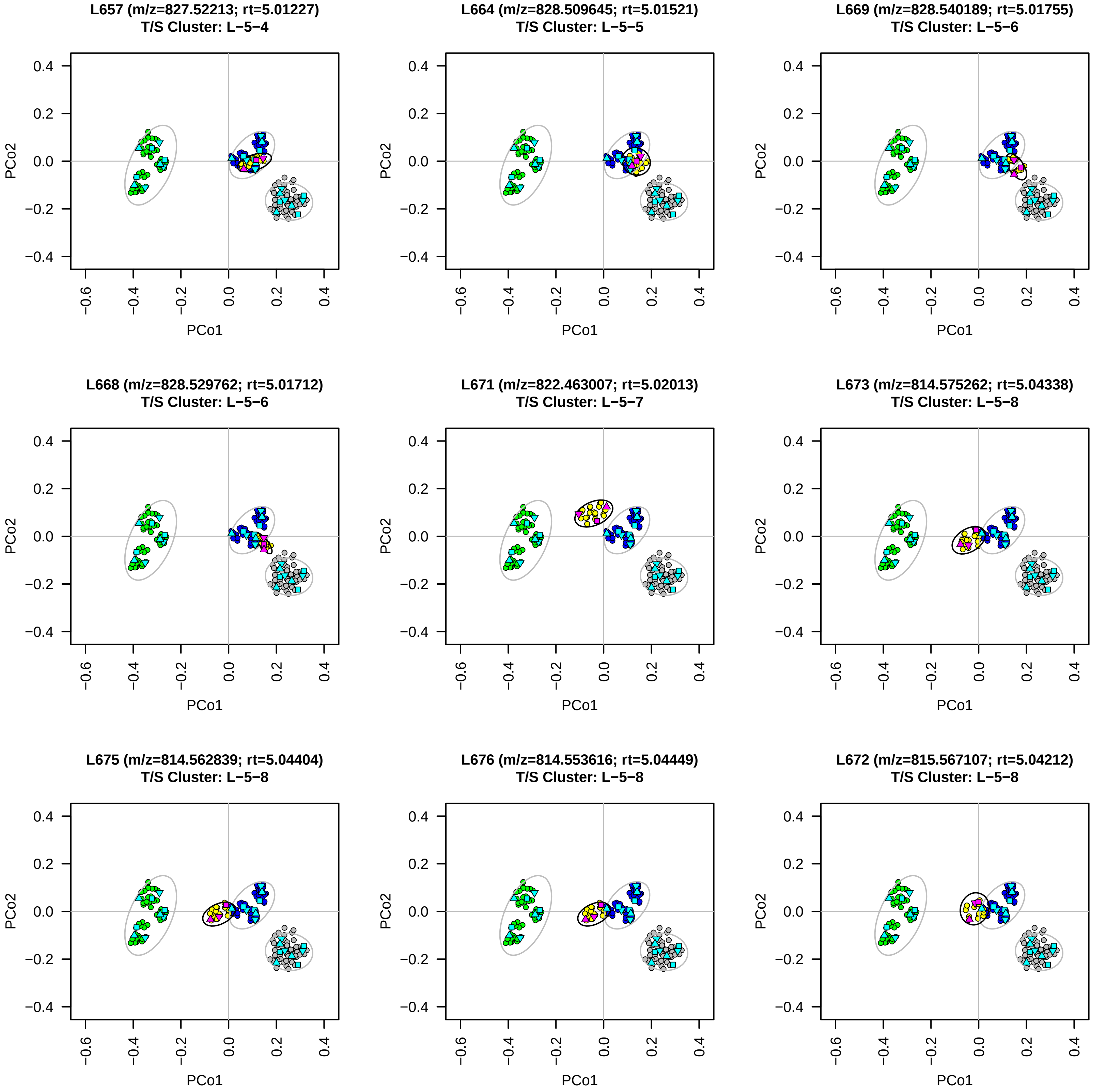
L657 (m/z=827.52213; rt=5.01227)
T/S Cluster: L−5−4
L664 (m/z=828.509645; rt=5.01521)
T/S Cluster: L−5−5
L669 (m/z=828.540189; rt=5.01755)
T/S Cluster: L−5−6
L668 (m/z=828.529762; rt=5.01712)
T/S Cluster: L−5−6
L671 (m/z=822.463007; rt=5.02013)
T/S Cluster: L−5−7
L673 (m/z=814.575262; rt=5.04338)
T/S Cluster: L−5−8
L675 (m/z=814.562839; rt=5.04404)
T/S Cluster: L−5−8
L676 (m/z=814.553616; rt=5.04449)
T/S Cluster: L−5−8
L672 (m/z=815.567107; rt=5.04212)
T/S Cluster: L−5−8
PCo1
PCo2
−0.6
−0.4
−0.2
0.0
0.2
0.4

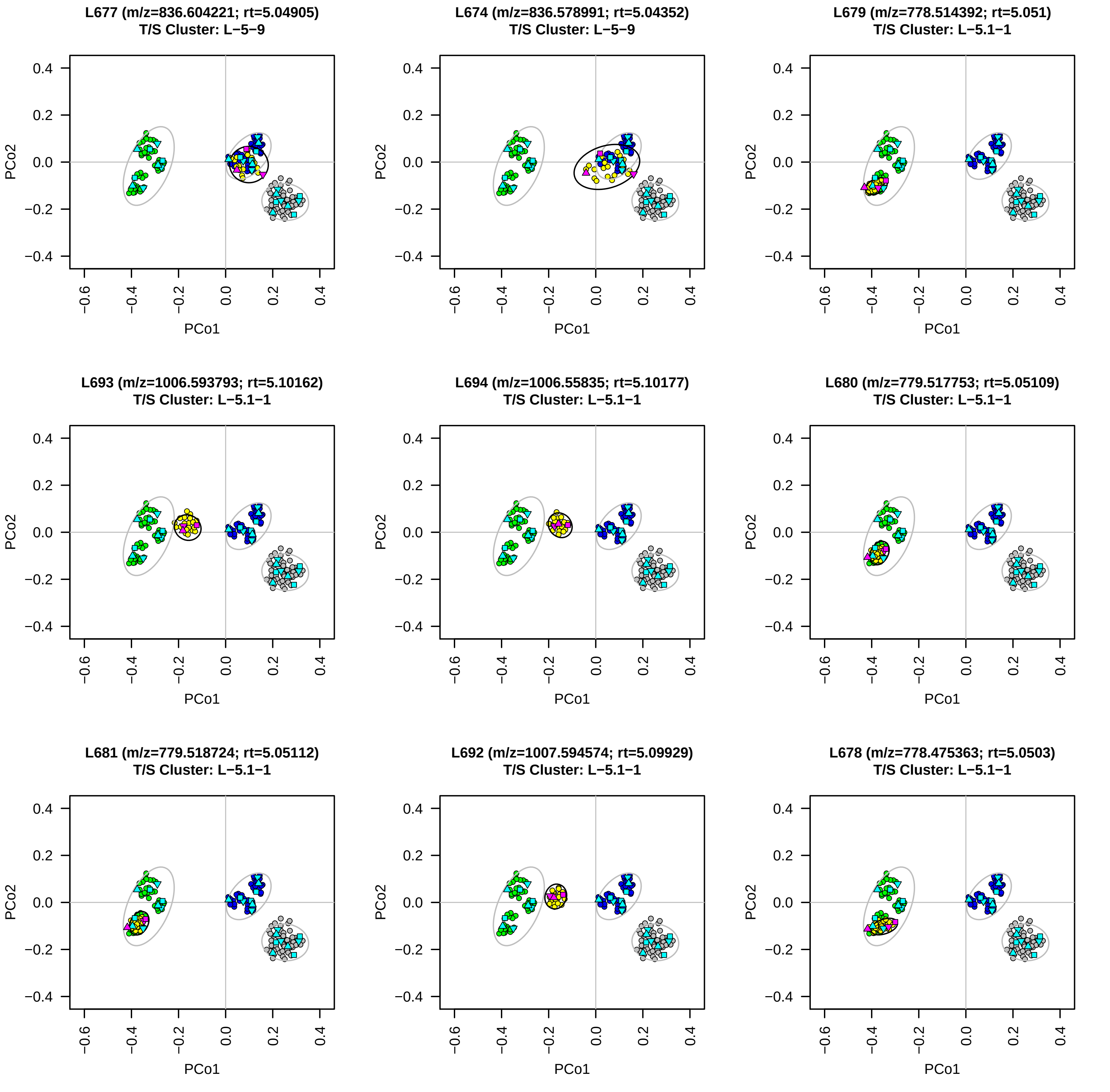
L677 (m/z=836.604221; rt=5.04905)
T/S Cluster: L−5−9
L674 (m/z=836.578991; rt=5.04352)
T/S Cluster: L−5−9
L679 (m/z=778.514392; rt=5.051)
T/S Cluster: L−5.1−1
L693 (m/z=1006.593793; rt=5.10162)
T/S Cluster: L−5.1−1
L694 (m/z=1006.55835; rt=5.10177)
T/S Cluster: L−5.1−1
L680 (m/z=779.517753; rt=5.05109)
T/S Cluster: L−5.1−1
L681 (m/z=779.518724; rt=5.05112)
T/S Cluster: L−5.1−1
L692 (m/z=1007.594574; rt=5.09929)
T/S Cluster: L−5.1−1
L678 (m/z=778.475363; rt=5.0503)
T/S Cluster: L−5.1−1
PCo1
PCo2
0.4
0.2
0.0
−0.2
−0.4
−0.6

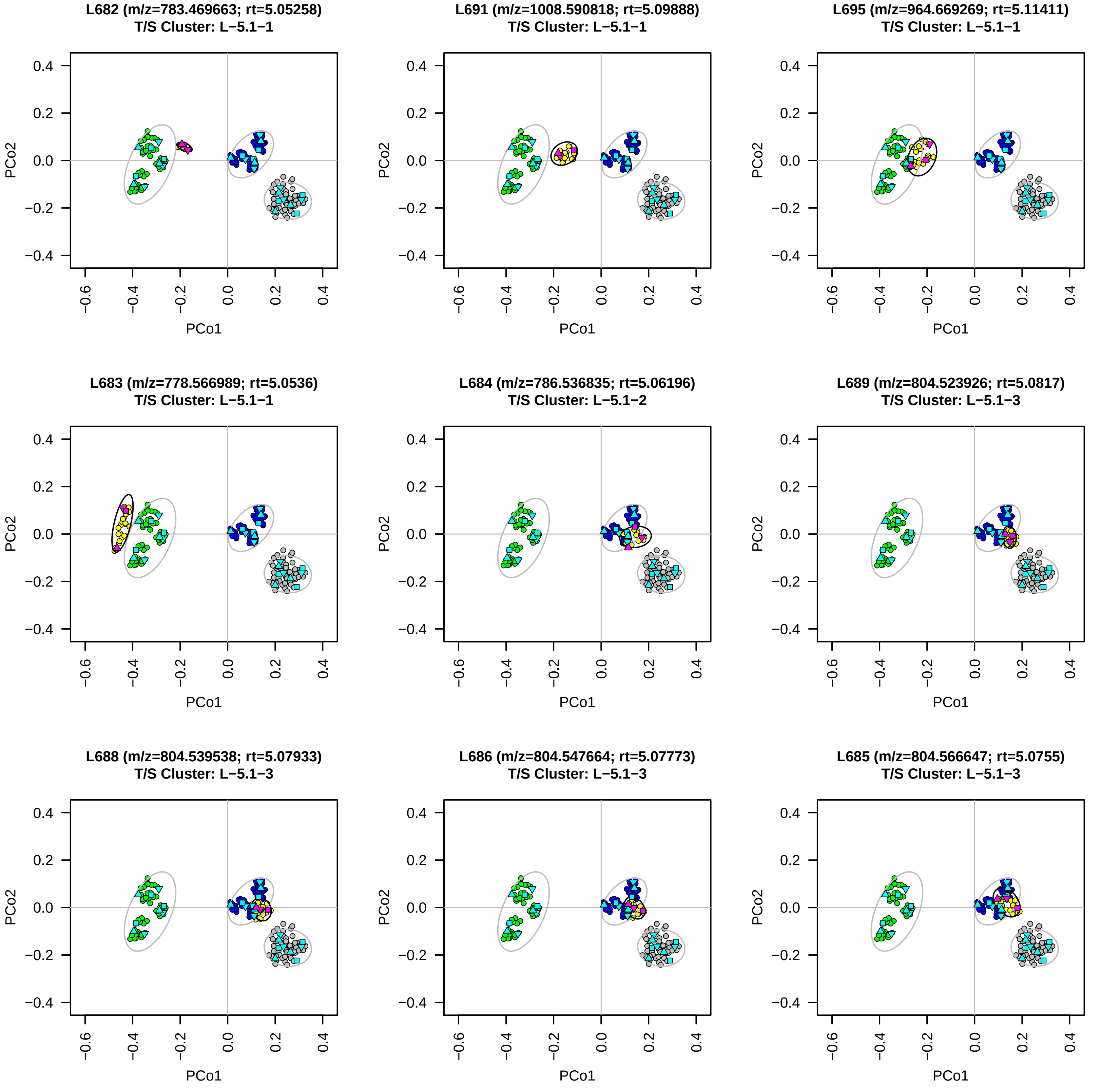
L682 (m/z=783.469663; rt=5.05258)
T/S Cluster: L−5.1−1
L691 (m/z=1008.590818; rt=5.09888)
T/S Cluster: L−5.1−1
L695 (m/z=964.669269; rt=5.11411)
T/S Cluster: L−5.1−1
L683 (m/z=778.566989; rt=5.0536)
T/S Cluster: L−5.1−1
L684 (m/z=786.536835; rt=5.06196)
T/S Cluster: L−5.1−2
L689 (m/z=804.523926; rt=5.0817)
T/S Cluster: L−5.1−3
L688 (m/z=804.539538; rt=5.07933)
T/S Cluster: L−5.1−3
L686 (m/z=804.547664; rt=5.07773)
T/S Cluster: L−5.1−3
L685 (m/z=804.566647; rt=5.0755)
T/S Cluster: L−5.1−3
PCo1
PCo2
−0.6
−0.4
−0.2
0.0
0.2
0.4

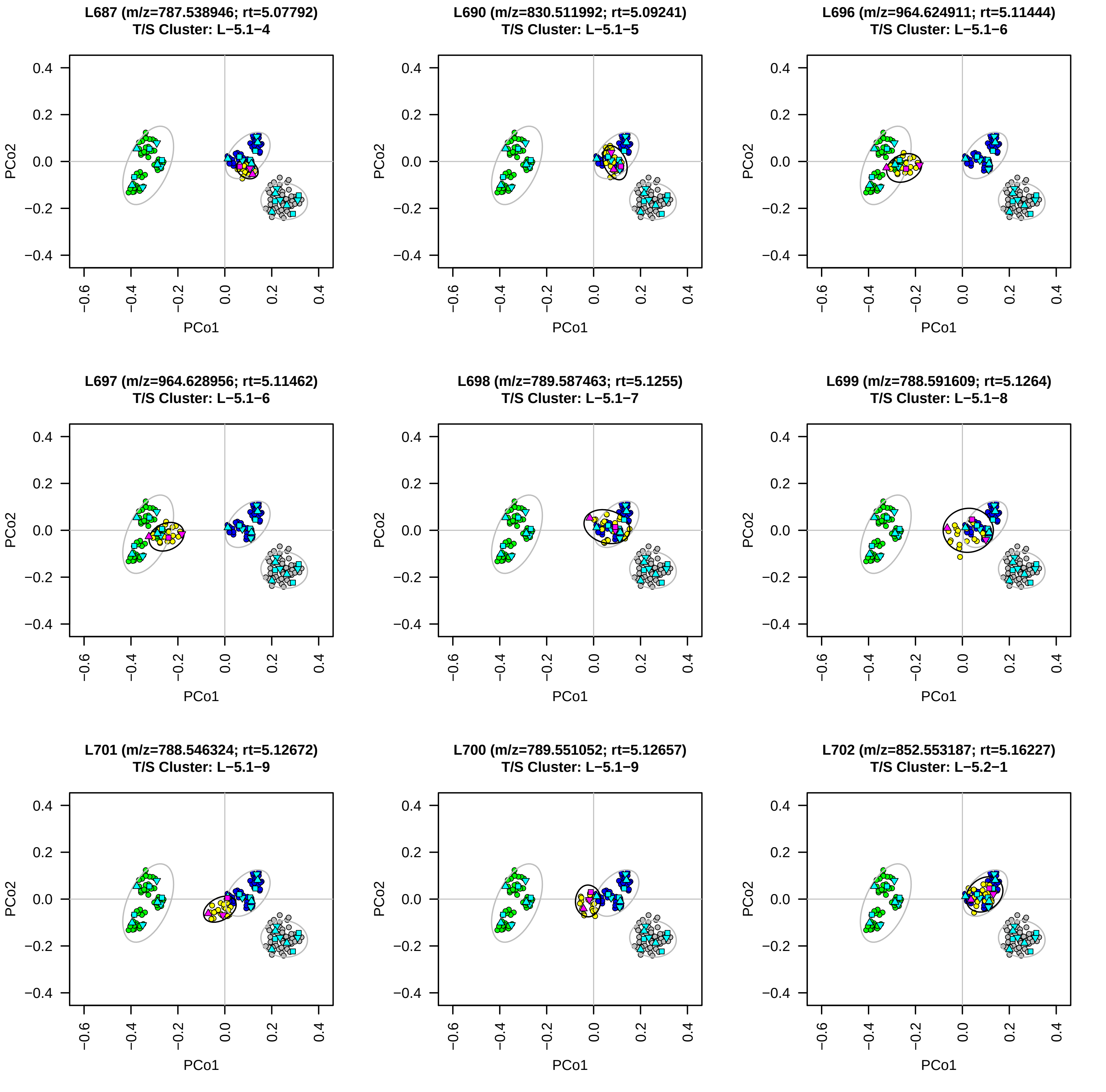
L687 (m/z=787.538946; rt=5.07792)
T/S Cluster: L−5.1−4
L690 (m/z=830.511992; rt=5.09241)
T/S Cluster: L−5.1−5
L696 (m/z=964.624911; rt=5.11444)
T/S Cluster: L−5.1−6
L697 (m/z=964.628956; rt=5.11462)
T/S Cluster: L−5.1−6
L698 (m/z=789.587463; rt=5.1255)
T/S Cluster: L−5.1−7
L699 (m/z=788.591609; rt=5.1264)
T/S Cluster: L−5.1−8
L701 (m/z=788.546324; rt=5.12672)
T/S Cluster: L−5.1−9
L700 (m/z=789.551052; rt=5.12657)
T/S Cluster: L−5.1−9
L702 (m/z=852.553187; rt=5.16227)
T/S Cluster: L−5.2−1
PCo1
PCo2
0.4
0.2
0.0
−0.2
−0.4
−0.6

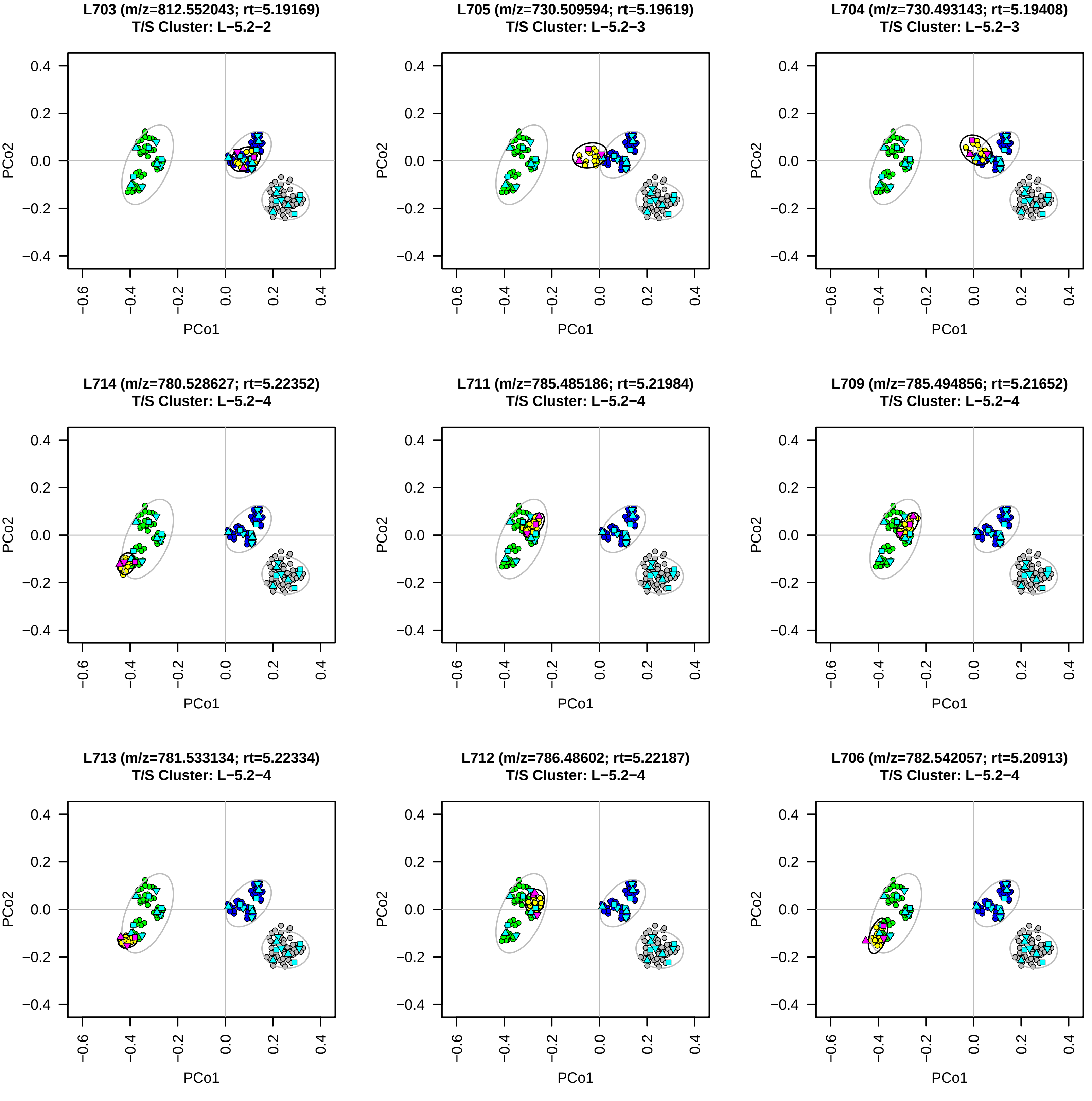

L703 (m/z=812.552043; rt=5.19169)
T/S Cluster: L−5.2−2
L705 (m/z=730.509594; rt=5.19619)
T/S Cluster: L−5.2−3
L704 (m/z=730.493143; rt=5.19408)
T/S Cluster: L−5.2−3
L714 (m/z=780.528627; rt=5.22352)
T/S Cluster: L−5.2−4
L711 (m/z=785.485186; rt=5.21984)
T/S Cluster: L−5.2−4
L709 (m/z=785.494856; rt=5.21652)
T/S Cluster: L−5.2−4
L713 (m/z=781.533134; rt=5.22334)
T/S Cluster: L−5.2−4
L712 (m/z=786.48602; rt=5.22187)
T/S Cluster: L−5.2−4
L706 (m/z=782.542057; rt=5.20913)
T/S Cluster: L−5.2−4
PCo1
PCo2

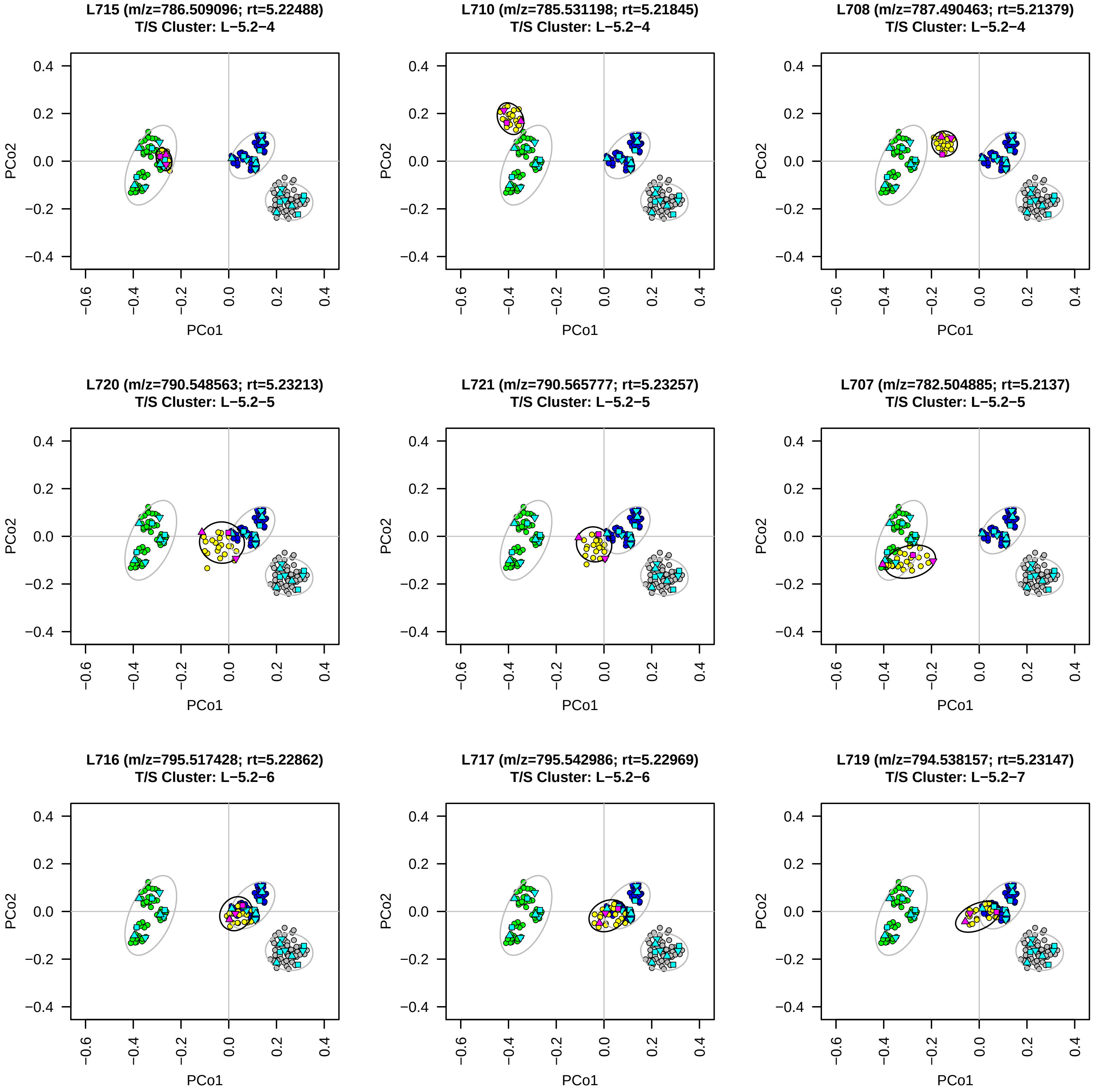
L715 (m/z=786.509096; rt=5.22488)
T/S Cluster: L−5.2−4
L710 (m/z=785.531198; rt=5.21845)
T/S Cluster: L−5.2−4
L708 (m/z=787.490463; rt=5.21379)
T/S Cluster: L−5.2−4
L720 (m/z=790.548563; rt=5.23213)
T/S Cluster: L−5.2−5
L721 (m/z=790.565777; rt=5.23257)
T/S Cluster: L−5.2−5
L707 (m/z=782.504885; rt=5.2137)
T/S Cluster: L−5.2−5
L716 (m/z=795.517428; rt=5.22862)
T/S Cluster: L−5.2−6
L717 (m/z=795.542986; rt=5.22969)
T/S Cluster: L−5.2−6
L719 (m/z=794.538157; rt=5.23147)
T/S Cluster: L−5.2−7
PCo1
PCo2

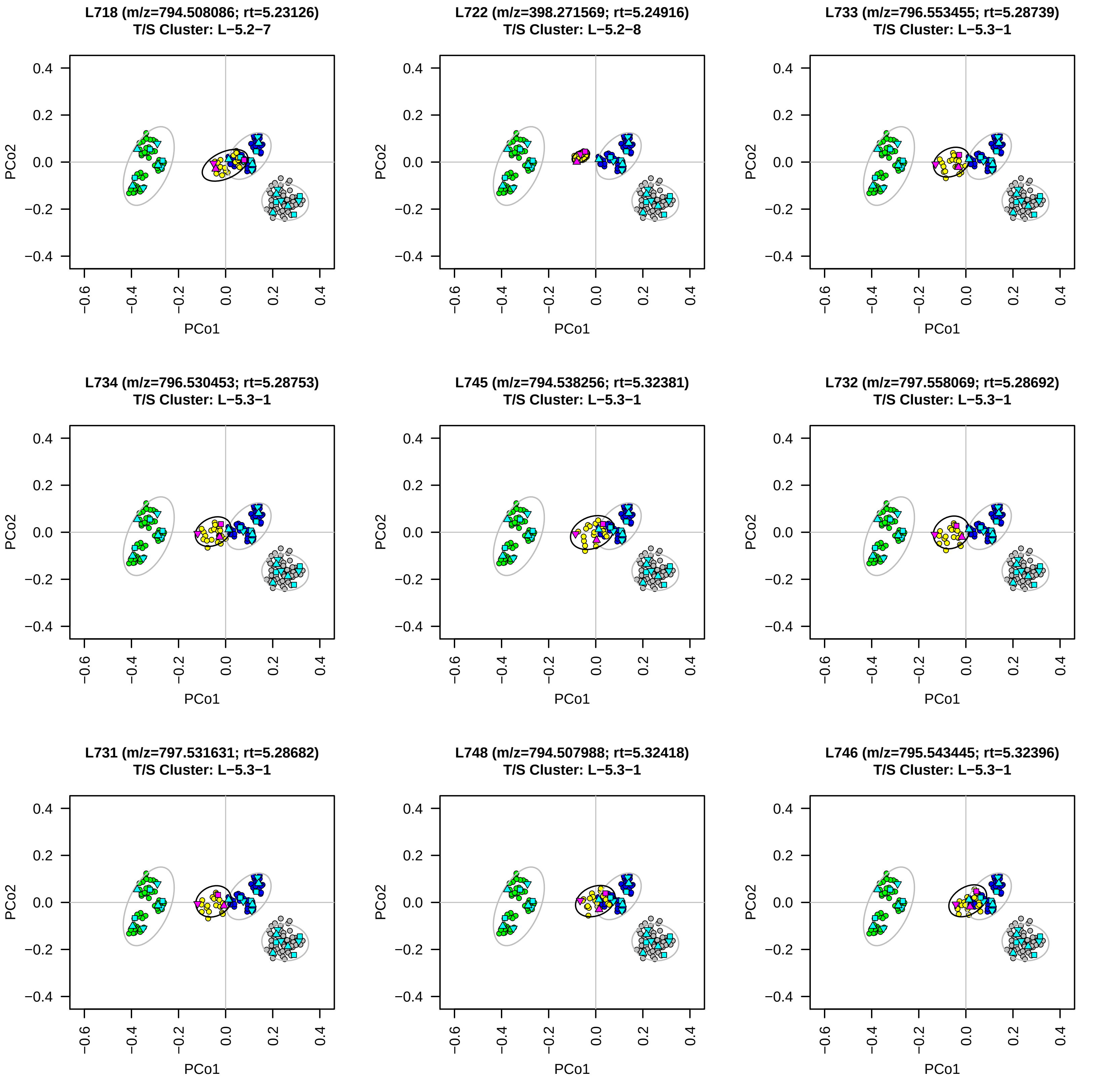
L718 (m/z=794.508086; rt=5.23126)
T/S Cluster: L−5.2−7
L722 (m/z=398.271569; rt=5.24916)
T/S Cluster: L−5.2−8
L733 (m/z=796.553455; rt=5.28739)
T/S Cluster: L−5.3−1
L734 (m/z=796.530453; rt=5.28753)
T/S Cluster: L−5.3−1
L745 (m/z=794.538256; rt=5.32381)
T/S Cluster: L−5.3−1
L732 (m/z=797.558069; rt=5.28692)
T/S Cluster: L−5.3−1
L731 (m/z=797.531631; rt=5.28682)
T/S Cluster: L−5.3−1
L748 (m/z=794.507988; rt=5.32418)
T/S Cluster: L−5.3−1
L746 (m/z=795.543445; rt=5.32396)
T/S Cluster: L−5.3−1
PCo1
PCo2
0.4
0.2
0.0
−0.2
−0.4
−0.6

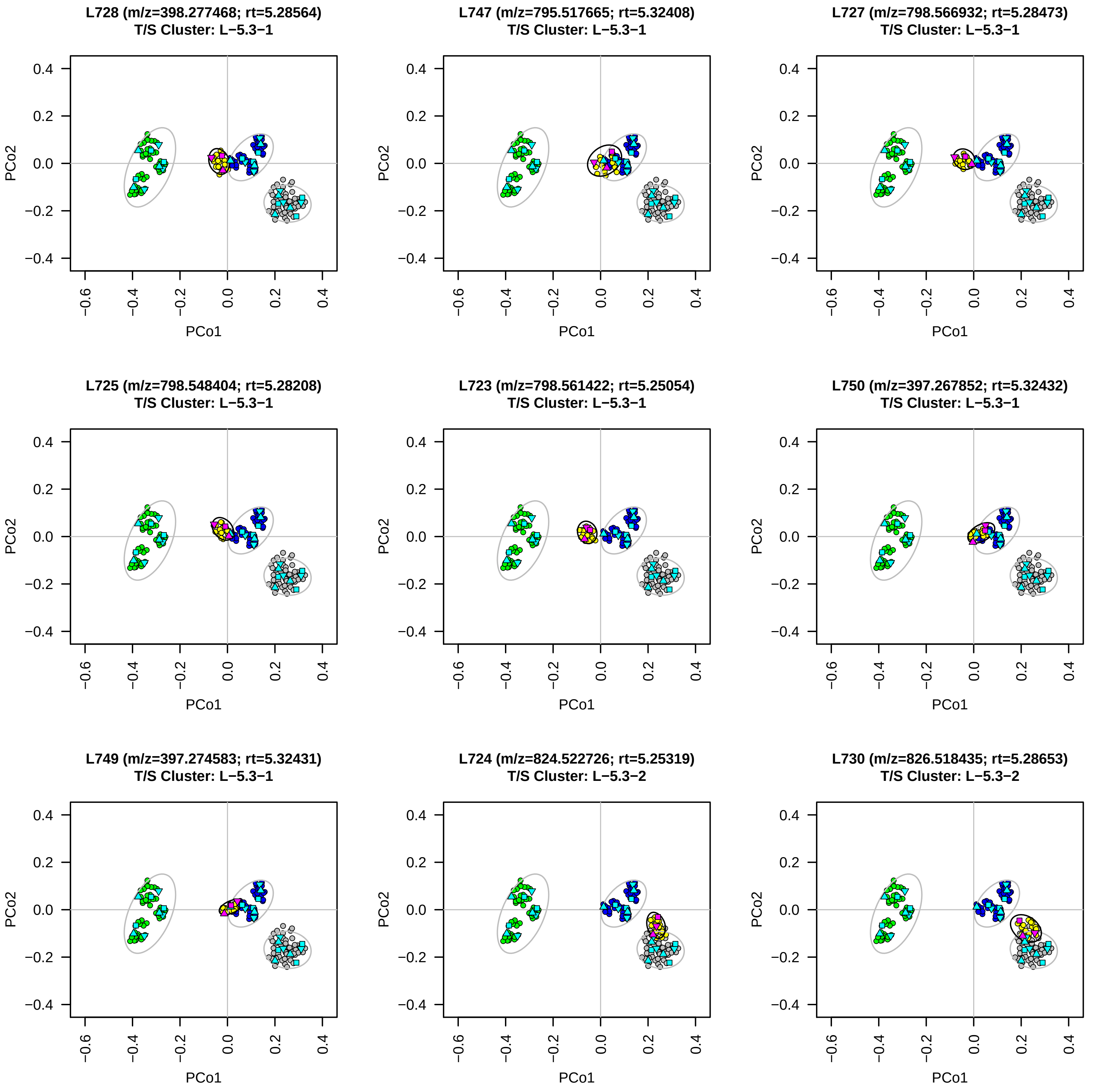
L728 (m/z=398.277468; rt=5.28564)
T/S Cluster: L−5.3−1
L747 (m/z=795.517665; rt=5.32408)
T/S Cluster: L−5.3−1
L727 (m/z=798.566932; rt=5.28473)
T/S Cluster: L−5.3−1
L725 (m/z=798.548404; rt=5.28208)
T/S Cluster: L−5.3−1
L723 (m/z=798.561422; rt=5.25054)
T/S Cluster: L−5.3−1
L750 (m/z=397.267852; rt=5.32432)
T/S Cluster: L−5.3−1
L749 (m/z=397.274583; rt=5.32431)
T/S Cluster: L−5.3−1
L724 (m/z=824.522726; rt=5.25319)
T/S Cluster: L−5.3−2
L730 (m/z=826.518435; rt=5.28653)
T/S Cluster: L−5.3−2
PCo1
PCo2
0.4
0.2
0.0
−0.2
−0.4
−0.6

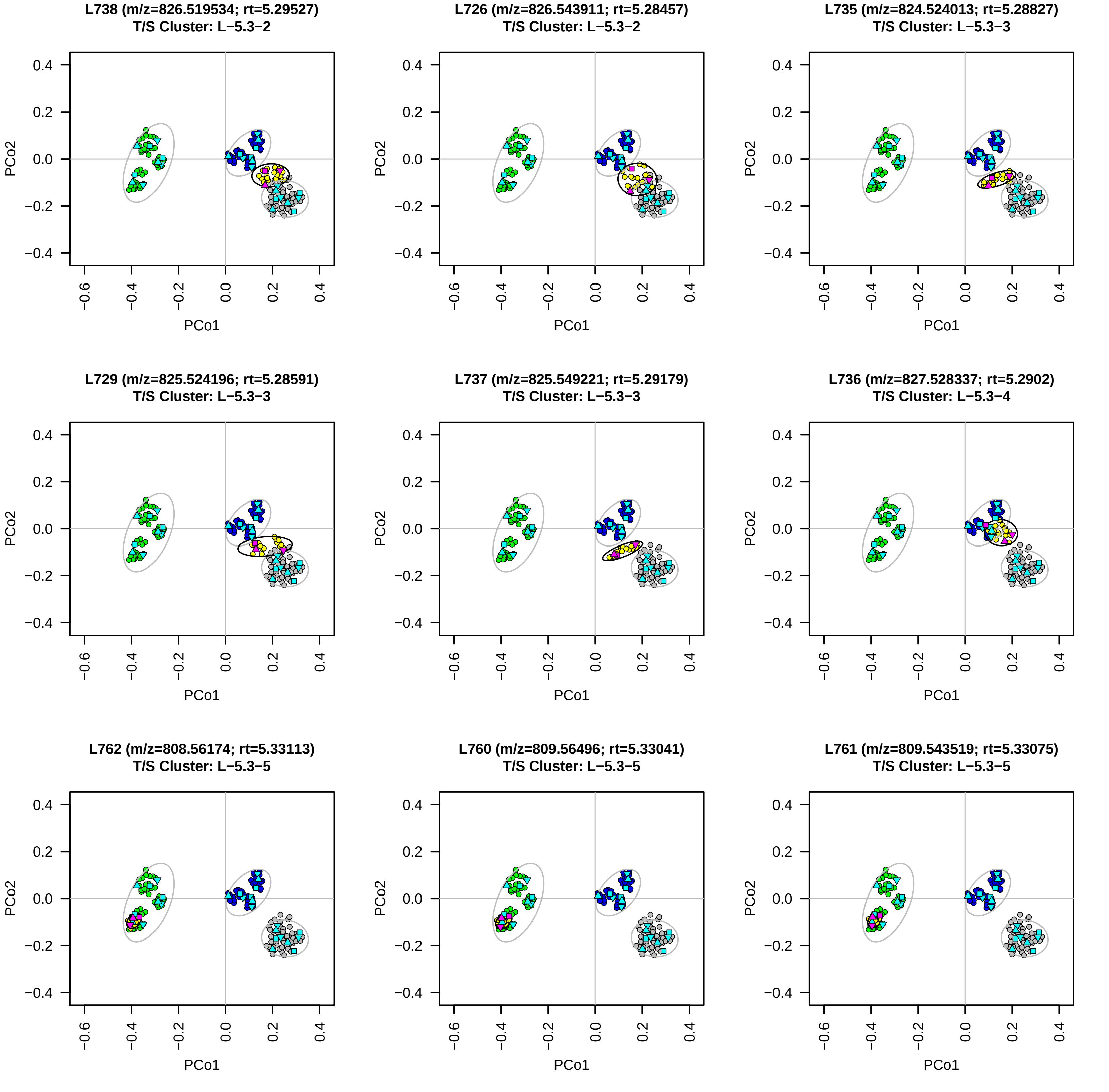
L738 (m/z=826.519534; rt=5.29527)
T/S Cluster: L−5.3−2
L726 (m/z=826.543911; rt=5.28457)
T/S Cluster: L−5.3−2
L735 (m/z=824.524013; rt=5.28827)
T/S Cluster: L−5.3−3
L729 (m/z=825.524196; rt=5.28591)
T/S Cluster: L−5.3−3
L737 (m/z=825.549221; rt=5.29179)
T/S Cluster: L−5.3−3
L736 (m/z=827.528337; rt=5.2902)
T/S Cluster: L−5.3−4
L762 (m/z=808.56174; rt=5.33113)
T/S Cluster: L−5.3−5
L760 (m/z=809.56496; rt=5.33041)
T/S Cluster: L−5.3−5
L761 (m/z=809.543519; rt=5.33075)
T/S Cluster: L−5.3−5
PCo1
PCo2
−0.6
−0.4
−0.2
0.0
0.2
0.4

L764 (m/z=813.517583; rt=5.33304)
T/S Cluster: L−5.3−5

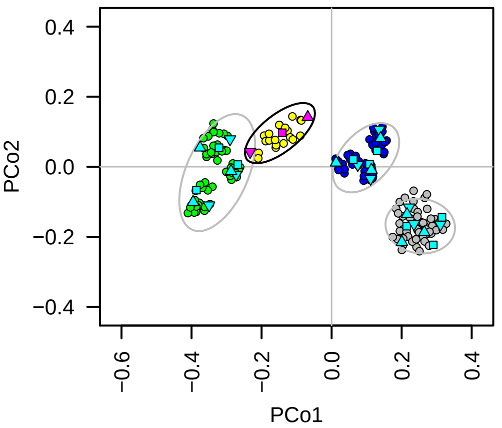

L765 (m/z=813.512556; rt=5.33335)
T/S Cluster: L−5.3−5

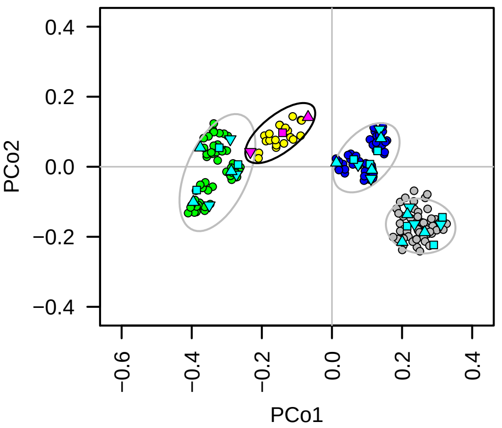

L766 (m/z=813.507899; rt=5.33343)
T/S Cluster: L−5.3−5

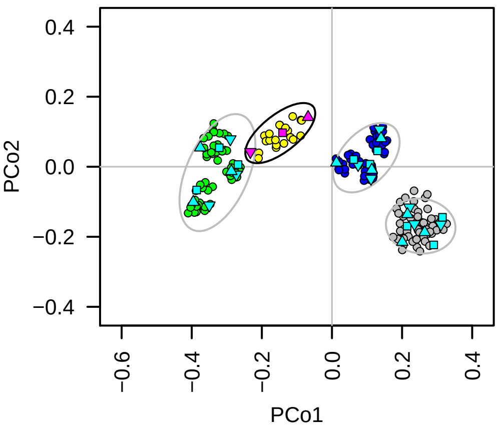

L763 (m/z=814.515083; rt=5.33139)
T/S Cluster: L−5.3−5

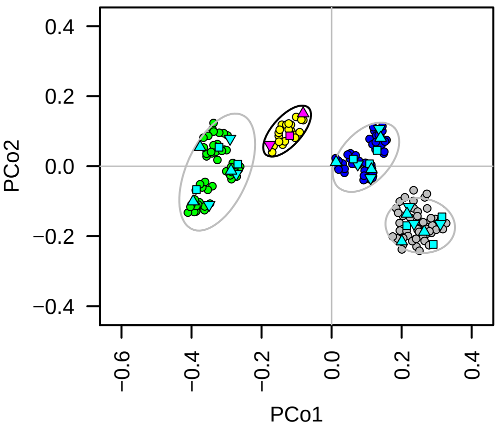

L752 (m/z=808.510759; rt=5.3291)
T/S Cluster: L−5.3−5

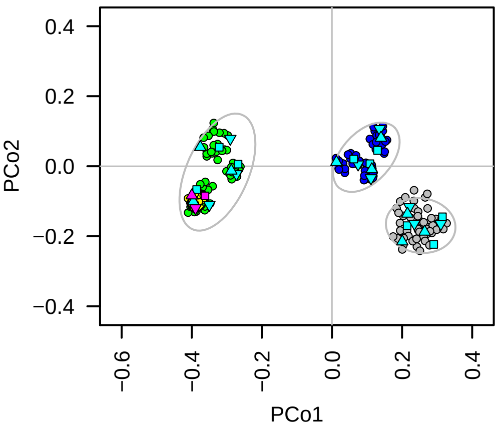

L744 (m/z=970.616092; rt=5.30917)
T/S Cluster: L−5.3−5

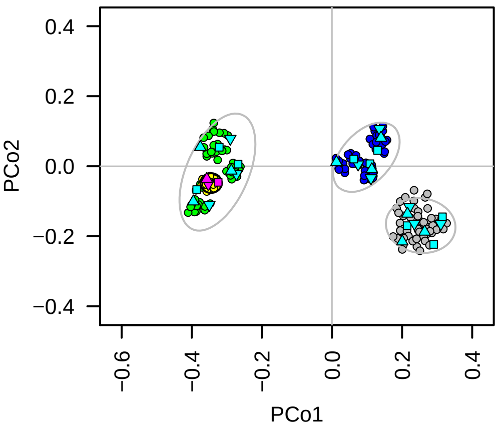

L758 (m/z=810.57049; rt=5.33026)
T/S Cluster: L−5.3−5

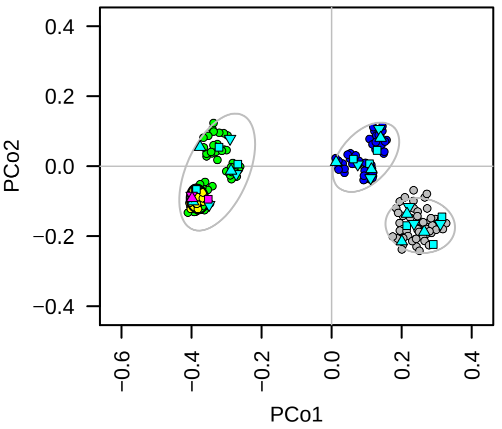

L754 (m/z=404.281494; rt=5.3301)
T/S Cluster: L−5.3−5

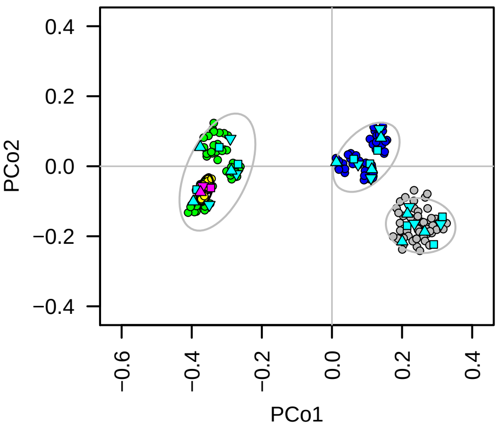

L755 (m/z=808.612885; rt=5.33015)
T/S Cluster: L−5.3−5

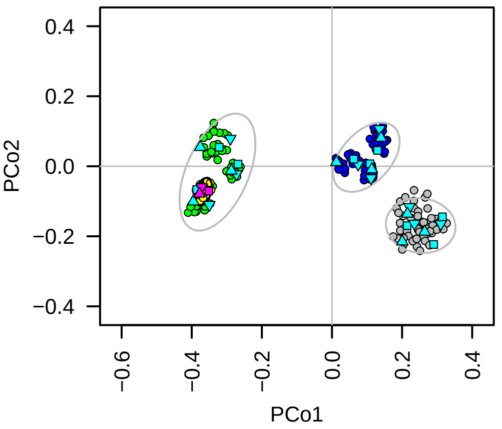

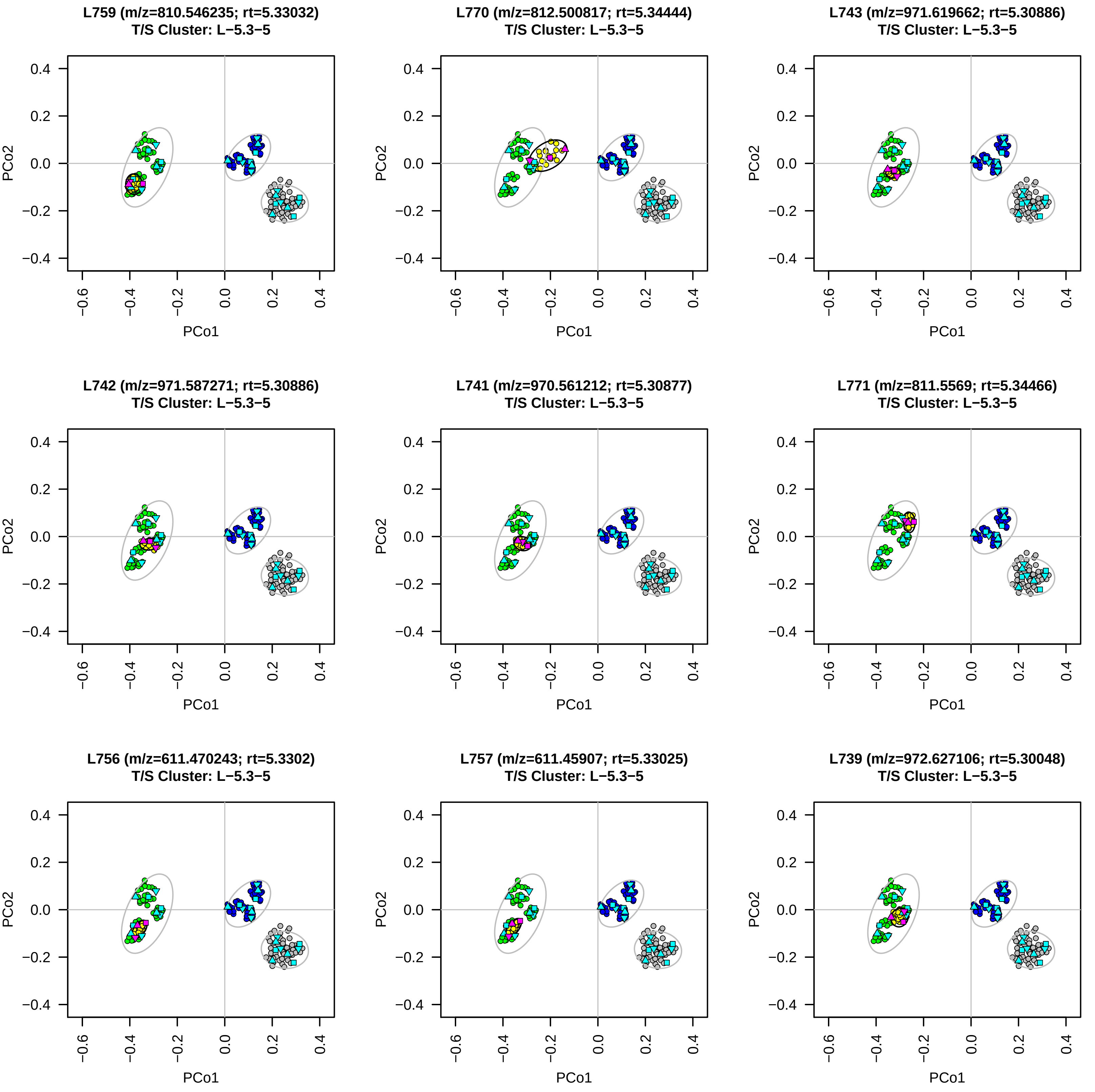
L759 (m/z=810.546235; rt=5.33032)
T/S Cluster: L−5.3−5
L770 (m/z=812.500817; rt=5.34444)
T/S Cluster: L−5.3−5
L743 (m/z=971.619662; rt=5.30886)
T/S Cluster: L−5.3−5
L742 (m/z=971.587271; rt=5.30886)
T/S Cluster: L−5.3−5
L741 (m/z=970.561212; rt=5.30877)
T/S Cluster: L−5.3−5
L771 (m/z=811.5569; rt=5.34466)
T/S Cluster: L−5.3−5
L756 (m/z=611.470243; rt=5.3302)
T/S Cluster: L−5.3−5
L757 (m/z=611.45907; rt=5.33025)
T/S Cluster: L−5.3−5
L739 (m/z=972.627106; rt=5.30048)
T/S Cluster: L−5.3−5
PCo1
PCo2
0.4
0.2
0.0
−0.2
−0.4
−0.6

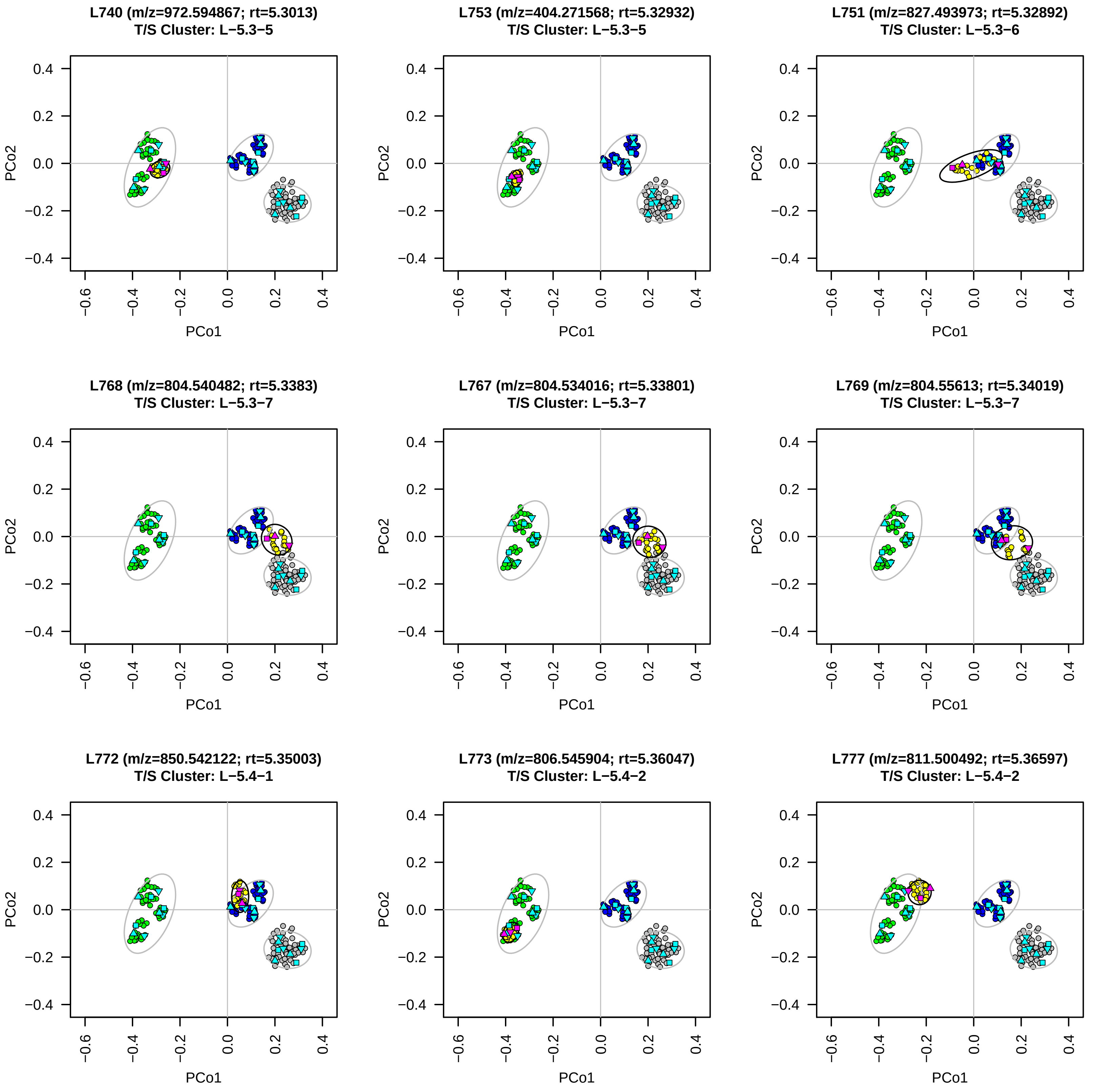
L740 (m/z=972.594867; rt=5.3013)
T/S Cluster: L−5.3−5
L753 (m/z=404.271568; rt=5.32932)
T/S Cluster: L−5.3−5
L751 (m/z=827.493973; rt=5.32892)
T/S Cluster: L−5.3−6
L768 (m/z=804.540482; rt=5.3383)
T/S Cluster: L−5.3−7
L767 (m/z=804.534016; rt=5.33801)
T/S Cluster: L−5.3−7
L769 (m/z=804.55613; rt=5.34019)
T/S Cluster: L−5.3−7
L772 (m/z=850.542122; rt=5.35003)
T/S Cluster: L−5.4−1
L773 (m/z=806.545904; rt=5.36047)
T/S Cluster: L−5.4−2
L777 (m/z=811.500492; rt=5.36597)
T/S Cluster: L−5.4−2
PCo1
PCo2
0.4
0.2
0.0
−0.2
−0.4
−0.6

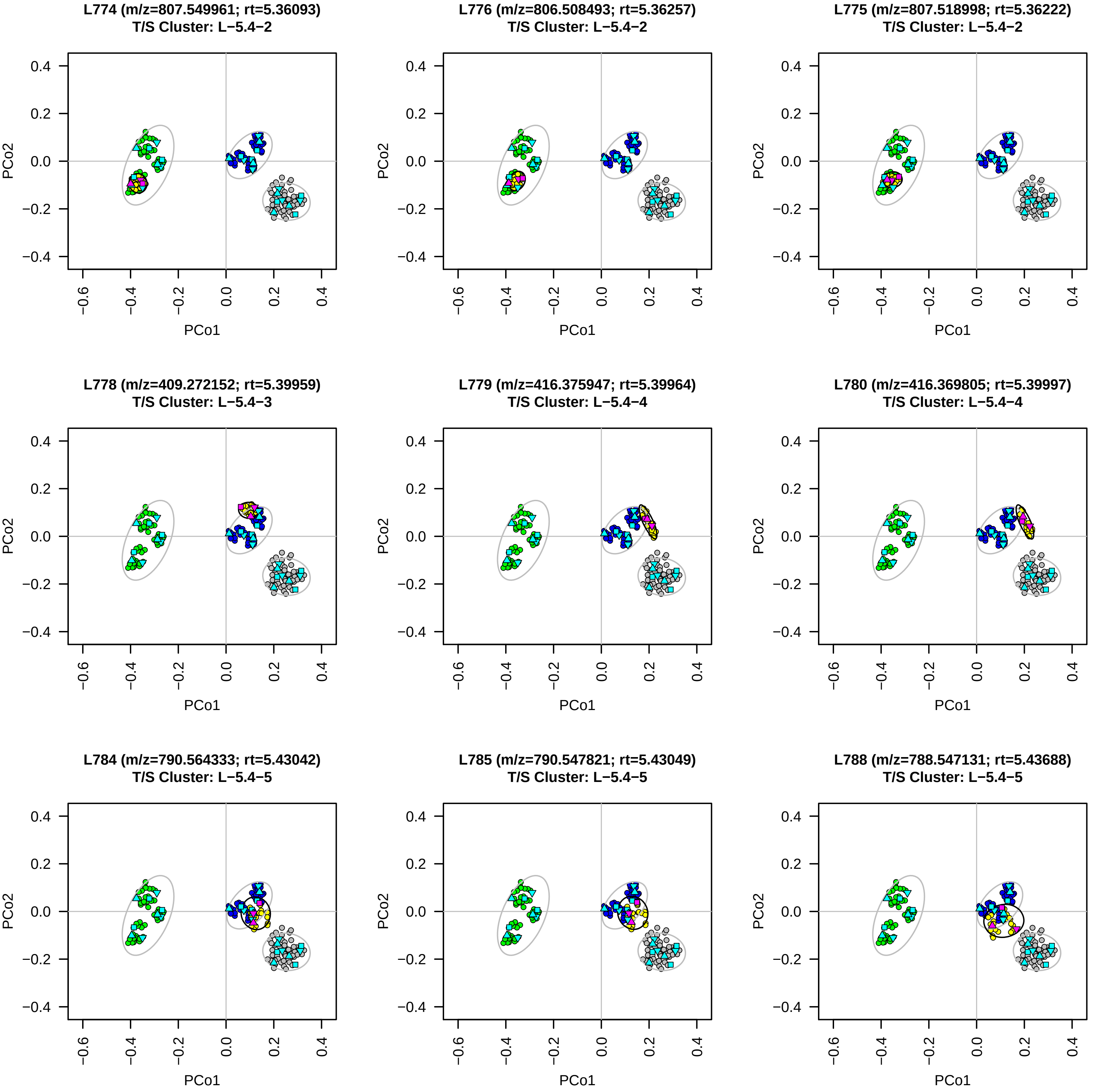
L774 (m/z=807.549961; rt=5.36093)
T/S Cluster: L−5.4−2
L776 (m/z=806.508493; rt=5.36257)
T/S Cluster: L−5.4−2
L775 (m/z=807.518998; rt=5.36222)
T/S Cluster: L−5.4−2
L778 (m/z=409.272152; rt=5.39959)
T/S Cluster: L−5.4−3
L779 (m/z=416.375947; rt=5.39964)
T/S Cluster: L−5.4−4
L780 (m/z=416.369805; rt=5.39997)
T/S Cluster: L−5.4−4
L784 (m/z=790.564333; rt=5.43042)
T/S Cluster: L−5.4−5
L785 (m/z=790.547821; rt=5.43049)
T/S Cluster: L−5.4−5
L788 (m/z=788.547131; rt=5.43688)
T/S Cluster: L−5.4−5
PCo1
PCo2
0.4
0.2
0.0
−0.2
−0.4
−0.6

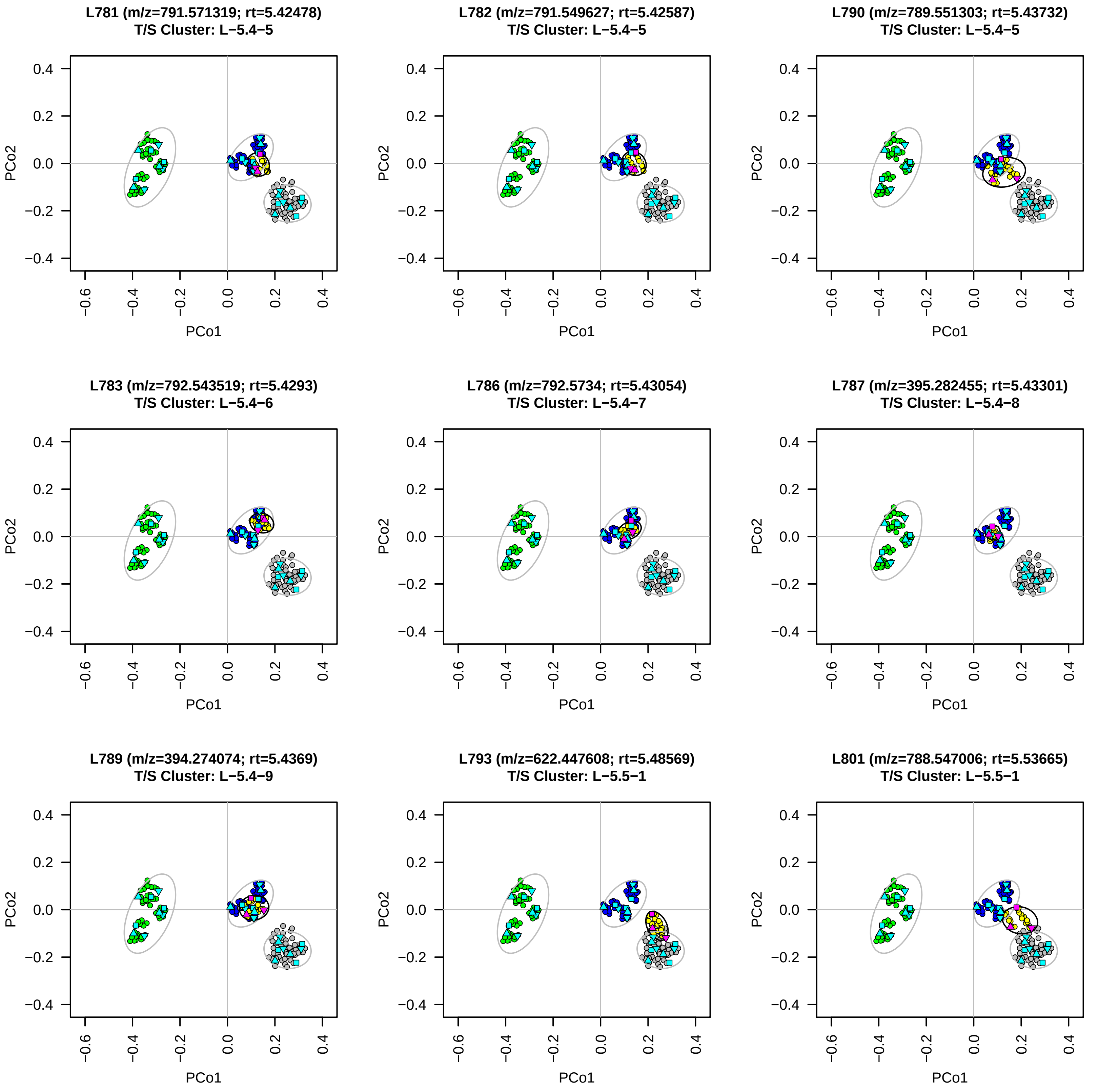
L781 (m/z=791.571319; rt=5.42478)
T/S Cluster: L−5.4−5
L782 (m/z=791.549627; rt=5.42587)
T/S Cluster: L−5.4−5
L790 (m/z=789.551303; rt=5.43732)
T/S Cluster: L−5.4−5
L783 (m/z=792.543519; rt=5.4293)
T/S Cluster: L−5.4−6
L786 (m/z=792.5734; rt=5.43054)
T/S Cluster: L−5.4−7
L787 (m/z=395.282455; rt=5.43301)
T/S Cluster: L−5.4−8
L789 (m/z=394.274074; rt=5.4369)
T/S Cluster: L−5.4−9
L793 (m/z=622.447608; rt=5.48569)
T/S Cluster: L−5.5−1
L801 (m/z=788.547006; rt=5.53665)
T/S Cluster: L−5.5−1
PCo1
PCo2
−0.6
−0.4
−0.2
0.0
0.2
0.4

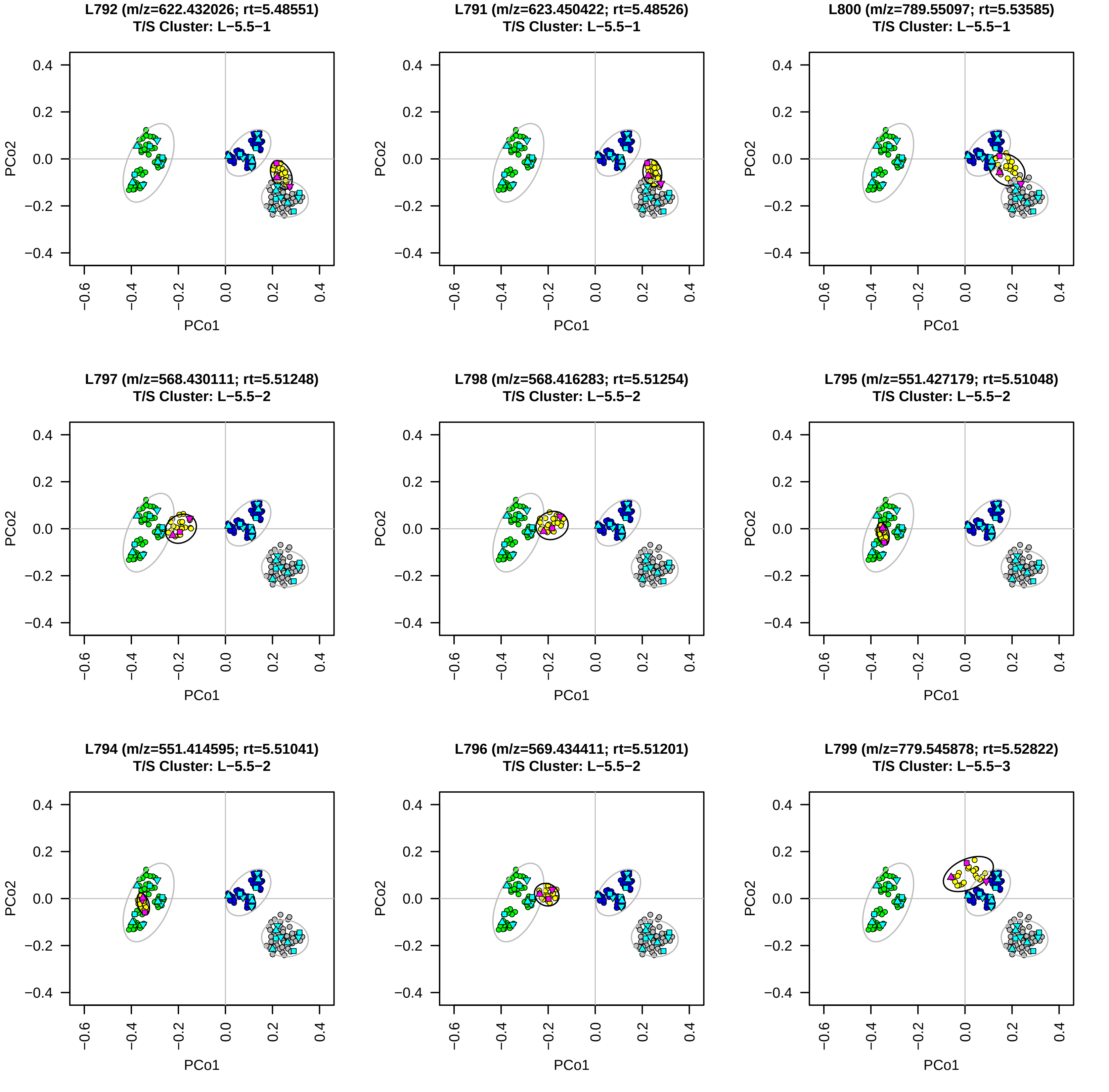
L792 (m/z=622.432026; rt=5.48551)
T/S Cluster: L−5.5−1
L791 (m/z=623.450422; rt=5.48526)
T/S Cluster: L−5.5−1
L800 (m/z=789.55097; rt=5.53585)
T/S Cluster: L−5.5−1
L797 (m/z=568.430111; rt=5.51248)
T/S Cluster: L−5.5−2
L798 (m/z=568.416283; rt=5.51254)
T/S Cluster: L−5.5−2
L795 (m/z=551.427179; rt=5.51048)
T/S Cluster: L−5.5−2
L794 (m/z=551.414595; rt=5.51041)
T/S Cluster: L−5.5−2
L796 (m/z=569.434411; rt=5.51201)
T/S Cluster: L−5.5−2
L799 (m/z=779.545878; rt=5.52822)
T/S Cluster: L−5.5−3
PCo1
PCo2

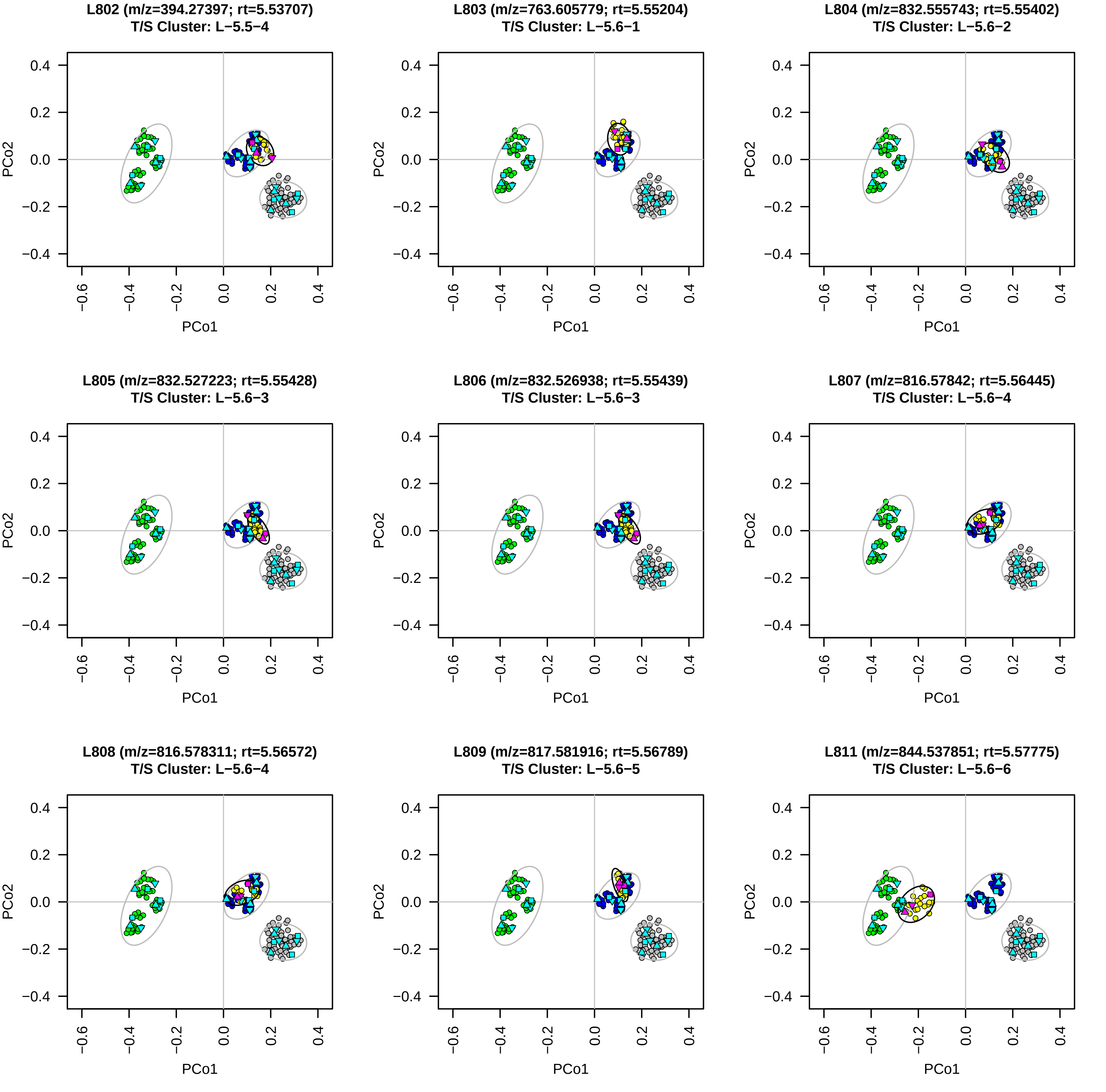
L802 (m/z=394.27397; rt=5.53707)
T/S Cluster: L−5.5−4
L803 (m/z=763.605779; rt=5.55204)
T/S Cluster: L−5.6−1
L804 (m/z=832.555743; rt=5.55402)
T/S Cluster: L−5.6−2
L805 (m/z=832.527223; rt=5.55428)
T/S Cluster: L−5.6−3
L806 (m/z=832.526938; rt=5.55439)
T/S Cluster: L−5.6−3
L807 (m/z=816.57842; rt=5.56445)
T/S Cluster: L−5.6−4
L808 (m/z=816.578311; rt=5.56572)
T/S Cluster: L−5.6−4
L809 (m/z=817.581916; rt=5.56789)
T/S Cluster: L−5.6−5
L811 (m/z=844.537851; rt=5.57775)
T/S Cluster: L−5.6−6
PCo1
PCo2

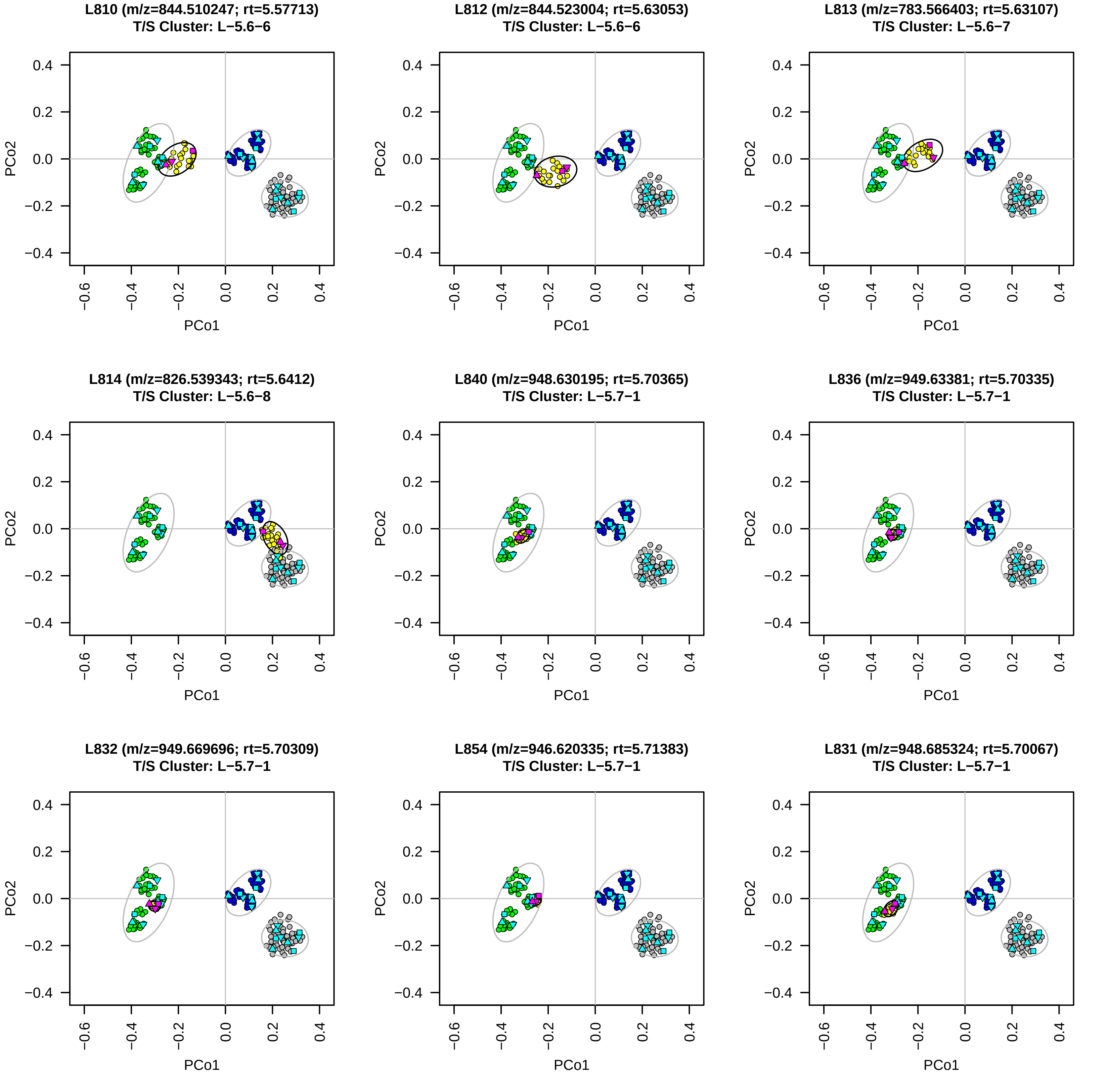
L810 (m/z=844.510247; rt=5.57713)
T/S Cluster: L−5.6−6
L812 (m/z=844.523004; rt=5.63053)
T/S Cluster: L−5.6−6
L813 (m/z=783.566403; rt=5.63107)
T/S Cluster: L−5.6−7
L814 (m/z=826.539343; rt=5.6412)
T/S Cluster: L−5.6−8
L840 (m/z=948.630195; rt=5.70365)
T/S Cluster: L−5.7−1
L836 (m/z=949.63381; rt=5.70335)
T/S Cluster: L−5.7−1
L832 (m/z=949.669696; rt=5.70309)
T/S Cluster: L−5.7−1
L854 (m/z=946.620335; rt=5.71383)
T/S Cluster: L−5.7−1
L831 (m/z=948.685324; rt=5.70067)
T/S Cluster: L−5.7−1
PCo1
PCo2

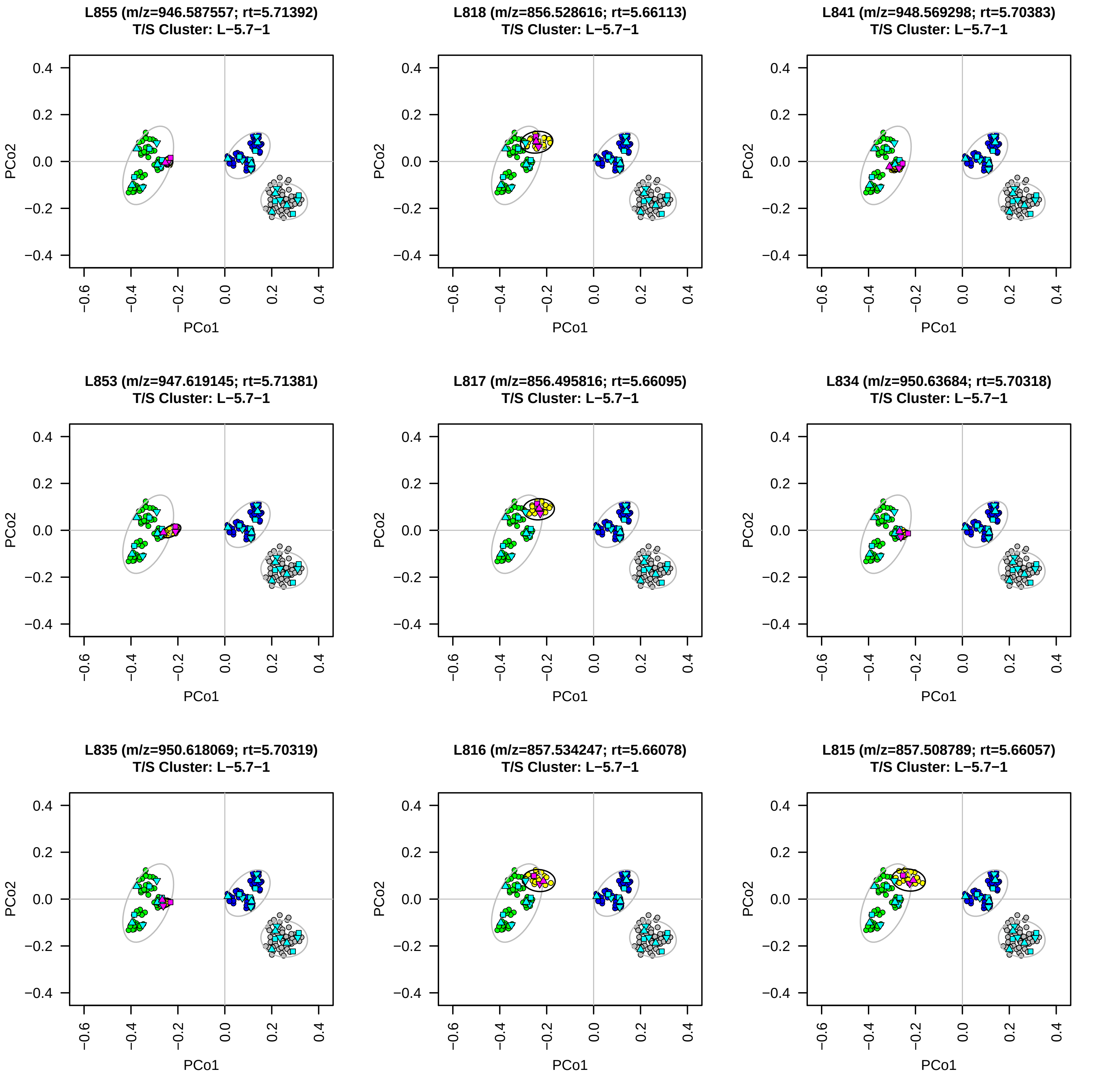
L855 (m/z=946.587557; rt=5.71392)
T/S Cluster: L−5.7−1
L818 (m/z=856.528616; rt=5.66113)
T/S Cluster: L−5.7−1
L841 (m/z=948.569298; rt=5.70383)
T/S Cluster: L−5.7−1
L853 (m/z=947.619145; rt=5.71381)
T/S Cluster: L−5.7−1
L817 (m/z=856.495816; rt=5.66095)
T/S Cluster: L−5.7−1
L834 (m/z=950.63684; rt=5.70318)
T/S Cluster: L−5.7−1
L835 (m/z=950.618069; rt=5.70319)
T/S Cluster: L−5.7−1
L816 (m/z=857.534247; rt=5.66078)
T/S Cluster: L−5.7−1
L815 (m/z=857.508789; rt=5.66057)
T/S Cluster: L−5.7−1
PCo1
PCo2
−0.6
−0.4
−0.2
0.0
0.2
0.4

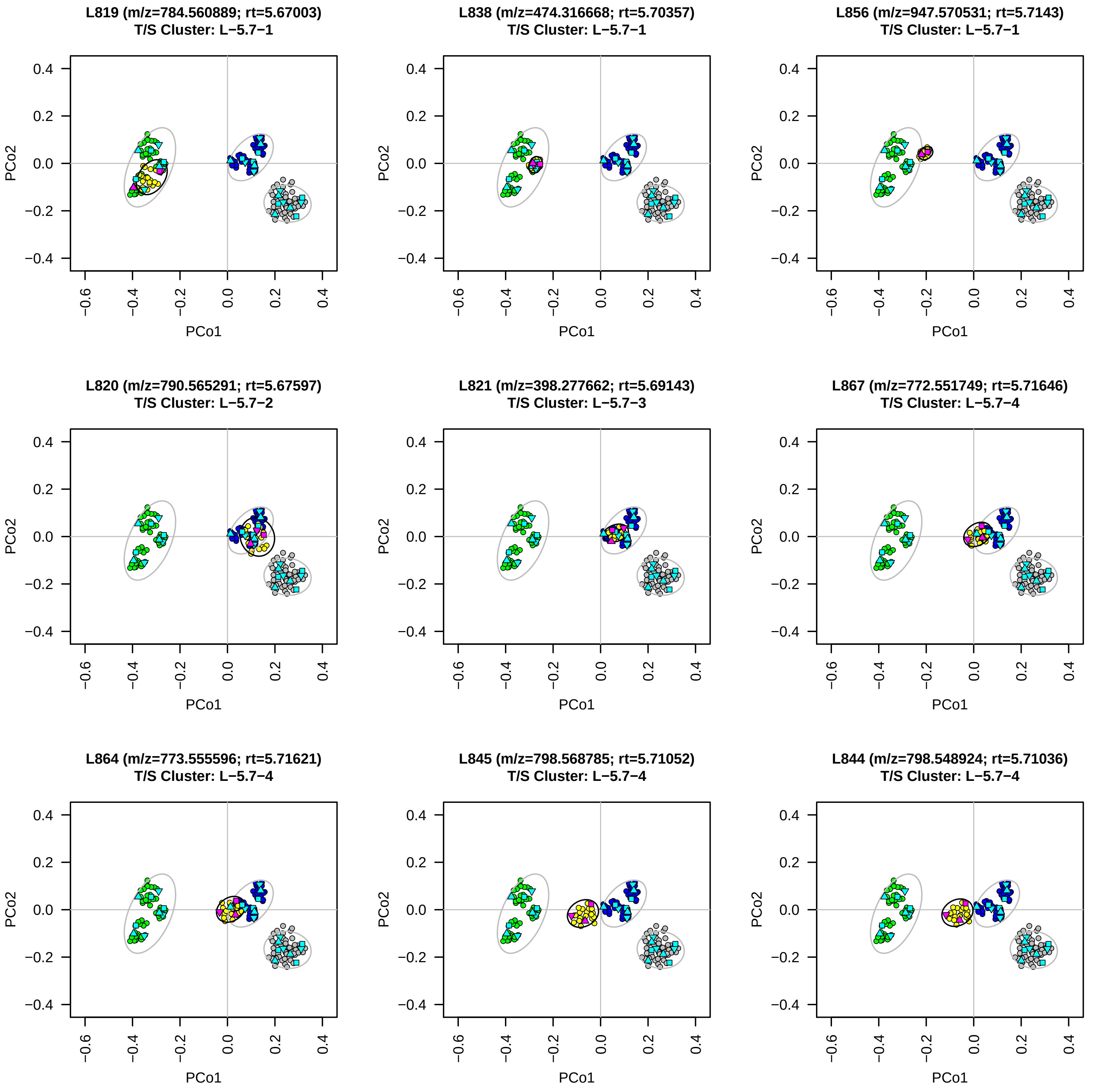
L819 (m/z=784.560889; rt=5.67003)
T/S Cluster: L−5.7−1
L838 (m/z=474.316668; rt=5.70357)
T/S Cluster: L−5.7−1
L856 (m/z=947.570531; rt=5.7143)
T/S Cluster: L−5.7−1
L820 (m/z=790.565291; rt=5.67597)
T/S Cluster: L−5.7−2
L821 (m/z=398.277662; rt=5.69143)
T/S Cluster: L−5.7−3
L867 (m/z=772.551749; rt=5.71646)
T/S Cluster: L−5.7−4
L864 (m/z=773.555596; rt=5.71621)
T/S Cluster: L−5.7−4
L845 (m/z=798.568785; rt=5.71052)
T/S Cluster: L−5.7−4
L844 (m/z=798.548924; rt=5.71036)
T/S Cluster: L−5.7−4
PCo1
PCo2
0.4
0.2
0.0
−0.2
−0.4
−0.6

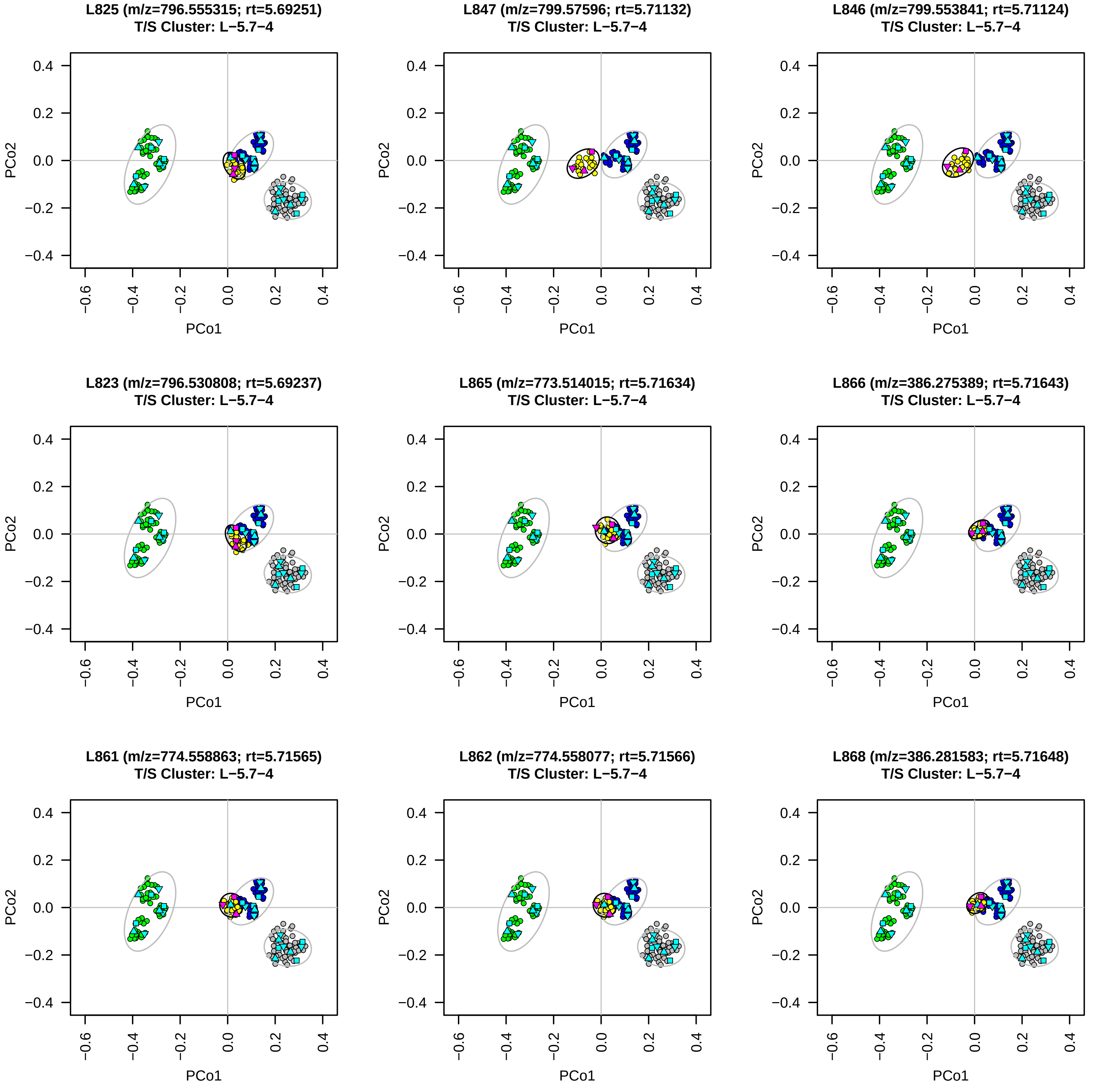

L825 (m/z=796.555315; rt=5.69251)
T/S Cluster: L−5.7−4
L847 (m/z=799.57596; rt=5.71132)
T/S Cluster: L−5.7−4
L846 (m/z=799.553841; rt=5.71124)
T/S Cluster: L−5.7−4
L823 (m/z=796.530808; rt=5.69237)
T/S Cluster: L−5.7−4
L865 (m/z=773.514015; rt=5.71634)
T/S Cluster: L−5.7−4
L866 (m/z=386.275389; rt=5.71643)
T/S Cluster: L−5.7−4
L861 (m/z=774.558863; rt=5.71565)
T/S Cluster: L−5.7−4
L862 (m/z=774.558077; rt=5.71566)
T/S Cluster: L−5.7−4
L868 (m/z=386.281583; rt=5.71648)
T/S Cluster: L−5.7−4
PCo1
PCo2

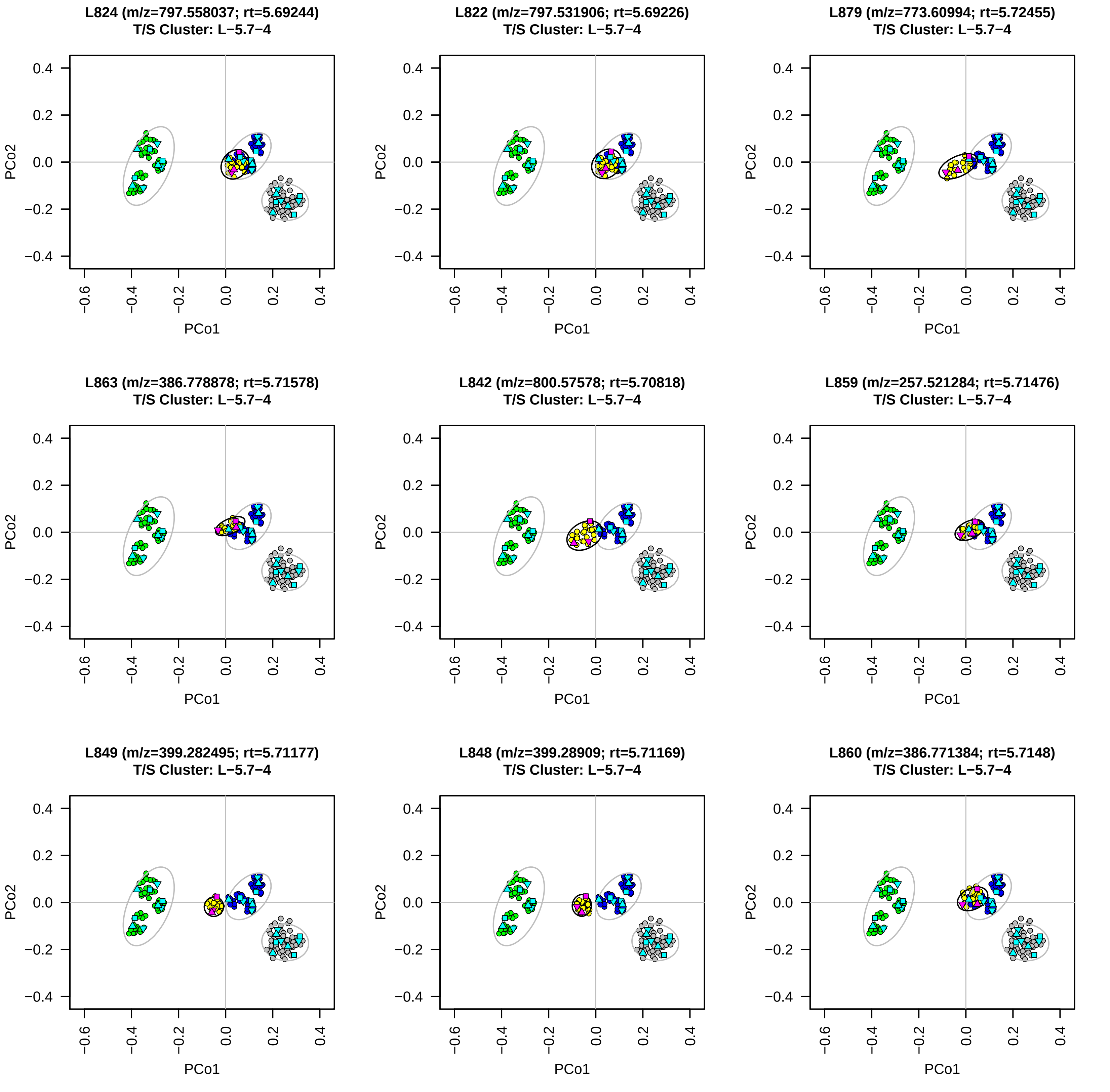
L824 (m/z=797.558037; rt=5.69244)
T/S Cluster: L−5.7−4
L822 (m/z=797.531906; rt=5.69226)
T/S Cluster: L−5.7−4
L879 (m/z=773.60994; rt=5.72455)
T/S Cluster: L−5.7−4
L863 (m/z=386.778878; rt=5.71578)
T/S Cluster: L−5.7−4
L842 (m/z=800.57578; rt=5.70818)
T/S Cluster: L−5.7−4
L859 (m/z=257.521284; rt=5.71476)
T/S Cluster: L−5.7−4
L849 (m/z=399.282495; rt=5.71177)
T/S Cluster: L−5.7−4
L848 (m/z=399.28909; rt=5.71169)
T/S Cluster: L−5.7−4
L860 (m/z=386.771384; rt=5.7148)
T/S Cluster: L−5.7−4
PCo1
PCo2

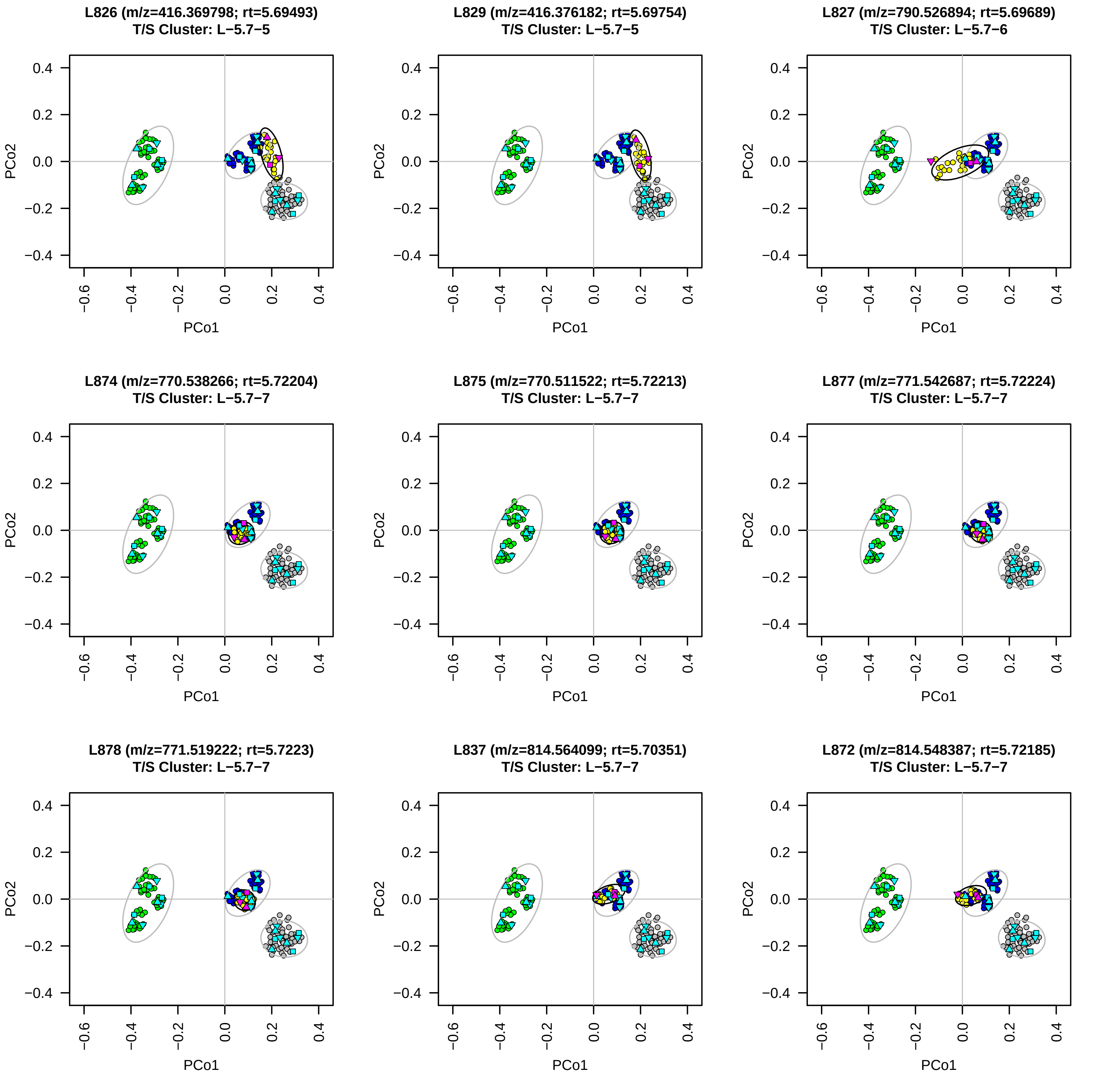
L826 (m/z=416.369798; rt=5.69493)
T/S Cluster: L−5.7−5
L829 (m/z=416.376182; rt=5.69754)
T/S Cluster: L−5.7−5
L827 (m/z=790.526894; rt=5.69689)
T/S Cluster: L−5.7−6
L874 (m/z=770.538266; rt=5.72204)
T/S Cluster: L−5.7−7
L875 (m/z=770.511522; rt=5.72213)
T/S Cluster: L−5.7−7
L877 (m/z=771.542687; rt=5.72224)
T/S Cluster: L−5.7−7
L878 (m/z=771.519222; rt=5.7223)
T/S Cluster: L−5.7−7
L837 (m/z=814.564099; rt=5.70351)
T/S Cluster: L−5.7−7
L872 (m/z=814.548387; rt=5.72185)
T/S Cluster: L−5.7−7
PCo1
PCo2
−0.6
−0.4
−0.2
0.0
0.2
0.4

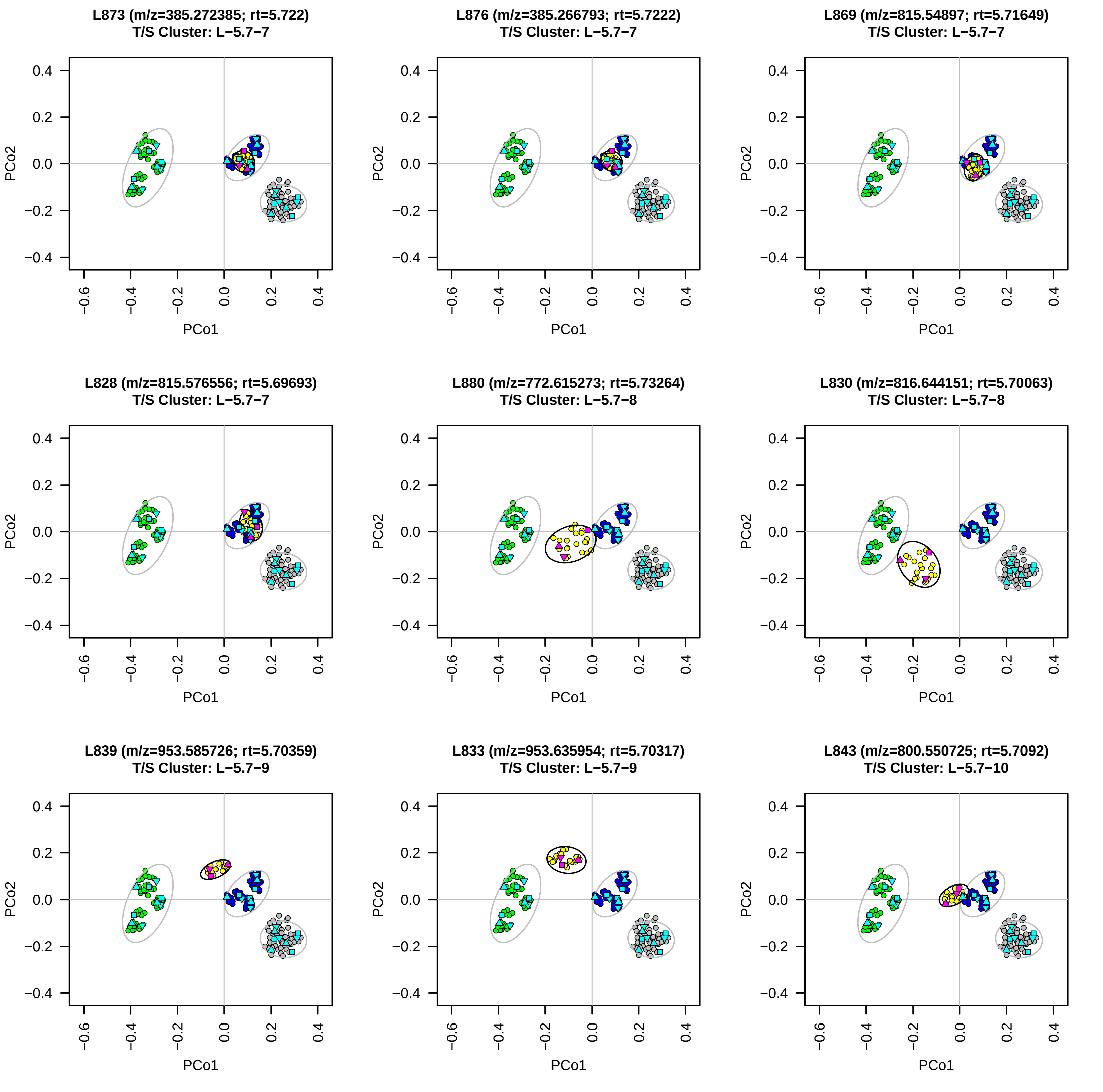
L873 (m/z=385.272385; rt=5.722)
T/S Cluster: L−5.7−7
L876 (m/z=385.266793; rt=5.7222)
T/S Cluster: L−5.7−7
L869 (m/z=815.54897; rt=5.71649)
T/S Cluster: L−5.7−7
L828 (m/z=815.576556; rt=5.69693)
T/S Cluster: L−5.7−7
L880 (m/z=772.615273; rt=5.73264)
T/S Cluster: L−5.7−8
L830 (m/z=816.644151; rt=5.70063)
T/S Cluster: L−5.7−8
L839 (m/z=953.585726; rt=5.70359)
T/S Cluster: L−5.7−9
L833 (m/z=953.635954; rt=5.70317)
T/S Cluster: L−5.7−9
L843 (m/z=800.550725; rt=5.7092)
T/S Cluster: L−5.7−10
PCo1
PCo2
−0.6
−0.4
−0.2
0.0
0.2
0.4

L850 (m/z=388.34341; rt=5.71303)
T/S Cluster: L−5.7−11

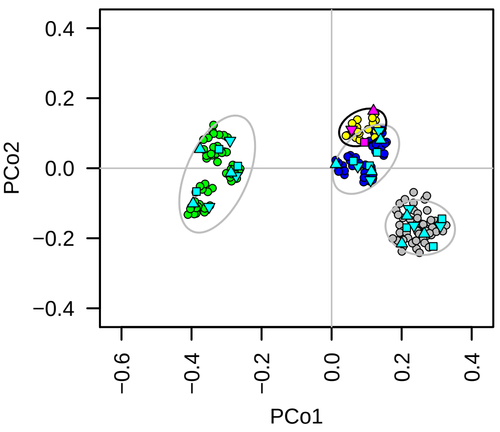

L852 (m/z=388.335179; rt=5.7135)
T/S Cluster: L−5.7−11

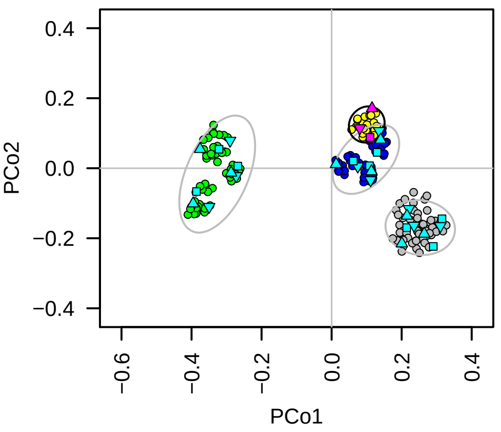

L851 (m/z=389.34674; rt=5.71319)
T/S Cluster: L−5.7−11

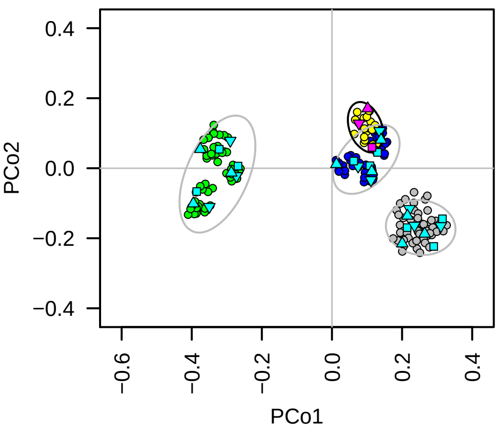

L857 (m/z=371.317694; rt=5.71444)
T/S Cluster: L−5.7−12

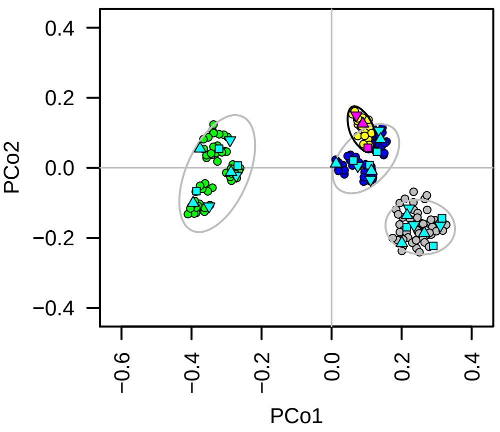

L858 (m/z=371.312064; rt=5.71453)
T/S Cluster: L−5.7−12

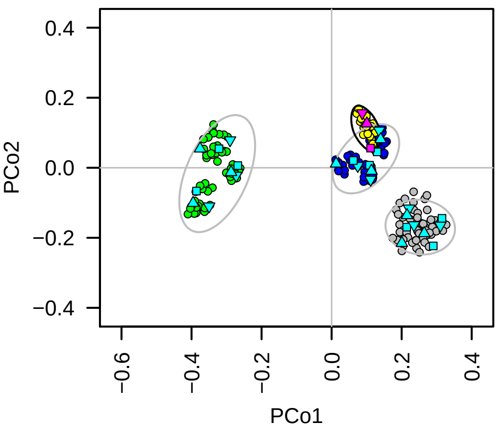

L870 (m/z=393.299471; rt=5.71804)
T/S Cluster: L−5.7−13

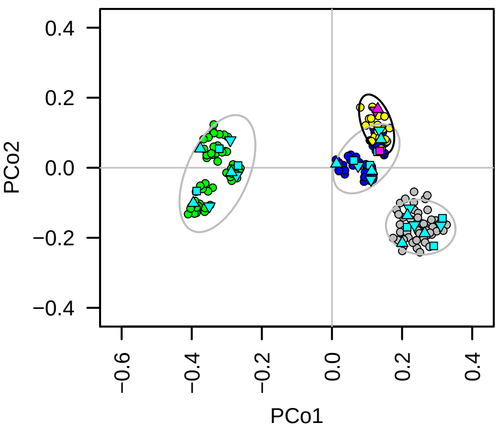

L871 (m/z=393.29258; rt=5.71864)
T/S Cluster: L−5.7−13

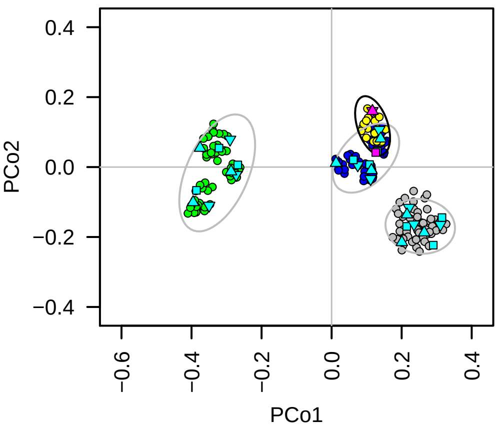

L881 (m/z=789.5178; rt=5.73359)
T/S Cluster: L−5.7−14

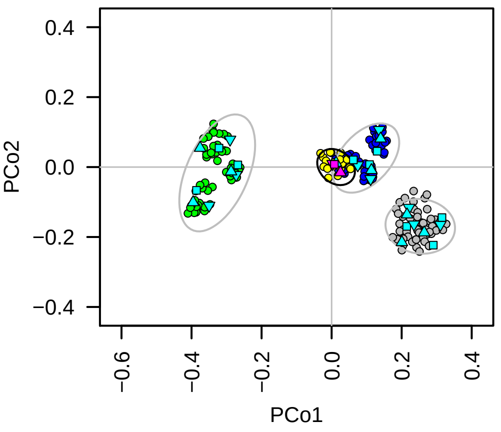

L882 (m/z=789.531233; rt=5.74776)
T/S Cluster: L−5.7−15

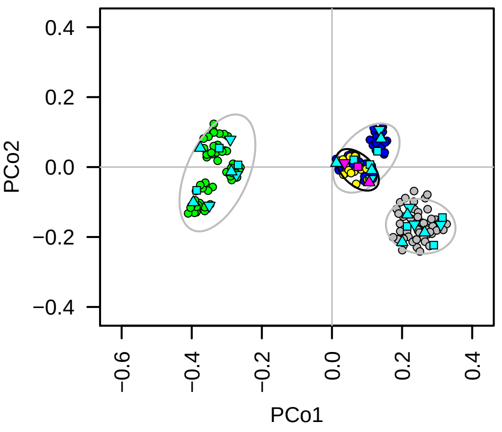

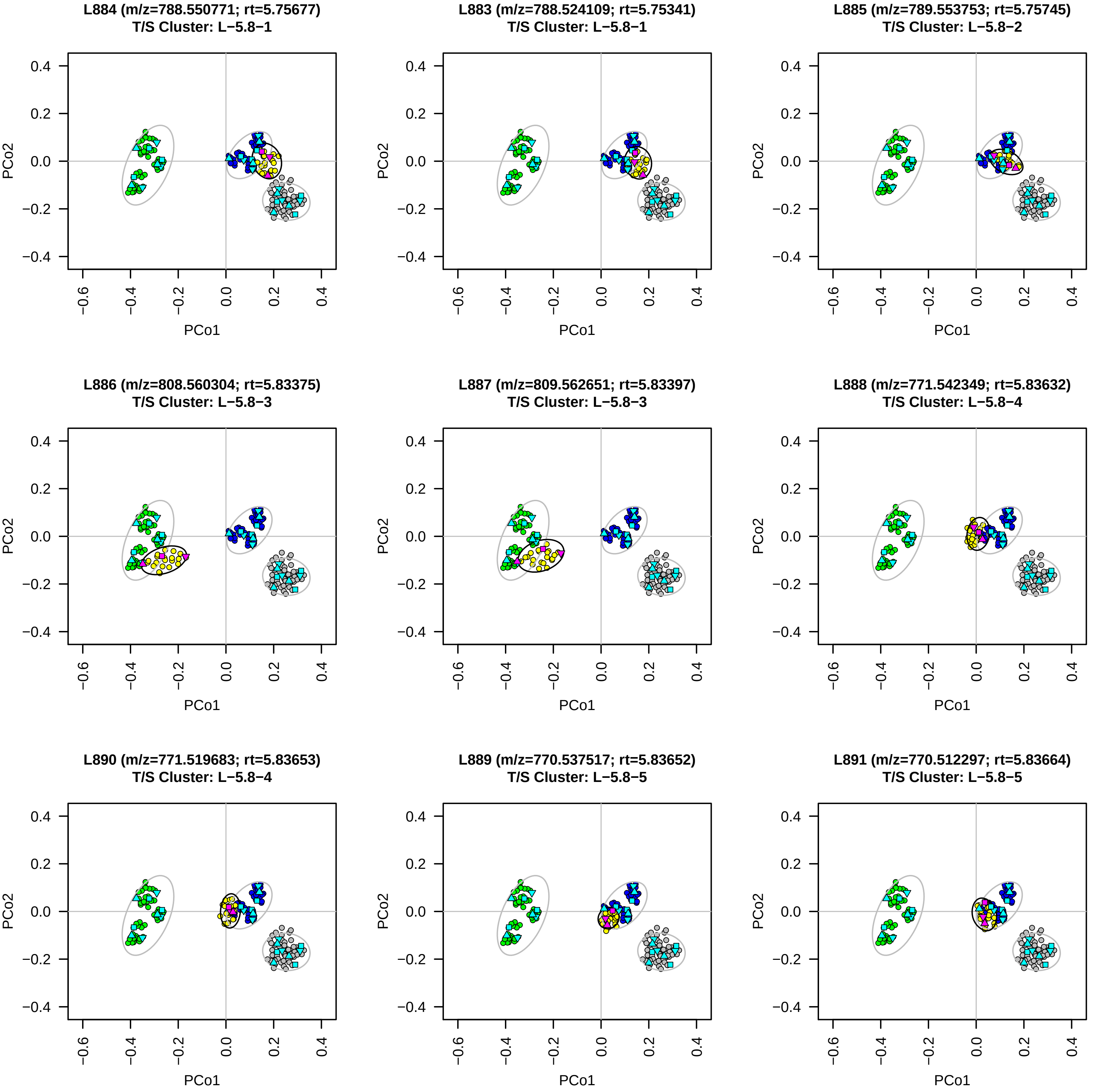
L884 (m/z=788.550771; rt=5.75677)
T/S Cluster: L−5.8−1
L883 (m/z=788.524109; rt=5.75341)
T/S Cluster: L−5.8−1
L885 (m/z=789.553753; rt=5.75745)
T/S Cluster: L−5.8−2
L886 (m/z=808.560304; rt=5.83375)
T/S Cluster: L−5.8−3
L887 (m/z=809.562651; rt=5.83397)
T/S Cluster: L−5.8−3
L888 (m/z=771.542349; rt=5.83632)
T/S Cluster: L−5.8−4
L890 (m/z=771.519683; rt=5.83653)
T/S Cluster: L−5.8−4
L889 (m/z=770.537517; rt=5.83652)
T/S Cluster: L−5.8−5
L891 (m/z=770.512297; rt=5.83664)
T/S Cluster: L−5.8−5
PCo1
PCo2
−0.6
−0.4
−0.2
0.0
0.2
0.4

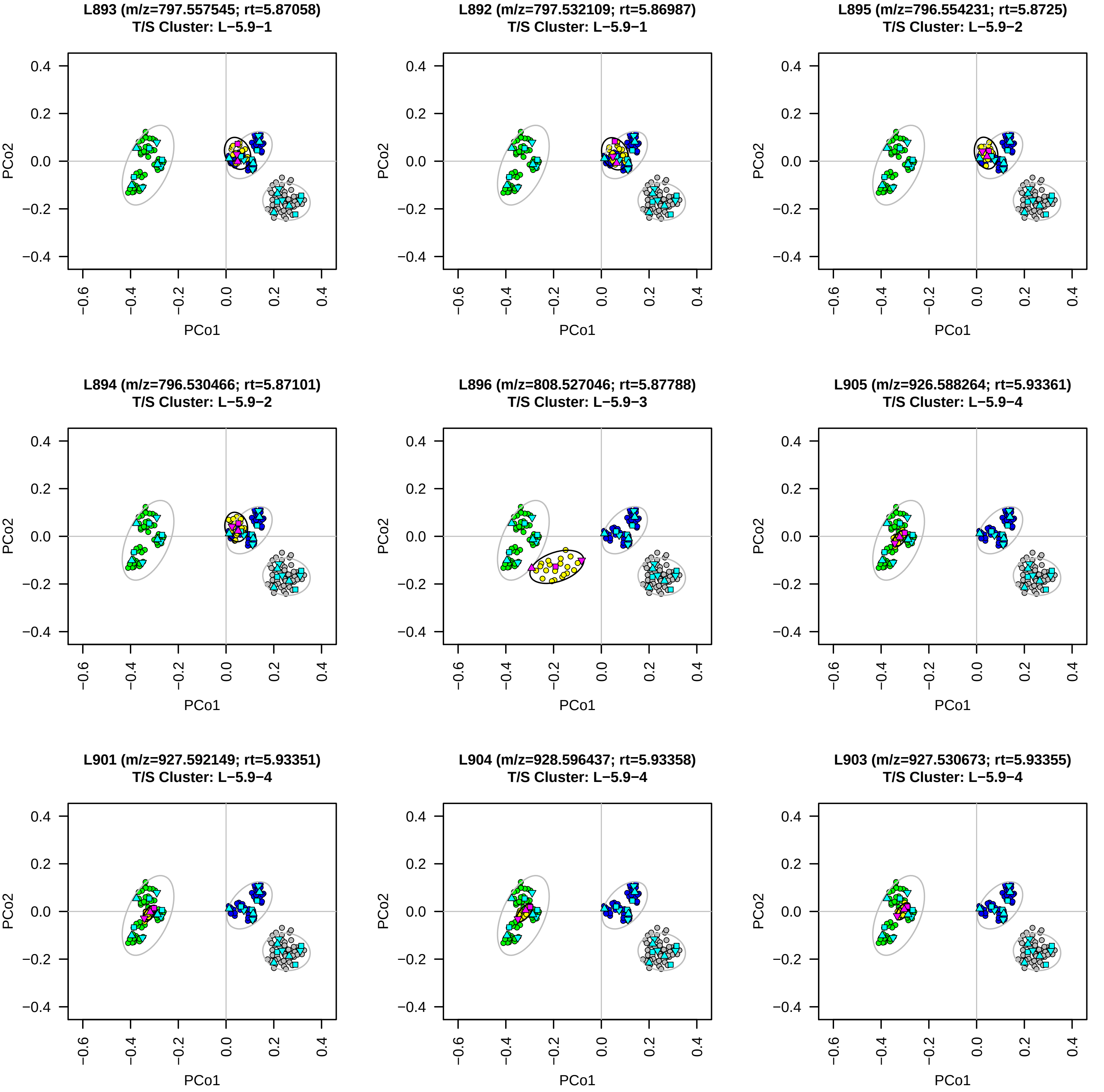
L893 (m/z=797.557545; rt=5.87058)
T/S Cluster: L−5.9−1
L892 (m/z=797.532109; rt=5.86987)
T/S Cluster: L−5.9−1
L895 (m/z=796.554231; rt=5.8725)
T/S Cluster: L−5.9−2
L894 (m/z=796.530466; rt=5.87101)
T/S Cluster: L−5.9−2
L896 (m/z=808.527046; rt=5.87788)
T/S Cluster: L−5.9−3
L905 (m/z=926.588264; rt=5.93361)
T/S Cluster: L−5.9−4
L901 (m/z=927.592149; rt=5.93351)
T/S Cluster: L−5.9−4
L904 (m/z=928.596437; rt=5.93358)
T/S Cluster: L−5.9−4
L903 (m/z=927.530673; rt=5.93355)
T/S Cluster: L−5.9−4
PCo1
PCo2

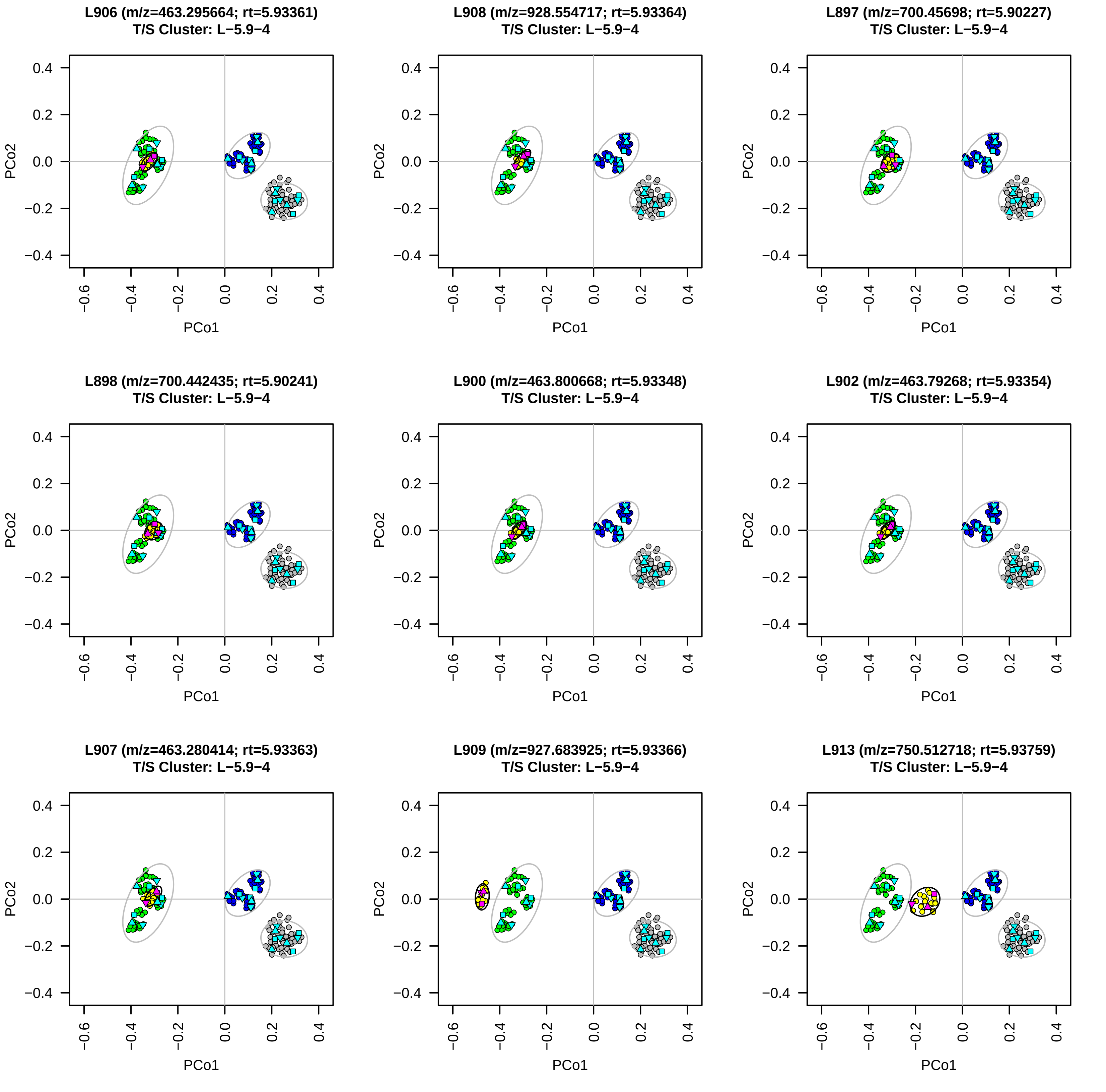
L906 (m/z=463.295664; rt=5.93361)
T/S Cluster: L−5.9−4
L908 (m/z=928.554717; rt=5.93364)
T/S Cluster: L−5.9−4
L897 (m/z=700.45698; rt=5.90227)
T/S Cluster: L−5.9−4
L898 (m/z=700.442435; rt=5.90241)
T/S Cluster: L−5.9−4
L900 (m/z=463.800668; rt=5.93348)
T/S Cluster: L−5.9−4
L902 (m/z=463.79268; rt=5.93354)
T/S Cluster: L−5.9−4
L907 (m/z=463.280414; rt=5.93363)
T/S Cluster: L−5.9−4
L909 (m/z=927.683925; rt=5.93366)
T/S Cluster: L−5.9−4
L913 (m/z=750.512718; rt=5.93759)
T/S Cluster: L−5.9−4
PCo1
PCo2

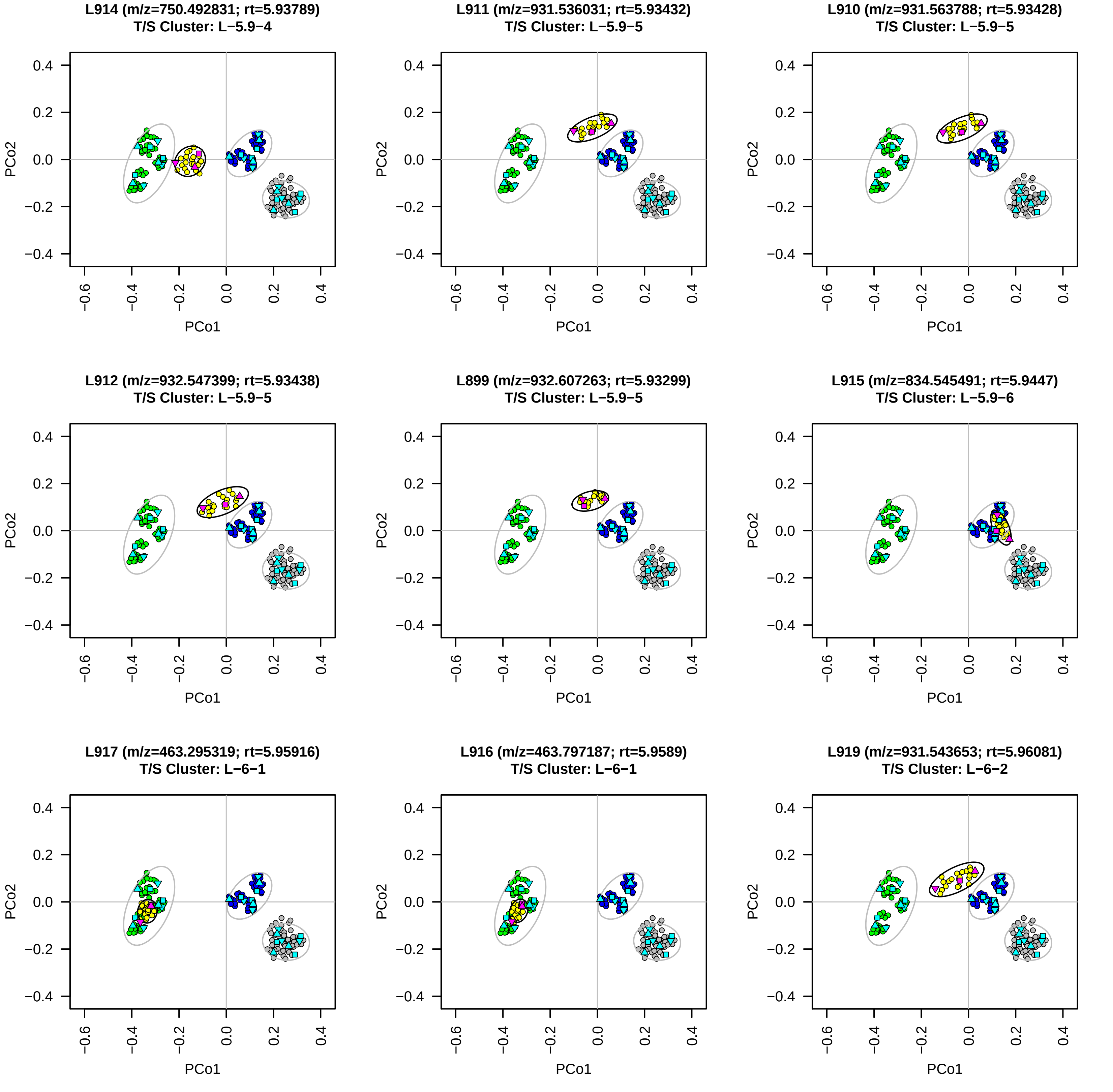

L914 (m/z=750.492831; rt=5.93789)
T/S Cluster: L−5.9−4
L911 (m/z=931.536031; rt=5.93432)
T/S Cluster: L−5.9−5
L910 (m/z=931.563788; rt=5.93428)
T/S Cluster: L−5.9−5
L912 (m/z=932.547399; rt=5.93438)
T/S Cluster: L−5.9−5
L899 (m/z=932.607263; rt=5.93299)
T/S Cluster: L−5.9−5
L915 (m/z=834.545491; rt=5.9447)
T/S Cluster: L−5.9−6
L917 (m/z=463.295319; rt=5.95916)
T/S Cluster: L−6−1
L916 (m/z=463.797187; rt=5.9589)
T/S Cluster: L−6−1
L919 (m/z=931.543653; rt=5.96081)
T/S Cluster: L−6−2
PCo1
PCo2
−0.6
−0.4
−0.2
0.0
0.2
0.4

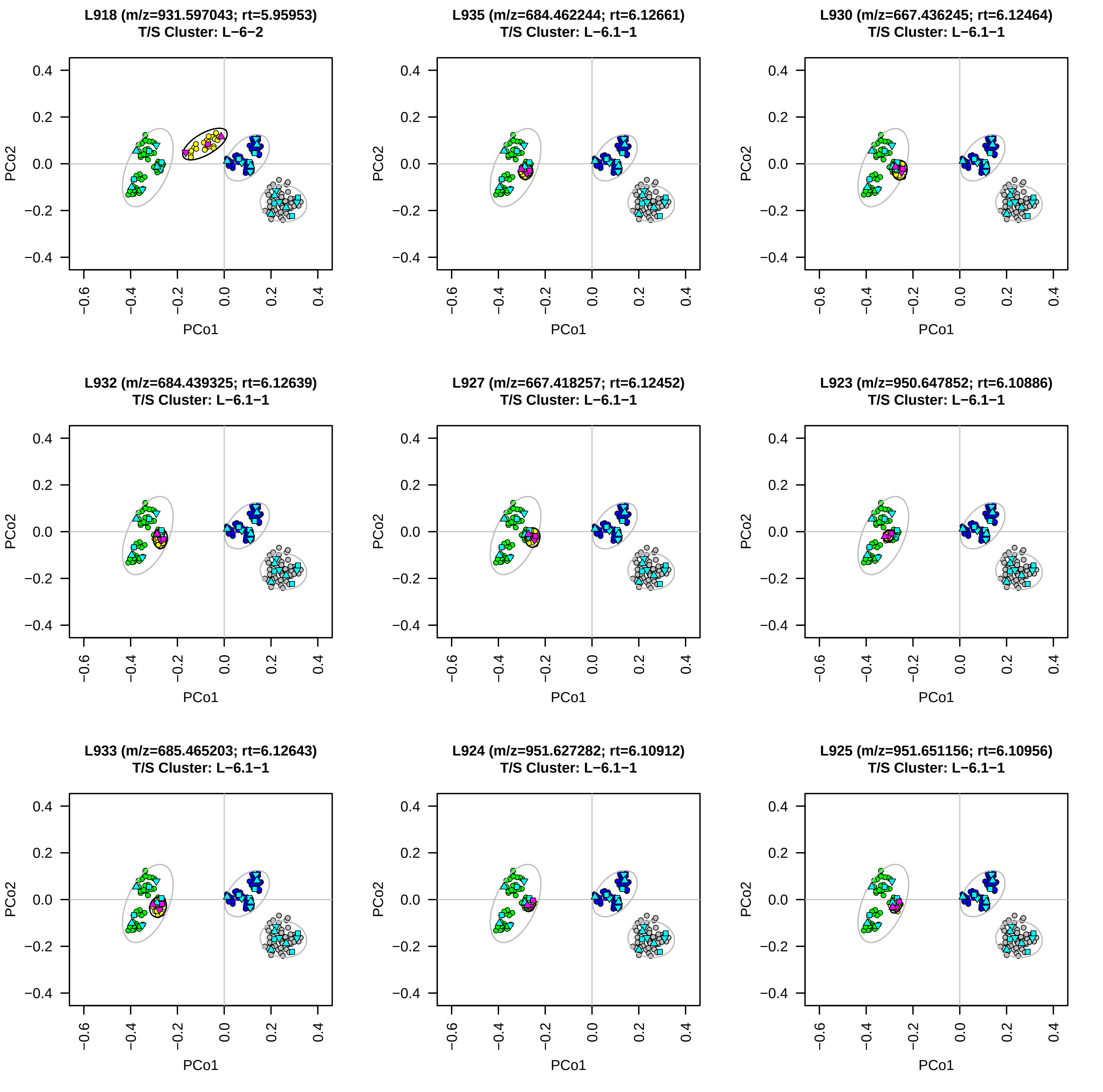
L918 (m/z=931.597043; rt=5.95953)
T/S Cluster: L−6−2
L935 (m/z=684.462244; rt=6.12661)
T/S Cluster: L−6.1−1
L930 (m/z=667.436245; rt=6.12464)
T/S Cluster: L−6.1−1
L932 (m/z=684.439325; rt=6.12639)
T/S Cluster: L−6.1−1
L927 (m/z=667.418257; rt=6.12452)
T/S Cluster: L−6.1−1
L923 (m/z=950.647852; rt=6.10886)
T/S Cluster: L−6.1−1
L933 (m/z=685.465203; rt=6.12643)
T/S Cluster: L−6.1−1
L924 (m/z=951.627282; rt=6.10912)
T/S Cluster: L−6.1−1
L925 (m/z=951.651156; rt=6.10956)
T/S Cluster: L−6.1−1
PCo1
PCo2
0.4
0.2
0.0
−0.2
−0.4
−0.6

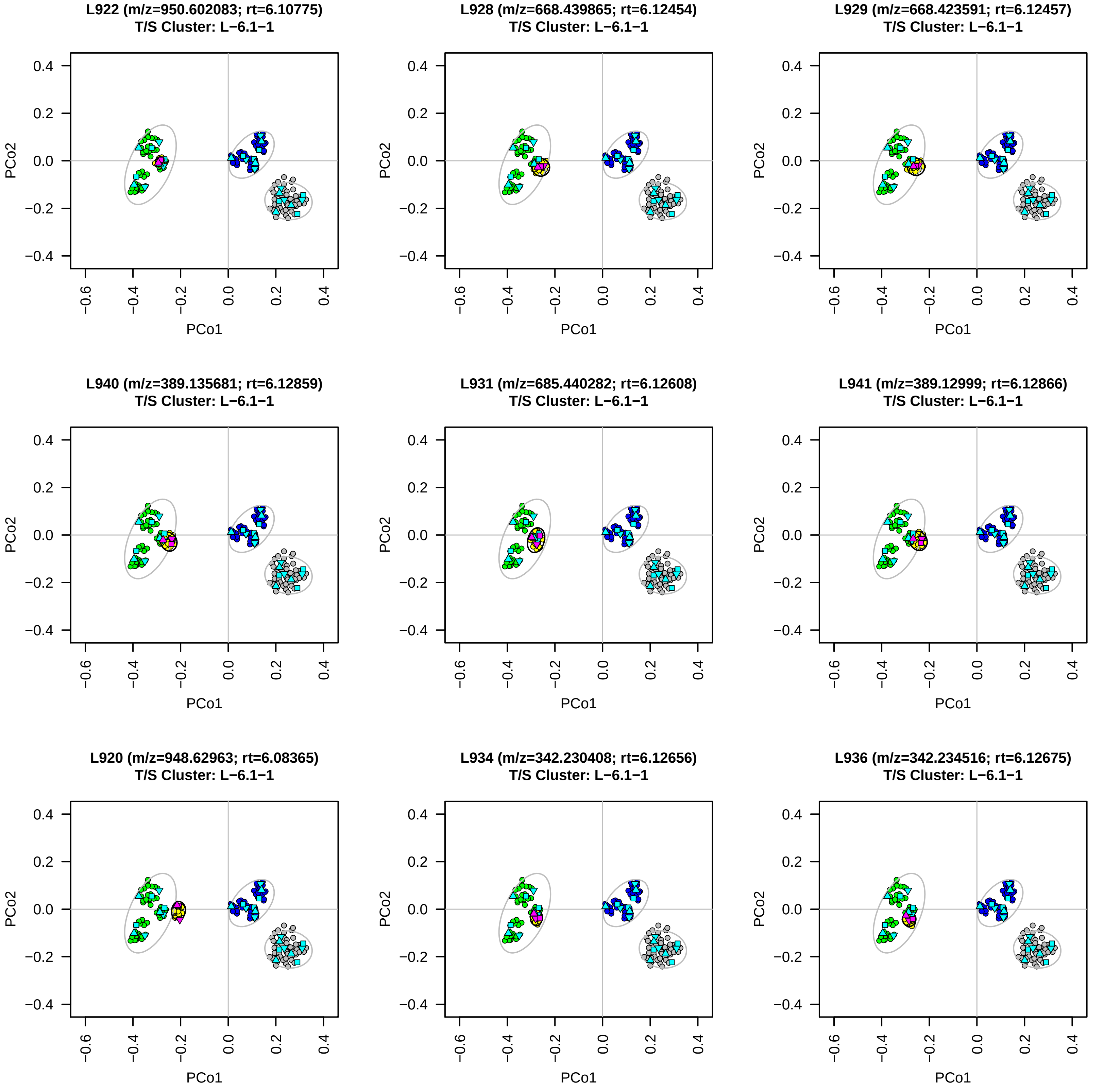
L922 (m/z=950.602083; rt=6.10775)
T/S Cluster: L−6.1−1
L928 (m/z=668.439865; rt=6.12454)
T/S Cluster: L−6.1−1
L929 (m/z=668.423591; rt=6.12457)
T/S Cluster: L−6.1−1
L940 (m/z=389.135681; rt=6.12859)
T/S Cluster: L−6.1−1
L931 (m/z=685.440282; rt=6.12608)
T/S Cluster: L−6.1−1
L941 (m/z=389.12999; rt=6.12866)
T/S Cluster: L−6.1−1
L920 (m/z=948.62963; rt=6.08365)
T/S Cluster: L−6.1−1
L934 (m/z=342.230408; rt=6.12656)
T/S Cluster: L−6.1−1
L936 (m/z=342.234516; rt=6.12675)
T/S Cluster: L−6.1−1
PCo1
PCo2
−0.6
−0.4
−0.2
0.0
0.2
0.4

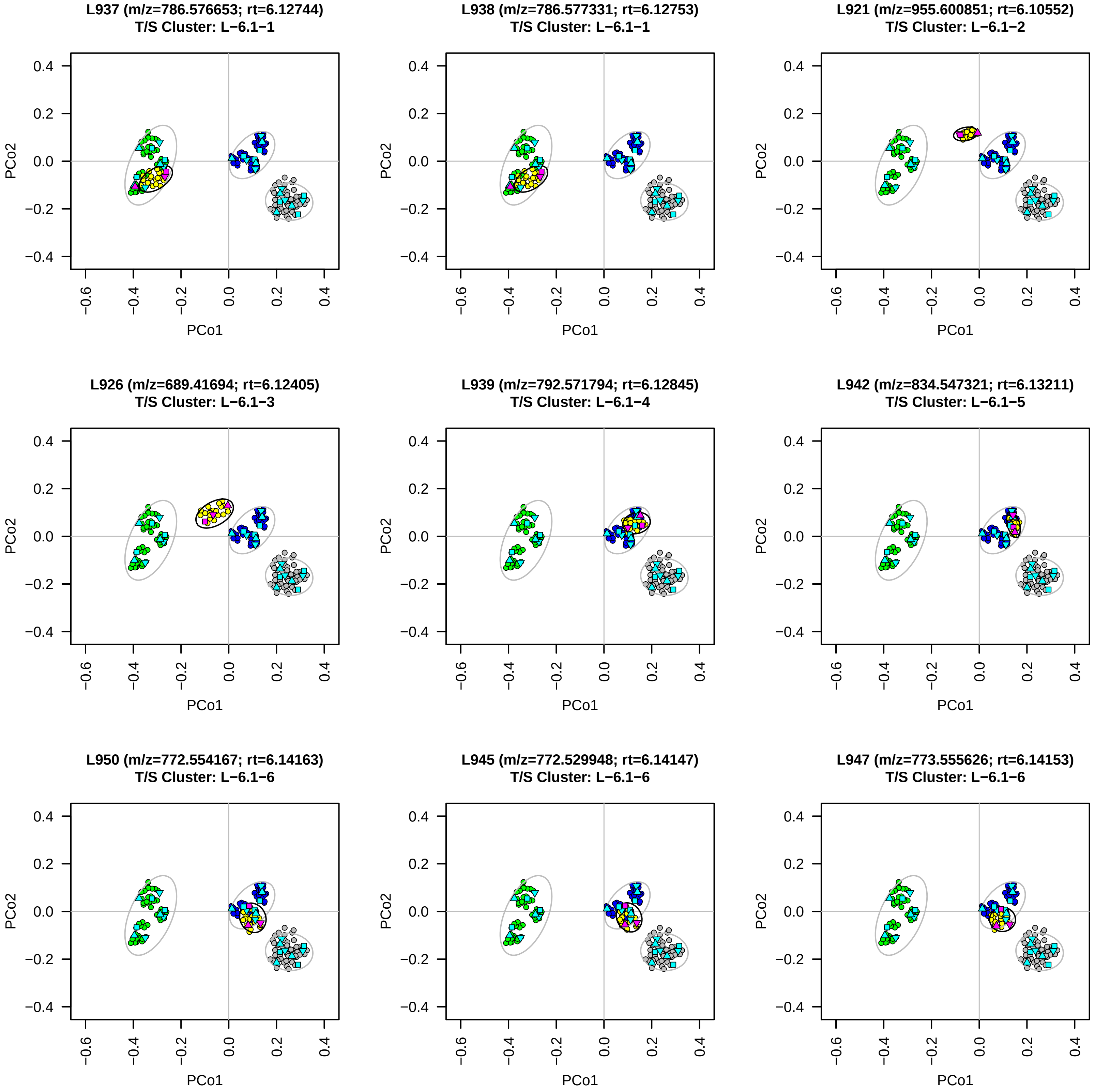
L937 (m/z=786.576653; rt=6.12744)
T/S Cluster: L−6.1−1
L938 (m/z=786.577331; rt=6.12753)
T/S Cluster: L−6.1−1
L921 (m/z=955.600851; rt=6.10552)
T/S Cluster: L−6.1−2
L926 (m/z=689.41694; rt=6.12405)
T/S Cluster: L−6.1−3
L939 (m/z=792.571794; rt=6.12845)
T/S Cluster: L−6.1−4
L942 (m/z=834.547321; rt=6.13211)
T/S Cluster: L−6.1−5
L950 (m/z=772.554167; rt=6.14163)
T/S Cluster: L−6.1−6
L945 (m/z=772.529948; rt=6.14147)
T/S Cluster: L−6.1−6
L947 (m/z=773.555626; rt=6.14153)
T/S Cluster: L−6.1−6
PCo1
PCo2
0.4
0.2
0.0
−0.2
−0.4
−0.6

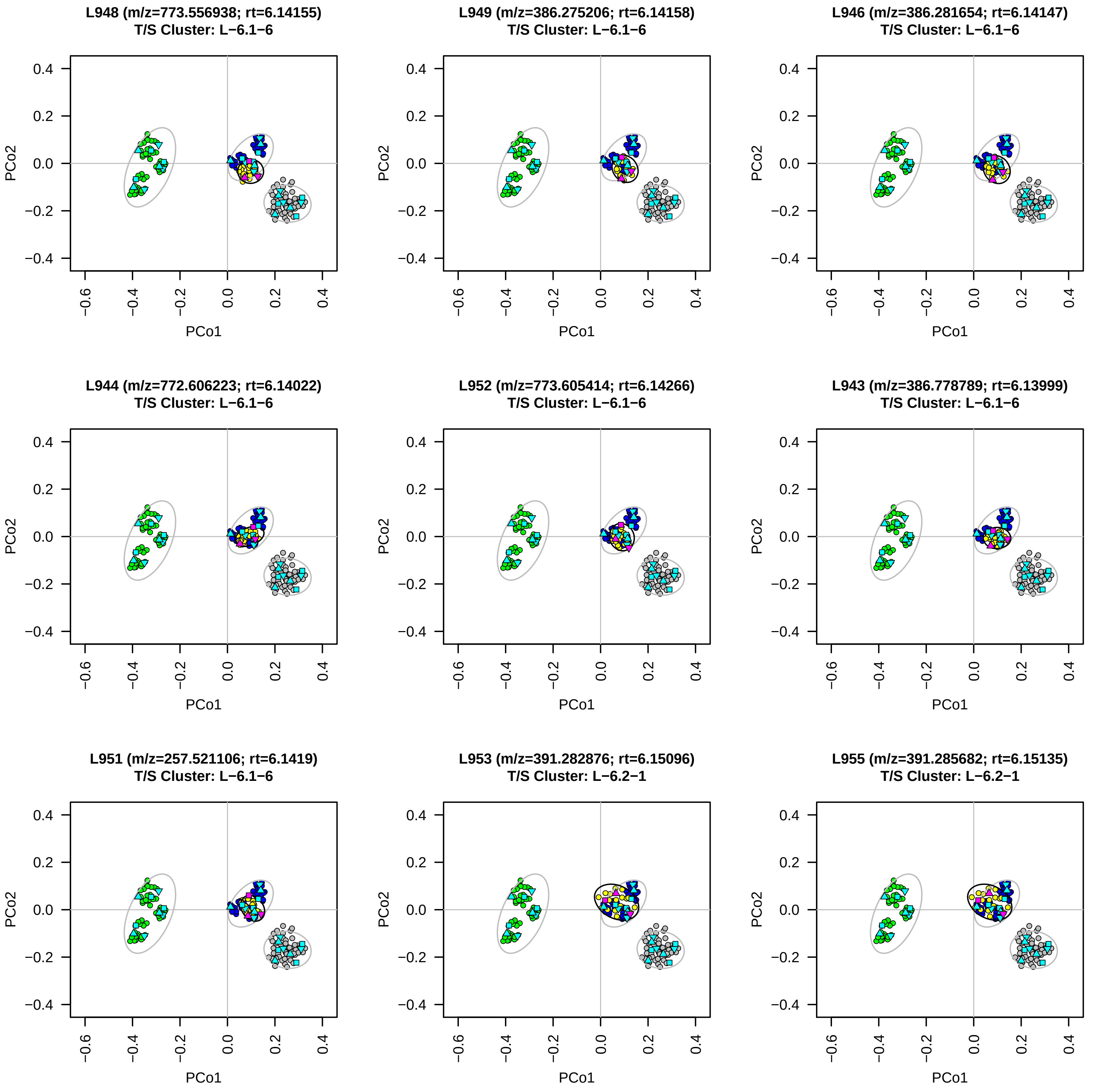
L948 (m/z=773.556938; rt=6.14155)
T/S Cluster: L−6.1−6
L949 (m/z=386.275206; rt=6.14158)
T/S Cluster: L−6.1−6
L946 (m/z=386.281654; rt=6.14147)
T/S Cluster: L−6.1−6
L944 (m/z=772.606223; rt=6.14022)
T/S Cluster: L−6.1−6
L952 (m/z=773.605414; rt=6.14266)
T/S Cluster: L−6.1−6
L943 (m/z=386.778789; rt=6.13999)
T/S Cluster: L−6.1−6
L951 (m/z=257.521106; rt=6.1419)
T/S Cluster: L−6.1−6
L953 (m/z=391.282876; rt=6.15096)
T/S Cluster: L−6.2−1
L955 (m/z=391.285682; rt=6.15135)
T/S Cluster: L−6.2−1
PCo1
PCo2
0.4
0.2
0.0
−0.2
−0.4
−0.6

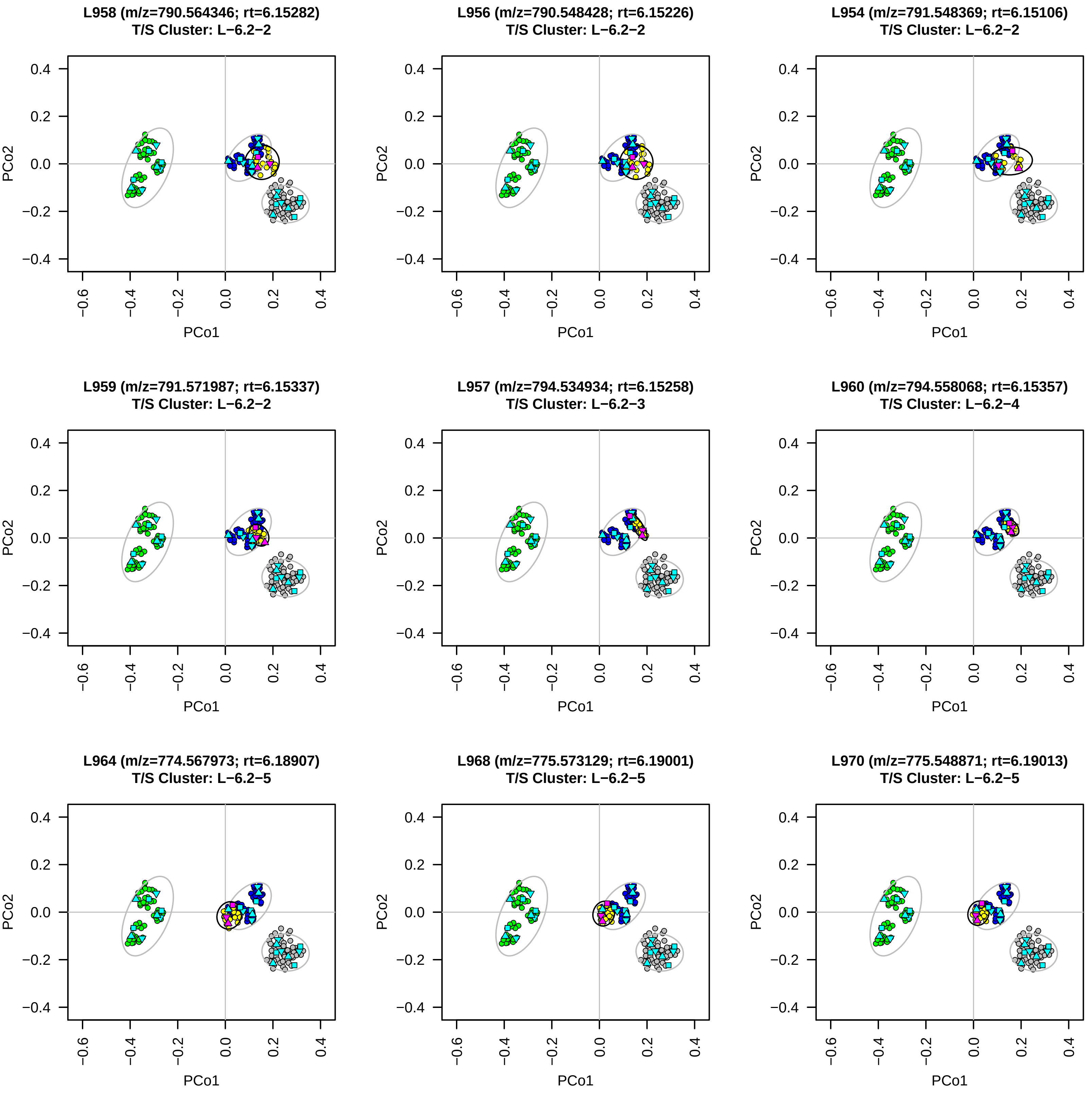
L958 (m/z=790.564346; rt=6.15282)
T/S Cluster: L−6.2−2
L956 (m/z=790.548428; rt=6.15226)
T/S Cluster: L−6.2−2
L954 (m/z=791.548369; rt=6.15106)
T/S Cluster: L−6.2−2
L959 (m/z=791.571987; rt=6.15337)
T/S Cluster: L−6.2−2
L957 (m/z=794.534934; rt=6.15258)
T/S Cluster: L−6.2−3
L960 (m/z=794.558068; rt=6.15357)
T/S Cluster: L−6.2−4
L964 (m/z=774.567973; rt=6.18907)
T/S Cluster: L−6.2−5
L968 (m/z=775.573129; rt=6.19001)
T/S Cluster: L−6.2−5
L970 (m/z=775.548871; rt=6.19013)
T/S Cluster: L−6.2−5
PCo1
PCo2
−0.6
−0.4
−0.2
0.0
0.2
0.4

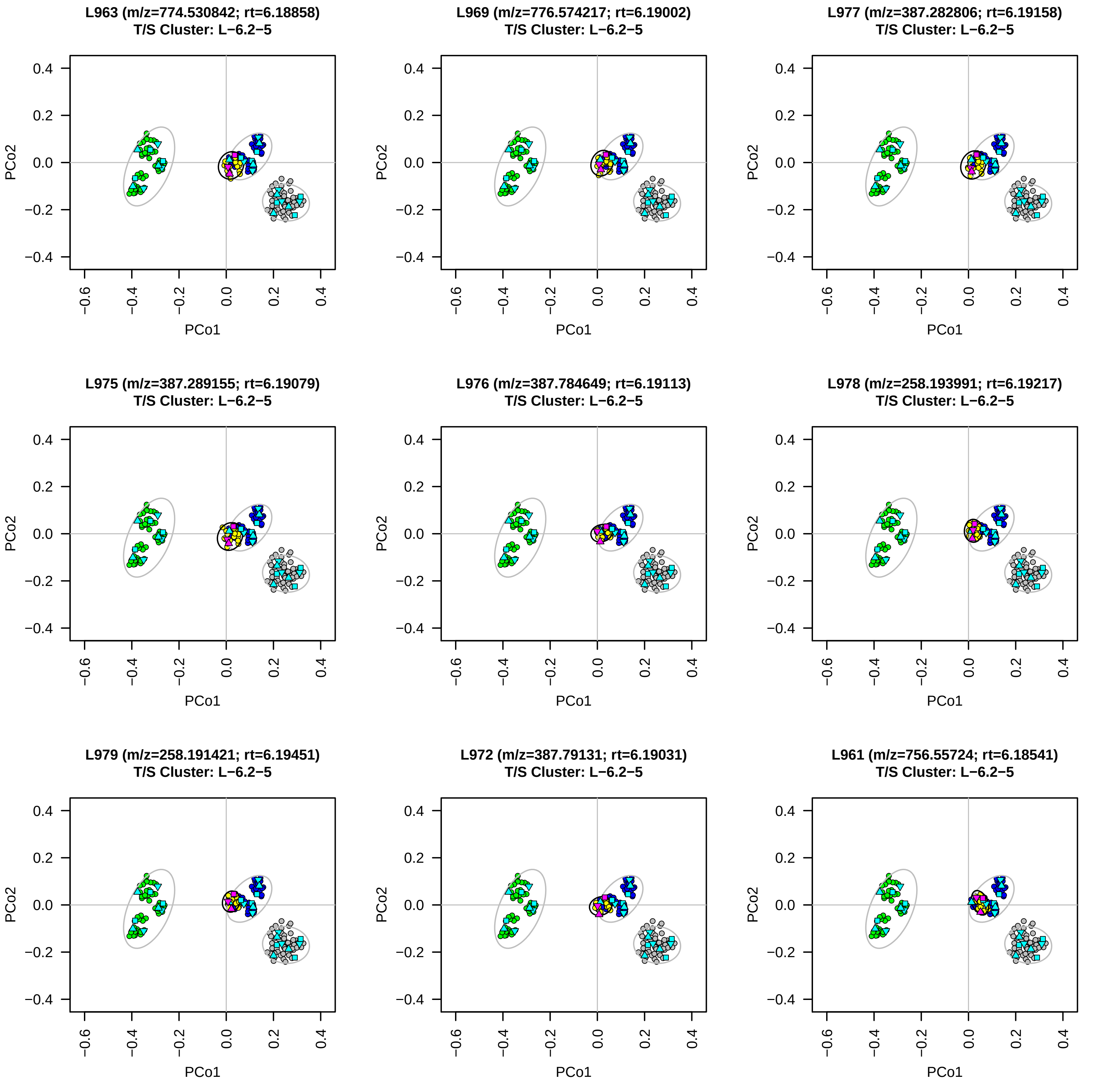
L963 (m/z=774.530842; rt=6.18858)
T/S Cluster: L−6.2−5
L969 (m/z=776.574217; rt=6.19002)
T/S Cluster: L−6.2−5
L977 (m/z=387.282806; rt=6.19158)
T/S Cluster: L−6.2−5
L975 (m/z=387.289155; rt=6.19079)
T/S Cluster: L−6.2−5
L976 (m/z=387.784649; rt=6.19113)
T/S Cluster: L−6.2−5
L978 (m/z=258.193991; rt=6.19217)
T/S Cluster: L−6.2−5
L979 (m/z=258.191421; rt=6.19451)
T/S Cluster: L−6.2−5
L972 (m/z=387.79131; rt=6.19031)
T/S Cluster: L−6.2−5
L961 (m/z=756.55724; rt=6.18541)
T/S Cluster: L−6.2−5
PCo1
PCo2
0.4
0.2
0.0
−0.2
−0.4
−0.6

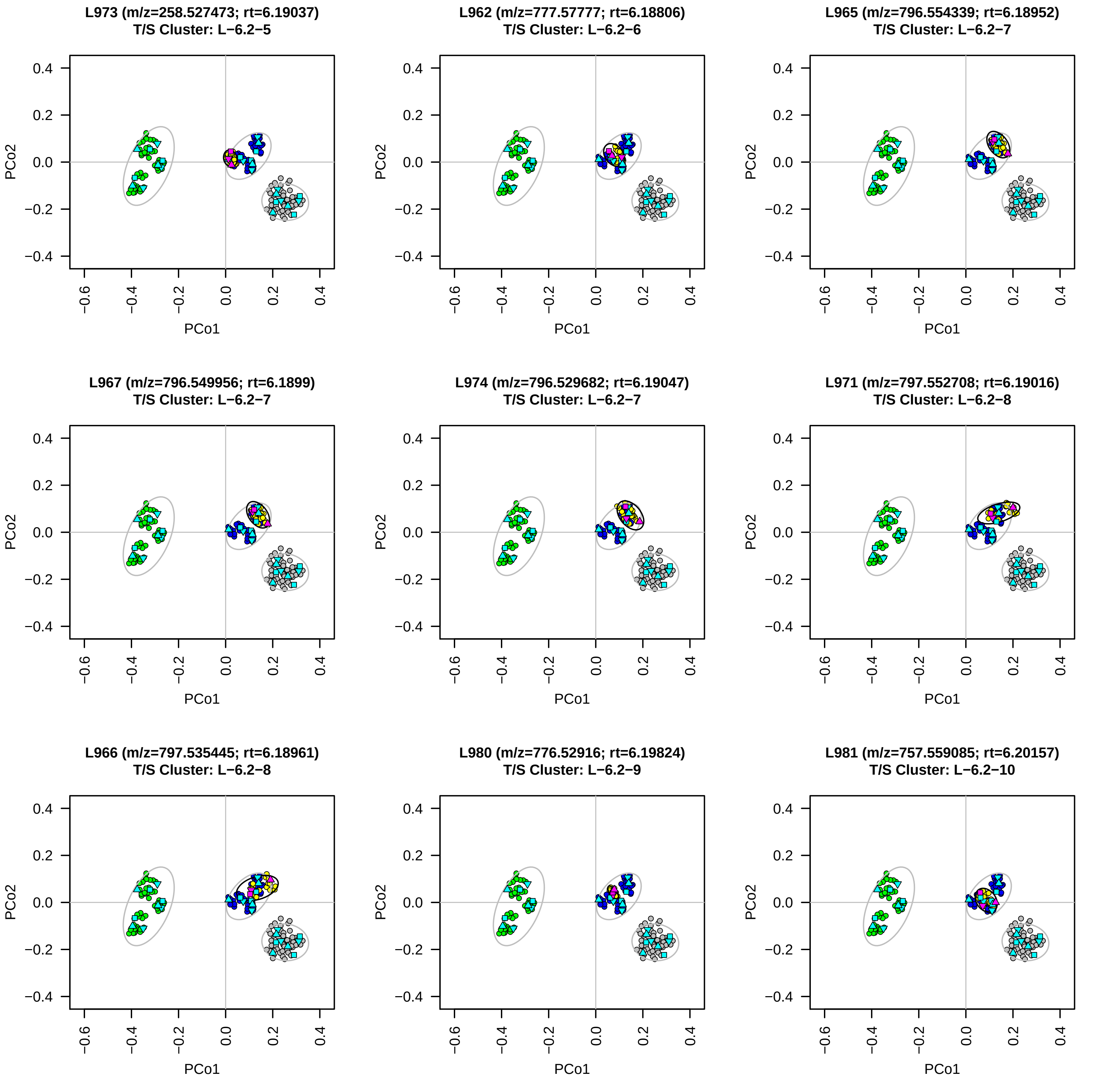
L973 (m/z=258.527473; rt=6.19037)
T/S Cluster: L−6.2−5
L962 (m/z=777.57777; rt=6.18806)
T/S Cluster: L−6.2−6
L965 (m/z=796.554339; rt=6.18952)
T/S Cluster: L−6.2−7
L967 (m/z=796.549956; rt=6.1899)
T/S Cluster: L−6.2−7
L974 (m/z=796.529682; rt=6.19047)
T/S Cluster: L−6.2−7
L971 (m/z=797.552708; rt=6.19016)
T/S Cluster: L−6.2−8
L966 (m/z=797.535445; rt=6.18961)
T/S Cluster: L−6.2−8
L980 (m/z=776.52916; rt=6.19824)
T/S Cluster: L−6.2−9
L981 (m/z=757.559085; rt=6.20157)
T/S Cluster: L−6.2−10
PCo1
PCo2
−0.6
−0.4
−0.2
0.0
0.2
0.4

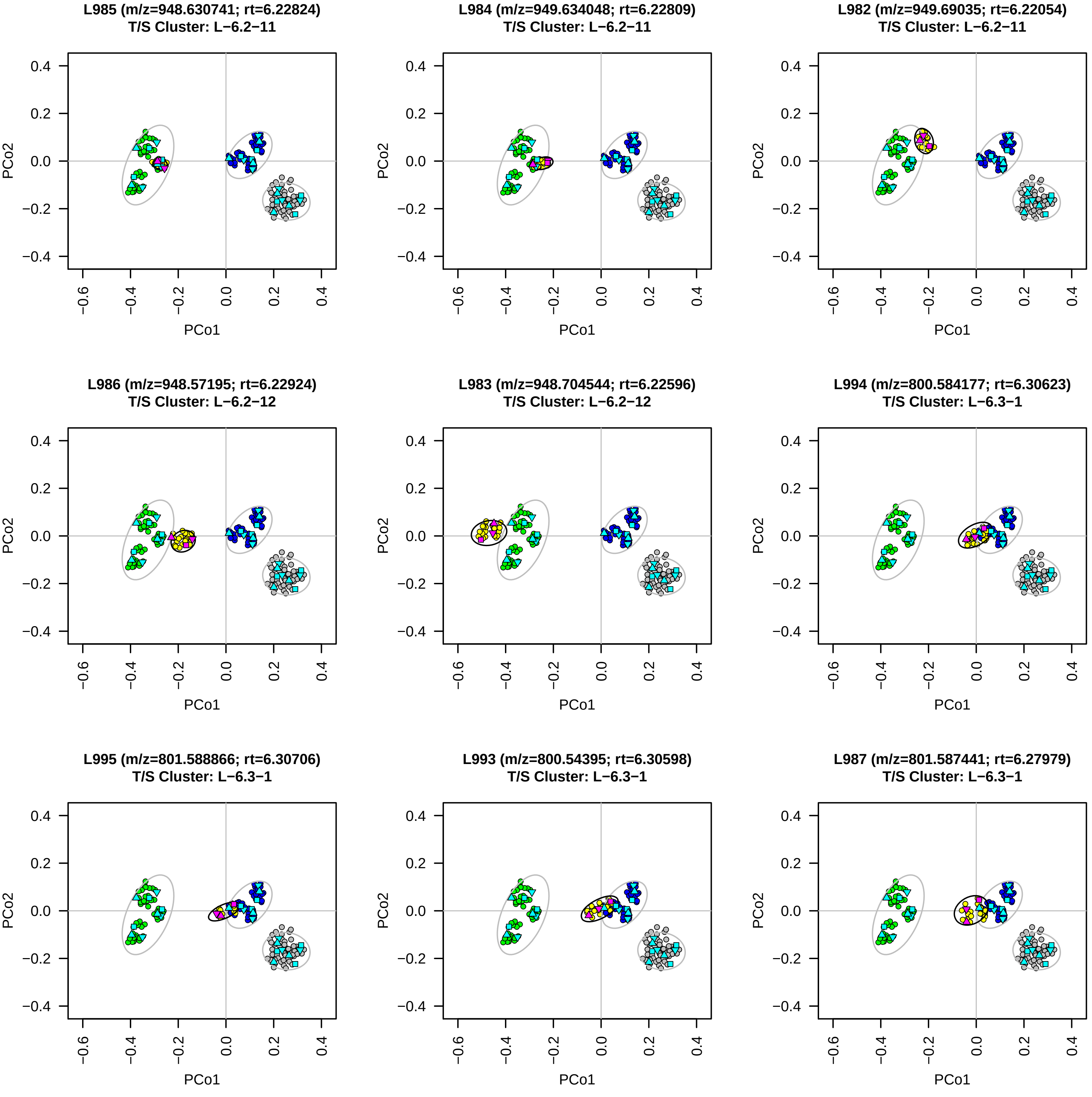
L985 (m/z=948.630741; rt=6.22824)
T/S Cluster: L−6.2−11
L984 (m/z=949.634048; rt=6.22809)
T/S Cluster: L−6.2−11
L982 (m/z=949.69035; rt=6.22054)
T/S Cluster: L−6.2−11
L986 (m/z=948.57195; rt=6.22924)
T/S Cluster: L−6.2−12
L983 (m/z=948.704544; rt=6.22596)
T/S Cluster: L−6.2−12
L994 (m/z=800.584177; rt=6.30623)
T/S Cluster: L−6.3−1
L995 (m/z=801.588866; rt=6.30706)
T/S Cluster: L−6.3−1
L993 (m/z=800.54395; rt=6.30598)
T/S Cluster: L−6.3−1
L987 (m/z=801.587441; rt=6.27979)
T/S Cluster: L−6.3−1
PCo1
PCo2
0.4
0.2
0.0
−0.2
−0.4
−0.6

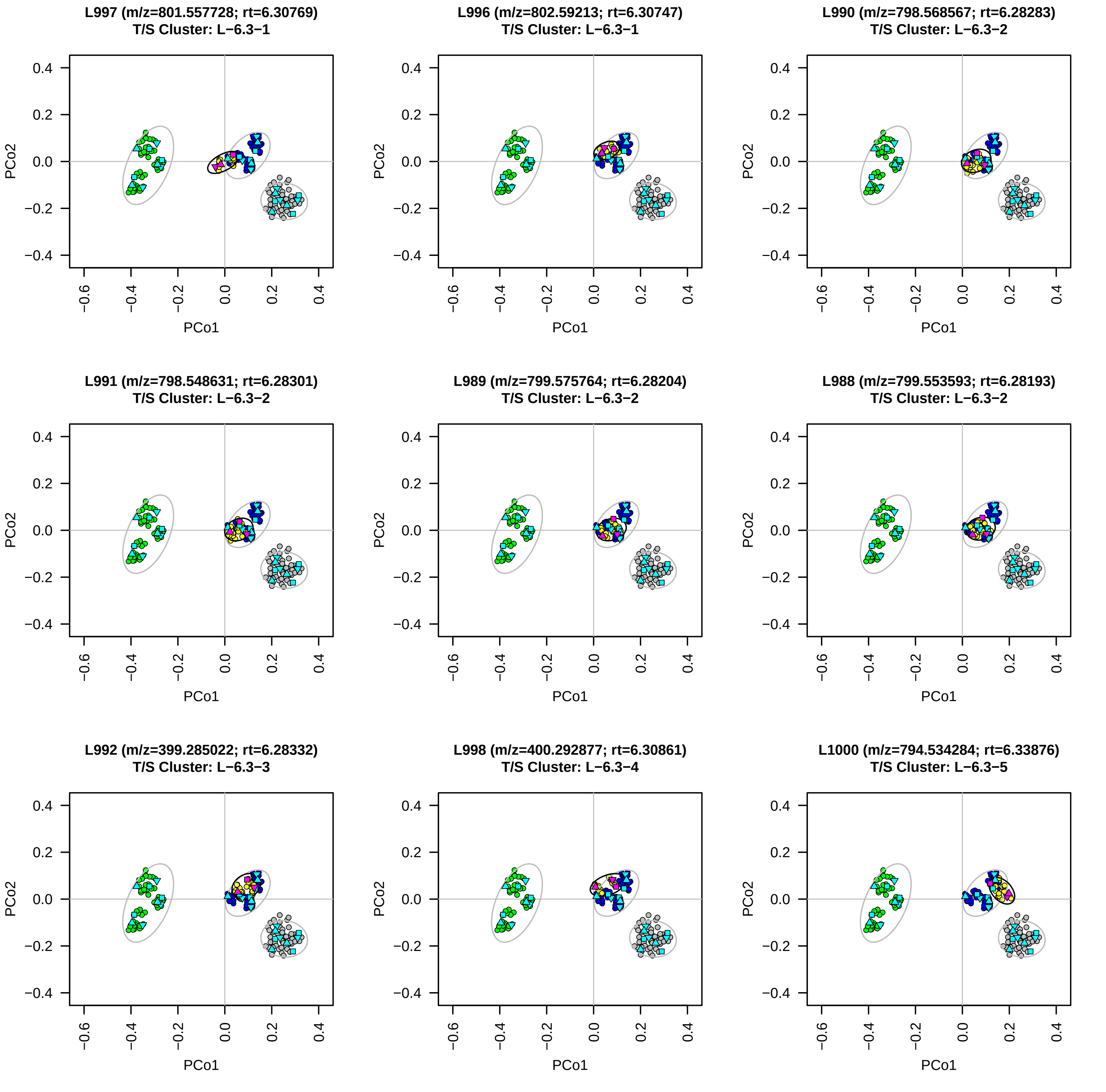
L997 (m/z=801.557728; rt=6.30769)
T/S Cluster: L−6.3−1
L996 (m/z=802.59213; rt=6.30747)
T/S Cluster: L−6.3−1
L990 (m/z=798.568567; rt=6.28283)
T/S Cluster: L−6.3−2
L991 (m/z=798.548631; rt=6.28301)
T/S Cluster: L−6.3−2
L989 (m/z=799.575764; rt=6.28204)
T/S Cluster: L−6.3−2
L988 (m/z=799.553593; rt=6.28193)
T/S Cluster: L−6.3−2
L992 (m/z=399.285022; rt=6.28332)
T/S Cluster: L−6.3−3
L998 (m/z=400.292877; rt=6.30861)
T/S Cluster: L−6.3−4
L1000 (m/z=794.534284; rt=6.33876)
T/S Cluster: L−6.3−5
PCo1
PCo2
0.4
0.2
0.0
−0.2
−0.4
−0.6

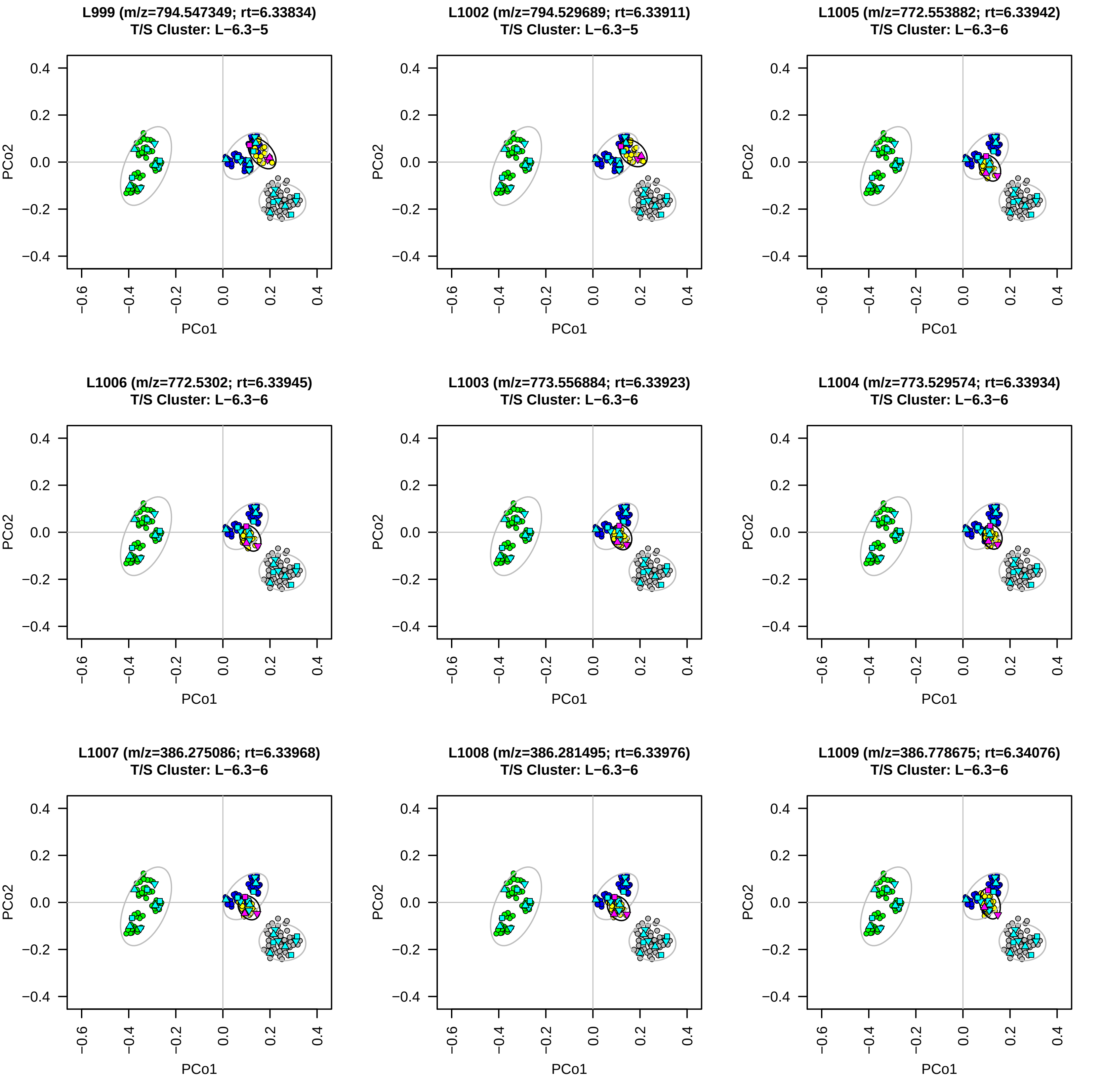
L999 (m/z=794.547349; rt=6.33834)
T/S Cluster: L−6.3−5
L1002 (m/z=794.529689; rt=6.33911)
T/S Cluster: L−6.3−5
L1005 (m/z=772.553882; rt=6.33942)
T/S Cluster: L−6.3−6
L1006 (m/z=772.5302; rt=6.33945)
T/S Cluster: L−6.3−6
L1003 (m/z=773.556884; rt=6.33923)
T/S Cluster: L−6.3−6
L1004 (m/z=773.529574; rt=6.33934)
T/S Cluster: L−6.3−6
L1007 (m/z=386.275086; rt=6.33968)
T/S Cluster: L−6.3−6
L1008 (m/z=386.281495; rt=6.33976)
T/S Cluster: L−6.3−6
L1009 (m/z=386.778675; rt=6.34076)
T/S Cluster: L−6.3−6
PCo1
PCo2
−0.6
−0.4
−0.2
0.0
0.2
0.4

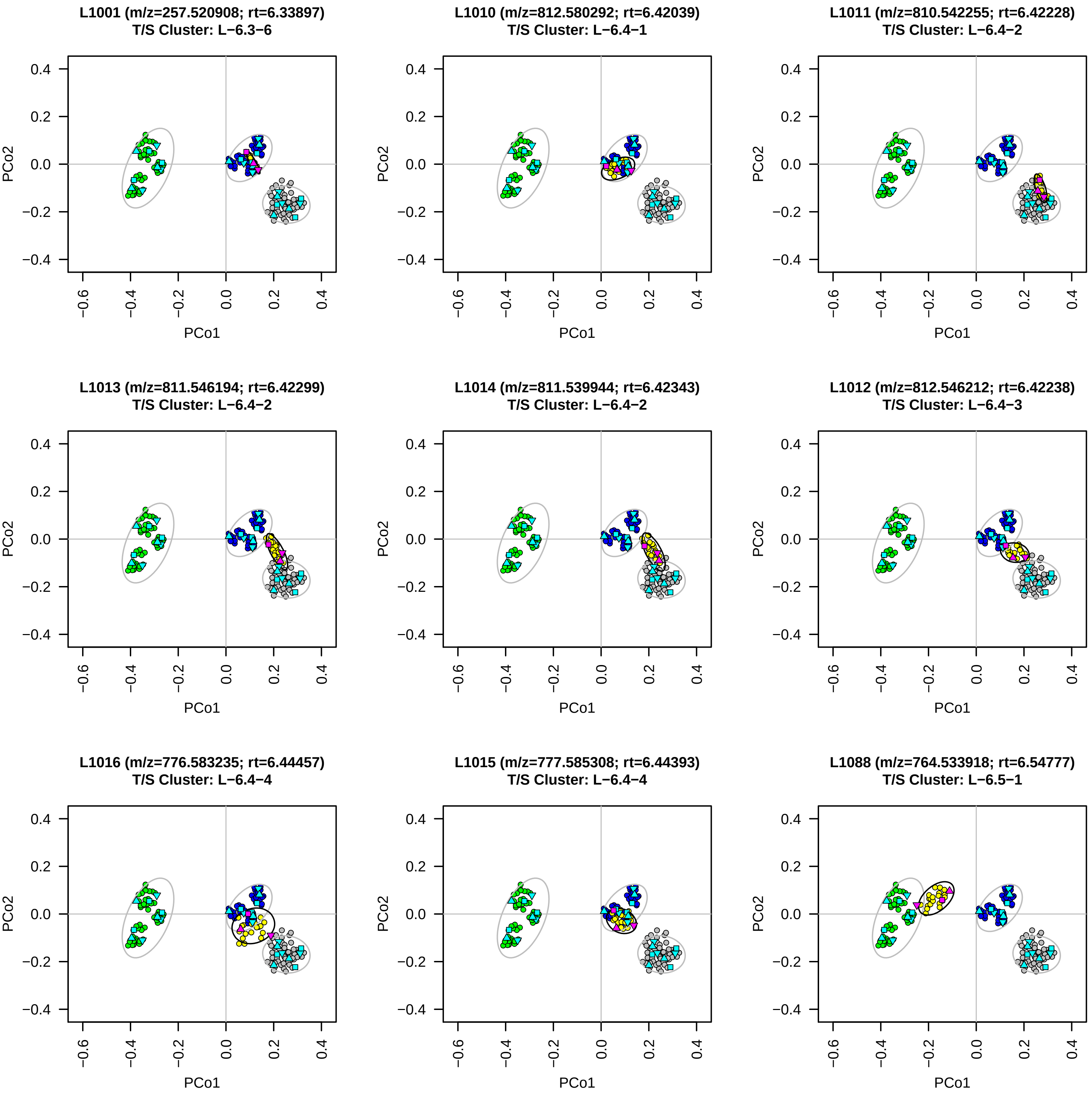
L1001 (m/z=257.520908; rt=6.33897)
T/S Cluster: L−6.3−6
L1010 (m/z=812.580292; rt=6.42039)
T/S Cluster: L−6.4−1
L1011 (m/z=810.542255; rt=6.42228)
T/S Cluster: L−6.4−2
L1013 (m/z=811.546194; rt=6.42299)
T/S Cluster: L−6.4−2
L1014 (m/z=811.539944; rt=6.42343)
T/S Cluster: L−6.4−2
L1012 (m/z=812.546212; rt=6.42238)
T/S Cluster: L−6.4−3
L1016 (m/z=776.583235; rt=6.44457)
T/S Cluster: L−6.4−4
L1015 (m/z=777.585308; rt=6.44393)
T/S Cluster: L−6.4−4
L1088 (m/z=764.533918; rt=6.54777)
T/S Cluster: L−6.5−1
PCo1
PCo2

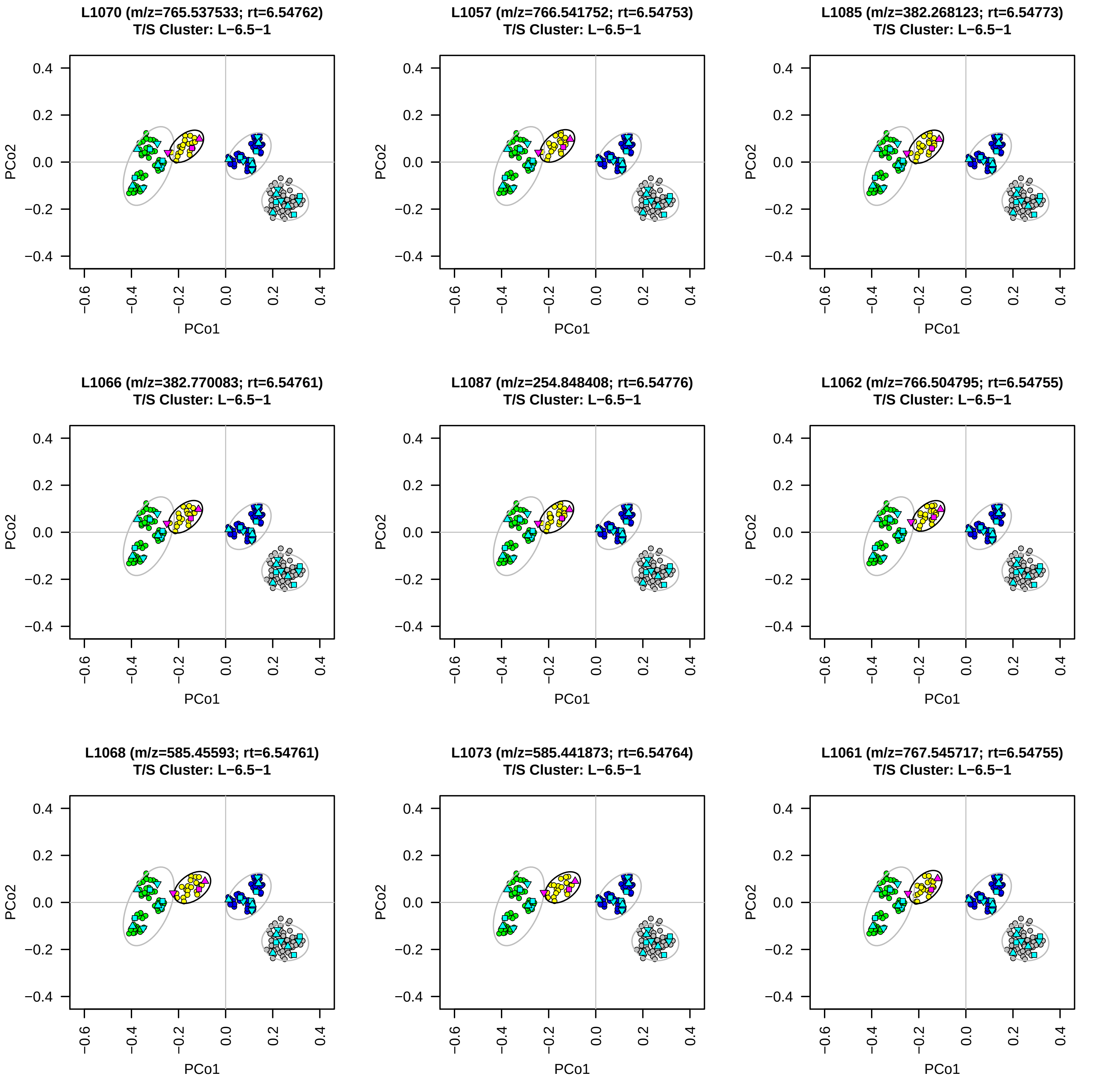
L1070 (m/z=765.537533; rt=6.54762)
T/S Cluster: L−6.5−1
L1057 (m/z=766.541752; rt=6.54753)
T/S Cluster: L−6.5−1
L1085 (m/z=382.268123; rt=6.54773)
T/S Cluster: L−6.5−1
L1066 (m/z=382.770083; rt=6.54761)
T/S Cluster: L−6.5−1
L1087 (m/z=254.848408; rt=6.54776)
T/S Cluster: L−6.5−1
L1062 (m/z=766.504795; rt=6.54755)
T/S Cluster: L−6.5−1
L1068 (m/z=585.45593; rt=6.54761)
T/S Cluster: L−6.5−1
L1073 (m/z=585.441873; rt=6.54764)
T/S Cluster: L−6.5−1
L1061 (m/z=767.545717; rt=6.54755)
T/S Cluster: L−6.5−1
PCo2
PCo1
0.4
0.2
0.0
−0.2
−0.4
−0.6

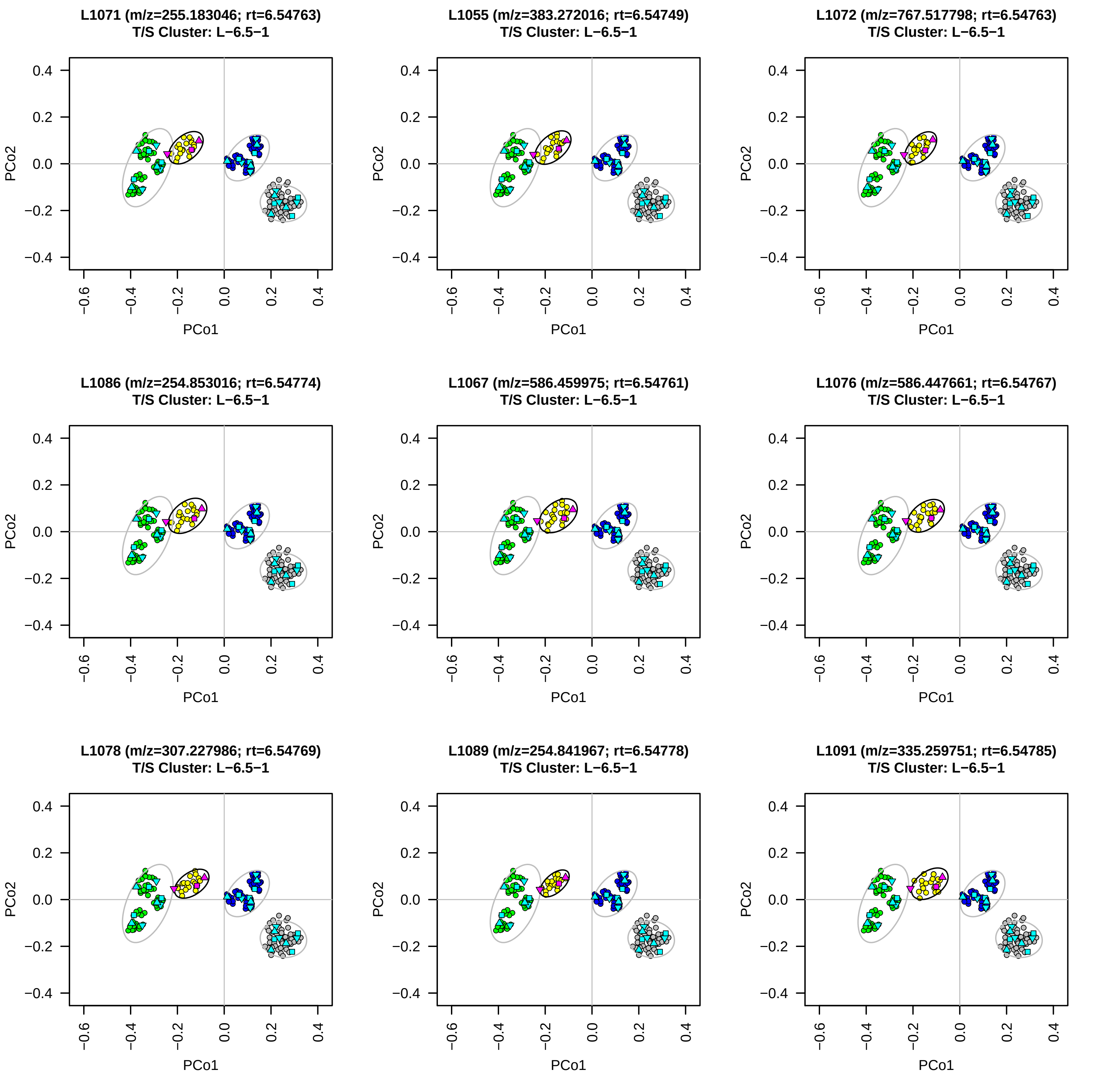
L1071 (m/z=255.183046; rt=6.54763)
T/S Cluster: L−6.5−1
L1055 (m/z=383.272016; rt=6.54749)
T/S Cluster: L−6.5−1
L1072 (m/z=767.517798; rt=6.54763)
T/S Cluster: L−6.5−1
L1086 (m/z=254.853016; rt=6.54774)
T/S Cluster: L−6.5−1
L1067 (m/z=586.459975; rt=6.54761)
T/S Cluster: L−6.5−1
L1076 (m/z=586.447661; rt=6.54767)
T/S Cluster: L−6.5−1
L1078 (m/z=307.227986; rt=6.54769)
T/S Cluster: L−6.5−1
L1089 (m/z=254.841967; rt=6.54778)
T/S Cluster: L−6.5−1
L1091 (m/z=335.259751; rt=6.54785)
T/S Cluster: L−6.5−1
PCo1
PCo2
0.4
0.2
0.0
−0.2
−0.4
−0.6

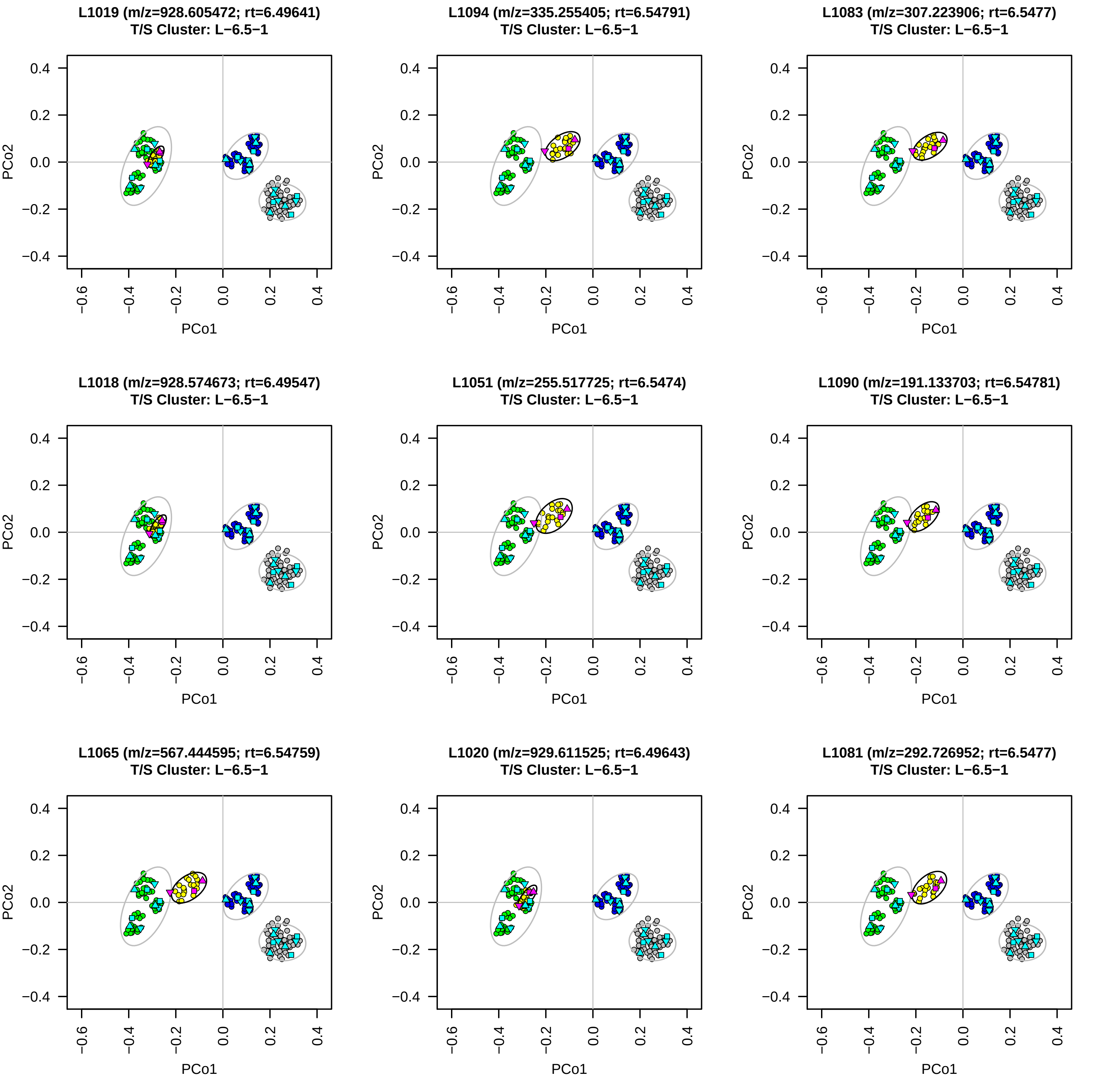
L1019 (m/z=928.605472; rt=6.49641)
T/S Cluster: L−6.5−1
L1094 (m/z=335.255405; rt=6.54791)
T/S Cluster: L−6.5−1
L1083 (m/z=307.223906; rt=6.5477)
T/S Cluster: L−6.5−1
L1018 (m/z=928.574673; rt=6.49547)
T/S Cluster: L−6.5−1
L1051 (m/z=255.517725; rt=6.5474)
T/S Cluster: L−6.5−1
L1090 (m/z=191.133703; rt=6.54781)
T/S Cluster: L−6.5−1
L1065 (m/z=567.444595; rt=6.54759)
T/S Cluster: L−6.5−1
L1020 (m/z=929.611525; rt=6.49643)
T/S Cluster: L−6.5−1
L1081 (m/z=292.726952; rt=6.5477)
T/S Cluster: L−6.5−1
PCo1
PCo2
0.4
0.2
0.0
−0.2
−0.4
−0.6

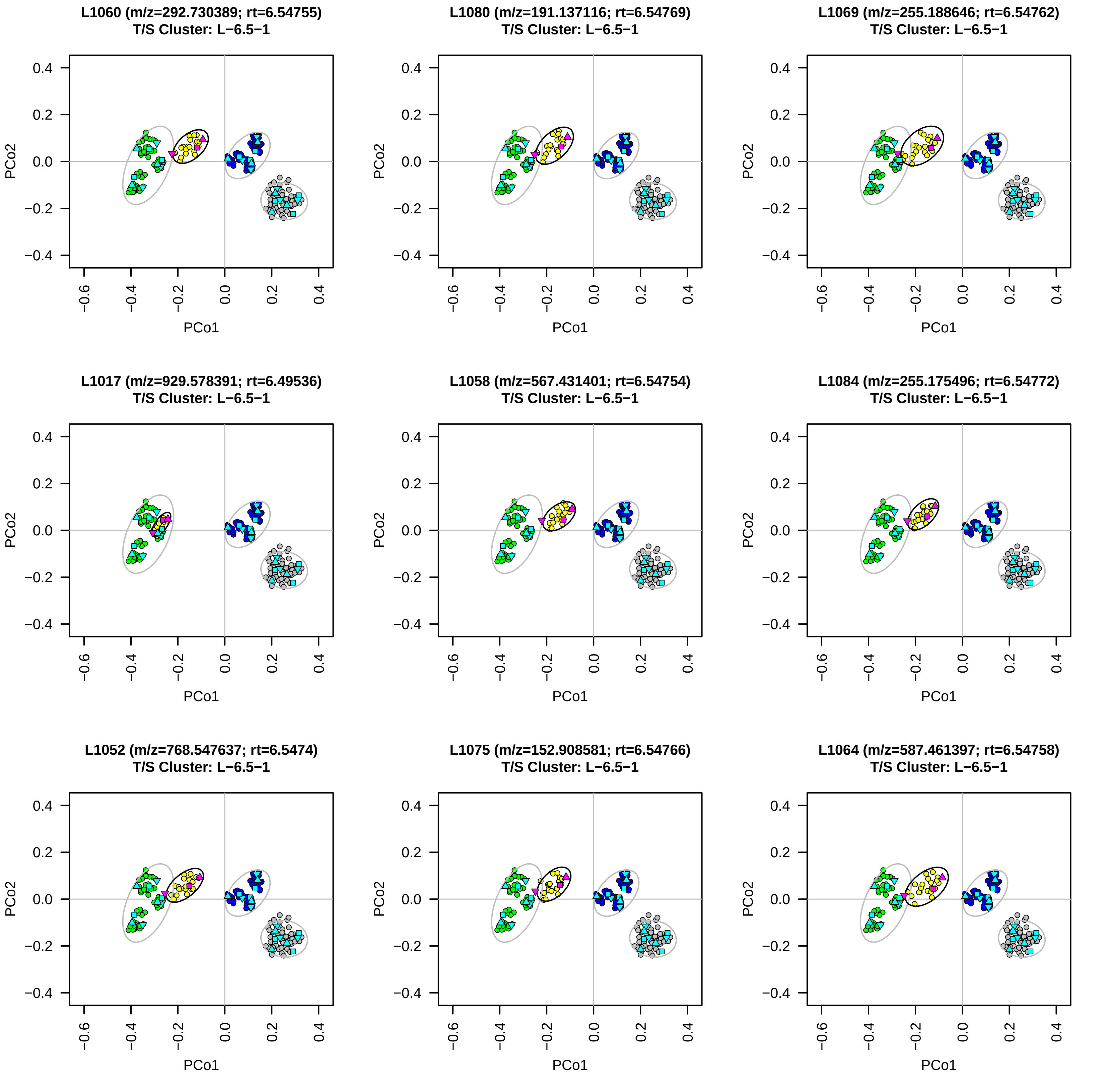
L1060 (m/z=292.730389; rt=6.54755)
T/S Cluster: L−6.5−1
L1080 (m/z=191.137116; rt=6.54769)
T/S Cluster: L−6.5−1
L1069 (m/z=255.188646; rt=6.54762)
T/S Cluster: L−6.5−1
L1017 (m/z=929.578391; rt=6.49536)
T/S Cluster: L−6.5−1
L1058 (m/z=567.431401; rt=6.54754)
T/S Cluster: L−6.5−1
L1084 (m/z=255.175496; rt=6.54772)
T/S Cluster: L−6.5−1
L1052 (m/z=768.547637; rt=6.5474)
T/S Cluster: L−6.5−1
L1075 (m/z=152.908581; rt=6.54766)
T/S Cluster: L−6.5−1
L1064 (m/z=587.461397; rt=6.54758)
T/S Cluster: L−6.5−1
PCo1
PCo2
0.4
0.2
0.0
−0.2
−0.4
−0.6

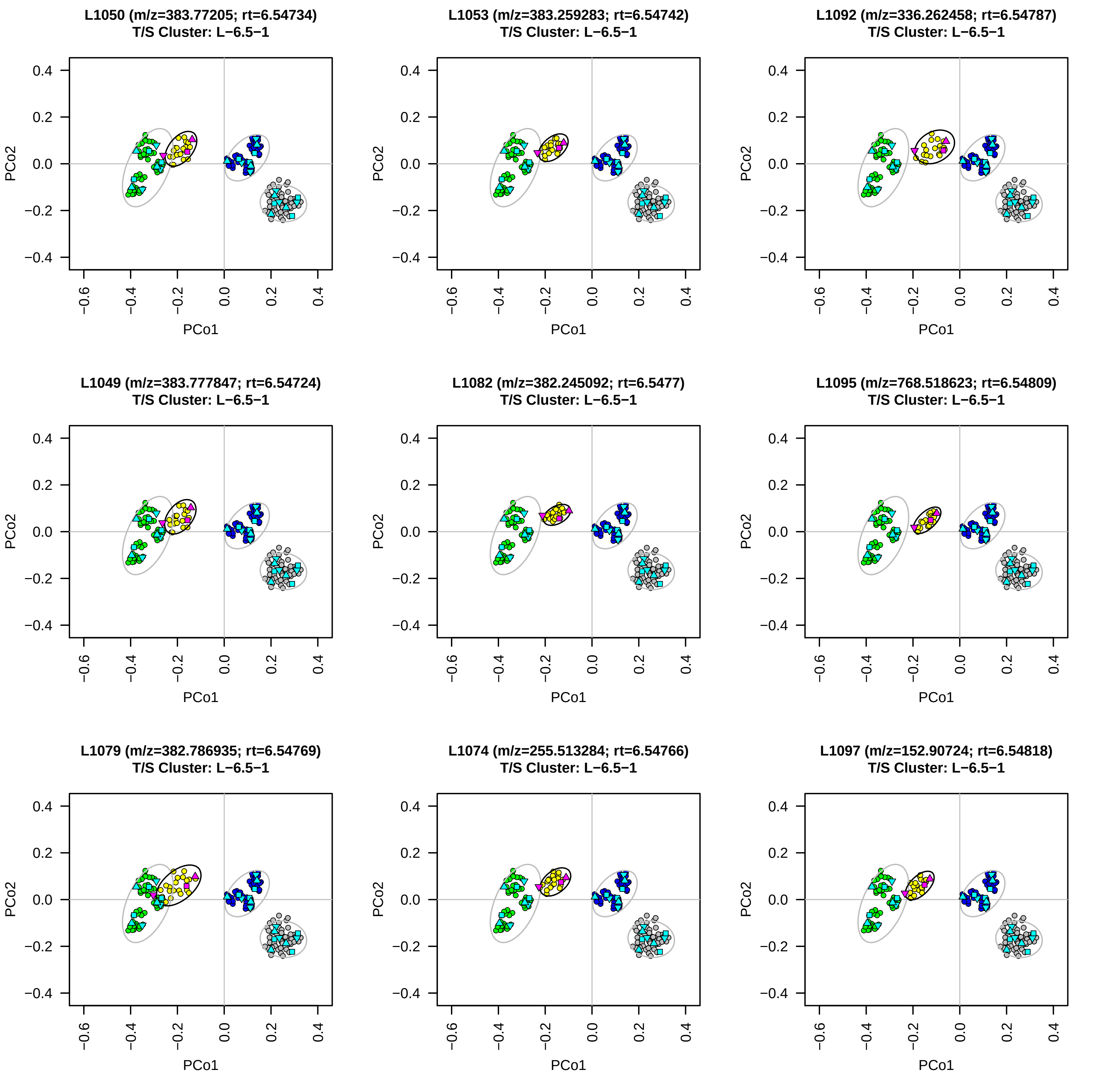
L1050 (m/z=383.77205; rt=6.54734)
T/S Cluster: L−6.5−1
L1053 (m/z=383.259283; rt=6.54742)
T/S Cluster: L−6.5−1
L1092 (m/z=336.262458; rt=6.54787)
T/S Cluster: L−6.5−1
L1049 (m/z=383.777847; rt=6.54724)
T/S Cluster: L−6.5−1
L1082 (m/z=382.245092; rt=6.5477)
T/S Cluster: L−6.5−1
L1095 (m/z=768.518623; rt=6.54809)
T/S Cluster: L−6.5−1
L1079 (m/z=382.786935; rt=6.54769)
T/S Cluster: L−6.5−1
L1074 (m/z=255.513284; rt=6.54766)
T/S Cluster: L−6.5−1
L1097 (m/z=152.90724; rt=6.54818)
T/S Cluster: L−6.5−1
PCo1
PCo2
0.4
0.2
0.0
−0.2
−0.4
−0.6

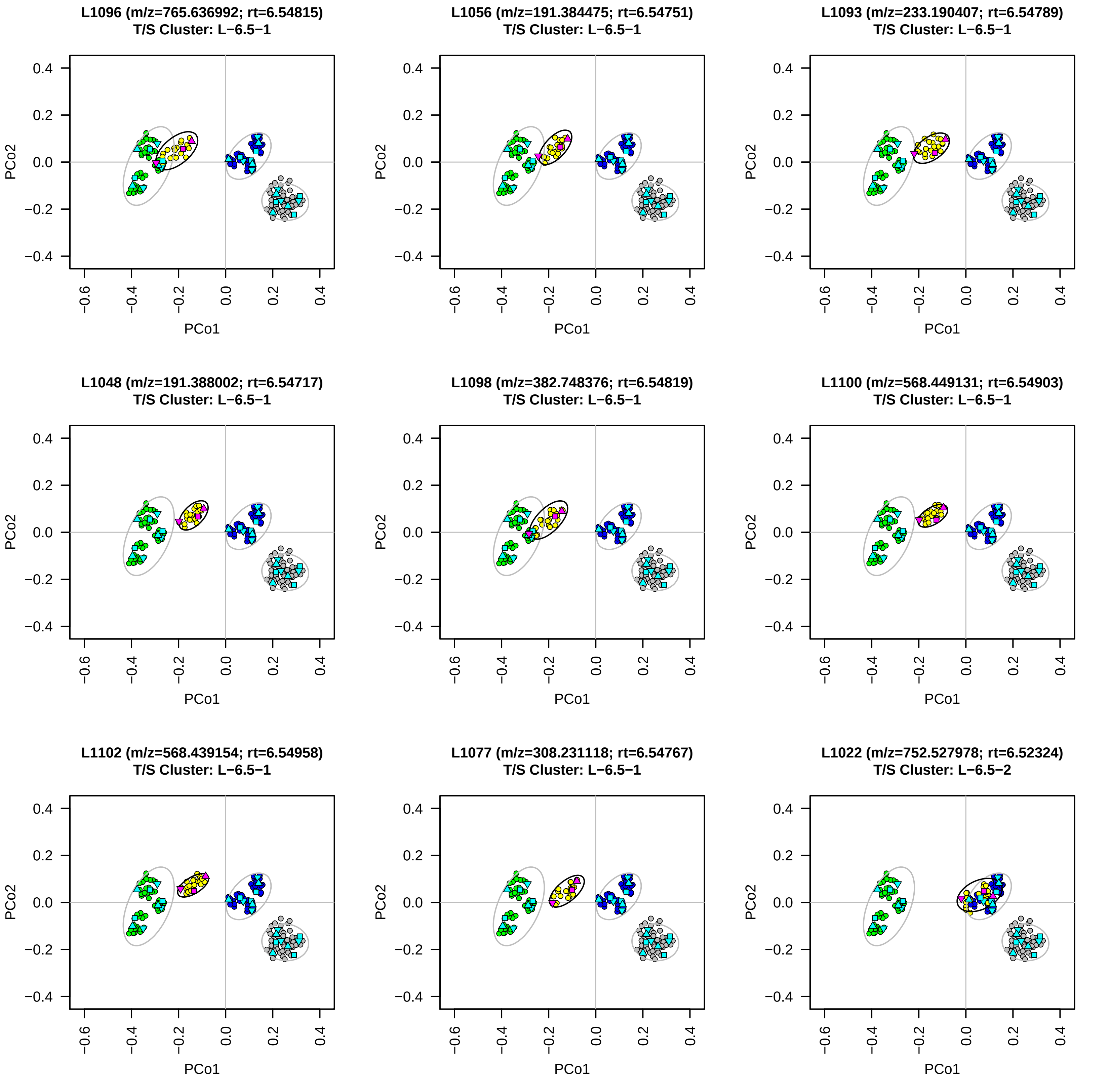
L1096 (m/z=765.636992; rt=6.54815)
T/S Cluster: L−6.5−1
L1056 (m/z=191.384475; rt=6.54751)
T/S Cluster: L−6.5−1
L1093 (m/z=233.190407; rt=6.54789)
T/S Cluster: L−6.5−1
L1048 (m/z=191.388002; rt=6.54717)
T/S Cluster: L−6.5−1
L1098 (m/z=382.748376; rt=6.54819)
T/S Cluster: L−6.5−1
L1100 (m/z=568.449131; rt=6.54903)
T/S Cluster: L−6.5−1
L1102 (m/z=568.439154; rt=6.54958)
T/S Cluster: L−6.5−1
L1077 (m/z=308.231118; rt=6.54767)
T/S Cluster: L−6.5−1
L1022 (m/z=752.527978; rt=6.52324)
T/S Cluster: L−6.5−2
PCo1
PCo2

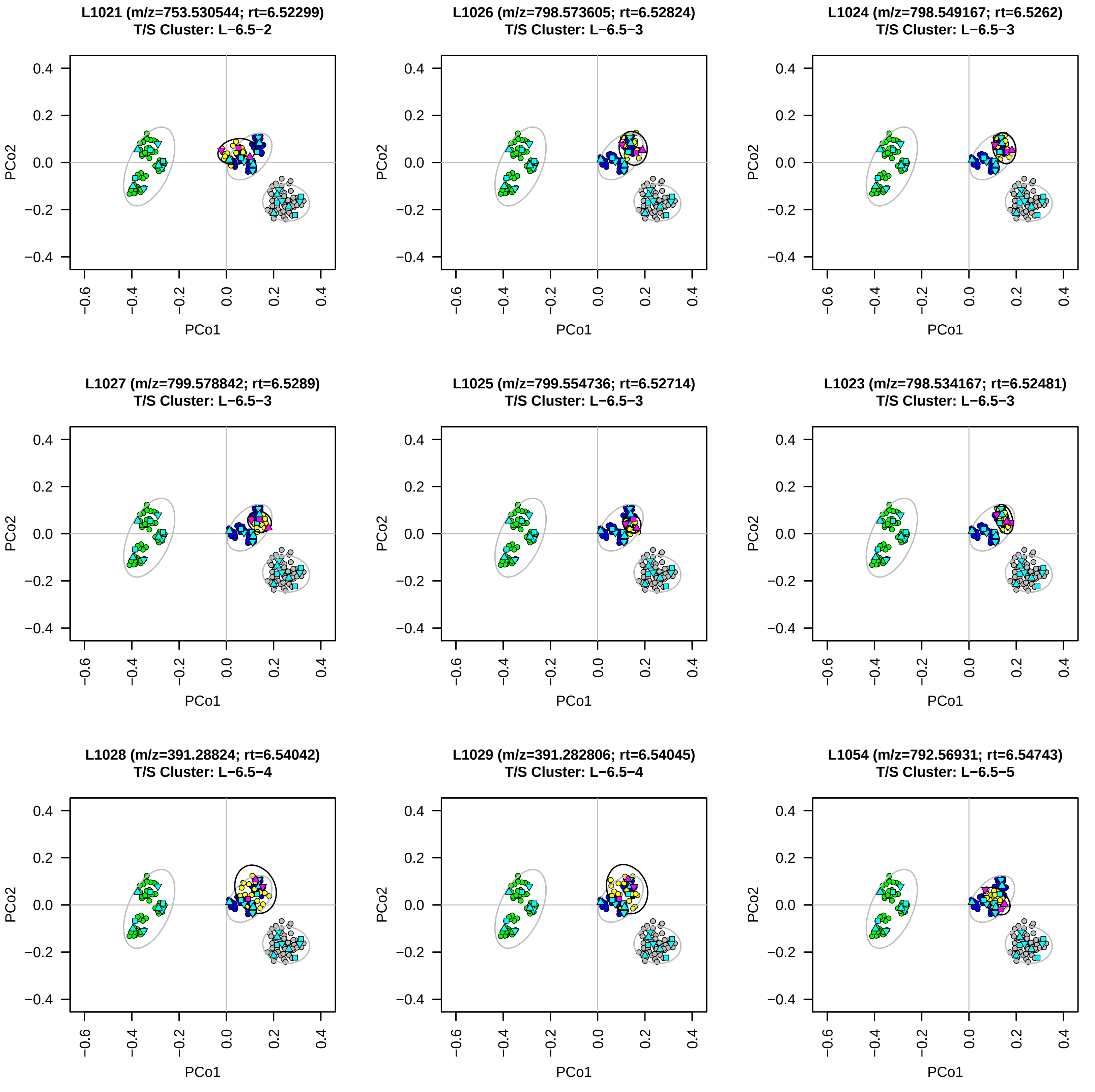

L1021 (m/z=753.530544; rt=6.52299)
T/S Cluster: L−6.5−2
L1026 (m/z=798.573605; rt=6.52824)
T/S Cluster: L−6.5−3
L1024 (m/z=798.549167; rt=6.5262)
T/S Cluster: L−6.5−3
L1027 (m/z=799.578842; rt=6.5289)
T/S Cluster: L−6.5−3
L1025 (m/z=799.554736; rt=6.52714)
T/S Cluster: L−6.5−3
L1023 (m/z=798.534167; rt=6.52481)
T/S Cluster: L−6.5−3
L1028 (m/z=391.28824; rt=6.54042)
T/S Cluster: L−6.5−4
L1029 (m/z=391.282806; rt=6.54045)
T/S Cluster: L−6.5−4
L1054 (m/z=792.56931; rt=6.54743)
T/S Cluster: L−6.5−5
PCo1
PCo2

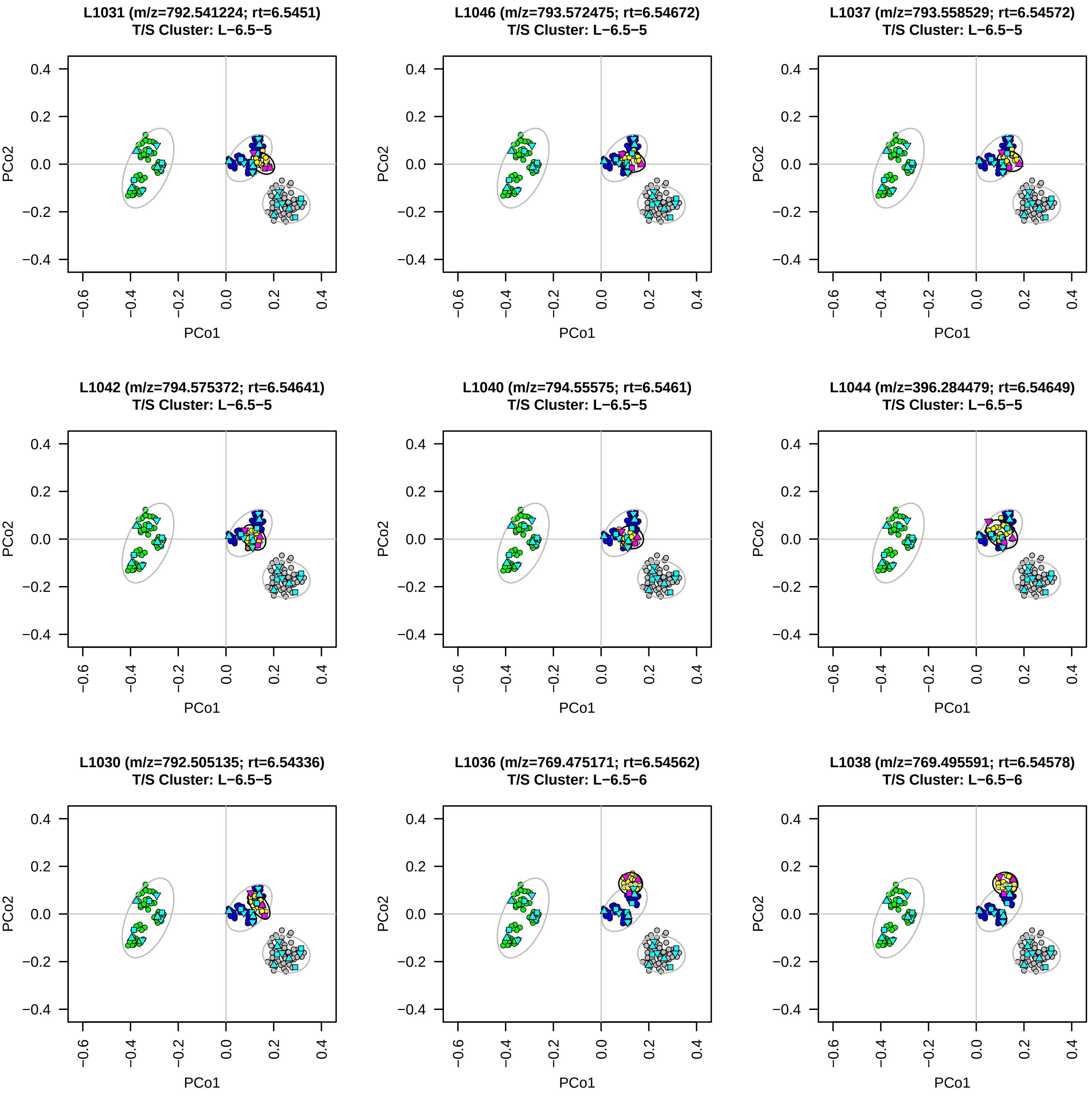
L1031 (m/z=792.541224; rt=6.5451)
T/S Cluster: L−6.5−5
L1046 (m/z=793.572475; rt=6.54672)
T/S Cluster: L−6.5−5
L1037 (m/z=793.558529; rt=6.54572)
T/S Cluster: L−6.5−5
L1042 (m/z=794.575372; rt=6.54641)
T/S Cluster: L−6.5−5
L1040 (m/z=794.55575; rt=6.5461)
T/S Cluster: L−6.5−5
L1044 (m/z=396.284479; rt=6.54649)
T/S Cluster: L−6.5−5
L1030 (m/z=792.505135; rt=6.54336)
T/S Cluster: L−6.5−5
L1036 (m/z=769.475171; rt=6.54562)
T/S Cluster: L−6.5−6
L1038 (m/z=769.495591; rt=6.54578)
T/S Cluster: L−6.5−6
PCo1
PCo2

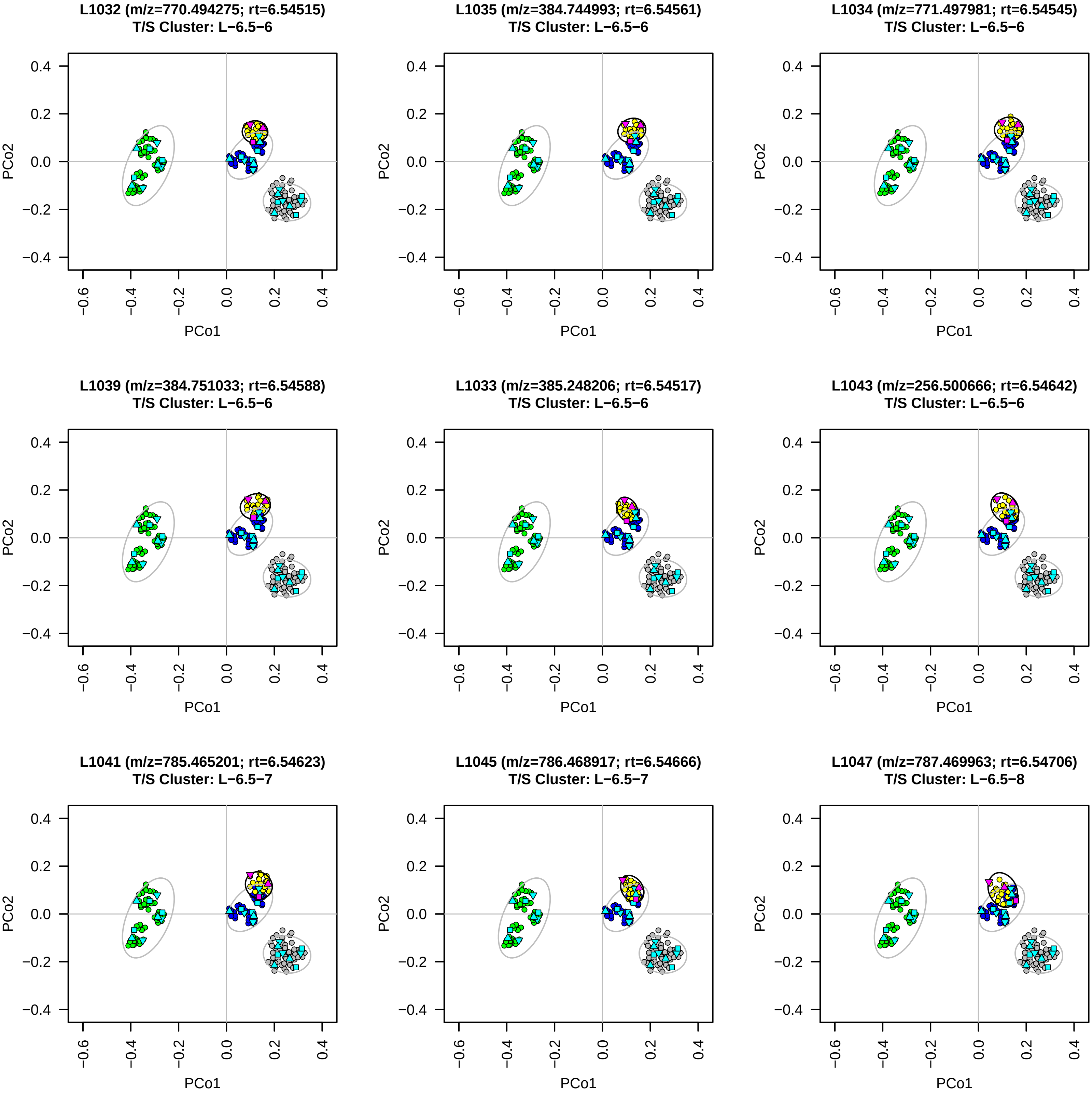
L1032 (m/z=770.494275; rt=6.54515)
T/S Cluster: L−6.5−6
L1035 (m/z=384.744993; rt=6.54561)
T/S Cluster: L−6.5−6
L1034 (m/z=771.497981; rt=6.54545)
T/S Cluster: L−6.5−6
L1039 (m/z=384.751033; rt=6.54588)
T/S Cluster: L−6.5−6
L1033 (m/z=385.248206; rt=6.54517)
T/S Cluster: L−6.5−6
L1043 (m/z=256.500666; rt=6.54642)
T/S Cluster: L−6.5−6
L1041 (m/z=785.465201; rt=6.54623)
T/S Cluster: L−6.5−7
L1045 (m/z=786.468917; rt=6.54666)
T/S Cluster: L−6.5−7
L1047 (m/z=787.469963; rt=6.54706)
T/S Cluster: L−6.5−8
PCo1
PCo2
−0.6
−0.4
−0.2
0.0
0.2
0.4

L1059 (m/z=806.561778; rt=6.54755)
T/S Cluster: L−6.5−9

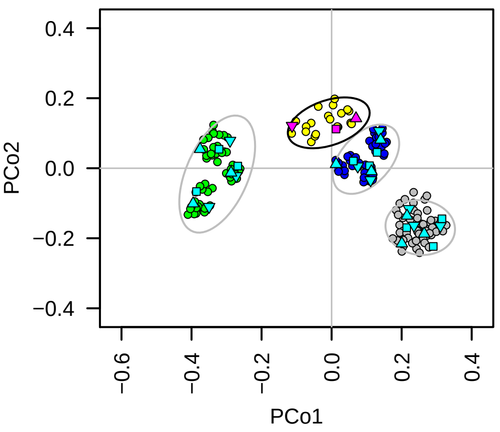

L1063 (m/z=806.561732; rt=6.54756)
T/S Cluster: L−6.5−9

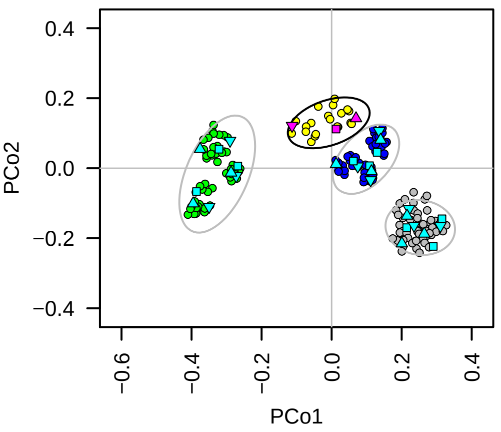

L1099 (m/z=805.563214; rt=6.549)
T/S Cluster: L−6.5−9

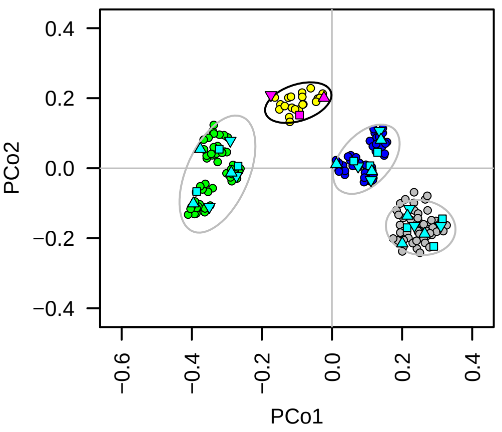

L1101 (m/z=806.526737; rt=6.54913)
T/S Cluster: L−6.5−10

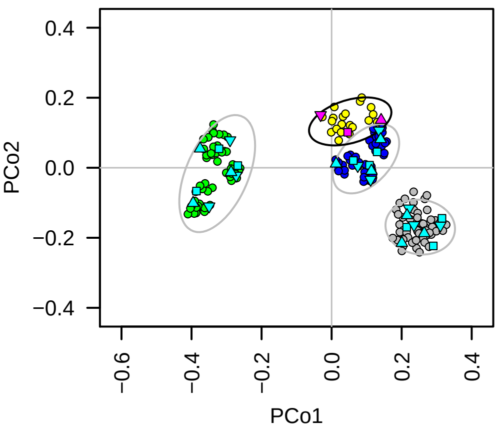

L1103 (m/z=772.511095; rt=6.55532)
T/S Cluster: L−6.6−1

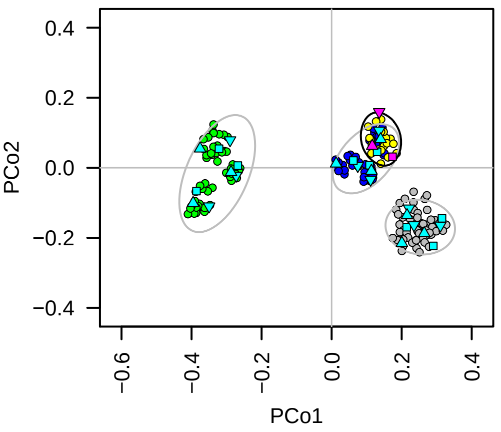

L1104 (m/z=437.745075; rt=6.56583)
T/S Cluster: L−6.6−2

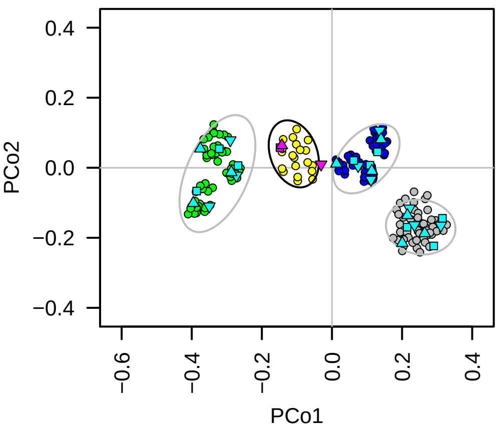

L1105 (m/z=388.341144; rt=6.56646)
T/S Cluster: L−6.6−3

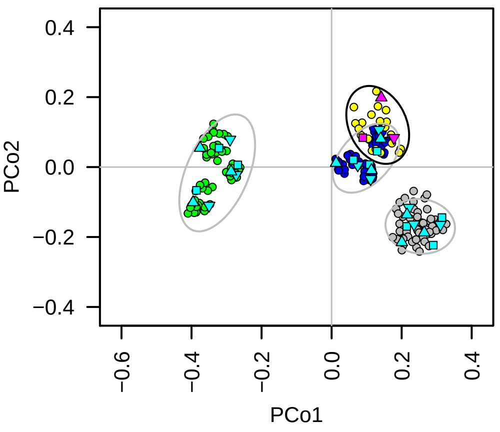

L1107 (m/z=388.345121; rt=6.56953)
T/S Cluster: L−6.6−3

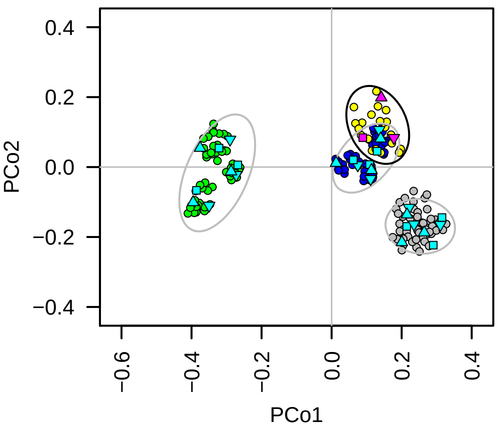

L1106 (m/z=800.587099; rt=6.56825)
T/S Cluster: L−6.6−4

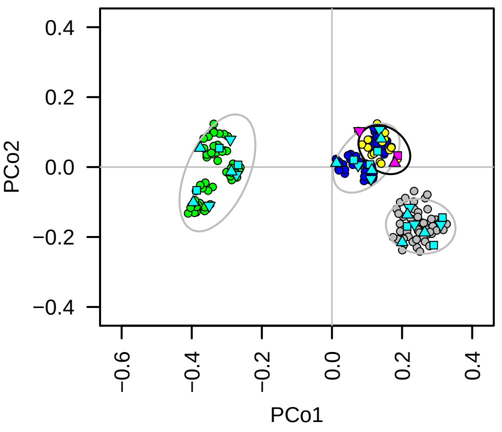

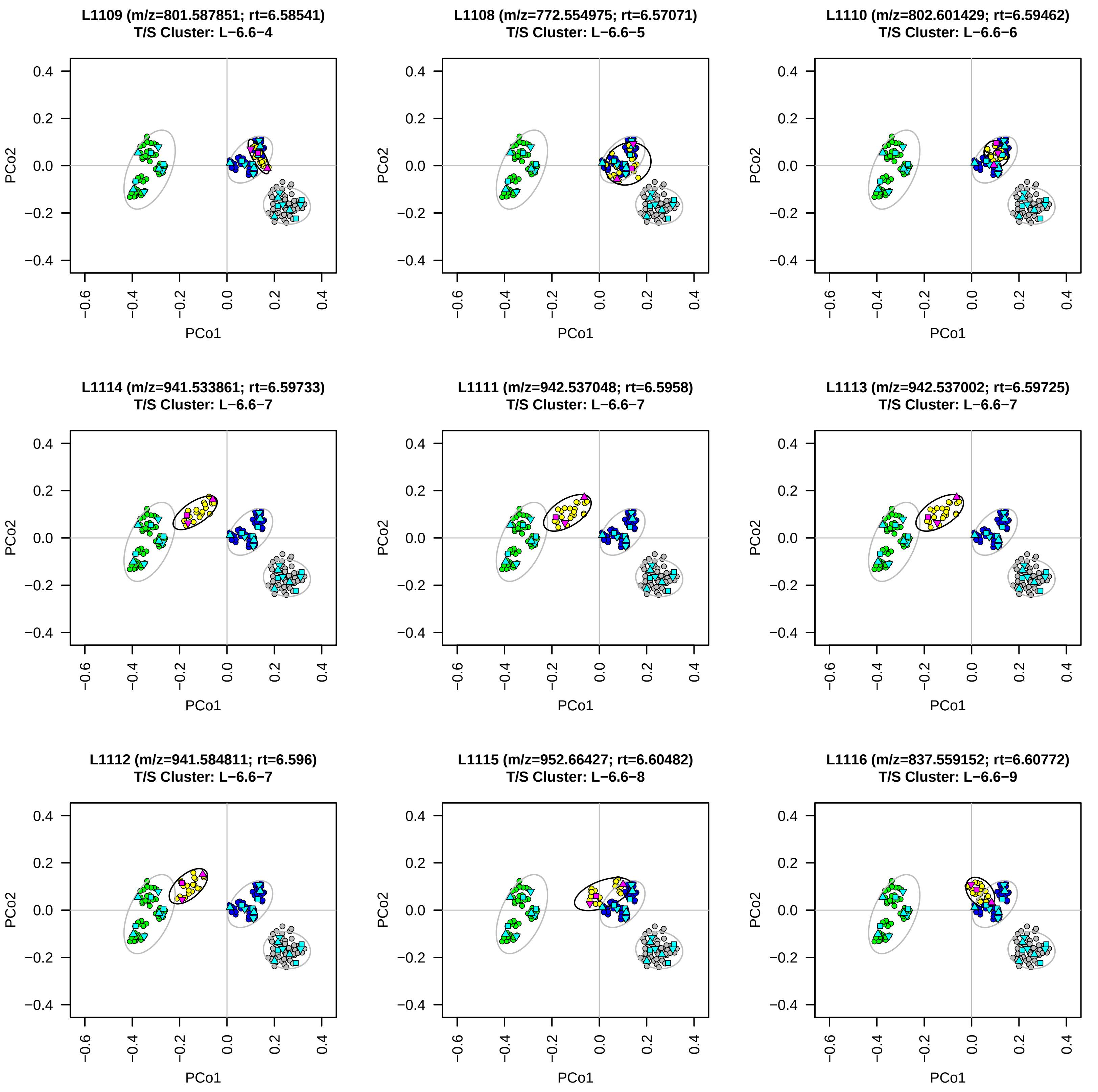
L1109 (m/z=801.587851; rt=6.58541)
T/S Cluster: L−6.6−4
L1108 (m/z=772.554975; rt=6.57071)
T/S Cluster: L−6.6−5
L1110 (m/z=802.601429; rt=6.59462)
T/S Cluster: L−6.6−6
L1114 (m/z=941.533861; rt=6.59733)
T/S Cluster: L−6.6−7
L1111 (m/z=942.537048; rt=6.5958)
T/S Cluster: L−6.6−7
L1113 (m/z=942.537002; rt=6.59725)
T/S Cluster: L−6.6−7
L1112 (m/z=941.584811; rt=6.596)
T/S Cluster: L−6.6−7
L1115 (m/z=952.66427; rt=6.60482)
T/S Cluster: L−6.6−8
L1116 (m/z=837.559152; rt=6.60772)
T/S Cluster: L−6.6−9
PCo1
PCo2

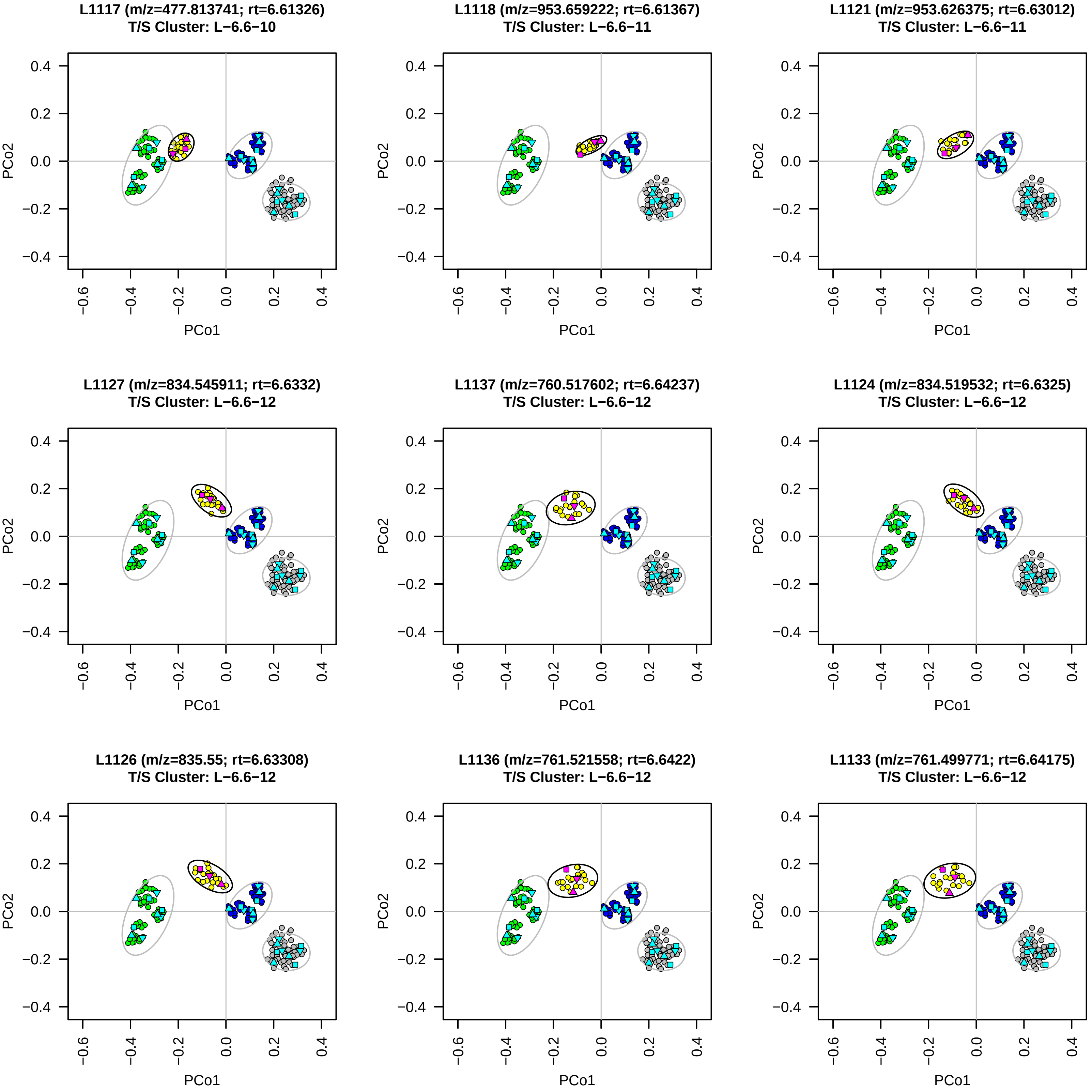
L1117 (m/z=477.813741; rt=6.61326)
T/S Cluster: L−6.6−10
L1118 (m/z=953.659222; rt=6.61367)
T/S Cluster: L−6.6−11
L1121 (m/z=953.626375; rt=6.63012)
T/S Cluster: L−6.6−11
L1127 (m/z=834.545911; rt=6.6332)
T/S Cluster: L−6.6−12
L1137 (m/z=760.517602; rt=6.64237)
T/S Cluster: L−6.6−12
L1124 (m/z=834.519532; rt=6.6325)
T/S Cluster: L−6.6−12
L1126 (m/z=835.55; rt=6.63308)
T/S Cluster: L−6.6−12
L1136 (m/z=761.521558; rt=6.6422)
T/S Cluster: L−6.6−12
L1133 (m/z=761.499771; rt=6.64175)
T/S Cluster: L−6.6−12
PCo1
PCo2
−0.6 −0.4 −0.2 0.0 0.2 0.4
−0.4 −0.2 0.0 0.2 0.4

L1123 (m/z=835.517989; rt=6.63203)
T/S Cluster: L−6.6−12

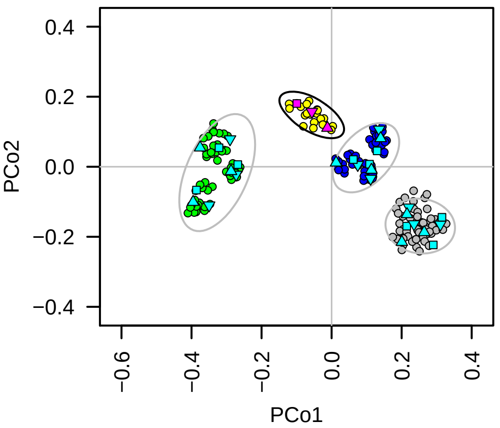

L1134 (m/z=760.474494; rt=6.64186)
T/S Cluster: L−6.6−12

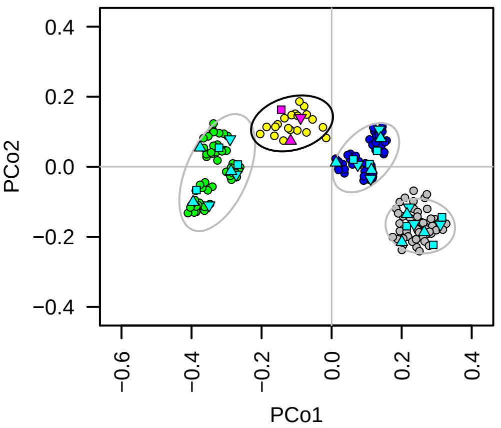

L1119 (m/z=836.552356; rt=6.62414)
T/S Cluster: L−6.6−12

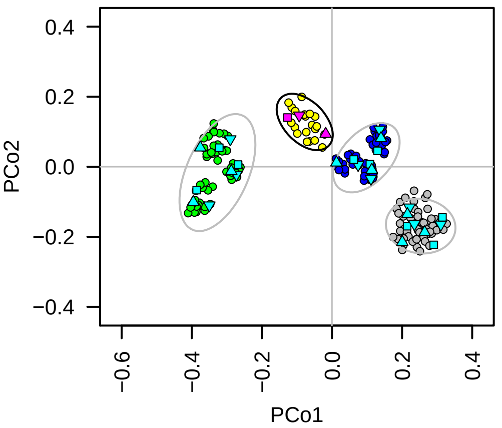

L1135 (m/z=380.258142; rt=6.64209)
T/S Cluster: L−6.6−12

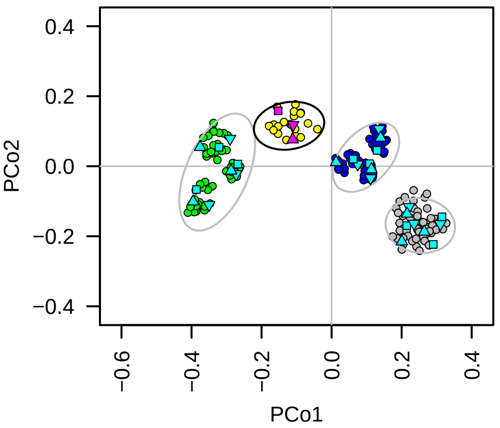

L1138 (m/z=380.261631; rt=6.64252)
T/S Cluster: L−6.6−12

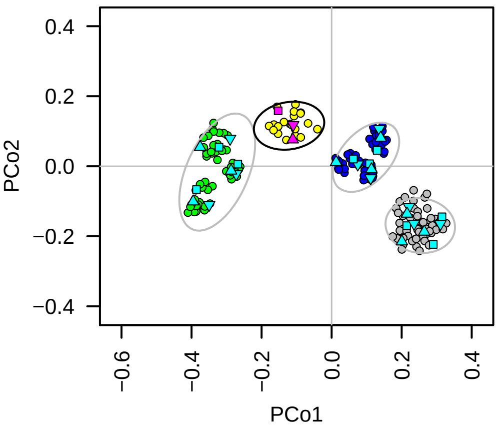

L1131 (m/z=762.525136; rt=6.64125)
T/S Cluster: L−6.6−12

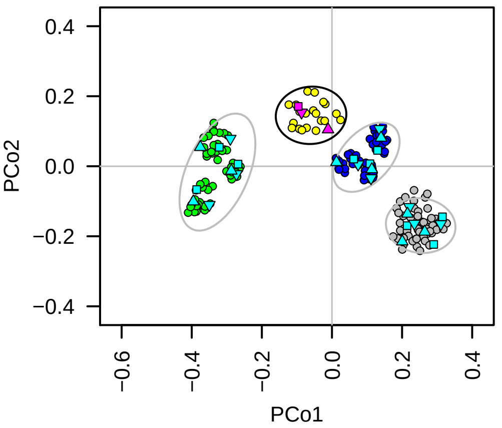

L1122 (m/z=417.272226; rt=6.63172)
T/S Cluster: L−6.6−12

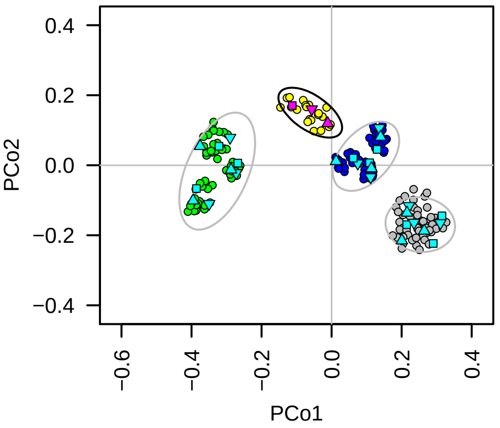

L1128 (m/z=417.274093; rt=6.64018)
T/S Cluster: L−6.6−12

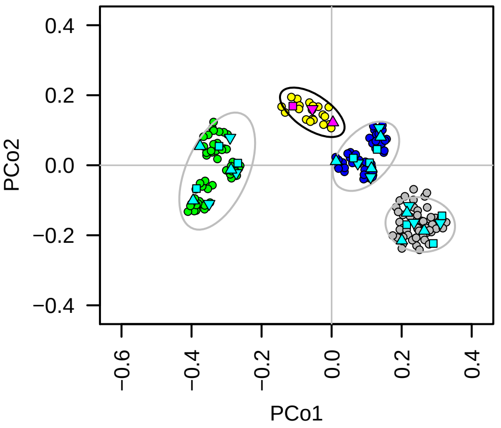

L1125 (m/z=417.278674; rt=6.63271)
T/S Cluster: L−6.6−12

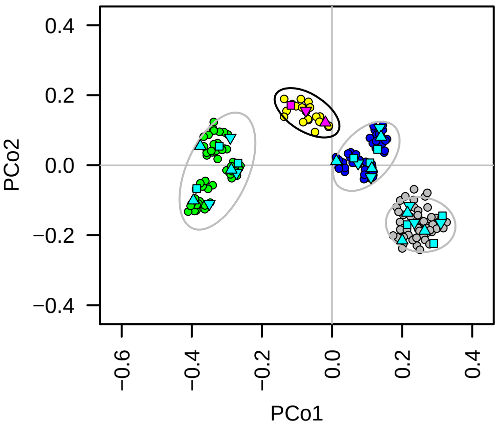

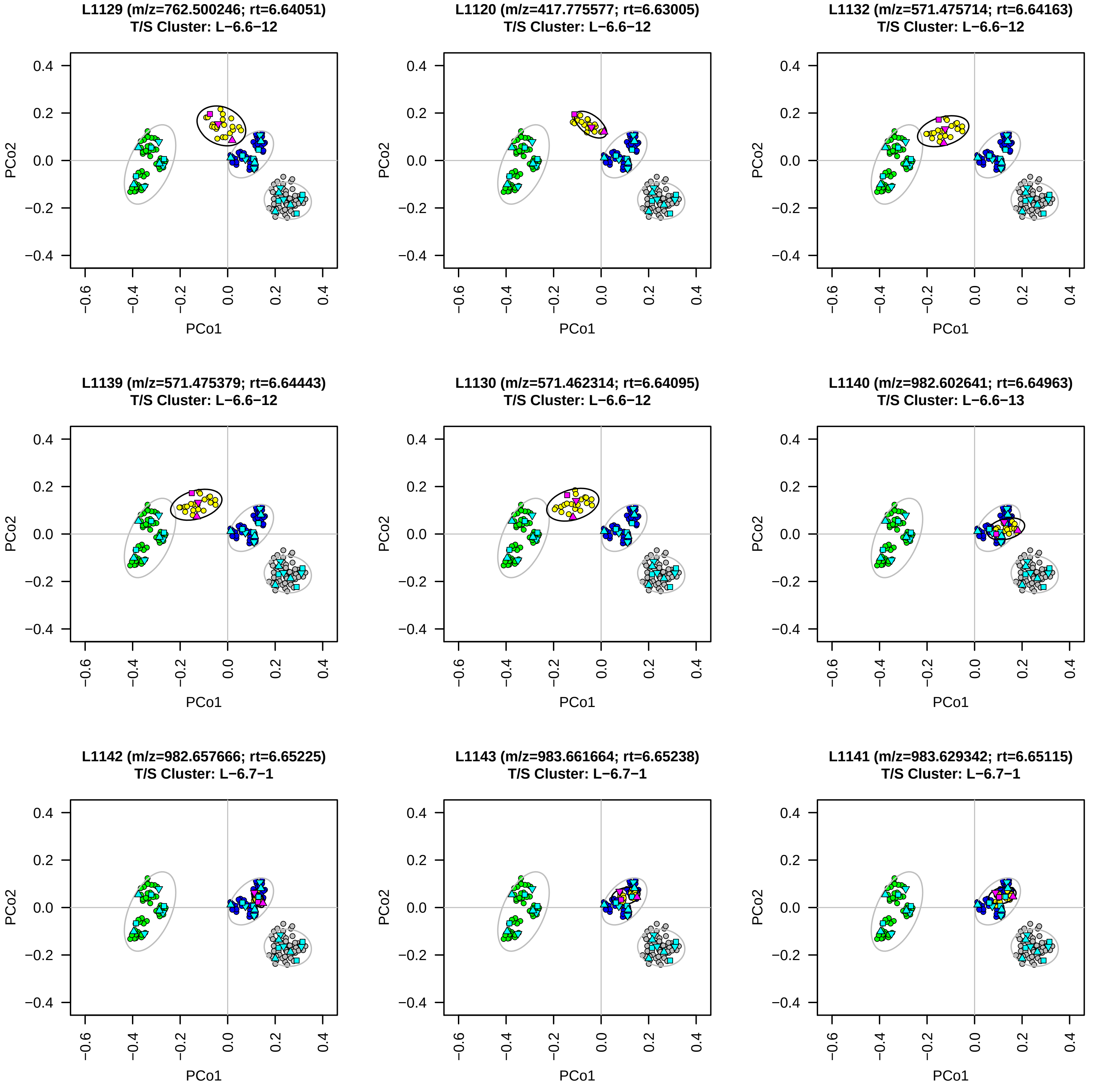
L1129 (m/z=762.500246; rt=6.64051)
T/S Cluster: L−6.6−12
L1120 (m/z=417.775577; rt=6.63005)
T/S Cluster: L−6.6−12
L1132 (m/z=571.475714; rt=6.64163)
T/S Cluster: L−6.6−12
L1139 (m/z=571.475379; rt=6.64443)
T/S Cluster: L−6.6−12
L1130 (m/z=571.462314; rt=6.64095)
T/S Cluster: L−6.6−12
L1140 (m/z=982.602641; rt=6.64963)
T/S Cluster: L−6.6−13
L1142 (m/z=982.657666; rt=6.65225)
T/S Cluster: L−6.7−1
L1143 (m/z=983.661664; rt=6.65238)
T/S Cluster: L−6.7−1
L1141 (m/z=983.629342; rt=6.65115)
T/S Cluster: L−6.7−1
PCo1
PCo2
−0.6
−0.4
−0.2
0.0
0.2
0.4

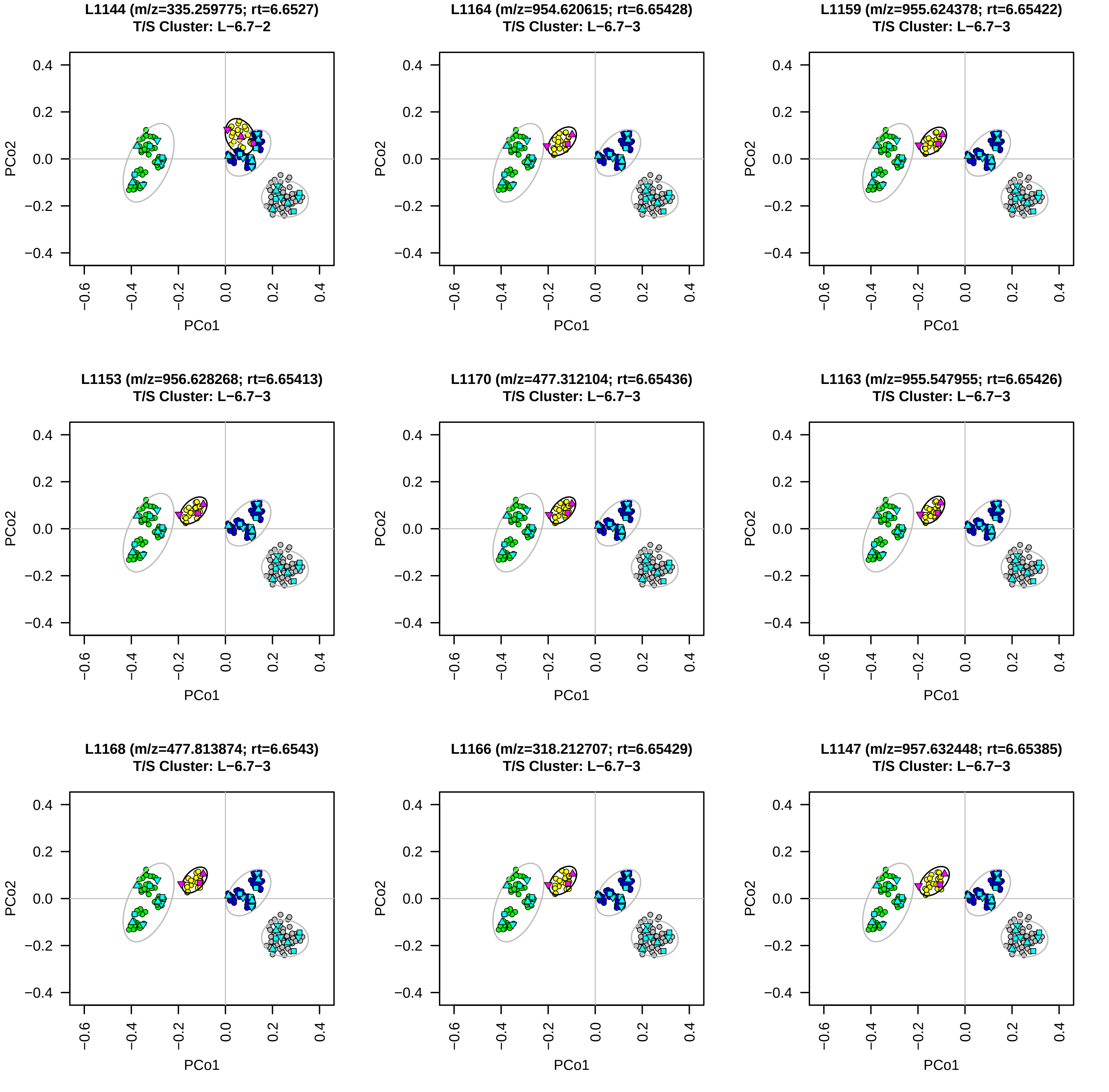
L1144 (m/z=335.259775; rt=6.6527)
T/S Cluster: L−6.7−2
L1164 (m/z=954.620615; rt=6.65428)
T/S Cluster: L−6.7−3
L1159 (m/z=955.624378; rt=6.65422)
T/S Cluster: L−6.7−3
L1153 (m/z=956.628268; rt=6.65413)
T/S Cluster: L−6.7−3
L1170 (m/z=477.312104; rt=6.65436)
T/S Cluster: L−6.7−3
L1163 (m/z=955.547955; rt=6.65426)
T/S Cluster: L−6.7−3
L1168 (m/z=477.813874; rt=6.6543)
T/S Cluster: L−6.7−3
L1166 (m/z=318.212707; rt=6.65429)
T/S Cluster: L−6.7−3
L1147 (m/z=957.632448; rt=6.65385)
T/S Cluster: L−6.7−3
PCo1
PCo2
−0.6
−0.4
−0.2
0.0
0.2
0.4

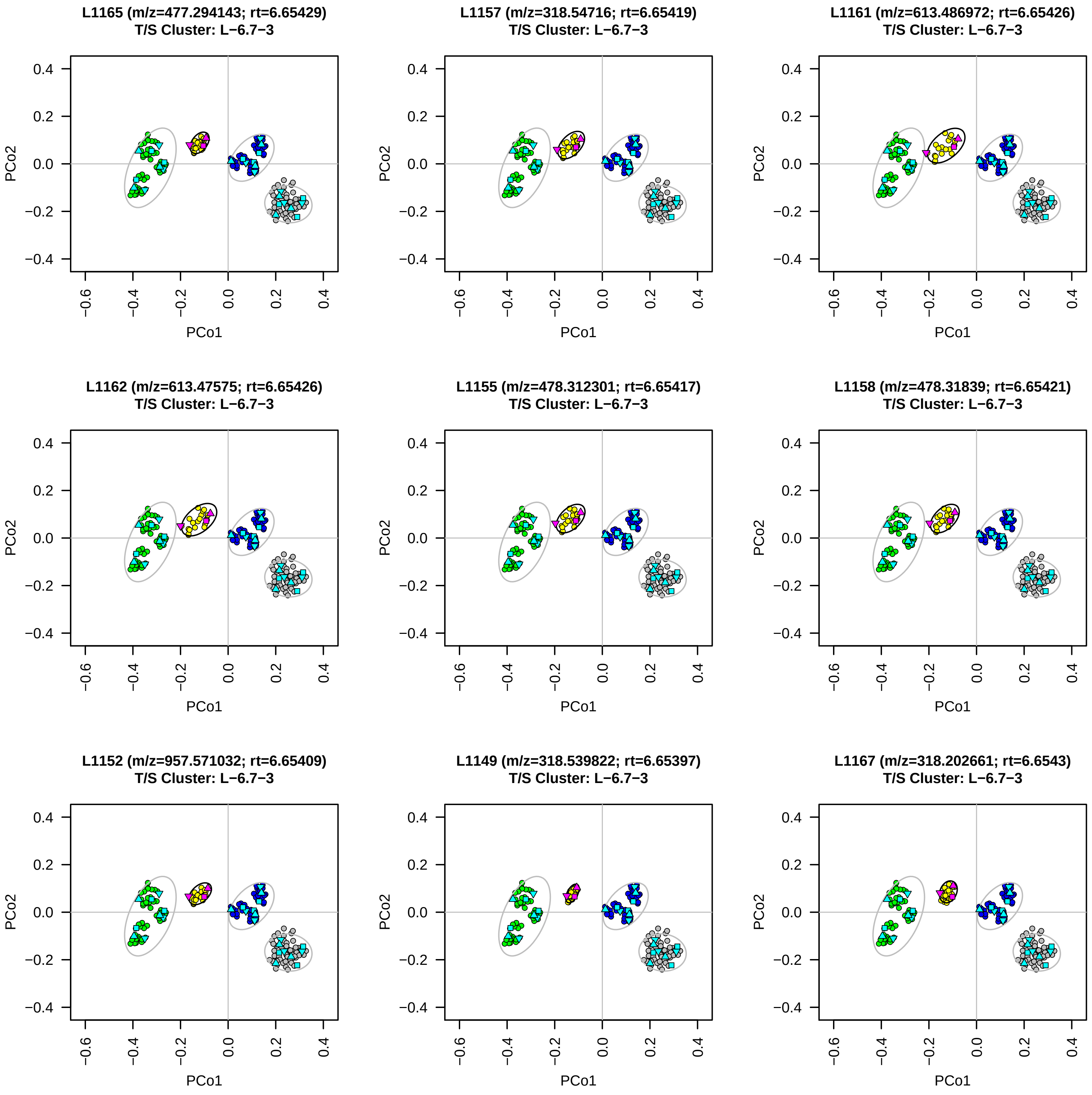
L1165 (m/z=477.294143; rt=6.65429)
T/S Cluster: L−6.7−3
L1157 (m/z=318.54716; rt=6.65419)
T/S Cluster: L−6.7−3
L1161 (m/z=613.486972; rt=6.65426)
T/S Cluster: L−6.7−3
L1162 (m/z=613.47575; rt=6.65426)
T/S Cluster: L−6.7−3
L1155 (m/z=478.312301; rt=6.65417)
T/S Cluster: L−6.7−3
L1158 (m/z=478.31839; rt=6.65421)
T/S Cluster: L−6.7−3
L1152 (m/z=957.571032; rt=6.65409)
T/S Cluster: L−6.7−3
L1149 (m/z=318.539822; rt=6.65397)
T/S Cluster: L−6.7−3
L1167 (m/z=318.202661; rt=6.6543)
T/S Cluster: L−6.7−3
PCo1
PCo2
0.4
0.2
0.0
−0.2
−0.4
−0.6

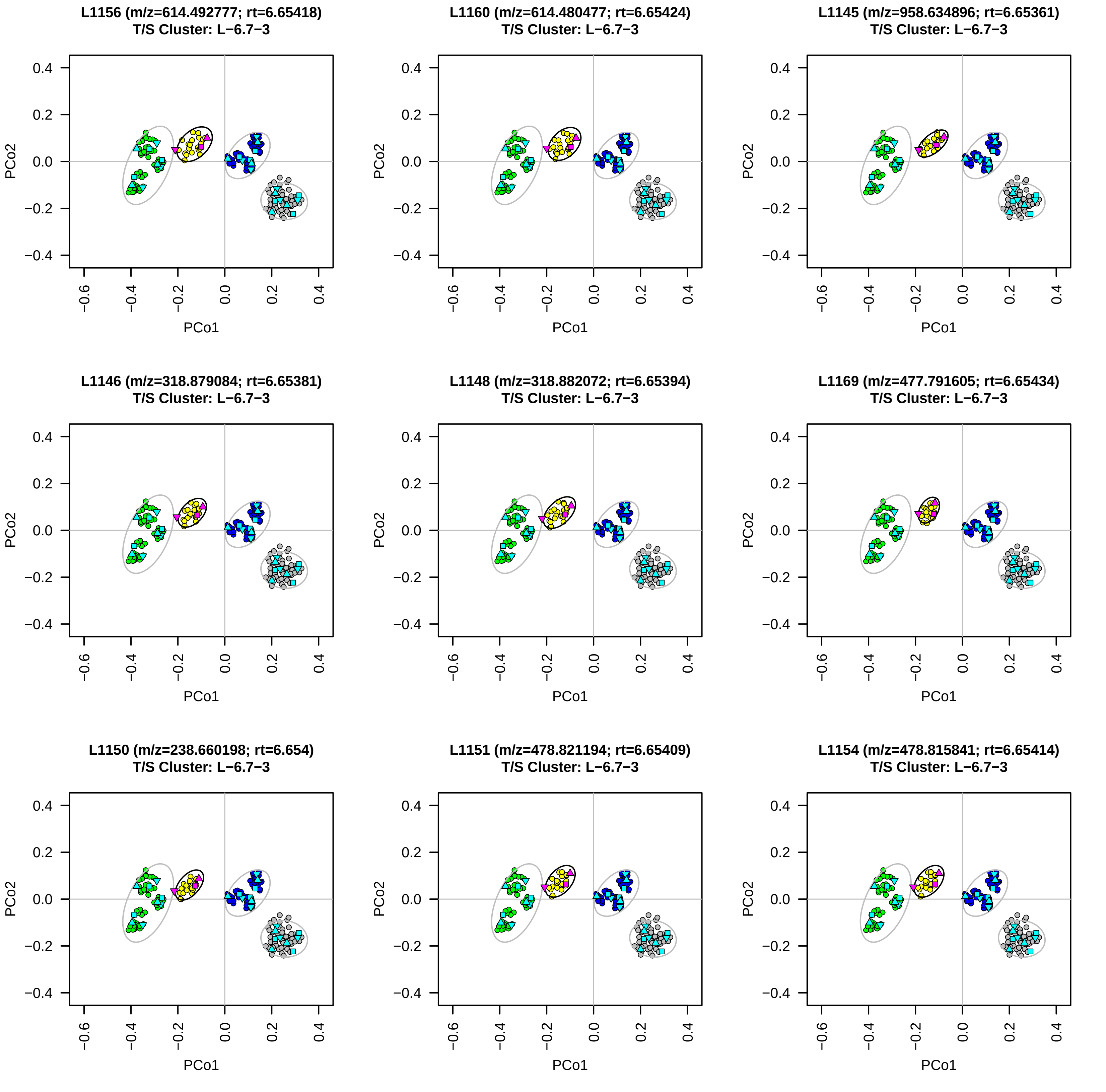
L1156 (m/z=614.492777; rt=6.65418)
T/S Cluster: L−6.7−3
L1160 (m/z=614.480477; rt=6.65424)
T/S Cluster: L−6.7−3
L1145 (m/z=958.634896; rt=6.65361)
T/S Cluster: L−6.7−3
L1146 (m/z=318.879084; rt=6.65381)
T/S Cluster: L−6.7−3
L1148 (m/z=318.882072; rt=6.65394)
T/S Cluster: L−6.7−3
L1169 (m/z=477.791605; rt=6.65434)
T/S Cluster: L−6.7−3
L1150 (m/z=238.660198; rt=6.654)
T/S Cluster: L−6.7−3
L1151 (m/z=478.821194; rt=6.65409)
T/S Cluster: L−6.7−3
L1154 (m/z=478.815841; rt=6.65414)
T/S Cluster: L−6.7−3
PCo1
PCo2
0.4
0.2
0.0
−0.2
−0.4
−0.6

L1171 (m/z=238.655423; rt=6.65462)
T/S Cluster: L−6.7−3

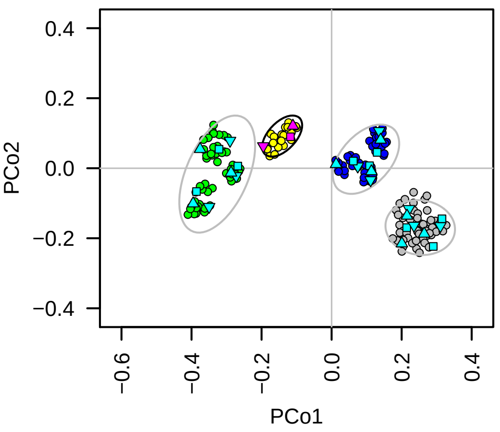

L1203 (m/z=800.525016; rt=6.6748)
T/S Cluster: L−6.7−4

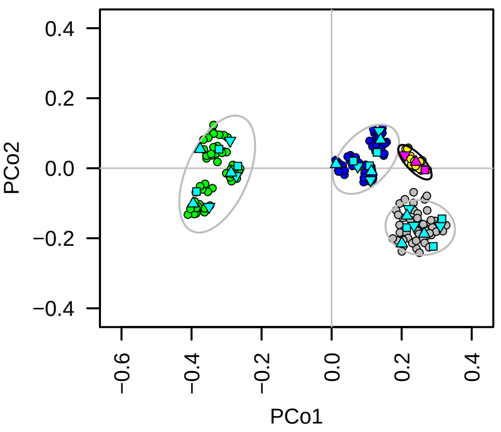

L1172 (m/z=801.537908; rt=6.65826)
T/S Cluster: L−6.7−4

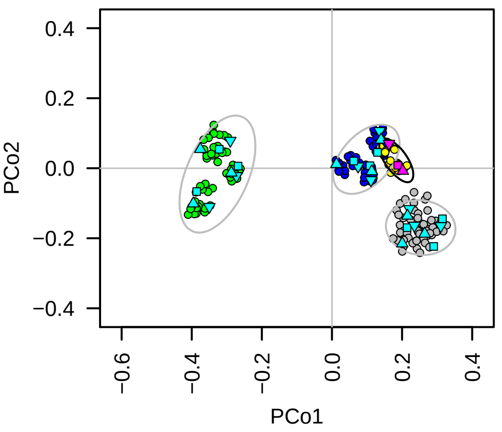

L1173 (m/z=391.288442; rt=6.65877)
T/S Cluster: L−6.7−5

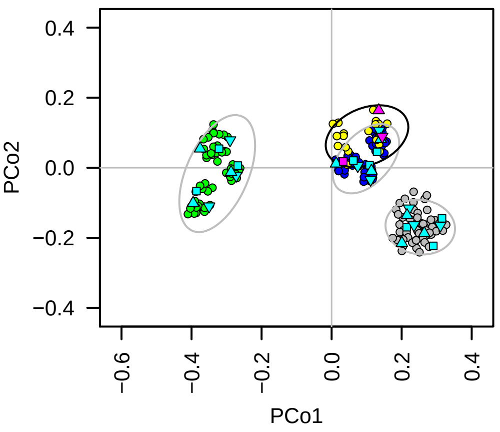

L1174 (m/z=391.283388; rt=6.65946)
T/S Cluster: L−6.7−5

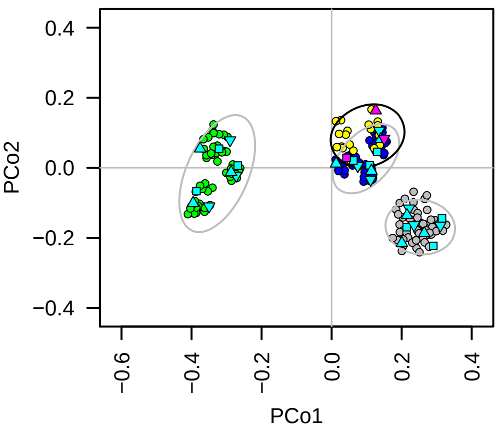

L1177 (m/z=959.582032; rt=6.66037)
T/S Cluster: L−6.7−6

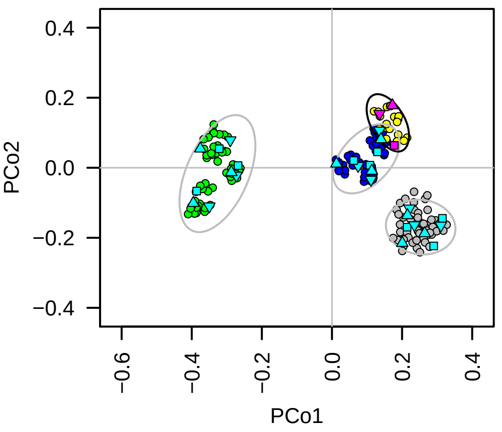

L1180 (m/z=959.563178; rt=6.66078)
T/S Cluster: L−6.7−6

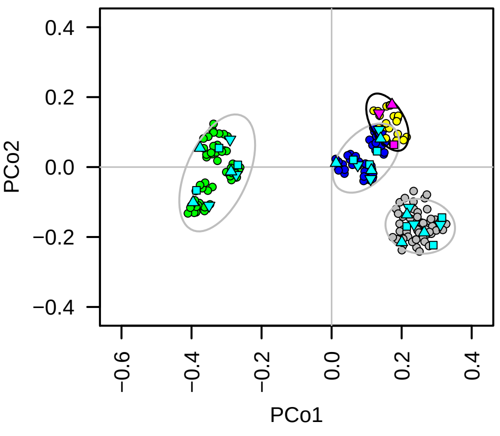

L1175 (m/z=960.585945; rt=6.65986)
T/S Cluster: L−6.7−6

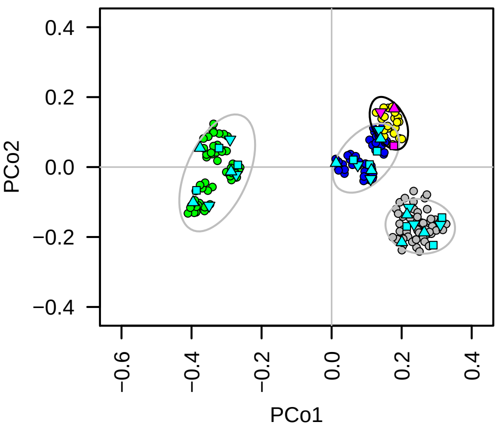

L1176 (m/z=960.567523; rt=6.66019)
T/S Cluster: L−6.7−6

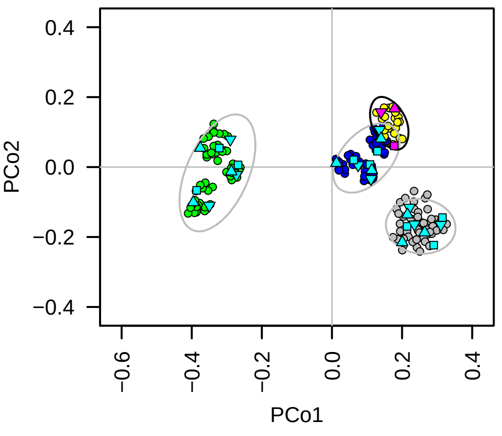

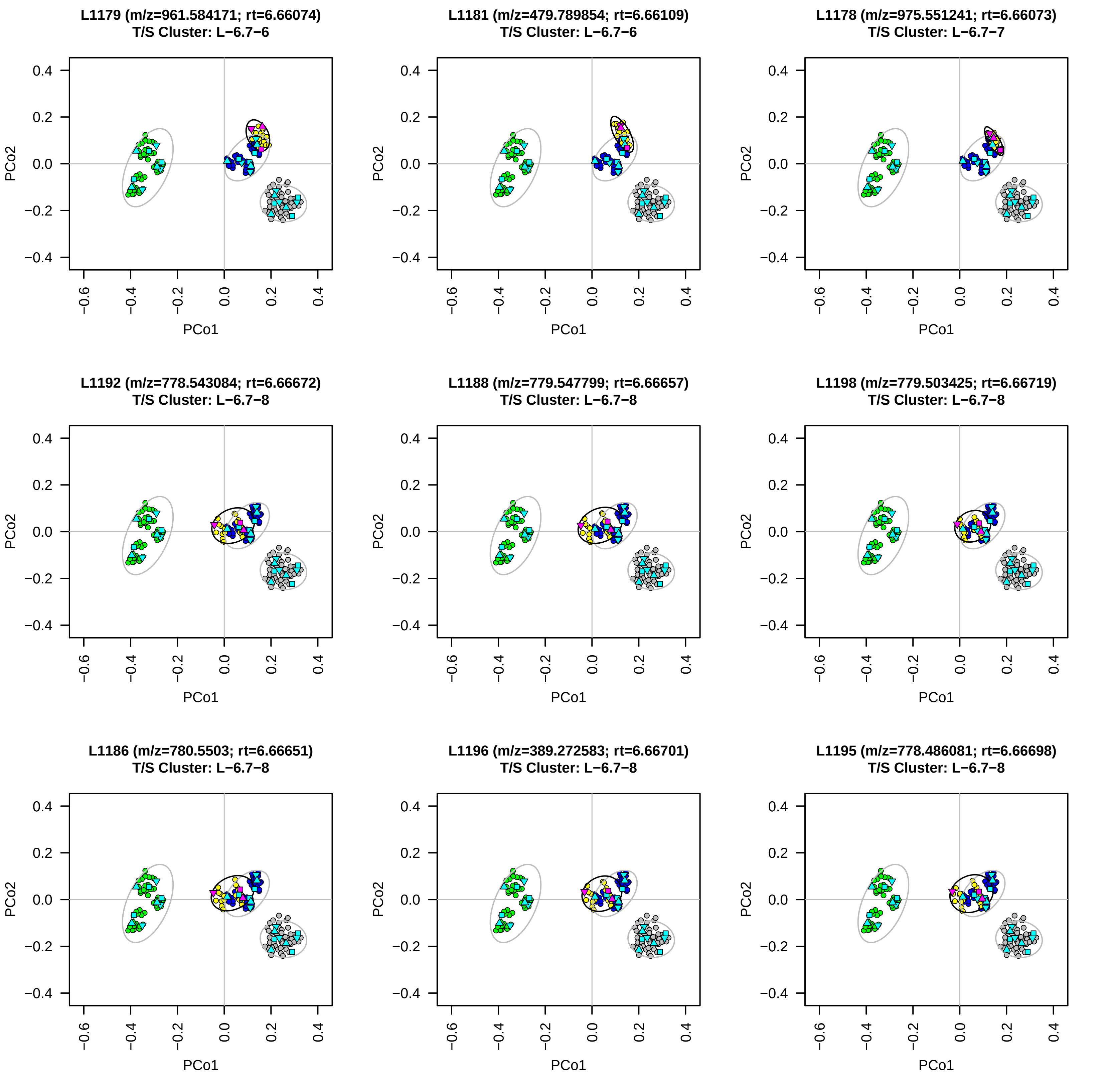
L1179 (m/z=961.584171; rt=6.66074)
T/S Cluster: L−6.7−6
L1181 (m/z=479.789854; rt=6.66109)
T/S Cluster: L−6.7−6
L1178 (m/z=975.551241; rt=6.66073)
T/S Cluster: L−6.7−7
L1192 (m/z=778.543084; rt=6.66672)
T/S Cluster: L−6.7−8
L1188 (m/z=779.547799; rt=6.66657)
T/S Cluster: L−6.7−8
L1198 (m/z=779.503425; rt=6.66719)
T/S Cluster: L−6.7−8
L1186 (m/z=780.5503; rt=6.66651)
T/S Cluster: L−6.7−8
L1196 (m/z=389.272583; rt=6.66701)
T/S Cluster: L−6.7−8
L1195 (m/z=778.486081; rt=6.66698)
T/S Cluster: L−6.7−8
PCo1
PCo2
0.4
0.2
0.0
−0.2
−0.4
−0.6

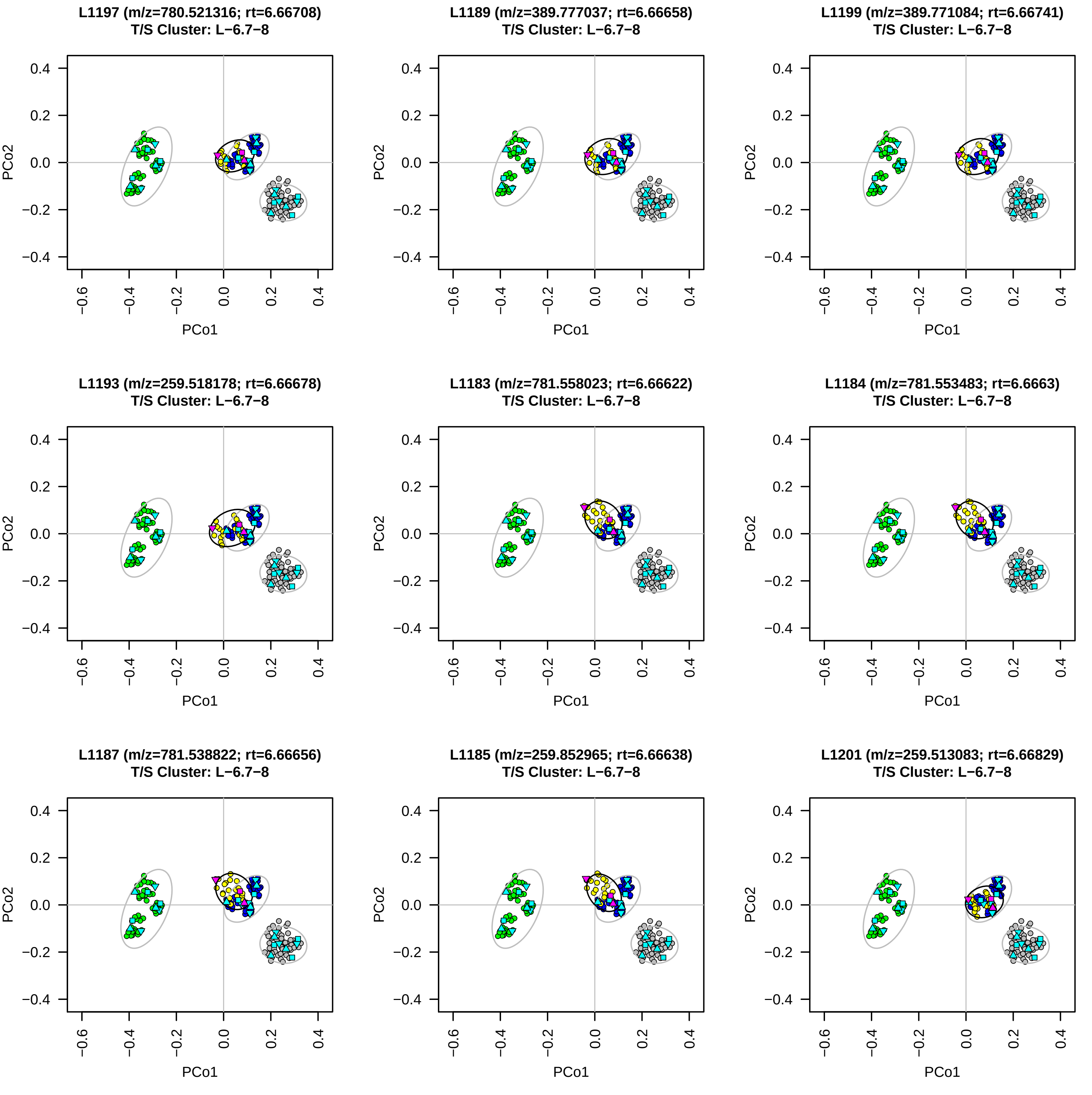
L1197 (m/z=780.521316; rt=6.66708)
T/S Cluster: L−6.7−8
L1189 (m/z=389.777037; rt=6.66658)
T/S Cluster: L−6.7−8
L1199 (m/z=389.771084; rt=6.66741)
T/S Cluster: L−6.7−8
L1193 (m/z=259.518178; rt=6.66678)
T/S Cluster: L−6.7−8
L1183 (m/z=781.558023; rt=6.66622)
T/S Cluster: L−6.7−8
L1184 (m/z=781.553483; rt=6.6663)
T/S Cluster: L−6.7−8
L1187 (m/z=781.538822; rt=6.66656)
T/S Cluster: L−6.7−8
L1185 (m/z=259.852965; rt=6.66638)
T/S Cluster: L−6.7−8
L1201 (m/z=259.513083; rt=6.66829)
T/S Cluster: L−6.7−8
PCo1
PCo2
−0.6
−0.4
−0.2
0.0
0.2
0.4

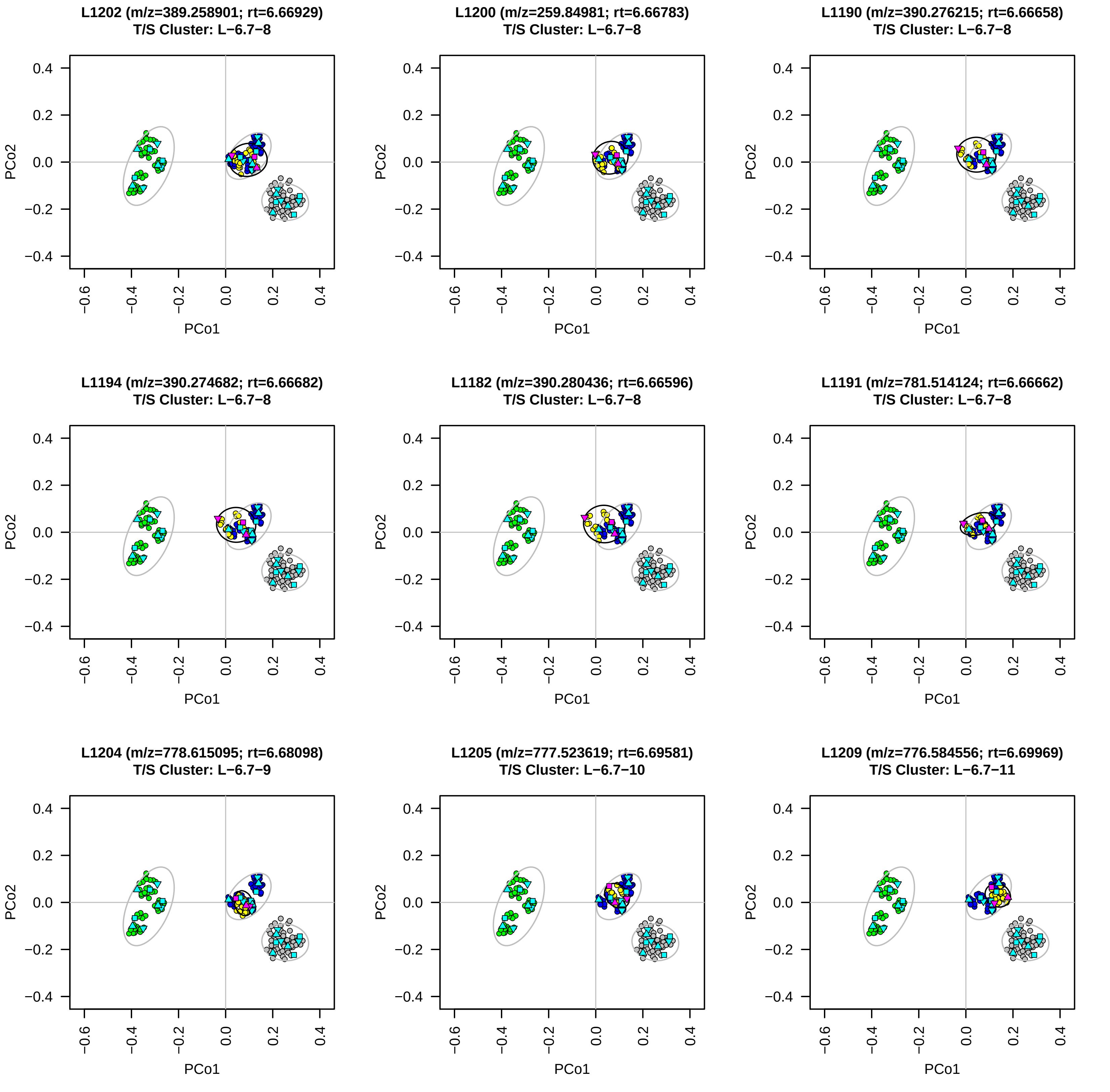
L1202 (m/z=389.258901; rt=6.66929)
T/S Cluster: L−6.7−8
L1200 (m/z=259.84981; rt=6.66783)
T/S Cluster: L−6.7−8
L1190 (m/z=390.276215; rt=6.66658)
T/S Cluster: L−6.7−8
L1194 (m/z=390.274682; rt=6.66682)
T/S Cluster: L−6.7−8
L1182 (m/z=390.280436; rt=6.66596)
T/S Cluster: L−6.7−8
L1191 (m/z=781.514124; rt=6.66662)
T/S Cluster: L−6.7−8
L1204 (m/z=778.615095; rt=6.68098)
T/S Cluster: L−6.7−9
L1205 (m/z=777.523619; rt=6.69581)
T/S Cluster: L−6.7−10
L1209 (m/z=776.584556; rt=6.69969)
T/S Cluster: L−6.7−11
PCo1
PCo2
−0.6
−0.4
−0.2
0.0
0.2
0.4

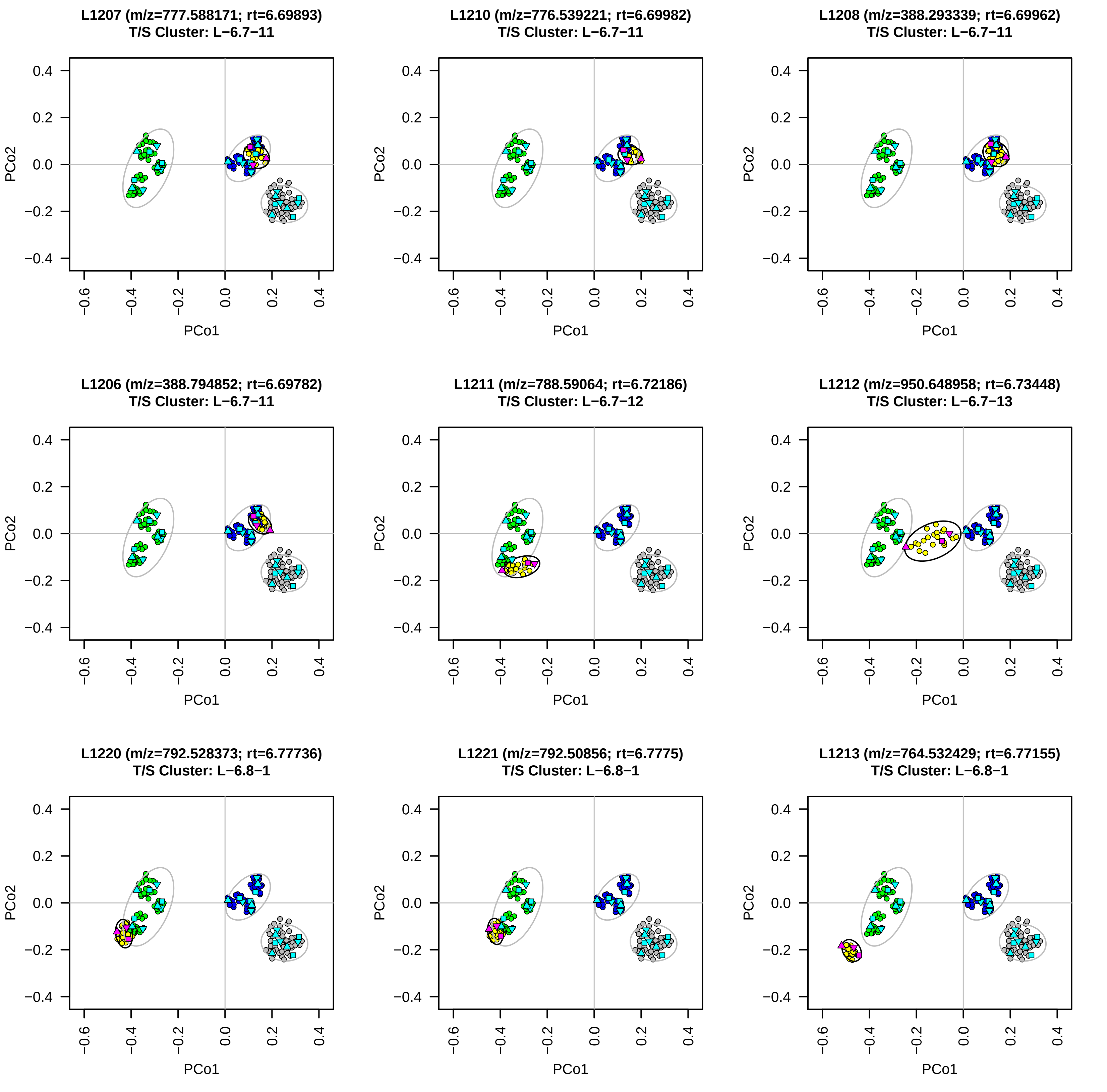

L1207 (m/z=777.588171; rt=6.69893)
T/S Cluster: L−6.7−11
L1210 (m/z=776.539221; rt=6.69982)
T/S Cluster: L−6.7−11
L1208 (m/z=388.293339; rt=6.69962)
T/S Cluster: L−6.7−11
L1206 (m/z=388.794852; rt=6.69782)
T/S Cluster: L−6.7−11
L1211 (m/z=788.59064; rt=6.72186)
T/S Cluster: L−6.7−12
L1212 (m/z=950.648958; rt=6.73448)
T/S Cluster: L−6.7−13
L1220 (m/z=792.528373; rt=6.77736)
T/S Cluster: L−6.8−1
L1221 (m/z=792.50856; rt=6.7775)
T/S Cluster: L−6.8−1
L1213 (m/z=764.532429; rt=6.77155)
T/S Cluster: L−6.8−1
PCo1
PCo2

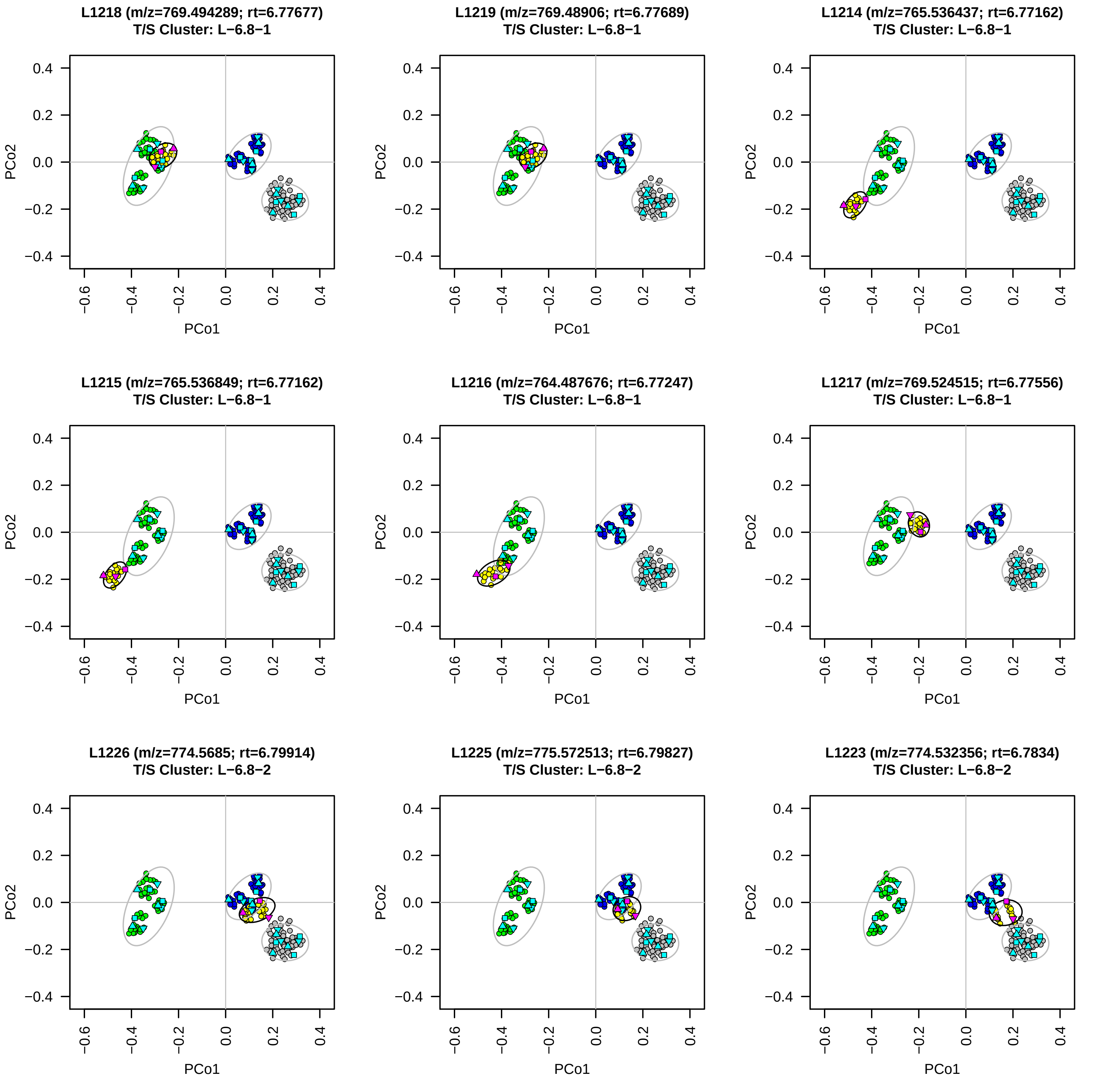
L1218 (m/z=769.494289; rt=6.77677)
T/S Cluster: L−6.8−1
L1219 (m/z=769.48906; rt=6.77689)
T/S Cluster: L−6.8−1
L1214 (m/z=765.536437; rt=6.77162)
T/S Cluster: L−6.8−1
L1215 (m/z=765.536849; rt=6.77162)
T/S Cluster: L−6.8−1
L1216 (m/z=764.487676; rt=6.77247)
T/S Cluster: L−6.8−1
L1217 (m/z=769.524515; rt=6.77556)
T/S Cluster: L−6.8−1
L1226 (m/z=774.5685; rt=6.79914)
T/S Cluster: L−6.8−2
L1225 (m/z=775.572513; rt=6.79827)
T/S Cluster: L−6.8−2
L1223 (m/z=774.532356; rt=6.7834)
T/S Cluster: L−6.8−2
PCo1
PCo2
0.4
0.2
0.0
−0.2
−0.4
−0.6

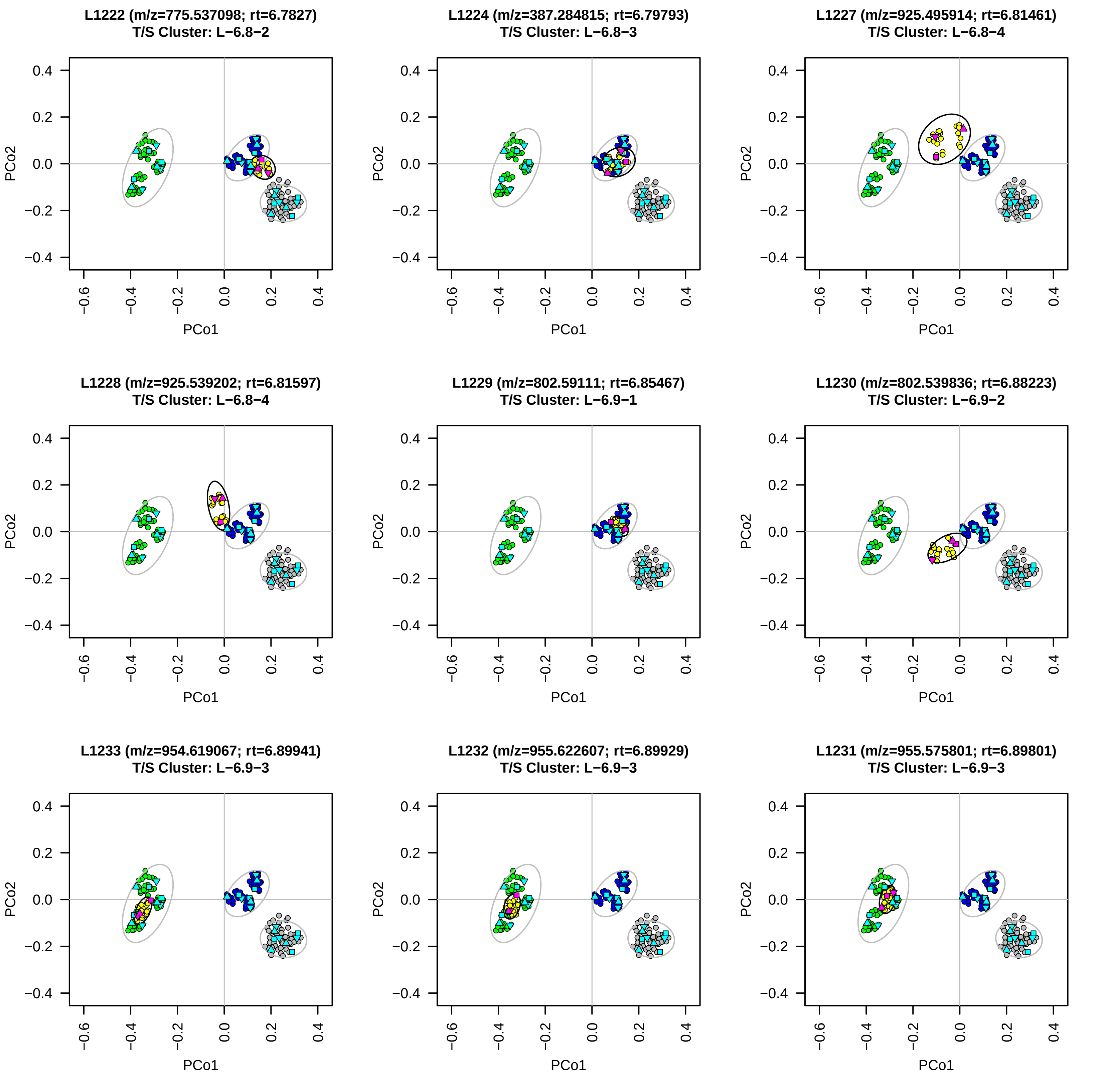
L1222 (m/z=775.537098; rt=6.7827)
T/S Cluster: L−6.8−2
L1224 (m/z=387.284815; rt=6.79793)
T/S Cluster: L−6.8−3
L1227 (m/z=925.495914; rt=6.81461)
T/S Cluster: L−6.8−4
L1228 (m/z=925.539202; rt=6.81597)
T/S Cluster: L−6.8−4
L1229 (m/z=802.59111; rt=6.85467)
T/S Cluster: L−6.9−1
L1230 (m/z=802.539836; rt=6.88223)
T/S Cluster: L−6.9−2
L1233 (m/z=954.619067; rt=6.89941)
T/S Cluster: L−6.9−3
L1232 (m/z=955.622607; rt=6.89929)
T/S Cluster: L−6.9−3
L1231 (m/z=955.575801; rt=6.89801)
T/S Cluster: L−6.9−3
PCo1
PCo2
−0.6
−0.4
−0.2
0.0
0.2
0.4

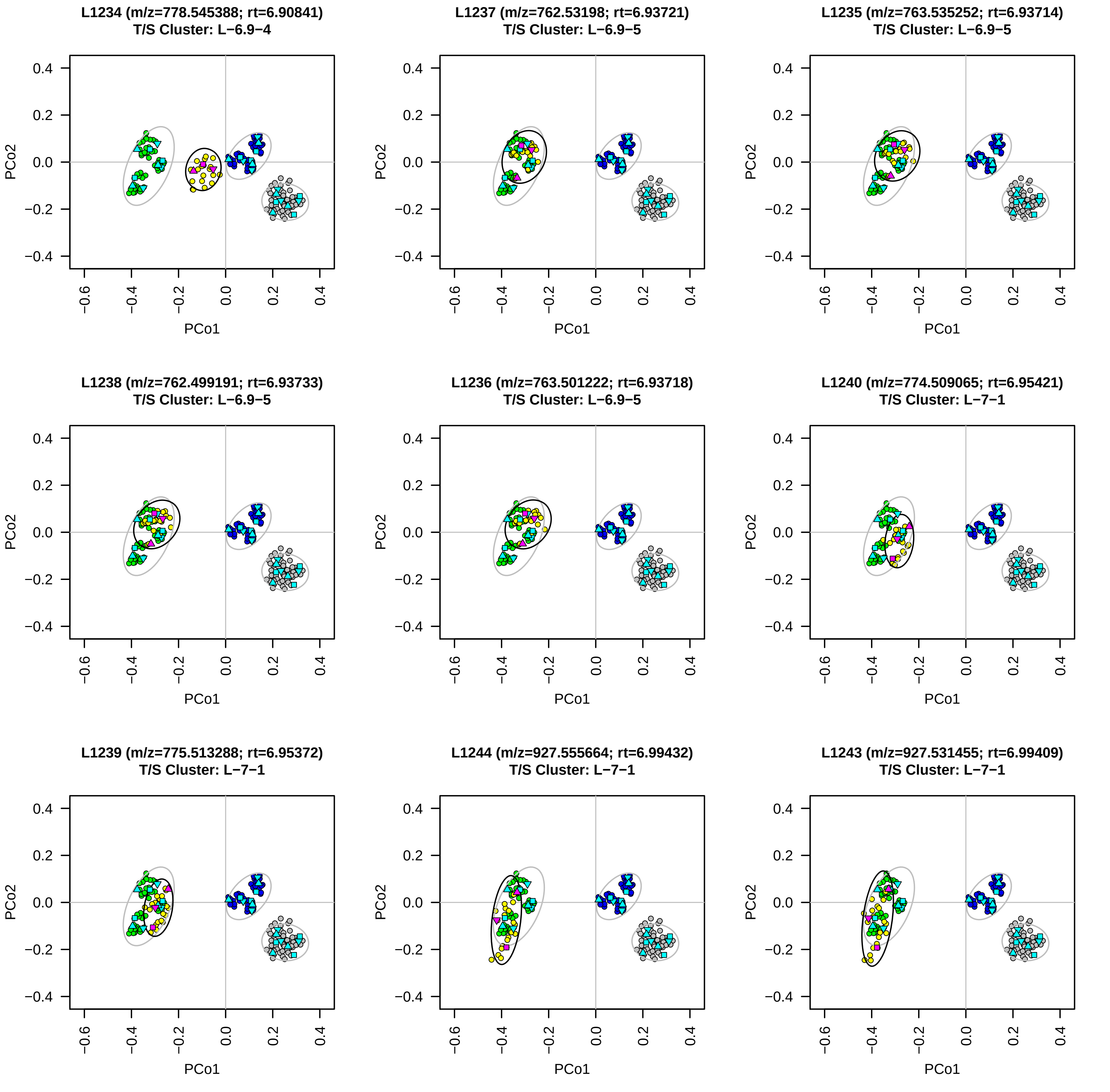
L1234 (m/z=778.545388; rt=6.90841)
T/S Cluster: L−6.9−4
L1237 (m/z=762.53198; rt=6.93721)
T/S Cluster: L−6.9−5
L1235 (m/z=763.535252; rt=6.93714)
T/S Cluster: L−6.9−5
L1238 (m/z=762.499191; rt=6.93733)
T/S Cluster: L−6.9−5
L1236 (m/z=763.501222; rt=6.93718)
T/S Cluster: L−6.9−5
L1240 (m/z=774.509065; rt=6.95421)
T/S Cluster: L−7−1
L1239 (m/z=775.513288; rt=6.95372)
T/S Cluster: L−7−1
L1244 (m/z=927.555664; rt=6.99432)
T/S Cluster: L−7−1
L1243 (m/z=927.531455; rt=6.99409)
T/S Cluster: L−7−1
PCo1
PCo2
−0.6
−0.4
−0.2
0.0
0.2
0.4

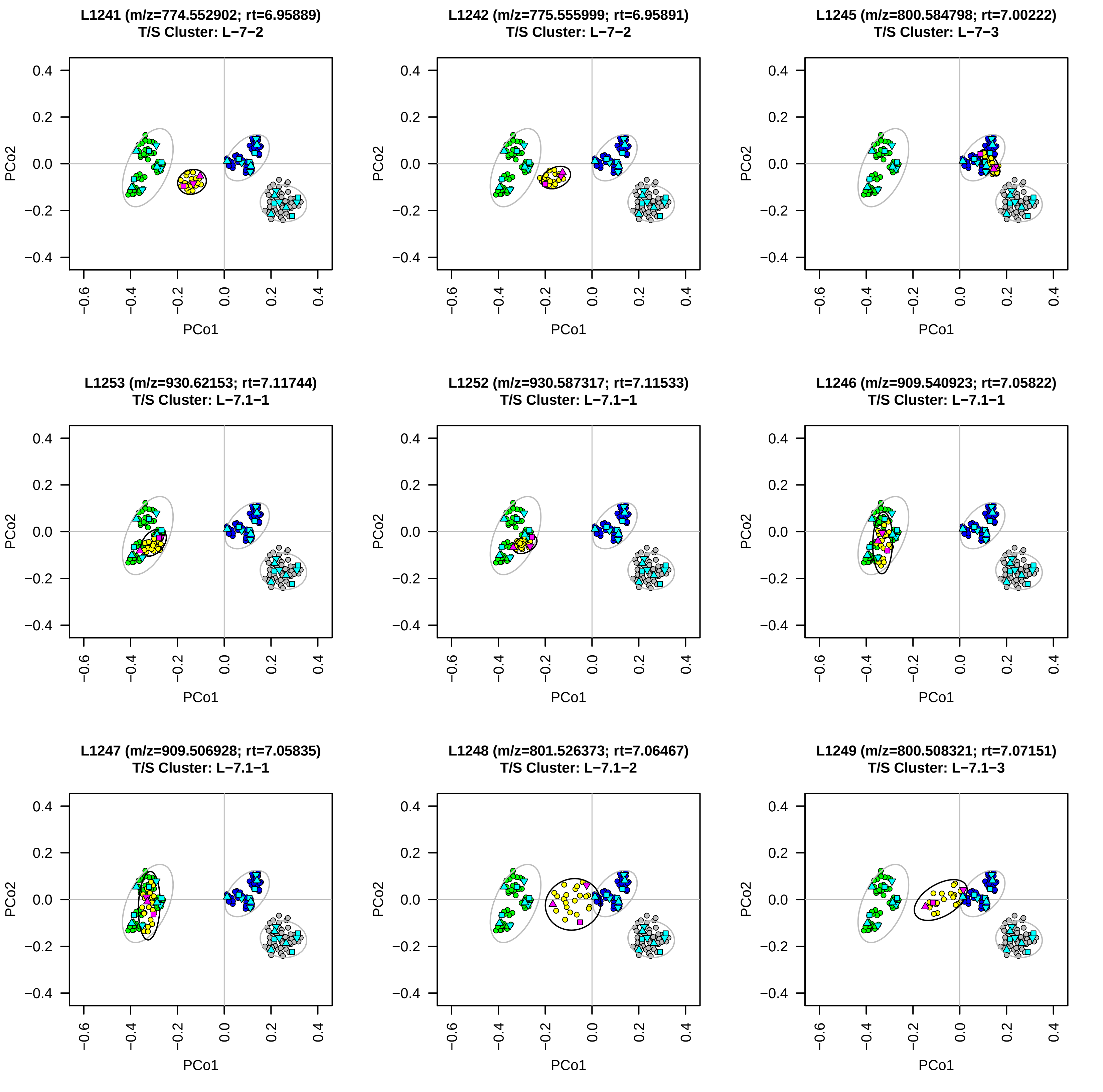
L1241 (m/z=774.552902; rt=6.95889)
T/S Cluster: L−7−2
L1242 (m/z=775.555999; rt=6.95891)
T/S Cluster: L−7−2
L1245 (m/z=800.584798; rt=7.00222)
T/S Cluster: L−7−3
L1253 (m/z=930.62153; rt=7.11744)
T/S Cluster: L−7.1−1
L1252 (m/z=930.587317; rt=7.11533)
T/S Cluster: L−7.1−1
L1246 (m/z=909.540923; rt=7.05822)
T/S Cluster: L−7.1−1
L1247 (m/z=909.506928; rt=7.05835)
T/S Cluster: L−7.1−1
L1248 (m/z=801.526373; rt=7.06467)
T/S Cluster: L−7.1−2
L1249 (m/z=800.508321; rt=7.07151)
T/S Cluster: L−7.1−3
PCo1
PCo2
0.4
0.2
0.0
−0.2
−0.4
−0.6

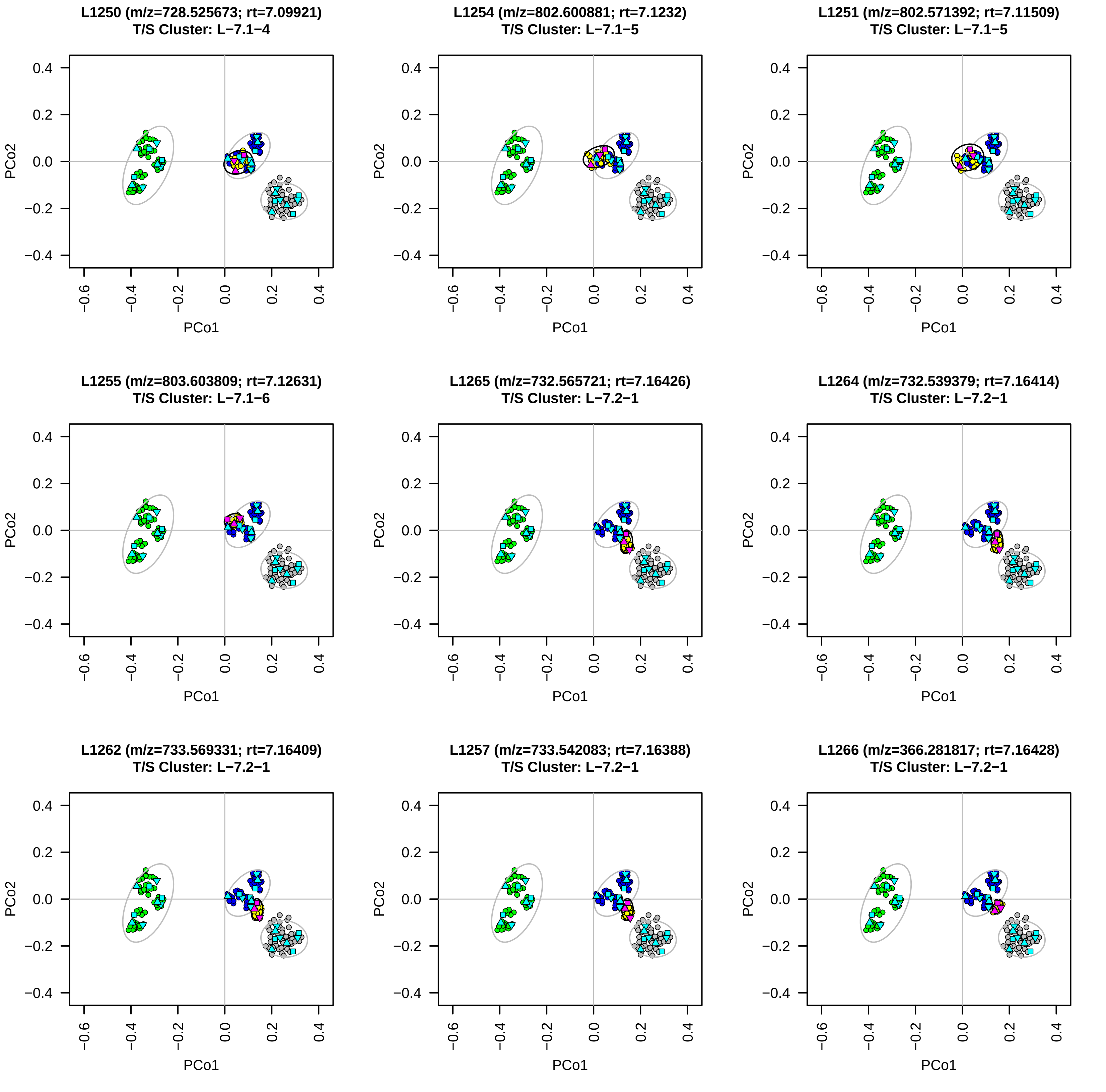
L1250 (m/z=728.525673; rt=7.09921)
T/S Cluster: L−7.1−4
L1254 (m/z=802.600881; rt=7.1232)
T/S Cluster: L−7.1−5
L1251 (m/z=802.571392; rt=7.11509)
T/S Cluster: L−7.1−5
L1255 (m/z=803.603809; rt=7.12631)
T/S Cluster: L−7.1−6
L1265 (m/z=732.565721; rt=7.16426)
T/S Cluster: L−7.2−1
L1264 (m/z=732.539379; rt=7.16414)
T/S Cluster: L−7.2−1
L1262 (m/z=733.569331; rt=7.16409)
T/S Cluster: L−7.2−1
L1257 (m/z=733.542083; rt=7.16388)
T/S Cluster: L−7.2−1
L1266 (m/z=366.281817; rt=7.16428)
T/S Cluster: L−7.2−1
PCo1
PCo2

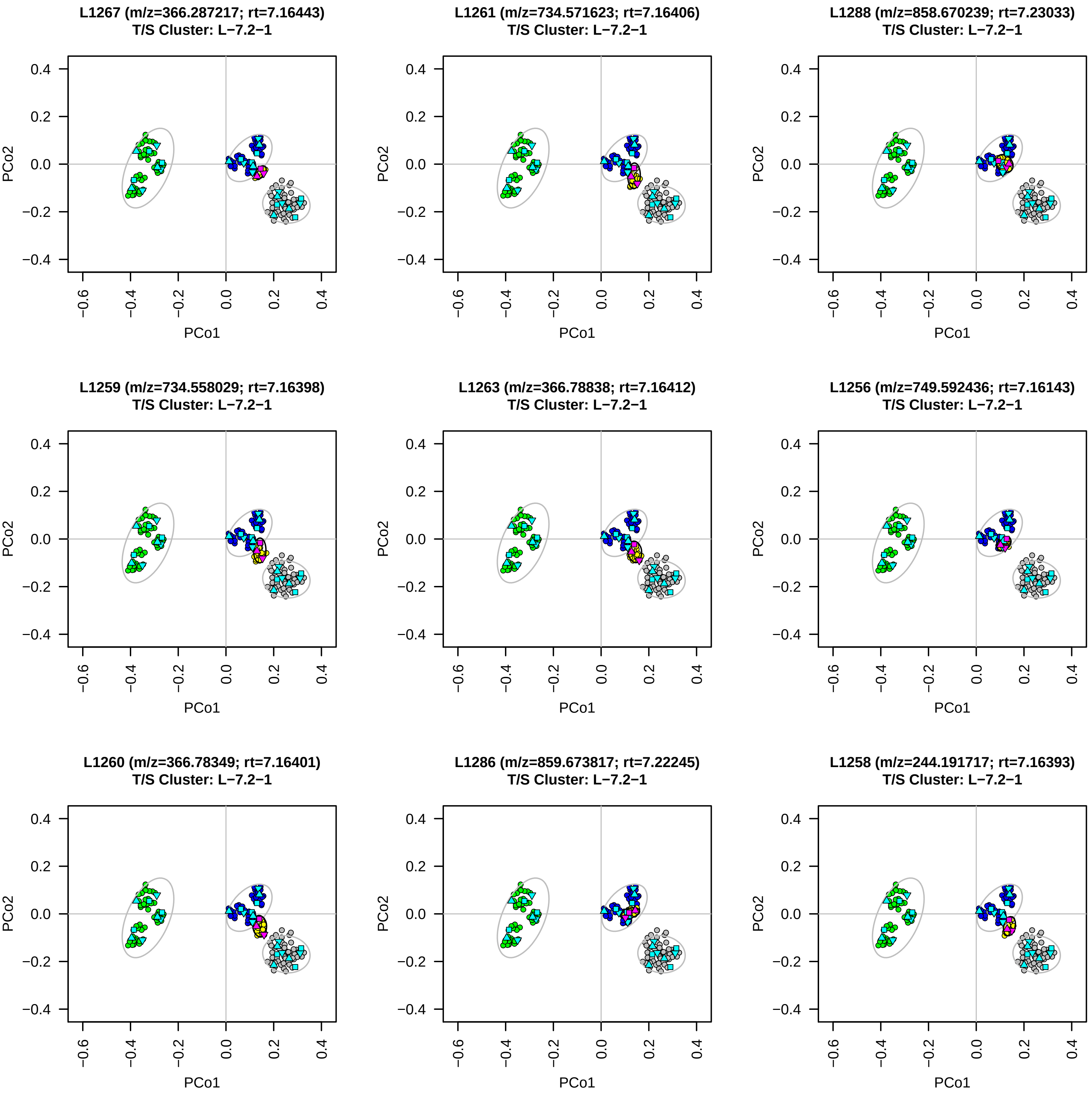
L1267 (m/z=366.287217; rt=7.16443)
T/S Cluster: L−7.2−1
L1261 (m/z=734.571623; rt=7.16406)
T/S Cluster: L−7.2−1
L1288 (m/z=858.670239; rt=7.23033)
T/S Cluster: L−7.2−1
L1259 (m/z=734.558029; rt=7.16398)
T/S Cluster: L−7.2−1
L1263 (m/z=366.78838; rt=7.16412)
T/S Cluster: L−7.2−1
L1256 (m/z=749.592436; rt=7.16143)
T/S Cluster: L−7.2−1
L1260 (m/z=366.78349; rt=7.16401)
T/S Cluster: L−7.2−1
L1286 (m/z=859.673817; rt=7.22245)
T/S Cluster: L−7.2−1
L1258 (m/z=244.191717; rt=7.16393)
T/S Cluster: L−7.2−1
PCo1
PCo2

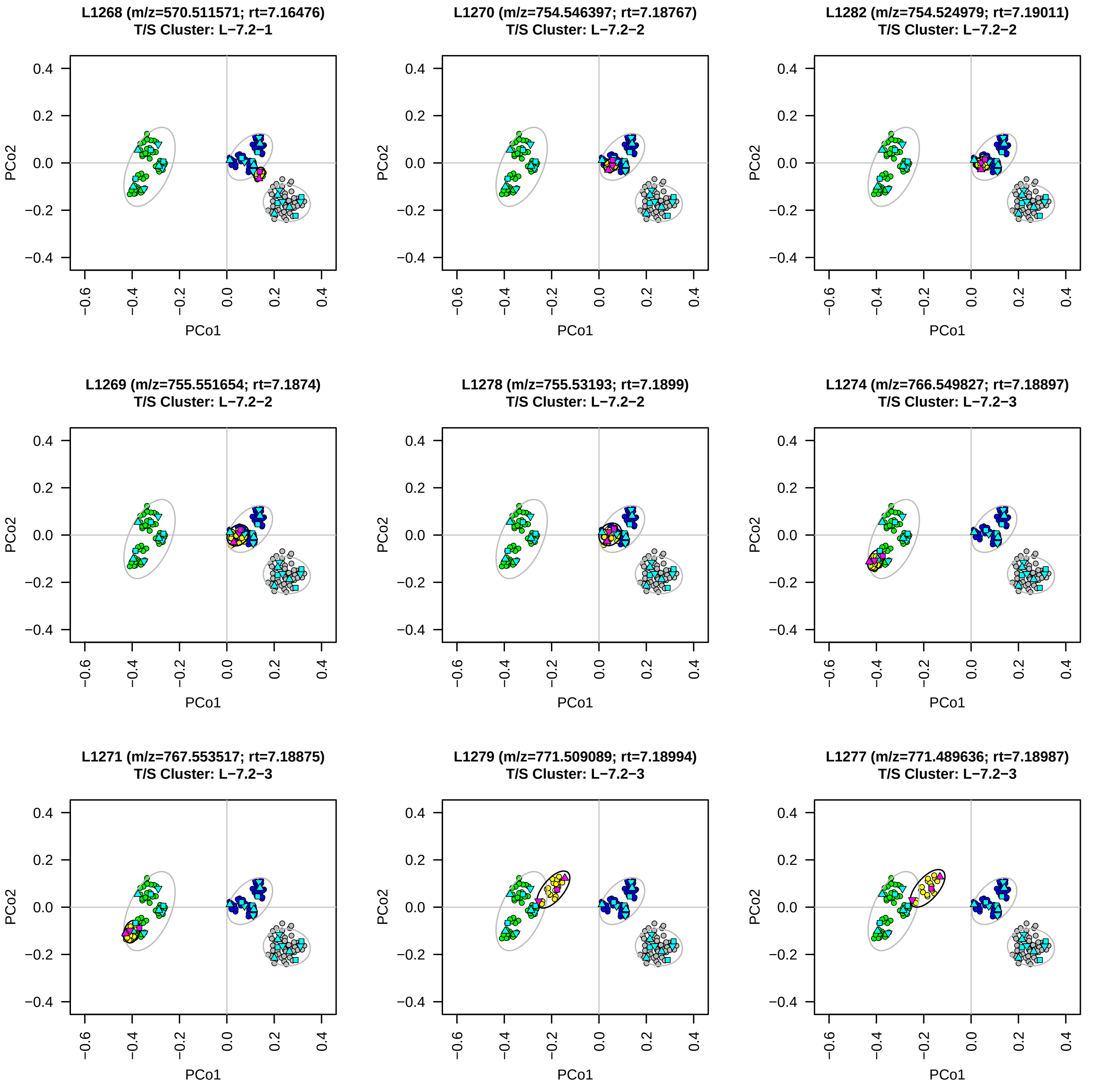
L1268 (m/z=570.511571; rt=7.16476)
T/S Cluster: L−7.2−1
L1270 (m/z=754.546397; rt=7.18767)
T/S Cluster: L−7.2−2
L1282 (m/z=754.524979; rt=7.19011)
T/S Cluster: L−7.2−2
L1269 (m/z=755.551654; rt=7.1874)
T/S Cluster: L−7.2−2
L1278 (m/z=755.53193; rt=7.1899)
T/S Cluster: L−7.2−2
L1274 (m/z=766.549827; rt=7.18897)
T/S Cluster: L−7.2−3
L1271 (m/z=767.553517; rt=7.18875)
T/S Cluster: L−7.2−3
L1279 (m/z=771.509089; rt=7.18994)
T/S Cluster: L−7.2−3
L1277 (m/z=771.489636; rt=7.18987)
T/S Cluster: L−7.2−3
PCo1
PCo2
−0.6
−0.4
−0.2
0.0
0.2
0.4

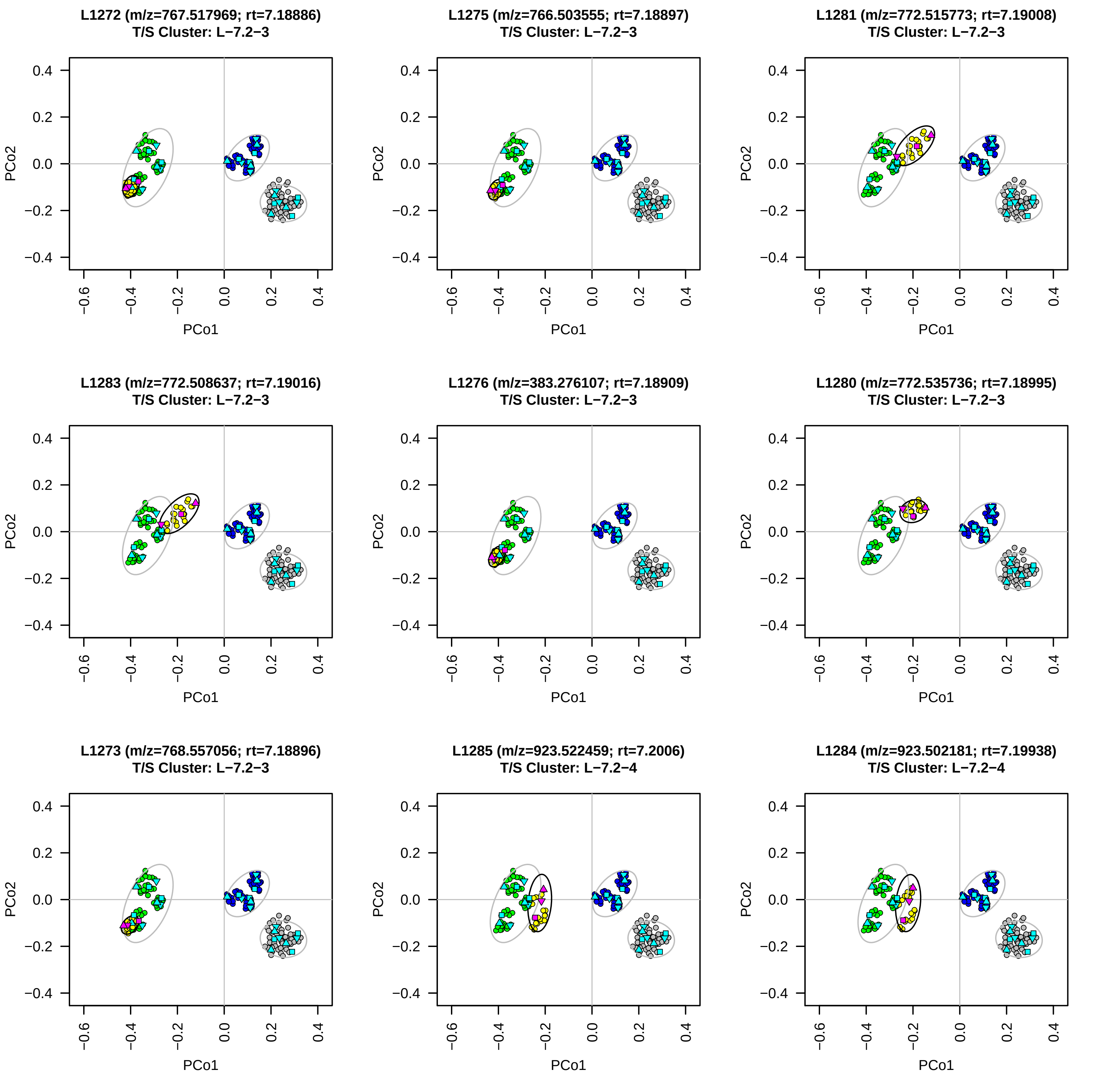
L1272 (m/z=767.517969; rt=7.18886)
T/S Cluster: L−7.2−3
L1275 (m/z=766.503555; rt=7.18897)
T/S Cluster: L−7.2−3
L1281 (m/z=772.515773; rt=7.19008)
T/S Cluster: L−7.2−3
L1283 (m/z=772.508637; rt=7.19016)
T/S Cluster: L−7.2−3
L1276 (m/z=383.276107; rt=7.18909)
T/S Cluster: L−7.2−3
L1280 (m/z=772.535736; rt=7.18995)
T/S Cluster: L−7.2−3
L1273 (m/z=768.557056; rt=7.18896)
T/S Cluster: L−7.2−3
L1285 (m/z=923.522459; rt=7.2006)
T/S Cluster: L−7.2−4
L1284 (m/z=923.502181; rt=7.19938)
T/S Cluster: L−7.2−4
PCo1
PCo2
−0.6
−0.4
−0.2
0.0
0.2
0.4

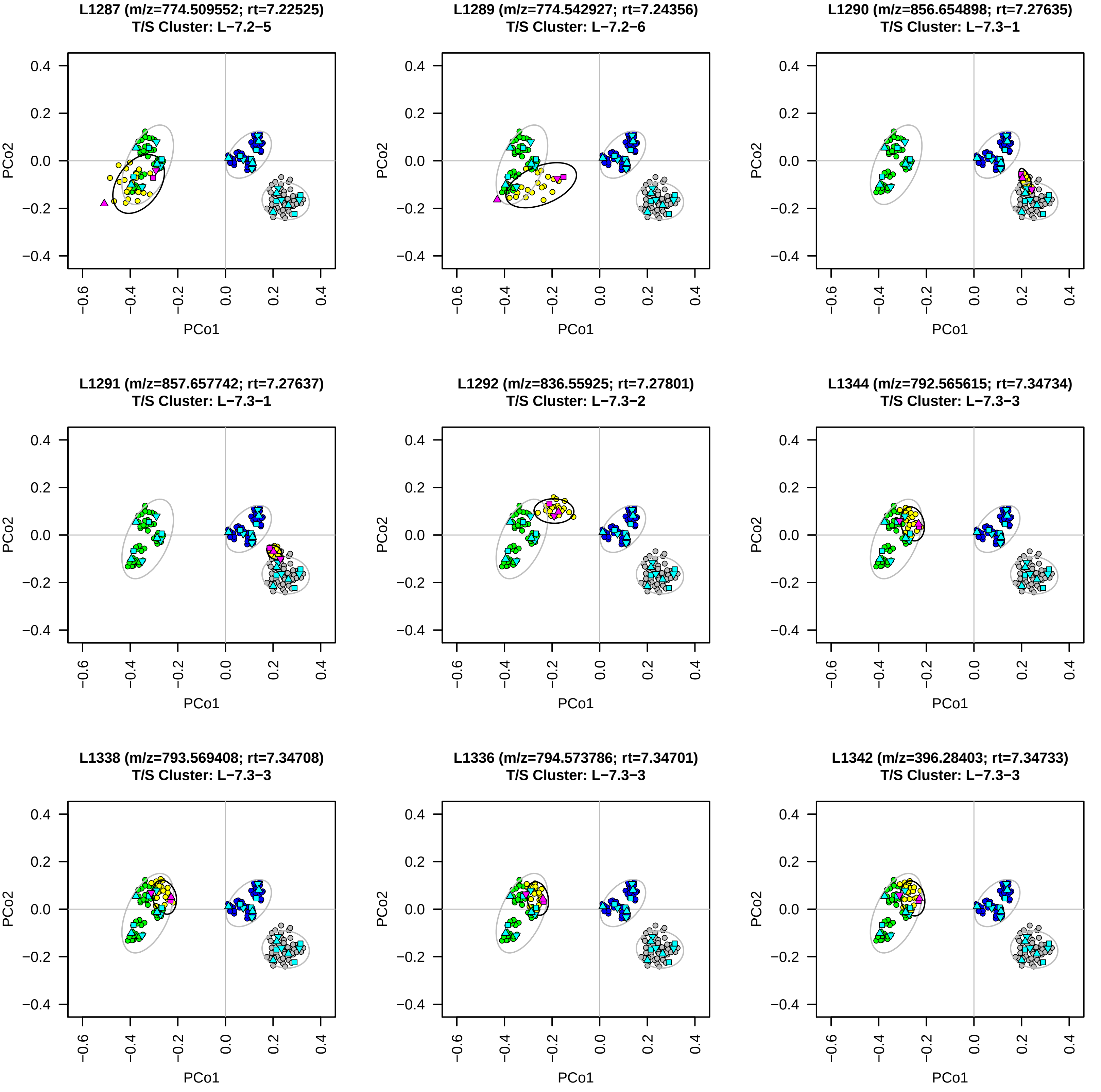
L1287 (m/z=774.509552; rt=7.22525)
T/S Cluster: L−7.2−5
L1289 (m/z=774.542927; rt=7.24356)
T/S Cluster: L−7.2−6
L1290 (m/z=856.654898; rt=7.27635)
T/S Cluster: L−7.3−1
L1291 (m/z=857.657742; rt=7.27637)
T/S Cluster: L−7.3−1
L1292 (m/z=836.55925; rt=7.27801)
T/S Cluster: L−7.3−2
L1344 (m/z=792.565615; rt=7.34734)
T/S Cluster: L−7.3−3
L1338 (m/z=793.569408; rt=7.34708)
T/S Cluster: L−7.3−3
L1336 (m/z=794.573786; rt=7.34701)
T/S Cluster: L−7.3−3
L1342 (m/z=396.28403; rt=7.34733)
T/S Cluster: L−7.3−3
PCo1
PCo2
−0.6
−0.4
−0.2
0.0
0.2
0.4

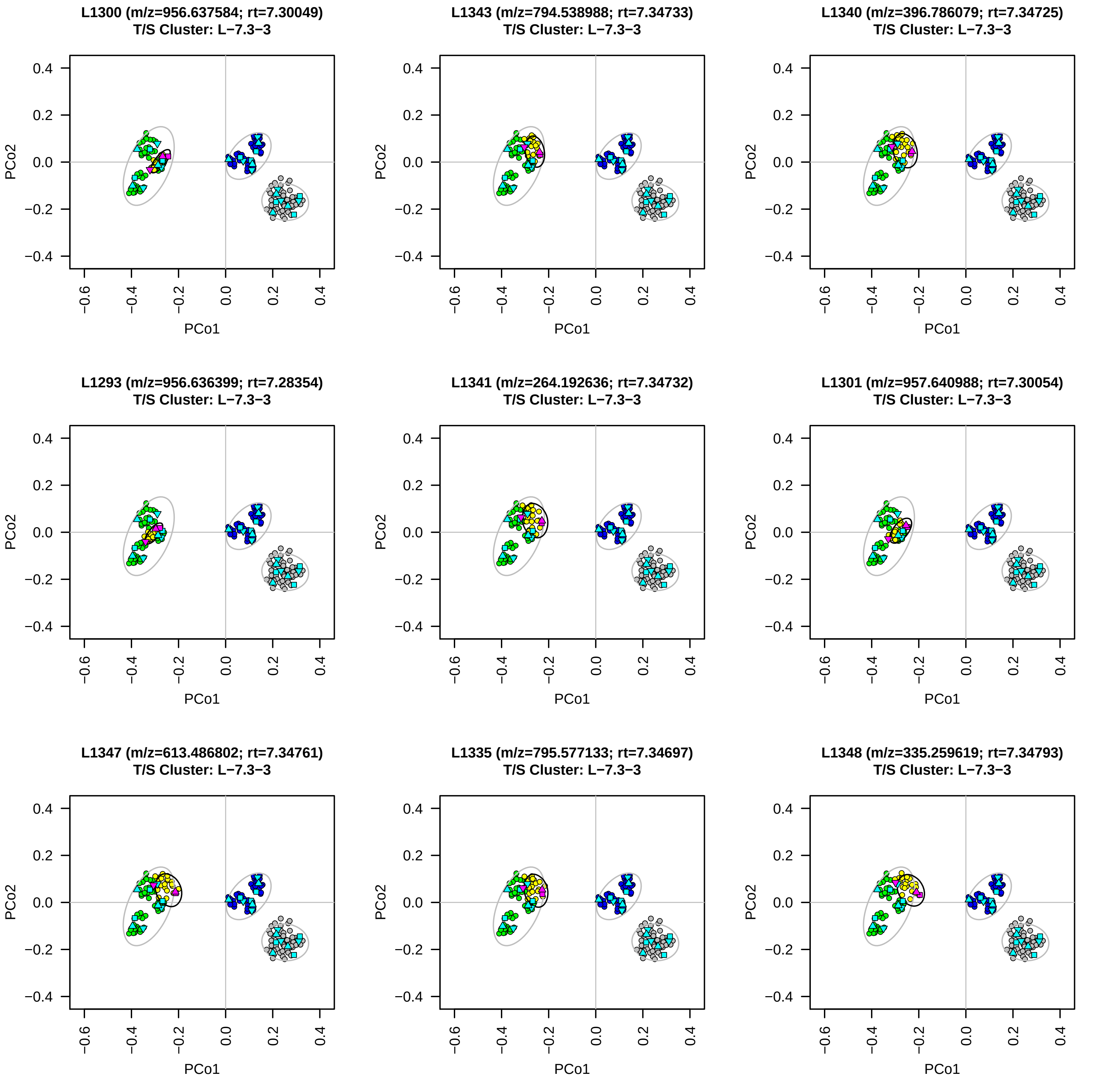
L1300 (m/z=956.637584; rt=7.30049)
T/S Cluster: L−7.3−3
L1343 (m/z=794.538988; rt=7.34733)
T/S Cluster: L−7.3−3
L1340 (m/z=396.786079; rt=7.34725)
T/S Cluster: L−7.3−3
L1293 (m/z=956.636399; rt=7.28354)
T/S Cluster: L−7.3−3
L1341 (m/z=264.192636; rt=7.34732)
T/S Cluster: L−7.3−3
L1301 (m/z=957.640988; rt=7.30054)
T/S Cluster: L−7.3−3
L1347 (m/z=613.486802; rt=7.34761)
T/S Cluster: L−7.3−3
L1335 (m/z=795.577133; rt=7.34697)
T/S Cluster: L−7.3−3
L1348 (m/z=335.259619; rt=7.34793)
T/S Cluster: L−7.3−3
PCo1
PCo2

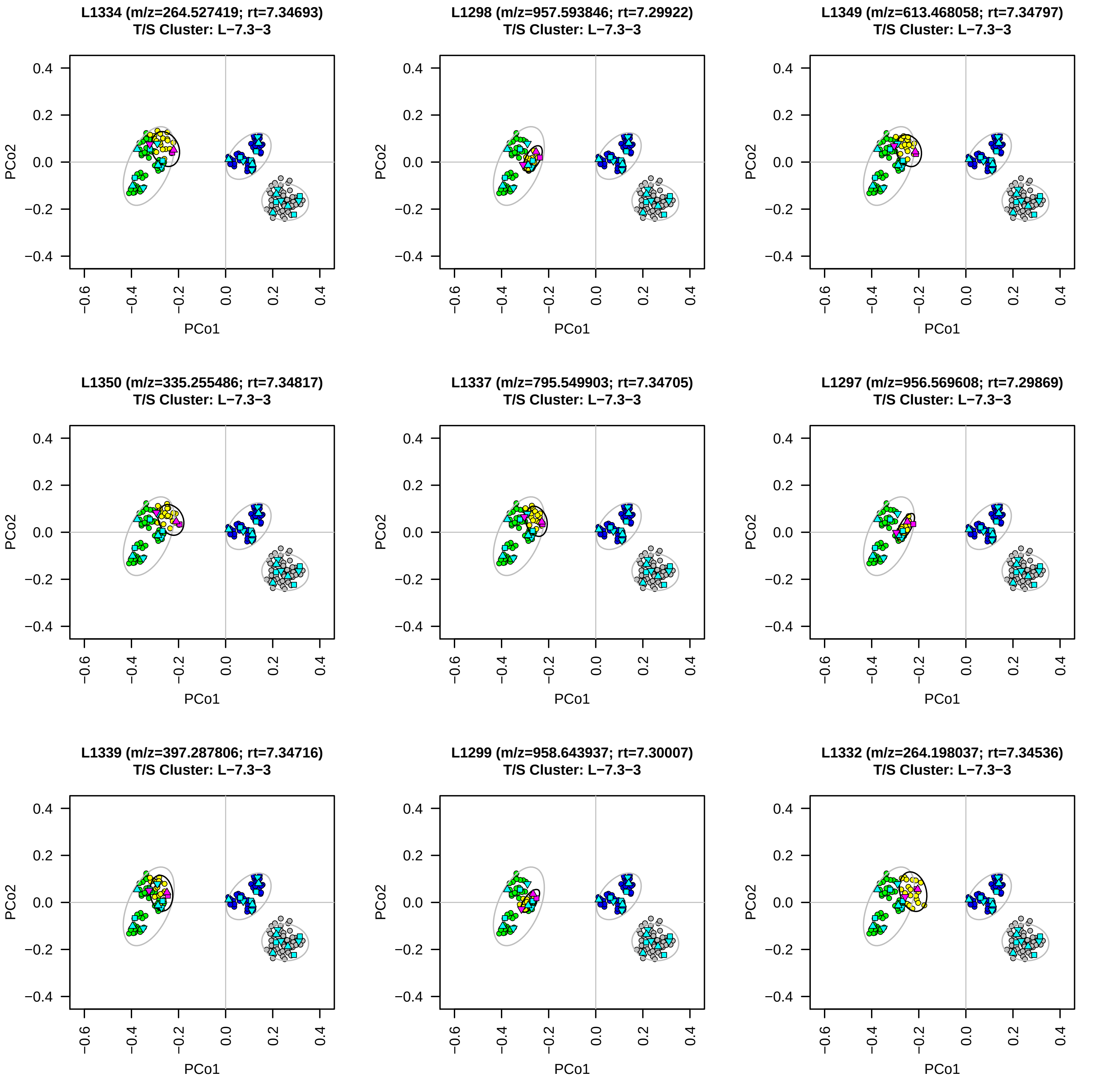
L1334 (m/z=264.527419; rt=7.34693)
T/S Cluster: L−7.3−3
L1298 (m/z=957.593846; rt=7.29922)
T/S Cluster: L−7.3−3
L1349 (m/z=613.468058; rt=7.34797)
T/S Cluster: L−7.3−3
L1350 (m/z=335.255486; rt=7.34817)
T/S Cluster: L−7.3−3
L1337 (m/z=795.549903; rt=7.34705)
T/S Cluster: L−7.3−3
L1297 (m/z=956.569608; rt=7.29869)
T/S Cluster: L−7.3−3
L1339 (m/z=397.287806; rt=7.34716)
T/S Cluster: L−7.3−3
L1299 (m/z=958.643937; rt=7.30007)
T/S Cluster: L−7.3−3
L1332 (m/z=264.198037; rt=7.34536)
T/S Cluster: L−7.3−3
PCo1
PCo2
−0.6
−0.4
−0.2
0.0
0.2
0.4

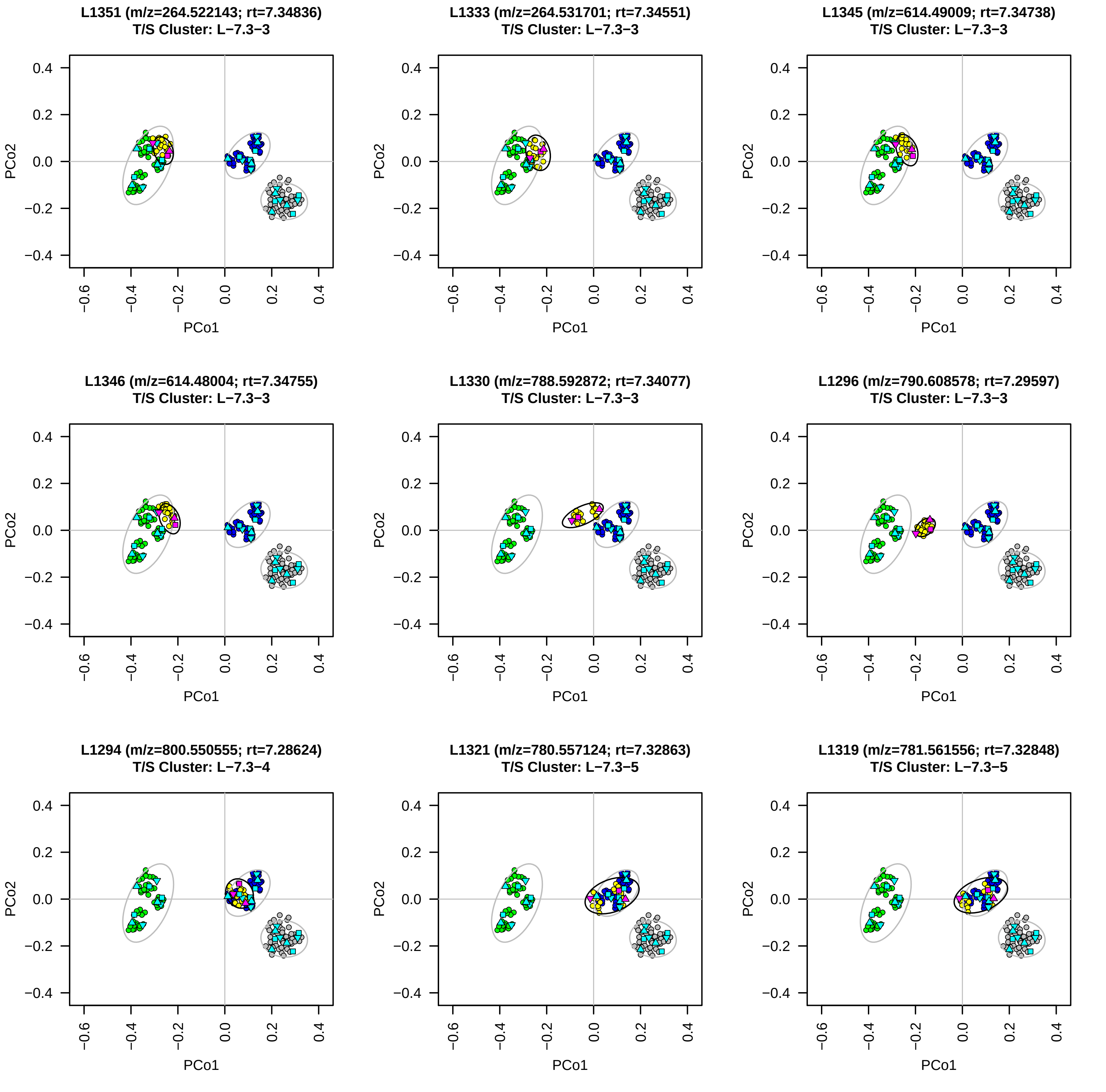
L1351 (m/z=264.522143; rt=7.34836)
T/S Cluster: L−7.3−3
L1333 (m/z=264.531701; rt=7.34551)
T/S Cluster: L−7.3−3
L1345 (m/z=614.49009; rt=7.34738)
T/S Cluster: L−7.3−3
L1346 (m/z=614.48004; rt=7.34755)
T/S Cluster: L−7.3−3
L1330 (m/z=788.592872; rt=7.34077)
T/S Cluster: L−7.3−3
L1296 (m/z=790.608578; rt=7.29597)
T/S Cluster: L−7.3−3
L1294 (m/z=800.550555; rt=7.28624)
T/S Cluster: L−7.3−4
L1321 (m/z=780.557124; rt=7.32863)
T/S Cluster: L−7.3−5
L1319 (m/z=781.561556; rt=7.32848)
T/S Cluster: L−7.3−5
PCo1
PCo2
−0.6
−0.4
−0.2
0.0
0.2
0.4

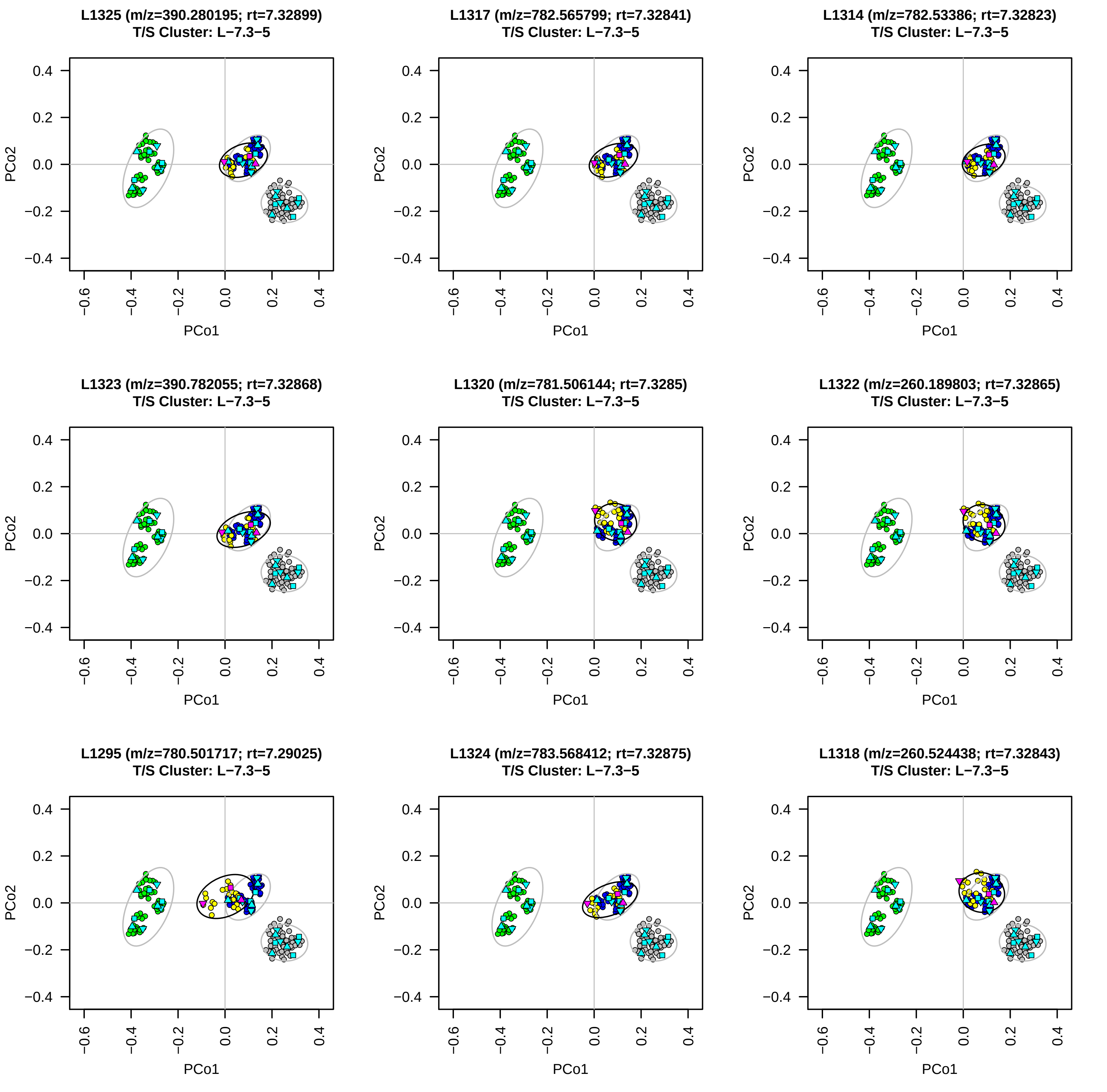
L1325 (m/z=390.280195; rt=7.32899)
T/S Cluster: L−7.3−5
L1317 (m/z=782.565799; rt=7.32841)
T/S Cluster: L−7.3−5
L1314 (m/z=782.53386; rt=7.32823)
T/S Cluster: L−7.3−5
L1323 (m/z=390.782055; rt=7.32868)
T/S Cluster: L−7.3−5
L1320 (m/z=781.506144; rt=7.3285)
T/S Cluster: L−7.3−5
L1322 (m/z=260.189803; rt=7.32865)
T/S Cluster: L−7.3−5
L1295 (m/z=780.501717; rt=7.29025)
T/S Cluster: L−7.3−5
L1324 (m/z=783.568412; rt=7.32875)
T/S Cluster: L−7.3−5
L1318 (m/z=260.524438; rt=7.32843)
T/S Cluster: L−7.3−5
PCo1
PCo2
−0.6 −0.4 −0.2 0.0 0.2 0.4
−0.4 −0.2 0.0 0.2 0.4

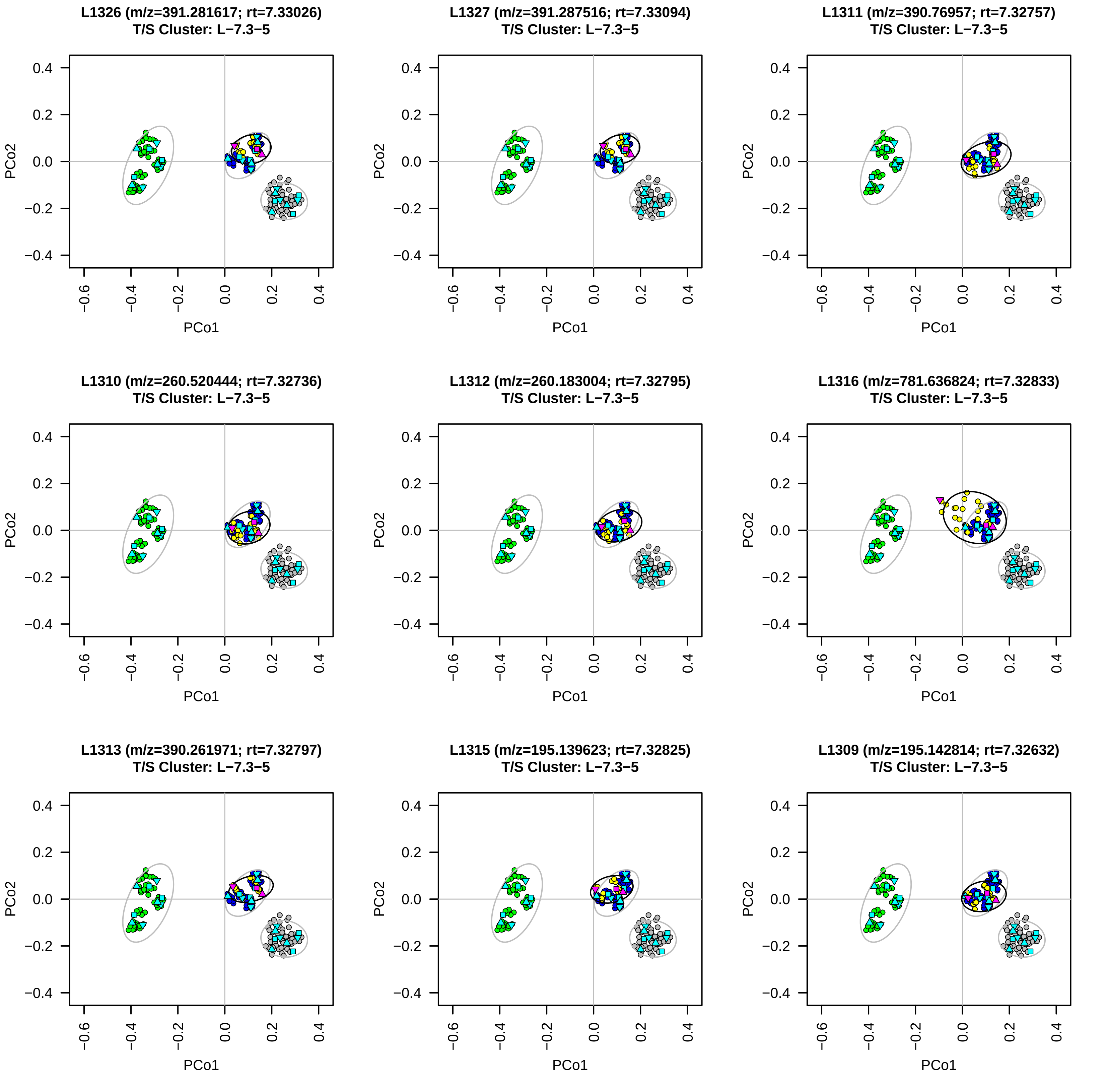
L1326 (m/z=391.281617; rt=7.33026)
T/S Cluster: L−7.3−5
L1327 (m/z=391.287516; rt=7.33094)
T/S Cluster: L−7.3−5
L1311 (m/z=390.76957; rt=7.32757)
T/S Cluster: L−7.3−5
L1310 (m/z=260.520444; rt=7.32736)
T/S Cluster: L−7.3−5
L1312 (m/z=260.183004; rt=7.32795)
T/S Cluster: L−7.3−5
L1316 (m/z=781.636824; rt=7.32833)
T/S Cluster: L−7.3−5
L1313 (m/z=390.261971; rt=7.32797)
T/S Cluster: L−7.3−5
L1315 (m/z=195.139623; rt=7.32825)
T/S Cluster: L−7.3−5
L1309 (m/z=195.142814; rt=7.32632)
T/S Cluster: L−7.3−5
PCo1
PCo2
0.4
0.2
0.0
−0.2
−0.4
−0.6

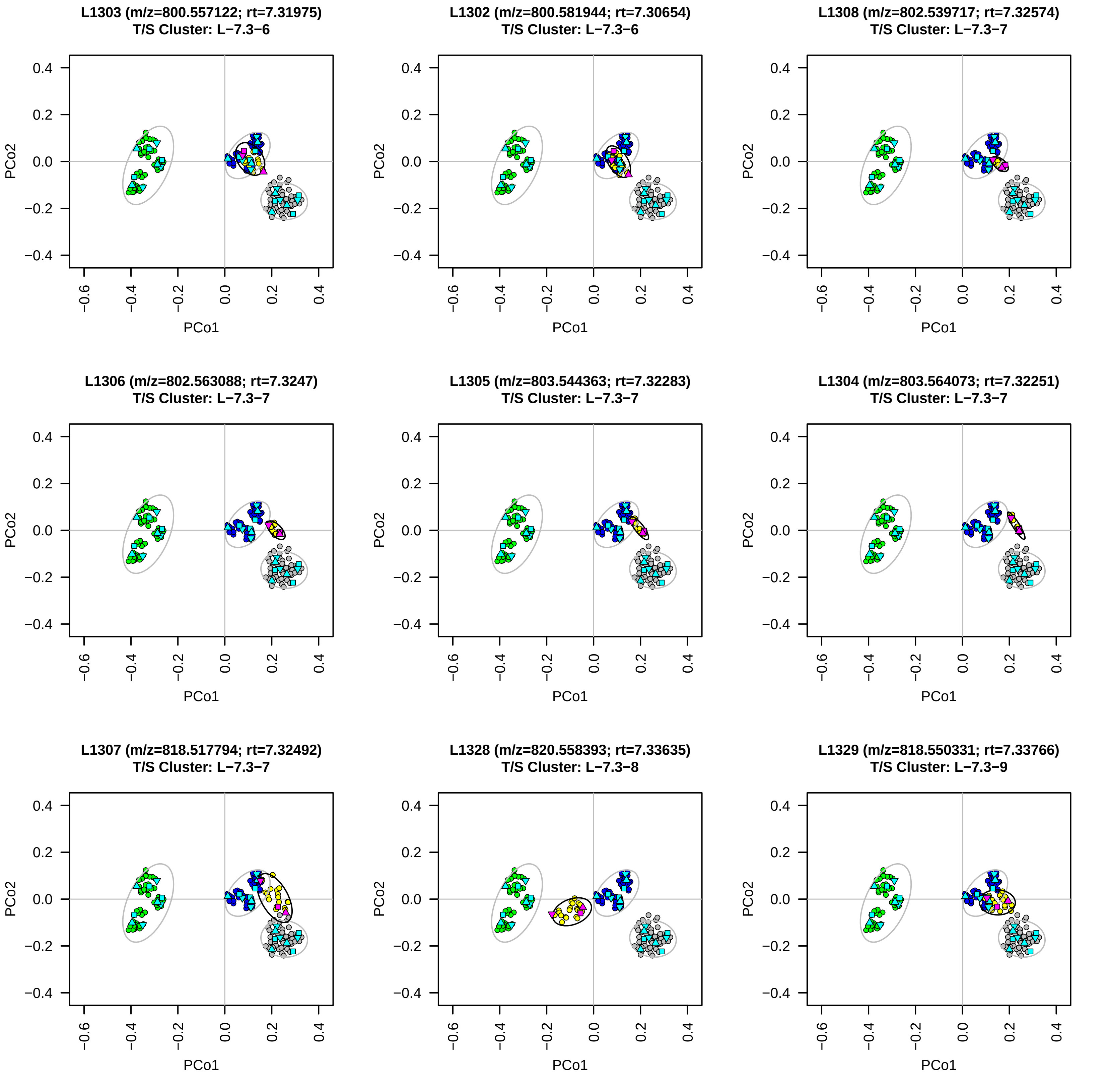

L1303 (m/z=800.557122; rt=7.31975)
T/S Cluster: L−7.3−6
L1302 (m/z=800.581944; rt=7.30654)
T/S Cluster: L−7.3−6
L1308 (m/z=802.539717; rt=7.32574)
T/S Cluster: L−7.3−7
L1306 (m/z=802.563088; rt=7.3247)
T/S Cluster: L−7.3−7
L1305 (m/z=803.544363; rt=7.32283)
T/S Cluster: L−7.3−7
L1304 (m/z=803.564073; rt=7.32251)
T/S Cluster: L−7.3−7
L1307 (m/z=818.517794; rt=7.32492)
T/S Cluster: L−7.3−7
L1328 (m/z=820.558393; rt=7.33635)
T/S Cluster: L−7.3−8
L1329 (m/z=818.550331; rt=7.33766)
T/S Cluster: L−7.3−9
PCo1
PCo2
−0.6
−0.4
−0.2
0.0
0.2
0.4

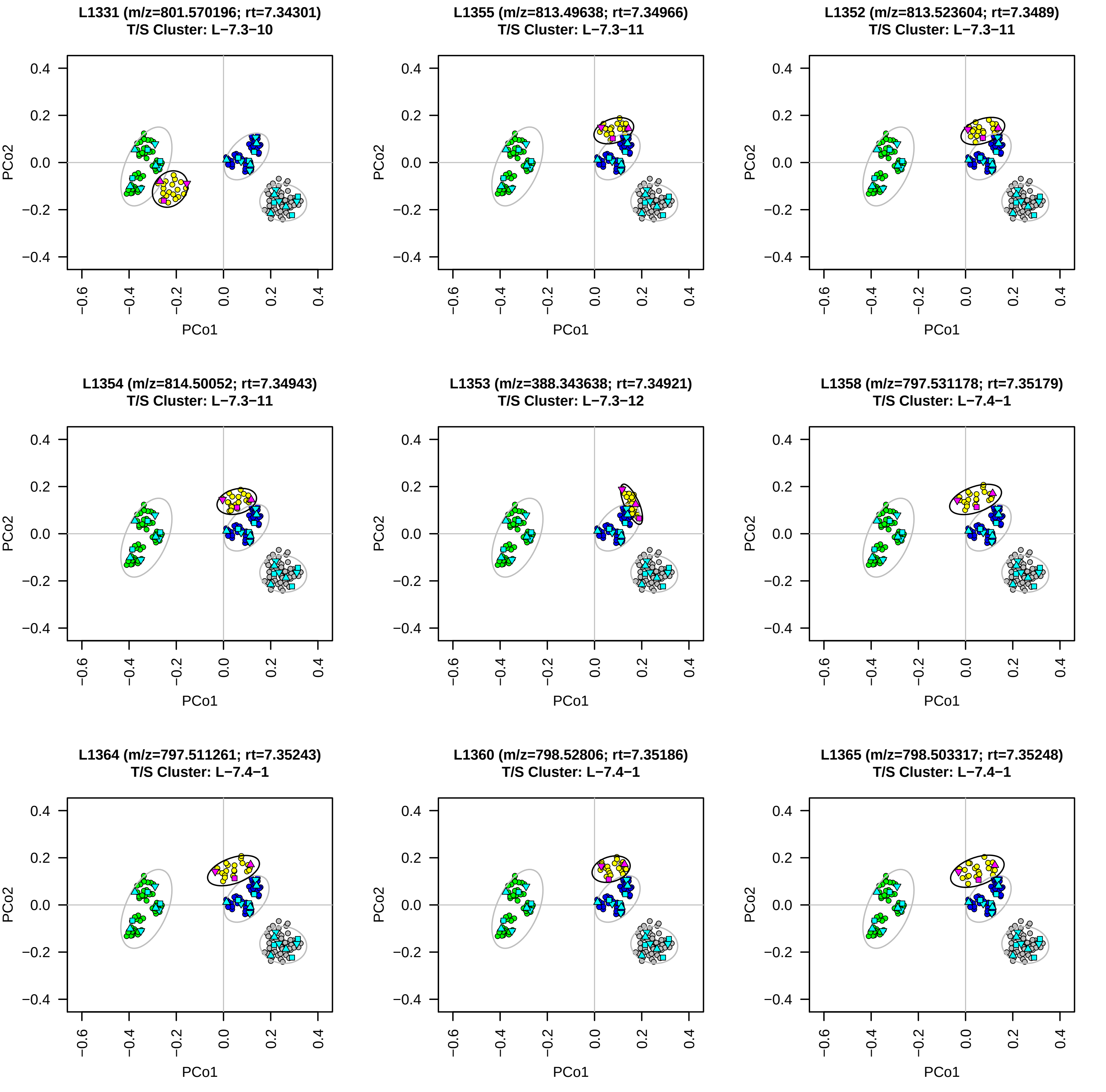
L1331 (m/z=801.570196; rt=7.34301)
T/S Cluster: L−7.3−10
L1355 (m/z=813.49638; rt=7.34966)
T/S Cluster: L−7.3−11
L1352 (m/z=813.523604; rt=7.3489)
T/S Cluster: L−7.3−11
L1354 (m/z=814.50052; rt=7.34943)
T/S Cluster: L−7.3−11
L1353 (m/z=388.343638; rt=7.34921)
T/S Cluster: L−7.3−12
L1358 (m/z=797.531178; rt=7.35179)
T/S Cluster: L−7.4−1
L1364 (m/z=797.511261; rt=7.35243)
T/S Cluster: L−7.4−1
L1360 (m/z=798.52806; rt=7.35186)
T/S Cluster: L−7.4−1
L1365 (m/z=798.503317; rt=7.35248)
T/S Cluster: L−7.4−1
PCo1
PCo2

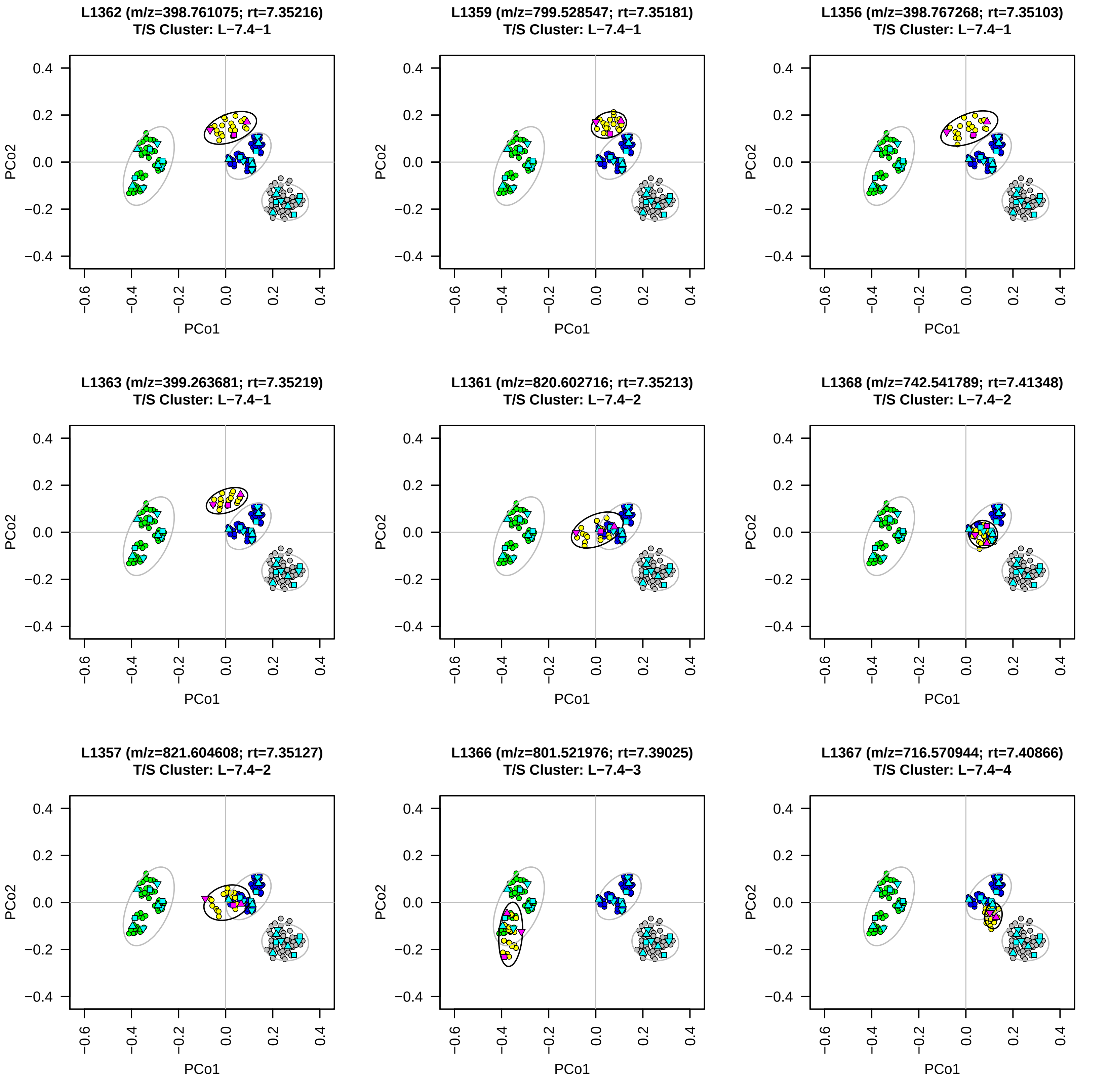
L1362 (m/z=398.761075; rt=7.35216)
T/S Cluster: L−7.4−1
L1359 (m/z=799.528547; rt=7.35181)
T/S Cluster: L−7.4−1
L1356 (m/z=398.767268; rt=7.35103)
T/S Cluster: L−7.4−1
L1363 (m/z=399.263681; rt=7.35219)
T/S Cluster: L−7.4−1
L1361 (m/z=820.602716; rt=7.35213)
T/S Cluster: L−7.4−2
L1368 (m/z=742.541789; rt=7.41348)
T/S Cluster: L−7.4−2
L1357 (m/z=821.604608; rt=7.35127)
T/S Cluster: L−7.4−2
L1366 (m/z=801.521976; rt=7.39025)
T/S Cluster: L−7.4−3
L1367 (m/z=716.570944; rt=7.40866)
T/S Cluster: L−7.4−4
PCo1
PCo2
0.4
0.2
0.0
−0.2
−0.4
−0.6

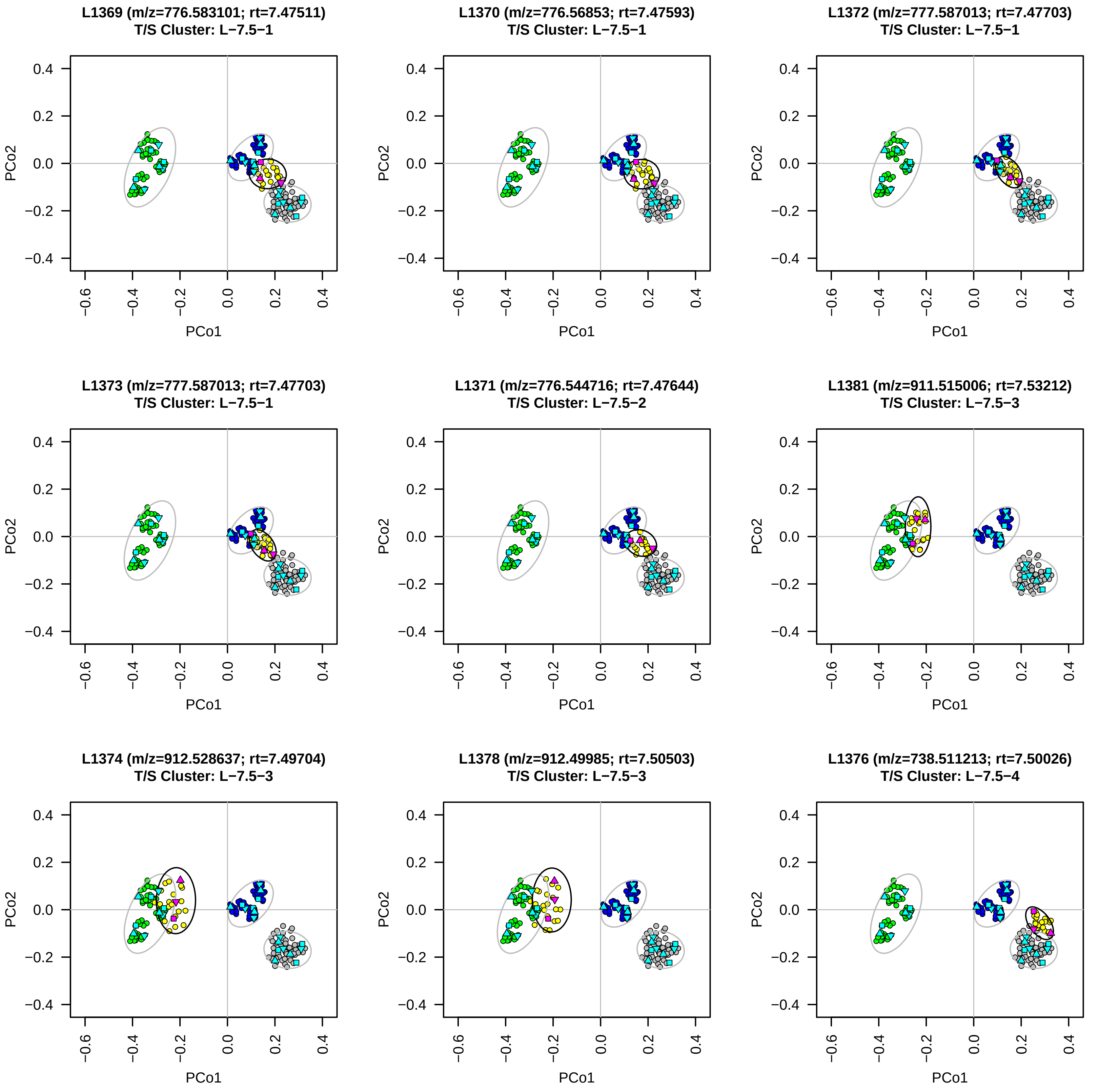

L1369 (m/z=776.583101; rt=7.47511)
T/S Cluster: L−7.5−1
L1370 (m/z=776.56853; rt=7.47593)
T/S Cluster: L−7.5−1
L1372 (m/z=777.587013; rt=7.47703)
T/S Cluster: L−7.5−1
L1373 (m/z=777.587013; rt=7.47703)
T/S Cluster: L−7.5−1
L1371 (m/z=776.544716; rt=7.47644)
T/S Cluster: L−7.5−2
L1381 (m/z=911.515006; rt=7.53212)
T/S Cluster: L−7.5−3
L1374 (m/z=912.528637; rt=7.49704)
T/S Cluster: L−7.5−3
L1378 (m/z=912.49985; rt=7.50503)
T/S Cluster: L−7.5−3
L1376 (m/z=738.511213; rt=7.50026)
T/S Cluster: L−7.5−4
PCo1
PCo2
−0.6
−0.4
−0.2
0.0
0.2
0.4

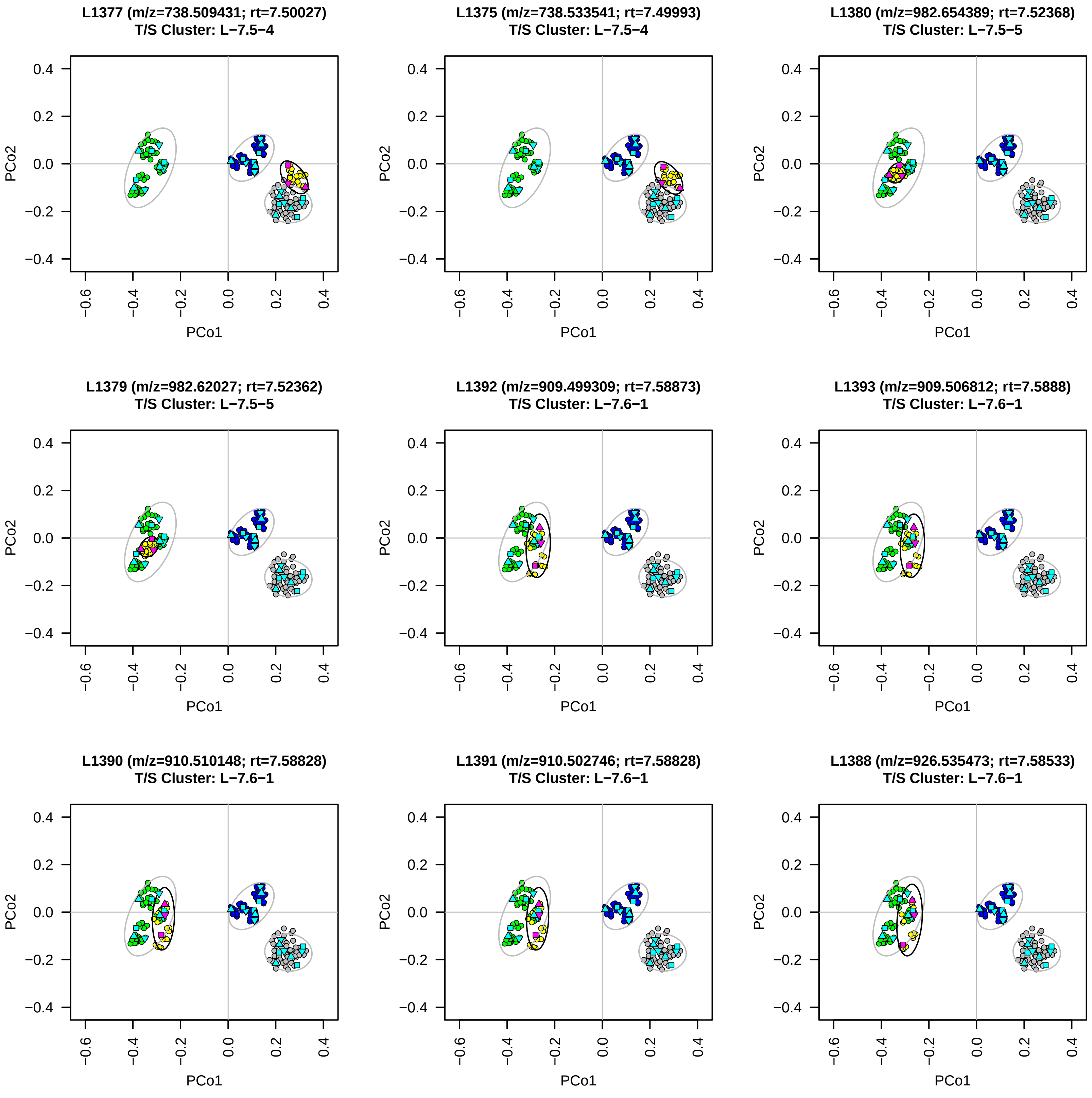

L1377 (m/z=738.509431; rt=7.50027)
T/S Cluster: L−7.5−4
L1375 (m/z=738.533541; rt=7.49993)
T/S Cluster: L−7.5−4
L1380 (m/z=982.654389; rt=7.52368)
T/S Cluster: L−7.5−5
L1379 (m/z=982.62027; rt=7.52362)
T/S Cluster: L−7.5−5
L1392 (m/z=909.499309; rt=7.58873)
T/S Cluster: L−7.6−1
L1393 (m/z=909.506812; rt=7.5888)
T/S Cluster: L−7.6−1
L1390 (m/z=910.510148; rt=7.58828)
T/S Cluster: L−7.6−1
L1391 (m/z=910.502746; rt=7.58828)
T/S Cluster: L−7.6−1
L1388 (m/z=926.535473; rt=7.58533)
T/S Cluster: L−7.6−1
PCo1
PCo2
−0.6
−0.4
−0.2
0.0
0.2
0.4

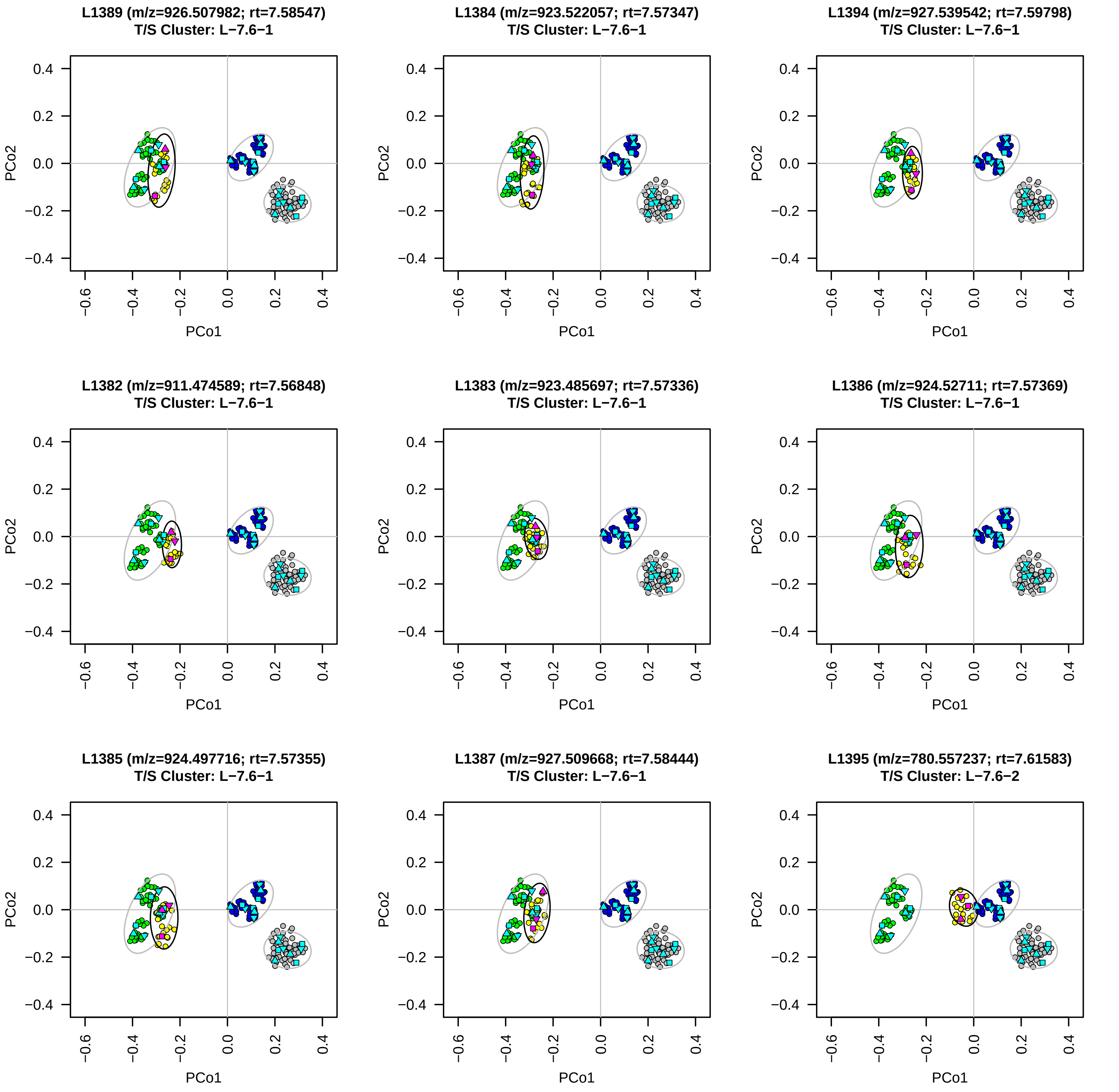
L1389 (m/z=926.507982; rt=7.58547)
T/S Cluster: L−7.6−1
L1384 (m/z=923.522057; rt=7.57347)
T/S Cluster: L−7.6−1
L1394 (m/z=927.539542; rt=7.59798)
T/S Cluster: L−7.6−1
L1382 (m/z=911.474589; rt=7.56848)
T/S Cluster: L−7.6−1
L1383 (m/z=923.485697; rt=7.57336)
T/S Cluster: L−7.6−1
L1386 (m/z=924.52711; rt=7.57369)
T/S Cluster: L−7.6−1
L1385 (m/z=924.497716; rt=7.57355)
T/S Cluster: L−7.6−1
L1387 (m/z=927.509668; rt=7.58444)
T/S Cluster: L−7.6−1
L1395 (m/z=780.557237; rt=7.61583)
T/S Cluster: L−7.6−2
PCo1
PCo2
−0.6
−0.4
−0.2
0.0
0.2
0.4

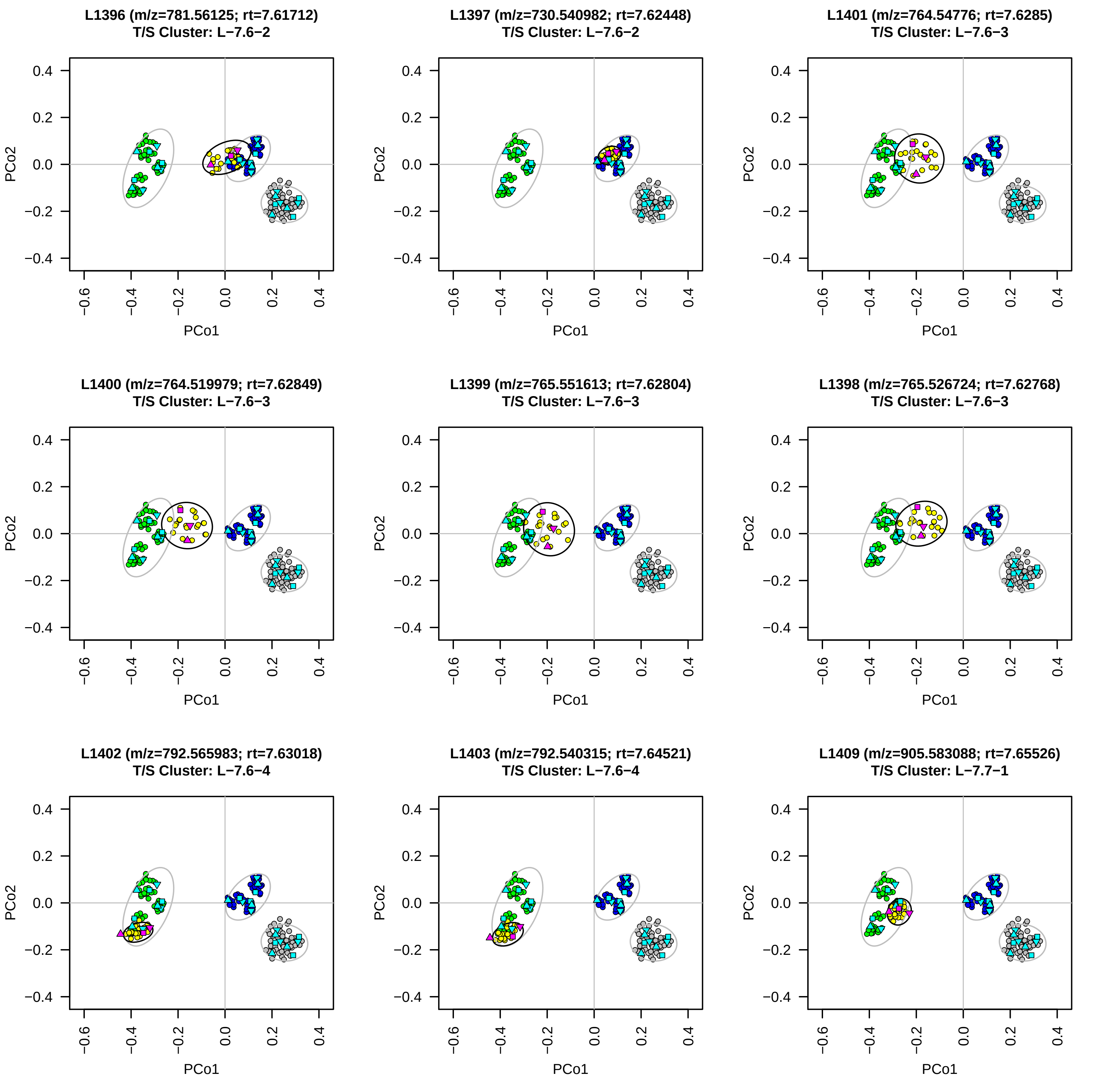
L1396 (m/z=781.56125; rt=7.61712)
T/S Cluster: L−7.6−2
L1397 (m/z=730.540982; rt=7.62448)
T/S Cluster: L−7.6−2
L1401 (m/z=764.54776; rt=7.6285)
T/S Cluster: L−7.6−3
L1400 (m/z=764.519979; rt=7.62849)
T/S Cluster: L−7.6−3
L1399 (m/z=765.551613; rt=7.62804)
T/S Cluster: L−7.6−3
L1398 (m/z=765.526724; rt=7.62768)
T/S Cluster: L−7.6−3
L1402 (m/z=792.565983; rt=7.63018)
T/S Cluster: L−7.6−4
L1403 (m/z=792.540315; rt=7.64521)
T/S Cluster: L−7.6−4
L1409 (m/z=905.583088; rt=7.65526)
T/S Cluster: L−7.7−1
PCo1
PCo2

L1411 (m/z=905.554105; rt=7.65544)
T/S Cluster: L−7.7−1

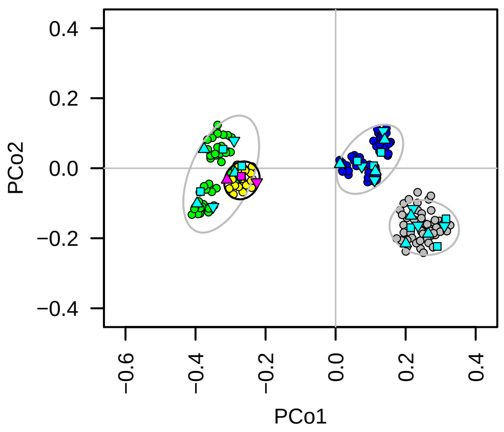

L1407 (m/z=906.587279; rt=7.65516)
T/S Cluster: L−7.7−1

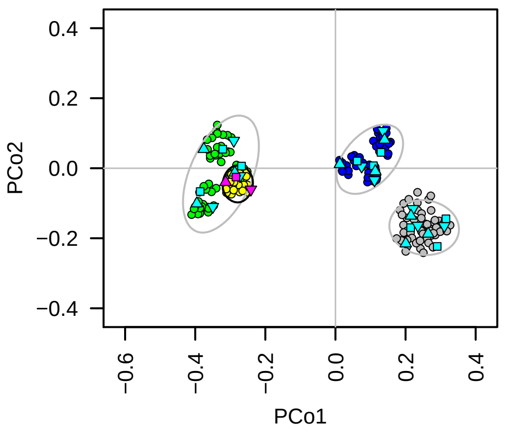

L1413 (m/z=905.536727; rt=7.65685)
T/S Cluster: L−7.7−1

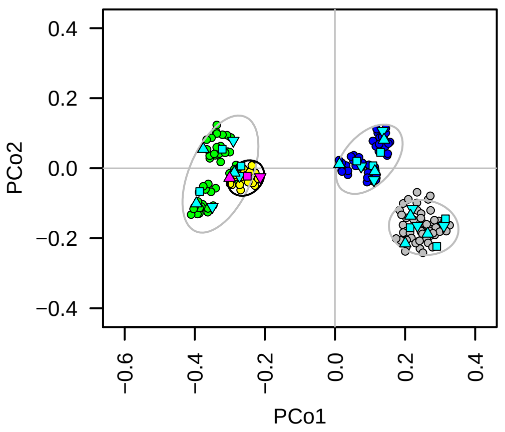

L1410 (m/z=906.550328; rt=7.65543)
T/S Cluster: L−7.7−1

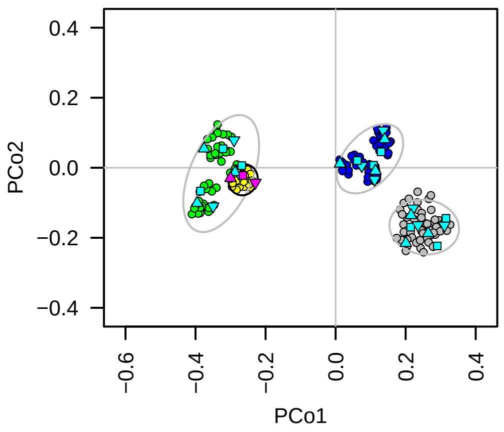

L1404 (m/z=907.589541; rt=7.65495)
T/S Cluster: L−7.7−1

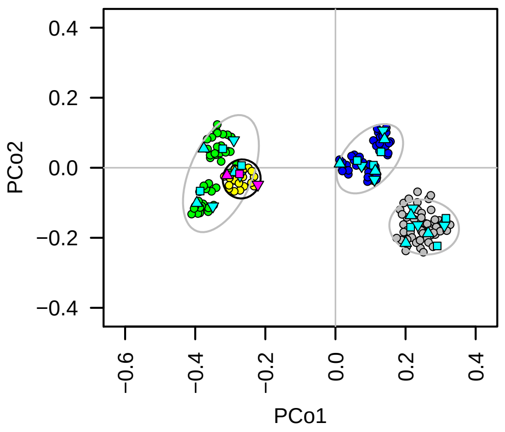

L1406 (m/z=452.791083; rt=7.65511)
T/S Cluster: L−7.7−1

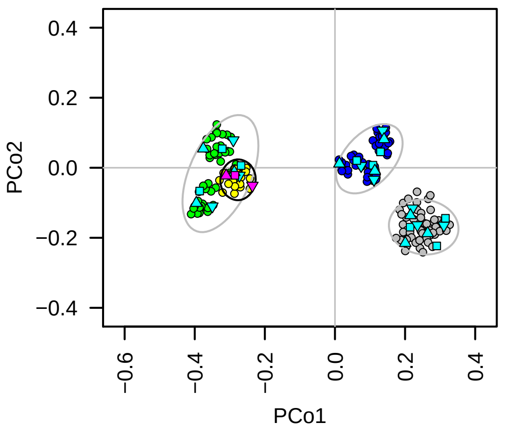

L1408 (m/z=452.795849; rt=7.65519)
T/S Cluster: L−7.7−1

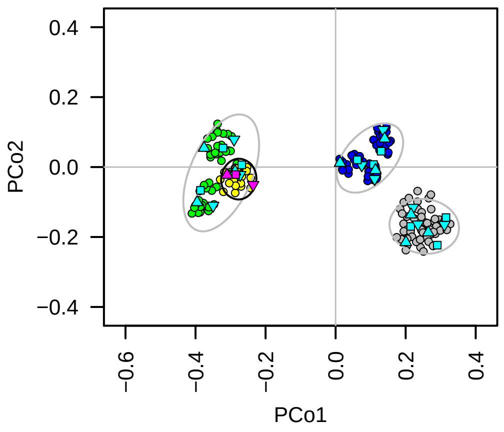

L1412 (m/z=452.792708; rt=7.65613)
T/S Cluster: L−7.7−1

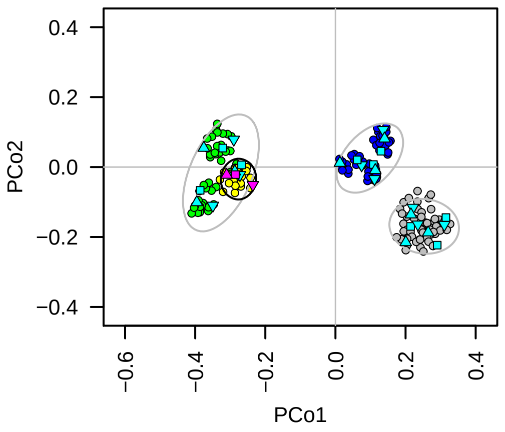

L1405 (m/z=907.548946; rt=7.6551)
T/S Cluster: L−7.7−1

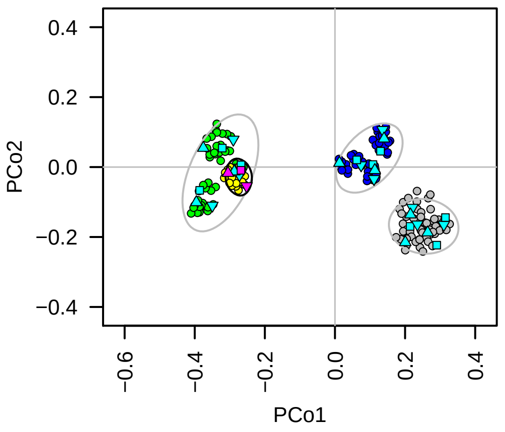

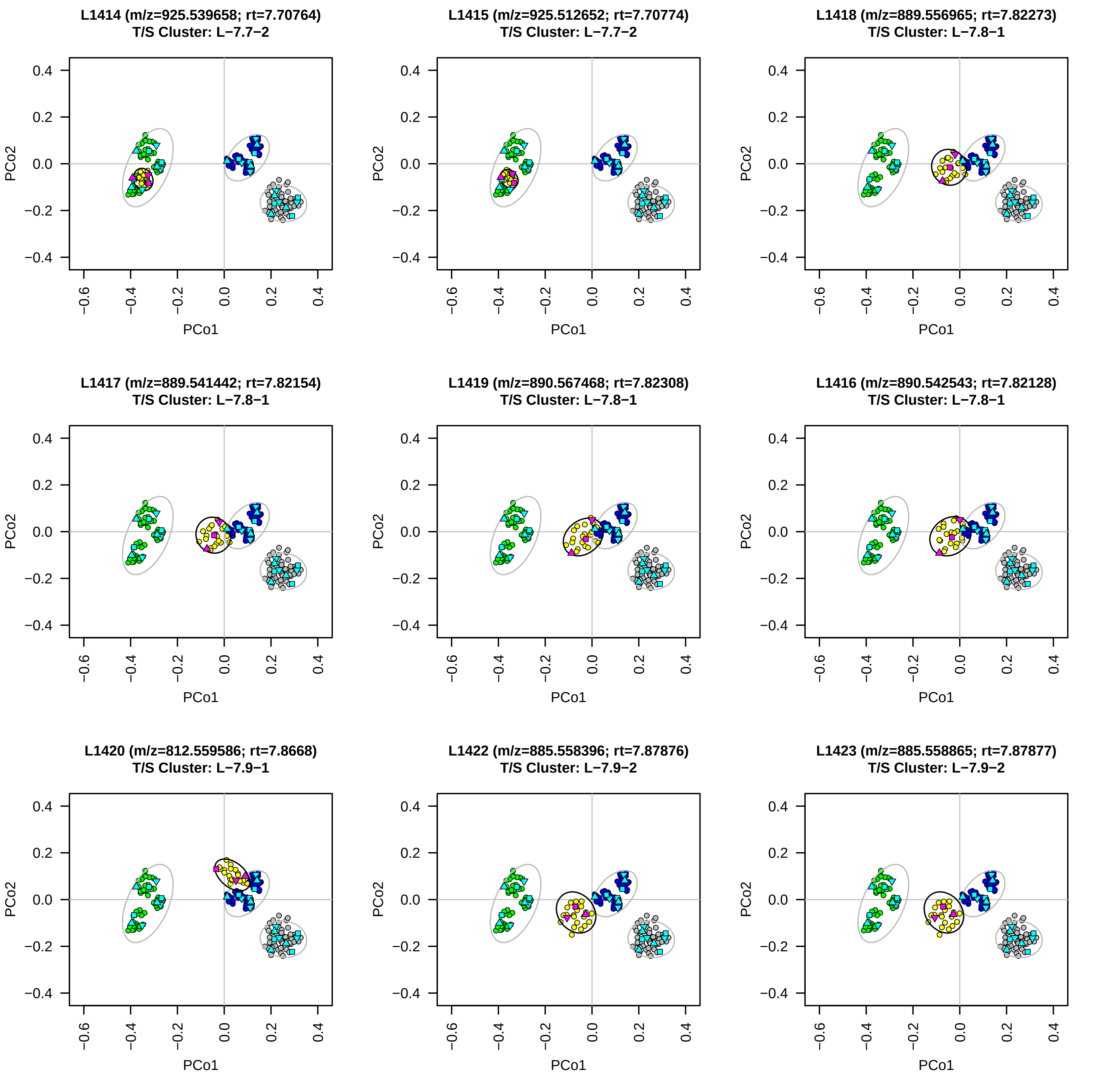
L1414 (m/z=925.539658; rt=7.70764)
T/S Cluster: L−7.7−2
PCo1
PCo2
L1415 (m/z=925.512652; rt=7.70774)
T/S Cluster: L−7.7−2
PCo1
PCo2
L1418 (m/z=889.556965; rt=7.82273)
T/S Cluster: L−7.8−1
PCo1
PCo2
L1417 (m/z=889.541442; rt=7.82154)
T/S Cluster: L−7.8−1
PCo1
PCo2
L1419 (m/z=890.567468; rt=7.82308)
T/S Cluster: L−7.8−1
PCo1
PCo2
L1416 (m/z=890.542543; rt=7.82128)
T/S Cluster: L−7.8−1
PCo1
PCo2
L1420 (m/z=812.559586; rt=7.8668)
T/S Cluster: L−7.9−1
PCo1
PCo2
L1422 (m/z=885.558396; rt=7.87876)
T/S Cluster: L−7.9−2
PCo1
PCo2
L1423 (m/z=885.558865; rt=7.87877)
T/S Cluster: L−7.9−2
PCo1
PCo2

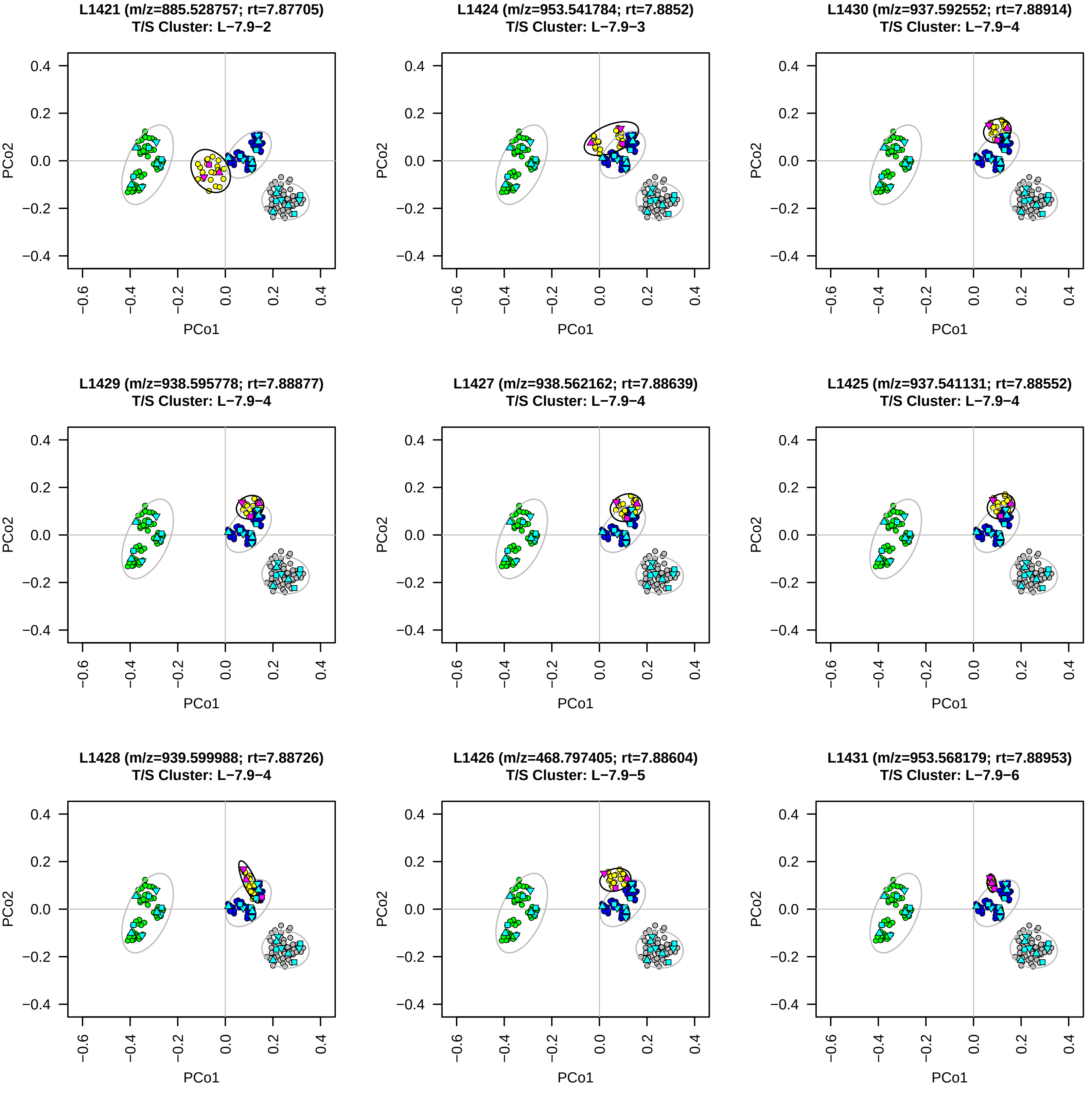
L1421 (m/z=885.528757; rt=7.87705)
T/S Cluster: L−7.9−2
L1424 (m/z=953.541784; rt=7.8852)
T/S Cluster: L−7.9−3
L1430 (m/z=937.592552; rt=7.88914)
T/S Cluster: L−7.9−4
L1429 (m/z=938.595778; rt=7.88877)
T/S Cluster: L−7.9−4
L1427 (m/z=938.562162; rt=7.88639)
T/S Cluster: L−7.9−4
L1425 (m/z=937.541131; rt=7.88552)
T/S Cluster: L−7.9−4
L1428 (m/z=939.599988; rt=7.88726)
T/S Cluster: L−7.9−4
L1426 (m/z=468.797405; rt=7.88604)
T/S Cluster: L−7.9−5
L1431 (m/z=953.568179; rt=7.88953)
T/S Cluster: L−7.9−6
PCo1
PCo2

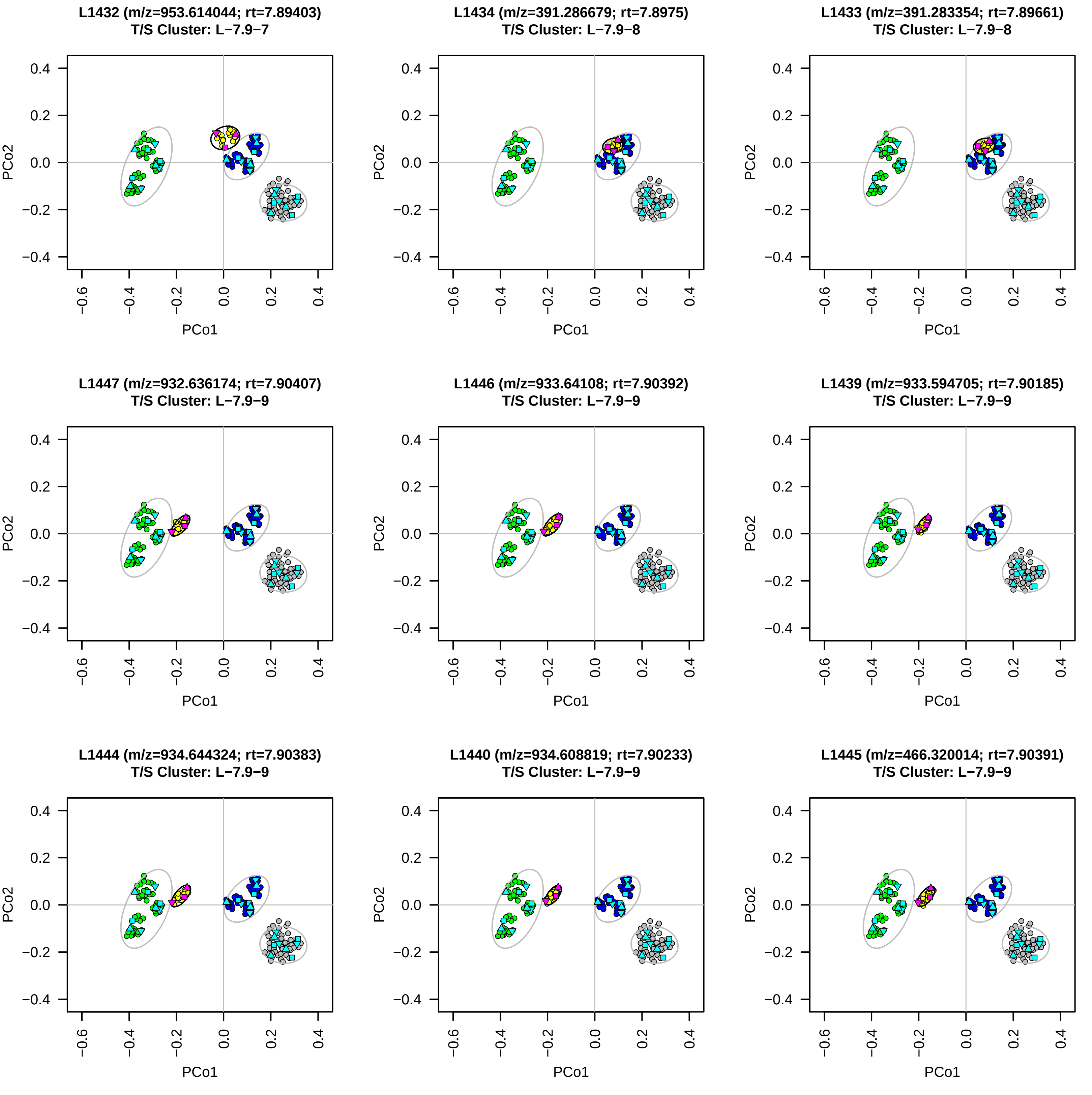
L1432 (m/z=953.614044; rt=7.89403)
T/S Cluster: L−7.9−7
L1434 (m/z=391.286679; rt=7.8975)
T/S Cluster: L−7.9−8
L1433 (m/z=391.283354; rt=7.89661)
T/S Cluster: L−7.9−8
L1447 (m/z=932.636174; rt=7.90407)
T/S Cluster: L−7.9−9
L1446 (m/z=933.64108; rt=7.90392)
T/S Cluster: L−7.9−9
L1439 (m/z=933.594705; rt=7.90185)
T/S Cluster: L−7.9−9
L1444 (m/z=934.644324; rt=7.90383)
T/S Cluster: L−7.9−9
L1440 (m/z=934.608819; rt=7.90233)
T/S Cluster: L−7.9−9
L1445 (m/z=466.320014; rt=7.90391)
T/S Cluster: L−7.9−9
PCo1
PCo2
−0.6
−0.4
−0.2
0.0
0.2
0.4

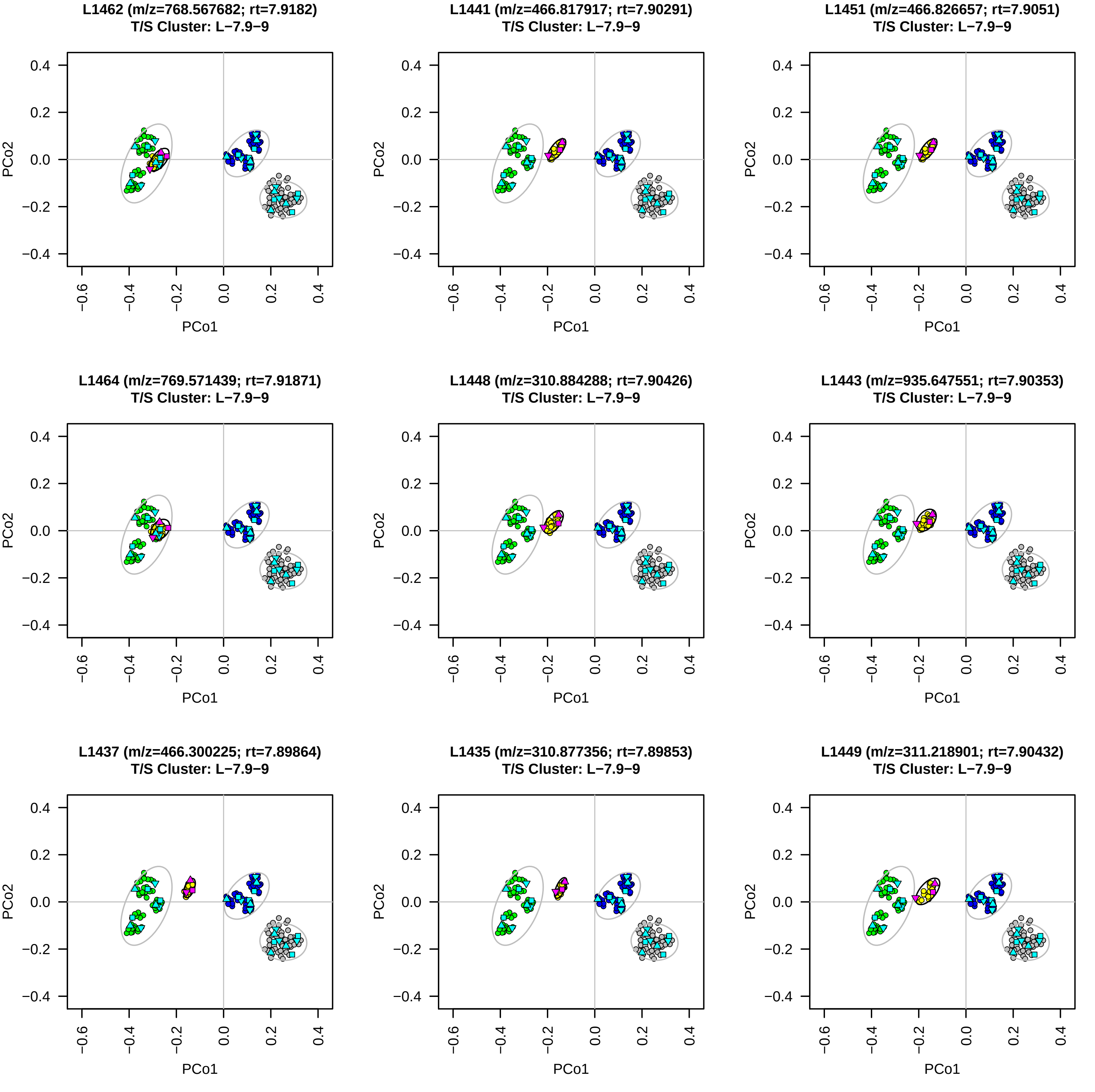

L1462 (m/z=768.567682; rt=7.9182)
T/S Cluster: L−7.9−9
L1441 (m/z=466.817917; rt=7.90291)
T/S Cluster: L−7.9−9
L1451 (m/z=466.826657; rt=7.9051)
T/S Cluster: L−7.9−9
L1464 (m/z=769.571439; rt=7.91871)
T/S Cluster: L−7.9−9
L1448 (m/z=310.884288; rt=7.90426)
T/S Cluster: L−7.9−9
L1443 (m/z=935.647551; rt=7.90353)
T/S Cluster: L−7.9−9
L1437 (m/z=466.300225; rt=7.89864)
T/S Cluster: L−7.9−9
L1435 (m/z=310.877356; rt=7.89853)
T/S Cluster: L−7.9−9
L1449 (m/z=311.218901; rt=7.90432)
T/S Cluster: L−7.9−9
PCo1
PCo2

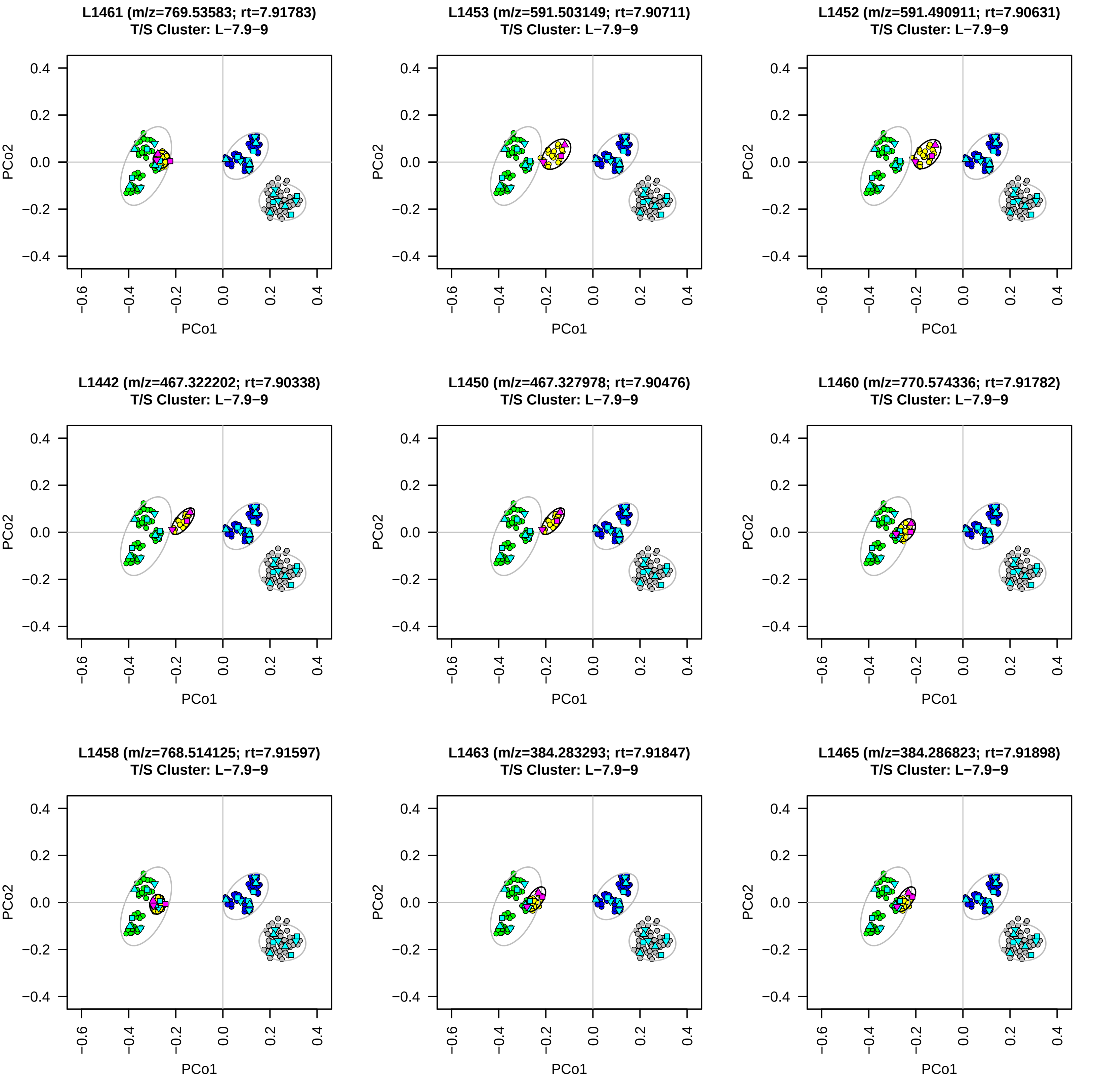
L1461 (m/z=769.53583; rt=7.91783)
T/S Cluster: L−7.9−9
L1453 (m/z=591.503149; rt=7.90711)
T/S Cluster: L−7.9−9
L1452 (m/z=591.490911; rt=7.90631)
T/S Cluster: L−7.9−9
L1442 (m/z=467.322202; rt=7.90338)
T/S Cluster: L−7.9−9
L1450 (m/z=467.327978; rt=7.90476)
T/S Cluster: L−7.9−9
L1460 (m/z=770.574336; rt=7.91782)
T/S Cluster: L−7.9−9
L1458 (m/z=768.514125; rt=7.91597)
T/S Cluster: L−7.9−9
L1463 (m/z=384.283293; rt=7.91847)
T/S Cluster: L−7.9−9
L1465 (m/z=384.286823; rt=7.91898)
T/S Cluster: L−7.9−9
PCo1
PCo2
0.4
0.2
0.0
−0.2
−0.4
−0.6

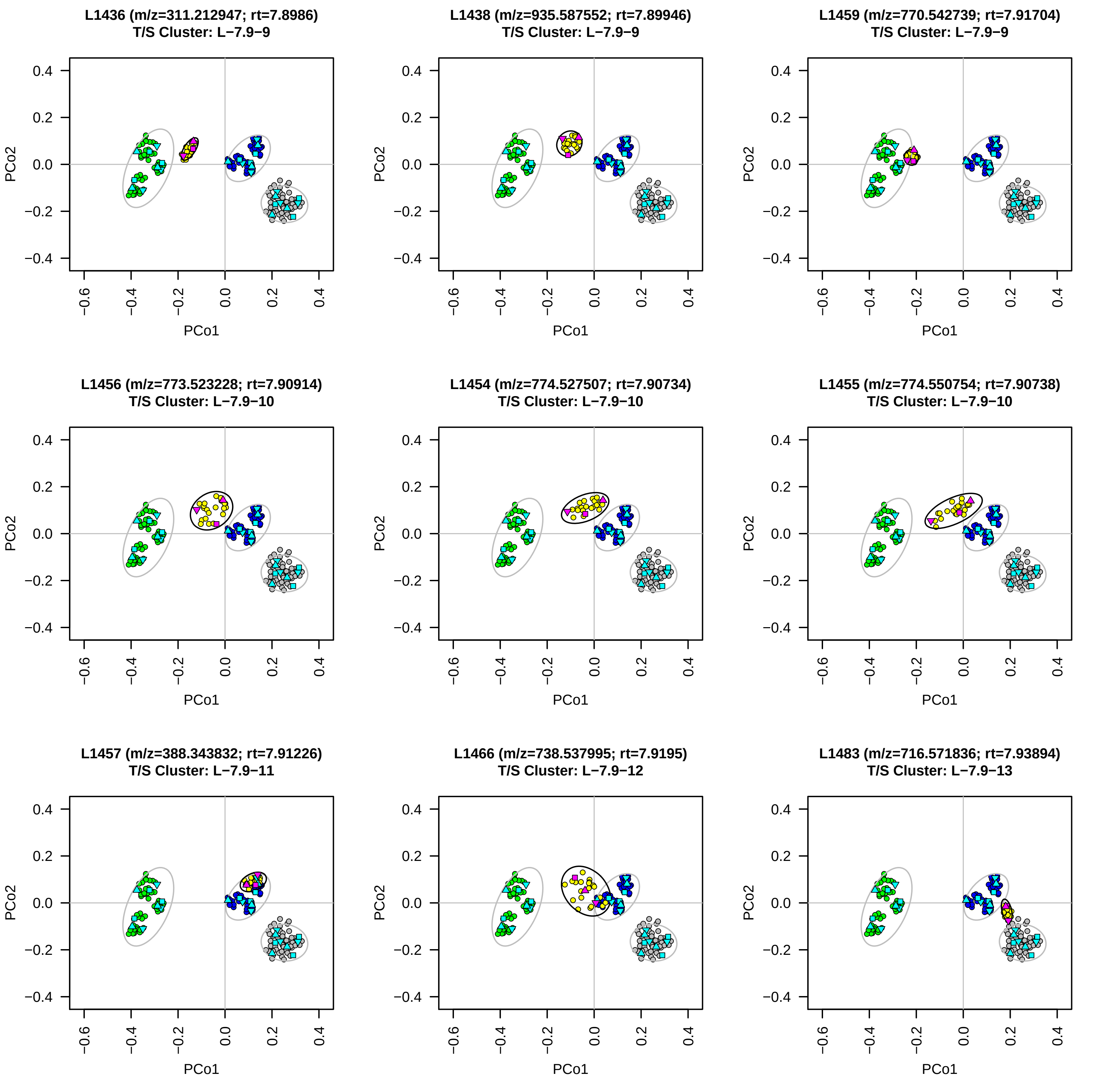
L1436 (m/z=311.212947; rt=7.8986)
T/S Cluster: L−7.9−9
L1438 (m/z=935.587552; rt=7.89946)
T/S Cluster: L−7.9−9
L1459 (m/z=770.542739; rt=7.91704)
T/S Cluster: L−7.9−9
L1456 (m/z=773.523228; rt=7.90914)
T/S Cluster: L−7.9−10
L1454 (m/z=774.527507; rt=7.90734)
T/S Cluster: L−7.9−10
L1455 (m/z=774.550754; rt=7.90738)
T/S Cluster: L−7.9−10
L1457 (m/z=388.343832; rt=7.91226)
T/S Cluster: L−7.9−11
L1466 (m/z=738.537995; rt=7.9195)
T/S Cluster: L−7.9−12
L1483 (m/z=716.571836; rt=7.93894)
T/S Cluster: L−7.9−13
PCo1
PCo2
0.4
0.2
0.0
−0.2
−0.4
−0.6

L1467 (m/z=716.541497; rt=7.93134)
T/S Cluster: L−7.9−13

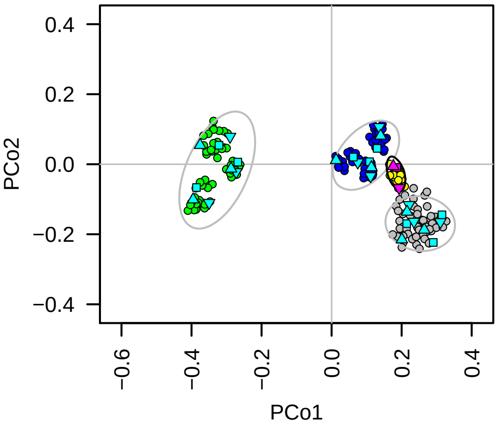

L1473 (m/z=717.576433; rt=7.93871)
T/S Cluster: L−7.9−13

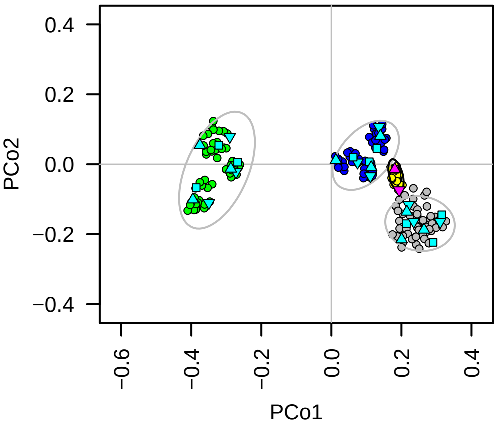

L1474 (m/z=717.55087; rt=7.93873)
T/S Cluster: L−7.9−13

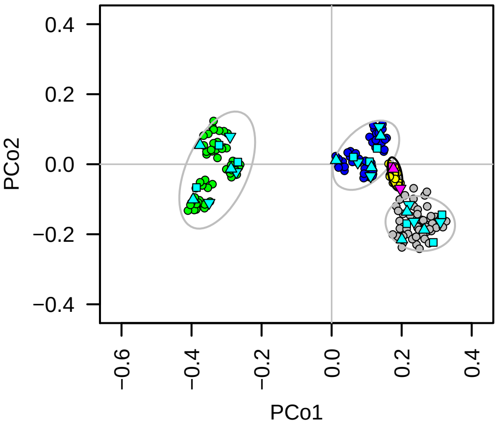

L1468 (m/z=716.525973; rt=7.93437)
T/S Cluster: L−7.9−13

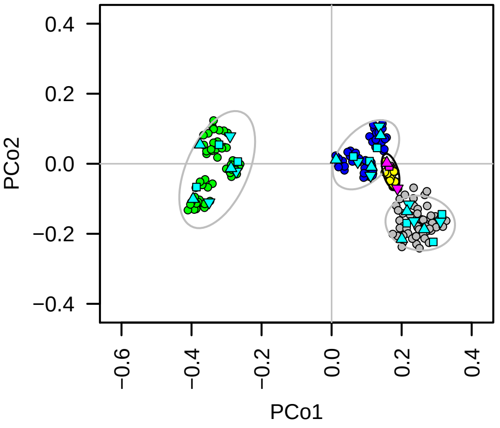

L1492 (m/z=358.288817; rt=7.93915)
T/S Cluster: L−7.9−13

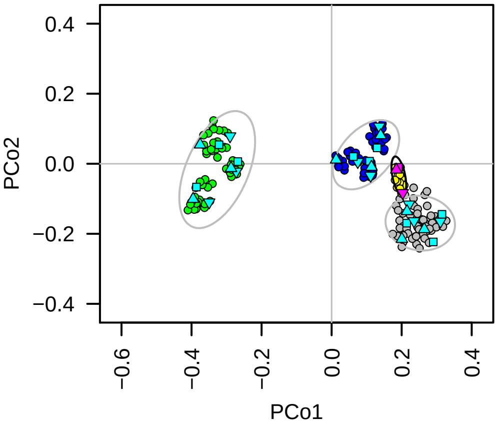

L1500 (m/z=358.284075; rt=7.93941)
T/S Cluster: L−7.9−13

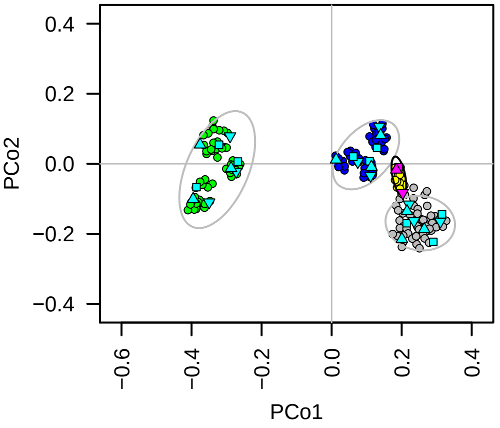

L1479 (m/z=718.580796; rt=7.93884)
T/S Cluster: L−7.9−13

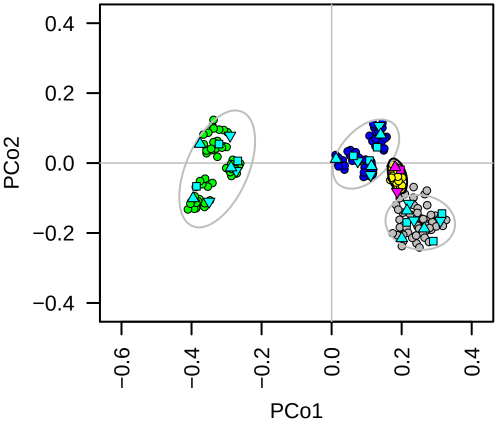

L1478 (m/z=718.562552; rt=7.93883)
T/S Cluster: L−7.9−13

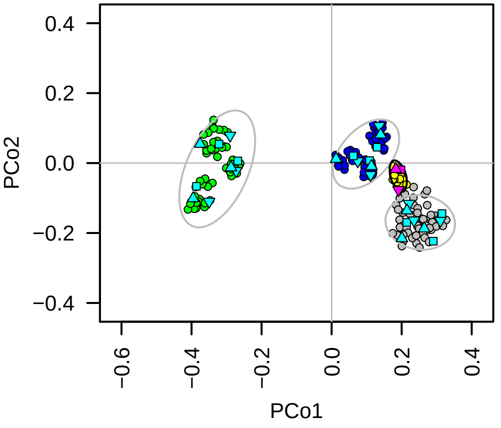

L1485 (m/z=358.792133; rt=7.93903)
T/S Cluster: L−7.9−13

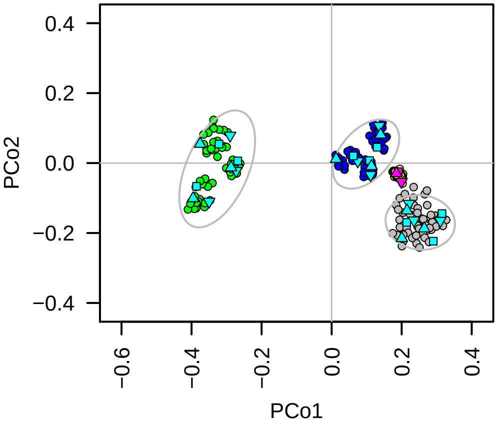

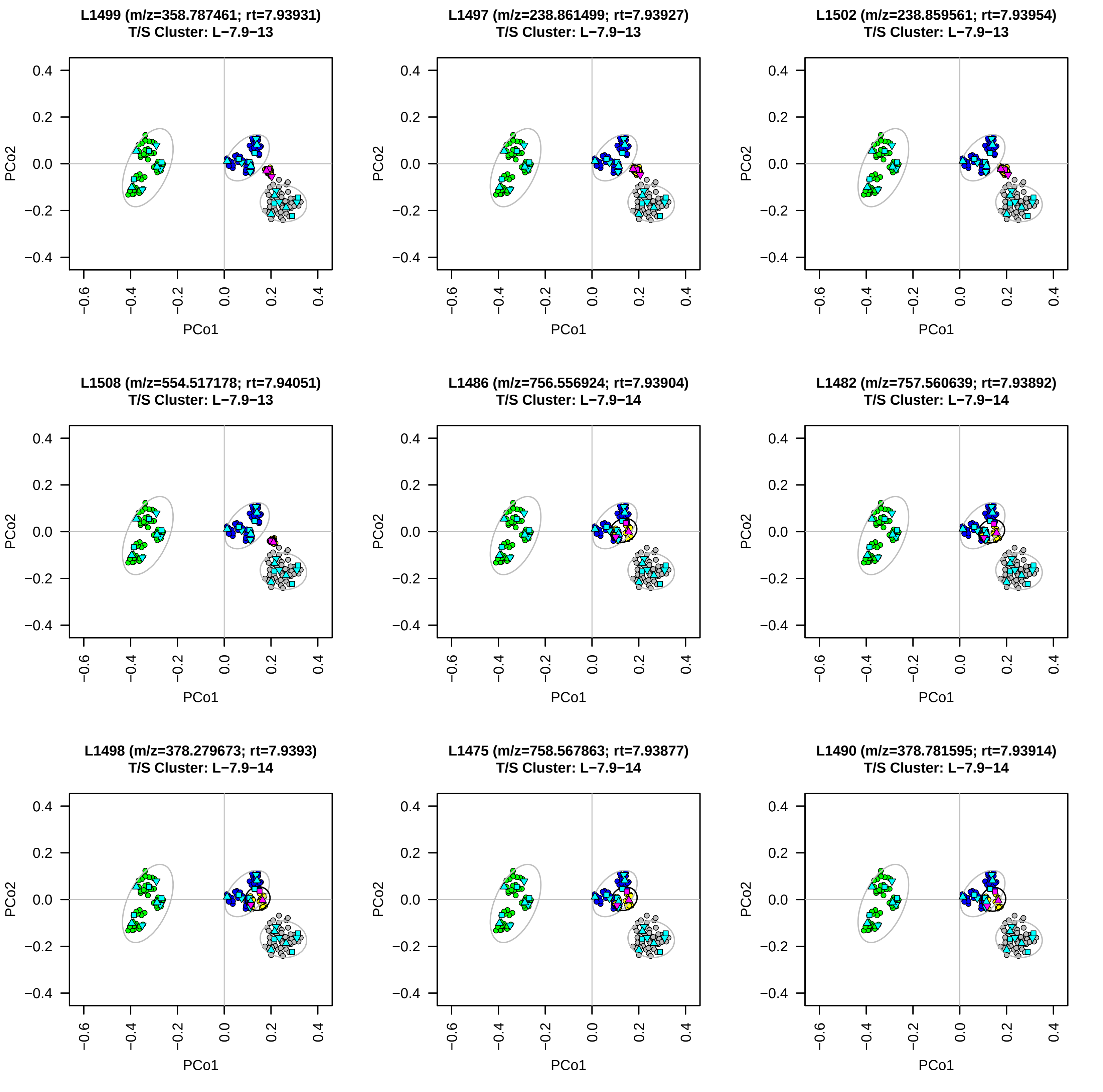
L1499 (m/z=358.787461; rt=7.93931)
T/S Cluster: L−7.9−13
L1497 (m/z=238.861499; rt=7.93927)
T/S Cluster: L−7.9−13
L1502 (m/z=238.859561; rt=7.93954)
T/S Cluster: L−7.9−13
L1508 (m/z=554.517178; rt=7.94051)
T/S Cluster: L−7.9−13
L1486 (m/z=756.556924; rt=7.93904)
T/S Cluster: L−7.9−14
L1482 (m/z=757.560639; rt=7.93892)
T/S Cluster: L−7.9−14
L1498 (m/z=378.279673; rt=7.9393)
T/S Cluster: L−7.9−14
L1475 (m/z=758.567863; rt=7.93877)
T/S Cluster: L−7.9−14
L1490 (m/z=378.781595; rt=7.93914)
T/S Cluster: L−7.9−14
PCo1
PCo2
−0.6
−0.4
−0.2
0.0
0.2
0.4

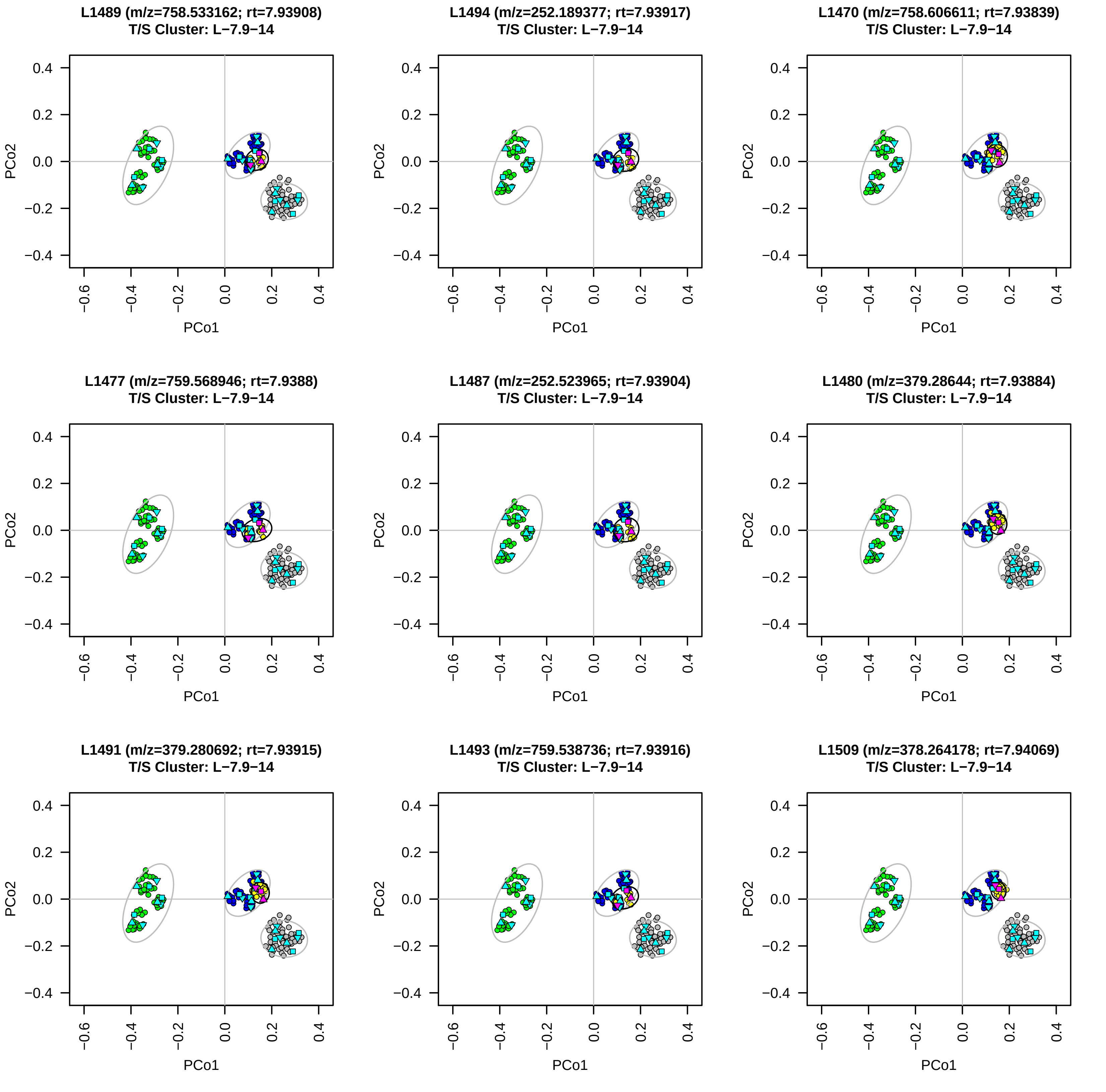
L1489 (m/z=758.533162; rt=7.93908)
T/S Cluster: L−7.9−14
L1494 (m/z=252.189377; rt=7.93917)
T/S Cluster: L−7.9−14
L1470 (m/z=758.606611; rt=7.93839)
T/S Cluster: L−7.9−14
L1477 (m/z=759.568946; rt=7.9388)
T/S Cluster: L−7.9−14
L1487 (m/z=252.523965; rt=7.93904)
T/S Cluster: L−7.9−14
L1480 (m/z=379.28644; rt=7.93884)
T/S Cluster: L−7.9−14
L1491 (m/z=379.280692; rt=7.93915)
T/S Cluster: L−7.9−14
L1493 (m/z=759.538736; rt=7.93916)
T/S Cluster: L−7.9−14
L1509 (m/z=378.264178; rt=7.94069)
T/S Cluster: L−7.9−14
PCo1
PCo2
0.4
0.2
0.0
−0.2
−0.4
−0.6

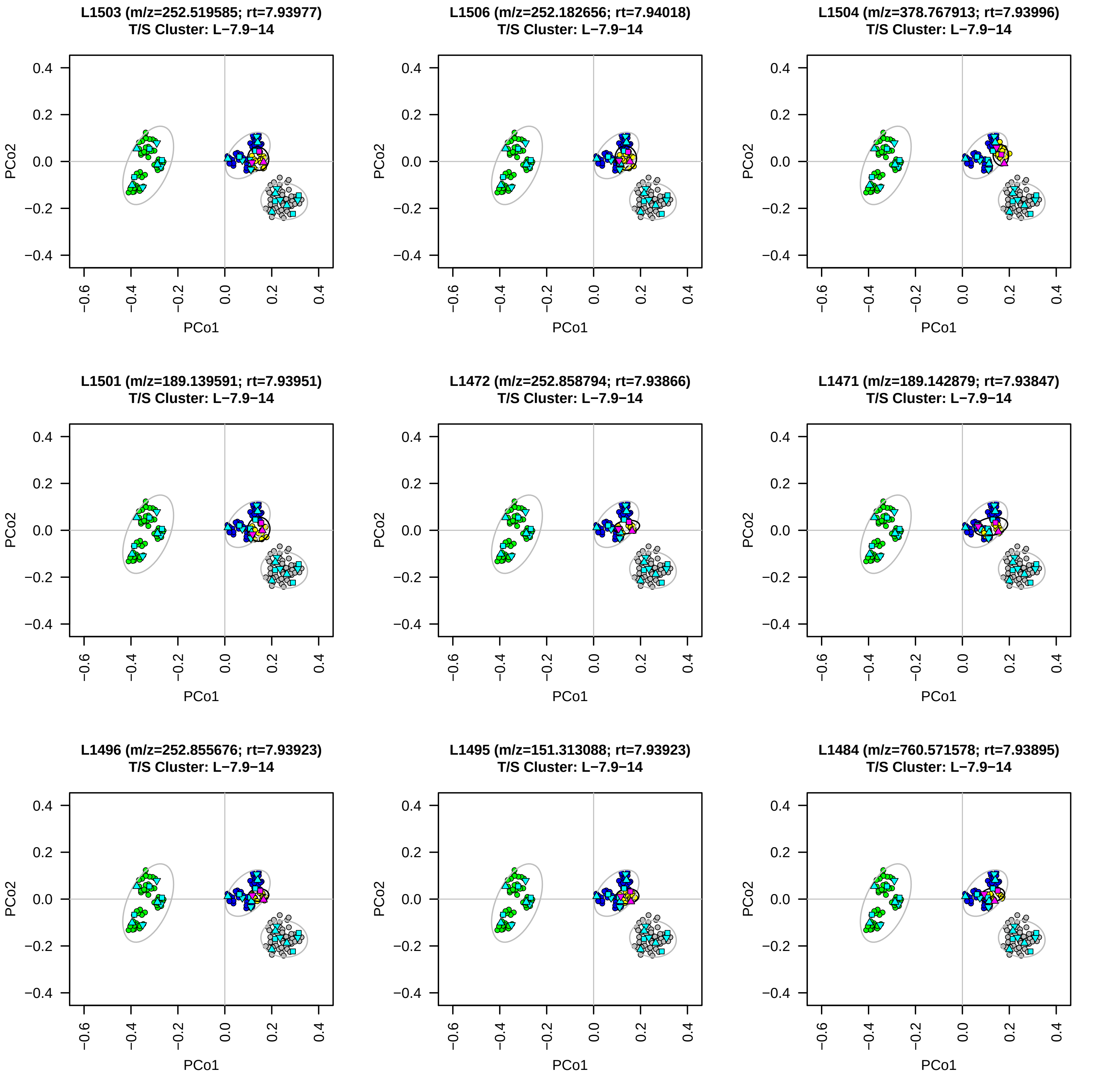
L1503 (m/z=252.519585; rt=7.93977)
T/S Cluster: L−7.9−14
L1506 (m/z=252.182656; rt=7.94018)
T/S Cluster: L−7.9−14
L1504 (m/z=378.767913; rt=7.93996)
T/S Cluster: L−7.9−14
L1501 (m/z=189.139591; rt=7.93951)
T/S Cluster: L−7.9−14
L1472 (m/z=252.858794; rt=7.93866)
T/S Cluster: L−7.9−14
L1471 (m/z=189.142879; rt=7.93847)
T/S Cluster: L−7.9−14
L1496 (m/z=252.855676; rt=7.93923)
T/S Cluster: L−7.9−14
L1495 (m/z=151.313088; rt=7.93923)
T/S Cluster: L−7.9−14
L1484 (m/z=760.571578; rt=7.93895)
T/S Cluster: L−7.9−14
PCo1
PCo2
0.4
0.2
0.0
−0.2
−0.4
−0.6
−0.4
−0.2
0.0
0.2
0.4

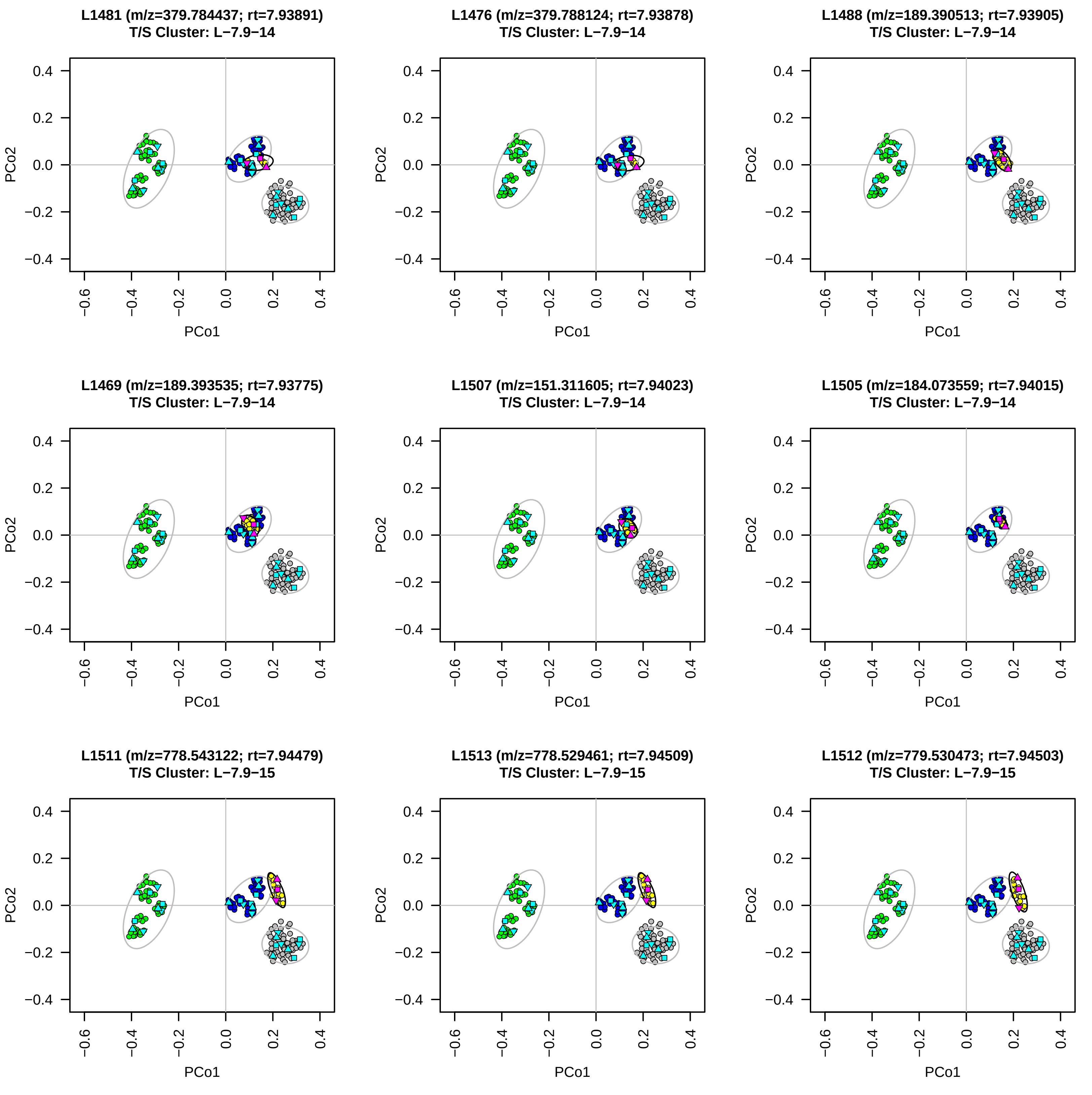
L1481 (m/z=379.784437; rt=7.93891)
T/S Cluster: L−7.9−14
L1476 (m/z=379.788124; rt=7.93878)
T/S Cluster: L−7.9−14
L1488 (m/z=189.390513; rt=7.93905)
T/S Cluster: L−7.9−14
L1469 (m/z=189.393535; rt=7.93775)
T/S Cluster: L−7.9−14
L1507 (m/z=151.311605; rt=7.94023)
T/S Cluster: L−7.9−14
L1505 (m/z=184.073559; rt=7.94015)
T/S Cluster: L−7.9−14
L1511 (m/z=778.543122; rt=7.94479)
T/S Cluster: L−7.9−15
L1513 (m/z=778.529461; rt=7.94509)
T/S Cluster: L−7.9−15
L1512 (m/z=779.530473; rt=7.94503)
T/S Cluster: L−7.9−15
PCo1
PCo2
−0.6
−0.4
−0.2
0.0
0.2
0.4

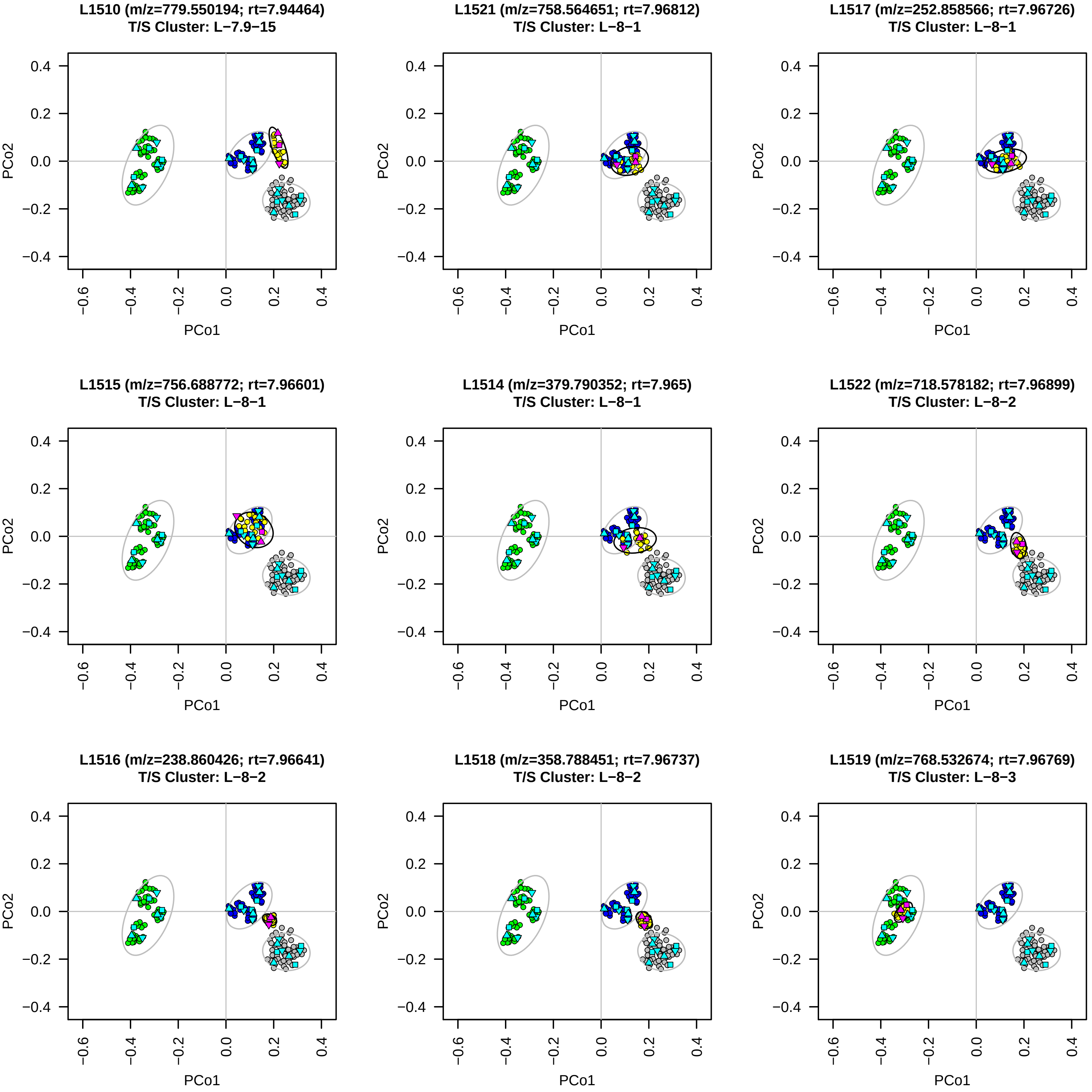
L1510 (m/z=779.550194; rt=7.94464)
T/S Cluster: L−7.9−15
L1521 (m/z=758.564651; rt=7.96812)
T/S Cluster: L−8−1
L1517 (m/z=252.858566; rt=7.96726)
T/S Cluster: L−8−1
L1515 (m/z=756.688772; rt=7.96601)
T/S Cluster: L−8−1
L1514 (m/z=379.790352; rt=7.965)
T/S Cluster: L−8−1
L1522 (m/z=718.578182; rt=7.96899)
T/S Cluster: L−8−2
L1516 (m/z=238.860426; rt=7.96641)
T/S Cluster: L−8−2
L1518 (m/z=358.788451; rt=7.96737)
T/S Cluster: L−8−2
L1519 (m/z=768.532674; rt=7.96769)
T/S Cluster: L−8−3
PCo1
PCo2
−0.6
−0.4
−0.2
0.0
0.2
0.4

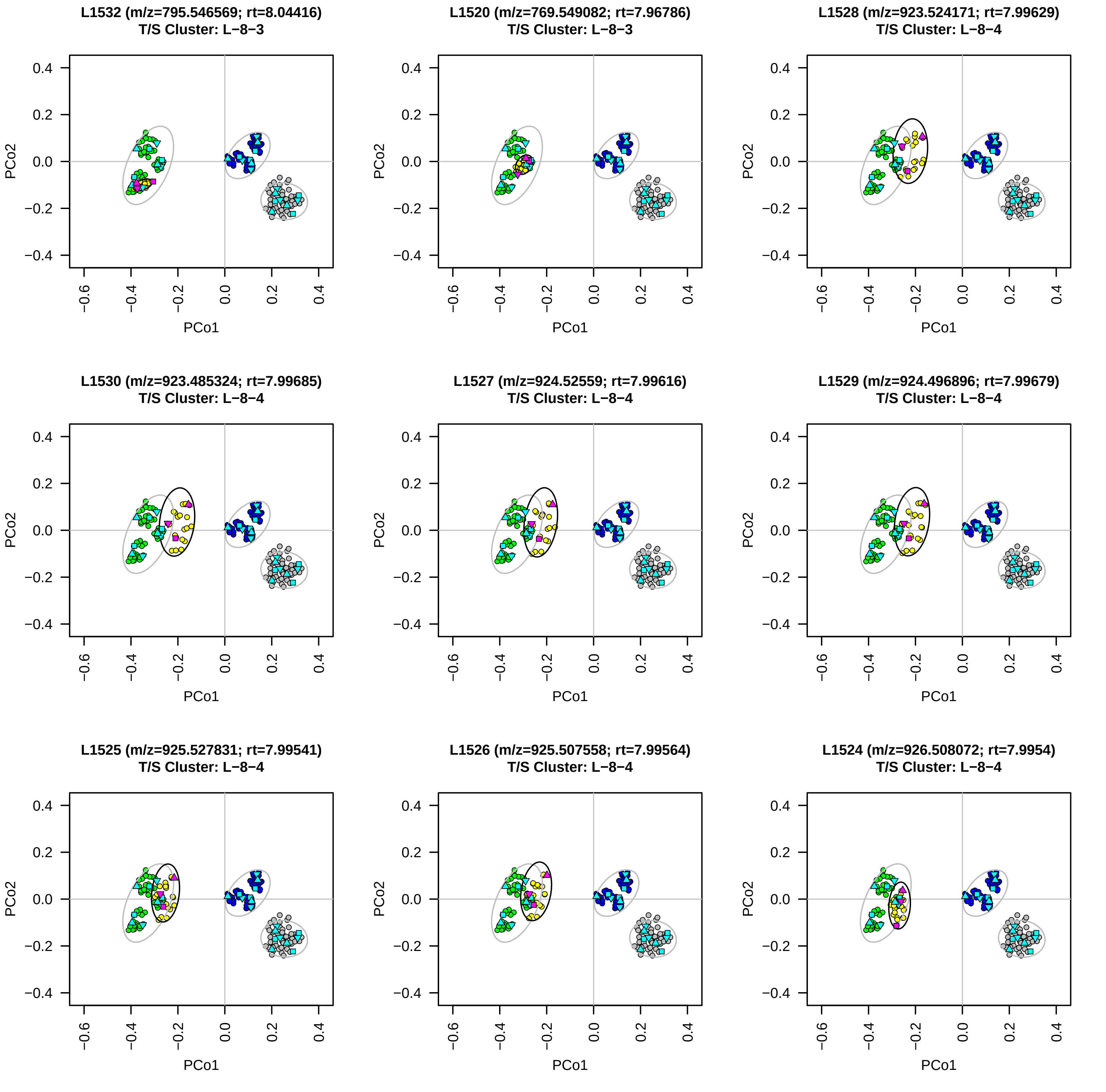
L1532 (m/z=795.546569; rt=8.04416)
T/S Cluster: L−8−3
L1520 (m/z=769.549082; rt=7.96786)
T/S Cluster: L−8−3
L1528 (m/z=923.524171; rt=7.99629)
T/S Cluster: L−8−4
L1530 (m/z=923.485324; rt=7.99685)
T/S Cluster: L−8−4
L1527 (m/z=924.52559; rt=7.99616)
T/S Cluster: L−8−4
L1529 (m/z=924.496896; rt=7.99679)
T/S Cluster: L−8−4
L1525 (m/z=925.527831; rt=7.99541)
T/S Cluster: L−8−4
L1526 (m/z=925.507558; rt=7.99564)
T/S Cluster: L−8−4
L1524 (m/z=926.508072; rt=7.9954)
T/S Cluster: L−8−4
PCo1
PCo2
−0.6
−0.4
−0.2
0.0
0.2
0.4

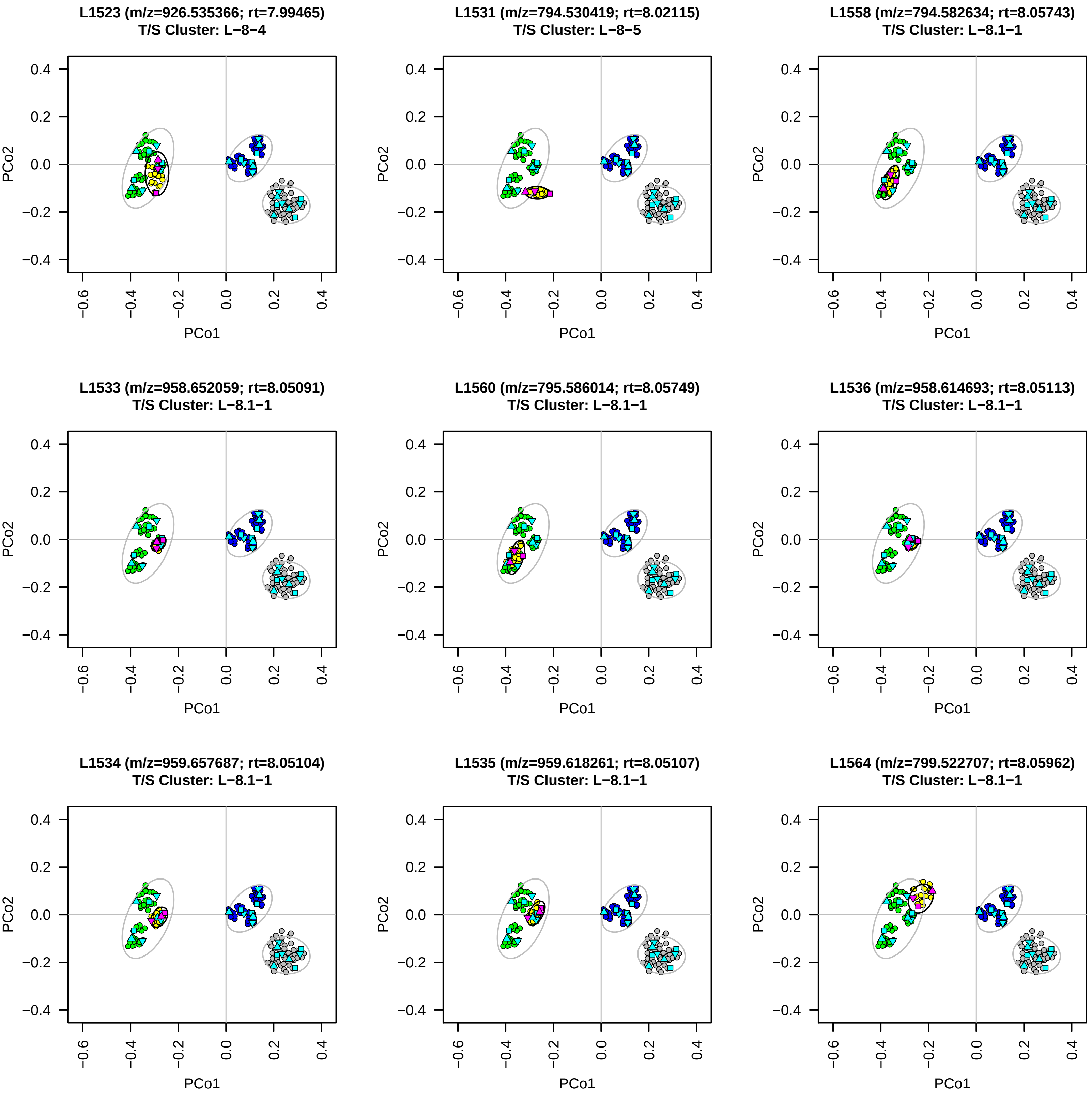
L1523 (m/z=926.535366; rt=7.99465)
T/S Cluster: L−8−4
L1531 (m/z=794.530419; rt=8.02115)
T/S Cluster: L−8−5
L1558 (m/z=794.582634; rt=8.05743)
T/S Cluster: L−8.1−1
L1533 (m/z=958.652059; rt=8.05091)
T/S Cluster: L−8.1−1
L1560 (m/z=795.586014; rt=8.05749)
T/S Cluster: L−8.1−1
L1536 (m/z=958.614693; rt=8.05113)
T/S Cluster: L−8.1−1
L1534 (m/z=959.657687; rt=8.05104)
T/S Cluster: L−8.1−1
L1535 (m/z=959.618261; rt=8.05107)
T/S Cluster: L−8.1−1
L1564 (m/z=799.522707; rt=8.05962)
T/S Cluster: L−8.1−1
PCo1
PCo2
0.4
0.2
0.0
−0.2
−0.4
−0.6

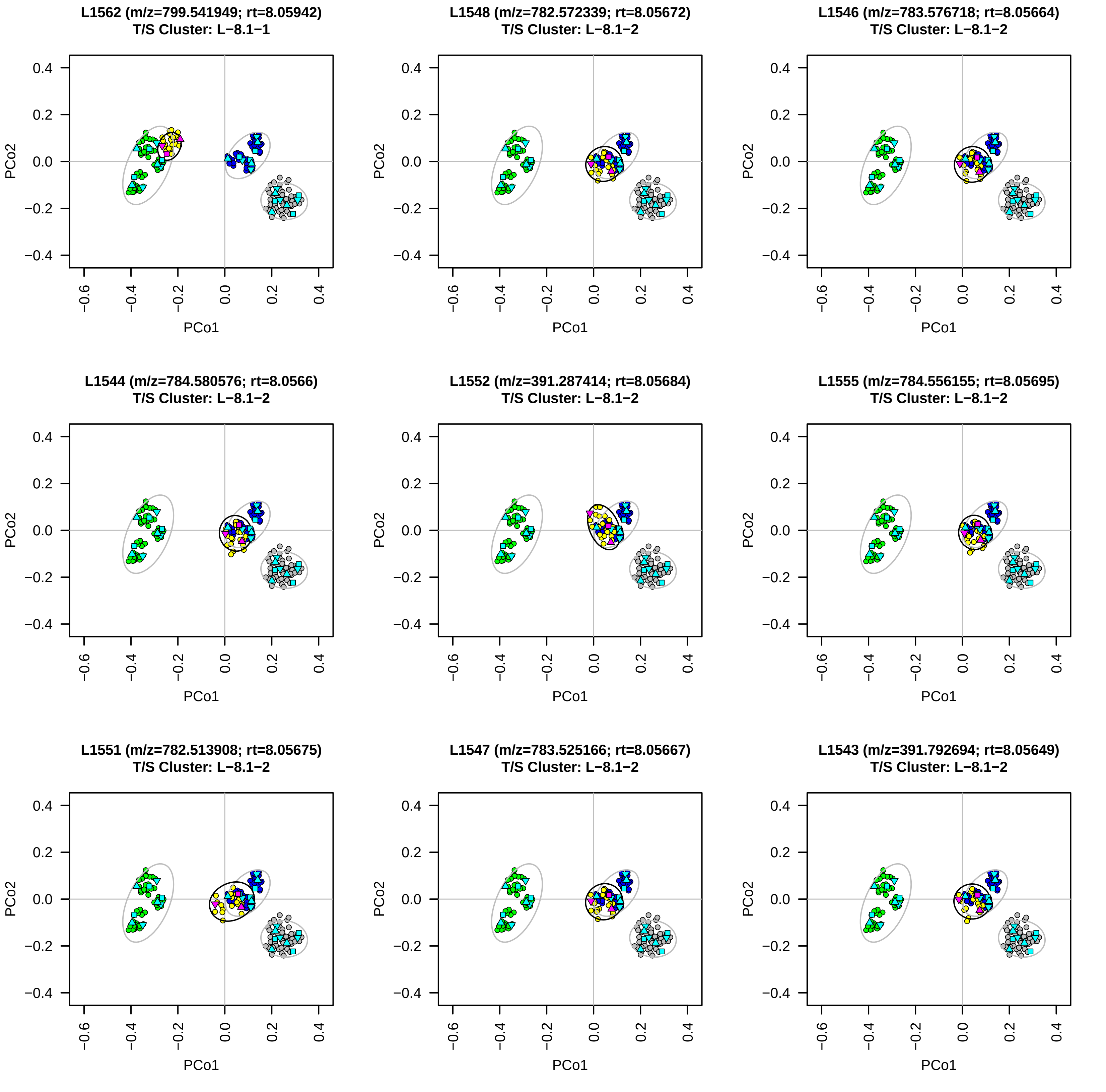
L1562 (m/z=799.541949; rt=8.05942)
T/S Cluster: L−8.1−1
L1548 (m/z=782.572339; rt=8.05672)
T/S Cluster: L−8.1−2
L1546 (m/z=783.576718; rt=8.05664)
T/S Cluster: L−8.1−2
L1544 (m/z=784.580576; rt=8.0566)
T/S Cluster: L−8.1−2
L1552 (m/z=391.287414; rt=8.05684)
T/S Cluster: L−8.1−2
L1555 (m/z=784.556155; rt=8.05695)
T/S Cluster: L−8.1−2
L1551 (m/z=782.513908; rt=8.05675)
T/S Cluster: L−8.1−2
L1547 (m/z=783.525166; rt=8.05667)
T/S Cluster: L−8.1−2
L1543 (m/z=391.792694; rt=8.05649)
T/S Cluster: L−8.1−2
PCo1
PCo2
−0.6
−0.4
−0.2
0.0
0.2
0.4

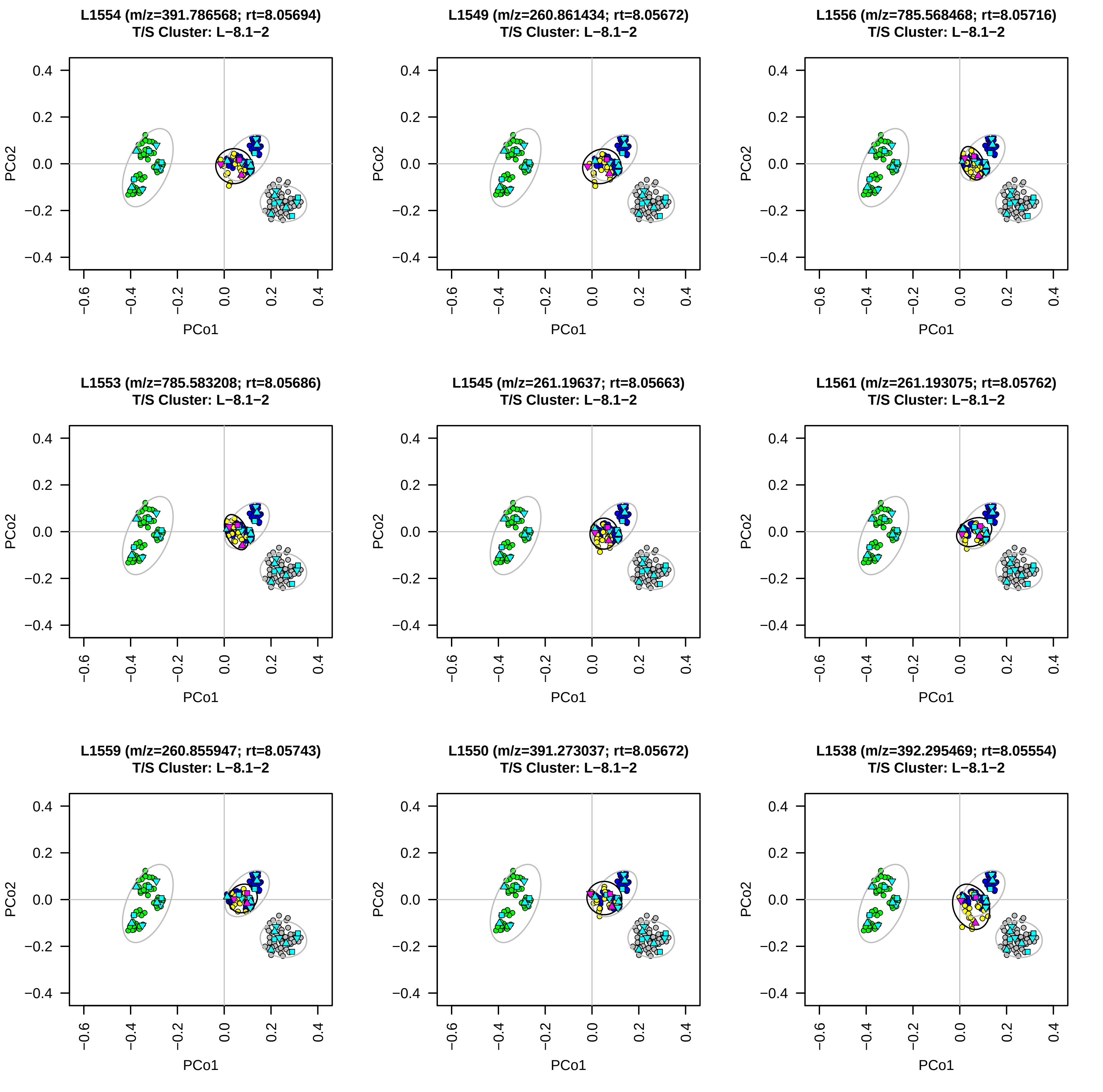
L1554 (m/z=391.786568; rt=8.05694)
T/S Cluster: L−8.1−2
L1549 (m/z=260.861434; rt=8.05672)
T/S Cluster: L−8.1−2
L1556 (m/z=785.568468; rt=8.05716)
T/S Cluster: L−8.1−2
L1553 (m/z=785.583208; rt=8.05686)
T/S Cluster: L−8.1−2
L1545 (m/z=261.19637; rt=8.05663)
T/S Cluster: L−8.1−2
L1561 (m/z=261.193075; rt=8.05762)
T/S Cluster: L−8.1−2
L1559 (m/z=260.855947; rt=8.05743)
T/S Cluster: L−8.1−2
L1550 (m/z=391.273037; rt=8.05672)
T/S Cluster: L−8.1−2
L1538 (m/z=392.295469; rt=8.05554)
T/S Cluster: L−8.1−2
PCo1
PCo2
−0.6
−0.4
−0.2
0.0
0.2
0.4

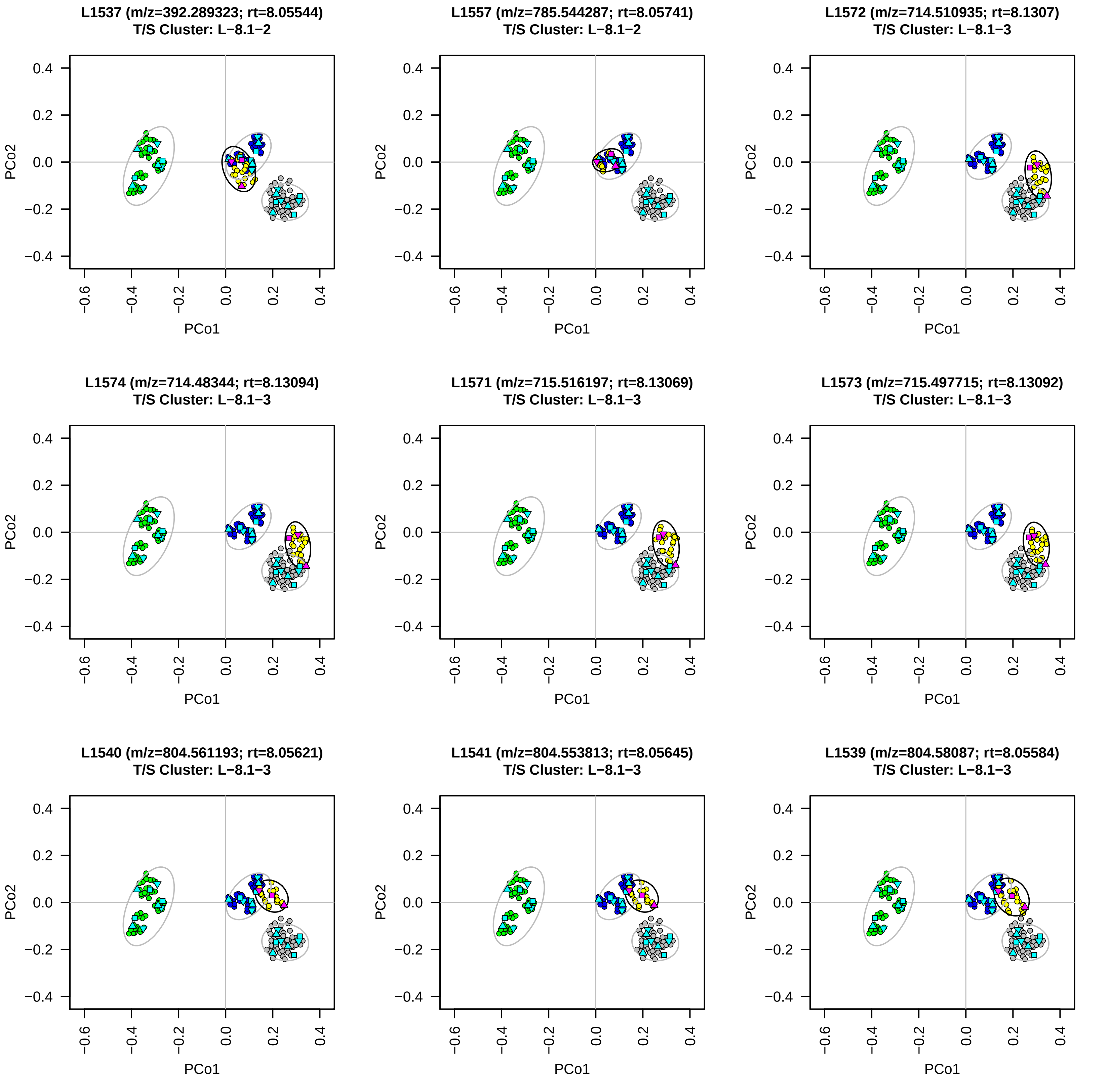
L1537 (m/z=392.289323; rt=8.05544)
T/S Cluster: L−8.1−2
L1557 (m/z=785.544287; rt=8.05741)
T/S Cluster: L−8.1−2
L1572 (m/z=714.510935; rt=8.1307)
T/S Cluster: L−8.1−3
L1574 (m/z=714.48344; rt=8.13094)
T/S Cluster: L−8.1−3
L1571 (m/z=715.516197; rt=8.13069)
T/S Cluster: L−8.1−3
L1573 (m/z=715.497715; rt=8.13092)
T/S Cluster: L−8.1−3
L1540 (m/z=804.561193; rt=8.05621)
T/S Cluster: L−8.1−3
L1541 (m/z=804.553813; rt=8.05645)
T/S Cluster: L−8.1−3
L1539 (m/z=804.58087; rt=8.05584)
T/S Cluster: L−8.1−3
PCo1
PCo2

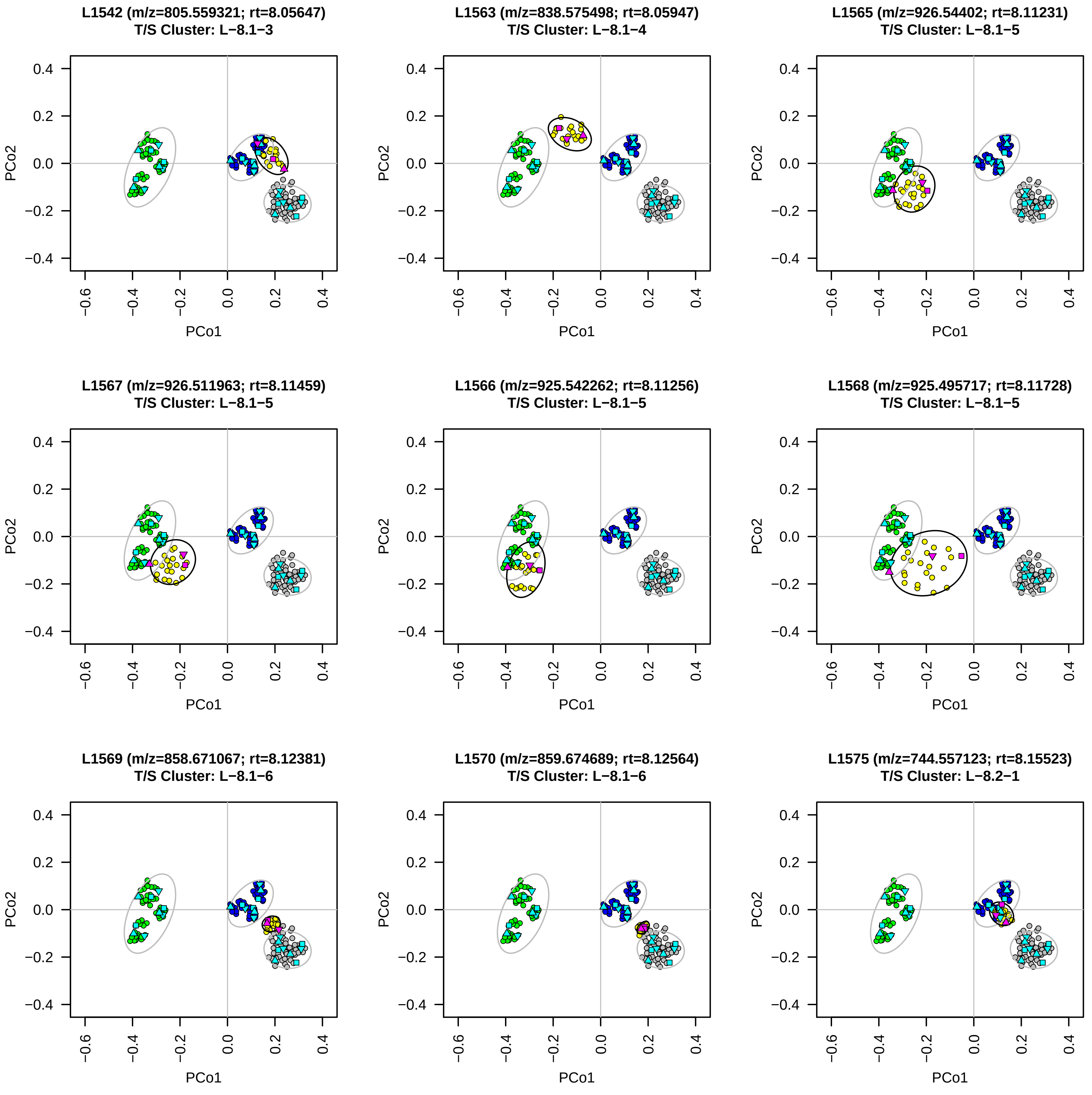
L1542 (m/z=805.559321; rt=8.05647)
T/S Cluster: L−8.1−3
L1563 (m/z=838.575498; rt=8.05947)
T/S Cluster: L−8.1−4
L1565 (m/z=926.54402; rt=8.11231)
T/S Cluster: L−8.1−5
L1567 (m/z=926.511963; rt=8.11459)
T/S Cluster: L−8.1−5
L1566 (m/z=925.542262; rt=8.11256)
T/S Cluster: L−8.1−5
L1568 (m/z=925.495717; rt=8.11728)
T/S Cluster: L−8.1−5
L1569 (m/z=858.671067; rt=8.12381)
T/S Cluster: L−8.1−6
L1570 (m/z=859.674689; rt=8.12564)
T/S Cluster: L−8.1−6
L1575 (m/z=744.557123; rt=8.15523)
T/S Cluster: L−8.2−1
PCo1
PCo2
0.4
0.2
0.0
−0.2
−0.4
−0.6

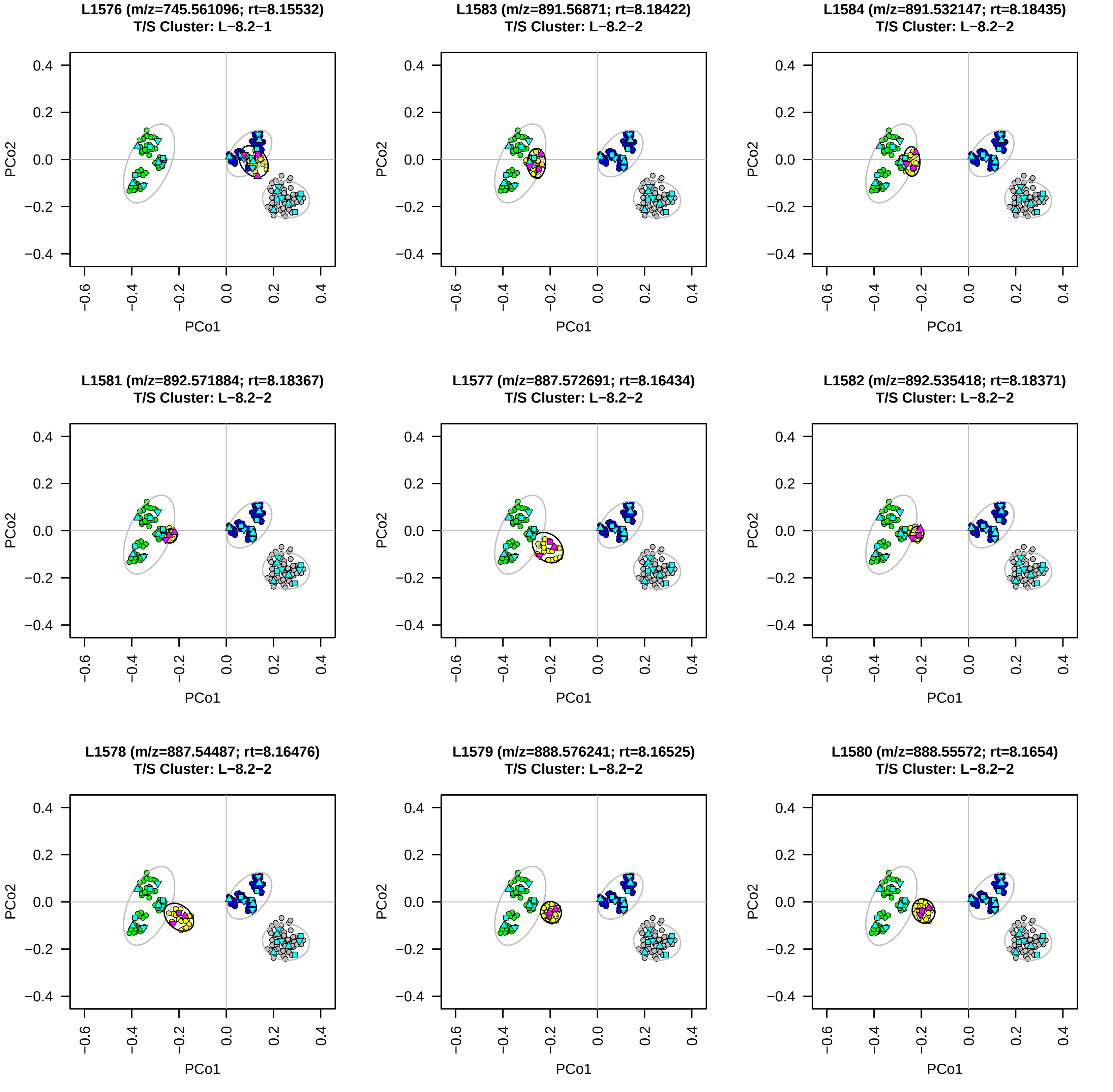

L1576 (m/z=745.561096; rt=8.15532)
T/S Cluster: L−8.2−1
L1583 (m/z=891.56871; rt=8.18422)
T/S Cluster: L−8.2−2
L1584 (m/z=891.532147; rt=8.18435)
T/S Cluster: L−8.2−2
L1581 (m/z=892.571884; rt=8.18367)
T/S Cluster: L−8.2−2
L1577 (m/z=887.572691; rt=8.16434)
T/S Cluster: L−8.2−2
L1582 (m/z=892.535418; rt=8.18371)
T/S Cluster: L−8.2−2
L1578 (m/z=887.54487; rt=8.16476)
T/S Cluster: L−8.2−2
L1579 (m/z=888.576241; rt=8.16525)
T/S Cluster: L−8.2−2
L1580 (m/z=888.55572; rt=8.1654)
T/S Cluster: L−8.2−2
PCo1
PCo2
0.4
0.2
0.0
−0.2
−0.4
−0.6

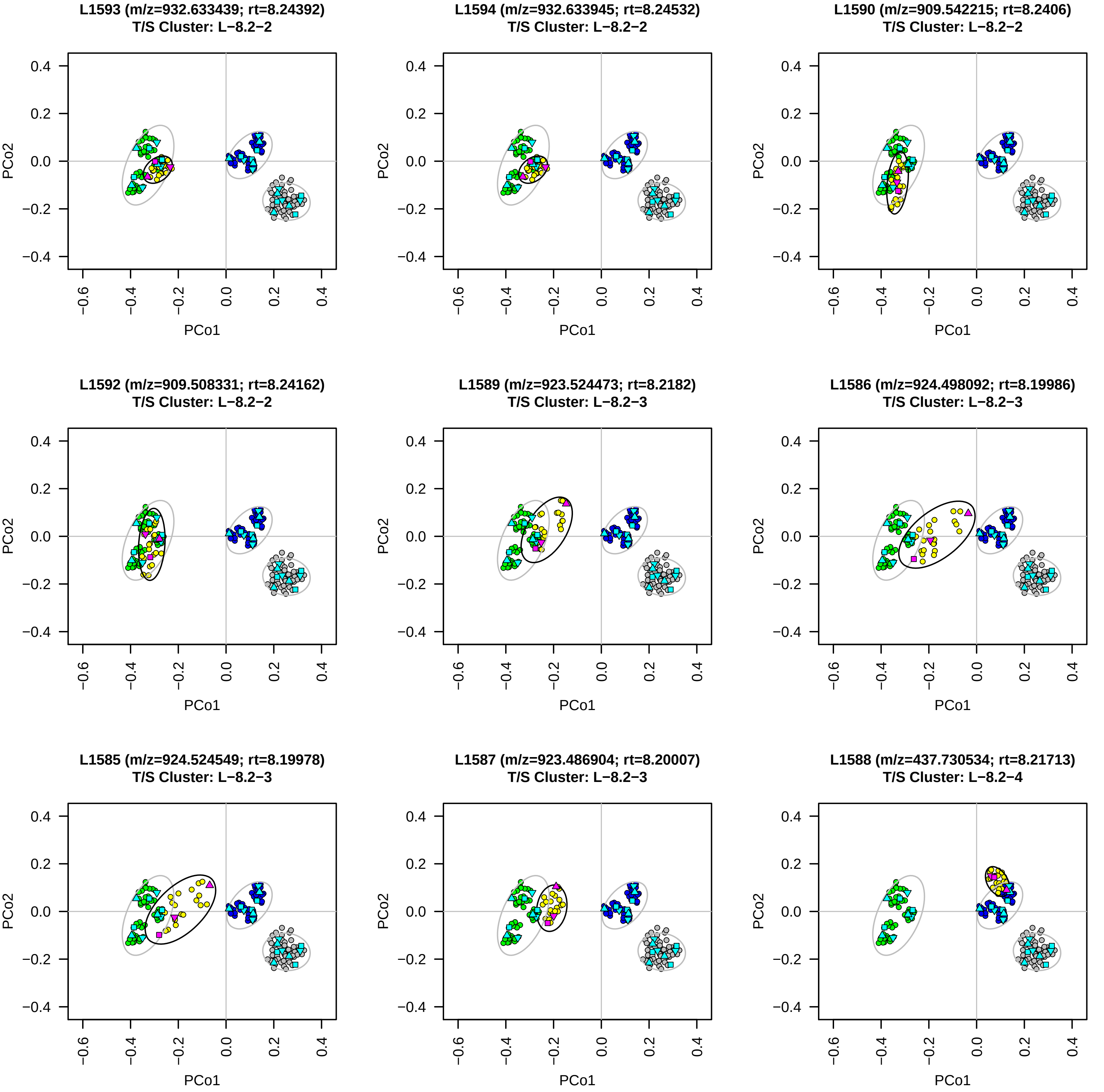
L1593 (m/z=932.633439; rt=8.24392)
T/S Cluster: L−8.2−2
L1594 (m/z=932.633945; rt=8.24532)
T/S Cluster: L−8.2−2
L1590 (m/z=909.542215; rt=8.2406)
T/S Cluster: L−8.2−2
L1592 (m/z=909.508331; rt=8.24162)
T/S Cluster: L−8.2−2
L1589 (m/z=923.524473; rt=8.2182)
T/S Cluster: L−8.2−3
L1586 (m/z=924.498092; rt=8.19986)
T/S Cluster: L−8.2−3
L1585 (m/z=924.524549; rt=8.19978)
T/S Cluster: L−8.2−3
L1587 (m/z=923.486904; rt=8.20007)
T/S Cluster: L−8.2−3
L1588 (m/z=437.730534; rt=8.21713)
T/S Cluster: L−8.2−4
PCo1
PCo2
−0.6
−0.4
−0.2
0.0
0.2
0.4

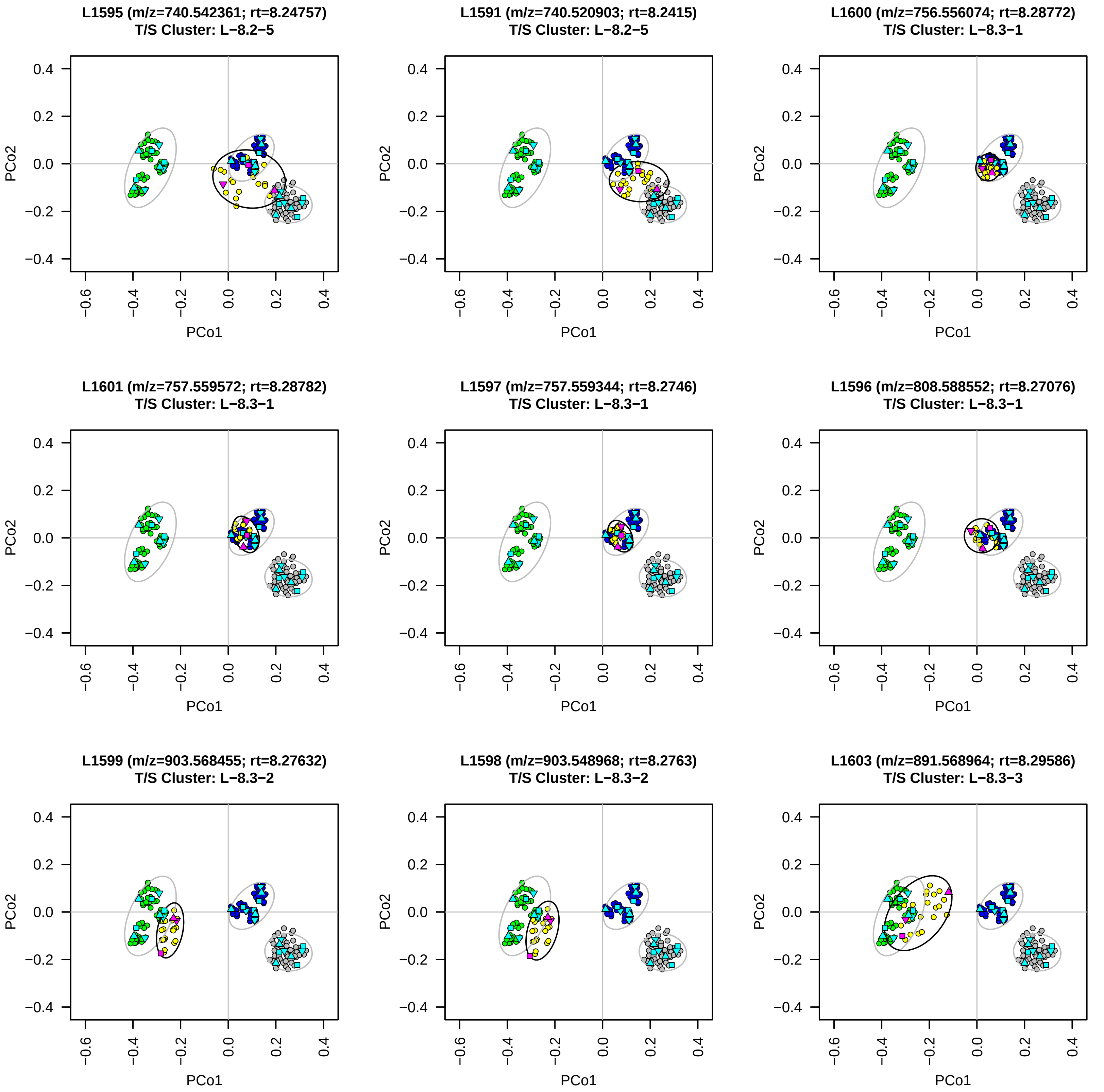

L1595 (m/z=740.542361; rt=8.24757)
T/S Cluster: L−8.2−5
L1591 (m/z=740.520903; rt=8.2415)
T/S Cluster: L−8.2−5
L1600 (m/z=756.556074; rt=8.28772)
T/S Cluster: L−8.3−1
L1601 (m/z=757.559572; rt=8.28782)
T/S Cluster: L−8.3−1
L1597 (m/z=757.559344; rt=8.2746)
T/S Cluster: L−8.3−1
L1596 (m/z=808.588552; rt=8.27076)
T/S Cluster: L−8.3−1
L1599 (m/z=903.568455; rt=8.27632)
T/S Cluster: L−8.3−2
L1598 (m/z=903.548968; rt=8.2763)
T/S Cluster: L−8.3−2
L1603 (m/z=891.568964; rt=8.29586)
T/S Cluster: L−8.3−3
PCo1
PCo2

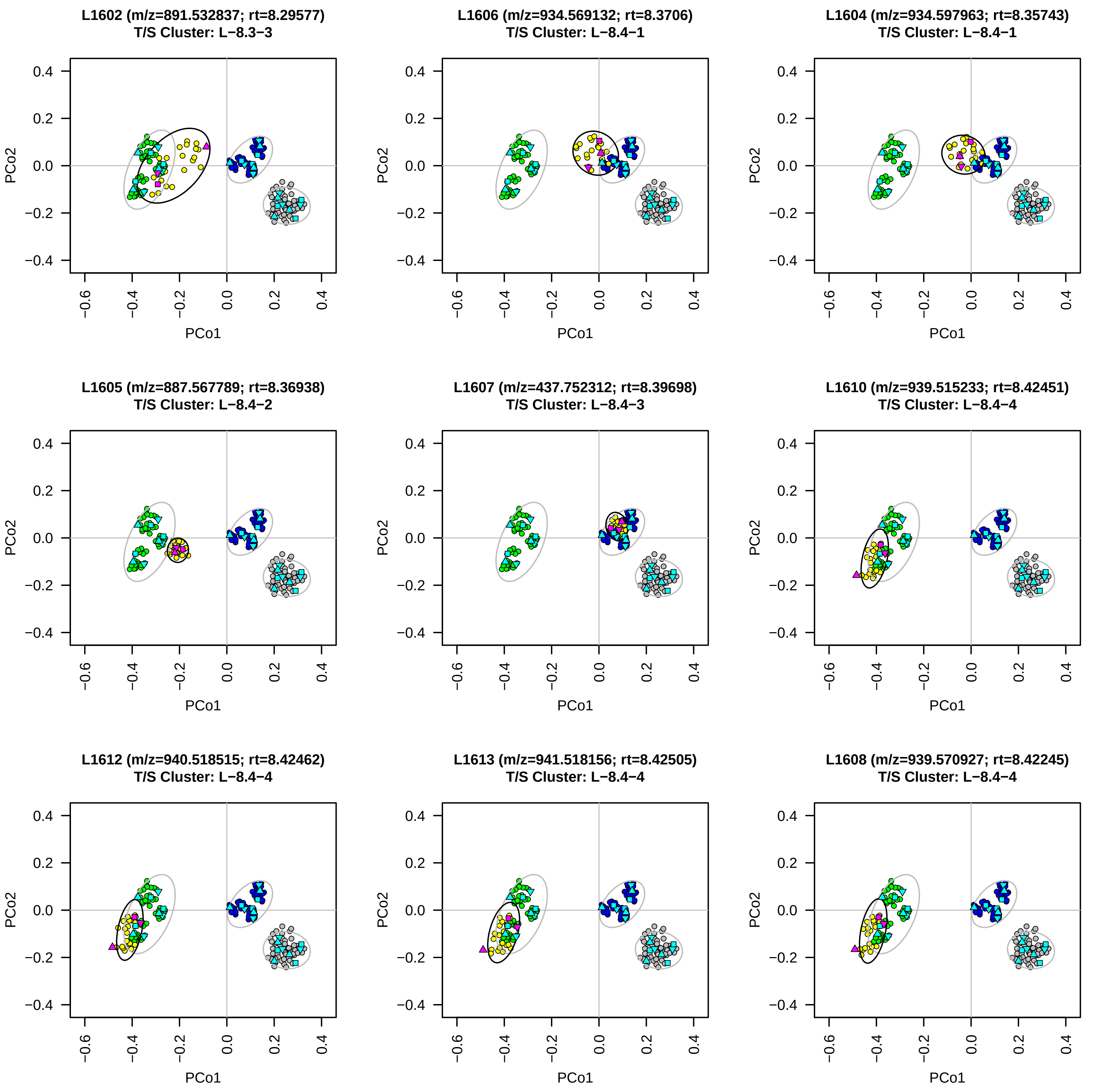
L1602 (m/z=891.532837; rt=8.29577)
T/S Cluster: L−8.3−3
L1606 (m/z=934.569132; rt=8.3706)
T/S Cluster: L−8.4−1
L1604 (m/z=934.597963; rt=8.35743)
T/S Cluster: L−8.4−1
L1605 (m/z=887.567789; rt=8.36938)
T/S Cluster: L−8.4−2
L1607 (m/z=437.752312; rt=8.39698)
T/S Cluster: L−8.4−3
L1610 (m/z=939.515233; rt=8.42451)
T/S Cluster: L−8.4−4
L1612 (m/z=940.518515; rt=8.42462)
T/S Cluster: L−8.4−4
L1613 (m/z=941.518156; rt=8.42505)
T/S Cluster: L−8.4−4
L1608 (m/z=939.570927; rt=8.42245)
T/S Cluster: L−8.4−4
PCo1
PCo2
0.4
0.2
0.0
−0.2
−0.4
−0.6

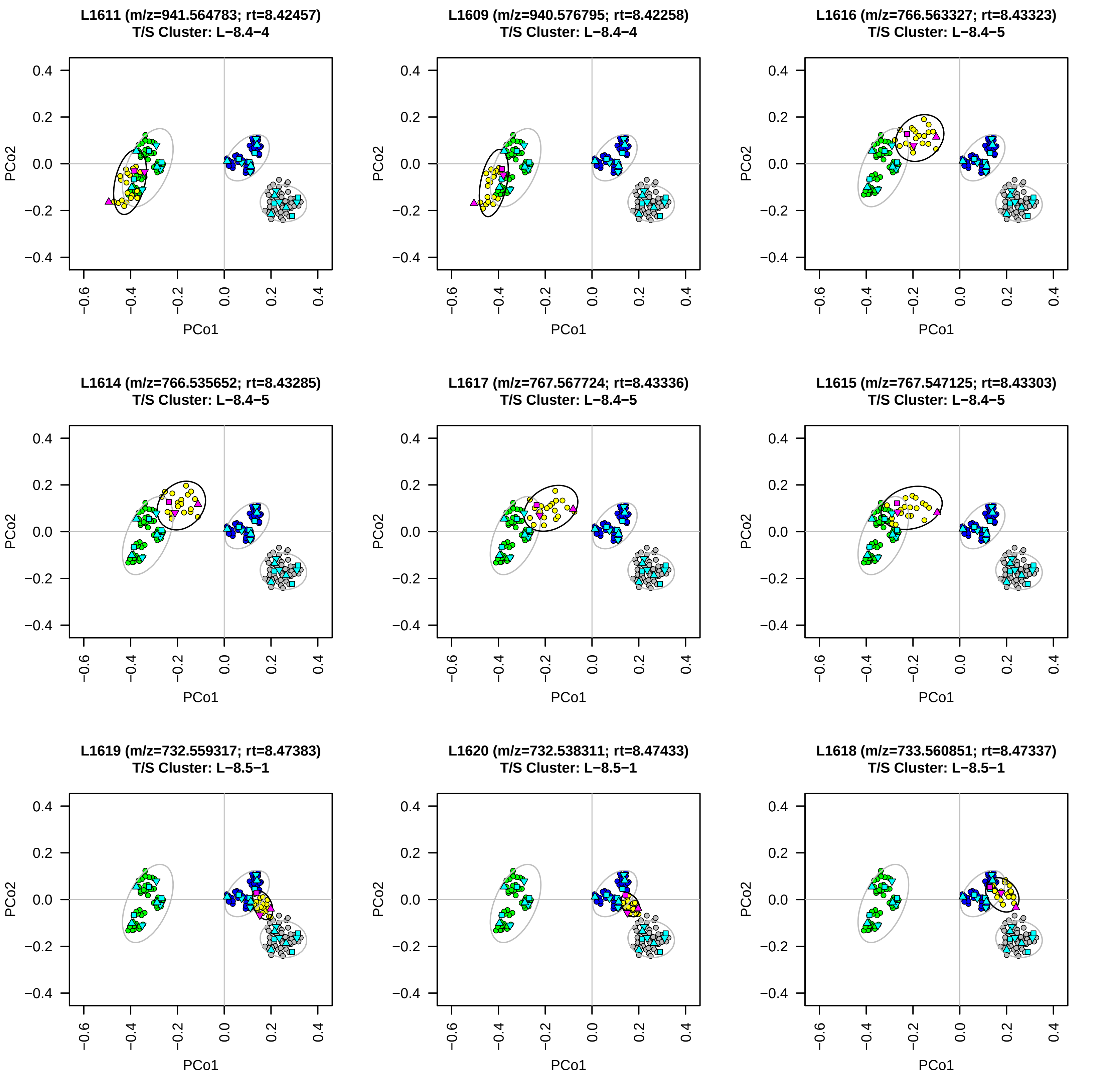

L1611 (m/z=941.564783; rt=8.42457)
T/S Cluster: L−8.4−4
L1609 (m/z=940.576795; rt=8.42258)
T/S Cluster: L−8.4−4
L1616 (m/z=766.563327; rt=8.43323)
T/S Cluster: L−8.4−5
L1614 (m/z=766.535652; rt=8.43285)
T/S Cluster: L−8.4−5
L1617 (m/z=767.567724; rt=8.43336)
T/S Cluster: L−8.4−5
L1615 (m/z=767.547125; rt=8.43303)
T/S Cluster: L−8.4−5
L1619 (m/z=732.559317; rt=8.47383)
T/S Cluster: L−8.5−1
L1620 (m/z=732.538311; rt=8.47433)
T/S Cluster: L−8.5−1
L1618 (m/z=733.560851; rt=8.47337)
T/S Cluster: L−8.5−1
PCo1
PCo2

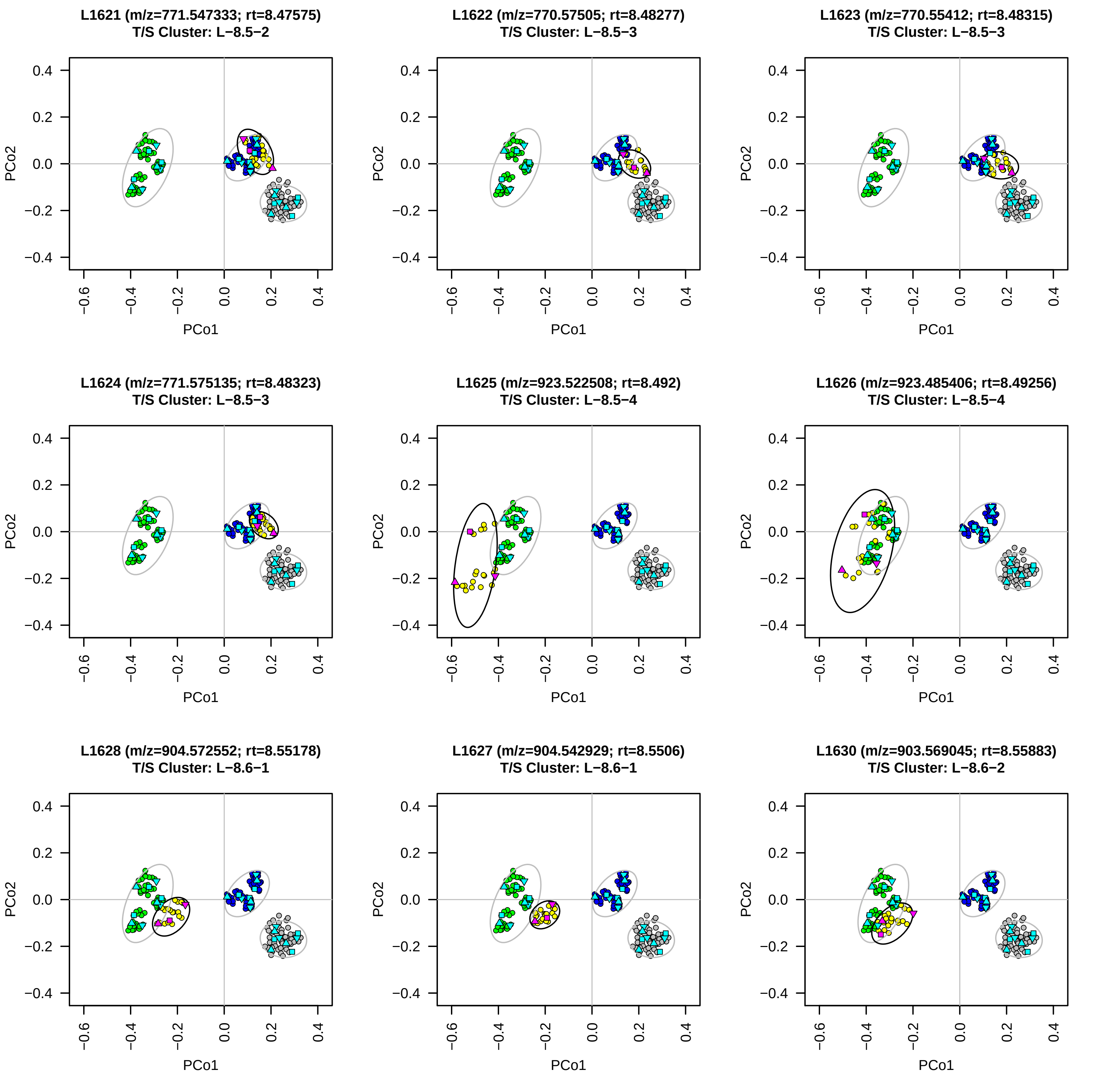
L1621 (m/z=771.547333; rt=8.47575)
T/S Cluster: L−8.5−2
L1622 (m/z=770.57505; rt=8.48277)
T/S Cluster: L−8.5−3
L1623 (m/z=770.55412; rt=8.48315)
T/S Cluster: L−8.5−3
L1624 (m/z=771.575135; rt=8.48323)
T/S Cluster: L−8.5−3
L1625 (m/z=923.522508; rt=8.492)
T/S Cluster: L−8.5−4
L1626 (m/z=923.485406; rt=8.49256)
T/S Cluster: L−8.5−4
L1628 (m/z=904.572552; rt=8.55178)
T/S Cluster: L−8.6−1
L1627 (m/z=904.542929; rt=8.5506)
T/S Cluster: L−8.6−1
L1630 (m/z=903.569045; rt=8.55883)
T/S Cluster: L−8.6−2
PCo1
PCo2

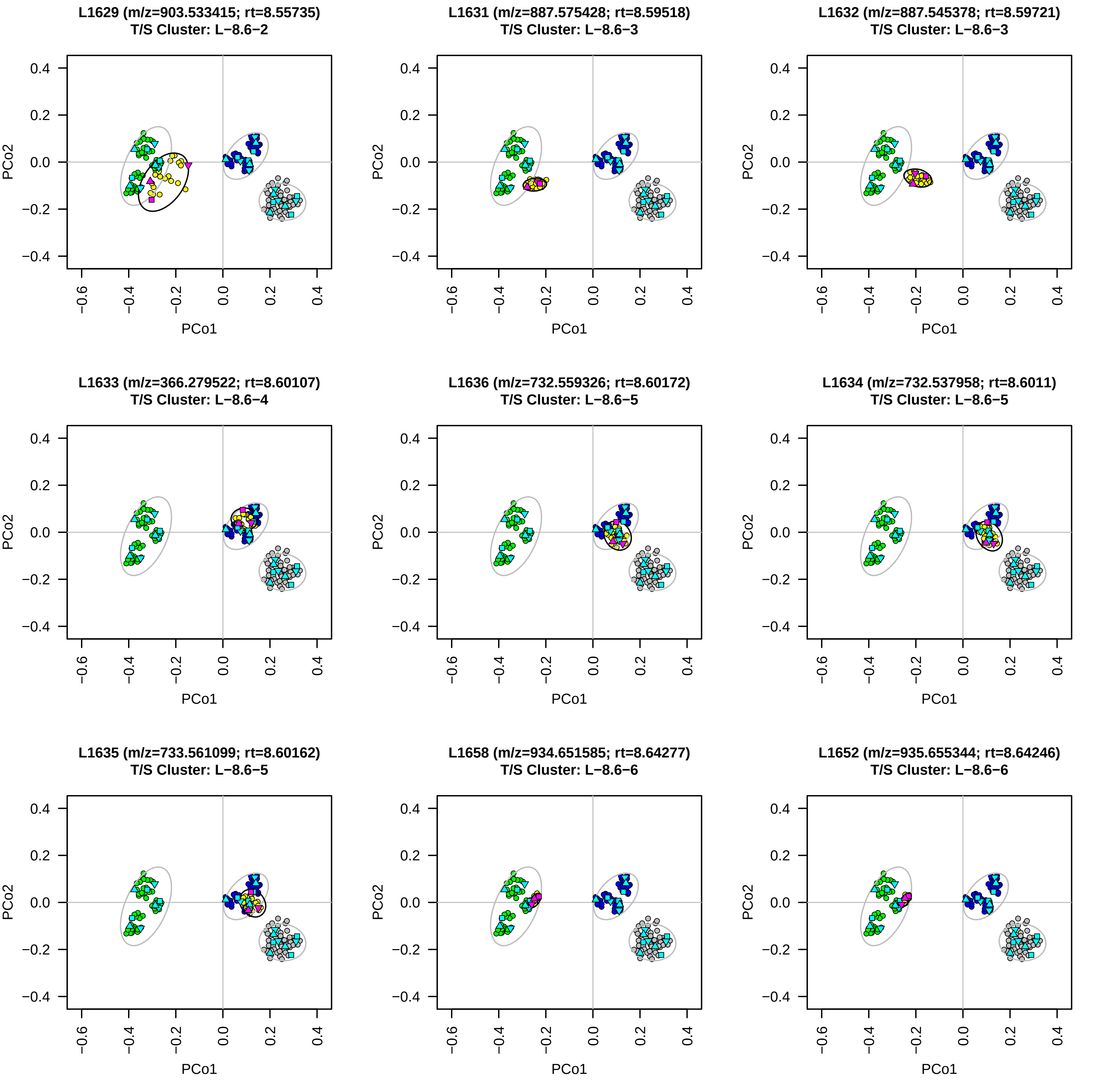

L1629 (m/z=903.533415; rt=8.55735)
T/S Cluster: L−8.6−2
L1631 (m/z=887.575428; rt=8.59518)
T/S Cluster: L−8.6−3
L1632 (m/z=887.545378; rt=8.59721)
T/S Cluster: L−8.6−3
L1633 (m/z=366.279522; rt=8.60107)
T/S Cluster: L−8.6−4
L1636 (m/z=732.559326; rt=8.60172)
T/S Cluster: L−8.6−5
L1634 (m/z=732.537958; rt=8.6011)
T/S Cluster: L−8.6−5
L1635 (m/z=733.561099; rt=8.60162)
T/S Cluster: L−8.6−5
L1658 (m/z=934.651585; rt=8.64277)
T/S Cluster: L−8.6−6
L1652 (m/z=935.655344; rt=8.64246)
T/S Cluster: L−8.6−6
PCo1
PCo2
−0.6
−0.4
−0.2
0.0
0.2
0.4

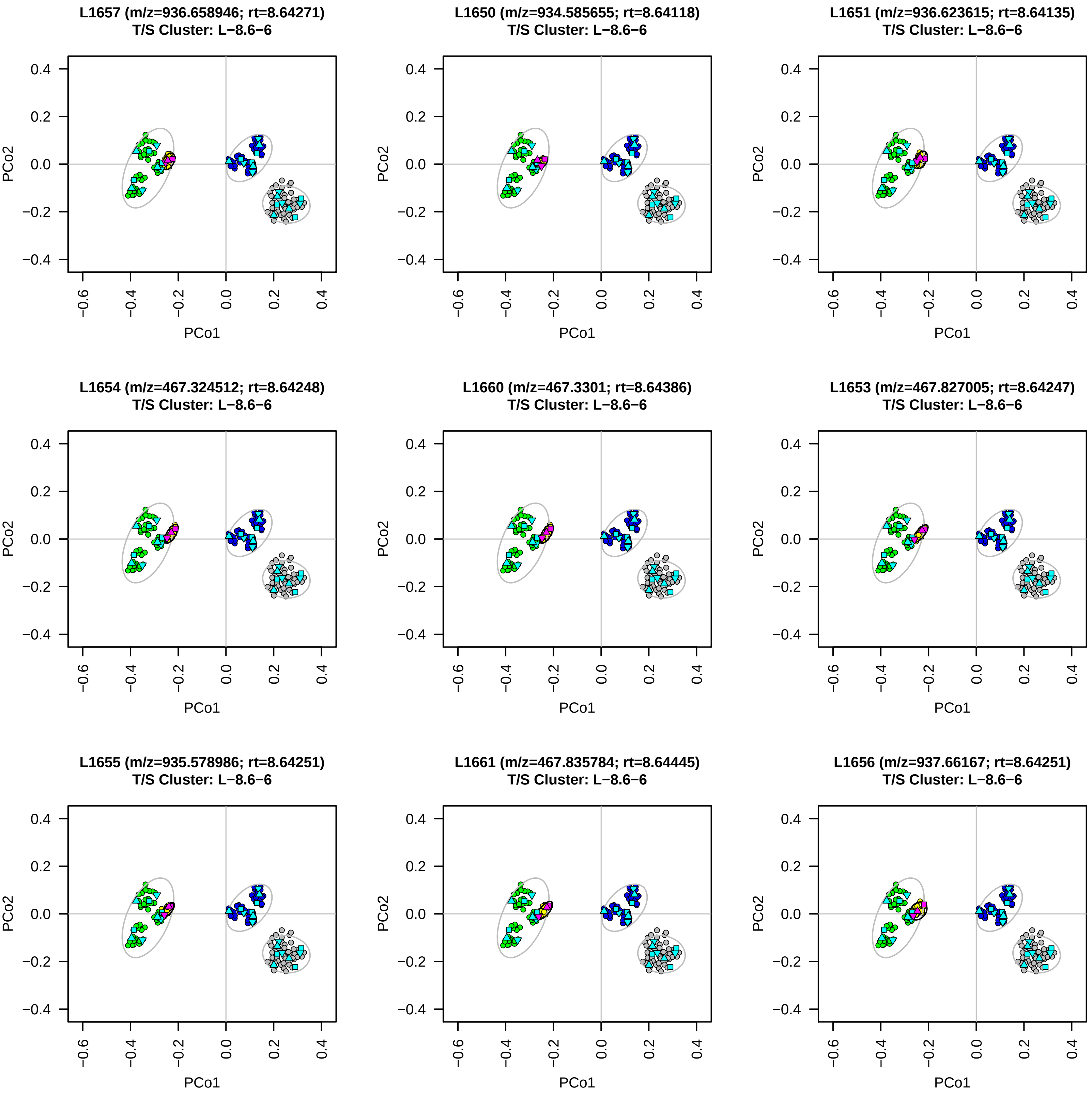
L1657 (m/z=936.658946; rt=8.64271)
T/S Cluster: L−8.6−6
L1650 (m/z=934.585655; rt=8.64118)
T/S Cluster: L−8.6−6
L1651 (m/z=936.623615; rt=8.64135)
T/S Cluster: L−8.6−6
L1654 (m/z=467.324512; rt=8.64248)
T/S Cluster: L−8.6−6
L1660 (m/z=467.3301; rt=8.64386)
T/S Cluster: L−8.6−6
L1653 (m/z=467.827005; rt=8.64247)
T/S Cluster: L−8.6−6
L1655 (m/z=935.578986; rt=8.64251)
T/S Cluster: L−8.6−6
L1661 (m/z=467.835784; rt=8.64445)
T/S Cluster: L−8.6−6
L1656 (m/z=937.66167; rt=8.64251)
T/S Cluster: L−8.6−6
PCo1
PCo2
0.4
0.2
0.0
−0.2
−0.4
−0.6

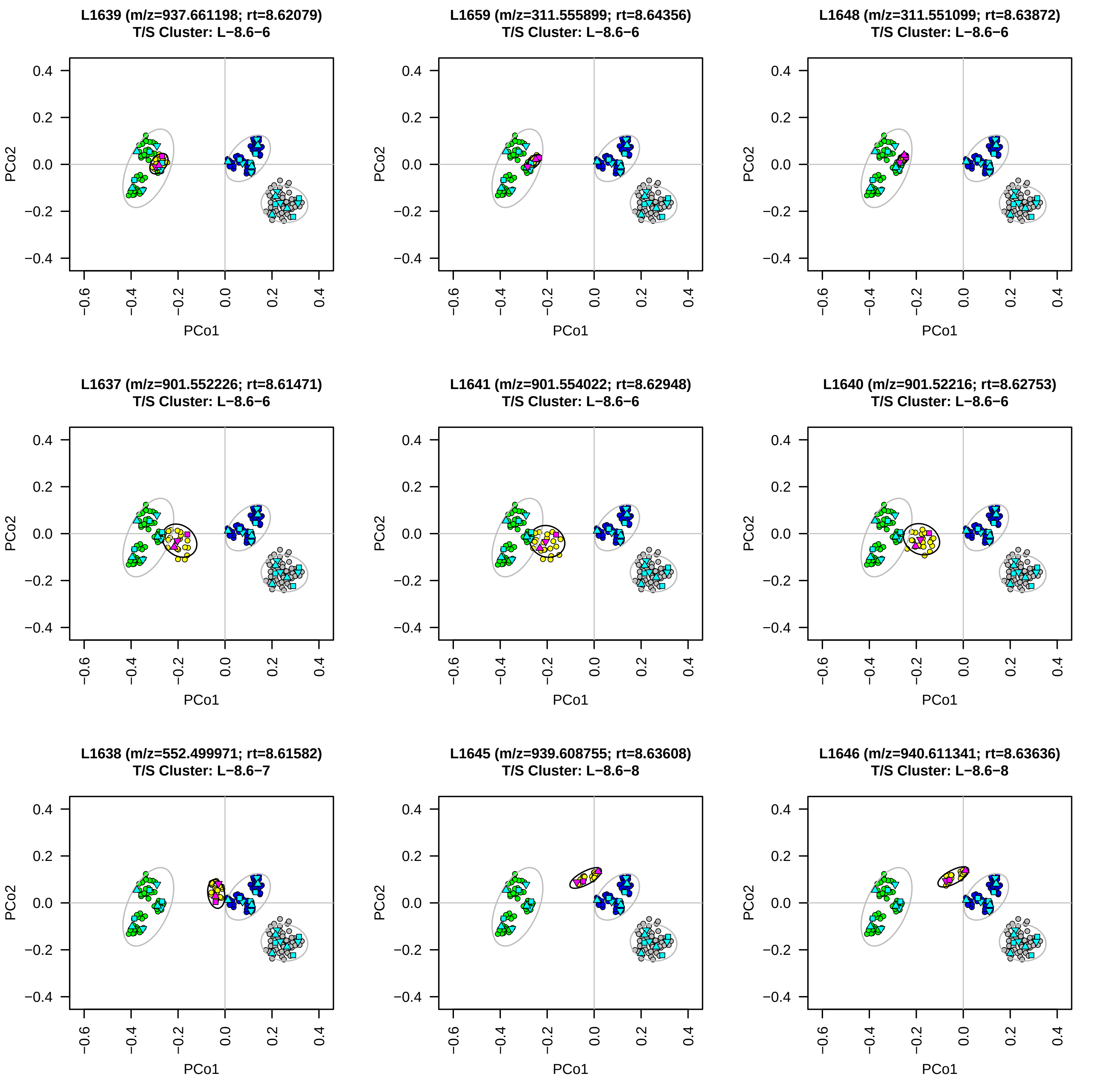
L1639 (m/z=937.661198; rt=8.62079)
T/S Cluster: L−8.6−6
L1659 (m/z=311.555899; rt=8.64356)
T/S Cluster: L−8.6−6
L1648 (m/z=311.551099; rt=8.63872)
T/S Cluster: L−8.6−6
L1637 (m/z=901.552226; rt=8.61471)
T/S Cluster: L−8.6−6
L1641 (m/z=901.554022; rt=8.62948)
T/S Cluster: L−8.6−6
L1640 (m/z=901.52216; rt=8.62753)
T/S Cluster: L−8.6−6
L1638 (m/z=552.499971; rt=8.61582)
T/S Cluster: L−8.6−7
L1645 (m/z=939.608755; rt=8.63608)
T/S Cluster: L−8.6−8
L1646 (m/z=940.611341; rt=8.63636)
T/S Cluster: L−8.6−8
PCo1
PCo2
0.4
0.2
0.0
−0.2
−0.4
−0.6

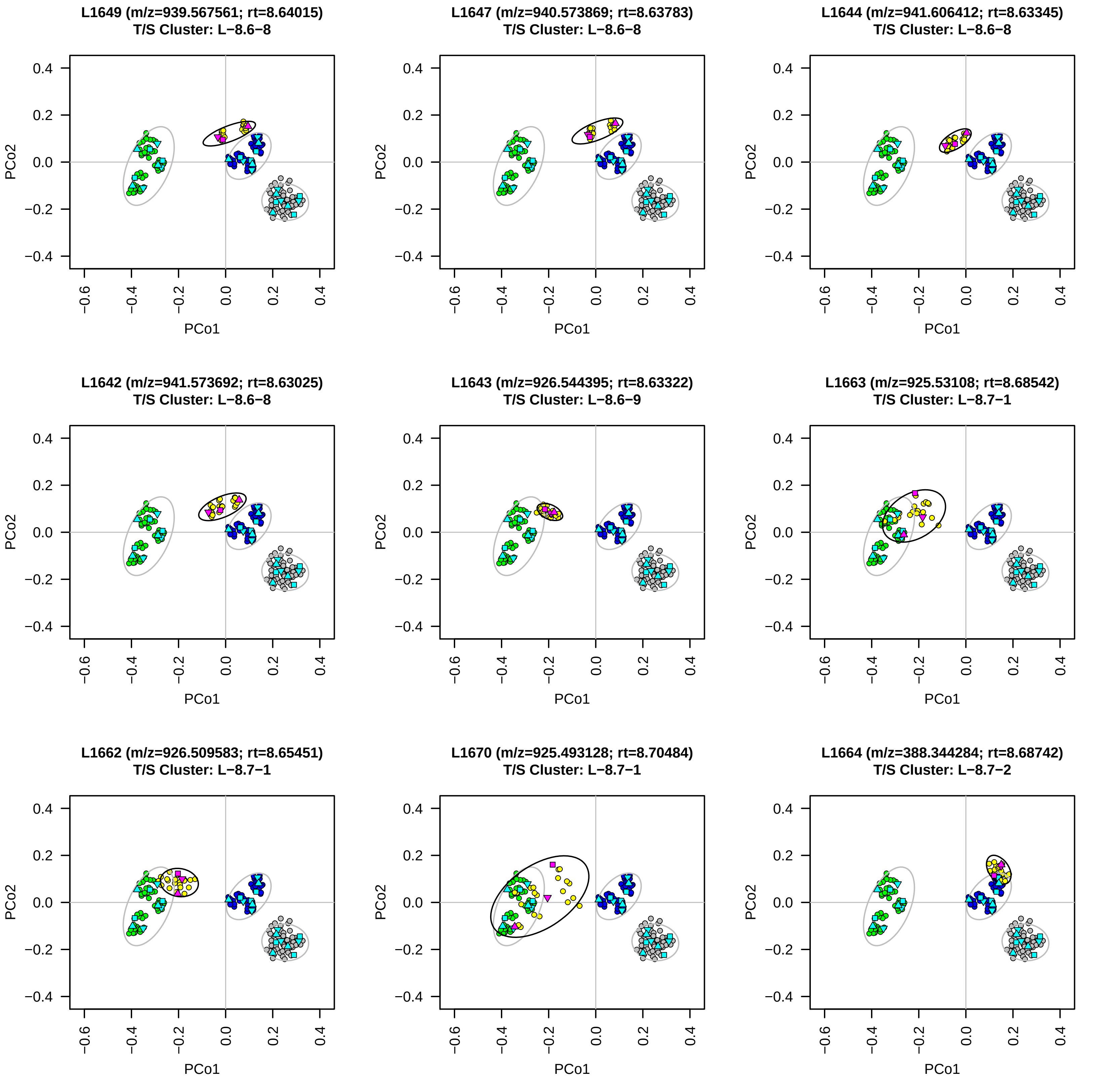

L1649 (m/z=939.567561; rt=8.64015)
T/S Cluster: L−8.6−8
L1647 (m/z=940.573869; rt=8.63783)
T/S Cluster: L−8.6−8
L1644 (m/z=941.606412; rt=8.63345)
T/S Cluster: L−8.6−8
L1642 (m/z=941.573692; rt=8.63025)
T/S Cluster: L−8.6−8
L1643 (m/z=926.544395; rt=8.63322)
T/S Cluster: L−8.6−9
L1663 (m/z=925.53108; rt=8.68542)
T/S Cluster: L−8.7−1
L1662 (m/z=926.509583; rt=8.65451)
T/S Cluster: L−8.7−1
L1670 (m/z=925.493128; rt=8.70484)
T/S Cluster: L−8.7−1
L1664 (m/z=388.344284; rt=8.68742)
T/S Cluster: L−8.7−2
PCo1
PCo2
−0.6
−0.4
−0.2
0.0
0.2
0.4

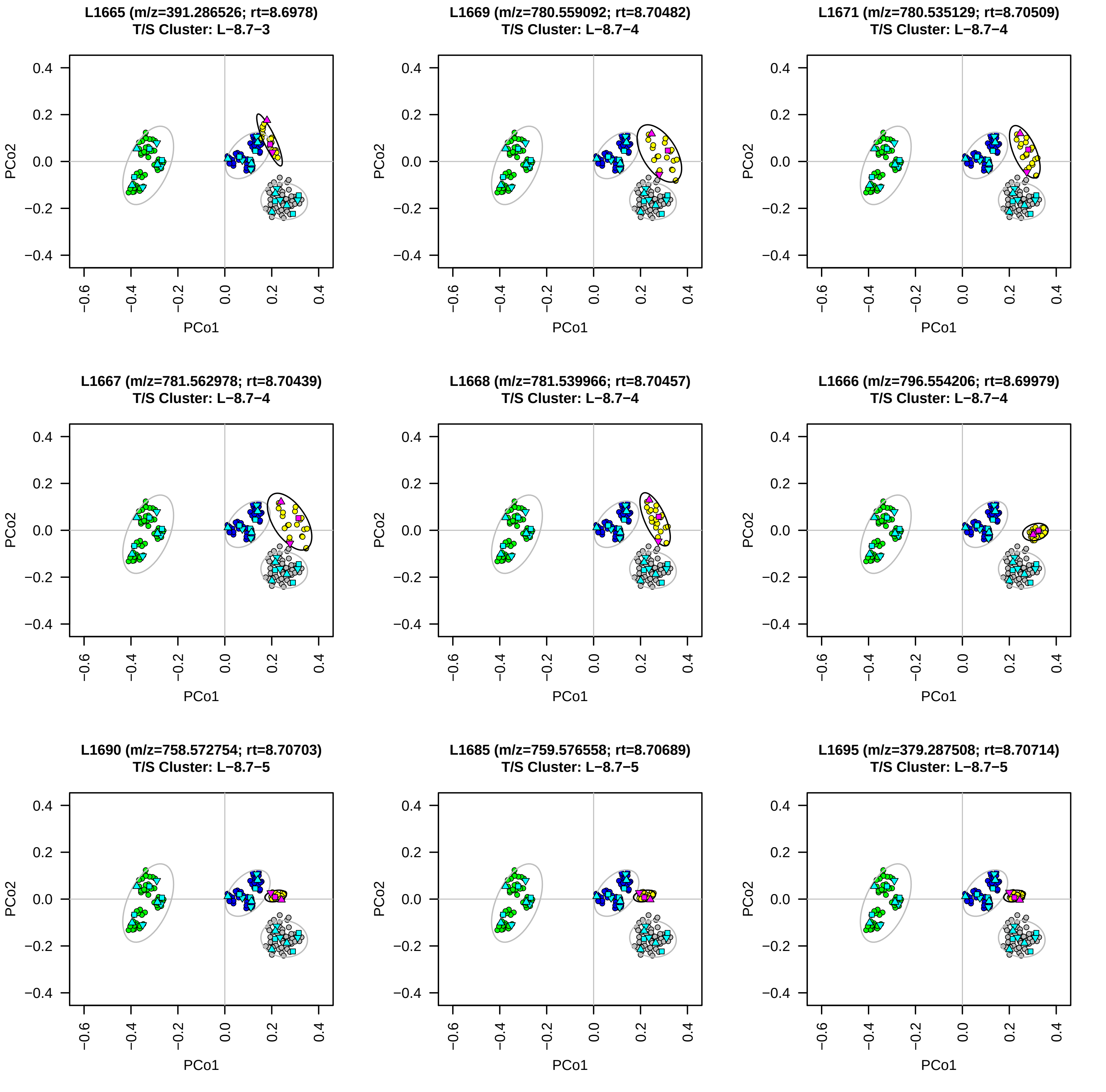
L1665 (m/z=391.286526; rt=8.6978)
T/S Cluster: L−8.7−3
L1669 (m/z=780.559092; rt=8.70482)
T/S Cluster: L−8.7−4
L1671 (m/z=780.535129; rt=8.70509)
T/S Cluster: L−8.7−4
L1667 (m/z=781.562978; rt=8.70439)
T/S Cluster: L−8.7−4
L1668 (m/z=781.539966; rt=8.70457)
T/S Cluster: L−8.7−4
L1666 (m/z=796.554206; rt=8.69979)
T/S Cluster: L−8.7−4
L1690 (m/z=758.572754; rt=8.70703)
T/S Cluster: L−8.7−5
L1685 (m/z=759.576558; rt=8.70689)
T/S Cluster: L−8.7−5
L1695 (m/z=379.287508; rt=8.70714)
T/S Cluster: L−8.7−5
PCo1
PCo2

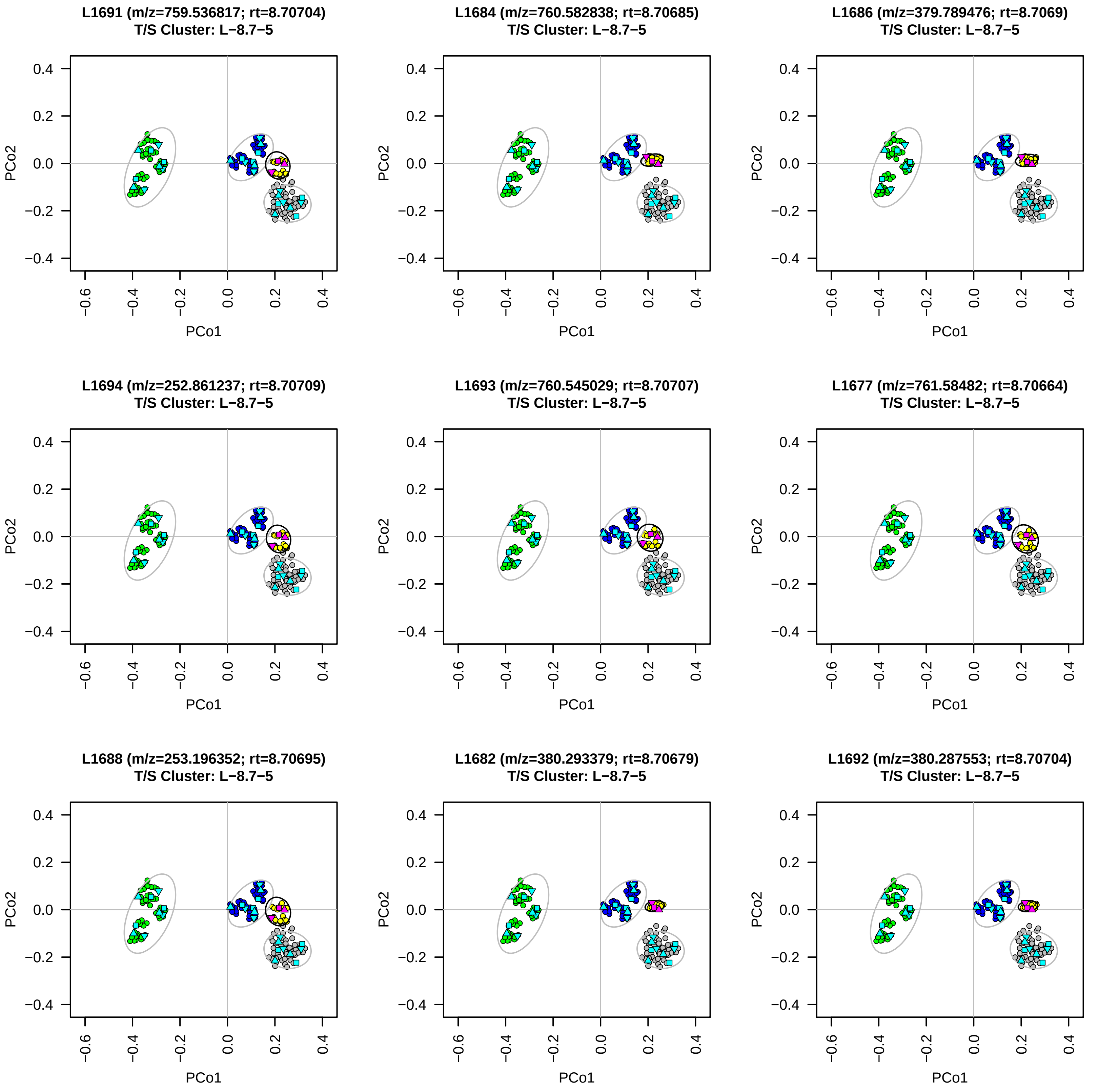
L1691 (m/z=759.536817; rt=8.70704)
T/S Cluster: L−8.7−5
L1684 (m/z=760.582838; rt=8.70685)
T/S Cluster: L−8.7−5
L1686 (m/z=379.789476; rt=8.7069)
T/S Cluster: L−8.7−5
L1694 (m/z=252.861237; rt=8.70709)
T/S Cluster: L−8.7−5
L1693 (m/z=760.545029; rt=8.70707)
T/S Cluster: L−8.7−5
L1677 (m/z=761.58482; rt=8.70664)
T/S Cluster: L−8.7−5
L1688 (m/z=253.196352; rt=8.70695)
T/S Cluster: L−8.7−5
L1682 (m/z=380.293379; rt=8.70679)
T/S Cluster: L−8.7−5
L1692 (m/z=380.287553; rt=8.70704)
T/S Cluster: L−8.7−5
PCo1
PCo2

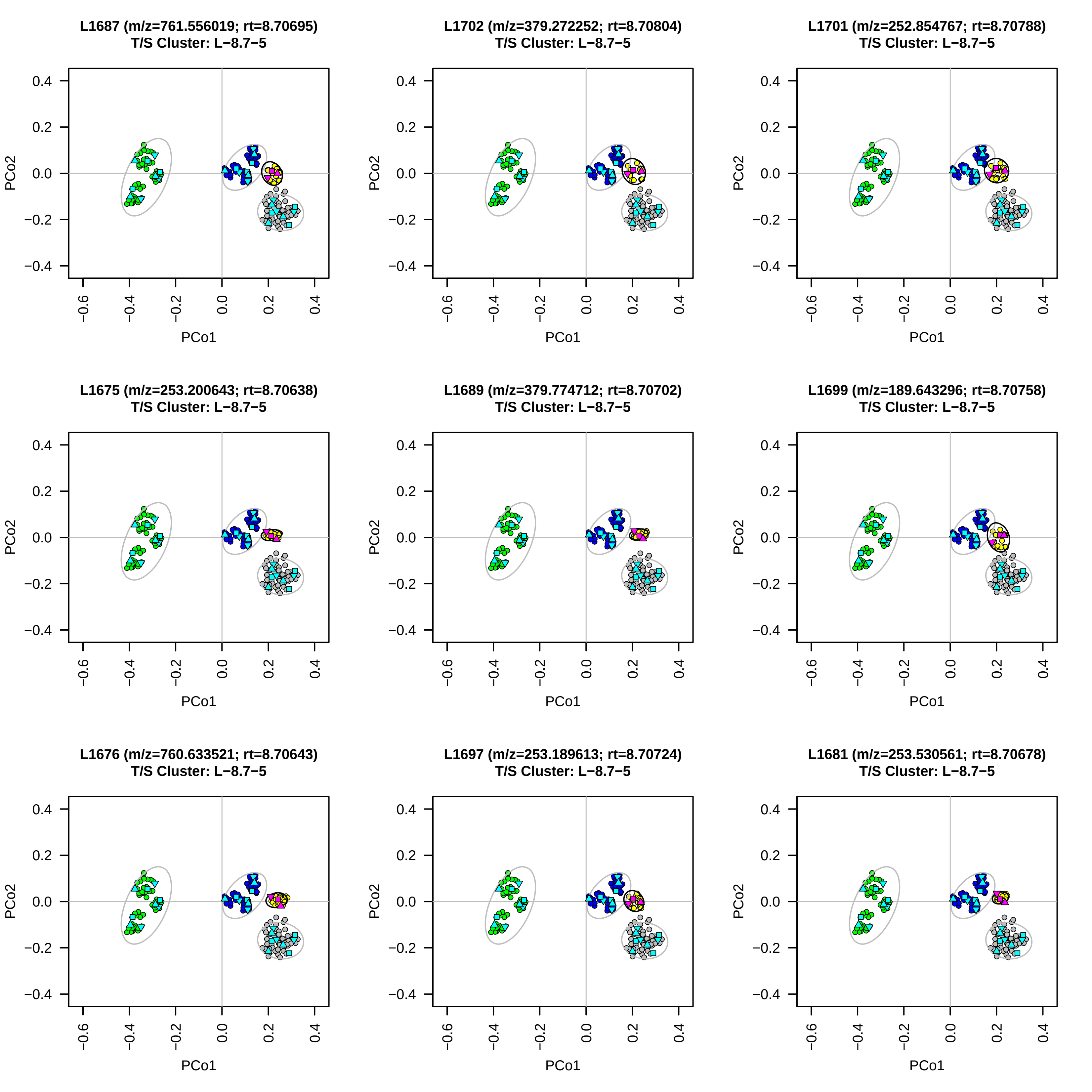
L1687 (m/z=761.556019; rt=8.70695)
T/S Cluster: L−8.7−5
L1702 (m/z=379.272252; rt=8.70804)
T/S Cluster: L−8.7−5
L1701 (m/z=252.854767; rt=8.70788)
T/S Cluster: L−8.7−5
L1675 (m/z=253.200643; rt=8.70638)
T/S Cluster: L−8.7−5
L1689 (m/z=379.774712; rt=8.70702)
T/S Cluster: L−8.7−5
L1699 (m/z=189.643296; rt=8.70758)
T/S Cluster: L−8.7−5
L1676 (m/z=760.633521; rt=8.70643)
T/S Cluster: L−8.7−5
L1697 (m/z=253.189613; rt=8.70724)
T/S Cluster: L−8.7−5
L1681 (m/z=253.530561; rt=8.70678)
T/S Cluster: L−8.7−5
PCo1
PCo2

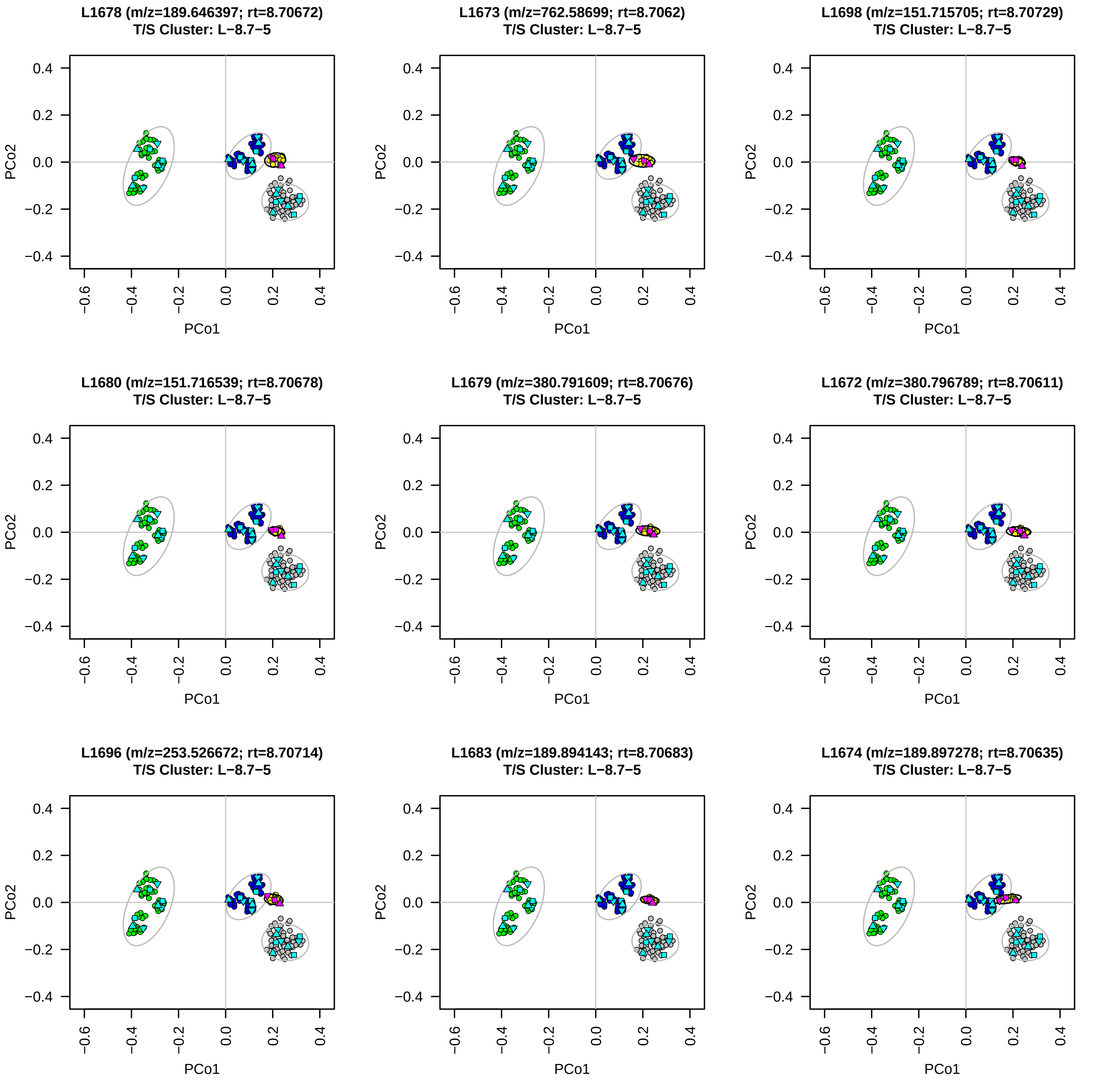
L1678 (m/z=189.646397; rt=8.70672)
T/S Cluster: L−8.7−5
L1673 (m/z=762.58699; rt=8.7062)
T/S Cluster: L−8.7−5
L1698 (m/z=151.715705; rt=8.70729)
T/S Cluster: L−8.7−5
L1680 (m/z=151.716539; rt=8.70678)
T/S Cluster: L−8.7−5
L1679 (m/z=380.791609; rt=8.70676)
T/S Cluster: L−8.7−5
L1672 (m/z=380.796789; rt=8.70611)
T/S Cluster: L−8.7−5
L1696 (m/z=253.526672; rt=8.70714)
T/S Cluster: L−8.7−5
L1683 (m/z=189.894143; rt=8.70683)
T/S Cluster: L−8.7−5
L1674 (m/z=189.897278; rt=8.70635)
T/S Cluster: L−8.7−5
PCo1
PCo2

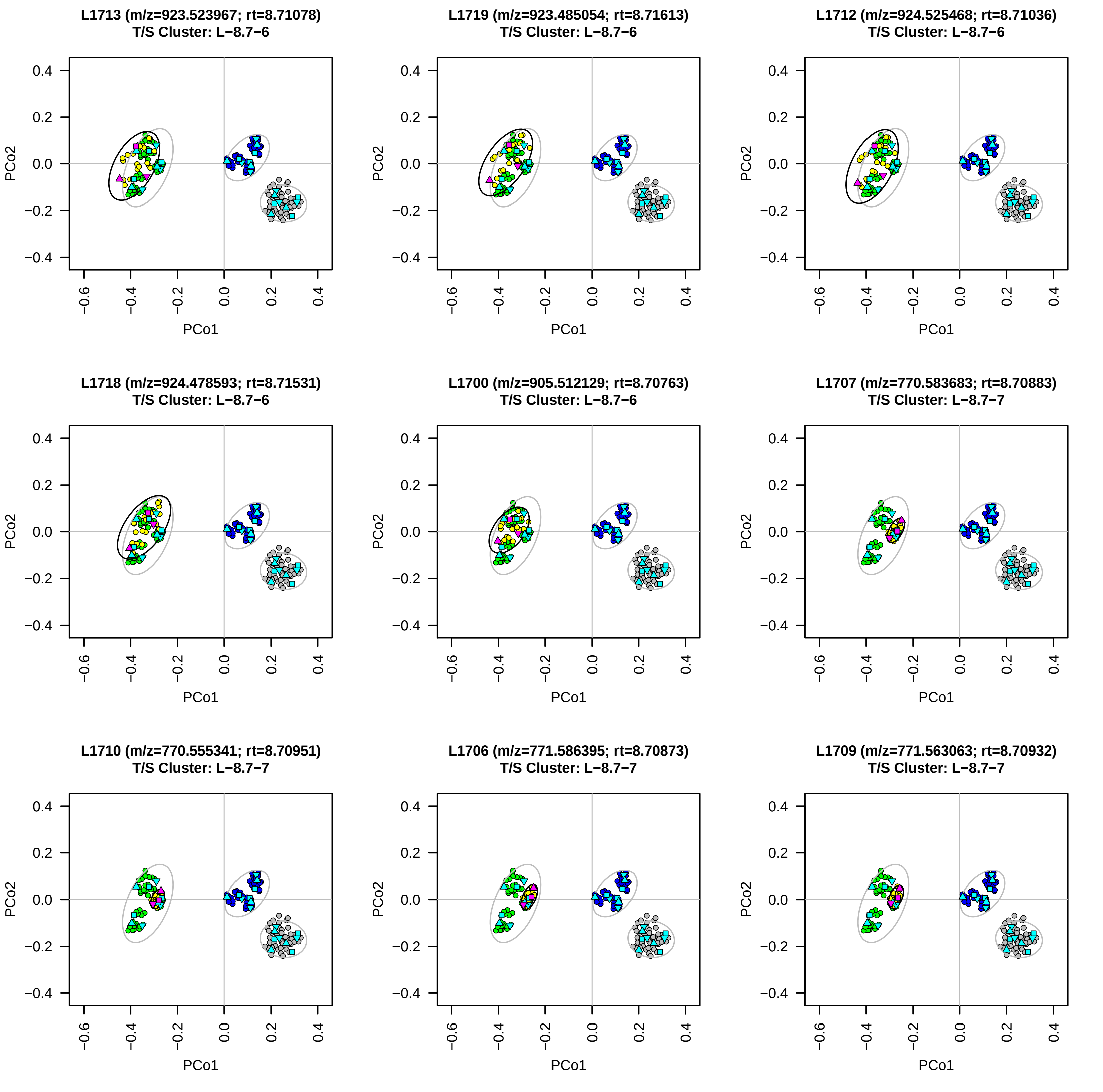

L1713 (m/z=923.523967; rt=8.71078)
T/S Cluster: L−8.7−6
L1719 (m/z=923.485054; rt=8.71613)
T/S Cluster: L−8.7−6
L1712 (m/z=924.525468; rt=8.71036)
T/S Cluster: L−8.7−6
L1718 (m/z=924.478593; rt=8.71531)
T/S Cluster: L−8.7−6
L1700 (m/z=905.512129; rt=8.70763)
T/S Cluster: L−8.7−6
L1707 (m/z=770.583683; rt=8.70883)
T/S Cluster: L−8.7−7
L1710 (m/z=770.555341; rt=8.70951)
T/S Cluster: L−8.7−7
L1706 (m/z=771.586395; rt=8.70873)
T/S Cluster: L−8.7−7
L1709 (m/z=771.563063; rt=8.70932)
T/S Cluster: L−8.7−7
PCo1
PCo2

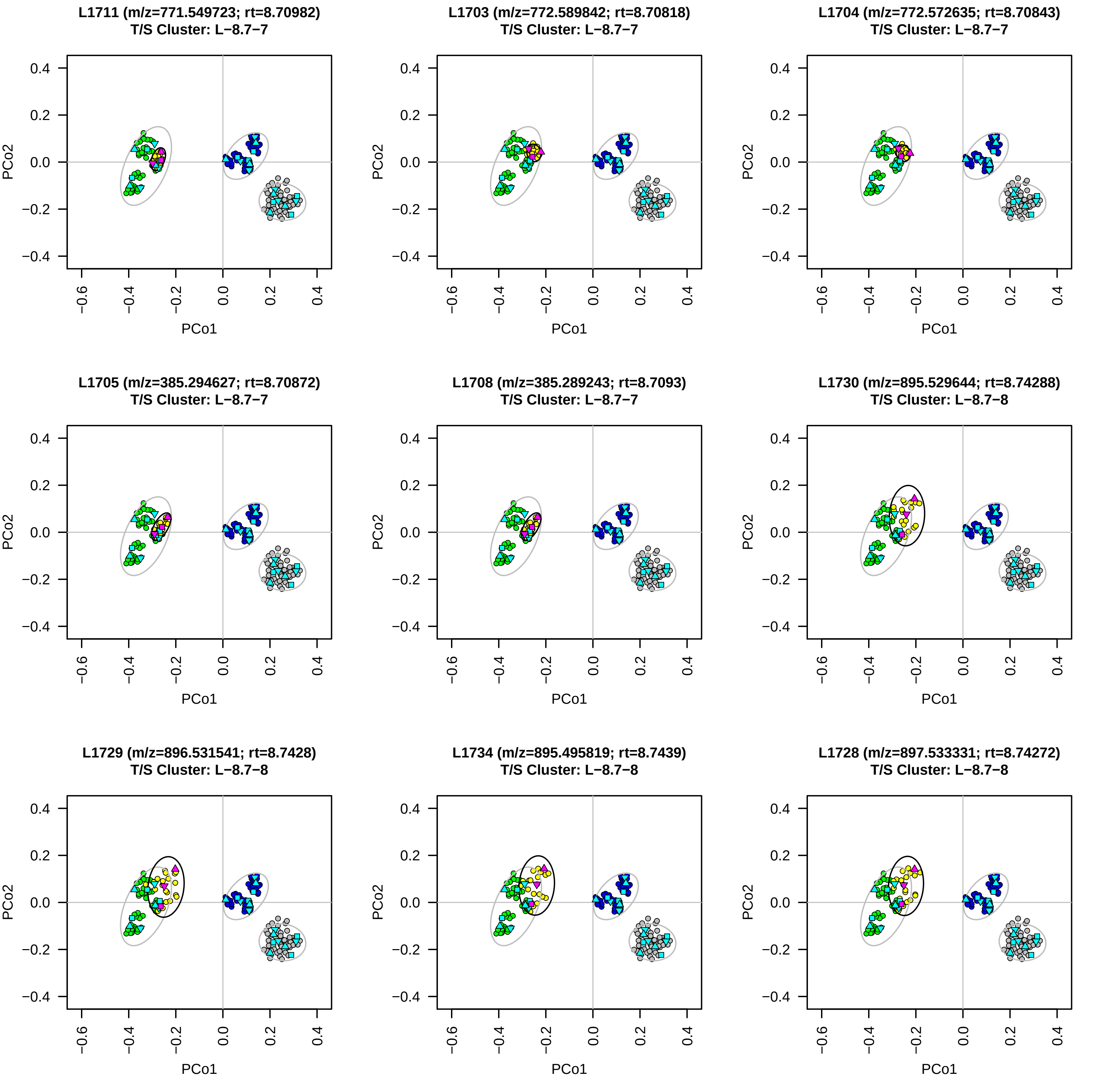
L1711 (m/z=771.549723; rt=8.70982)
T/S Cluster: L−8.7−7
L1703 (m/z=772.589842; rt=8.70818)
T/S Cluster: L−8.7−7
L1704 (m/z=772.572635; rt=8.70843)
T/S Cluster: L−8.7−7
L1705 (m/z=385.294627; rt=8.70872)
T/S Cluster: L−8.7−7
L1708 (m/z=385.289243; rt=8.7093)
T/S Cluster: L−8.7−7
L1730 (m/z=895.529644; rt=8.74288)
T/S Cluster: L−8.7−8
L1729 (m/z=896.531541; rt=8.7428)
T/S Cluster: L−8.7−8
L1734 (m/z=895.495819; rt=8.7439)
T/S Cluster: L−8.7−8
L1728 (m/z=897.533331; rt=8.74272)
T/S Cluster: L−8.7−8
PCo1
PCo2
−0.6
−0.4
−0.2
0.0
0.2
0.4

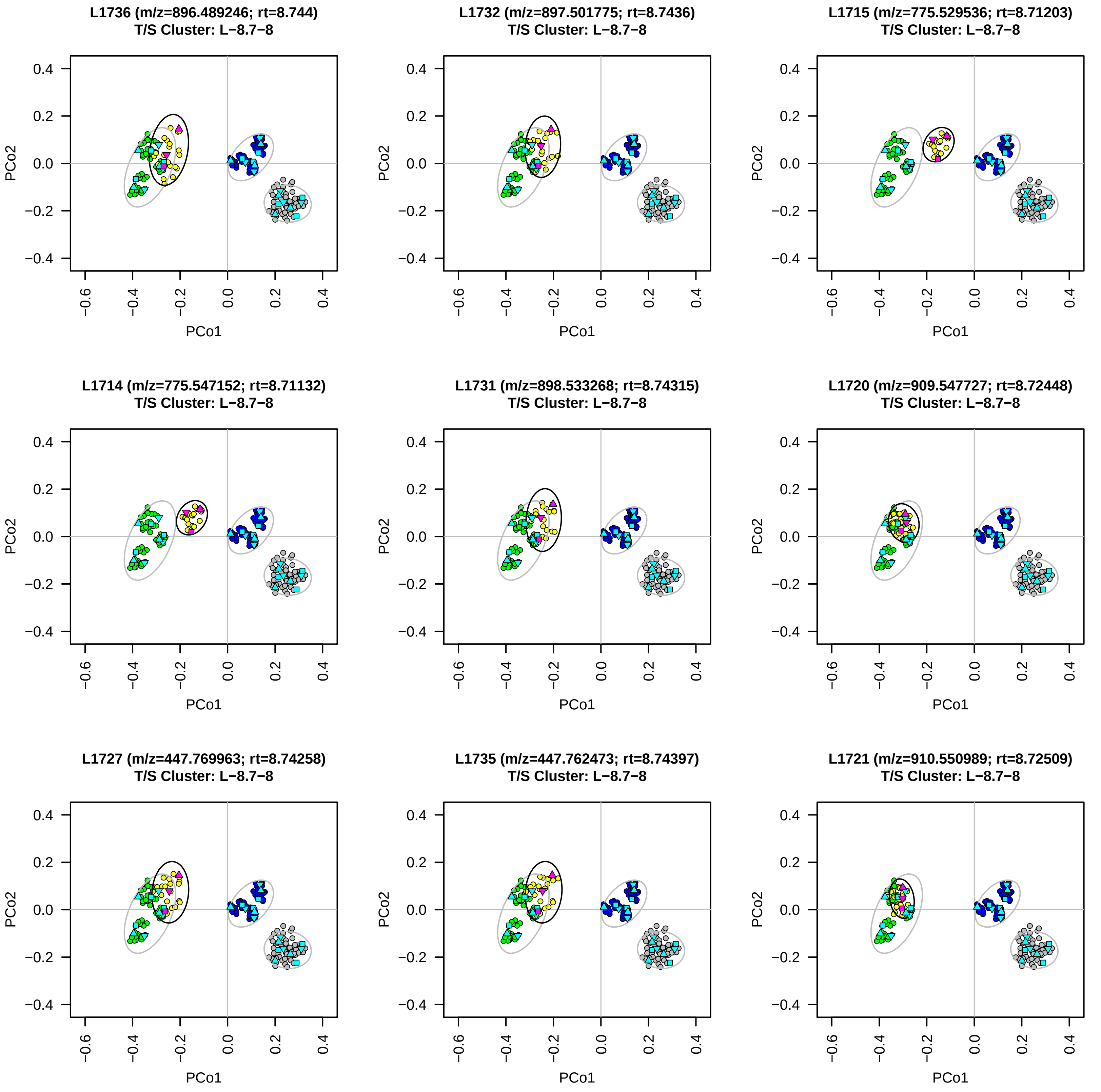
L1736 (m/z=896.489246; rt=8.744)
T/S Cluster: L−8.7−8
L1732 (m/z=897.501775; rt=8.7436)
T/S Cluster: L−8.7−8
L1715 (m/z=775.529536; rt=8.71203)
T/S Cluster: L−8.7−8
L1714 (m/z=775.547152; rt=8.71132)
T/S Cluster: L−8.7−8
L1731 (m/z=898.533268; rt=8.74315)
T/S Cluster: L−8.7−8
L1720 (m/z=909.547727; rt=8.72448)
T/S Cluster: L−8.7−8
L1727 (m/z=447.769963; rt=8.74258)
T/S Cluster: L−8.7−8
L1735 (m/z=447.762473; rt=8.74397)
T/S Cluster: L−8.7−8
L1721 (m/z=910.550989; rt=8.72509)
T/S Cluster: L−8.7−8
PCo1
PCo2
−0.6
−0.4
−0.2
0.0
0.2
0.4

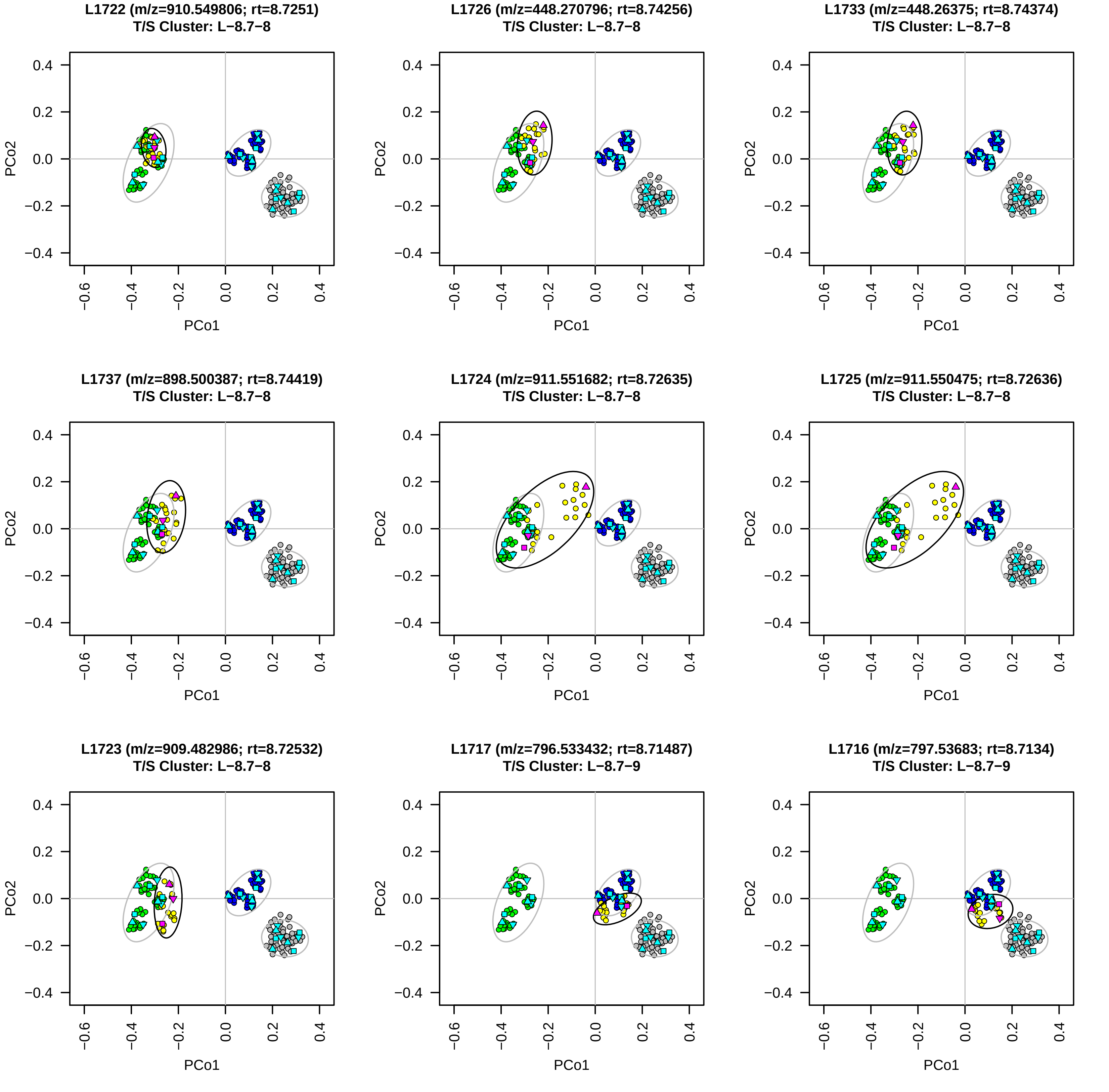
L1722 (m/z=910.549806; rt=8.7251)
T/S Cluster: L−8.7−8
L1726 (m/z=448.270796; rt=8.74256)
T/S Cluster: L−8.7−8
L1733 (m/z=448.26375; rt=8.74374)
T/S Cluster: L−8.7−8
L1737 (m/z=898.500387; rt=8.74419)
T/S Cluster: L−8.7−8
L1724 (m/z=911.551682; rt=8.72635)
T/S Cluster: L−8.7−8
L1725 (m/z=911.550475; rt=8.72636)
T/S Cluster: L−8.7−8
L1723 (m/z=909.482986; rt=8.72532)
T/S Cluster: L−8.7−8
L1717 (m/z=796.533432; rt=8.71487)
T/S Cluster: L−8.7−9
L1716 (m/z=797.53683; rt=8.7134)
T/S Cluster: L−8.7−9
PCo1
PCo2

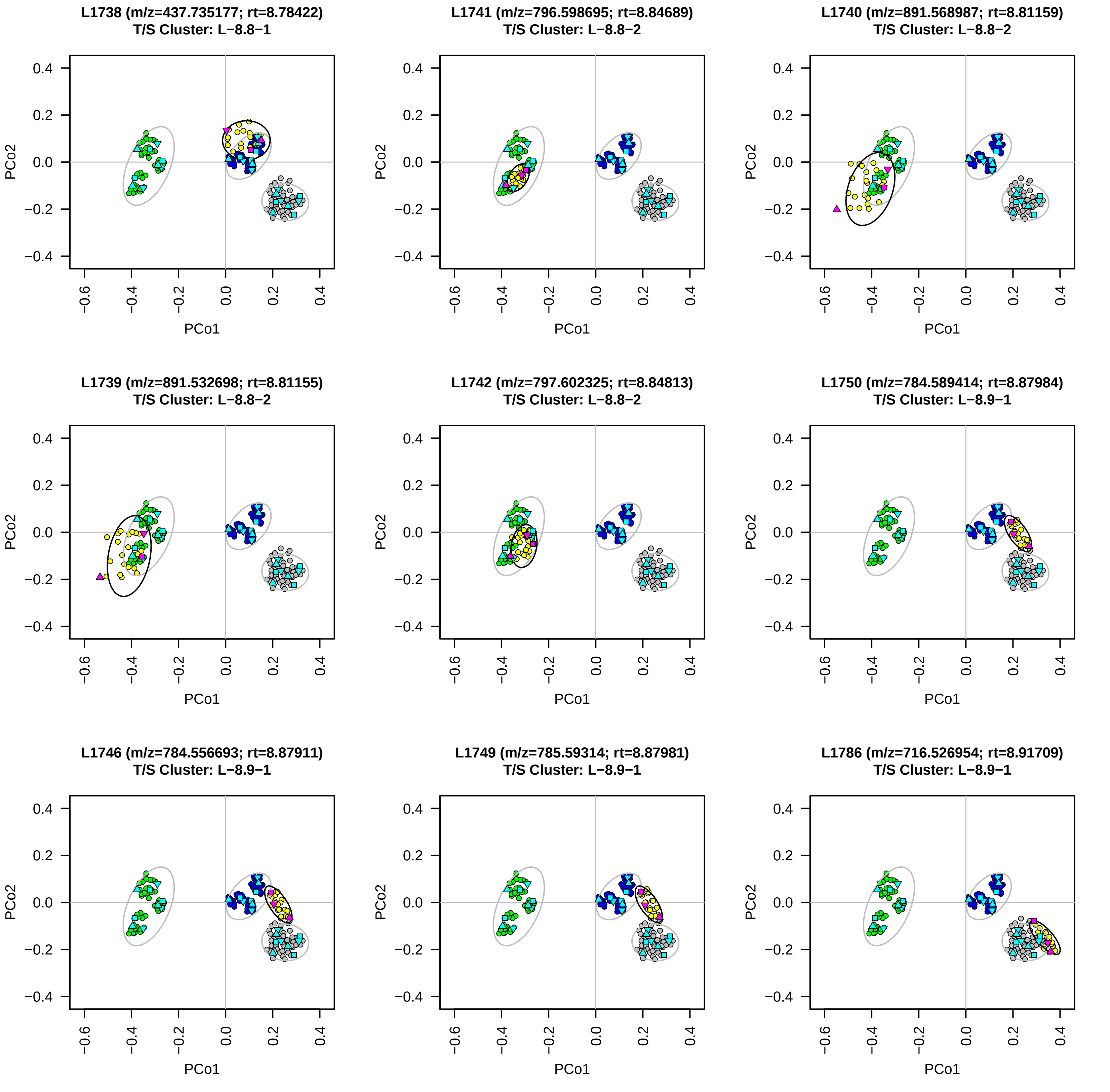
L1738 (m/z=437.735177; rt=8.78422)
T/S Cluster: L−8.8−1
L1741 (m/z=796.598695; rt=8.84689)
T/S Cluster: L−8.8−2
L1740 (m/z=891.568987; rt=8.81159)
T/S Cluster: L−8.8−2
L1739 (m/z=891.532698; rt=8.81155)
T/S Cluster: L−8.8−2
L1742 (m/z=797.602325; rt=8.84813)
T/S Cluster: L−8.8−2
L1750 (m/z=784.589414; rt=8.87984)
T/S Cluster: L−8.9−1
L1746 (m/z=784.556693; rt=8.87911)
T/S Cluster: L−8.9−1
L1749 (m/z=785.59314; rt=8.87981)
T/S Cluster: L−8.9−1
L1786 (m/z=716.526954; rt=8.91709)
T/S Cluster: L−8.9−1
PCo1
PCo2
−0.6
−0.4
−0.2
0.0
0.2
0.4

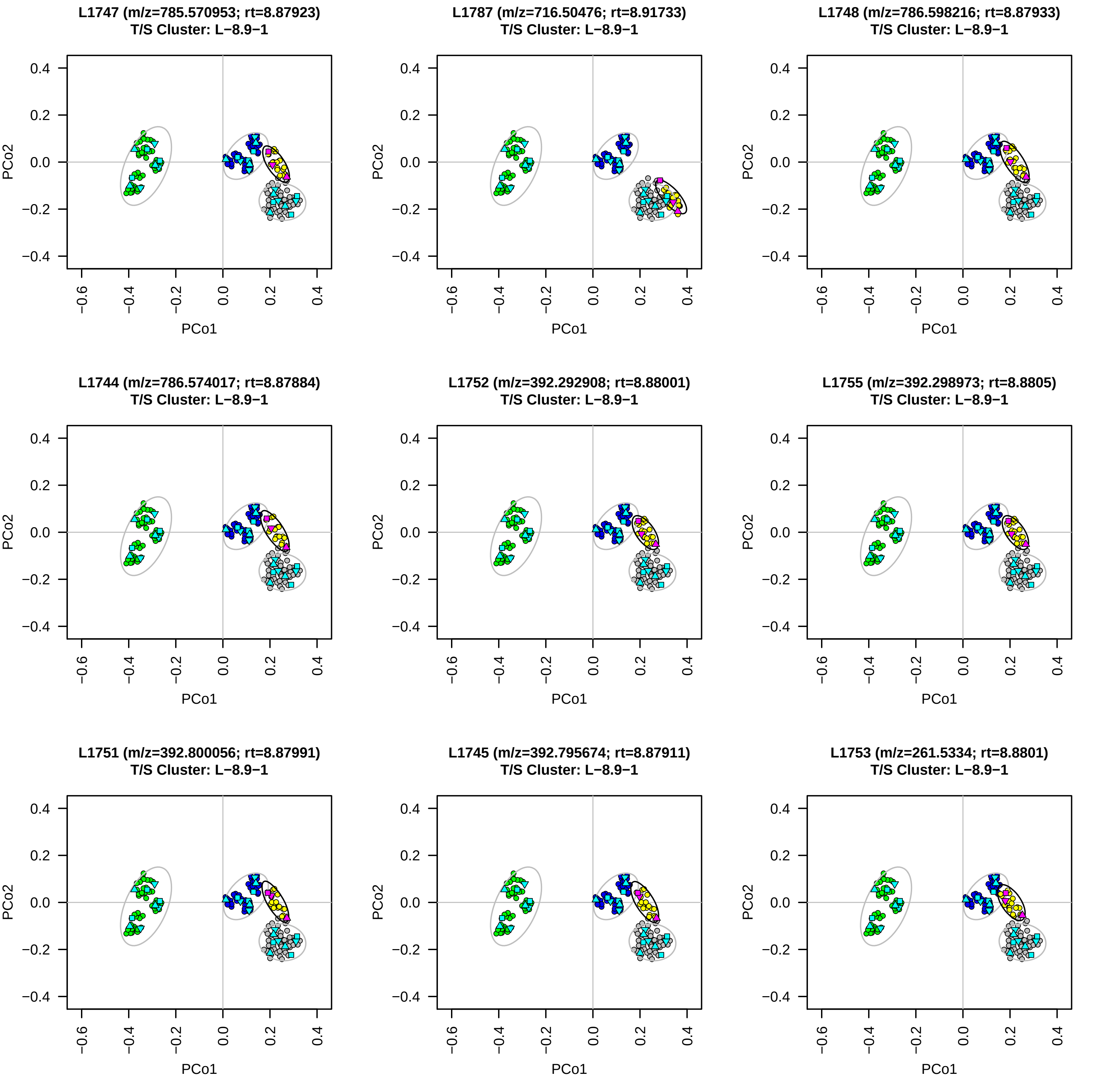
L1747 (m/z=785.570953; rt=8.87923)
T/S Cluster: L−8.9−1
L1787 (m/z=716.50476; rt=8.91733)
T/S Cluster: L−8.9−1
L1748 (m/z=786.598216; rt=8.87933)
T/S Cluster: L−8.9−1
L1744 (m/z=786.574017; rt=8.87884)
T/S Cluster: L−8.9−1
L1752 (m/z=392.292908; rt=8.88001)
T/S Cluster: L−8.9−1
L1755 (m/z=392.298973; rt=8.8805)
T/S Cluster: L−8.9−1
L1751 (m/z=392.800056; rt=8.87991)
T/S Cluster: L−8.9−1
L1745 (m/z=392.795674; rt=8.87911)
T/S Cluster: L−8.9−1
L1753 (m/z=261.5334; rt=8.8801)
T/S Cluster: L−8.9−1
PCo1
PCo2
0.4
0.2
0.0
−0.2
−0.4
−0.6

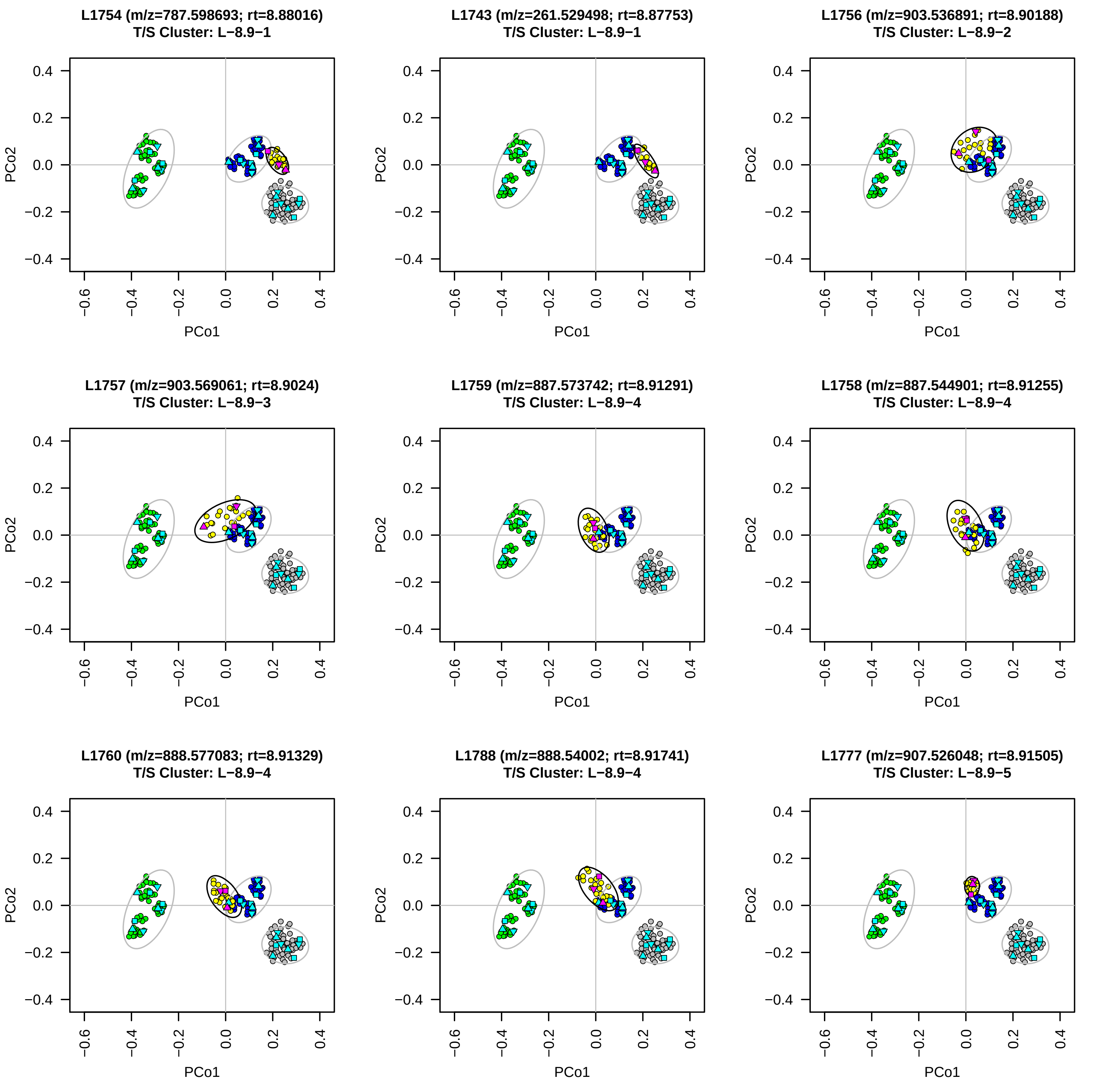
L1754 (m/z=787.598693; rt=8.88016)
T/S Cluster: L−8.9−1
L1743 (m/z=261.529498; rt=8.87753)
T/S Cluster: L−8.9−1
L1756 (m/z=903.536891; rt=8.90188)
T/S Cluster: L−8.9−2
L1757 (m/z=903.569061; rt=8.9024)
T/S Cluster: L−8.9−3
L1759 (m/z=887.573742; rt=8.91291)
T/S Cluster: L−8.9−4
L1758 (m/z=887.544901; rt=8.91255)
T/S Cluster: L−8.9−4
L1760 (m/z=888.577083; rt=8.91329)
T/S Cluster: L−8.9−4
L1788 (m/z=888.54002; rt=8.91741)
T/S Cluster: L−8.9−4
L1777 (m/z=907.526048; rt=8.91505)
T/S Cluster: L−8.9−5
PCo1
PCo2
0.4
0.2
0.0
−0.2
−0.4
−0.6

L1768 (m/z=908.52917; rt=8.9148)
T/S Cluster: L−8.9−5

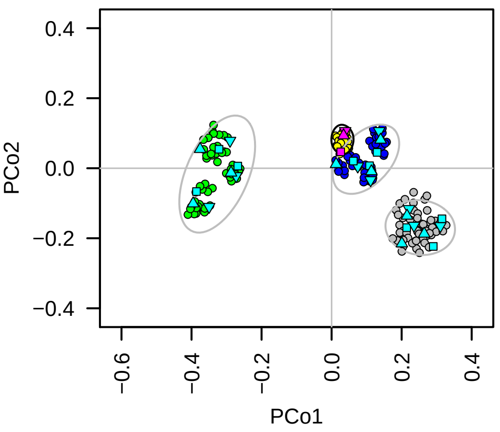

L1783 (m/z=909.528911; rt=8.91609)
T/S Cluster: L−8.9−5

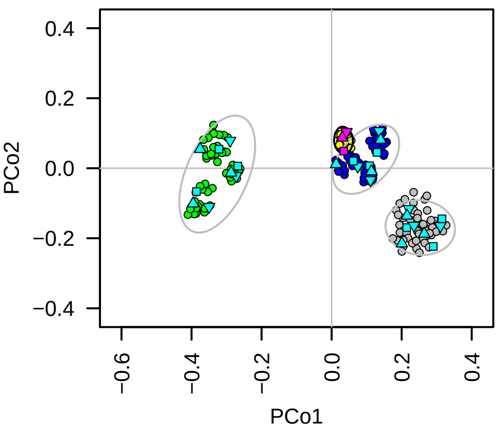

L1779 (m/z=909.503706; rt=8.91523)
T/S Cluster: L−8.9−5

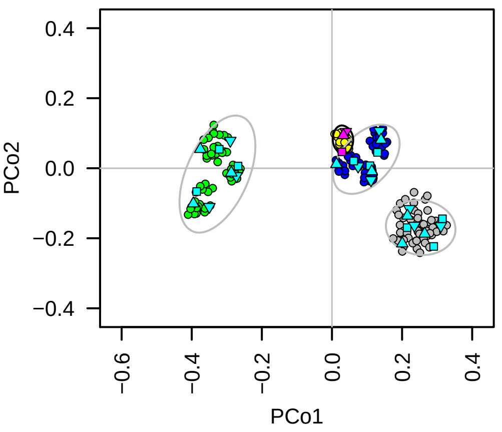

L1780 (m/z=907.464676; rt=8.91527)
T/S Cluster: L−8.9−5

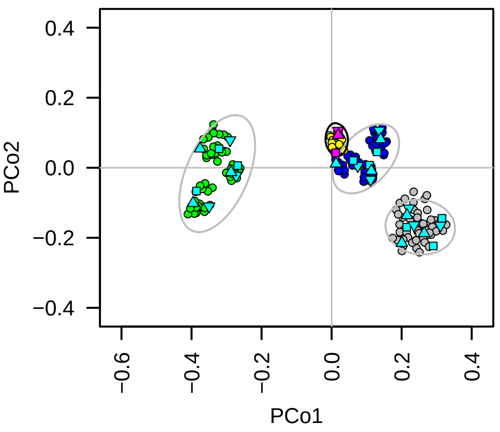

L1778 (m/z=453.761839; rt=8.91517)
T/S Cluster: L−8.9−5

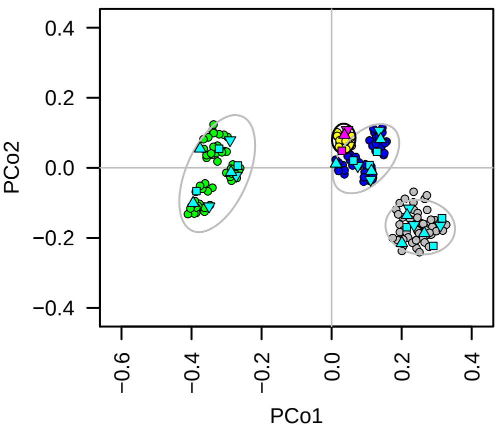

L1773 (m/z=453.770355; rt=8.91495)
T/S Cluster: L−8.9−5

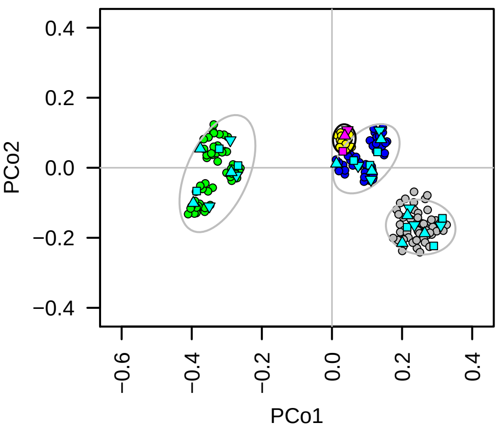

L1771 (m/z=908.461527; rt=8.91494)
T/S Cluster: L−8.9−5

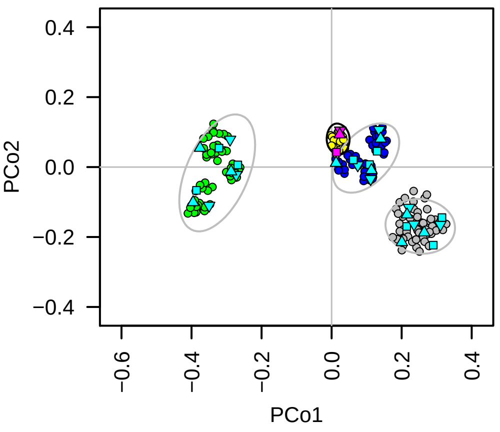

L1764 (m/z=454.265875; rt=8.91431)
T/S Cluster: L−8.9−5

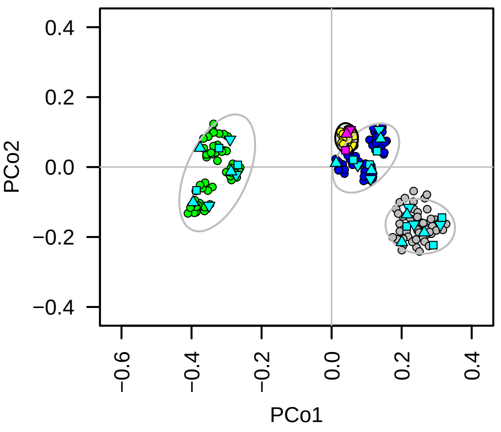

L1769 (m/z=454.270404; rt=8.91482)
T/S Cluster: L−8.9−5

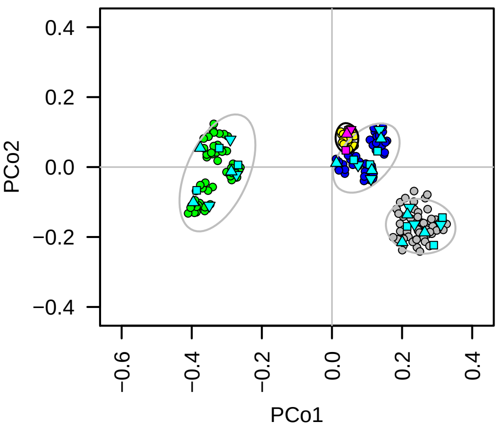

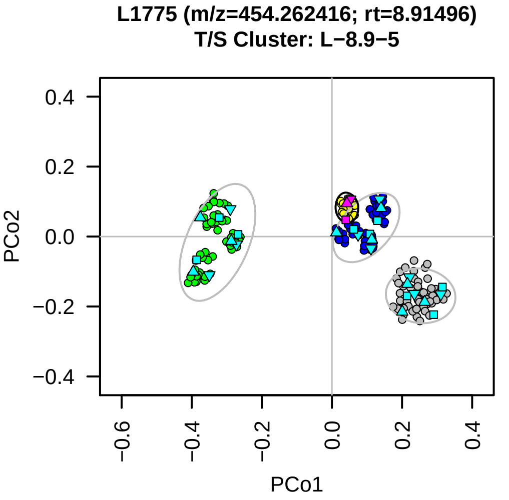
L1775 (m/z=454.262416; rt=8.91496)
T/S Cluster: L−8.9−5
PCo2
PCo1
0.4 0.2 0.0 −0.2 −0.4
−0.6 −0.4 −0.2 0.0 0.2 0.4

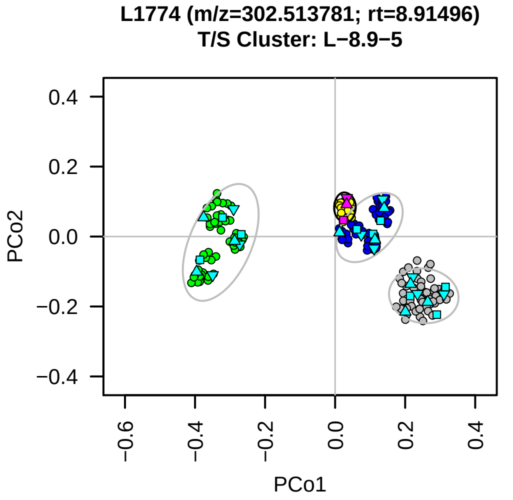
L1774 (m/z=302.513781; rt=8.91496)
T/S Cluster: L−8.9−5
PCo2
PCo1
0.4 0.2 0.0 −0.2 −0.4
−0.6 −0.4 −0.2 0.0 0.2 0.4

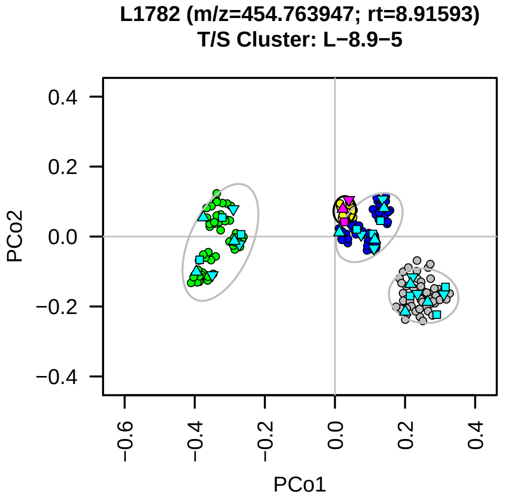
L1782 (m/z=454.763947; rt=8.91593)
T/S Cluster: L−8.9−5
PCo2
PCo1
0.4 0.2 0.0 −0.2 −0.4
−0.6 −0.4 −0.2 0.0 0.2 0.4

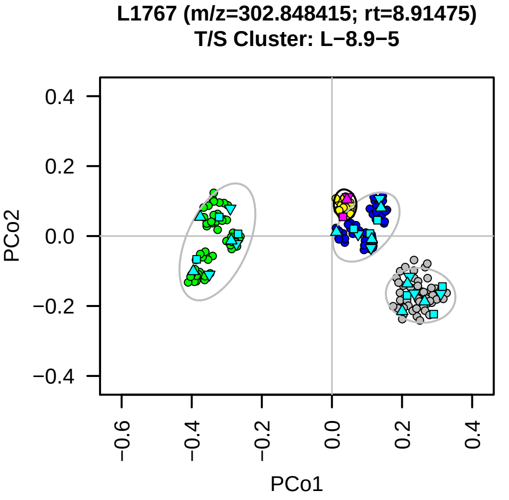
L1767 (m/z=302.848415; rt=8.91475)
T/S Cluster: L−8.9−5
PCo2
PCo1
0.4 0.2 0.0 −0.2 −0.4
−0.6 −0.4 −0.2 0.0 0.2 0.4

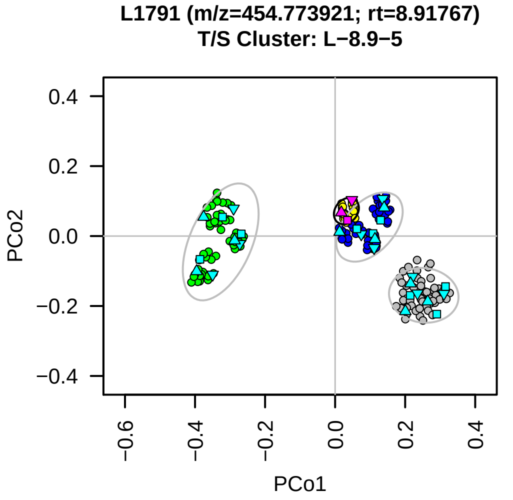
L1791 (m/z=454.773921; rt=8.91767)
T/S Cluster: L−8.9−5
PCo2
PCo1
0.4 0.2 0.0 −0.2 −0.4
−0.6 −0.4 −0.2 0.0 0.2 0.4

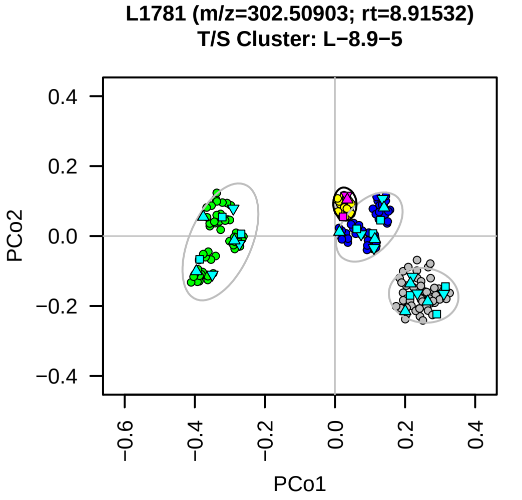
L1781 (m/z=302.50903; rt=8.91532)
T/S Cluster: L−8.9−5
PCo2
PCo1
0.4 0.2 0.0 −0.2 −0.4
−0.6 −0.4 −0.2 0.0 0.2 0.4

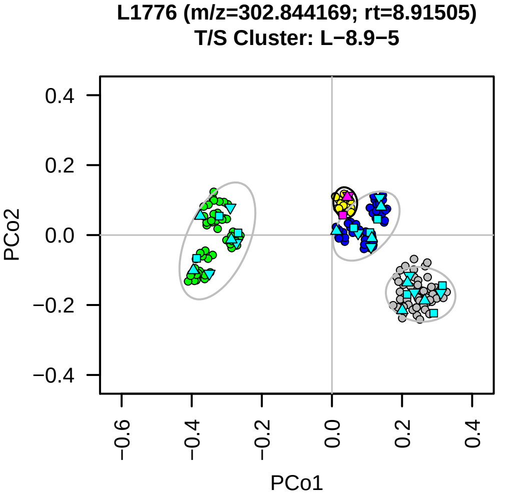
L1776 (m/z=302.844169; rt=8.91505)
T/S Cluster: L−8.9−5
PCo2
PCo1
0.4 0.2 0.0 −0.2 −0.4
−0.6 −0.4 −0.2 0.0 0.2 0.4

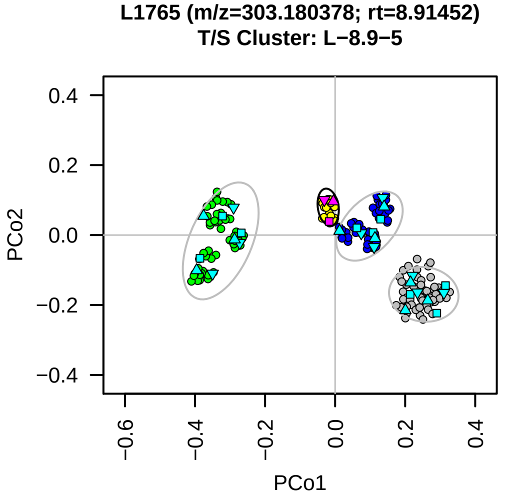
L1765 (m/z=303.180378; rt=8.91452)
T/S Cluster: L−8.9−5
PCo2
PCo1
0.4 0.2 0.0 −0.2 −0.4
−0.6 −0.4 −0.2 0.0 0.2 0.4

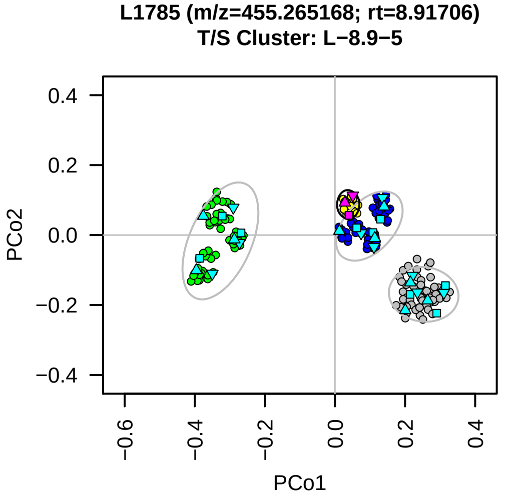
L1785 (m/z=455.265168; rt=8.91706)
T/S Cluster: L−8.9−5
PCo2
PCo1
0.4 0.2 0.0 −0.2 −0.4
−0.6 −0.4 −0.2 0.0 0.2 0.4

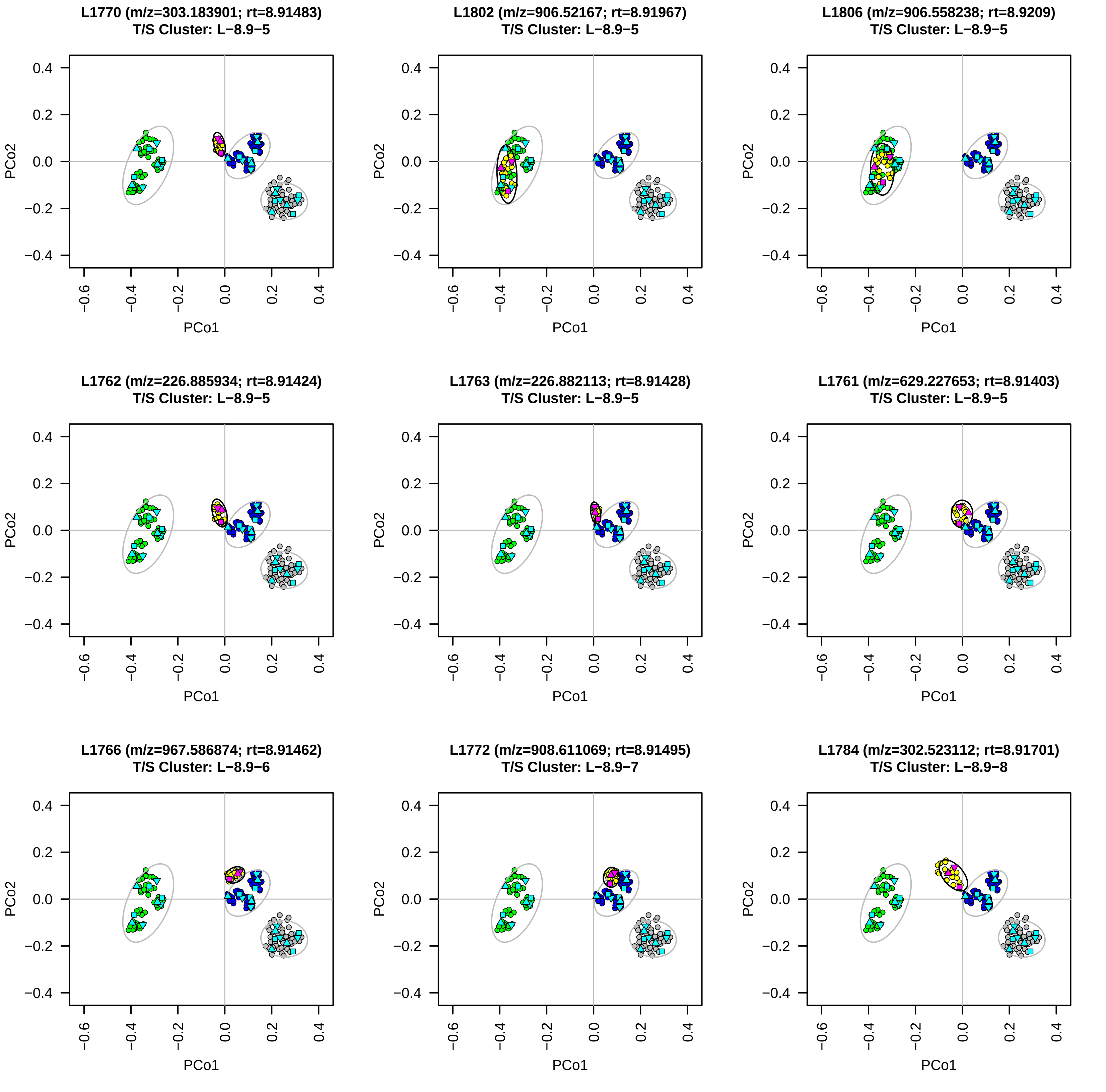
L1770 (m/z=303.183901; rt=8.91483)
T/S Cluster: L−8.9−5
L1802 (m/z=906.52167; rt=8.91967)
T/S Cluster: L−8.9−5
L1806 (m/z=906.558238; rt=8.9209)
T/S Cluster: L−8.9−5
L1762 (m/z=226.885934; rt=8.91424)
T/S Cluster: L−8.9−5
L1763 (m/z=226.882113; rt=8.91428)
T/S Cluster: L−8.9−5
L1761 (m/z=629.227653; rt=8.91403)
T/S Cluster: L−8.9−5
L1766 (m/z=967.586874; rt=8.91462)
T/S Cluster: L−8.9−6
L1772 (m/z=908.611069; rt=8.91495)
T/S Cluster: L−8.9−7
L1784 (m/z=302.523112; rt=8.91701)
T/S Cluster: L−8.9−8
PCo1
PCo2
0.4
0.2
0.0
−0.2
−0.4
−0.6

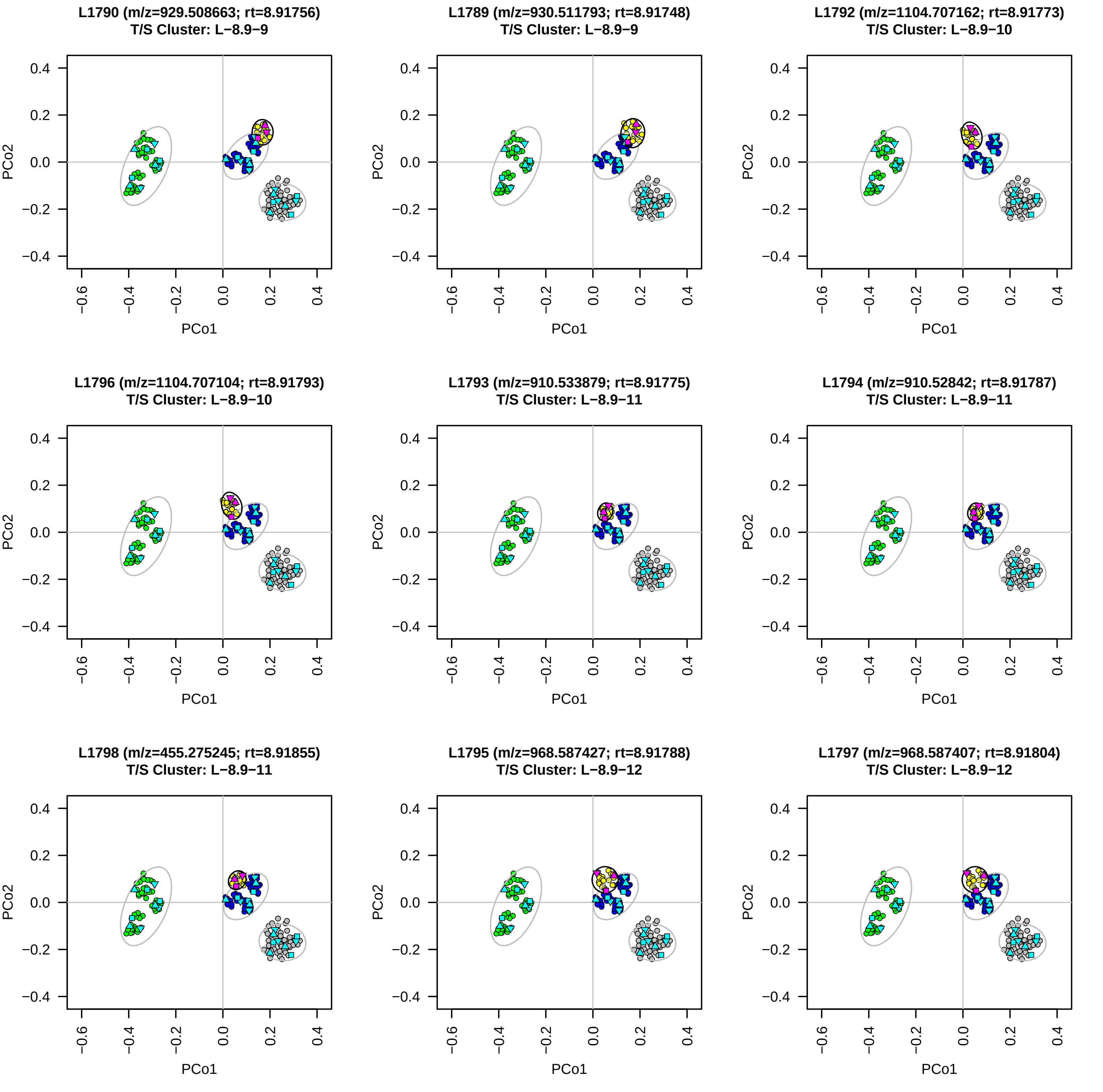
L1790 (m/z=929.508663; rt=8.91756)
T/S Cluster: L−8.9−9
L1789 (m/z=930.511793; rt=8.91748)
T/S Cluster: L−8.9−9
L1792 (m/z=1104.707162; rt=8.91773)
T/S Cluster: L−8.9−10
L1796 (m/z=1104.707104; rt=8.91793)
T/S Cluster: L−8.9−10
L1793 (m/z=910.533879; rt=8.91775)
T/S Cluster: L−8.9−11
L1794 (m/z=910.52842; rt=8.91787)
T/S Cluster: L−8.9−11
L1798 (m/z=455.275245; rt=8.91855)
T/S Cluster: L−8.9−11
L1795 (m/z=968.587427; rt=8.91788)
T/S Cluster: L−8.9−12
L1797 (m/z=968.587407; rt=8.91804)
T/S Cluster: L−8.9−12
PCo1
PCo2
−0.6
−0.4
−0.2
0.0
0.2
0.4

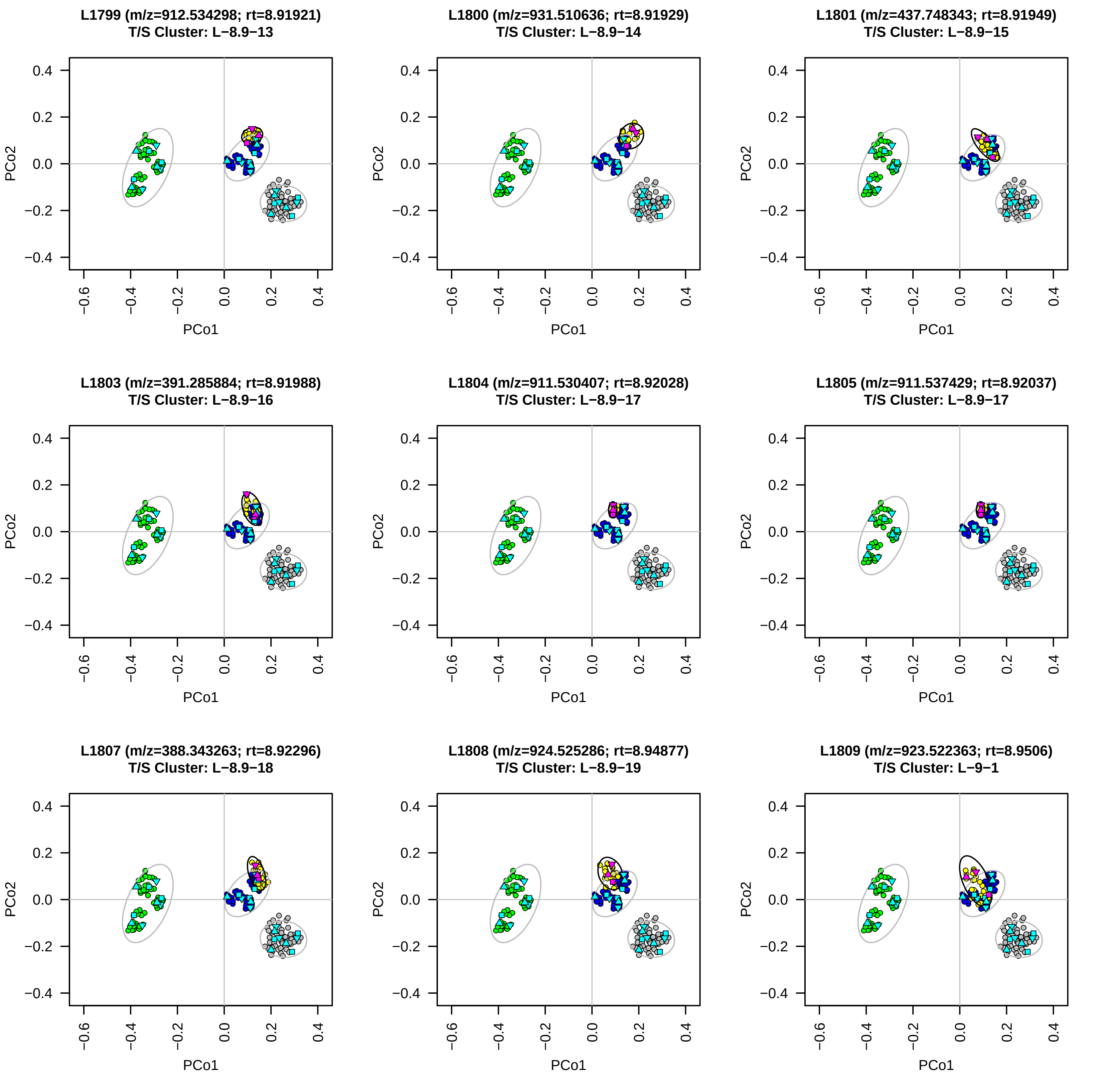
L1799 (m/z=912.534298; rt=8.91921)
T/S Cluster: L−8.9−13
L1800 (m/z=931.510636; rt=8.91929)
T/S Cluster: L−8.9−14
L1801 (m/z=437.748343; rt=8.91949)
T/S Cluster: L−8.9−15
L1803 (m/z=391.285884; rt=8.91988)
T/S Cluster: L−8.9−16
L1804 (m/z=911.530407; rt=8.92028)
T/S Cluster: L−8.9−17
L1805 (m/z=911.537429; rt=8.92037)
T/S Cluster: L−8.9−17
L1807 (m/z=388.343263; rt=8.92296)
T/S Cluster: L−8.9−18
L1808 (m/z=924.525286; rt=8.94877)
T/S Cluster: L−8.9−19
L1809 (m/z=923.522363; rt=8.9506)
T/S Cluster: L−9−1
PCo1
PCo2

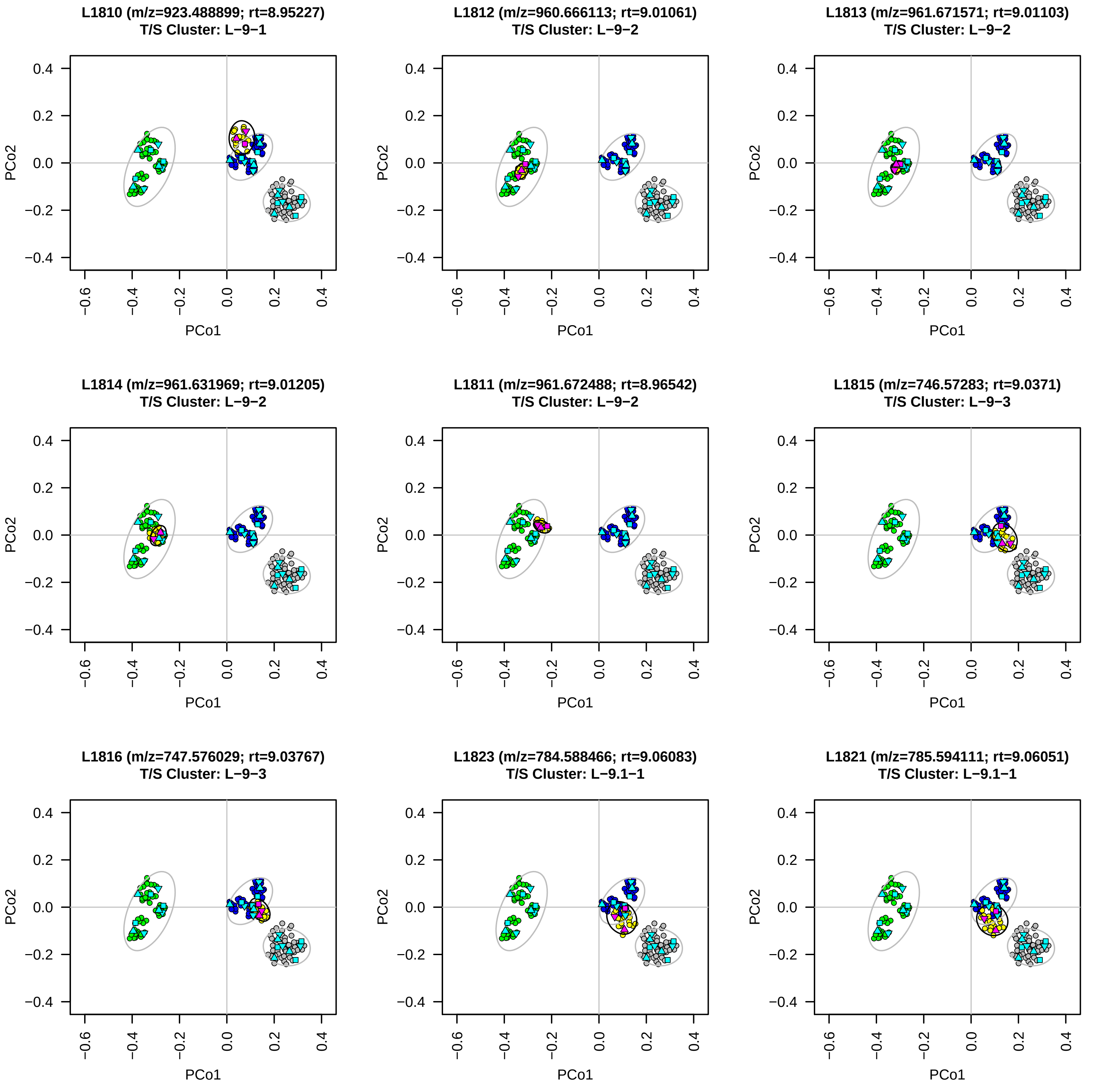
L1810 (m/z=923.488899; rt=8.95227)
T/S Cluster: L−9−1
L1812 (m/z=960.666113; rt=9.01061)
T/S Cluster: L−9−2
L1813 (m/z=961.671571; rt=9.01103)
T/S Cluster: L−9−2
L1814 (m/z=961.631969; rt=9.01205)
T/S Cluster: L−9−2
L1811 (m/z=961.672488; rt=8.96542)
T/S Cluster: L−9−2
L1815 (m/z=746.57283; rt=9.0371)
T/S Cluster: L−9−3
L1816 (m/z=747.576029; rt=9.03767)
T/S Cluster: L−9−3
L1823 (m/z=784.588466; rt=9.06083)
T/S Cluster: L−9.1−1
L1821 (m/z=785.594111; rt=9.06051)
T/S Cluster: L−9.1−1
PCo1
PCo2
0.4
0.2
0.0
−0.2
−0.4
−0.6

L1822 (m/z=784.556825; rt=9.06078)
T/S Cluster: L−9.1−1

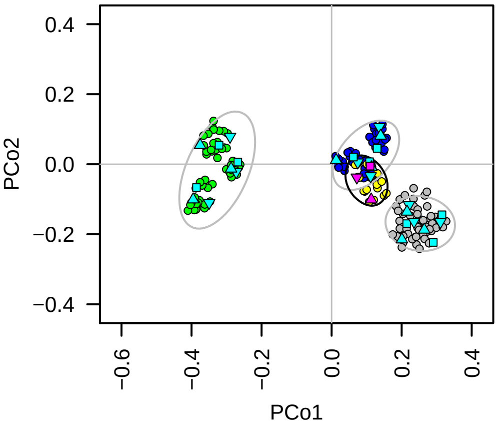

L1820 (m/z=785.570877; rt=9.06048)
T/S Cluster: L−9.1−1

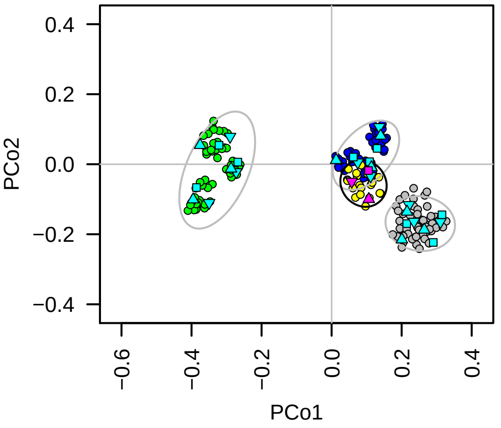

L1829 (m/z=758.572127; rt=9.09258)
T/S Cluster: L−9.1−1

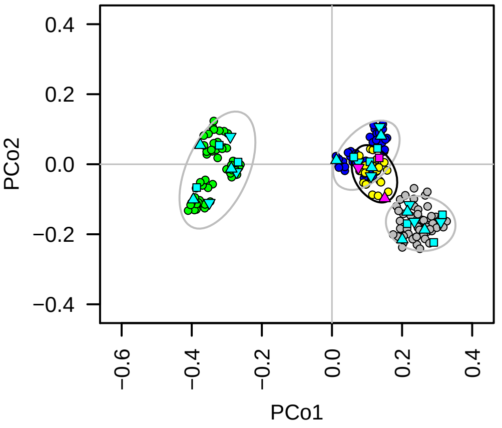

L1819 (m/z=786.598008; rt=9.05923)
T/S Cluster: L−9.1−1

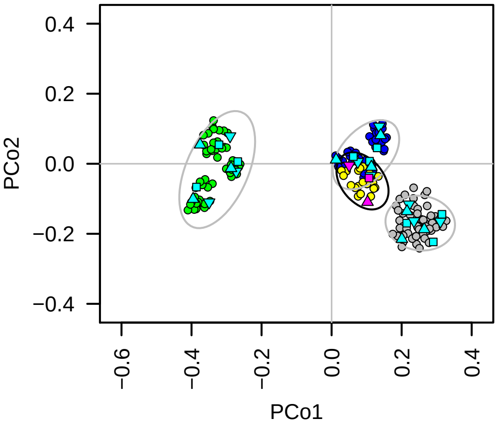

L1824 (m/z=392.298975; rt=9.06084)
T/S Cluster: L−9.1−1

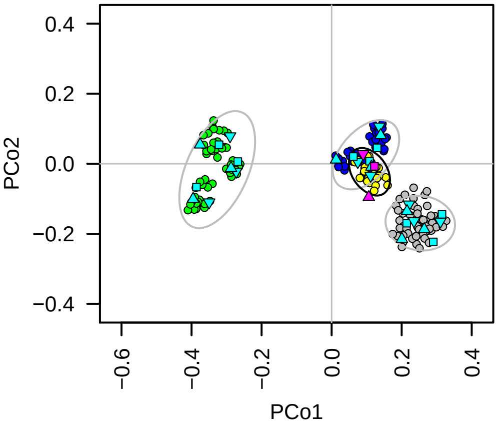

L1825 (m/z=392.292751; rt=9.06091)
T/S Cluster: L−9.1−1

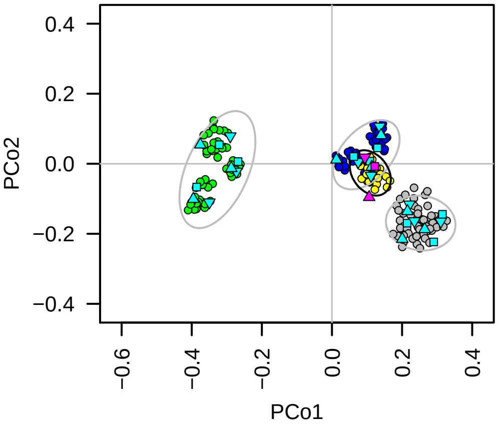

L1817 (m/z=786.574369; rt=9.05738)
T/S Cluster: L−9.1−1

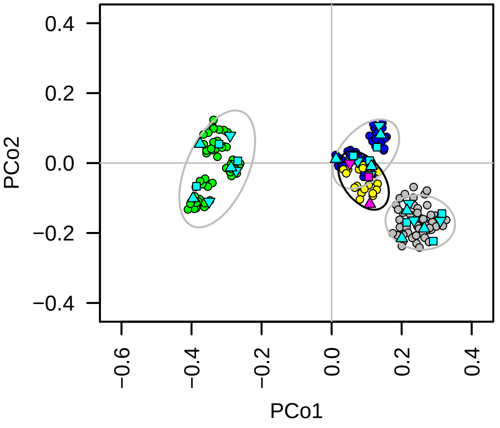

L1828 (m/z=759.576187; rt=9.09158)
T/S Cluster: L−9.1−1

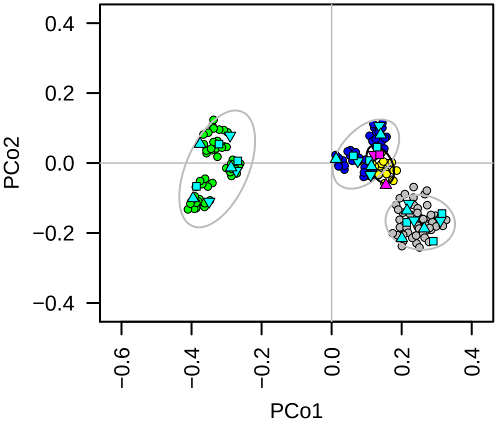

L1826 (m/z=392.796745; rt=9.06093)
T/S Cluster: L−9.1−1

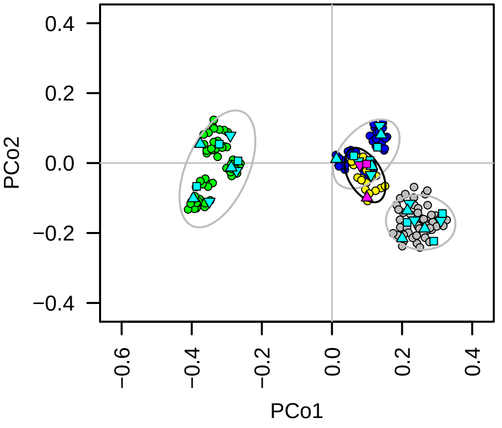

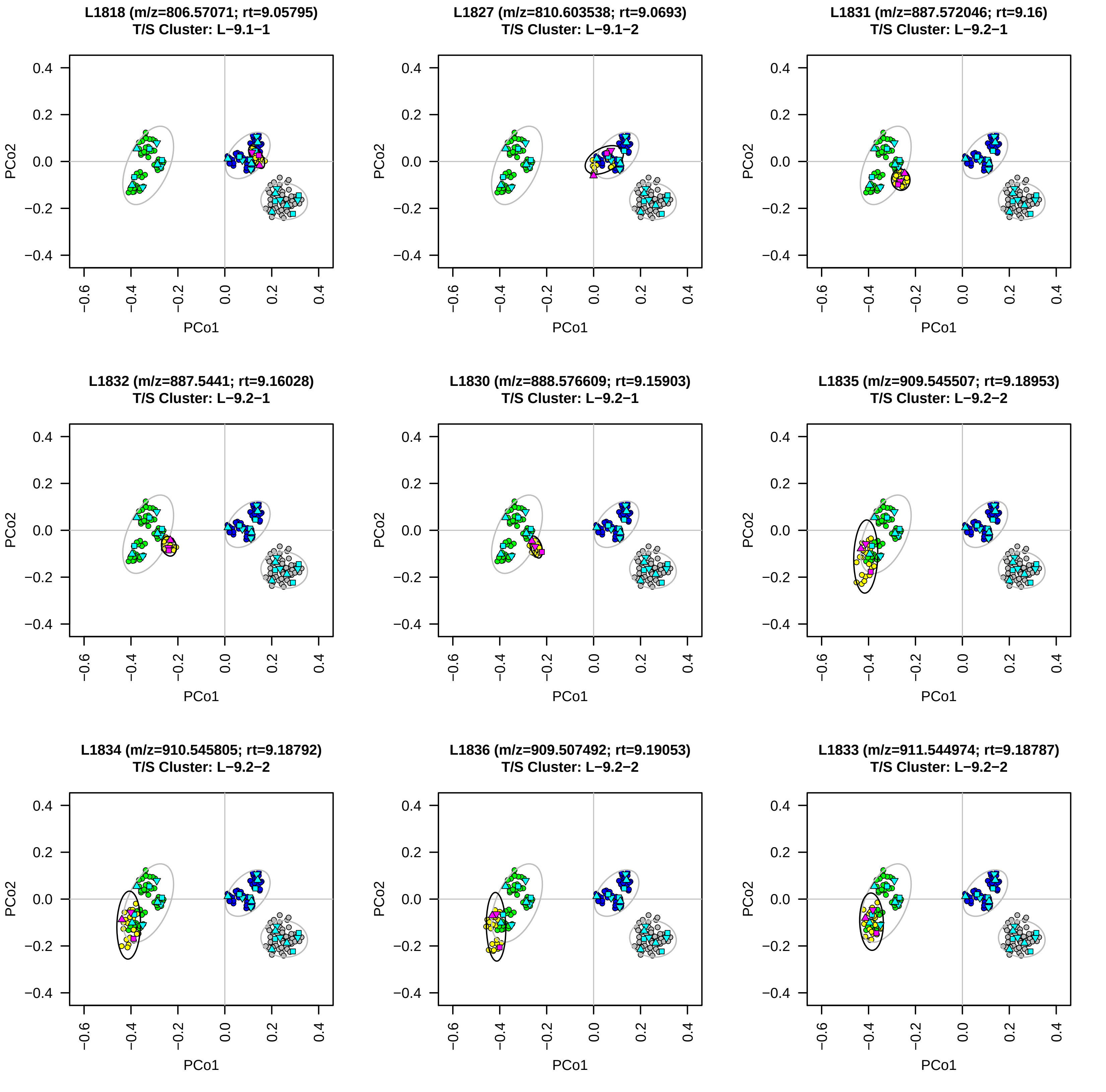

L1818 (m/z=806.57071; rt=9.05795)
T/S Cluster: L−9.1−1
L1827 (m/z=810.603538; rt=9.0693)
T/S Cluster: L−9.1−2
L1831 (m/z=887.572046; rt=9.16)
T/S Cluster: L−9.2−1
L1832 (m/z=887.5441; rt=9.16028)
T/S Cluster: L−9.2−1
L1830 (m/z=888.576609; rt=9.15903)
T/S Cluster: L−9.2−1
L1835 (m/z=909.545507; rt=9.18953)
T/S Cluster: L−9.2−2
L1834 (m/z=910.545805; rt=9.18792)
T/S Cluster: L−9.2−2
L1836 (m/z=909.507492; rt=9.19053)
T/S Cluster: L−9.2−2
L1833 (m/z=911.544974; rt=9.18787)
T/S Cluster: L−9.2−2
PCo1
PCo2

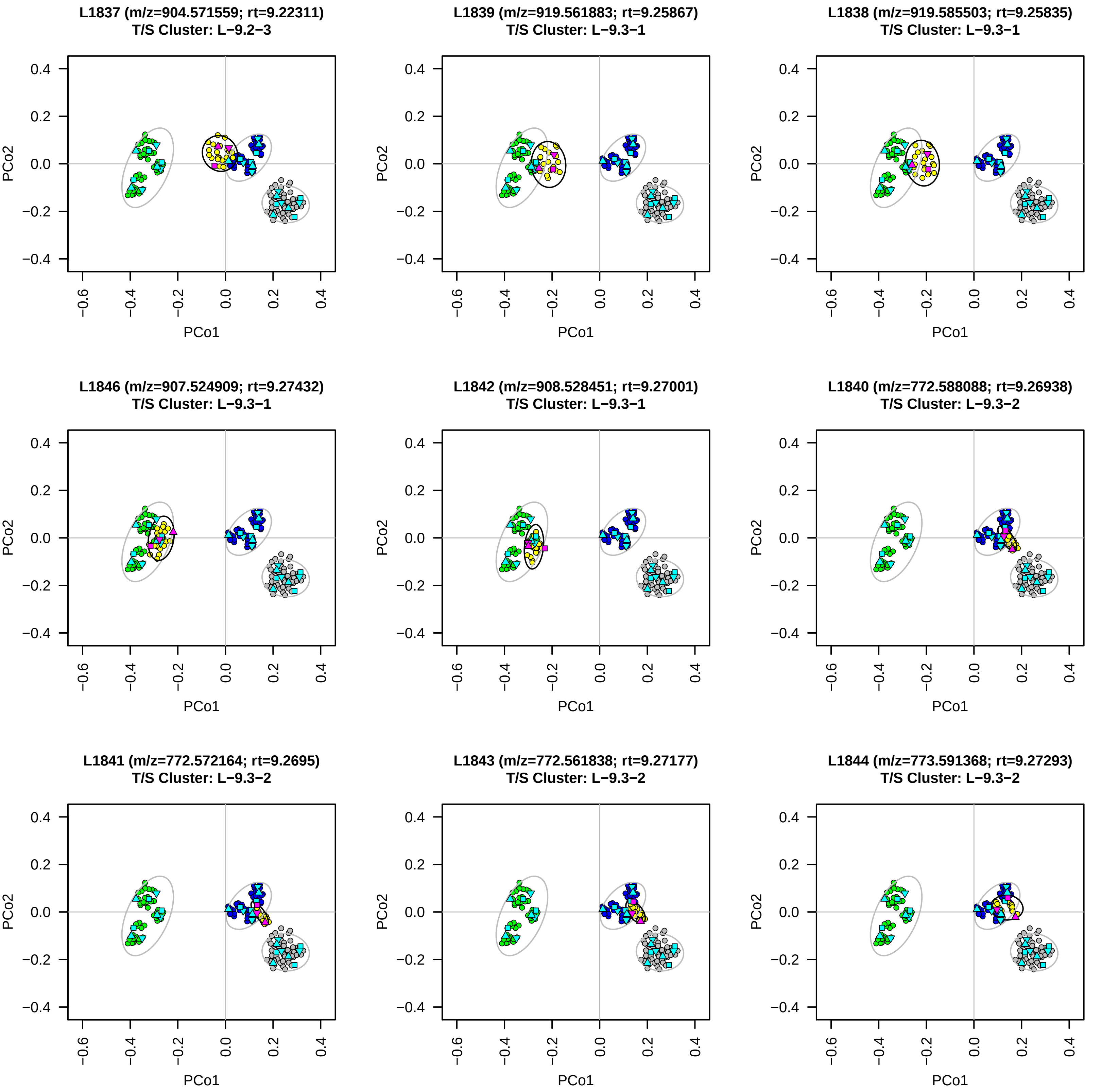
L1837 (m/z=904.571559; rt=9.22311)
T/S Cluster: L−9.2−3
L1839 (m/z=919.561883; rt=9.25867)
T/S Cluster: L−9.3−1
L1838 (m/z=919.585503; rt=9.25835)
T/S Cluster: L−9.3−1
L1846 (m/z=907.524909; rt=9.27432)
T/S Cluster: L−9.3−1
L1842 (m/z=908.528451; rt=9.27001)
T/S Cluster: L−9.3−1
L1840 (m/z=772.588088; rt=9.26938)
T/S Cluster: L−9.3−2
L1841 (m/z=772.572164; rt=9.2695)
T/S Cluster: L−9.3−2
L1843 (m/z=772.561838; rt=9.27177)
T/S Cluster: L−9.3−2
L1844 (m/z=773.591368; rt=9.27293)
T/S Cluster: L−9.3−2
PCo1
PCo2
0.4
0.2
0.0
−0.2
−0.4
−0.6
−0.4
−0.2
0.0
0.2
0.4

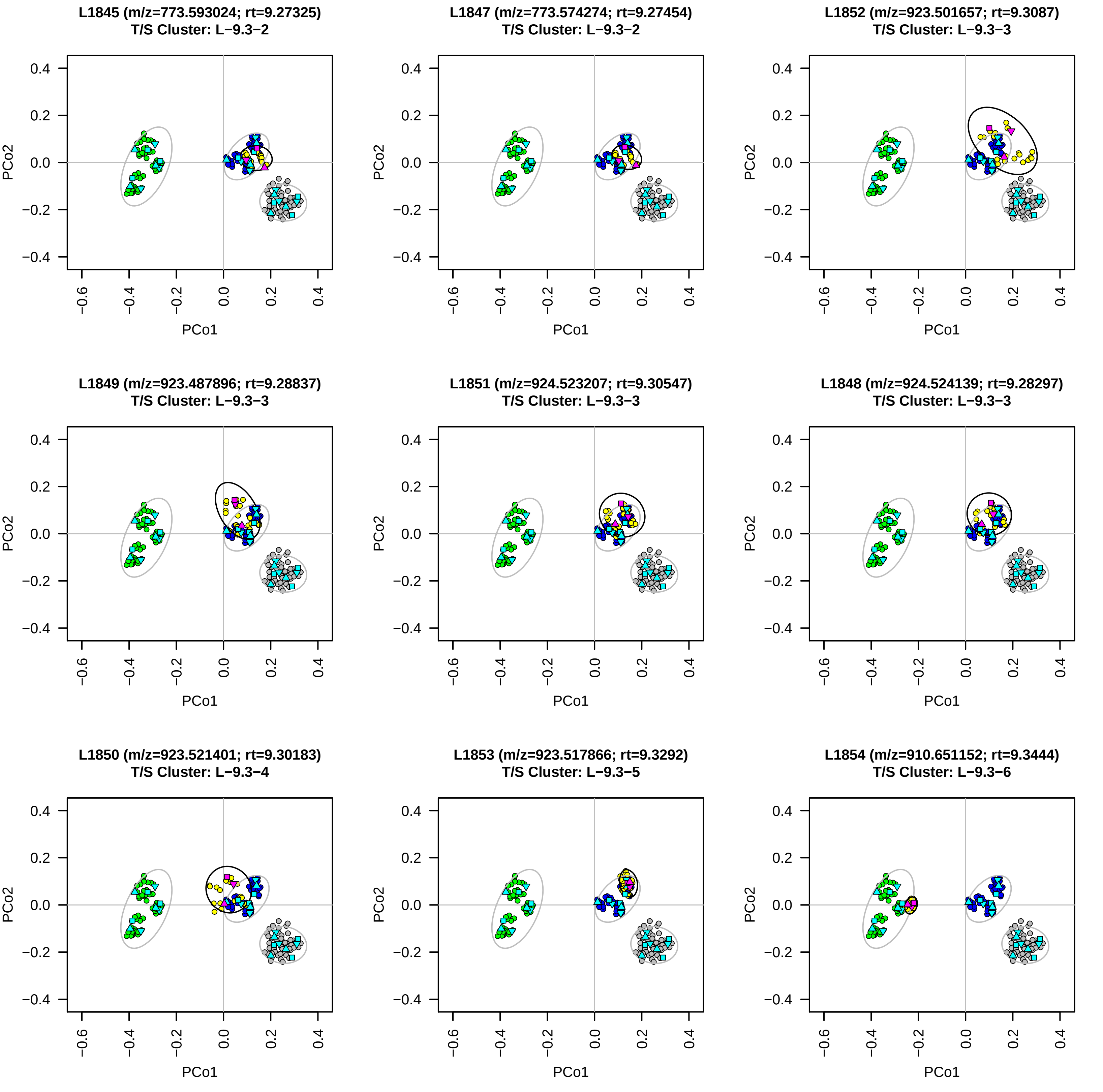
L1845 (m/z=773.593024; rt=9.27325)
T/S Cluster: L−9.3−2
L1847 (m/z=773.574274; rt=9.27454)
T/S Cluster: L−9.3−2
L1852 (m/z=923.501657; rt=9.3087)
T/S Cluster: L−9.3−3
L1849 (m/z=923.487896; rt=9.28837)
T/S Cluster: L−9.3−3
L1851 (m/z=924.523207; rt=9.30547)
T/S Cluster: L−9.3−3
L1848 (m/z=924.524139; rt=9.28297)
T/S Cluster: L−9.3−3
L1850 (m/z=923.521401; rt=9.30183)
T/S Cluster: L−9.3−4
L1853 (m/z=923.517866; rt=9.3292)
T/S Cluster: L−9.3−5
L1854 (m/z=910.651152; rt=9.3444)
T/S Cluster: L−9.3−6
PCo1
PCo2
0.4
0.2
0.0
−0.2
−0.4
−0.6

L1855 (m/z=911.654746; rt=9.34441)
T/S Cluster: L−9.3−6

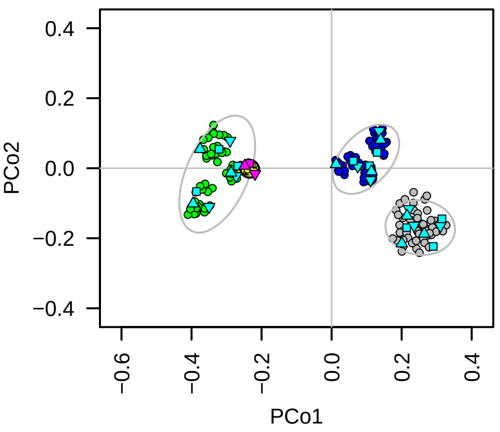

L1856 (m/z=915.606045; rt=9.34536)
T/S Cluster: L−9.3−6

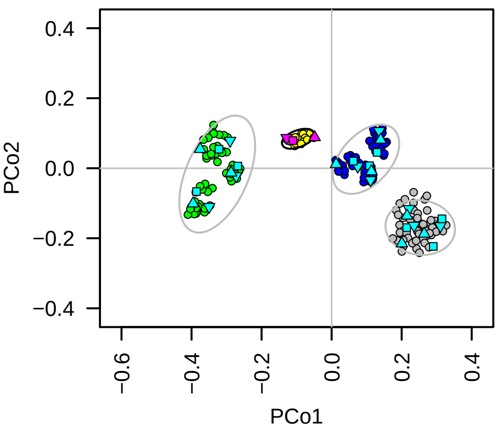

L1861 (m/z=907.52572; rt=9.4348)
T/S Cluster: L−9.4−1

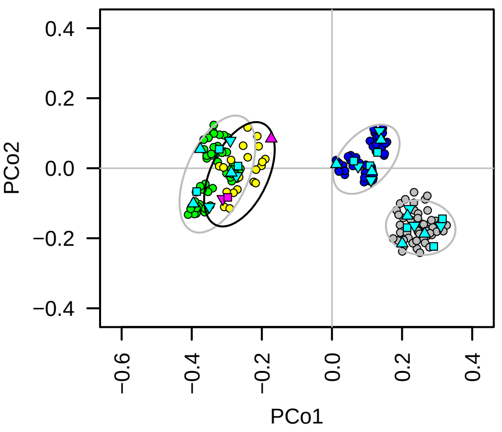

L1859 (m/z=908.528466; rt=9.43462)
T/S Cluster: L−9.4−1

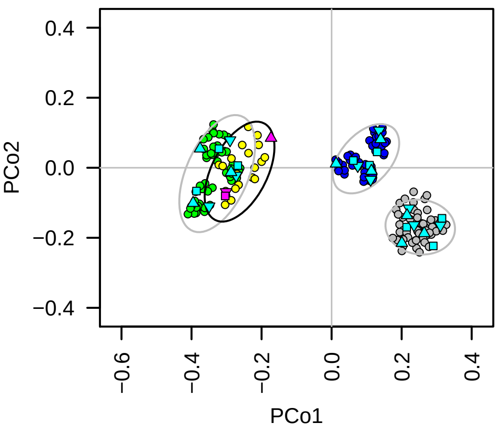

L1869 (m/z=909.503828; rt=9.43847)
T/S Cluster: L−9.4−1

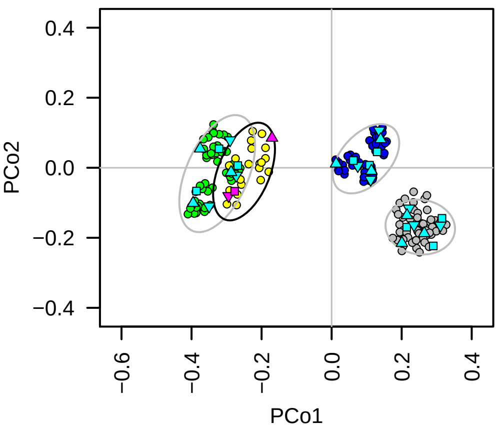

L1858 (m/z=907.466113; rt=9.43455)
T/S Cluster: L−9.4−1

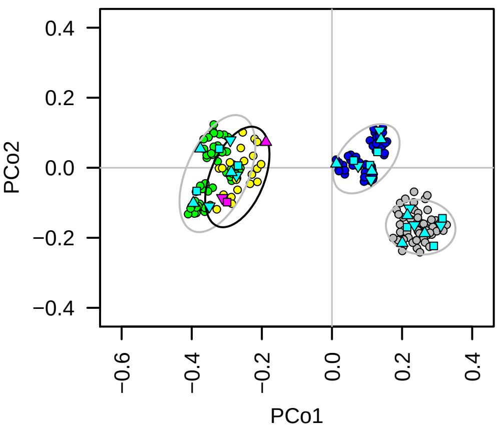

L1866 (m/z=908.589221; rt=9.43549)
T/S Cluster: L−9.4−1

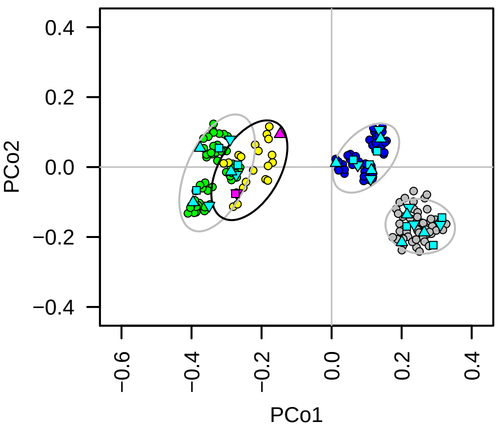

L1860 (m/z=453.761788; rt=9.43474)
T/S Cluster: L−9.4−1

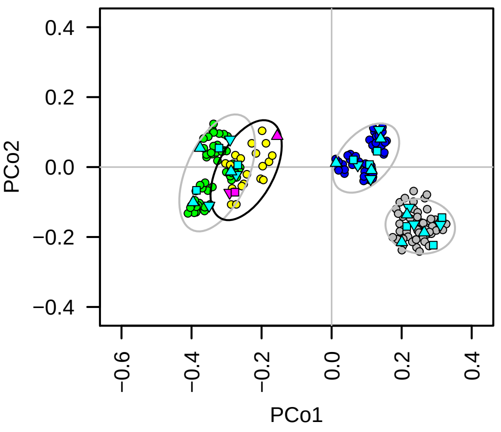

L1862 (m/z=908.46245; rt=9.43485)
T/S Cluster: L−9.4−1

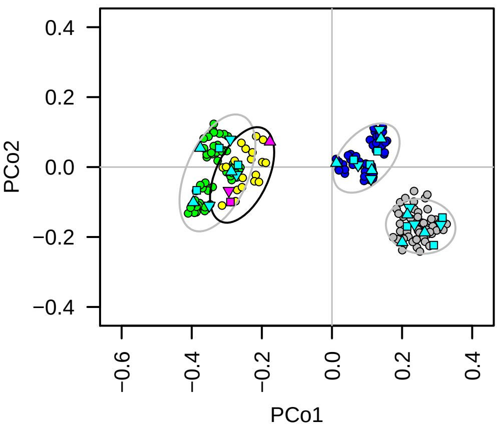

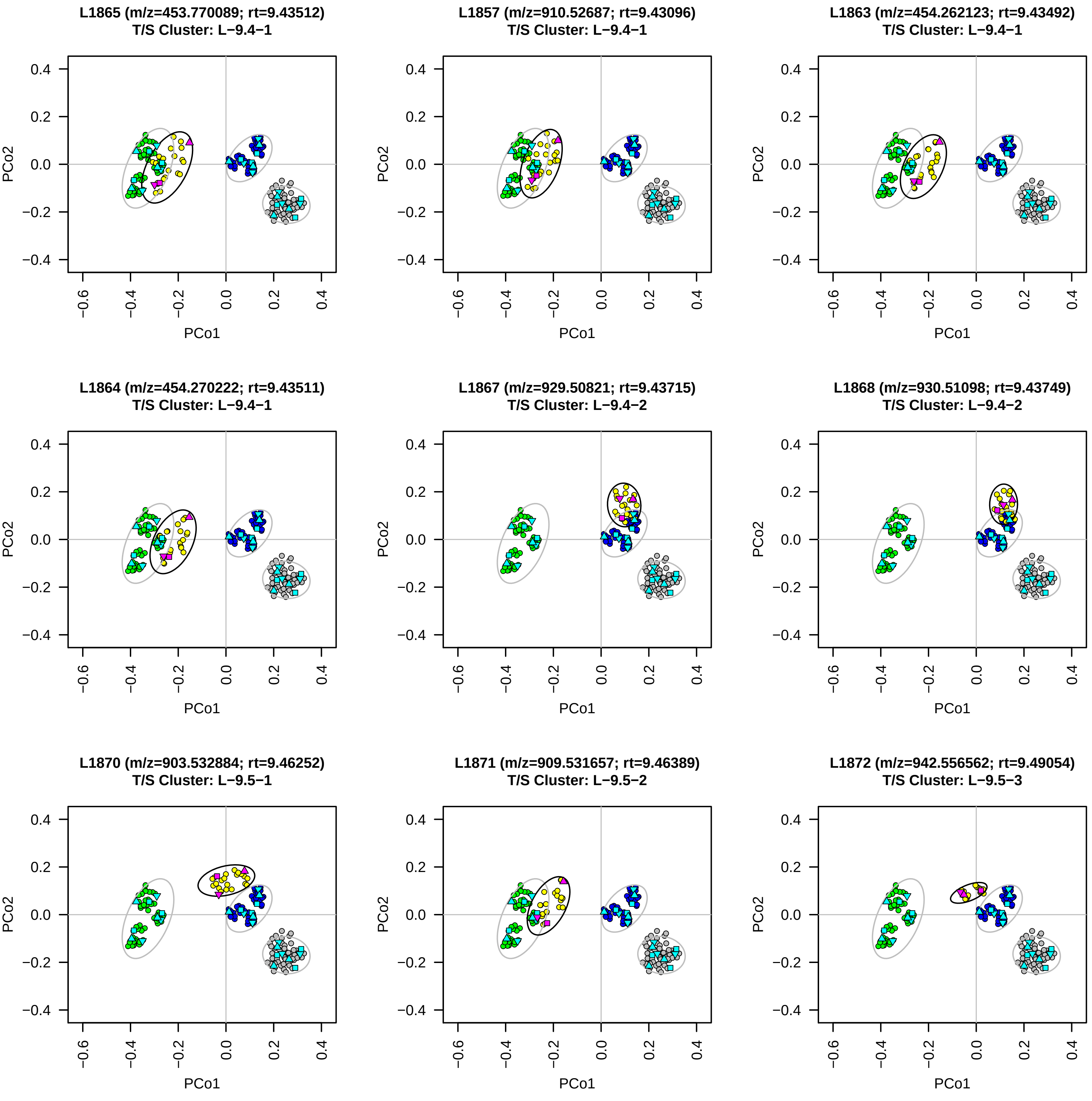
L1865 (m/z=453.770089; rt=9.43512)
T/S Cluster: L−9.4−1
L1857 (m/z=910.52687; rt=9.43096)
T/S Cluster: L−9.4−1
L1863 (m/z=454.262123; rt=9.43492)
T/S Cluster: L−9.4−1
L1864 (m/z=454.270222; rt=9.43511)
T/S Cluster: L−9.4−1
L1867 (m/z=929.50821; rt=9.43715)
T/S Cluster: L−9.4−2
L1868 (m/z=930.51098; rt=9.43749)
T/S Cluster: L−9.4−2
L1870 (m/z=903.532884; rt=9.46252)
T/S Cluster: L−9.5−1
L1871 (m/z=909.531657; rt=9.46389)
T/S Cluster: L−9.5−2
L1872 (m/z=942.556562; rt=9.49054)
T/S Cluster: L−9.5−3
PCo1
PCo2
−0.6
−0.4
−0.2
0.0
0.2
0.4

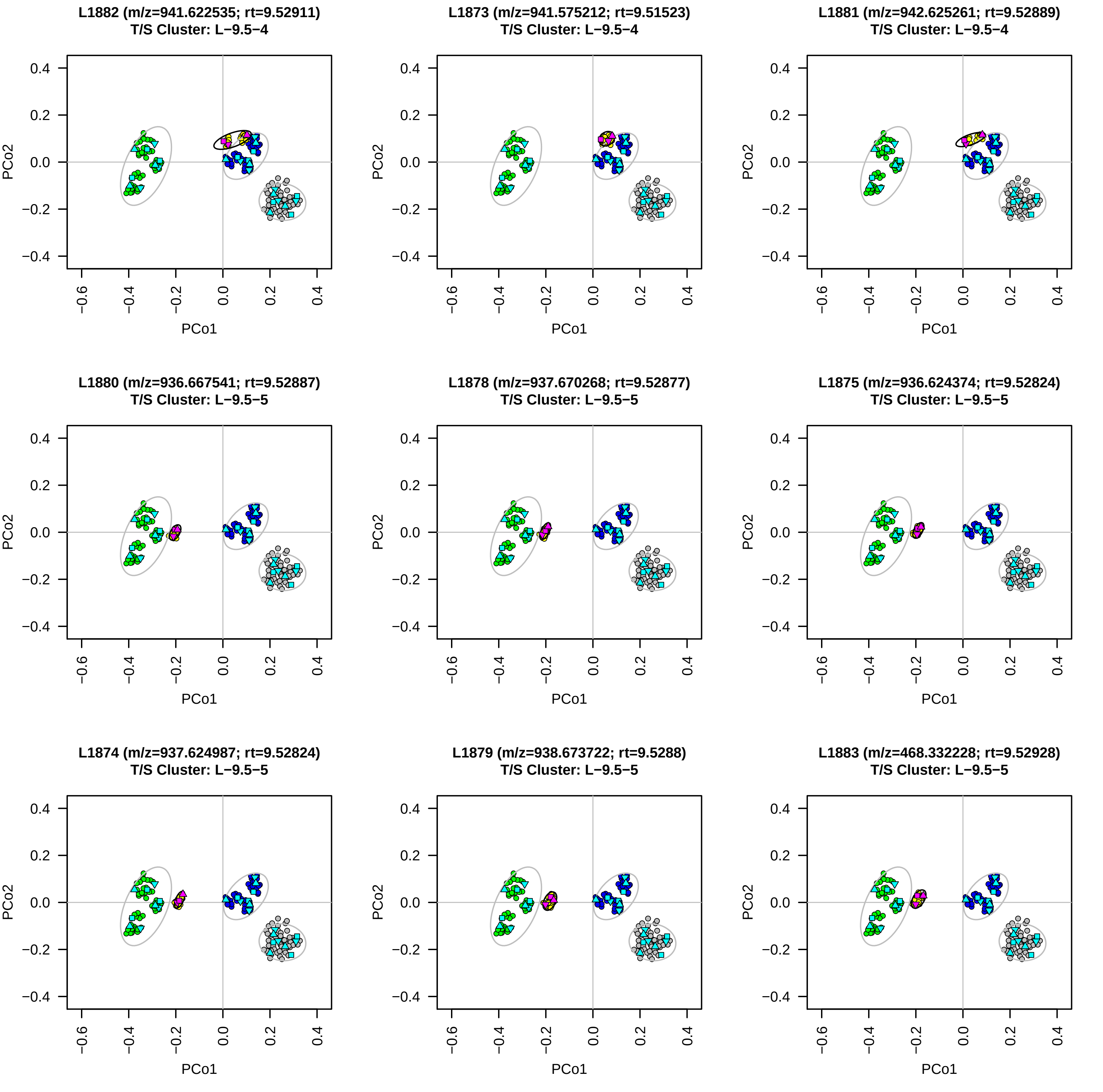
L1882 (m/z=941.622535; rt=9.52911)
T/S Cluster: L−9.5−4
L1873 (m/z=941.575212; rt=9.51523)
T/S Cluster: L−9.5−4
L1881 (m/z=942.625261; rt=9.52889)
T/S Cluster: L−9.5−4
L1880 (m/z=936.667541; rt=9.52887)
T/S Cluster: L−9.5−5
L1878 (m/z=937.670268; rt=9.52877)
T/S Cluster: L−9.5−5
L1875 (m/z=936.624374; rt=9.52824)
T/S Cluster: L−9.5−5
L1874 (m/z=937.624987; rt=9.52824)
T/S Cluster: L−9.5−5
L1879 (m/z=938.673722; rt=9.5288)
T/S Cluster: L−9.5−5
L1883 (m/z=468.332228; rt=9.52928)
T/S Cluster: L−9.5−5
PCo1
PCo2
0.4
0.2
0.0
−0.2
−0.4
−0.6

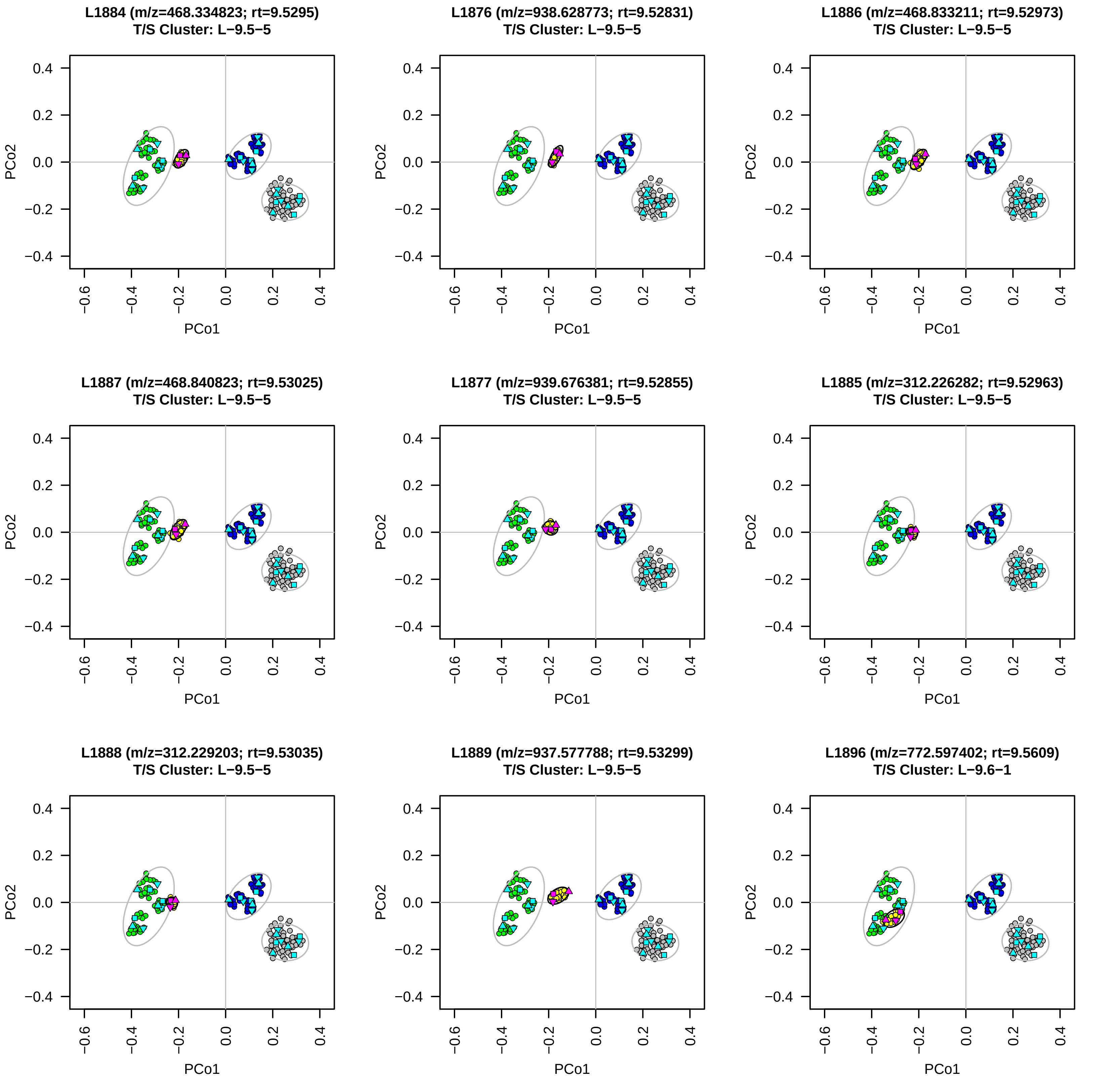
L1884 (m/z=468.334823; rt=9.5295)
T/S Cluster: L−9.5−5
L1876 (m/z=938.628773; rt=9.52831)
T/S Cluster: L−9.5−5
L1886 (m/z=468.833211; rt=9.52973)
T/S Cluster: L−9.5−5
L1887 (m/z=468.840823; rt=9.53025)
T/S Cluster: L−9.5−5
L1877 (m/z=939.676381; rt=9.52855)
T/S Cluster: L−9.5−5
L1885 (m/z=312.226282; rt=9.52963)
T/S Cluster: L−9.5−5
L1888 (m/z=312.229203; rt=9.53035)
T/S Cluster: L−9.5−5
L1889 (m/z=937.577788; rt=9.53299)
T/S Cluster: L−9.5−5
L1896 (m/z=772.597402; rt=9.5609)
T/S Cluster: L−9.6−1
PCo1
PCo2
0.4
0.2
0.0
−0.2
−0.4
−0.6

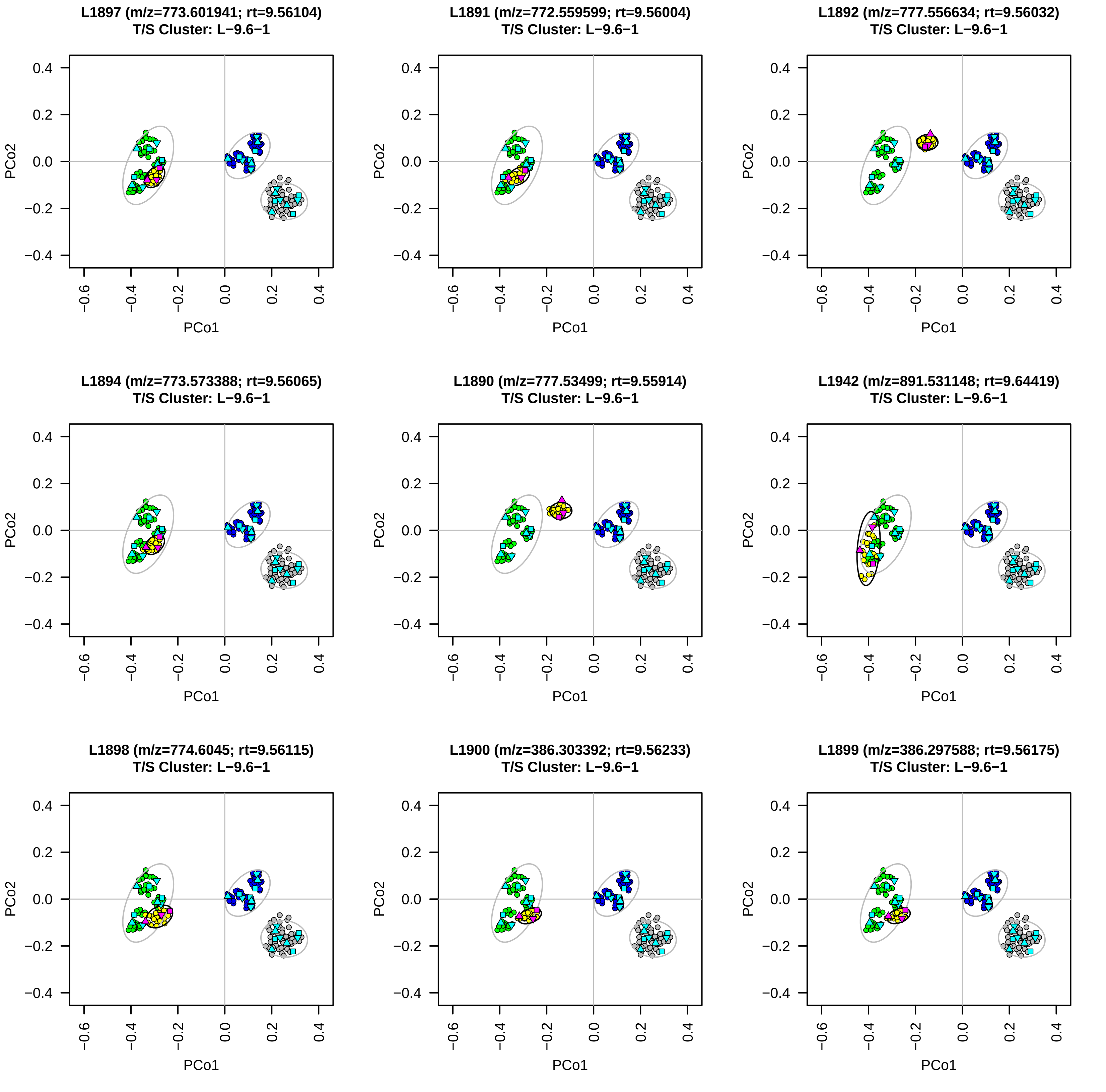
L1897 (m/z=773.601941; rt=9.56104)
T/S Cluster: L−9.6−1
L1891 (m/z=772.559599; rt=9.56004)
T/S Cluster: L−9.6−1
L1892 (m/z=777.556634; rt=9.56032)
T/S Cluster: L−9.6−1
L1894 (m/z=773.573388; rt=9.56065)
T/S Cluster: L−9.6−1
L1890 (m/z=777.53499; rt=9.55914)
T/S Cluster: L−9.6−1
L1942 (m/z=891.531148; rt=9.64419)
T/S Cluster: L−9.6−1
L1898 (m/z=774.6045; rt=9.56115)
T/S Cluster: L−9.6−1
L1900 (m/z=386.303392; rt=9.56233)
T/S Cluster: L−9.6−1
L1899 (m/z=386.297588; rt=9.56175)
T/S Cluster: L−9.6−1
PCo1
PCo2

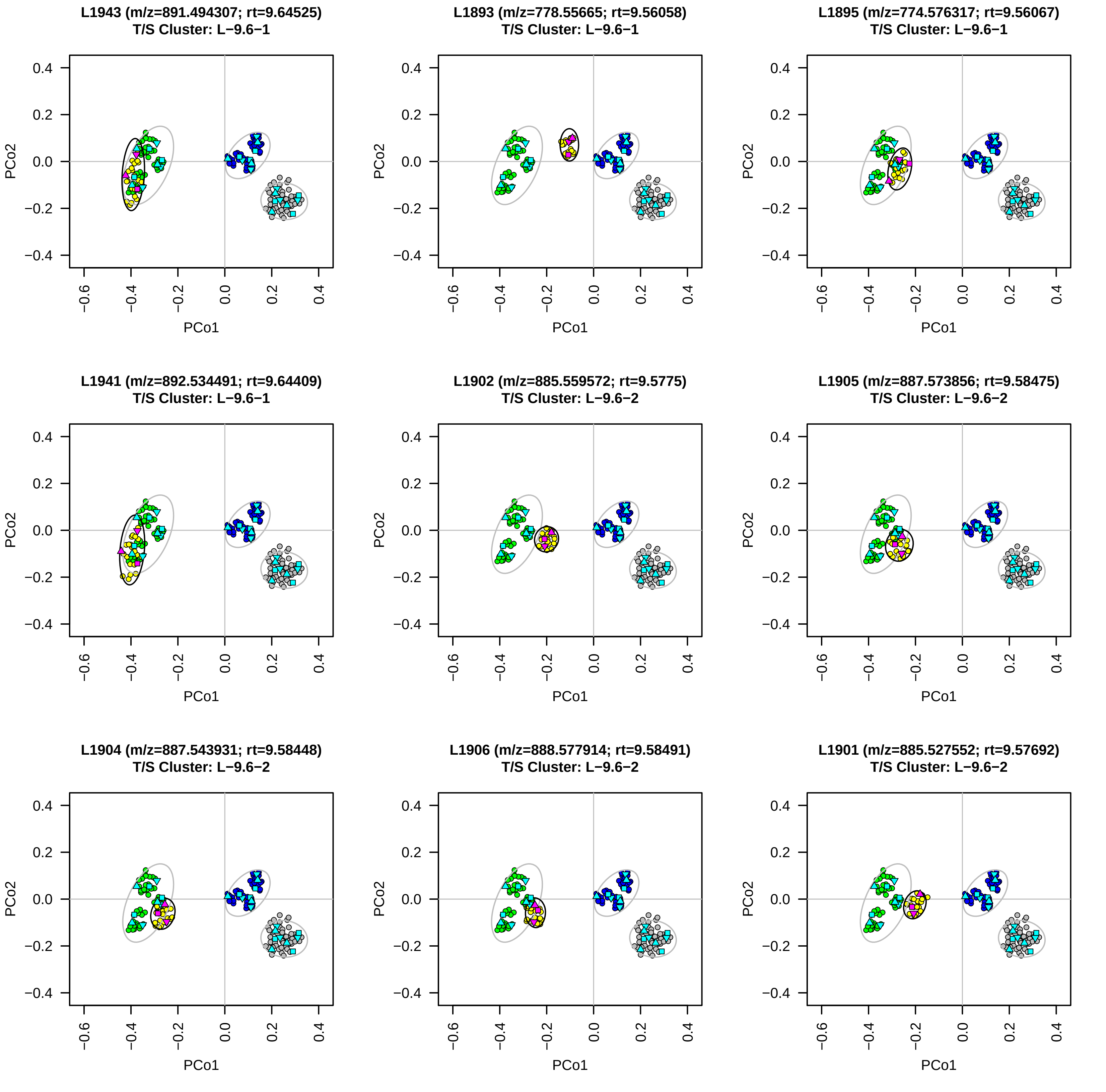
L1943 (m/z=891.494307; rt=9.64525)
T/S Cluster: L−9.6−1
L1893 (m/z=778.55665; rt=9.56058)
T/S Cluster: L−9.6−1
L1895 (m/z=774.576317; rt=9.56067)
T/S Cluster: L−9.6−1
L1941 (m/z=892.534491; rt=9.64409)
T/S Cluster: L−9.6−1
L1902 (m/z=885.559572; rt=9.5775)
T/S Cluster: L−9.6−2
L1905 (m/z=887.573856; rt=9.58475)
T/S Cluster: L−9.6−2
L1904 (m/z=887.543931; rt=9.58448)
T/S Cluster: L−9.6−2
L1906 (m/z=888.577914; rt=9.58491)
T/S Cluster: L−9.6−2
L1901 (m/z=885.527552; rt=9.57692)
T/S Cluster: L−9.6−2
PCo1
PCo2
0.4
0.2
0.0
−0.2
−0.4
−0.6

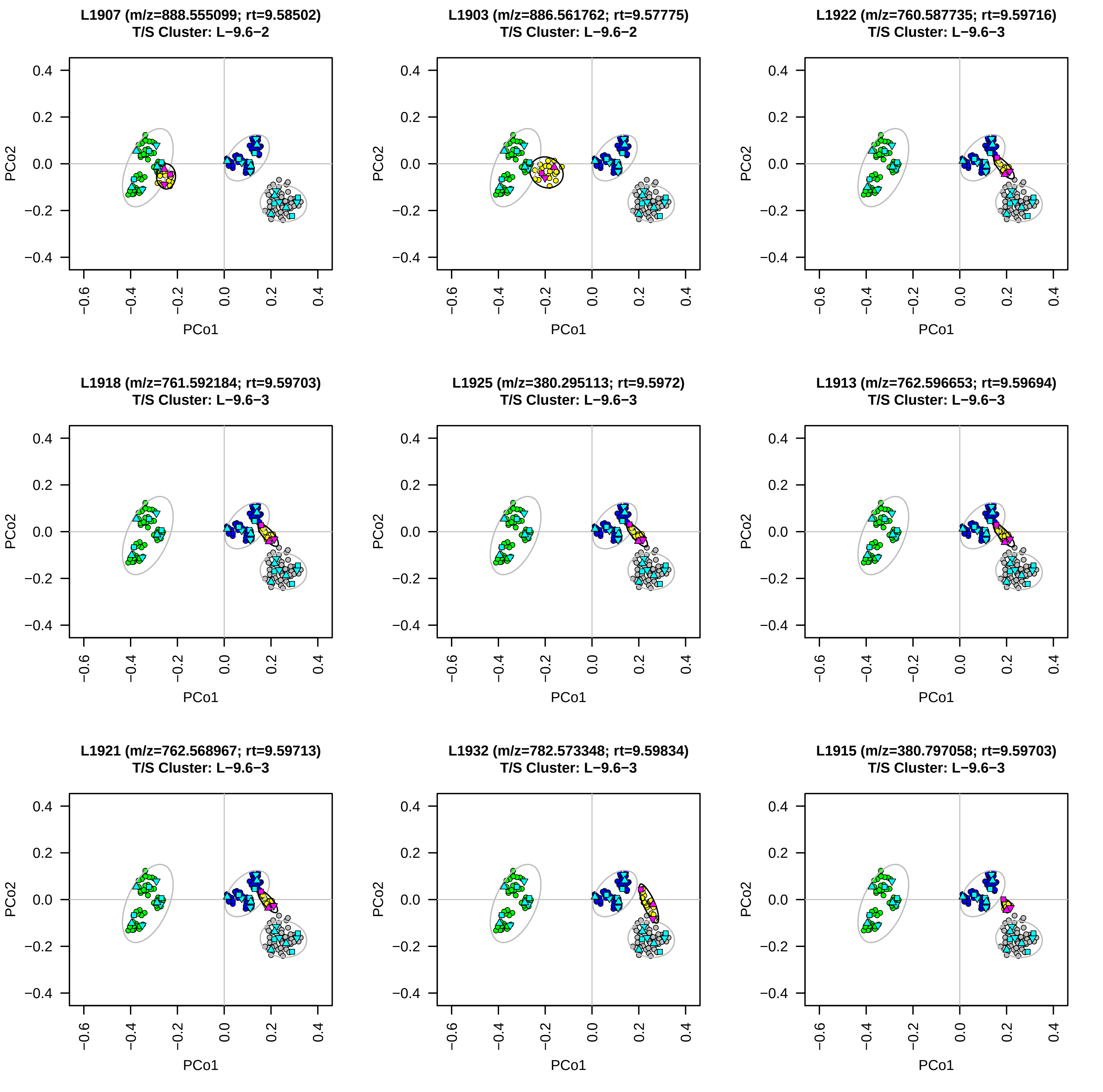
L1907 (m/z=888.555099; rt=9.58502)
T/S Cluster: L−9.6−2
L1903 (m/z=886.561762; rt=9.57775)
T/S Cluster: L−9.6−2
L1922 (m/z=760.587735; rt=9.59716)
T/S Cluster: L−9.6−3
L1918 (m/z=761.592184; rt=9.59703)
T/S Cluster: L−9.6−3
L1925 (m/z=380.295113; rt=9.5972)
T/S Cluster: L−9.6−3
L1913 (m/z=762.596653; rt=9.59694)
T/S Cluster: L−9.6−3
L1921 (m/z=762.568967; rt=9.59713)
T/S Cluster: L−9.6−3
L1932 (m/z=782.573348; rt=9.59834)
T/S Cluster: L−9.6−3
L1915 (m/z=380.797058; rt=9.59703)
T/S Cluster: L−9.6−3
PCo1
PCo2
−0.6
−0.4
−0.2
0.0
0.2
0.4

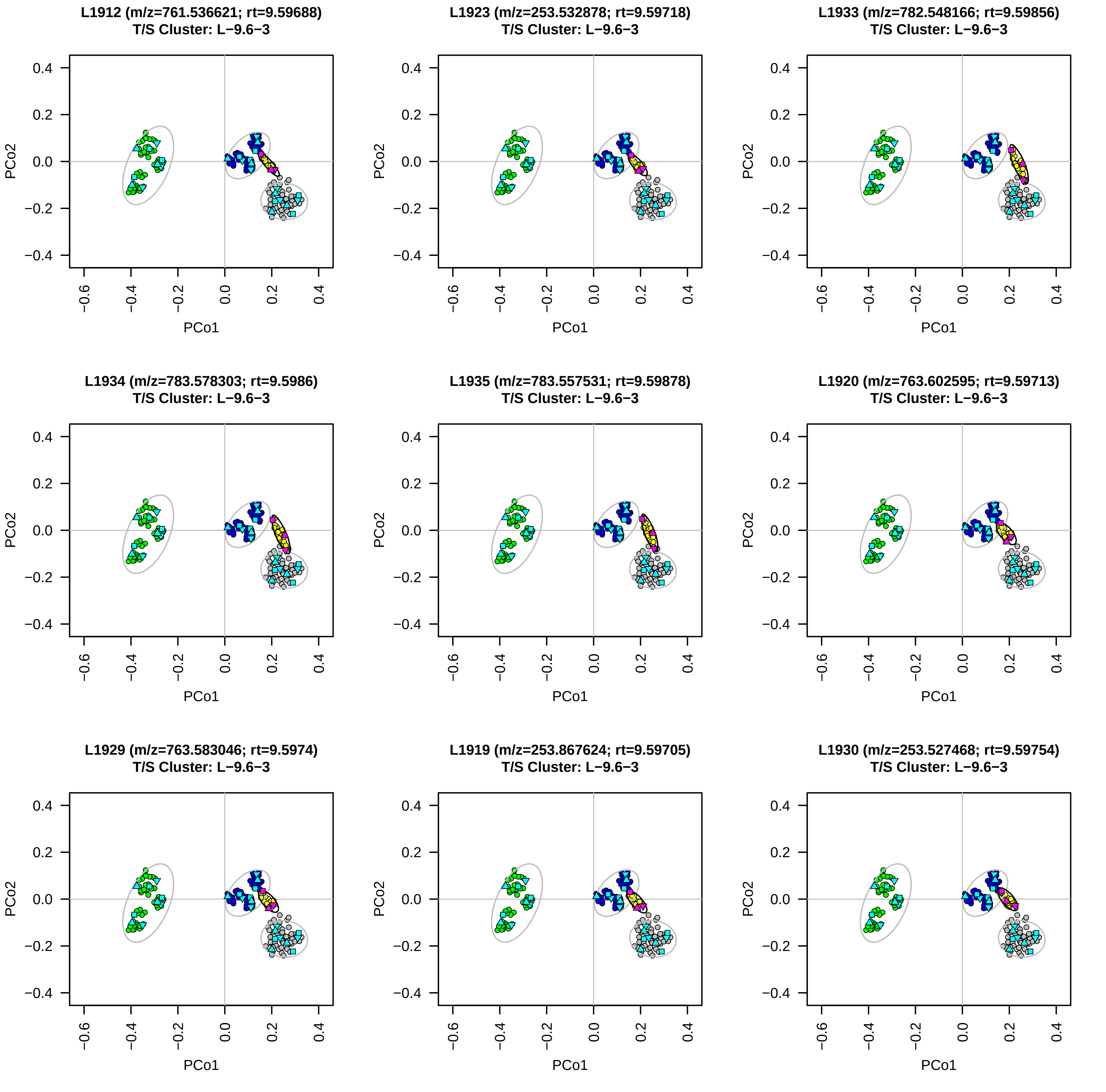
L1912 (m/z=761.536621; rt=9.59688)
T/S Cluster: L−9.6−3
L1923 (m/z=253.532878; rt=9.59718)
T/S Cluster: L−9.6−3
L1933 (m/z=782.548166; rt=9.59856)
T/S Cluster: L−9.6−3
L1934 (m/z=783.578303; rt=9.5986)
T/S Cluster: L−9.6−3
L1935 (m/z=783.557531; rt=9.59878)
T/S Cluster: L−9.6−3
L1920 (m/z=763.602595; rt=9.59713)
T/S Cluster: L−9.6−3
L1929 (m/z=763.583046; rt=9.5974)
T/S Cluster: L−9.6−3
L1919 (m/z=253.867624; rt=9.59705)
T/S Cluster: L−9.6−3
L1930 (m/z=253.527468; rt=9.59754)
T/S Cluster: L−9.6−3
PCo1
PCo2

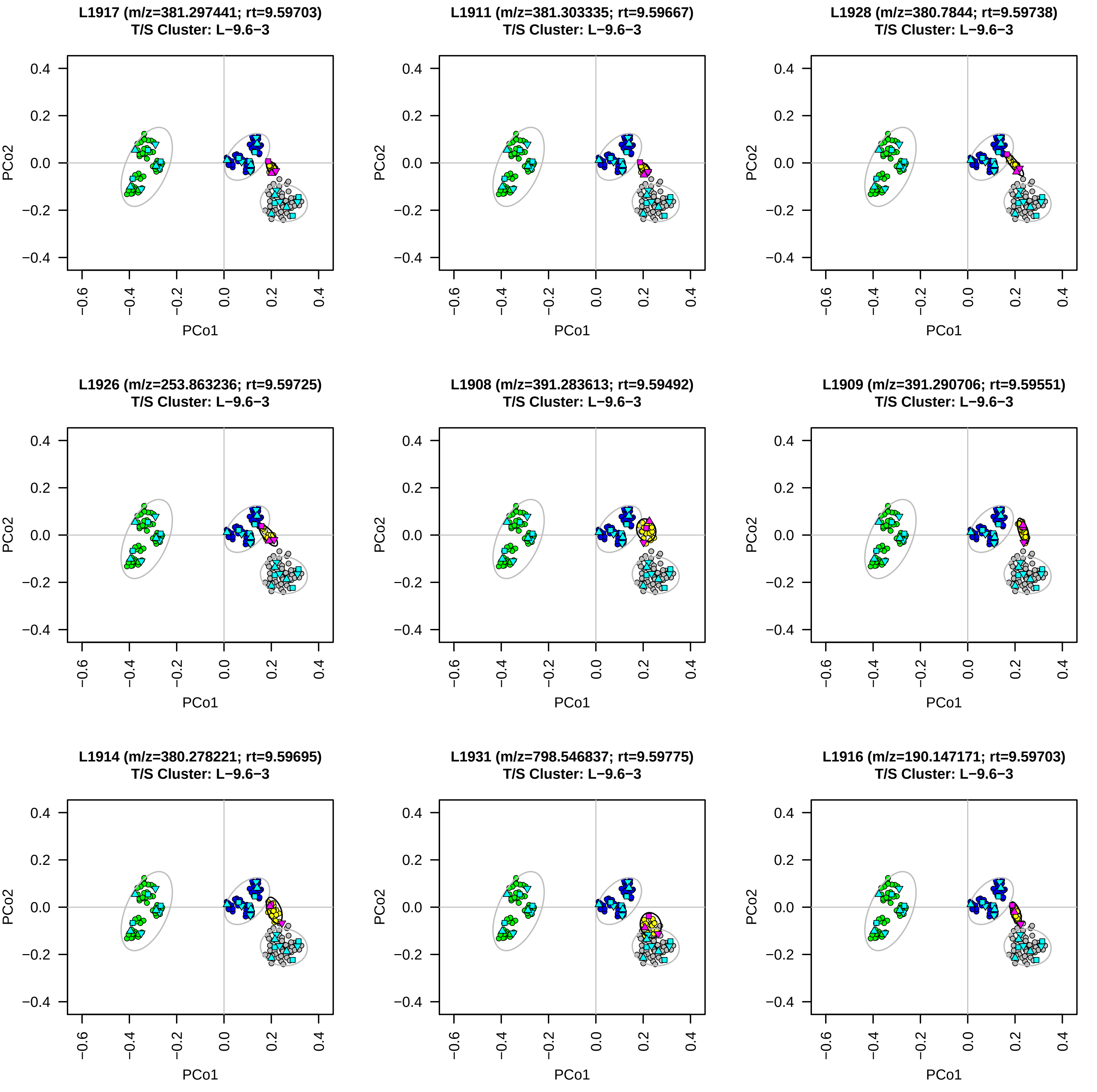

L1917 (m/z=381.297441; rt=9.59703)
T/S Cluster: L−9.6−3
L1911 (m/z=381.303335; rt=9.59667)
T/S Cluster: L−9.6−3
L1928 (m/z=380.7844; rt=9.59738)
T/S Cluster: L−9.6−3
L1926 (m/z=253.863236; rt=9.59725)
T/S Cluster: L−9.6−3
L1908 (m/z=391.283613; rt=9.59492)
T/S Cluster: L−9.6−3
L1909 (m/z=391.290706; rt=9.59551)
T/S Cluster: L−9.6−3
L1914 (m/z=380.278221; rt=9.59695)
T/S Cluster: L−9.6−3
L1931 (m/z=798.546837; rt=9.59775)
T/S Cluster: L−9.6−3
L1916 (m/z=190.147171; rt=9.59703)
T/S Cluster: L−9.6−3
PCo1
PCo2

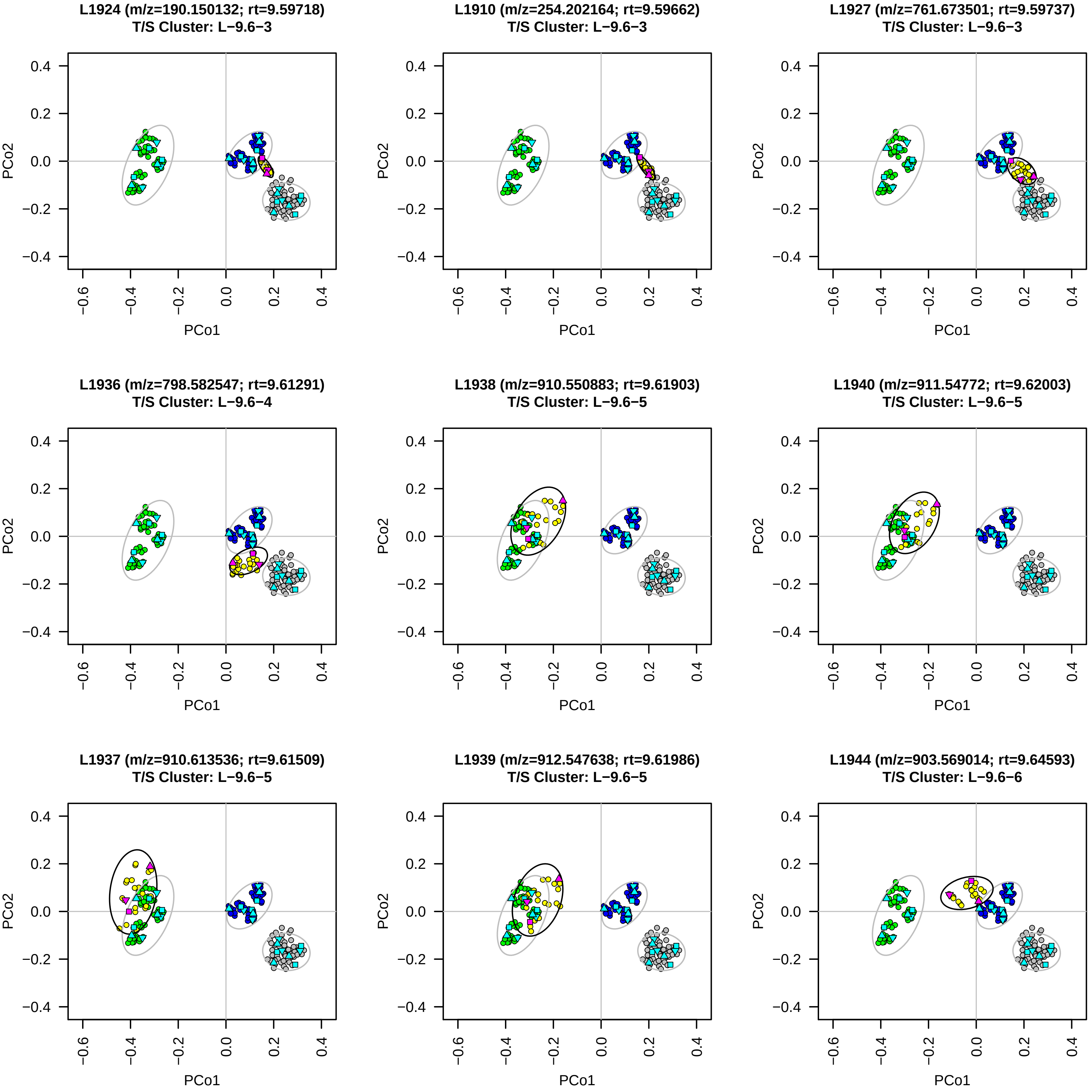
L1924 (m/z=190.150132; rt=9.59718)
T/S Cluster: L−9.6−3
L1910 (m/z=254.202164; rt=9.59662)
T/S Cluster: L−9.6−3
L1927 (m/z=761.673501; rt=9.59737)
T/S Cluster: L−9.6−3
L1936 (m/z=798.582547; rt=9.61291)
T/S Cluster: L−9.6−4
L1938 (m/z=910.550883; rt=9.61903)
T/S Cluster: L−9.6−5
L1940 (m/z=911.54772; rt=9.62003)
T/S Cluster: L−9.6−5
L1937 (m/z=910.613536; rt=9.61509)
T/S Cluster: L−9.6−5
L1939 (m/z=912.547638; rt=9.61986)
T/S Cluster: L−9.6−5
L1944 (m/z=903.569014; rt=9.64593)
T/S Cluster: L−9.6−6
PCo1
PCo2
−0.6
−0.4
−0.2
0.0
0.2
0.4

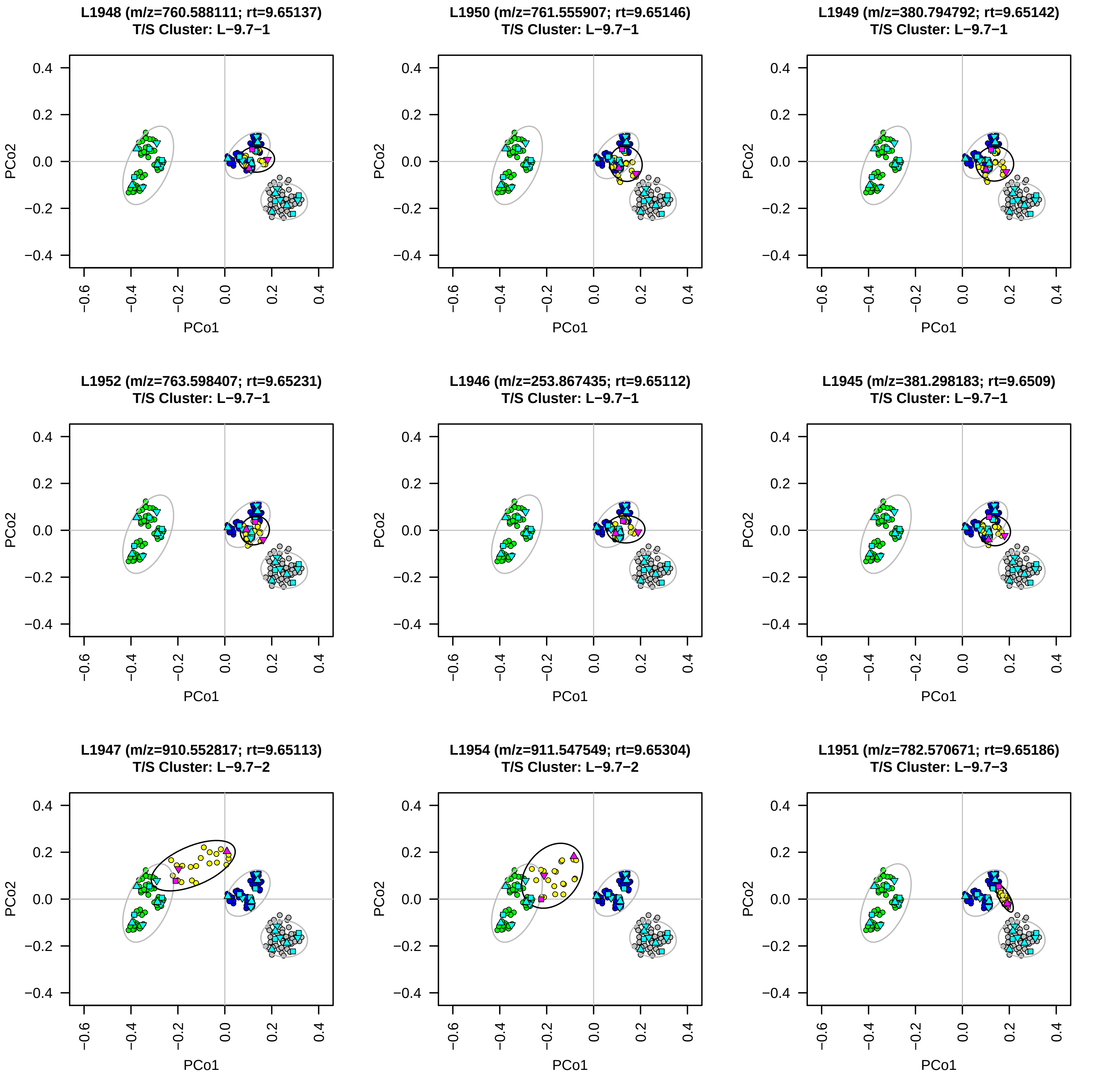
L1948 (m/z=760.588111; rt=9.65137)
T/S Cluster: L−9.7−1
L1950 (m/z=761.555907; rt=9.65146)
T/S Cluster: L−9.7−1
L1949 (m/z=380.794792; rt=9.65142)
T/S Cluster: L−9.7−1
L1952 (m/z=763.598407; rt=9.65231)
T/S Cluster: L−9.7−1
L1946 (m/z=253.867435; rt=9.65112)
T/S Cluster: L−9.7−1
L1945 (m/z=381.298183; rt=9.6509)
T/S Cluster: L−9.7−1
L1947 (m/z=910.552817; rt=9.65113)
T/S Cluster: L−9.7−2
L1954 (m/z=911.547549; rt=9.65304)
T/S Cluster: L−9.7−2
L1951 (m/z=782.570671; rt=9.65186)
T/S Cluster: L−9.7−3
PCo1
PCo2
0.4
0.2
0.0
−0.2
−0.4
−0.6

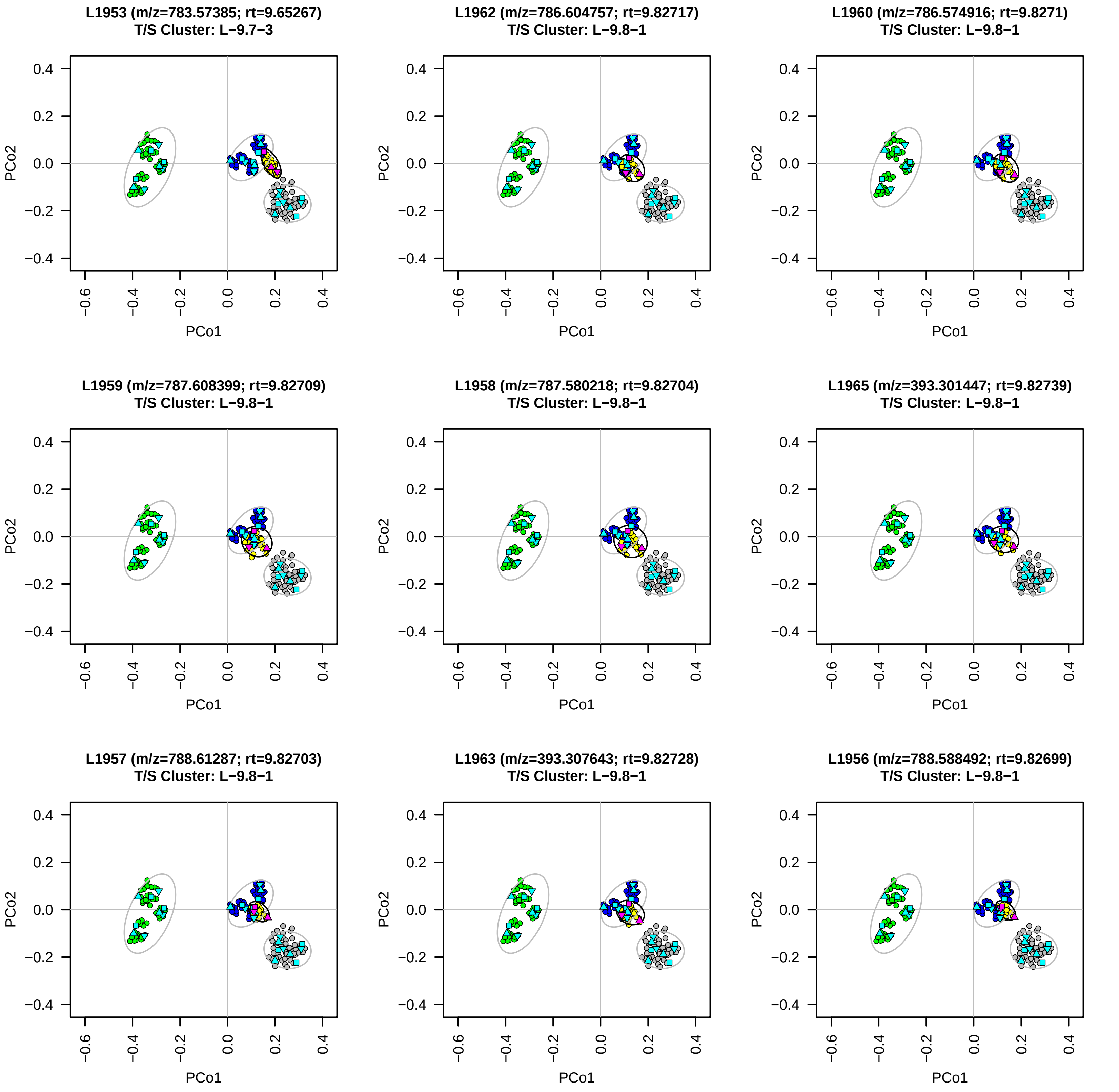
L1953 (m/z=783.57385; rt=9.65267)
T/S Cluster: L−9.7−3
L1962 (m/z=786.604757; rt=9.82717)
T/S Cluster: L−9.8−1
L1960 (m/z=786.574916; rt=9.8271)
T/S Cluster: L−9.8−1
L1959 (m/z=787.608399; rt=9.82709)
T/S Cluster: L−9.8−1
L1958 (m/z=787.580218; rt=9.82704)
T/S Cluster: L−9.8−1
L1965 (m/z=393.301447; rt=9.82739)
T/S Cluster: L−9.8−1
L1957 (m/z=788.61287; rt=9.82703)
T/S Cluster: L−9.8−1
L1963 (m/z=393.307643; rt=9.82728)
T/S Cluster: L−9.8−1
L1956 (m/z=788.588492; rt=9.82699)
T/S Cluster: L−9.8−1
PCo1
PCo2

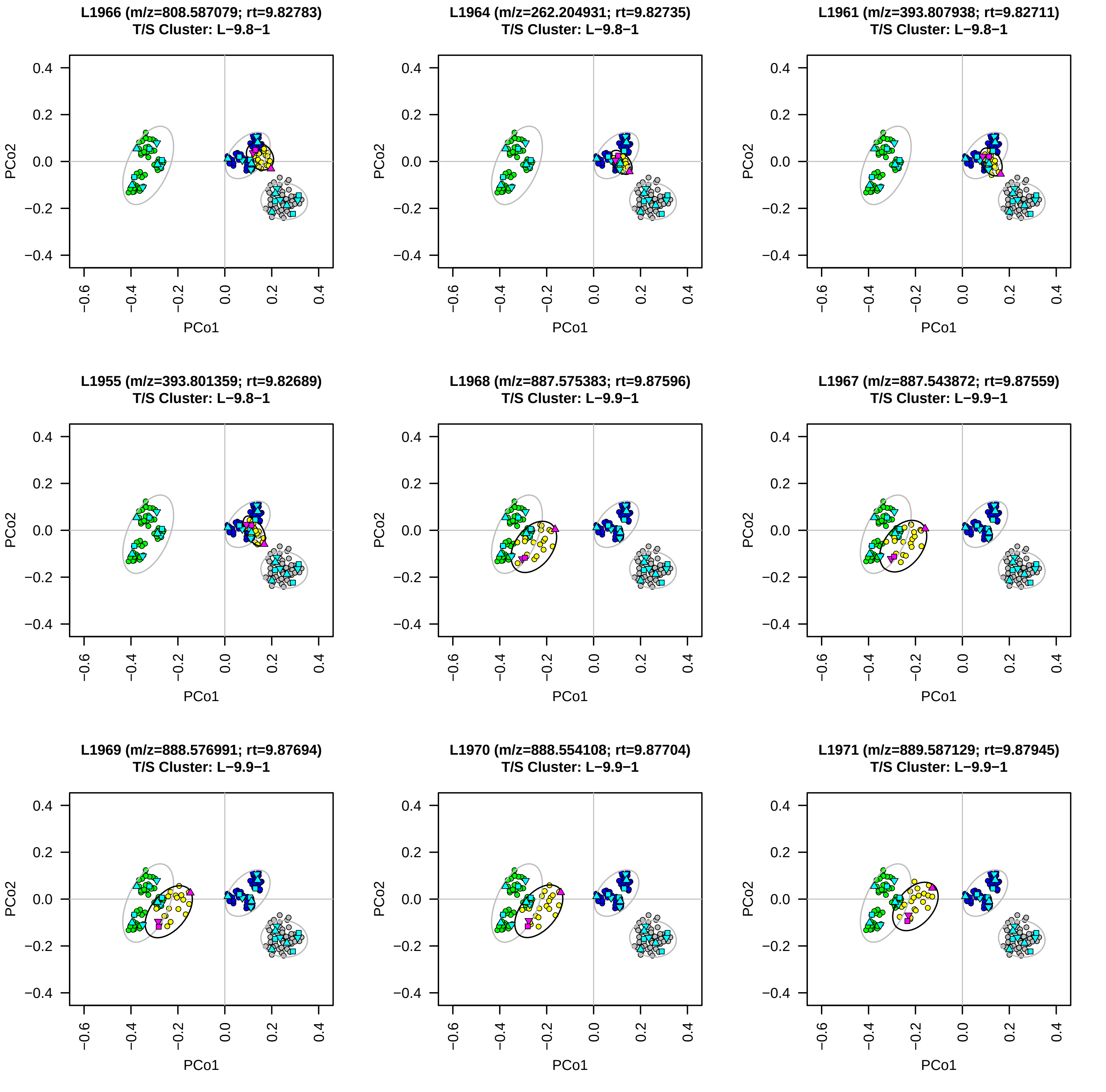
L1966 (m/z=808.587079; rt=9.82783)
T/S Cluster: L−9.8−1
L1964 (m/z=262.204931; rt=9.82735)
T/S Cluster: L−9.8−1
L1961 (m/z=393.807938; rt=9.82711)
T/S Cluster: L−9.8−1
L1955 (m/z=393.801359; rt=9.82689)
T/S Cluster: L−9.8−1
L1968 (m/z=887.575383; rt=9.87596)
T/S Cluster: L−9.9−1
L1967 (m/z=887.543872; rt=9.87559)
T/S Cluster: L−9.9−1
L1969 (m/z=888.576991; rt=9.87694)
T/S Cluster: L−9.9−1
L1970 (m/z=888.554108; rt=9.87704)
T/S Cluster: L−9.9−1
L1971 (m/z=889.587129; rt=9.87945)
T/S Cluster: L−9.9−1
PCo1
PCo2
−0.6
−0.4
−0.2
0.0
0.2
0.4

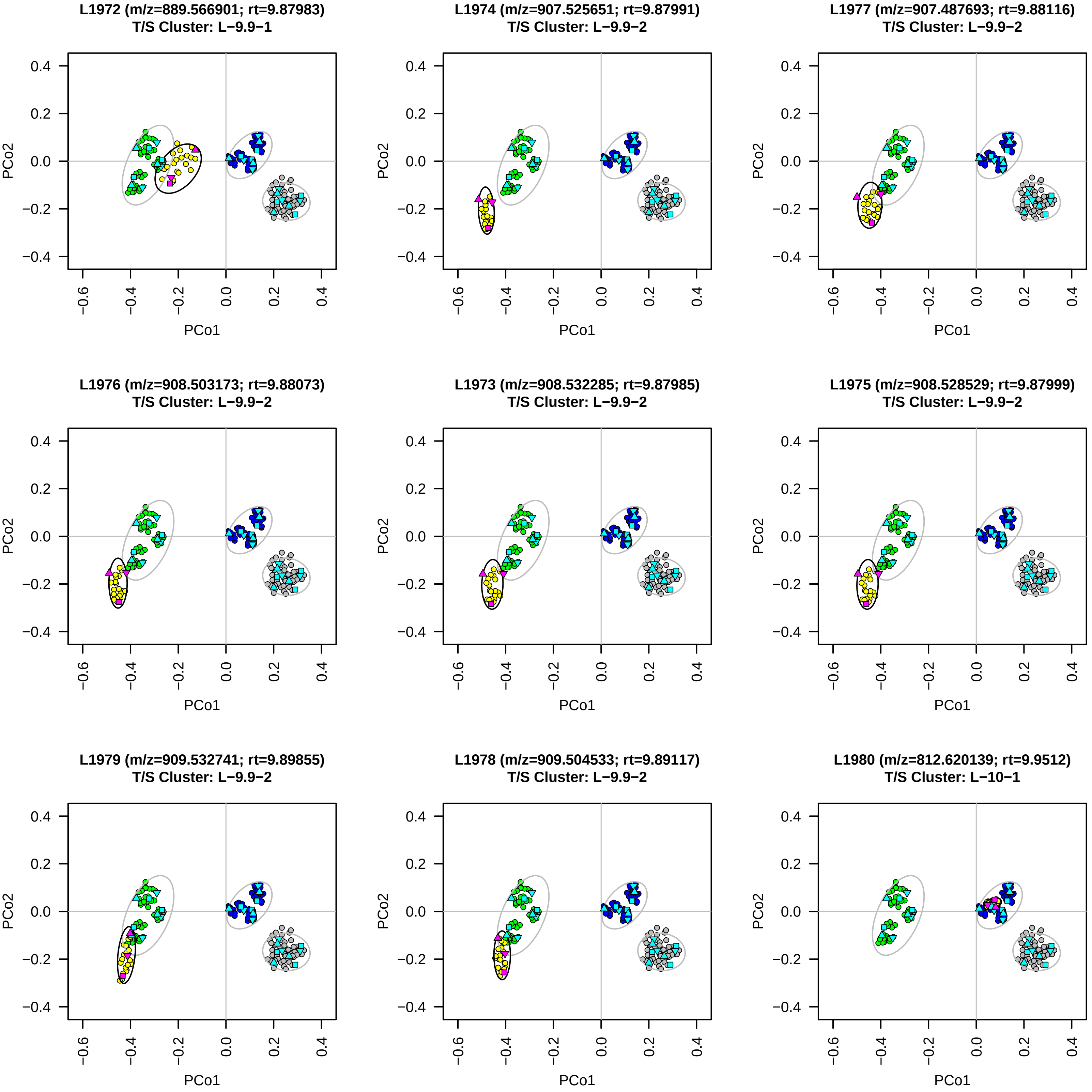
L1972 (m/z=889.566901; rt=9.87983)
T/S Cluster: L−9.9−1
L1974 (m/z=907.525651; rt=9.87991)
T/S Cluster: L−9.9−2
L1977 (m/z=907.487693; rt=9.88116)
T/S Cluster: L−9.9−2
L1976 (m/z=908.503173; rt=9.88073)
T/S Cluster: L−9.9−2
L1973 (m/z=908.532285; rt=9.87985)
T/S Cluster: L−9.9−2
L1975 (m/z=908.528529; rt=9.87999)
T/S Cluster: L−9.9−2
L1979 (m/z=909.532741; rt=9.89855)
T/S Cluster: L−9.9−2
L1978 (m/z=909.504533; rt=9.89117)
T/S Cluster: L−9.9−2
L1980 (m/z=812.620139; rt=9.9512)
T/S Cluster: L−10−1
PCo1
PCo2
0.4
0.2
0.0
−0.2
−0.4
−0.6

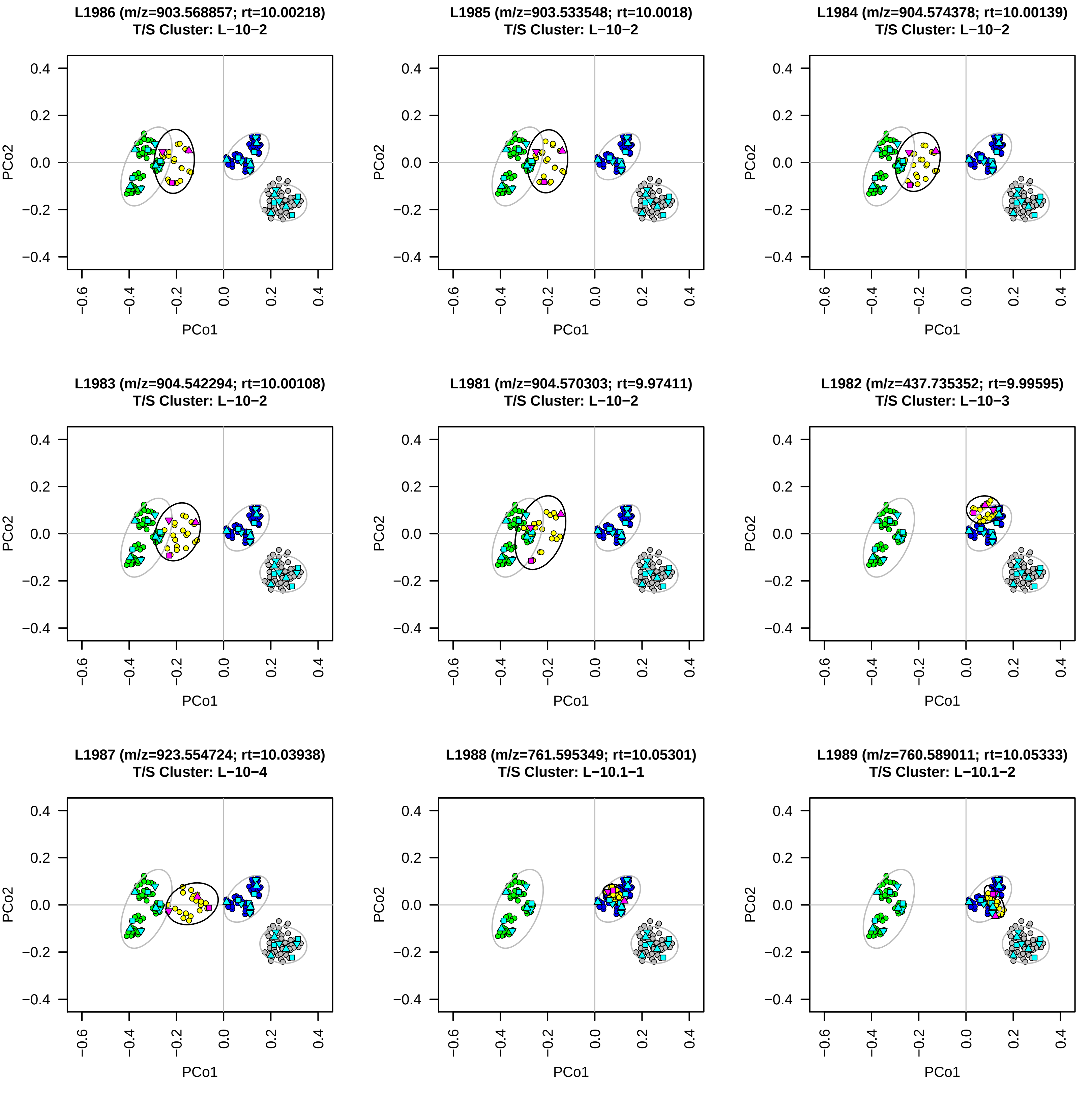
L1986 (m/z=903.568857; rt=10.00218)
T/S Cluster: L−10−2
L1985 (m/z=903.533548; rt=10.0018)
T/S Cluster: L−10−2
L1984 (m/z=904.574378; rt=10.00139)
T/S Cluster: L−10−2
L1983 (m/z=904.542294; rt=10.00108)
T/S Cluster: L−10−2
L1981 (m/z=904.570303; rt=9.97411)
T/S Cluster: L−10−2
L1982 (m/z=437.735352; rt=9.99595)
T/S Cluster: L−10−3
L1987 (m/z=923.554724; rt=10.03938)
T/S Cluster: L−10−4
L1988 (m/z=761.595349; rt=10.05301)
T/S Cluster: L−10.1−1
L1989 (m/z=760.589011; rt=10.05333)
T/S Cluster: L−10.1−2
PCo1
PCo2
0.4
0.2
0.0
−0.2
−0.4
−0.6

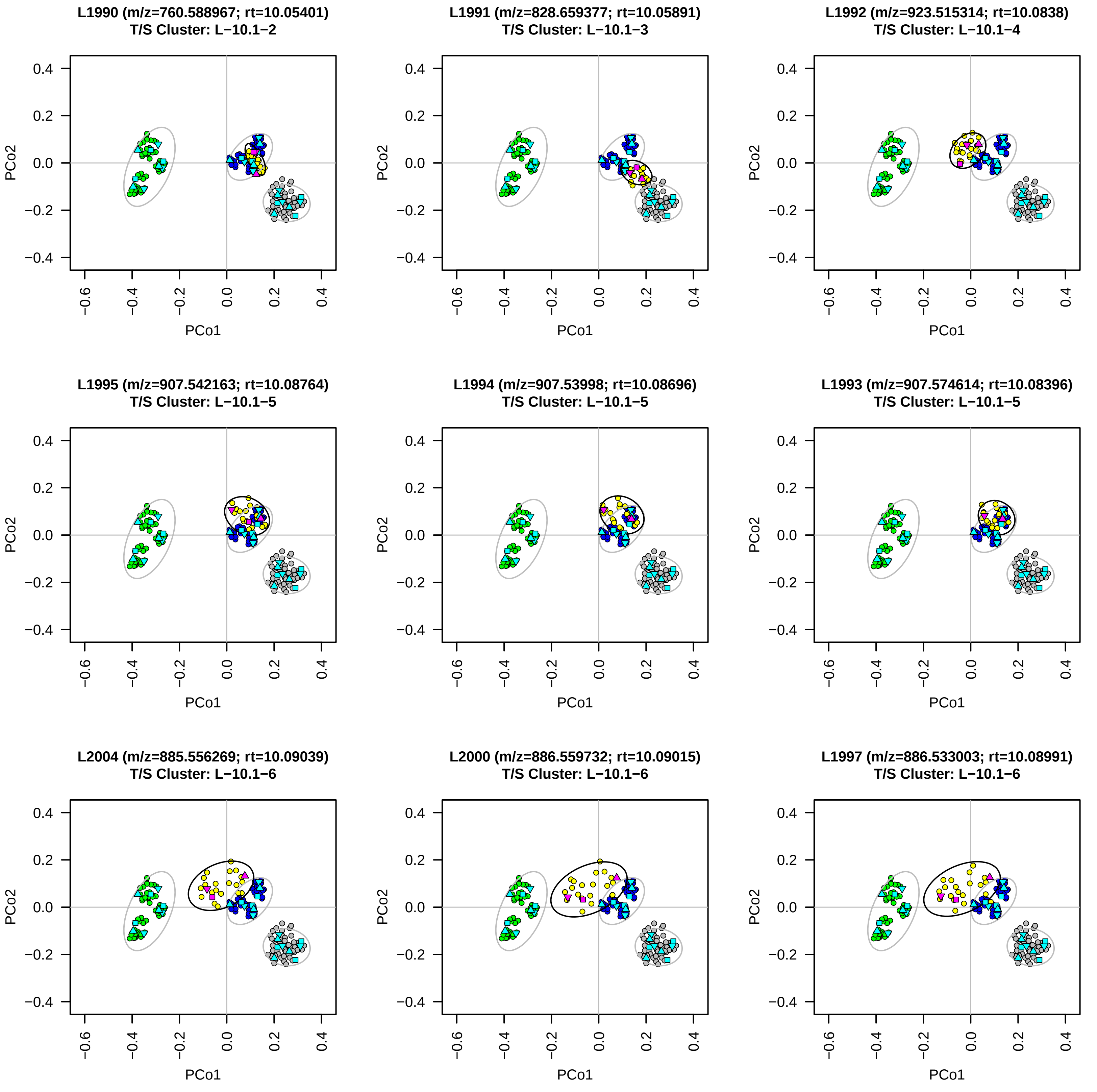
L1990 (m/z=760.588967; rt=10.05401)
T/S Cluster: L−10.1−2
L1991 (m/z=828.659377; rt=10.05891)
T/S Cluster: L−10.1−3
L1992 (m/z=923.515314; rt=10.0838)
T/S Cluster: L−10.1−4
L1995 (m/z=907.542163; rt=10.08764)
T/S Cluster: L−10.1−5
L1994 (m/z=907.53998; rt=10.08696)
T/S Cluster: L−10.1−5
L1993 (m/z=907.574614; rt=10.08396)
T/S Cluster: L−10.1−5
L2004 (m/z=885.556269; rt=10.09039)
T/S Cluster: L−10.1−6
L2000 (m/z=886.559732; rt=10.09015)
T/S Cluster: L−10.1−6
L1997 (m/z=886.533003; rt=10.08991)
T/S Cluster: L−10.1−6
PCo1
PCo2
0.4
0.2
0.0
−0.2
−0.4
−0.6

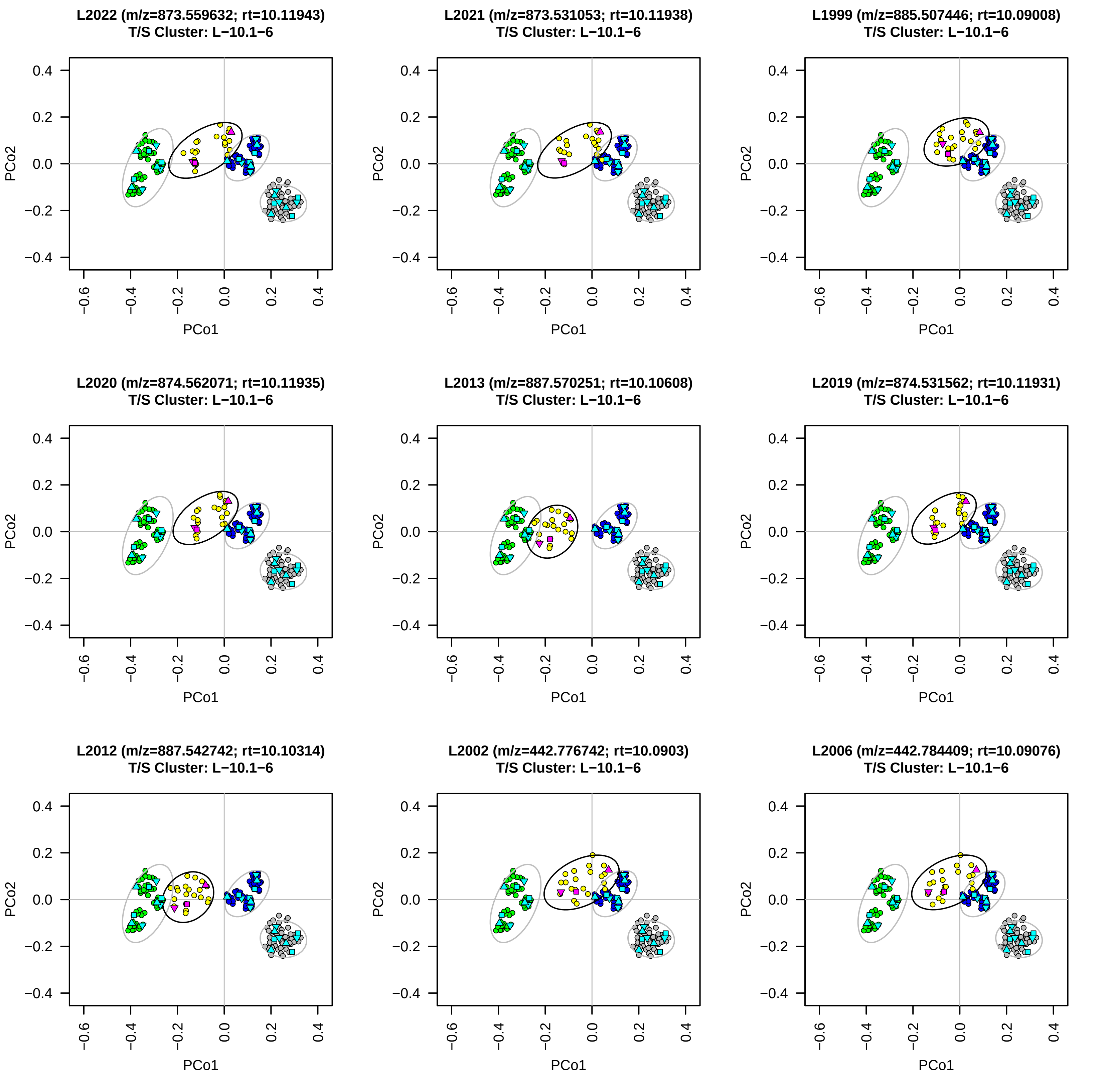
L2022 (m/z=873.559632; rt=10.11943)
T/S Cluster: L−10.1−6
L2021 (m/z=873.531053; rt=10.11938)
T/S Cluster: L−10.1−6
L1999 (m/z=885.507446; rt=10.09008)
T/S Cluster: L−10.1−6
L2020 (m/z=874.562071; rt=10.11935)
T/S Cluster: L−10.1−6
L2013 (m/z=887.570251; rt=10.10608)
T/S Cluster: L−10.1−6
L2019 (m/z=874.531562; rt=10.11931)
T/S Cluster: L−10.1−6
L2012 (m/z=887.542742; rt=10.10314)
T/S Cluster: L−10.1−6
L2002 (m/z=442.776742; rt=10.0903)
T/S Cluster: L−10.1−6
L2006 (m/z=442.784409; rt=10.09076)
T/S Cluster: L−10.1−6
PCo1
PCo2
−0.6
−0.4
−0.2
0.0
0.2
0.4

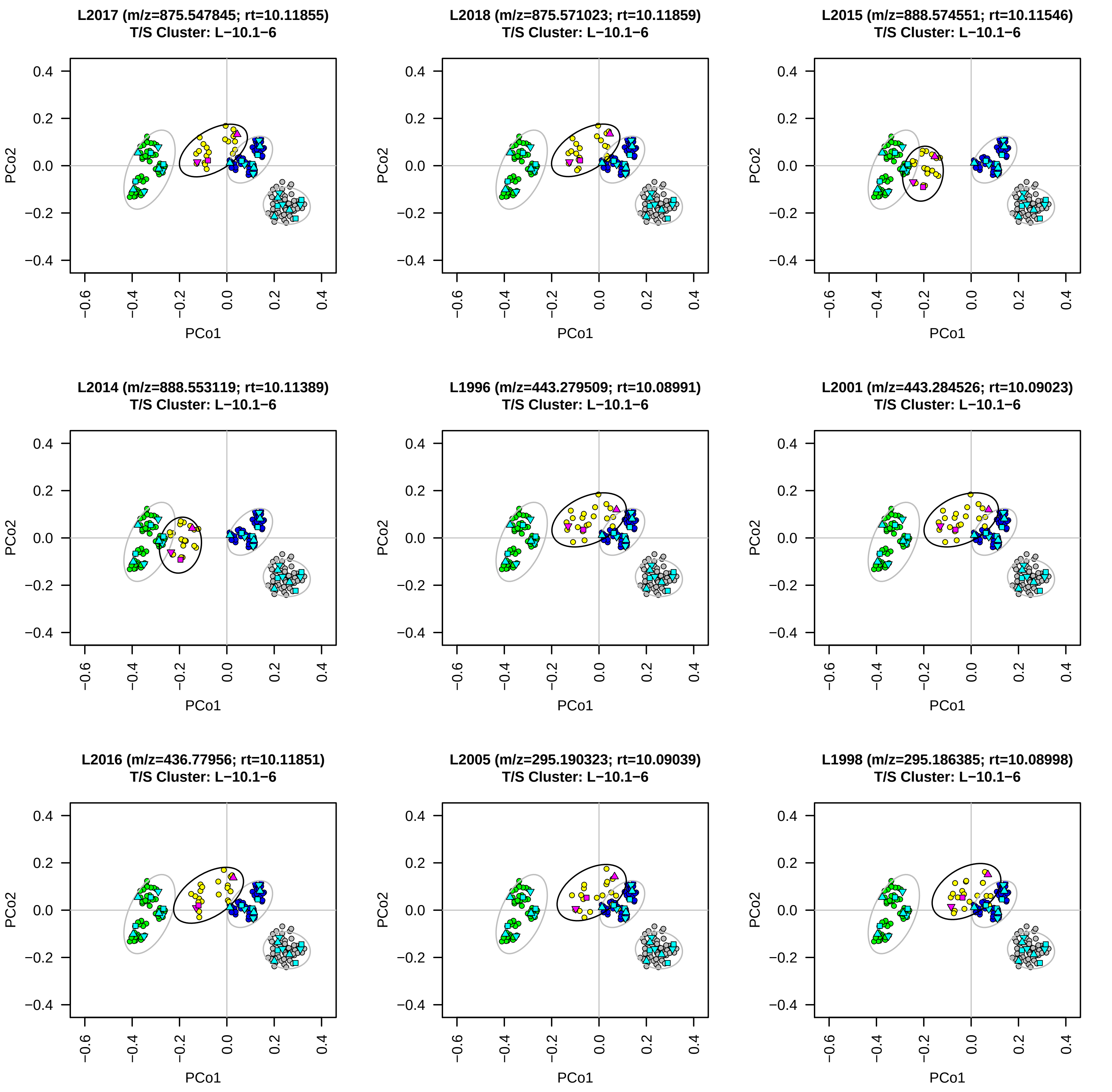
L2017 (m/z=875.547845; rt=10.11855)
T/S Cluster: L−10.1−6
L2018 (m/z=875.571023; rt=10.11859)
T/S Cluster: L−10.1−6
L2015 (m/z=888.574551; rt=10.11546)
T/S Cluster: L−10.1−6
L2014 (m/z=888.553119; rt=10.11389)
T/S Cluster: L−10.1−6
L1996 (m/z=443.279509; rt=10.08991)
T/S Cluster: L−10.1−6
L2001 (m/z=443.284526; rt=10.09023)
T/S Cluster: L−10.1−6
L2016 (m/z=436.77956; rt=10.11851)
T/S Cluster: L−10.1−6
L2005 (m/z=295.190323; rt=10.09039)
T/S Cluster: L−10.1−6
L1998 (m/z=295.186385; rt=10.08998)
T/S Cluster: L−10.1−6
PCo1
PCo2
−0.6
−0.4
−0.2
0.0
0.2
0.4

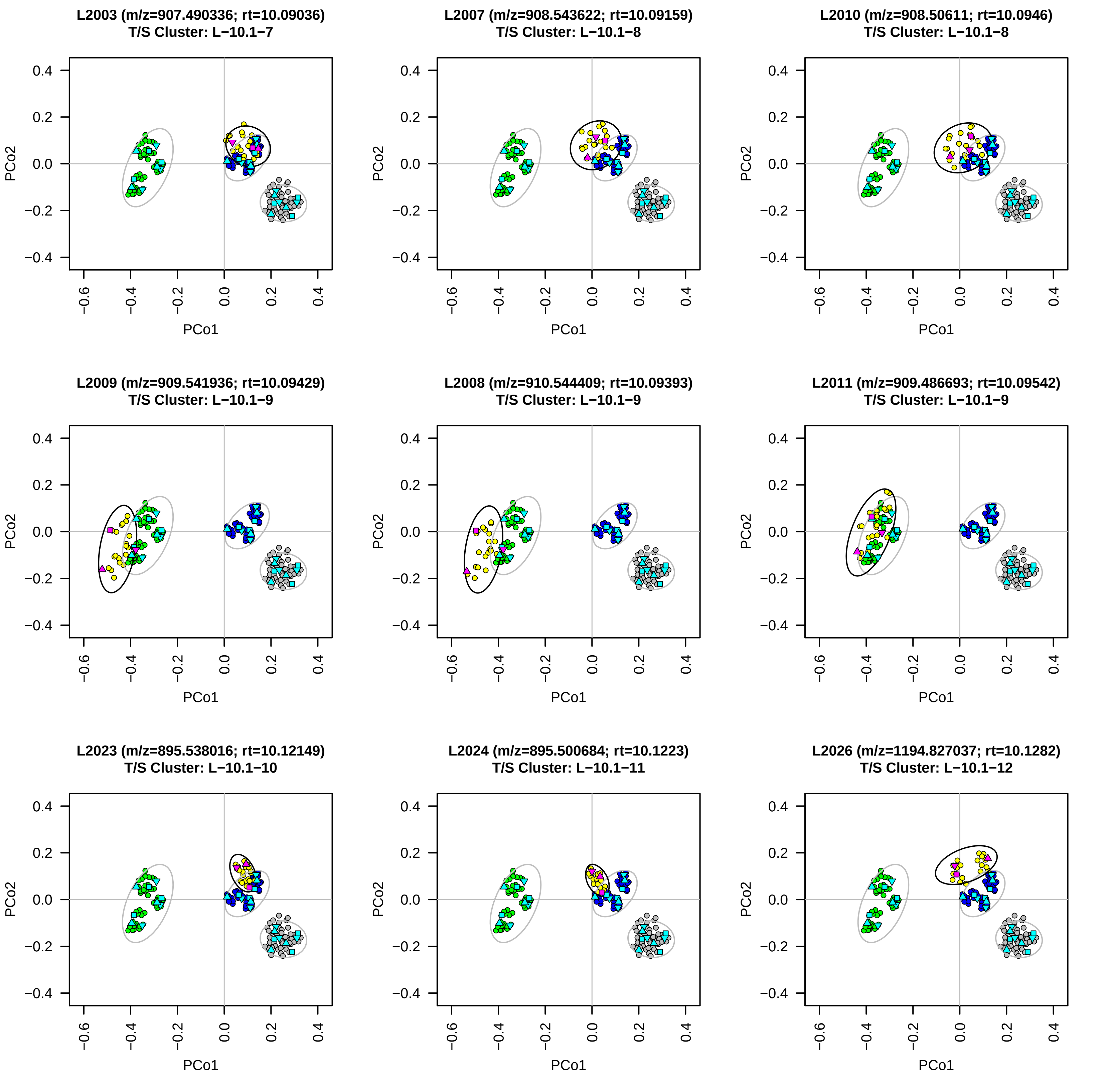

L2003 (m/z=907.490336; rt=10.09036)
T/S Cluster: L−10.1−7
L2007 (m/z=908.543622; rt=10.09159)
T/S Cluster: L−10.1−8
L2010 (m/z=908.50611; rt=10.0946)
T/S Cluster: L−10.1−8
L2009 (m/z=909.541936; rt=10.09429)
T/S Cluster: L−10.1−9
L2008 (m/z=910.544409; rt=10.09393)
T/S Cluster: L−10.1−9
L2011 (m/z=909.486693; rt=10.09542)
T/S Cluster: L−10.1−9
L2023 (m/z=895.538016; rt=10.12149)
T/S Cluster: L−10.1−10
L2024 (m/z=895.500684; rt=10.1223)
T/S Cluster: L−10.1−11
L2026 (m/z=1194.827037; rt=10.1282)
T/S Cluster: L−10.1−12
PCo1
PCo2
0.4
0.2
0.0
−0.2
−0.4
−0.6

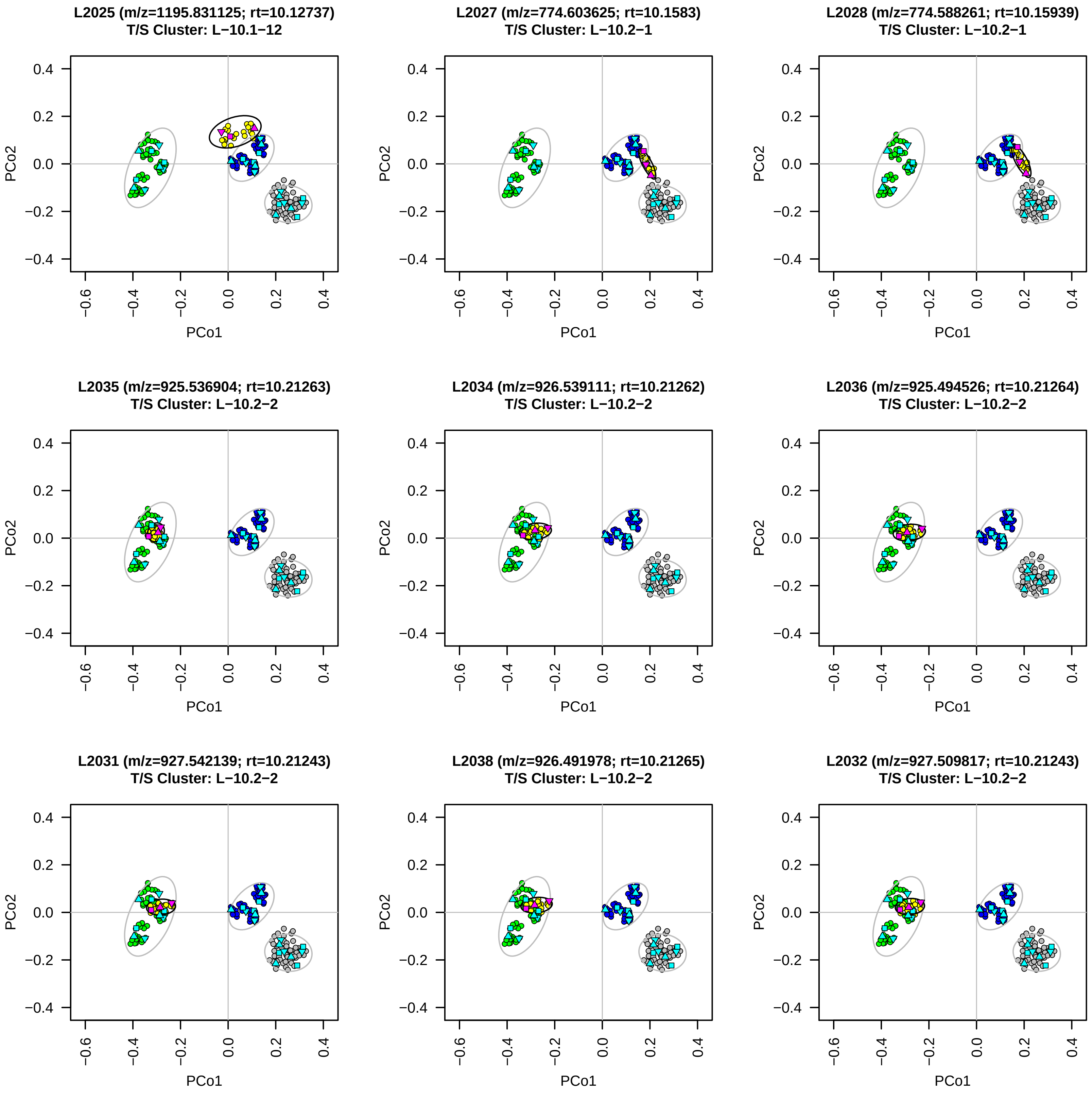
L2025 (m/z=1195.831125; rt=10.12737)
T/S Cluster: L−10.1−12
L2027 (m/z=774.603625; rt=10.1583)
T/S Cluster: L−10.2−1
L2028 (m/z=774.588261; rt=10.15939)
T/S Cluster: L−10.2−1
L2035 (m/z=925.536904; rt=10.21263)
T/S Cluster: L−10.2−2
L2034 (m/z=926.539111; rt=10.21262)
T/S Cluster: L−10.2−2
L2036 (m/z=925.494526; rt=10.21264)
T/S Cluster: L−10.2−2
L2031 (m/z=927.542139; rt=10.21243)
T/S Cluster: L−10.2−2
L2038 (m/z=926.491978; rt=10.21265)
T/S Cluster: L−10.2−2
L2032 (m/z=927.509817; rt=10.21243)
T/S Cluster: L−10.2−2
PCo1
PCo2
0.4
0.2
0.0
−0.2
−0.4
−0.6

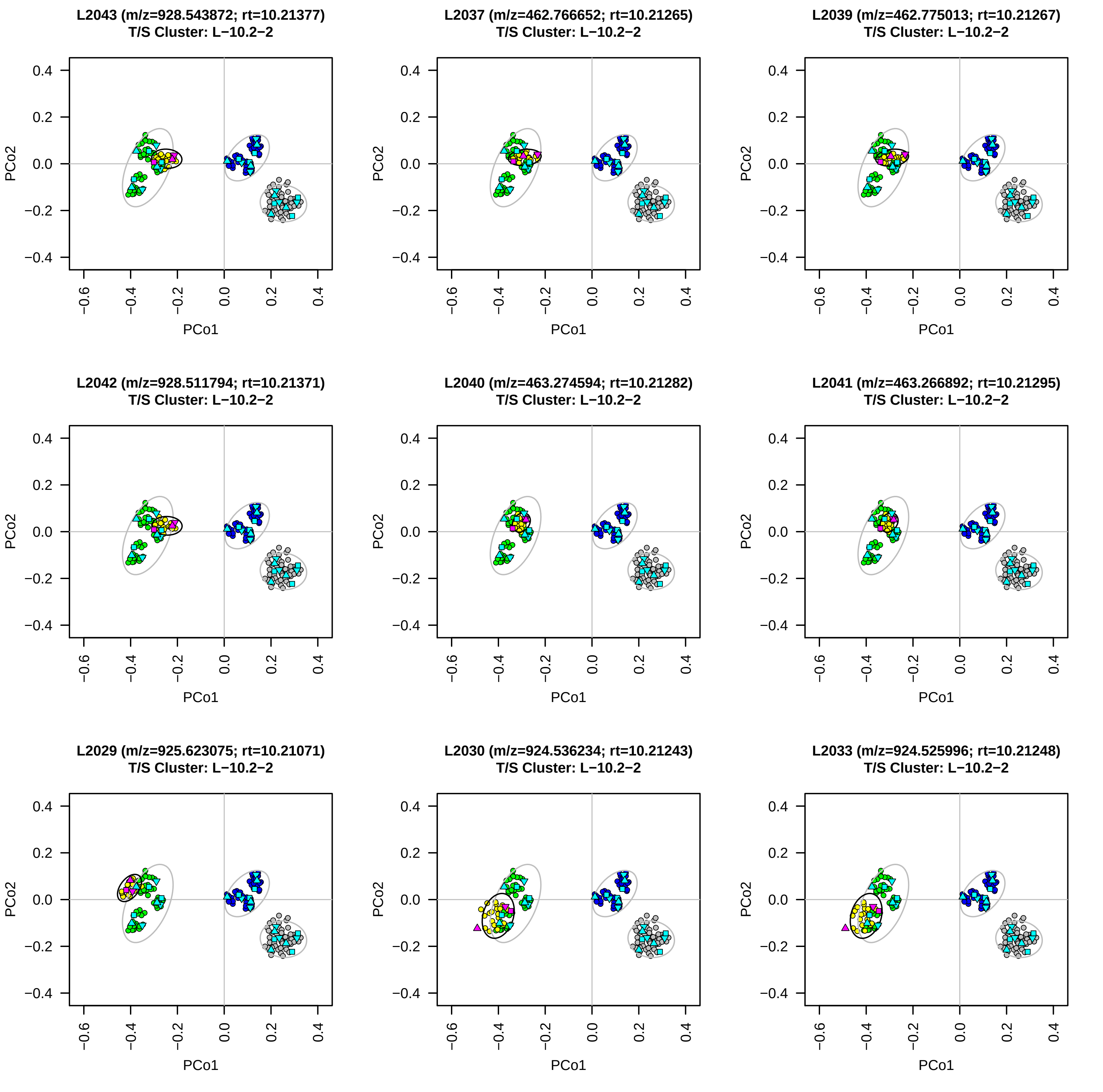
L2043 (m/z=928.543872; rt=10.21377)
T/S Cluster: L−10.2−2
L2037 (m/z=462.766652; rt=10.21265)
T/S Cluster: L−10.2−2
L2039 (m/z=462.775013; rt=10.21267)
T/S Cluster: L−10.2−2
L2042 (m/z=928.511794; rt=10.21371)
T/S Cluster: L−10.2−2
L2040 (m/z=463.274594; rt=10.21282)
T/S Cluster: L−10.2−2
L2041 (m/z=463.266892; rt=10.21295)
T/S Cluster: L−10.2−2
L2029 (m/z=925.623075; rt=10.21071)
T/S Cluster: L−10.2−2
L2030 (m/z=924.536234; rt=10.21243)
T/S Cluster: L−10.2−2
L2033 (m/z=924.525996; rt=10.21248)
T/S Cluster: L−10.2−2
PCo1
PCo2

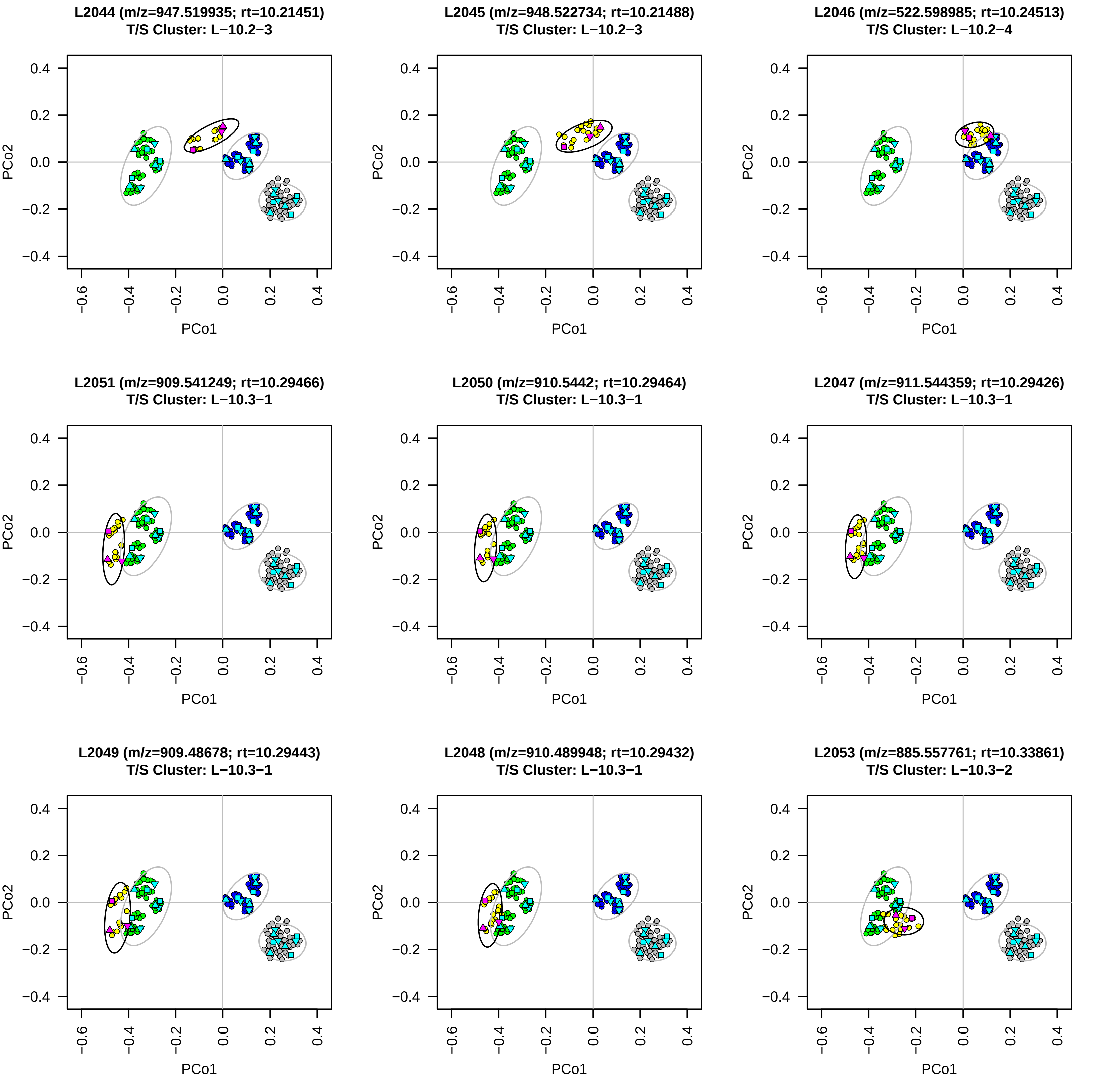
L2044 (m/z=947.519935; rt=10.21451)
T/S Cluster: L−10.2−3
L2045 (m/z=948.522734; rt=10.21488)
T/S Cluster: L−10.2−3
L2046 (m/z=522.598985; rt=10.24513)
T/S Cluster: L−10.2−4
L2051 (m/z=909.541249; rt=10.29466)
T/S Cluster: L−10.3−1
L2050 (m/z=910.5442; rt=10.29464)
T/S Cluster: L−10.3−1
L2047 (m/z=911.544359; rt=10.29426)
T/S Cluster: L−10.3−1
L2049 (m/z=909.48678; rt=10.29443)
T/S Cluster: L−10.3−1
L2048 (m/z=910.489948; rt=10.29432)
T/S Cluster: L−10.3−1
L2053 (m/z=885.557761; rt=10.33861)
T/S Cluster: L−10.3−2
PCo1
PCo2
−0.6
−0.4
−0.2
0.0
0.2
0.4

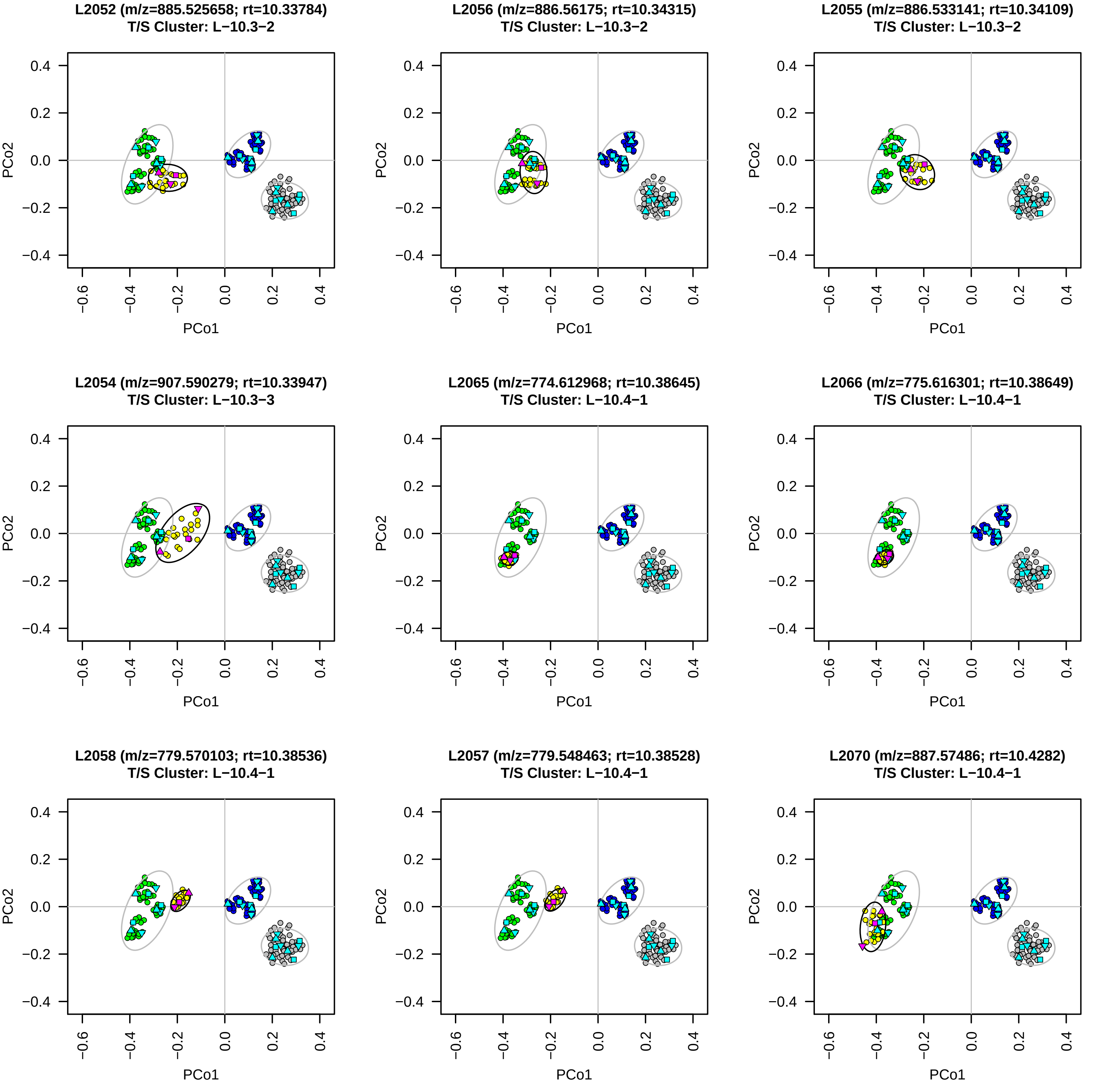
L2052 (m/z=885.525658; rt=10.33784)
T/S Cluster: L−10.3−2
L2056 (m/z=886.56175; rt=10.34315)
T/S Cluster: L−10.3−2
L2055 (m/z=886.533141; rt=10.34109)
T/S Cluster: L−10.3−2
L2054 (m/z=907.590279; rt=10.33947)
T/S Cluster: L−10.3−3
L2065 (m/z=774.612968; rt=10.38645)
T/S Cluster: L−10.4−1
L2066 (m/z=775.616301; rt=10.38649)
T/S Cluster: L−10.4−1
L2058 (m/z=779.570103; rt=10.38536)
T/S Cluster: L−10.4−1
L2057 (m/z=779.548463; rt=10.38528)
T/S Cluster: L−10.4−1
L2070 (m/z=887.57486; rt=10.4282)
T/S Cluster: L−10.4−1
PCo1
PCo2
−0.6
−0.4
−0.2
0.0
0.2
0.4

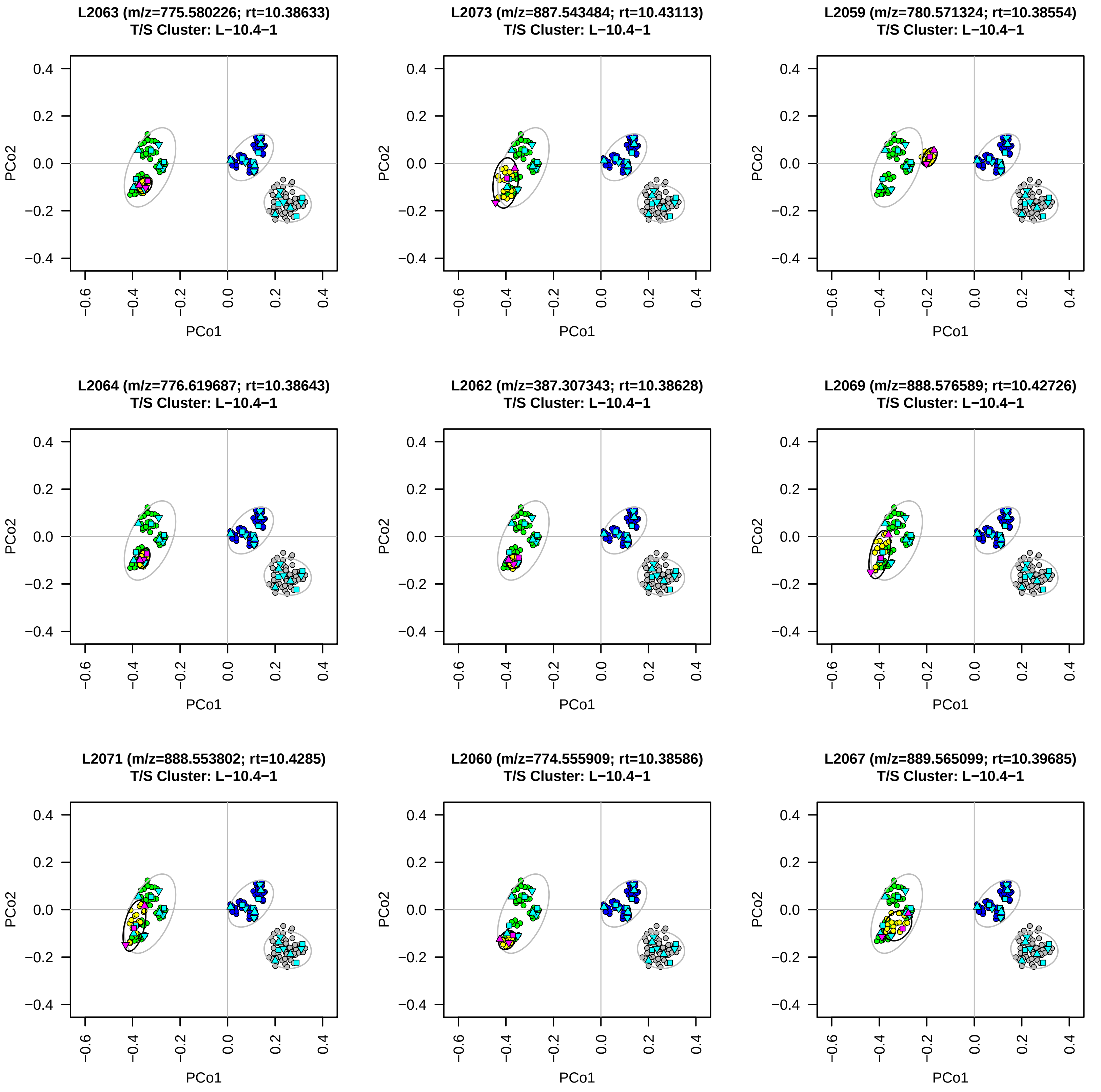

L2063 (m/z=775.580226; rt=10.38633)
T/S Cluster: L−10.4−1
L2073 (m/z=887.543484; rt=10.43113)
T/S Cluster: L−10.4−1
L2059 (m/z=780.571324; rt=10.38554)
T/S Cluster: L−10.4−1
L2064 (m/z=776.619687; rt=10.38643)
T/S Cluster: L−10.4−1
L2062 (m/z=387.307343; rt=10.38628)
T/S Cluster: L−10.4−1
L2069 (m/z=888.576589; rt=10.42726)
T/S Cluster: L−10.4−1
L2071 (m/z=888.553802; rt=10.4285)
T/S Cluster: L−10.4−1
L2060 (m/z=774.555909; rt=10.38586)
T/S Cluster: L−10.4−1
L2067 (m/z=889.565099; rt=10.39685)
T/S Cluster: L−10.4−1
PCo1
PCo2

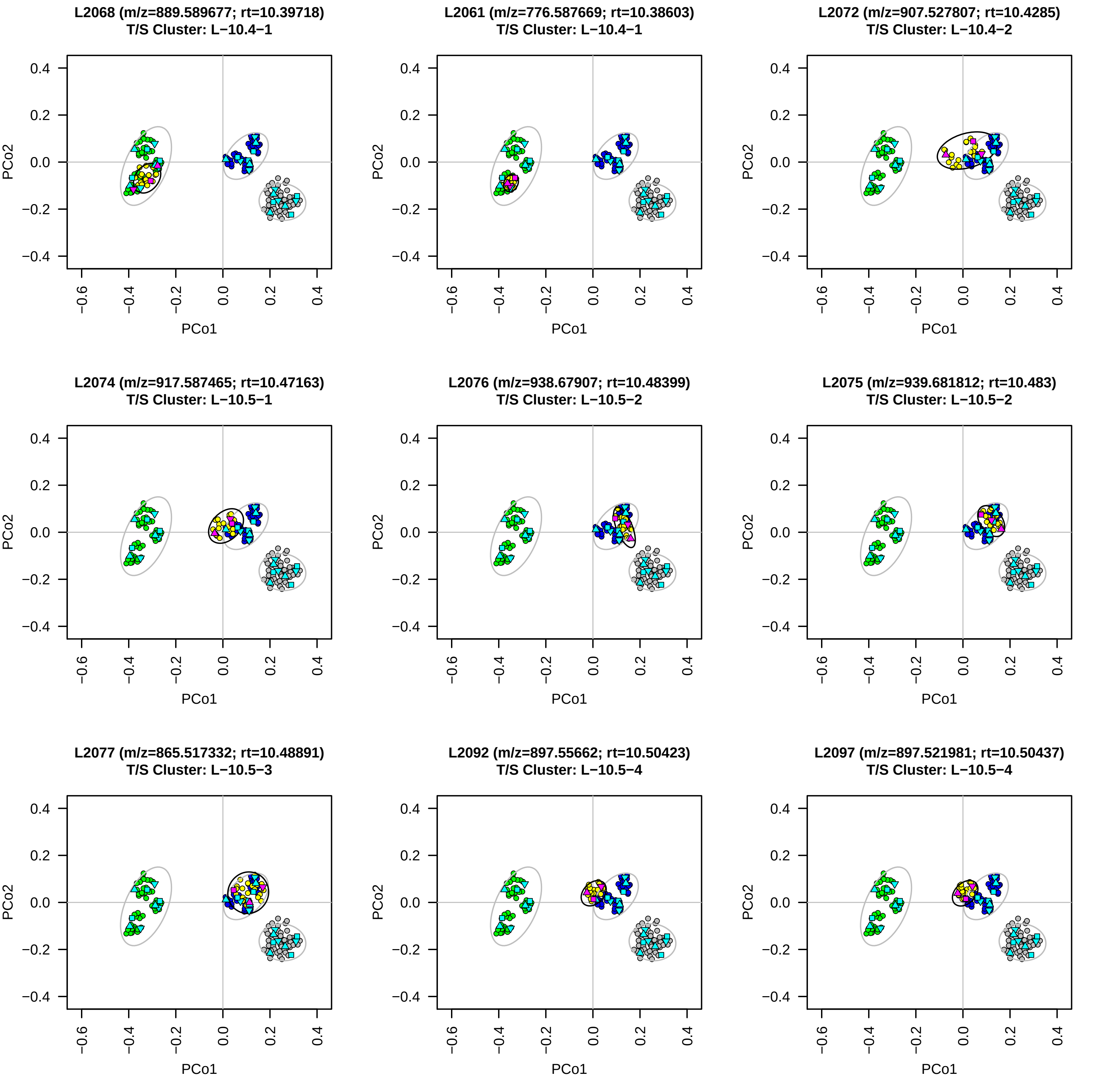
L2068 (m/z=889.589677; rt=10.39718)
T/S Cluster: L−10.4−1
L2061 (m/z=776.587669; rt=10.38603)
T/S Cluster: L−10.4−1
L2072 (m/z=907.527807; rt=10.4285)
T/S Cluster: L−10.4−2
L2074 (m/z=917.587465; rt=10.47163)
T/S Cluster: L−10.5−1
L2076 (m/z=938.67907; rt=10.48399)
T/S Cluster: L−10.5−2
L2075 (m/z=939.681812; rt=10.483)
T/S Cluster: L−10.5−2
L2077 (m/z=865.517332; rt=10.48891)
T/S Cluster: L−10.5−3
L2092 (m/z=897.55662; rt=10.50423)
T/S Cluster: L−10.5−4
L2097 (m/z=897.521981; rt=10.50437)
T/S Cluster: L−10.5−4
PCo1
PCo2
−0.6
−0.4
−0.2
0.0
0.2
0.4

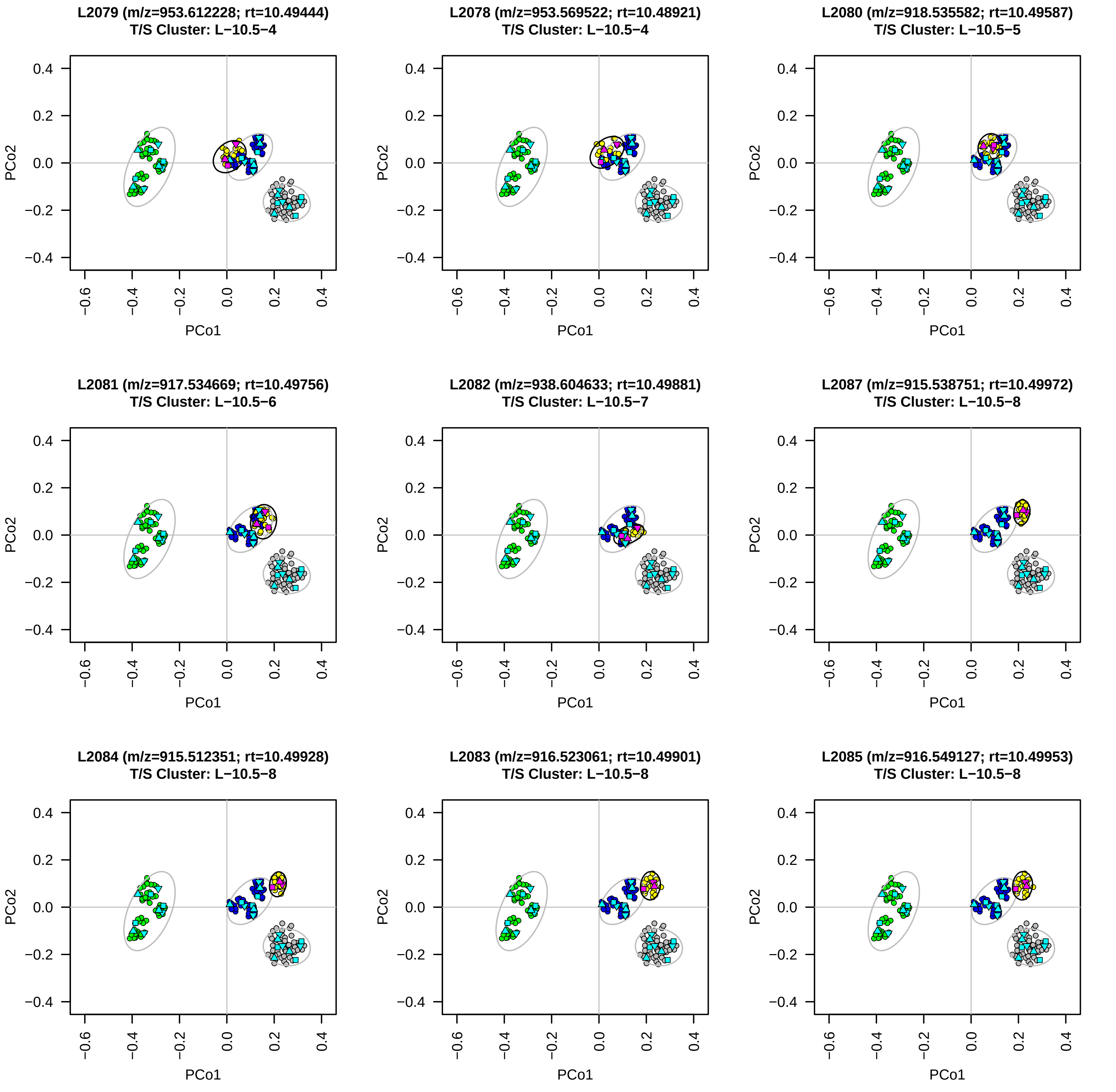
L2079 (m/z=953.612228; rt=10.49444)
T/S Cluster: L−10.5−4
L2078 (m/z=953.569522; rt=10.48921)
T/S Cluster: L−10.5−4
L2080 (m/z=918.535582; rt=10.49587)
T/S Cluster: L−10.5−5
L2081 (m/z=917.534669; rt=10.49756)
T/S Cluster: L−10.5−6
L2082 (m/z=938.604633; rt=10.49881)
T/S Cluster: L−10.5−7
L2087 (m/z=915.538751; rt=10.49972)
T/S Cluster: L−10.5−8
L2084 (m/z=915.512351; rt=10.49928)
T/S Cluster: L−10.5−8
L2083 (m/z=916.523061; rt=10.49901)
T/S Cluster: L−10.5−8
L2085 (m/z=916.549127; rt=10.49953)
T/S Cluster: L−10.5−8
PCo1
PCo2
−0.6
−0.4
−0.2
0.0
0.2
0.4

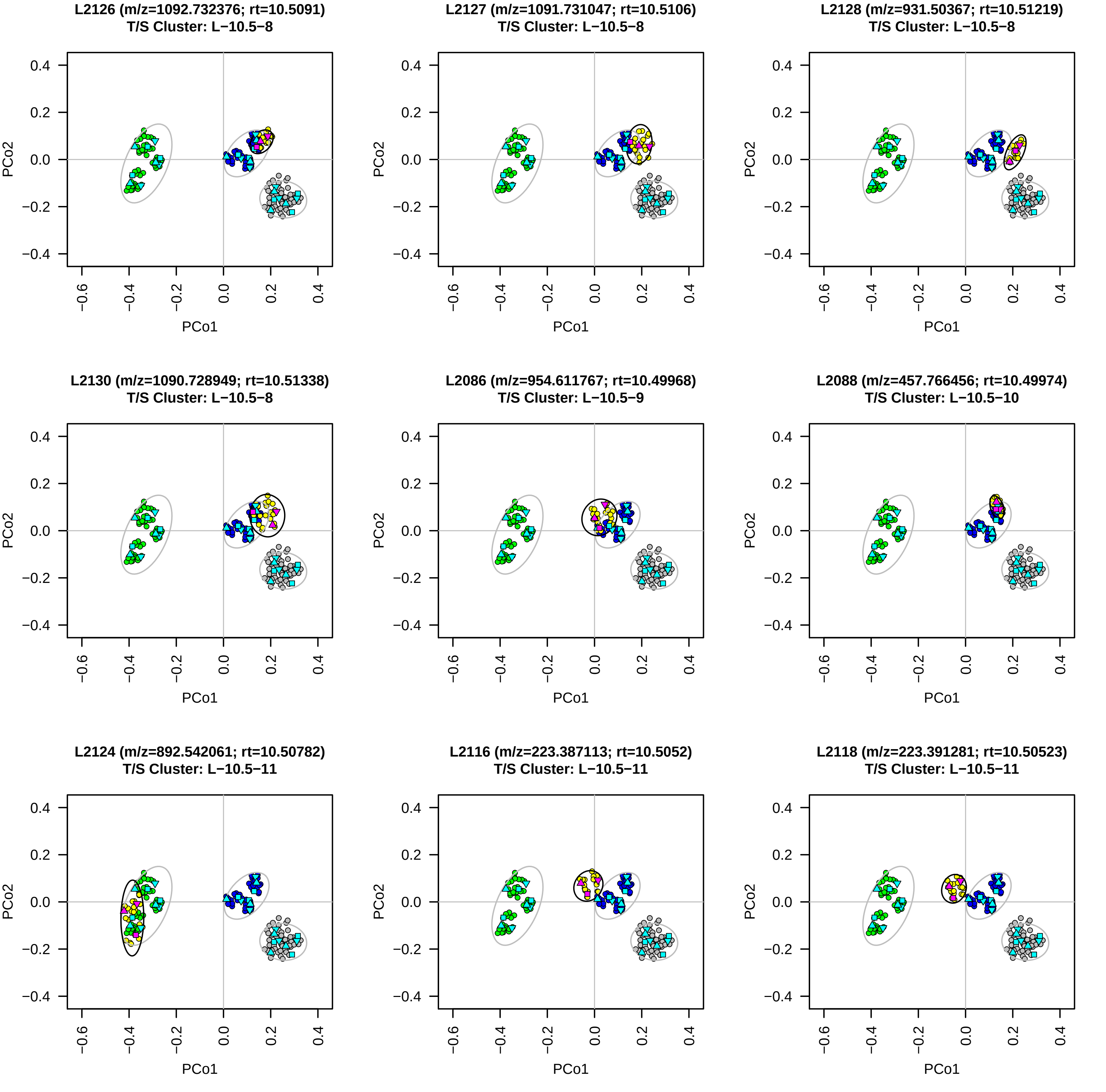
L2126 (m/z=1092.732376; rt=10.5091)
T/S Cluster: L−10.5−8
L2127 (m/z=1091.731047; rt=10.5106)
T/S Cluster: L−10.5−8
L2128 (m/z=931.50367; rt=10.51219)
T/S Cluster: L−10.5−8
L2130 (m/z=1090.728949; rt=10.51338)
T/S Cluster: L−10.5−8
L2086 (m/z=954.611767; rt=10.49968)
T/S Cluster: L−10.5−9
L2088 (m/z=457.766456; rt=10.49974)
T/S Cluster: L−10.5−10
L2124 (m/z=892.542061; rt=10.50782)
T/S Cluster: L−10.5−11
L2116 (m/z=223.387113; rt=10.5052)
T/S Cluster: L−10.5−11
L2118 (m/z=223.391281; rt=10.50523)
T/S Cluster: L−10.5−11
PCo1
PCo2
0.4
0.2
0.0
−0.2
−0.4
−0.6

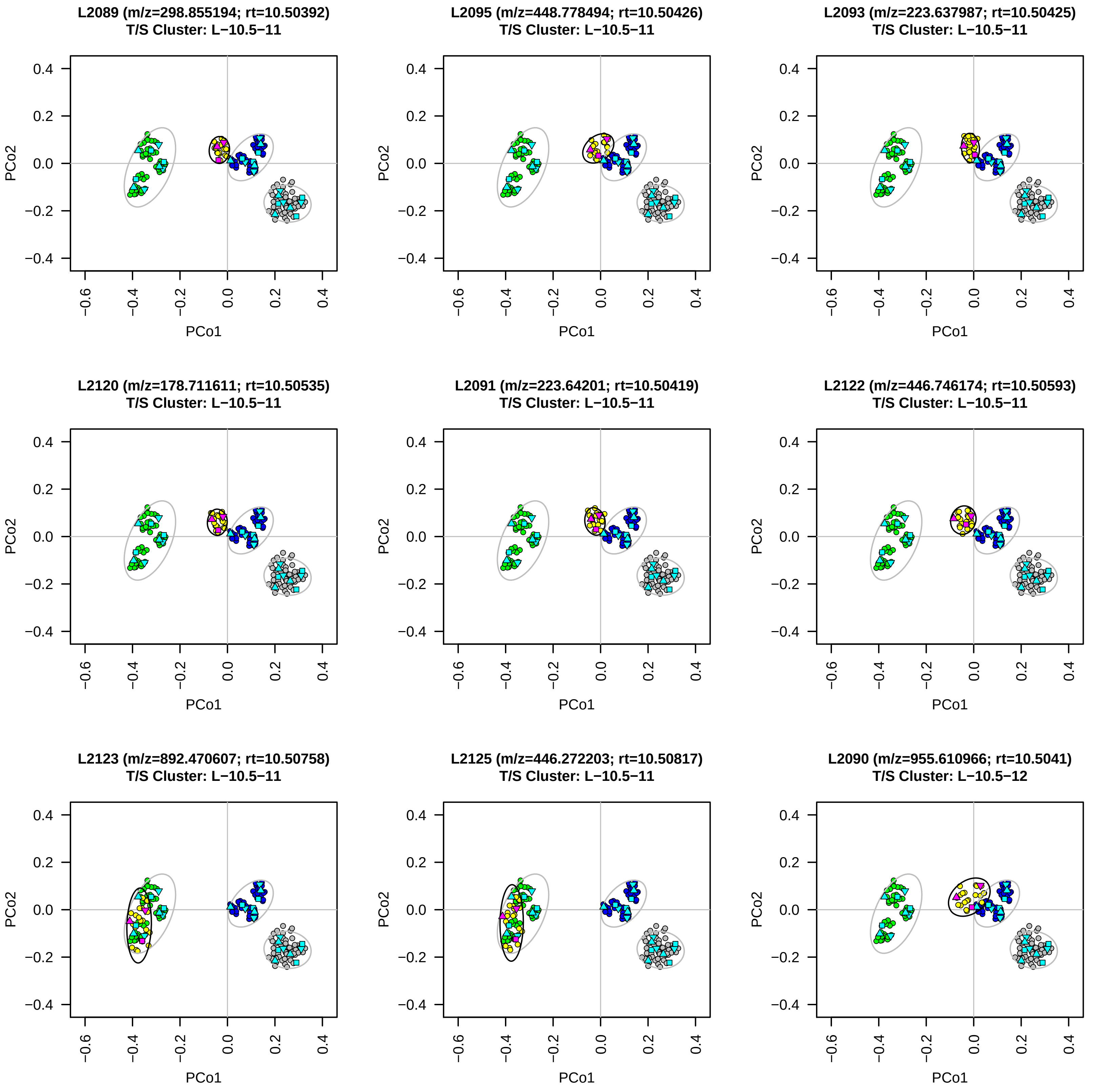
L2089 (m/z=298.855194; rt=10.50392)
T/S Cluster: L−10.5−11
L2095 (m/z=448.778494; rt=10.50426)
T/S Cluster: L−10.5−11
L2093 (m/z=223.637987; rt=10.50425)
T/S Cluster: L−10.5−11
L2120 (m/z=178.711611; rt=10.50535)
T/S Cluster: L−10.5−11
L2091 (m/z=223.64201; rt=10.50419)
T/S Cluster: L−10.5−11
L2122 (m/z=446.746174; rt=10.50593)
T/S Cluster: L−10.5−11
L2123 (m/z=892.470607; rt=10.50758)
T/S Cluster: L−10.5−11
L2125 (m/z=446.272203; rt=10.50817)
T/S Cluster: L−10.5−11
L2090 (m/z=955.610966; rt=10.5041)
T/S Cluster: L−10.5−12

L2114 (m/z=893.546107; rt=10.50511)
T/S Cluster: L−10.5−13

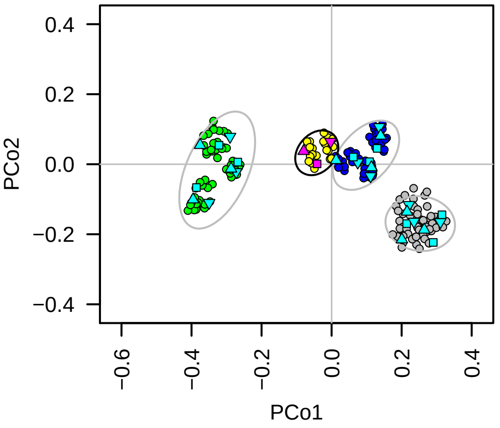

L2110 (m/z=894.549312; rt=10.50491)
T/S Cluster: L−10.5−13

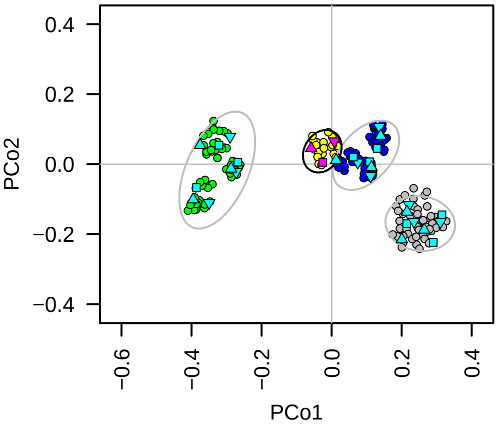

L2105 (m/z=895.550026; rt=10.50472)
T/S Cluster: L−10.5−13

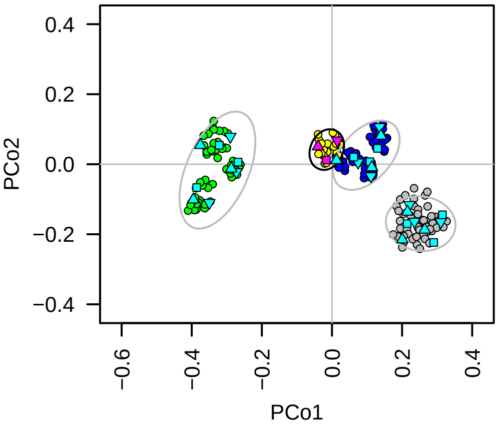

L2115 (m/z=446.774522; rt=10.50511)
T/S Cluster: L−10.5−13

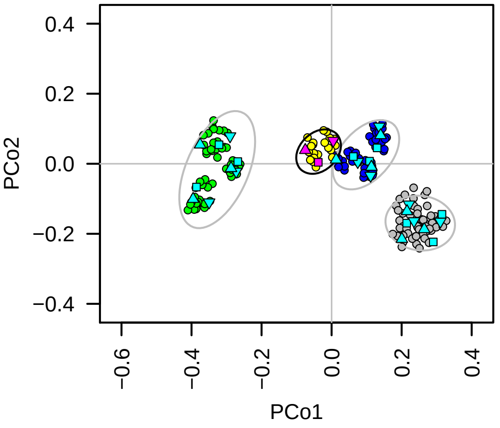

L2098 (m/z=896.552351; rt=10.50448)
T/S Cluster: L−10.5−13

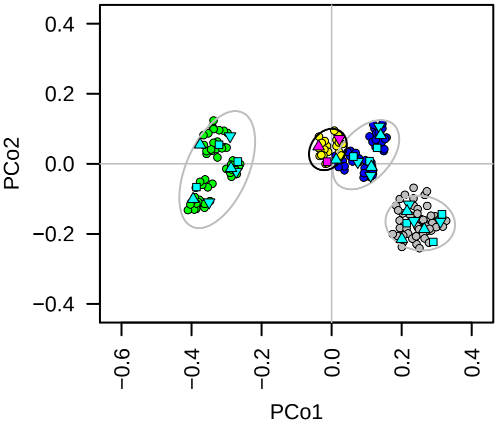

L2112 (m/z=894.484266; rt=10.50504)
T/S Cluster: L−10.5−13

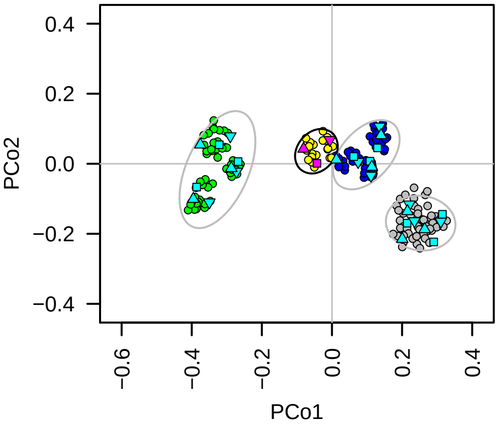

L2111 (m/z=895.496217; rt=10.50494)
T/S Cluster: L−10.5−13

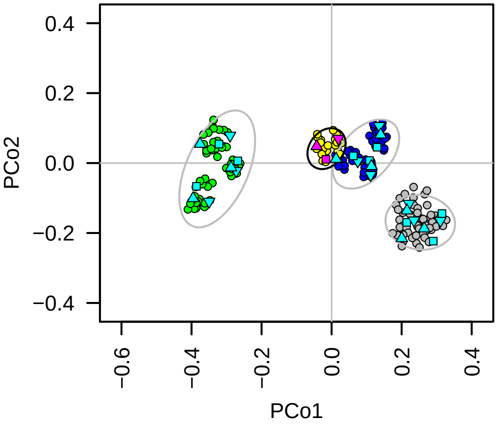

L2108 (m/z=447.276261; rt=10.50486)
T/S Cluster: L−10.5−13

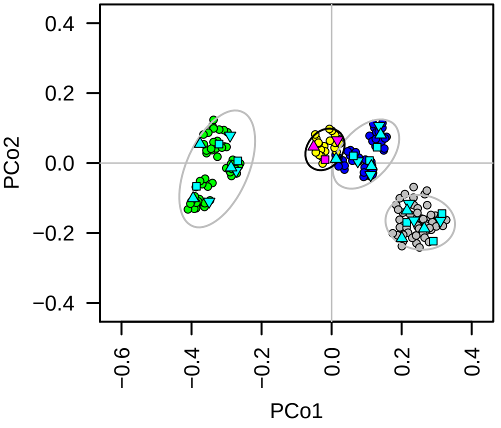

L2103 (m/z=896.508779; rt=10.50464)
T/S Cluster: L−10.5−13

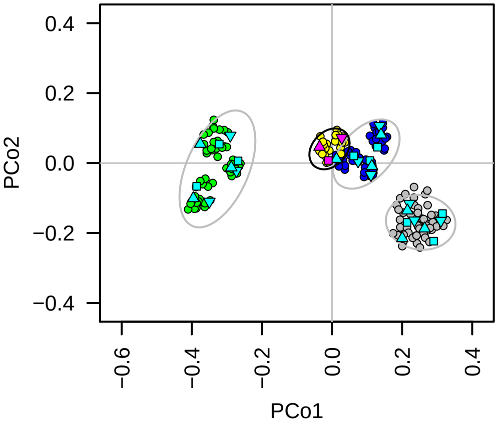

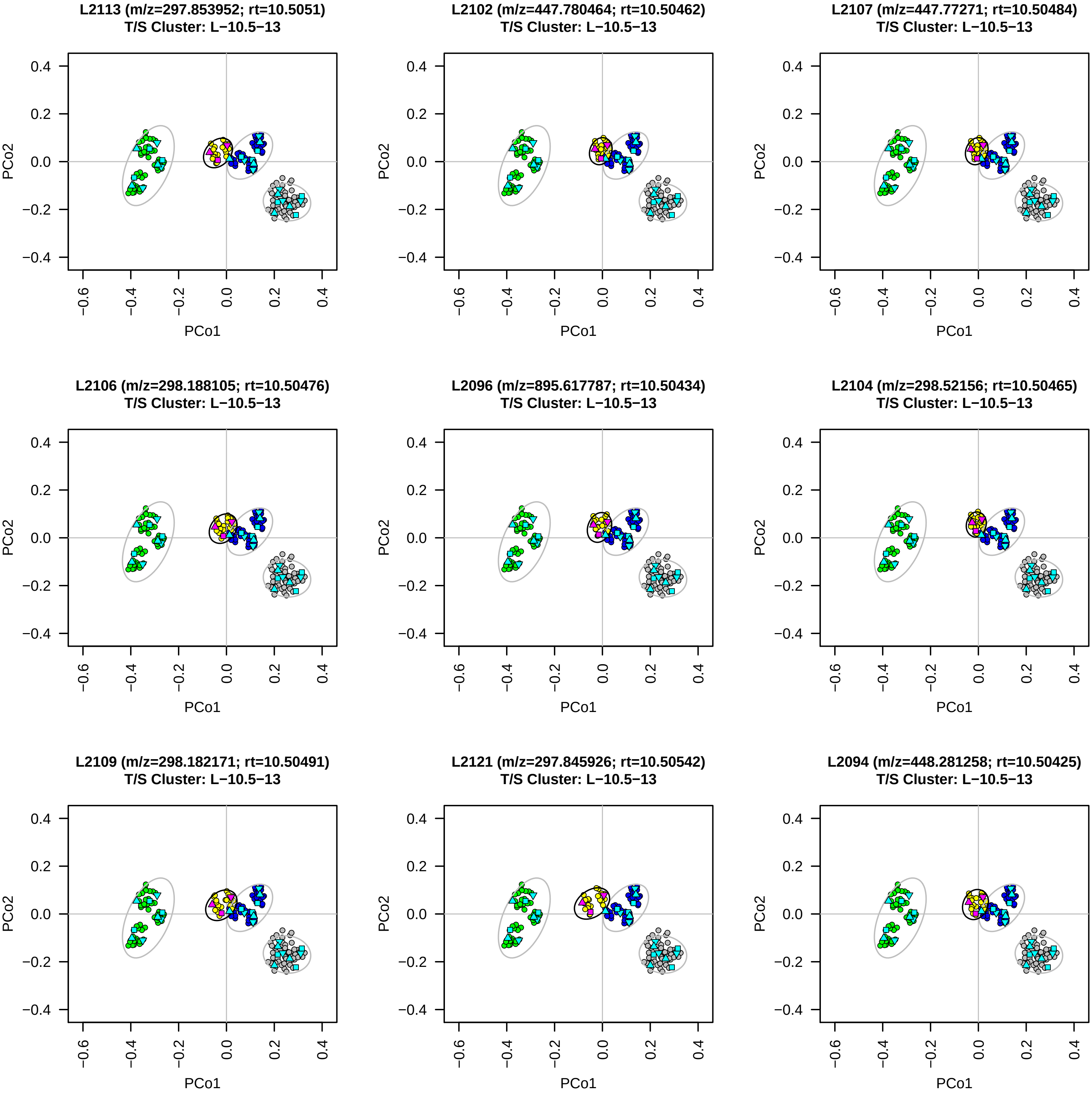
L2113 (m/z=297.853952; rt=10.5051)
T/S Cluster: L−10.5−13
L2102 (m/z=447.780464; rt=10.50462)
T/S Cluster: L−10.5−13
L2107 (m/z=447.77271; rt=10.50484)
T/S Cluster: L−10.5−13
L2106 (m/z=298.188105; rt=10.50476)
T/S Cluster: L−10.5−13
L2096 (m/z=895.617787; rt=10.50434)
T/S Cluster: L−10.5−13
L2104 (m/z=298.52156; rt=10.50465)
T/S Cluster: L−10.5−13
L2109 (m/z=298.182171; rt=10.50491)
T/S Cluster: L−10.5−13
L2121 (m/z=297.845926; rt=10.50542)
T/S Cluster: L−10.5−13
L2094 (m/z=448.281258; rt=10.50425)
T/S Cluster: L−10.5−13
PCo1
PCo2

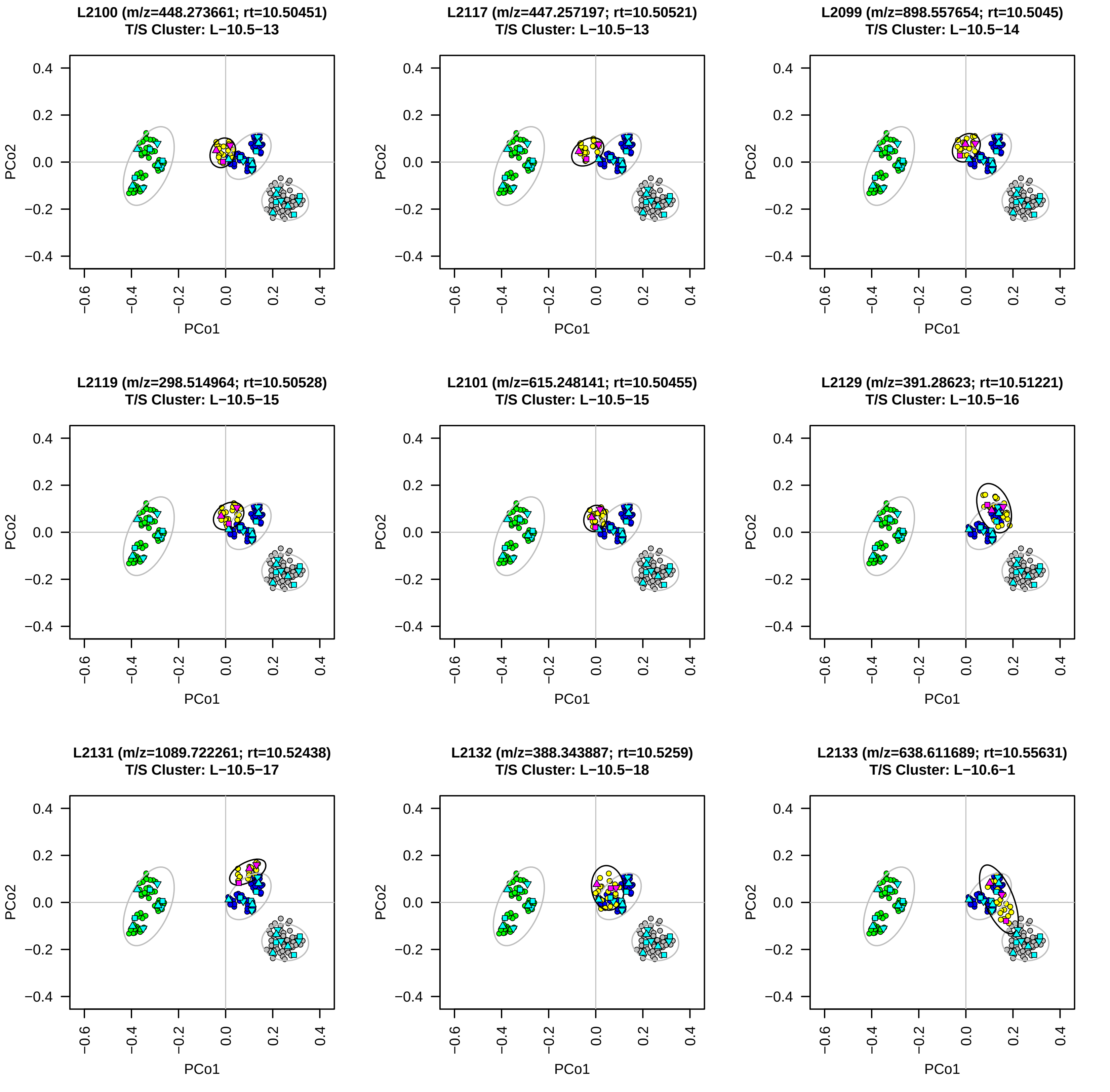
L2100 (m/z=448.273661; rt=10.50451)
T/S Cluster: L−10.5−13
L2117 (m/z=447.257197; rt=10.50521)
T/S Cluster: L−10.5−13
L2099 (m/z=898.557654; rt=10.5045)
T/S Cluster: L−10.5−14
L2119 (m/z=298.514964; rt=10.50528)
T/S Cluster: L−10.5−15
L2101 (m/z=615.248141; rt=10.50455)
T/S Cluster: L−10.5−15
L2129 (m/z=391.28623; rt=10.51221)
T/S Cluster: L−10.5−16
L2131 (m/z=1089.722261; rt=10.52438)
T/S Cluster: L−10.5−17
L2132 (m/z=388.343887; rt=10.5259)
T/S Cluster: L−10.5−18
L2133 (m/z=638.611689; rt=10.55631)
T/S Cluster: L−10.6−1
PCo1
PCo2
0.4
0.2
0.0
−0.2
−0.4
−0.6

L2136 (m/z=816.662962; rt=10.58508)
T/S Cluster: L−10.6−2

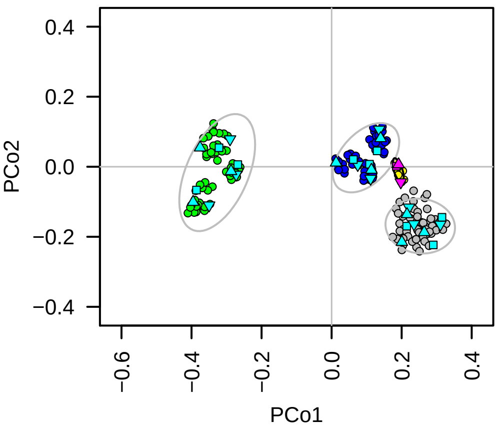

L2139 (m/z=816.638622; rt=10.58577)
T/S Cluster: L−10.6−2

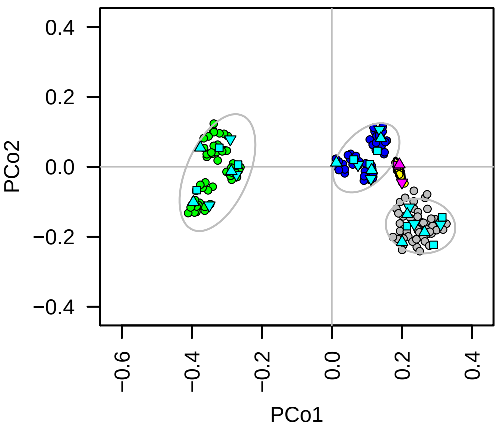

L2135 (m/z=817.666837; rt=10.58493)
T/S Cluster: L−10.6−2

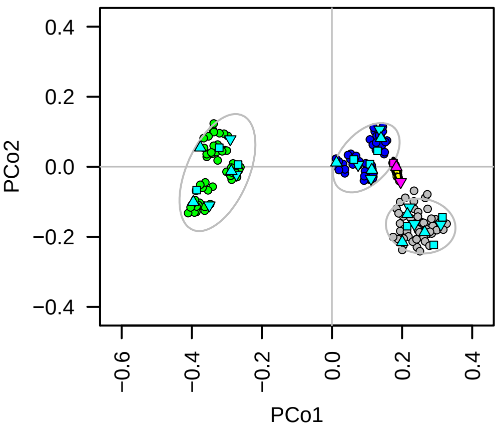

L2138 (m/z=817.642509; rt=10.58553)
T/S Cluster: L−10.6−2

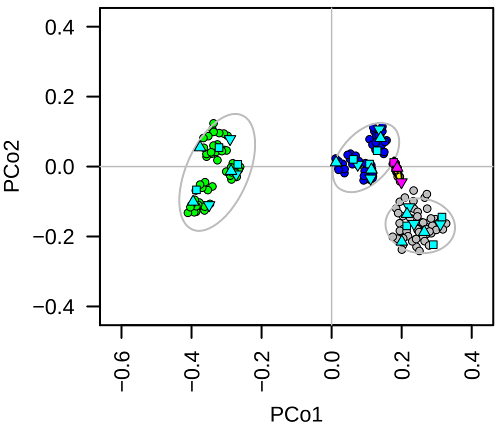

L2134 (m/z=818.666694; rt=10.58444)
T/S Cluster: L−10.6−2

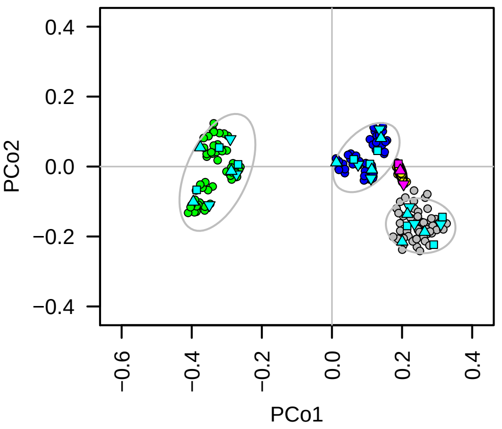

L2137 (m/z=408.331242; rt=10.58535)
T/S Cluster: L−10.6−2

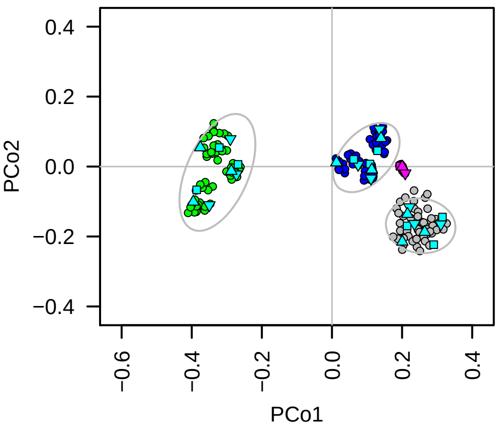

L2140 (m/z=429.852138; rt=10.60934)
T/S Cluster: L−10.6−3

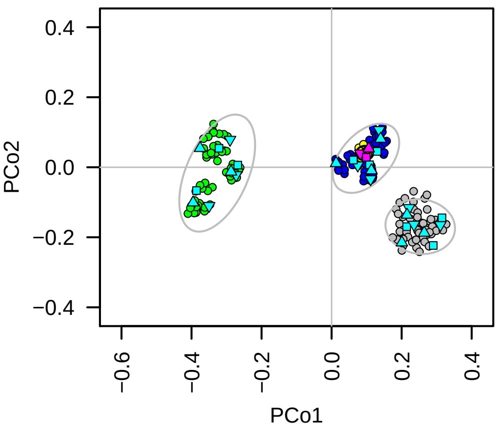

L2156 (m/z=842.677186; rt=10.61318)
T/S Cluster: L−10.6−4

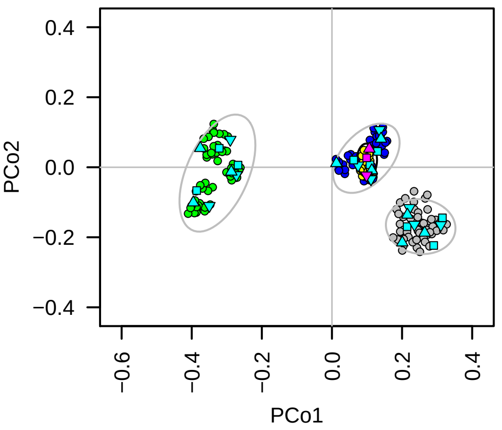

L2160 (m/z=842.646601; rt=10.61369)
T/S Cluster: L−10.6−4

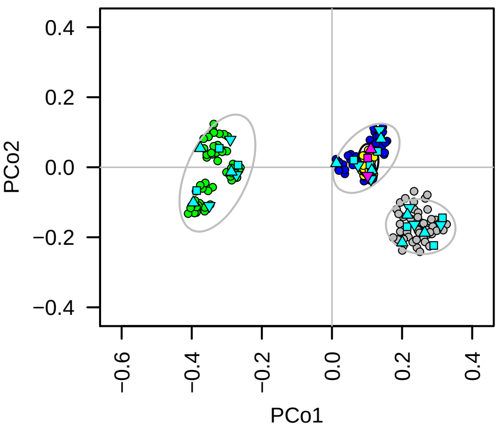

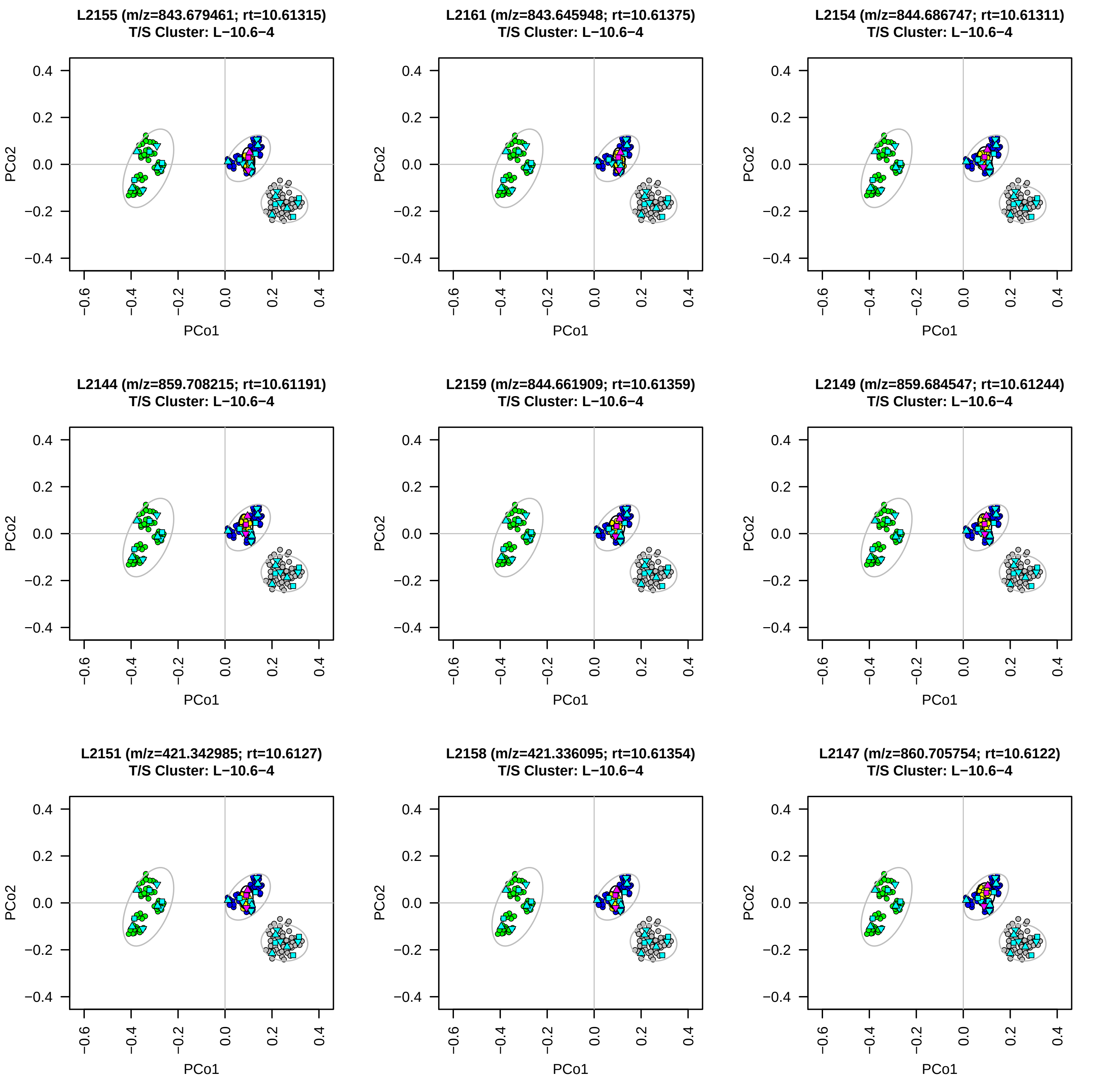
L2155 (m/z=843.679461; rt=10.61315)
T/S Cluster: L−10.6−4
L2161 (m/z=843.645948; rt=10.61375)
T/S Cluster: L−10.6−4
L2154 (m/z=844.686747; rt=10.61311)
T/S Cluster: L−10.6−4
L2144 (m/z=859.708215; rt=10.61191)
T/S Cluster: L−10.6−4
L2159 (m/z=844.661909; rt=10.61359)
T/S Cluster: L−10.6−4
L2149 (m/z=859.684547; rt=10.61244)
T/S Cluster: L−10.6−4
L2151 (m/z=421.342985; rt=10.6127)
T/S Cluster: L−10.6−4
L2158 (m/z=421.336095; rt=10.61354)
T/S Cluster: L−10.6−4
L2147 (m/z=860.705754; rt=10.6122)
T/S Cluster: L−10.6−4
PCo1
PCo2

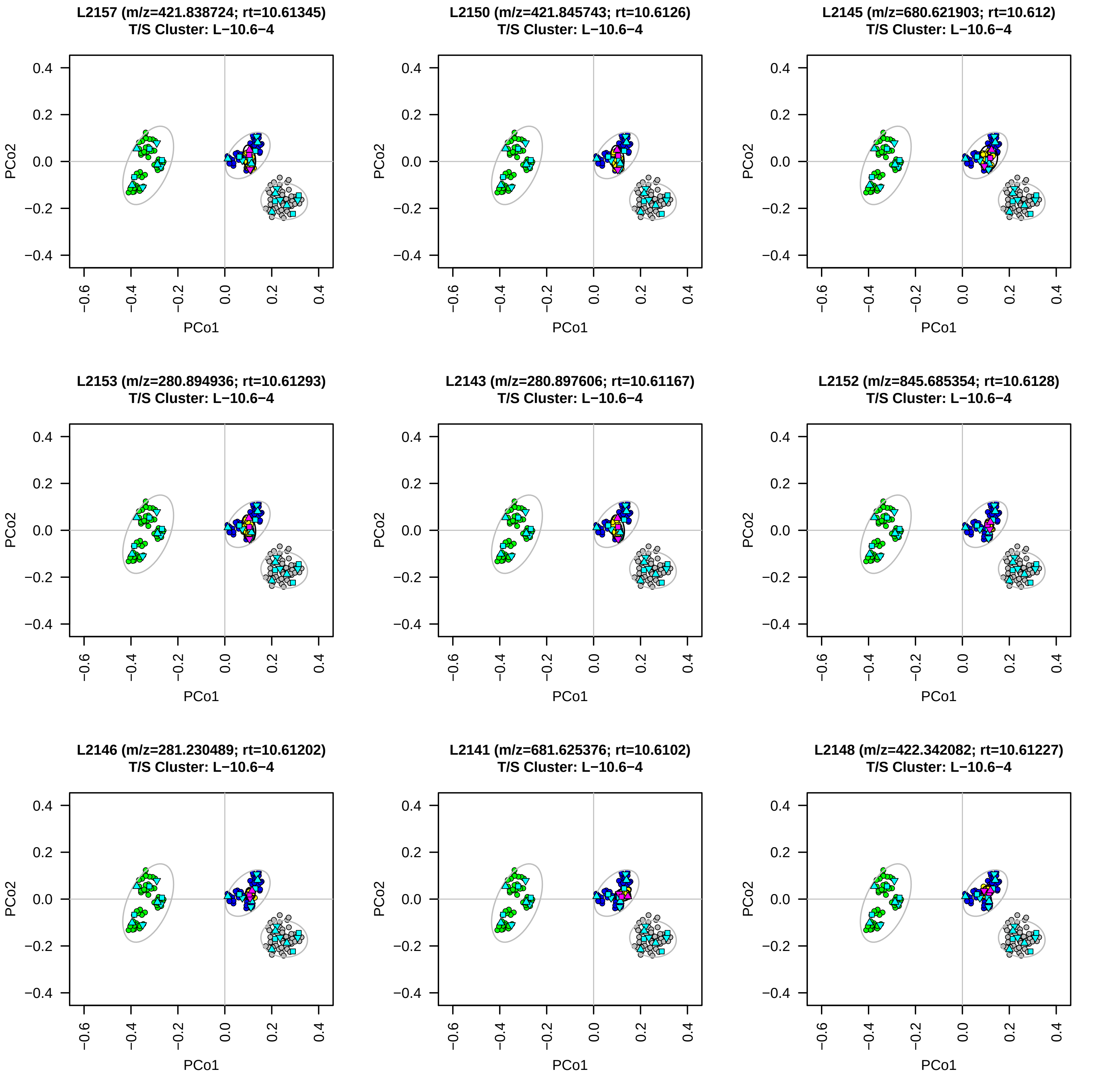
L2157 (m/z=421.838724; rt=10.61345)
T/S Cluster: L−10.6−4
L2150 (m/z=421.845743; rt=10.6126)
T/S Cluster: L−10.6−4
L2145 (m/z=680.621903; rt=10.612)
T/S Cluster: L−10.6−4
L2153 (m/z=280.894936; rt=10.61293)
T/S Cluster: L−10.6−4
L2143 (m/z=280.897606; rt=10.61167)
T/S Cluster: L−10.6−4
L2152 (m/z=845.685354; rt=10.6128)
T/S Cluster: L−10.6−4
L2146 (m/z=281.230489; rt=10.61202)
T/S Cluster: L−10.6−4
L2141 (m/z=681.625376; rt=10.6102)
T/S Cluster: L−10.6−4
L2148 (m/z=422.342082; rt=10.61227)
T/S Cluster: L−10.6−4
PCo1
PCo2

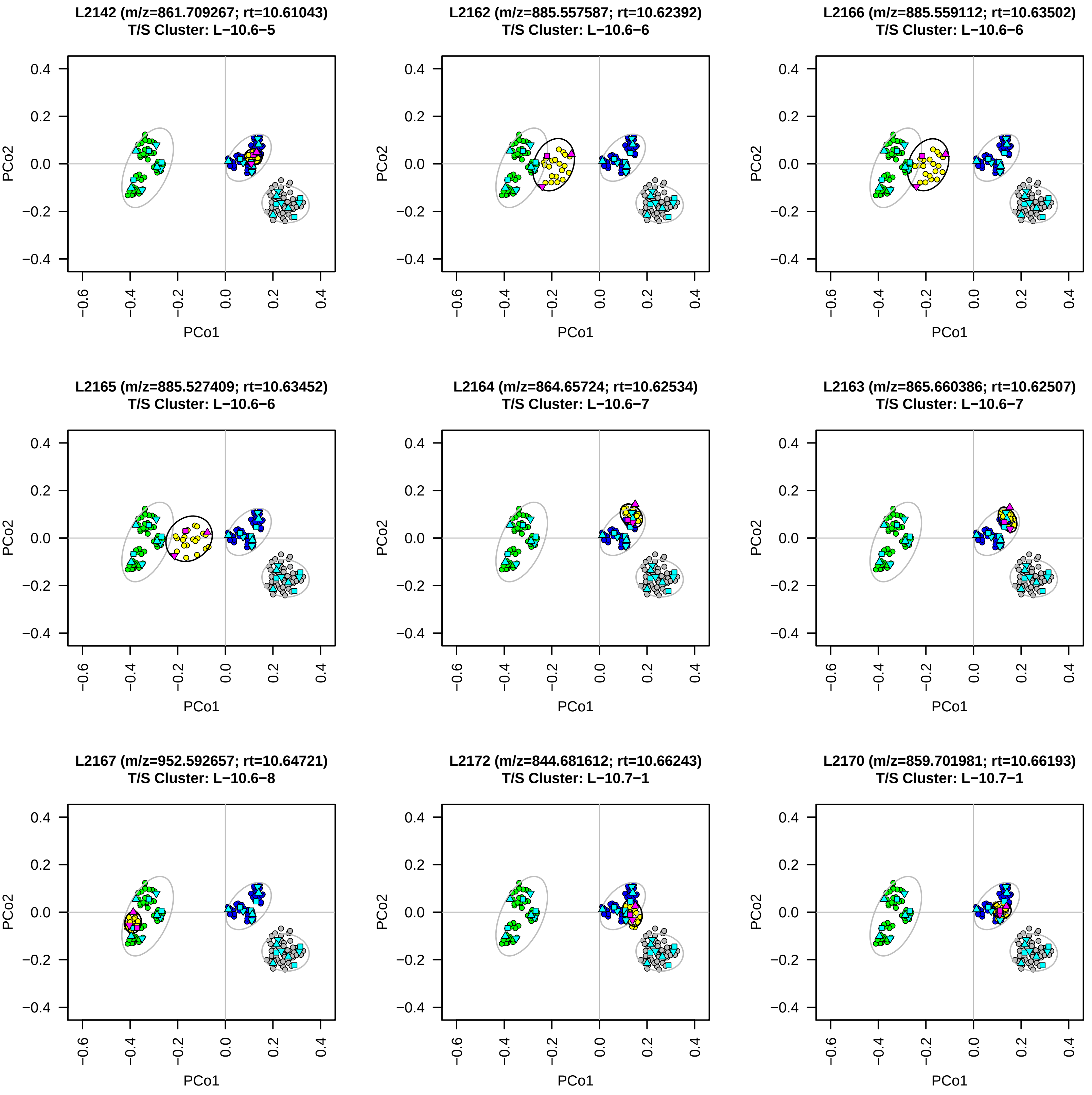
L2142 (m/z=861.709267; rt=10.61043)
T/S Cluster: L−10.6−5
L2162 (m/z=885.557587; rt=10.62392)
T/S Cluster: L−10.6−6
L2166 (m/z=885.559112; rt=10.63502)
T/S Cluster: L−10.6−6
L2165 (m/z=885.527409; rt=10.63452)
T/S Cluster: L−10.6−6
L2164 (m/z=864.65724; rt=10.62534)
T/S Cluster: L−10.6−7
L2163 (m/z=865.660386; rt=10.62507)
T/S Cluster: L−10.6−7
L2167 (m/z=952.592657; rt=10.64721)
T/S Cluster: L−10.6−8
L2172 (m/z=844.681612; rt=10.66243)
T/S Cluster: L−10.7−1
L2170 (m/z=859.701981; rt=10.66193)
T/S Cluster: L−10.7−1
PCo1
PCo2

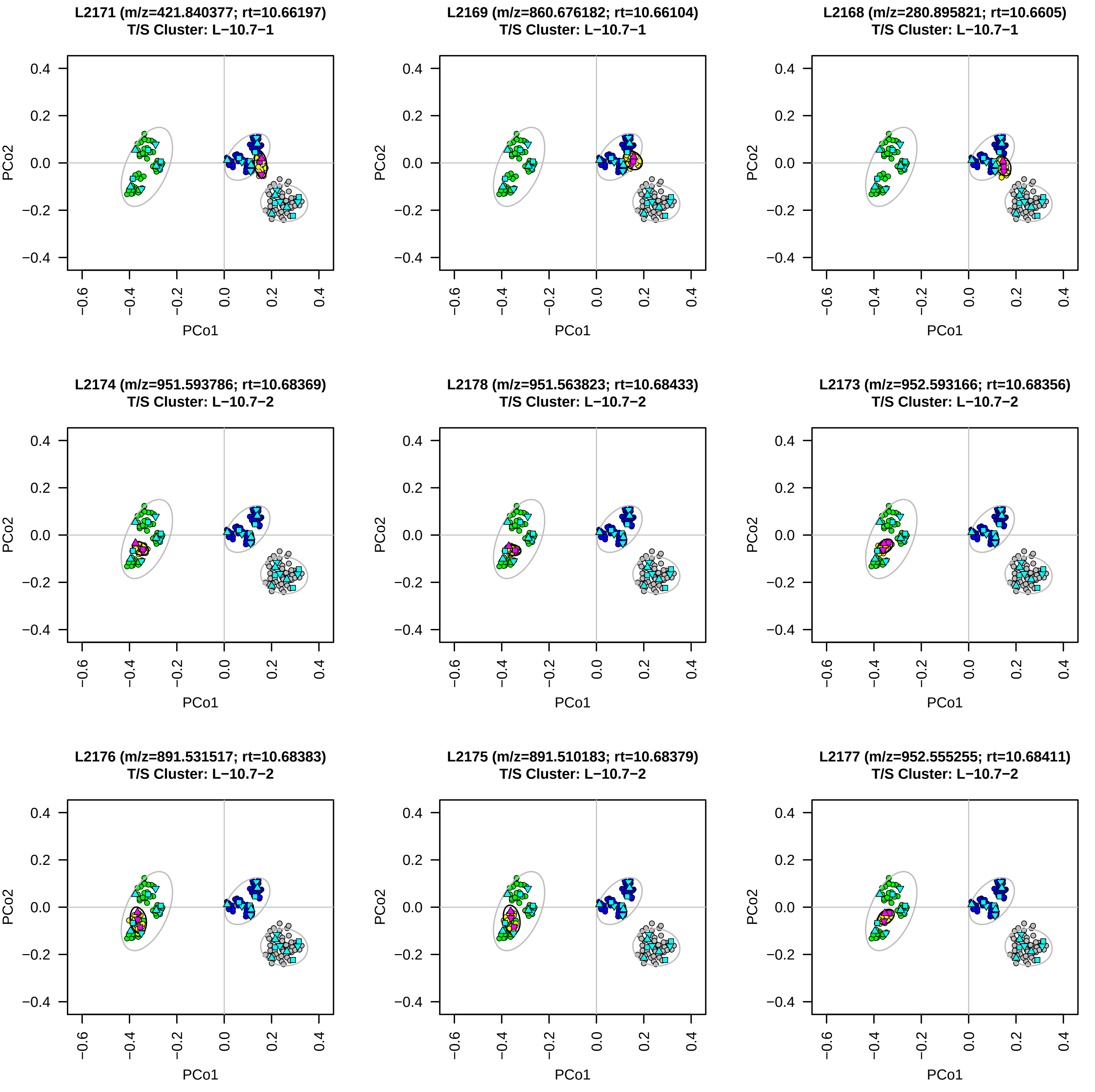
L2171 (m/z=421.840377; rt=10.66197)
T/S Cluster: L−10.7−1
L2169 (m/z=860.676182; rt=10.66104)
T/S Cluster: L−10.7−1
L2168 (m/z=280.895821; rt=10.6605)
T/S Cluster: L−10.7−1
L2174 (m/z=951.593786; rt=10.68369)
T/S Cluster: L−10.7−2
L2178 (m/z=951.563823; rt=10.68433)
T/S Cluster: L−10.7−2
L2173 (m/z=952.593166; rt=10.68356)
T/S Cluster: L−10.7−2
L2176 (m/z=891.531517; rt=10.68383)
T/S Cluster: L−10.7−2
L2175 (m/z=891.510183; rt=10.68379)
T/S Cluster: L−10.7−2
L2177 (m/z=952.555255; rt=10.68411)
T/S Cluster: L−10.7−2
PCo1
PCo2
−0.6
−0.4
−0.2
0.0
0.2
0.4

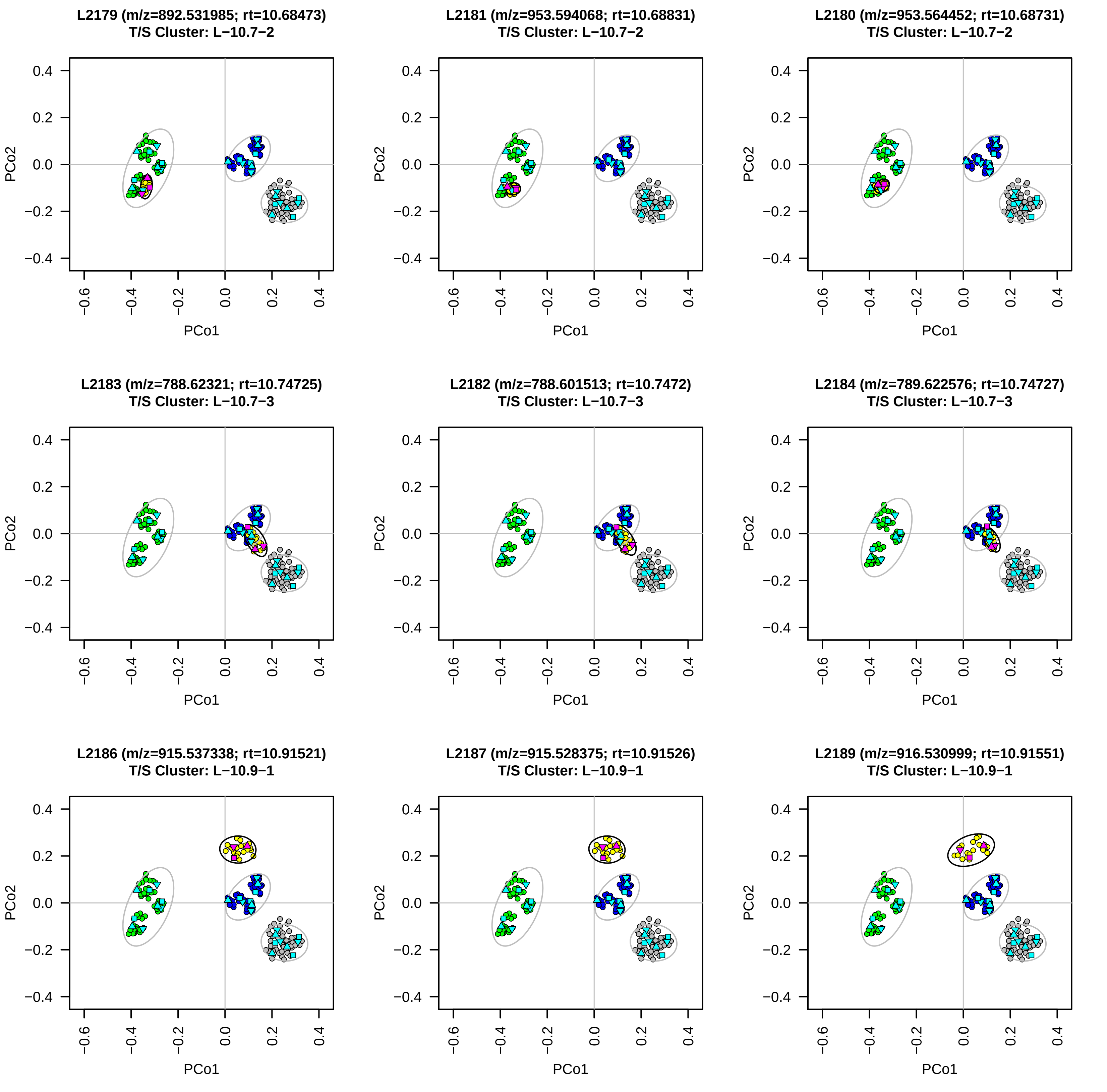
L2179 (m/z=892.531985; rt=10.68473)
T/S Cluster: L−10.7−2
L2181 (m/z=953.594068; rt=10.68831)
T/S Cluster: L−10.7−2
L2180 (m/z=953.564452; rt=10.68731)
T/S Cluster: L−10.7−2
L2183 (m/z=788.62321; rt=10.74725)
T/S Cluster: L−10.7−3
L2182 (m/z=788.601513; rt=10.7472)
T/S Cluster: L−10.7−3
L2184 (m/z=789.622576; rt=10.74727)
T/S Cluster: L−10.7−3
L2186 (m/z=915.537338; rt=10.91521)
T/S Cluster: L−10.9−1
L2187 (m/z=915.528375; rt=10.91526)
T/S Cluster: L−10.9−1
L2189 (m/z=916.530999; rt=10.91551)
T/S Cluster: L−10.9−1
PCo1
PCo2
0.4
0.2
0.0
−0.2
−0.4
−0.6

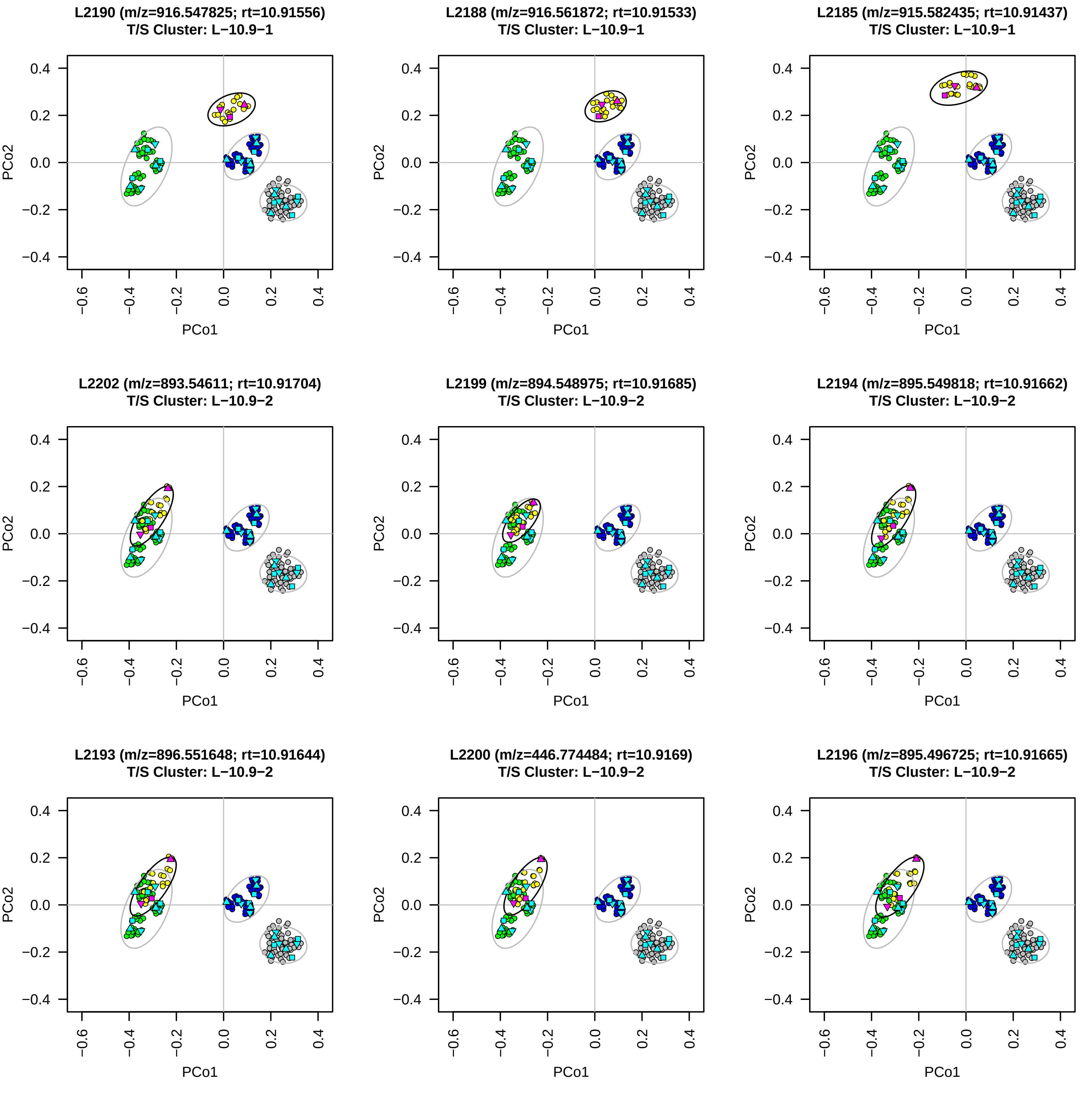
L2190 (m/z=916.547825; rt=10.91556)
T/S Cluster: L−10.9−1
L2188 (m/z=916.561872; rt=10.91533)
T/S Cluster: L−10.9−1
L2185 (m/z=915.582435; rt=10.91437)
T/S Cluster: L−10.9−1
L2202 (m/z=893.54611; rt=10.91704)
T/S Cluster: L−10.9−2
L2199 (m/z=894.548975; rt=10.91685)
T/S Cluster: L−10.9−2
L2194 (m/z=895.549818; rt=10.91662)
T/S Cluster: L−10.9−2
L2193 (m/z=896.551648; rt=10.91644)
T/S Cluster: L−10.9−2
L2200 (m/z=446.774484; rt=10.9169)
T/S Cluster: L−10.9−2
L2196 (m/z=895.496725; rt=10.91665)
T/S Cluster: L−10.9−2
PCo1
PCo2
−0.6
−0.4
−0.2
0.0
0.2
0.4

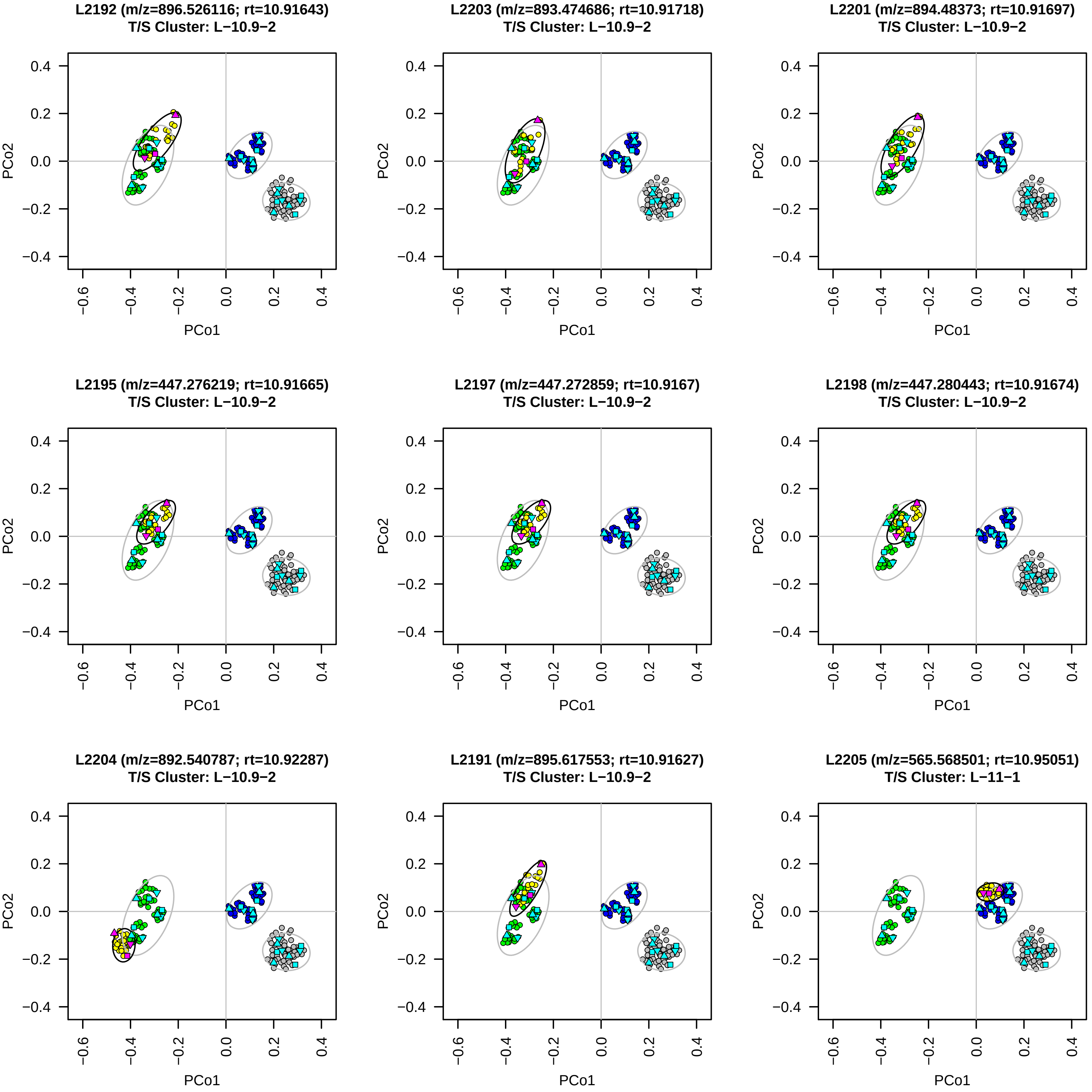
L2192 (m/z=896.526116; rt=10.91643)
T/S Cluster: L−10.9−2
L2203 (m/z=893.474686; rt=10.91718)
T/S Cluster: L−10.9−2
L2201 (m/z=894.48373; rt=10.91697)
T/S Cluster: L−10.9−2
L2195 (m/z=447.276219; rt=10.91665)
T/S Cluster: L−10.9−2
L2197 (m/z=447.272859; rt=10.9167)
T/S Cluster: L−10.9−2
L2198 (m/z=447.280443; rt=10.91674)
T/S Cluster: L−10.9−2
L2204 (m/z=892.540787; rt=10.92287)
T/S Cluster: L−10.9−2
L2191 (m/z=895.617553; rt=10.91627)
T/S Cluster: L−10.9−2
L2205 (m/z=565.568501; rt=10.95051)
T/S Cluster: L−11−1
PCo1
PCo2
0.4
0.2
0.0
−0.2
−0.4
−0.6

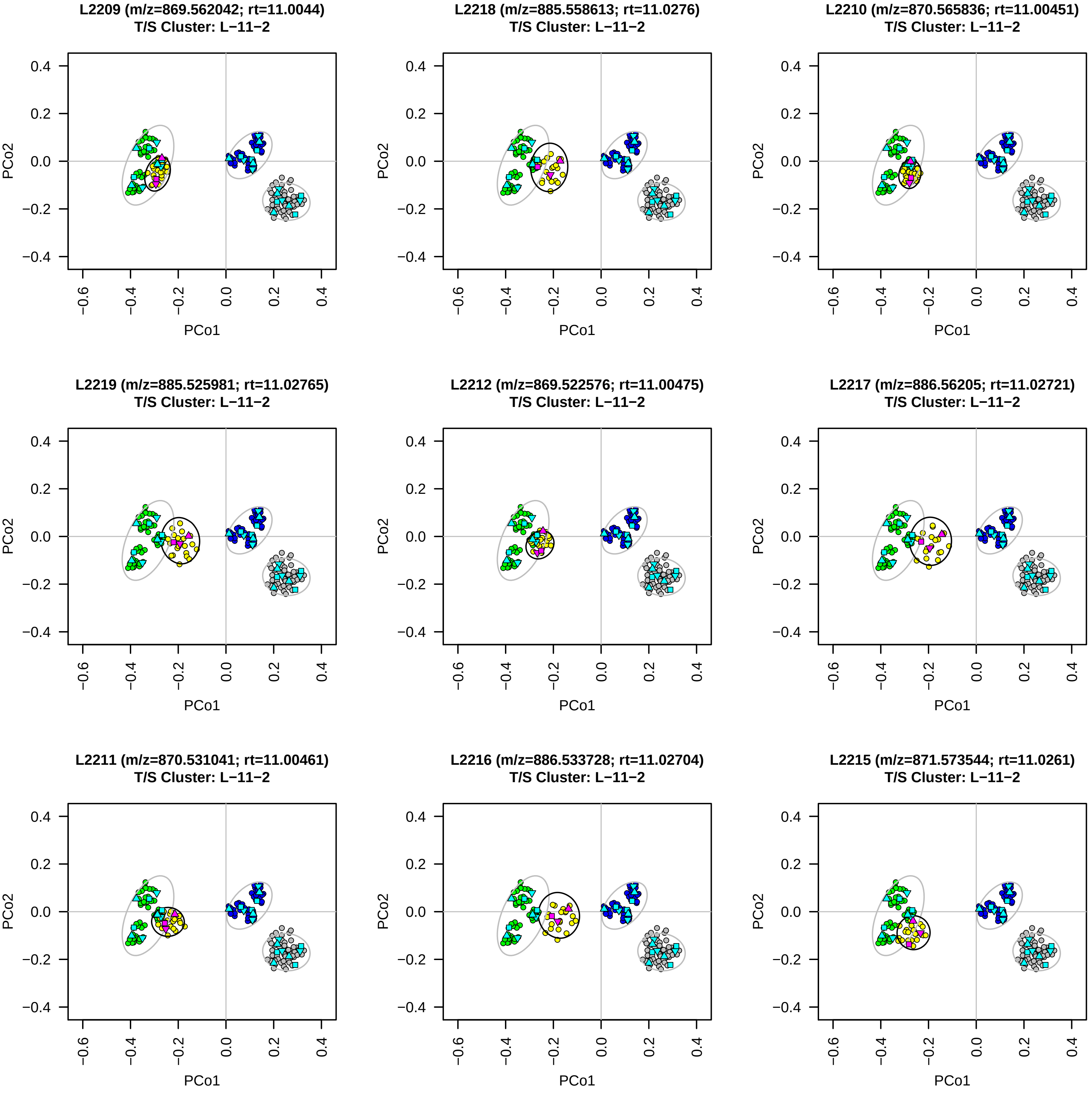
L2209 (m/z=869.562042; rt=11.0044)
T/S Cluster: L−11−2
L2218 (m/z=885.558613; rt=11.0276)
T/S Cluster: L−11−2
L2210 (m/z=870.565836; rt=11.00451)
T/S Cluster: L−11−2
L2219 (m/z=885.525981; rt=11.02765)
T/S Cluster: L−11−2
L2212 (m/z=869.522576; rt=11.00475)
T/S Cluster: L−11−2
L2217 (m/z=886.56205; rt=11.02721)
T/S Cluster: L−11−2
L2211 (m/z=870.531041; rt=11.00461)
T/S Cluster: L−11−2
L2216 (m/z=886.533728; rt=11.02704)
T/S Cluster: L−11−2
L2215 (m/z=871.573544; rt=11.0261)
T/S Cluster: L−11−2
PCo1
PCo2
0.4
0.2
0.0
−0.2
−0.4
−0.6

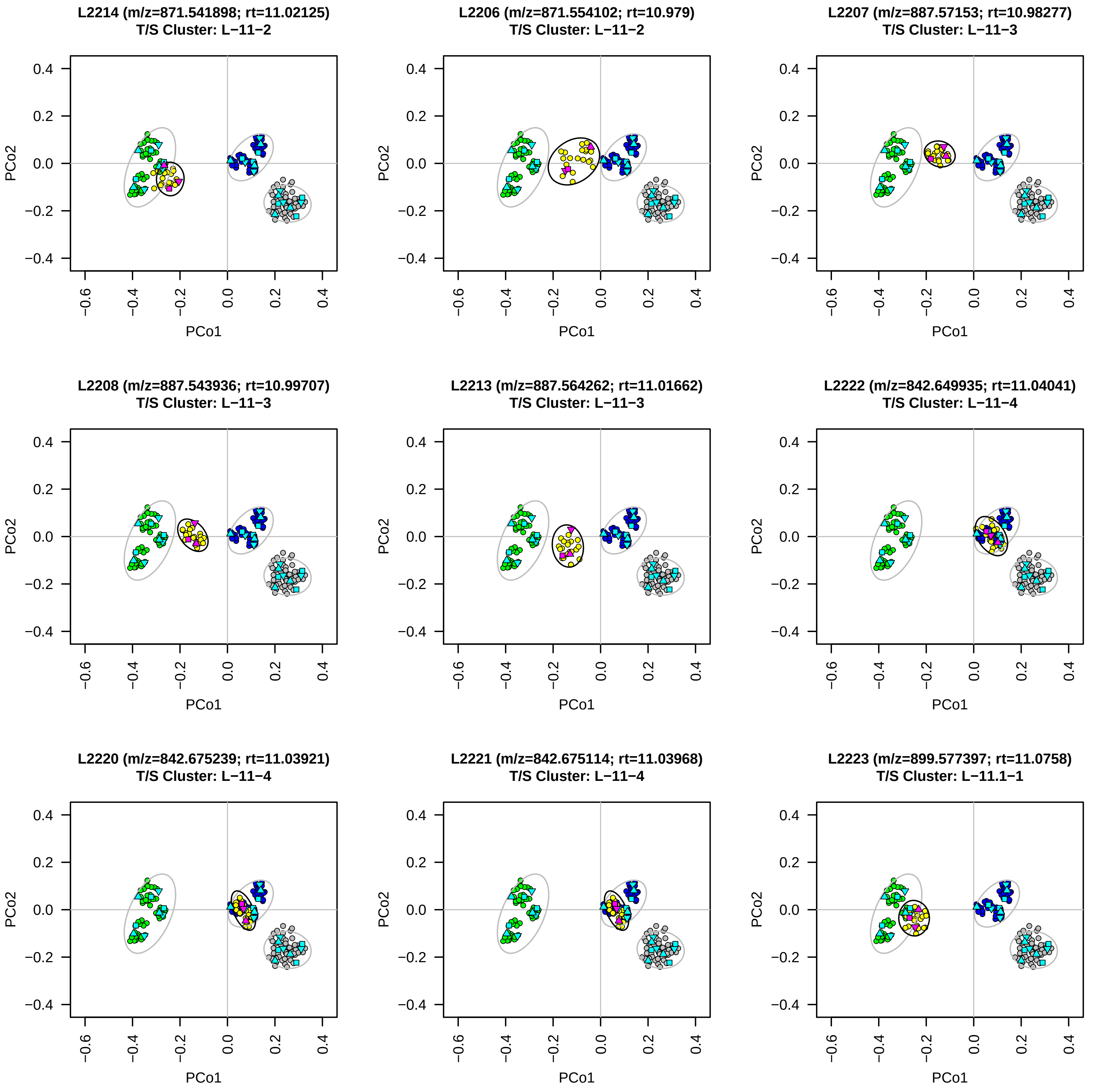
L2214 (m/z=871.541898; rt=11.02125)
T/S Cluster: L−11−2
L2206 (m/z=871.554102; rt=10.979)
T/S Cluster: L−11−2
L2207 (m/z=887.57153; rt=10.98277)
T/S Cluster: L−11−3
L2208 (m/z=887.543936; rt=10.99707)
T/S Cluster: L−11−3
L2213 (m/z=887.564262; rt=11.01662)
T/S Cluster: L−11−3
L2222 (m/z=842.649935; rt=11.04041)
T/S Cluster: L−11−4
L2220 (m/z=842.675239; rt=11.03921)
T/S Cluster: L−11−4
L2221 (m/z=842.675114; rt=11.03968)
T/S Cluster: L−11−4
L2223 (m/z=899.577397; rt=11.0758)
T/S Cluster: L−11.1−1
PCo1
PCo2
0.4
0.2
0.0
−0.2
−0.4
−0.6

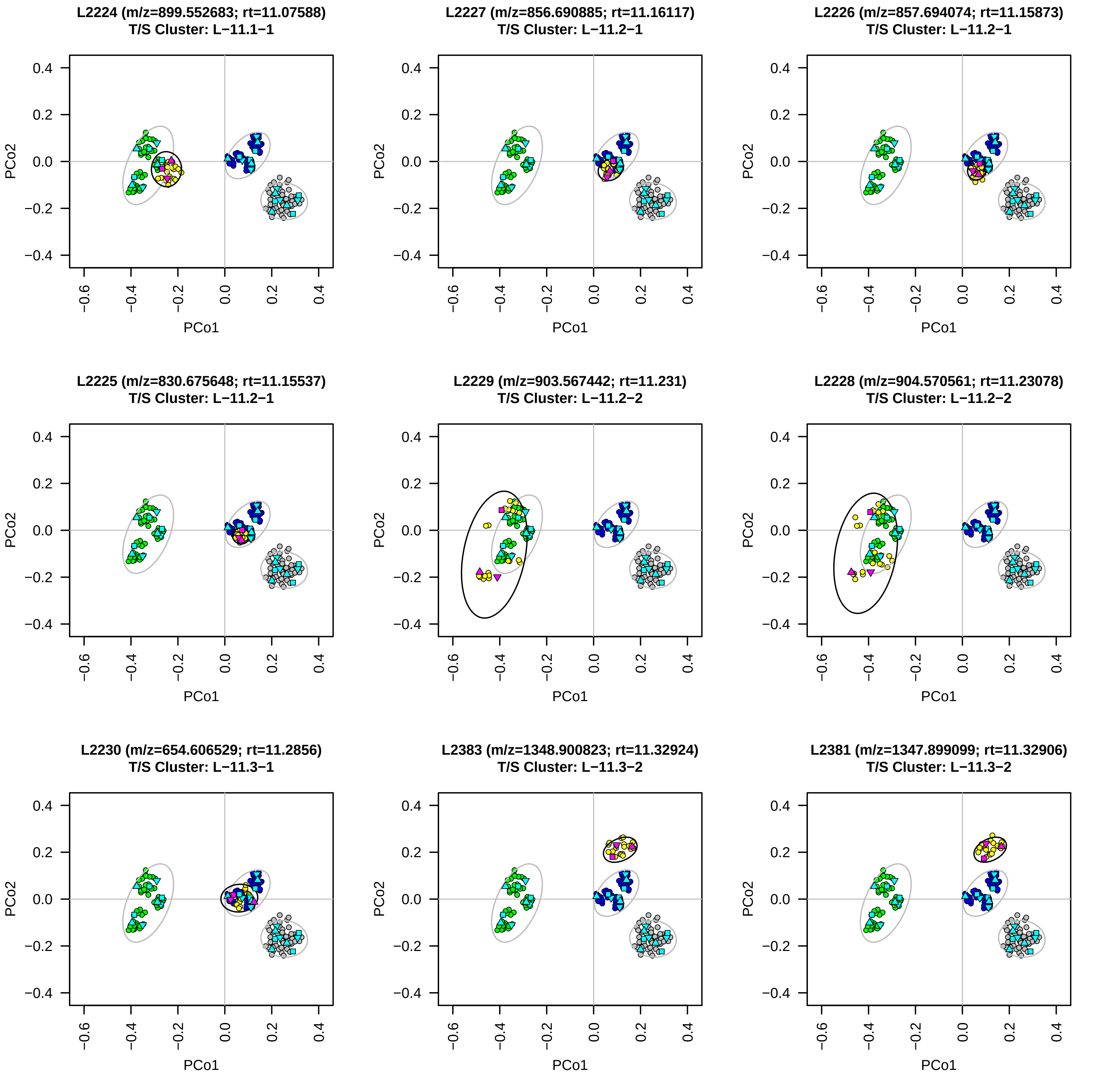
L2224 (m/z=899.552683; rt=11.07588)
T/S Cluster: L−11.1−1
L2227 (m/z=856.690885; rt=11.16117)
T/S Cluster: L−11.2−1
L2226 (m/z=857.694074; rt=11.15873)
T/S Cluster: L−11.2−1
L2225 (m/z=830.675648; rt=11.15537)
T/S Cluster: L−11.2−1
L2229 (m/z=903.567442; rt=11.231)
T/S Cluster: L−11.2−2
L2228 (m/z=904.570561; rt=11.23078)
T/S Cluster: L−11.2−2
L2230 (m/z=654.606529; rt=11.2856)
T/S Cluster: L−11.3−1
L2383 (m/z=1348.900823; rt=11.32924)
T/S Cluster: L−11.3−2
L2381 (m/z=1347.899099; rt=11.32906)
T/S Cluster: L−11.3−2
PCo1
PCo2
−0.6
−0.4
−0.2
0.0
0.2
0.4

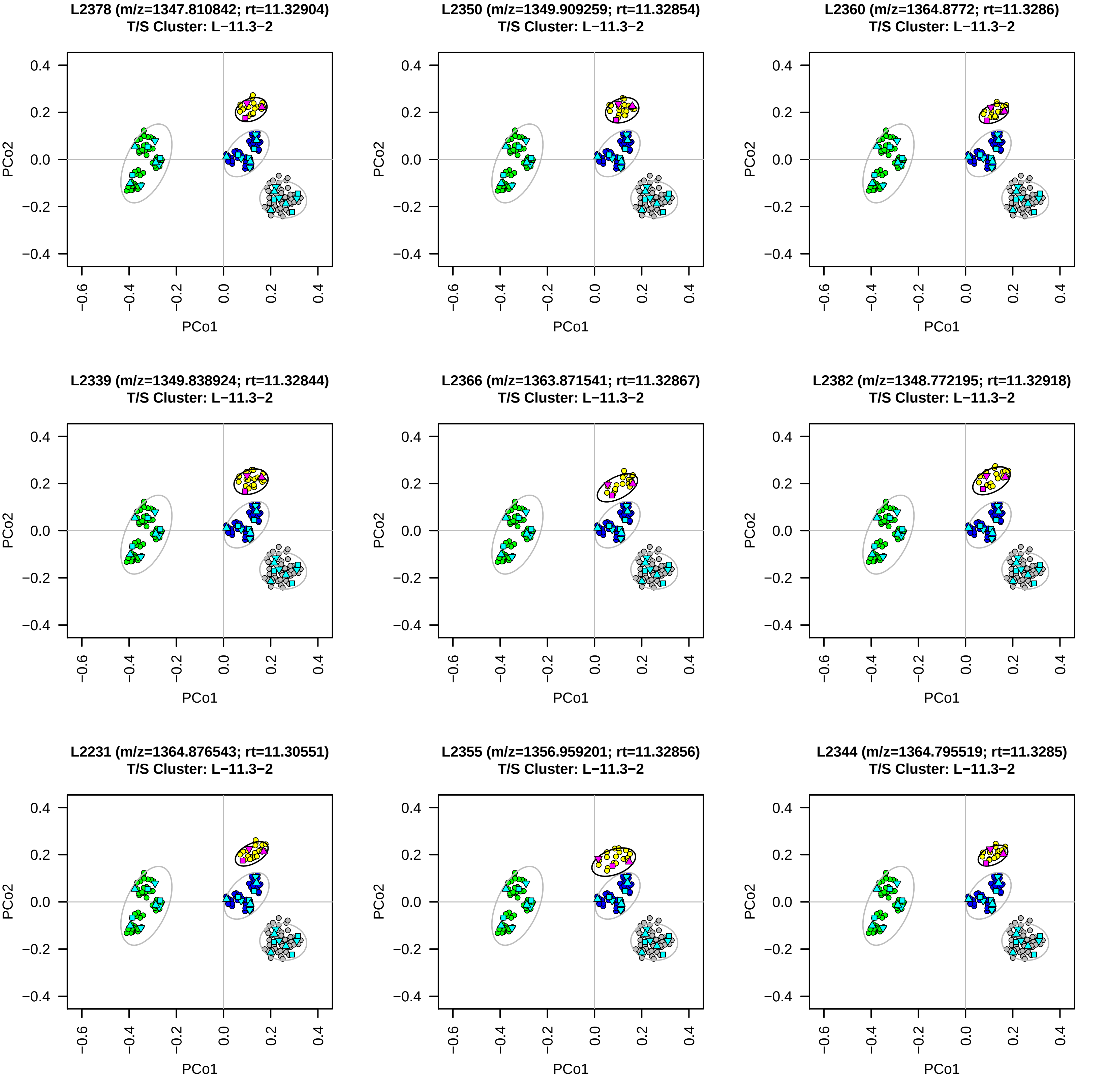

L2378 (m/z=1347.810842; rt=11.32904)
T/S Cluster: L−11.3−2
L2350 (m/z=1349.909259; rt=11.32854)
T/S Cluster: L−11.3−2
L2360 (m/z=1364.8772; rt=11.3286)
T/S Cluster: L−11.3−2
L2339 (m/z=1349.838924; rt=11.32844)
T/S Cluster: L−11.3−2
L2366 (m/z=1363.871541; rt=11.32867)
T/S Cluster: L−11.3−2
L2382 (m/z=1348.772195; rt=11.32918)
T/S Cluster: L−11.3−2
L2231 (m/z=1364.876543; rt=11.30551)
T/S Cluster: L−11.3−2
L2355 (m/z=1356.959201; rt=11.32856)
T/S Cluster: L−11.3−2
L2344 (m/z=1364.795519; rt=11.3285)
T/S Cluster: L−11.3−2
PCo1
PCo2
0.4
0.2
0.0
−0.2
−0.4
−0.6

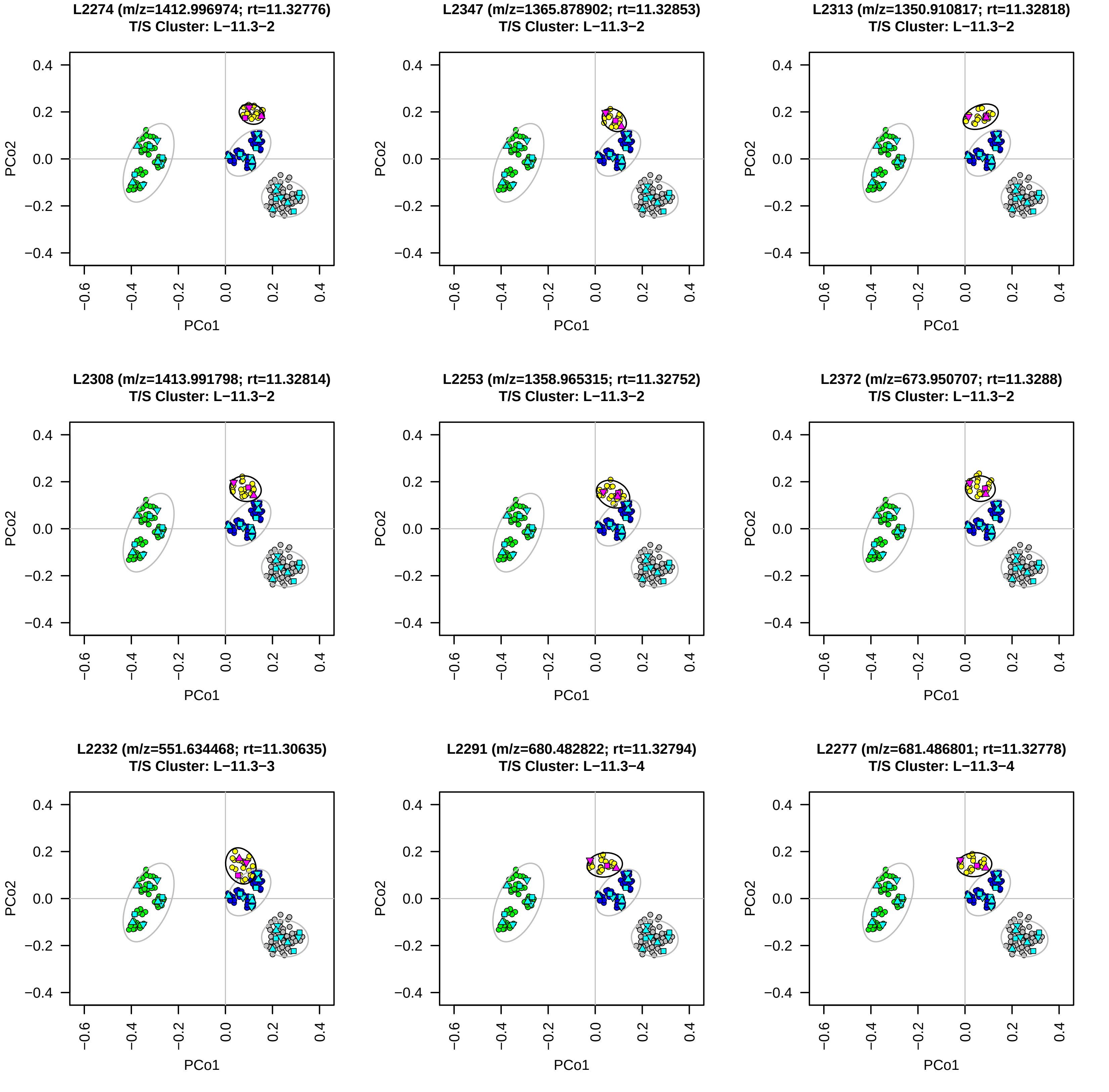

L2274 (m/z=1412.996974; rt=11.32776)
T/S Cluster: L−11.3−2
L2347 (m/z=1365.878902; rt=11.32853)
T/S Cluster: L−11.3−2
L2313 (m/z=1350.910817; rt=11.32818)
T/S Cluster: L−11.3−2
L2308 (m/z=1413.991798; rt=11.32814)
T/S Cluster: L−11.3−2
L2253 (m/z=1358.965315; rt=11.32752)
T/S Cluster: L−11.3−2
L2372 (m/z=673.950707; rt=11.3288)
T/S Cluster: L−11.3−2
L2232 (m/z=551.634468; rt=11.30635)
T/S Cluster: L−11.3−3
L2291 (m/z=680.482822; rt=11.32794)
T/S Cluster: L−11.3−4
L2277 (m/z=681.486801; rt=11.32778)
T/S Cluster: L−11.3−4
PCo1
PCo2
−0.6
−0.4
−0.2
0.0
0.2
0.4

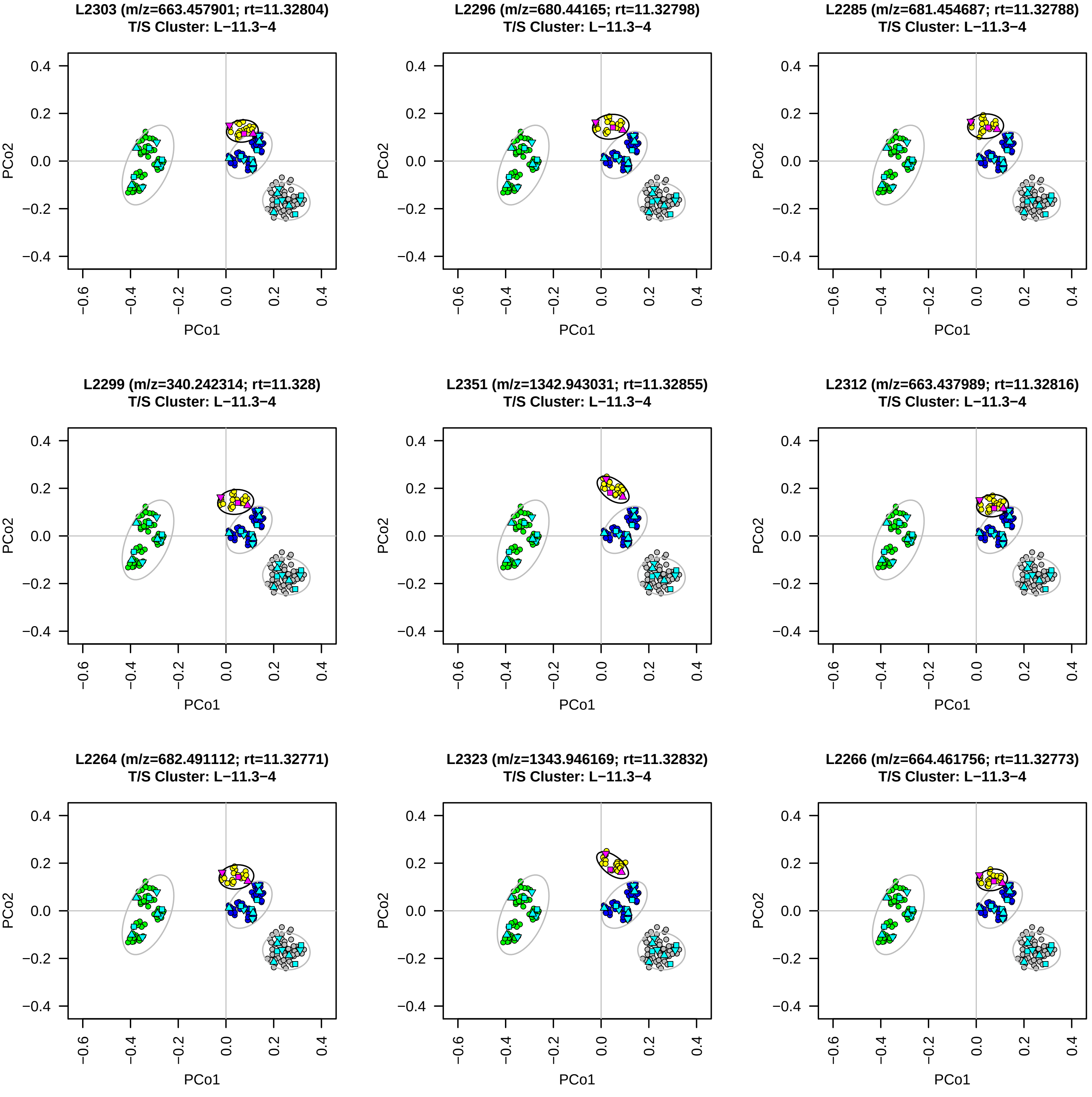
L2303 (m/z=663.457901; rt=11.32804)
T/S Cluster: L−11.3−4
L2296 (m/z=680.44165; rt=11.32798)
T/S Cluster: L−11.3−4
L2285 (m/z=681.454687; rt=11.32788)
T/S Cluster: L−11.3−4
L2299 (m/z=340.242314; rt=11.328)
T/S Cluster: L−11.3−4
L2351 (m/z=1342.943031; rt=11.32855)
T/S Cluster: L−11.3−4
L2312 (m/z=663.437989; rt=11.32816)
T/S Cluster: L−11.3−4
L2264 (m/z=682.491112; rt=11.32771)
T/S Cluster: L−11.3−4
L2323 (m/z=1343.946169; rt=11.32832)
T/S Cluster: L−11.3−4
L2266 (m/z=664.461756; rt=11.32773)
T/S Cluster: L−11.3−4
PCo1
PCo2

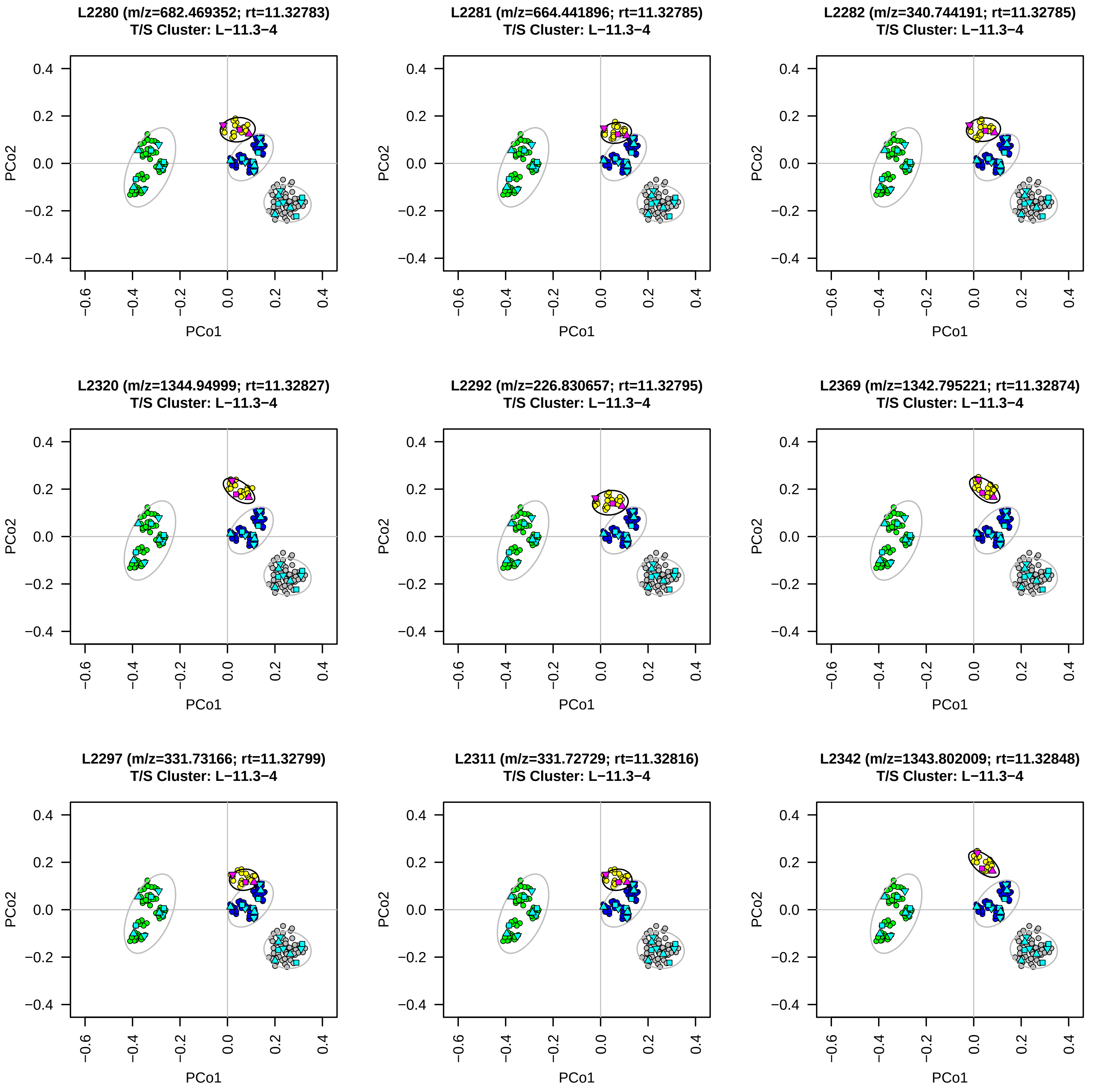
L2280 (m/z=682.469352; rt=11.32783)
T/S Cluster: L−11.3−4
L2281 (m/z=664.441896; rt=11.32785)
T/S Cluster: L−11.3−4
L2282 (m/z=340.744191; rt=11.32785)
T/S Cluster: L−11.3−4
L2320 (m/z=1344.94999; rt=11.32827)
T/S Cluster: L−11.3−4
L2292 (m/z=226.830657; rt=11.32795)
T/S Cluster: L−11.3−4
L2369 (m/z=1342.795221; rt=11.32874)
T/S Cluster: L−11.3−4
L2297 (m/z=331.73166; rt=11.32799)
T/S Cluster: L−11.3−4
L2311 (m/z=331.72729; rt=11.32816)
T/S Cluster: L−11.3−4
L2342 (m/z=1343.802009; rt=11.32848)
T/S Cluster: L−11.3−4
PCo1
PCo2
0.4
0.2
0.0
−0.2
−0.4
−0.6

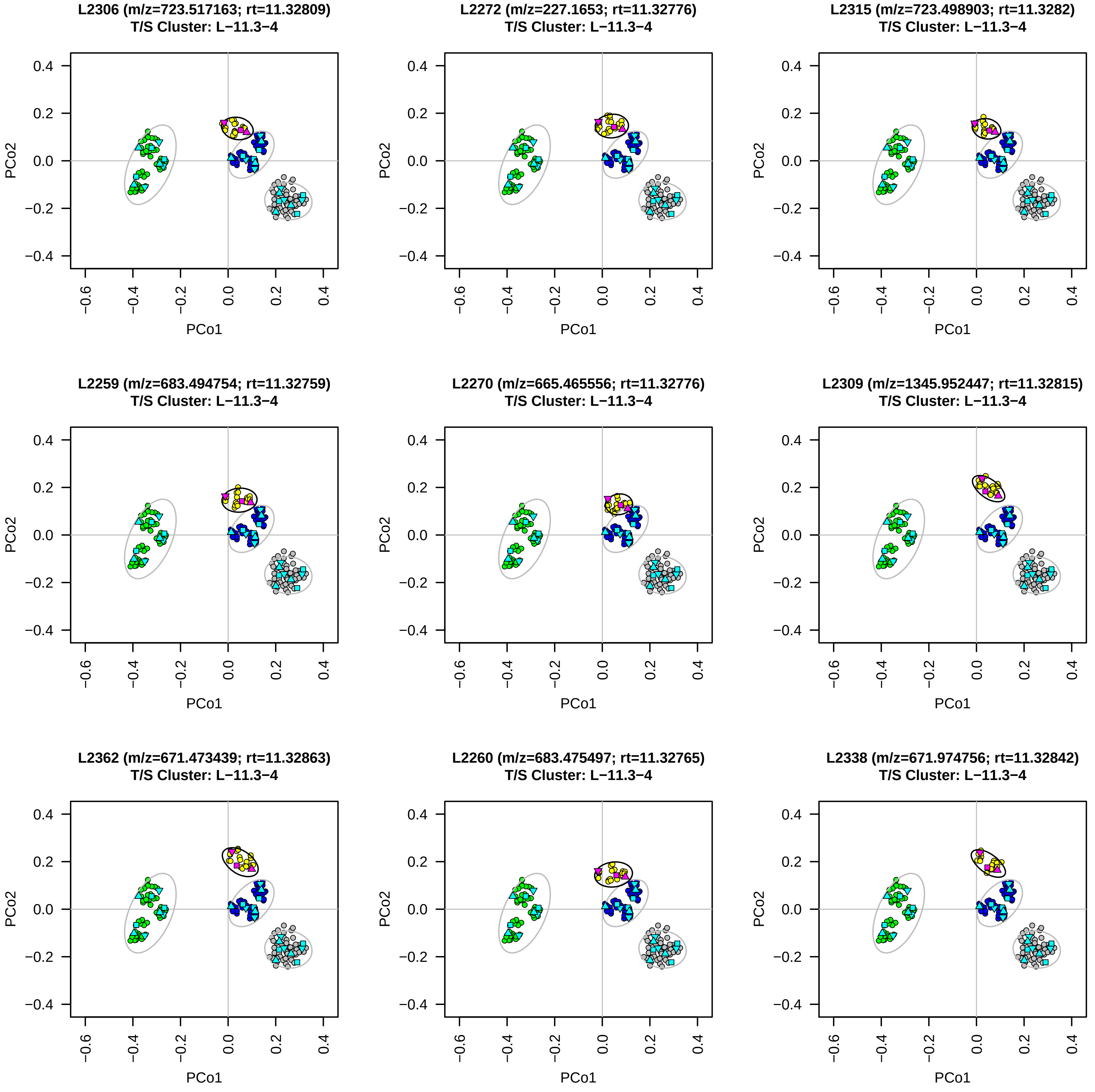
L2306 (m/z=723.517163; rt=11.32809)
T/S Cluster: L−11.3−4
L2272 (m/z=227.1653; rt=11.32776)
T/S Cluster: L−11.3−4
L2315 (m/z=723.498903; rt=11.3282)
T/S Cluster: L−11.3−4
L2259 (m/z=683.494754; rt=11.32759)
T/S Cluster: L−11.3−4
L2270 (m/z=665.465556; rt=11.32776)
T/S Cluster: L−11.3−4
L2309 (m/z=1345.952447; rt=11.32815)
T/S Cluster: L−11.3−4
L2362 (m/z=671.473439; rt=11.32863)
T/S Cluster: L−11.3−4
L2260 (m/z=683.475497; rt=11.32765)
T/S Cluster: L−11.3−4
L2338 (m/z=671.974756; rt=11.32842)
T/S Cluster: L−11.3−4
PCo1
PCo2
0.4
0.2
0.0
−0.2
−0.4
−0.6
−0.4
−0.2
0.0
0.2
0.4

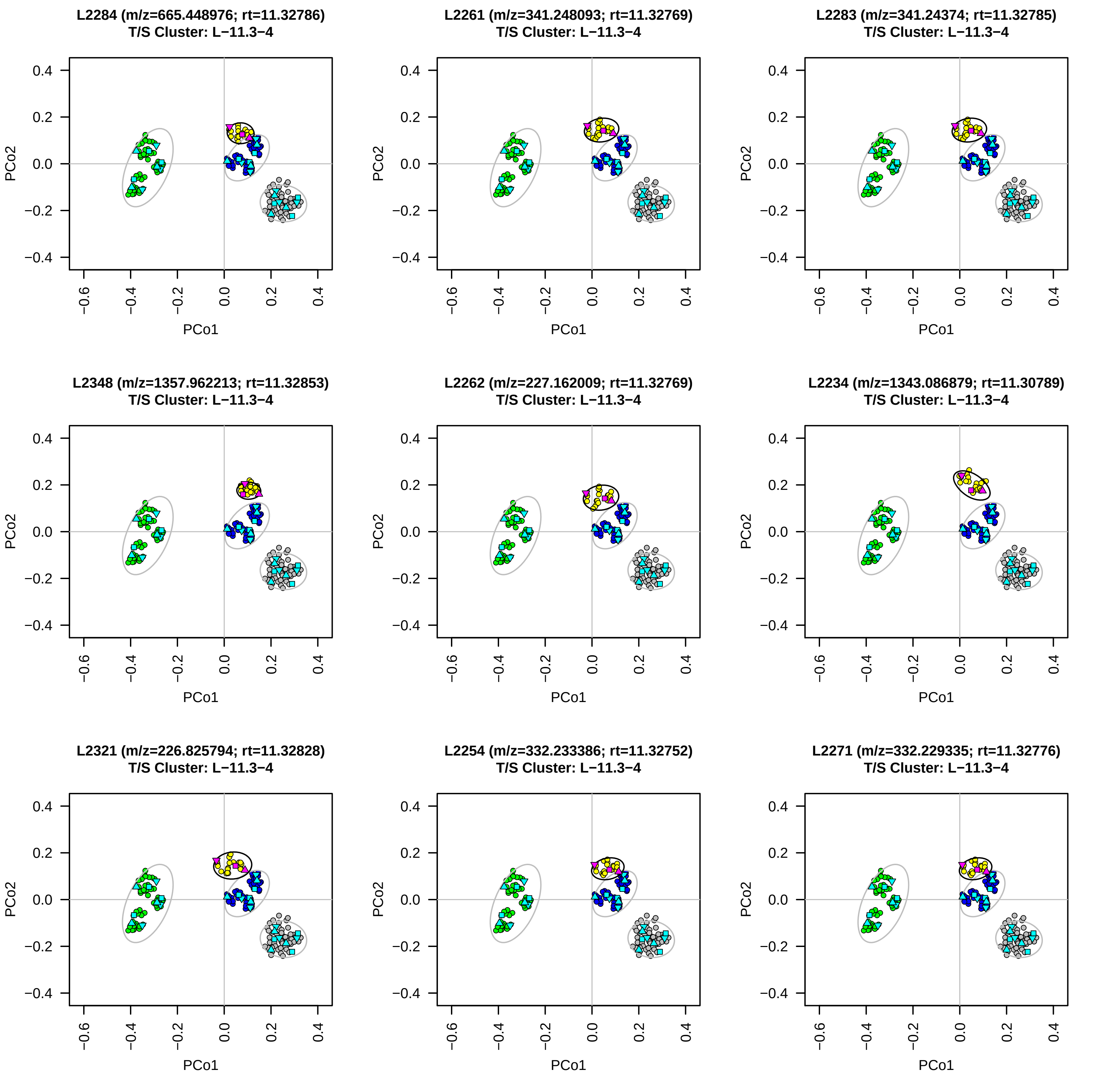
L2284 (m/z=665.448976; rt=11.32786)
T/S Cluster: L−11.3−4
L2261 (m/z=341.248093; rt=11.32769)
T/S Cluster: L−11.3−4
L2283 (m/z=341.24374; rt=11.32785)
T/S Cluster: L−11.3−4
L2348 (m/z=1357.962213; rt=11.32853)
T/S Cluster: L−11.3−4
L2262 (m/z=227.162009; rt=11.32769)
T/S Cluster: L−11.3−4
L2234 (m/z=1343.086879; rt=11.30789)
T/S Cluster: L−11.3−4
L2321 (m/z=226.825794; rt=11.32828)
T/S Cluster: L−11.3−4
L2254 (m/z=332.233386; rt=11.32752)
T/S Cluster: L−11.3−4
L2271 (m/z=332.229335; rt=11.32776)
T/S Cluster: L−11.3−4
PCo1
PCo2
0.4
0.2
0.0
−0.2
−0.4
−0.6

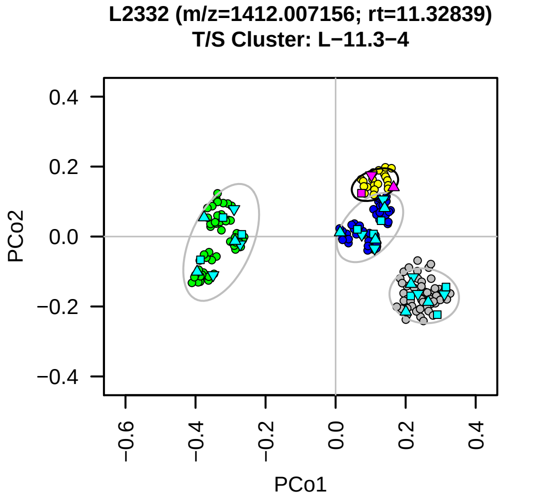
L2332 (m/z=1412.007156; rt=11.32839)
T/S Cluster: L−11.3−4
PCo1
PCo2

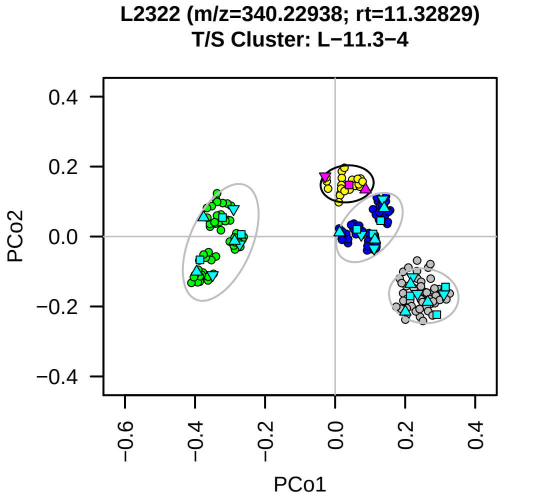
L2322 (m/z=340.22938; rt=11.32829)
T/S Cluster: L−11.3−4
PCo1
PCo2

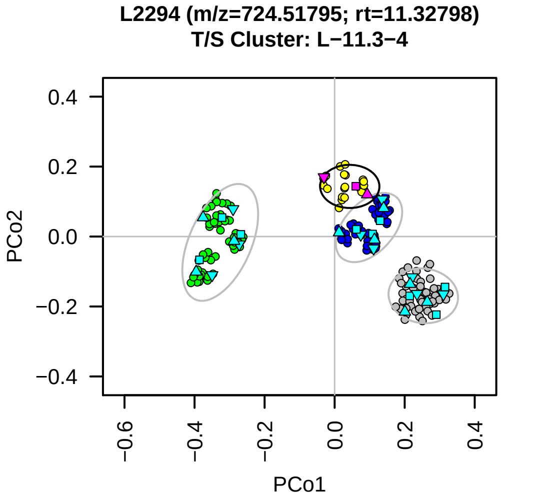
L2294 (m/z=724.51795; rt=11.32798)
T/S Cluster: L−11.3−4
PCo1
PCo2

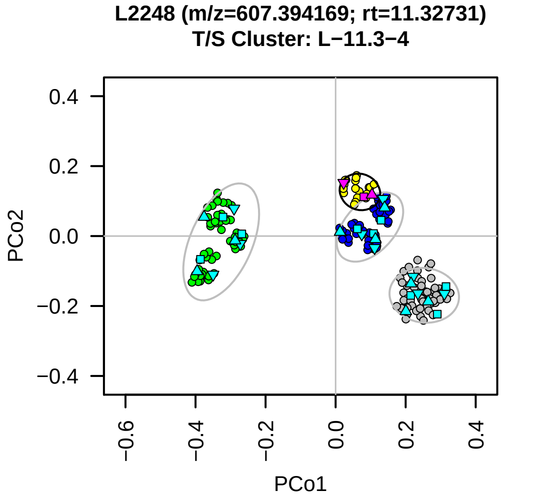
L2248 (m/z=607.394169; rt=11.32731)
T/S Cluster: L−11.3−4
PCo1
PCo2

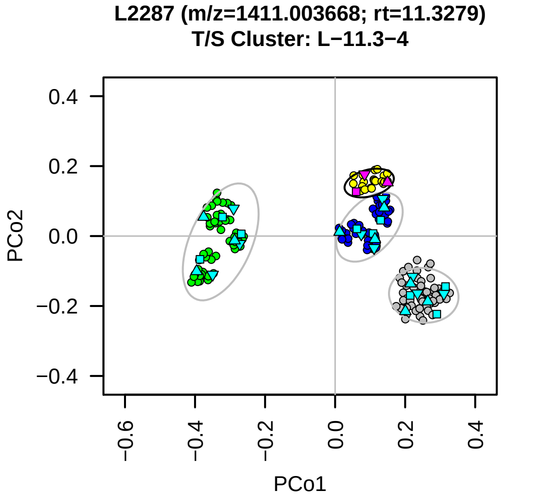
L2287 (m/z=1411.003668; rt=11.3279)
T/S Cluster: L−11.3−4
PCo1
PCo2

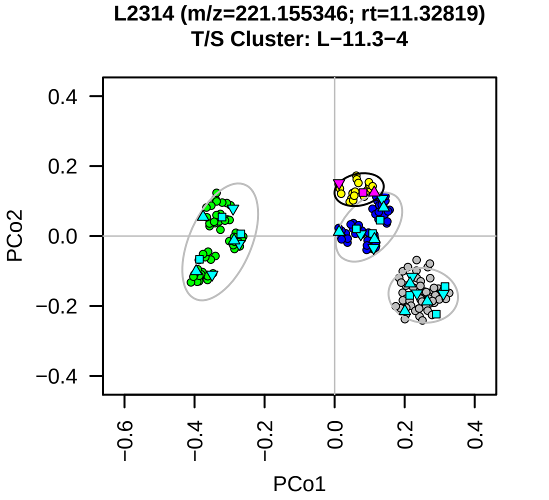
L2314 (m/z=221.155346; rt=11.32819)
T/S Cluster: L−11.3−4
PCo1
PCo2

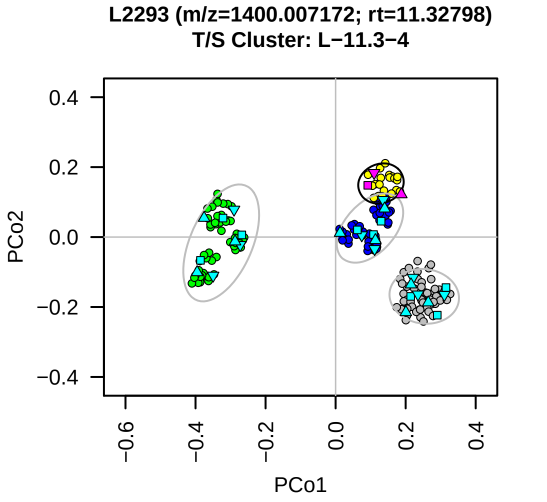
L2293 (m/z=1400.007172; rt=11.32798)
T/S Cluster: L−11.3−4
PCo1
PCo2

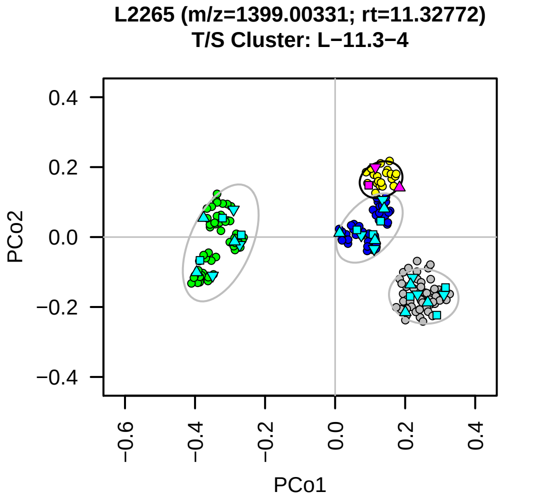
L2265 (m/z=1399.00331; rt=11.32772)
T/S Cluster: L−11.3−4
PCo1
PCo2

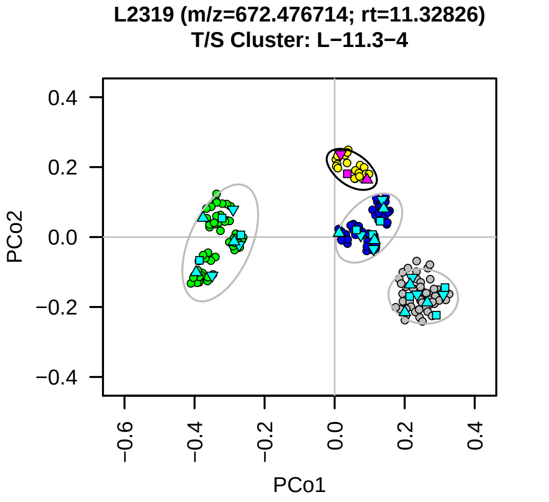
L2319 (m/z=672.476714; rt=11.32826)
T/S Cluster: L−11.3−4
PCo1
PCo2

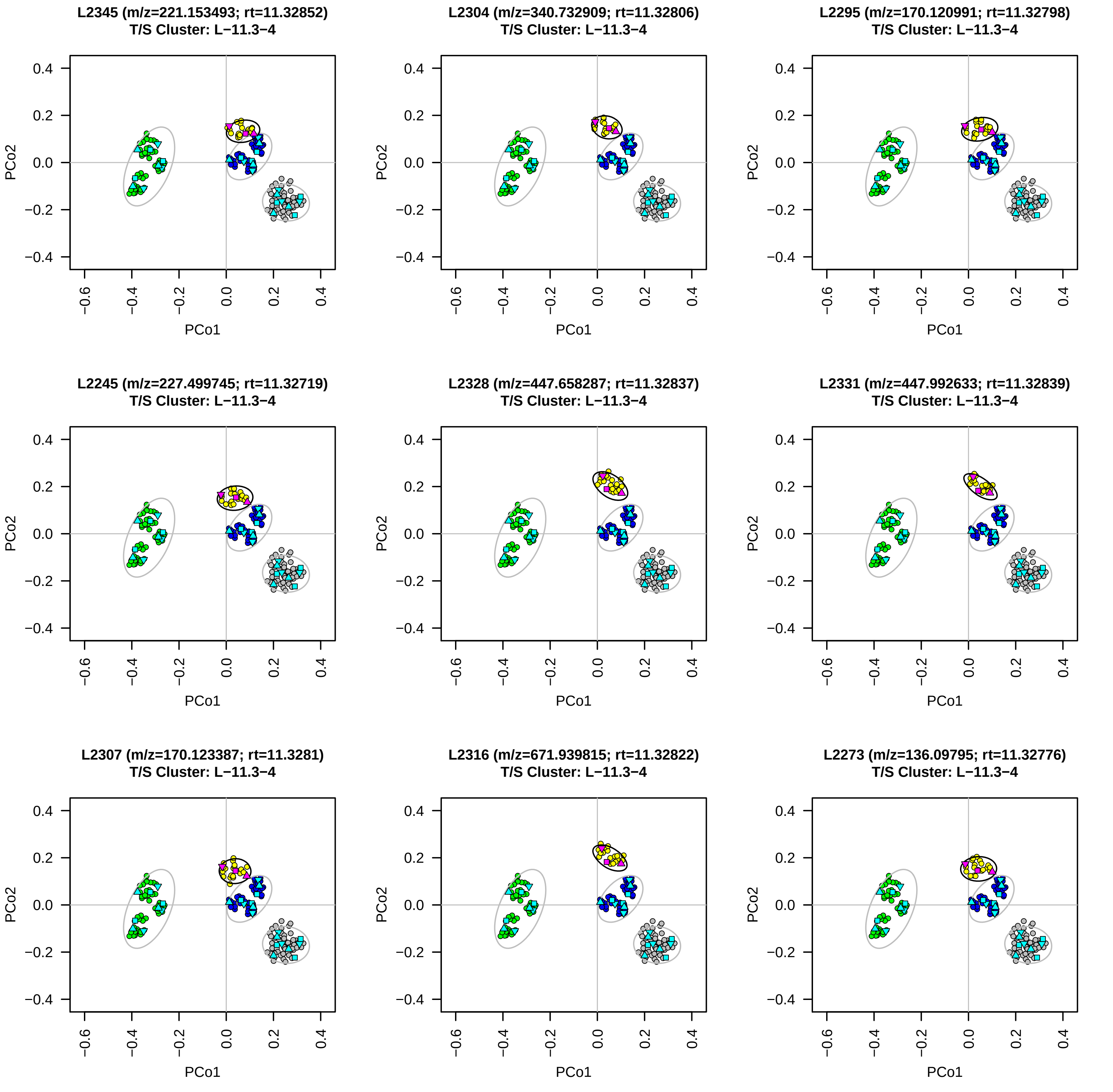
L2345 (m/z=221.153493; rt=11.32852)
T/S Cluster: L−11.3−4
L2304 (m/z=340.732909; rt=11.32806)
T/S Cluster: L−11.3−4
L2295 (m/z=170.120991; rt=11.32798)
T/S Cluster: L−11.3−4
L2245 (m/z=227.499745; rt=11.32719)
T/S Cluster: L−11.3−4
L2328 (m/z=447.658287; rt=11.32837)
T/S Cluster: L−11.3−4
L2331 (m/z=447.992633; rt=11.32839)
T/S Cluster: L−11.3−4
L2307 (m/z=170.123387; rt=11.3281)
T/S Cluster: L−11.3−4
L2316 (m/z=671.939815; rt=11.32822)
T/S Cluster: L−11.3−4
L2273 (m/z=136.09795; rt=11.32776)
T/S Cluster: L−11.3−4
PCo1
PCo2

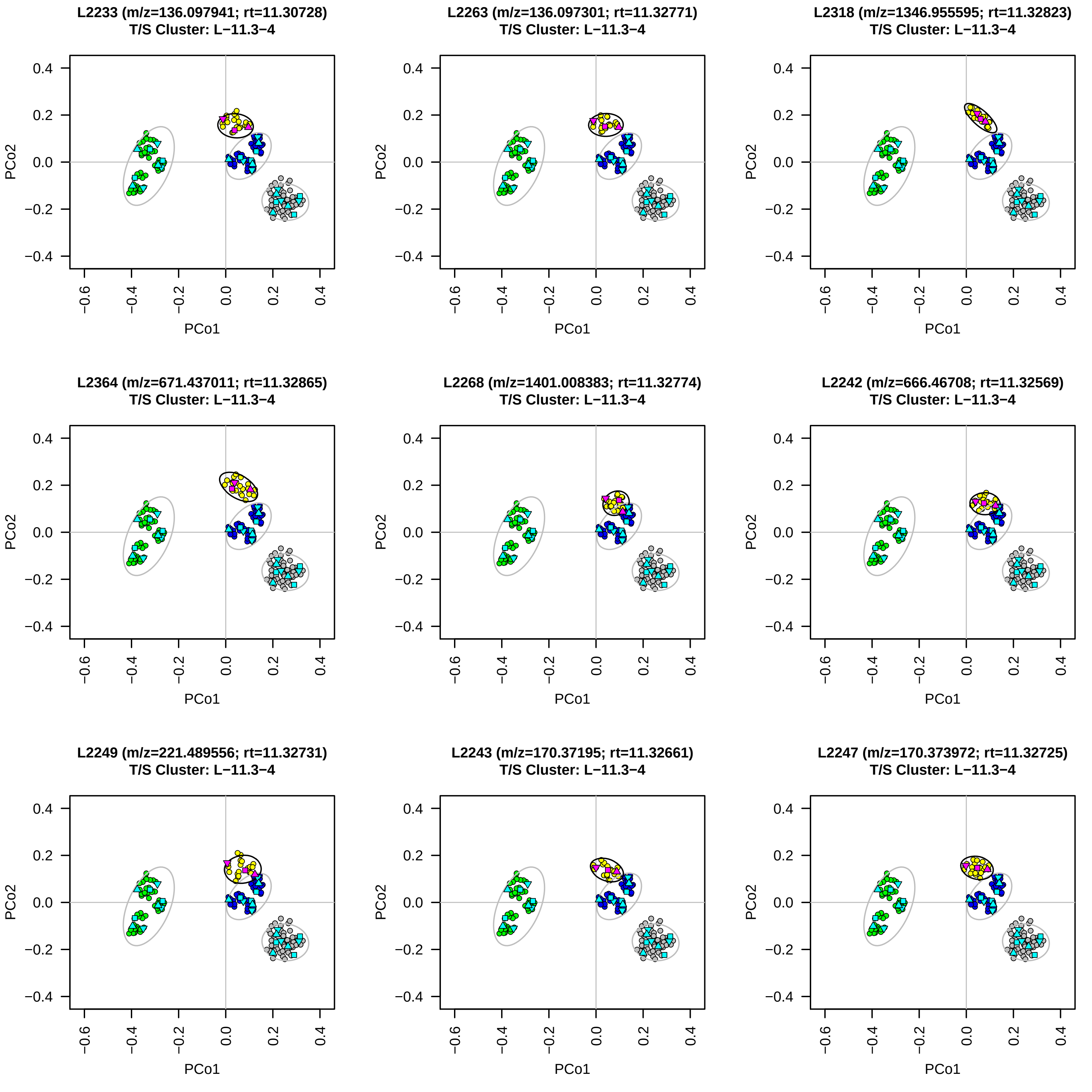

L2233 (m/z=136.097941; rt=11.30728)
T/S Cluster: L−11.3−4
L2263 (m/z=136.097301; rt=11.32771)
T/S Cluster: L−11.3−4
L2318 (m/z=1346.955595; rt=11.32823)
T/S Cluster: L−11.3−4
L2364 (m/z=671.437011; rt=11.32865)
T/S Cluster: L−11.3−4
L2268 (m/z=1401.008383; rt=11.32774)
T/S Cluster: L−11.3−4
L2242 (m/z=666.46708; rt=11.32569)
T/S Cluster: L−11.3−4
L2249 (m/z=221.489556; rt=11.32731)
T/S Cluster: L−11.3−4
L2243 (m/z=170.37195; rt=11.32661)
T/S Cluster: L−11.3−4
L2247 (m/z=170.373972; rt=11.32725)
T/S Cluster: L−11.3−4
PCo1
PCo2
−0.6
−0.4
−0.2
0.0
0.2
0.4

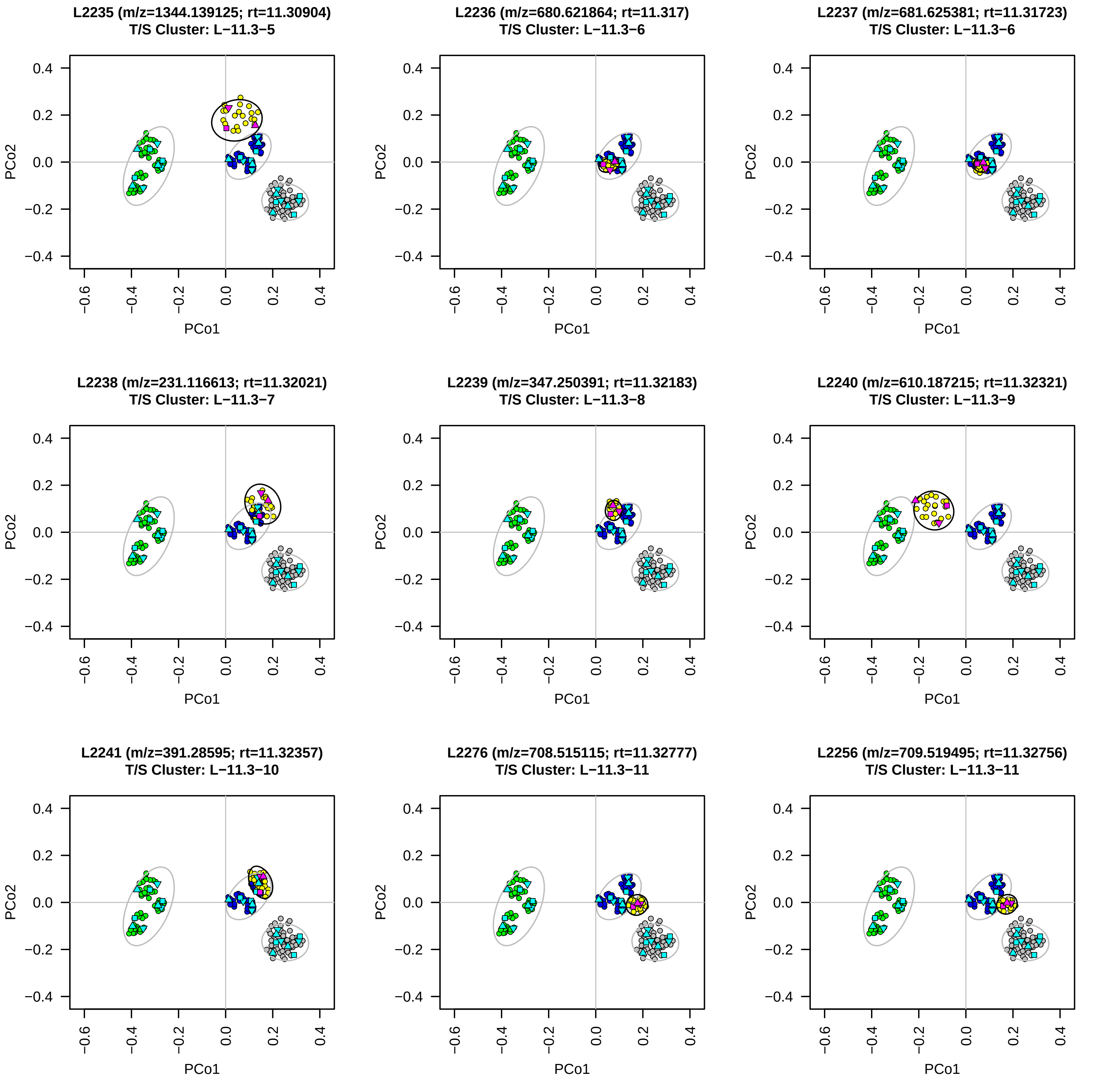
L2235 (m/z=1344.139125; rt=11.30904)
T/S Cluster: L−11.3−5
L2236 (m/z=680.621864; rt=11.317)
T/S Cluster: L−11.3−6
L2237 (m/z=681.625381; rt=11.31723)
T/S Cluster: L−11.3−6
L2238 (m/z=231.116613; rt=11.32021)
T/S Cluster: L−11.3−7
L2239 (m/z=347.250391; rt=11.32183)
T/S Cluster: L−11.3−8
L2240 (m/z=610.187215; rt=11.32321)
T/S Cluster: L−11.3−9
L2241 (m/z=391.28595; rt=11.32357)
T/S Cluster: L−11.3−10
L2276 (m/z=708.515115; rt=11.32777)
T/S Cluster: L−11.3−11
L2256 (m/z=709.519495; rt=11.32756)
T/S Cluster: L−11.3−11
PCo1
PCo2
−0.6
−0.4
−0.2
0.0
0.2
0.4

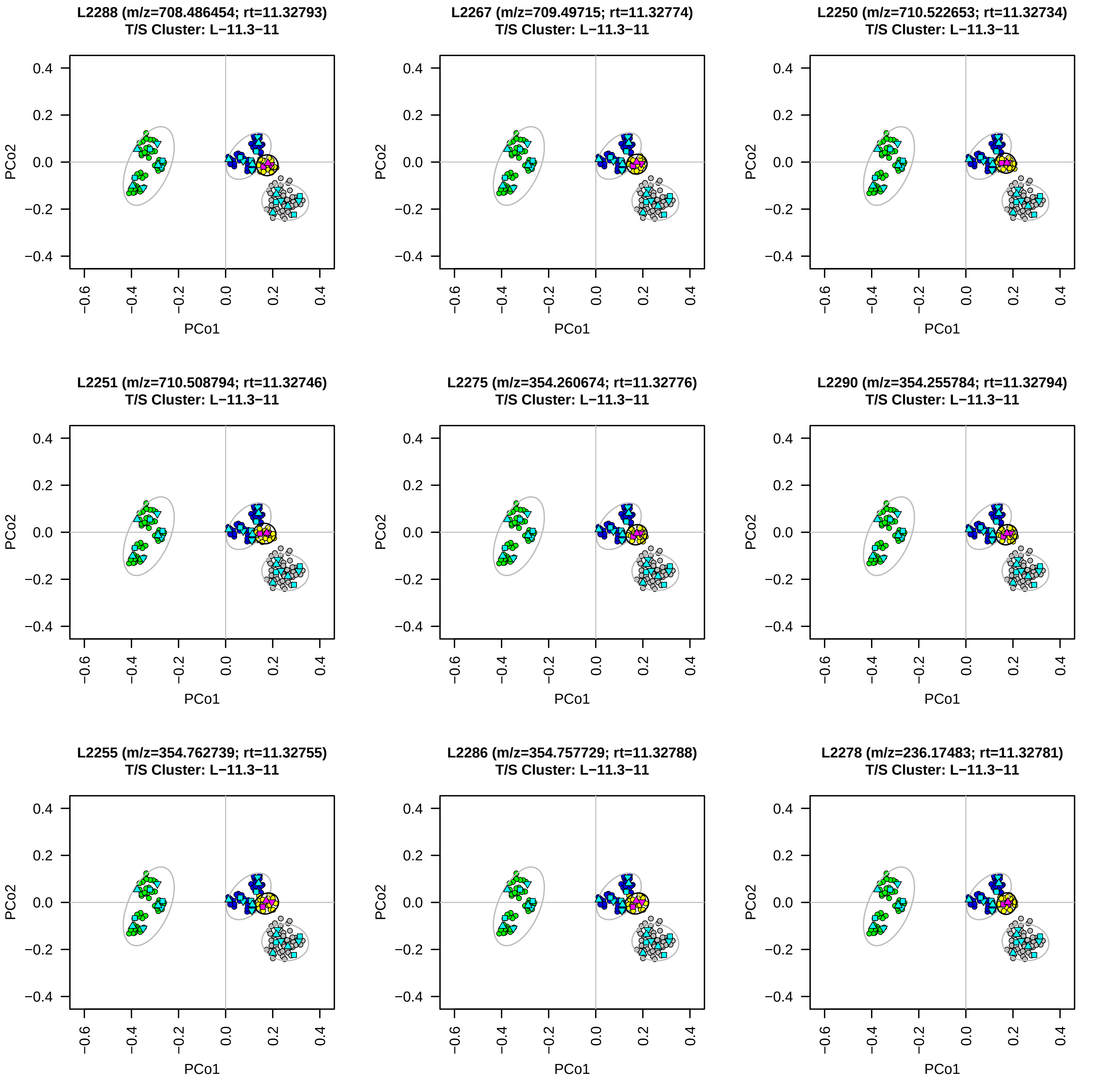
L2288 (m/z=708.486454; rt=11.32793)
T/S Cluster: L−11.3−11
L2267 (m/z=709.49715; rt=11.32774)
T/S Cluster: L−11.3−11
L2250 (m/z=710.522653; rt=11.32734)
T/S Cluster: L−11.3−11
L2251 (m/z=710.508794; rt=11.32746)
T/S Cluster: L−11.3−11
L2275 (m/z=354.260674; rt=11.32776)
T/S Cluster: L−11.3−11
L2290 (m/z=354.255784; rt=11.32794)
T/S Cluster: L−11.3−11
L2255 (m/z=354.762739; rt=11.32755)
T/S Cluster: L−11.3−11
L2286 (m/z=354.757729; rt=11.32788)
T/S Cluster: L−11.3−11
L2278 (m/z=236.17483; rt=11.32781)
T/S Cluster: L−11.3−11
PCo1
PCo2
−0.6
−0.4
−0.2
0.0
0.2
0.4

L2310 (m/z=236.171988; rt=11.32815)
T/S Cluster: L−11.3−11

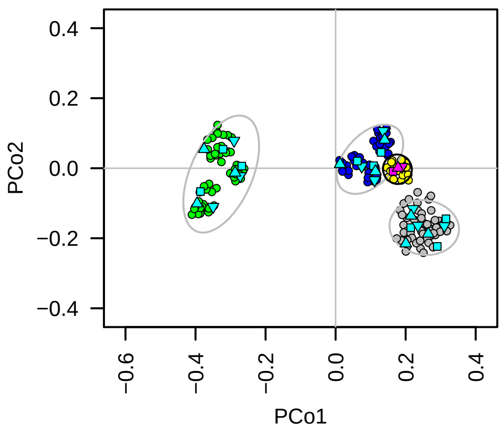

L2258 (m/z=236.50934; rt=11.32756)
T/S Cluster: L−11.3−11

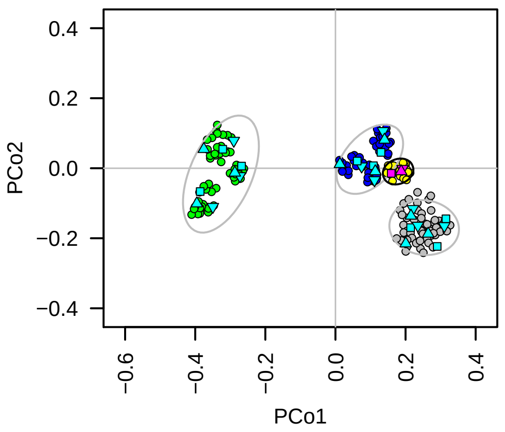

L2246 (m/z=711.524857; rt=11.32724)
T/S Cluster: L−11.3−11

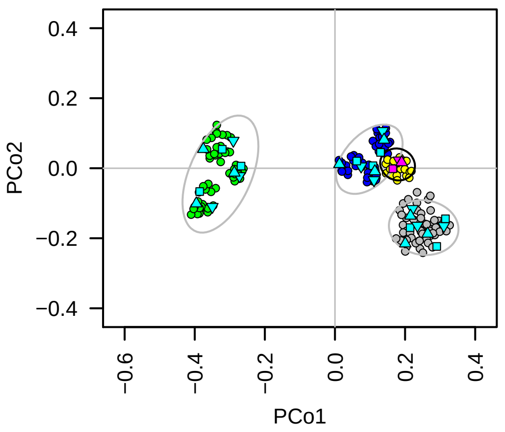

L2244 (m/z=355.261902; rt=11.32663)
T/S Cluster: L−11.3−11

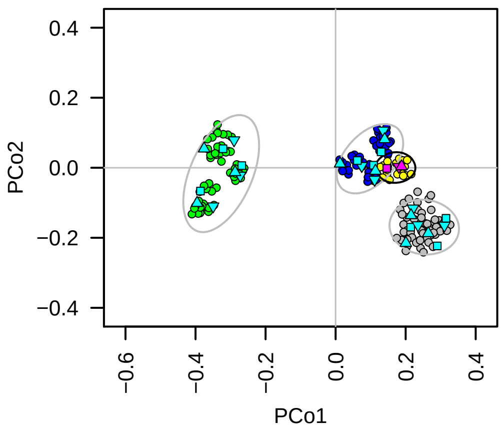

L2252 (m/z=737.549495; rt=11.32747)
T/S Cluster: L−11.3−12

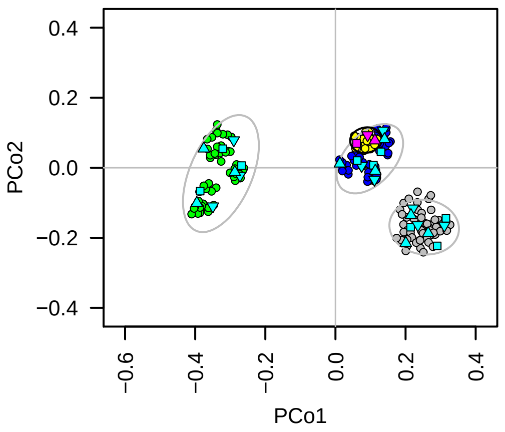

L2257 (m/z=751.528498; rt=11.32756)
T/S Cluster: L−11.3−13

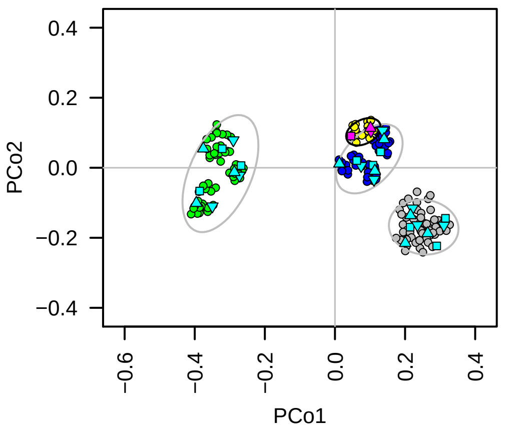

L2300 (m/z=694.489188; rt=11.328)
T/S Cluster: L−11.3−14

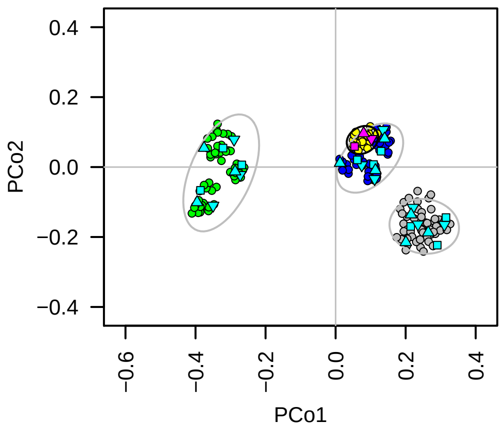

L2269 (m/z=694.504541; rt=11.32775)
T/S Cluster: L−11.3−14

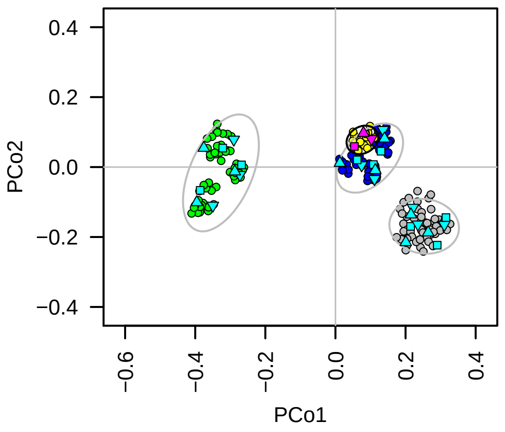

L2375 (m/z=685.439087; rt=11.32886)
T/S Cluster: L−11.3−15

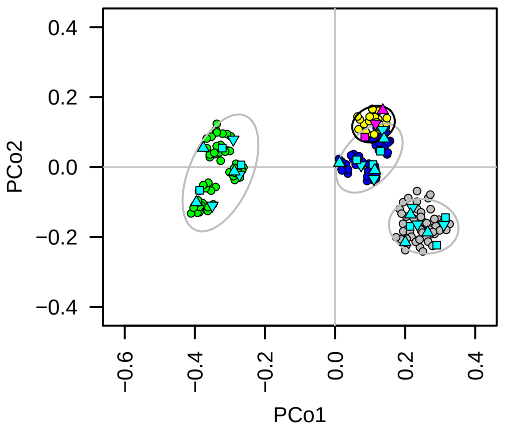

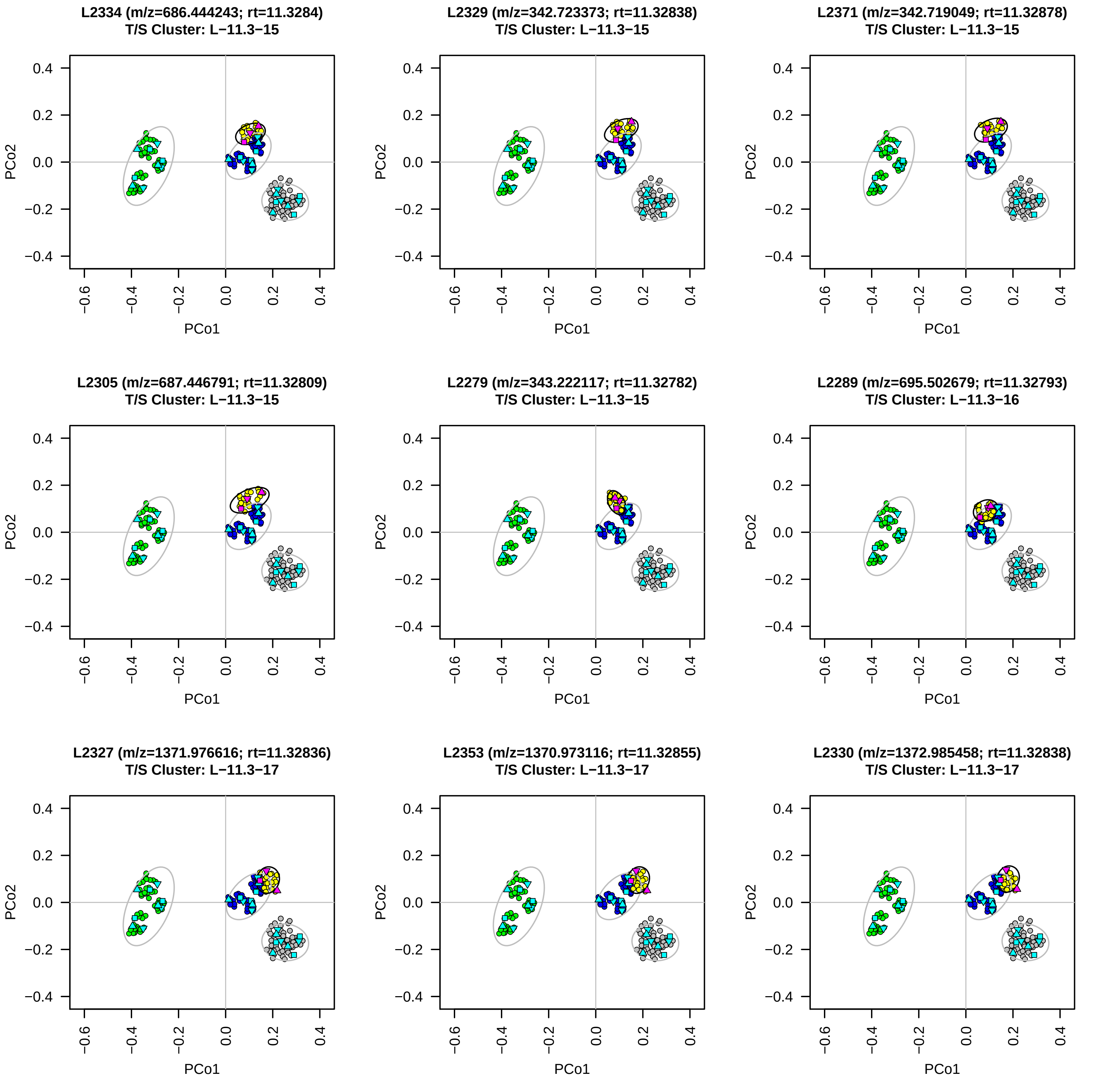
L2334 (m/z=686.444243; rt=11.3284)
T/S Cluster: L−11.3−15
L2329 (m/z=342.723373; rt=11.32838)
T/S Cluster: L−11.3−15
L2371 (m/z=342.719049; rt=11.32878)
T/S Cluster: L−11.3−15
L2305 (m/z=687.446791; rt=11.32809)
T/S Cluster: L−11.3−15
L2279 (m/z=343.222117; rt=11.32782)
T/S Cluster: L−11.3−15
L2289 (m/z=695.502679; rt=11.32793)
T/S Cluster: L−11.3−16
L2327 (m/z=1371.976616; rt=11.32836)
T/S Cluster: L−11.3−17
L2353 (m/z=1370.973116; rt=11.32855)
T/S Cluster: L−11.3−17
L2330 (m/z=1372.985458; rt=11.32838)
T/S Cluster: L−11.3−17
PCo1
PCo2
−0.6
−0.4
−0.2
0.0
0.2
0.4

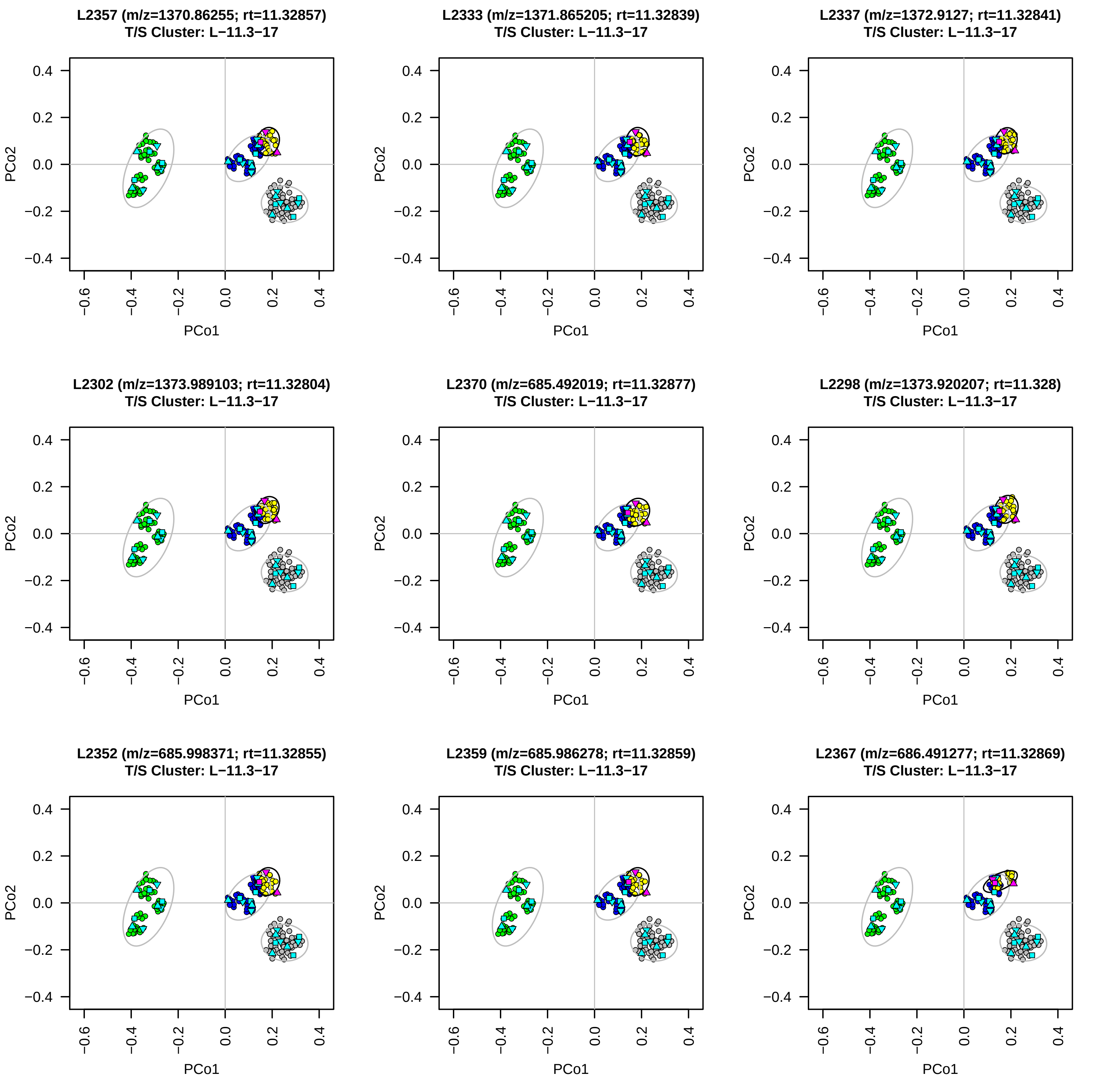
L2357 (m/z=1370.86255; rt=11.32857)
T/S Cluster: L−11.3−17
L2333 (m/z=1371.865205; rt=11.32839)
T/S Cluster: L−11.3−17
L2337 (m/z=1372.9127; rt=11.32841)
T/S Cluster: L−11.3−17
L2302 (m/z=1373.989103; rt=11.32804)
T/S Cluster: L−11.3−17
L2370 (m/z=685.492019; rt=11.32877)
T/S Cluster: L−11.3−17
L2298 (m/z=1373.920207; rt=11.328)
T/S Cluster: L−11.3−17
L2352 (m/z=685.998371; rt=11.32855)
T/S Cluster: L−11.3−17
L2359 (m/z=685.986278; rt=11.32859)
T/S Cluster: L−11.3−17
L2367 (m/z=686.491277; rt=11.32869)
T/S Cluster: L−11.3−17
PCo1
PCo2
0.4
0.2
0.0
−0.2
−0.4
−0.6

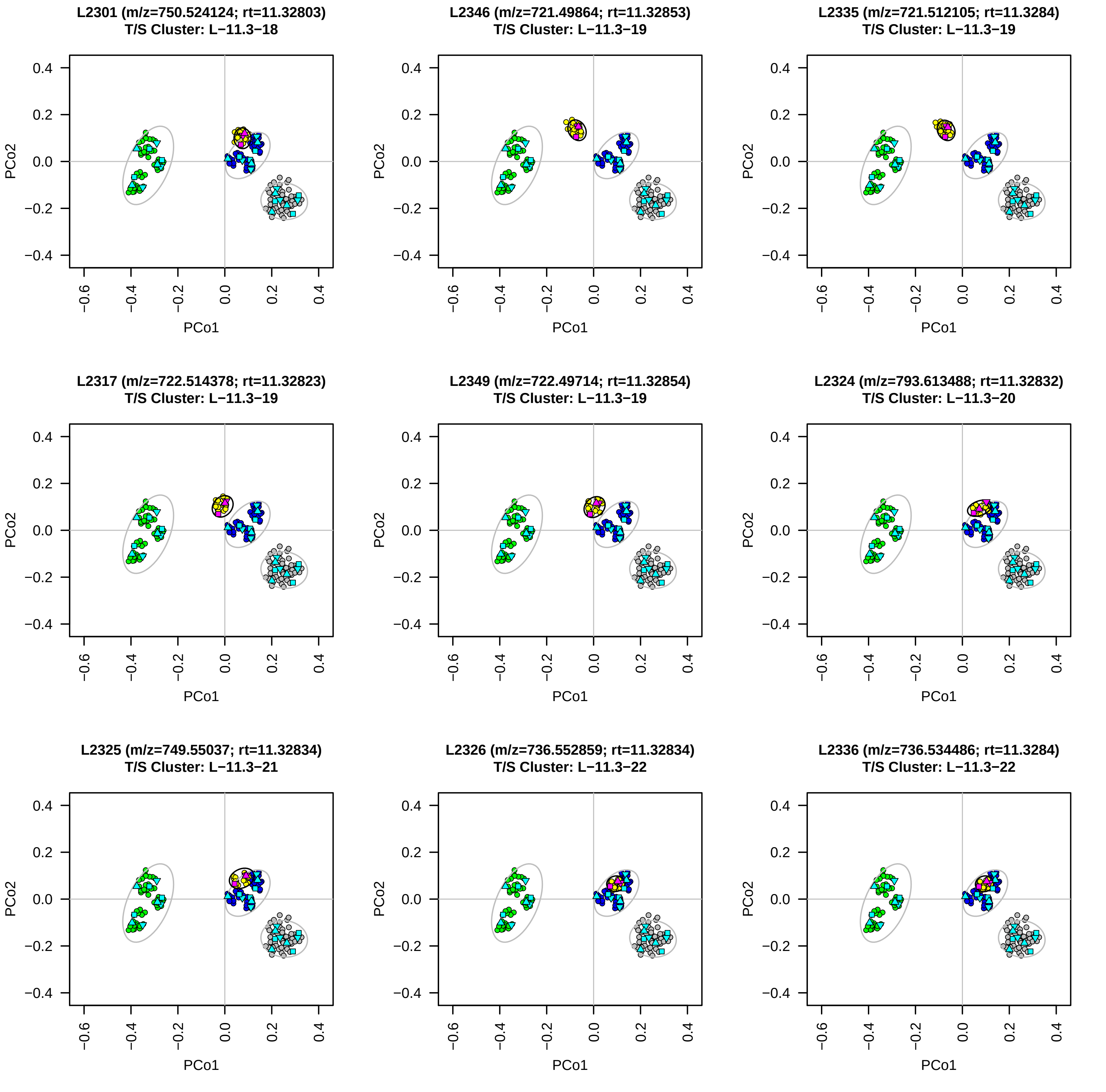
L2301 (m/z=750.524124; rt=11.32803)
T/S Cluster: L−11.3−18
L2346 (m/z=721.49864; rt=11.32853)
T/S Cluster: L−11.3−19
L2335 (m/z=721.512105; rt=11.3284)
T/S Cluster: L−11.3−19
L2317 (m/z=722.514378; rt=11.32823)
T/S Cluster: L−11.3−19
L2349 (m/z=722.49714; rt=11.32854)
T/S Cluster: L−11.3−19
L2324 (m/z=793.613488; rt=11.32832)
T/S Cluster: L−11.3−20
L2325 (m/z=749.55037; rt=11.32834)
T/S Cluster: L−11.3−21
L2326 (m/z=736.552859; rt=11.32834)
T/S Cluster: L−11.3−22
L2336 (m/z=736.534486; rt=11.3284)
T/S Cluster: L−11.3−22
PCo1
PCo2
−0.6
−0.4
−0.2
0.0
0.2
0.4

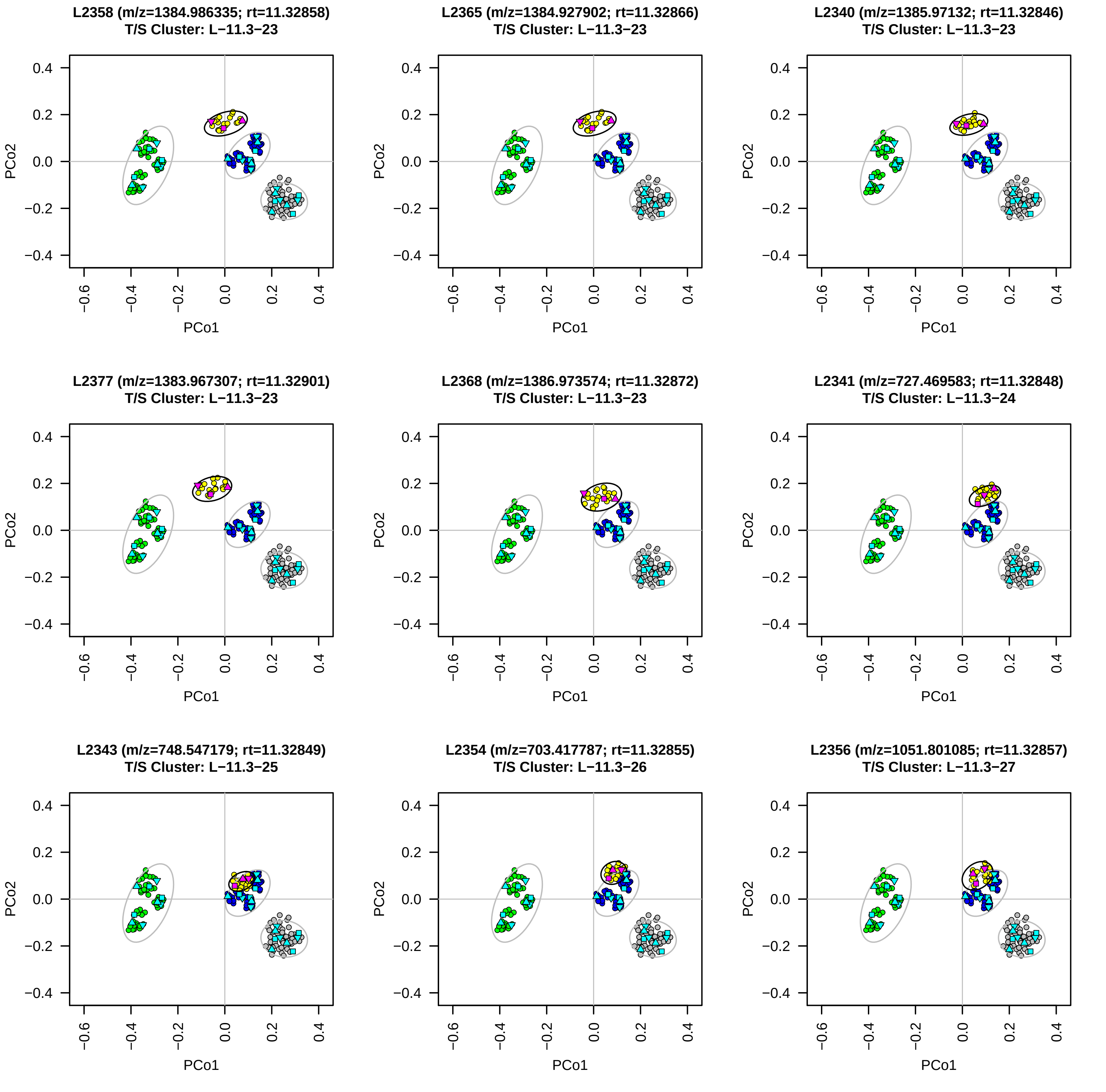
L2358 (m/z=1384.986335; rt=11.32858)
T/S Cluster: L−11.3−23
L2365 (m/z=1384.927902; rt=11.32866)
T/S Cluster: L−11.3−23
L2340 (m/z=1385.97132; rt=11.32846)
T/S Cluster: L−11.3−23
L2377 (m/z=1383.967307; rt=11.32901)
T/S Cluster: L−11.3−23
L2368 (m/z=1386.973574; rt=11.32872)
T/S Cluster: L−11.3−23
L2341 (m/z=727.469583; rt=11.32848)
T/S Cluster: L−11.3−24
L2343 (m/z=748.547179; rt=11.32849)
T/S Cluster: L−11.3−25
L2354 (m/z=703.417787; rt=11.32855)
T/S Cluster: L−11.3−26
L2356 (m/z=1051.801085; rt=11.32857)
T/S Cluster: L−11.3−27
PCo1
PCo2
−0.6
−0.4
−0.2
0.0
0.2
0.4

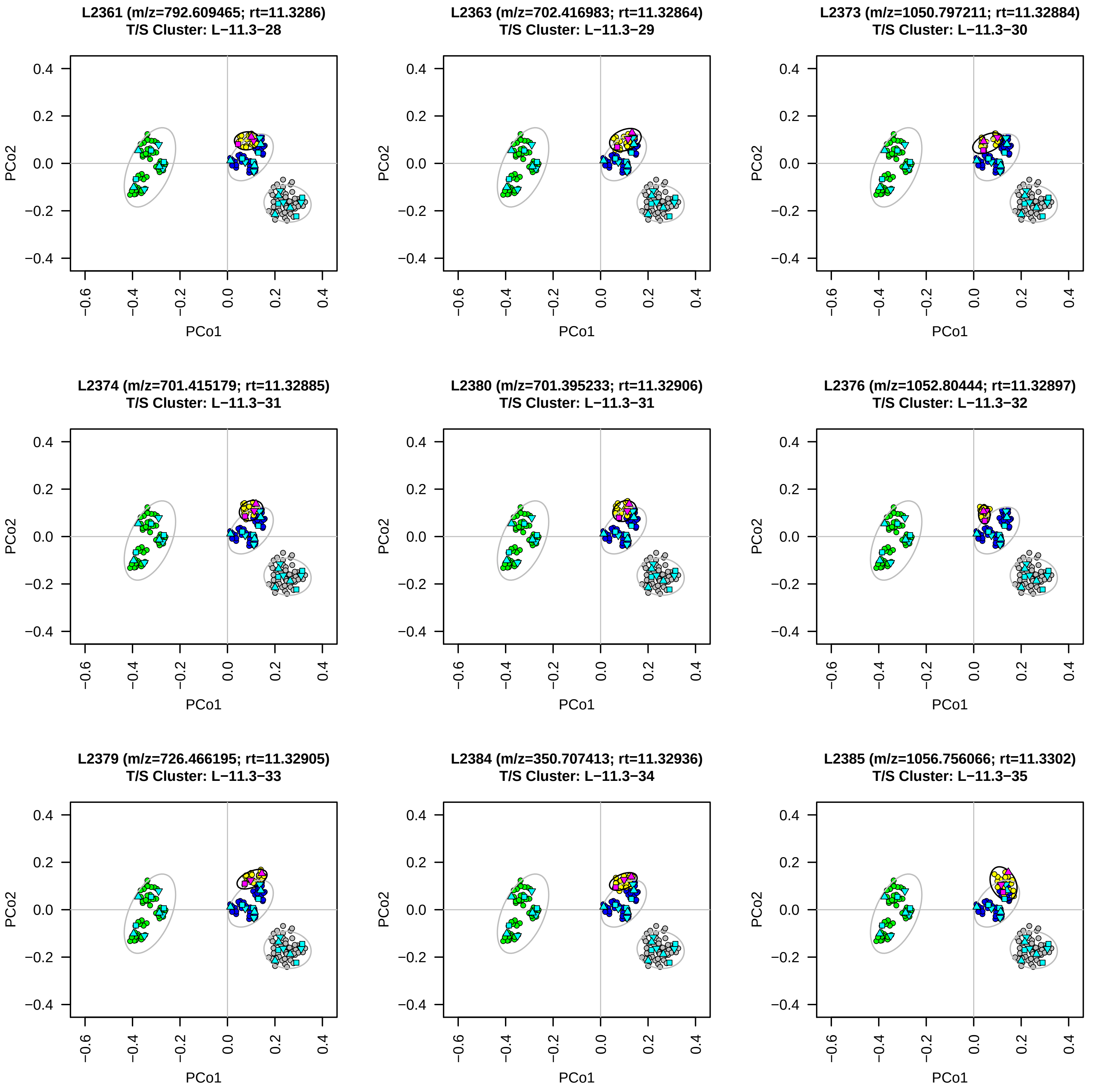
L2361 (m/z=792.609465; rt=11.3286)
T/S Cluster: L−11.3−28
L2363 (m/z=702.416983; rt=11.32864)
T/S Cluster: L−11.3−29
L2373 (m/z=1050.797211; rt=11.32884)
T/S Cluster: L−11.3−30
L2374 (m/z=701.415179; rt=11.32885)
T/S Cluster: L−11.3−31
L2380 (m/z=701.395233; rt=11.32906)
T/S Cluster: L−11.3−31
L2376 (m/z=1052.80444; rt=11.32897)
T/S Cluster: L−11.3−32
L2379 (m/z=726.466195; rt=11.32905)
T/S Cluster: L−11.3−33
L2384 (m/z=350.707413; rt=11.32936)
T/S Cluster: L−11.3−34
L2385 (m/z=1056.756066; rt=11.3302)
T/S Cluster: L−11.3−35
PCo1
PCo2

L2386 (m/z=1055.752726; rt=11.33073)
T/S Cluster: L−11.3−36

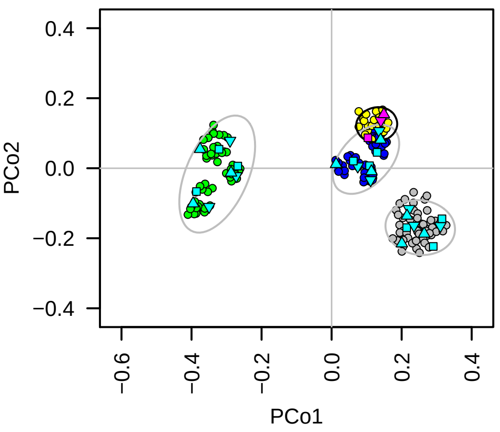

L2387 (m/z=1213.08701; rt=11.33755)
T/S Cluster: L−11.3−37

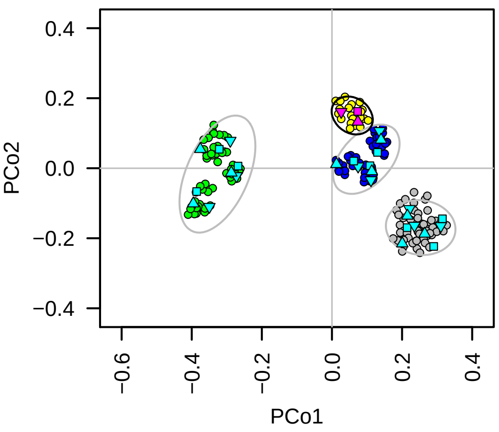

L2388 (m/z=1214.090706; rt=11.33923)
T/S Cluster: L−11.3−38

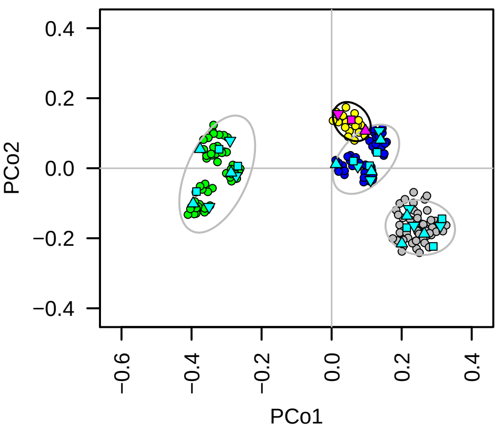

L2389 (m/z=550.632; rt=11.34431)
T/S Cluster: L−11.3−39

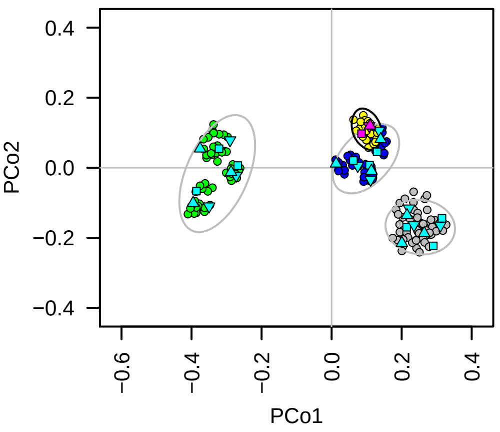

L2392 (m/z=550.619373; rt=11.34469)
T/S Cluster: L−11.3−39

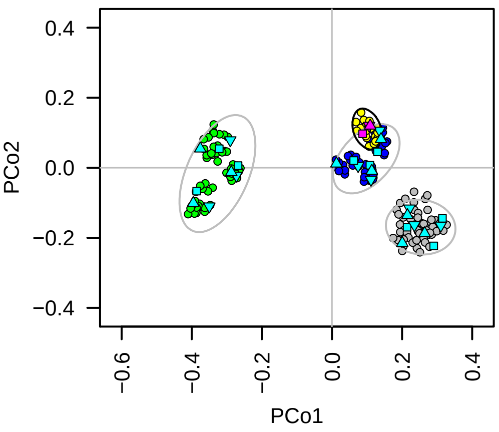

L2390 (m/z=551.636876; rt=11.34439)
T/S Cluster: L−11.3−39

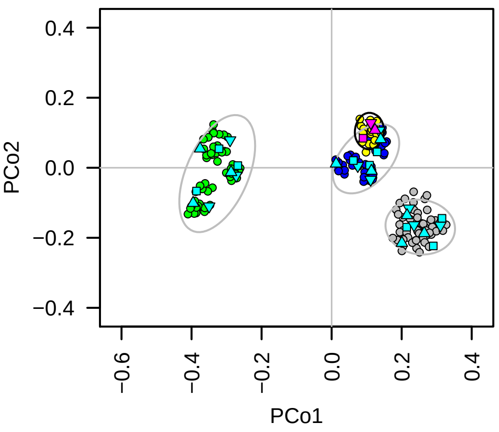

L2391 (m/z=551.626819; rt=11.34467)
T/S Cluster: L−11.3−39

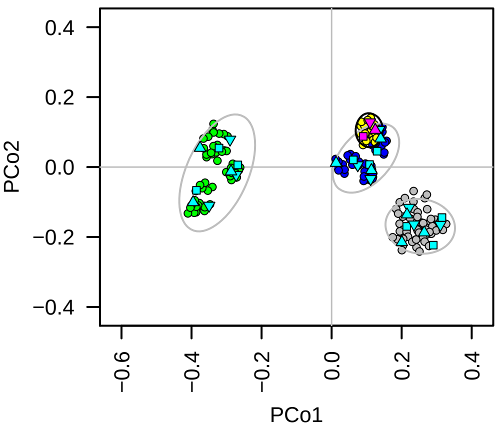

L2393 (m/z=275.315883; rt=11.3459)
T/S Cluster: L−11.3−39

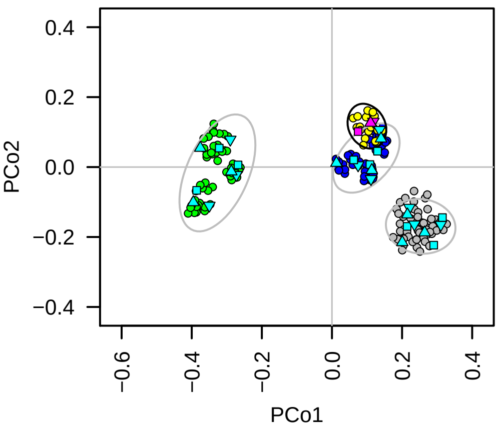

L2395 (m/z=869.551428; rt=11.37292)
T/S Cluster: L−11.4−1

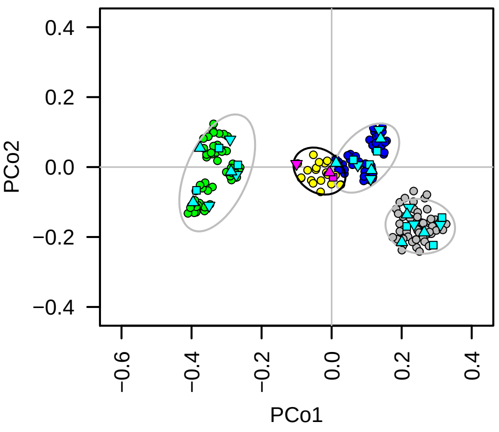

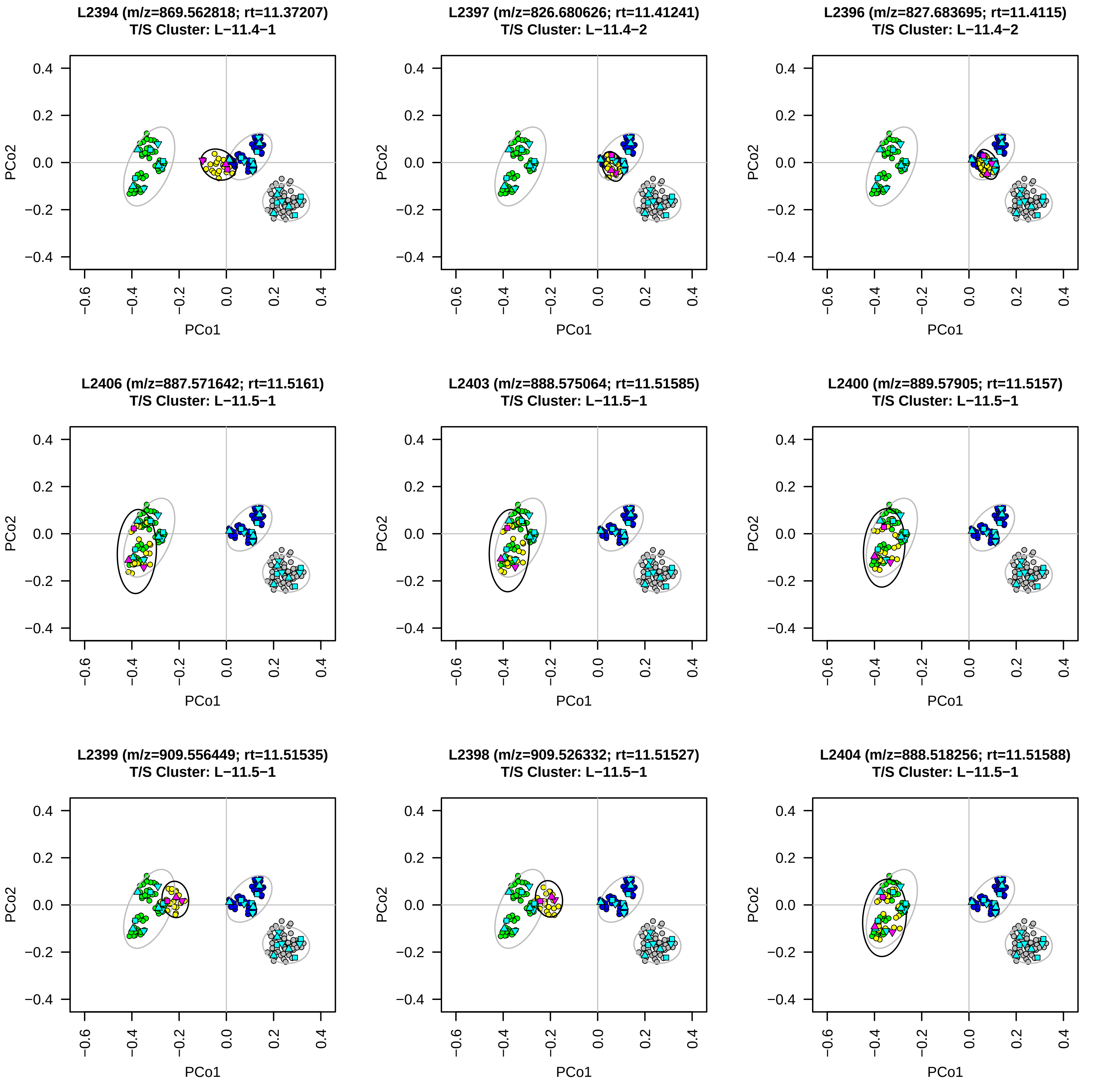
L2394 (m/z=869.562818; rt=11.37207)
T/S Cluster: L−11.4−1
L2397 (m/z=826.680626; rt=11.41241)
T/S Cluster: L−11.4−2
L2396 (m/z=827.683695; rt=11.4115)
T/S Cluster: L−11.4−2
L2406 (m/z=887.571642; rt=11.5161)
T/S Cluster: L−11.5−1
L2403 (m/z=888.575064; rt=11.51585)
T/S Cluster: L−11.5−1
L2400 (m/z=889.57905; rt=11.5157)
T/S Cluster: L−11.5−1
L2399 (m/z=909.556449; rt=11.51535)
T/S Cluster: L−11.5−1
L2398 (m/z=909.526332; rt=11.51527)
T/S Cluster: L−11.5−1
L2404 (m/z=888.518256; rt=11.51588)
T/S Cluster: L−11.5−1
PCo1
PCo2
0.4
0.2
0.0
−0.2
−0.4
−0.6

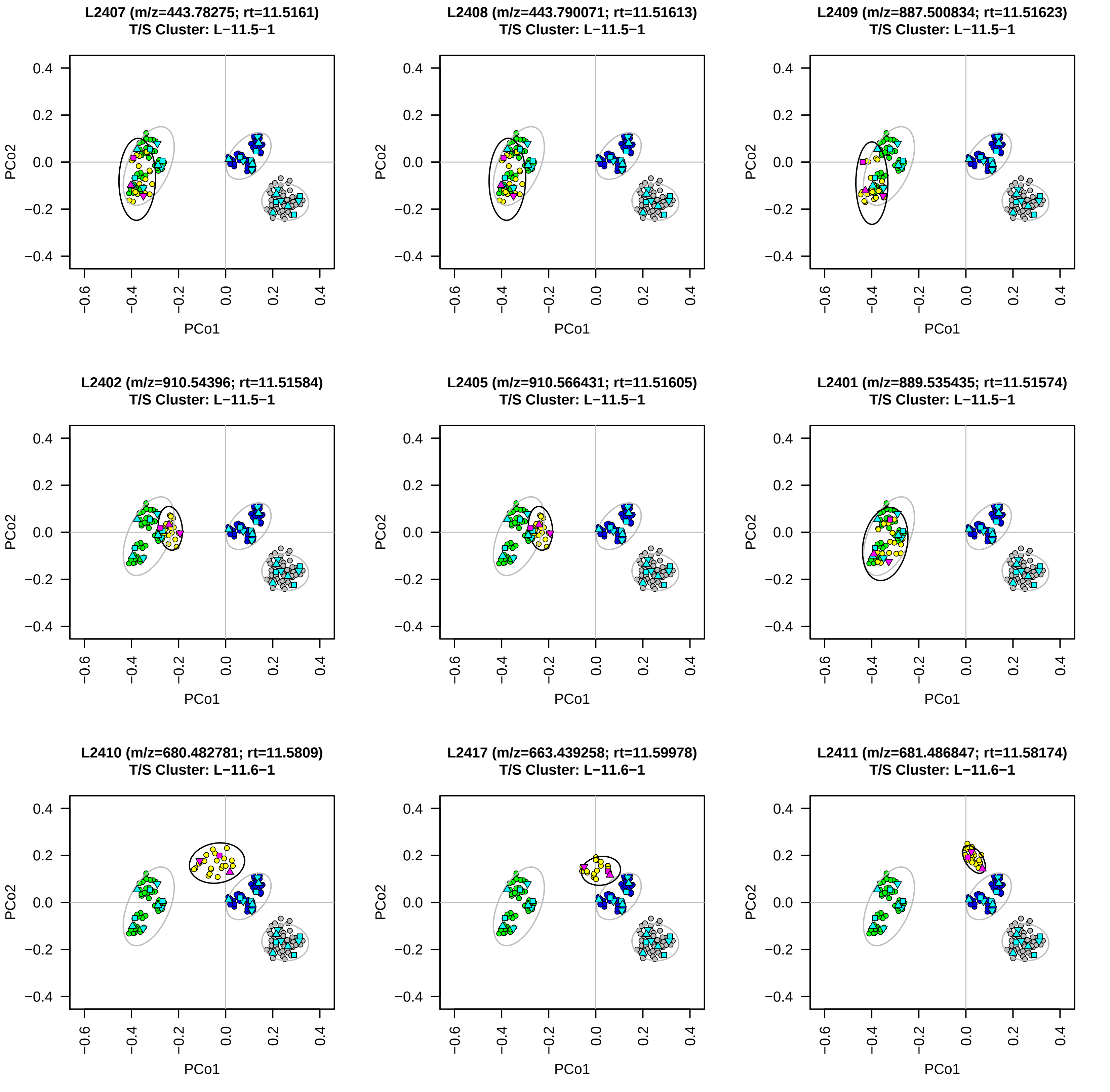

L2407 (m/z=443.78275; rt=11.5161)
T/S Cluster: L−11.5−1
L2408 (m/z=443.790071; rt=11.51613)
T/S Cluster: L−11.5−1
L2409 (m/z=887.500834; rt=11.51623)
T/S Cluster: L−11.5−1
L2402 (m/z=910.54396; rt=11.51584)
T/S Cluster: L−11.5−1
L2405 (m/z=910.566431; rt=11.51605)
T/S Cluster: L−11.5−1
L2401 (m/z=889.535435; rt=11.51574)
T/S Cluster: L−11.5−1
L2410 (m/z=680.482781; rt=11.5809)
T/S Cluster: L−11.6−1
L2417 (m/z=663.439258; rt=11.59978)
T/S Cluster: L−11.6−1
L2411 (m/z=681.486847; rt=11.58174)
T/S Cluster: L−11.6−1
PCo1
PCo2

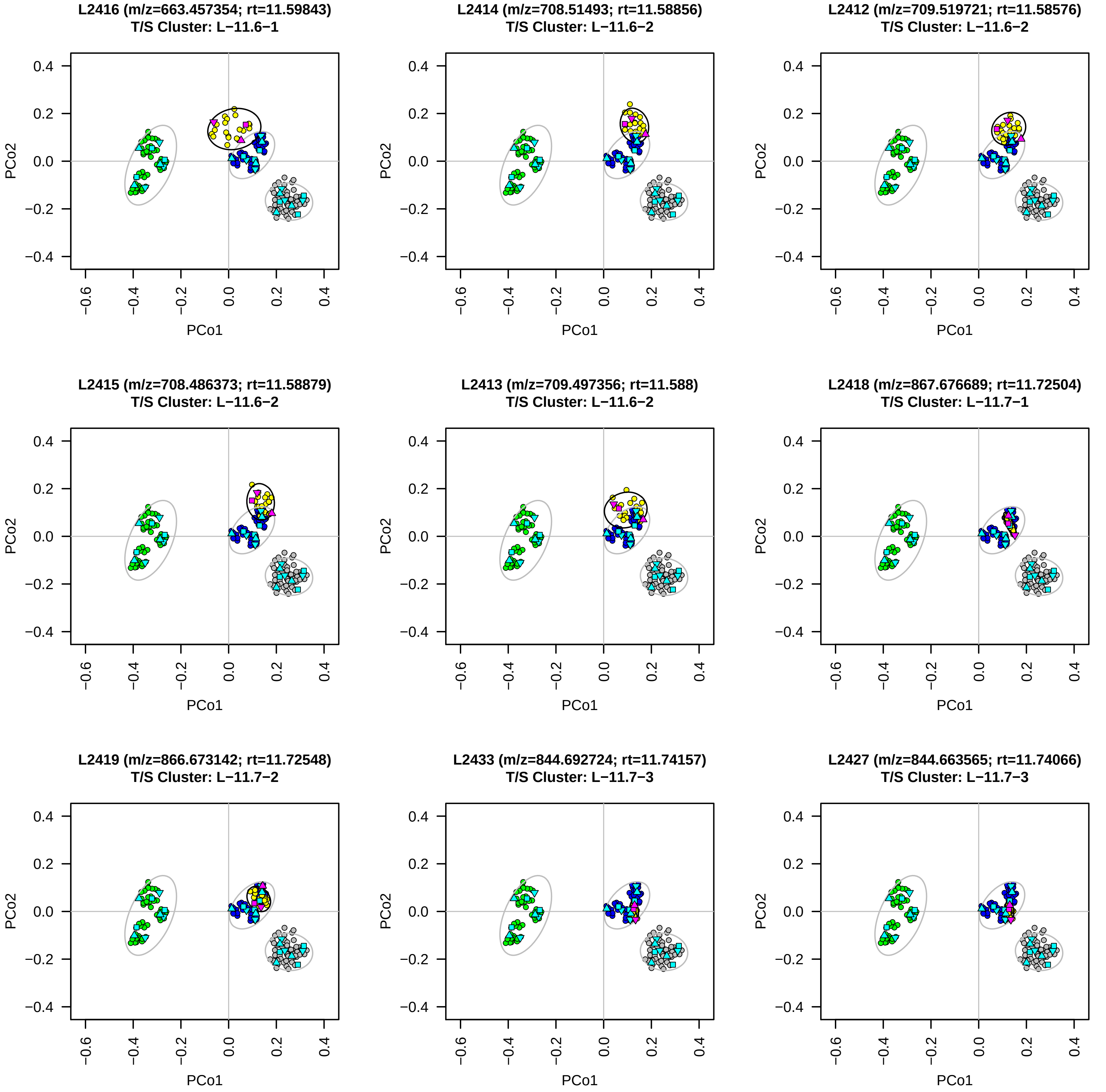
L2416 (m/z=663.457354; rt=11.59843)
T/S Cluster: L−11.6−1
L2414 (m/z=708.51493; rt=11.58856)
T/S Cluster: L−11.6−2
L2412 (m/z=709.519721; rt=11.58576)
T/S Cluster: L−11.6−2
L2415 (m/z=708.486373; rt=11.58879)
T/S Cluster: L−11.6−2
L2413 (m/z=709.497356; rt=11.588)
T/S Cluster: L−11.6−2
L2418 (m/z=867.676689; rt=11.72504)
T/S Cluster: L−11.7−1
L2419 (m/z=866.673142; rt=11.72548)
T/S Cluster: L−11.7−2
L2433 (m/z=844.692724; rt=11.74157)
T/S Cluster: L−11.7−3
L2427 (m/z=844.663565; rt=11.74066)
T/S Cluster: L−11.7−3
PCo1
PCo2
0.4
0.2
0.0
−0.2
−0.4
−0.6

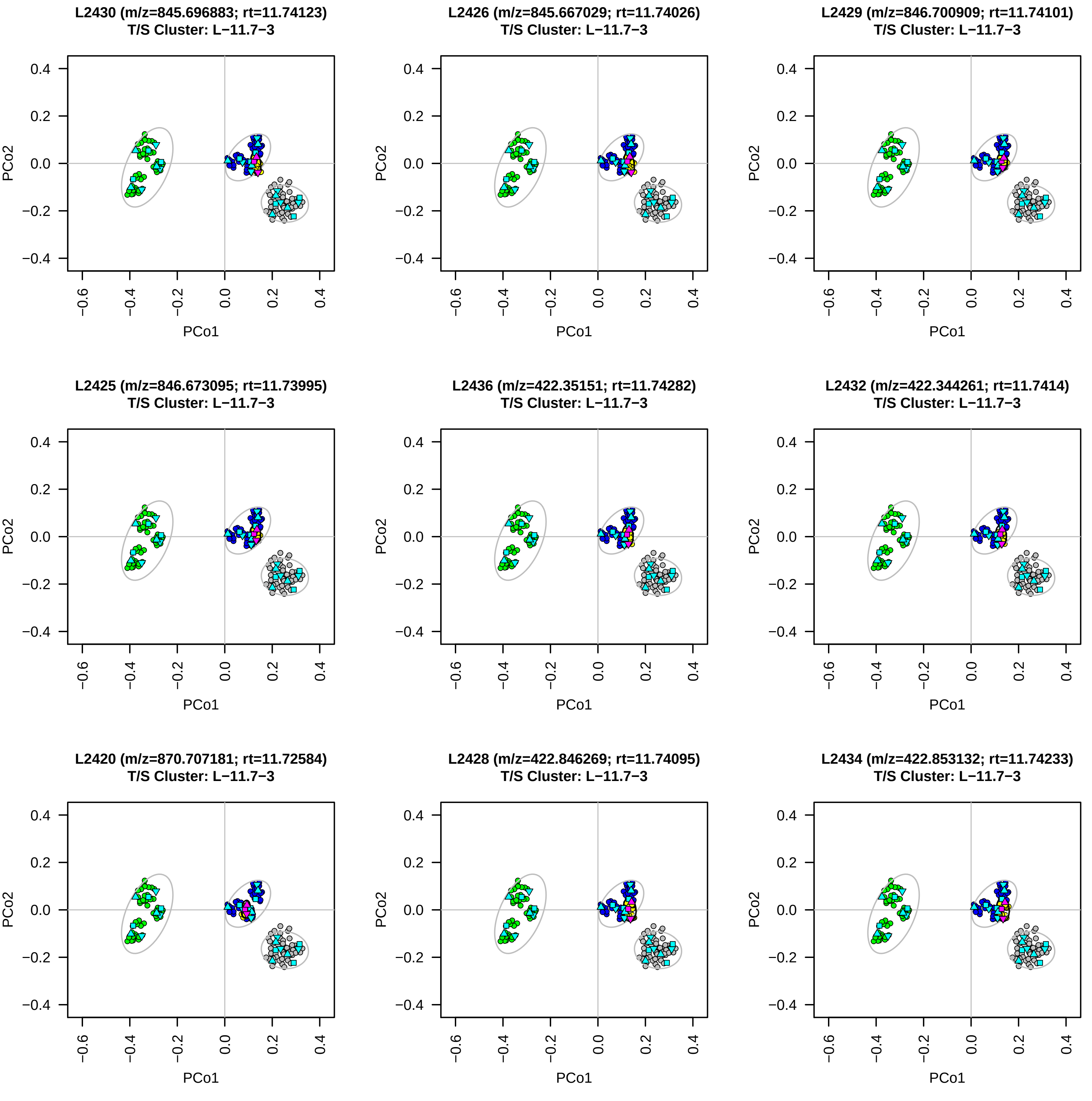
L2430 (m/z=845.696883; rt=11.74123)
T/S Cluster: L−11.7−3
L2426 (m/z=845.667029; rt=11.74026)
T/S Cluster: L−11.7−3
L2429 (m/z=846.700909; rt=11.74101)
T/S Cluster: L−11.7−3
L2425 (m/z=846.673095; rt=11.73995)
T/S Cluster: L−11.7−3
L2436 (m/z=422.35151; rt=11.74282)
T/S Cluster: L−11.7−3
L2432 (m/z=422.344261; rt=11.7414)
T/S Cluster: L−11.7−3
L2420 (m/z=870.707181; rt=11.72584)
T/S Cluster: L−11.7−3
L2428 (m/z=422.846269; rt=11.74095)
T/S Cluster: L−11.7−3
L2434 (m/z=422.853132; rt=11.74233)
T/S Cluster: L−11.7−3
PCo1
PCo2
−0.6
−0.4
−0.2
0.0
0.2
0.4

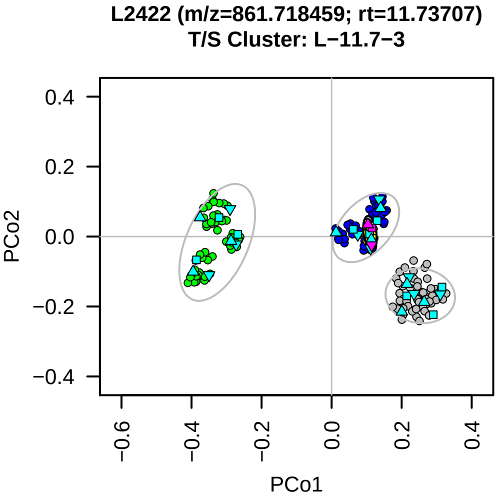
L2422 (m/z=861.718459; rt=11.73707)
T/S Cluster: L−11.7−3
PCo2
PCo1

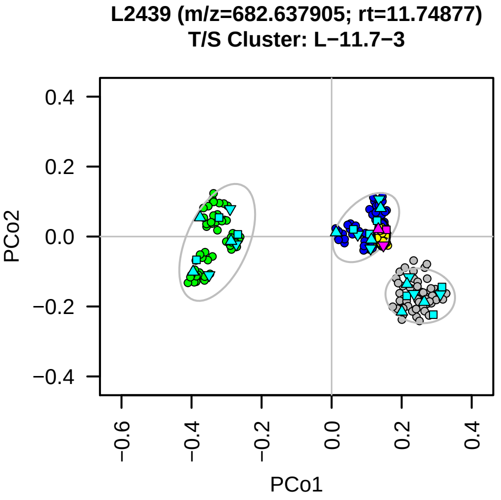
L2439 (m/z=682.637905; rt=11.74877)
T/S Cluster: L−11.7−3
PCo2
PCo1

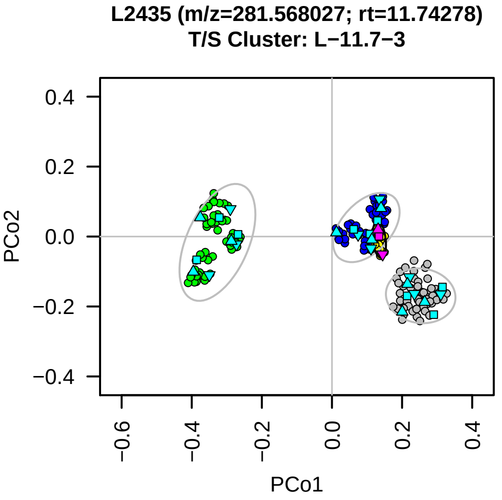
L2435 (m/z=281.568027; rt=11.74278)
T/S Cluster: L−11.7−3
PCo2
PCo1

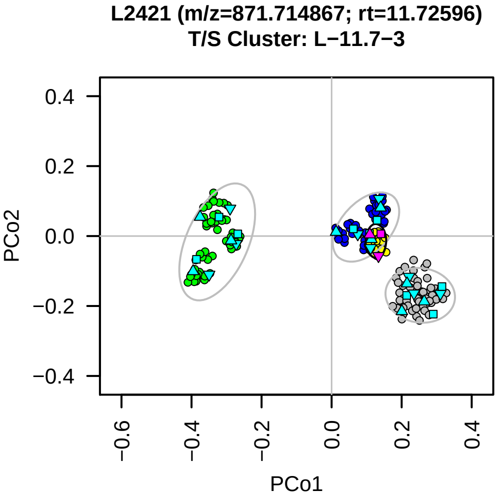
L2421 (m/z=871.714867; rt=11.72596)
T/S Cluster: L−11.7−3
PCo2
PCo1

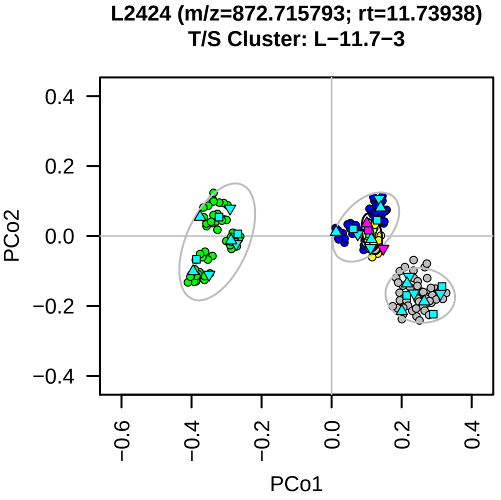
L2424 (m/z=872.715793; rt=11.73938)
T/S Cluster: L−11.7−3
PCo2
PCo1

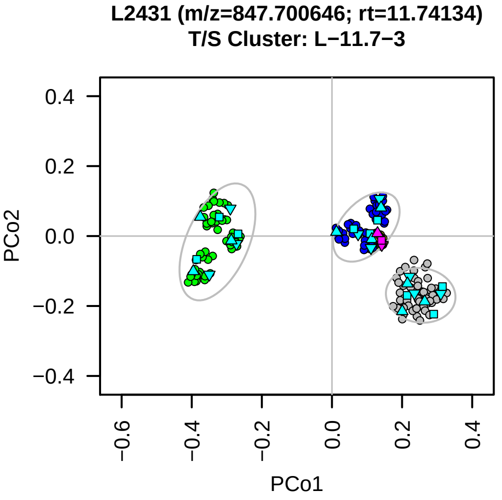
L2431 (m/z=847.700646; rt=11.74134)
T/S Cluster: L−11.7−3
PCo2
PCo1

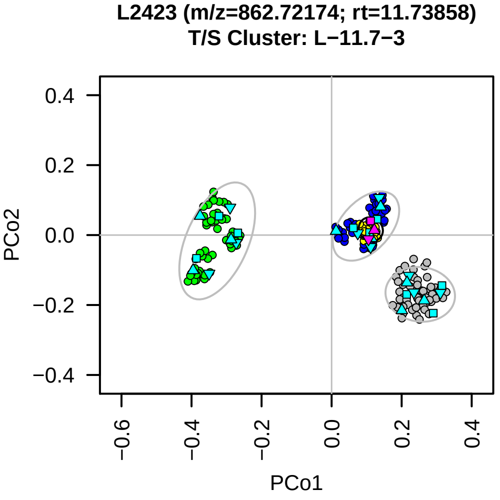
L2423 (m/z=862.72174; rt=11.73858)
T/S Cluster: L−11.7−3
PCo2
PCo1

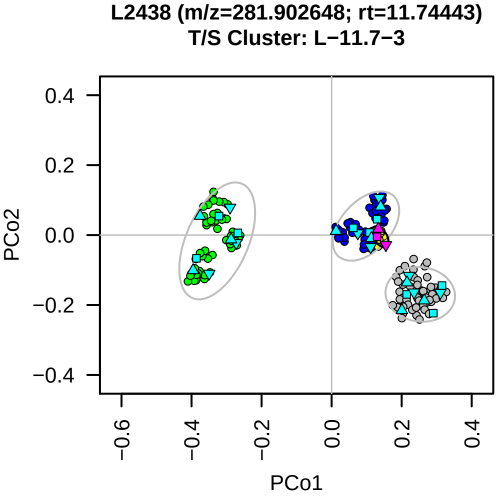
L2438 (m/z=281.902648; rt=11.74443)
T/S Cluster: L−11.7−3
PCo2
PCo1

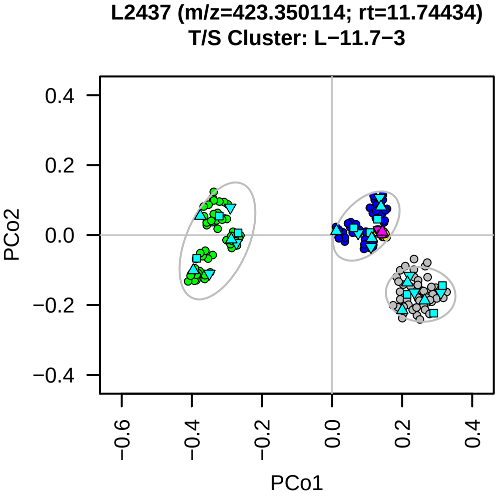
L2437 (m/z=423.350114; rt=11.74434)
T/S Cluster: L−11.7−3
PCo2
PCo1

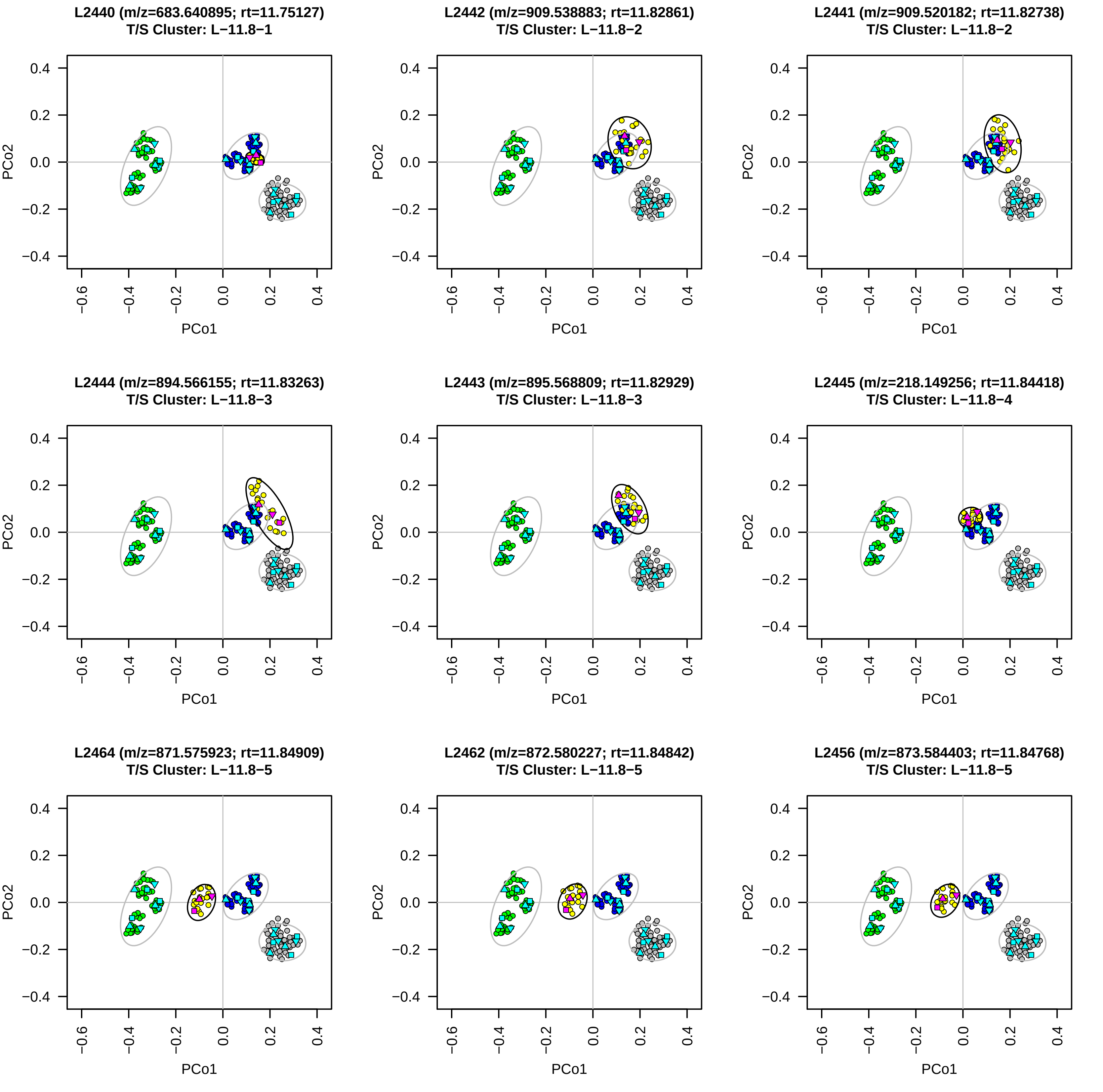
L2440 (m/z=683.640895; rt=11.75127)
T/S Cluster: L−11.8−1
L2442 (m/z=909.538883; rt=11.82861)
T/S Cluster: L−11.8−2
L2441 (m/z=909.520182; rt=11.82738)
T/S Cluster: L−11.8−2
L2444 (m/z=894.566155; rt=11.83263)
T/S Cluster: L−11.8−3
L2443 (m/z=895.568809; rt=11.82929)
T/S Cluster: L−11.8−3
L2445 (m/z=218.149256; rt=11.84418)
T/S Cluster: L−11.8−4
L2464 (m/z=871.575923; rt=11.84909)
T/S Cluster: L−11.8−5
L2462 (m/z=872.580227; rt=11.84842)
T/S Cluster: L−11.8−5
L2456 (m/z=873.584403; rt=11.84768)
T/S Cluster: L−11.8−5
PCo1
PCo2
−0.6
−0.4
−0.2
0.0
0.2
0.4

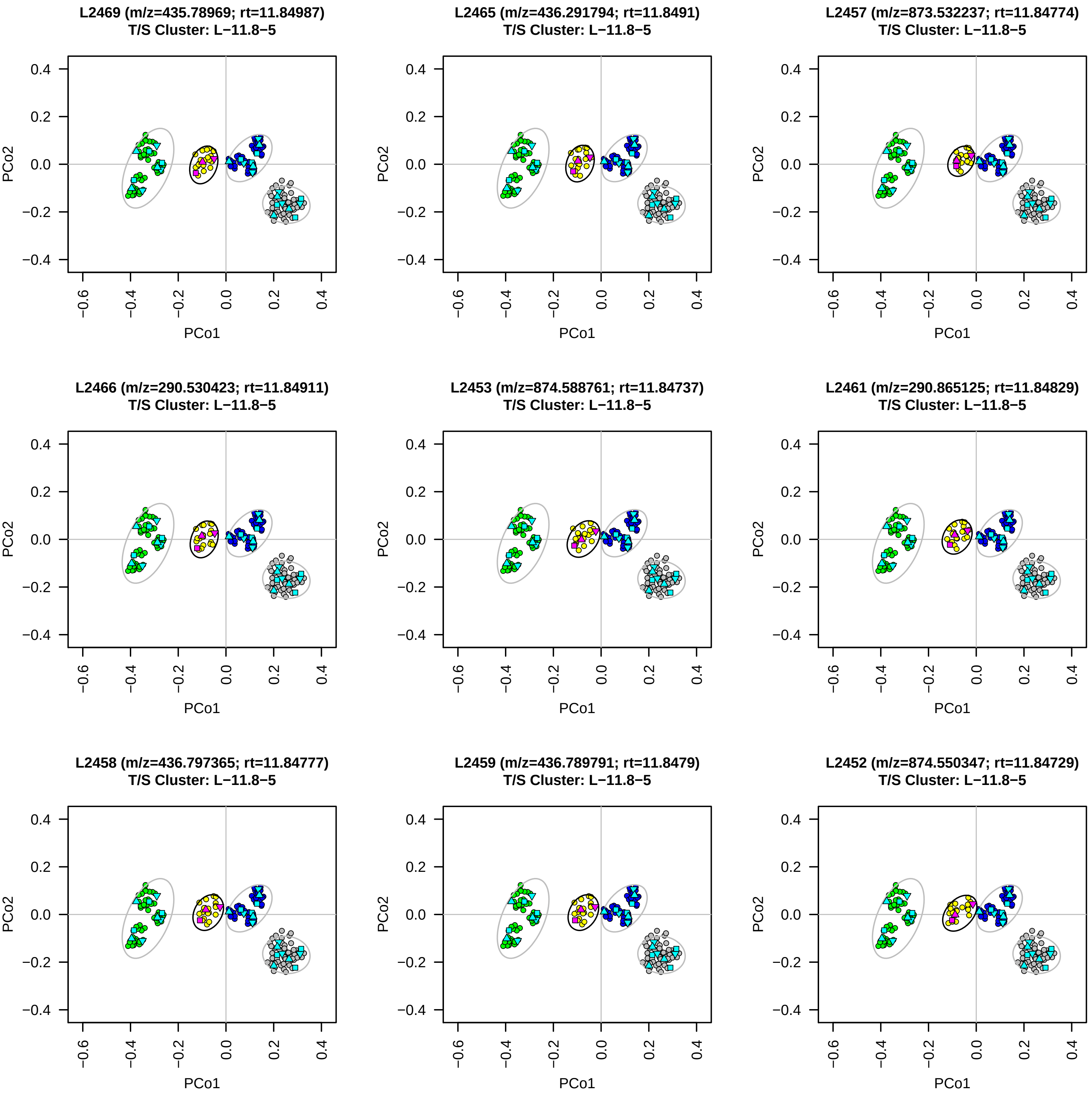
L2469 (m/z=435.78969; rt=11.84987)
T/S Cluster: L−11.8−5
L2465 (m/z=436.291794; rt=11.8491)
T/S Cluster: L−11.8−5
L2457 (m/z=873.532237; rt=11.84774)
T/S Cluster: L−11.8−5
L2466 (m/z=290.530423; rt=11.84911)
T/S Cluster: L−11.8−5
L2453 (m/z=874.588761; rt=11.84737)
T/S Cluster: L−11.8−5
L2461 (m/z=290.865125; rt=11.84829)
T/S Cluster: L−11.8−5
L2458 (m/z=436.797365; rt=11.84777)
T/S Cluster: L−11.8−5
L2459 (m/z=436.789791; rt=11.8479)
T/S Cluster: L−11.8−5
L2452 (m/z=874.550347; rt=11.84729)
T/S Cluster: L−11.8−5
PCo1
PCo2
−0.6
−0.4
−0.2
0.0
0.2
0.4

L2463 (m/z=290.859394; rt=11.84906)
T/S Cluster: L−11.8−5

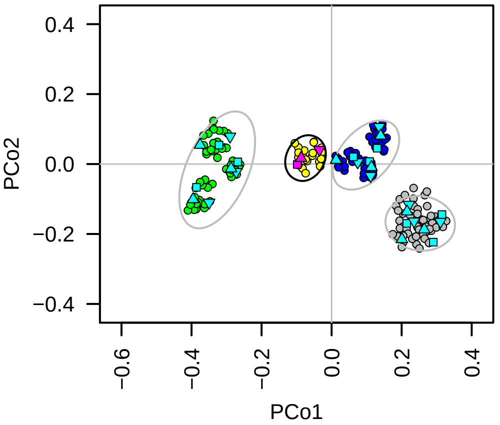

L2467 (m/z=436.275827; rt=11.84914)
T/S Cluster: L−11.8−5

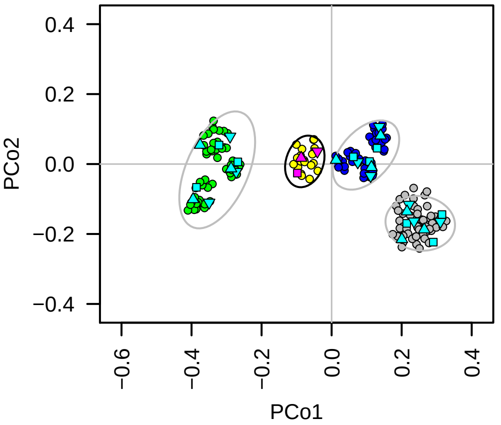

L2451 (m/z=291.20023; rt=11.84631)
T/S Cluster: L−11.8−5

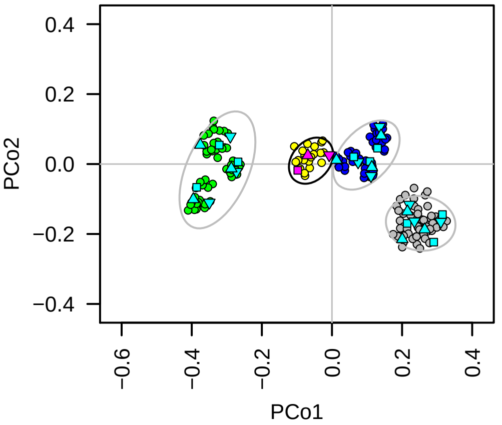

L2454 (m/z=875.592652; rt=11.84757)
T/S Cluster: L−11.8−5

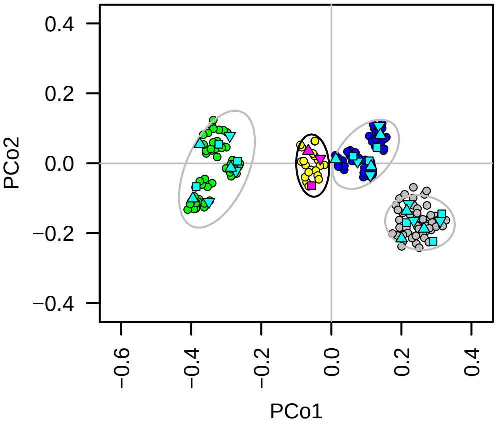

L2450 (m/z=291.196179; rt=11.84605)
T/S Cluster: L−11.8−5

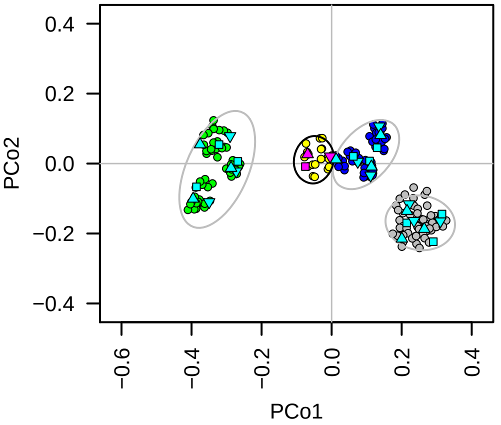

L2448 (m/z=437.29867; rt=11.84559)
T/S Cluster: L−11.8−5

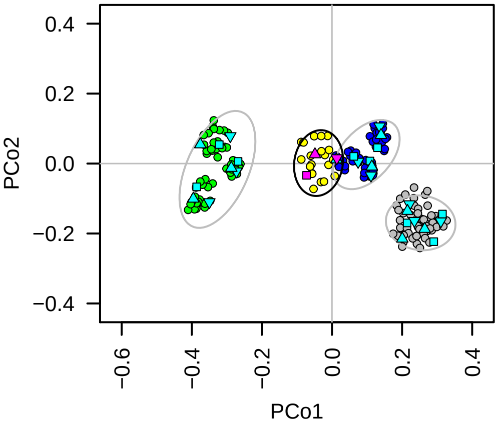

L2447 (m/z=437.289763; rt=11.84557)
T/S Cluster: L−11.8−5

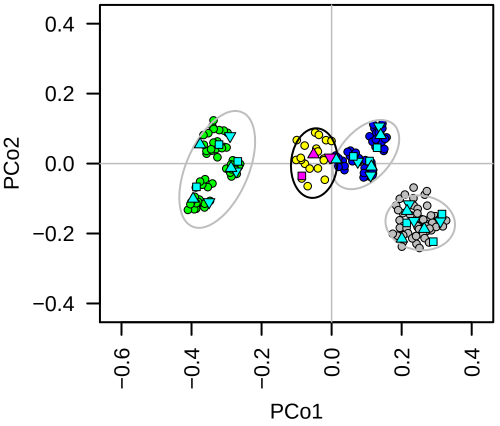

L2468 (m/z=217.894515; rt=11.84943)
T/S Cluster: L−11.8−5

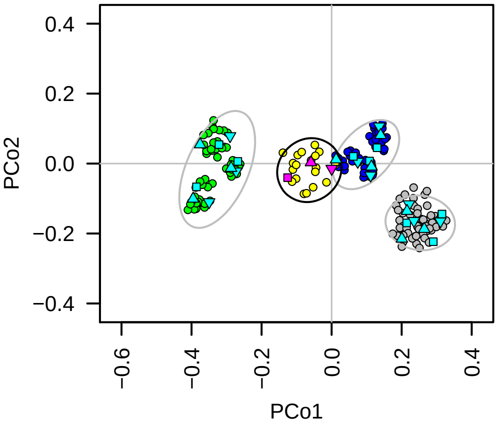

L2446 (m/z=217.898746; rt=11.84515)
T/S Cluster: L−11.8−5

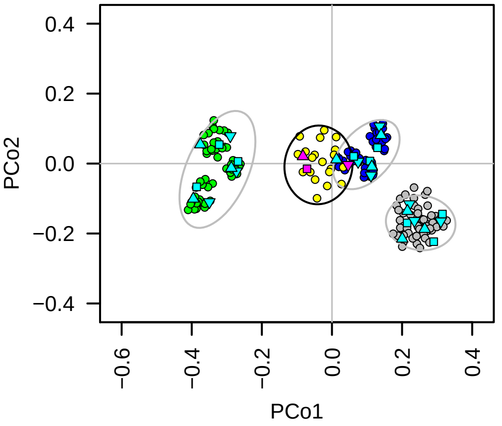

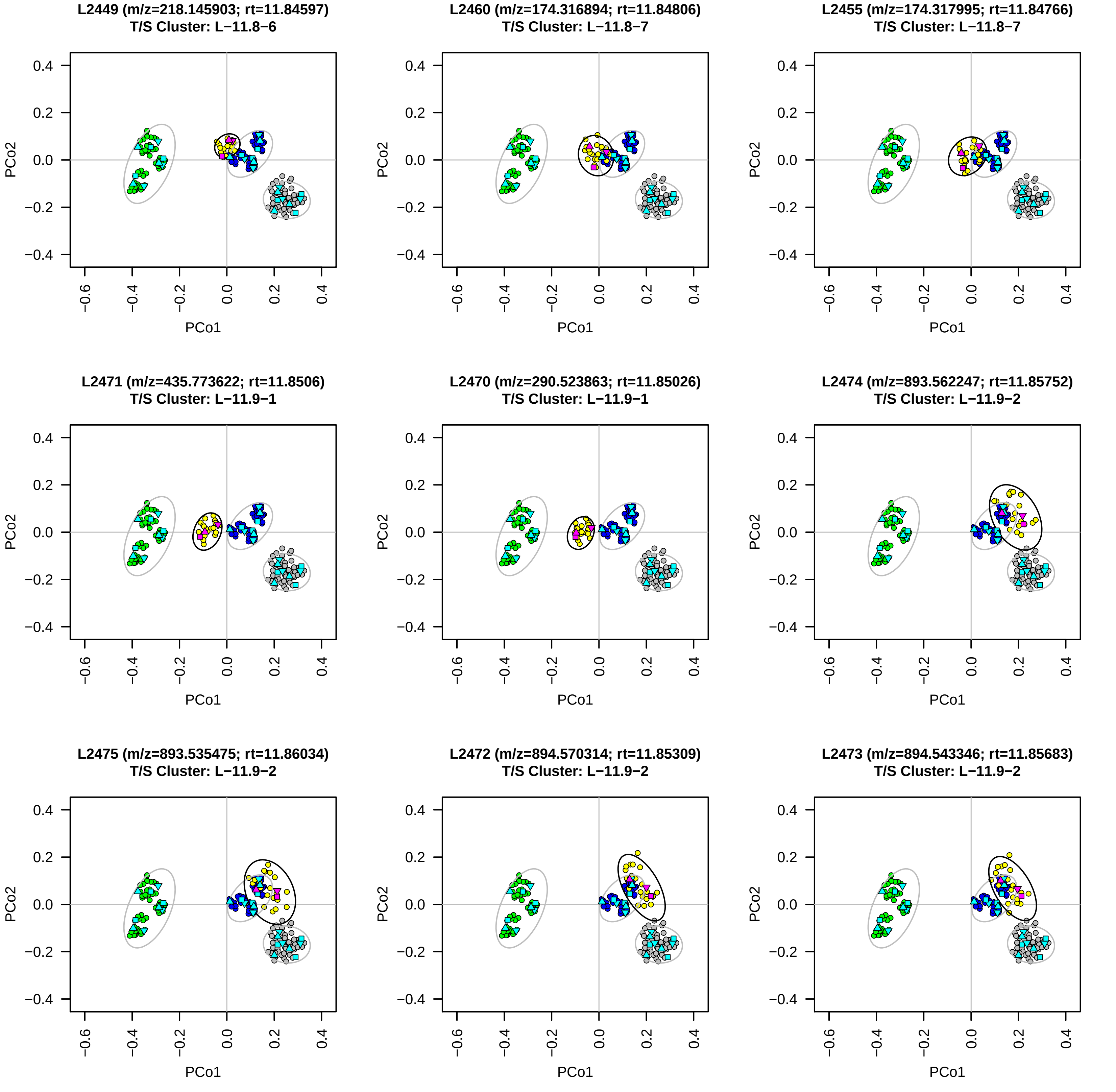
L2449 (m/z=218.145903; rt=11.84597)
T/S Cluster: L−11.8−6
L2460 (m/z=174.316894; rt=11.84806)
T/S Cluster: L−11.8−7
L2455 (m/z=174.317995; rt=11.84766)
T/S Cluster: L−11.8−7
L2471 (m/z=435.773622; rt=11.8506)
T/S Cluster: L−11.9−1
L2470 (m/z=290.523863; rt=11.85026)
T/S Cluster: L−11.9−1
L2474 (m/z=893.562247; rt=11.85752)
T/S Cluster: L−11.9−2
L2475 (m/z=893.535475; rt=11.86034)
T/S Cluster: L−11.9−2
L2472 (m/z=894.570314; rt=11.85309)
T/S Cluster: L−11.9−2
L2473 (m/z=894.543346; rt=11.85683)
T/S Cluster: L−11.9−2
PCo1
PCo2
0.4
0.2
0.0
−0.2
−0.4
−0.6

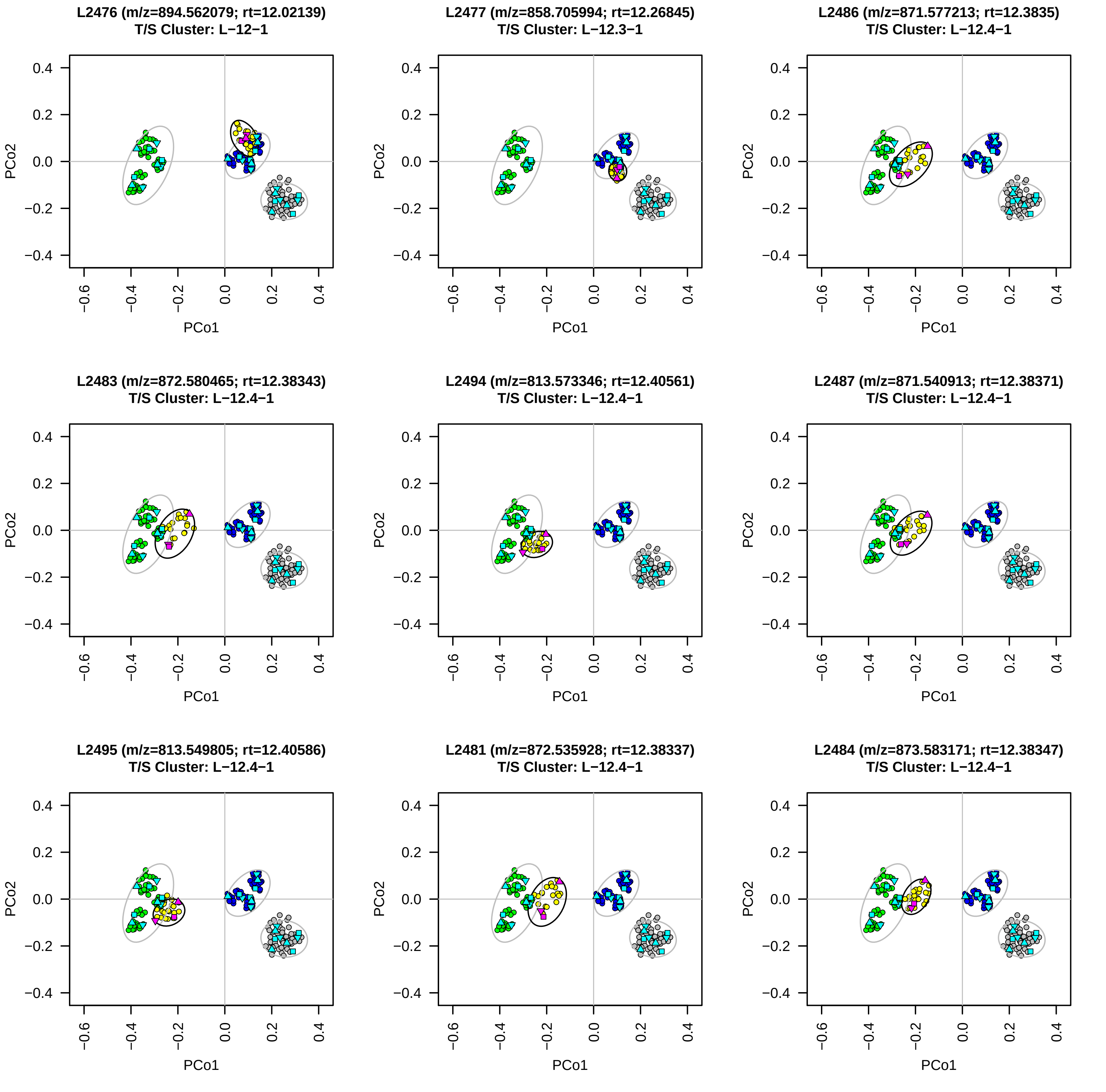
L2476 (m/z=894.562079; rt=12.02139)
T/S Cluster: L−12−1
L2477 (m/z=858.705994; rt=12.26845)
T/S Cluster: L−12.3−1
L2486 (m/z=871.577213; rt=12.3835)
T/S Cluster: L−12.4−1
L2483 (m/z=872.580465; rt=12.38343)
T/S Cluster: L−12.4−1
L2494 (m/z=813.573346; rt=12.40561)
T/S Cluster: L−12.4−1
L2487 (m/z=871.540913; rt=12.38371)
T/S Cluster: L−12.4−1
L2495 (m/z=813.549805; rt=12.40586)
T/S Cluster: L−12.4−1
L2481 (m/z=872.535928; rt=12.38337)
T/S Cluster: L−12.4−1
L2484 (m/z=873.583171; rt=12.38347)
T/S Cluster: L−12.4−1
PCo1
PCo2
0.4
0.2
0.0
−0.2
−0.4
−0.6

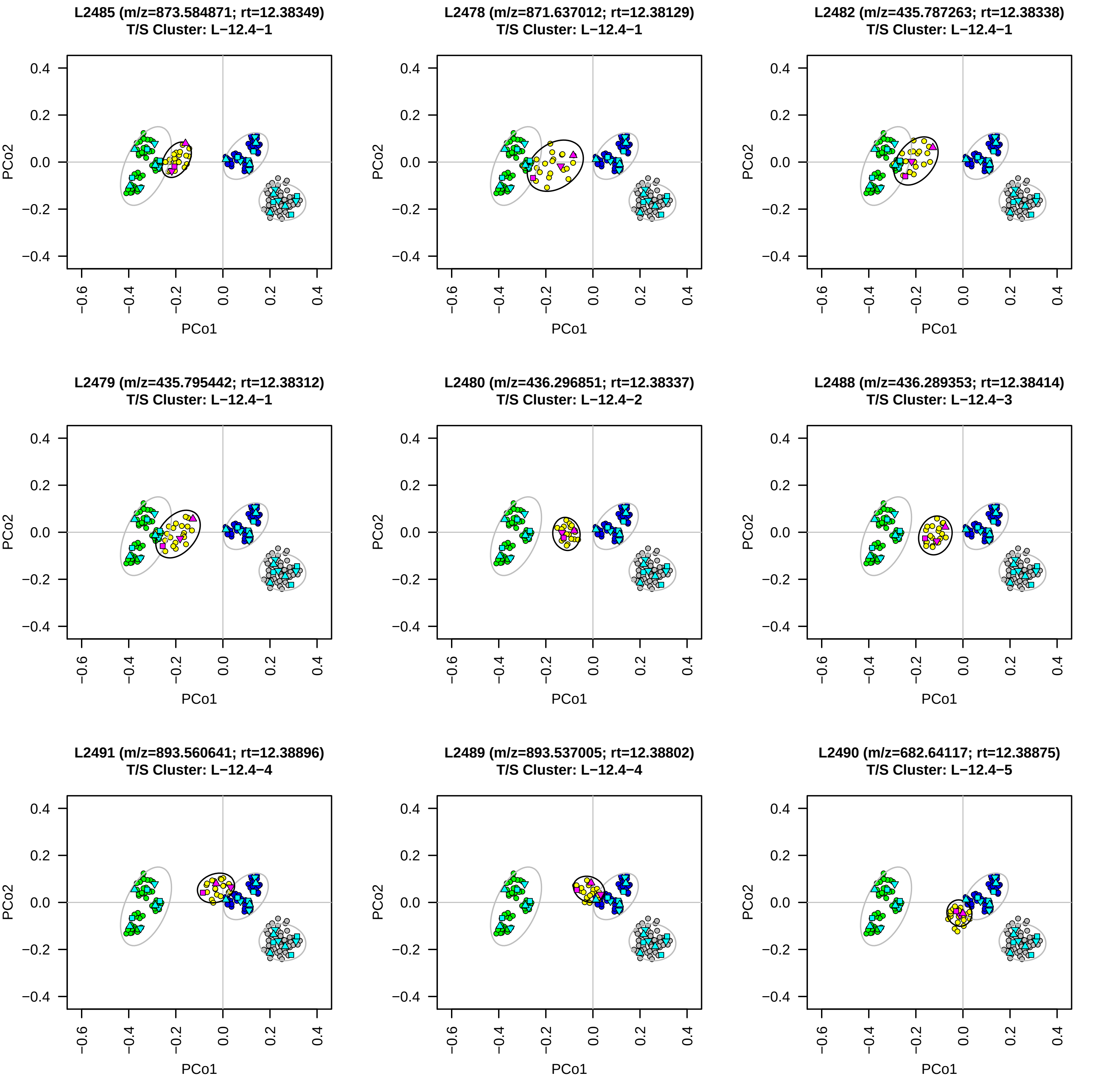
L2485 (m/z=873.584871; rt=12.38349)
T/S Cluster: L−12.4−1
L2478 (m/z=871.637012; rt=12.38129)
T/S Cluster: L−12.4−1
L2482 (m/z=435.787263; rt=12.38338)
T/S Cluster: L−12.4−1
L2479 (m/z=435.795442; rt=12.38312)
T/S Cluster: L−12.4−1
L2480 (m/z=436.296851; rt=12.38337)
T/S Cluster: L−12.4−2
L2488 (m/z=436.289353; rt=12.38414)
T/S Cluster: L−12.4−3
L2491 (m/z=893.560641; rt=12.38896)
T/S Cluster: L−12.4−4
L2489 (m/z=893.537005; rt=12.38802)
T/S Cluster: L−12.4−4
L2490 (m/z=682.64117; rt=12.38875)
T/S Cluster: L−12.4−5
PCo1
PCo2
0.4
0.2
0.0
−0.2
−0.4
−0.6

L2492 (m/z=682.62611; rt=12.38932)
T/S Cluster: L−12.4−5

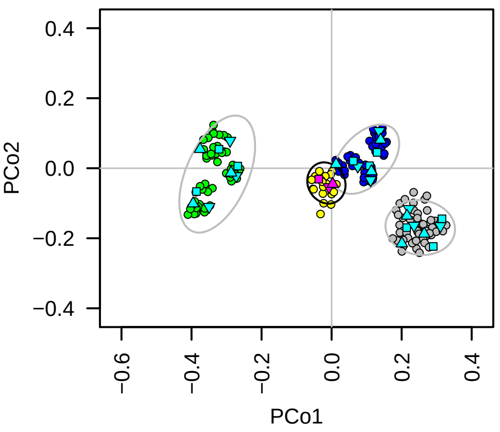

L2493 (m/z=683.640331; rt=12.39029)
T/S Cluster: L−12.4−5

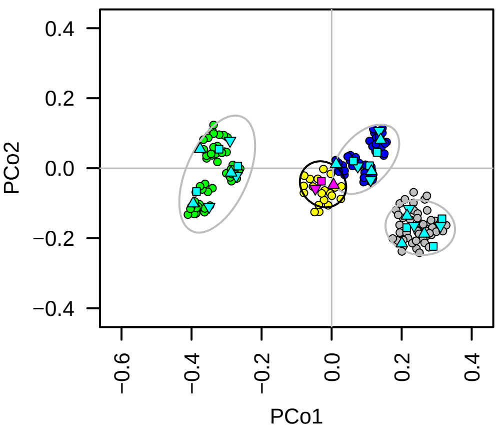

L2496 (m/z=828.695897; rt=12.50723)
T/S Cluster: L−12.5−1

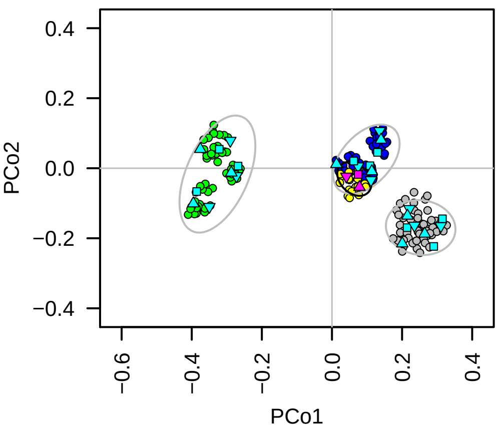

L2497 (m/z=437.746443; rt=12.52308)
T/S Cluster: L−12.5−2

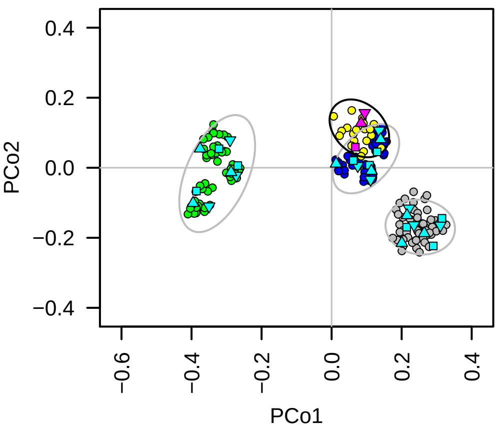

L2500 (m/z=684.207611; rt=12.76659)
T/S Cluster: L−12.8−1

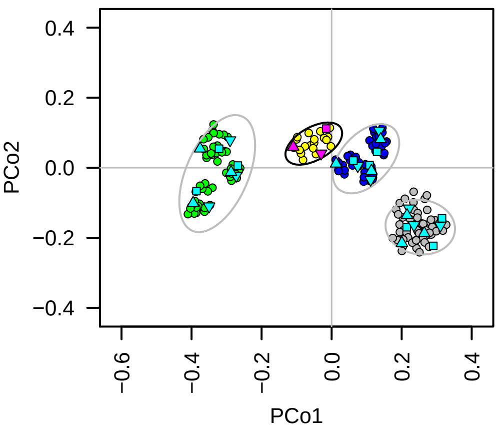

L2504 (m/z=684.188612; rt=12.76663)
T/S Cluster: L−12.8−1

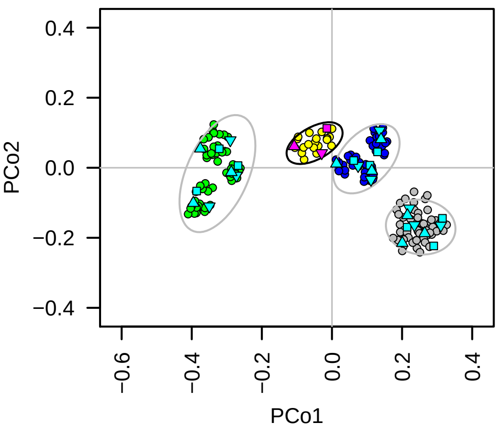

L2501 (m/z=685.208026; rt=12.7666)
T/S Cluster: L−12.8−1

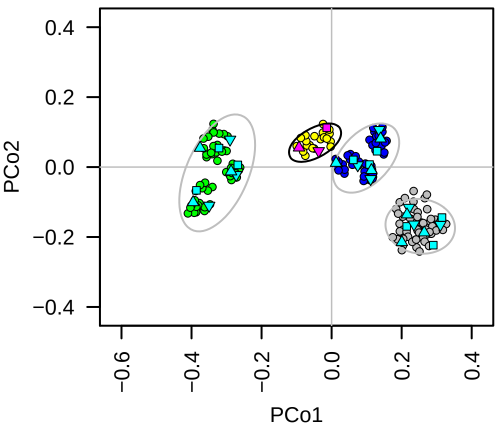

L2503 (m/z=685.188831; rt=12.76662)
T/S Cluster: L−12.8−1

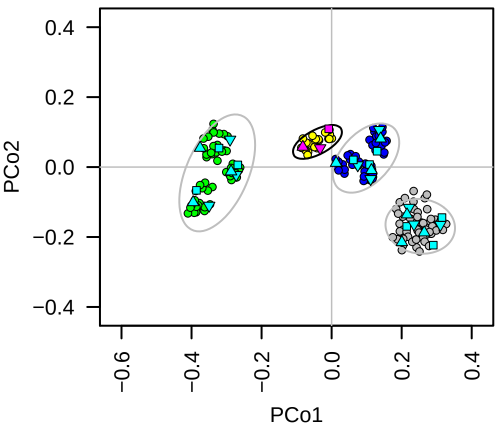

L2499 (m/z=686.206737; rt=12.76649)
T/S Cluster: L−12.8−1

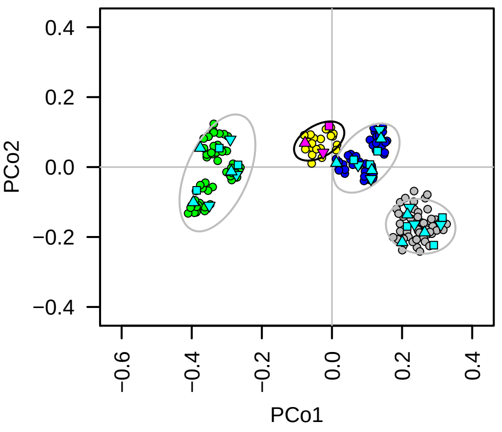

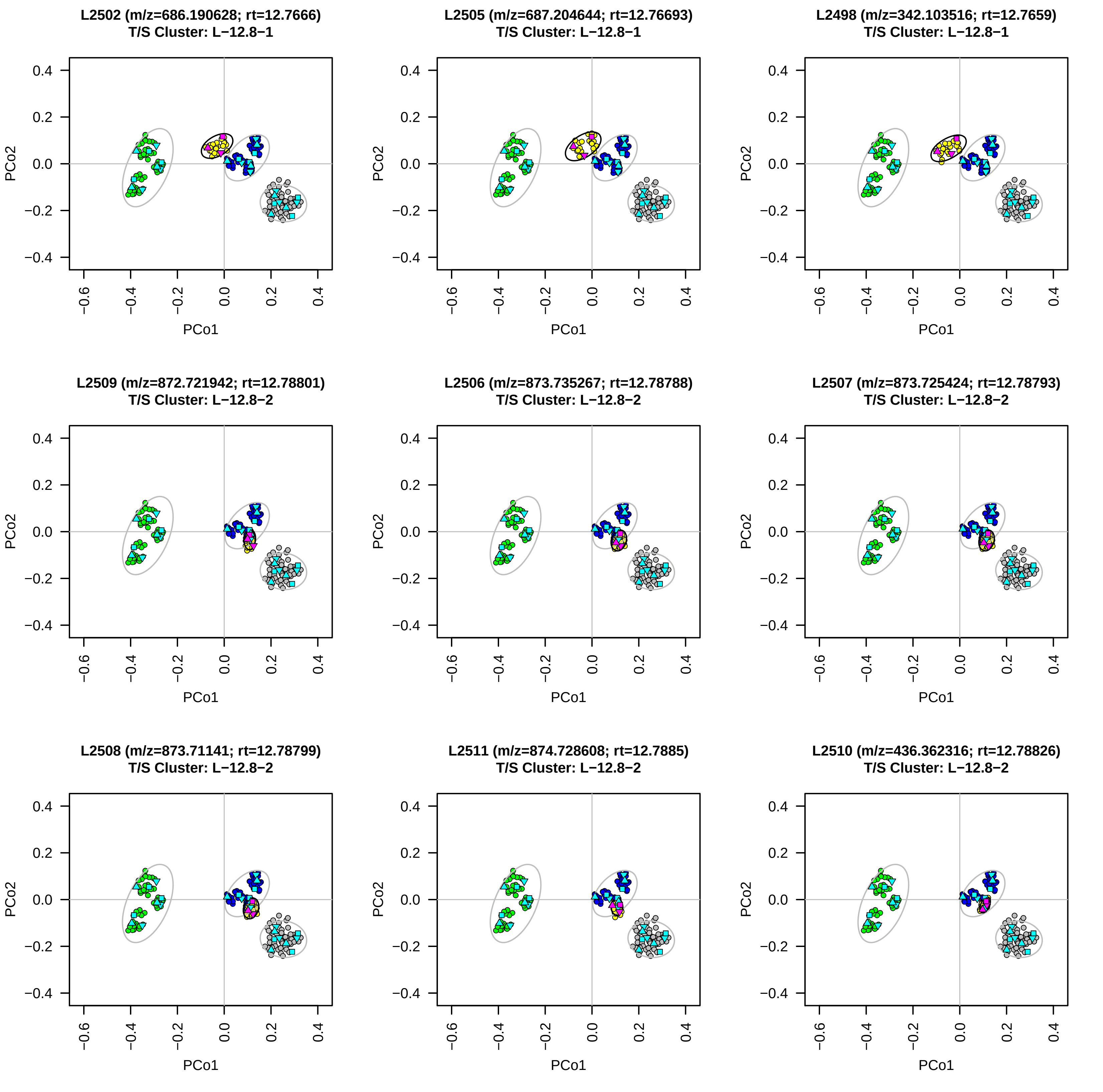
L2502 (m/z=686.190628; rt=12.7666)
T/S Cluster: L−12.8−1
L2505 (m/z=687.204644; rt=12.76693)
T/S Cluster: L−12.8−1
L2498 (m/z=342.103516; rt=12.7659)
T/S Cluster: L−12.8−1
L2509 (m/z=872.721942; rt=12.78801)
T/S Cluster: L−12.8−2
L2506 (m/z=873.735267; rt=12.78788)
T/S Cluster: L−12.8−2
L2507 (m/z=873.725424; rt=12.78793)
T/S Cluster: L−12.8−2
L2508 (m/z=873.71141; rt=12.78799)
T/S Cluster: L−12.8−2
L2511 (m/z=874.728608; rt=12.7885)
T/S Cluster: L−12.8−2
L2510 (m/z=436.362316; rt=12.78826)
T/S Cluster: L−12.8−2
PCo1
PCo2
0.4
0.2
0.0
−0.2
−0.4
−0.6

L2512 (m/z=894.70347; rt=12.78985)
T/S Cluster: L−12.8−3

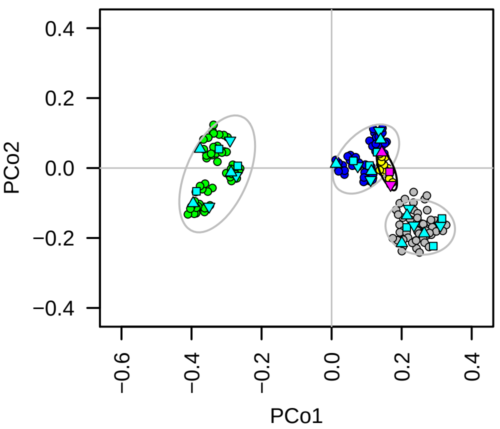

L2513 (m/z=895.706831; rt=12.79005)
T/S Cluster: L−12.8−3

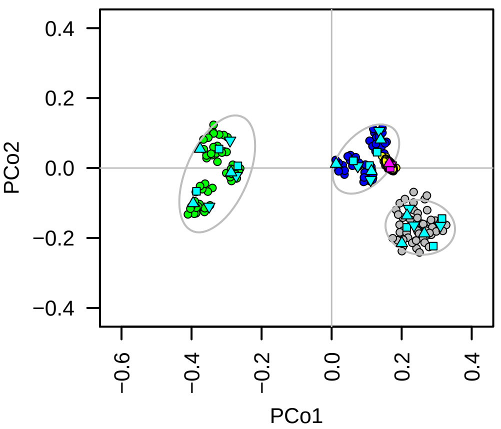

L2514 (m/z=437.746816; rt=13.65571)
T/S Cluster: L−13.7−1

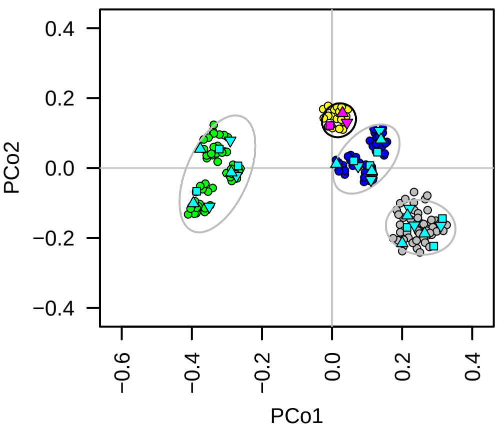

L2516 (m/z=900.753482; rt=13.7002)
T/S Cluster: L−13.7−2

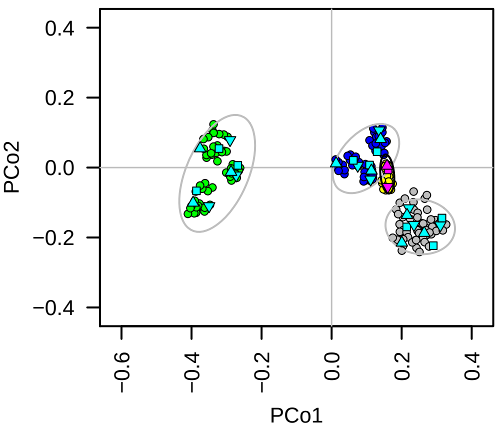

L2517 (m/z=901.756297; rt=13.70076)
T/S Cluster: L−13.7−2

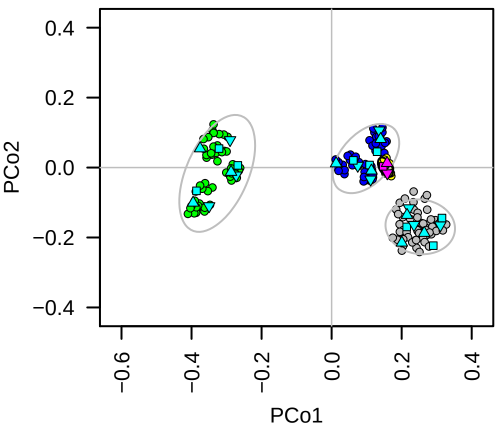

L2515 (m/z=832.705531; rt=13.69529)
T/S Cluster: L−13.7−2

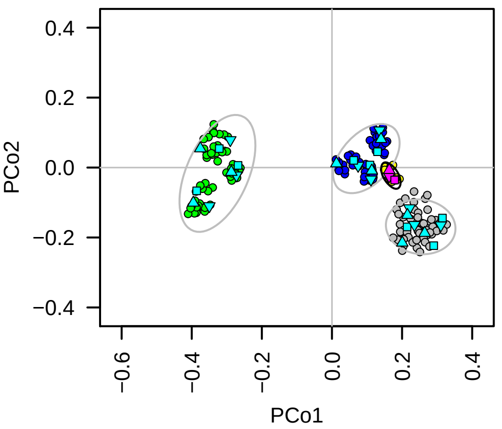

L2523 (m/z=758.225413; rt=14.00396)
T/S Cluster: L−14−1

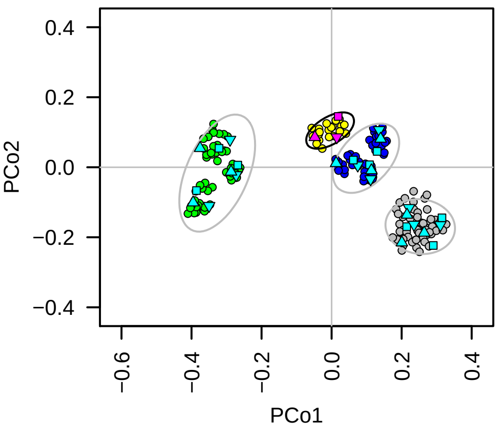

L2520 (m/z=759.227015; rt=14.0039)
T/S Cluster: L−14−1

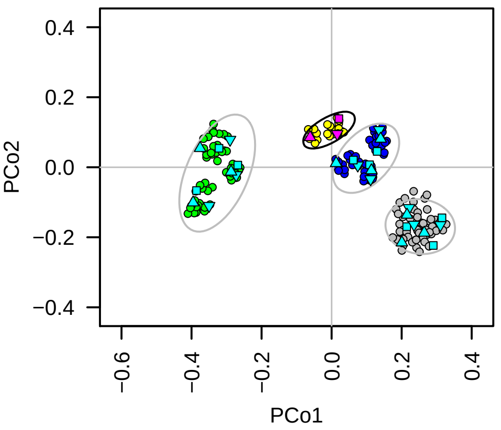

L2522 (m/z=758.199033; rt=14.00395)
T/S Cluster: L−14−1

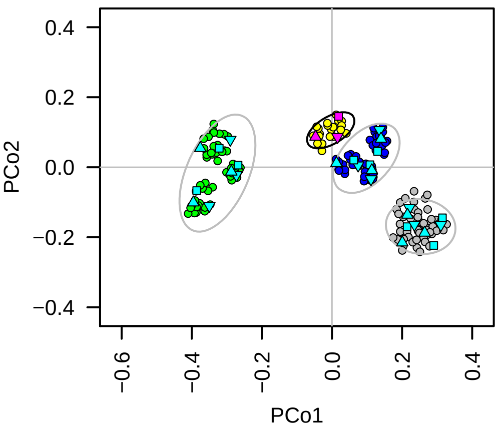

L2529 (m/z=760.227771; rt=14.00421)
T/S Cluster: L−14−1

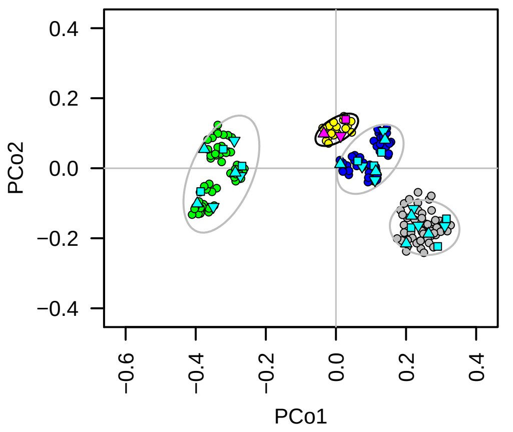

L2521 (m/z=759.202655; rt=14.00394)
T/S Cluster: L−14−1

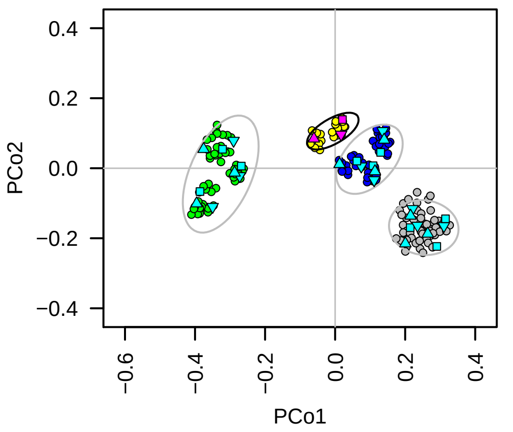

L2528 (m/z=760.207673; rt=14.00417)
T/S Cluster: L−14−1

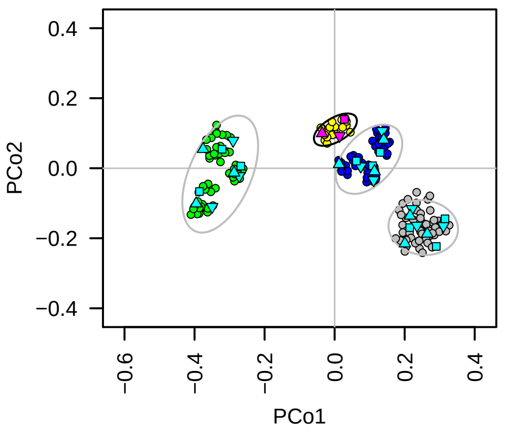

L2526 (m/z=761.232285; rt=14.00406)
T/S Cluster: L−14−1

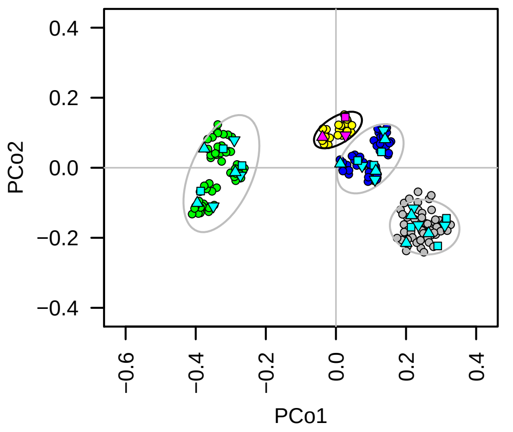

L2527 (m/z=761.213707; rt=14.00409)
T/S Cluster: L−14−1

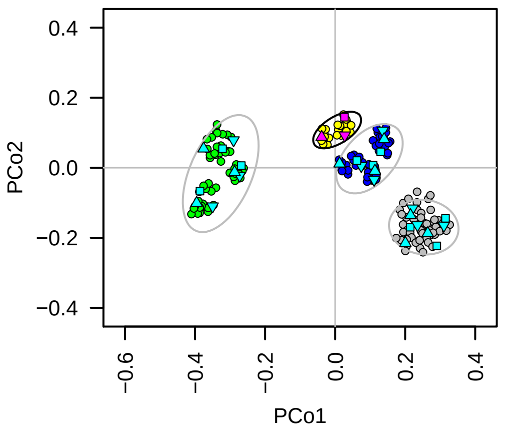

L2525 (m/z=762.222238; rt=14.00405)
T/S Cluster: L−14−1

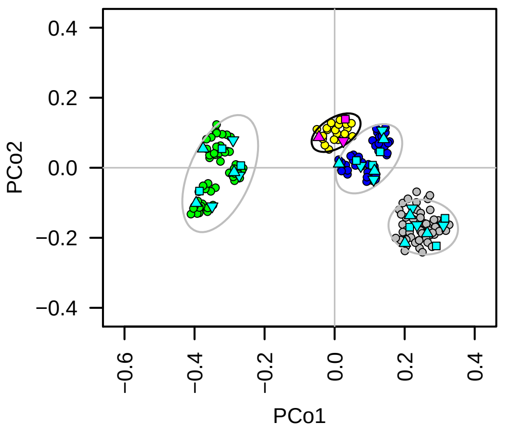

L2524 (m/z=379.113243; rt=14.004)
T/S Cluster: L−14−1

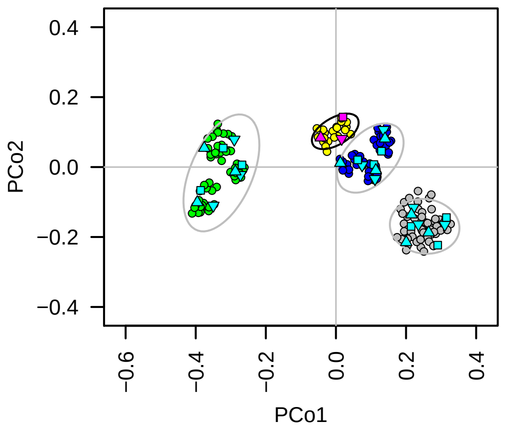

L2519 (m/z=379.613469; rt=14.00343)
T/S Cluster: L−14−1

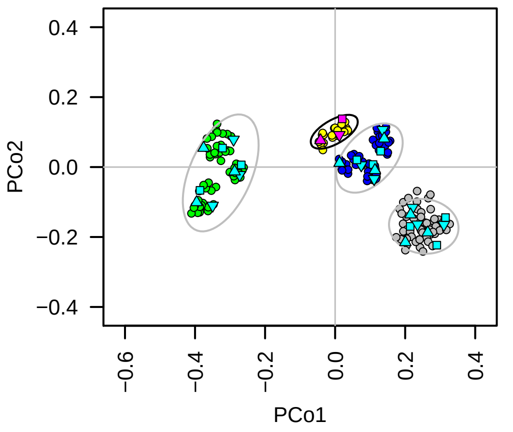

L2518 (m/z=380.112362; rt=14.00327)
T/S Cluster: L−14−1

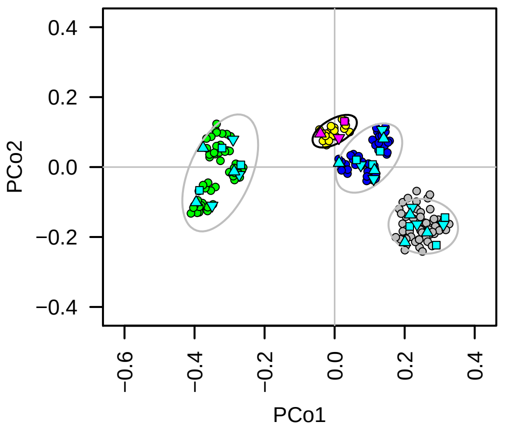

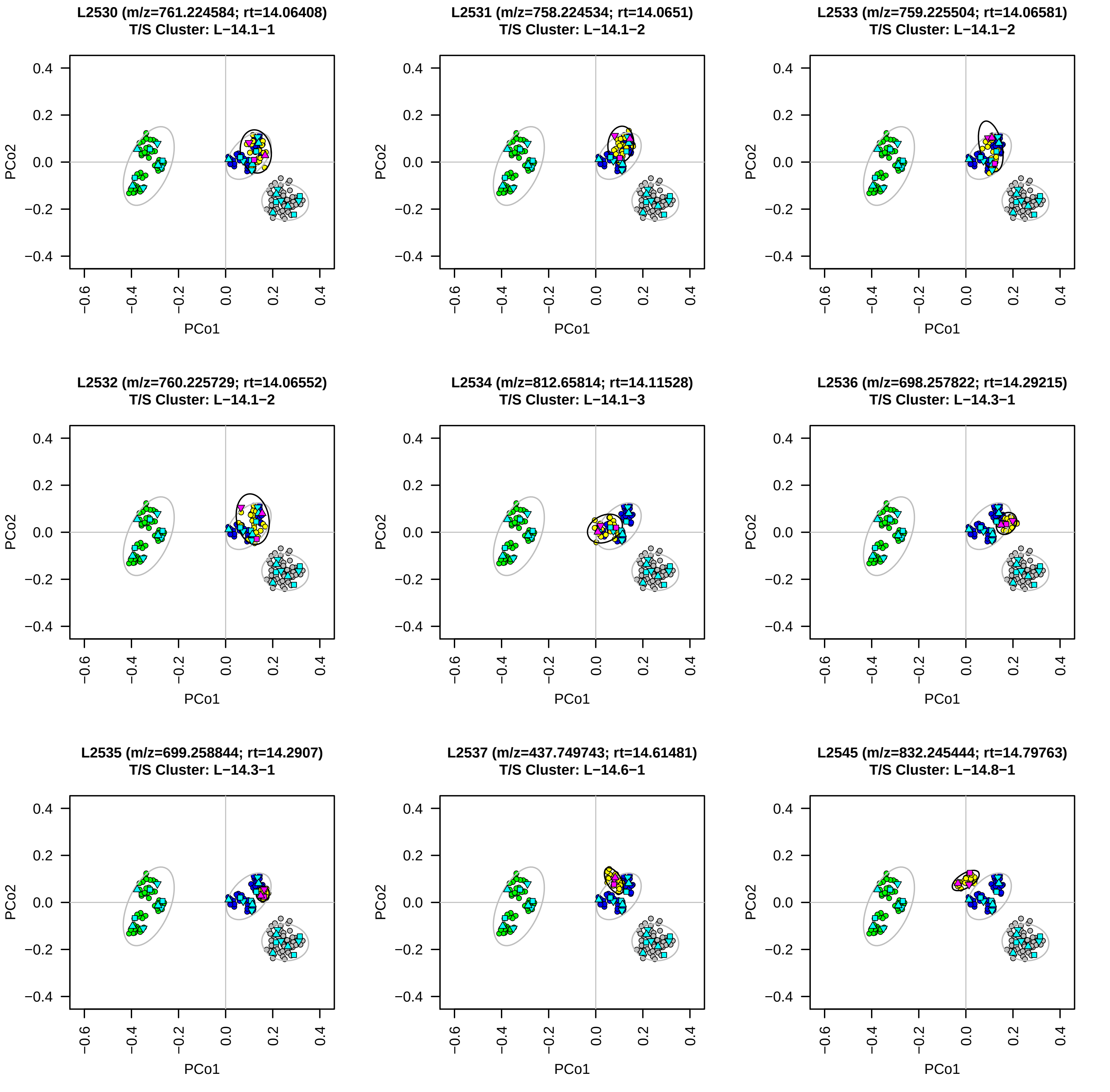
L2530 (m/z=761.224584; rt=14.06408)
T/S Cluster: L−14.1−1
L2531 (m/z=758.224534; rt=14.0651)
T/S Cluster: L−14.1−2
L2533 (m/z=759.225504; rt=14.06581)
T/S Cluster: L−14.1−2
L2532 (m/z=760.225729; rt=14.06552)
T/S Cluster: L−14.1−2
L2534 (m/z=812.65814; rt=14.11528)
T/S Cluster: L−14.1−3
L2536 (m/z=698.257822; rt=14.29215)
T/S Cluster: L−14.3−1
L2535 (m/z=699.258844; rt=14.2907)
T/S Cluster: L−14.3−1
L2537 (m/z=437.749743; rt=14.61481)
T/S Cluster: L−14.6−1
L2545 (m/z=832.245444; rt=14.79763)
T/S Cluster: L−14.8−1
PCo1
PCo2
−0.6
−0.4
−0.2
0.0
0.2
0.4

L2543 (m/z=833.247558; rt=14.79759)
T/S Cluster: L−14.8−1

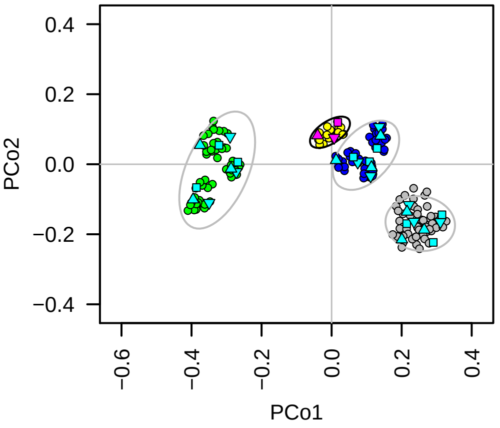

L2546 (m/z=832.215688; rt=14.79764)
T/S Cluster: L−14.8−1

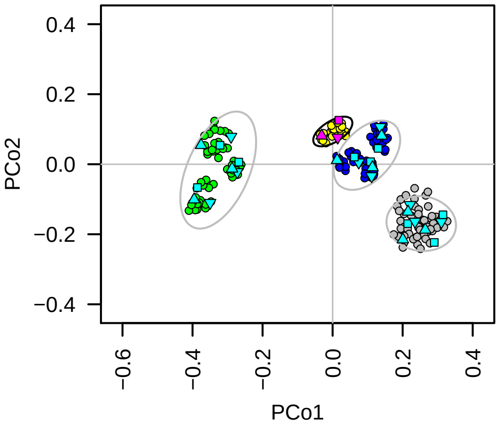

L2544 (m/z=833.222129; rt=14.7976)
T/S Cluster: L−14.8−1

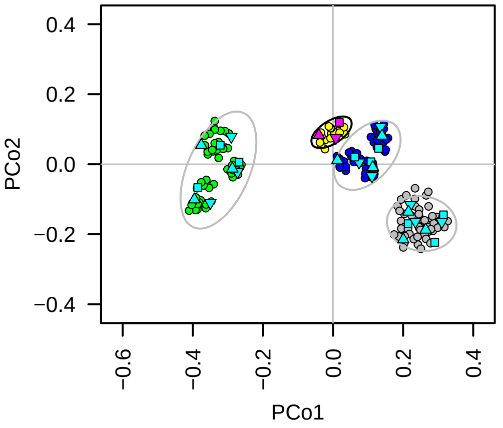

L2542 (m/z=834.245074; rt=14.79748)
T/S Cluster: L−14.8−1

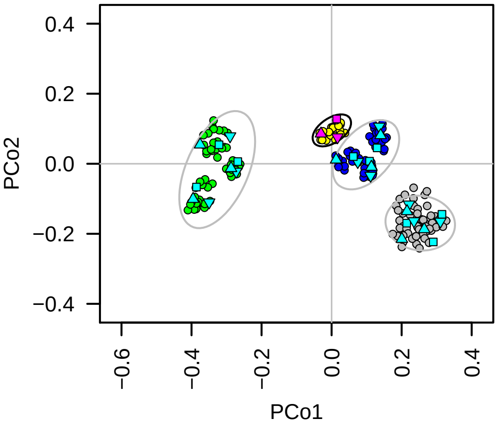

L2547 (m/z=834.216779; rt=14.79768)
T/S Cluster: L−14.8−1

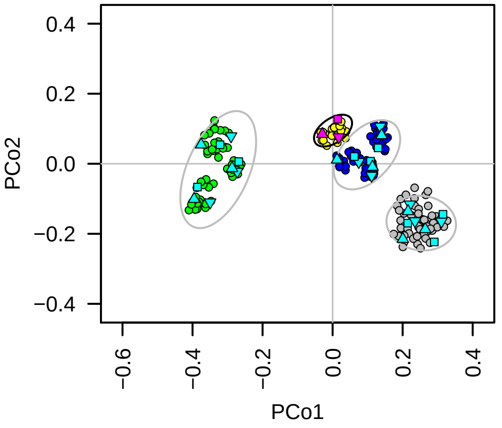

L2548 (m/z=835.249403; rt=14.79769)
T/S Cluster: L−14.8−1

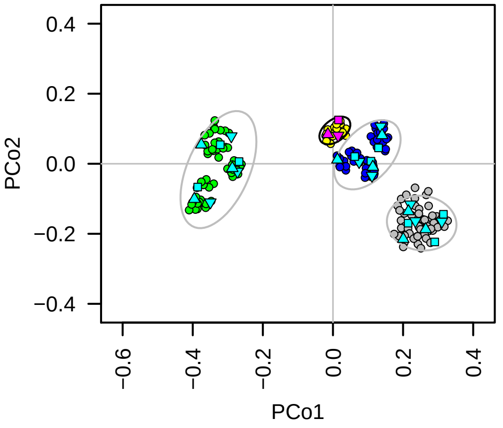

L2549 (m/z=835.225941; rt=14.79777)
T/S Cluster: L−14.8−1

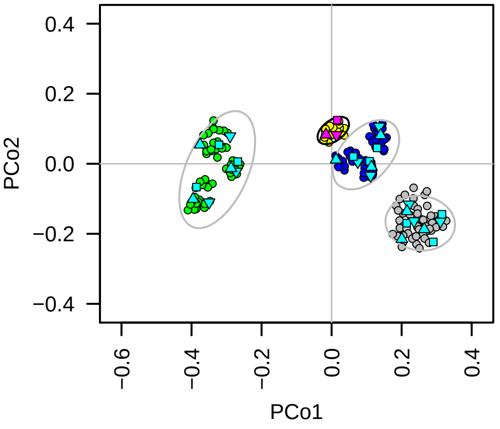

L2550 (m/z=836.241445; rt=14.7978)
T/S Cluster: L−14.8−1

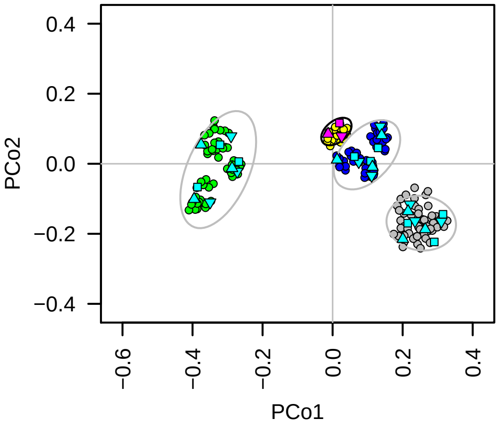

L2541 (m/z=416.122983; rt=14.79698)
T/S Cluster: L−14.8−1

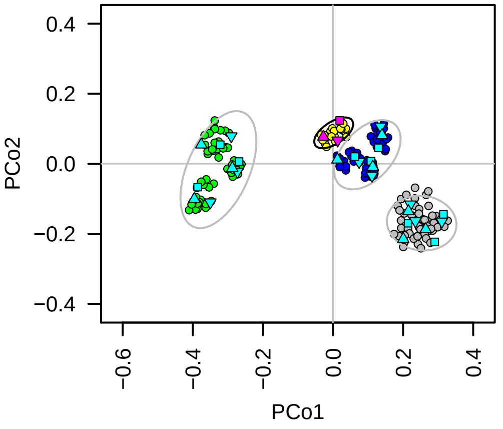

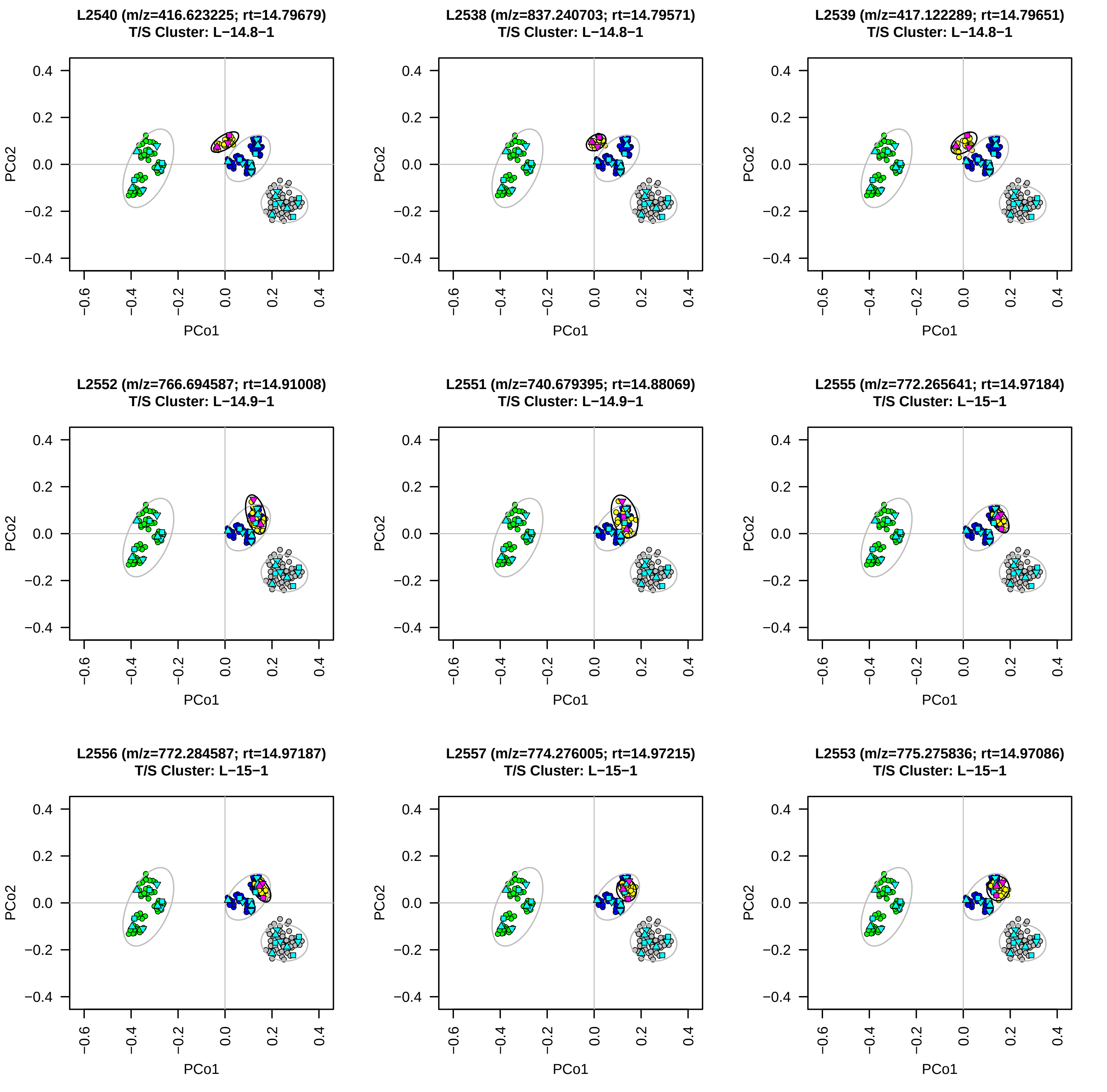
L2540 (m/z=416.623225; rt=14.79679)
T/S Cluster: L−14.8−1
L2538 (m/z=837.240703; rt=14.79571)
T/S Cluster: L−14.8−1
L2539 (m/z=417.122289; rt=14.79651)
T/S Cluster: L−14.8−1
L2552 (m/z=766.694587; rt=14.91008)
T/S Cluster: L−14.9−1
L2551 (m/z=740.679395; rt=14.88069)
T/S Cluster: L−14.9−1
L2555 (m/z=772.265641; rt=14.97184)
T/S Cluster: L−15−1
L2556 (m/z=772.284587; rt=14.97187)
T/S Cluster: L−15−1
L2557 (m/z=774.276005; rt=14.97215)
T/S Cluster: L−15−1
L2553 (m/z=775.275836; rt=14.97086)
T/S Cluster: L−15−1
PCo1
PCo2
0.4
0.2
0.0
−0.2
−0.4
−0.6

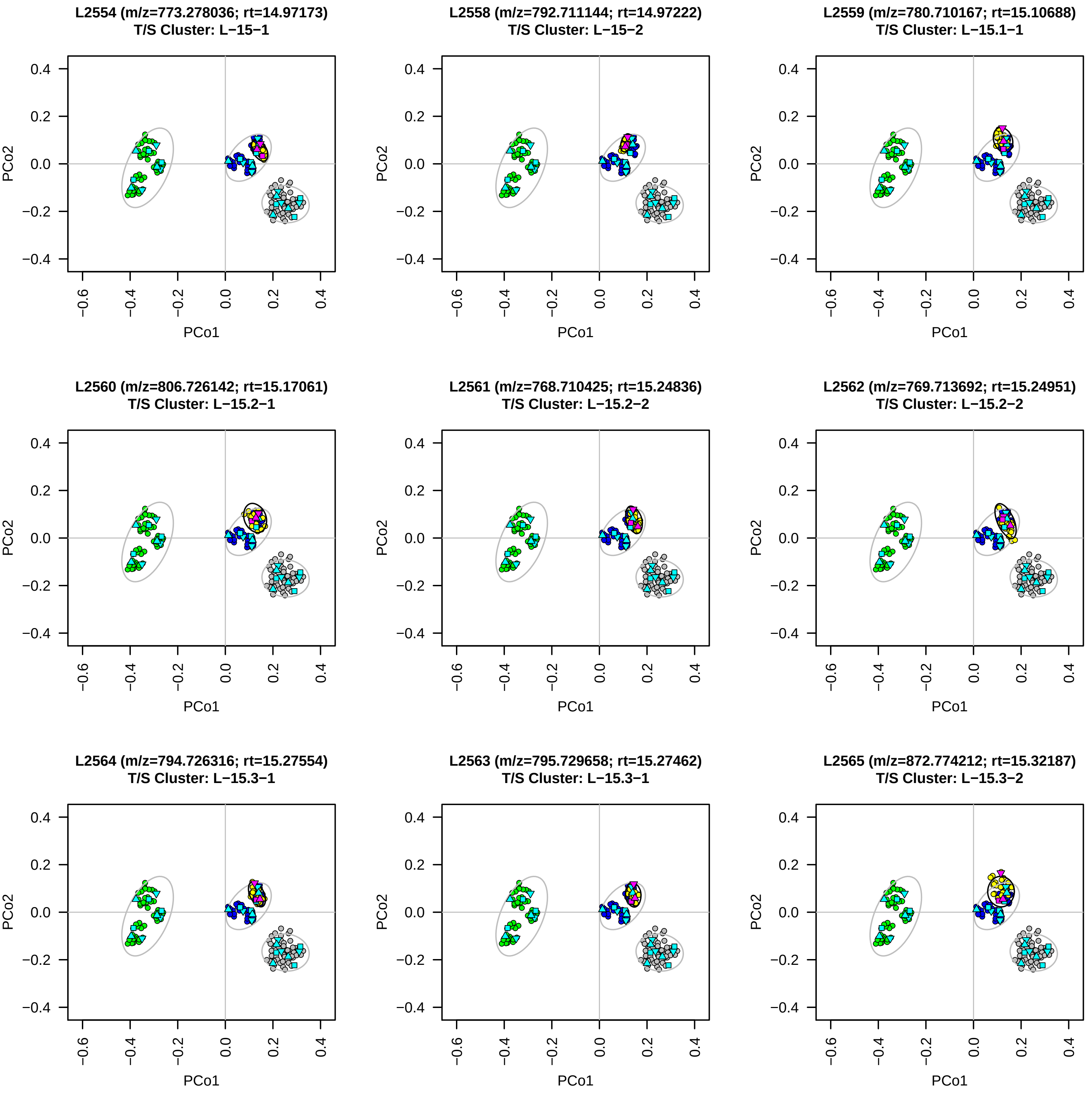
L2554 (m/z=773.278036; rt=14.97173)
T/S Cluster: L−15−1
L2558 (m/z=792.711144; rt=14.97222)
T/S Cluster: L−15−2
L2559 (m/z=780.710167; rt=15.10688)
T/S Cluster: L−15.1−1
L2560 (m/z=806.726142; rt=15.17061)
T/S Cluster: L−15.2−1
L2561 (m/z=768.710425; rt=15.24836)
T/S Cluster: L−15.2−2
L2562 (m/z=769.713692; rt=15.24951)
T/S Cluster: L−15.2−2
L2564 (m/z=794.726316; rt=15.27554)
T/S Cluster: L−15.3−1
L2563 (m/z=795.729658; rt=15.27462)
T/S Cluster: L−15.3−1
L2565 (m/z=872.774212; rt=15.32187)
T/S Cluster: L−15.3−2
PCo1
PCo2
0.4
0.2
0.0
−0.2
−0.4
−0.6

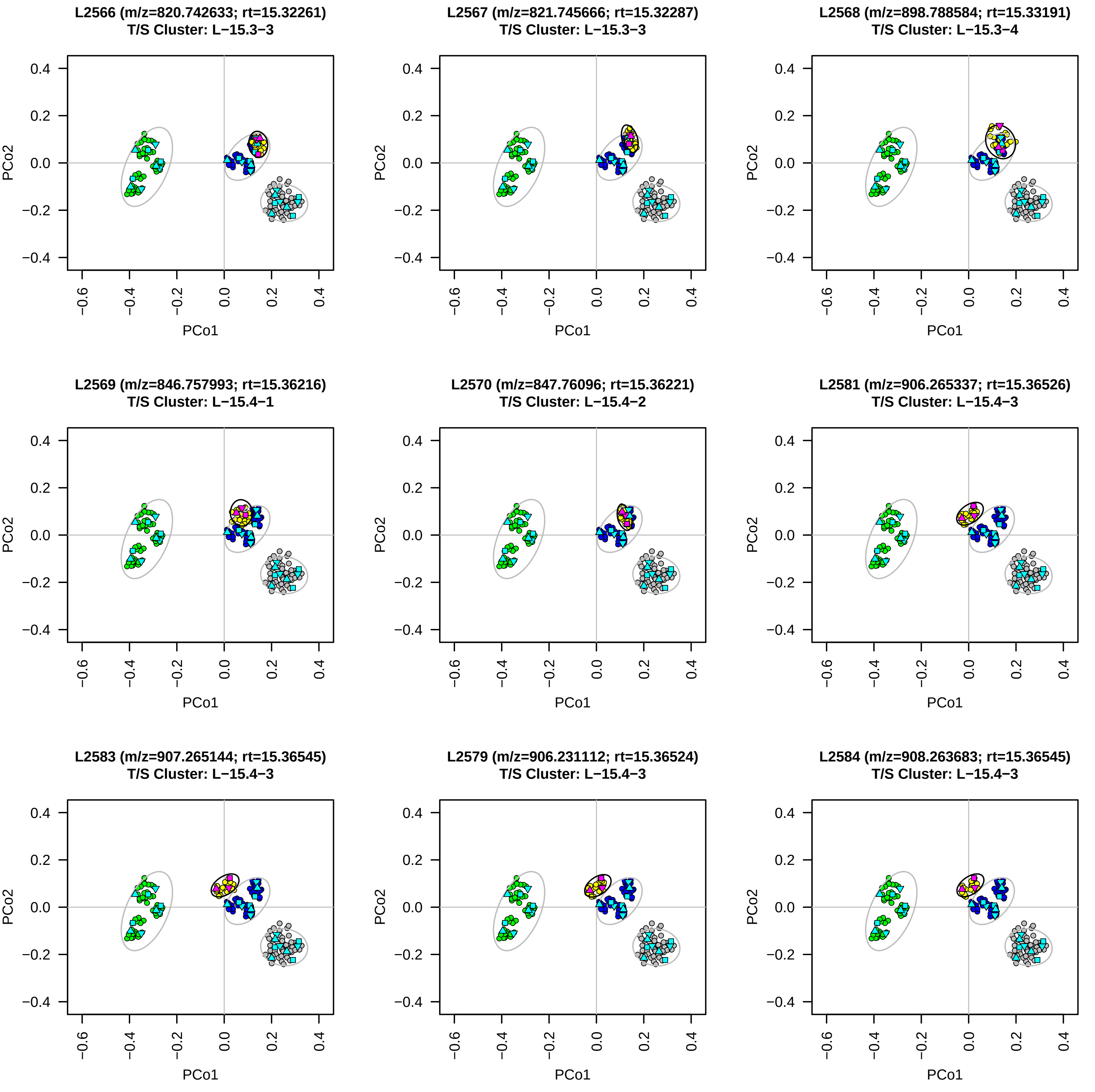
L2566 (m/z=820.742633; rt=15.32261)
T/S Cluster: L−15.3−3
L2567 (m/z=821.745666; rt=15.32287)
T/S Cluster: L−15.3−3
L2568 (m/z=898.788584; rt=15.33191)
T/S Cluster: L−15.3−4
L2569 (m/z=846.757993; rt=15.36216)
T/S Cluster: L−15.4−1
L2570 (m/z=847.76096; rt=15.36221)
T/S Cluster: L−15.4−2
L2581 (m/z=906.265337; rt=15.36526)
T/S Cluster: L−15.4−3
L2583 (m/z=907.265144; rt=15.36545)
T/S Cluster: L−15.4−3
L2579 (m/z=906.231112; rt=15.36524)
T/S Cluster: L−15.4−3
L2584 (m/z=908.263683; rt=15.36545)
T/S Cluster: L−15.4−3
PCo1
PCo2
−0.6
−0.4
−0.2
0.0
0.2
0.4

L2586 (m/z=907.227608; rt=15.36564)
T/S Cluster: L−15.4−3

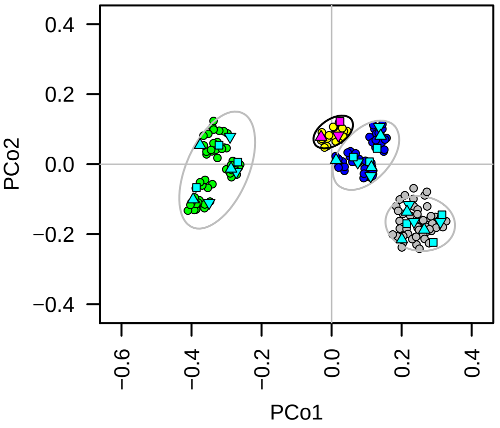

L2585 (m/z=908.225764; rt=15.3655)
T/S Cluster: L−15.4−3

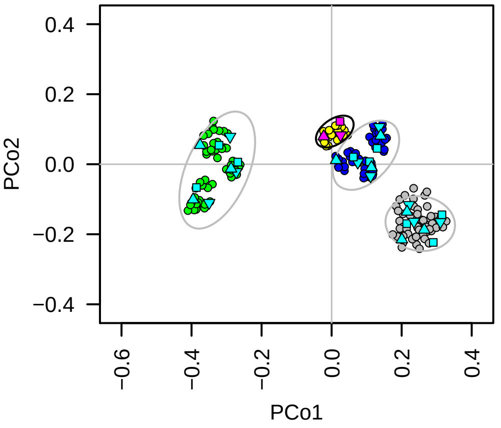

L2580 (m/z=909.269026; rt=15.36525)
T/S Cluster: L−15.4−3

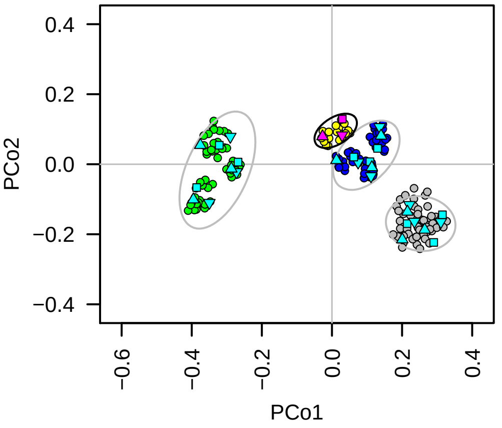

L2582 (m/z=909.241436; rt=15.36536)
T/S Cluster: L−15.4−3

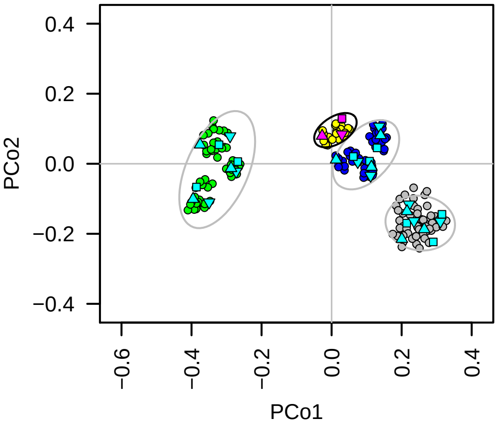

L2587 (m/z=910.260951; rt=15.36564)
T/S Cluster: L−15.4−3

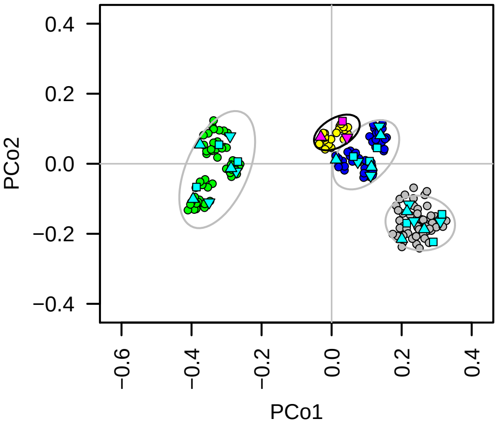

L2577 (m/z=453.130129; rt=15.36479)
T/S Cluster: L−15.4−3

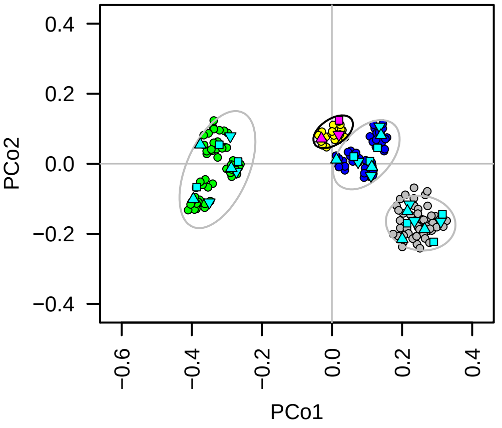

L2576 (m/z=453.137476; rt=15.36476)
T/S Cluster: L−15.4−3

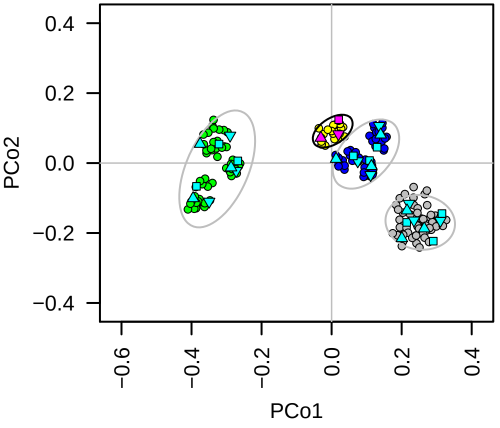

L2575 (m/z=911.259746; rt=15.36475)
T/S Cluster: L−15.4−3

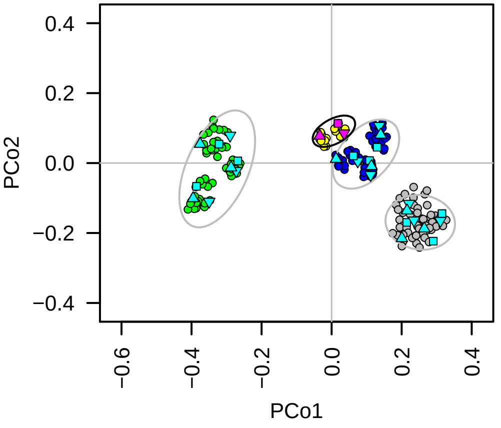

L2574 (m/z=453.633135; rt=15.36469)
T/S Cluster: L−15.4−3

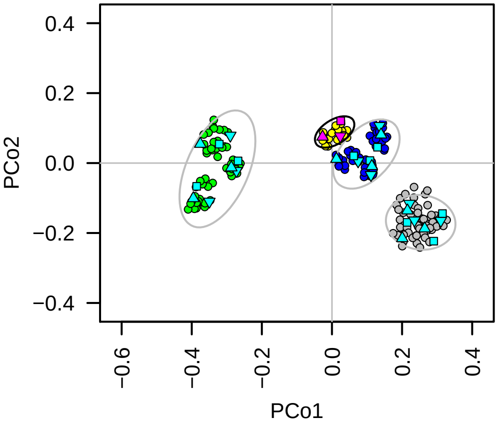

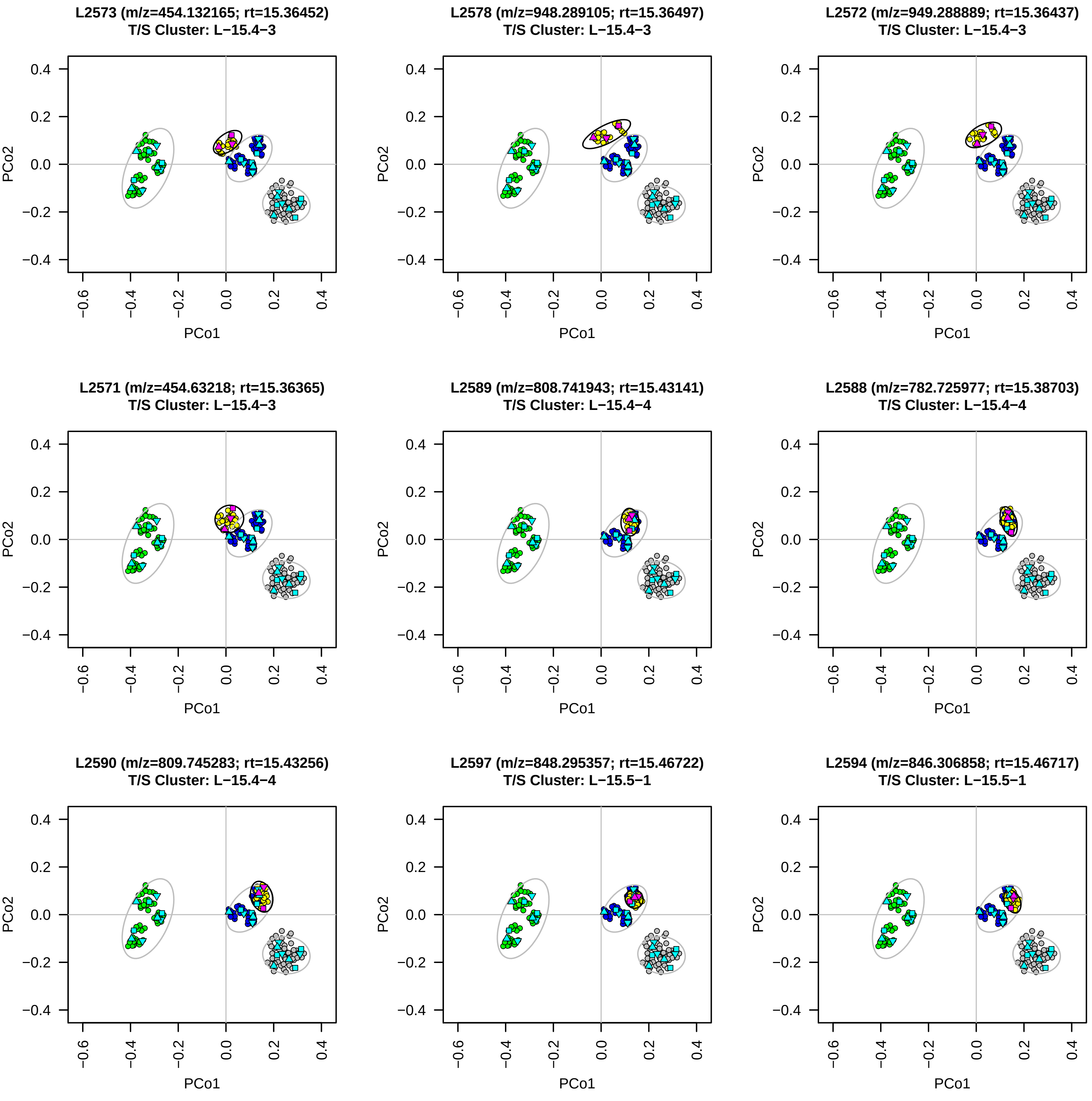
L2573 (m/z=454.132165; rt=15.36452)
T/S Cluster: L−15.4−3
L2578 (m/z=948.289105; rt=15.36497)
T/S Cluster: L−15.4−3
L2572 (m/z=949.288889; rt=15.36437)
T/S Cluster: L−15.4−3
L2571 (m/z=454.63218; rt=15.36365)
T/S Cluster: L−15.4−3
L2589 (m/z=808.741943; rt=15.43141)
T/S Cluster: L−15.4−4
L2588 (m/z=782.725977; rt=15.38703)
T/S Cluster: L−15.4−4
L2590 (m/z=809.745283; rt=15.43256)
T/S Cluster: L−15.4−4
L2597 (m/z=848.295357; rt=15.46722)
T/S Cluster: L−15.5−1
L2594 (m/z=846.306858; rt=15.46717)
T/S Cluster: L−15.5−1
PCo1
PCo2
0.4
0.2
0.0
−0.2
−0.4
−0.6

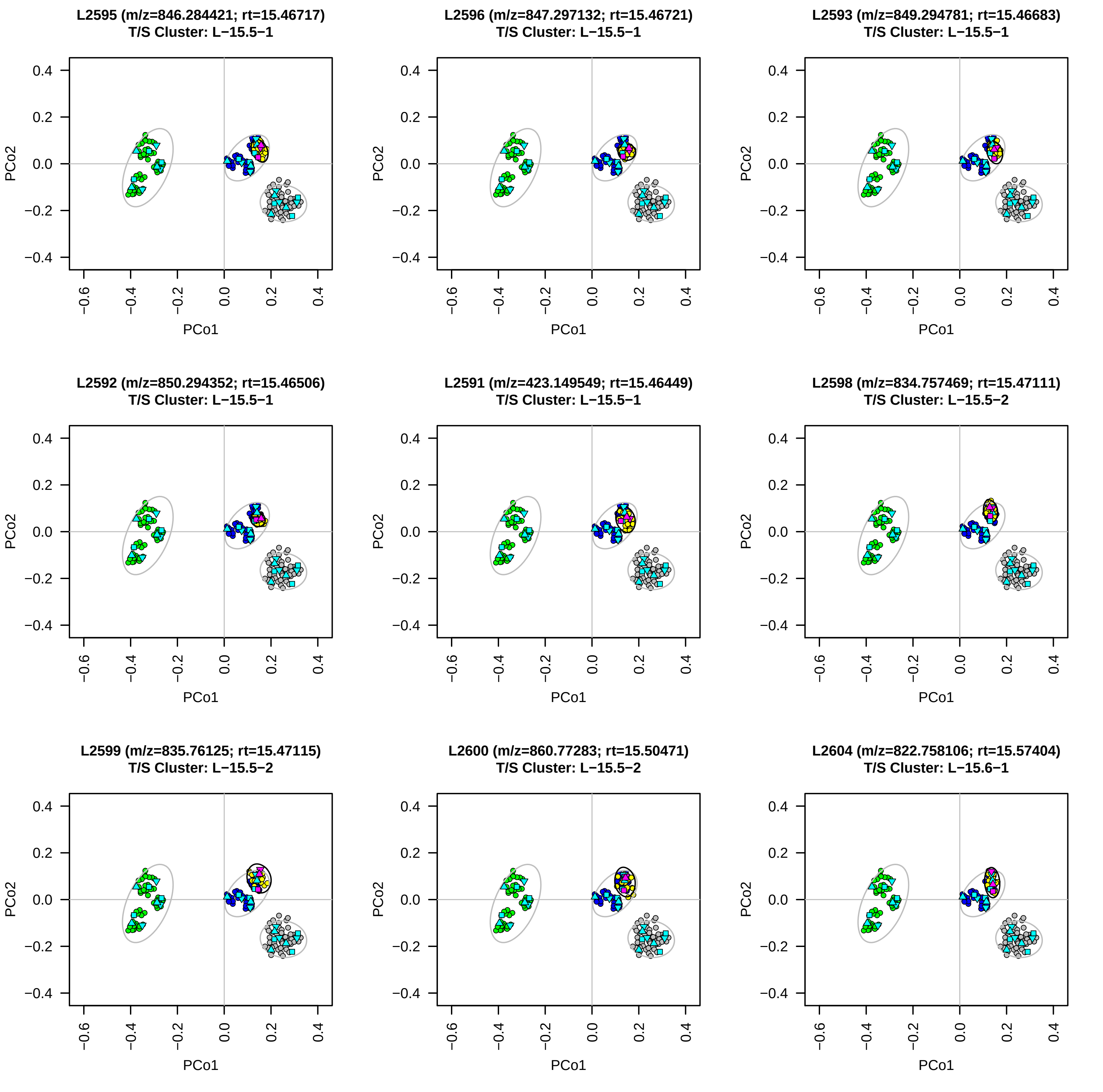
L2595 (m/z=846.284421; rt=15.46717)
T/S Cluster: L−15.5−1
L2596 (m/z=847.297132; rt=15.46721)
T/S Cluster: L−15.5−1
L2593 (m/z=849.294781; rt=15.46683)
T/S Cluster: L−15.5−1
L2592 (m/z=850.294352; rt=15.46506)
T/S Cluster: L−15.5−1
L2591 (m/z=423.149549; rt=15.46449)
T/S Cluster: L−15.5−1
L2598 (m/z=834.757469; rt=15.47111)
T/S Cluster: L−15.5−2
L2599 (m/z=835.76125; rt=15.47115)
T/S Cluster: L−15.5−2
L2600 (m/z=860.77283; rt=15.50471)
T/S Cluster: L−15.5−2
L2604 (m/z=822.758106; rt=15.57404)
T/S Cluster: L−15.6−1
PCo1
PCo2
0.4
0.2
0.0
−0.2
−0.4
−0.6

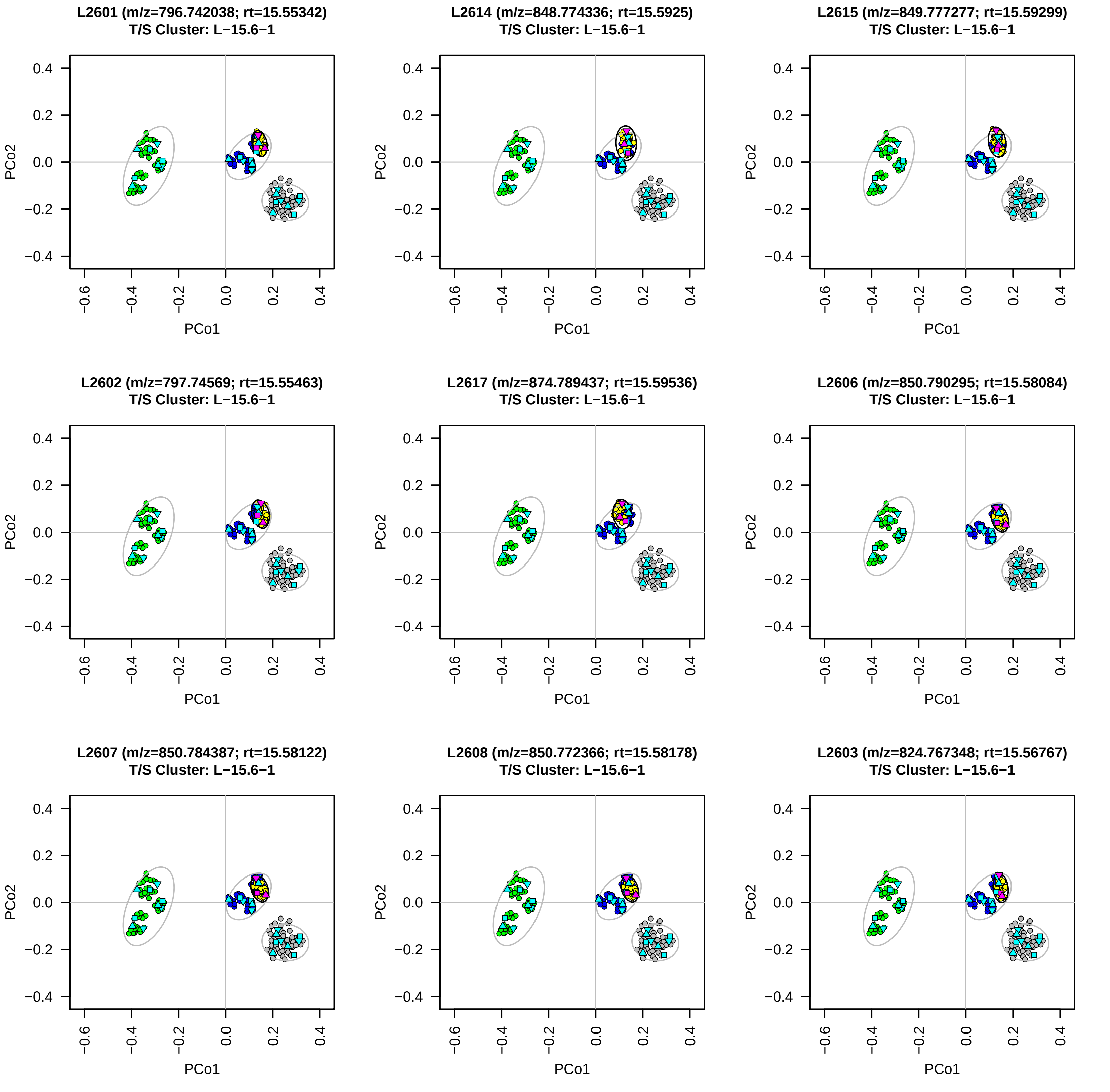

L2601 (m/z=796.742038; rt=15.55342)
T/S Cluster: L−15.6−1
L2614 (m/z=848.774336; rt=15.5925)
T/S Cluster: L−15.6−1
L2615 (m/z=849.777277; rt=15.59299)
T/S Cluster: L−15.6−1
L2602 (m/z=797.74569; rt=15.55463)
T/S Cluster: L−15.6−1
L2617 (m/z=874.789437; rt=15.59536)
T/S Cluster: L−15.6−1
L2606 (m/z=850.790295; rt=15.58084)
T/S Cluster: L−15.6−1
L2607 (m/z=850.784387; rt=15.58122)
T/S Cluster: L−15.6−1
L2608 (m/z=850.772366; rt=15.58178)
T/S Cluster: L−15.6−1
L2603 (m/z=824.767348; rt=15.56767)
T/S Cluster: L−15.6−1
PCo1
PCo2
0.4
0.2
0.0
−0.2
−0.4
−0.6

L2611 (m/z=876.793438; rt=15.58888)
T/S Cluster: L−15.6−1

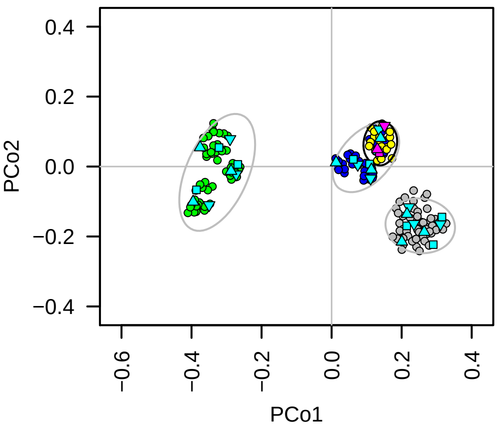

L2612 (m/z=876.800256; rt=15.589)
T/S Cluster: L−15.6−1

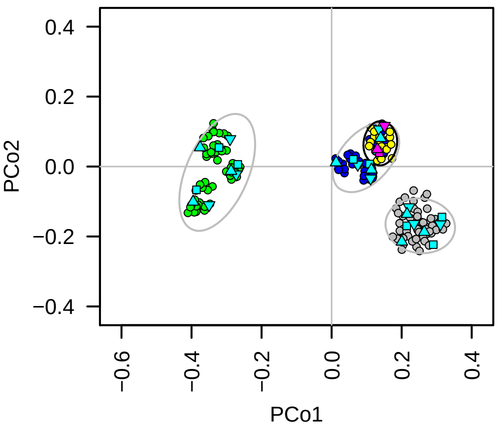

L2613 (m/z=876.801058; rt=15.58918)
T/S Cluster: L−15.6−1

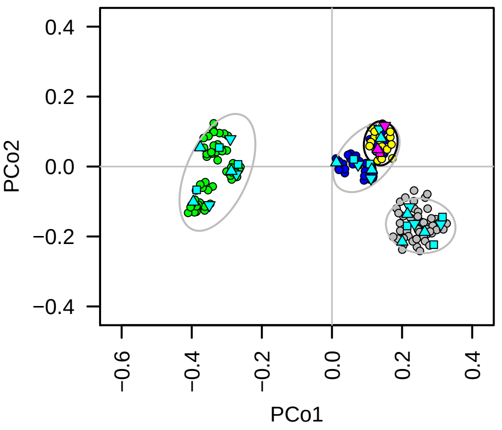

L2605 (m/z=823.761539; rt=15.5744)
T/S Cluster: L−15.6−2

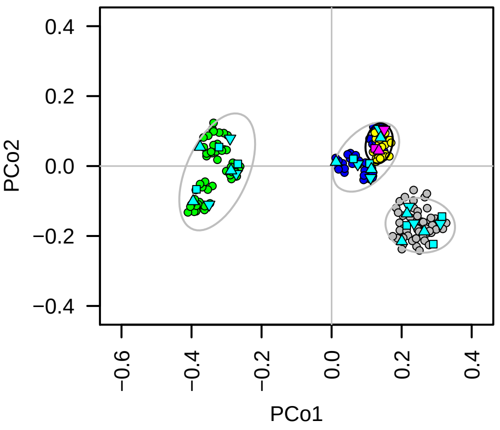

L2610 (m/z=900.804609; rt=15.58792)
T/S Cluster: L−15.6−3

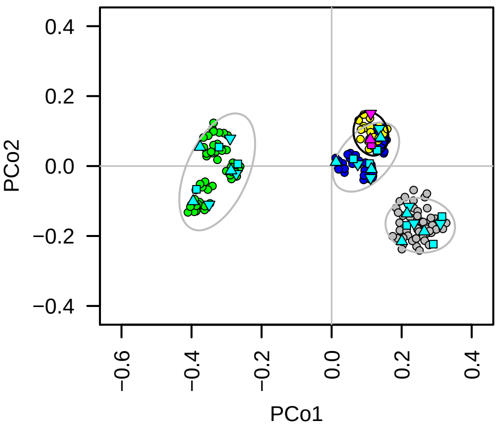

L2609 (m/z=901.808391; rt=15.5867)
T/S Cluster: L−15.6−3

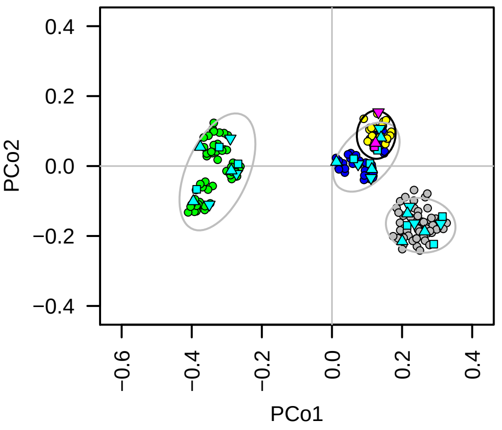

L2616 (m/z=875.792653; rt=15.59453)
T/S Cluster: L−15.6−4

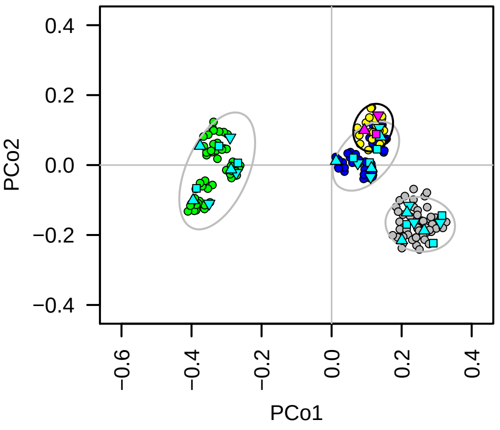

L2618 (m/z=1425.41018; rt=15.60846)
T/S Cluster: L−15.6−5

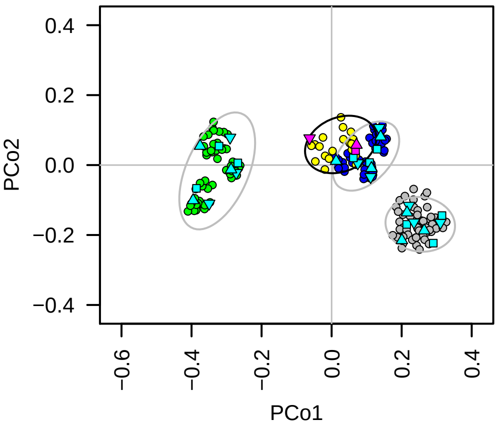

L2622 (m/z=836.773468; rt=15.70573)
T/S Cluster: L−15.7−1

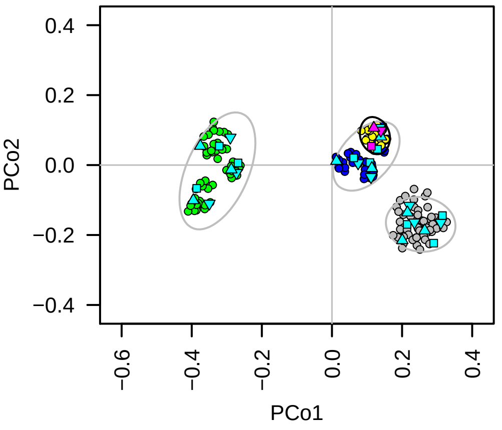

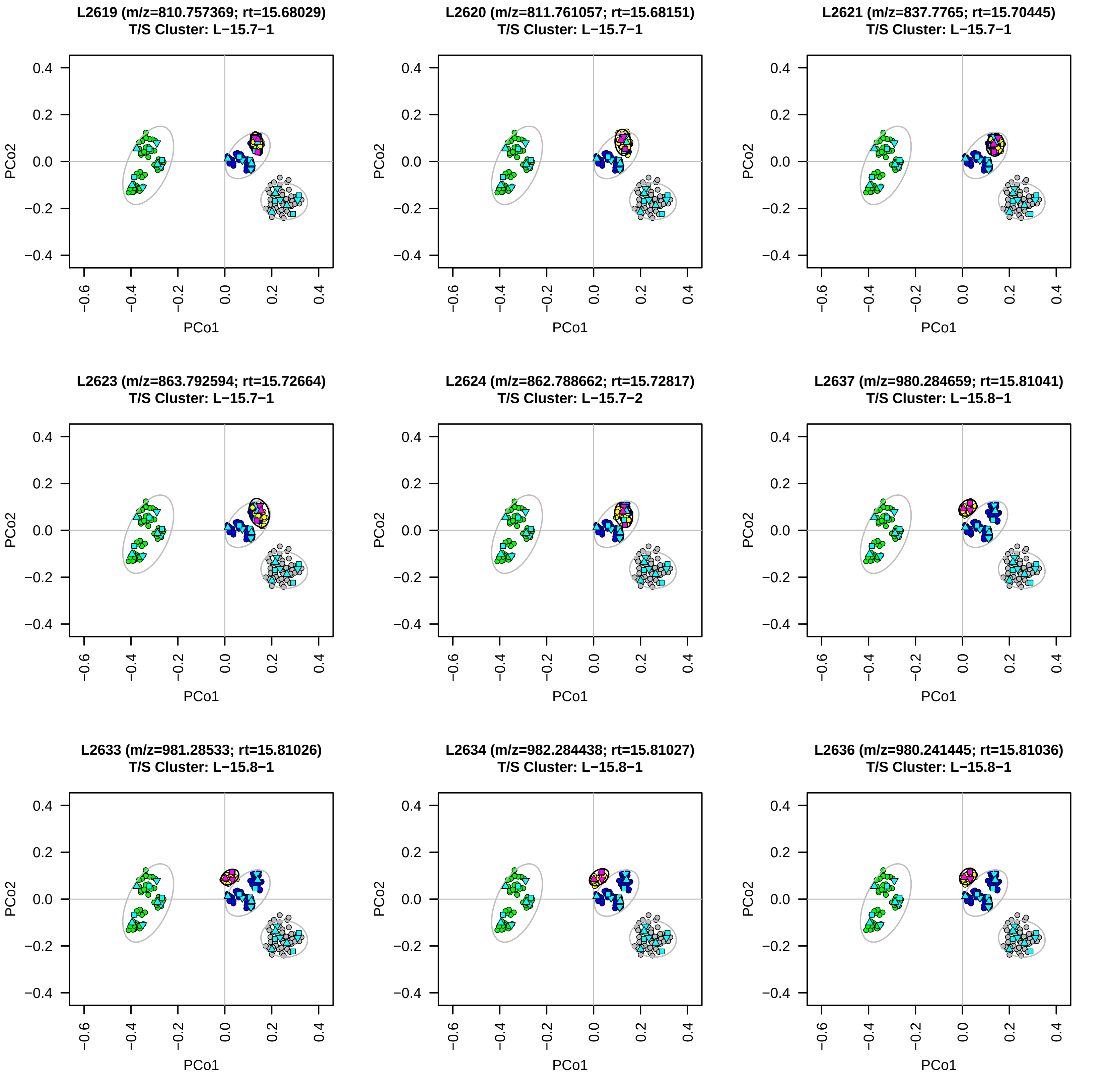
L2619 (m/z=810.757369; rt=15.68029)
T/S Cluster: L−15.7−1
L2620 (m/z=811.761057; rt=15.68151)
T/S Cluster: L−15.7−1
L2621 (m/z=837.7765; rt=15.70445)
T/S Cluster: L−15.7−1
L2623 (m/z=863.792594; rt=15.72664)
T/S Cluster: L−15.7−1
L2624 (m/z=862.788662; rt=15.72817)
T/S Cluster: L−15.7−2
L2637 (m/z=980.284659; rt=15.81041)
T/S Cluster: L−15.8−1
L2633 (m/z=981.28533; rt=15.81026)
T/S Cluster: L−15.8−1
L2634 (m/z=982.284438; rt=15.81027)
T/S Cluster: L−15.8−1
L2636 (m/z=980.241445; rt=15.81036)
T/S Cluster: L−15.8−1
PCo1
PCo2

L2635 (m/z=982.246206; rt=15.81029)
T/S Cluster: L−15.8−1

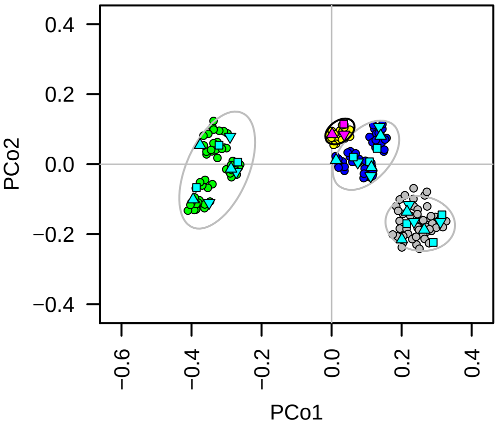

L2632 (m/z=981.243326; rt=15.8102)
T/S Cluster: L−15.8−1

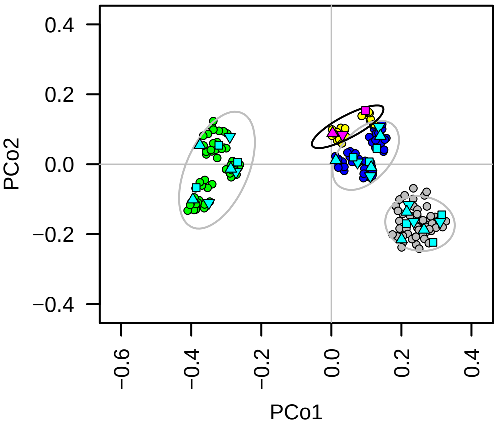

L2630 (m/z=983.286233; rt=15.81018)
T/S Cluster: L−15.8−1

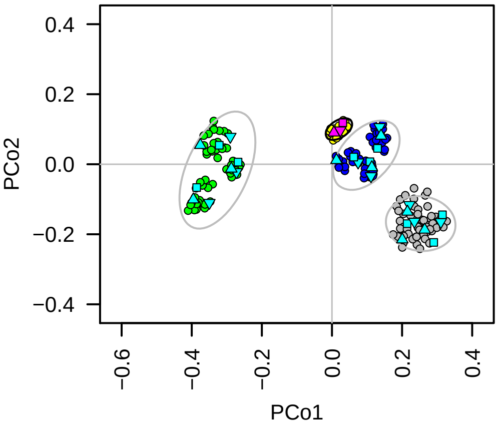

L2631 (m/z=983.251016; rt=15.81019)
T/S Cluster: L−15.8−1

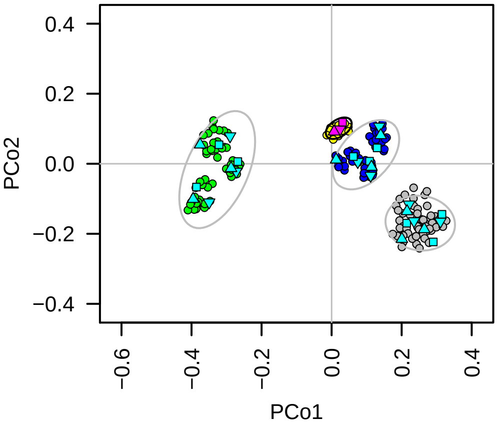

L2638 (m/z=984.280565; rt=15.81061)
T/S Cluster: L−15.8−1

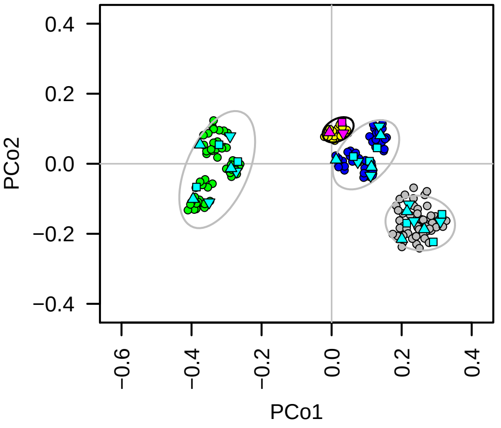

L2629 (m/z=985.280045; rt=15.80964)
T/S Cluster: L−15.8−1

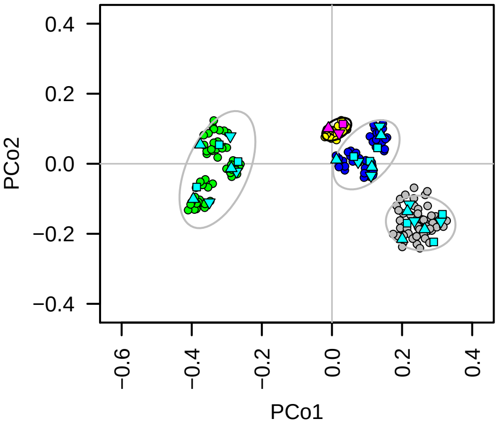

L2628 (m/z=490.142898; rt=15.80961)
T/S Cluster: L−15.8−1

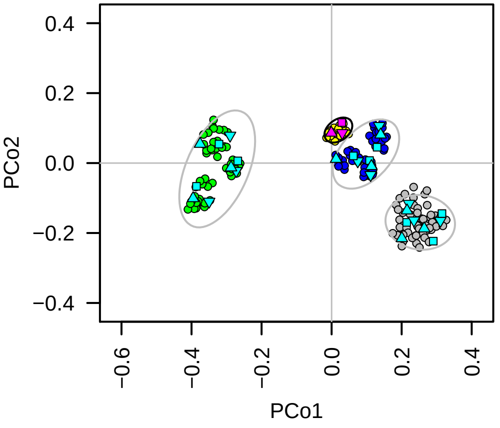

L2627 (m/z=490.643362; rt=15.80954)
T/S Cluster: L−15.8−1

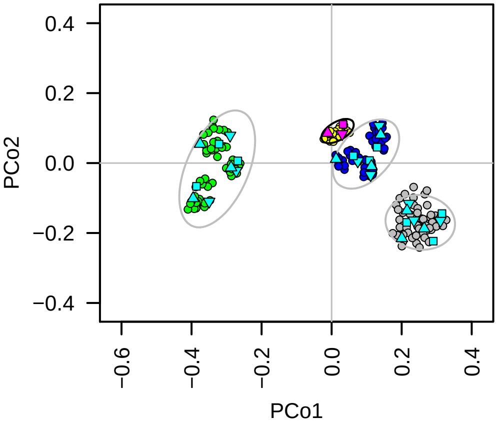

L2626 (m/z=491.142426; rt=15.8093)
T/S Cluster: L−15.8−1

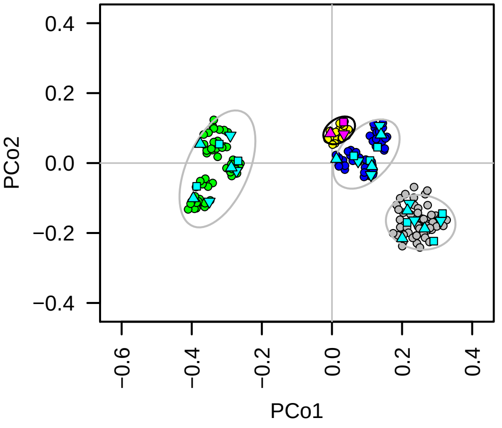

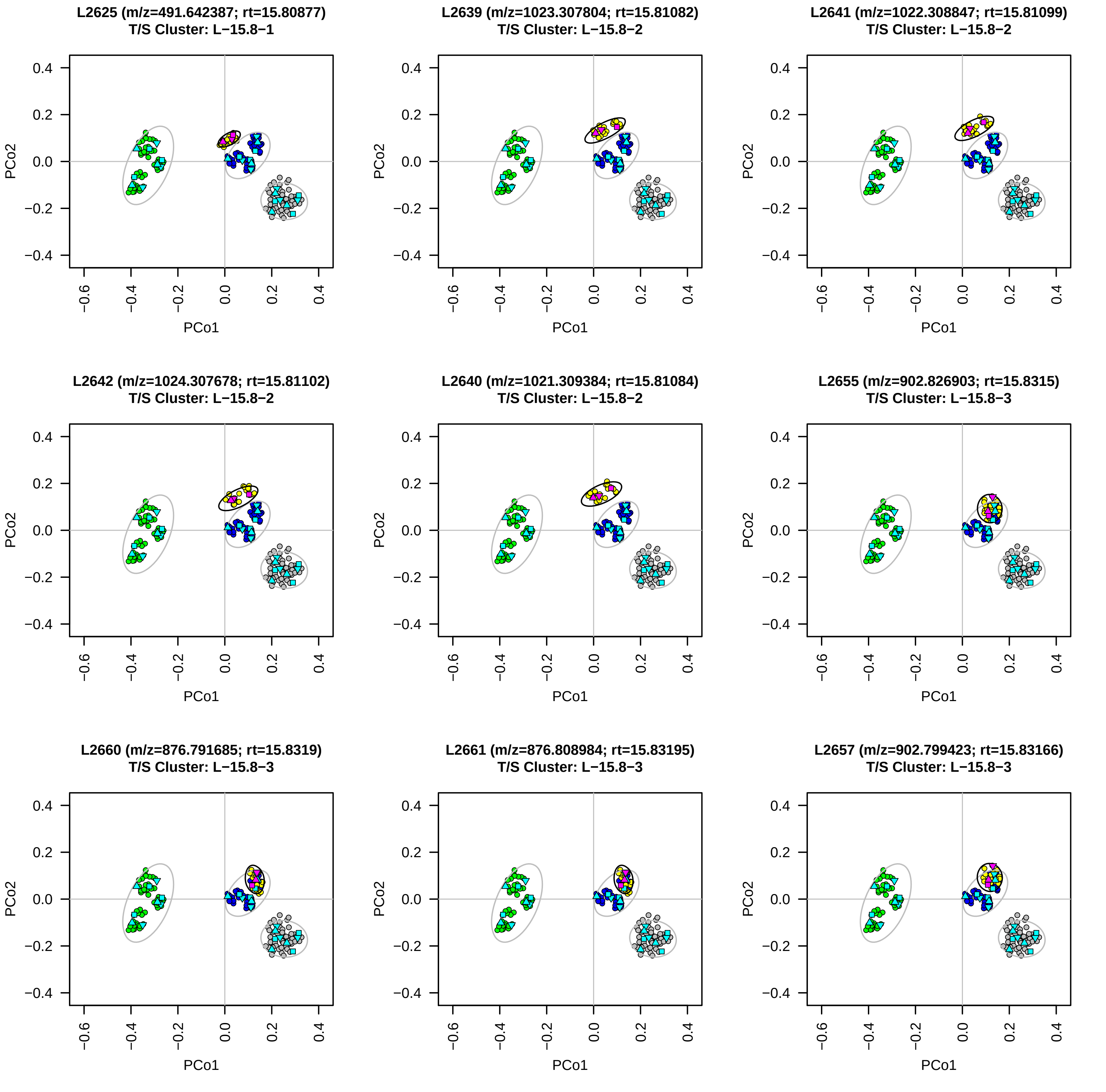
L2625 (m/z=491.642387; rt=15.80877)
T/S Cluster: L−15.8−1
PCo2
PCo1
L2639 (m/z=1023.307804; rt=15.81082)
T/S Cluster: L−15.8−2
PCo2
PCo1
L2641 (m/z=1022.308847; rt=15.81099)
T/S Cluster: L−15.8−2
PCo2
PCo1
L2642 (m/z=1024.307678; rt=15.81102)
T/S Cluster: L−15.8−2
PCo2
PCo1
L2640 (m/z=1021.309384; rt=15.81084)
T/S Cluster: L−15.8−2
PCo2
PCo1
L2655 (m/z=902.826903; rt=15.8315)
T/S Cluster: L−15.8−3
PCo2
PCo1
L2660 (m/z=876.791685; rt=15.8319)
T/S Cluster: L−15.8−3
PCo2
PCo1
L2661 (m/z=876.808984; rt=15.83195)
T/S Cluster: L−15.8−3
PCo2
PCo1
L2657 (m/z=902.799423; rt=15.83166)
T/S Cluster: L−15.8−3
PCo2
PCo1

L2650 (m/z=850.795801; rt=15.82894)
T/S Cluster: L−15.8−3

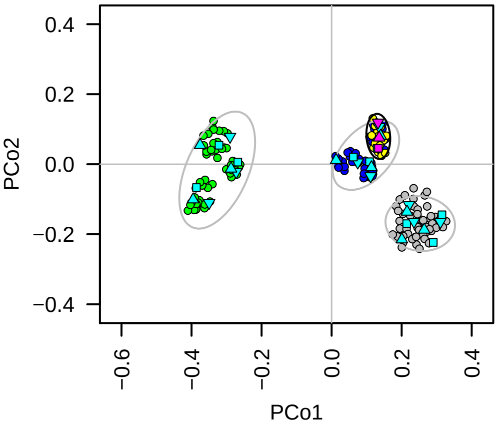

L2651 (m/z=850.771665; rt=15.82902)
T/S Cluster: L−15.8−3

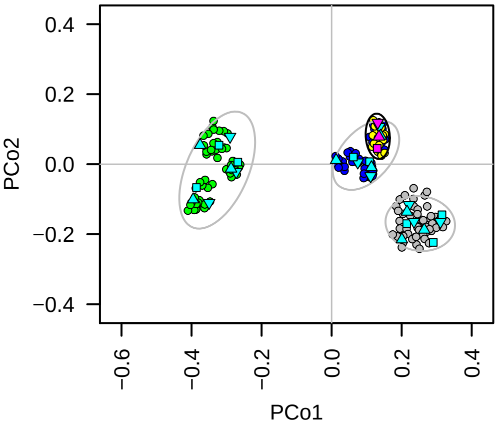

L2656 (m/z=903.824875; rt=15.83162)
T/S Cluster: L−15.8−3

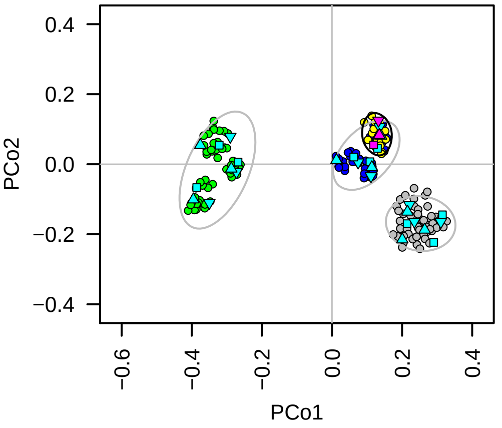

L2658 (m/z=877.797545; rt=15.83183)
T/S Cluster: L−15.8−3

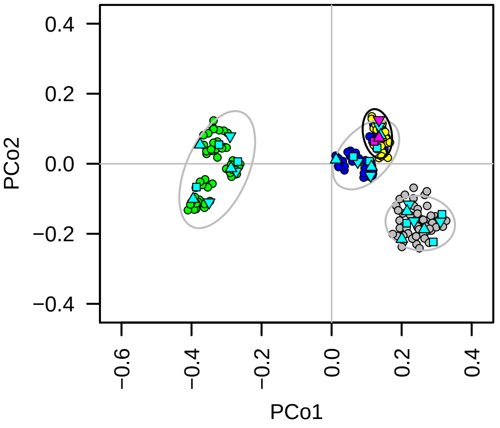

L2659 (m/z=877.820599; rt=15.83185)
T/S Cluster: L−15.8−3

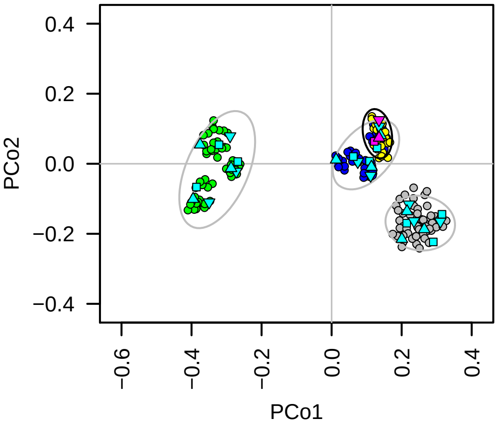

L2643 (m/z=824.774097; rt=15.82282)
T/S Cluster: L−15.8−3

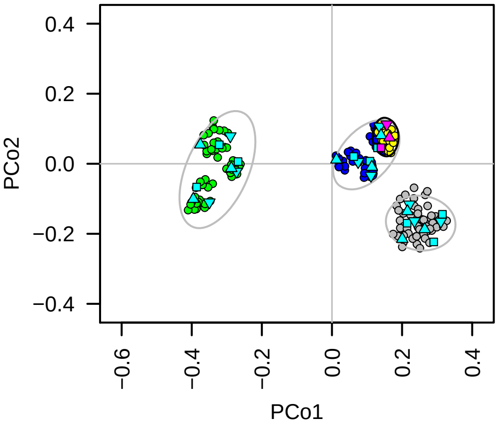

L2648 (m/z=851.784543; rt=15.82865)
T/S Cluster: L−15.8−3

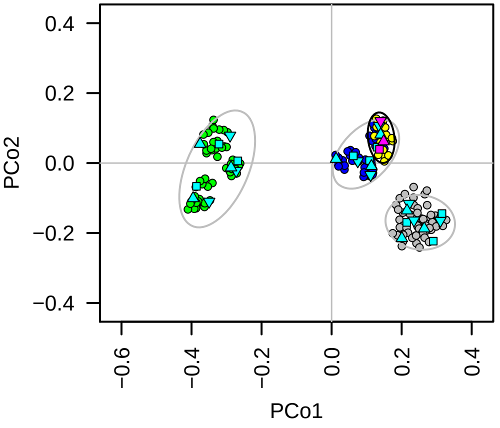

L2649 (m/z=851.806162; rt=15.82873)
T/S Cluster: L−15.8−3

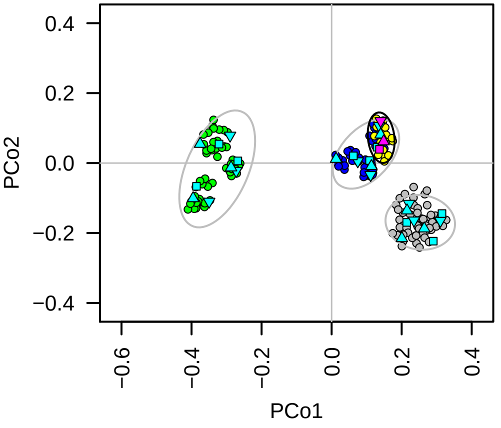

L2644 (m/z=825.777321; rt=15.82297)
T/S Cluster: L−15.8−3

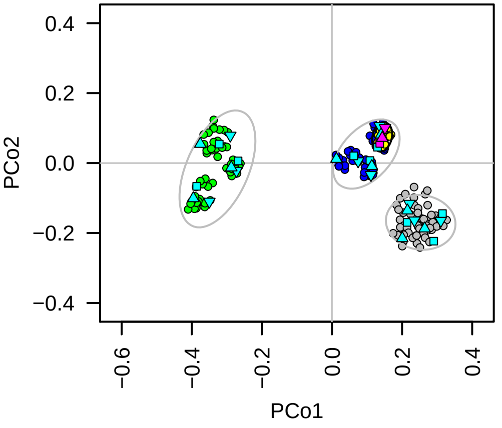

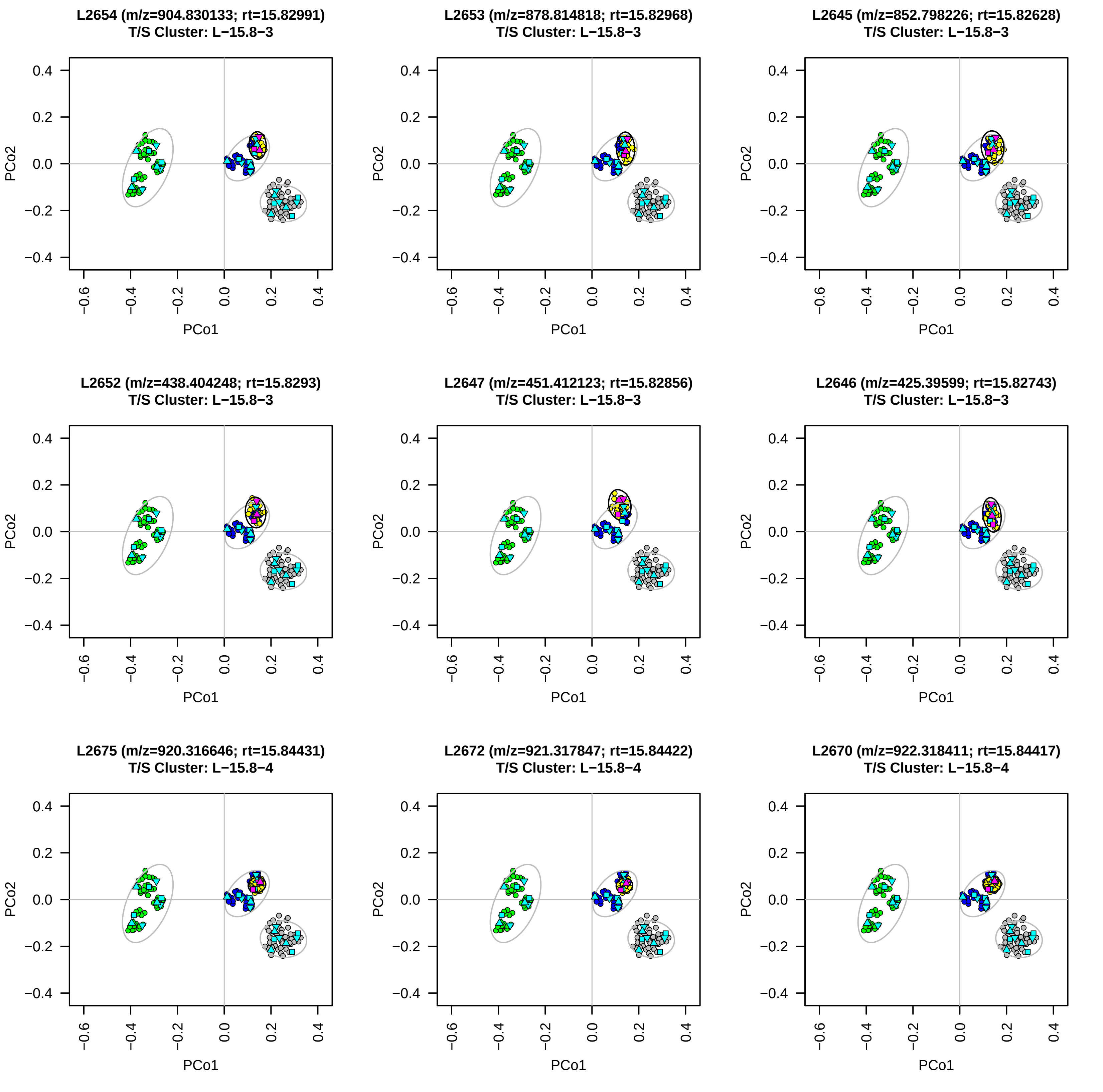

L2654 (m/z=904.830133; rt=15.82991)
T/S Cluster: L−15.8−3
L2653 (m/z=878.814818; rt=15.82968)
T/S Cluster: L−15.8−3
L2645 (m/z=852.798226; rt=15.82628)
T/S Cluster: L−15.8−3
L2652 (m/z=438.404248; rt=15.8293)
T/S Cluster: L−15.8−3
L2647 (m/z=451.412123; rt=15.82856)
T/S Cluster: L−15.8−3
L2646 (m/z=425.39599; rt=15.82743)
T/S Cluster: L−15.8−3
L2675 (m/z=920.316646; rt=15.84431)
T/S Cluster: L−15.8−4
L2672 (m/z=921.317847; rt=15.84422)
T/S Cluster: L−15.8−4
L2670 (m/z=922.318411; rt=15.84417)
T/S Cluster: L−15.8−4
PCo1
PCo2
−0.6
−0.4
−0.2
0.0
0.2
0.4

L2674 (m/z=922.286687; rt=15.84429)
T/S Cluster: L−15.8−4

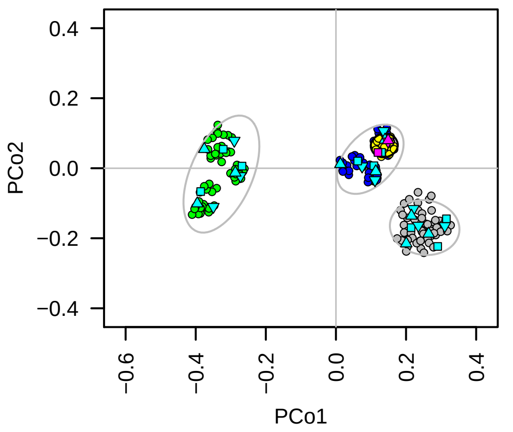

L2679 (m/z=920.274095; rt=15.84454)
T/S Cluster: L−15.8−4

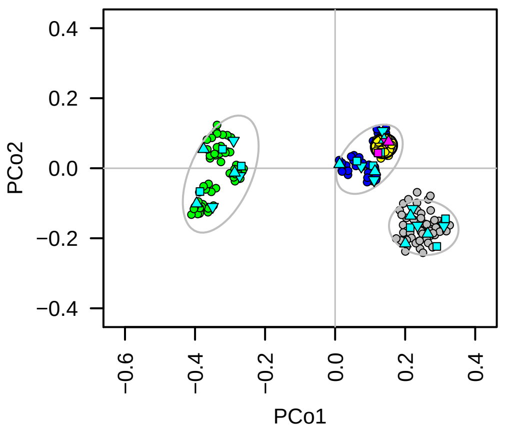

L2677 (m/z=921.27974; rt=15.84438)
T/S Cluster: L−15.8−4

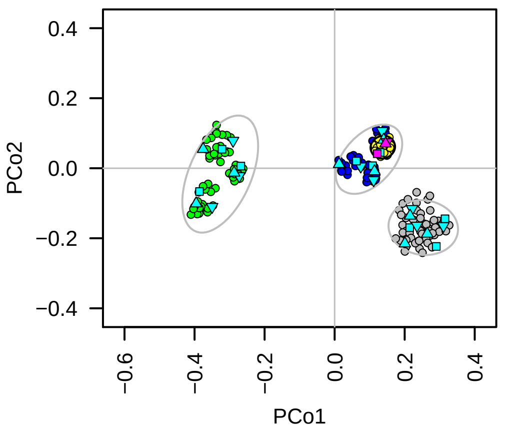

L2676 (m/z=923.294113; rt=15.84434)
T/S Cluster: L−15.8−4

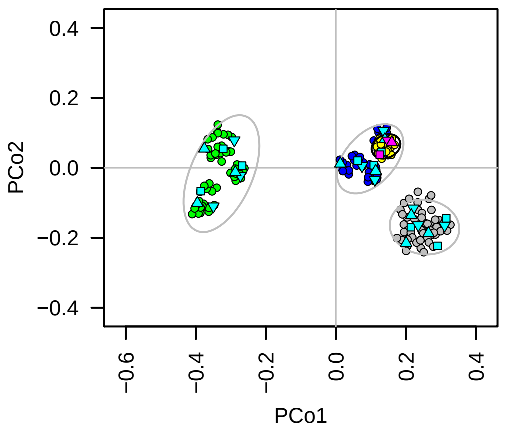

L2671 (m/z=923.321978; rt=15.84418)
T/S Cluster: L−15.8−4

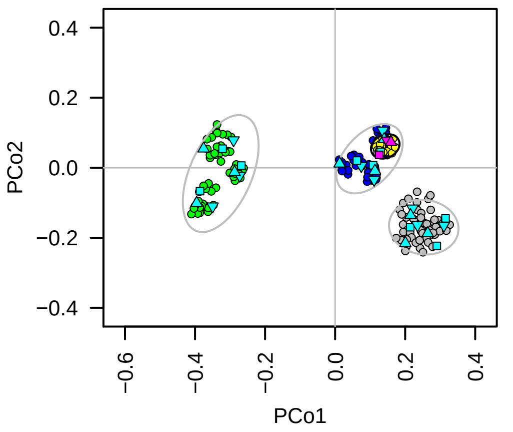

L2678 (m/z=924.313517; rt=15.84451)
T/S Cluster: L−15.8−4

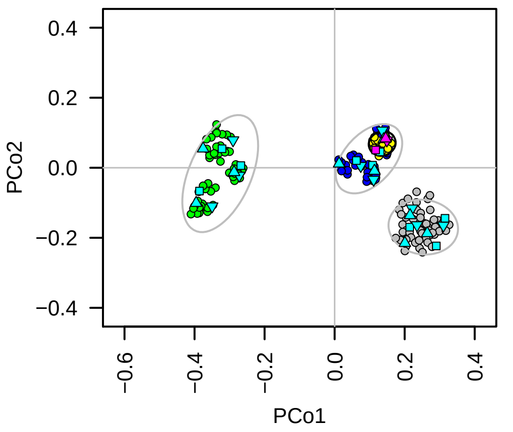

L2668 (m/z=925.312678; rt=15.84396)
T/S Cluster: L−15.8−4

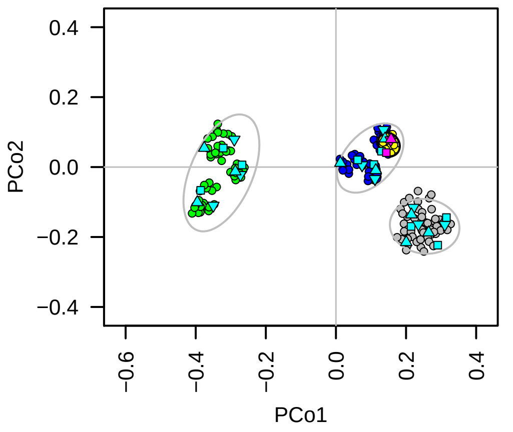

L2673 (m/z=460.164306; rt=15.84426)
T/S Cluster: L−15.8−4

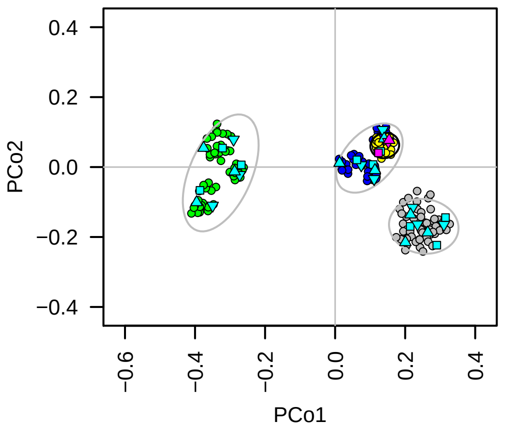

L2680 (m/z=460.156474; rt=15.84455)
T/S Cluster: L−15.8−4

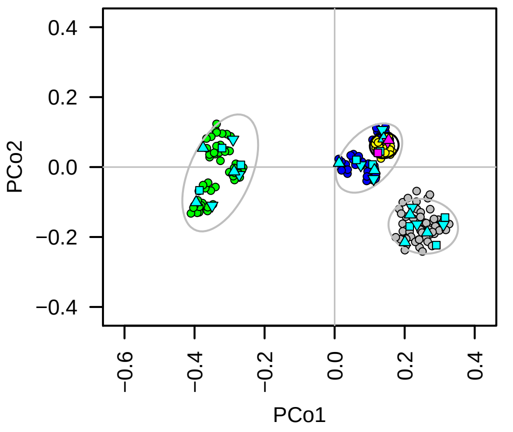

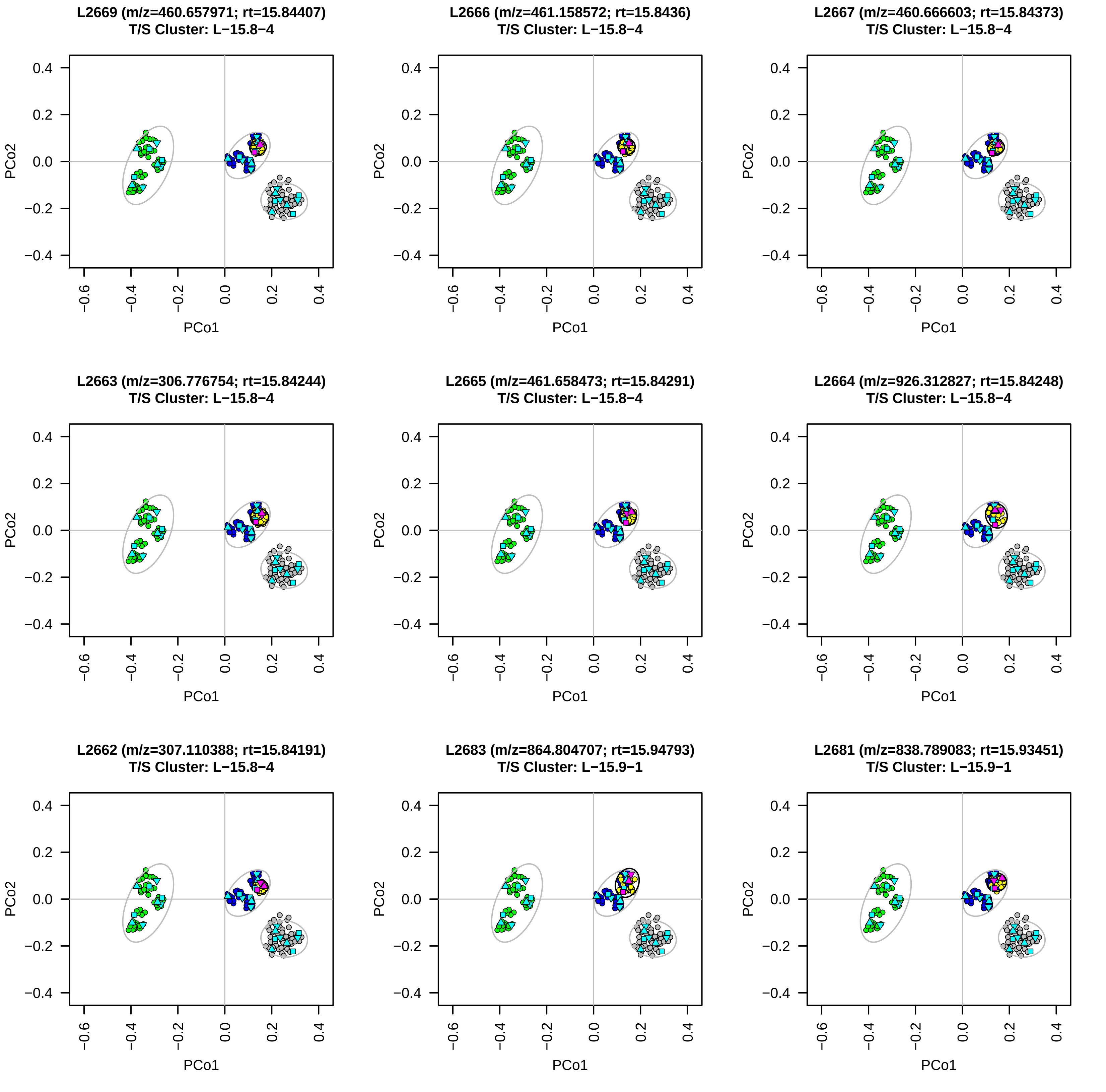
L2669 (m/z=460.657971; rt=15.84407)
T/S Cluster: L−15.8−4
L2666 (m/z=461.158572; rt=15.8436)
T/S Cluster: L−15.8−4
L2667 (m/z=460.666603; rt=15.84373)
T/S Cluster: L−15.8−4
L2663 (m/z=306.776754; rt=15.84244)
T/S Cluster: L−15.8−4
L2665 (m/z=461.658473; rt=15.84291)
T/S Cluster: L−15.8−4
L2664 (m/z=926.312827; rt=15.84248)
T/S Cluster: L−15.8−4
L2662 (m/z=307.110388; rt=15.84191)
T/S Cluster: L−15.8−4
L2683 (m/z=864.804707; rt=15.94793)
T/S Cluster: L−15.9−1
L2681 (m/z=838.789083; rt=15.93451)
T/S Cluster: L−15.9−1
PCo1
PCo2
−0.6 −0.4 −0.2 0.0 0.2 0.4
−0.4 −0.2 0.0 0.2 0.4

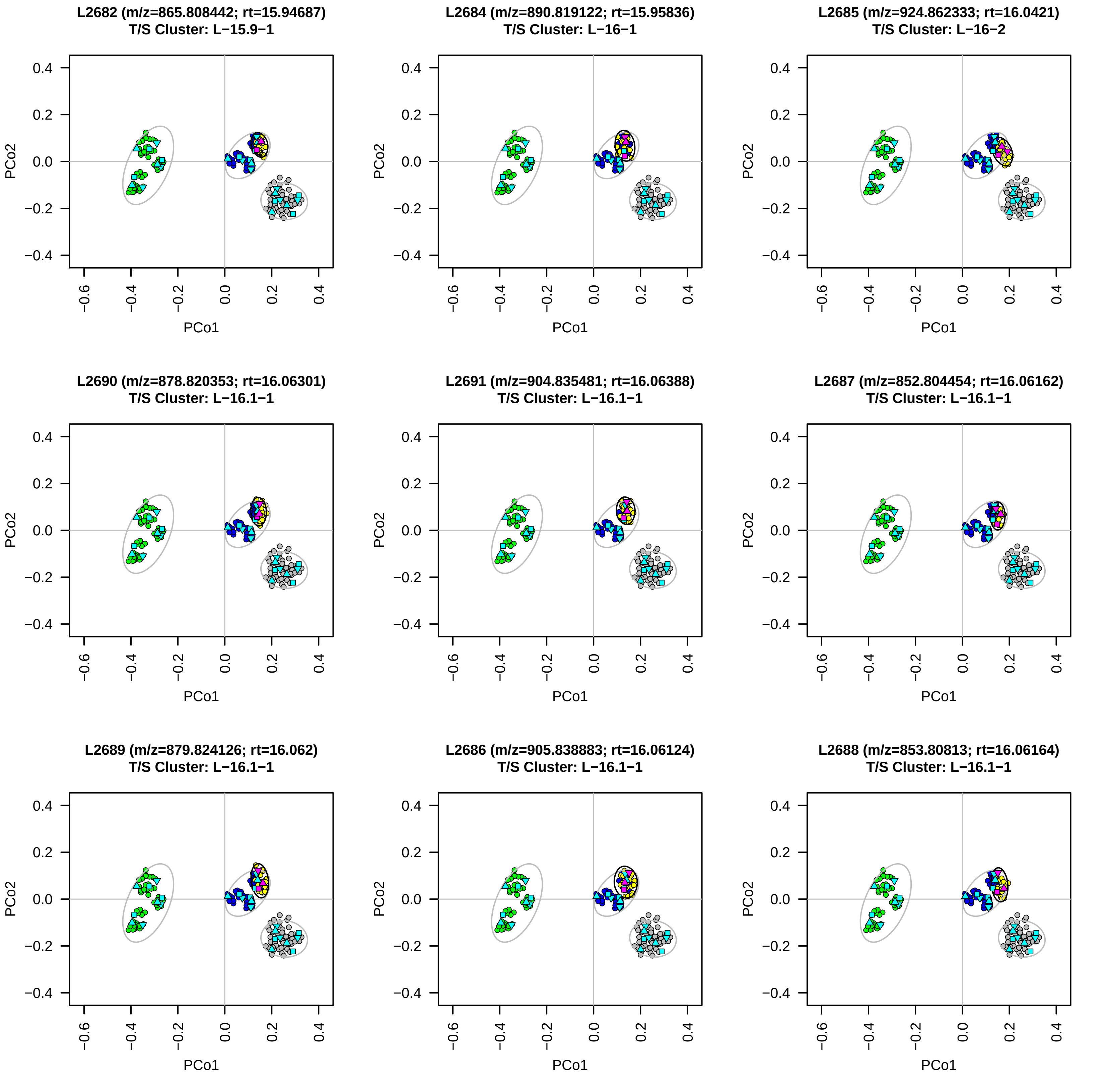
L2682 (m/z=865.808442; rt=15.94687)
T/S Cluster: L−15.9−1
L2684 (m/z=890.819122; rt=15.95836)
T/S Cluster: L−16−1
L2685 (m/z=924.862333; rt=16.0421)
T/S Cluster: L−16−2
L2690 (m/z=878.820353; rt=16.06301)
T/S Cluster: L−16.1−1
L2691 (m/z=904.835481; rt=16.06388)
T/S Cluster: L−16.1−1
L2687 (m/z=852.804454; rt=16.06162)
T/S Cluster: L−16.1−1
L2689 (m/z=879.824126; rt=16.062)
T/S Cluster: L−16.1−1
L2686 (m/z=905.838883; rt=16.06124)
T/S Cluster: L−16.1−1
L2688 (m/z=853.80813; rt=16.06164)
T/S Cluster: L−16.1−1
PCo1
PCo2
0.4
0.2
0.0
−0.2
−0.4
−0.6

L2700 (m/z=995.338274; rt=16.15619)
T/S Cluster: L−16.2−1

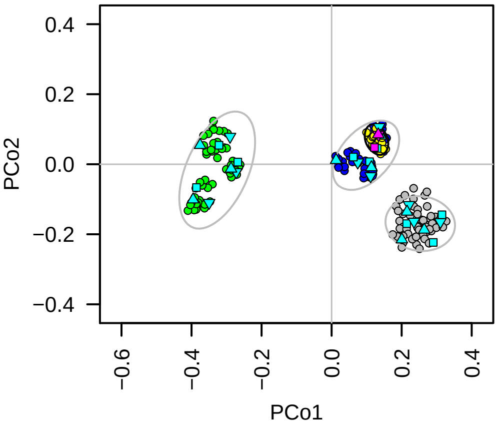

L2703 (m/z=996.339399; rt=16.1563)
T/S Cluster: L−16.2−1

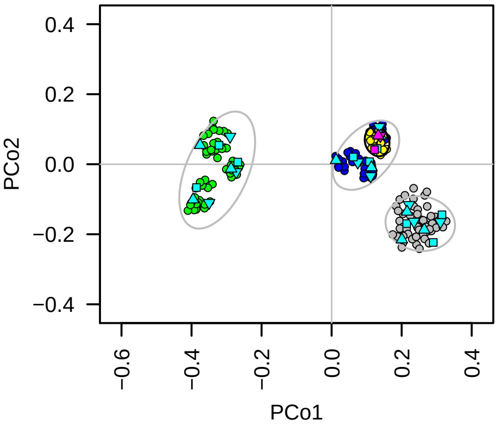

L2702 (m/z=994.342891; rt=16.15623)
T/S Cluster: L−16.2−1

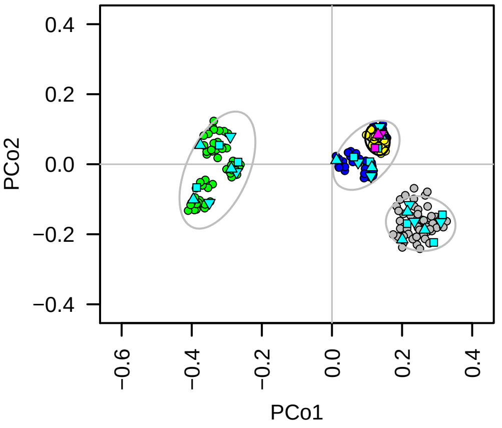

L2698 (m/z=994.309468; rt=16.15615)
T/S Cluster: L−16.2−1

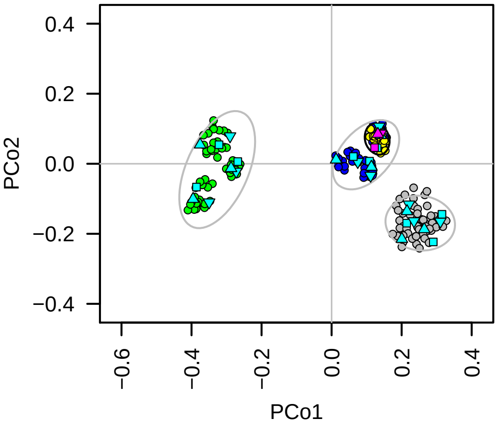

L2699 (m/z=995.297071; rt=16.15616)
T/S Cluster: L−16.2−1

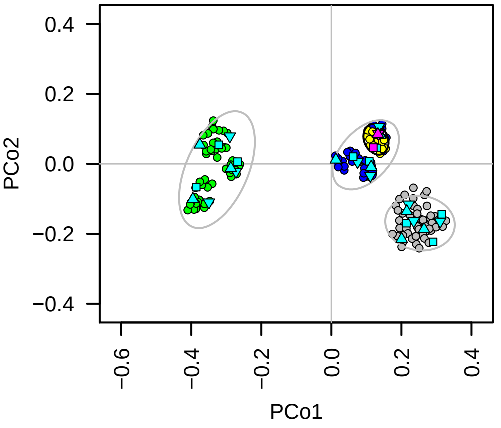

L2701 (m/z=996.303748; rt=16.15622)
T/S Cluster: L−16.2−1

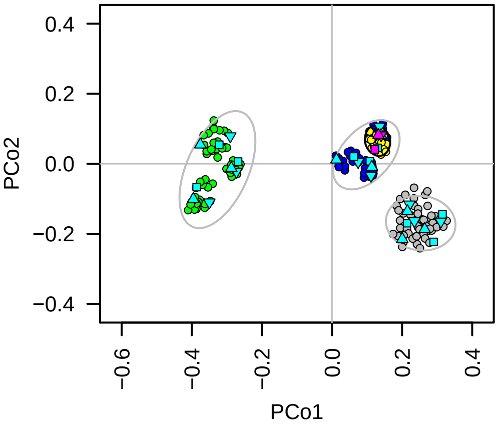

L2705 (m/z=997.311782; rt=16.15636)
T/S Cluster: L−16.2−1

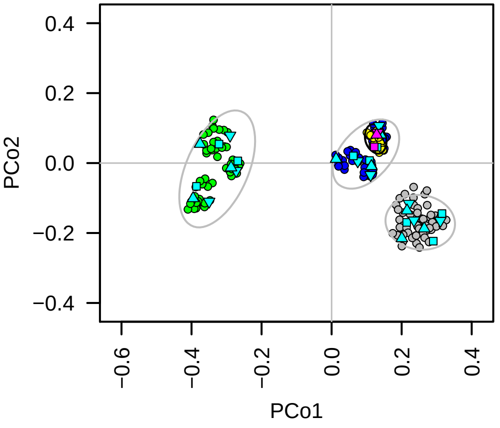

L2706 (m/z=997.343689; rt=16.1564)
T/S Cluster: L−16.2−1

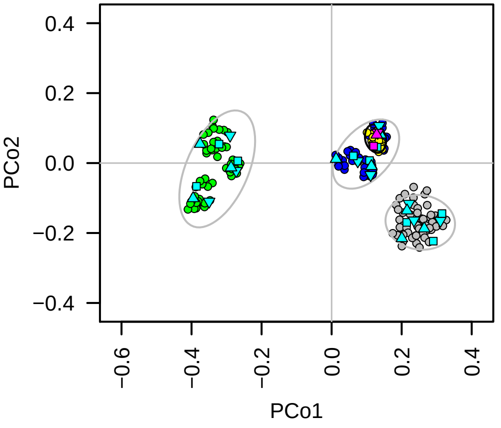

L2704 (m/z=998.332735; rt=16.15635)
T/S Cluster: L−16.2−1

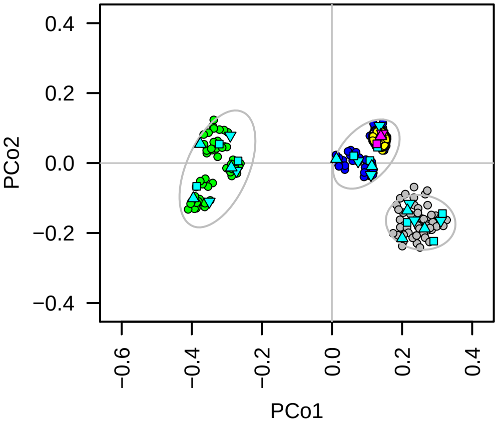

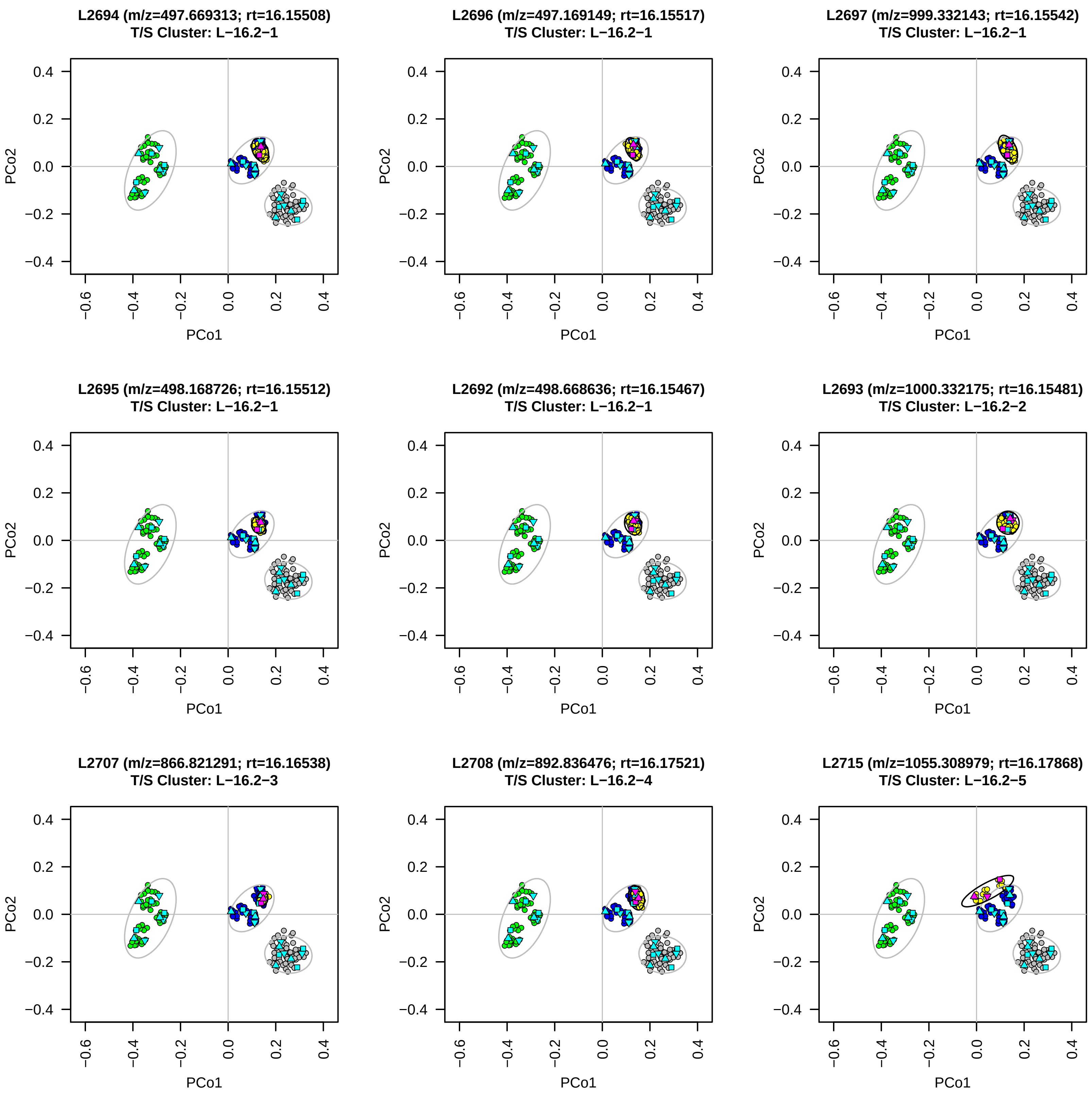
L2694 (m/z=497.669313; rt=16.15508)
T/S Cluster: L−16.2−1
L2696 (m/z=497.169149; rt=16.15517)
T/S Cluster: L−16.2−1
L2697 (m/z=999.332143; rt=16.15542)
T/S Cluster: L−16.2−1
L2695 (m/z=498.168726; rt=16.15512)
T/S Cluster: L−16.2−1
L2692 (m/z=498.668636; rt=16.15467)
T/S Cluster: L−16.2−1
L2693 (m/z=1000.332175; rt=16.15481)
T/S Cluster: L−16.2−2
L2707 (m/z=866.821291; rt=16.16538)
T/S Cluster: L−16.2−3
L2708 (m/z=892.836476; rt=16.17521)
T/S Cluster: L−16.2−4
L2715 (m/z=1055.308979; rt=16.17868)
T/S Cluster: L−16.2−5
PCo1
PCo2
−0.6
−0.4
−0.2
0.0
0.2
0.4

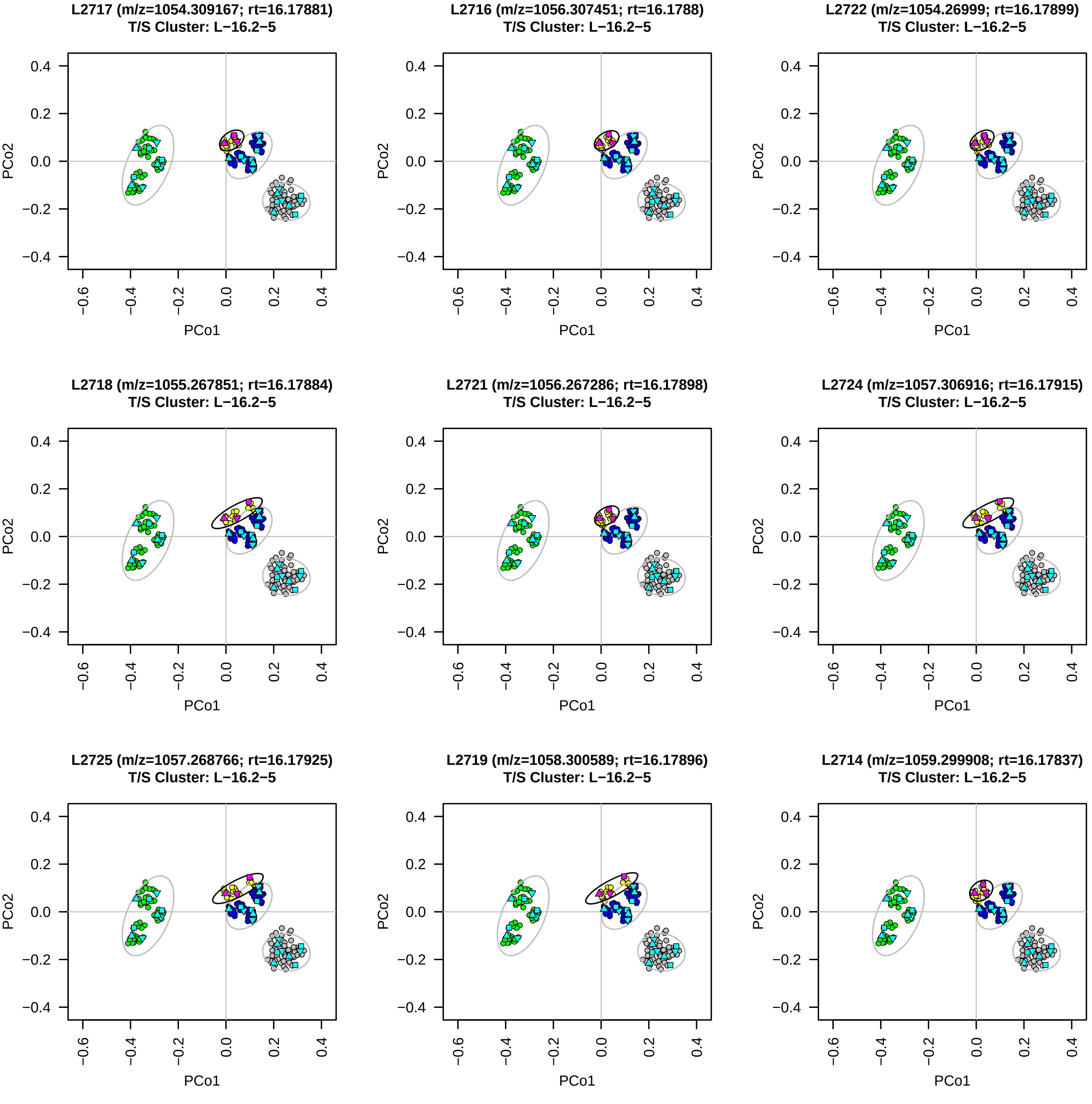

L2717 (m/z=1054.309167; rt=16.17881)
T/S Cluster: L−16.2−5
PCo2
PCo1
L2716 (m/z=1056.307451; rt=16.1788)
T/S Cluster: L−16.2−5
PCo2
PCo1
L2722 (m/z=1054.26999; rt=16.17899)
T/S Cluster: L−16.2−5
PCo2
PCo1
L2718 (m/z=1055.267851; rt=16.17884)
T/S Cluster: L−16.2−5
PCo2
PCo1
L2721 (m/z=1056.267286; rt=16.17898)
T/S Cluster: L−16.2−5
PCo2
PCo1
L2724 (m/z=1057.306916; rt=16.17915)
T/S Cluster: L−16.2−5
PCo2
PCo1
L2725 (m/z=1057.268766; rt=16.17925)
T/S Cluster: L−16.2−5
PCo2
PCo1
L2719 (m/z=1058.300589; rt=16.17896)
T/S Cluster: L−16.2−5
PCo2
PCo1
L2714 (m/z=1059.299908; rt=16.17837)
T/S Cluster: L−16.2−5
PCo2
PCo1

L2712 (m/z=527.152642; rt=16.17782)
T/S Cluster: L−16.2−5

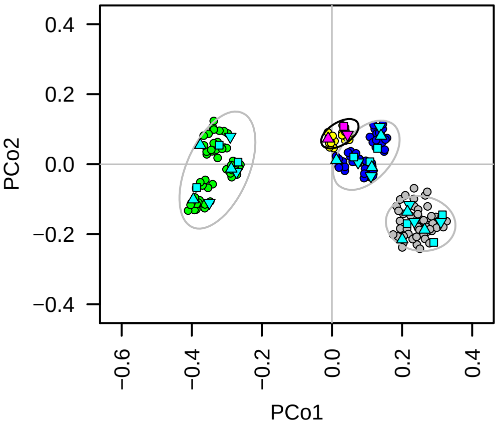

L2710 (m/z=527.653239; rt=16.17764)
T/S Cluster: L−16.2−5

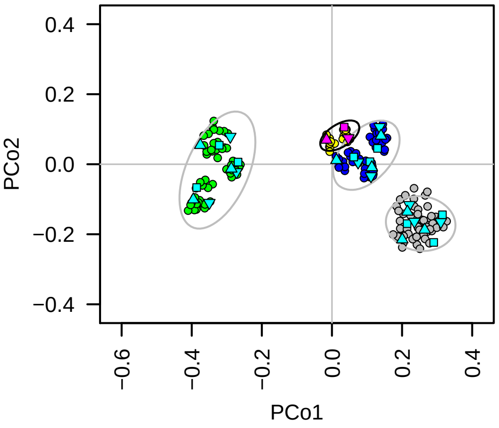

L2711 (m/z=528.152414; rt=16.1777)
T/S Cluster: L−16.2−5

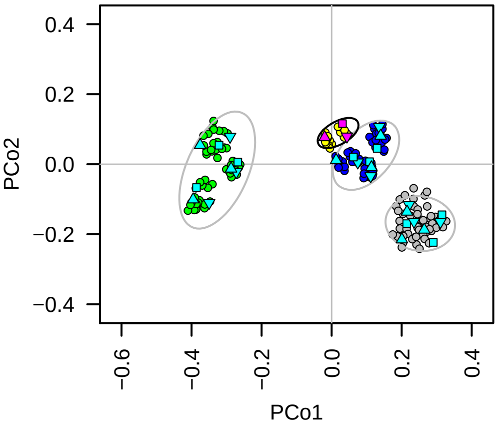

L2713 (m/z=1060.299516; rt=16.17787)
T/S Cluster: L−16.2−5

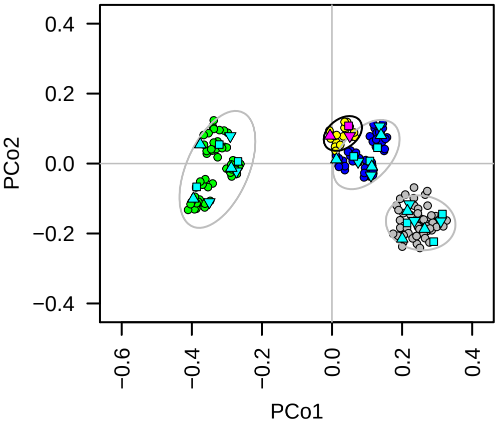

L2709 (m/z=528.652767; rt=16.17727)
T/S Cluster: L−16.2−5

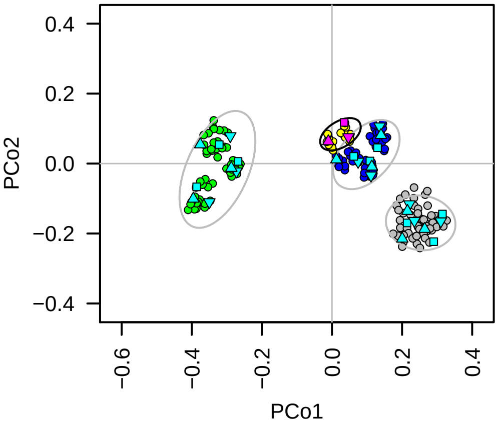

L2726 (m/z=1096.329156; rt=16.17966)
T/S Cluster: L−16.2−6

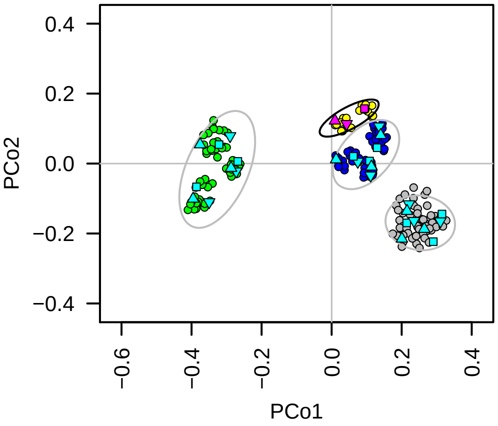

L2727 (m/z=1097.328083; rt=16.18005)
T/S Cluster: L−16.2−6

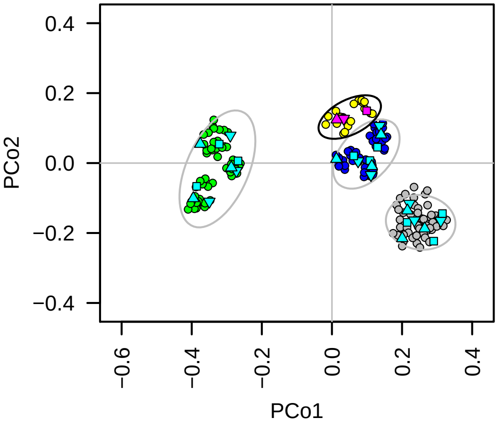

L2723 (m/z=1098.327838; rt=16.17909)
T/S Cluster: L−16.2−6

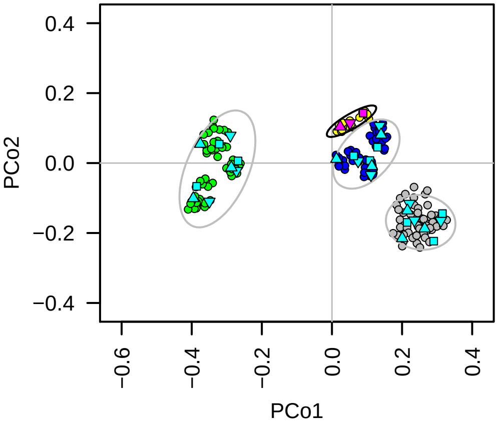

L2720 (m/z=1095.329566; rt=16.17897)
T/S Cluster: L−16.2−6

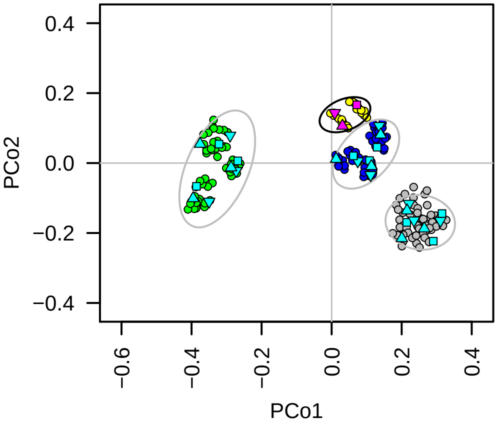

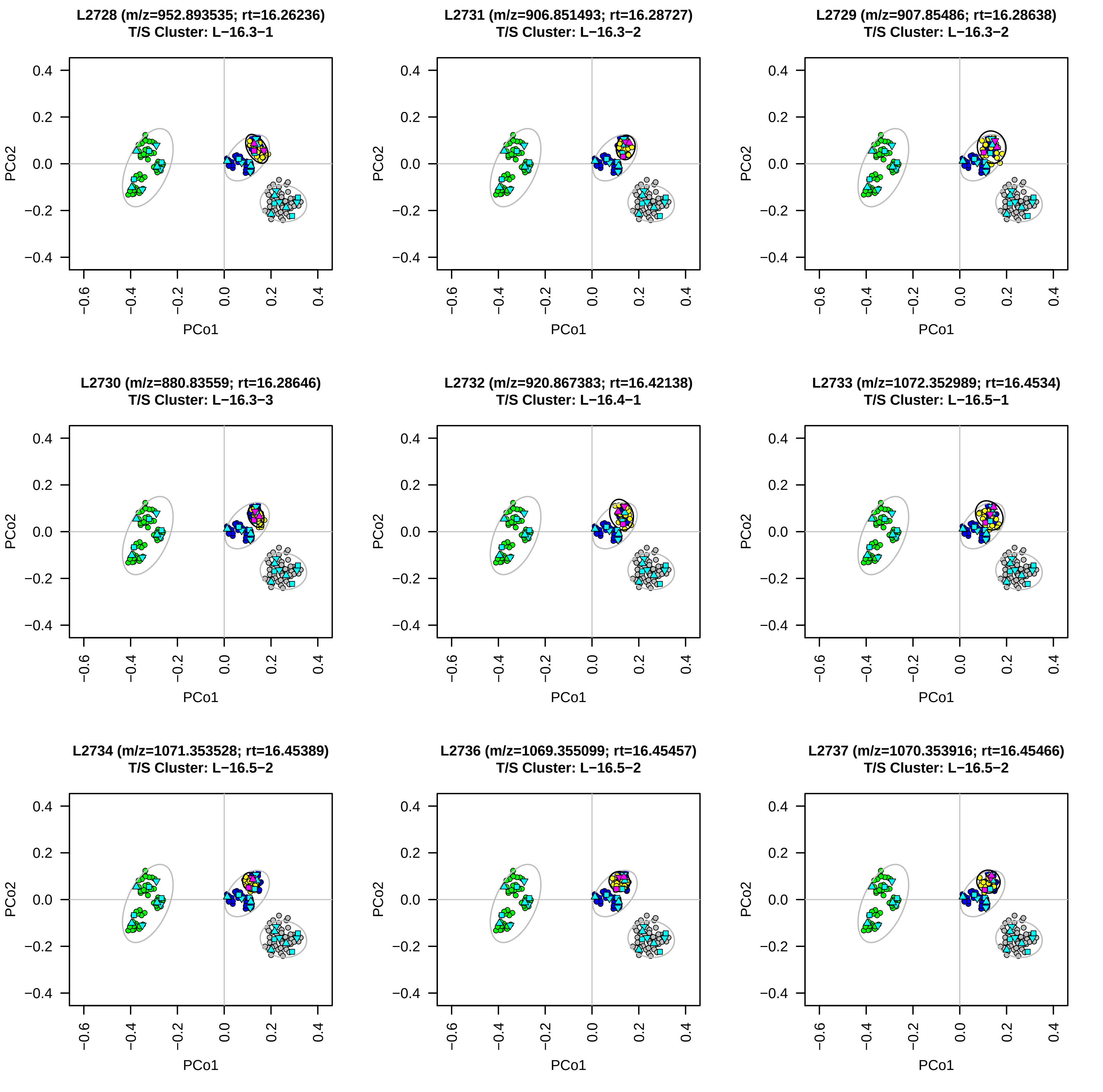
L2728 (m/z=952.893535; rt=16.26236)
T/S Cluster: L−16.3−1
L2731 (m/z=906.851493; rt=16.28727)
T/S Cluster: L−16.3−2
L2729 (m/z=907.85486; rt=16.28638)
T/S Cluster: L−16.3−2
L2730 (m/z=880.83559; rt=16.28646)
T/S Cluster: L−16.3−3
L2732 (m/z=920.867383; rt=16.42138)
T/S Cluster: L−16.4−1
L2733 (m/z=1072.352989; rt=16.4534)
T/S Cluster: L−16.5−1
L2734 (m/z=1071.353528; rt=16.45389)
T/S Cluster: L−16.5−2
L2736 (m/z=1069.355099; rt=16.45457)
T/S Cluster: L−16.5−2
L2737 (m/z=1070.353916; rt=16.45466)
T/S Cluster: L−16.5−2
PCo1
PCo2
0.4
0.2
0.0
−0.2
−0.4
−0.6

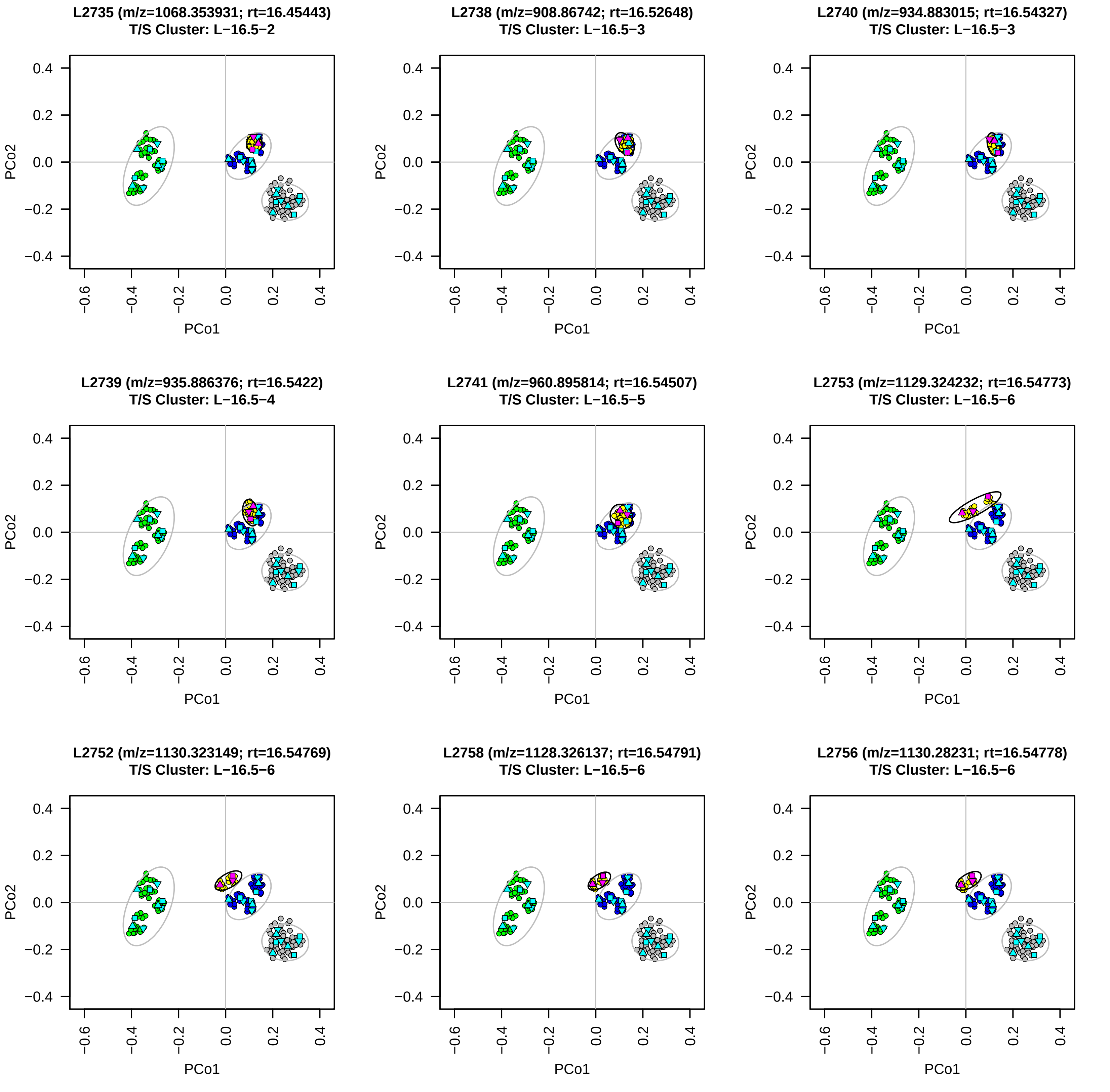
L2735 (m/z=1068.353931; rt=16.45443)
T/S Cluster: L−16.5−2
L2738 (m/z=908.86742; rt=16.52648)
T/S Cluster: L−16.5−3
L2740 (m/z=934.883015; rt=16.54327)
T/S Cluster: L−16.5−3
L2739 (m/z=935.886376; rt=16.5422)
T/S Cluster: L−16.5−4
L2741 (m/z=960.895814; rt=16.54507)
T/S Cluster: L−16.5−5
L2753 (m/z=1129.324232; rt=16.54773)
T/S Cluster: L−16.5−6
L2752 (m/z=1130.323149; rt=16.54769)
T/S Cluster: L−16.5−6
L2758 (m/z=1128.326137; rt=16.54791)
T/S Cluster: L−16.5−6
L2756 (m/z=1130.28231; rt=16.54778)
T/S Cluster: L−16.5−6
PCo1
PCo2
0.4
0.2
0.0
−0.2
−0.4
−0.6

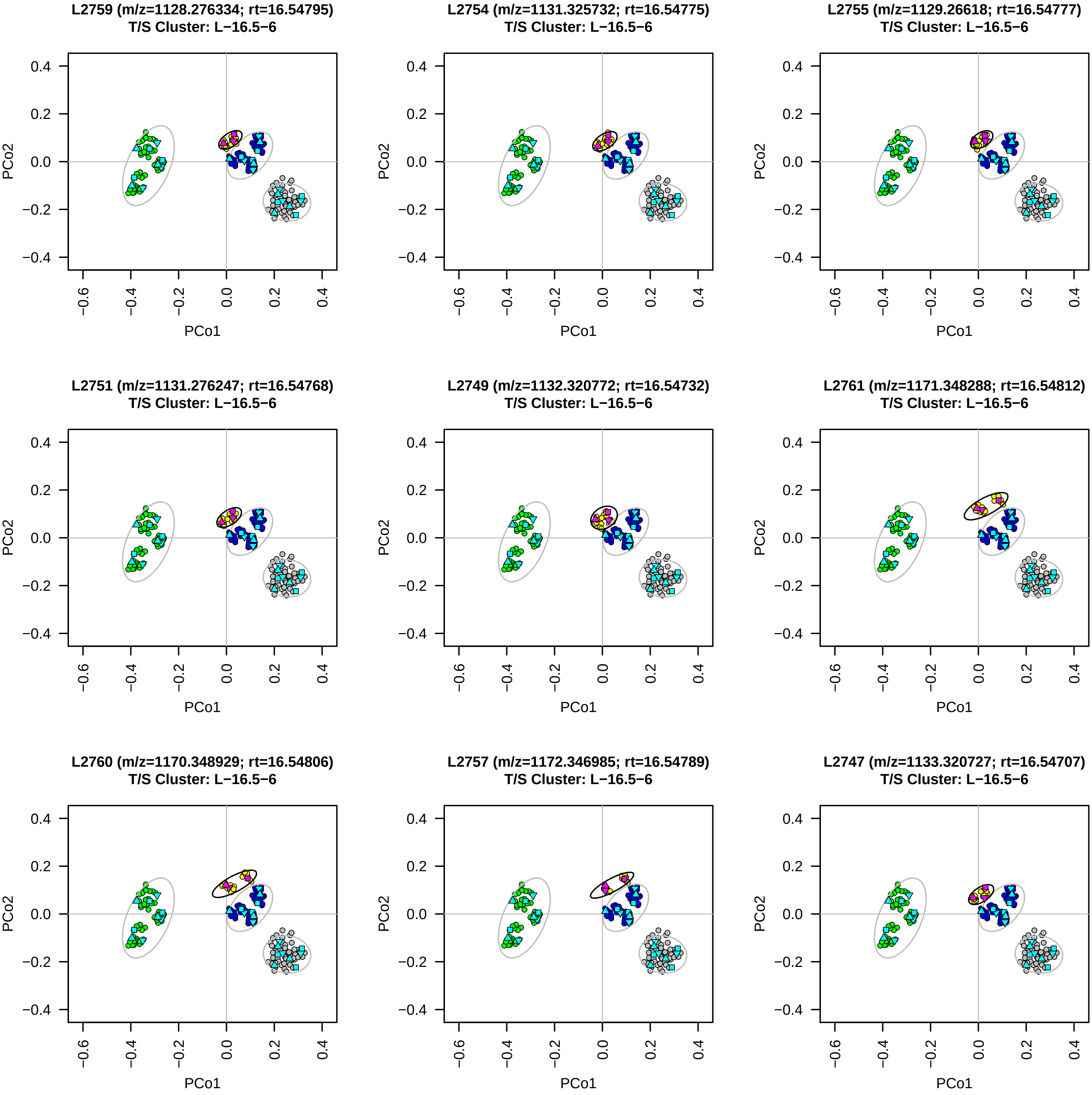
L2759 (m/z=1128.276334; rt=16.54795)
T/S Cluster: L−16.5−6
L2754 (m/z=1131.325732; rt=16.54775)
T/S Cluster: L−16.5−6
L2755 (m/z=1129.26618; rt=16.54777)
T/S Cluster: L−16.5−6
L2751 (m/z=1131.276247; rt=16.54768)
T/S Cluster: L−16.5−6
L2749 (m/z=1132.320772; rt=16.54732)
T/S Cluster: L−16.5−6
L2761 (m/z=1171.348288; rt=16.54812)
T/S Cluster: L−16.5−6
L2760 (m/z=1170.348929; rt=16.54806)
T/S Cluster: L−16.5−6
L2757 (m/z=1172.346985; rt=16.54789)
T/S Cluster: L−16.5−6
L2747 (m/z=1133.320727; rt=16.54707)
T/S Cluster: L−16.5−6
PCo1
PCo2
0.4
0.2
0.0
−0.2
−0.4
−0.6

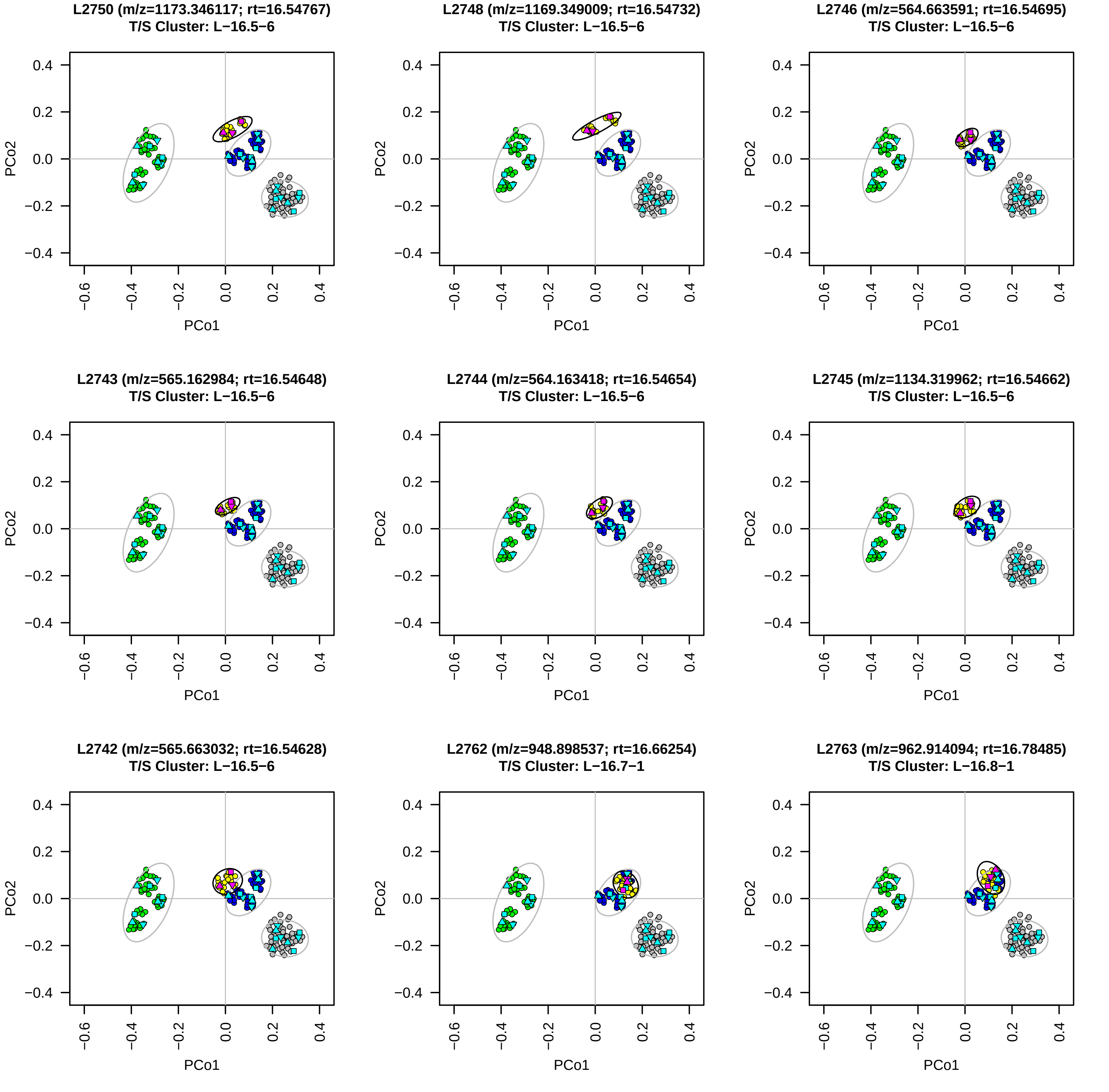
L2750 (m/z=1173.346117; rt=16.54767)
T/S Cluster: L−16.5−6
L2748 (m/z=1169.349009; rt=16.54732)
T/S Cluster: L−16.5−6
L2746 (m/z=564.663591; rt=16.54695)
T/S Cluster: L−16.5−6
L2743 (m/z=565.162984; rt=16.54648)
T/S Cluster: L−16.5−6
L2744 (m/z=564.163418; rt=16.54654)
T/S Cluster: L−16.5−6
L2745 (m/z=1134.319962; rt=16.54662)
T/S Cluster: L−16.5−6
L2742 (m/z=565.663032; rt=16.54628)
T/S Cluster: L−16.5−6
L2762 (m/z=948.898537; rt=16.66254)
T/S Cluster: L−16.7−1
L2763 (m/z=962.914094; rt=16.78485)
T/S Cluster: L−16.8−1
PCo1
PCo2
−0.6
−0.4
−0.2
0.0
0.2
0.4

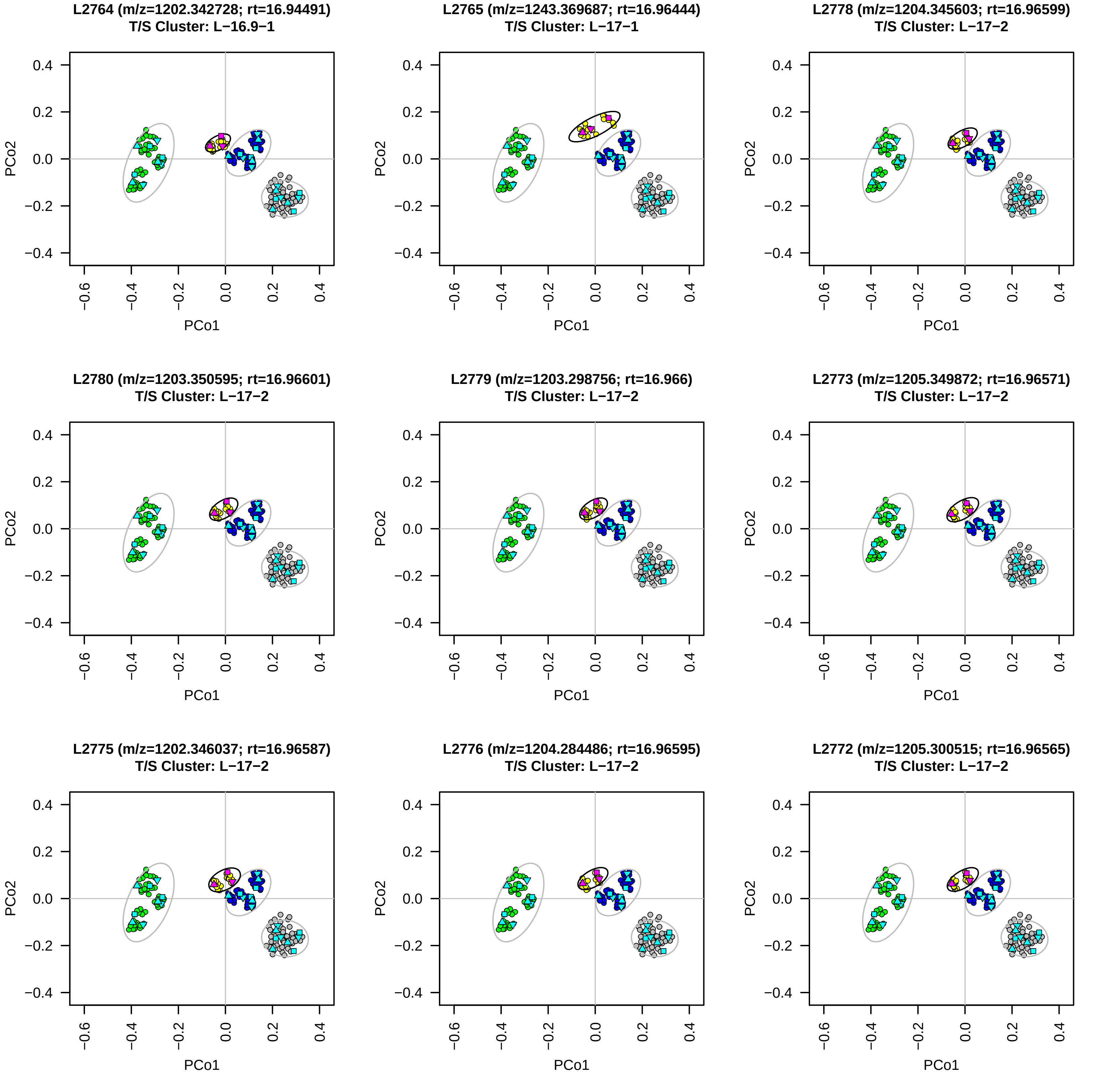
L2764 (m/z=1202.342728; rt=16.94491)
T/S Cluster: L−16.9−1
L2765 (m/z=1243.369687; rt=16.96444)
T/S Cluster: L−17−1
L2778 (m/z=1204.345603; rt=16.96599)
T/S Cluster: L−17−2
L2780 (m/z=1203.350595; rt=16.96601)
T/S Cluster: L−17−2
L2779 (m/z=1203.298756; rt=16.966)
T/S Cluster: L−17−2
L2773 (m/z=1205.349872; rt=16.96571)
T/S Cluster: L−17−2
L2775 (m/z=1202.346037; rt=16.96587)
T/S Cluster: L−17−2
L2776 (m/z=1204.284486; rt=16.96595)
T/S Cluster: L−17−2
L2772 (m/z=1205.300515; rt=16.96565)
T/S Cluster: L−17−2
PCo1
PCo2
0.4
0.2
0.0
−0.2
−0.4
−0.6

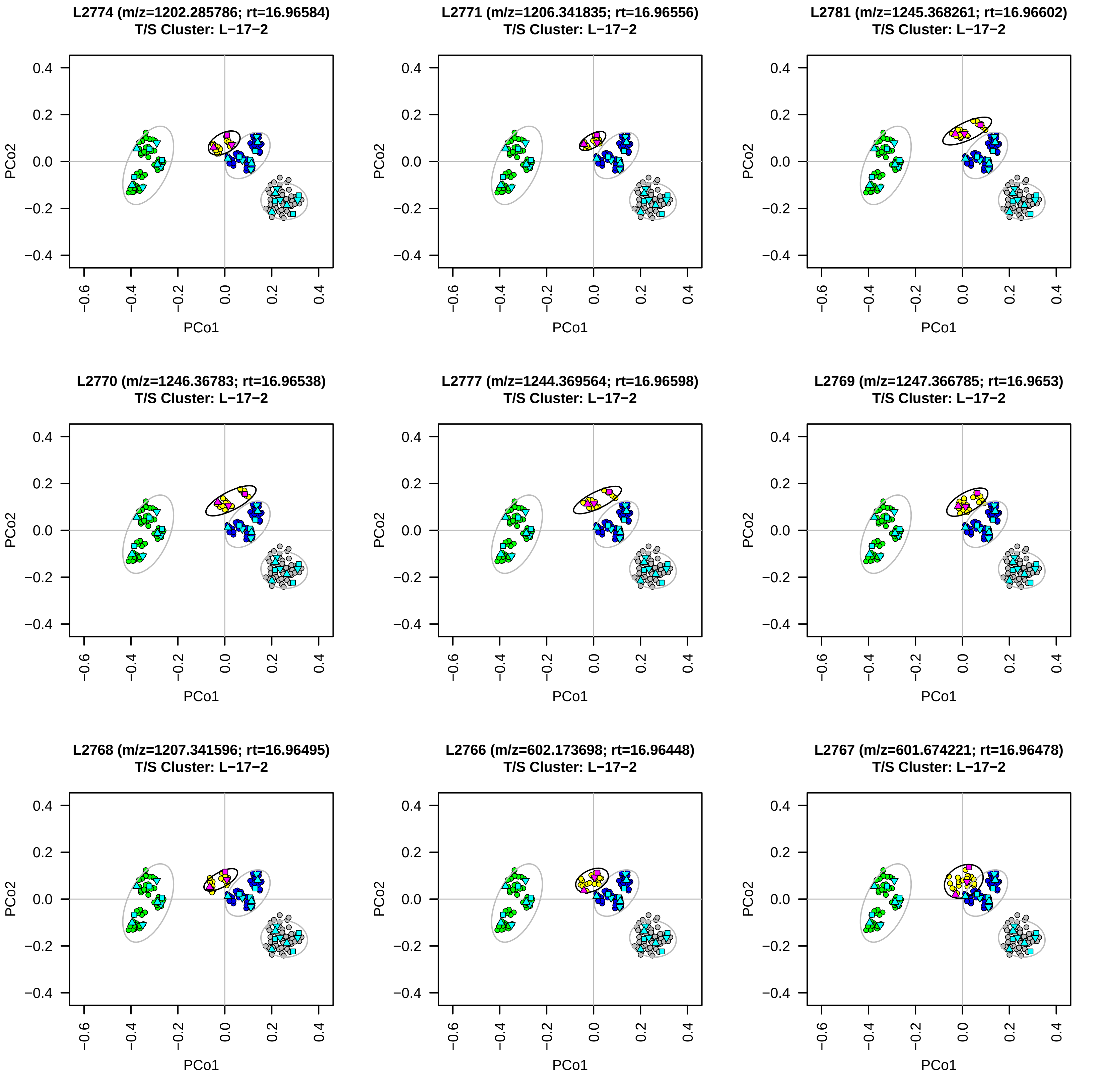
L2774 (m/z=1202.285786; rt=16.96584)
T/S Cluster: L−17−2
L2771 (m/z=1206.341835; rt=16.96556)
T/S Cluster: L−17−2
L2781 (m/z=1245.368261; rt=16.96602)
T/S Cluster: L−17−2
L2770 (m/z=1246.36783; rt=16.96538)
T/S Cluster: L−17−2
L2777 (m/z=1244.369564; rt=16.96598)
T/S Cluster: L−17−2
L2769 (m/z=1247.366785; rt=16.9653)
T/S Cluster: L−17−2
L2768 (m/z=1207.341596; rt=16.96495)
T/S Cluster: L−17−2
L2766 (m/z=602.173698; rt=16.96448)
T/S Cluster: L−17−2
L2767 (m/z=601.674221; rt=16.96478)
T/S Cluster: L−17−2
PCo1
PCo2

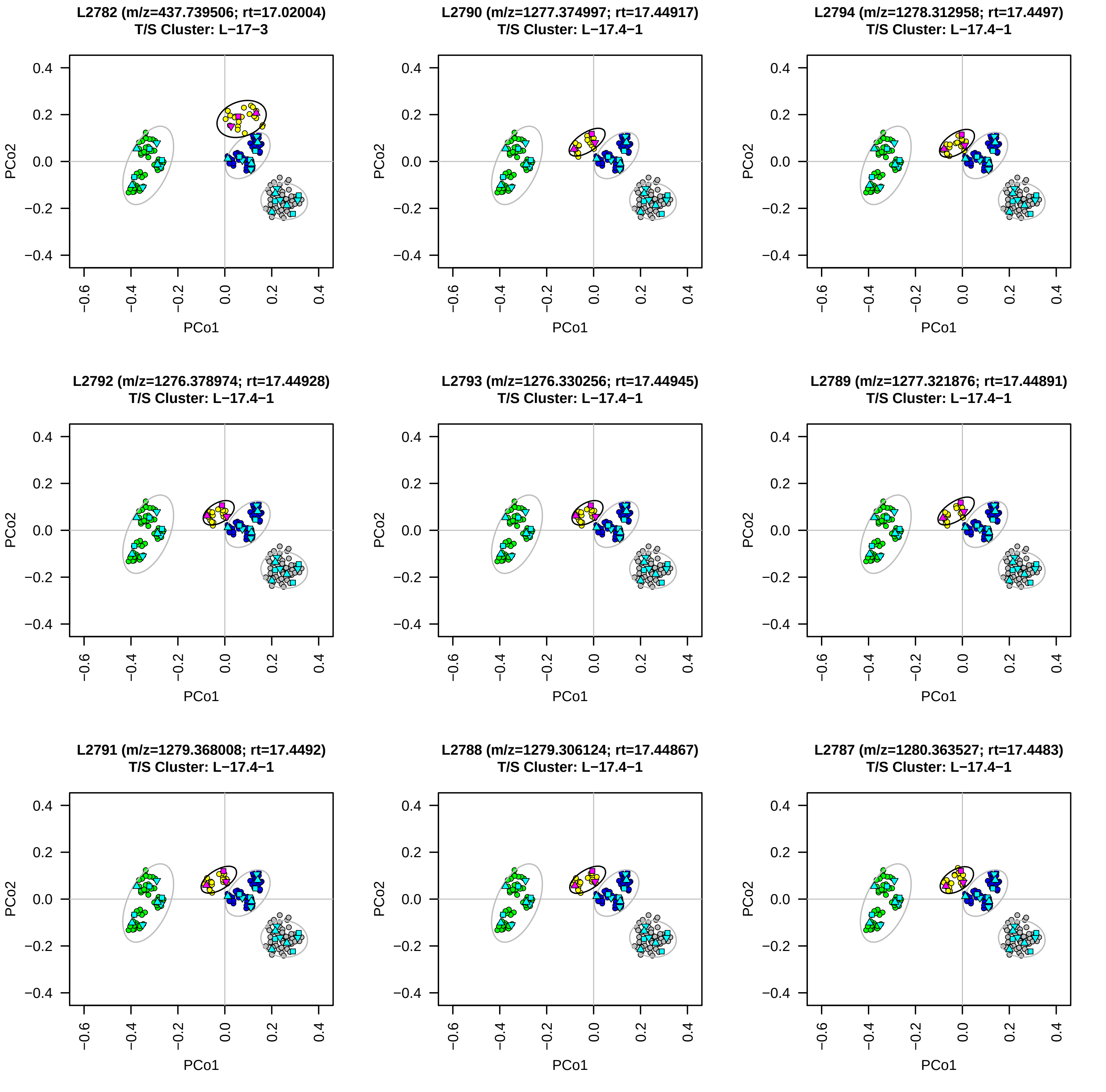
L2782 (m/z=437.739506; rt=17.02004)
T/S Cluster: L−17−3
L2790 (m/z=1277.374997; rt=17.44917)
T/S Cluster: L−17.4−1
L2794 (m/z=1278.312958; rt=17.4497)
T/S Cluster: L−17.4−1
L2792 (m/z=1276.378974; rt=17.44928)
T/S Cluster: L−17.4−1
L2793 (m/z=1276.330256; rt=17.44945)
T/S Cluster: L−17.4−1
L2789 (m/z=1277.321876; rt=17.44891)
T/S Cluster: L−17.4−1
L2791 (m/z=1279.368008; rt=17.4492)
T/S Cluster: L−17.4−1
L2788 (m/z=1279.306124; rt=17.44867)
T/S Cluster: L−17.4−1
L2787 (m/z=1280.363527; rt=17.4483)
T/S Cluster: L−17.4−1
PCo1
PCo2

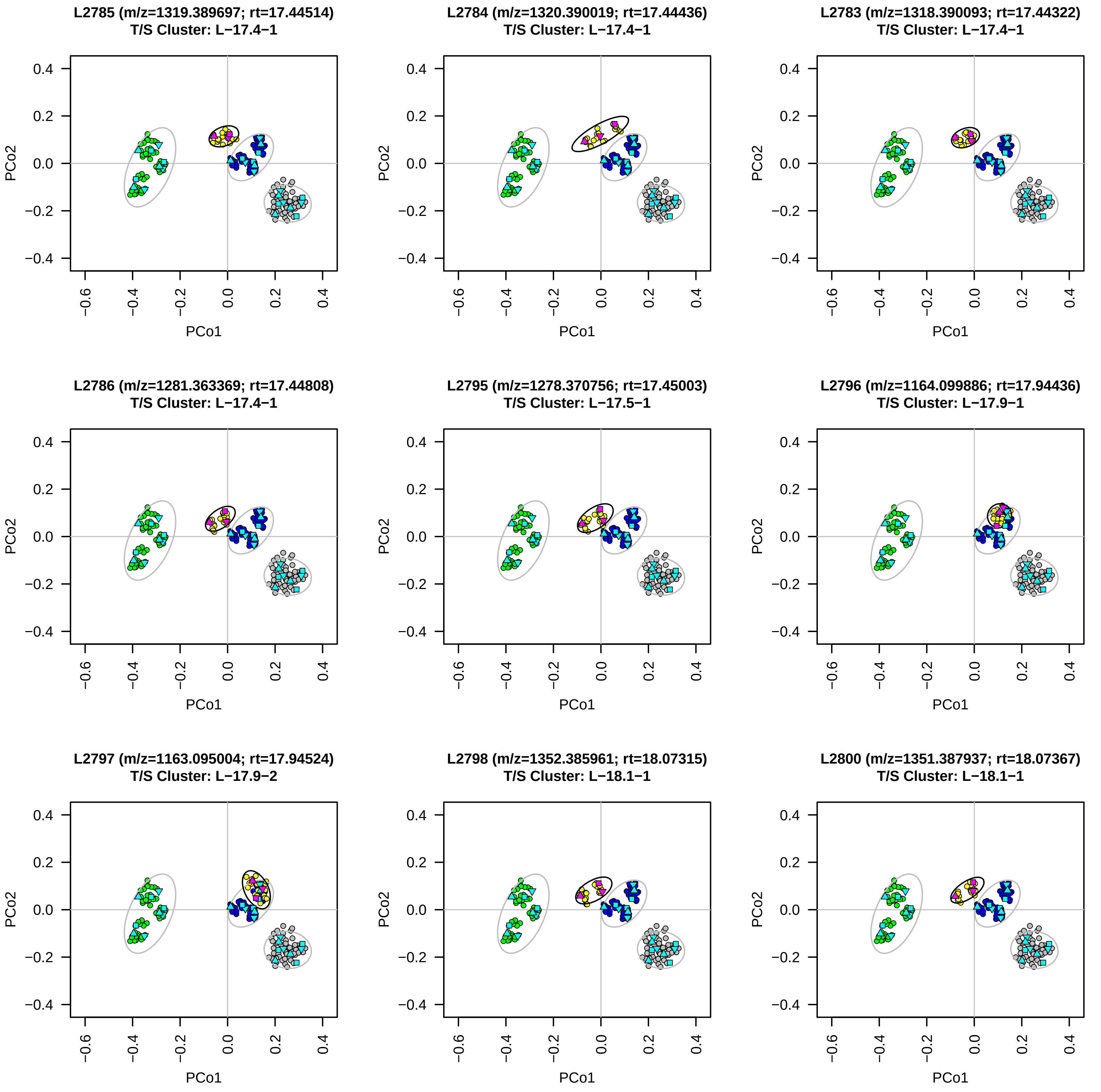
L2785 (m/z=1319.389697; rt=17.44514)
T/S Cluster: L−17.4−1
L2784 (m/z=1320.390019; rt=17.44436)
T/S Cluster: L−17.4−1
L2783 (m/z=1318.390093; rt=17.44322)
T/S Cluster: L−17.4−1
L2786 (m/z=1281.363369; rt=17.44808)
T/S Cluster: L−17.4−1
L2795 (m/z=1278.370756; rt=17.45003)
T/S Cluster: L−17.5−1
L2796 (m/z=1164.099886; rt=17.94436)
T/S Cluster: L−17.9−1
L2797 (m/z=1163.095004; rt=17.94524)
T/S Cluster: L−17.9−2
L2798 (m/z=1352.385961; rt=18.07315)
T/S Cluster: L−18.1−1
L2800 (m/z=1351.387937; rt=18.07367)
T/S Cluster: L−18.1−1
PCo1
PCo2
−0.6
−0.4
−0.2
0.0
0.2
0.4

L2801 (m/z=1353.387303; rt=18.07392)
T/S Cluster: L−18.1−1

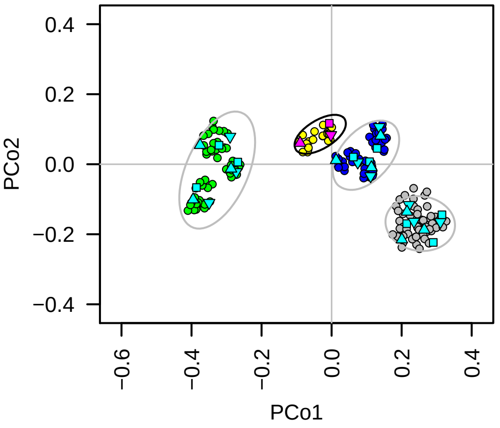

L2799 (m/z=1350.386176; rt=18.07326)
T/S Cluster: L−18.1−1

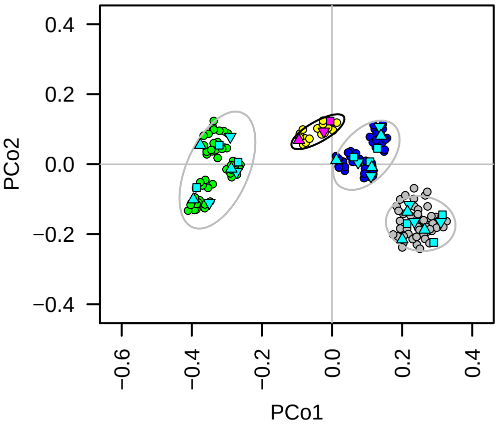

L2802 (m/z=1354.386521; rt=18.07501)
T/S Cluster: L−18.1−1

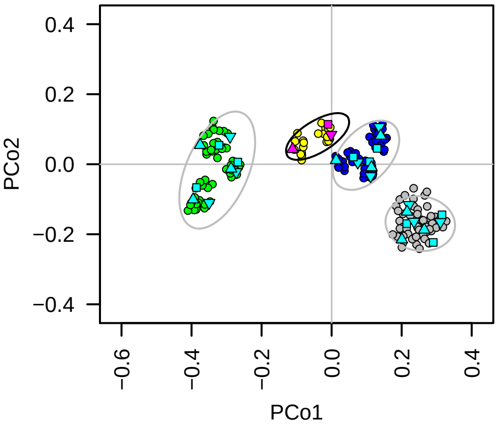

L2803 (m/z=1192.1306; rt=18.4148)
T/S Cluster: L−18.4−1

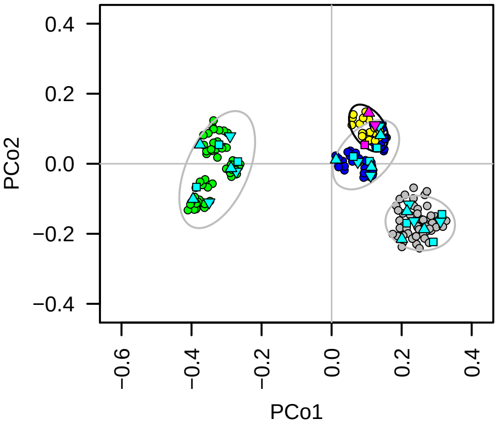

L2804 (m/z=1191.127696; rt=18.41561)
T/S Cluster: L−18.4−2

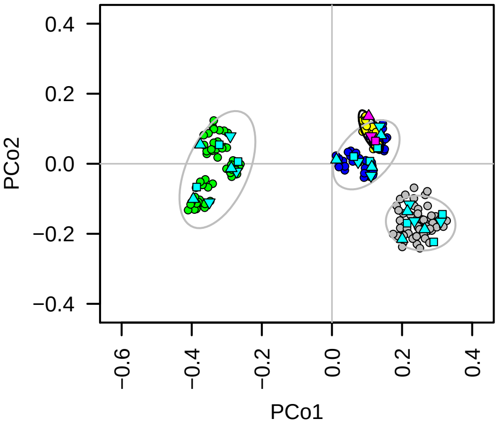

Supplement: Data S6 — Scatter plots of analytes and compartment-specific markers in the principal coordinates space for visual assessment of subcellular location. (PDF) [file pone.0017806.s014.pdf]
